# Evolutionary history of the human multigene families reveals widespread gene duplications throughout the history of animals

**DOI:** 10.1186/s12862-019-1441-0

**Published:** 2019-06-20

**Authors:** Nashaiman Pervaiz, Nazia Shakeel, Ayesha Qasim, Rabail Zehra, Saneela Anwar, Neenish Rana, Yongbiao Xue, Zhang Zhang, Yiming Bao, Amir Ali Abbasi

**Affiliations:** 1 National Center for Bioinformatics, Programme of Comparative and Evolutionary Genomics, Faculty of Biological Sciences, Quaid-i-Azam University, Islamabad, 45320 Pakistan; 2 BIG Data Center, Beijing Institute of Genomics, Chinese Academy of Sciences, Beijing 100101; University of Chinese Academy of Sciences, Beijing, 100049 China

**Keywords:** Human, Whole genome duplications, Segmental duplications, Paralogons, Paralogy regions, Vertebrate, Multigene families, Phylogenetic analysis

## Abstract

**Background:**

The hypothesis that vertebrates have experienced two ancient, whole genome duplications (WGDs) is of central interest to evolutionary biology and has been implicated in evolution of developmental complexity. Three-way and Four-way paralogy regions in human and other vertebrate genomes are considered as vital evidence to support this hypothesis. Alternatively, it has been proposed that such paralogy regions are created by small-scale duplications that occurred at different intervals over the evolution of life.

**Results:**

To address this debate, the present study investigates the evolutionary history of multigene families with at least three-fold representation on human chromosomes 1, 2, 8 and 20. Phylogenetic analysis and the tree topology comparisons classified the members of 36 multigene families into four distinct co-duplicated groups. Gene families falling within the same co-duplicated group might have duplicated together, whereas genes belong to different co-duplicated groups might have distinct evolutionary origins.

**Conclusion:**

Taken together with previous investigations, the current study yielded no proof in favor of WGDs hypothesis. Rather, it appears that the vertebrate genome evolved as a result of small-scale duplication events, that cover the entire span of the animals’ history.

**Electronic supplementary material:**

The online version of this article (10.1186/s12862-019-1441-0) contains supplementary material, which is available to authorized users.

## Background

To elucidate the genetic underpinnings of major changes in organismal make up and the origination of ample new traits during the evolutionary history of vertebrates, Susumu Ohno in the year 1970 put forward the hypothesis that two rounds of whole genome duplications (WGDs) occurred early in vertebrate evolution. This hypothesis is popularly termed as “2R hypothesis” (two rounds of WGDs) and is believed to be the most rational explanation for the complexity of modern-day vertebrate genome [1]. The 2R has been under immense scrutiny over the past couple of decades [2–9]. The occurrence of intra-genomic conserved syntenic blocks (paralogy groups/paralogons) in vertebrate genomes is presented as the most credible proof furthering the ancient WGDs [10, 11]. Markedly, the presence of four potential quadruplicated regions on *Homo sapiens* autosomes (Hsa) 1/6/9/19 (MHC bearing paralogon), Hsa 4/5/8/10 (FGFR bearing chromosomes), Hsa 1/2/8/20 and Hsa 2/7/12/17 (HOX-cluster bearing chromosomes), is considered as an outcome of two consecutive rounds of WGDs [12]. However, alternatively it is hypothesized that the excess of paralogy regions in the human and other vertebrate genomes is due to higher instance of local duplications, translocations and chromosomal restructuring that occurred extensively at different intervals during early vertebrate history, thus nullifying the Ohno’s postulation [13].

In order to evaluate the mechanisms behind the formation of vertebrate paralogy regions, our research group has continuously been putting efforts in assembling and dating the gene duplications that occurred during the animal’s evolutionary history [3, 4, 7, 14–17]. Previously, we investigated the evolutionary histories of 11 multigene families (40 human genes) with triplicated or quadruplicated presence on Hsa 1/2/8/20. The results achieved were in contrast with 2R hypothesis, suggesting that the paralogy fragments on human chromosomes 1, 2, 8 and 20 are an outcome of small-scale duplication events which scattered across the history of metazoans [3, 4, 14, 17, 18].

In this study, we furthered our efforts [14] to analyze the evolutionary history of 25 human multigene families with three or fourfold distribution on Hsa 1/2/8/20. A robust and detailed phylogenomic analysis was carried out by using the recently available well-annotated and high-quality genome sequence data from a wide range of metazoans [19–21]. The topology comparison approach was particularly applied on the phylogenetic data of total 36 families (25 present data and 11 previous data) to classify the genes that might have duplicated together early in vertebrate history [3, 14]. In addition, relative timing approach was employed to estimate the timings of gene duplication events. In sync with the previous results [14], it appeared that the triplicated or quadruplicated gene families residing on Hsa 1/2/8/20 have not arisen simultaneously through 2R. Rather, phylogenetic data clarifies that the tetra-paralogy blocks on the human genome have resulted from independent duplications, segmental duplications and genomic restructuring events that had occurred at broadly different time points during the course of animal evolution.

## Results

**Fig. 1:**
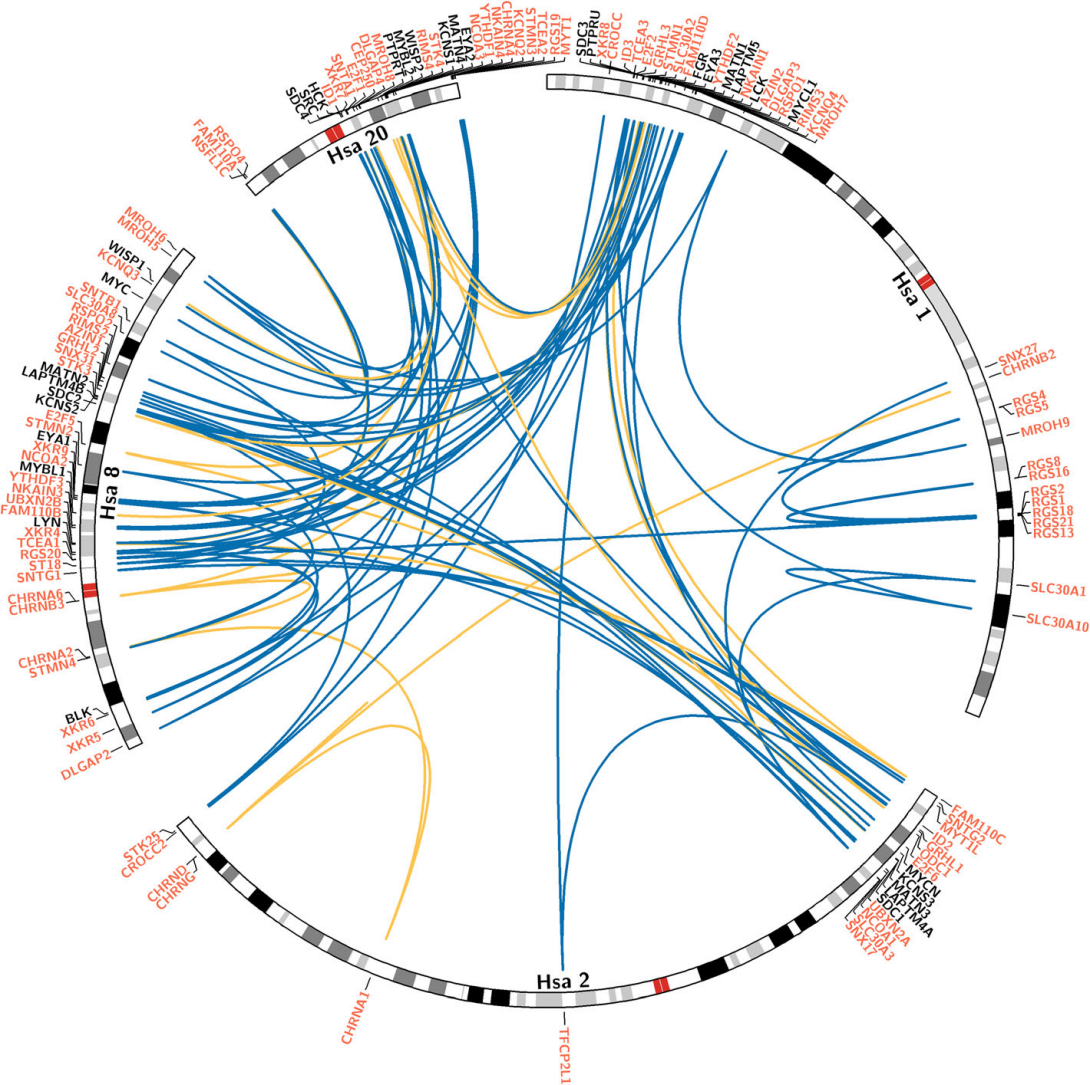
Evolutionary history of human tetra-paralogon Hsa 1/2/8/20. A circular view of human chromosomes shows the paralogons detected among human chromosomes 1/2/8/20, including the synteny relationship among 36 distinct multigene families: 11 families from previously published data that are labeled in black [14], whereas the 25 families analyzed in the present study that are labeled in green. Blue lines connect positions on ideograms for gene families with 3-fold representation, while yellow lines connect families with four-fold representation on these chromosomes. Detailed information about each family is given in Table 1

**Fig. 2:**
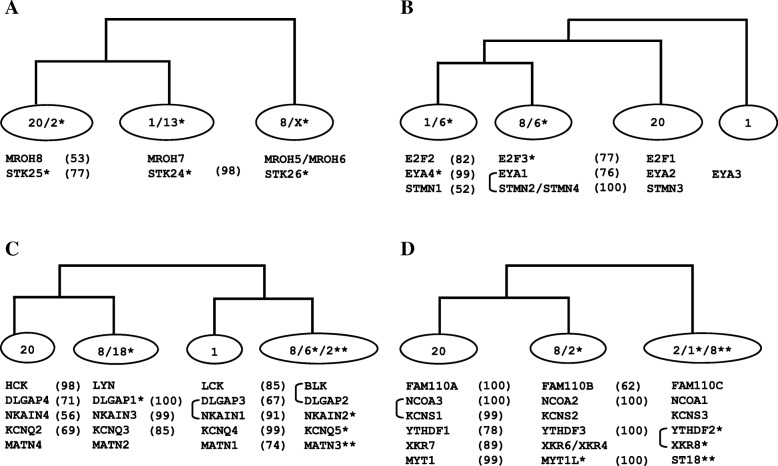
The human genes duplicated in parallel lie in respective co-duplicated groups. Consistencies in phylogenetic tree topologies of families (analyzed in this and our previous study) with at least threefold representation on human tetra-paralogon Hsa1/2/8/20 (**a**) Schematic topology of MROH and STK families; **b** schematic topology of E2F, EYA and STMN families; **c** schematic topology of HCK, DLGAP, NKAIN, KCNQ and MATN gene families; **d** schematic topology of FAM110, NCO, KCNS, YTHDF, XKR and MYT gene families. For each case, the percentage bootstrap values of internal branches are provided in parentheses except for gene families exhibiting slightly lower bootstrap values (≤50%).The connecting bars on the left portray the close physical associations of relevant genes. Asterisk symbol * designate the relevant chromosomes

For investigating the validity of whole genome duplications (WGDs) hypothesis, which strongly supports that fourfold paralogons in the human genome had been formed by polyploidization events, we undertook phylogenetic analyses for 25 gene families (see details in Methods). Each of these chosen subset of multigene families have at least threefold portrayal on one of the paralogy regions in human genome that comprises of segments from human chromosomes 1, 2, 8 and 20 (Fig. 1; Table 1). By employing currently available wide range of sequenced vertebrate and invertebrate genomes, orthologous sequence data was gathered. (Additional file 1). This wider set of taxonomic representation in the sequence data enabled us to perform a robust phylogenetic examination based on NJ and ML methods (Additional files 2, 3 and 4). Given the phylogenetic data, we next determine the co-duplication events by employing the topology comparison approach [3, 17, 22] (Fig. 2). The phylogenetic tree topology comparison approach takes into account uniformity among tree branching pattern of distinct but physically linked gene families as a proof of their joint origin, thus displaying co-duplicated groups [13, 23]. In contrast, the non-uniform tree topologies of physically linked distinct families suggest the incongruent duplication histories of concerned genes [16]. For this purpose, only those sections of 36 phylogenies were chosen for which there is a strong bootstrap support for at least two gene duplication events within the time frame that divided the teleosts and vertebrates from tetrapods and invertebrates respectively (proposed timing of WGDs) (Additional file 5: Table S1). Among them 11 families were published previously by our research group [14].

**Table 1:** List of human gene families used in the phylogenetic analysis

Gene family	Members	Chr location	Human protein accession No.	Number of included taxa	Number of sequences included
Antizyme Inhibitor	AZIN2	1p35.1	Q96A70	25	54
	ODC1	2p25	P11926		
	AZIN1	8q22.3	O14977		
Cholinergic Receptors Nicotinic subunits	CHRNB2	1q21.3	P17787	32	123
	CHRNG	2q37.1	P07510		
	CHRND	2q37.1	Q07001		
	CHRNA1	2q31.1	P02708		
	CHRNA2	8p21	Q15822		
	CHRNA6	8p11.21	Q15825		
	CHRNB3	8p11.2	Q05901		
	CHRNA4	20q13.33	P43681		
	CHRNA3	15q24	P32297		
	CHRNB4	15q24	P30926		
	CHRNB1	17p13.1	P11230		
	CHRNE	17p13.2	Q04844		
	CHRNA5	15q24	P30532		
Ciliary Rootlet Coiled-Coil Protein	CROCC	1p36.13	Q5TZA2	28	42
	CROCC2	2q37.3	H7BZ55		
	CEP250	20q11.22	Q9BV73		
Discs, large (Drosophila) Homolog-associated Protein	DLGAP3	1p35.3-p34.1	O95886	25	85
	DLGAP1	18p11.31	O14490		
	DLGAP5	14q22.3	Q15398		
	DLGAP2	8p23	Q9P1A6		
	DLGAP4	20q11.23	Q9Y2H0		
E2F Transcription Factor	E2F2	1p36	Q14209	31	84
	E2F6	2p25.1	O75461		
	E2F5	8q21.2	Q15329		
	E2F1	20q11.2	Q01094		
	E2F3	6p22	O00716		
	E2F4	16q22.1	Q16254		
Family with Sequence Similarity 110	FAM110D	1p36.11	Q8TAY7	25	56
	FAM110C	2p25.3	Q1W6H9		
	FAM110B	8q12.1	Q8TC76		
	FAM110A	20p13	Q9BQ89		
Grainyhead like Transcription factor	GRHL3	1p36.11	Q8TE85	26	57
	TFCP2L1	2q14	Q9NZI6		
	GRHL1	2p25.1	Q9NZI5		
	GRHL2	8q22.3	Q6ISB3		
	TFCP2	12q13	Q12800		
	UBP1	3p22.3	Q9NZI7		
Inhibitor of DNA Binding protein	ID3	1p36.13-p36.12	Q02535	35	65
	ID2	2p25	Q02363		
	ID1	20q11	P41134		
	ID4	6p22.3	P47928		
Maestro Heat-like Repeat-containing Protein Family	MROH9	1q24.3	Q5TGP6	22	46
	MROH7	1p32.3	Q68CQ1		
	MROH6	8q24.3	A6NGR9		
	MROH5	8q24.3	Q6ZUA9		
	MROH8	20q11.22	Q9H579		
Myelin Transcription Factor	MYT1L	2p25.3	Q9UL68	22	48
	ST18	8q11.23	O60284		
	MYT1	20q13.33	Q01538		
Nuclear Receptor Coactivator	NCOA1	2p23	Q15788	22	54
	NCOA2	8q13.3	Q15596		
	NCOA3	20q12	Q9Y6Q9		
Na+/K+ Transporting ATPase Interacting Protein	NKAIN1	1p35.2	Q4KMZ8	24	46
	NKAIN3	8q12.3	Q8N8D7		
	NKAIN4	20q13.33	Q8IVV8		
	NKAIN2	6q21	Q5VXU1		
Potassium Voltage-Gated Channel subfamily Q	KCNQ4	1p34	P56696	28	67
	KCNQ3	8q24	O43525		
	KCNQ2	20q13.3	O43526		
	KCNQ5	6q14	Q9NR82		
	KCNQ1	11p15.5	P51787		
Regulator of G-protein Signalling	RGS13	1q31.2	O14921	31	101
	RGS8	1q25	P57771		
	RGS1	1q31	Q08116		
	RGS18	1q31.2	Q9NS28		
	RGS16	1q25-q31	O15492		
	RGS21	1q31.2	Q2M5E4		
	RGS4	1q23.3	P49798		
	RGS2	1q31	P41220		
	RGS20	8q11.23	O76081		
	RGS19	20q13.33	P49795		
	RGS17	6q25.3	Q9UGC6		
	RGS3	9q32	P49796		
	RGS5	1q23.1	O15539		
Regulating Synaptic Membrane Exocytosis Protein	RIMS3	1p34.2	Q9UJD0	27	49
	RIMS2	8q22.3	Q9UQ26		
	RIMS4	20q13.12	Q9H426		
	RIMS1	6q12-q13	Q86UR5		
R-Spondin Homolog	RSPO1	1p34.3	Q2MKA7	31	60
	RSPO2	8q23.1	Q6UXX9		
	RSPO4	20p13	Q2I0M5		
	RSPO3	6q22.33	Q9BXY4		
Solute Carrier Family	SLC30A2	1p35.3	Q9BRI3	23	74
	SLC30A10	1q41	Q6XR72		
	SLC30A1	1q32.3	Q9Y6M5		
	SLC30A3	2p23.3	Q99726		
	SLC30A8	8q24.11	Q8IWU4		
	SLC30A4	15q21.1	O14863		
Syntrophin, Gamma	SNTG2	2p25.3	Q9NY99	28	81
	SNTG1	8q11.21	Q9NSN8		
	SNTB1	8q23-q24	Q13884		
	SNTA1	20q11.2	Q13424		
	SNTB2	16q22.1	P49711		
	GOPC	6q21	Q9HD26		
Sorting Nexin Family	SNX27	1q21.3	Q96L92	29	43
	SNX17	2p23.3	Q15036		
	SNX31	8q22.3	Q8N9S9		
Stathmin	STMN1	1p36.11	P16949	22	63
	STMN2	8q21.13	Q93045		
	STMN4	8p21.2	Q9H169		
	STMN3	20q13.3	Q9NZ72		
Serine/Threonine-Protein Kinase	STK25	2q37.3	O00506	25	72
	STK3	8q22.2	Q13188		
	STK4	20q11.2-q13.2	Q13043		
	STK24	13q31.2-q32.3	Q9Y6E0		
	STK26	Xq26.2	Q9P289		
Transcription Elongation factor A (SII) Protein	TCEA3	1p36.12	O75764	22	51
	TCEA1	8q11.2	P23193		
	TCEA2	20q13.33	Q15560		
	TCEANC	Xp22.2	Q8N8B7		
UBX Domain-Containing Protein	UBXN2A	2p23.3	P68543	22	32
	UBXN2B	8q12.1	Q14CS0		
	NSFL1C	20p13	Q9UNZ2		
X Kell Blood Group Precursor-related Family	XKR8	1p35.3	Q9H6D3	24	101
	XKR9	8q13.3	Q5GH70		
	XKR6	8p23.1	Q5GH73		
	XKR4	8q12.1	Q5GH76		
	XKR5	8p23.1	Q6UX68		
	XKR7	20q11.21	Q5GH72		
YTH Domain-Containing Family Protein	YTHDF2	1p35	Q9Y5A9	24	50
	YTHDF3	8q12.3	Q7Z739		
	YTHDF1	20q13.33	Q9BYJ9

MROH and STK gene family members has threefold representation on Hsa 1/2/8/20 paralogon and diversified by at least two vertebrate specific duplication events (Additional file 2). Assuming three independent gene translocation events in STK gene family, congruent but asymmetrical topologies of the type ((Hsa20/2 Hsa1/13) Hsa8/X) are recovered for these two gene families (Fig. 2a). This pattern indicates that the subset members of MROH and STK families might have duplicated in block through segmental duplication (SD) events.

E2F family has fourfold representation, whereas EYA and STMN families has threefold representation on tetra-paralogon Hsa 1/2/8/20. Assuming two independent gene translocation events revealed congruent and asymmetrical topologies of the type (((Hsa1/6 Hsa8/6) Hsa20) for E2F, EYA and STMN families (Fig. 2b; Additional file 2).

MATN family has fourfold presense, whereas HCK, DLGAP, NKAIN and KCNQ families has threefold portrayal on tetra-paralogy regions residing on Hsa 1/2/8/20. By assuming five gene translocation events, congruent and symmetrical topology of the type ((A, B) (C, D)) i.e. ((Hsa20-Hsa8/18) (Hsa1-Hsa8/6/2)) is recovered for HCK, DLGAP, NKAIN, KCNQ, and MATN families (Fig. 2c; Additional file 2).

FAM110 family has fourfold depiction whereas NCOA, KCNS, YTHDF, XKR, and MYT families has threefold distribution on Hsa 1/2/8/20. Each of these five families experienced at least two vertebrate specific duplication events (Additional file 2). By assuming four independent gene translocation events, members of these five families constitute the fourth co-duplicated group with an asymmetrical tree topology of the type ((Hsa20-Hsa8/2) Hsa2/1/8) (Fig. 2d).

Phylogenetic trees of eight gene families (CHRN, RGS, GRHL, RIMS, RSPO, ID, TCEA, and SNT) involve complex histories with majority of duplications occurred anciently prior to vertebrate–invertebrate split. CHRN family appear to have diversified by in total twelve duplications, six of them predate the vertebrate-invertebrate split (Additional file 2). RGS family tree indicates 10 duplication events, five of them occurred earlier than vertebrate-invertebrate split (Additional file 2). The tree topology pattern of GRHL indicates in total six duplications, two of them occured at least prior to protostome–deuterostome split (Additional file 2). The tree topology of RIMS family reveals three duplication events, one of them occurred earlier than Bilaterian–Nonbilaterian divergence (Additional file 2). RSPO arose by three independent gene duplication events, one of them happened prior to the divergence of echinoderms from vertebrates (Additional file 2). Vertebrate ID family tree revealed three independent gene duplication events, two of them occurred prior to hemichordates-vertebrates split (Additional file 2). Members of TCEA family arose by four duplications, three of them occurred earlier than vertebrate-cephalochordate split (Additional file 2). SNT paralogs experienced five duplications, four of them occurred prior to protostomes and deuterostomes split (Additional file 2).

Phylogenetic tree topologies of five families (AZIN, CRO, SLC, SNX and UBXN) reveal no evidence for vertebrate specifc gene duplications. All of these families are diversified by duplications that predates the vertebrate-invertebrate split (Additional file 2).

**Fig. 3:**
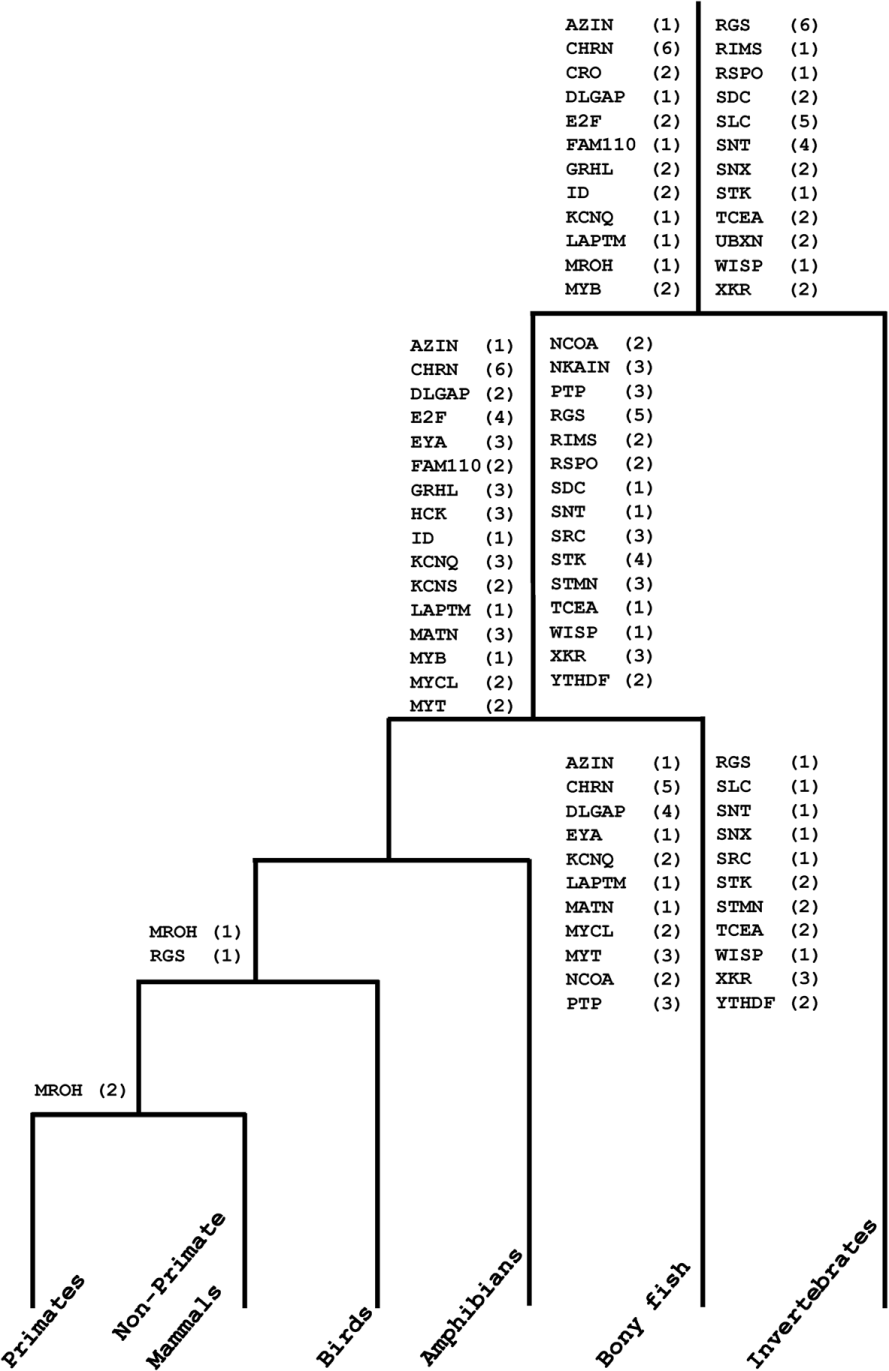
The relative timings of gene duplication events. For the 36 multigene families analyzed in this study, 52 gene duplications are detected before the invertebrate-vertebrate divide and 74 duplications are detected after invertebrate-vertebrate and before tetrapod-bony fish divergence. Only four tetrapod specific duplication events are detected. The numbers enclosed in the parentheses following gene family names represent the count of duplications experienced by family. Gene families are ordered alphabetically

Estimation of gene duplication events with respect to relative timing of speciations provides a bird’s eye view to all the duplications that occurred in a particular time window [24]. Taken together the phylogenetic histories of 36 families (25 present data and 11 previously analyzed); in total 172 duplication events are recovered (Fig. 3). It appears that 52 of these duplication events occurred earlier than invertebrate-vertebrate- split, whereas 74 duplications are identified at the root of vertebrate history prior to tetrapod-teleost- divergence. Furthermore, 42 teleost fish specific and only 4 tetrapod specific duplication events are detected (Fig. 3).

## Discussion

Different post genomic methods like, genome wide pairwise comparisons and genome self comparisons have been robustly utilized in order to analyze the evolutionary basis for the origination of paralogy blocks in vertebrate genomes [11]. Evolutionary events in the recent vertebrate history has been successfully highlighted by these approaches, as the identity of recently duplicated intra-genomic and inter-genomic conserved syntenic segments and thus the patterns of evolution preceeding their origin are not vagued by evolutionary divergence, and genomic anomalies like chromosomal breakage and rearrangements [25]. For instance, complex pattern of segmental duplications (SDs) has been witnessed as a result of inter-genomic and intra-genomic comparisons in primates [26–29]. These large duplicated segments range in size from 300 kb to 1 Mb, position on at least two different genomic locations and possess more than 90% sequence identity [30]. Comparative data has implicated numerous roles to these SDs, such as creating new genes, expanding gene families and catalyzing large-scale hominoid specific chromosomal reorganization [31].

Conflictingly, carrying out inter-genomic and intra-genomic map comparisons have not proven useful in prediction of evolutionary processes that have arisen in early vertebrate history [32]. The reason lies in the fact that anciently duplicated genomic blocks have undergone events such as sequence variation, multiple chromosomal breakages, gene rearrangement events and modification of karyotype [32].

Phylogenetic investigation of multigene families is considered as the most reliable approach to estimate the existence of ancient intra-genomic synteny blocks or paralogons [16]. Evolutionary mechanisms behind the origin of anciently duplicated regions are captured more adequately by this approach: firstly, by estimating the relative timing of gene duplication events. This startegy can provide a bird’s eye view to all the duplications that happened in a specific time frame. For example, if the phylogenies designate that the bulk of the paralogy regions arose before the split of teleost-tetrapod and after the vertebrate-invertebrate- divergence, this advocates that large-scale gene duplications have occurred between these speciation events [24]. Secondly, the creation of paralogy regions can be scrutinized by combining the information from the global physical structuring of gene families comprising of paralogons with their phylogenetic tree topologies [13]. Distinct but physically linked multigene families (bearing human paralogons) showing coherence among the topologies would suggest that these families might have arisen jointly through segmental duplication events. This approach is elaborated and applied in previous studies [7, 16, 23].

In the earlier studies, various human tetra-paralogons, e.g. Hsa 4/5/8/10 (FGFR-paralogon), Hsa 2/7/12/17 (HOX-paralogon), and Hsa 1/6/9/19 (MHC-paralogon) have been examined to test the legitimacy of 2R hypothesis [4, 7, 14, 17, 23]. In this study, we assess the history of one of the most extensively cited paralogy region, which involves segments of human chromosomes 1, 2, 8 and 20 [14] (Additional files 2, 3 and 4). Taken together with our previous findings, this study estimated the history of 36 multigene families (25 present study and 11 from previous work) with at least threefold distribution on Hsa 1/2/8/20 [14] (Fig. 1; Table 1). In total, our data for this particular human paralogon involves 165 human genes and 2240 protein sequences (Additional file 1) [14]. The topology comparison approach is applied to test the WGD hypothesis (Fig. 2). Hence, the careful analysis resulted in the categorization of 36 phylogenies into four distinct co-duplicated groups, where the component gene families were expanded through duplications that could have happened within the time frame of invertebrate-vertebrate and bony fish-tetrapod- divergence (Additional file 5: Table S1). Distinct gene families within a co-duplicated group could have diversified concurrently by segmental duplications, whereas distinct co-duplicated groups might have been created through discrete duplication events [13]. The retrieval of large co-duplicated groups in this study shows that ancient segmental duplications (aSDs) and rearrangement events played an essential role in modeling the paralogy segments belonging to human chromosomes 1/2/8/20 (Fig. 2). Interestingly, compatible and symmetrical topologies of the type ((AB) (CD)) are gained for the HCK, DLGAP, NKAIN, KCNQ, and MATN gene families (co-duplicated group 3) (Fig. 2c). This pattern is usually measured as an outcome of WGD events [12]. However, here we affirm that sub-chromosomal duplications might be a more balanced clarification for such symmetrical topology trends [6, 7, 14]. For example, tandem duplications occurring in two rounds embracing several unrelated genes would result in a genomic segment with specific paralogous gene-quartets organized in a tandem pattern. Genomic breakage of such larger segments into smaller subsegments via chromosomal deterioration and restructuring could result in paralogy blocks seen in human and other vertebrate genomes [14].

## Conclusion

The present study examined the vertebrate polyploidy proposal by scrutinizing the phylogenomic history of human tetra-paralogon Hsa1/2/8/20. Estimation of gene duplication number with respect to speciation and topology comparison approach revealed no evidence in favor of Ohno’s 2R model. Instead, taken together with previous results from HOX paralogon [16] (63 gene families), FGFR paralogon [4] (80 gene families) and MHC paralogon [23] (40 gene families), the present data (36 families from Hsa 1/2/8/20) suggests that vertebrate genome in its early history was shaped by small-scale events, such as duplication of independent genes, chromosomal segments and rearrangements.

## Methods

### Data collection

Gene families with triplicated or quadruplicated presence on Hsa 1/2/8/20 were recognized by scanning the maps of human genome sequence at Ensembl genome browser [33–35]. A total of 25 gene-families (in total 125 known protein-coding genes) were identified. Among these gene families, 3 families have quadruplicated representation while the 22 families have triplicated presence on Hsa 1/2/8/20 (Fig. 1; Table 1).

The closest putative orthologs of human protein sequences in other animal species were acquired using BLASTP [36] in the Ensembl genome browser [33]. In attempts to obtain sequence data from those organisms still not available at Ensembl, a BLASTP search was carried out against the protein databases available at the National Center for Biotechnology Information [37] and the Joint Genome Institute [http://www.jgi.doe.gov/]. In total, 1605 amino acid sequences from 46 metazoan species were selected for phylogenomic investigation (Additional file 1). Further confirmation of the common ancestry of the putative orthologs was obtained by clustering homologous proteins within phylogenetic trees. The phylogenetic tree topology of each gene family was validated with the detailed comparison against a well established metazoan specie tree [38, 39]. Protein sequences whose placement within a tree was in disagreement with the conventional animal history were removed from the analysis.

The list of sequences used in the analysis (from 46 species including 25 tetrapods, 5 teleost fish, and 16 invertebrates) is provided in Additional file 1. The species that were selected for analysis included *Homo sapiens* (Human), *Mus musculus* (Mouse), *Pan troglodytes* (Chimpanzee), *Gorilla gorilla* (Gorilla), *Callithrix jacchus* (Marmoset), *Pongo abelii* (Orangutan), *Macaca mulatta* (Macaque), *Rattus norvegicus* (Rat), *Oryctolagus cuniculus* (Rabbit), *Taeniopygia guttata* (Zebra finch), *Gallus gallus* (Chicken), *Canis familiaris* (Dog), *Felis catus* (Cat), *Bos taurus* (Cow), *Loxodonta Africana* (Elephant), *Equus caballus* (Horse), *Myotis lucifugus* (Microbat), *Dasypus novemcinctus* (Armadillo), *Pteropus vampyrus* (Megabat), *Ornithorhynchus anatinus* (Platypus), *Monodelphis domestica* (Opossum), *Pelodiscus sinensis* (Chinese softshell turtle), *Anolis carolinensis* (Lizard), *Erinaceus europaeus* (Hedgehog), *Xenopus tropicalis* (Frog), *Danio rerio* (Zebrafish), *Takifug urubripes* (Fugu), *Tetraodon nigroviridis* (Tetraodon), *Gasterosteus aculeatus* (Stickleback), *Oryzias latipes* (Medaka), *Branchiostoma floridae* (Amphioxus), *Ciona intestinalis* (Ascidian), *Ciona savignyi* (Ascidian), *Saccoglossus kowalevskii, Ptychodera flava, Strongylocentrotu spurpuratus* (Sea urchin), *Caenorhabditis elegans* (Nematode), *Anopheles gambiae* (Mosquito), *Drosophila melanogaster* (Fruit fly), *Apis mellifera* (Honey bee), *Capitella teleta* (Capitella), *Octopus bimaculoides* (Octopus), *Hydra magnipapillata* (Hydra) and *Nematostella vectensis* (Sea anemone), *Trichoplax adhaerens* (Trichoplax), and *Amphimedon queenslandica* (Sponge).

### Alignment and phylogenetic analysis

Phylogenetic analysis for each gene family was performed using MEGA version 5 [40]. Multiple sequence alignment program CLUSTALW [41] was used to align the protein sequences. Alignment quality has much impact on accurate inference of phylogeny. Homologous protein sequences often evolve under different evolutionary pressure in some regions of protein in different species [42–44]. Furthermore, regional rate heterogeneity affect the whole alignment and ultimaley phylogenetic reconstructuction [44, 45]. Therefore, multiple sequence alignment of each gene family was trimmed to eliminate all of positions containing gaps and missing data. Only unambiguous portions of sequence alignments are used for phylogenetic analyses. Phylogenetic analyses were performed using Neighbor-Joining (NJ) approach [46–48]. The JTT (Jones-Taylor-Thornton) matrix-based method and uncorrected proportion (*p*) of amino acid differences were employed as amino acid substitution models. Results obtained with both the methods are given in Additional files 2 and 3.The authenticity of clustering patterns in resulting trees was evaluated by bootstrap method (1000 pseudo-replicates) [49], which produced the bootstrap probability values for each interior branch in the phylogenetic tree. Each of the phylogenetic tree reconstruction methods has its own limitation, therefore, to systematically check and validate NJ based trees, Maximum Likelihood (ML) based phylogenies are also constructed using Whelan and Goldman (WAG) model of amino acid replacement [50]. The phylogenetic trees with the highest log likelihood scores are selected as final trees. Initial tree(s) for ML were generated automatically by applying NJ and BioNJ methods to a matrix of pairwise distances calculated using JTT model, and then selecting a toplogy with superior loglikelihood value [47, 51]. Heuristic searches starting with the initial trees were conducted with Nearest Neighbor Interchange [NNI] [40]. The topological reliability of each ML tree was evaluated by bootstrap method on the basis of 1000 pseudoreplicates [49]. The ML based trees are provided in Additional file 4.

The gene duplications relative to the divergence of major animal taxa were estimated by investigating the branching order of phylogenetic trees [4, 13, 18]. The phylogenetic topology of each family was compared with that of all other families to assess the consistencies in gene duplication events [16]. Gene families with consistent tree topologies are placed in respective co-duplicated groups [13].

Among the tree topologies of 25 gene families, the phylogenies of five families (*MYT*, *NCOA*, *STMN*, *NKAIN* and *YTHDF*) were rooted with invertebrate sequences, whereas *CRO*, *ID*, *MROH*, *RSPO*, *FAM110*, *TCEA*, *RIMS*, *KCNQ* and *CHRN* families were rooted with both invertebrate and vertebrate sequences. In case of *UBXN* and *E2F* families the vertebrate sequences served as outgroup. The phylogenies of *SNX*, *RGS*, *GRHL*, *AZIN*, *DLGAP*, *STK*, *SLC*, *SNT*, and *XKR* families contained two sub families, each of them served to root the other.

## Additional files

Additional file 1: Complete list of protein sequences used in this study (PDF 1724 kb)

Additional file 2: Neighbor Joining Trees of gene families (residing on human chromosomes 1/2/8/20) using p-distance method. (PDF 4993 kb)

Additional file 3: Neighbor Joining Trees of gene families (residing on human chromosomes 1/2/8/20) using JTT method. (PDF 3402 kb)

Additional file 4: Maximum likelihood Trees of gene families (residing on human chromosomes 1/2/8/20) based on WAG model. (PDF 3836 kb)

Additional file 5: **Table S1.** Summary of the Phylogenetic analysis of gene families with three or more members are residing on human chromosomes 1/2/8/20. (PDF 73 kb)

## Data Availability

The datasets analyzed during the current study are available in the Ensembl database (http://www.ensembl.org), NCBI database (https://www.ncbi.nlm.nih.gov/) and as supplementary information.

## Abbreviations

aSDs: Ancient segmental duplications
Hsa: *Homo sapiens* autosomes
JTT: Jones-Taylor-Thornton
ML: Maximum Likelihood
NJ: Neighbor-Joining
SDs: Segmental duplications
WAG: Whelan and Goldman
WGDs: Whole genome duplications
