# Supplementary material for: Evolutionary history of the human multigene families reveals widespread gene duplications throughout the history of animals
Source: BMC Evol Biol. 2019 Jun 20;19:128. doi: 10.1186/s12862-019-1441-0 (PMC6585022; doi:10.1186/s12862-019-1441-0)
Supplement: Supplementary file 1 — Complete list of protein sequences used in this study (PDF 1724 kb) [file 12862_2019_1441_MOESM1_ESM.pdf]

Additional file 1: Complete list of protein sequences used in this study

Antizyme Inhibitor-AZIN

```
>Homo sapiens_AZIN1(ENSP_321507)
MKGFIDDANYSVGLLDEGTNLGNVIDNYVYEHTLTGKNAFFVGDLGKIVKKHSQWQNVVA
QIKPFYTVKCNSAPAVLEILAALGTGFACSSKNEMALVQELGVPPENIIYISPCKQVSQI
KYAAKVGVNILTCDNEIELKKIARNHPNAKVLLHIATEDNIGGEEGNMKFGTTLKNCRHL
LECAKELDVQIIGVKFHVSSACKESQVYVHALSDARCVFDMAGEIGFTMNMLDIGGGFTG
TEFQLEEVNHVISPLLDIYFPEGSGVKIISEPGSYYVSSAFTLAVNIIAKKVVENDKFPS
GVEKTGSDEPAFMYYMNDGVYGSFASKLSEDLNTIPEVHKKYKEDEPLFTSSLWGPSCDE
LDQIVESCLLPELNVGDWLIFDNMGADSFHEPSAFNDFQRPAIYYMMSFSDWYEMQDAGI
TSDSMMKNFFFVPSCIQLSQEDSFSAEA

>Macaca mulatta_AZIN1(ENSMMUP_1788)
MKGFIDDANYSVGLLDEGTNLGNVIDNYVYEHTLTGKNAFFVGDLGKIVKKHSQWQNVVA
QIKPFYTVKCNSAPAVLEILAALGTGFACSSKNEMALVQELGVSPENIIYISPCKQVSQI
KYAAKVGVNIMTCDNEIELKKIARNHPNAKVLLHIATEDNIGGEEGNMKFGTTLKNCKHL
LECAKELDVQIIGVKFHVSSACKESQVYVHALSDARCVFDMAGEIGFTMNMLDIGGGFTG
TEFQLEEVNHVISPLLDIYFPEGSGVKIISEPGSYYVSSAFTLAVNIIAKKVVENDKFPS
GVEKTGSDEPAFMYYMNDGVYGSFASKLSEDLNTIPEVHKKYKEDEPLFTSSLWGPSCDE
LDQIVESCLLPELNVGDWLIFDNMGADSFHEPSAFNDFQRPAIYYMMSFSDWYEMQDAGI
TSDSMMKNFFFVPSCIQLSQEDSFSAEA

>Callithrix jacchus_AZIN1(ENSCJAP_42044)
MKGFIDDANYSVGLLDEGTNLGNVIDNYVYEHTLTGKKAFFVGDLGKIVKKHSQWQNVVA
QIKPFYTVKCNSAPAVLEILAALGTGFACSSKNEMALVQELGVSPENIIYISPCKQVSQI
KYAAKVGVNIMTCDNEIELKKIARNHPNAKVLLHIATEDNIGGEEGNMKFGTTLKNCRHL
LECAKELDVQIIGVKFHVSSACKESQVYVHALSDARCVFDMAGEIGFTMNMLDIGGGFTG
TEFQLEEVNHVISPLLDIYFPEGSGVKIISEPGSYYVSSAFTLAVNIIAKKVVENDKFSS
GVEKTGSDEPAFMYYVNDGVYGSFASKLSEDLNAIPEVHKKYKEDEPLFTSSLWGPSCDE
LDQIVESCLLPELNVGDWLIFDNMGADSFHEPSAFNDFQRPAIYYMMSFSDWYEMQDAGI
TSDAMMKNFFFVPSCIQLTQEDSFSTEA

>Mus musculus_AZIN1(ENSMUSP_105958)
MKGFIDDANYSVGLLDEGTNLGNVIDNYVYEHTLTGKNAFFVGDLGKIVKKHSQWQTVVA
QIKPFYTVKCNSTPAVLEILAALGTGFACSSKNEMALVQELGVSPENIIFTSPCKQVSQI
KYAAKVGVNIMTCDNEIELKKIARNHPNAKVLLHIATEDNIGGEDGNMKFGTTLKNCRHL
LECAKELDVQIIGVKFHVSSACKEYQVYVHALSDARCVFDMAGEFGFTMNMLDIGGGFTG
TEIQLEEVNHVISPLLDIYFPEGSGIQIISEPGSYYVSSAFTLAVNIIAKKVVENDKFSS
GVEKNGSDEPAFVYYMNDGVYGSFASKLSEDLNTIPEVHKKYKEDEPLFTSSLWGPSCDE
LDQIVESCLLPELNVGDWLIFDNMGADSFHEPSAFNDFQRPAIYFMMSFSDWYEMQDAGI
TSDAMMKNFFFAPSCIQLSQEDSFSTEA

>Rattus norvegicus_AZIN1(ENSRNOP_69785)
MKGFIDDANYSVGLLDEGTNLGNVIDNYVYEHTLTGKNAFFVGDLGKIVKKHSQWQNVVA
QIKPFYMVKCNSTPAVLEILAALGTGFACSSKNEMALVQELGVSPENIIYTSPCKQASQI
KYAAKVGVNIMTCDNEVELKKIARNHPNAKVLLHIATEDNIGGEDGNMKFGTTLKNCRHL
LECAKELDVQIIGVKFHISSACKEYQVYVHALSDARCVFDMAGEFGFTMNMLDIGGGFTG
TEIQLEEVNHVISPLLDIYFPEGSGIQIISEPGSYYVSSAFTLAVNIIAKKVVENDKLSS
GVEKNGSDEPAFVYYMNDGVYGSFASKLSEDLNTVPEVHKKYKEDEPLFTSSLWGPSCDE
LDQIVESCLLPELSVGDWLIFDNMGADSLHGPSAFSDTQRPAIYFMMSLSDWYEMQDAGI
TSDAMMKNFFFAPSCIQLSQEDNFSTEA
```

>Canis familiaris_AZIN1(ENSCAFP_907)
MKGFLDDANYSVGLLDEGTNLGNVIDNYVYEHTLTGKNAFFVGDLGKIVKKHSQWQNIVA
QIKPFYTVKCNSTPAVLEILAALGTGFACSSKTEMALVQELGVSPENIIYISPCKQVSQI
KYAAKIGVNTMTCDNEVELKKIARNHPNAKVLLHIATEDNIGGEEGNMKFGTTLKNCRHL
LECAKELDVQIIGVKFHVSSACKESQVYVHALSDARCVFDMAGEFGFTMNMLDIGGGFTG
TEFQLEEVNHVISPLLDVYFPEGSGIKIISEPGSYYVSSAFTLAVNIIAKKVVENDKFPS
GVEKTGSDEPAFMYYMNDGIYGSFASKLSEDLNTIPEVHKKYKKDEPLFTSSLWGPSCDE
LDQIVESCLLPELNVGDWLIFDNMGADSLHEPSAFNDFQRPAIYYMMSFSDWYEMQDAGL
TSDTMMKNFFFVPCIQLSQEDSFSSEA

>Felis catus_AZIN1(ENSFCAP_9312)
MKGFIDDANYSVGLLDEGTNLGNVIDNYVYEHTLTGKNAFFVGDLGKIVKKHSQWQNVVA
QIKPFYTVKCNSTPAVLEILAALGTGFACSSKTEMALVQELGVSPENIIYISPCKQVSQI
KYAAKIGVNIMTCDNEVELKKIARNHPNAKVLLHIATEDNIGGEEGNMKFGTTLKNCRHL
LECAKELDVQIIGVKFHVSSTCKESQVYVHALSDARCVFDMAGEFGFTMNMLDIGGGFTG
SEFQLEEVNHVISPLLDVYFPEGSGIKIISEPGSYYVSSAFTLAVNIIAKKVVENDKFSS
GVEKTGSDEPAFMYYMNDGVYGSFASKLSEDLNTIPEVHKKYKEDEPLFTSSLWGPSCDE
LDQIVENCLLPELNVGDWLIFDNMGADSLHEPSAFNDFQRPAIYYMMSFNDWYEMQDAGI
TSDTMMKNFFFVPSCIQLSQEDGFSSEA

>Myotis lucifugus_AZIN1(ENSMLUP_1554)
MKGFIDDANYFVSLLDEGTNLGDVIDNYVNEHTLTGKNAFFVGDLGKIVKKHSQWQNVVA
QIKPFYTVKCNSTPAVLEILAALGTGFACSSKTEMALVQELGVSPENIIYISPCKQVSQI
KYAAKVGVNIMTCDSEVELKKIARNHPNAKVLLHIATEDNTGDEEGNMKFGATLKNCRHI
LECAKELDVQIIGVKFHVSSACKESQVYVHALSDARCVFDMAGEFGFTMNILDIGGGFTG
TEFQLEEVNHVISPLLDVYFPEGSGIKIIAEPGSYYVSSAFTLAVNIIAKEVVESDKFSS
GVEKTGSDEPAFVYYMNDGVYGSFASKLSEDLNTIPEVHKKYKDDEPLFTSSLWGPSCDE
LDHIVEKCLLPELNVGDWLIFDNMGADSLHEPSAFNDRQRPAIYYMMSFSDWYEMQDAGI
TSDTMKNFFFVPSCIQLSQEDSFSTEA

>Monodelphis domestica_AZIN1(ENSMODP_7263)
MKGFIEDANYSVGLLDEGTHLGNVIDNYVYEHTLTGKKAFFVGDLGKIVKKHSQWQNVVA
QIKPFYTVKCNSAPAVLEILAALGTGFACSSKNEMALVQELGVSPENIIDISPCKQSSQI
KYAAKTGVNIMSCDNEIELKKIARNHPNAKLLLHIATEDISGDEESNMKFGTTLKNCRHL
LECAKELDVQIVGVKFHVSSSCKEPQVYIHALSDARCVFDMAGEFGFKMTILDIGGGFTG
SEFQLQEVNHVISPLLDVYFPEGSGIKVISEPGSYYVSSAFTLAVNIIAKKVVENDVVSS
GVGKTGSNEPAFMYYMNDGVYGSFASKLSENLNTIPEVHKKYKKDAPLFTSSLWGPSCDE
LDQIVENCLLPELNVGDWLIFDNMGADTLHEPSAFNDFQRPTIYYMMSFSDWYEMQDAGI
TSDTMMKNFFFVPSCIQLSQDDSSFSTEA

>Gallus gallus_AZIN1(ENSGALP_35817)
MKGFLEDANYSIGLLDEGATLADVIDNCIYEHTHTGKRAFYVGDLGKLVKKNIQWQNVMA
PIKPFYTVRCNSTPGVLEILGTLGIGFACSSKSEMALVQGLGISPENIIYTNPCKQASQI
KYAAKAGINIMTCDSDIELKKIARNHPNAKLLLHIATEDITADEEMNMKFGTTLKNCRHL
MECAKEFGVQIVGVKFHVSGSCKELQTYIHAISDARCVFDMAEEFGFKMNMLDIGGGFTG
SELQLEEVNHVIRPLLDVYFPKESGIQVIAEPGCYYVSSAFTLAVNVIAKKAVDCDKLLP
SGVEQARNDDEPVFTYYINDGVYGSFASKLSEKLYTNPEVHKKYKEDEPLFASSLWGPSC
DELDQIVENCLLPELSVGDWLIFDNMGSGTLGEQSTFNDYQRPLIYYMMSFSDWDEMQDA
GIASDTLMKNFFFVPSCIQLSPEDRFSTAA

>Anolis carolinensis_AZIN1(ENSACAP_9060)
MKGFLEDANYSIGLLDEGVNVAEVIDNCIYEHTINGKNGFFVGDLGKLIKHHIHWQNVMA
PIKPFYNVKCNSTPGVLEILAALGTGFACSSKNEMALVQDLGISSENILFSNPCKQASQI
KYAAKTGVNVLTCDNELELKKIARNHPNAKLLLHIATEDINGGEEMNMMFGTTLKNCRHL
MECAKELNVQIIGVKFNVSNSCKEPQVYKHAISDARCVFDMAEEFGFTMNMLNIGSGFTD
YEFDLEKINHVVSPLLDIYFPEESGVNVIAEAGCYYVSSAFTLAVNIIAKKAVQSTEPLL
FKTEQTQGDDQVFMYYINDGVYGSFASKLSDNLTTIPEVHKKYKEDEPLFASSLWGPSCD
ELDQIVENCLLPELNVGDWLLFDNMGSGTLGEQSAFNDFQRPPVYYIMSISNWCEMQDAG
FTCETLMKNFFFVPSCIQLSPEDGFSLAA

>Xenopus tropicalis_AZIN1(ENSXETP_8894)
MKGFIEDTNYSIGLLDDTATPRDVIDNYIYEHTLMGKNAFFVADLGKIVKKHFKWKNIMG
RVNPFYTVRCNSSPAVLEILAALSMGFACANKNEMSLVYDLGIPMENAVYTNPCKQASQI
KHAAKIGVNLMTCDSETELKKIARNHFNAKLLLHIATEGISGEEDMNMTFGTTLKNCRHL
LECAKELSVEVVGVKFHVSSSNNPQTYIHALSDARCVFDMAEELGFKMNILDIGGISENE
AQLEELYQAVSPLLDVYFPEGSGTRIIAEPGSFYVSSAFTLAVNVMAKNVIEHDQHLPSA
GKQISNKPAFIYCMNEGVYGSFARKLSEKLNTAPEVHKKYKDDEPLFASSLLGPSYDELD
VIVEHCLLPELEVGDWVVFENMGCGSLNEQSPFADFEKPSLYNFMSFSDWYEIQDTGNTS
ESLMKSLCVPCFHLGEDQFCTV

>Danio rerio_AZIN1a(ENSDARP_122392)
MKGFVDAPNYFIELLEGGTTLENVIDNHIYEQNLAEKNAFVVADLGALMRQHVVWKTTMP
LVRPFYPVSCNSSPVVIELLSALGVGFVCANKAETTLVLSHDVPPENIILSGVCKQLSLI
KHAAKTGINFLLCENEAELRKIARAHPEAKLLLQVSTEAQIEEASVTIGCSLKGCRHLLE
LAKELNVSVAGVTFQVPASCKDPQAYTHALSDARCIFDMGKELGFDMNILDIGGGFSGSE
FQLKQVHSVIRPLLEAYFPSESGVSIIAEPGNFFVFSCFTLAVNVIGKKAAYRNLHTSTH
DKLTSSDAPEFVYYMNDGVYGSFMGRLFGNIISTPSVHKMSLTLDEPVFSSSLWGPSCDP
LDQVVERCLLPELAVGDWLVFSNMGASGQEGPATFSDTDQPPVYYTISTDDWFEMQEAGI
SLDDTMKNLSLVQFGL

>Danio rerio_AZIN1b(ENSDARP_69192)
MSMKGSLDELHYSVELLEGSAALRDVIDKHIYDQTFAQKSAFFAADLGVIVQQQARWRNK
MDQIRPFYTVKSNSSPVVVELLASLGTGFICSNKHELDLVKGFGVSSQDIILGGTCKQLS
HIKYAAKHNIPLLVCDNEAEMRKIARCHPKAKVLLLLTSESCCEKEEMAIPFGSTLKDCR
RLLECAAGELNLQVTGVKFNIPSCCQEVEKFSRAVSEARCVFDMGEELGFEMNILDIGGG
FDGSEEQLDKVNQMLKPMLDMYFPRSTGLSIIAEPGAYYVSSAFTLAVNIIDKKTVARDC
SAQPHDALSANDEPEFLYHINDGVYGSFASKLLCEDSIATPLAHKHVSADEPLFSSSLWG
PSEDDLDLVMERCLLPELSVGDWLLFRNAGASALGSAFSHGDQHKPPVFYSITEYDWQEI
HNSGVTVDVRMKNFSLVPCCLQPNMSGASISTPA

>Takifugu rubripes_AZIN1(ENSTRUP_25228)
MKELTNELQHSVSLLEGNASLCDVIDNHIYEQTLAGKNAFFIADLGVVMRQHVRWRTHMA
QIRPYYSVRCNSRPTVIDILAALGTGFICTNKTELELVQSRGVPSEDIIFSGVSKQLSQL
KYAAKNGIDLLVCDNEVELCKISRCHPSAKLLLQVSTEAFCQDNDLVMTFGCSLKDCRHL
LERAKELGVQVVGVRFNISSSCEDPQLYAHAMSDARCVFDMGEEIGFNMNILDIGGGFNG
CETQLELVNNTVMSMVDLYFPPSSGVCIIAEPGSFFVSSAFTLAVNVISKEVVARDSHDQ
AHDEPSPNDEPAFQYFMKEGVYGSFASKLCETQIVAPSVHKSSCLDAALFSSSLWGPSGD
DMDQVVEHCLLPELNIGDWLIFSQAGAYSLGQPLCSTDDSPPPHVCYVMSSADWFEMQDI
GITYEGTLKNMALVPYFLDSCQTEAALSVPA

>Tetraodon nigroviridis_AZIN1a(ENSTNIP_12033)
MKGLADKPSYTIELLEGGVTLEDVIDGHICEQTLVDKTAFVVGDLGALMRQHVRWQSISP
RLQPYFPVKCNSSPAVIEVMASLGLGFVCVNKAEMSLVLEHGVPPEAVILSGVCKQQAHI
KYAAKNNIQNLVCENEAELFKISRLHPNAKLLLQLSTQAHAAETSMTFGFSLKSSRHLLE
AAKQLGLQVVGVSFHIPSSCQDLQEAYTHALSDARCVFDMGVDLGFNMNVLDIGSGFTGS
EFQLRQVESALKPLLDTYFSPLSGVQVLAQPGNFYVASAFCLAVTIMGKKLVNPRWDNLA
QGEDNEGTEFLYYMNEGIYGPFSCKLLGNSISAPSVHKHALCAEEAMYPSSLWGPSLDQL
DQVVERCLLPELSVGDWLLFPNMGACGLEDFGSLQLPVYYTVSTSDWFEMQEAGVALDNA
VKNFSMVQCSA

>Tetraodon nigroviridis_AZIN1b(ENSTNIP_12033)
MKGLADKPSYTIELLEGGVTLEDVIDGHICEQTLVDKTAFVVGDLGALMRQHVRWQSISP
RLQPYFPVKCNSSPAVIEVMASLGLGFVCVNKAEMSLVLEHGVPPEAVILSGVCKQQAHI
KYAAKNNIQNLVCENEAELFKISRLHPNAKLLLQLSTQAHAAETSMTFGFSLKSSRHLLE
AAKQLGLQVVGVSFHIPSSCQDLQEAYTHALSDARCVFDMGVDLGFNMNVLDIGSGFTGS
EFQLRQVESALKPLLDTYFSPLSGVQVLAQPGNFYVASAFCLAVTIMGKKLVNPRWDNLA
QGEDNEGTEFLYYMNEGIYGPFSCKLLGNSISAPSVHKHALCAEEAMYPSSLWGPSLDQL
DQVVERCLLPELSVGDWLLFPNMGACGLEDFGSLQLPVYYTVSTSDWFEMQEAGVALDNA
VKNFSMVQCSA

>Gasterosteus aculeatus_AZIN1(ENSGACP_6108)

MKGIADEPQYSVGLLEAGTALCDVINQHIDEQTLSEKNAFFVADLGVVMRQHVRWRTHMP
QIRPYYPVRCNSSPAVIEVLAVLGAGFICTNKSELELVQSHGVPSEDIIYSGICKQVSQI
KYAAKNGIDLLVCDNEAELRKISRCHPNAKLLLQVSTEASIQGDEMSMAFGCSLKDCRHL
LEKAKELGVQVVGVRCHISSACEDDQVYVHAISDARCVFDMGEDIGFDMTILDIGGGFSG
SENQLEVINKAVMSMADLYFPPSTGVSIIGEPGSFFVSSAFTLVVNVISKEVVARDRQDQ
ARDDPSPNDEPEFQYHLSEGVYGPFASKLSETLIATPSVYKPPQDAPLFSCSLWGPSGDD
LDQVVEHCLLPELNAGDWLLFTHAGAYSTGHPLCTATDSPPPPEYYIISSTDWFEMQDIG
VAHEATLKNFSLVPYFLAYCQTAAGRSVPA

>Oryzias latipes_AZIN1a(ENSORLP_3831)
MKGIADEPQYSVGLLDGGTALCDVIDNYIDEQTVSEKNAFFVADLGAVMRQHVRWRTRMP
LIRPYYAVRCNSSPAVIELLASLGSGFTCSNKSDLELVLSHGVSPEDIIYSGVCKQVSHI
KYAATNGIDLLVCDNEAELRKISRCHPGAKLLLQVSTEASNPEEGTSVTFGCSLKDSRCL
LQRAKELGVQVVGVRSNISSSCEDDQVYLRAISDARCIYDLGAEMGFNVNILDIGGGFNG
AEAQLDLIHSAVMSVVDLYFPSSSGVSLIAEPGSFFVSSAFTLAVSVISKKVVARDAKDQ
EREEPSPDEEPEIQYVLNEGVYGSFSSKLSETPIGAPSVHKKTLVDAPLFSSSLWGPSGD
DLDQVVEHCLLPELHIGDWLVFKHAGAFSLGQPLLASTDSPPPPIFYFIFSRDWFEMQDT
GVMHDTTLKNLSLVPYFFNSSQTGAALSVPA

>Oryzias latipes_AZIN1b(ENSORLP_15618)
MKGFADKPNYIIELLDGSVTLEDVIDGHICDQTVMEKSAFVVGDLGALMQQHACWQSVVP
RVLPYYPVKCNSSPVVIEVLASLGLGFVCANKAEVGLVLEHGVPPENIILSSGFKQLAHI
KYAAKNNIQHLVCENEAELSKIARLHPSAKLLLQLTTESQAAENSMAFGFSLKNCRHLLE
KAKDLGIDVVGAAFHIPSSCEDLQPAYTHALSDARCVFDMGLDLGFNMHILDIGGGFTGS
EFQLKQVESAINTLLDAYFPQLSGVQVLAQPGNFYVASAFSLAVNIIGKKIKTQSWNCLS
QGINSGEKKEGTEFLYYMNEGVYGPFSHKLLGHPIAAPSLHKHAFCADEAVYPSSLWGPS
LDQLDQVVEHCLLPELSVADWLIFSNMGTCGLEDLNCLSSSIDFPVYYIISTSDWYKMEE
AGVALNNPVKNFSMLQYSA

>Homo sapiens_AZIN2(ENSP_362542)
MAGYLSESDFVMVEEGFSTRDLLKELTLGASQATTDEVAAFFVADLGAIVRKHFCFLKCL
PRVRPFYAVKCNSSPGVLKVLAQLGLGFSCANKAEMELVQHIGIPASKIICANPCKQIAQ
IKYAAKHGIQLLSFDNEMELAKVVKSHPSAKMVLCIATDDSHSLSCLSLKFGVSLKSCRH
LLENAKKHHVEVVGVSFHIGSGCPDPQAYAQSIADARLVFEMGTELGHKMHVLDLGGGFP
GTEGAKVRFEEIASVINSALDLYFPEGCGVDIFAELGRYYVTSAFTVAVSIIAKKEVLLD
QPGREEENGSTSKTIVYHLDEGVYGIFNSVLFDNICPTPILQKKPSTEQPLYSSSLWGPA
VDGCDCVAEGLWLPQLHVGDWLVFDNMGAYTVGMGSPFWGTQACHITYAMSRVAWEALRR
QLMAAEQEDDVEGVCKPLSCGWEITDTLCVGPVFTPASIM

>Gorilla gorilla_AZIN2(ENSGGOP_14531)
MAGYLSESDFVMVEEGFSTRDLLKELTLGASQATTDEVAAFFVADLGAIVRKHFCFLKCL
PRVRPFYAVKCNSSPGVLKVLAQLGLGFSCANKAEMELVQHIGIPASKIICANPCKQIAQ
IKYAAKHGIQLLSFDNEMELAKVVKSHPSAKMVLCIATDDSHSLSRLSLKFGVSLKSCRH
LLENAKKNHAEVVGVSFHIGSGCPDPQAYAQSIADARLVFEMGTELGHKMHVLDLGGGFP
GTEGAKVRFEEIASMINSALDLYFPEGCGVDIFAELGRYYVTSAFTVAVSIIAKKEVLLD
QPGREAPLPPPHIATCAACEPSPPAEENGSTSKTIVYHLDEGVYGIFNSVLFDNICPTPI
LQKKPSTEQPLYSSSLWGPAVDGCDCVAEGLWLPQLHVGDWLVFDNMGAYTVGMVSPFGE
TQACHITYAMSRVAWEALRRQLMAAEQEDDVEGVCKPLSCSWEITDTLCVGPVFTPASIM

>Pongo abelii_AZIN2(ENSPPYP_1819)
MAGYLSESDFVMVEEGFSTRDLLKELTLGASQATTDQVAAFFVADLGAIVRKHFCFLKCL
PRVRPFYAVKCNSSPGVLKVLAQLGLGFSCANKAEMELVQHIGVPASKIICANPCKQIAQ
IKYAAKHGIRLLSFDNEMELAKVVKSHPSAKMVLCIATDDSHSLSRLSLKFGVSLKSCRH
LLENAKKSHVEVVGVSFHIGSGCPDPQAYAQSIADARLVFEMGTELGHKMHILDLGGGFP
GTEGTKVRFEEIASMINSALDLYFPEGCGVDIFAELGRYYVTSAFTVAVSITAKKEVLLD
QPGREEENGSTRKTIVYHLDEGVYGIFNSVLFDNICPIPILQKKPSTEQPLYSSSLWGPA
VDGCDCVAEGLWLPQLHVGDWLVFDNMGAYTVGMGPPKGGTQACHITYAMSRVAWEALGS
S

>Mus musculus_AZIN2(ENSMUSP_30581)
MAGYLSESDFVMVEEGFSTRDLLEELTLGASQATSGKVAAFFVADLGAVVRKHFCFLKHL

PRVRPFYAVGCNSSLGVLKVLAELGLGFSCANKAEMELVQHIGVPASKIICANPCKQVAQ
IKYAAKHGVRLLSFDNEVELAKVVKSHPSAKMVLCIATQDSHSLNHLSLRFGASLKSCRH
LLENAKKSHVEVVGVSFHIGSGCPDPQAYAQSIADARLVFQMGEELGHTMNILDLGGGFP
GLEGAKVRFEEMASVINSALDLYFPEGCGVDILAELGRYYVTSAFTVAVSIVAKREVLDQ
ASREEQTGAAPKSIVYYLDEGVYGVFNSVLFDNTCPTPALQKKPSADQPLYSSSLWGPAV
EGCDCVAEGLWLPQLQVGDWLVFDNMGAYTVDTKSLLGGTQARRVTYAMSRLAWEALRGQ
LLPAEEDQDAEGVCKPLSCGWEITDTLCVGPVFTPASIM

>Rattus norvegicus_AZIN2(ENSRNOP_72209)
MAGYLSESDFVMVEEGFSTRDLLEELTLGASQATTGKVAAFFVADAVVRKHFCFLKYLPR
VRPFYAVRCNSSLGVLKVLAELGLGFSCASKAEMELVQHIGVPASKIICANPCKQVAQIK
YAAKHGVRLLSFDNEVELAKVVKSHPSAKMVLCIATQDSHSLNHLSLRLRASLKSCRHLL
ENAKQSHVEVVGVSFHIGSGCPDPQAYAQSIADARLVFQMGAELGHTMNILDLGGGFPGL
EGAKVRFEEMASVINSALDLYFPEGCCVDILAELGRYYVTSAFTVAVSIVAKKEVLTSRE
EQTGAAPKSIVYHLDEGVYGVFNSVLFDNTCPTPALQKKPSADQPLYSSSLWGPAVDGCD
CVAEGLWLPQLQVGDWLVFDNMGAYTVDTKSLLGGKPFKGSCCLQKKTRTPRVCANLCPA
AGRSQTACVWALSSPQQASCEWACFPRESQRGQGDASGEVGEAASWYPLARTLVLALPPH
APPVVFLPCK

>Canis familiaris_AZIN2(ENSCAFP_15252)
MAGYLSETDFVMVEEGFSTRDLLEELTLGASQATAGDVAAFFVADLGAVVRKHFCLLKCL
PRVRPFYTVKCNSSPGVLKVLAELGLGFSCANKAEMELVQRIGVPASKIVYANPCKQIAQ
IKYAAKHGVRLLSFDNEVELAKVVKSHPSAKMVLCIATDDSHSLSHLSFKFGASLKSCRH
LLENAKRSHVEVVGVSFHVGSGCPDLHAYTQSIADARLVFEMGAELGHRMHILDLGGGFP
GLEGAKVRFEEIATVINSALDLYFPEGCGVDILARLGRYYVTSAFTLAVSIIAKKEVLLD
QPGREEETGSTPKTIVYHLGEGVYGIFNSVLFDNTCPTPILQKKSSTEQPLYSSSLWGPV
ADGSDCVAEGLWLPQLHVGDWLVFENMGAYTVGMGSLFGGTQTCRVTYAMSRVAWEALRG
QLLPAEQDEDTEGMCKPLSCGWEITDTLCVGPVFTPASIM

>Felis catus_AZIN2(ENSFCAP_24006)
MAGYLSETDFVMVEEGFSTRDLLEELTLGASQATTGDVAAFFVADLGAVVRRHFCFLKCL
PRVRPFYTVKCNSSPGVLKVLAELGLGFSCANKAEMELVQRIGIPAGKIIYANPCKQIAQ
IKYAAKHGVRLLSFDNELELAKVVKSHPSAKMVLHIAAEDSPSRLSFKFGASLKSCRHLL
ENAKRSHMEVVGVSFHVGTGCPDLQAYTQSIADARLVFEMGTELGHRMHILDLGGGFPGL
EGAKVRLEEICSVINSALDLYFPEGCGVDILAELGRYYVTSAFTLAVSIIAKKEVLLDQP
GREEESGSTPKTIVYHLGEGVYGIFNSVLFDNTCPTPILQKKSSMEQPLYSSSLWGPVVD
GCDCIAEGLWLPQLHVGDWLVFENMGAYTVGMGSLIEGAQTCRVTYAMSRVAWEALRGQL
LPAEQDEDTEGMCKPLSCGWEITDTLCVGPVFTPASIM

>Myotis lucifugus_AZIN2(ENSMLUP_729)
MAGYLSETDFVMVEEGFSTRDLLEELTMGASQATTKSKAALFCLALSGCLPRHHLLCFLG
KLRCLPMPPSWFLVSSGCGPGSAHLVISISLGTCLWKAEMELVQRIGVPASKIIFANPCK
QIAQIKYAAKYGIQLLSFDNEMELAKVVKNHSSAKLLLCIATDDSHSQSGLSLKFGALLD
SCRHLLQNAKKSHVEVVGVSFHVGSCCPDPEAYAQFIADARIVFEMGAELGHRMHILDLG
GGFPGVEGAKVRFEEIASVINSALDLYFPEGCGVDIFAKLGRYYVTSAFTLAVSIIAKKE
VLLDQPGREEETGSAPKTIVYQLDEGIYGIFNSVLFDNTCPTPVLQKKPSTEQPLYSSSL
WGPAADGCDCVAEGLWLPQLHIGDWLVFENMGAYTVGMGSLFRGIQTCRITYAMSRVAWK
ALREQPLPAEQDEDAEGMCKPLSCGWEITDTLCVGPVFTPASIICVRLLCSAKGLVSQIS
PQASVPGLLGRVSCQVKVGSELLPF

>Monodelphis domestica_AZIN2(ENSMODP_21660)
MLRPQGRAGAAGGAEAPVSDSLAGETYGQKPCKCVLRGLSSPAAATPRGVAQVLSSPASP
PSIAPGSAPPQGGRGSDEGTSFFVADLGDVVKKHLRFLKNLPRVRPFYVVKCNSSPGVLR
ALAELGLGFSCANKAEMELLRHIGVPAHKIIYTSPCKQAAQVKQAARLGIRMLSFDNEVE
LAKVARSHPTARMVLRIATDDSCSPARLSMKLGATLKSCRHLLETAKKMNMEVVGVSFHV
GSGCSSDPQAFTQSIADARLVFEMGTELGYRMHILDLGGGFPGTEDSKVRFEEMASVINS
ALDMYFPEGCGVDILAEPGRYYVASAFTLAVRIVAKKEVPSDQTGLDEEDGASPKSIVYH
ISEGVYGAFNCVFFDNACPMPILQKKPSPDQRVYSSSLWGPTCDDLDHVAEGLCLPELQL
GDWLIFENMGAYTVGTTASCSGQQTCQTTYAMSRVTWEAVRGRLLPPEEDTEDMCKPLSC
GWEIADTLCVGPVFAPTSII

>Gallus gallus_AZIN2(ENSGALP_5708)
MNGYLNESNFMMVEEGFTSRDLLESTLVELCRTGDQQAFFVADLGDIVKKHLHFLKALPR
VKPYFPVKCNSSGGVIRLLAELGAGFACANKTEIAEVQSIGVPADKIFYSSPCKQIAHIK
YAASHGVQLMTFDNEVELSKVARSHPQARMLLGIAADSSPSAHPSMTFGATLKSCRCLLE
SAKEQDVEVVGISFHLGRHSLEPQVFAQAVAEAQLAFDMGAELGYRMHLLDIGGGFPGTE
DTRAQFEEIAAVINSALDMYFPEGCGVEIIATPGRYYVTSAFTFAASITSKEDVPVEQPG
SDGEESGSKRSFVYHLSNGIYGTFSCVLFDGPCPKPLLHKKPCSEQLLCSSSLRGPLGHA
EDQITDDVELPELQDGDWLIFQDMGAYSIATSSLCGGCPQPHVTYAMSRLAWKALQLLQG
KPPPAEDDHESACTPLSCGWEMVESLCVPPVFTPAGII

>Taeniopygia guttata_AZIN2(ENSTGUP_1370)
MNGYLNESNFLMVEEGFTTRDLLENLLGEPCQESDQQAFFVADLGDIVKKHLRFLKALPR
VKPYFPVKCNGSEGVIRLLAELGAGFACANKAEITRVQGIGVPADRIFYSSPCKQVAHIR
YAAAHGVRLMAFDNEVELSKVARSHPRARMLLGITADSTPPAHPSMTFGTTLKSCRHLLE
TAKEQAVEVVGISFHLGSRGLEPQAWAQAVATAQLVFDMGTELGHHMHLLDIGGGFPGTE
DTRALLEEMAAGINSALDLYFPEGSGVDIVARPGRYYVTSAFTLAVSITALEETPMEQPG
SDGRCDSRTCPLSLSLLPNVSSVSLSPLPVPTCPPYVSLNPPEPSPSTHSSSLQSPRGHG
EERIAEGLELPQLHVGDWLILGTGSYSMPSSSLLTAGPQARVTYAMSRVAWEGGQGWVRP
PQAEDDRESLCMPLSCGWEMAETLCVTPVFAPAGII

>Anolis carolinensis_AZIN2(ENSACAP_3333)
MTRCLDESDIVMVEEGFTTKDVLENLLLEVDQTGNREPFFVADLEEVVKKHLRLLKALPR
IKPFYALKCNRSKGVVQTLAGLGAGFSCANKTEMALVKSVGVPAERIICASPCKQLSLIK
YAASQGVQLMTFDNEVELGKVARSHPSARMLLCLATDDSRSSTHLSIKFGATLKTCRHLL
ETAKEMKVEVVGISFHLGSSCPDPQIFTQSIADARLVFEMGAEVGYKMHLLDIGGGFPAF
EESRGRFEETSAVVNSALDLYFPEGCGVEIMAELGRYYVDSAFTLAVNIVAKKEVTLDQV
SSEDEDPGGKKSFVYHLNDGVYGSFGSVIFNNACPIPILHQKPSPDQRLHSSSFWGPTGD
GLDRLAEGVDLPELHVGDWLVFENMGAYAVPPSASLSSGPPARIHYAMSRVAWEAVQLLW
GKLLHTEEEEEEGRESACTPLSCGWEITDSLCAAPVFAPASIM

>Homo sapiens_ODC1(ENSP_385333)
MNNFGNEEFDCHFLDEGFTAKDILDQKINEVSSSDDKDAFYVADLGDILKKHLRWLKALP
RVTPFYAVKCNDSKAIVKTLAATGTGFDCASKTEIQLVQSLGVPPERIIYANPCKQVSQI
KYAANNGVQMMTFDSEVELMKVARAHPKAKLVLRIATDDSKAVCRLSVKFGATLRTSRLL
LERAKELNIDVVGVSFHVGSGCTDPETFVQAISDARCVFDMGAEVGFSMYLLDIGGGFPG
SEDVKLKFEEITGVINPALDKYFPSDSGVRIIAEPGRYYVASAFTLAVNIIAKKIVLKEQ
TGSDDEDESSEQTFMYYVNDGVYGSFNCILYDHAHVKPLLQKRPKPDEKYYSSSIWGPTC
DGLDRIVERCDLPEMHVGDWMLFENMGAYTVAAASTFNGFQRPTIYYVMSGPAWQLMQQF
QNPDFPPEVEEQDASTLPVSCAWESGMKRHRAACASASINV

>Pan troglodytes_ODC1(ENSPTRP_20031)
MNNFGNEEFDCHFLDEGFTAKDILDQKIHEVSSSDDKDAFYVADLGDILKKHLRWLKALP
RVTPFYAVKCNDSKAIVKTLAAIGTGFDCASKTEIQLVQSLGVLPERIIYANPCKQVSQI
KYAANNGVQMMTFDSEVELMKVARAHPKAKLVLRIATDDSKAVCRLSVKFGATLRTSRLL
LERAKELNIDVVGVSFHVGSGCTDPETFVQAISDARCVFDMGAEVGFSMYLLDIGGGFPG
SEDVKLKFEEITGVINPALDKYFPSDSGVRIIAEPGRYYVASAFTLAVNIIAKKIVLKEQ
TGSDDEDESSEQTFMYYVNDGVYGSFNCILYDHAHVKPLLQKRPKPDEKYYSSSIWGPTC
DGLDRIVERCDLPEMHVGDWMLFENMGAYTVAAASTFNGFQRPTIYYVMSGPAWQLMQQF
QNPDFPPEVEEQDASTLPVSCAWESGMKRHRAACASASINV

>Pongo abelii_ODC1(ENSPPYP_14127)
MNNFGNEEFDCHFLDEGFTAKDILDQKINEVSSSDDKDAFYVADLGDILKKHLRWLKAVP
RVTPFYAVKCNDSKAIVKTLAAVGTGFDCASKTEIQLVQSLGVPPERIIYANPCKQVSQI
KYAANNGVQMMTFDSEVELMKVARAHPKAKLVLRIATDDSKAVCRLSVKFGATLRTSRLL
LERAKELNIDVVGVSFHVGSGCTDPETFVQAISDARCVFDMGAEVGFSMYLLDIGGGFPG
SEDVKLKFEEITSVINPALDKYFPSDSGVRIIAEPGRYYVASAFTLAVNIIAKKMVLKEQ
TGSDDEDESSEQTFMYYVNDGVYGSFNCILYDHAHVKPLLQKRPKPDEKYYSSSIWGPTC
DGLDRIVERCDLPEMHVGDWMLFENMGAYTVAAASTFNGFQRPTIYYVMSGPAWQLMQQF
QNPDFPPEVEEQDASTLPVSCARESGMKRHRAACASASINV

>Mus musculus_ODC1(ENSMUSP_128661)

MSSFTKDEFDCHILDEGFTAKDILDQKINEVSSSDDKDAFYVADLGDILKKHLRWLKALP
RVTPFYAVKCNDSRAIVSTLAAIGTGFDCASKTEIQLVQGLGVPAERVIYANPCKQVSQI
KYAASNGVQMMTFDSEIELMKVARAHPKAKLVLRIATDDSKAVCRLSVKFGATLKTSRLL
LERAKELNIDVIGVSFHVGSGCTDPETFVQAVSDARCVFDMATEVGFSMHLLDIGGGFPG
SEDTKLKFEEITSVINPALDKYFPSDSGVRIIAEPGRYYVASAFTLAVNIIAKKTVWKEQ
PGSDDEDESNEQTFMYYVNDGVYGSFNCILYDHAHVKALLQKRPKPDEKYYSSSIWGPTC
DGLDRIVERCNLPEMHVGDWMLFENMGAYTVAAASTFNGFQRPNIYYVMSRPMWQLMKQI
QSHGFPPEVEEQDDGTLPMSCAQESGMDRHPAACASARINV

>Rattus norvegicus_ODC1(ENSRNOP_7259)
MGSFTKEEFDCHILDEGFTAKDILDQKINEVSSSDDKDAFYVADLGDVLKKHLRWLKALP
RVTPFYAVKCNDSRAIVSTLAAIGTGFDCASKTEIQLVQGLGVPPERIIYANPCKQVSQI
KYAASNGVQMMTFDSEIELMKVARAHPKAKLVLRIATDDSKAVCRLSVKFGATLKTSRLL
LERAKELNIDVIGVSFHVGSGCTDPETFVQAVSDARCVFDMGTEVGFSMYLLDIGGGFPG
SEDTKLKFEEITSVINPALDKYFPSDSGVRIIAEPGRYYVASAFTLAVNIIAKKTVWKEQ
TGSDDEDESNEQTLMYYVNDGVYGSFNCILYDHAHVKALLQKRPKPDEKYYSSSIWGPTC
DGLDRIVERCSLPEMHVGDWMLFENMGAYTVAAASTFNGFQRPNIYYVMSRSMWQLMKQI
QSHGFPPEVEEQDVGTLPMSCAQESGMDRHPAACASASINV

>Canis familiaris_ODC1(ENSCAFP_32017)
MNNFNNAEFDCHFLDEGFTAKDILDQKINEVSSSDDKDAFYVADLGDILKKHLRWLKALP
RVTPFYAVKCNDSRTIVKTLAAIGTGFDCASKTEIQLVQSLGVPPERIIYANPCKQVSQI
KYASNNGVQMMTFDSEVELMKVARAHPKAKLVLRIATDDSKAVCRLSVKFGATLKTSRLL
LERARELNIDVIGVSFHVGSGCTDPETFVQAISDARCVFDMGAEVGFNMYLLDIGGGFPG
SEDVKLKFEEITSVINPALDKYFPADSGVRVIAEPGRYYVASAFTLAVNIIAKKLVLKEQ
TGSDDEDESSEQTFMYYVNDGVYGSFNCILYDHAHVKPLLQKRPKPDEKYYSTSIWGPTC
DGLDRIVERCDLPEIHVGDWMLFENMGAYTVAAASTFNGFQRPTIYYVMSGPTWQLMQQI
QNHDFPPEVEEQDVSTLPVSCAWESGMKRPPAACASASINV

>Felis catus_ODC1(ENSFCAP_17722)
MNSFSNAEFDCHFLDEGFTAKDILDQKINEVSSSDDKDAFYVADLGDILKKHLRWLKALP
RVTPFYAVKCNDSRTIVKTLAAIGTGFDCASKTEIQLVQSLGVPPERIIYANPCKQVSQI
KYAANNGVQMMTFDSEVELMKVARAHPKAKLVLRIATDDSKAVCRLSVKFGATLKTSRLL
LERARELNIDVIGVSFHVGSGCTDPETFVQAISDARCVFDMGAEVGFNMCLLDIGGGFPG
SEDVKLKFEEITSVINPALDKYFPADSGVRVIAEPGRYYVASAFTLAVNIIAKKLVVKEQ
TGSDDEDESSEQTFMYYVNDGVYGSFNCILYDHAHVKPLLQKRPKPDEKYYSTSIWGPTC
DGLDRIVERCDLPEMHVGDWMLFENMGAYTVAAASTFNGFQRPTIYYVMSGPTWQRMQQI
QNHDFPPEAEEQDASALPVSCARESGMKRPPAACASASINV

>Myotis lucifugus_ODC1(ENSMLUP_19721)
MNNVSNEEFDCHFLDEGFTAKDILDQKINEVSSSDDKDAFYVADLGDILKKHLRWLKALP
RVTPFYAVKCNDSRTIVKTLAAIGTGFDCASKTEIQLVQSLGVPPERIIYANPCKQVSQI
KYAANNGVQMMTFDSEVELMKVARAHPKAKLVLRIATDDSKAVCRLSVKFGATLKTSRLL
LERAKELNIDVIGVSFHVGSGCTDPETFVQAISDARCVFDMGAEVGFNMYLLDIGSGFPG
SEDAKLKFEEITSVINPALDKYFPSDSGVTVIAEPGRYYVASAFTLAVNIIAKKLVLKEQ
TGSDDEEESSEKTFMYYVNDGVYGSFNCILYDHAHVKPLLQKRPKPDEKYYSSSIWGPTC
DGLDRIVERCGLPEMHVGDWMLFENMGAYTVAAASTFNGFQRPTIYYVMSGPTWQLMQQI
QNRDFPPEAEEDAVALPVSCAWESGMELLPAACASAHVNV

>Monodelphis domestica_ODC1(ENSMODP_18485)
MNSFSNDEFDFNFLDEGFTAKDILDQKINEVSSSDDKDAFYVADLGDILKKHLRWYKALP
RVTPFYAVKCNDSRAILKTLAAVGAGFDCASKTEIQLVQSLGVPPERIIYANPCKQVSQI
KYAANNGVQMMTFDSEVELMKVARAHPKAKLVLRIATDDSKAVCRLSVKFGATLKTSRLL
LERAKELNIDVIGVSFHVGSGCTDPETFVQAVSDARCVFDMGAEFGFNMYLLDIGGGFPG
SEDVKLKFEEISSVINPALDKYFPPDSGVRIIAEPGRYYVASAFTLAVNIIAKKLVLKEQ
TGSDDEDEANDQTFMYYVNDGVYGSFNCILFDHAHVKPLLQKRPKPDEKYYSSSIWGPTC
DGLDRIVERCDLPEMHVGDWMLFENMGAYTVAAASTFNGFQRPTIYYVMSGPAWQCMQQI
KNQGFPAEVEDQDINTLPLSCAWESGMEHHSTTCTSASVNV

>Gallus gallus_ODC1(ENSGALP_26476)
MSGIMGSFSKEELEFTFLDEGFTAKDILDQKINEVSSSDDKDAFYVADLGDIVKKHMRWH

KALPRVTPFYAVKCNDSEAVVKTLAVLGAGFDCASKTEIQLVQSIGVPPERIIYANPCKQ
LSQIKHAANSGVRMMTFDSEVELMKIARAHPKAKLVLRITTDDSKAVCRLSVKFGATLKT
SRLLLERAKELDLAIVGVSFHVGSGCTDPETFVQAISDARCVFDMGAELGFNMYLLDIGG
GFPGSEDVKLKFEEITSVINPALDKYFPLDSEVTIIAEPGRYYVASAFTLAVNIIAKKIV
SKEQTGSDDEDDVNDKTLMYYVNDGVYGSFNCILYDHAHVKPVLQKRPKPDDGCYSCSIW
GPTCDGLDRIVERCNMPELQVGDWILFENMGAYTVAAASTFNGFQRPTIHYVMSRPAWQL
MQQIKEQEFLAEVEEQDVASLPLSCACESGIEYPATCASASINV

>Taeniopygia guttata_ODC1(ENSTGUP_13433)
MSNFSKEEFELTFLDEGFTAKDILDQKINEVSSSDDKDAFYVADLGDIVKKHVRWHKALP
RVTPFYAVKCNDSKAVVKTLAVLGAGFDCASKTEIQLVQSIGVPPERIIYANPCKQVSQI
KHAASSGVQMMTFDSEVELMKVARAHPKAKLVLRITTDDSKAVCRLSVKFGATLKTSRLL
LERAKELDLAIVGVSFHVGSGCTDPETFVQAISDARCVFDMGAELGFSMYLLDIGGGFPG
SEDVKLKFEEITSVINPALDKYFPSDSGINIIAEPGRYYVASAFTLAVNIIAKKIVLKEQ
TGSDDEDDANDKTLMYYVNDGVYGSFNCILYDHAHVKPVLQKRPKPDDSCYSCSIWGPTC
DGLDRIVERFNMPELQVGDWILFENMGAYTVAAASTFNGFQRPTIHYVMSRPAWQLMQQI
KEQGFLAEVEEQDVASLPVSCAWESGIEYPATCASASINV

>Anolis carolinensis_ODC1(ENSACAP_12470)
MTEPLTCFLKEGNLMCQHWEICLLHLRNITPSKMNGFSGEEFSFTFLDEGFTAKDIVDQK
INEVSSSDDKDAFYVADLGDVLKKHLRWYKALPRVSPFYAVKCNDSIAVVKILAALGAGF
DCASKTEIQLVQSVGVPPERIIYANPCKQVSQIKHAANNGVQMMTFDSEVELMKVARAHP
KAKLVLRIATDDSKAVCRLSVKFGATLKTSRLLLERAKELDLDIIGVSFHVGSGCTDPET
FVQAISDARCVFDMGAELGFSMYLLDIGGGFPGSEDVKLKFEEITNVINPALDKYFPSDS
GVKIIAEPGRYYVASAFTLAVNIIAKKVVIKEQTGSDDEEEQNDKTLMYYVNDGVYGSFN
CILYDHAHVKPVLQKRPKPDERYYSCSIWGPTCDGLDRIVELCAMPELQVGDWMLFENMG
AYTVAAASTFNGFQRPTIYYVMSRPAWQLMEQIKEQGFQAEVEEQEVTLPLSCAWESGIE
HYPAACASASINV

>Xenopus tropicalis_ODC1(ENSXETP_7603)
MNSFSNDDFDFSFLEEGFCARDIVEQKINEVSLSDDKDAFYVADLGDIVKKHVRWFKALP
RVTPFYAVKCNDSKAIVKTLSLLGAGFDCASKTEIQLVQSIGVSSERIIYANPCKQVSQI
KYAASCGVEKMTFDSEVELMKVARNHPNAKLVLRIATDDSKAVCRLSVKFGATLKTSRLL
LERAKELNVDIIGVSFHVGSGCTDPQTYVQAVSDARCVFDMGAELGFNMHLLDIGGGFPG
SEDVKLKFEEITSVINPALDKYFPVDSGVTIIAEPGRYYVASAFTLAVNIIAKKVMVNEQ
SGSDDEEDAANDKTLMYYVNDGVYGSFNCILFDHAHVKPVLTKKPKPDESFYSSSIWGPT
CDGLDRIVERFDLPELQVGDWMLFENMGAYTVAASSTFNGFQRPTLYYVMSRPHWQLMHD
IKEHGILPEVPELSAHRVSCAQESGIELAPTICPTASINV

>Danio rerio_ODC1(ENSDARP_124918)
MTACTGSDFDFAFLEEGFCARDIVEQKINESSLSDDKDAFYVADLGDVLKKHLRWLRVLP
RITPFYAVKCNDSRAVVTTLASLGAGFDCASKTEIQIVQSVGVDPSRIIYANPCKQVSQI
KYASAHGVQMMTFDSEVELMKVARSHENAKLVLRIATDDSKAVCRLSVKFGATLKSSRLL
LERAKELGLDVIGVSFHVGSGCTDPETYSQAISDARYVFDIGAELGYNMSLLDIGGGFPG
SEDTKLKFEEIAAVINPALDKYFPVDSGVRIIAEPGRYYVASAYTLAVNIIAKKVIMKEQ
SASDEEEDVSNDRTLMYYVNDGVYGSFNCILYDHAHVLPTLHKKPKPDERMYPCSIWGPT
CDGLDRIVEQCSLPDMQVGDWLLFENMGAYTVAASSTFNGFQKPDIYYIMSRTAWQCMQQ
IRAQGIPALPLEEPSAGNVPSHCGRESSLDVPAKPCPTQVL

>Takifugu rubripes_ODC1(ENSTRUP_17393)
VGVTMNTEMEFSFLEEGFSAKDIVEQKIKESSVTDDRDAFYVCDLGDVLKKHVRWMRALP
RITPFYAVKCNDSLSVLKTLASLGTGFDCASKTEIQLVQSLGVDPSKIIYANPCKQVSQI
KYASAHGVKMMTFDSEVELMKVARCHDNAKLVLRIATDDSKAVCRLSVKFGVPVKACRGL
LERAKKLGLDVIGVSFHVGSGCTDPKAYIQAIADARCVFDIGNELGFKMDLLDIGGGFPG
SDDAELQFEEITAVINPTLEKYFPADSDVKIIAEPGRFYVASAYTLVVNIIAKKVILDED
SDEEDERTNEKTVMYYVNDGVYGSFNCILYDHAHCMPTLHKKRKPDEVRYPSSIWGPTCD
GLDRIVELCSLPDLQVGDWLVFENMGAYTVAASSTFNGFQKPDLHYIMSRPAWQSVQQIS
QGMLPPAEESSLFEAVGCGRESSLDIPMKSCQANVV

>Tetraodon nigroviridis_ODC1(ENSTNIP_4466)
LCVTMNAEMEFSFLEEGFSAKDIVEQKIKESSLTVTVPPVRDPFSLQACVERRLLFCFSS

VLRTLASLGTGFDCASKTEIQLVQSLGVDAGRIIYANPCKQASQIKYAAAHGVQMMTFDS
EVELMKVARCHDNAKLVLRIATDDSKAVCRLSVKFGVPVKACRGLLERARELGLDVIGVS
FHVGSGCTDPNAYTQAIADARCVFDIGNELGFKMDLLDIGGGFPGSDDAALQFEEITAVI
NPALDKYFPADTEVKVIAEPGRFYVASAYTLVVNIIAKKVIEDEDEDEEDERSGEKTVMY
YVNDGVYGSFNCILYDHAHCMPTLHKKRKPDEVRYPSSIWGPTCDGLDRIVELCSLPDLQ
VGDWLVFENMGAYTVAASSTFNGFQKPDLHYIMSRRAW

>Gasterosteus aculeatus_ODC1(ENSGACP_17174)
MNTSTSTEFDFSFLEEGFSARDIVEQKINESSTTDDRDAFYVCDLGDVLKKHLRWVRALP
RVTPFYAVKCNNSRAVVMTLASLGAGFDCASKNEIHLVQSLGVDPSRIIYANPCKQVSQI
KYASAHGVRMMTFDSEVELMKVARCHENAKLVLRIATDDSKAVCRLSVKFGAPLKACRGL
LECAKKLQLDVIGVSFHVGSGCTDPQTYSQAIADARCVFDMGDELGFNMDLLDIGGGFPG
SDEAELKFEEITGVISPALDKFFPADTGIKVIAEPGRFYVASAYTLVVNIIAKKAIVDDE
SASDEEDEGTNDTTLMYYVNDGVYGSFNCILYDHAHCLPTLLKKPKPDEAMYTCSIWGPT
CDGLDRIVEQCDLPDLQVGDWLVFDNMGAYTVAASSTFNGFQKPDIYYVMSRTAWQQVQR
ICSQGLPPPAEESCVLDVPACCGRQSSMDQHPLVINC

>Oryzias latipes_ODC1(ENSORLP_22229)
VTMNTEVPTLEFSFLEEGFSAWDIVEQKINESSLTDDRDAFYVCDLGDVLKKHLRWTRAL
PRVTPFYAVKCNDSRAVVMTLASLGTGFDCASKTEIQLVQSLGVHPNRIIYANPCKQVSQ
MKYASAHGVEMMTFDSEVELMKVARYHDNAKLVLRIATDDSKAVCRLSVKFGAPIKACRG
LLERAKELGLDVVGVSFHVGSGCTDAGTFVKAIADARCVFDMGEELGFQMDLLDIGGGFP
GSEDTELSFEEITATINPALDKYFPVDSGVRIIAEPGRFYVASAYTLVVNIIAKKVIIDE
EAPIDEEDNKTLMYYVNDGVYGSFNCILYDHAHCYPVLHKKPKPDETMYSCSIWGPTCDG
LDRIVEQCCLPDMQVGDWLVFKNMGAYTVAASSTFNGFQRPDIHYVMSRPAWQYVQQIFA
QGVLPPPLEGSSQLEIPACCGRGSTFDMPAKPCPANIV

>Ciona intestinalis_(ENSCINP_16169)
MKQDCSVETSAVEIYPTINTSEIIASKILNDGREDHDNGFFICDLGDVMKKYIRWQKNFP
RVIPHYAIKCNPDEILLKMLIGLGTGFDCASKSEIRQMLALGARPEQIIFANPCKQASHV
RYARDNNVKRIVFDNVEELIKMKKNFPTADLVLRIQTDDSQSICRFSMKYGAHPDVCKQL
LDTAHKMNLNVVGISFHVGSGCQDVQAFVKALQNARVLFDYAECIGYKLNLLDIGGGFPG
TEDVNLKFEEIASVVNMTLKELFPMEEFPHVDVIAEPGRYFAASAYTLASNIIAKREVMR
DVTEETVCEQTAYMYYINDGLYGSFNCLFYDHAHVTPKLLKEPSEIDMLYSSSVWGPTCD
GLDRICELVQLPQLDVGNWILWEDMGAYTMAAGSSFNGFKTSKVYYVISDLEKHVLRNCK
MRIP

>Ciona savignyi_(ENSCSAVP_17223)
DHDDAFFVCDLGDVVKKYVRWQKNFPRIVPYYAIKCNPDETMLKLMIGLGAGFDCASKAE
IRQMLSLGASPEKIIFANPCKQTSHVRYAKENNIKPVVFDNKEELVKMKKNYPNADLVLR
IQTDDSQSVCPFSMKYGAHPDSCPQLLDTAYKMGLNVVGISFHVGSGCTDVQAFVKAIQN
ARVLFDYAESIGYHFTLLDIGGGFPGTENVDLKFEEIASMVNLALDDLFPKSDFPHVQII
AEPGRYFAASAYTLATNIIAKRAVLQGVPGEEADSPKAFMYYVNDGIYGSFNCLFFDHAQ
VTPQILKQTKQNGVRFSSSIWGPTCDGLDQICEHVMLPELTTGNWIIWEDMGAYTISAGS
AFNGFKTTKMFYVIS

>Nematostella vectensis_(XP_001636251)
MKQSIGTDLAIEIVKDGLSHRDVITEKIDSLGREEKDDAFYIVDLADVVLKHKKWVQLLPRVEPFYAMKC
NNDPAVLKLLAGLGLGFDCASKGEIQTILGLGVSPKKIVYANPCKQASHIKYASQHDVAMTTFDNEMELH
KIKKLYPDAQLILRIRTDDSKSLCQLGIKFGAPLKQCRHLLKVAQDLGLNVVGVSFHVGSGCFDASAYAT
AVIAARSVFNLAVQFGFNCTLLDIGGGFPGDPNAAITFEEICTTINPLLEEHFPPSSGVRIIAEPGRYYS
SSSFTLAVNVYSRREVQSSSVLAEDVTAPENNGNQGFMYYVNDGVYGSFNCLIFDHATVEAIPVKESSGQ
PLYQTSIWGPTCDSMDCITKNMMLPKVEVGEWLFFKYMGAYTQSAASTFNGFKKPTKFYLCTEELW

>Trichoplax adhaerens_(XP_002114448)
MSNPTIYYQLSKDKDDPFYIVNVGNILKKHKTWLSLLPRVEPFYAVKCNDDRNVLATLSALGIGFDCASM
AEIKAVLKLGVPPSKIVYANPCKQASHLKFAAKREVSMMTFDNEPELHKVKQLHPTAKLILRLLTDDSKS
LCRLGQKYGAPLKTVPNLLQVARNLDLNVVGISFHVGSGCYDASAFSSAVEIAKTAFDYGKSMGFQFDIL
DIGGGFPGSPRATVSFEEISATLNKALNKHFPVESGVRIIAEPGRYYVASAFNLAVNVIAKRQVNDDNTQ
AQGKQHAYRNQKYMYYVNDGVYGSFNCLLYDHAIVYPKTLGRDNEQQYSSSIWGPTCDGLDCVMKECMLP
ELDVGDWMYFEDMGAYTAAAASNFNGFQKPLAYYVIEEKVWALRKERNRNKPLPTS

Cholinergic Receptors Nicotinic subunits-CHRN

>Homo sapiens_CHRNB2(ENSP_357461)
MARRCGPVALLLGFGLLRLCSGVWGTDTEERLVEHLLDPSRYNKLIRPATNGSELVTVQL
MVSLAQLISVHEREQIMTTNVWLTQEWEDYRLTWKPEEFDNMKKVRLPSKHIWLPDVVLY
NNADGMYEVSFYSNAVVSYDGSIFWLPPAIYKSACKIEVKHFPFDQQNCTMKFRSWTYDR
TEIDLVLKSEVASLDDFTPSGEWDIVALPGRRNENPDDSTYVDITYDFIIRRKPLFYTIN
LIIPCVLITSLAILVFYLPSDCGEKMTLCISVLLALTVFLLLISKIVPPTSLDVPLVGKY
LMFTMVLVTFSIVTSVCVLNVHHRSPTTHTMAPWVKVVFLEKLPALLFMQQPRHHCARQR
LRLRRRQREREGAGALFFREAPGADSCTCFVNRASVQGLAGAFGAEPAPVAGPGRSGEPC
GCGLREAVDGVRFIADHMRSEDDDQSVSEDWKYVAMVIDRLFLWIFVFVCVFGTIGMFLQ
PLFQNYTTTTFLHSDHSAPSSK

>Pan troglodytes_CHRNB2(ENSPTRP_2339)
MARRCGPVALLLGFGLLRLCSGVWGTDTEERLVEHLLDPSRYNKLIRPATNGSELVTVQL
MVSLAQLISVHEREQIMTTNVWLTQEWEDYRLTWKPEEFDNMKKVRLPSKHIWLPDVVLY
NNADGMYEVSFYSNAVVSYDGSIFWLPPAIYKSACKIEVKHFPFDQQNCTMKFRSWTYDR
TEIDLVLKSEVASLDDFTPSGEWDIVALPGRRNENPDDSTYVDITYDFIIRRKPLFYTIN
LIIPCVLITSLAILVFYLPSDCGEKMTLCISVLLALTVFLLLISKIVPPTSLDVPLVGKY
LMFTMVLVTFSIVTSVCVLNVHHRSPTTHTMAPWVKVVFLEKLPALLFMQQPRHHCARQR
LRLRRRQREREGAGALFFREAPGADSCTCFVNRASVQGLAGAFGAEPAPVAGPGRSGEPC
GCGLREAVDGVRFIADHMRSEDDDQSVSEDWKYVAMVIDRLFLWIFVFVCVFGTIGMFLQ
PLFQNYTTTTFLHSDHSAPSSK

>Mus musculus_CHRNB2(ENSMUSP_29562)
MARCSNSMALLFSFGLLWLCSGVLGTDTEERLVEHLLDPSRYNKLIRPATNGSELVTVQL
MVSLAQLISVHEREQIMTTNVWLTQEWEDYRLTWKPEDFDNMKKVRLPSKHIWLPDVVLY
NNADGMYEVSFYSNAVVSYDGSIFWLPPAIYKSACKIEVKHFPFDQQNCTMKFRSWTYDR
TEIDLVLKSDVASLDDFTPSGEWDIIALPGRRNENPDDSTYVDITYDFIIRRKPLFYTIN
LIIPCVLITSLAILVFYLPSDCGEKMTLCISVLLALTVFLLLISKIVPPTSLDVPLVGKY
LMFTMVLVTFSIVTSVCVLNVHHRSPTTHTMAPWVKVVFLEKLPTLLFLQQPRHRCARQR
LRLRRRQREREGAGTLFFREGPAADPCTCFVNPASMQGLAGAFQAEPAAAGLGRSMGPCS
CGLREAVDGVRFIADHMRSEDDDQSVREDWKYVAMVIDRLFLWIFVFVCVFGTIGMFLQP
LFQNYTATTFLHSDHSAPSSK

>Canis familiaris_CHRNB2(ENSCAFP_25294)
MATRSGPAALLLGFGLLGLHAGVWGTDTEERLVEHLLDPSRYNKLIRPATNGSELVTVQL
MVSLAQLISVHEREQIMTTNVWLTQEWEDYRLTWKPEEFDNMKKVRLPSKHIWLPDVVLY
NNADGMYEVSFYSNAVVSYDGSIFWLPPAIYKSACKIEVKHFPFDQQNCTMKFRSWTYDR
TEIDLVLKSDVASLDDFTPSGEWDIVALPGRRNENPDDSTYVDITYDFIIRRKPLFYTIN
LIIPCVLITSLAILVFYLPSDCGEKMTLCISVLLALTVFLLLISKIVPPTSLDVPLVGKY
LMFTMVLVTFSIVTSVCVLNVHHRSPTTHTMAPWVKVVFLEKLPTLLFMQQPRHRCARQR
LRLRRRQREREAGALLFREAPGESYSGSARGGVGTDGCGLRSAVDGVRFIADHVRNEDDD
QSVSEDWKYVAMVIDRLFLWIFVFVCVFGTIGMFLQPLFQNYSAATFLHTDHSAPSAK

>Xenopus tropicalis_CHRNB2(ENSXETP_8352)
YISCRDFAFAFGTGCLGTDTEERLVEHLLDPSRYNKLIRPATNGSEQVTVQLMVSLAQLI
SVHEREQIMTTNVWLTQEWEDYRLTWDPAEFDDMKKVRLPSKHIWLPDVVLYNNADGMYE
VSFYSNAVVSHDGSIFWLPPAIYKSACKIEVKHFPFDQQNCTMKFRSWTYDRTELDLVLR
SDVASLDDFTPSGEWDIIALPGRRNENPNDSTYVDITYDFIIRRKPLFYTINLIIPCILI
TSLAILVFYLPSDCGEKMTLCISVLLALTVFLLLISKIVPPTSLDVPLVGKYLMFTMVLV
TFSIVTSVCVLNVHHRSPTTHTMPPWVKVVFLDKLPTLLFMKQPRQNCARQRLRQQRQSQ
ERAAGSFFLRDGARSCTCYVNQASVKKYGAQLSELPEGVNGFRDRQGKVRQCLCGLQEAV
DGVRFIADHMKSEDDDQSVSEDWKYVAMVIDRLFLWIFVFVCVFGTIGMFLQPLFQNYTT
NALVHMNHAAKKMK

>Danio rerio_CHRNB2(ENSDARP_41624)
MMALWTLFCILAIVKSGYGADTEERLVEHLLNPAHYNKLIRPATNGSEVVTVQLMVSLAQ
LISVHEREQIMTTNVWLTQEWQDYRLTWSPEEFDGMKKVRLPSKHIWLPDVVLYNNADGM
YEVSFYSNAVVSYDGSVFWLPPAIYKSACKIEVKHFPFDQQNCTLSFRSWTYDRTEVDLV
LRADVASMDDFTPSGEWDIIALPGRRNENPSDPTYVDITYDFIIRRKPLFYTINLIIPCV
LITSLAILVFYLPSDCGEKMTLCISVLLALTVFLLLISKIVPPTSLDVPLVGKYLMFTMV
LVTFSIVTSVCVLNVHHRSPTTHTMPPWVKLVFLNKLPALLFMRQPRNSSERQRLRQRRR
ANEQQEGGRGGLAAGLMAGLGLSGSSSGSKENNEACTCYVNRASVKQFGAELGGGGGGGG
STDGLNGIREGGRETREGGANPLQGSALPRGKQQPPTVLTQALLAQACPGFEEAVDGVRF
IANHMKSEDDDRSVSEDWKYVAMVIDRLFLWIFVFVCVFGTIGMFLQPLFQNYTAKTITH
TPG

>Takifugu rubripes_CHRNB2(ENSTRUP_14017)
TAVLPSSLHPSIQALLFQYAWMVGSDTEERLVEHLLNPAHYNKLIRPATNGSELVTVQLM
VSLAQLISVHEREQVMTTNVWLTQEWQDYRLTWIPEEFDGMLKVRLPSKHIWLPDVVLYN
NADGVYEVSFYSNAVVSYDGSIFWLPPAIYKSACKIEVKHFPFDQQNCTLRFRSWTYDRT
EIDLVLRADVASMDDFTPSGEWDIIALPGRRNENPGDPTYVDITYDFIIRRKPLFYTINL
IIPCVLITSLAILVFYLPSDCGEKMTLCISVLLALTVFLLLISKIVPPTSLDVPLVGKYL
MFTMVLVTFSIVTSVCVLNVHHRSPTTHTMPPWVKMVFLQKLPALLFMRQPRNSCERQRL
RQRRRAQEHKEGGIFGKEDSDPCTCYVNRASVKQFGGDLGGAGGSMDGLNRARDGREAQA
CPGFEEAVEGVRFIANHMKSEDDDQSVSEDWKYVAMVIDRLFLWIFVFVCVFGTMGMFMQ
PLFQNYTAKTINSSPG

>Tetraodon nigroviridis_CHRNB2(ENSTNIP_21501)
GLCADTEERLVEHLLNPAHYNKLIRPATNGSELVTVQLMVSLAQLISVHEREQVMTTNVW
LTQEWQDYRLTWIPEEFDGMLKVRLPSKHIWLPDVVLYNNADGVYEVSFYSNAVVSYDGS
IFWLPPAIYKSACKIEVKHFPFDQQNCTLRFRSWTYDRTEIDLVLRADVASMDDFTPSGE
WDIIALPGRRNENPGDPTYVDITYDFIIRRKPLFYTINLIIPCVLITSLAILVFYLPSDC
GEKMTLCISVLLALTVFLLLISKIVPPTSLDVPLVGKYLMFTMVLVTFSIVTSVCVLNVH
HRSPTTHTMPPWVKMVFLQKLPALLFMRQPRNSCERQRLRQRRRAQEQKGGGRCGEPGGL
MVGNGLGNAGGSGGGTTTGLFGKEDSDPCTCYVNRASVKQFGGDLGSAGGGSMDGLNRAR
EAREGGSGNQSRGKPGTGAPALSHALLAQACPGFEEAVEGVRFIANHMKSEDDDQSVSED
WKYVAMVIDRLFLWIFVFVCVFGTMGMFLQPLFQNYTAKTINSSPG

>Gasterosteus aculeatus_CHRNB2(ENSGACP_16024)
GLGADTEERLVEHLLNPAHYNKLIRPATNGSELVTVQLMVSLAQLISVHEREQVMTTNVW
LTQEWQDYRLTWVPEEFDGMLKVRLPSKHIWLPDVVLYNNADGVYEVSFYSNAVVSYDGS
IFWLPPAIYKSACKIEVKHFPFDQQNCTLRFRSWTYDRTEIDLVLRADVASMDDFTPSGE
WDIIALPGRRNENPADPTYVDITYDFIIRRKPLFYTINLIIPCVLITSLAILVFYLPSDC
GEKMTLCISVLLALTVFLLLISKIVPPTSLDVPLVGKYLMFTMVLVTFSIVTSVCVLNVH
HRSPTTHTMPPWVKLVFLNKLPALLFMRQPRNSCERQRLRQRRRAQEQKEGGRSGEAGAL
MVGLGLGNASGNRGGTSTGVFSKEDSDPCTCYVNRASVKQFGGDLALLAQACPGFEEAVE
GVRFIANHMKSEDDDQSVSEDWKYVAMVIDRLFLWIFVFVCVFGTLGMFMQPLYQNYT

>Homo sapiens_CHRNG(ENSP_374145)
MHGGQGPLLLLLLLAVCLGAQGRNQEERLLADLMQNYDPNLRPAERDSDVVNVSLKLTLT
NLISLNEREEALTTNVWIEMQWCDYRLRWDPRDYEGLWVLRVPSTMVWRPDIVLENNVDG
VFEVALYCNVLVSPDGCIYWLPPAIFRSACSISVTYFPFDWQNCSLIFQSQTYSTNEIDL
QLSQEDGQTIEWIFIDPEAFTENGEWAIQHRPAKMLLDPAAPAQEAGHQKVVFYLLIQRK
PLFYVINIIAPCVLISSVAILIHFLPAKAGGQKCTVAINVLLAQTVFLFLVAKKVPETSQ
AVPLISKYLTFLLVVTILIVVNAVVVLNVSLRSPHTHSMARGVRKVFLRLLPQLLRMHVR
PLAPAAVQDTQSRLQNGSSGWSITTGEEVALCLPRSELLFQQWQRQGLVAAALEKLEKGP
ELGLSQFCGSLKQAAPAIQACVEACNLIACARHQQSHFDNGNEEWFLVGRVLDRVCFLAM
LSLFICGTAGIFLMAHYNRVPALPFPGDPRPYLPSPD

>Gorilla gorilla_CHRNG(ENSGGOP_2069)
MHGGQGPLLLLLLLAVCLGAQGRNQEERLLADLMQNYDPNLRPAERDSDVVNVSLKLTLT
NLISLNEREEALTTNVWIEMQWCDYRLRWDPRDYEGLWVLRVPSTMVWRPDIVLENNVDG
VFEVALYCNVLVSPDGCIYWLPPAIFRSACSISVTYFPFDWQNCSLIFQSQTYSTNEIDL
QLSQEDGQTIEWIFIDPEAFTENGEWAIQHRPAKMLLDPAAPAQEAGHQKVVFYLLIQRK

PLFYVINIIAPCVLISSVAILIYFLPAKAGGQKCTVAINVLLAQTVFLFLVAKKVPETSQ
AVPLISKYLTFLLVVTILIVVNAVVVLNVSLRSPHTHSMARGVRKVFLRLLPQLLRMHVR
PLAPAAVQDTQSRLQNGSSGRSITTGEEVALCLPRSELLFQQWQRQGLVAAALEKLEKGP
ELGPSQFCGSLKQAAPAIQACVEACNLIACARHQQSHFDNGNEEWFLVGRVLDRVCFLAM
LSLFVCGTAGIFLMAHYNRVPALPFPGDPHPYLPSPD

>Mus musculus_CHRNG(ENSMUSP_27470)
MQGGQRPQLLLLLLAVCLGAQSRNQEERLLADLMRNYDPHLRPAERDSDVVNVSLKLTLT
NLISLNEREEALTTNVWIEMQWCDYRLRWDPKDYEGLWILRVPSTMVWRPDIVLENNVDG
VFEVALYCNVLVSPDGCIYWLPPAIFRSSCSISVTYFPFDWQNCSLVFQSQTYSTSEINL
QLSQEDGQAIEWIFIDPEAFTENGEWAIRHRPAKMLLDSVAPAEEAGHQKVVFYLLIQRK
PLFYVINIIAPCVLISSVAILIYFLPAKAGGQKCTVATNVLLAQTVFLFLVAKKVPETSQ
AVPLISKYLTFLMVVTILIVVNSVVVLNVSLRSPHTHSMARGVRKVFLRLLPQLLRMHVR
PLAPAAVQDARFRLQNGSSSGWPIMAREEGDLCLPRSELLFRQRQRNGLVQAVLEKLENG
PEVRQSQEFCGSLKQASPAIQACVDACNLMARARRQQSHFDSGNEEWLLVGRVLDRVCFL
AMLSLFICGTAGIFLMAHYNQVPDLPFPGDPRPYLPLPD

>Xenopus tropicalis_CHRNG(ENSXETP_30553)
RGGMDTVLLLVLICISAALCHNEEEGLLNDLMKNYNKNLRPAEKDGDIVSVSIKLTLTNL
ISLNEREEALTTNVWVEVQWRDYRLSWDPNEYYGISMMRIPSTSVWLPDVGLENNVDGTF
DIALYANTLVSSDGSMYWLPPAIYRSSCPVVVTYFPFDWQNCSMVFQSQTYSANEIELLL
TVDEQTIEWIEIDPEAFTENGEWAIKHMPAKKIINHRLTPDDLNYQQIVFYLIIQRKPLF
YIININIIVPCVLISFVSILVYFLPAKAGGQKCTVSINILLAQTVFLFLIAQKIPETSTAVP
LIVKYLTFLMVVTITIVANAVIVLNISLRTPNTHSMSSTVRELCLRTVPRLLRMHLRPSD
AAPPLAPLMRRSSSVGLMIKADEYMLRKPRSQLMFEKQKERDGLMKVVLDKIGRGMENNT
SDDLVHSLKHAAPEIRACVEACCHIASATREKNEFKSTGDNDWKCQSKVL

>Danio rerio_CHRNG(ENSDARP_110378)
MDQNLSSKIIWLWILLFRFSGALCNLEGSLHRDLMVGYNKNIRPVESHEDIIDVKIKMTL
TNLISLNEKEETLTTCVWIEMQWRDYRLRWANRTGFEVYENITRMRLPSKTIWLPDIGLE
NNVDGRFEVALYANTLIDPDGSVYWLPPAIYRSSCAIKVNYFPFDWQNCSMVFRSQTYNS
NEITLMLSDEDNVTMEWIEIDPEAFTENGEWMIKHRPAKKVINKRYRPDELEHQEIIFFL
IIQRKPLFYVINIIVPCVLFSSLGLLVYFLPAKAGGQKCTMTICILLGQTVFLFLIAKKV
PETSQAVPLIGKYLMFVMSVTTITVMNCVVVLNVSLRTPNTHPMSNTIRKVLLNILPRVL
RMTMQRWTPEQEEGDFKMFALGNGTPLRRRRRSSLGLIAKADEYMFRTARSELMFSRLKE
RKGLLKNTLEKIQNGLGGNTAQDLECSLAAASPEVQQCVSSCKHIAESTKHQNDFQSKNE
EWFLVARVIDRMCFFVMALLFILGTIGIFLTGHFNQAPSQPFPGDPKKYLPEISAGDIPG

>Takifugu rubripes_CHRNG(ENSTRUP_36755)
MDSGCPLSLSLFIRVFMISAAASAVNLEGELFKDLMKGYNKNVRPTEKSGDITQVDIKLT
LTNLISLNEKEEALTTSVWIELQWCDYRLRWDQPPRSTLYGNITKPLRIPSKILWLPDII
LENNVDGQFEVALYCNALVSPDGCVYWLPPAIYRSACPITVNYFPFDWQNCTMVFRSQTY
SANEIDMVLKVEDNHTLEWVDIDPEAFTENGEWAIKHRPAKKVINSQYTKDDREYQEVIF
FLIIQRKPLFYIINIIAPCVLFSSLCLLVYYLPAKAGGQKCTMSVATLLGQTVFLFLIAK
KVPETSKAVPLIAKYLMFVMSVTTMVVMNCVIVLNVSLRTPSTHIMTDAVRKVLLNILPR
LLKMQMRPWTPNNDSTPEIRNRRRSSMTLITKAEEYAMKTARSELMFSRLKERNGLMKSV
LEKLHDGLDGGTPEQLGISLAKASPEVKKCVASCKHIAETARQQSNFQNENEEWFLVARV
IDRACFIVMASVFFIGTIGVFLMGHFNQPPSAPFPRDAKTYLPPIN

>Tetraodon nigroviridis_CHRNG(ENSTNIP_1763)
SGCRLSLSFFVTVFTISAAASAVNLERELFKDLMKGYNKHVRPTEKSGDITQVDIKMTLT
NLISLNEKEEALTTSVWIELQWCDYRLRWDQPPRAALYGNITKPLRIPSRIIWLPDIILE
NNVDGQFEVALYCNALVSPDGCVYWLPPAIYRSACPITVNYFPFDWQNCTMVFRSQTYSA
NEINMVLKVEDNHTLEWVDIDPEAFTENGEWAIKHRPAKKVINRQYTKDDQEYQEVIFFL
IIQRKPLFYIINIIVPCVLFSSLCLLVYYLPAKAGGQKCTMSIATLLGQTVFLFLIAKKV
PETSKAMPLIAKYLMFVMSVTTMVVMNCVIVLNVSLRTPSTHIMTDTVRKILLNILPRLL
KMRMRPWTPNKDSAFPCRRRSSMTLITKAEEYVMKTARSELMFSRLKERNGLMKSVLEKL
HEGLDGGTPEQLSISLAKASPEVKKCVASCKHIAETARQHNNFQNENEEWFLVARVIDRV
CFIVMASVFFIGTMGVFLMGHFNQPPSTPFPGDVKMYLPP

>Gasterosteus aculeatus_CHRNG(ENSGACP_19898)

VIHILTIFICIEKTSAINLEGELFKDLMKGYNKNVRPMENGSDVTQVAIKMTLTNLISLN
EKEEALTTGVWIGMQWCDYRLRWDRPPRSALYGSITSQLRLPSKSIWLPDIILENNVDGN
FEVALYCNALVSPNGCVNWLPPAIYRSACAITVNYFPFDWQNCTMVFHSQTYSANEIKLV
LTEEDDLTLEWVDIDPEAFTENGEWAIKHRPAKKLINTDYNKDELEYQEVVFFLIIQRKP
LFYVINIIAPCVLFSSLGLLVYFLPAKAGGQKCTMSIATLLGQTVFLFLIAKKVPETSRA
VPLIGKYLMFVMSVTTMVVMNCVIVLNVSLRTPNTHKMTDKVRKILLNILPRLLNMQMRP
WTPDGDNADTRRRSSMTLITKAEEYAMKTARSELMFAALKERNGLMRSVLEKLKDELEGG
TAEQLSASLGKASSQLQQCVSSCKHIAKTARQQNNFKKENEEWFLVAKVIDRVCFIVMVS
VFFIGTIGIFLTGHFNQPPSSPFLGDPKTYLPPTNNLTDRVENGR

>Homo sapiens_CHRND(ENSP_258385)
MEGPVLTLGLLAALAVCGSWGLNEEERLIRHLFQEKGYNKELRPVAHKEESVDVALALTL
SNLISLKEVEETLTTNVWIEHGWTDNRLKWNAEEFGNISVLRLPPDMVWLPEIVLENNND
GSFQISYSCNVLVYHYGFVYWLPPAIFRSSCPISVTYFPFDWQNCSLKFSSLKYTAKEIT
LSLKQDAKENRTYPVEWIIIDPEGFTENGEWEIVHRPARVNVDPRAPLDSPSRQDITFYL
IIRRKPLFYIINILVPCVLISFMVNLVFYLPADSGEKTSVAISVLLAQSVFLLLISKRLP
ATSMAIPLIGKFLLFGMVLVTMVVVICVIVLNIHFRTPSTHVLSEGVKKLFLETLPELLH
MSRPAEDGPSPGALVRRSSSLGYISKAEEYFLLKSRSDLMFEKQSERHGLARRLTTARRP
PASSEQAQQELFNELKPAVDGANFIVNHMRDQNNYNEEKDSWNRVARTVDRLCLFVVTPV
MVVGTAWIFLQGVYNQPPPQPFPGDPYSYNVQDKRFI

>Mus musculus_CHRND(ENSMUSP_72983)
MAGPVLTLGLLAALVVCALPGSWGLNEEQRLIQHLFNEKGYDKDLRPVARKEDKVDVALS
LTLSNLISLKEVEETLTTNVWIDHAWVDSRLQWDANDFGNITVLRLPPDMVWLPEIVLEN
NNDGSFQISYACNVLVYDSGYVTWLPPAIFRSSCPISVTYFPFDWQNCSLKFSSLKYTAK
EITLSLKQEEENNRSYPIEWIIIDPEGFTENGEWEIVHRAAKLNVDPSVPMDSTNHQDVT
FYLIIRRKPLFYIINILVPCVLISFMINLVFYLPGDCGEKTSVAISVLLAQSVFLLLISK
RLPATSMAIPLVGKFLLFGMVLVTMVVVICVIVLNIHFRTPSTHVLSEGVKKFFLETLPK
LLHMSRPAEEDPGPRALIRRSSSLGYICKAEEYFSLKSRSDLMFEKQSERHGLARRLTTA
RRPPASSEQVQQELFNEMKPAVDGANFIVNHMRDQNSYNEEKDNWNQVARTVDRLCLFVV
TPVMVVGTAWIFLQGVYNQPPLQPFPGDPFSYSEQDKRFI

>Felis catus_CHRND(ENSFCAP_5273)
TARTPSWGVAGSPQLSPAPWPGRISRQPPSRSPLPAPHPSSCPSPGSWGLNEEERLIRHL
FEEKGYNKELRPVAHKDESVEVSLALTLSNLISLKEVEETLTTNVWMEHGWTDPRLQWDA
EEFGNISVLRLPPDMLWLPEIVLENNNDGSFQISYACNVLVYPSGYVYWLPPAIFRSSCP
ISVTYFPFDWQNCSLKFSSLKYTAKEITLSLKQEEKEGRSYPVEWIVIDPAGFTENGEWE
IVHRPARINVDPSVPLDSPGHQDVTFYLIIRRKPLFYIINILVPCVLISFMINLVFYLPA
DSGEKTSMAISVLLAQSVFLLLISKRLPATSMAIPLIGKFLLFGMVLVTMVVVICVIVLN
IHFRTPSTHVLSEGVKKLFLETLPKRLHMSRPAEDGPSPGALVRRSSSLGYISKAEEYFL
LKSRSDLMFEKQSERHGLARRLTTARRPPAGSEQAQQELFSQLKPAVDGANFIVNHMRDQ
NNYNEEKDCWNRVARTVDRLCLFVVTPIMVVGTAWIFLQGAYNQPPPQPFPGDPFSYHEK
DKRFA

>Xenopus tropicalis_CHRND(ENSXETP_30556)
HAWLWLFDLLCILVLVPGCLSESEEERLLNHIFGERGYRKELRPVEHTGETVNVSLALTL
SNLISLKEADETLTTNVWVELGWYDKRLAWDMEEFHHINIIRVPPDMVWQPQLILENNNN
GVFEVAYYSNVLVNSDGFMYWLPPAIFQTSCSINVNYFPFDWQNCSLKFSSLTYNAKEIN
LQLRQDQDEESQRNYPVEWIIIDPEGFTENGEWEIVHIPAKKNIDRSLTPESTKYQDITF
YLIIKRKPLFYIINILAPCVLIALMANLVFYLPADSGEKMTLAISVLLAQSVFLLLISQR
LPETSFAIPLISKYLMFIMVLVTIVVVSCVIVLNLHFRTPSTHVITERMKEIFLNKLPRI
LHMSRPAEPEPEPWSGVLLRRSSSVGYIVKAEEYYSVKSRSELMFEKQSERHGLTSRATP
ARVNPLNSNNSQDQLYGEIKPAIDGANFIVKHMRDKNDYNEEKDNWYRIARTVDRLCLFL
VTPVMIIGTLWIFLGGAYNLPPSLPFPGDPFDYNKEHKRLI

>Tetraodon nigroviridis_CHRNDa(ENSTNIP_8045)
ARGCNLSRGRNSAMHLRVCVCVSGCWCRNEEERLIHHLFKELGYNKELRPVRRHEDAVDV
QLALTLSNLISLKEVDETLLTNVWIEHTWTDYRLSWNLSAFDGISMLRLPSNMVWLPEIV
LENNNDAQFKVAYYSNVLVSSDGLCYWLPPAIFRSSCSISVKYFPFDWQNCTLKFTSLTY
NAKEIRMLLKEDALNGTAYTVEWIVIDPASFTENGEWEIIHRPAKRNTYKHIPMESNKHQ
DITFYLVIRRKPLFYVVNIIIPCVLISFLALLVYYLPADSGEKMTLSISVLLAQSVFLLL

ISQRLPETSMSVPLIVKYLMFIMVGVTVVVLNCVVVLNLHFRTPSTHVMSGWTKRLFLER
LPRLLRMSRPAETEAYWDGVLPRRSSSVGYIASAEEYYSVKSRSELMFEKQSERHGLVTR
VTHAAMKPQDEGGVTDQLYAEIKPAVDGANYIIKHMRNKNDYNEEKDNWSGIARTVDRLC
LFLVTPLMTVGTVIIFTGICNQPPHLPFKGDPHDYREENPRLL

>Tetraodon nigroviridis_CHRNDb(ENSTNIP_913)
GCWCRNEEERLIHHLFKELGYNKELRPVRRHEDAVDVQLALTLSNLISLKEVDETLLTNV
WIEHTWTDYRLSWNLSAFDGISMLRLPSNMVWLPEIVLENNNDAQFKVAYYSNVLVSSDG
LCYWLPPAIFRSSCSISVKYFPFDWQNCTLKFTSLTYNAKEIRMLLKEDALNGTAYTVEW
IVIDPASFTENGEWEIIHRPAKRNTYKHIPMESNKHQDITFYLVIRRKPLFYVVNIIIPC
VLISFLALLVYYLPADSGEKMTLSISVLLAQSVFLLLISQRLQKHPCLFLSSYLMFIMVG
VTVVVLNCVVVLNLHFRTPSTHVMSGWTKRLFLERLPRLLRMSRPAETEAYWDGVLPRRS
SSVGYIASAEEYYSVKSRSELMFEKQSERHGLVTRGRSDRSAVRRDQAGCGRSNYIIKHM
RNKNDYNEEKDNWSGIARTVDRLCLFLVTPLMTVGTVIIFTGICNQPPHLPFKGDPHDYR
EENPRLL

>Homo sapiens_CHRNA1(ENSP_261007)
MEPWPLLLLFSLCSAGLVLGSEHETRLVAKLFKDYSSVVRPVEDHRQVVEVTVGLQLIQL
INVDEVNQIVTTNVRLKQGDMVDLPRPSCVTLGVPLFSHLQNEQWVDYNLKWNPDDYGGV
KKIHIPSEKIWRPDLVLYNNADGDFAIVKFTKVLLQYTGHITWTPPAIFKSYCEIIVTHF
PFDEQNCSMKLGTWTYDGSVVAINPESDQPDLSNFMESGEWVIKESRGWKHSVTYSCCPD
TPYLDITYHFVMQRLPLYFIVNVIIPCLLFSFLTGLVFYLPTDSGEKMTLSISVLLSLTV
FLLVIVELIPSTSSAVPLIGKYMLFTMVFVIASIIITVIVINTHHRSPSTHVMPNWVRKV
FIDTIPNIMFFSTMKRPSREKQDKKIFTEDIDISDISGKPGPPPMGFHSPLIKHPEVKSA
IEGIKYIAETMKSDQESNNAAAEWKYVAMVMDHILLGVFMLVCIIGTLAVFAGRLIELNQ
QG

>Pan troglodytes_CHRNA1(ENSPTRP_21633)
MEPWPLLLLFSFCSAGLVLGSEHETRLVAKLFKDYSSVVRPVEDHRQVVEVTVGLQLIQL
INVDEVNQIVTTNVRLKQGDMVDLPRPSCVILGVPLFSHLQNEQWVDYNLKWNPDDYGGV
KKIHIPSEKIWRPDLVLYNNADGDFAIVKFTKVLLEYTGHITWTPPAIFKSYCEIIVTHF
PFDEQNCSMKLGTWTYDGSVVAINPESDQPDLSNFMESGEWVIKESRGWKHSVTYSCCPD
TPYLDITYHFVMQRLPLYFIVNVIIPCLLFSFLTGLVFYLPTDSGEKMTLSISVLLSLTV
FLLVIVELIPSTSSAVPLIGKYMLFTMVFVIASIIITVIIINTHHRSPSTHVMPNWVRKV
FIDTIPNIMFFSTMKRPSREKQDKKIFTEDIDISDISGKPGPPPMGFHSPLIKHPEVKSA
IEGIKSTTVTMKSDQESNYAAAEWKYVAMVMDHILLGVFMLVCIIGTLAVFAGRLIELNQ
QG

>Felis catus_CHRNA1(ENSFCAP_13080)
MRVFVEGGEQNLTVCVSFAVSAGLVLGSEHETRLVAKLFEDYNSVVRPVEDHRQAVEVTV
GLQLIQLINVDEVNQIVTTNVRLKQQWVDYNLKWNPEDYGGVKKIHVPSEKIWRPDFVLY
NNADGDFAIVKFTKVLLDYTGHITWTPPAIFKSYCEIIVTHFPFDEQNCSMKLGTWTYDG
SVVAINPESDQPDLSNFMESGEWVIKESRGWKHWVFYACCPTTPYLDITYHFVMQRLPLY
FIVNVIIPCLLFSFLTGLVFYLPTDSGEKMTLSISVLLSLTVFLLVIVELIPSTSSAVPL
IGKYMLFTMVFVIASIIITVIVINTHHRSPSTHVMPNWVRKVFIDTIPNIMFFSTMKRPS
REKQDKKIFTEDIDISDISGKPGPPPMGFHSPLIKHPEVKSAIEGVKYIAETMKSDQESN
NAAEEWKYVAMVMDHILLGVFMLVCIIGTLAVFAERMKTMVRITPPAPQS

>Anolis carolinensis_CHRNA1(ENSACAP_5260)
MDVCCLPLLLLLGPAVLVRCSSHETRLVSDLFSKYNKVVRPVSNHRDPVRVTVGLQLIQL
INVDEVNQIVTTNVRLKQQWMDVNLKWNPEDYGGVKQIRIPSEDIWRPDLVLYNNADGDF
AINKFTKILLNYTGHITWTPPAIFKSYCEIIVTHFPFDEQNCSMKLGTWTYDGTMVVINP
ESDRPDLSNYMGSGEWIMKDYRGWKHEVTYACCPDLPYLDITYHFVMLRLPLYFIVNVII
PCLLFSFLTGLVFYLPTDSGEKMTLSISVLLSLTVFLLVIVELIPSTSSAVPLIGKYMLF
TMVFVIASIIITVIVINTHHRNPSTHTMPQWVRKIFIDTIPNLMFFSTMKRPSKNKQEKK
IFSEDIDISEISGKQGPATVNFQSSLIKNPEVKSAIEGIKYIAETMKSDQESNNAAEEWK
FVAMVVDHLLLGIFMSVCIIGTLAVFAGRLIELHQQG

>Danio rerio_CHRNA1(ENSDARP_21860)
MNYFILILPILPYLYGPAVCSEDETRLVKTLFTGYNKVVRPVSHFKDPVVVTVGLQLIQL
ISVDEVNQIVTSNVRLKQQWKDVHLQWNPDDYGGIRKIRIPSTDLWKPDLVLYNNADGDF

AIVHETKVLLEHTGMITWTPPAIFKSYCEIVVLHFPFDLQNCSMKLGTWTYDGNLVIINP
DSDRPDLSNFMESGEWVMKDYRSWKHWVYYACCPDTPYLDITYHFLLLRLPLYFIVNVII
PCMLFSFLTGLVFYLPTDSGEKMTLSISVLLSLTVFLLVIVELIPSTSSAVPLIGKYMLF
TMIFVIASIIITVIVINTHHRSPSTHIMPAWVRKIFIDTIPNMMFFSTMKRPSQERQEKR
LFPADFDISDISGKPMPASVTYHSPITKNPDVRSAIEGVKYIADTMKSDEESNNAAEEWK
FVAMVLDHILLCVFMAVCIIGTLGVFAGRLIELSML

>Takifugu rubripes_CHRNA1(ENSTRUP_21949)
LHLCDFSSGVETLAGTCASEDETRLVKTLFTGYNKVVRPVNHFSEPVVVTVGLQLIQLIS
VDEVNQIVTSNVRLRQQWVDVNLNWNPDDYGGIRKIRVPSTDIWKPDLVLYNNADGDFAI
IHETKVLLEHTGKIIWNPPAIFKSYCEIIVLHFPFDLQNCSMKLGTWTYDGLLVVINPDS
DRPDLSNFMESGEWVLKDYRSWKHWVYYTCCPDTPYLDITYHFLMLRLPLYFIVNVIIPC
MLFSFLTGLVFYLPTDSGEKMTLSISVLLSLTVFLLVIVELIPSTSSAVPLIGKYMLFTM
VFVIASIIITVIVINTHHRSPSTHTMPPWIRKVFIETIPNMMFFSTMKRPSQEIRQKRLF
PTDMDISDISGNPTPTSVTYQSPIVKNPDVRSAIEGVKYIAETMKSDEESNNAAEEWKFV
AMVLDHILLCVFMAVCIIGTLAVFAGRLIELN

>Tetraodon nigroviridis_CHRNA1a(ENSTNIP_12737)
FTLRVSGGSLWFRAHLPERSQHFSRSPVLCSEDETRLVKTLFTGYNKVVRPVNHFSEPVV
VTVGLQLIQLINVDEVSQIVTSNVRLRQQWVDVNLNWNPDDYGGIRKIRVPSSEIWKPDL
VLYNNADGDFAIIHETKVLLEHTGKIIWNPPAIFKSYCEIIVLHFPFDVQNCSMKLGTWT
YDGLLVVINPDSDRPDLSNFMESGEWVLKDYRSWKHWVYYTCCGDTPYLDITYHFLMQRL
PLYFIVNVIIPCMLFSFLTGLVFYLPTDSGEKMTLSISVLLSLTVFLLVIVELIPSTSSA
VPLIGKYMLFTMVFVIASIIITVIVINTHHRSPSTHTMPPWIRKVFIETIPNMMFFSTMK
CPSQEIRQKRLFPTDMDISDISGNPTPTSVTYQSPIVKNPDVRSAIEGVKYIAETMKADE
ESNNAAEEWKFVAMVLDHILLCVFMAVCIIGTVAVFAGRLIELNMP

>Tetraodon nigroviridis_CHRNA1b(ENSTNIP_20072)
MNTTLFSFHIILLAGAAWASSNETRLVKNLFAGYNKVVRPVTHFKDPVVVTVGLQLIQLI
NVDEVNQIVSSNVRLKQQWKDVNLHWNPDDYGGIKKIRVPSTDIWRPDLVLYNNADGDFA
IVHETKVLLEHTGMITWNPPAIFKSYCEIIVLHFPFDLQNCSMKLGTWTYDGNLVVVNPD
SDRPDLSNFMESGEWVMKDYRGWKHWVYYACCPDTPYLDITYHFLMLRLPLYFIVNVIIP
CMLFSFLTGLVFYLPTDSGEKMTLSISVLLSLTVFLLVIVELIPSTSSAVPLIGKYMLFT
MVFVIASIIITVIVINTHHRSPSTHTMPDWVRRIFIETIPNIMFFSTMKRPGKEKQRKGI
QPPDFDISEISGNQATAVPYETRITKNPDVRSAIEGVKYIAETMKSDEESNNAAEEWKFV
AMVLDHILLCVFMAVCLVGTLGVFAGRLIELSML

>Gasterosteus aculeatus_CHRNA1a(ENSGACP_15736)
PVSCSEAETRLVKKLFTGYSKVVRPVDHFSDAVVVSVGLQLIQLISVDEVNQIVTSNVRL
KQRWRDVNLNWDPDDYGGIRKIRVPSTDIWKPDLVLYNNADGDFAIIHQTKVLLEHTGMI
TWNPPAIFKSYCEIIVLHFPFDLQNCSMKLGTWTYDGLLVVINPDSARPDLSNFMESGEW
VLKDYRSWKHTVNYSCCPNKPYLDITYHFLMLRLPLYFIVNVIIPCMLFSFLTGLVFYLP
TDSGEKMTLSISVLLSLTVFLLVIVELIPSTSSAVPLIGKYMLFTMVFVIASIIITVIVI
NTHHRSPSTHTMPAWIRKVFIETIPNMMFFSTMKRPSLEIRQMRMFPTDMDISDISGNPA
PTSDTYVSPITKNPDVRRAIEGVKYIAETMKSDEESNNAAEEWKFVAMVLDHILLCVFMA
VCIIGTLAVFAGRLIELNTL

>Gasterosteus aculeatus_CHRNA1b(ENSGACP_6143)
MARMKPVFFIFYLVILAGAAWGSSDETRLVKTLFTGYNKVVRPVNHFKDPVVVTVGLQLI
QLISVDEVNQIVSSNVRLKQQWKDVNLQWKPEDYSGIKKIRVPSTDIWRPDLVLYNNADG
DFAIVHETKVLLEHTGMITWNPPAIFKSYCEIIVLHFPFDLQNCSMKLGTWTYDGNLVVV
NPDSERPDLSNFMESGEWVMKDFRGWKHWVYYACCPDTPYLDITYHFLMLRLPLYFIVNV
IIPCMLFSFLTGLVFYLPTDSGEKMTLSISVLLSLTVFLLVIVELIPSTSSAVPLIGKYM
LFTMVFVIASIIITVIVINTHHRSPSTHTMPNWVRTVFIETIPNIMFFSTMKRPGKEKQA
KSSIYGADFDISDISGNQTTSITYQSPITKNPDVRSAIEGVKYIAETMKSDEESNNAAEE
WKFVAMVLDHILLCVFMAVCLIGTLGVFAGRLIELSML

>Oryzias latipes_CHRNA1a(ENSORLP_1705)
LGFTMYKYILKVLILFSDCCSRSVLGSEYETRLVKTLFTGYNKVVRPVTHFSQAVEVTVG
LQLIQLISVDEVNQIVTSNVRVKQQWTDVNLKWNPDDYGGIKKIRVPSTDIWKPDLVLYN
NADGDFAIIHETKVLLEYSGKITWNPPAIFKSYCEIIVLHFPFDLQNCSMKLGIWSYDGL

LVVINPESDKPDLSNFMESGEWVLKDSRCWKHWVQYTCCQDTPYLDITYHFLMLRLPLYF
IVNVIIPCLLFSFLTALVFYLPTDSGEKMTLSISVLLSLTVFLLVIVELIPSTSSAVPLI
GKYMLFTMVFVIASIIITVIVINVHHRSPSTHTMPQWIRKVFIETIPNMMVFSTMKRPGQ
EIRQRRFFPTDMDISDISGNLPPTSITYQSPIIKNPDVRSAIEGVKHIAETMKLDEESNN
AAEEWKFVAMVLDHILLCVFMGVCIIGTVAVFAGRLIELNM

>Oryzias latipes_CHRNA1b(ENSORLP_21899)
MGTLFFAFQVIILAGAAFASSDETRLVKTLFTGYNKVVRPVNHYKDPVVVTVGLQLIQLI
SVDEVNQIVSSNVRLKQQWKDVNLQWNPEDYGGIRKIRVPSTDIWRPDLVLYNNADGDFA
IVHETKVLLEHTGMITWNPPAIFKSYCEIIVLHFPFDLQNCTMKLGTWTYDGNLVVVNPD
SDRPDLSNFMESGEWVMKDYRGWKHWVYYACCQDTPYLDITYHFLMLRLPLYFIVNVIIP
CMLFSFLTGLVFYLPTDSGEKMTLSISVLLSLTVFLLVIVELIPSTSSAVPLIGKYMLFT
MVFVIASIIITVIVINTHHRSPSTHTMPDWVRKVFIETIPNLMFFSTMKRPAKEKQNKPV
YTAEFDISEISGNQATAVHYQSPLTKNPDVRSAIEGVKYIAETMKSDEESNNAAEEWKFV
AMVLDHILLCVFMGVCLIGTLGVFAGRLIELSTY

>Homo sapiens_CHRNA2(ENSP_385026)
MGPSCPVFLSFTKLSLWWLLLTPAGGEEAKRPPPRAPGDPLSSPSPTALPQGGSHTETED
RLFKHLFRGYNRWARPVPNTSDVVIVRFGLSIAQLIDVDEKNQMMTTNVWLKQEWSDYKL
RWNPTDFGNITSLRVPSEMIWIPDIVLYNNADGEFAVTHMTKAHLFSTGTVHWVPPAIYK
SSCSIDVTFFPFDQQNCKMKFGSWTYDKAKIDLEQMEQTVDLKDYWESGEWAIVNATGTY
NSKKYDCCAEIYPDVTYAFVIRRLPLFYTINLIIPCLLISCLTVLVFYLPSDCGEKITLC
ISVLLSLTVFLLLITEIIPSTSLVIPLIGEYLLFTMIFVTLSIVITVFVLNVHHRSPSTH
TMPHWVRGALLGCVPRWLLMNRPPPPVELCHPLRLKLSPSYHWLESNVDAEEREVVVEEE
DRWACAGHVAPSVGTLCSHGHLHSGASGPKAEALLQEGELLLSPHMQKALEGVHYIADHL
RSEDADSSVKEDWKYVAMVIDRIFLWLFIIVCFLGTIGLFLPPFLAGMI

>Macaca mulatta_CHRNA2(ENSMMUP_28337)
MGPSRPAFLSFTKLSLWWLLLTPAGGEEAKRPPPRAPGDPLSSPSPTALPQGGSHTQAED
RLFKHLFRGYNRWARPVPNTSDVVIVRFGLSIAQLIDVDEKNQMMTTNVWLKQEWSDYKL
RWNPADFGNITSLRVPSEMIWIPDIVLYNNADGEFAVTHMTKAHLFSTGTVHWVPPAIYK
SSCSIDVTFFPFDQQNCKMKFGSWTYDKAKIDLEQMEQTVDLKDYWESGEWAIVNATGTY
NSKKYDCCAEIYPDVTYAFVIRRLPLFYTINLIIPCLLISCLTVLVFYLPSDCGEKITLC
ISVLLSLTVFLLLITEIIPSTSLVIPLIGEYLLFTMIFVTLSIVITVFVLNVHHRSPSTH
TMPRWVRGALLGCVPRWLLMNRPLPSLELCHPPGLKLSPSYHWLETNMDAEEREVVVEEE
DRWACAGHVASSVGTLCSHGHLHSGASGPKAEAVLQEGELLLSPRMQKALEGVHYIADHL
RSEDADSSVKEDWKYVAMVIDRIFLWLFIIVCFLGTIGLFLPPFLAGMI

>Mus musculus_CHRNA2(ENSMUSP_22620)
MAPSHPAFQFWIHLYLWCLLLMPAVLAQQGSHTHAEDRLFKHLFGGYNRWARPVPNTSDV
VIVRFGLSIAQLIDVDEKNQMMTTNVWLKQEWNDYKLRWDPAEFGNITSLRVPSEMIWIP
DIVLYNNADGEFAVTHMTKAHLFFTGTVHWVPPAIYKSSCSIDVTFFPFDQQNCKMKFGS
WTYDKAKIDLEQMERTVDLKDYWESGEWAIINATGTYNSKKYDCCAEIYPDVTYYFVIRR
LPLFYTINLIIPCLLISCLTVLVFYLPSECGEKITLCISVLLSLTVFLLLITEIIPSTSL
VIPLIGEYLLFTMIFVTLSIVITVFVLNVHHRSPSTHNMPNWVRVALLGRVPRWLMMNRP
LPPMELHGSPGLKLSPTYHWLETNMDAEEREETEEEEEEEEDENICMCAGLPDSSMGVLY
GHGSLHLRAMGPEAKTPSQASEILLSPQIQKALEGVHYIADHLRSEDADSSVKEDWKYVA
MVVDRIFLWLFIIVCFLGTIGLFLPPFLAGMI

>Felis catus_CHRNA2(ENSFCAP_17705)
MGPSRSAVLSAMRLGLWWLLLSPADLMGEGPKHPPPWTPGDPTLPSCPQGASRTQAEDRL
FKHLFRGYNRWARPVPNTSDVVIVRFGLSIAQLIDVDEKNQMMTTNVWLKQEWNDYKLHW
NPADFGNITSLRVPSEMIWIPDIVLYNNADGEFAVTHMTKAHLFSTGTVHWVPPAIYKSS
CSIDVTFFPFDQQNCKMKFGSWTYDKAKIDLEQTEQTVDLKDYWESGEWAIINATGTYNT
KKYDCCAEIYPDVTYYFVIRRLPLFYTINLIIPCLLISCLTVLVFYLPSDCGEKITLCIS
VLLSLTVFLLLITDIIPSTSLVIPLIGEYLLFTMIFVTLSIVITVFVLNVHHRSPRTHTM
PHWVRVAFLGCVPRWLLMNRPVPPLEPRDPPDLKPSSSHYWLETNVDAEEREEEKEEEDR
WGWAAHVSPSMGVLHGHGGLHQGASGPRVEVQLQEGGLLLSPRIQKALEGVHYIADHLRS
EDADSSVKEDWKYVAMVIDRIFLWLFIIVCFLGTIGLFLPPFLAGMI

>Monodelphis domestica_CHRNA2(ENSMODP_19892)

LTMPPPPLKMSDVDVFVLRHASHALDEDRLFKKLFRGYNRWSRPVQNSSDVVIIRFGLSI
AQLIDVDEKNQMMTTNVWLKQEWSDYKLRWNPTDYGNITSIRVPSELIWIPDIVLYNNAD
GEFIVTHMTKAHLFFTGTVKWAPPAIYKSSCSIDVTFFPFDQQNCKMKFGSWTYDKAKID
LEQMEQMVDLKDYWESGEWAIINAVGTYNTKKYDCCVEIYPDITYFFIIRRLPLFYTINL
IIPCLLISCLTVLVFYLPSDCGEKITLCISVLLSLTVFLLLITEIIPSTSLVIPLIGEYL
LFTMIFVTLSIVITVFVLNVHHRSPGTHNMPHWVRVAFLGCIPRWLWMNRPLPVLEFHDN
PGQKLSSTHHWLDTNVDEDKKSEENWEFSGHSSPVIDTLYHHCGHHHESPCFKAGTQLPE
TEFLLPPSIQKALEGVHYIADHLRTEDADFSVKEDWKYVAMVIDRIFLWMFIIICFLGTI
GLFLPPLLA

>Danio rerio_CHRNA2a(ENSDARP_28704)
MAENRIKTLLNNLWMLILIADHVQPVLSDGWIHAHAEDKLFHKLFIGYNKWSRPVRNISD
VVIVKFGLSIAQLIDVDEKNQMMTTNVWLKQEWNDYKLRWNPSEFDNVTSIRVPSEMIWV
PDIVLYNNADGEFAVTHMTKAHLFHTGKVSWVPPAIYKSSCSIDVTFFPFDQQNCKMKFG
SWTYDKAKIDLEQIEGTVDLKDYWESGEWAIINAVGTYNTKKYDCCHEIYPDITYFFIIR
RLPLFYTINLIIPCLLISCLTVLVFYLPSDCGEKITLCISVLLSLTVFLLLITEIIPSTS
LVIPLIGEYLLFTMIFVTLSIVITVFVLNVHHRSPSTHSMPRWVHSVFLDHIPRWLFMRR
PEPEKKPKKTANLSTSKSWFQQETGVDGLVCQDMEFGIGLSTSISSPSATSLHLSDSEPL
LQKYELQHLGHVVNLCQHPAKLGNLVSEPGLLFSPCVLRALEGVRYIADHLRAEDEDFSV
KEDWKYVAMVIDRIFLWMFIIVCLLGTIGLFLPPWLSGMI

>Danio rerio_CHRNA2b(ENSDARP_119522)
MASFTNQLVFLRITLLCTIICESISCCEKTHSHAEDELFKKLFDGYNKWSRPVPNTSDVV
IVKFGLSIAQLIDVDEKNQMMTTNVWLKQEWNDYKLRWKPSDYDNVTSIRVPSELIWVPD
IVLYNNADGEFAVTHMTKAHLFHTGKVRWVPPAIYKSSCSIDVTFFPFDQQNCKMKFGSW
TYDKAKIDLERIENTVDLKDYWESGEWAIINAVGTYNTKKYDCCHEIYPDITYFFIIRRL
PLFYTINLIIPCLLISCLTVLVFYLPSDCGEKITLCISVLLSLTVFLLLITEIIPSTSLV
IPLIGEYLLFTMIFVTLSIVITVFVLNVHHRSPSTHKMPCWVHSVFLDLIPRWLFMRRPA
PDSRRRQKLLLLQRQVPGMTARPANASLSTSTSWFRGQNSGEDESCRHFSYKDLELGTLS
SAISLSLQASSPCPLGLTPPPLQKFGPAPRLEVGTASRQLSGRANVTKRLDNMIPHDNPG
FPLSPSVLQALEGVHYIADHLRAEDADFSVKEDWKYVAMVIDRIFLWMFIIVCLLGTIGL
FLPPWLAGMI

>Takifugu rubripes_CHRNA2(ENSTRUP_19923)
MKKLQSFTLCFTCHLIVLCIRSVSAVLCSQKTHSHAEDDLFKKLFAGYNKYSRPVPNVTD
VVIVKFGLSIAQLIDVDEKNQMMTTNVWLKQEWNDYKLRWRPSDYDNVTSIRVPSELIWV
PDIVLYNNADGEFAVTHMTKAHLFYNGKILWVPPAIYKSSCSIDVTFFPFDQQNCKMKFG
SWTYDKAKIDLERIENTVDLNNYWESGEWAIINAVGTYNTKKYDCCHEIYPDITYFFIIR
RLPLFYTINLIIPCLLISCLTVLVFYLPSDCGEKITLCISVLLSLTVFLLLITEIIPSTS
LVIPLIGEYLLFTMIFVTLSIVITVFVLNVHHRSPSTHKMPRWVHSVFLDLIPRWLFMRR
PAPDGRRRRLLLLQQEVMAERRRGRLTGYKSGNYLSTSANWLMDGSLSDEPNRNSYEDLE
LGRARITAQRLTKADSTVSDSSFLLSPSIIRALEGVHYIADHLRAEDADFSVKEDWKYVA
MVIDRIFLWMFIIVCLLGTIGLFLPPWLAGMI

>Tetraodon nigroviridis_CHRNA2(ENSTNIP_22729)
VLCSQKMHSHSEDALFKKLFAGYNKYSRPVPNVTDVVIVKFGLSIAQLIDVDEKNQMMTT
NVWLKQEWNDYKLRWRPSDYDNVTSIRVPSELIWVPDIVLYNNADGEFAVTHMTKAHLFH
NGKIRWVPPAIYNSSCIIDVTFFPFDQHTARCKFGPCRYDKAKLTCYWESGEWAIINAVG
TYNTKKYDCCHEIYPDITYFFIIRRLPLFYTINLIIPCLLISCLTVLVFYLPSDCGEKIT
LCISVLLSLTVFLLLITEIIPSTSLVIPLIGEYLLFTMIFVTLSIVITVFVLNVHHRSPS
THKMPRWVHSVFLDLIPRWLFMRRPAPEGRRRRLLLLQQPPSPRPLGTSPPRQQNSQKKQ
EVPAGTNQQLSGARTTAQRLTKADSTGSDSYFLLSPSIIRALEGVHYIADHLRAEDADFS
VKEDWKYVAMVIDRIFLWMFIIVCLLGTIGLFLPPWLAGMI

>Gasterosteus aculeatus_CHRNA2(ENSGACP_14086)
YTIKYEPLRMILFNVSAVLCHEKSHSHAEDDLFKTLFAGYNKWSRPVSNISDVVIVKFGL
SIAQLIDVDEKNQMMTTNVWLKQEWNDYKLRWKPSDYDNVTSIRVPSELIWVPDIVLYNN
ADGEFAVTHMTKAHLFHNGKVRWVPPAIYKSSCSIDVTFFPFDQQNCKMKFGSWTYDKAK
IDLERTENTVDLNNYWESGEWAIINAVGTYNTKKYDCCHEIYPDITYFFIIRRLPLFYTI
NLIIPCLLISCLTVLVFYLPSDCGEKITLCISVLLSLTVFLLLITEIIPSTSLVIPLIGE
YLLFTMIFVTLSIVITVFVLNVHHRSPSTHKMPHWVHSVFLNLIPRWLFMRRPAPDVRRR

RLALMQQDAALVRRQGRAGGYKRPSNCLSTSATWSGGSRVNLTQRAVKADNAVSDSTFLL
SPSVIRALEGVHYIADHLRAEDADFSVKEDWKYVAMVIDRIFLWMFIIVCLLGTIGLFLP
PWLA

>Oryzias latipes_CHRNA2(ENSORLP_11046)
FCYDKSHAHAEEELFQTLFTGYNKWSRPVPNISDVVIVKFGLSIAQLIDVDEKNQMMTTN
VWLKQEWNDYKLRWKPSDYDNVTSIRVPSELIWVPDIVLYNNADGEFAVTHMTKAHLFHT
GKVRWVPPAIYKSSCSIDVTFFPFDQQNCKMKFGSWTYDKAKIDLERIENTVDLNNYWES
GEWAIINAVGTYNTKKYDCCHEIYPDITYFFIIRRLPLFYTINLIIPCLLISCLTVLVFY
LPSDCGEKITLCISVLLSLTVFLLLITEIIPSTSLVIPLIGEYLLFTMIFVTLSIVITVF
VLNVHHRSPSTHKMPRWVHTVFLDLIPRWLFMRRPAPNGRRRRLLLLQQDGALGRLQPRV
SALDISTSSTWLREGAVLEKTERSCYEDLELGTLQSYFSFRPPSPRPPGQTPPQQLKTPQ
NSQSWREGGGGPNRQMGGARVDSPKVQCAESGSLLSPCVMRALEGVHYIADHLRAEDADF
SVKEDWKYVAMVIDRIFLWMFIIVCLLGTIGLFLPPWLAGMI

>Homo sapiens_CHRNA6(ENSP_276410)
MLTSKGQGFLHGGLCLWLCVFTPFFKGCVGCATEERLFHKLFSHYNQFIRPVENVSDPVT
VHFEVAITQLANVDEVNQIMETNLWLRHIWNDYKLRWDPMEYDGIETLRVPADKIWKPDI
VLYNNAVGDFQVEGKTKALLKYNGMITWTPPAIFKSSCPMDITFFPFDHQNCSLKFGSWT
YDKAEIDLLIIGSKVDMNDFWENSEWEIIDASGYKHDIKYNCCEEIYTDITYSFYIRRLP
MFYTINLIIPCLFISFLTVLVFYLPSDCGEKVTLCISVLLSLTVFLLVITETIPSTSLVV
PLVGEYLLFTMIFVTLSIVVTVFVLNIHYRTPTTHTMPRWVKTVFLKLLPQVLLMRWPLD
KTRGTGSDAVPRGLARRPAKGKLASHGEPRHLKECFHCHKSNELATSKRRLSHQPLQWVV
ENSEHSPEVEDVINSVQFIAENMKSHNETKEVEDDWKYVAMVVDRVFLWVFIIVCVFGTA
GLFLQPLLGNTGKS

>Pongo abelii_CHRNA6(ENSPPYP_20814)
MPTSKGQGFLHGGLCLWLCVFTPFFKGCAGCVTEERLFHKLFSHYNQFIRPVENVSDPVT
VHFEVAITQLANVDEVNQIMETNLWLRHIWNDYKLRWDPTEYDGIETLRVPADKIWKPDI
VLYNNAVGDFQVEGKTKALLKYNGMITWTPPAIFKSSCPMDITFFPFDHQNCSLKFGSWT
YDKAEIDLLIIGSKVDMNDFWENSEWEIIDASGYKHDIKYNCCEEIYTDITYSFYIRRLP
MFYTINLIIPCLFISFLTVLVFYLPSDCGEKVTLCISVLLSLTVFLLVITETIPSTSLVV
PLVGEYLLFTMIFVTLSIMVTVFVLNIHYRTPTMHTMPRWVKTVFLKLLPQVLLMRWPVD
KTRGTGSDAMPRGLARRPAKGKLASREEPRHLKECFHCHKSNELATSKRRLSHQPLQWMV
ENSEHSPEVEDVINSVQFIAENMKSHNETKQVEDDWKYVAMVVDRVFLWVFIIVCVFGTA
GLFLQPLLGNTGKS

>Mus musculus_CHRNA6(ENSMUSP_33882)
MLNSRDQGNLHSGLCLWLCGFLALFKGSTGCESEEQLFHRLFAHYNRFIRPVENVSDPVT
VHFELAITQLANVDEVNQIMETNLWLRHIWKDYRLRWDPTEYDGIETLRVPADNIWKPDI
VLYNNAVGDFQVEGKTKALLKYDGVITWTPPAIFKSSCPMDITFFPFDHQNCSLKFGSWT
YDKAEIDLLIIGSKVDMNDFWENSEWEIVDASGYKHDIKYNCCEEIYTDITYSFYIRRLP
MFYTINLIIPCLFISFLTVLVFYLPSDCGEKVTLCISVLLSLTVFLLVITETIPSTSLVI
PLVGEYLLFTMIFVTLSIVVTVFVLNIHYRTPATHTMPKWVKTIFLQAFPSILMMRKPLD
KTKEAGGVKDPKSHTKRPAKVKFTHRGESKLLKECHHCQKSSDIAPGKRRSSQQPARWVA
ENSEHSSDVEDVIESVQFIAENMKSHNETNEVEDDWKYMAMVVDRVFLWVFIIVCVFGTV
GLFLQPLLGNTGKS

>Canis familiaris_CHRNA6(ENSCAFP_8128)
MLTSKGQGFLHFGLCLCSCIFIPCLKGSAGCASEERLFHKLFSHYNQFIRPVENVSDPVT
VHFEVAITQLANVDEVNQIMETNLWLRHIWNDYKLRWNPMEYDGIETLRVPADRIWKPDI
VLYNNAVGDFQVEGKTKALLKYDGMIIWTPPAIFKSSCPMDITFFPFDHQNCSLKFGSWT
YDKAEIDLLIIGSKVDMNDFWENSEWEIVDASGYKHDIKYNCCEEIYTDITYSFYIRRLP
MFYTINLIIPCLFISFLTVLVFYLPSDCGEKVTLCISVLLSLTVFLLVITETIPSTSLVI
PLVGEYLLFTMIFVTLSIAVTVFVLNIHYRTPATHTMPKWVKTVFLQLLPQILMMRRPLD
KMRKTSSDKNSKGISSRPTKVNFDNYRETKLPKECCHCHKSSKLATSKRRLSHQSLQWMT
ENSEHWPDVEDVINSVQFIAENMKNQNETKEVEDDWKYVAMVVDRVFLWVFIFVCVFGTA
GLFLQPLLGNTGKS

>Myotis lucifugus_CHRNA6(ENSMLUP_4768)
MLTSKGQGCLHCGLCLWLYIFIPFFKDGAGCASEERLFHKLFSHYNQLIRPVENVSEPVT

VHFEVAITQLANVDEVNQIMETNLWLRHIWNDYKLRWDPTEFDGIETIRVPADKIWKPDI
VLYNNAVGDFQVKGKTKALLKYDGMITWTPPAIFKSSCPMDITFFPFDHQNCSLKFGSWT
YDKAEIDLLIIGSKVDMNDFWENSEWEIVDASGYKHDIKYNCCEEIYTDITYSFYIRRLP
MFYTINLIIPCLFISFLTVLVFYLPSDCGEKVTLCISVLLSLTVFLLVITETIPSTSLVI
PLVGEYLLFTMIFVTLSIAVTVFVLNIHYRTPTTHTMPDWVKMVFLQLLPQILMMKRPLD
KTRKTSSDKNPKSISGRPTKVKFDNRREPKLLKECCHCHKSHQLATSKRRLSHQPLQWMT
GNTEHSPEVEDVINSVQFIAENMKNQNETKEVEDDWKYVAMVVDRVFLWIFIIVCVFGTA
GLFLQPLLGNTGKS

>Homo sapiens_CHRNB3(ENSP_289957)
MLPDFMLVLIVLGIPSSATTGFNSIAENEDALLRHLFQGYQKWVRPVLHSNDTIKVYFGL
KISQLVDVDEKNQLMTTNVWLKQEWTDHKLRWNPDDYGGIHSIKVPSESLWLPDIVLFEN
ADGRFEGSLMTKVIVKSNGTVVWTPPASYKSSCTMDVTFFPFDRQNCSMKFGSWTYDGTM
VDLILINENVDRKDFFDNGEWEILNAKGMKGNRRDGVYSYPFITYSFVLRRLPLFYTLFL
IIPCLGLSFLTVLVFYLPSDEGEKLSLSTSVLVSLTVFLLVIEEIIPSSSKVIPLIGEYL
LFIMIFVTLSIIVTVFVINVHHRSSSTYHPMAPWVKRLFLQKLPKLLCMKDHVDRYSSPE
KEESQPVVKGKVLEKKKQKQLSDGEKVLVAFLEKAADSIRYISRHVKKEHFISQVVQDWK
FVAQVLDRIFLWLFLIVSVTGSVLIFTPALKMWLHSYH

>Callithrix jacchus_CHRNB3(ENSCJAP_14325)
MLTDFMLVLIVLGIPSSATAGFSSIAENEDALLRHLFQGYQKWVRPVLHSNDTIKVYFGL
KISQLVDVDEKNQLMTTNVWLKQEWTDHKLRWNPDDYGGIHSIKVPSESLWLPDIVLFEN
ADGRFEGSLMTKVIVKSNGTVVWTPPASYKSSCTMDVTFFPFDRQNCSMKFGSWTYDGTM
VDLILINENVDRKEFFDNGEWEILNAKGMKGNRRDGVYSYPFITYSFVLRRLPLFYTLFL
IIPCLGLSFLTVLVFYLPSDEGEKLSLSTSVLVSLTVFLLVIEEIIPSSSKVIPLIGEYL
LFIMIFVTLSIIVTVFVINVHHRSSSTYQPMAPWVKRLFLQKLPKLLCMKDHVDRYSIPE
KEESQPVVKSKVLEKKKHKQLSDGEKVLVAFLEKAADSIRYISSHVKKEHFISQVVQDWK
FVAQVLDRIFLWLFLIVSVTGSVLIFTPALKMWLHSYH

>Gallus gallus_CHRNB3(ENSGALP_24774)
MLCLMLCVLCWSRSDVAALGSVAENEDALLRHLFQGYQKWVRPVENSNDTIKVLFGLKIS
QLVDVDEKNQLMTTNVWLKQEWMDHKLSWNPEEYGGITAIRVPSESLWLPDIVLFENADG
RFEGSLMTKAIVKYNGVVTWMPPASYKSSCTMDVTFFPFDRQNCSMKFGSWTYDGSMVDL
ILVDENVDRKDFFDNGEWEILNAKGMKGNRKDGLYSYPFVTYSFVLRRLPLFYTLFLIIP
CLGLSFLTVLVFYLPSDEGEKLSLSTSVLVSLTVFLLVIEEIIPSSSKVIPLIGEYLLFI
MIFVTLSIIVTVFVINVHHRSSATYHPMAPWVKRLFLQKLPRLLCMKGHVDRYSFSDTEE
KETTLKSKLPGKQKHKQAKDGEKVVIAFLEKAADSIRYISRHVKKEHFIRQVVQDWKFVA
QVLDRIFLWLFLVVSVTGSVLIFTPALQMWLNSTL

>Xenopus tropicalis_CHRNB3(ENSXETP_22596)
TMKMFIYACTNIIFIPSGFAKFHPLAEIEDALLRKLFAGYQKWVRPVLNPNSTIKVNFGL
KISQLVDVDEKKQLMTTNVWLRQEWMDMKLRWNPKDYGGITYIRVPSESLWLPDIVLFEN
ADGRFEGSLMTKAIIKYDGTVTWTPPASYKSSCTMDVTFFPFDRQNCSMKFGSWTYDGNM
VDLILLDENVDRKDFFDNGEWDILNATGLKGMRKDGLYSYPFITYSFVLRRLPLFYTLFL
IIPCLGLSFLTVLVFYLPSDEGEKLSLSTSVLVSLTVFLLVIEEIIPSSSKVIPLIGEYL
LFIMIFVTLSIIVTVFVINVHHRSSATYHPMAPWVKRLFLEKLPKLLCMKGHVDRFSFFD
SESDVSSAKSKPSGKRKNIHASDGEKVLIAFLEKAADSIKYISTHVKKEHFIRQVVQDWK
FVAQVLDRIFLWLFLSVSITGSVLIFTPALQMWLNSHV

>Danio rerio_CHRNB3a(ENSDARP_69164)
MKLQISGILLVAAVAYATIEAPEEFVSLAEMEDTLLRNLFRGYQKWVRPVLHANDTITVR
FGLKISQLVDVDEKNQLMTTNVWLWEEWTDYKLRWNPEDYGGITSIRVPSETIWLPDIVL
YENADGRFEGSLMTKAIVRFNGTIMWTPPASYKSACTMDVTFFPFDRQNCSMKFGSWTYD
GTMVDLTLVDAYVDRKDFFDNGEWEILNATGQRGSRRDGIYSYPYLTYSFILKRLPLFYT
LFLIIPCLGLSFLTVLVFYLPSDEGEKLSLSTSVLVSLTVFLLVIEEIIPSSSKVIPLIG
EYLLFIMIFVTFSIIVTVFVINVHHRSSATYHPMAPWVKSLFLQRLPRLLCMRGHTDRYQ
YPDIELRSPELKRGMKKGQQKSAGGGRAGVKEDENQAWIALLEKATHSVHYISRHIRKEH
FIREVVQDWKFVAQVLDRIFLWAFLTVSVLGTILIFTPALQMFFSTS

>Danio rerio_CHRNB3b(ENSDARP_50036)
MTLTVIGLFTLFTSIFATTPAREFVSLAEREDALLRDLFQGYQRWVRPILHANHTVKVRF

GLKISQLVDVDEKNQLMTTNVWLYEEWLDYKLRWNPENYGGITFIRVPSESIWLPDIVLY
ENADGRFEGSIMTKAIVRYNGLITWTPPASYKSACTMDVTFFPFDRQNCSMKFGSWTYDG
NMVELVLVNHQVDRSDFFDNGEWEILSATGHKGSRRDGHYSYPYITYSFILKRLPLFYTL
FLIIPCLGLSFLTVLVFYLPSDEGEKVSLSTSVLVSLTVFLLVIEEIIPSSSKVIPLIGE
YLLFIMIFVTLSIIVTVFVINVHHRSSATYHPMSPWVRTLFLQRLPDLLCMRGNTDRYHY
PQMEPQSPDLKPRSKKGPPGPEGDGQALINMLEQATNSVRYISRHIKKEHFIREVVQDWK
FVAQVLDRIFLWAFLTVSVLGTILIFTPAVKMFLSTPPPPSP

>Takifugu rubripes_CHRNB3a(ENSTRUP_15529)
PPLLSVLLSVPIPLAPAEEDFVSLAELEDSLLRNLFKGYQKWVRPVQHANDTITVRFGLK
ISQLVDVDEKNQLMTTNVWLWQEWIDVKLKWNPDDYGGITAIRVPSETIWLPDIVLYENA
DGRFEGSLMTKAIVRWDGTITWTPPASYKSACNMDVTFFPFDRQNCSMKFGSWTYDGNMV
DLVLVDHYVDRKDFFDNGEWEILNATGVKGSRRDEVYWYPFVTYSFILKRLPLFYTLFLI
IPCLGLSFLTVLVFYLPSDEGEKLSLSTSVLVSLTVFLLVIEEIIPSSSKVIPLIGEYLL
FIMIFVTFSIIVTVFVINVHHRSSATYHPMAPWVKSLFLQRLPRLLCMRGHTDRYNFPDN
EMRSPEPKPRRAIGRRGVQGKEDENQAWLAMLEKATNSVRYISQHIRKEHFIREVVQDWK
FVAQVLDRIFLWAFLTVSILGTVLIFTPALKMYLHKMFH

>Takifugu rubripes_CHRNB3b(ENSTRUP_4037)
HLPDQLVCAKTSLSAPAQSEFVSLAEMEDALLRNLFQGYQRWVRPIQHANDTIKVRFGLK
ISQLVDVDEKNQLMTTNVWLCQEWIDFKLRWNPDKYGGITSIRVPSENLWLPDIVLYENA
DGRFEGSLMTKAIVRYNGAITWTPPASYKSACTMDVTFFPFDRQNCTMKFGSWTYDGTMV
DLILIDKQVDRKDFFDNGEWEILSATGAKGNRKDGLYSYPFITYSFILKRLPLFYTLFLI
IPCLGLSFLTVLVFYLPSDEGEKLSLSTSVLVSLTVFLLVIEEIIPSSSKVIPLIGEYLL
FIMIFVTLSIIVTVFVINVHHRSSATYHPMSPWVRTLFLQKLPSLLCMRGHTDRYHYPEL
GPESPEPKPRSGGRRGGQRISSGGTYGQASSEGKDDEDWATMLDKAIFSVRYISRHIRKE
HFIREVVQDWKFVAQVLDRIFLWAFLTVSVLGTILIFTPALQKFMQVLP

>Tetraodon nigroviridis_CHRNB3a(ENSTNIP_16128)
LLVASVCFFPVFHMFAPAQSEFVSLAEMEDALLRNLFQGYQRWVRPIQHANDTIKVRFGL
KISQLVDVDEKNQLMTTNVWLCQEWIDYKLRWNPDKYGGITSIRVPSENLWLPDIVLYEN
ADGRFEGSLMTKAIVRYNGAITWTPPASYKSACTMDVTFFPFDRQNCTMKFGSWTYDGTM
VDLILIDKQVDRKDFFDNGEWEILSATGAKGNRKDGLYSYPFITYSFILKRLPLFYTLFL
IIPCLGLSFLTVLVFYLPSDEGEKLSLSTSVLVSLTVFLLVIEEIIPSSSKVIPLIGEYL
LFIMIFVTLSIIVTVFVINVHHRSSATYHPMSPWVRTLFLQKLPRLLCMRGHTDRYHYPE
LGPESPEPRVRAGGRRGGQRVGSGSHGQTPPEGKDDEEWASMLDKAIYSVRYISRHIRKE
HFIREVVQDWKFVAQVLDRIFLWAFLTVSVLGTILIFTPALQKFMKVLPQSSS

>Tetraodon nigroviridis_CHRNB3b(ENSTNIP_14999)
QEDFVSLAELEDSLLRNLFRGYQKWVRPVQHANDTITVRFGLKISQLVDVDEKNQLMTTN
VWLWQEWVDVKLKWNPDDYGGITAIRVPSETIWLPDIVLYENADGRFEGSLMTKAIVRWD
GTITWTPPASYKSACTMDVTFFPFDRQNCSMKFGSWTYDGNMVDLVLVDHYVDRKDFFDN
GEWEILNATGVKGSRRDGIYWYPFVTYSFILKRLPLFYTLFLIIPCLGLSFLTVLVFYLP
SDEGEKLSLSTSVLVSLTVFLLVIEEIIPSSSKVIPLIGEYLLFIMIFVTFSIIVTVFVI
NVHHRSSATYHPMAPWVKSLFLQRLPRLLCMRGHTDRYHFPDMEMHSPELKPRRAVGRRG
VQGQQKGPLAGKEDENQAWLAMLEKATNSVRYISQHIRKEHFIREVVQDWKFVAQVLDRI
FLWAFLTVSILGTVLIFTPALQMYLSTP

>Gasterosteus aculeatus_CHRNB3a(ENSGACP_24319)
QDDVVSLAEMEDSLLRNLFRGYQKWVRPVLHSNDTITVCFGLKISQLVDVDEKNQLMTTN
VWLWQEWVDLKLKWNPDDYGGITSIRVPSETIWLPDIVLYENADGRFEGSLMTKAIVRWD
GTITWTPPASYKSACTMDVTFFPFDRQNCSMKFGSWTYDGNMVDLVLVDHYVDRKDFFDN
GEWEILNATGQRGSRRDEVYWYPFVTYSFILKRLPLFYTLFLIIPCLGLSFLTVLVFYLP
SDEGEKLSLSTSVLVSLTVFLLVIEEIIPSSSKVIPLIGEYLHHRSSSTYHPMAPWVKSL
FLQRLPRLLCMRGHTDRYHFEDMEMHSPEVKTRRGVGRRGAPGQRGPVSGKEEENRAWLA
MLEKATSSVRYISRHIKKEHFIREVVQDWKFVAQVLDRILLWAFLTVSILGTVLIFTPAL
QMYFSTP

>Gasterosteus aculeatus_CHRNB3b(ENSGACP_23418)
MKWTVLFLLLVCPLSLAEIHAPSEFVSLAEMEDALMRNLFQGYQRWVRPIQHANDTVRVR
FGLKISQLVDVDEKNQLMTTNVWLWQEWIDYKLRWNPDKYGGITSIRVPSEIIWLPDIVL

YENADGRFEGSLMTKAIVKFNGVITWTPPASYKSACTMDVTFFPFDRQNCTMKFGSWTYD
GHMVDLVLIDNQVDRRDFFDNGEWEILSATGARGTRKDDMYTYPFLTYSFILKRLPLFYT
LFLIFPCLGLSFLTVLVFYLPSDEGEKLSLSTSVLVSLTVFLLVIEEIIPSSSKVIPLIG
EYLLRSSATYHPMSPWVRNLFLQKLPRFLCMRGHTDLYHYPELAPESPEMKPRSGGRKGG
QRANSGARGQTASEGKEDEAWATMLDKAVYSVRYISRHIRKEHFIREVVQDWKFVAQVLD
RIFLWAFLTVSILGTVLIFTPALYSFFKIP

>Oryzias latipes_CHRNB3a(ENSORLP_17917)
FPLLPVQENVLSLAELEDSLLRNLFKGYQKWVRPVQHANDTITVRFGLKISQLVDVDEKN
QLMTTNVWLWQEWIDVKLKWNPDDYGGITSIRVPSETIWLPDIVLYENADGRFEGSLMTK
AIVRWDGTITWTPPASYKSSCTMDVTFFPFDRQNCSMKFGSWTYDGNMVDLVLVDHYVDR
KDFFDNGEWEILNATGQRGSRRDEMYWYPFLTYSFILKRLPLFYTLFLIIPCLGLSFLTV
LVFYLPSDEGEKLSLSTSVLVSLTVFLLVIEEIIPSSSKVIPLIGEYLLFIMIFVTFSII
VTVFVINVHHRSSATYHPMAPWVKSLFLQKLPRLLCMRGHTDRYHFPDIEMRSPELKTRR
GAGRRAVPGGMEDDSQAWLAMLEKATNSVHYISRHIRKEHFIREVVQDWKFVAQVLDRIF
LWAFLTVSILGTVLIFTPALQSYLSTP

>Oryzias latipes_CHRNB3b(ENSORLP_4018)
LTVFLLLLLSACPQLFAAITAPKRFVSLAEMEDDLLKNLFQGYQRWVRPIQRPNDTVRVR
FGLKISQLVDVDEKNQLMTTNVWLRQEWIDYKLRWNPDKYGGITSIRVPSENIWLPDIVL
YENADGKFEGSLMTKAIVKFNGAVTWMPPASYKSACTMDVTFFPFDRQNCSMKFGSWTYD
GNMVDLVLMDSQVDRKDFFDNGEWEILSATGAKGNRKDGLYFYPFITCSFILKRLPLFYT
LFLIIPCLGLSFLTVLVFYLPSDEGEKLSLSTSVLVSLTVFLLVIEEIIPSSSKVIPLIG
EYLLFIMIFVTLSIIVTVFVINVHHRSSATYHPMSPWVRSLFLQKLPRLLCMRGHADRYH
YPELAPESPALKSRSGGRRGIQRMSSGGQQSTYDGKEEEAYSTMLEKAIYSVRYISRHIR
KDHFIREVVQDWKFVAQVLDRIFLWIFLSVSVLGTILIFTPALQMFMKIPPPAPSE

>Homo sapiens_CHRNA4(ENSP_359285)
MELGGPGAPRLLPPLLLLLGTGLLRASSHVETRAHAEERLLKKLFSGYNKWSRPVANISD
VVLVRFGLSIAQLIDVDEKNQMMTTNVWVKQEWHDYKLRWDPADYENVTSIRIPSELIWR
PDIVLYNNADGDFAVTHLTKAHLFHDGRVQWTPPAIYKSSCSIDVTFFPFDQQNCTMKFG
SWTYDKAKIDLVNMHSRVDQLDFWESGEWVIVDAVGTYNTRKYECCAEIYPDITYAFVIR
RLPLFYTINLIIPCLLISCLTVLVFYLPSECGEKITLCISVLLSLTVFLLLITEIIPSTS
LVIPLIGEYLLFTMIFVTLSIVITVFVLNVHHRSPRTHTMPTWVRRVFLDIVPRLLLMKR
PSVVKDNCRRLIESMHKMASAPRFWPEPEGEPPATSGTQSLHPPSPSFCVPLDVPAEPGP
SCKSPSDQLPPQQPLEAEKASPHPSPGPCRPPHGTQAPGLAKARSLSVQHMSSPGEAVEG
GVRCRSRSIQYCVPRDDAAPEADGQAAGALASRNTHSAELPPPDQPSPCKCTCKKEPSSV
SPSATVKTRSTKAPPPHLPLSPALTRAVEGVQYIADHLKAEDTDFSVKEDWKYVAMVIDR
IFLWMFIIVCLLGTVGLFLPPWLAGMI

>Mus musculus_CHRNA4(ENSMUSP_66338)
MEIGGSGAPPPLLLLPLLLLLGTGLLPASSHIETRAHAEERLLKRLFSGYNKWSRPVANI
SDVVLVRFGLSIAQLIDVDEKNQMMTTNVWVKQEWHDYKLRWDPGDYENVTSIRIPSELI
WRPDIVLYNNADGDFAVTHLTKAHLFYDGRVQWTPPAIYKSSCSIDVTFFPFDQQNCTMK
FGSWTYDKAKIDLVSMHSRVDQLDFWESGEWVIVDAVGTYNTRKYECCAEIYPDITYAFI
IRRLPLFYTINLIIPCLLISCLTVLVFYLPSECGEKVTLCISVLLSLTVFLLLITEIIPS
TSLVIPLIGEYLLFTMIFVTLSIVITVFVLNVHHRSPRTHTMPAWVRRVFLDIVPRLLFM
KRPSVVKDNCRRLIESMHKMANAPRFWPEPESEPGILGDICNQGLSPAPTFCNRMDTAVE
TQPTCRSPSHKVPDLKTSEVEKASPCPSPGSCHPPNSSGAPVLIKARSLSVQHVPSSQEA
AEGSIRCRSRSIQYCVSQDGAASLTESKPTGSPASLKTRPSQLPVSDQTSPCKCTCKEPS
PVSPITVLKAGGTKAPPQHLPLSPALTRAVEGVQYIADHLKAEDTDFSVKEDWKYVAMVI
DRIFLWMFIIVCLLGTVGLFLPPWLAGMI

>Monodelphis domestica_CHRNA4(ENSMODP_21042)
MRLQGPQGSALLLLLLRGLLILGVLLPASGHVETRAHAEERLLKKLFSGYNKWSRPVANI
SDVVLVRFGLSIAQLIDVDEKNQMMTTNMWVKQEWQDYKLRWDPMDYENVTSIRIPSELI
WRPDIVLYNNADGDFAVTHLTKAHLFYDGRIKWTPPAIYKSSCSIDVTFFPFDQQNCTMK
FGSWTYDKAKIDLVSMHSHVDQLDYWESGEWVIISAVGNYNIKKYECCTETYSDITYSFI
IRRLPLFYTINLIIPCLLISCLTVLVFYLPSDCGEKITLCISVLLSLTVFLLLITEIIPS
TSLVIPLIGEYLLFTMIFVTLSIIITVFVLNVHHRSPRTHTMPAWVRRVFLDIIPRLLFM
KRPGVVKDNCRKLVESMHKLAGGPRFWPQPEPNFVTSPSTSPQTPERSPSASFCVHLEDP

VKPQAVCKSPSGQYTVLQRPEPGKGSPCSPPTPHPPLNNSHPSGFSKVRSLSVQQMYSSN
KVEEGSIRCRSRSIQYCSLQEDSSQANNGQSTSSPASLRSQKDEVQPQEKPSPCKCKCKK
VKATPVPDPVTKPRSTKEQHALPMSPALKLAVEGVQYIADHLKAEDTDFSVKEDWKYVAM
VIDRIFLWMFVIVCLLGTVGLFLPPWLAGMI

>Danio rerio_CHRNA4(ENSDARP_22694)
MRAAWRVFFLLCVHLQLAYVFSMSPAHAHAEERLLQSLFINYNKLSRPVANISDVVLVHF
GLSIAQLIDVDEKNQMMTTNVWVKQEWNDYKLRWNPEEYENVTSIRIPSEIIWRPDIVLY
NNADGDFAVTHLTKAQVFYNGRIKWKPPAIYKSSCSIDVTFFPFDQQNCKMKFGSWTYDR
AKIDLISMASDVDQMDYWESGEWVIINAVGTYNIKKYECCTEIYPDITYSFIIRRLPLFY
TINLIIPCLLISCLTVLVFYLPSECAEKITLCISVLLSLTVFLLLITEIIPSTSLVIPLI
GEYLLFTMIFVTLSIIITVFVLNVHHRSSRTHSMPHWVRQLFLHLVPRYLFMKRPPASGK
RNCGKLIEMMHKQANSQSMFLRNNILDISPQSPIPHLGPTSAAQQDQVQQDSTSPKPLFF
CSSPSSQYSILQEENSQTPQTPVLSYTCAYTNSIFASSTTSFPDTLLGTLLHTIPQDGAK
STECDFQGHSAESVFMEAKESVLGAGSCQHCLTSEGTSLQKLDLDEAGRCTRHNMPSAQI
FSPSSDGSTDLNAAEPVSNLSQSLLKALEGVQYIADHLRAEDSDFSVREDWKYVAMVIDR
IFLWMFVLVCILGTVGLFLPPWLAGMI

>Takifugu rubripes_CHRNA4(ENSTRUP_25718)
TEWISSGLDPPALPHHLHFIWITSSSLAGFSQVAPRAHAEERLLQDLFAHYNKLSRPVEN
TTDTVLVHFGLSIAQLIDVDEKNQMMTTNVWVKQEWNDYKLRWNPEEYENVTSIRIPSEI
IWRPDIVLYNNADGDFAVTHLTKAQLFYDGRIKWMPPAIYKSSCSIDVTFFPFDQQSCKM
KFGSWTYDRAKIDLISMASDVDQMDYWESGEWVIVNAVGKYNTKKYECCTEIYSDITYYF
IIRRLPLFYTINLIIPCLLISCLTVLVFYLPSQCGEKITLCISVLLSLTVFLLLITEIIP
STSLVIPLIGEYLLFTMVFVTLSIIITVFVLNVHHRSPQTHGMPHWVRRVFLDFVPKVLF
MKRPPGTAKQHCKKLIEMMHRPATVSGPSPSPPSSPLANCNKDNQSLNANTFCQSISGRY
SLLSEKQLLHGHKSAASSSSQLSLPPTLPLGLLHKRTNKESNTMASIGRSLSVEHMFDGQ
KEAPCRGRYRRRSRSFQYCCLHDEGAGLTRATWGQKKHPSADHLVEIIMAESTKDTSSLQ
DFEVSTISPAMQRAIEGVQYIADHLRAEDADFSVKEDWKYVAMVIDRIFLWMFVLVCILG
SVGLFLPPWLAGMI

>Tetraodon nigroviridis_CHRNA4(ENSTNIP_6787)
IQVAPRARRRRLLRDLFAHYNRLSRPVENTTDTVLVHFGLSIAQLIDVDEKNQMMTTNVW
VKQEWNDYKLRWNPEEYENVTSIRIPSEIIWRPDIVLYNNADGDFAVTHLTKAQLFYDGR
IKWMPPAIYKSSCSIDVTFFPFDQQSCKMKFGSWTYDRAKIDLVSMASDVDQMDYWESGE
WVIVNAVGKYNIKKYECCTEIYSDITYYFIIRRLPLFYTINLIIPCLLISCLTVLVFYLP
SQCGEKITLCISVLLSLTVFLLLITEIIPSTSLVIPLIGEYLLFTMVFVTLSIIITVFVL
NVHHRSPQTHGMPHWVRRVFLDFVPKVLFMKRPPGTAKQHCKKLIEMMHRPATVSEVENS
SSFWTGLETELRQMGQVQKTIPKALTDSPSIRISSPSPPSSPLVNCNKGNQSLNANTFCQ
SISGQYSLLSEKKLLHGHKTAAFSSSQLSLPPTLPLGRLHNHTDEESNTIALNGRSLSVE
HMFDDRKEASRPGGYRRRSRSFQYCCLHDEGVGLTRTTGEMKKQPSADHLGETIMAESTK
DTSSLQNIELSTISPAMQRAIEGVQYIADHLRAEDSDFSVKEDWKYVAMVIDRIFLWMFI
LVCILGSVGLFLPPWLAGMI

>Gasterosteus aculeatus_CHRNA4(ENSGACP_6142)
LPPLLSYLVANLCLSRTHVRPRAHAEERLLQDLFARYNKLSRPVENTSDTVLVHFGLSIA
QLIDVDEKNQMMTTNVWVKQEWSDYKLRWKPEDYENVSSIRIPSEIIWRPDIVLYNNADG
DFAVTHLTKAHLFHDGRIKWMPPAIYKSSCSIDVTFFPFDQQSCKMKFGSWTYDRAKIDL
ISMASDVDQMDYWESGEWVIINAVGKYNTKKYECCAEIYSDITYYFIIRRLPLFYTINLI
IPCLLISCLTVLVFYLPSQCGEKITLCISVLLSLTVFLLLITEIIPSTSLVIPLIGEYLL
FTMVFVTLSIVITVFVLNVHHRSPQTHSMPHWVRRVFLDMVPRVLFMKRPPGTAKQHCKK
LIEMMHRPTAISATGNSLAYWTGLETGLRQMCQAENALPKTPCDSPSIHVCSPAPPSSPP
EDCEEDDRLLKANLSLPPALPLGPLRSFSQEEPNAMAPNGRSLSAEQMFDQQGDLSQRAG
HRCRSRSFQYCCLHGEGPGLMAVAGWVKKQAPTNHPAGRVTGESTKDASTQQDIVIPTIS
PAVQRAIEGVQYVADHLRAEDADFSVKEDWKYVAMVIDRIFLWMFVLVCILGSVGLFLPP
WLAGMI

>Oryzias latipes_CHRNA4(ENSORLP_1058)
SSQIEPRAHAEERLLQNLFARYNKLSRPVQNTSDTVLVHFGLSIAQLIDVDEKNQMMTTN
VWLKQEWNDYKLRWNPEDYENVTSIRIPSEIIWRPDIVLYNNHSADGDFAVTHLTKAHLF
YDGQIKWMPPAIYKSSCSIDVTFFPFDQQSCKMKFGSWTYDRAKIDLISMASDVDQMDYW

ESGEWVIVNAVGKYNTKKYECCAEIYADITYYFIIRRLPLFYTINLIIPCLLISCLTVLV
FYLPSQCGEKITLCISVLLSLTVFLLLITEIIPSTSLVIPLIGEYLLFTMVFVTLSIIIT
IFVLNVHHRSPQTHGMPHWVRRVFLDLVPRVLFMKRPPGTAKQHCKRLIEMMHCPTTISA
TGNSQSFWTGLDSELRQMTQVHNSLPKVDSPRILVCSPSPSSSPVDDCSDEDHPLKADIF
CQTPSGQYSPLLEHQALCRHISTTHSAPQLSMPLGPFPCLSREHPSPLARNGRSLSVEQM
CGKQGQPPQRSAHRCRSQSFQYCCLHNDGAAGFTGQTNKTPEQLTETLAAEFAKDASTQL
DNMAPIVSPAMQRAIEGVQYIADHLRAGDADFSVKEDWKYVAMVIDRIFLWMFVLVCILG
SVGLFLPPWLAGMI

>Homo sapiens_CHRNA3(ENSP_315602)
MGSGPLSLPLALSPPRLLLLLLLSLLPVARASEAEHRLFERLFEDYNEIIRPVANVSDPV
IIHFEVSMSQLVKVDEVNQIMETNLWLKQIWNDYKLKWNPSDYGGAEFMRVPAQKIWKPD
IVLYNNAVGDFQVDDKTKALLKYTGEVTWIPPAIFKSSCKIDVTYFPFDYQNCTMKFGSW
SYDKAKIDLVLIGSSMNLKDYWESGEWAIIKAPGYKHDIKYNCCEEIYPDITYSLYIRRL
PLFYTINLIIPCLLISFLTVLVFYLPSDCGEKVTLCISVLLSLTVFLLVITETIPSTSLV
IPLIGEYLLFTMIFVTLSIVITVFVLNVHYRTPTTHTMPSWVKTVFLNLLPRVMFMTRPT
SNEGNAQKPRPLYGAELSNLNCFSRAESKGCKEGYPCQDGMCGYCHHRRIKISNFSANLT
RSSSSESVDAVLSLSALSPEIKEAIQSVKYIAENMKAQNEAKEIQDDWKYVAMVIDRIFL
WVFTLVCILGTAGLFLQPLMAREDA

>Oryctolagus cuniculus_CHRNA3(ENSOCUP_4351)
MGAPPPPPPSPVLLLLLLLLPPVVSASEAEHRLFERLFEDYNEIIRPVANVSDPVIIQFE
VSMSQLVKVDEVNQIMETNLWLKQIWNDYKLKWNPSDYNGAEFMRVPAQKIWKPDIVLYN
NAVGDFQVDDKTKALLKYTGEVTWIPPAIFKSSCKIDVTYFPFDYQNCTMKFGSWSYDKA
KIDLVLIGSSMNLRDYWESGEWAIIKAPGYKHDIKYNCCEEIYTDITYSLYIRRLPLFYT
INLIIPCLLISFLTVLVFYLPSDCGEKVTLCISVLLSLTVFLLVITETIPSTSLVIPLIG
EYLLFTMIFVTLSIVITVFVLNVHYRTPTTHTMPAWVKTVFLNFLPRVMFMTRPASDGDS
AQMPQLLAGAELSNLGCFGRTEPKACKEGCPCQDGACGYRHHRRVKMSNFGANLTRSSSL
ESVDAVLSLSALSPEIREAIRSVQYIAENMKAQNEAKEIQDDWKYVAMVIDRIFLWVFIL
VCILGTAGLFLQPLMARDDA

>Taeniopygia guttata_CHRNA3(ENSTGUP_3582)
GCGGSEAEHRLYAALFKSYNQFVRPVKNVSDPVIIQFEVSMSQLVKVDEVNQIMETNLWL
KHIWNDYKLRWNPADYGGAEFIRVPSGQIWKPDIVLYNNAVGDFQVDDKTKALLKYTGDV
TWIPPAIFKSSCKIDVTYFPFDYQNCTMKFGSWSYDKAKIDLVLIGSTMNLKDYWESGEW
AIIKAPGYKHDIKYNCCEEIYPDITYSLYIRRLPLFYTINMIIPCLLISFLTVLVFYLPS
DCGEKVTLCISVLLSLTVFLLVITETIPSTSLVIPLIGEYLLFTMIFVTLSIVITVFVLN
VHYRTPKTHTMPVWVKTIFLNLLPRIMFMTRPASDEENNQKPKPLFTSEFSNLNCINGSE
TKSCKDGFACQDMACSCCQYQRTNSESVDPMLSFSVLSPEMRDAIESVKYIAENMKMQNE
AKEIQDDWKYVAMVIDRIFLWVFILVCILGTAGLFLQPLM

>Pelodiscus sinensis_CHRNA3(ENSPSIP_19032)
QTSAPLSLPSTAFSKEVSLLILPATLSVSNCSEAEHRLFASLFANYSQFIRPVENVSDPA
IIQFEVSMSQLVKVDEVNQIMETNLWLKHIWNDYKLKWNPEEYGGAEFIRVPSDSIWKPD
IVLYNNAVGDFQVDDKTKALLKYTGEVTWIPPAIFKSSCKIDVTYFPFDYQNCTMKFGSW
SYDKAKIDLVLIGSTMNLKDYWESGEWAIIKAPGYKHDIKYNCCEELYPDITYSLYIRRL
PLFYTINMIIPCLLISFLTVLVFYLPSDCGEKVTLCISVLLSLTVFLLVITETIPSTSLV
IPLIGEYLLFTMIFVTLSIVITVFVLNVHYRTPKTHTMPEWVKTIFLNLLPKIMFMTRPA
SDEETTQKLKPLYSVELSDLNCFSSSETKCCKEGFICQDTACSCCRYQRIKFSDFSGNLT
RSSSYESVDALLSLSVLSPEIRDAIESVKYIAENMKMQNEAKEIQDDWKYVAMVIDRIFL
WVFILVCILGTAGLFLQPLMTRDEI

>Danio rerio_CHRNA3(ENSDARP_132461)
MQLIHFIIASTIQLCVSVSGVCCSEAEHRLFSVIFSSYNQYIRPVENVSDPVVVQFEVSM
SQLVKVDEVNQIMETNLWLRHIWNDYKLRWDPKDFGGVEFIRVPSNKIWKPDIVLYNNAV
GDFQVDDKTKALLRFNGDVTWIPPAIFKSSCKIDVTYFPFDYQNCTMKFGSWTYDKAKID
LVLIGSTINLKDFWESGEWTIIDAPGYKHDIKYNCCEEIYTDITYSLYIRRLPLFYTINM
IIPCLLISFLTVLVFYLPSDCGEKVTLCISVLLSLTVFLLVITETIPSTSLVIPLIGEYL
LFTMIFVTLSIVITVFVLNVHYRTPKTHTMPCWVRRVFLSLLPRVMFMTRPEKDQEVPAK
HKQTSVCRGPQLLLCPTELNEASRTAFLCREHHCWKKHISNMTSEGAEDGGSPCSSSESL
DGFLCMSAVSPQVREAIESVKYIAENMRLQNEAKEVQDDWKYVAMVIDRIFLWVFVLVCI

LGTAGLFLQPLLLGEDM

>Takifugu rubripes_CHRNA3(ENSTRUP_9649)
LLFLLPGGCCSEAEHKLFSVIFSNYNQYIRPVENVTDPVIVQFEVSMSQLVKVDEVNQIM
ETNLWLRHIWNDYKLRWPKEYGGVEYIRVPSSRIWKPDIVLYNNAVGDFQVDDKTKALLR
YNGDVTWIPPAIFKSSCKIDVTYFPFDYQNCTMKFGSWTYDKAKIDLVLIGSTMNLKDFW
ETGDWLIIDAPGYKHDIKYNCCEEIYTDITYSLYIRRLPLFYTINLIIPCLLISFLTVLV
FYLPSDCGEKVTLCISVLLSLTVFLLVITETIPSTSLVIPLIGEYLLFTMIFVTLSIVIT
VFVLNVHYRTPKTHTMPCWVRAVFLELLPRVMFMTRPERHAFQVLRRPLSNTELSNLNNL
SGDPSKGSTSLCCEGRCNCCWYQRSRGSVAKVSGGSQRSSSESLNGGLMPLLPLSPEVRE
AIESVRYIAENMRLQNEAKEVQDDWKYVAMVIDRIFLWVFVLVCILGTAGLFLQPLLLAE
DI

>Tetraodon nigroviridis_CHRNA3(ENSTNIP_2840)
LLFRLPGGSCSEAEHKLFSVIFSNYNQYIRPVENVTDPVIVQFEVSMSQLVKVDEVNQIM
ETNLWLRHIWNDYKLRWNPKEYGGVEYIRVPSSRIWKPDIVLYNNAVGDFQVDDKTKALL
RYNGDVTWIPPAIFKSSCKIDVTYFPFDYQNCTMKFGSWTYDKAKIDLVLIGSTMNLKDF
WETGDWLIIDAPGYKHDIKYNCCEEIYTDITYSLYIRRLPLFYTINLIIPCLLISFLTVL
VFYLPSDCGEKVTLCISVLLSLTVFLLVITETIPSTSLVIPLIGEYLLFTMIFVTLSIVI
TVFVLNVHYRTPKTHTMPRWVRAVFLELLPRVMFMIRPEREPEQGRGAGNAQDMHPRSCS
VHSSELSNLNNLSGDLTKGSTSLCCEGRCNCCWYQRSKVSGGSQCSSSDSLNGGLMPQLP
LSPEVREAIESVRYIAENMRLQNEAKEVQDDWKYVAMVIDRIFLWVFVLVCILGTAGLFL
QPLLLAEDI

>Gasterosteus aculeatus_CHRNA3(ENSGACP_21875)
AEHKLFSVIFSSYNQYIRPVENVTDPVVVQFEVSMSQLVKVDEVNQIMETNLWLRHIWND
YKLRWNPKDFGGVDFIRVPSNRIWKPDIVLYNNAVGDFQVDDKTKALLRFNGDVTWIPPA
IFKSSCKIDVTYFPFDYQNCTMKFGSWTYDKAKIDLVLIGSTINLKDFWESGEWTIIDAP
GYKHDIKYNCCEEIYTDITYSLYIRRLPLFYTINMIIPCLLISFLTVLVFYLPSDCGEKI
TLCISVLLSLTVFLLVITETIPSTSLVIPLIGEYLLFTMIFVTLSIVITVFVLNVHYRTP
KTHTMPCWVRTVFLGLLPRVMFMTRPETDAERVLAAAGDVQTTHLRPCAIHPSGALQKQH
KPQAPPPPSVVSSLTNRQRLFNNTELSDLNNLSGDPAKGAGSALLCCKGHCNCCWRQRSS
KLPADSGGGSGGLGSMGALSGGSAVGGGSHCSSSESVAGGTMSMLPLSPEVQEAIESVKY
IAENMRLQNEAKEVQDDWKYVAMVIDRIFLWVFVLVCILGTAGLFLQPLLLWDDI

>Homo sapiens_CHRNB4(ENSP_261751)
MRRAPSLVLFFLVALCGRGNCRVANAEEKLMDDLLNKTRYNNLIRPATSSSQLISIKLQL
SLAQLISVNEREQIMTTNVWLKQEWTDYRLTWNSSRYEGVNILRIPAKRIWLPDIVLYNN
ADGTYEVSVYTNLIVRSNGSVLWLPPAIYKSACKIEVKYFPFDQQNCTLKFRSWTYDHTE
IDMVLMTPTASMDDFTPSGEWDIVALPGRRTVNPQDPSYVDVTYDFIIKRKPLFYTINLI
IPCVLTTLLAILVFYLPSDCGEKMTLCISVLLALTFFLLLISKIVPPTSLDVPLIGKYLM
FTMVLVTFSIVTSVCVLNVHHRSPSTHTMAPWVKRCFLHKLPTFLFMKRPGPDSSPARAF
PPSKSCVTKPEATATSTSPSNFYGNSMYFVNPASAASKSPAGSTPVAIPRDFWLRSSGRF
RQDVQEALEGVSFIAQHMKNDDEDQSVVEDWKYVAMVVDRLFLWVFMFVCVLGTVGLFLP
PLFQTHAASEGPYAAQRD

>Danio rerio_CHRNB4(ENSDARP_139554)
XGVCADSEERLMNWLLGKHRYNKLIRPALNRTERVTVLLQVSLAQLISVNEREQIMTTNV
WLTQNWNDYRLSWDPAEYEGIDKLRIPSRHIWLPDIVLYNNADGTYEVTVFTNAIVLFNG
SIAWLPPAIYKSACKIEVKHFPFDQQNCTLKFRSWTYDHTEIDLLLKSDAASMDDFTPSG
EWDILALPGRRTVSPLDATYVDLTYDFIIKRKPLFYTINLIIPCVLITSLAILVFYLPSD
CGEKMTLCISVLLALTVFLLLISKIVPPTSLDVPLIGKYLMFTMVLVTFSIITSVCVLNV
HHRSPSTHSMPAWVKIVFLVRLPALLFMQRPRGHSARQRLRQNRRLLGLADTPGRSVMCS
SASALLAASSASALSSPGLLYNKGHFGSRRAEGLNNGHGSAQDLKGNRLYCEWGSEIQEA
VDGVRYVADHMMGNDDDQSVIEDWKYVAMVVDRLFLWIFVIVCVVGTLGLFLQPLFQNQT
LAIQQPNTEPARRDGM

>Takifugu rubripes_CHRNB4(ENSTRUP_9641)
LWGRLMTVLSLNSLIYHGVPSGCRCADSEERLMNWLLGKNRYNPLIRPASNRTERVPVKL
QVSLAQLISVNEREQIMTTNLWLMQHWVDYRLSWDPAKYEGINKLRIPSRLIWLPDIVLY
NNADGTYEVTVFTNAIVLFNGSINWLPPAIYKSACKIEVKHFPFDQQNCTLKFRSWTYDR

TEIDLVLKTDAASMDDFTPSGEWDILALPGRRTVNPLDPTYVDLTYDFIIKRKPLFYTIN
LIIPCILITSLAILVFYLPSDCGEKMTLCISVLLALTVFLLLISKIVPPTSLDVPLIGKY
LMFTMVLVTFSIITSVCVLNVHHRSPSTHTMPSWVKLIFLVKLPSLLFIRRPQNNSARQR
LQSQRCQRAKRDILGLGCQPKSSITMLSSALLSPGSVFSTPGQKNVAPSLSLNITQNLQQ
SSGADWAADVQEALSGVRFVAEHMMEDDDDQSVIEDWKYVAMVVDRMFLWIFVIVCVVGT
LGLFLQPVFQNPITPIQQPSSDM

>Gasterosteus aculeatus_CHRNB4(ENSGACP_21862)
FGCEYTTCAFKLWNINVSPGSRCAESEERLMNWLLGKDRYNLLIRPAVNRTERVPVKLQV
SLAQLISVNEREQIMTTNVWLTQHWVDYRLSWDPAKYEGIDKLRIPSRHIWLPDIVLYNN
ADGTYEVTVFTNAIVIFNGSINWLPPAIYKSACKIEVKHFPFDQQNCTLKFRSWTYDHTE
IDLILKSEVASMDDFTPSGEWDILALPGRRTVNPLDPTYVDLTYDFIIKRKPLFYTINLI
IPCVLITSLAILVFYLPSDCGEKMTLCISVLLALTVFLLLISKIVPPTSLDVPLIGKYLM
FTMVLVTFSIITSVCVLNVHHRSPSTHTMPSWVKLIFLVKLPALLFMRRPQNNSASQRLR
RQRCLRARKSIGSSGYPDAAKTGFILSPSALKGDLRSTDYIPSGQDLKQSTSSDWAADVQ
EAVNGVRFVAEHMMGDDDDQSVIEDWKYVAMVVDRMFLWIFVIVCVVGTLGLFLQPVFQT
QIVPNQQ

>Oryzias latipes_CHRNB4(ENSORLP_16438)
CKSADSEERLMNMLLGKHRYNPLIRPAINRTERVEVKLQVSLAQLISVNEREQIMTTNVW
ITQHWVDYRLSWDPAKYEGIQKLRIPSRHVWLPDIVLYNNADGIYEVTVFTNVIVLFNGS
VNWLPPAIYKSACKIEVKHFPFDLQNCTLKFRSWTYDHTEIDLILKSEAASMDDFTPSGE
WDIIALPGRRTVNPTDPTYVDLTYDFIIKRKPLFYTINLIIPCILITSLAILVFYLPSDC
GEKMTLCISVLLALTVFLLLISKIVPPTSLDVPLIGKYLMFTMVLVTFSIITSVCVLNVH
HRSPSTHTMPSWVKLIFLKKLPALLFMKRPNNFSGCQRLRQQRCLRARRSILGLDYPNAS
KACFTLSSSALLPSDSAFSTPRHKNLAPTMGYGLSRRKGDLSSSDYPHISLTPTQDYQQK
CKSDWTADVKEAVKGVRFVAEHMMADDDDQSVIEDWKYVAMVVDRMFLWIFVIVCVVGTL
GLFLQPLFQSQMITSQQPSSDKPRI

>Homo sapiens_CHRNB1(ENSP_304290)
MTPGALLMLLGALGAPLAPGVRGSEAEGRLREKLFSGYDSSVRPAREVGDRVRVSVGLIL
AQLISLNEKDEEMSTKVYLDLEWTDYRLSWDPAEHDGIDSLRITAESVWLPDVVLLNNND
GNFDVALDISVVVSSDGSVRWQPPGIYRSSCSIQVTYFPFDWQNCTMVFSSYSYDSSEVS
LQTGLGPDGQGHQEIHIHEGTFIENGQWEIIHKPSRLIQPPGDPRGGREGQRQEVIFYLI
IRRKPLFYLVNVIAPCILITLLAIFVFYLPPDAGEKMGLSIFALLTLTVFLLLLADKVPE
TSLSVPIIIKYLMFTMVLVTFSVILSVVVLNLHHRSPHTHQMPLWVRQIFIHKLPLYLRL
KRPKPERDLMPEPPHCSSPGSGWGRGTDEYFIRKPPSDFLFPKPNRFQPELSAPDLRRFI
DGPNRAVALLPELREVVSSISYIARQLQEQEDHDALKEDWQFVAMVVDRLFLWTFIIFTS
VGTLVIFLDATYHLPPPDPFP

>Gorilla gorilla_CHRNB1(ENSGGOP_22459)
MTPGALLMLLGALGAPLAPGVRGSEAEGRLREKLFSGYDSSVRPAREVGDRVRVSVGLIL
AQLISLNEKDEEMSTKVYLDLEWTDYRLSWDPAEHDGIDLLRITAESVWLPDVVLLNNND
GNFDVALDISVVVSSDGSVRWQPPGIYRSSCSIQVTYFPFDWQNCTMVFSSYSYDSSEVS
LQTRLGPDGQGHQEIHIHEGTFIATSSLTSYLPGQYIIVIEKSVFEWPGNMITDYAIELS
FSSALQGEKMGLSIFALLTLTVFLLLLADKVPETSLSVPIIIKYLMFTMVLVTFSVILSV
VVLNLHHRSPHTHQMPLWVRQIFIHKLPLYLRLKRPKPERDLMPEPPHCSSPGSDWGRGT
DEYFIRKPPSDFLFPKPNRFQPELPAPDLRRFIDGPNRAVALLPELREVVSSISYIARQL
QEQEDHDALKEDWQFVAMVVDRLFLWTFIIFTSVGTLVIFLDATYHLPPPDPFP

>Mus musculus_CHRNB1(ENSMUSP_47270)
MALGALLLLLGVLGTPLAPGARGSEAEGQLIKKLFSNYDSSVRPAREVGDRVGVSIGLTL
AQLISLNEKDEEMSTKVYLDLEWTDYRLSWDPAEHDGIDSLRITAESVWLPDVVLLNNND
GNFDVALDINVVVSFEGSVRWQPPGLYRSSCSIQVTYFPFDWQNCTMVFSSYSYDSSEVS
LKTGLDPEGEERQEVYIHEGTFIENGQWEIIHKPSRLIQLPGDQRGGKEGHHEEVIFYLI
IRRKPLFYLVNVIAPCILITLLAIFVFYLPPDAGEKMGLSIFALLTLTVFLLLLADKVPE
TSLAVPIIIKYLMFTMVLVTFSVILSVVVLNLHHRSPHTHQMPFWVRQIFIHKLPPYLGL
KRPKPERDQLPEPHHSLSPRSGWGRGTDEYFIRKPPSDFLFPKLNRFQPESSAPDLRRFI
DGPTRAVGLPQELREVISSISYMARQLQEQEDHDALKEDWQFVAMVVDRLFLWTFIVFTS
VGTLVIFLDATYHLPPPEPFP

>Danio rerio_CHRNB1(ENSDARP_92302)
MKAQDWIIVTCCLCGLGAMTGATEMEDKLMKKLFSAYNSKVRPARNPEERVVVRIGMILS
SFVSLNMKDEEMNTIVMMNLEWNDYRLSWNPKDYGGVDVLRISSGKVWLPDIVLINNNDG
VFGVALQVHVQAYSNGRVTWTPPALYKSSCGVKVTYFPFDWQNCTMVFRSYTYDSSEVDL
QHALDKRGKEIKEIILDTGFSESGEWHIRHKVSRKNVREDLYEDITFYLIIERKPMFYVV
NIVLPCILITIIAIFNFYLPPDAGEKMGLSINVLLTLTVFLLLLANKIPETSLGVPIIVN
YLMITMILVTFSVILSVVVLNLHHRSPNTHQMPLWVRKIFIHMLPPYLGMLRPKVETPLF
LEKPAKKENIQAISRVADEYFIRKPSNMNFLFPKPHRYQPDGQCTDLRKFIDGPSHYLTL
PPELKTAVEAITYIAEQLQAEKDYEALKEDWQYVAMVADRLFLWIFVIFTTLGTLGIFTD
ASFNATPTDPFP

>Takifugu rubripes_CHRNB1a(ENSTRUP_10238)
STMKYLLLLPLSCCLCSLIPLGGASEVEKTLMKTLFSNYTLKVRPAISPEQRVVVRVGMI
LSSFVGLNMKNEEMSTIVVMNLEWTDYRLSWSPKQYGGIEVLRIPSGKVWLPDIVLINNN
DGVFHVALHVHVQVYSNGRVTWTPPALYCSSCGVKVEYFPFDWQNCSMQFRSYTYDSTEI
DVQYALNLRGQEIREIQLDEAFTEGGEWYIKHRPSRKVMLDDEYEGMVFYLIIERKPLYY
VLNIILPCILITIIAIFNFYLPPDAGEKMGLSINVLLTLTVFLLLLADKIPETSLGVPII
VNYIMLTMILVTFSVILSVVVLNLHHRSPNTHQMPMWVRKIFIHLLPPYLCMMRPKVEAP
LYLEALPRREKKVTTVSKVADEYFIRKPDMSVLFPKPNRFHSEGLCTDLRKFIDGPSSYL
SLPEELKSAIEAITYIAEALQAEKDYEALKKDWQYVAMVVDRMFLWIFVIFTTVGTLAIF
AEASFNRAPANPFP

>Takifugu rubripes_CHRNB1b(ENSTRUP_10240)
TSYLLFTQLIFGVLLLLAGASEVEKTLMKTLFSNYTLKVRPAISPEQRVVVRVGMILSSF
VGLNMKNEEMSTIVVMNLEWTDYRLSWSPKQYGGIEVLRIPSGKVWLPDIVLINNNDGVF
HVALHVHVQVYSNGRVTWTPPALYCSSCGVKVEYFPFDWQNCSMQFRSYTYDSTEIDVQY
ALNLRGQEIREIQLVYNISPEGGEWYIKHRPSRKVMLDDEYEGMVFYLIIERKPLYYVLN
IILPCILITIIAIFNFYLPPDAGEKMGLSINVLLTLTVFLLLLADKIPETSLGVPIIVNY
IMLTMILVTFSVILSVVVLNLHHRSPNTHQMPMWVRKIFIHLLPPYLCMMRPKVEAPLYL
EALPRREKKVTTVSKVADEYFIRKPDMSVLFPKPNRFHSEGLCTDLRKFIDGPSSYLSLP
EELKSAIEAITYIAEALQAEKDYEALKKDWQYVAMVVDRMFLWIFVIFTTVGTLAIFAEA
SFNRAPANPFP

>Tetraodon nigroviridis_CHRNB1(ENSTNIP_8624)
AFEVESALLKNLFSNYTLKVRPAVSPEQRVVVRVGMILSSFVGLNMKNEEMSTIVVMNLE
WTDYRLSWSPKDYDGIEVLRIPSGKIWLPDIVLINNNDGVFEVALQVHVQVYSNGRVTWT
PPALYCSSCGVKVEYFPFDWQNCSMQFRSYTYDSTEIDIQYALDSKGREIKEIQLDEAYT
EGGEWYIKHRPSRKVMLDDEYEGMVFYLIIERKPLYYVLNIILPCILITIIAIFNFYLPP
DAGEKMGLSINVLLTLTVFLLLLADKPETSLGVPIIVNYIMFTMILVTFSVILSVVVLRL
HHRSHNTHQMPMWVRRIFIHLLPPYLCMMRPQVETPLYLEVLPRREKKIPASSKVADEYF
IRKPDLSVLFPKPNRFHSDGLCTDLRKFIDGPSSYLSLPEELKSAIEAITYIAEALQAEK
DYEALKKDWQYVAMVVDRMFLVLFVIFTTVGTLAIFTQASFNRSPGNPFPVTPDTWFL

>Gasterosteus aculeatus_CHRNB1(ENSGACP_26349)
PLCPTGASDMEKTLLKKLFTDYNLKVRPASSPEERVVVRVGMVLSSFVGLNMKNEEMSTI
VVMNLEWTDHRLSWKREEYDGIEVMRIPAGKVWLPDIVLINNNDGVFDVALHVHVQAYSN
GRVTWTPPALYCSSCGVKVTYFPFDWQNCTMQFQSYTYDSTEIDVQYALDSKRNEIREIQ
LDEAYTEGGEWTIKHKPCRKNMNDDQYEVMTFYLIIERKPLYYVLNIILPCILITIIAIF
NFYLPPDAAPGEKMGLSINVLLTLTVFLLLLADKIPETSLGVPIIVNYIMFTMILVTFSV
ILSVVVLNLHHRSPSTHHMMPMWVRKIFIHLLPPYLYMLRPKVETPLSLEFMPRREKKVFT
ISKADEYFIRKPDSSILFPKPNRFHPEGMCTDLRKYIDGPSSYLTLPDELKSAIEAITFI
AEALQAEKDFEALREDWQYVAMVVDRMFLWIFVIFTTVGTLAIFADASFHRTPTDPFK

>Oryzias latipes_CHRNB1(ENSORLP_4440)
MTLLFPVGHLGVPYRSRLLGAAQVEQALMKSLFSDYNLKVRPAHVPESRVVVRVGMILSS
FVGLNMKNEEMSTTVVMNLEWTDYRLTWNPKQHDGIKMLRVPSGKVWLPDIVLINNNDGV
FDVALRVHVLVYSDGRITWTPPALYLSSCGVKVTYFPFDWQNCTMQFRSYTYDSTEIDLQ
YALDSKGKEIREIQLDEGFSESGEWHIRHKPSRKIMKDDLYEEMTFYLIIERKPLYFNFY
LPPDAGEKMGLSINVLLTLTVFLLLLADKIPETSLGVPIIVKYIMFTMILVTCSVILSVV
VLNLHHRSPNTHMMPMWIRKIFLHMLPPYLFMRRPKQENLQYLESVPRREMKVMAISKAA
DEYFIRKPDSSILFPKPNRFHSDGMYTDLRKFIDGPSTYLALPDELKTAIDAITYIAEAL

QAEKDYEALKEDWQFVAMVVDRMFLWIFVVFTTLGTLAIFIDASFNHTPKDPFK

>Homo sapiens_CHRNE(ENSP_293780)
MARAPLGVLLLLGLLGRGVGKNEELRLYHHLFNNYDPGSRPVREPEDTVTISLKVTLTNL
ISLNEKEETLTTSVWIGIDWQDYRLNYSKDDFGGIETLRVPSELVWLPEIVLENNIDGQF
GVAYDANVLVYEGGSVTWLPPAIYRSVCAVEVTYFPFDWQNCSLIFRSQTYNAEEVEFTF
AVDNDGKTINKIDIDTEAYTENGEWAIDFCPGVIRRHHGGATDGPGETDVIYSLIIRRKP
LFYVINIIVPCVLISGLVLLAYFLPAQAGGQKCTVSINVLLAQTVFLFLIAQKIPETSLS
VPLLGRFLIFVMVVATLIVMNCVIVLNVSQRTPTTHAMSPRLRHVLLELLPRLLGSPPPP
EAPRAASPPRRASSVGLLLRAEELILKKPRSELVFEGQRHRQGTWTAAFCQSLGAAAPEV
RCCVDAVNFVAESTRDQEATGEEVSDWVRMGNALDNICFWAALVLFSVGSSLIFLGAYFN
RVPDLPYAPCIQP

>Mus musculus_CHRNE(ENSMUSP_99616)
MAGALLGALLLLTLFGRSQGKNEELSLYHHLFDNYDPECRPVRRPEDTVTITLKVTLTNL
ISLNEKEETLTTSVWIGIDWHDYRLNYSKDDFAGVGILRVPSEHVWLPEIVLENNIDGQF
GVAYDSNVLVYEGGYVSWLPPAIYRSTCAVEVTYFPFDWQNCSLIFRSQTYNAEEVEFIF
AVDDDGNTINKIDIDTAAFTENGEWAIDYCPGMIRRYEGGSTEGPGETDVIYTLIIRRKP
LFYVINIIVPCVLISGLVLLAYFLPAQAGGQKCTVSINVLLAQTVFLFLIAQKIPETSLS
VPLLGRYLIFVMVVATLIVMNCVIVLNVSLRTPTTHATSPRLRQILLELLPRLLGSSPPP
EDPRTASPARRASSVGILLRAEELILKKPRSELVFEGQRHRHGTWTAALCQNLGAAAPEI
RCCVDAVNFVAESTRDQEATGEELSDWVRMGKALDNVCFWAALVLFSVGSTLIFLGGYFN
QVPDLPYPPCIQP

>Bos taurus_CHRNE(ENSBTAP_42804)
MAGALLCALLLLQLLGRGEGKNEELRLYHYLFDTYDPGRRPVQEPEDTVTISLKVTLTNL
ISLNEKEETLTTSVWIGIDWQDYRLNYSKGDFGGVETLRVPSELVWLPEIVLENNIDGQF
GVAYEANVLVSEGGYLSWLPPAIYRSTCAVEVTYFPFDWQNCSLVFRSQTYNAEEVEFVF
AVDDEGKTISKIDIDTEAYTENGEWAIDFCPGVIRRHDGDSAGGPGETDVIYSLIIRRKP
LFYVINIIVPCVLISGLVLLAYFLPAQAGGQKCTVSINVLLAQTVFLFLIAQKTPETSLS
VPLLGRYLIFVMVVATLIVMNCVIVLNVSLRTPTTHAMSPRLRYVLLELLPQLLGSGAPP
EIPRAASPPRRASSLGLLLRAEELILKKPRSELVFEQQRHRHGTWTATLCQNLGAAAPEI
RCCVDAVNFVASSTRDQEATGEEVSDWVRMGKALDSICFWAALVLFLVGSSLIFLGAYFN
RVPQLPYPPCM

>Danio rerio_CHRNE(ENSDARP_116514)
MAVAERFGRTCVVAIALLATVLQVTGNEESQLIADKFKNYSKNIRPARHADEKVKVQVKL
TLTNLISLNEKEETLTTNVWIEIQWHDYRLAWNTSEYHDIEQIRVPYYTVWLPDIVLENN
IDGKFDVAYYANVLISSDGSMYWLPPAIYRSTCAIEITYFPFDWQNCTLVFRSQTYSANE
IDIVLAEDENGKPIEWVDIDPEAFTENGEWAIKHRPARKLTNQRYSPDDLEYQEVYYNII
IQRKPLFYVINIIILPCSLISSLVVLAYFLPAKAGGQKLTVSISVLLAQTVFLILISQKIP
ETSLSVPLIGKYLIFVMSMTTLIVTNCIIVLNYSLRSPSTHNMSQSIKHIFLEVVPRYLG
MAPFVDEGDQGGVYEMRERRRSSFGLMQRAEEYVLKQPRSEMMFDKQRERHGLMRTIVDE
IDVSSTANLYKSLAKTAPEIKECVDACNFIAESTKQQNDTGSEMESWVLIGKMIDKVCFW
AAISLFSIGTIAIFLMGHYNQAPEYPFAWENKKYVPE

>Takifugu rubripes_CHRNE(ENSTRUP_41477)
SLLGSQSLVSLFRAVQSNEETELIKDLFKDYNKNIRPVVHPEDKVEVQIKLTLTNLISLN
EKEETLTTNVWIEIQWLDYRLSWNESKYYGIQVIRVPCKTVWLPDIVLENNIDGKFDVAY
YANVLISSNGWMYWLPPAIYRSTCSIEITYFPFDYQNCTLAFRSQTYSANEVDLILALTE
ENGTIEWVDIDPEAFTENGEWAIVHRPARKMVNMRYSPDDLEYQEILFNLVIQRKPLFYI
INLILPCSLISSLVVLAYFLPAQAGGQKLTVSISVLLAQTVFLFLIAQKVPETSLSVPLI
GKYLIFVMCVTTLIATNQIVVLNFSLRSPNTHTMSQTIKHVFLEMVPRFLCMPPLVDESE
ATTEVNGIRERRRSSFGLMQRAEEYVLKQPRSEMMFDKQRERHGLTTANLYKSLAQAAPE
IKQCVDACNFIADSKRQQNTIGSEIESWVLIGKMIDKVCFWAAILLFIIGTVAIFLMGHF
NRAPDFPFPGQNKK

>Tetraodon nigroviridis_CHRNE(ENSTNIP_14726)
NEETELIKDLFKDYNKKIRPVVHPEDKVEVQIKLTLTNLISLNEKEETLTTNVWIEIQWI
DYRLSWNESEYYGINIIRVPCKTVWLPDIVLENNIDGKFDVAYYANVLVSSNGGMYWLPP
AIYRSTCAIEITYFPFDYQNCTLAFRSQTYSANEVELILTIGEKNETIEWVDIDPEAFTE

NGEWAIVHRPARKVINTRYSPDDLEYQEIMFNLVIQRKPLFYIINVILPCSLISSLVVLA
YFLPAQAGGQKLTVSISVLLAQTVFLFLIAQKVPETSLSVPLIGKYLIFVMCVTTLIATN
QIVVLNFSLRSPSTHTMSQTIKHVFLELVPRLLCMPPLVDEGEATAVVNGIRERRRSSFG
LMQRAEEYVLKQPRSEMMFDKQRERHGLTRSIVDNIDVTSTANLYKSLAQAAPEIKQCVD
ACNFIADSIRQQNTIGSEIENWVLIGKMIDKVCFWAAILLFIIGTVAIFFMGHFNRAPDF
PFPGEDKKYVPS

>Gasterosteus aculeatus_CHRNE(ENSGACP_26955)
MAESRFWIFFGVVAVLGTLTARVQCNEESELIRDLFKGYNKNIRPVVHPEDKLTIQIKLT
LTNLISLNEKEETLTTNVWIEIQWVDYRFVWNTSNYHGIDVIRVPCKTIWLPDIVLENNI
DGKFDVAYYANVLISYDGSMYWLPPAIYRSTCAIEITYFPFDYQNCTLAFRSQTYSANEV
DLILAVGDEMETIEWVDIDPQAFTENGEWAIVHRPGRKMINPRYTPDDLEYQEILFNLVI
QRKPLFYIINIILPCSLISSLVVLAYFLPAQAGGQKLTVSISVLLAQTVFLFLIAQKIPE
TSLSVPLIGKYLIFVMSVTTLIATNQIVVLNFSLRSPSTHTMSNTIKHVFLEMVPRFLGM
PPLVDDTEVTTVVNGVRERRRSSFGLLQRAEEYVLKQPRSEMMFDKQRERHGLTRSIVDD
MDVSSTANLYKSLAQAAPEIKQCVDACNFIAESTRQQNSIGSEIESWVLIGKMIDKVCFW
AAISLFIIGTVWIFLTGHFNRAPEFPFPGQSKKYVPS

>Oryzias latipes_CHRNE(ENSORLP_10079)
MPVFSSPLLQYIMIEIVHCNEESQLIGDLFRNYNKNIRPVEHPEDKVQVMIKLTLTNLIS
LNEKEETLTTNVWIEIQWTDYRLVWNTSQYYGIKTIRVPYNTVWLPDIVLENNIDGKFDV
AYYANVLISNEGWMYWLPPAIYRSTCAIEITYFPFDYQNCTLAFRSQTYSAKEVDLVLAT
GDEDVTIEWVDIDPEAFTENGEWAIVHRPARKVINTRYSPDDLEYQEIIFNLVIQRKPLF
YIINVILPCSLISSLVVLAYFLPAQAGGQKLTVSISVLLAQTVFLFLIAQKTPETSLAVP
LIGKYLIFVMCVTTLIATNQIVVLNFSLRSPSTHTMSYSIKHLCLEMVPRFLGMSPLVDD
SEVTAEVNGIRERRRSSFGLMQRAEEYVLKQPRSEMMFDKQRERHGLTRSIVDNIDVSST
ANLYKSLAQAAPEIKQCVDACNFIAESTKQQNDIGSVXESWVLIGKMIDKVCFWAAMLLF
SGTVGIFLTGHVNKAPEFPFPGEDKKYVP

>Homo sapiens_CHRNA5(ENSP_299565)
MAARGSGPRALRLLLLVQLVAGRCGLAGAAGGAQRGLSEPSSIAKHEDSLLKDLFQDYER
WVRPVEHLNDKIKIKFGLAISQLVDVDEKNQLMTTNVWLKQEWIDVKLRWNPDDYGGIKV
IRVPSDSVWTPDIVLFDNADGRFEGTSTKTVIRYNGTVTWTPPANYKSSCTIDVTFFPFD
LQNCSMKFGSWTYDGSQVDIILEDQDVDKRDFFDNGEWEIVSATGSKGNRTDSCCWYPYV
TYSFVIKRLPLFYTLFLIIPCIGLSFLTVLVFYLPSNEGEKICLCTSVLVSLTVFLLVIE
EIIPSSSKVIPLIGEYLVFTMIFVTLSIMVTVFAINIHHRSSSTHNAMAPLVRKIFLHTL
PKLLCMRSHVDRYFTQKEETESGSGPKSSRNTLEAALDSIRYITRHIMKENDVREVVEDW
KFIAQVLDRMFLWTFLFVSIVGSLGLFVPVIYKWANILIPVHIGNANK

>Xenopus tropicalis_CHRNA5(ENSXETP_51511)
MPWCAEYLLWLFLCLDILHGACGISEPSYIAKSEDRLFKYLFQQQDYQRWVRPVEHLNDT
VKVKFGLAITQLVDVDEKNQLMTTNVWLKQEWVDVKLRWDPNEFAGITSIRVPSDSIWIP
DIVLYDNADGRFEGSVTKAVVRYDGTINWTPPANYKSSCTIDVTFFPFDLQNCSMKFGSW
TYDGSQVDIILEDYHVDKRDFFDNGEWEIVNATGNKGNRTDGCCFYPYITYSFIIKRLPL
FYTLFLIIPCIGLSFLTILVFYLPSNEGEKISLCTSVLVSLTVFLLVIEEIIPSSSKVIP
LIGEYLVFTMIFVTLSIVLTVFAINIHNRSSATHNAMAPWVRKIFLHKLPKLLCMRSHVD
RYFTRKDETGKVRGPESSRNTLEAALDSIRYITRHVMKEHEVQEVVEDWKFVAQVLDRMF
LWTFLLVSVIGSLFLFIPVIHKWANIIVPVHIGNMYGDKNT

>Danio rerio_CHRNA5(ENSDARP_136216)
MVTALSLVRALLPLVCSAVLCSAIGPPMLSSYAKTEDKLFKHLFSNYQKWVRPIEDLNTT
IQVKFGLAISQLVDVDEKNQLMTTNVWMKQEWTDLKLRWDPDHYLGITSIRVPSDSIWIP
DIVLYDNADGNFEATVTKAVVRFDGTISWTPPANYKSACTIDVTFFPFDLQNCSMKFGSW
TYDGSQVDILLEDVHVDKRDYFDNGEWEIVKATGNRGLRRDGTFFYPSITYSFIIRRLPL
FYTLFLIIPCIGLSFLTVLVFYLPSNGGEKISLCTSVLVSLTVFLLVIEEIIPSSSKVIP
LIGEYLVFTMIFVTLSIVITVFAINIHHRSSSTHHSMAPWVRRIFLHHLPRLLCMRSHVD
RYATSAGTRTGGLGYGKSLSQDTNQLDAEHNLQAALESIRYITRHVVKENEVREVVQDWK
FVAQVLDRVFLWAFLLVSLLGSALLFIPVIYKWASIIVPSYAEITS

>Takifugu rubripes_CHRNA5(ENSTRUP_13990)
STEIRISMTVNLWVITVFLHLLEVPKLSSCAKAEDKLFKYLFGNYQKWVRPVDHLNQTIR

VKFGLAISQLVDVDEKNQRMTTNVWMKQEWTDMKLRWNPEEYAGITIIRVPSDRIWRPDV
VLYDKSSDGRFEGTITKAVVKYDGTITWTAPANYKSACTIDVTFFPFDLQNCSMKFGSWT
YDGSQVDIILEDFHVDKRDYFDNGEWEIVKATGSRGLRMDGSTSYPTITYFFIIRRLPLF
YTLFLIIPCIGLSFLTILVFYLPSNGGEKISLCTSVLVSLTVFLLVIEEIIPSSSKAIPL
IGEYLVFTMIFVTLSIVITVFAINVHHRSSSTHNDMAPWVRRVFLHHLPKLLCMRSHVDR
YATSGAARTRGLGMAEGSEPELTPLFYTRQNLKAALDSIRYITMHVVKENEVREYPHARG
LSQQVSKSLNSFQAQTCSQTCRFLPSIDMKKKKAKEIHFKVVQDWKFVAQVLDRMFLWAF
LLVSILGSALLFIPVIYKWASIIV

>Tetraodon nigroviridis_CHRNA5(ENSTNIP_14923)
VPKLSSYAKAEDKLLKYLFGNYQKWVRPVDHLNQTIRVKFGLAISQLVDVDEKNQRMTTN
VWMKQEWTDMKLRWSPEEYVGITIIRVPSDRIWRPDVVLYDNSDGRFEGTVTKAVVKHDG
TITWTAPANYKSACTIDVTFFPFDLQNCSMKFGSWTYDGSQVDIILEDFHVDKRDYFDNG
EWEIVKATGSRGLRTDGSASYPTITYFFIIRLPLFYTLFLIIPCIGLSFLTILVFYLPSN
GGEKISLCTSVLVSLTVFLLVIEEIIPSSSKAIPLIGEYLVFTMIFVTLSIVITVFAINV
HHRSSSTHNDMAPWVRRVFLHRLPKLLCMRHVDRYATSGASRARALGMAESTPELTPLLY
SRQSLKAALDSIRYITMHVVKENEVREVVQDWKFVAQVLDRMFLWAFLLVSILGSAILFI
PVIYKWASIIVPNHETMVFGN

>Gasterosteus aculeatus_CHRNA5(ENSGACP_21887)
MCHSLRVPKLSSFAKAEDKLFKYLFGNYQKWVRPVEYLNQTISVKFGLAISQLVDVDEKN
QQMTTNVWMKQEWIDRKLRWNPDDYLGITTIRVPSNRIWLPDVVLYDNSDGRFEATVTKA
VVKYNGTISWTPPANYKSACTIDVTFFPFDLQNCSMKFGSWTYDGSQVDITLEEFHVDKK
DYFDNGEWEIVKATGSRGLRTDSSSTYPTITYFFIIRRLPLFYTLFLIIPCIGLSFLTIL
VFYLPSNGGEKISLCTSVLVSLTVFLLVIEEIIPSSSKAIPLIGEYLVFTMIFVTLSIVI
TVFAINIHHRSSSTHHNMAPWVRRIFLHRLPKLLCMRSHADRYATAGVARTGGAEREGKM
KDSAPELTSLLYTKHNLQAALESIRYITMHVVKENEVREVVQDWKFVAQVLDRMFLWAFL
LVSILGSALLFIPVIYKWANIIVPKHFGS

>Oryzias latipes_CHRNA5(ENSORLP_24913)
VPKLSSYAKAEDTLFKHLFGTYQKWVRPIEYLNQTIPVKFGLAISQLVDVDEKKQLMTTN
VWLKQEWIDMKLRWKPEDYLGITTIRVPSDRIWLPDVVLYDNSDGRFEGTVTKAVVKYDG
TISWNPPANFKSACTIDVTFFPFDLQNCSMKFGSWTYDGSQVDIILEDFHVDKKDYFDNG
EWEIVKATGSRGLRTDGSTSYPTITYFFIIRRLPLFYTLFLIIPCIGLSFLTILVFYLPS
NGNEKISLCTSVLVSLTVFLLVIEEIIPSSSKAIPLIGEYLVFTMIFVTLSIIITVFAIN
IHYRSSSTHHDMAPWVRRIFLHRLPKLLCMRSHVDRYATAGLAGKGKVTVQEKTKDSTPD
LTHILHTRHNLQTALESIRYITMHVVKENQVREVVQDWKYVAQVLDRMFLWAFFLVSVLG
SALLFVPVIYKWASIIVPDYAGS

>Ciona intestinalis_(ENSCINP_4364)
MGSRCWLIAVCLCYVIRECLSAHAEEKLLRHLLDPVRYNKLIRPSESITSKVDVHFDLII
SQLIGINEREQYMITKVWLPQVWVDKRLSWQPMRYENITKVQLPSNMIWLPDVVLYNNAD
GMYEVSLQSNAIILNNGTVTWMPPAIYKSACKIKVRHFPFDEQNCTMMFRSWTYNRLDIE
LKLKNPNATLDDFTPSGEWDILQTPGSVNVNPNDETYQDLLYSLIIRRKPLFYTINLIIP
CILITSLAILVFYLPSDCGEKMTLCISVLLALTVFLLLISKIVPPTSLDVPLIGKYLMFT
MMLVTLSIVASVCVLNIHHRSPSTHTMPPWIRTIFLNHLPPLLGMERPGTDDRCRCKVND
ATYGVDVTELSGSDVIRNRKRGKSEVKLPRDVSSALEGVKFIADHLRAEDENKNVHEDWK
YVAMVIDRMFLFVFVGVCAIGTIGIFIQPFV

>Ciona intestinalis_(ENSCINP_17902)
MINCLLIFLLLPITKTSAAKFESTEGRLANYLLNGYNPQFRPYNKKYENKTFVDVMMTLS
QIISVKEKSQEFTTSVYMQYDWLDDRFGWVPDDYDGITTVRIPCASIWVPRIVLQNSIDG
NFDVSLRTNALIQHDGKVSWLPPALYKSTCTIEVALFPFDWQNCTMVFRSATYDRTEMEF
GIPIDTVYIDLSAFQSNGEWDILQRPVKINSNENDKTYQDITFYFIIRRKPQFYIINLII
PCILMSSLSCFVFYLPSISCEKMTLSISILLGETVFLFLIAQRMPETSLAVPLIAGYLLF
VMILVIISVICSVVVCNVHYRSNTTHSMPDWIVWIFMEKLAPLLKVERPSEEESGACSAR
PRRCSSVSTRKAMKQRSEWKPPSEILFDHQASRLGFRARHYLNEYGMTGINVAAPENGSF
VPPSFKKLPKGFQPAADSIDYIANQKKEENEMGQSQDDWGYVALILDRLLLWVYLATFTL
GTLFFFLQTKFAYYPEKLN

>Ciona savignyi_(ENSCSAVP_750)

ECTSAHAEEELLRHLLDSGRYNKLIRPSQSNFDLIISQLIGINEREQFMITKVWLPQVWV
DERLSWQPTEYENITKVQLPSDMIWLPDIVLYNNADGTYEVSLQSNAIILNNGTVTWMPP
AIYKSACKIKVRHFPFDEQNCTMMFRSWTYDRDDIELKLMNPNATLDDFTPSGEWDILAT
PGMMNLNADDPSYQDLLYSLVIRRKPLFYTVNLIIPCILITSLAILVFYLPSDCGEKMTL
CISVLLALTVFLLLISKIVPPTSLDVPLIGKYLMFTMMLVTFSIIASVCVLNIHHRSPST
HEMSPWIRRTFLQVIPPLIGMQRPGTDQCSYNKYKVDVSDLSGSDVIRKRKQPEVQLSRD
ISSALEGVKLISDHLKAEDRNKNVSFIGYLYILEYLIFRLTRMLIRGYISFIVLLDSPTP
AVHVQFRSQGWIRQHLFNLSSCELVIHEDWKYVAMVIDRMFLFVFVVVCAVGTVSIFLQP
FV

>Ciona savignyi_(ENSCSAVP_1475)
LHTGFILLVLAASAASVLSEFQSSESRLMKRLLNGYNNQFRPYNVEYENKTLVSMGMTLS
QIISVKEKSQELTTSVYMTFDWVDNRFSWDPEEYEGITIIRIPPYAIWSPRIVLQNSIDG
NFDVSVPVMALIKYTGEVQWIPPGLYKSTCTIVVALFPFDWQNCTMVFRSATYDRSEMEF
GLSYGDSIYVDPEAFKENGEWKIVQRPVRINSDSSDPTYQDVTFYLIIRRKPQFYVINLI
IPCILMSSLSCFVFYLPSVSCEKMTLSISVLLGETVFLFLIAQRVPETSLAVPLIAGYLL
FVMILVIISVISSVVVCNVHYRSNTTHSMPDWVVWFFMEKLAPILRVERPSEEESGACSA
RPRRCSSVSARKAMRQRKEWKSPSEIMFDHQASRLGFRRRHVVNHYGMTEVNVASPENDF
EPAAESINYIANQLKEENDSAQSKDDWGYVALILDRILLWIYFAAFSLGTLFFFLQSTFA
YYPEQ

>Ciona savignyi_(ENSCSAVP_3483)
TEEKLFKRLFDDYNIWIRPVANSNQSVLIAFELFVSQLVKVDEINQIMMTNLWLKQEWND
NKLRWRPEDYGGVKYLRVPSEFIWKPDIVLYNTAVGKFQVDQNTKALLRYNGDVSWMPPA
IFKSSCSIDVTYFPFDTQNCTMKFGSWTYDKARVNLVIKGNEANRKEYWESGEWELLDVL
GYRHERKYNCCPEIYPDVTYSFHIRRLPLFYMINLIIPCLLISFLTVLVFYLPSDCGEKI
TLCISVLLSLTVFLLVITEIIPSTSLVIPLIGQYLLFTMIFVTLSIVITVFVLNVHFRSE
NTHTMPPWVRRVFLECLPKASCFSHLFHSSISSHNHNNVNKSLAHSATSLTHHSTSPTHN
TTTAICHMNRVSNAEKTEKTPDSALPKEVQRAIESVMYIANHLKVEDESTEIENDWKYVG
MVIDRIFLWIFIVVCVLGTTGIFLRPLLDDTHVG

>Branchiostoma floridae_(XP_002594600)
MLVTKVNVSFCRSHTETLAAEAEERLVQRLFNESRYNPLIRPARYNGEKVLVHFRLSISQLINVDEKNQK
MKTNVWLHQEWFDYRLSWDPREYDNIKVIRVPSEIVWRPEIVLFNNANGQYDVQLMPKCLIYSGGNITWI
PPAIYESACQIDVRFFPFDQQNCSMKFASWTYDGTELDLELKDTNAVMDDFKKNGEWDILRLPGRKSSHA
GDPLPYVYFDFILMRRPLFYTINLIIPCILITSLSVLVFYLPSDCGEKITLSISVLLALTVFLLLIADII
PPTSLDVPLIGKYIMFTMIFVTFTIVTTVYILNVHHRHPSTHTMPPWVRTVFLDKLPRLLLMKRPNQCRK
YALRVSESSARNPHEDFPEDHTDSPYYSPRTRPAHTHYKQIDLPPEVPSDLPPELKKAVKNVRYIADYFR
SHDQDLSISEDWKYVAMVIDRIFLWTFVSVCFIGTLGLFLQPLL

>Branchiostoma floridae_(XP_002603628)
MFLPGGVATQSEERLFRDLFRDYNKWIRPVANISNIVVIEFGLAIAQLLDVDEKNQIMTTNVWLKQKWKD
SKLCWDPDEYHGVSVIRVPSDMIWLPDIVLYNNADGNYAVKHNTKAQISSDGMVTWEPPAVYKSSCEIEV
TYFPFDQQNCSMKFGSWTYDGFQIDLMMMDDHVDQGGFSESGEWVIIDAPGQKNIIKYECCEEVYPDITF
FFVIRRLPLFYTINLIVPCALISFLTVLVFYLPSDCGEKITLCISVLLSLTVFLLLITEIIPATSIVIPV
IGEYLLFTMIFVTLSIVITVFVLNIHYRSPSTHRMPVWVRKIFIDILPRLLSDVYDYESKTLPRMTTPKM
RSRHGAKVSVTNVSGNPGVGGAEKYNTCDSRPSQSQARAQFQSYQYSPDILRAAQGVNYIAETLRAKDEA
DSVNEDWKYVAMVIDRIFLWIFVSMCILGTIGLFLQPLPGFIS

>Drosophila melanogaster_(FBpp0306936)
MGSVLFAAVFIALHFATGGLANPDAKRLYDDLLSNYNRLIRPVGNNSDRLTVKMGLRLSQ
LIDVNLKNQIMTTNVWVEQEWNDYKLKWNPDDYGGVDTLHVPSEHIWLPDIVLYNNADGN
YEVTIMTKAILHHTGKVVWKPPAIYKSFCEIDVEYFPFDEQTCFMKFGSWTYDGYMVDLR
HLKQTADSDNIEVGIDLQDYYISVEWDIMRVPAVRNEKFYSCCEEPYLDIVFNLTLRRKT
LFYTVNLIIPCVGISFLSVLVFYLPSDSGEKISLCISILLSLTVFFLLLAEIIPPTSLTV
PLLGKYLLFTMMLVTLSVVVTIAVLNVNFRSPVTHRMAPWVQRLFIQILPKLLCIERPKK
EEPEEDQPPEVLTDVYHLPPDVDKFVNYDSKRFSGDYGIPALPASHRFDLAAAGGISAHC
FAEPPLPSSLPLPGADDDLFSPSGLNGDISPGCCPAAAAAAAADLSPTFEKPYAREMEKT
IEGSRFIAQHVKNKDKFESVEEDWKYVAMVLDRMFLWIFAIACVVGTALIILQAPSLYDQ
SQPIDILYSKIAKKKFELLKMGSENTL

>Apis mellifera_(NP_001011575)
MFKMQILTLGVLFNTLHIIYSVAGLKIFEANPDTKRLYDDLLSNYNRLIRPVMNNTETLTVQLGLKLSQL
IEMNLKNQVMTTNVWVEQRWNDYKLKWNPEEYGGVEMLYVPSENIWLPDIVLYNNADGNYEVTLMTKATL
KYTGDVSWKPPAIYKSSCEINVEYFPFDEQSCIMKFGSWTYNGAQVDLKHMKQEAGSNLVAKGIDLSDFY
LSVEWDILEVPASRNEEYYPCCTEPYSDITFNITMRRKTLFYTVNLIIPCVGITFLTVLVFYLPSDSGEK
VSLCSSILLSLTVFFLLLAEIIPPTSLAIPLLGKYLLFTMILVTLSIWITVCVLNVHFRSPSTHNMSPWV
RQVFLNWMPRLLMMRRTPYSTPEYDDTYMDSGYTNEIDFSFPDSVSDYPLELKGSPDGFESVTSQYKNIR
EDDARHIPHASVTDSENTVPRYLSPDVISALKGVRFIAQHIKNADKDNEVIEDWKFVAMVLDRLFLWVFT
LACTAGTLGIIFQAPSLYDTREPVDQQLSGISPRNYMYPNIDISPEG

>Anopheles gambiae_(XP_311918)
MGSVLLTAVFIALQFATGLANPDSKRLYDDLLSNYNRLIRPVGNNSDRLTVKMGLRLSQLIDVNLKNQIM
TTNVWVEQEWNDYKLKWNPDDYGGVDTLHVPSEHIWLPDIVLYNNADGNYEVTIMTKAILHHTGKVVWKP
PAIYKSFCEIDVEYFPFDEQTCFMKFGSWTYDGYMVDLRHLQQTPDSDNIDIGIDLQDYYISVEWDIMKV
PAVRNEKFYSCCEEPYPDIIFNITLRRKTLFYTVNLIIPCVGISFLSVLVFYLPSDSGEKISLCISILLS
LTVFFLLLAEIIPPTSLTVPLLGKYLLFTMMLVTLSVVVTIAVLNVNFRSPVTHRMAPWVHRVFIELLPK
VLCIERPKKEDEPSDNDDQTPTDVLTDVFQVPPDVEKYVGFCGKEYGTDFDIPALPPSRFDVAASGGVGP
CFGEPPLPALPLPGGDDDLFSPTGNGDMSPTCCGDLSPTFEKPLLREMEKTIEASRFVAQHVRNKDKFES
VKEDWKYVALVLDRLFLWIFTIACVLGTCLIILQAPSLYDNTQPIDAMYSKIAKKKMELLKMGSENV

>Caenorhabditis elegans_(T08G11)
MRTNRLSWILVLSVVIFLVIINTINASDDEERLMVDVFRGYNSLIQPVRNSSELPLIVKM
ALQLVLLINVDEKDQVMHTNVWLTLQWHDFQMKWNPVNYGEIKQIRVSPDKVWLPDIVLF
NNADGNYEVSFMCNVVINHKGDMLWVPPAIYKSSCIIDVEFFPFDEQVCTLVFGSWTYNE
NEIKLEFVQAELVDVSEYSASSIWDVIDVPASLVNKRSRIEFQVRIRRKTLFYTVVLIIP
TVLMAFLSMAVFFLPTDSGEKITLTISVLLSIVVFLLLVSKILPPTSSTIPLMAKYLLLT
FVLNVITILVTVIIINVYFRGPRTHRMPQWVRVVFLQFLPKLVCMKRPKSASERSAVRSG
MAQLPGVGQFTLSPSAHHPLCPSADDRTTTIRNTASNETSAYYPLSTDALRAIDAIEYIT
EHLKRDEQHKSFRDDWKYVAMIIDRLLLYVFFGITVGGTCGILFSAPHVFQRIDQQEMLD
RLKEKYDTASNIP

>Trichoplax adhaerens_(XP_002111826)
MVATAQKNCYIYAILFASILSHSICNAQKTLFSVLEQQQTQNAVMISTNGDAIIAGLLPAHYNYEDGKHK
CSELDTYGIHLMMAMIFAINDINEQSNLLPDVKLGYDIRDTCHNSLRVSKEIMALLMQYLPFKEGKYDGF
FCEKIKYDEANEETVRTYGPIIGVIGTTMSDSTIATANTIGNFGVIEITPSALAVTQSDNQPPNLYSTQP
SFYDHLVVISKIIIALDMTRICLITIEPPETSYRMDLLRTILQDAKICIAQEFSITGDRMSLAMARVIQN
LKANNKLQVVVLLGPYEKGVRFMKEAERQNLTGRTWIGAGEWVKSPAIYSIRKEVMGGLLAVSTQTFRSV
LITKHLENYVTSDTRNVHLDQLWKKLYNCTPNEENCEVRLGSYEIIDEKKSSCEYYQSELNEDIIYVIDA
VYAIAHALHQTLNCTIEFCNSVETFNHTIFNYHLQRVEFSNHANQPVEFQQNKGVRHILIQNLHHDDNCS
HFVEVARWNPFESEMIMDTNKIRWRNGESTTIPTEVYETACTPGDRKHSFDQESCYYVCLKCGPSLVSNG
TTSQCFKCSPGFKPTNNRSACQEIPPTKLDWRNPVNLTILIFTLLSMLTLLFIVIIFFKYSNTPIVKASN
KIMSHIQLLSVALLFILGGSYQFAVTTSLCWFQFAICSVSFPLCEACLLAKTKHVANVFTLSSRIQKVDK
SSRLQSRWCKSLIPILFVLSITAIPIIIWLIFTIIDPPNAIPDYRYENEVHILCSSKLHLGLGLVVGFTL
VLAIICTILAWKTRKLPDNFSEARYIFICSFSTLVIVALSVPGYYSTVGLLQATFASIAVITFGLSILLL
LFIPKIYTILFRPDLNTKEAMLRSIQEFTFGADTRIHTKPK

>Amphimedon queenslandica_(XP_011410551)
MSSSRSLSSSRSKSHLSKSSKFESTAELPSEVMVNTPEQLSWQVLSQTQVARFKALSPTELTKELSTVLS
LSKHEEDLQQAAILDYYVSSFVFAQEKDFTDEQLSSFFTIIHKLLANIREKRTTLQDNLNELKSYFAGVG
VSDQTCLKCFTPKVAMATISYIHTSLVQHYTLFEYILQEKQEDEFIQTQLLVSLPPDSSTLSPPPLEESV
TEDFYNRLVSTAPAGSPSQTQLGSPTTHSPPHSIQGTDMSSPTGAIPPTLLLNLRPEDVKMMTEKLLEET
VTPYQDEMLRKLVDRETSYKSKLDKLEKSLT

Ciliary Rootlet Coiled-Coil Protein-CRO

>Homo sapiens_CROCC(ENSP_364691)
MSLGLAGAQEVELTLETVIQTLESSVLCQEKGLGARDLAQDAQITSLPALIREIVTRNLS
QPESPVLLPATEMASLLSLQEENQLLQQELSRVEDLLAQSRAERDELAIKYNAVSERLEQ
ALRLEPGELETQEPRGLVRQSVELRRQLQEEQASYRRKLQAYQEGQQRQAQLVQRLQGKI
LQYKKRCSELEQQLLERSGELEQQRLRDTEHSQDLESALIRLEEEQQRSASLAQVNAMLR
EQLDQAGSANQALSEDIRKVTNDWTRCRKELEHREAAWRREEESFNAYFSNEHSRLLLLW

RQVVGFRRLVSEVKMFTERDLLQLGGELARTSRAVQEAGLGLSTGLRLAESRAEAALEKQ
ALLQAQLEEQLRDKVLREKDLAQQQMQSDLDKADLSARVTELGLAVKRLEKQNLEKDQVN
KDLTEKLEALESLRLQEQAALETEDGEGLQQTLRDLAQAVLSDSESGVQLSGSERTADAS
NGSLRGLSGQRTPSPPRRSSPGRGRSPRRGPSPACSDSSTLALIHSALHKRQLQVQDMRG
RYEASQDLLGTLRKQLSDSESERRALEEQLQRLRDKTDGAMQAHEDAQREVQRLRSANEL
LSREKSNLAHSLQVAQQQAEELRQEREKLQAAQEELRRQRDRLEEEQEDAVQDGARVRRE
LERSHRQLEQLEGKRSVLAKELVEVREALSRATLQRDMLQAEKAEVAEALTKAEAGRVEL
ELSMTKLRAEEASLQDSLSKLSALNESLAQDKLDLNRLVAQLEEEKSALQGRQRQAEQEA
TVAREEQERLEELRLEQEVARQGLEGSLRVAEQAQEALEQQLPTLRHERSQLQEQLAQLS
RQLSGREQELEQARREAQRQVEALERAAREKEALAKEHAGLAVQLVAAEREGRTLSEEAT
RLRLEKEALEGSLFEVQRQLAQLEARREQLEAEGQALLLAKETLTGELAGLRQQIIATQE
KASLDKELMAQKLVQAEREAQASLREQRAAHEEDLQRLQREKEAAWRELEAERAQLQSQL
QREQEELLARLEAEKEELSEEIAALQQERDEGLLLAESEKQQALSLKESEKTALSEKLMG
TRHSLATISLEMERQKRDAQSRQEQDRSTVNALTSELRDLRAQREEAAAAHAQEVRRLQE
QARDLGKQRDSCLREAEELRTQLRLLEDARDGLRRELLEAQRKLRESQEGREVQRQEAGE
LRRSLGEGAKEREALRRSNEELRSAVKKAESERISLKLANEDKEQKLALLEEARTAVGKE
AGELRTGLQEVERSRLEARRELQELRRQMKMLDSENTRLGRELAELQGRLALGERAEKES
RRETLGLRQRLLKGEASLEVMRQELQVAQRKLQEQEGEFRTRERRLLGSLEEARGTEKQQ
LDHARGLELKLEAARAEAAELGLRLSAAEGRAQGLEAELARVEVQRRAAEAQLGGLRSAL
RRGLGLGRAPSPAPRPVPGSPARDAPAEGSGEGLNSPSTLECSPGSQPPSPGPATSPASP
DLDPEAVRGALREFLQELRSAQRERDELRTQTSALNRQLAEMEAERDSATSRARQLQKAV
AESEEARRSVDGRLSGVQAELALQEESVRRSERERRATLDQVATLERSLQATESELRASQ
EKISKMKANETKLEGDKRRLKEVLDASESRTVKLELQRRSLEGELQRSRLGLSDREAQAQ
ALQDRVDSLQRQVADSEVKAGTLQLTVERLNGALAKVEESEGALRDKVRGLTEALAQSSA
SLNSTRDKNLHLQKALTACEHDRQVLQERLDAARQALSEARKQSSSLGEQVQTLRGEVAD
LELQRVEAEGQLQQLREVLRQRQEGEAAALNTVQKLQDERRLLQERLGSLQRALAQLEAE
KREVERSALRLEKDRVALRRTLDKVEREKLRSHEDTVRLSAEKGRLDRTLTGAELELAEA
QRQIQQLEAQVVVLEQSHSPAQLEVDAQQQQLELQQEVERLRSAQAQTERTLEARERAHR
QRVRGLEEQVSTLKGQLQQELRRSSAPFSPPSGPPEK

>Pan troglodytes_CROCC(ENSPTRP_423)
MSLGLAGAQEVELTLETVIQTLESSVLCQEKGLGARDLAQDAQITSLPARIREIVTRNLS
RPESPVLLPATEMASLLSLQEENQLLQQELSRVEDLLAQSRAERDELAIKYNAVSERLEQ
ALQLESGELETQEPRGLVRQSVELRRQLQEEQASYRRKLQAYQEGQQRQAQLVQRLQGKI
LQYKKRCSELEQQLLERSGELEQQRLRDTEHSQDLESALIRLEEEQQRSASLAQVNAMLR
EQLDQAGSANQALSEDIRKVTNDWTRCRKELEHREAAWRREEESFNAYFSNEHSRLLLLW
RQVVGFRQLVSEVKMFTARDLLQLGGELARTSRAVQEAGLGLSTGLRLAESRAEAALEKQ
ALLQAQLEEQLRNKVLREKDLVQQQMQSDLDKADLSARVTELALAVERLQKQNLEKDQVN
KDLTEKLEALESLRLQEQAALETEDGEGLQQTLRDLAQAVLSDSESGVQLSGSERTADAS
NGSLRGLSGQRTPSPPRRSSPGRGRSPRRGPSPACSDSSTLALIHSALHKRQLQVQDMRG
RYEASQDLLGTLRKQLSDSESERRALEEQLQRLRDKTDGAMQAHEDAQREVQRLRSANEL
LSREKSNLAHSLQVAQQQAEELRQEREKLQAAQEELRRQRDRLEEEQEDAVQDGVRVRRE
LERSHRQLEQLEGKRSVLAKELVEVREALSRATLQRDMLQAEKTEVAEALTKAEAGRVEL
ELSMTRLRAEEASLQDSLSKLSALNESLAQDKLDLNRLVAQLEEEKSALQGRQRQAEQEA
TVAREEQERLEELRLEQEVARQGLEGSLRVAEQAQEALEQQLPTLRHERSRLQEQLAQLS
RQLSGREQELEQARREAQRQVEALERAAREKEALAKERAGLAVQLVAAEREGRTLSEEAT
RLRLEKEALEGSLFEVQRQLAQLEARREQLEAEGQALLLAKETLTGELAGLRQQIIATQE
KASLDKELMAQKLVQAEREAQASLREQRAAHEEDLQRLQREKEAAWRELEAERAQLQSQL
QREQEELLARLEAEKEELSEEIAALQQERDEGLLLAESEKQQALSLKESEKTALSEKLMG
TRHSLATISLEMERQKRDAQSRQEQDRSTVNALTSELRDLRAQREEAAAAHAQEVRRLQE
QARDLGKQRDSCLLEAEELRTQLRLLEDARDGLRRELLEAQRKLRESQEGREVQRQEAGE
LRRSLGEGAKEREALRRSNEELRSAVKKAESERISLKLANEDKEQKLALLEEARTAVGKE
AGELRTGLQEVERSRLEARRELQELRRQMKMLDSENTRLGRELAELQGRLALGERAEKES
RRETLGLRQRLLKGEASLEVMRQELQVAQRKLQEQEGEFRTRERRLLGSLEEARGTEKQQ
LDHARGLELKLEAARAEAAELGLRLSAAEGRAQGLEAELARVEVQRRAAEAQLGGLRSAL
RRGLGLGRAPSPAPRPVPGSPARDAPAGGSGEGLSSPSTLECSPGSQPPSPGPATSPASP
DLDPEAVRGALREFLQELRSAQRERDELRTQTSALNRQLAEMEAERDSATSRARQLQKAV
AESEEARRSVDGRLSGVQAELALQEESVRRSERERRATLDQVATLERSLQATESELRASQ
EKISKMKANETKLEGDKRRLKEVLDASESRTVKLELQRRSLEGELQRSRLGLSDREAQAQ
ALQDRVDSLQRQVADSEVKAGTLQLTVERLNGALAKVEESEGALRDKVRGLTEALAQSSA
SLNSTRDKNLHLQKALTACEHDRQVLQERLDAARQALSEARKQSSSLGEQVQTLRGEVAD

LELQRVEAEGQLQQLREVLRQRQEGEAAALNTVQKLQDEQRLLQERLGSLQRALAQLEAE
KREVERSALRLEKDRVALRRTLDKVEREKLRSHEDTVRLSAEKGRLDRTLTGAELELAEA
QRQIQQLEAQVVVLEQSHSPAQLEVDAQQQQLELQQEVERLRSAQAQTERTLEARERAHR
QRVRGLEEQVSTLKGQLQQELRRSSAPFSPPSGPPEK

>Gorilla gorilla_CROCC(ENSGGOP_15837)
MSLGLAGAQEVELTLETVIQTLESSVLCQEKGLGARDLAQDAQITSLPARIREIVTRNLS
RPESPVLLPATEMASLLSLQEENQLLQQELSRVEDLLAQSRAERDELAIKYNAVSERLEQ
ALRLESGELETQEPRGLVRQSVELRRQLQEEQASYRRKLQAYQEGQQRQAQLVQRLQGKI
LQYKKRCSELEQQLLERSGELEQQRLRDTEHSQDLEGALIRLEEEQQRSASLAQVNAMLR
EQLDQAGSANQALSEDIRKVTSDWTRSRKELEHREAAWRREEESFNAYFSNEHSRLLLLW
RQVVGFRRLVSEVKMFTERDLLQLGGELARTSRAVQEAGLGLSTDLRLAESRAEAALEKQ
ALLQAQLEEQLRDKVLREKDLAQQQMQSDLDKADLSARVTELSLAVKRLQKQNLEKDQVN
KDLTEKLEALESLRLQEQAALETEDGEGLQQTLRDLAQAVLSDSESGVQLSGSERTTDAS
NGSLRGLSGQRTPSPPRRSSPGRGRSPRRGPSPACSDSSTLALIHSALHKRQLQVQDMRG
RYEASQDLLGTLRKQLSDSESERRALEEQLQRLRDKTDGAMQAHEDAQREVQRLRSANEL
LSREKSNLAHSLQVAQQQAEELRQEREKLQAAQEELWCQRDRLEEEQDDAVQDGARVRRE
LERSHRQLEQLEGKRSVLAKELVEVREALSRATLQRDMLQAEKAEVAEALTKAEAGRVEL
ELSMTKLRAEEASLQDSLSKLSALNESLAQDKLDLNRLVAQLEEEKSALQGRQRQAEQEA
TVAREEQERLEELRLEQEVARQGLEGSLRVAEQAQEALEQQLPTLRHERSRLQEQLAQLS
RQLSGREQELEQARREAQRQVEALERAAREKEALAKERAGLAVQLVAAEREGRTLSEEAT
RLRLEKEALEGSLFEVQRQLAQLEARREQLEAEGQALLLAKETLTGELAGLRQQIIATQE
KASLDKELMAQKLVQAEWEAQASLWEQQAAHEEDFQRLQHEKEAAWRELEAERAQLQSQL
QREQEELLARLEAEKEELSEEIAALQQERDEGLLLAESEKQQALSLKESEKTALSEKLMG
TRHSLATISLEMERQKRDAQSRQEQDRSTVNALTSELRDLRAQREEAAAAHTQEVRRLQE
QARDLGKQRDSCLREAEELRTQLRLLEDARDGLRRELLEAQRKLRESQEGREVQRQEAGE
LRRSLGEGAKEREALRRSNEELRSAVKKAESERISLKLANEDKEQKLALLEEARTAVGKE
AGELRTGLQEVERSRLEARRELQELRRQMKMLDSENTRLGRELAELQGRLALGERAEKES
RRETLGLRQRLLKGEASLEVMRQELQVAQRKLQEQEGEFRTRERRLLGSLEEARGTEKQQ
LDHARGLELKLEAARAEAAELGLRLSAAEGRAQGLEAELARVEVQRRAAEAQLGGLRSAL
RRGLGLGRAPSPAPRPVPGSPARDAPAGGSGEGLSSPSTLECSPGSQPPSPGPATSPASP
DLDPEAVRGALREFLQELRSAQRERDELRTQTSALNRQLAEMEAERDSATSRARQLQKAV
AESEEARRSVDGRLSGVQAELALQEESVRRSERERRATLDQVATLERSLQATESELRASQ
EKISKMKANETKLEGDKRRLKEVLDASESRTVKLELQRRSLEGELQRSRLGLSDREAQAQ
ALQDRVDSLQRQVADSEVKAGTLQLTVERLNGALAKVEESEGALRDKVRGLTEALAQSSA
SLNSTRDKNLHLQKALTACEHDRQVLQERLDAARQALSEARKQSSSLGEQVQTLRGEVAD
LELQRVEAEGQLQQLREVLRQRQEGEAAALNTVQKLQDERRLLQERLGSLQRALAQLEAE
KREVERSALRLEKDRVALRRTLDKVEREKLRSHEDTVRLSAEKGRLDRTLTGAELELAEA
QRQIQQLEAQVVVLEQSHSPAQLEVDAQQQQLELQQEVERLRSAQAQTERTLEARERAHR
QRVRGLEEQVSTLKGQLQQELRRSSAPFSPPSGPPEK

>Mus musculus_CROCC(ENSMUSP_99549)
MSLGLAGSLQAQLALEIVIQSLENCVLGPNQEKSLSVQNRVQDFQGASLLVCAREVIASN
LSRPETPAPLQVPEMASLLSLQEENQLLQQELSRVEDLLAQSRAERDELAIKYNAVNERL
EQAVRLETGELEAQEPRGLVRQSVELRRQLQEEQSSYRRKLQAYQEGQQRQAQLVQRLQA
KILQYKKQCSELEKQLMDRSTELEQQRLRDTEHSQDLDSALLRLEEEQQRSASLAQVNAM
LREQLDQANLANQALSEDIRKVTSDWTRSCKELEQREAVWRREEESFNTYFSSEHSRLLR
LWRQVMGLRRQASEVKMGTERDLLQLGGELVRTSRAVQELGLGLSASLHRAESKAEAALE
KQKLLQAQLEEQLQAKLLREKDLAQLQVQSDLDKADLSARVTELALSVEHLQNQNSEKDQ
VNRTLSDKLEALESLRLQEQTTLDTEDGEGLQQTLRDLAQAALSDTESGVQLSSSSERTAD
TSDGSLRGFSGQRTPTPPRHSPGRGRSPRRGLSPACSDSSTLTLIHSALHKRQLQVQDMR
GRYEASQELLGSVRKQLSDSEGERRGLEEQLQRLRDQTAASAQAQEDAQREAQRLRSANE
LLSREKGNLTHSLQVTQQQAKELRQELEKLQAAQEELKRQHNQLEDAQEDSVQEGARARR
ELERSHRQLEQLEVKRSGLTKELVEVREALSCAILQRDVLQTEKAEVAEALTKAEAGRAQ
LELSLTKLRAEEASLRDSLSKMSALNESLAQDKLELNRLIAQLEEEKVALLGRQQQAEHA
TTMAVEKQELLEQLRLEQEVERQGLQGSLCVAEQAREALEQQILVLRSERSHLQEQLAQL
SRQLSGRDQELEQALRESQRQVEALERAAREKEAMAKERAGLAVKLAAAEREGRTLSEEA
IRLRLEKEALESSLFDVQRQLAQLEARREQLEADSQALLLAKETLTGELAGLRQQVTSTE
EKAALDKELMTQKLVQAEREAQASLREQRAAHEEDLQRLQHEKEAAWRELQAERAQLQGQ
LQQEREELLARMEAEKEELSKEIAALQQERDEGLLLAESEKQQALSLKESEKTALSEKLM
GTRHSLAAISLEMERQKRDAQSRQEQDRNTLNALTSELRDLRAQLEEATAAHAQTVKELE

ERTGNLGRQREACMREAEELRTQLRVLEDTRDGLRRELLEAQRKGRDSQDSSEAHRQEAS
ELRRSLSEGAKEREALRRSNEELRSAVKKAESERISLKLANEDKEQKLALLEEARVSVAK
EAGELRASLQEVERSRLEARRELQELRRQMKTLDSDNGRLGRELADLQGRLALGERTEKE
SRREALGLRQRLLKGESSLEALKQELQGSQRKLQEQEAEFRARERGLLGSLEEARGAEKR
LLDSARSLELRLEAVRAETSELGLRLSAAEGRAQGLEVELARVEAQRRVAEAQLGGLRSA
LRRGLGLGRVSSSPAREAPAGGSGDGLSSPSPLEYSPRSQPPSPGLIASPAPPDLDPEAV
RDALRDFLQELRSAQRERDELKVQTSTLSQQLVEMEAERDHAASRAKQLQKAVAESEEAW
RSADRRLSGAQAELALQEESVRRSKRECRATLDQMAVLERSLQATESELRASQEKVSKMK
ATEAKLESDKRRLKEVLDASESRSIKLELQRRALEGELQRSRLGLGDREAHAQALQDRVD
SLQRQVADSEVKAGTLQLTVERLSGALAKVEESEGNLRSKVQSLTDALTQSSASLSSTQD
KNLHLQKALSTCEHDRQVLQERLDAARQALSEARRQSSSLGEQVQTLRGELASLELQRGD
AEGQLQQLQQALRQRQEGEAMALRSVQKLQEERRLLQERLGSLQRALAQLEAEKRDLERS
ALQFDKDRVALRKTLDKVEREKLRSHEDTLRLNAERGRLDRTLTGAELDLAEAQQQIQHL
EAQVDVALEGNHNPVQPEAGEQQLELQQEVERLRSAQVQTERTLEARERAHRQRVSGLEE
QVSTLKAQLHQELRRSSASVSLPPGTPEK

>Rattus norvegicus_CROCC(ENSRNOP_52694)
MASLLSLQEENQLLQQELSRVEDLLAQSRAERDELAIKYNAINERDTEHSQDLDRALLRL
EEEQQRSASLAQVNDMLREQLDQANLANQTLSEDICKVTSDWTRSCKELEQREATWRREE
ESFNAYFSSEHSRLLLLWRQVMGLRRMASEVKMGTERDLLQLGGELVRTSRAVQEVGLGL
SASLQRAESRAEAALEKQKLLQAQLEEQLRAKLLREKDLAQLQVQSDLDKADLSARVTEL
ALSVEHLQNQNTEKDQVNRTLSDKLEALESLRLQEQTTLDTEDGEGLQQTLRDLAQAALS
DTESGVQLSNSERTADTSDGSFRGLFGQRTPTPPRHSSPGRGRSPRRGLSPACSDSSTLT
LIHSALHKRQLQVQDMRGRYEASQDLLGSVRKQLSDSEGERRGLEEQLQRLRDQTATSVQ
AQEDAQREAQRLRSANEILSREKGNLSHSLQVAQQQAEDLRQELEKLQAAQEELRRQHTQ
LEDQQEDTVQEGARARRELERSHRQLEQLEVKRSGLTKELVEVREALSCAVLQRDVLQTE
KAEVAEALTKAEAGRAQLELSVTKLRAEEASLRDSLSKMSALNESLAQDKLELNRLIAQL
EEEKAALLGRQQQAEHATSLAVEKQERLEQLRLEQEVERQGLEGSLCVAEQAREALGQQI
LVLRSERSHLQEQVAQLSRQLNGRDQELDQALRESQRQVEALERAAREKEAMAKERAGLA
VQLAAAEREGRTLSEETIRLRLEKEALESSLFDVQRQLAQLEARREQLEADSQALLLAKE
TLTGELAGLRQQVTATEEKAALDKELMTQKLVQAERETQASLREQRAAHEEDLQRLQREK
EAAWRELQAERAQLQGQLQQEREELLARMEAEKEELSEEIAALQQERDEGLLLAESEKQQ
ALSLKESEKTALSEKLMGTRHSLAAISLEMERQKRDAQSRQEQDRNTVNALTSELRDLRA
QLEEATAAHAQQVKELQEQTGNLGRQREACMREAEELRTQLRLLEDTRDGLRRELLEAQR
KVRDSQDSSEAHRQEASELRRSLSEGTKEREALRRSNEELRTAVKKAESERISLKLANED
KEQKLALLEEARMSVAKEAGELRASLQEVERSRLEARRELQELRRQMKTLDSDNGRLGRE
LADLQSRLALGERTEKESRREVLGLRQKVLKGESSLEALKQELQGSQRKLQEQEAEFRAR
ERGLLGSLEEARGAEKKLLDSARSLELRLEGVRAETSELGLRLSAAEGRAQGLEVELARV
EAQRRVAEAQLGGLRSALRRGLGLGRVSSSPAREAPAGGSGDGLSSPSPLEYSPRSQPPS
PGPVASPAPPDLDPEAVRDALRDFLQELRSAQRERDELRVQTSTLSQQLAEMEAERDHAA
SRAKQLQKAVAESEEAWRSADRRLSGAQAELALQEESVRRSRRECRATLDQMAVLERSLQ
ATESELRASQEKVNKMKATEVKLESDKRRLKEVLDASESRSIKLELQRRALEGELQRSRL
GLGDREAHAQALQDRVDSLQRQVADSEVKAGTLQLTVERLSGALAKVEESEGTLRSKVQS
LTDALAQSSASLTSSQDKNLYLQKALSTCEHDRQVLQERLDAARQALSEARRQSSSLGEQ
VQTLRGELANLELQRGDAEGQLQQLQQVLRQRQEGEAVALRSVQKLQEERRLLQERLGSL
QRALAQLEAEKRELERSALQLDKDRVALRKTLDKVEREKLRSHEDTLRLNAERGRLDRTL
TGAELDLAEAQQQIQHLEAQVVEVLERNHSPVQVEADEQHLELQQEVERLRSAQVRTERT
LEARERAHRQRVSGLEEQVSTLKAQLHQERHRSSASMSPPSGTPEK

>Canis familiaris_CROCC(ENSCAFP_42060)
MSLGLEESSEAQPTLETVIQTLESSVLRAGQEEGLSAQDPAQDAPRTAGLPARIREIVTR
NLSQPDSQAPLPATEMASVLSLQEENQLLQQELSRVEDLLAQSRAERDELAIKYNAVSER
LEQAVRLESGELETPEPRGLVRQSVELRRQLQEEQASYRRKLQAYQEGQQRQAQLVQRLQ
AKTLQYKKKCSELEQQLVERSTELERQRLRDTEHSHDLESALVRLEEEQQRSASLAQVNS
MLREQLDQASSANQALSEDIRKVTTDWTRCRKELEQREAAWRREEESFNSYITNEHSRLL
LLWRQVVGVRRLVSEVKMSTERDLLHLGGELARASRAIQEADLGLSAGLRLAESRAEAAL
EKQALLQTQLEEQLRDKVLQEKDLAQLKVQSNLDKANLSARVTELALTVERLQNQNLEKD
QVNKVLTEKLEALESLRLQEQAAVETEDGEGLQQTLRDLAQAVLSDVDSGVQLSGSERTA
DASDGSLRGLSGPRTPSPPRRASPGRGRSPRRGPSPACSDSSTLALIHSALHKRQLQIQD
MRGRYEASQDLLGTLRKQLSDSEGERRSLEEQLQRLRDKTDGATQAHEDAQREAQRLRST
IGLLSREKDSLACSLQAAQQQAEELQQEREKLQAAQEELRRQRDQLEEEREATAQDSART

RRELERSHRQLEQLEVRRSGLAKELVEVREALSCATLQRDVLEAEKAEAEALSKAEAGRV
ELELSMTKLRVEEASLRDSLSKLSALNESLAQDKLGLNRLVAQLEEEKAALLGRQRQVEQ
EASLAREEQERLEQLRLEQEVEQQGLEGSLRVAEQAREALEDQLPTLRQERCRLQEQLAQ
LSRQLSGREQELEQARRETQRQVEALDRASREKEALARERAGLAVQLAAAEREGRTLSEE
ATRLRLEKEALEGSLFEVRRQLAQLEARREQLEADGQALLLTKEALTGELVGLRQQVTTT
EEKAALDKELMAQKLVQAEREAQASLREQRVAHEEDLQRLQQEKEAAWRELEAERAQLQS
QLQREREELLARLEAEKEELSEEIAALQQERDEGLLLAESEKQQALSLKESEKTALSEKL
MGTQHSLAAISLEMERQKRDAQSRQEQDRSTVNALMSELRDLRAQLEEAADTHAQEVKRL
QEQARNLERQRESSTREAEELRTQLRLLEDARDGLRRELLEAQRQVREGQDGREAQRQEA
SELRRSLSEGVQEREALRRTNEELRAAVKKAESERISLKLANEDKEQKLALLTEARVAVG
KEAEELRAGLQEVERSRLEARRELQELRRQMKMLDSENARLGRELVELQGRLTLGERTEK
EGRREALGLRQKLLKGEASMEAVRQELQGAQRKLQEQEGEFRARERGLLGSLEEARGAEK
QQLDHARSLELKLEAARAEIAELGLRLSAAEGRGQGLEAELARVEAQRRADEVQLGGLRS
ALRRGLGLGRSPSPPPLPSTSFPTGSAPAGGSGEGLRSPSPLERSPGCEPPSPGPTTSPA
SPDLDPEAVRGALREFLQELRSTQRQRDELRAQMSALSRQLAELEAERDNATSRVRQLQK
AVAESEEARRGVDARLSGAQAQLALQEESVRRSERERRAALDQVATLERSLQATESELRA
SQEKISKMKANEGRLENDKRRLKEVLDASEGRTIKLELQRRSLEGELQRSRLGLSDREAQ
AQALQDRVASLQRQVADSEVKAGTLQLTVERLSGALVKVEESEGLLRDKVQGLTEALAQN
SASLTSSQDKNLHLQKALTACEHDRQVLQERLEAARQALSEARKQSSSLGEQVQTMRGEL
ADLELQRAEAEGQLQQLQEVLRQRQEGEAAALHTVQKLRDERRLLQERLDSLQGALAQRE
AEKREVERSALRLEKDRVALKRMLDKVEREKLRSHEDTVRLNAERGRLDRTLTGAELELA
EAQRQIQLLEAQVVALEQDQSPGRLEADEQRQLELQQEVERLRSAQVRTERTLEARERAH
RQRVRGLEEQVSTLKGQLHQELRRTSASFPSASGP

>Felis catus_CROCC(ENSFCAP_9450)
MSLGLEESVEAQLALETVIQTLESSVLRAGREKGLSEQDPAQDSQLACLPARIREIITRN
LSQPENQAPLPAAEMASVLSLQEENQLLQQELSRVEDLLAQSRAERDELAIKYNAVSERL
EQAVRLESGEPETQEPTGLVRQSVELRRQLQEEQASYRRKLQAYQEGQQRQAQLVQRLQA
KTLQYKKKCSELEQRCPTSPTQDTEHSHDLESALIRLEEEQQRSSSLAQVNSMLREQLDQ
ANSANQALSEDIRKVTSDWTRSRKELEQREAAWRREEESFNTYISNEHSRLLLLWRQVVG
LRRLVSEVKMSTERDLLQLGGELARTSRAIQEADLGLSAGLRLAESRAEAALEKQTLLQA
QLEEQLRDKVLQEKDLAQLQVQSNLDKANLSARVTELALTVERLQNQNVEKDRVNKALTE
KLEALESLRLQEQAALETEDGEGLQQTLRDLAQAVLSDMESGVQLSGSERTGDASDGSLR
GLSGPRTPSPPRRSSPGRGRSPRRGPSPACSDSSTLALIHSALHKRQLQVQDMRGRYEAS
QDLLGTLRKQLSDSEGERRSLEEQLQRLRDKTSGATQAHEDAQREAQRLRGAIDLLNREK
DSLARSLQAAQQQAEELRQEREKLQAAQEELRRQRDRLEEEREDTARDSERTRRELERSH
RQLEQLEMKRSGLAKELVEVREALSCATLQRDMLQAEKAEVAEALTKAEAGRVELELSMT
KLRVEEASLRDSLSKLSALNESLAQDKLGLNRLVAQLEEEKAALLGRQRQVEQEACLARE
AQERLEHLRLEQEVEQQGLEGSLRVAEQAREALEHQLPTLRQERCRLQEQLAQLSRQLSG
REQELEQARRETQRQVEALERAAREKEALAKERAGLAVQLAAAERESRTLSEEATRLRLE
KEALESSLFEVQRQLAQLEARREQLEADGQALLLAKETLTGELVGLRQQMTTTEEKVALD
KELMAQKLVQAEREAQASLREQRAAHEEDLQRLQREKEAAWRELEAERAQLQGQMHRERE
ELLARLEAEKEELSEEIAALQQERDEGLLLAESEKQQALSLKESEKTALSEKLMGTQHSL
AAVSLDLERHKRDAQSRQEQDRSTVNALTSELRDLRAQLEEAAAAHAQEAKRLQEQARNL
DRQRESSVREAEELRTQLRLLEDARDGLRRELLEAQRKVRDSQDGREAQRQEASELRRSL
GEGVKEREALRRSNEELRAAVKKAESERISLKLANEDREQKLVLLTEARVAAGREAEELR
AGLQEVERSRLEARRELQELRRQMKVLDGENTRLGRELVELQGRLALGERAEKEGRREAL
GLRQKLLKGEASLEAIRQELQGAQRKLQEQEVEFRARERGLLGSLEEALCPGSPFADFCS
SGCFILKEVLVYTARAGVGVFVWEMESPGEEVVVGRHPAPGPVAVDGAVGQLRCHLRRQE
GLGPALSMPPPPGSGEGLSSPSPLERSPGSEPPSPGPATSPASPDLDPEAVRGALREFLQ
ELRSAQRQRDELQAQTGVLSRQLAEMEAERDSATSRARQLQKAVADGAEGEWRGACRPPR
VSSGPASSCVKEGGQQSSPRDDTQALLYHTMQLLGLALGSDAAEQEKISKMKANEVKLES
DKRRLKEVLDASESRTIKLELQRRSLEGELQRSRLGLSDREAQAQALQDRVDSLQRQVAD
SEVKAGTLQLTVERLNGALAKMEESEGALRDKAQGLSEALAQSSANLTSTQDKNLHLQKA
LTACEHDRQVLQERLEAARQALSEARKQSSSLSEQVQTMRGELADLEVQRAEAEGQLQQL
QEVLRQRQEGEAAALHAVQKLQEERRLLQERLDGLQRALAQLEAEKREVERSALRLEKDR
VALKRMLDKVEREKLRSHEDTVRLSAEKGRLDRTLTGAELELAEAQRQIQLLEAQVVALE
QDQSPARLEAAEQQQREVEREVERLRSAQVQTERTLEARERAHRQRVRGLEEQVSTLKGQ
LQQELRRSSASFPQTSGPPGE

>Myotis lucifugus_CROCC(ENSMLUP_28)

MSLGLGGSLEAQLALETVIQALESSVLGPGQETGLSVQDPAQDCQPAPLPALLREIVTGS
FSKPESPALLSATEMASMLSLQEENQLLQQELSRVEDLLAQSRAERDELAIKYNAVSERL
EQAVRLESGELETQEPRGLVRQSVELRRQLQEEQASYRRKLQAYQEGQQRQAQLVQRLQA
KILQYKKKGSELEQQLLERSTELEQQRLRDTEHSRDLESALIRLEEEQQRSSSLAQVNAM
LREQLDQASSANQALREDIRKVTSDWSRSRKELEQREVAWRREEESFNAYFSNEHSRLLL
LWRQVVGVRRLVNEVKMSTERDLLQLGGELARTSRAVQEAGLGLSAGLQLAESRAEAALE
KQALLQAQLEEQLRGKVLQEEGLAQLQMQSNLDKADFNARMTELALTVERLQNQNLEKDR
VNKALTEKLEALEALQLQEQPALDTEEGEGLQQTLRDLAQAVLSDMESGVQLSGSEHTAD
ASEGSLRGLSGPRTPSPSRRSSPGRGRSPRRGPSPACSDSSVLALIHSALHKRQLQVQDM
RGRYEASQDLLGTLRKQLSDSEGERRALEDQLQRQRDKTDGATQAQEDAQREAQRLRSAI
DLLNREKDSLAHSLQAAQQQAEELRQEREKLQVTQEELRRQRDQLEEEKEDTEQDRARAR
RELERSTRQLEQLEVKRSGLAKELVEVREALSCATLQRDMLQAEKAEVAEALTKAEAGRM
DLELAMTKLRAEEASLRDALSKLSALNESLAQDKLGLNHLVAQLEEEKAALLGQQRRVEQ
EATLAREEQERLEQLRLEQEVDRQGLEGSLRVAEQAREALEHQVPTLRQERCRLQEQLAQ
LSRQLSGREHELEQARRETQRQVEALERATREKEALAKERAGLAVQLAAAEREGRTLLEE
ATRLRLEKEALESSLFEVQRQLAQLEARREQLEADGQALLLAKEALTGELAGLRQQMTMA
EEKAALDKELMAQKLVQAEREAQASLREQRAAHEEDVQRLQREKEAAWRELEAERAQLQS
QLQREREELLARLEAEKEELSEEILPSEQSPQRPHVSADPPQKEALSLKESEKTALTEKL
VGTQHSLTAISLEMERQKRDAQSRQEQDRGTMNTLTSELRDLRAQLEEAAAAHAQEVKRL
QEQARDLGRQRESCVREAEELRTQLRLLEDARDGLRRELLEAQRKVRESQDGREAQRQEA
AELRRSLGEGAKEREALRRSNEELRAAVKKAESERISLKLANDDKEQKLALLEEARVAMG
KEAGDLRAGLQEVERSRLEARRELQELRRQVPSAARHPPRGGQGLAQQTGNKLVGMCTET
WASGHRVGRWPCWLSPSHLRPLPLPQLQGAQRKLQEQEGEFRARERGLVGNLEEARSAEK
QQLDHARSLELKLEAARAEAAELGLRLSAAEGRGQGLEVELARVKAQRRAAEAQLGGLRS
ALRRVLGLGRAPSPALSSTGRQALGSLARCPPIRGSGDGPSSPSPLERSPGSEPPSPGPA
TSPTSPDLDPEAVRGALRDFLQELRSTQRERDEFRAQTSALSRQLVEMEAERDSATLEGK
AAAEGSIARRSVDGRLSGAQAELALQEDSVRRSERERRAALDQVAALERSLQATESELRA
SQEKISKMKANEVKLESDKRRLKEVLDASESRTIKLELQRRSLEGELQRSRLGLSDREAQ
AQALQERVDSLQRQVADSEVKAGTLQLTVERLNGALAKVEESEGALRDKVKGLSEALAQN
SASLTSSVGNLKELINEREGLWASPAVWLTLQERLEAARQALSEARKQSGALAEQVQTMR
GEMADLELQRAEAEGQLQQLREVLRQRQEAEAAALHTVQKLQDERRRLQERLGGLQRALA
QLEAEKREVERSALRLEKDRVALKKTLDKVEREKLRSHEDTVRLSAEKGRLDRTLTGAEL
ELAEAQRQIQLLEAQVVVLEQDQSPVGLEALEAEEQQQLELQQEVERLRSAQVQTERTLE
ARERAHRQRVRGLEEQVSTLKGHLQQELRRSSASFSPASGPPEK

>Monodelphis domestica_CROCC(ENSMODP_39331)
MSSRDEGPPVASPKLETVIQKLESSLLTSEEEKDLTVQDPGQDGAPSRLPACIREIVTRN
LSEPENPETVQTPEMASLLSLQEENHILQQELSRVEDLLAQSRAERDELAIKYNAISERL
EQSLRLESGEREPLGYGVLGQQTLELRRQLEEEQANYKRKLQAYQEGQQRQAQLVQKLQA
KVLQYKKKCGDVEQQLLEKSTELEQQKLAQEEEHSQDLENALIRLEEEQQRSASLLQVNA
MLREQLDQASVANQALTDDIRKVTSDWTRSRGELEQREAEWRREEESFNAYFSNEHSRLL
LLWRQVVGFRRLVSEMKTSTERDLSQLGIELARTSRTIHSASLGLSGSLRLAESSSEAAM
EKAALLRAQLEEQLRDKVHDLIQLQVKSDLEKAELSSRVHELTLLVEQLQKQNSEKDRAN
ETLTQKVEALEAVRLQEQAAMEAEEVEALRTESEVLQQTLRDLAQAVLSDTDSGIQMGGL
ERTPETSDGSLRGLSSLRTPSPLRRSSPRPSGSPRPSLSPAFSDSTLALVHSALHKRQMQ
VQVGSPATAEPCPVSKGKEKEDVGPSRGFPFCSWSGDGGRS

>Takifugu rubripes_CROCC(ENSTRUP_26160)
EKKLTVRGPSPDAPPACLPARVREIVTKNLSESRELPQSLAAGAMSSVMSLQEENRVLQG
ELARLEDLLAHSRADRDELAIKYSAISERDEQSSNLEDALIRLEEEQQRSSSLSAVNAML
REQLEQAGIANEALSQDIRRLTTDWTKAREELEQKESDWRREEESFHSYFSSEHSRLLML
WRQVVGFRRNVCELKNGTERDLSDMRNELARTCHSVQVSCSSLSSVLHSREGGAVLALER
EKSLRLQLEQQLRERVGEMMNFQTKADAEKSGLSLRLSDLLRESERLKGQIEERDTEIAV
LMRKLKEQSVNDETDMQMMKTHTESLLDTLRDIAQVSCNTNMLAVRGRGLLQEGDSSSEA
DQENSGTPLLALFHSSSTRRSTSPSRSSSQALLTDSALCALRSAVTNKTLQLQDVRGRLI
SSQSSIQQLRKQLSESDLAKREAEQRIQSLQRERDAAQREKETTLKEKERLKEDRDVLAS
EKVSLEKVVQAAQGSSQLLEMESKKLQLIVASAQRERDHEREQKEAAVLERDRAKAETQR
IQKQLEQSESRLSSQRGELSAVRETHQKGEVERQVLEGERAQLSEALARAESRNAELSLL
LNKLQSEEAALRDSLAKVGSLNESLAQDKADLNTYILQLEEEKSVLQAQKREAQQQKLTI
RDELRLEQDRLELDAARITLQHSLQDVELSQVGVDAELQSLREEKVKLQERVTQLCSEVS
SLGSELSLAKGEGQRSEVALEEAGRSRAELARDKAALVVQLTASEKENTALSEELAAFRS

ERESLETSLFEMQQQLVQAESRREQLEAENQNLRLRSETAAVELRRLRSDGENVLAQAER
EKQALTQTLNAAQLEAQQALHKAVSEHQEEVERLVSEKEALRDSLLMEQESALRKLRQEM
EEPLHRAERERE ELQEELRSLQHDRDQSLLQAETEKQQALSLKETEKAVLSDRVSCLQAE
LTAAALEAERMSREAALYKEQEQIRVAALNSELQALRSQLEDASSLHERELQSAREACTD
LQSHTDVALKELDQCRAALAANEETRDQLKRDVTDAERRQNQTVDTLETCRREVSELRRN
LADITHEKDTLSHSNILLRETVRSAESERISVKRQHEEKEQKLSVLEESFASVQKEVTEL
RGCLREVERSRLEARRELQELRRKLKVLDGEKEQKEREVAELQTRLLLEEQREEEKAKEL
FAVRHKLTEAETARDFLKKELSMTQKRLVESESSWRSGERELSAQLQEARGCEKKLQDEA
KNLALRTQAAQDSAAQSGLLLSETQGRLAATEAELIRAEAGKKDLEFRLNSIQSALTRTL
GIGASGRRGRGRSPGGSSTSPGTISRHQSISPLRTSLSPPKEIQVCTPDTTLNSGAASPE
RGEAPLPLPQPELDPDALRSDLRDFMQELRETQRERDDAKCRVGLLERELEEMLGDQDST
QSRLTHLQKAFQEYQEGKRGLEERLSTTQILLQQQEEAVRRADRDRRAVTDRLRDLERAL
QVSETDKKNMQDQLNKQRAAEMRLDSERRRLREALEAAEARATRVELGRRSLEGELQRLK
LSLGDRETESQNSQERYDSLMKQLSEGEARTSLLQRETERLSHSLLKSQEDESILKEKIS
SLRKSLQEAAASHSSHQSRLAALQKMLTEVEHDKRLLQEQLEEARVSAAEGKRSSASISE
RIQSLQGELSHSERRREELEAELRSTQELLQQRTASLTEAQRSAQSAQVERATVEERLRG
LQRAVAMLETEKKDAERQAVRLEKDKNALRNTLDKVERQKLMTEESSMRLSAAKGRLDRS
LNTAEQELQEAQQQILMLQTQLADLEQSHGLCESLVRQREEAQREAERLRSSFREAEQTL
GARERAHRHRVKGLEEQVSTLKEQLLQEMKRRQPSLPSPLMPAGN

>Tetraodon nigroviridis_CROCC(ENSTNIP_17688)
QRLEESVLSEEKKLTVRGPSPDGPPACLPARVREIVTKNLSESACVSGAGAMSSVMSLQE
ENRVLQGELARLEDLLAHSRADRDELAIKYGAISERLEQALRFDTADGDCDAAEPRSLAQ
QTLDLRRRLEEEQAAYKRKLTAYQEGQQRQAQLVQKLQAKVLQYKKRCGDLEQILQEKTS
ELDKHLLSQDEQSSNLEDALIRLEEEQQRSSSLSAVNAMLREQLEQAGVANEALSQDIRR
LTADWTRAREELEQKESDWRREAESFHTYFSSEHSRLLMLWRQVVGFRRNVCELKNGAER

>Gasterosteus aculeatus_CROCC(ENSGACP_1486)
AMSASGNSDSDSNKLESVIQRLEESVLSEEKRLTVRGASPDAPPTCLPARVREIVTKNLN
ETCEWPQSALPSCITSGAMSSVVSLQEENRVLQGELARLEDLLAHSRADRDELAIKYGAI
SERLEQALRFETGDGDHDSPESLGLVQQNVDLRRRLEEEQASYKRKLTAYQEGQQRQAQL
VQKLQAKVLQYKKRCGDLEQMVQERSTELEKHRLSQDESSSNLEDALIRLEEEQQRSSSL
SAVNAMLREQLEQAGLANEALSQDIRRLTADWTKAREELEQKESDWRREEESFHSYFSTE
HSRLLMLWRQVVGFRRHVCELKSATERDLSDMRNELARASHSAQVSCAGLSAALQGREGG
AALALEREKALRLQLEQQLRERVAETMTLQTRADSERSELNVRLTESAREAERLKGQIEE
RDREMVVLVRRLEEQSGHNESDMQAMRSHTETLLDTMRDIAQTVLSDTESSSEADQDNSG
VPLLALIRGFSSHRTSSPRRSSSPSRPSSLAPPSEAALSALCSAVTNKTLHLQDVRGRLL
SAQSAVQQLRKQLSEADSAKRDSEQRNQHLQRERDAAQREREITQRDKDRLKQERDTLAS
EKLSLEKNLQAAQSSTQILQMDCEKLQLTVASTQRERAHDREEKEAANQERERAKAETQR
IQKQWDQSESRASAQRAELSAVKETYQQAEIERQLLERENAQLTEALARAEGSNAELSLL
LNKLQSEEAALRDSLAKMGSMNEGLAQDKTQLNNYILQLEEDKARLQAQKREAEQEKLTI
RDELVRLEQDRLELDSGHTTLHQSLQDAELSRAGTEAELQGLRAETFKLRETVTQLCAEV
TSLESELSLARGESQRNQVALEETGRGRAELVRDKAALVVQLTASERENAVLSEELTAFR
SEREQLETSLFEAQQQLVQVESRREQLETENQNLRVRCATAAADLRRERSDGENVVTQAE
RDKKLLTQTLNAAQLEAQQALRKAISDHQEEVERLVSEKEVVRHSLLMEHEGTLRRLHME
AEDQLRRAEREKEELLDELRSLQHDRDQSLLQAETEKQQALSLKEAEKTVLSDRVSSLQA
ELSAAAVEAERMSREAAQYKEQEQIRIGSLTSELLELRSQLEDAASVHERELHSLQETCT
DLQSRADVALKELDQCRASHLAHEDSRDQLRRELLDCERRLNQCQDAAENSRREGTELRR
NLADVSKERDTLGQSNTQLRVTLRSAETERISVKRQCEEKEQRLAVLEDNLSCNLKEVTD
LRSCLREVERSRLEARRELQELRRQLKVLDGEKEQMGREVAELRRRVSLEEQREEERGKE
LFTLKQKLTEAETARDSLKKEISATQKRLAESESGWRGCERNLTAQLQEARGCEKKLQDE
AKNLALRAQAAQDSAAASSLQLSEAQGRLAATEAELTRAEAGRRDLEYRLSSLQSALTRT
LGIGAGGRGGRGRSPGGRSPGGSSTPPGPMSRHHSVSPLRSSLSPPKDDPELDPDTLRSD
LRDFLQELREAQRERDDARCQLGGLRRELEELSGGRESALIRLTHLENMLQECQEGKRGV
DERLTTTQVLLQQQEEVVRRGDRERRALSDRVNDLERALQTSEMDRKHTQDQLNKQRATE
VRLEAEKRRLREALEAAEARATRVELGRRSLEGELQRFKLSFGDREADSQASQGRHDALL
KQVAEGDACVSLLQRDVERLSQALLKAQDGESFLREKTSSLKKSLQEAEASHSSTQSCLA
SVQKMLSVAEQDKRLLQERMEEARGSLVEAKRNMVILTERVQSLQSELDQSELKREAVEA
ELNNAKEALRQRSASAAEAQRSAQSAQKERAAFEERLRGLQRAVAMLETEKKDAERQAVR
LEKDKDALRNTLDKVERQKLKTEEGSMRLSAEKSRLDRSLNTTEQEVQEAQQQILILQTQ
LAETEQSHGLCESLARQRDDAQREAERLKAGLRDAERTLGTRERAHRHRVKGLEEQVSTL

KEQLQQEMKRRQPSLPSSLLSAGK

>Oryzias latipes_CROCC(ENSORLP_22962)
KAMSAAENSDSDPSKLESVIRRLEESVLSEEKRLTVRGPSPDDPPACLPARVREIVTKNL
SDSSLMCVCAAGEMSSLMSLQEENRVLQGELARLEDLLAHSRADRDELAIKYGAISERLE
QALRSETGEGEADSQASRSLAQQNVELRRRLDEEQAAYRRKLTAYQEGQQRQAQLVQKLQ
AKVLQYKKRCGDLEQKLQEKSSELEERSETSNGRHHEDSSSSLEEALLHLEEEQQRSSSL
SAVNAMLREQLEQAGLANEALSRDIRRLTADWTKAREELEQKESDWRREEESFHSYFSSE
HGRLLTFWRQVVGFRRHVCEMKSSAERDLSDMRGELVRMSHLVQVSCAGLSAVLRSQEAG
TALALEQERALRDQLEQQLRGQVKETMNLQSRADAERSELSLRLSDAAQENERLKSQMEE
RDREIDNLSRRLEEQSRSDETDSQLLRAHCNALLDTLKDIAQTVLSDGESSSEADPDNSA
VVLQALIRDSSPQRSSQTSSSLSPLPEAALSALRSVVTNMMLQLQDVRGRLLSAQSSAQK
FRKQLSEIDSAKRDADQRNQALQRDKDAALRERETAQREKEQLKTMSPTPSEKANLEKAV
QALHSSNQMLQMDYEKLQLAVASAQRERDEREEKEAAIHDGERWKTEIQRIQKQRDQSE
SRASAQRGELSAVLQTHQQGEVERQLLEQESAHLSEALARAASTNKELSLHLSKLQYEDA
ALRDSLTKMGNLNEGLVQDKVDLNKYILQLEEEKAVLQAQKREAEQEKLSIRDELVRQEQ
NRLELESSRVTLQQSLQDTEFSRVGVEAELQNLRAERLKLQEKVAQLCGEVASLDSELSL
AKGEGQRSEMALEEAGRSRAELTKDKAALVVQLTASERENAMLAEELTAFRSERETLETS
LFDAQQQLLQAESCREQLEAENQNLRVRCETATVELRRLRSDAENQLAQAERDKQALTQA
LSAAQLEAQQASRKATSEHQEEVERLALEKEVVRHSLQMEHETAVRKLRQEVEDQLQKAR
REKEELQDGLRALQHERDQSLLQAETEKQQALSLKEAEKTVLLEKVSGLQADLSALSLEA
ERTAREAAQYKEQEQIRVGALTSELQELRSQMEDAAAGHERDILSLNETCADLRSRADVA
LKELEQCRASLSAAEENREQLRRDFLETERRLTQTQEASENHRREGAELRRSLCDVTRER
DALSQSNAQLREALRSAETERISVKRQCEEKEQRLVVLEESLSSSQKEVAELRSCLREVE
RSRLEARRELQELRRQLKVLDADREQKGRELAELQTRLSLEEQREEERGKEVFILKQKLA
ETQTARESLKRELSMTQKRLVESESGWRSCERELTAQLQEARGCEKKLQDEAKNLSVRAQ
AAQESAAQCSLQLSEAQGRLTATEAELTRAEASKRDLEFRLSSLQSALTRTLGIGASGRA
SRGRSPGGSSASPGSISRHHSISPLRPSLSPPKGGGTPLPPAQPELDPDTLRSGLRDFLQ
ELRDAHRQRDDAQCQLMTLQRELEEQKLERECAENRSTQLQNSLKECQEGKRGLDDRLTS
TQILLQQQEDAVKRVDRERRALADRVRELERSLHTAEMEKKHTQEQLNKQRATEARLEAE
RKRLREALEAAEARATRVELGRRSLEGELQRLKLGLGDREAESQASQERHDCLLRQVAEG
EAQVSLLQTEVDRLSQALLKAQEGEAALKQKVTTLSQSLQEAAADHSSTQTRLTALQKTL
SLVEQDKRLLQEKMDEARTSVTEAKRSMFALTERVQSLQGELTQSELLREELEAELKNTQ
EALRQRSGSLAETQHSIQKAQTERAAVEERLLALQRAVALLETEKKDAERQAARLEKDKD
ALRNTLDKVERQKLKCEEGSMRLSAEKTRLDRSLNAVEQELQEAQQQMLMLQTQLAEMEQ
AHSGCEGLLRQRDEAQQEAEKLRNSCKDLERTLSTRERAHRHRVKGLEEQQVSTLKEQLQ
QEMKRRQPPLPSTFTST

>Homo sapiens_CROCC2(ENSP_397968)
MSSASSEPGNGDASQQPLLGLDTVIQRLEDTILSPTASREDRALTVRGEGRQASPTPVPT
RIREIVAGSLSEEPPQAGVQEPTATVARVQEENELLQEELTRLGDLLAQASAERDELASR
CRVVSEQLQARLETTEAQLRRSELEHSVDLEEALGRLEAAEERSTGLCQVNALLREQLEH
MKKANDALGRELAGMTGSVQRLQGELELRRWAQRQTRSGGLGQPRDLLLLWRQAVVLGTD
LAELRVATERGLADLQADTARTARRLHTACLNLDSNLRLSASSTASTLGQQLRDKAGEML
QLQGRWDAEKVALQARLSEQTLLVEKLTEQNEQKAKTIAALRTDLQNLVAQEDARCLELA
GSSITELGEPRRPLRSPQRATSPHQGASPPHICSPATLDPALQAMRAAIERRWRREQELC
LQLKSSQALVASLQEQLSESRRELWAAQKLQQERAREQAREREALRGQLEAQRLEVQQCR
ASCKLLGREKAALEMVVEELKGKADAADAEKQGLEAEAAELQRSLLLQAERREELALRRE
RSCRALETSQGRLQQLEEKVSGLREELASVREALSTAQLQRDVVESEREGLRSALARAEC
SNADLELLVRRLKSEGVEQRDSLAAMAALMEGLAQDKSALNHLALQLEQERDQLREQRKT
LEQERARAGEQLAQAEQQLALERAERRGLQQACGRLEQRQEQLEGQAALLGREKAQLQEQ
VGQVTCQKQALEEQLAQSLQDQEAQMGTLQQALQGKDALSEERAQLLAKQEALERQGRLA
AEEAADLRVERDSLESSLLEAQQLATKLQEQLEEEARSAGLARQALQVEMEQLQSDWEVQ
EMKLRQDTVRLQRQVAQQEREAQRALESQALAHREALAQLQREKETLSLTLAEEKEVARC
QLEQEKELVTKSAAEREALKGEIQSLKQERDESLLQLEHKMQQVMALSLKETERSLLSEE
LSRARRTLERVQQEAQSQQEQAQATISATTEELKALQAQFEDAITAHQRETTALRESLQD
LAAERGDVEREAERLRAQLTVAQEGLAALRQELQGVEESREGLHREAQEARRALSDEARE
KDVLLLFNSELRATICRAEQEKASFKRSKEEKEQKLLILEEAQAALQQEASALRAHLWEL
EQAGGDARQELRELHRQVRTLKAENQRRSGEAHELQAQCSQEVLELRRQAAKAEAKHEGA
RKEVLGLQRKLAEVEAAGEAHGQRLQEHLRESRGAEQTLRAELHSVTRKLQEASGVADAL
QARLDQACHRIHSLEQELAQAEGARQDAEAQLGRLCSTLRRGLGLQRQSPWASPEPQGSP

TKGSDSSQALPGQQGTSPPARPHSPLRWPSPTPGGRSSELMDVATVQDILRDFVQKLREA
QRERDDSRIQMATLSSRLSEAECRCARAQSRVGQLQKALAEAEEGQRRVEGALSSARAAR
ALQKEALRRLELEHLASVRAAGQEKRRLQEQLETLRQALEESRRHSQGLAKQGKLLEEQL
TNLEHRCQKAEVSLEPLRQMEQETLKREEDVARLGAEKEQLDQSLNSLHQEVDGALRQNQ
QLQAQMTEMEQAHTQRLQDLTAQHQRDLATEAERLHGARPQATQALESQEWTHQQQVKVL
EEQVASLKEQLDQEVQWRQQAHLGQAFQTGHAQRD

>Pan troglodytes_CROCC2(ENSPTRP_61067)
SRSTGLCQVNALLREQLEHMKKANDALGRELAGMTGSVQRLQGELELRRWAQRQVLPQPT
RPGGLGQPRDLLLLWRQAVVLGTDLAELRVATERGLADLQADTARTARHLHTACLNLDSN
LRLSASSTASTLGQQLDKVGEMLQLQGRWDAEKVALQARLSEQTLLVEKLTEQNEQKAKT
VAALRTDLQNLVGGRDGGGCVAGVCHPCSGVSPATGSLVAQEDARCLELAGSSITELGEP
RRPLRIPQRATSPHQGASPPHICSPAALDPALQAMRAAIERRWRREQELCLQLKSSQALV
ASLREQLSESRRELWAAQKLQQERAREQAREQEALRGQLEAQRLEVQQCRASCKLLGREK
AALEMVVEELKGKADAADAEKQGLEAEAAELQRSLLLQAERREELALRRERSCRALETSQ
GRLQQLEEKVSGLREELASAREALSTAQLQRDVVESEREGLRSALARAECSNADLELLVR
RLKSEGVEQRDSVAAMAALMEGLAQDKSALNHLALQLEQERDQLREQRKTLEQERARAGE
QLAQAEQQLALERAERRGLQQACGRLEQQQEQLEGQAALLGREKARLQERVGQVTCQKQA
LEEQLAQSLQDQEAQMGTLQQALQGKDALSEERAQLLAKQEALERQGRLAAEEAADLRVE
RDSLESSLLEAQQLATKLQEQLEEEARSAGLARQALQVEMEQLQSDWEVQEMKLWQDTVR
LQRQVAQQEREAQRALESQASAHREALAQLQKEK

>Macaca mulatta_CROCC2(ENSMMUP_41485)
MSSASSEPGNGDASQKPLLGLDVVIQRLEDTILSPTASREDRALTMRGEGRRASPTPVPA
HIREIVASSLSEEPPQAGVQEPTATMARVQEENELLQEELTRLGDLLAQAGVERDELASR
CRVVSEQLQARLETTEAQLRRSELEHSVDLEEALGRLEVAEERSTGLCQVNALLREQLEH
MKKANDALARELAGTTGSVQRLQGELELRRWAQRQTRPGGLEKPRDLLLLWRQAGVLCGT
DGTRGLADLQADTAKTARRLHTACLNLDSNLRLSASSTASTLGQQLRDKVGEMLQLQVRW
DTEKVALQARLSEQTLLVEKLTEQNEQKARTIASLRTDLQSLVAQEDTQCLELSWSITEL
GEPRRPLRSPQRATSPHQGASPPHTHSPATLDPVLQAVRAAIERRRRREQELCLQLESSQ
AVAAGLQEQLSECRQELWAAQKLQQERAREQAREQEALWGQLEAQRLEVQQCRASCKLLG
REKAALEMVVEELKGKADAADAEKQGLEAEATELQRSLLLQAERREELALQRERSCRALE
TSQGRLQQLEEKVSGLREELASAREALSTAQLQRDVVESEREGLRSALARAECSNADLEL
LVRRLKSEGVEQRDSLAAMAALMEALAQDKSDLNHLTLQLEQERDQLREQQKTLEQEQVR
AREQLAQAEQQLLLEQAECRGLQQACGRLEQQQEQLEGQAALLGREKAQLRDQVGQVTCQ
KQALEEQLAQSLQDQEAQMDTLQQALQDKDALSEERAQLLAKQEALERQGRLAAEEAADL
RYARQDGPRSLPSHTPSSPALLRAPRGGPGLQSRGSMVEPLELLLGPHGEREAGHRLRSP
SFRNMNMLRPGEPLHSAFCHSLFFEGGFIPPPSLPNFLTSIPCPVQALSLKEAERSLLSE
ELSRARRVLERVQQEAQSQQEQAQATISSTTEELKALQAQFEDAITAHQRETTALCDSLR
DLSAERGDVEREAERLRAQLTMAQEGLAALRQELQSIEESREGLRREAQEARQALSDEAR
EKDVLLLFNSELRATICRAEREKASFKRSKEEKEQKLLVLEEARAALQQEASALRARLRE
LEQERGDARQELQELHRQVRTLKAENQRRSGEAHELQVQCSQEVLELRRQAAKAEAKREG
ARKEVLGLQRKLAEVEAAGEAHGQRLQEHLQESRGAEQTLRAELHRVTRKLQEASGVADA
LQARLDQACHRIHSLEQELAQAEGARRDAEAQLGRLCSTLRRGLGLWRQSPWVSPEQPGS
PTKGSDGSQALPGHQGASPPARPHSPLRWPSPTPGGRSSELVDVATVQDVLRDFVQKLRE
AQRERDDSRVQMATLSSRLSEAECRCARAQSRVGQLQKALAEAEEGDLLGIGVLSTIRTS
LXXXXXXXXXALRRLEAEHLASVRAAGQEKRRLQEQLDMLHQALDESRRHNQGLAKKGKL
LEEQLTNLEHRCQEAEGSLEPLRQTEQETLKREEDVVRLGAEKEQLDRSLNSLHKEVDGV
RRQNHQLQAQMVEMEQAHTQQLRDLAAQHQRDLATEAERLHGDQSQATQALESQEWTHQQ
RVKMLEEQVAGLKEQLDQEVQRRQQAHLDQAFQTG

>Felis catus_CROCC2(ENSFCAP_23758)
VSLWTAVFQRLEDTVLSPMASREDRALTVRGEGWPALPTPVPARIREIVAGSLRDEPHQG
LQQPPAVSAGVQEENERLQEELSRLGELLAQADAEQDELAGRYHAVSARSFRGLRLLRQD
WAGDAPCGVLRAPGMSGSTPTPAVFRVLLAPAGSQEGCRLWAGLVQLWEGSVPQPPCLLA
RWVTLTLQLQARLETTEARLRRSELEHSADLEEALGRLEAAEQRSTGLSQVNTLLREQLG
HMKKANDRLAEELARTTDGVLHLRGQLELTEARRRAETQIRRPRRGGSQDFLLLWRQVTA
LRVQLAELRTTAERALTDMRAEMARTAGRLQTACLALGSRLRLAASSAAGALEQRLRDQV
RETLQLQGRWDAEKVALQARLSEQTLLVEKLTEQNSKKERTISSLKMDVQKLESRRSRGQ
RAADTLEDQVEALQRVLKSITEVLAHLGPSRGTLKVAQEDAGCPERACSGGPEAQEAQGQ
VRRPGSSASPHRGVSPTRAHALDALHPALQAVHAAIERWRLLEQELRLQLGSSQAVAARL

REQLSEAQRELQAARRLLQERAQEQVREPEDLLRELEAQSLEAQRCRESSELLQREKRAL
ETAVEEMRGKAACADAEKQRLEAENTELHRSLLLWAEQKEELVQQGKRGRRELETSQGRL
EQLEEKVSGLKKELVSAQEALNSARLQRDVLESEREGLHRALARAVGTGLPSLTPSHTRL
SVPSPLVTLPPLLGARAWTRTPPCPHPRQLEQERERLRERQKALEQERAGALERLARAEQ
QLGPAQQACGRLEERLQGREAAFERQRARLQEQVGQVTCKKQALEEQLAQSLQDREAQRD
TLQRTLQEKEALSEERAQLLAKQEALERQVRLTAEEAAELRAERDSLERSLFETQQLVMQ
LQAQREQLEGQARSMRLARQALQVEMQQLRSAWEVQETKLQWDVGRLQRQVAQQERDLQL
ALESQALAHHEDLARLQREKESLSLSLTEEKEVAAHRLEQEKKLVAKNAAKREALKEEIQ
SLKHERDESLLQLENEMQEALSLKEEERRLLREELFRAMQELGQVRQEAQSQQKQAEATV
SATAAELKALQARFEDAILAHQREAAALRDSLREMAAERSNAGREAEGLRAQLDEAREAL
AVLRGELQGSEESREGLRREALEARRALGEEAREKAVLRSSNTELRAALRRAEQDKASFQ
RSKEEREQKVLVLEEARAAAQKEACELRASLREVQQARADACRELQELGTQVNVLRAENP
RKGRESSEPGDRSGCDQGLQRQTRWPQWRRRDGWLQRGRRSALMTLPQVLRLRRELEEAE
AGAEARKQQLEEGLCRSRGAERTLRAELHMVTGKLQQAGSVADGLQARLDEAGRRLHSLE
GELARAEGARRHAEAQLGRLWSTLCRGLGLRGPGPSGSPEWPGFPSTGSESIQSAGPPTR
SSSPLRWPSPAPGDPCPDVEDVASVRDALTDFVQKLRVAQRERDDWHLQVVSLSGRLSEA
ERERAQAQSRAARLQEALAEAEEGGMSVEGERRRAQAARALQEEALRSLEEQHRAGRRAA
SWEKRRLQ

>Bos taurus_CROCC2(ENSBTAP_6833)
PAGLREPLASLGSGPEEHTLLQDELSRLEDLLAQAGAERDELAGRYHTVNERGGEMLGTR
GQWEYKPQKPCSQIRQPKRGCRQLSPEDSPVVWASSRGFPATRPTWGLQAHLCQAARPAG
LGSSRPGGSLPSQLQARLQATTAQLRRSELERSMDLEDALGRLEAAEQRSTSLSQVNALL
REQLEQLRKAHDRLAEELARTTGSVPRLWAELELREAQRRTHREAGRARSGQPQDTLLQR
QVTALRGRLAELRAATEKGLADLRADVARMARRLHTACLNLSSNLRLTAGSEAAALEQQL
RDKVREMLQLQGCWAAEKAALQARLAEQTLLVEKLTEQNASKERAISSLRMDVQRLESQH
GGGGPTAPEDSRAEVETLRLLLDSITQVQAQVAQADAGSEELARSSGAEGEGAQGQRGSP
LHAGNSLRAESPADPDSALWAVRQAIQRWRQREQELRQQLESSEARATGLQEQLSESQRE
LQASRSLLQEERQDLRGKLEAQSREAQWSRMTSELLGREKTALEETVEELRGEAATSHAE
KQTLEARNAELQRSLRQCTEQKEALERQGERGRRALESSQGRLEQLEGTVSGLKTELVSA
QEALDSARLQRDILESEREGLHGALARAESSKADLELLVSRLKAEGVEQRDSLAKMAAVT
EALAQDKGSLNHLVLQLEQERDELRAQQQTLAQGQAGTREQLAQAERQVELLRAERRGLQ
RACGRLEERLERLEGQATRLRRERAQLQEQVGQVTCKKQALEKQLAQSLQDQEAQMAALQ
RALQEKEALSEERVQLLAKQEALEKQGQLMAEEAADLRAERDALESSLFEAQQLASQLQA
RQEQLEGEARGARLARQALQGAAPMKLEQLQSVQEAQETRLRRQAAQQERDAQLALESRA
LAHREDLARLQREKETLSLSLMEEKEAAARRLKQEKELVAKNATRRAALKEEIQSLRRER
DESLLQLQHEKQQSPQPAQERPTPVTQALLLRDAELSRLRGELQLVRQEAQGQQEQAEAT
ISTVASELKALQAQFEDAISAHQTEARALRETLRALVAQRSSTGREAETLRAQLDEAHEA
LAALHRELQGSEETQEGLQKEARDARRALADAAREKDALRDSNTELRATLRRAEQEKASF
KRSTEEREQKVLVLEEAWASAQKQAGELRASLREAERAAVDTQQQLQELRRQVTTLEAEN
QRKSRELVRLQTRGAQEPSRQEALALQTLVAEAKAAREDSQWEVRRLRRMLAEVEAQAKT
REKHLERQLCESRGSERALWAELRGVARKLLQADGVADSLQARPDRACGRAHGLKQEPAR
AEGTQRETEDQRGQLWSMLHPSVGLHGWSPVASPEPAGPPSRGQCPPRPPSHPPGAPQPP
RGSPSLPEKGARASMRDAQRDLTQKLWDAQWEGAGRRLSLGLQAFCLSPLALGEAESEPT
WALSRIGQLQKALAVAEKGWHQVEDKLSSAQAMWALQEEALPR

>Equus caballus_CROCC2(ENSECAP_21159)
AERDELASRHHAVSERDLEHSVDLEEALGRLEAAEQRSTGLSQVNALLREQLEHLKKAND
TLAEELARTTGSVLHLRGELELREAQRRTERQNSNWYLSKWEPRDFLLMWRQVTALRAHL
AELRAATERGLADMRADTARTARRLHRACLNLDSHLRLSASRAADALEQRALQGTRLEQQ
LRDKVREMLQLQGRWDTEKVALQARWVPGSAGPPGLSEQMLLVEKLTEQNSKKERTISSL
KMDVQKLVRGAGRQRPCRGVSVRLAEGSALAAGVPAWRRPAGSGRPEGPSGDRLRSPPRT
ASPYRGVSPPRTRSLAASDPALRAVQAAIQRWRLKEQELRLQLESSQAVVAGLREQLSES
QQELRASRRLLQERAQEQAREYEDLLGKLEAQSREAQHCRATSELLGREKKALESVVEEL
RGKANTWDVERQRLETKNAELQRSLLLWAGQKEELVQRGQRGRRELETSQGRLEQLEEKV
SWLKKELLSAREALNTAQLQRDVLESEREGLRGALARAESGSADLELLVTRLKSEGVEQR
DSLAKMAALMEGLAQDKGTLNHLVLQLEQERDQLREQQKALEQERAGAQEQLAQAEQQLE
LVRAERRGLQQACGRLEEQLEQLEGRAAQLRRERAQLQEELGQVTCKKQALEEQLAQSLQ
DREAQMATLQRTLKEKEALSEERTQLLAQQEALERQGRLTAEEAADLRAERDSLESSLLE
AQQLAMQLQAQQEQLEGEAQSARLARQALQVEMEQLKSTWEVQETKLQWDVGRLQRQVAQ
QERDMQLALESQALAHREDLARLQREKETLSLSLTEEKEVAARRLEQEKELLAKSAAKRE

VLKEEIQSLKHERDESLLQLEHEMQQALSLKEAERSLLREELSRATWELERLQQEAQSRE
EQAEATISATTAELKALQAQFEDAISAHQTEAAALSKSLREMAAERSNAGREAERLRAQL
EEAQEGLAALRQELQGSEESREGLRREALEARQALGDETREKDVLQLSNAELRAAVRRAE
QEKASFKRSKDEKEQKVLVLEEAQAAAQKQAGELRASLREAEQARGEARRELQELCRQVK
TLEAENRRRSQEVSRLQARVAQDAQRQQQSRQEALELQRQVAEGEAAQKEPLRSLLQVLR
LQRKLAEVEAGGEARERQLEERLHESRGTEQSLRAELRGVTGQLQQAGGVADSLRTRLDE
ACRRVHSLEQQLAHTEGARQDAESQLGRLWSTLRRGLGLGARSPSTSPERPGSPTKGQCS
DGSEGHSGRHSASPPGPARPSHGPRLHPEIHGPEVDVASVRDTLRDFVQLRDAQRERDNW
RLQVASLSSRLSEAESERARAQSRMRQLQKALAEAEEGWRQAEGETSRARAAQALQEEAL
QRLQTE

>Loxodonta africana_CROCC2(ENSLAFP_23187)
ETTEARLRRSELEHSVDLEEALGRLEAAEQRSTGLAQVNTLLREQLEHMKKANDALAEEL
ARTAGGVLRLRGELGRREERRQPNMESNGFNCVYWTGESQNLLPLWRQATALQAYLAELR
AATERGLADMRAEAARTARRLHTACLNLDPNLRLSAHSAAGAQEQQLRDKVQEMLALQGC
WDAEKVALQARLSEQTQLVERLTEENTEKEGTIRALTVEVQGLESRRGRGQPSMDDLRDQ
VDALRQVLNSITKVPLPSPALQGLRVFRKKGTELVQSGSTEGYEMLGQPKRPLHTTYPGP
GGLSPPQAQSPAALDPALRAVRVAIERRQQREQELRWKLEASQAVAAGLREQLSECRQEL
LASKGPLWEQAWEHEALLGELEAHRWEAQGCRASEERLGREKAALEAVVEELRGQVDISA
VERCRLEAENVELQRSLLLCAEQGNELAQQEQRSRRELDTSQGHLEQLEEKVSGLKKELV
LAREALNATQLQRDVLEGENEGLRSALARAESSNADRELLVTRLKSEGVEQRDSLANMAT
LMEGLAQDKGTLNHLILQQLEQERDRLREQQRVLEQEQARSLEQLARAEQQLAREQAERR
SLPEGSVQLAREAQLQSRWPVRHLWAGLQCQLPQVTCGKQALEEQLAQSLQDREAQMDTL
QRAMQEKEALSEERAQLLAKQEALERHSRLMAKETADLRVERDSLESSLFEARQLVSKLQ
TQQEQLEGEAQSARLMRQALQVDMEQLKSTGEVRETKLQWDLEQLRRQVAQQERDSQLAL
ESQAVAHREDLARLQREKRPGKVYKMLSKCRMAGTEAQRGRELKGCLCRFLPPPHSSNVQ
RGAASSRAPVSFTCTPSCHLQALSLKEAERNLLREELSTATQELERARQEAQGQQERAKA
TISTVTEELRVLQAQFTDAISTHQKESAALSTSLREVAAERSSMGREAERLRAQLDVAQE
GLAVLRRELQGVEESCEGLRREALEARRALADEAREKDVLQLSNTELRASIRTAEQEKAS
FKRSKEEHEQKVLILEEAHAAARKEAGELRAGLREVERARADARRELQGLRRQVKALEAE
SQRKGQEVGELQARAARDTQLQEQGQQEALELQRRAAELEVAYEGARKEVLGLQRKLAAA
EASGEAQEKQLTAQLRDSRAAEQTLQAEVRSVARKLRLADGTAEGLRARLDSACSRIRGL
EQELAQAEGARRDVETQLDRLCSTLRRSLGLQGQSPVASPERSSSPTKGSDGSQAQTWRQ
STSPPAESCSQLRWPSPGARDVEVLDVVCVRNALRDFVQKLWDAQRERDDARCQAVSLSS
QLSEAESARARAQSRTAQLQEALAEAEEGQRRAEAEVSSVQAARALQEEALSRLEMEHLA
STRAADKERCRLQVEAQLEWASELAGTRRRYLELPVAELPRTSAELRATSVKGNAQEELG
SRGAGAAGLLRRGDGLQSVISQSAVDERRAALTVTRYGAAREESNWSPCSPRREGRGLCF
PLAASALQLILIRKPLLFYEKMCTSGKWSFSNLQEQLDALHQALDESRRHSQGLAATGRL
LEGQLAGLERRCQEAEGTLEPLRQVLRRRQRGEQAAAAAEKRVLREQTAALRTERARLQG
QLAALRARLAQVPSAGPGVGRTRNPGRGSQRRTERETMRREGTAARLEAKKEQLDQSLSA
LHQEVNGALRQNQQLQAQMAELEQAHTQRLQELAAQHQQDLAAEAEQLHGAQLQAAQALE
FRERTHQQRVKVLEQQVASLREQLDQEEQRRRQSPQAAGSAPPSQ

>Dasypus novemcinctus_CROCC2(ENSDNOP_27065)
VLQRLETTVLSPTASREDRALTVRGDGRGASPTPVPARIREIVAGSLSEEPPQGVREPPG
AAARVREENTLLQEELSRLEDLLAQTGAERDELASKYHAVSERSRSCSKRPPGSERVKLG
RGSVVQMGKLRQESDDARYARAPSLCQPFLERNGFAAGLGAGGHGLRAARCGQLQARLGS
TGARLRRSELEHSADLEEALGRLEAAEQRSTGLAQVNALLREQLQHMKTANDRLAEELAR
TTGSVLRLQGQLERGQAWRQAEREFIYFFNIQCIFVKMSFYLSGLGFIRLLVSLIVFITL
DDILVLGATLKKANCGSKSTGEIFSPSAALQNSRQSVPSLPAVFGLAMRVQLLEYHLHVA
QRAVGAGRKHIPRGASSARCHWLNEGVGAPQMWNKGRGSDNGEVAAQEARCHGAQQAVDS
LKDKVGSLQHALSSVSKVAQADTEHPQPAWSSSAEGEEVRGWPRSPLPTATPPPRGRSPP
RACSPAAPDQALQAVQTALERRRQQEQELCLRLESSQEAAAGLREQLSESRRELRASWRE
CERLRGQLEARRCGAARELRDREKEALETALEQLRAKADAADAEKHRLEAANAELRRSLR
GRTEQQEELARRGARSQRELEASQGRLEQLEDKVSGLKKELVAAREALSAAQLRQDVLEG
EGEALRGALARAESSNTALELLVTRLKSEGVAQRDSLAKMAALMEGLAQDKGTLNHLVLQ
LEQERDRLQERQKALEQAGAGAREQLAGVEQQLERERAEKQGLQQACGRLEEQQGQLEGQ
AAQLRHERAQLQEQVDQVFGTRSGGESPLSCLRCPAEAEVSSVSRRAGQSEACKHTGGLA
PVCPPLSRNGMAASERYGPRRAARDSLESSLFEARQLATQLQAQQAQLEAEAQGARRAWR
DLQVEMEQLKSTWEVQGTKLRWELEQSQRQVARLERDSQLALDSQALAHREDLAQLQREK
ETLTVALAEEKEAAACRLEQERELVAKTAGEREALRDEVRSLKQEWDESLLQLEQELQQA

LSLKEAERSLLSQELSGATQQLEQAGQEARSRQEQAEATVSAMAEELRALQAQFEEAIST
HQREAATLNGSLREAAAECSSVGREAEQLRAQLDVAQEGLAALRRELQSSEERGKGLRGE
VLEARRALGDEAREKDVLQRSNAELRAAVRQAEQDRASFKRSKEEKEQKLLVLEEARAAA
QKEAGELRASLREAEKARADVHRDLQELRRQVRTLEAENQRKGQEVSTLQAQRAQDAERR
RQSQREALELQRAGAEAEAARKEVLQLQRKLAEAEAGAEGHLRESRAAERALRAEADGLR
ARLDVACASVCSLEQELARAEGARRDAEAQLCRLGSVLRRGLALGGRSTPASPARPGSPT
KALQPPCPAGSDGLQSASPPAGPRSPLRGSSPAPRDHSAEAVDVAAVQGALTDLVQRLRG
AHWERDDARLQVVGLSARLGEAESGRARALSHAGQLQRALAEAEEGQRRAEAELSSARAA
GTLQEEQLRAGAAEKRHLQKVVAYLHFSCQCSSQPSASWEEARSPAYCDDLKLYGSQKKT
PEPLLASSEAPPTLLCQRAVLQGQNEPQSRILEEESSEPRPLPRHVSGIAKKGAQPRDGA
GCSREPTKRSSGQLAKKAGSRPALPSLGVRGSARVVVTIQNTLAPACFPPLCIPLTARQG
DPVSPLPQEQLEALRQALEDSRRHGQGLGARGRQLEAQLADLGRRCQEADGALEPLRQVL
RRRQRSDQAAERRVLREETAALRTERTRLQGELAALRARLAQVRPVPVRAQRDTVPGCRR
QVFRTESETLRREGDTARLGAKKEQLDQSLSSLRQEVDGALRQSQQLQAQVAELEQAHRR
RRELATQHQHDLAAEAEQPPRAQLQAAQALASPGQTHQRRVKVLEEQVASLKEQLDQEAQ
Q

>Myotis lucifugus_CROCC2(ENSMLUP_17303)
VFQRLEDTVLSPTASKEDRALTVRGEGWQASPTPLPARIREIVASSLGEEPPQGVREPPG
ASAQVQEESELLQGELSRLEDLLAQVGAERDELASRYHMVSERLRNSCDPWPGSSILCAF
QGVRQAAARGRFGYKVRGVGSGGNPPPPCPQGLSSLPQHPRLCSESAHLLQAQLQTTEAR
LRRSELEHSVDLEEALGRLEAAEQRSTGLSQVNTLLREQLEHMKKANDALAEELARTTGS
VLHLRGELELREAQRWTERQTRRSHLGVQPQDFLLLWRQVTALRTHVAELRSASERSLTD
MQAEAARTARRLHTACLNLNSNLQLSASSSAGALERQALQGAWLEQQLRDKVREMLQLQG
RWDTEKVALQARLSEQKLLVEKLTEQNSEKERTISSLRMDVQRLESWRSGGQLAADNLKE
EVKSLRRVLNIITEVAQADSRCPELAQSSSTDREKAQRPLRSPPRTSSLHWGASPPRAHS
RASPDPALRAVQAAIERRRQQEQELRLQLKSSQAVTARLREQLSERQRELRASRRLLQER
EQERGDLLDRLEAQSQKAQHFRAASELLGREKKALELEMEELRGKMDVWDMDKQKREAEI
AELQRSLLLWTQQKEELEQQGERGRRELQTSQGRLEQLEEKVSGLKKELVSAREALNAAR
LQRDVLESEREALHGTLARNADLELLVTRLKSEGVQQRDSLAKMATLMEGLAKDKGSLTH
QVLQSMEAQVCGAWAWRHRCVGHPPPPRQLQQEGDQLREQEQVLRQERADARERLALAEQ
QLEQLEGQADRLQRERAQLQEQVGQVTCKKQALEEQLAQSLRDQEAQMDTLRTALQEKEA
LLEERVQLLAKQEALERQGRLTAEEAADLRYPGAYGQGVCAWPCLREAAVWQGAAEETWR
ASGRRKVLGRESSLVELEQLKGAWEVQETKLEWDIGQLRRQMAQQEREAQLALESQALAH
CEDLARLQREKETLSLSLTEEKKVAACRLEQEKELVAKSAAKREALKEEIQSLKHERDES
LLQLEHEMQQCDTVKYSLSSEWKDKHWVCAHSCSTLRFLRRRKKRXRKAQGTRNRATVRA
TAAELKALQAQFEEAISAHERETSALNHSLRKMAAERSNAGREAEQLWAQLGEAQEGLAV
LRRELQGSEDSLEGLRREVVDAHRALGDEALEKDMLQRSNAELQAAVCKAEQEKASFQRA
KEEKEQKVLVLEEARAAAQKEAGKLRASLQEAERAQLDTCRELRELRSQVKMLETENQRK
SQEVCQLQAQCAQDSQRQQQSWREALELQRQAAEVDAAREDAQKEVLRLQQKLAEVKERL
CESHRAEQSLRAELHGATMKLQQASSVTDSLQARLDRACHRVHSLEQELARAEGARRDAQ
GQLSRLWSTLCCELGLRAQGPSASPKRPGSPTKGLDGSQGHSGRHSASPPARSRSPLRWP
SPAPGQQGPDMDVASVQDALRDFMQKLRDAQQERDNWRAQVRHLSSWLREVERERARAQS
HVGQLKTALAQAEEGWRQAEREAGSAQATRALQKEVLQRLEREHLASVRTAGQEKRRLQV
GTPHPRAGRGK

>Homo sapiens_CEP250(ENSP_380661)
METRSPGLNNMKPQSLQLVLEEQVLALQQQMAENQAASWRKLKNSQEAQQRQATLVRKLQ
AKVLQYRSWCQELEKRLEATGGPIPQRWENVEEPNLDELLVRLEEEQQRCESLAEVNTQL
RLHMEKADVVNKALREDVEKLTVDWSRARDELMRKESQWQMEQEFFKGYLKGEHGRLLSL
WREVVTFRRHFLEMKSATDRDLMELKAEHVRLSGSLLTCCLRLTVGAQSREPNGSGRMDG
REPAQLLLLLAKTQELEKEAHERSQELIQLKSQGDLEKAELQDRVTELSALLTQSQKQNE
DYEKMIKALRETVEILETNHTELMEHEASLSRNAQEEKLSLQQVIKDITQVMVEEGDNIA
QGSGHENSLELDSSIFSQFDYQDADKALTLVRSVLTRRRQAVQDLRQQLAGCQEAVNLLQ
QQHDQWEEEGKALRQRLQKLTGERDTLAGQTVDLQGEVDSLSKERELLQKAREELRQQLE
VLEQEAWRLRRVNVELQLQGDSAQGQKEEQQEELHLAVRERERLQEMLMGLEAKQSESLS
ELITLREALESSHLEGELLRQEQTEVTAALARAEQSIAELSSSENTLKTEVADLRAAAVK
LSALNEALALDKVGLNQQLLQLEEENQSVCSRMEAAEQARNALQVDLAEAEKRREALWEK
NTHLEAQLQKAEEAGAELQADLRDIQEEKEEIQKKLSESRHQQEAATTQLEQLHQEAKRQ
EEVLARAVQEKEALVREKAALEVRLQAVERDRQDLAEQLQGLSSAKELLESSLFEAQQQN
SVIEVTKGQLEVQIQTVTQAKEVIQGEVRCLKLELDTERSQAEQERDAAARQLAQAEQEG

KTALEQQKAAHEKEVNQLREKWEKERSWHQQELAKALESLEREKMELEMRLKEQQTEMEA
IQAQREEERTQAESALCQMQLETEKERVSLLETLLQTQKELADASQQLERLRQDMKVQKL
KEQETTGILQTQLQEAQRELKEAARQHRDDLAALQEESSSLLQDKMDLQKQVEDLKSQLV
AQDDSQRLVEQEVQEKLRETQEYNRIQKELEREKASLTLSLMEKEQRLLVLQEADSIRQQ
ELSALRQDMQEAQGEQKELSAQMELLRQEVKEKEADFLAQEAQLLEELEASHITEQQLRA
SLWAQEAKAAQLQLRLRSTESQLEALAAEQQPGNQAQAQLASLYSALQQALGSVCESR
PELSGGGDSAPSVWGLEPDQNGARSLFKRGPLLTALSAEAVASALHKLHQDLWKTQQTRD
VLRDQVQKLEERLTDTEAEKSQVHTELQDLQRQLSQNQEEKSKWEGKQNSLESELMELHE
TMASLQSRLRRAELQRMEAQGERELLQAAKENLTAQVEHLQAAVVEARAQASAAGILEED
LRTARSALKLKNEEVESERERAQALQEQGELKVAQGKALQENLALLTQTLAEREEEVETL
RGQIQELEKQREMQKAALELLSLDLKKRNQEVDLQQEQIQELEKCRSVLEHLPMAVQERE
QKLTVQREQIRELEKDRETQRNVLEHQLLELEKKDQMIESQRGQVQDLKKQLVTLECLAL
ELEENHHKMECQQKLIKELEGQRETQRVALTHLTLDLEERSQELQAQSSQIHDLESHSTV
LARELQERDQEVKSQREQIEELQRQKEHLTQDLERRDQELMLQKERIQVLEDQRTRQTKI
LEEDLEQIKLSLRERGRELTTQRQLMQERAEEGKGPSKAQRGSLEHMKLILRDKEKEVEC
QQEHIHELQELKDQLEQQLQGLHRKVGETSLLLSQREQEIVVLQQQLQEAREQGELKEQS
LQSQLDEAQRALAQRDQELEALQQEQQQAQGQEERVKEKADALQGALEQAHMTLKERHGE
LQDHKEQARRLEEELAVEGRRVQALEEVLGDLRAESREQEKALLALQQQCAEQAQEHEVE
TRALQDSWLQAQAVLKERDQELEALRAESQSSRHQEEAARARAEALQEALGKAHAALQGK
EQHLLEQAELSRSLEASTATLQASLDACQAHSRQLEEALRIQEGEIQDQDLRYQEDVQQL
QQALAQRDEELRHQQEREQLLEKSLAQRVQENMIQEKQNLGQEREEEEIRGLHQSVRELQ
LTLAQKEQEILELRETQQRNNLEALPHSHKTSPMEEQSLKLDSLEPRLQRELERLQAALR
QTEAREIEWREKAQDLALSLAQTKASVSSLQEVAMFLQASVLERDSEQQRLQDELELTRR
ALEKERLHSPGATSTAELGSRGEQGVQLGEVSGVEAEPSPDGMEKQSWRQRLEHLQQAVA
RLEIDRSRLQRHNVQLRSTLEQVERERRKLKREAMRAAQAGSLEISKATASSPTQQDGRG
QKNSDAKCVAELQKEVVLLQAQLTLERKQKQDYITRSAQTSRELAGLHHSLSHSLLAVAQ
APEATVLEAETRRLDESLTQSLTSPGPVLLHPSPSTTQAASR

>Pan troglodytes_CEP250(ENSPTRP_46519)
METRSPGLNNMKPQSLQLVLEEQVLALQQQMAENQAASWRKLKNSQEAQQRQATLVRKLQ
AKVLQYRSWCQELEKRLEATGGPIPQRWENVEEPNLDELLVRLEEEQQRCESLAEVNTQL
RLHMEKADVVNKALREDVEKLTVDWSRARDELMRKESQWQMEQEFFKGYLKGEHGRLLSL
WREVVTFRRHFLEMKSATDRDLMELKAEHVRLSGSLLTCCLRLTVGAQSREPNGSGRMDG
REPAQLLLLLAKTQELEKEAHERSQELIQLKSQGDLEKAELQDRVTELSALLTQSQKQNE
DYEKMIKALRETVEILETNHTELMEHEASLSRNAQEEKLSLQQVIKDITQVMVEEGDNIA
QGCGHENSLELDSSIFSQFDYQDADKALTLVRSVLTRRRQAVQDLRQQLAGCQEAVNLLQ
QQHDQWEEEGKALRQRLQKLTGERDTLAGQTVDLQGEVDSLSKERELLQKAREELRQQLE
VLEQEAWRLRRVNVELQLQGDSAQGQKEEQQEELHLAVRERERLQEMLMGLEAKQSESLS
ELITLREALESSHLEGELLRQEQTEVTAALARAEQSIAELSSSENTLKTEVADLRAAAVK
LSALNEALALDKVGLNQQLLQLEEENQSVCSRMEAAEQARNALQVDLAEAEKRREALWEK
NTHLEAQLQKAEEAGAELQADLRDIQEEKEEIQKKLSESRHQQEAATTQLEQLHQEAKRQ
EEVLARAVQEKEALVREKAALEVRLQAVERDRQDLAEQLQGLSSAKELLESSLFEAQQQN
SVIEVTKGQLEVQIQTVTQAKEVIQGEVRCLKLELDTERSQAEQERDAAARQLAQAEQEG
KTALEQQKAAHEEEVNQLREKWEKERSWHQQELAKALESLKREKMELEMRLKEQQTEMEA
IQAQREEERTQAESALCQMQLETEKERVSLLETLLQTQKELADASQQLERLRQDMKVQKL
KEQETTGILQTQLQEAQRELKEAAWQHRDDLAALQEESSSLLQDKMDLQKQVEDLKSQLV
AQDDSQRLVEQEVQEKLRETQEYNRIQKELEREKASLTLSLMEKEQRLLVLQEADSIRQQ
ELSALRQDMQEAQGEQKELSAQMELLRQEVKEKEADFLAQEAQLLEELEASHITEQQLRA
SLWAQEAKAAQLQLRLRSTESQLEALAAEQQPGNQAQAQLASLYSALQQALGSVCESRPE
LSGGGDSAPSLWGLEPDQNGARSLFKRGPLLTALSAEAVASALHKLHQDLWKTQQTRDVL
RDQVQKLEERLTDTEAEKSQVHTELQDLQRQLSQNQEEKSKWEGKQNSLESELMELHETM
ASLQSRLRRAELQRMEAQGEQELLQAAKENLTAQVEHLQAAVVEARAQASAAGILEEDLR
TARSALKLKNEEVESERERAQALQEQGELKVAQGKALQENLALLTQTLAEREEEVETLRG
QIQELEKQREMQKAALELLSLDLKKRNQEVDLQQEQIQELEKCRSVLEHLPMAVQEREQK
LTVQREQIRELEKDRETQRNVLEHQLLELEKKDQMIESQRGQVQDLKKQLVTLECLALEL
EENHHKMECQQKLIKELEGQRETQRVALTHLTLDLEERSQELQAQSSQIHDLESHSTVLA
RELQERDQEVKSQREQIEELQRQKEHLTQDLERRDQELMLQKERIQVLEDQRTRQTKILE
EDLEQIKLSLRERGRELTTQRQLMQERAEEGKGPSKAQRGSLEHMKLILRDKEKEVECQQ
EHIHELQELKDQLEQQLQGLHRKVGETSLLLSQREQEIVVLQQQLQEAREQGQLKEQSLE
SQLDEAQRALAQRDQELEALQQEQQQAQGQEERVKEKADALQGALEQAHMTLKERHGELQ
DHKEQARRLEEELAVEGRRVQALEEVLGDLRAESREQEKALLALQQQCAEQAQEHEVETR

ALQDSWLQAQAVLKERDQELEALRAESQSSRHQEEAARARAEALQEALGKAHAALQGKEQ
HLLEQAELSRSLEASTATLQASLDACQAHTRQLEEALRIQEGEIQDQDLRYQEDVQQLQQ
ALAQRDEELRHQQEREQLLEKSLAQRVQENMIQEKQNLGQEREEEEIRGLHQSVRELQLT
LAQKEQEILELRETQQRNNLEALPHSHKTSPMEEQSLKLDSLEPRLQRELERLQAALRQT
EAREIEWREKAQDLALSLAQTKASVSSLQEVAMFLQASVLERDSEQQRLQDELELTRRAL
EKERLHSPGATSTAELGSRGEQGVQLGEVSGVEAEPSLAGMEKQSWRQRLEHLQQAVAQL
EIDRSRLQRHNVQLRSTLEQVERERRKLKREAMRAAQAGSLEISKATASSPTQQDGRGQK
NSDAKCVAELQKEVVLLQAQLTLERKQKQDYITRSAQTSRELAGLHHSLSHSLLAVAQAP
EATVLEAETRRLDESLTQSLTSPGPVLLHPSPSTTQAASR

>Gorilla gorilla_CEP250(ENSGGOP_8041)
MKPQSLQLVLEEQVLALQQQMAENQAASWRKLKNSQEAQQRQATLVRKLQAKVLQYRSWC
QELEKRLEATGELTVKRWENVEEPNLDELLVRLEEEQQRCESLAEVNTQLRLHMEKADVV
NKALREDVEKLTVDWSRARDELMRKESQWQMEQEFFKGYLKGEHGRLLSLWREVVTFRRH
FLEMKSATDRDLTELKAEHVRLSGSLLTCCLRLTVGAQSREPNGSGRMDGREPAQLLLLL
AKTQELEKEAHERSQELIQLKSQGDLEKAELQDRVTELSALLTQSQKQNEDHEKMIKALR
ETVEILVHDPLLWKGVGHGLVDSLCFMICMQCVLTREELANSEYPRDSDNEAMRSTLGLK
HPFTVQRQHPSPIYSPPSPKGEDDADKALTLVRSVLTRRRQAVQDLRQQLAGCQEAVNLL
QQQHDQWEEEGKALRQRLQKLTGERDTLAAQTVDLQGEVDSLSKERELLQKAREELRQQL
EVLEQEAWRLRRVNVELQLQGDSAQGQKEEQQEELHLAVRERERLHREVSLSFEMLMGLE
AKQSESLSELITLREALESSRLEGELLRQEQTEVTSALARAEQSIAELSNSENTLKTEVA
DLRAAAVKLSALNEALALDKVGLNQQLLQLEEENQSVCSRMEAAEQARNALQVDLAEAEK
RREALWEKNTHLEAQLQKAEEAGAELQADLRDIQEEKEEIQKKLSESRHQQEAATTQLEQ
LHQEAKRQEEVLARAVQEKEALVREKAALEVRLQAVERDRQDLAEQLQGLSSAKELLESS
LFEAQQQNSVIEVTKGQLEVQIQTVTQAKEVIQGEVRCLKLELDTERSQAEQERDAAARQ
LAQAEQEGKTALEQQKAAHEEEVQICGQEKERSWHQQELAKALESLEREKMELEMRLKEQ
QTEMEAIQAQREEERTQAESALCQMQLETEKERVSLLETLLQTQKELADASQQLERLRQD
MKVQKLKEQETTGILQTQLQEAQRELKEAARQHRDDLAALQEESSSLLQDKMDLQKQVED
LKSQLVAQDDSQRLVEQEVQEKLRETQEYNRIQKELEREKARRVLGLGLKELINSTSQLE
SRTEKMRNSRLAMPFLSQDVLRDQVQKLEERLTDTEAEKSQVHTELQDLQRQLSQNQKEK
SKWEGKQNSLESELMELHETMASLQSRLRRAELQRMEAQGERELLQAAKENLTAQVEHLQ
AAVVEARAQEREQKLTVQREQIRELEKDRETQRNVLEHQLLELEKKDQMIESQRGQVQDL
KKQLVTLECLALELEENHHKMECQQKLIKELEGQRETQRVALTHLTLDLERARPGADHSE
AADAGTGRGREGPKAYPRTPGAQRPAGAAAPGPAQSQLDEAQRALAQRDQELEALQQEQQ
QAQGQEGALEQAHMTLKERHGELQDHKEQARRLKEELAVEGRRVQALEEVLGDLRAESRE
QEKESQSSRHQEEAAWARAEALQEALGKVHAALQGKEQHLLEQAELSRSLEASTATLQAS
LDACQAHTRQLEEALRIREGEIQDQDLRYQEDVQQLQQALAQRDEELRHQQEREQLLEPR
LQRELERLQAALRQTEAREIEWREKAQDLALSLAQTKASVNSLQEVAMFLQASVLERDSE
QQRLQDELELTRRALEKERLHSPGMEKQSWRQRLEHLQQAVARLEIDRSRLQRHNVQLRS
TLEQ

>Mus musculus_CEP250(ENSMUSP_105247)
METGSPGLNMKPQSLQLVLEGQVLALQQQMAENQAASWRKLKNSQEAQKRQATLVRKLQA
KVLQYRSWCQDLEKRLEATGGLIPQRWESVEEPNLEQLLIRLEEEQQRCESLVEVNTELR
LHMEKADVVNKALQEDVEKLTVDWSRARDELVRKESQWRMEQEFFKGYLRGEHGRLLNLW
REVVTFRRHFLKMKSATDRDLTELKAEHARLSGSLLTCCLRLTLRAQSRESSGSGRTEES
EPARLLLLVAKTQALEKEAHEKSQELMQLKSHGDLEKAELQDRVTELSALLTQSQKQNED
YEKMVKALRETMEILVCGPLCCRGAKKGLAGSLLFVDK

>Rattus norvegicus_CEP250(ENSRNOP_52126)
MEKADVVNKALQEDVEKLTVDWSRARDELMRKESQWRMEQEFFKGYLRGEHGRLLSVWRE
VVTFRRHFLKMKSATDRDLTELKAEHARLSGSLLTCCLRLTLRAQSRDSSGSGRSDESEP
AQLLLLVAKTQALEKEAHEKSQELMQLKSQGDLEKAELQDRVTELSALLTQSQKQNKDYE
EMVKALRGTMEILETNHAELMEHEASLSRNAQEEKLSLQHVIKDITQALASVEEEDTVTQ
TPGSENSLQSDYNDLSQFDSQDPNKALTLVHSVLTRRQQAVQDLRQQLSGCQEAMSFLQQ
QHNQWEEEGKALRERLQKLTGERDALAGQTVDLQGEVDSLSKERELLQTARGELQQQLEV
LEQEAWRLRRVNVELQLQGDSAQGEKLEQQEELHLAVRERERLQETLVGLEAKQSESLSE
LIALREALESSRLAGELLKQEQEEVAAALARAEQSIVELSSSENSLKAEVADLRAAAVKL
GALNEALALDKVALNQQLLQLEQENQSMCSRVEAAEQLRSALQVDLAEAERSREALWEEK
THLETQLRKAEETGAELQAELRGAREEKEELKEKLNEAHHQQEAATAQLQQLHQDAERQE
EALARAGQEKEALVRERAALEVRLQAVERDRQDLAEQVLGLRSAKEQLEGNLFEAQQQNS

VIQVTKSQLEVQIQTIIQAKEVIQGEVKCLKLELDTERSHAEQERDAVARQLAQAEQEGQ
AALERQKVAHEEEVNRLQEKWEKERALLQQKLDKTLETLERERTELEMKLREQHTETEAL
RAQREEERTQADSALYQMQLETEKERVSLLETLLQTQKELADASQQLERLRQDMKVQKLK
EQETTGMLQAQLRGAQQELKEAIQQHRDDLAAFQKDKIDLQKQVEDLTSQLVTHNDSQRL
VKQEIEEKVREAQECSRIQKELEKEKASLTLSLVEKEKRLLVLQEADSVRQRELVSLRQD
IQEAQEGQRELGVQVELLRQEVKEKEADFVAREAQLLEELEASRIAERQVRASLWAQEAK
AAQLQLQLRSTEAQLEALVAEQQPENQAQAQLRSLYSVLQQALGSACERRPELRGGGDSV
PTPWGSDPDQNGASRLLKRGPLLTALSAEAVALALQKLHQDLWKAQQARDDLRDQVQKLV
QRLTDTEAQKSQVHSELQDLQRQLSQSQEEKSKWEGKQNSLESELRDLHETMASLQSRLR
QAELQKMEAQNDRELLQASKEKLSAQVEHLQACVTEARAQASAAGVLEEDLRTARSALKL
KNEEIESERERAQALQEQGELKVAQGKALQENLTLLAQTLSNREREVETLQAEVQELEKQ
REMQKAALELLSLDLKKRSREVDLQQEQIQELEQCRSVLEHLPMAVQEREQKLSVQRDQI
RELENDREAQRTALEHQLLDLEQKAQVIESQRGQIQDLKKQLGTLECLALELEESHRKVE
GQQKVITELEGQRELQRVALTHLTLDLEERSQELQTQSSQLHELENHSTRLAKELQERDQ
EVMSQRQQIEELQKQKEQLTQDLERKGQELVLQKERIQVLEDQRTLQTKILEEDLEQIKH
SLRERGQELASQWQLMQERAEDGKSPSKAQRGSLEHLKLILRDKEKEVECQQERIQELQE
HMGQLEQQLQGLHRKVGETSLLLTHREQETATLQQRLQEAKEQGELREQVLQGQLEEAQR
ALAQRDHELEALRQEQQQTRGQEESMKQKTSALQAALEQAHVTLKERQGELEEHRGQVQR
LQEELIVEGRQVRALEEVLGDLRAESREHEKTVLALQQRCAEQAQEHEAEARTLQDSWLQ
AQATLAEQEQELEALRAENQYSRQQEEAAFGQAEALQEALSKAQATLQEKEQRLLEQAEL
SCTLEASTATLQATLDTCQARARQLEEALRVREGEIQTQALQHQEAMQHLQQELCQKEED
LRQQDEQRQLLEKAVAQRSQENGIQEKQSLEQEKEEETRGLLESLKELQLTVAQKEEEIL
MLREAQQRQNQEASSPSHRSFPAEKPSLQLLLAQQDLERLQNALRQTEAREIEWREKAQG
LALSLAQSKASISSLQEIAMFLQASVLERESEQQRLQEELVLSRQALEEQQSRGPHSSSR
ADQGPKAGQDTEPGEVIEAEPSPGVGEKEQLRQRLERLQQAVAELEVDRSKLQCHNAQLR
TTLEQVERERRKLKRDSLRASRAGSLEARETMNSSPTQQDGRGSQRGSSDSMLMVELQRE
VALLRAQLALERKQRQDYIARSVQTSRELAGLHHSLSHSLLTVAQAPEATVLEAETKKLD
ESLNQSLTSPAPFLHPSLDTTQNTHR

>Canis familiaris_CEP250(ENSCAFP_40980)
METRGPGLNNMKPQSLQLVLEEQVLALQQQMAENQAASWRKLKNSQEAQQRQATLVRKLQ
AKVLQYRTWCQELEKQLEATGGPTPQRWESMEEPDLEQLLVRLEEEQQRCESLAEVNTQL
RLHMEKADMVNKALREDVENLTVDWSRARDELRRKESQWQMEQEFFKGYLKGEHGRLLGL
WREVVTFRRHFLEMKSATDRDLTELKAEHVKLSGSLLTCCLRLTVGTQSRESDGSGRRDG
SEPTQLLLLLTKTQELEKEAHERSQELIQLKSRGDLEKAELQDQVTELSALLIQARKQNE
EYEKMLGALRETVEILETNHAELMEHEVSLSKNAQEEKLSLQQVIRDITQVMVEEGDSMT
QGCGRDSSLELDPSGLSSQFDSQDPDKALTLVRSVVTQRRQAVQDLRQQLSACQEAMSSL
RQQHNQWEEEGEALRQRLQKLTGERDTLAGQTSDLQGEVESLSKERELLQKTREELQQQL
EVLEQEAWRLRRTNMELQLQGDSVQGEKEEQQEELHLAVRERERLQETLASLEAKQSESL
SELIIILREALESCHLEGELLRQEQTEVTAALARAEQSVAELSSSENSLKAEVADLRAATI
KLSALNEALALDKVGLNQQLLQLEQENQSVCHRMEATEQARNALQLGLAEAERSREALQE
KNTHLEAQLQKAEERGAELQADLRAIQDEKEEIQEKLSEARHQQEAALAQLDQLRQETKR
QEEVLAREVQEKEALVRERAALEVRLQAVERDRQDLAEQLLGLSSAKEQLESTLFEAQQQ
NSLVEVTKGQLEVQIQTVIRAKEVIQGEVRCLKLELDSERNQAEQERETAARRLAQAEQE
GQTALQQQKSAHEEEVNRLQEKWEKERSWHQQELDKALESLEKEKMELETRLREQQAEAE
AIRTQREEERAEAESALCQMQLETEKERVSLLETLLQTQKELADASQQLERLRQDMKVQK
LKEQETTQILQAQLREARGELEQAAQQNRDDLVAVQEECGALLQAKMDLQKRVEELKSQL
VSRDDSRRLVEQEVQEKLREAQEYSRIQKELEREKASLIQSLMEKEQRLLVLQEADSVRQ
QELSSLRQDMQESQEGQKELSTQVELLKQEVKEKEADFLAQEAQLLEELETSQVTEQRLR
ASLRALEAKAAQVQLRLRSTENQLAALVAEQQPGHQAQAQLASLCSVLQQALGFACESRP
ELRGGGDSASLWGPEPDQNGTGILLKRGPLLTALSAEAVASALQKLHQDLWKTQQARDDL
REQALKLEQRLTDTEAEKSQVCTELQDLQRQLSQNQEEKSKWEGKQISLESELTELHGTV
ASLQSRLQQAELQGLEAQDELELTRQALEKEQLLSSSSTSRAEQRPREEVSEVEAEPSLG
LEERQLWGQRLEYLQQAVAQLEIDRSRLQHHNVQLRATLEQVERERRKLKRESMRVSRTG
GLEVKEAAASSPTQQDGRGGQKGSSDDKQMAELQKEVAMLRGQLALERKQRQDYIARSVQ
TSRELAGLHHSLSHSLLAVAQAPEATVLEAETRKLDESLTQSLTSPGPALLCPSPSTIQA
ISR

>Felis catus_CEP250(ENSFCAP_2972)
METGGPGSNNMKPQSLQLVLEKQVLALQQQMAENQAISWRKLKSSQEAQQRQAALVRKLQ
AKVLQYRTWCQELEKRLEASGAKGPISPRWESVEEPSLEQLLVRLEEEQQRCESLAEVNT

QLRLHMEKADMVNKALREDVEKLTVDWSRARDELMRKESQWQMEQEFFKGYLKGEHGRLL
GLWREVVTFRRHFLEMKSATDRDLTELKAEHVRLSGSLLTCCLRLTVGARSGEPDGSGRL
DGSESTQLLLLLTKTQKLEKEVHERSQELIQLKSQGDLEKAGLQDRVTELSVLLTQAQKQ
NEDYEKMLKALREAMEILEKNHAELMDHEASLSRNAQEEKLSLQQVIRDITQVMVEEGDG
MTPGCGHESSLELDPSGDSSQFDSQDPGKALTLVRSVVTRRRQAVQDLRQQLSGCQEAVS
SLRRQHDQWEEEVKALRQRLQKLTGERDTLAGQTTDLQGEVESLSKERELLQKTREELQQ
QLEVLEQEAWRLRRTNMELQLQGDSAQGEKEEQQEELHLAVRERKRLQETLAGLEAKQSE
SLSELITLREALESSHLERELLRQEQTEVTAALARAERCVAELSSSENSLKAEVADLRAA
AAKLSALNEALALDKVGLNQQLLQLEQENQSMCSRMEAAEQARSTLQLGLAEAERSRDAL
QEKNTHLEVQLQKAEETGAELRADLRGIQEEKEEIQEKLSEAHHQQEAAFAQLEQLHQEM
KRQDEVLAREVQEKEALVRERAGLEVRLQAVERDRQDLSEQLLGLSSAKEQLESNLFEAQ
QQNSLIEVTKGQLEIQIQTVTRAKEVIQGEVRCLKLELDTERSRAEQERENAARQLAQAE
QEGHTALQQQKSAHEEEVNRLQEKWEKERSRHQQELGKALESLEREKMELEMRLTEQQAE
TEAIRTQREEERAEAESALCQMQLETEKERVSLLETLLQTQKELAEASQQLERLRQDMKV
QKLKEQETTELLQTQLRDARQELEQAAQRDRGDLAGLREECRALLQAKTDLQEQVEDLKS
QLVSKDDSQRLVEQEIKEKLREAQEYSQIQKELESEKASLIQSLVEKEQRLLVLQEADSV
RQQELSSLRQDLQEAQGGQKELSAQVELLKQEVKEKEADFLAQEAQLLEELEASRVTEQQ
LRASLRALETKAAQVQLRLRSTENQLAALAAEQQPGQQAQAQLASLCSVLQQALGSACEN
RPELHGGGDSASLGAPEPDQNGARILFKRGPLLTALSAEAVASALHKLHQDLWKTQQARD
DLREQAQKLEQRLTDTEAEKSQVCTELQHLQRKLSQNQEEKSKWEGKQISLECELTELHE
TVASLQSRLWQAELQGIEAQNERELLQAAKENLMAQVERLQASVAEARAQASAAGALEED
LRNARSALKLKSKEAETERERAQALQEQGELKVAHGKALQENLAILAQTLSKREGEVETL
RGNIQELEKQREMQKAALEVLSLDLKKRNQEVDVQQEQIQELEKCRSVLEHLPMAVQEQE
QKLAVQREQIKELEKDRETQRNILEHQLLELEKKAQVIESQKGQIQDLKKQLVTLECLAL
ELEENHHKMECQQKAIEELEDQREVQRLALTHLTLDLEERSQELQAQSSQIHELESRSTL
LARELQDKDQELQSQREQIEELQRQKEHLTQDLERRDQDMVLQRERIQVLEDQRTLQTKI
LEEDLGQIKLSLRERGRELASQRPLMQERAEEGKGQSKAQRGSLEHMKLILRDKEKEVEC
QQERIQELKEHKGHLEQQLQGLHRKVGETSLLLTQREQELVVLQRHLQEAREQGQLKEQS
LQGHLEETQRALAQRDQELEALQQQQQQVQGQEESMKEKASTLQRALEQAHAVLKERQGE
LEDHKEHVRRLREELVVEGQRVQALEEVLGDLRAESREQEEALLALRQQCAERAQEHEVE
IRGLQDSLLQAEAMLKERGRELEAVQADGRSSQHREETARARAEALQEALSEAQATLQEK
EQRLLGQAELSRNLEASTATLQAALDSCQAQARQLEEALRRREGEIRDRDLRHQEAVQQL
QRALAQRDDELSHQKRQGQLLEQSLAQRDREDQGKQGEGREREEEEMRGLRESLRELQLT
LAQKEEEILGLREAQQRKNLEDSPHSHTASPAEEPSTQFDALGPRLQQELERLQAALRQT
EAREIEWREKAQDLALSLAQSKASVSNLQEVAMLLQASVLERDSERQRLQDELELTRQAL
EKERLFSPSLTSRAERGPREEVGVQLEEVPGVEAEPSPGMEEKQLWGQRLEQLQQAVAQL
EIDRSRLQRHNVQLRATLEQVERERRRLKRDSMRALRTGILEVSEATQQDARGGQKGTSD
AKHMAELQKEVALLRAQLALERKQRQDYIARSTQTSRELAGLHHSLSHSLLAVAQTPEAT
VLEAETRKLDESLTQSLTSPGPVLLCPSPTQATSR

>Myotis lucifugus_CEP250(ENSMLUP_14802)
METGGPGLNNMKPQSLQLVLEDQVLALQQQMAENQAASWRKLKSSQEAQQRQAVLVRKLQ
AKVLQYRSWCQELEKQLEATGGPIPQMWDSVKEPNLEQLLMRLEEEQQRCESLAEVNTQL
RLHMEKADVVNKALREDVEKLTVDWSRARDELIRKESQWRMEQEFFKGYLKGEHGRLLSL
WREVVTFRRHFLEMKSATDRDLTELKAEHVKLSGSLLTCCLRLTVGTQSRESDGSGRLDG
SAPAQLLLQLAKTQELEKEAHERSQELIQLKSQGDLEKAELQDRVTELSALLTQSQKQNE
DYEKMVKALRETMEILETNHAELMEHEASLSRNAQEEKLSLQQVIKDITQVMVDEGDNMS
QGSGHESTLELDYSDFSSQFDSQDPDKAVTLVRSVLTQRRQAVQDLRQQLSGCQEAVSFL
QQQHNQWEEEGEALRQRLQKLTGERDTLAGQTVDLQGEVDSLSKERALLQETREELQQQL
AVLEQEAWRLRRTNMELQLQGDSAQGEKEEQQEELHLAVRERQRLQEMLAGLEAKQSESL
SELITLRENLESSRLEGELLRQEQTEVTAALARAEQSIAELSSSENSLKAEVADLRAAAA
KLSALNEALALDKVALNQQLLQLEQENQSVCSRMEAAEQARNTLQVGLAEVERSREALWE
KNTHLEAQLQKAEETGAQLQADLRSIQEEKEELQEKLSEARHQQEAASAQLEQLHQEAKR
QEEVLAREVQEKEALVREKAALEVRLQAGERDRQDLSDQLLGLSSAKEFLESSLFEAQQQ
NSLIEVTKGQLEVQIQTVTQAKEVIQGEVRCLKLELDTERNRAEQERDTAARQLAQAEQE
GQTALQQLNSAHEEEVNRLQEKLEKECSRHQQELDKALETLEKEKMELEMRLREQQAETE
ALRTQKEEERAKAESALCQMQLETEKERVSLLETLLQTQKELADASQQLERLRQDMKVQK
LKEQETIGMLQTQLREARRELEQAAQQHRDDLTALQGESSSLLQDKIDLQKQVEDLKSQL
VSKDDSRRLVEQEIQEKRREAQECSRIQKELERDKASLTLSLVEKEQRLLVLEEADSVRQ
QELRSLHQDLQEAQGGQKELSAQVELLKQEVKEKEANFLAQEAQLLEELEASRVTEQQLR
ASLWAQEAKEAQLQLRLRSMESQLEALAAEQQPGHHAQAQLASLCSVLQQALGSVCESRP

ELSGGGDSAPSFWGPEPDQNGTRILFKRGPLLTALSAEAVASALHKLHQDLRKTQQARDD
LRNQAQKLKQHLTDTEAEKSQVQTELRDLQRQLSQNQEEKSKWEEKQKSLESELMELHET
VASLQSRLRRAELQGMEAQNERQLLQAAKENLTAQVQHLQASVTEARAQASAAGVLEEDL
RTARSALKLKNEEVESERERAQALREQGELKVAQGKALQENLAILAQALSEREGEVEALQ
EKIQELEKQREMQKAALELLSLDLKKRNQEVDLQQEQIQELERCRSVLEHLPMAVQEREQ
KLTVQREQIKELEKDQETQRNVVEHQLLELEKRDQVIESQKGQIQDLKKQLVTLECLALE
LEENHRKVECQQKAIEELESQREVQRVALTHLTLDLEERSQELQVLSSQVHELESHSSLL
AGELQEKDQEVKSQREQIEELQRQKERLTQDLQRRDQEMVLQKERIQVLEDQRTLQTKIL
EEDLEQIKLSLRERGRELASQRQLMQERAEEGKGQNKAQRGSLEHMKLILRDKEKEVECQ
QERIQELQEHKGQLEQQLQGLHRKVGETSLLLTQREQEIVVLQQHLQEAREQGELKEKSL
QGQLEEAQRALAQRDQELEALQQQQQAQGQEEVVKEKAGTLQRALEQAHMTLRERQGEL
EDQREHVQRLQEELTVEGQRAQALEEVLGDLRAEFQKQEKTLLALQQQCAERAQEHEAEA
KALQDSWLQAEAMLKQRDQELEALRADSQSSQHQEEAARGQVEALQEALSKAQAALQEKE
QHLLGQAELSRSLEASTATLQAALDSCQAQARQLEEVLRKREGEIQDRDLRHQAAVQELQ
QALAQRDGELRHQRKQRQLLEKSLAQRLQEDEREEEMRGLYESLRELQLTLAQREEEILE
LREQRKNLEASAHSRKASPVEEPSTKLDSLGPRLQQELERLQAVLRQTEAREIEWREKAQ
DLALSLAQSKASVSSLQEVAMFLQASVLERDSEQQRLQDELELTRQALEKERLHSPGPTS
RTEQGPRGEAGGQLGQVSGGEAEPSAGTQEKQPWEQRLEHLQQAVAQLEVDRSRLQHHNI
QLRTTLEQVERERRKLKREFLRTSRSGVLETSEATASSPTPQVYLFIDCSLSCSLPLGFP
AQQVALLRAQLALERKQKQDYIARSVQTSRELAGLHHSLSHSLLAVAQAPEATVLEAETR
KLDESLTQSLTSPGPVLLCPSPSPSPRASQ

>Monodelphis domestica_CEP250(ENSMODP_2186)
MEARDQGHNMKPQPLNMVLEKQILALQQQMAENQAASWHKLKISQEAQQRQAVLVRKLQA
KVLQYRNWCQELEKRLEDMGGPMPKRWTSAEEPRLEQLLIRLEEEHQRSEGLVKVNTQLQ
EHLEEADVVNKALQEDVGKLTVDWSQAQDELARRQSDWQMEREFFKGYLKGEHGRLLSLW
REVVTFRRHFLEMKTATDRDLAELKAEQVKISGSLLTSCLHLSLELQSREAQGSGRLAGN
EQVHLQLVAKTQELEKEIHKRSQELILLKARGNMEKTELQDRVTELSALLAQSKKQNEEQ
EKMLKALKDTMDILETNRSELMEHEASLSRNSQKEKLSLQQVIKDITQVVVDDEDSENQS
FGLESSLEMESSIFCQLRSEDPDKAVTLVRSILAKKHQAVQGLRQQLSTCQEAVSFWQHQ
HTQWEEEGEALRQRLQKLTGERDVLAGQTQGLQGEVDSLNRDRGHLEKAREELQQQLEIL
EQEAWRLRRLNTELQLQGDLAQGEKVEQEEELQQAVRERDCLQETLKGLEARQSSSLSEL
ISLKEALEKSHLEGELLNQERAEMARALAKAEQSIAELTGTERTLKAEVADLRDAAAKLS
ALNEALALDKVELNQQLLQLEQENQSVHGKVEASEKARSAVQMELDESEQRKEALLEEKA
CLEMQLQKSKDAEAELQGDLSREREEKIETQEKLNEAQRQLEVATTELEQLHQETTRQEL
LREKVIQEKEILVRDKAALEVHLKALERDQQDLSEQLLELSSAKELLETNLFEAQQQGSL
MEITKEQLMMQIQTVTQAKEVIQGEVKCLQMELEAQRQQAEQEQDTAAKHRDQMEQESQA
ALQQQRATYEEELHLLQEERMDQIKHRQELEATLEQMKREKVDLEETCEKKLRELQEESE
AALAQRIKDHAQAESNRCQMQLEMEKERMSLLETLLKTQNELADAGHQLEQLRQDIKDQK
LKEQENMEDLWAQLQEAQKTLMETRRKHHNDLAALEEEKSILLQQKENLEQQVQDLKAQL
VTSEDSCRLAEHEAQKHLQEVQQCTHIQEEREQEKASLVQSLAEKEQRLLILQEADSLQQ
QELSSLRQEVKEAQEEQEQLSAQVLFLKEEMQKKEASLLDQEILLEKLGASKKTEQQLRA
SLWAQEAKAAQLQLQLCSVESQVGALILEQQNGHQAQAQLMGLYSILQQAFGSAIESRPE
LSGAGDASPLSWSPRTLEPGQENFLKNQIAVTNFTTEDVASLLHKLQRDLWQARHTRDDM
KCQVEKLEQHLSQAEAERNQFSTEIQELQSLLSQNQKEKLKWEGEQSSLETELAVLQERV
ASLQNSLKRSEQQRTEAQNEQATLWAANEKLVSEVEHLQASVAVTKAQANAREALEAELR
ATHSALRMKNEEAESEREKAEALRKKLAQGKALQENVDLLTSTLSKREKELEALQGVIQE
LEKQRESQMVMQEHLSLNLKKRSQELESRQEQIQELEKQKAGLEHLPVALEQKKKQIRLQ
REQIKELEGKQETQGNILEHQALDLQKKTQTLESQREHIQDLENQLVTLEHLVLEVKKNH
QEIESQKKKIEDLENQREMQRVTLTHLTLDLEEKSQEIQSQNKKIKELEKHNALLTVNLE
ERCQEMKSYRDQVEELKKQKDQLTWDLEKKNQELVLQEEKILVLEDQRMLQNRVLEEDFE
QMKLALREKDQEIESQRQLMIEREEEEKGQAKAQRSSLEHMKLILRDKEKEVEYQRERIH
MFQQYGEQREEQLQGLRRKIGEMTLLLTQKDQELETQQQHIRRVQERRESRERALQDQLE
RVQEALQTKVQDLEALQQKAQLQQESQEEQMSTLQAALEQARAALEEHEEAIEAHKEQNQ
KVQQQQTAAELQVQALEELLGDLRAEFQEQEKVLETLRQQCTEQGHEQETQAWHQHTEAR
QWEKTTAGQVQALKEALRVAQASLQKKDQELLQQAELSQRLEGSAAALQVALDSCQTQAR
HLEEALREREEEIREQQLQHQEMVQQLQLALAQRDGELRQLKEKGQQQEAALNQSDKGEE
KEEKVLRQNLRHLQLTLSRKEFQLQETHRRHGQESYYESHSPEKMEDGEEEEVYGTGTME
KMSLREISPVSMEEAKLQEELERVKNVLRQTEAREIEWQEKAQALAFSLAESEASICNLR
EAALFLQAKTAEQNTEQLQLEEQLQLTRQALEDEQSHNRGSAWRTEPEIQGDEGTSQRKA
TGPEDDGSCTELEKTQQWQQRLKYLLHAMASLEHERNRLQRHNFQLRVTLEQVERERRKL

KRNFNRMTHTTPPLPRETTALTPGQKQEKGGQENSSELKHVAELQRQVTALQTQLALERQ
QKQDYISRCVQTSRELTDLHHNLSNSLWAVARAPEANILEAEARKLDKSLTQSLTSSAPS
PPCCTPRSAQATSRS

>Gallus gallus_CEP250(ENSGALP_55060)
MAAREAARGQACLRRRLHGAEEAQQQQAVLVRKLQEKLLQYRSWCRELEQRLEAGGVSVQ
GSLSYRWETTEDHSLEKALLQLEEEQQRCENLAEVNTLLREHLDKANEVNLALKEDVGKL
TADWMRAREELELKESEWRSEREFYDSYLRGEHNRLLSLWRQVLTFRRHFLEMKTATDRD
LSELKAEQMRLSGSILVSCFRLNSGVQLWESVTLGRPVLKDQAQQQVEREINQEALEVMC
LQVDEDLEKKELQDRVMELSALLVQSQKQNEEKEKTVKTLNDTVEMLEASRLEVEYEASL
TKNAKEENLALQKLIKEITEVVLDDSDSTVGIVQRPESSNVLSCLSSLDAEHAFVLVQET
LARRQKAIQALKEELSTRQDSISFLQHQHRQQEEKCKKLQQRLEQLEEENKMSNSHQQHL
KSLVLALKCDCENLEKIRGELQQKLELSEQEASRLRQSNTELQLKEDSAQGEKVEQQLAM
ERAHHDHELVLKDLAALEEKHSLLQNELVAAREKLEESHLQRDLLKQEKHELTVALEKAE
KSVAALTGAQNKLNSEIADLHTAAAKMSSINEALALDKVELNKLVLQLEQENELLSDKVN
EMERAKVSEQEKITLYKRTNEELCAEKSHLEKLLKKAEEQQEELQMQLRILAEEKEETLE
KLNQVYRQQESASSGLEQLRQQSSRQEDAVAKVSKEKEFLVHEKAALEVRLAAVERDRQG
LSEQLAEARSVKDTLESSLFEAQQQLSHLEITKSQLEIQLHTVVQAKEVIQGEVKCLQYE
LETERCLMKQEQKNMAQRLIQIEEQHNNTLKLQQTDHEVEINKLLQDLASEREGRQLELQ
EMLELWEKEKAEAEGQHEKKLFHMKEKVATMQAQQEEERTRVESANQEILTEKENEKKAL
LETLLQTQGELTEACHQLEQLRQEVKEQQEYEQNITEKLQAELQETHCKIKMVENMHKEE
MENIKEEKNILLQQRDDLQKQVEELTSQLAASEESHQAIGHKAQQELSEAQELSRQKVLE
VVHLQKILEEERGQWEEVEHQNKELQVCLQSLEGERSRWEEVERHNTEFQASLKALESER
ERLSLSLEQKELSLKTLEENNLVQQNEVSKLHSAIQQAQQLHSDHRREIQELNNQVQTLQ
EVVLEKEASLAAREKQLLQDLEESRAGERCLRDSLHVLEAEMAELHLRLCSTENRAKALA
TECQQANNAHCEAQSQLDKLHLVLHHVLCDSTDKSRDLVAWSSGKSGRDENYVECLGMKE
NMEGRDGSSQSY

>Anolis carolinensis_CEP250(ENSACAP_5625)
MEGDQAAHSMKMESAHLSDDQLVSVHQQMIESQASYRNKLQNSQEAQHRQAILVQKLQGK
VLQYRNWCRDLEQRLEASRASYSLRQDNKKDHSLEKALIQLEEEQQRCQNLAGVNILLRD
HLDKANEVNGALREDVSKLTADWTKARDELEYKESEWHKERKFFESYMRAEHDRIIGIWR
QVVTLHRYFLEMKTATDRDLSELKVEQMRLSGSILISCFQLSSDTRLLEMSNLESSVLKG
SLQQQQQQLYLKPEKTDMEKEIYQQTQAIMNLRVKGDLAREDLQSRVIELTALLEQSQKE
NEEKEKTVKTLRDTLEKSNHLELEYEQEQSRSKTVNEEILSLRHVIKEITEAVLNGNDAT
VGKPQVEQSAELGHSLQLDPGGLDGTLLLVREALMKRSNEVQHLRQQLLASQNSITSLEK
QQEQQEEHSRFQGQRLEQLEKERETFTSQIQHQQSTIETYSRDYATLEKSRTELQQHLEV
LEQEVWHLRQNNTELQLKGDMVNGEKEEQQQELQRMMREREYIQEDRNALEERHSSLKNE
LIALREALEKSHLDGELMKQEKYELASALEQTEQSLAQLTSAHNKLKAETADLQGATAKM
SALNEALALDKVGLHQIILKMEQESQMLSDNVDRLEKMKSLSQKQLKQAEQTNEELCTEK
IRLEKLLQKAEELQEGLQDELKMLKEEQKETYEKLSQVHHQHEKSISSLEQAHQESNRLG
EMYAKVSKEKEKLMCEKATLEVRLGAVEREKSALTEQVTELRSVKDLLESSLFQAQQQVS
QLEVAKGQLEMKVHAISQAKEVIQGEVKCLRIELEAEKSRIEQENEERVQLLSQAEQKHQ
ETLSHRQIVHKEEMNAVFQELEGERERHRVELEEALKRSAKEKEESESTCEQKLLELQKN
ITAIQTRMEDELFKAQNAKQELLFEKENERKSLLDELLQTKDKLTSTCQQLEYFRQEIKK
HQEEAQVTVQGLEADLKDAQHRMKTLEKKHKEEIKSLKEEINILLQQRDALRSQVQELES
QLLIAKDSCQSISQEAQHHLREGQELSKQKELEIAHLRKMLEAERKRCEEISHQSIELQS
QKEEMEHRNTELQAMLQNQWEAEKHLNRELQRKQEEVQRQCATLQALEREQNLWVEADRQ
NSEQHNVLQRQLEEAKFQNKQLQTQKREADLQNAKLLHQKEELECQNAQLKDALQSQREE
AECESTILQSHWEEAQQRYSELQNWKEKIEDENAELQALLSHQREKMESQNMELQSKQKE
IEHQNIVLQKMKEERSHWEEVEDQNSELKAALQTMEQKRASLALSLKETEDRAKKLQESD
DAHKDEIAKLSTALQLTEQLLAEYRKEMQELNNEIQSLKQKTAEKEAALVAQQEQLIQDV
EESHTSEQRLKDSVQVLEAEVSTLRQSLRSTESRAEALASECQRACSARWEAQSQLSKLH
SVLQYMLCNGLEVKVEHGGHGDNNIWNSLSLRTEQDKLIRPALSQSKDFSTELTVERVTE
AIQKLRKDLWQSRLERDEVRNNMEKLKQQLIEKEGERDRISAKAQELQKWFNESQEEKNK
AEGKRGSLESALKTEVIAFKEENVTLRRRLITLEKTLESTEKQRKDMLDERDSLQMAKDK
LSWELDLLRESIRASEIQTNAMEKINQSLEQELQTALSALKTKNEELETQQKKLATLHQE
AEIGKTLQENVDRLTASLEKKEEEIKFQLQQIKDLERHREVQKTTMEHLAESLEKRTQET
ECQKNQIEEVKKQEKMQKADLERLNLELEKSLQIMQSQQEKIQKLEDLTQTQSTILKSLT
LDLEAKVKETQIKEEKIQILEKNESSQIGSLLKELGDLKMQLTEKDWELASQKQLLQECE
EQGKKQEKCLHTRLEHMKAILMDKEREVASREEQIAGFQQHEAQRDSHLCDLNEKVKELA

FTVAQRDEELESQRQQIREMEGEMETELKTLRELTAKTQLSLKERNIELEFQRKHAEEVE
RQIKILLGDVQISTAVLNLEDNNAEFQKDQKEQLKKQTVLNMLEQLRVLLIRKEREIKSL
KEQQDEHKEENEKTVREMQTNLQQALENKEVQLQCLKEENRQLHQLEEKAAAQTKSVMEN
LEFTRTSLHDREKEIQSLKGHIREISRQKEVEQNKAVSLLNDLNKIKQSRTEKENELLRQ
VEHIRTYQVKLEATQAELQACQTRVNELQSVIKQKEEENERDFKRLRHKHEPVQENDKHP
GENEEYLEEKQRKKGEEMDAQNKQEKIKDEMKMLQENIQQLQHLLKKKDDEVEWQKRKVE
ELKERVKSKEKELLISSEHLKEIVSFLNLQDNEDDNLQSHIQKLHMWKELEIVRSKALQE
KDQWLTRQKEKIQQLEDEKKVKKQELDHVTAILKKTESGEIEWREKAQRLDLSLAQSEEA
LRALREEMTIMQNMVSERDRDRHNLQEQLDTAFRTLEEERTATKALVGDVDQFISEKHLP
EILGKEGEEAMRKGGKLSWVDERRFLCRRLQLLQQAVARLENDYRVLEQHNTQLREALKQ
VEHERRRLKRSCGDLSLSFGLSQINAGPLKIPSIEKEETQVLHKELEKLQKQVFVLQKQL
SLQKKQKQDYIDSCARTNHELSGLHQELSDSLAAVAKEPEATVLALETQKLDETLNYSLS
LASGSCNGFFAGKHLISSTPKRLQQKDSK

>Ciona intestinalis_(ENSCINP_32049)
MLREQLVSANDSNAALADDLRKVTDDWNDVKQELKTKESEWREEQENFSSYFGDEHSRLL
ALWRAVVQIRRGFGDLKSNTQRDLAQAINDVQRGARQLHASCLNLNANQSVQDTSAKVSL
EKERGEKGDVEEQLRRKVKEMLSMQSDFDTEKARLNGRINELTVAYDRAKSQLSDKDRTI
DDLRTP

>Strongylocentrotus purpuratus_(XP_011663951)
MLREQLDQATEANRSLNHEIDQLSNEVKRSQDDLDNRETEWRDEEKSFNEYFNSEHSRLLVLWRQIVAFR
RNFAEMKSSTERDLTQMRSDVTRTSRNTQSACQNLQSASQGSETGAVMALEREKGEKMQLESQMREKLKD
MLDMQAKFDAEKGDLTTRINELTIANERYKIQVAEKENQLRQRSASAAGGDHFDSAQSEASQEAYMLPGA
DGGSLRAVQEECEALHMALRDIAQAVIQDADASAADMSEFAAGQEGDVTLDVSLASPYAKSTPLRPLSPT
RYNRHRSPSPRRPVTPSRRTASPSRARSPGFPDSTYSAVQAALQKRQLQVQELKARLNSTRDQASTLKKN
LEGSENERRQTERAVDAHRDNLSVSQRQLEEIKRDRDRLRNSLEATGSEKSGLENLRQSLNAQIESLNVE
NERLQAANSDLQRQRDHLEDEREDREKDSIRQKKEIERSHKLLEQMEGKNSNLKEDIVTLKEALNKAVLE
KDVLEQEKAEISESLARLEVQRAELEVELNRLRTEEAGLRDSLLKMQALNEGLGQDKIELNKIIMQLEAE
KNRLIEDKDNIHHERAAIRDELMRVESEKVDTETEKMGLNQSLSLMEDNRTRLEEEMSLLNREKGELGDN
LSQLSRQKNSLAEELLQTRRDVERQIEAVTRIAKEKEELTKEKAELIVQLTASERENRAQAEVIAAMNTD
KESLERTLYEYQQSLAKLEAKRTQLESELQEQGVSKEGVTVELARQRKEMEIEMTRYQKDLELLNQRMVQ
QERDTQLALKQRQQAHDEDVERLNRERESLKLAMEAEKEDLVRKTNQEREELNGRYMQEKEELTEDLMGL
QRERDESLLLAENDKQQSLSLAQTERNQLVEKLNSSQRDMANASMEMDRIKREAFTRAETDKEAIRDVQD
ELKELRARFEEGTNVRERQAKDLSNQIKDLQKVKEALLREVNELKTQLKMAEESRDGVRRELIEAHRKIR
EGDEGREIQRKENMELKRQMNDEVREKDAINRANEELRQKVKKVETDRIQLNRNVEERTQKIAVLEESKT
AIQKEAGDLRASLREVEKSRLEARRELQELRRQVKTLDTDKAKLTKDIHDLQNRVARDDEKEEENRKEIY
ALKQKVMETEAERESARKDAQNLTRRFGDLEEELRLKEKDYAMSVDEARSAERRISERLRTTENALDETK
ADLGDLKLKLSAAEGRVNGLESQLAQVEGAKQEVEFKLGSLHSTLRRTLGIGAGGMGSSLSLASSTSPGP
RSSLRDRSPSPVRARRTRSPVKGFDNTFASTADGFGTPIPRTGSPELQDRSASPLPTRSTSPTRMEQLVA
DIDPETVRIALKDFSQQLKDTERERDEAFCRIRLITQELAENEAARHRVDQRLQQFQKSLTEAEEGKRGA
DGRLSSAQTALMLQEETIRRSERDRKGMQEKINTLERSLQSAEAEKRNQSDRIAKMNQGSQRMEGDKRSL
RETLEASENRCTQLELKRRALEGDLQRSHLSMNDKETESQVLQDRIEALMRQIQDLESKNTSLQLTVDRL
SSSLARSEEGEHTLKDQLQSLSLSLNDTAMESTQKQEQLHHLQQAVANSEKDRRVLQERLDAARHGASDA
KKQNHQLIERVQQGQNDLAECELRRAELEQQVRSSQQLVKQHQESEETTMTQIQRFQAEKRSLKEHVTTL
QKAYSNLETEKREVQRSAGRLEKDKTALKKTLDKVEREKMRHEEAVMRATSEKGALDQSLTSLDQENIEL
QRQTQALQATLAETEQQHAQRLIELTTRHRQETEIETERLRTAQLQAERTLEARERAHRQRVKGLEEQNS
TLKDQMHHEVRKRQQYISMSSKTNDEIQDLRNMLSDSLHSVARDPTLDPVLLEHETRKLDESMGYSSSLR
RSARGASPPTSLSPRLAKDRNRSISPVRLGKPASTSTPGMDSKLRSSSGRTRSARGLSPSSLRGSLRK

>Drosophila melanogaster_(FBpp0308351)
MQAYRDNFKQTPCPSAVDLQAAGPAHQSATASRLPFSRSQRGRDPSASGASVSASVAGGL
TPRMMSPGPPGAGGGGGGGVSGGGSGDPSALLRQNQELRQRLADESHSYRRRLDTYKQAQ
HNQANLVSRLQSKIQQYRQRCSDLEERMHETIKPTAGVGPKLTTGPTNQVLCSTSLTLGQ
SSLPCSSSLDSPPPSCSRDYVDDVLVTGGGAGAAELCRKLEEEHQRCEQIVAQNSALRQQ
LEESNRTNEALTNDLQKLTNDWASLRDELLIKEDEFKEEEQAFKDYYNSEHNRLLKMWRE
VVAVKRSFKEMQTAMKAEVAKMGQEINCVGKDINGSNATVAFAVQQAKRAADDELKQSQR
SNDELQNQLATLKVQYESARHEIMERDQRLLELMNQLKKLEDRCAQAESQAALASRYSDE
IERLNNSMREIAQAVVQDAENADREADAEVTGGVMQHMHLTRDAASVVGGAGGAGSTAGG
GGKSPRRNSTRASQAFAEGTISAVQAALHKYQLALHDMQVKFQNTSETLRTTKAQLETSE
GTKQLLTTKMQQLTEKLDSSNSKLSELLQERESLQRGLDDIRVQKQQSEMGRADINSAFE

NLSSDYEKMQLNCGKLQKRIDSMEEDKKAVELEIQRILKDKNITELNLRSEEDRSSRLRE
ETISLREELNRVSLNRDLLEQQRIESDNLINLLEKQKSDLEYDLDKLLLEKCDLQEKHEK
LSNNSCSTSDELKSVQNCLQEAQEERKKLRIQSVDQCNEIGELKKELAILDKARLELETD
NLSAGEKLKCLQLEKEKILQDLACVTRDRGDIHNQLTAMCRKKEALNEELMRTRQRLEQT
TETNSRLNRNLEEMVKDVEEKQVVIDLHEKDTHRLNELLAALRSEKESLESVLFDTNTSL
EATEERRSQLERDLQEALVREESLKNHVARLQKELEQCQRKAQETKTQLLNAARAAESDF
NQKIANLQACAEEAAKRHGEEILQLRNALEKRMQQALQALQTAKDDEIEKLQERLATLQA
HLESLVQQHEEALIRAESEKQQALLIAHRDKQAVAERLEAVSRDLKTEQESLDRSRREAN
ARDEKQRAAIAQLKDEMVQMRTKEEEHKIKLEECIRKQELQLSSLREERESLCRVSEELK
MEIRLKEDRMESTNNELQDALRKSKEGEGFIDSLRKELTDCRRQLADSNIERDKYSGSNK
ELRDHVKRVESAKREQARAIEEALQKISNLEDTKNSLENERTRLSTILKETENHFTKTTQ
DLNATKAQLQKAQVEFAQKDEGGKELQCKLVAEVELKERAQQELCQIKKQLSDLEANLCA
TRQELGRARCQNNQEEHRFHAREQELAQRLEEGRGREKRLEDQKHNLEVCLADATQQIQE
LKARLGGAEGRIRALDEQLSCVELHKRDTEQKLSSVVHTLRRIAGIQVDGSVNLSHRLLS
PSRRFSPSRSCGDYDNRSTSQCPDGPIDVDPDLVRKGVRNLMHQVAQLEREKDDYKSQLG
AAKKQLQDAADQQLRCDAKLGKLQAMLRNLQEEKSNLETDRKMKISAIQALEEKLKHRND
ECQMLRERLAQTEMQLAATSEENGQNEERLEKSRQQCSKLDNEKRQLQEELAKVEGRASK
LELQRVAMEGDLTRLQMALQEKDCSIRQMAERLENQNRALTQLEDRCTALKSTVDQLKER
LQKSAVSETQLRGEIKTLQKELSEQGHCSQANEDKLKLVQKSLQTAENEKRILTERLDSA
QTNLNELRRSQQAQLDGNQRLQEQVTDLEVQRSALESQLRIAKWNQESGGDKDLTNGNGG
GNGEEELSRQLKSSQREKSELRSKLQTLQDKVKQLECDRKSKFSGGNAYDRAEKSNSFYG
GAAESGEFDSNRYDVGGGNAGGGSFNCGLDHSVIEQETRDLRLKVRRLETLLAEKESELA
RCKARMNDSAKCHDGLDGDRYRSAQMHAEKLLDAREQSHRQQVLRLENQISMLREQLAQE
AKRRQQYILRSSKANREMQHLRSTLGDSLRNVSQHPVDPHLLESESRRLDSAVSMSLPPS
SCRDYDRD

>Apis mellifera_(XP_006559453)
MSRPLPKNISQLSARGQMERRRPPFLRGPSRMREKIERGIGSKMDSTGSTLSGTGDCLSEEDLSPDALVR
QNYELRHRLEEEAANYKRRLDTYRQAQQHQAALVSRLQAKVLQYKQKCSELENQMAESIVYDSNKVVSSA
PPTTSVLDAAHQTLREIREEQIYDLDTALKKLSEERKRCEKLLQLNTTLKDQLEESHQTNEALTNDLQKL
SNDWDILREELAIKEEEWREEEQAFNDYYTSEHNRLLILWRDVVSVKRLFADIKSSTERDLLKMKNNIIS
TFNDVSSACNNTGFAMRMQAAMHPMISQQVQQAQEQVTTDLKTELITLKQQYDVAQNEIRIKEEKINQLI
RDVHSLEERCGEAETEIHRNTRLQDDIEILQSALRDIAHAVIQDAESREIESTQAPPHLHLSPTGPIPQK
SPKRGTRSNTIPAFAESTISAVQAALHKYQLTIHELQVKLQTNKEQLQNTRKQCENAEENVKTLEKKAEE
LIIELDAVRLHCSQLNQEKDMLQKGLDTIRIEKNTLEKTRIELNNMMENLNNDCEKLQKANNKLQKICDN
LEDEKLYLQSELNRLSKDVELRELNLRSEEDRCSKMREELLTLREELNKTYLSKDMLEQQKLETDGLISQ
IEKSKGDLELELERVLLEKSDVQEVLMKLETVCSNHEQEKQRLQEELKKVTEEKNKLANQCTDQQGDLGS
LRKELLQAEQTRLDLESEKVTLNEKVKFLEIEKEKIEIELAQVTRERGDFNNQLSVLARKKETLNEELMR
LKQRLEQANEMNGRINRNLEDLVKDNEEKQVLLETNEKEVQRLQEQLASMRSEKETLEGVLFDTQTSLEN
MHVKKTQLEKEQKELLIKQESLKGQVERLMKDLENSEKRIHEVKQTLTQQSGDQEAEFQQIISNVKKHSE
ENIKKLNEEKEQIKINLEKRLQQSLLQVTGEKDNEINQLQQRIDEMQQHIENLCQQHEEVLLRAENDKQQ
ALLIAHHDQQALMEKLETVLHEMEEEKNNVERVKREAAVKTEQERNNINQLRDELNRLKTKLDETRLKTD
EEKIKLDLKIEELWKERELAQRESEELQVQLHMTEDKVDSLQNQLHETIRKLKDAENLNETLRKELVDIR
RQLGDSTYEKEKYNSSNKELREHVKRIESEKREQNRILEESYQKISALEDMKVNVDAERSRLQAQIRDME
KEMLQLQKQLHFTQDELQKSHQSNAQAQNDEKELQARLTNETEERERLQLQLHQVKKQLMDVDNSLKVTR
QELGRLRSRADEENERWRVREQELVVRLEDSRCRERKLEDQKHNLEVCLADASQQIQELKARLGGSEGRV
RALDAQLSQLEITKKEVEQKLSSVGSTLRRIAGIQMDGSVNIPFKLLSPSRRWSPARAQDHIDSSRDVIL
DVDPEIIRKGVRSLMQQVAQIERERDDYKTELCNLKKQLDENQEIQNRSDIQINTLLTNIRMLQDEKNSL
EVKLSQKQSGYEMQSEECEQLREKIINLELMISNNSEEKIQFEEKLDKLKQVLNKVENEKRNLQEELSKS
ESRATKLELQRMSLEGDLQRLQMMFQEKDANIHKLQERNDTQNRTMTTLEERCTSLKSMVEQLNLALEKA
STTENELKNEINSMQHNIMELTTTLQTSNEKNKQLQKQISNAENERRILSERIESMQQSLNDLKHTNQTL
TDQITRLQNELANNEVQRCALESQLRIVAYPTQEENINKDEELLRQLQIAQRERSEMRGKMEALNDKMKL
LEADKRNLERQLSLFKSTNRSKSYERYEKAHTELLGTSFDIDHYEQENRELRLKVRRLETQLAEKEAELI
RLKSSYTHTHSVFDFNRDRTGEIERLRAAQLQAEKLLEAREQSHRQQVLRLENQIQLLREQLNQEIKRRQ
LYVLRSSRAGREMQQLRQALGDSLRTVAQDPSLDAVLLEHEARKLDSTLTSTTSLPPSLALPPPPSYDRS
STPAQLK

>Anopheles gambiae_(XP_311738)
MTTVLNGKDCAPSLGLRHSEGGGGGGGGSALADRSKKPFSRPFNKSRLQHEPSSTTTGGGVSGTGGPPAT
TARMPSSTGSSSGPPSAADTSALIRQNNELRHRLQEEANNYRRRLETYKQAQNNQAALVSRLQAKVLQYK
QRCSDLEQLPPSAGSCSGGGGGGGGGRYAGGSGSREPSPGSRHLHHAHHAPSPPPSAGGGGGGGPICTDV

TGGPLSLPSACPVARERARSRSRSQSPCRKYSEGSHDEIRHQLDEERRRCEKLLVENSCLRQQLEESHRT
NEALTNDLQKLTNDWESLRDELLSKEDEWKEEEQAFNDYYNNEHTRLLKMWREVVQVRRMFNEMTSTTKM
ELGRMHLELSGTVREVSGACSGVSVNLKQSSKFEEAQQMHIERENIELKSQLSELRLQFEAAKQEIGQRD
QRLQQMAQELKLLDDRYTQADSQAAQAHRMNDELERLQAALRDIAHAVVQDAETSASADMGGCGGGSAGE
GGQHHLHLSQPIVSPGVAPRSPKRASGGMAGTGGSLRASQAFAEGTISAVQAALHKYQLAIHDLQVKLQT
NNDALKATRKQYDSCEHARDTLSGKVSELTEKLDSANHQLSELYKERDSLQKTLDGLRSDKHTVERGKAE
LNSIVDSLNTDYEKLQNVNSKLQKMYDALEEEKKMIELELQRVHKDKDIQELNLRAEEERCSRLREETIT
LREELNKLYLSRDLLEQQRIESDGLLNMLEKQKLELEFELDRVTGERNELHSTLEKRTNSNEHQEQEIRQ
LKTTVAQLDEERGKLRAQSSDQSADLASLKKELISAEQARLDLDSEKLAISERLKCLEMEKDKIEAELSC
VARERNDLSNQLATISRKKETIGEEVMRLRQRLEQSNEMNGRLNRSLEELVKESEEKTVTIEGHEKELQR
LQEQLASLRSEKESLEAVLFDTNTSLEEAEAKKDALERENQDLLIKQESHKALIARLNKDLENAERRAQD
IKIQLTNAAANQEAEFLQKLSNLRTFSEENIKKLNDEKEQIRQSLEKRMQQSLQALESAKDAEIRQLREQ
FETLQLHLDALNQQHEEVILRAENEKQQALLIAHRDKQAVIEKLEVTARELKNELENGDRLKREMAARQE
KDRTTIGCLRDEVTKMRTKAEEARIRAEEEMNRLEVVAGSLREEKETLLKDAEELKVQLRLSEDRCDALN
HQLQDTHRKLKEAENSTDATRKDLTDTRRTLADSNIERDKYANSNKELRDHVKRVEEQRRQQARTIEDAL
AKISSLEETRNGLEQDKVRLQTILKETETNVTKLNQELSNAQTDIQKFQTDTTQKDASEKELQARLANEG
EEKERVLAELHQIKKQMADLEGTLCATRQELGRARCKANQDEHRFHQREQELCARNEEGRGREKRLEDQK
HNLEVCLADATQQIQELKARLGGAEGRVRALDEQLVQLECQKKEVENKLSSIGHTLRRIAGIQMDGSVSL
PYRLMSPSRRYSPARGGTGGRSEGGEHHHHDRHHHGHHHHHHGHETRSLSGENVLIDVDPEMVRKGVRSL
MQQIAHIERERDDFKVQLCTARKQLQEGSDAQLRLETKINKLQQHVRAVHEDKTNLEARLAQKTSALQST
EETLRQKSDELTALREKATQLEQSLTSTSEERTHLEERLDKCRQSGARLESDKRHLQDELARTEARATKL
DLQRVALEGDIQRLQMAMQEKDCTVRNQQERLENQQRSLTQLEDRCVALKSTVDQLKERLQAAAITETEL
RGEISGLQRHNADQSHTFALGQDKLKQLQKTLSGSENERRVLAERLDAAQHTINELRRNQQASQDSTTRL
QEQLAECEVQKSTLESQLRLAKWNQETSEQLAATGGNLGGGSGGAGGNTSGDQELTRQLISSQRERNELR
NKVEALNEKIRTLERDQQRPAGKYDRSEKKSGEYDSNRIESTTYSCGLDHAQLEQEGRELRMKVRRLETL
LAEKEAELARSRAKLLESPSKASSLGGGSVGADVERYRSAQVQAERLLDAREQSHRQQVTRLENQIAMLR
EQLAQEAKRRQQYILRSSRAGREMQQLRQTIGDSLRNVAQDPLDTSLLESEARRLDSAVSMSLPPTTSAT
GRGRGEYDRSLSPTFR

>Capitella teleta_(ELU12564)
MSTDSTDYSGSRSYKRRSGGGGWERTSRKVENLAHSLKDTNRNLKAVDKMLLDYKDASEQQNSVIDKLQG
DVSRSRHRIRDEKARIRGSSLRGCSEEDLSRDSRRRSRMPTSPLRNYDRAASDHEFADDWQPTRNSVRFY
DDMDTPPRLHRLHQSVRDVSSEQLRLEDDLQQEILRRHRIETDHNKTLAALLEQTKQADPALNSVEQRLQ
SIQDELRSERTFLRRKQDEVGKVSQELKQQPANVVYDGRVNAVENEAQLRARLTQVETQKTAMESELERT
KQRLEQVDGNKSTLITEVNDMRRQLSRVEHERDQLGLQLEQRERDLKAQLKTRSLDEQQDGLREQEIKRL
KQDLEEARSQLTKSASVVGDYGELRRELDKSERLRSQLSDHIQVGVALF

>Hydra magnipapillata_(XP_012555552)
MSFLDEKPPSDKSNDEDLPSLQSIIKRLEENEIKIPTKETLTNSEIPISMRDVTKVMSNDVADAEKLIEE
NRLLYDDLLWSQNQLSLMKSEKSSLNSSKLPLYNDYMRMQSPVNSSYNNESVRIQSPVSSFLSVENYILQ
INQLKQKIEDDHSNYKRKLHAYQEGQEKQSELIQKLQSKVQQYKMRNNELEMIANDKSNELEELRDKLQV
TIASAKEYEKRLMEFDEDRSNEMENYAVQLEEEKQRNVNLQHANNMLHEQLEQATSANSHLSLETQKLSI
ELTKSCDELKHRDSEEENYFTEEHRKLVNLWNSIDSFRSAFNIVKLETQKDLLQVKAEFSKSARTIQAAS
MELEKTQRGLSTVNQNTLLEEQAQREKVESQLREKIRELIDLQATLNKMKDHYEIRLDEITNSLTESKAR
VDEQQKTIGLYKNKLKQLGIEYSETTENWKNDSQVNIEAMREEIEVLQSTLYELANCILEDADSTSSSLD
YLLSKVEAEIDTDNDIDPSSKVKDIVQSSSPLIHVKLRSKSPVRARSPAYIQSLVAGSQSAMKKRQLQLQ
EQKRQLDIAVMDKQSFKRQFDISSSKLQQLNTAYDELKTDHLSLQSKYNKSNEDCNKYRNQLQIIEKEKQ
SFENMKNHLQNKVEEYEHKVNQLENQISYLKNNHENESEKKDSIMNEVSRLSNESQQLQKVIADLECKQS
MLKEECVDLKEKLNRASLEKDVFLQENTHLQELLKSAQDERDDLENDVNKLKMDEVQLKDALVKLQTLNE
ALGEDKLNLGKIVLQVEAERDQIYKEKENLESEKLGLKEELLRIDKEKASLESERKGILEAINLNDATIE
KLEEQLQEACFSKNKMNDTLNNVVREKEVSQYELVSLRLEVERLQNSLNRLLKEKEELTKENSLISVKLS
GNLKEIQALTESNALLKSEKETLESYLYELQHLVGKLEVRKEQLETSNHEVHLAKDTIAVELAHLKKENE
IQTAKLNKENELLQQKLSLLERERDLSLSRLKEQHEDQIHQLAMDKDKMLTAKKEEHLIVINELKEEMED
YQIKAERDRLALINEVASIQRERDDLLLQAEAEKQAHLRAADSEKSLLVEKITKLKDDLDSANEELEILK
RDLEERAAREKDLLNDSAHEIEKCKENLEETVNLYEAKLKDLVDSHDKLLKNKEELYGELSNAKFQLKIL
NENHEAAQKECKELTNKVIDLNGKLDDASKHTLELKRSLKDAEVEKMLVLSGAEELREAFKQVESEKMTY
KKNYEDSKKLASILDDTNVMQTRETDDLRHMISELETARQETRKEVQQLQNRVRLLDTECVKCKKDLNIA
KDQLNKTNSLLEEERLDLINTKNKLRECETSKDAIKAEMQALATKAREHNDEFGLREKDLLMALEDARQT
EVKVYDKMRQLENLLDVSNQEKSDLKLKLSSVEGRNLGLEDQLTKLEALKNDLDSKLSNVYSALRRTLGI
KPNQSINSINLLVHLRPSQMSLRNVAHLTLGNRVYLQSSHDQSSEFDETPPSKNVSFETSNEMDPEYVRN
SLREFAQKFKDLEHERDEALINIGGLQRQLENLEMDRLDYENRLSVLQKSLQDTAEDKRGADGRLSSAQT

ALLLQEETIRRLERERKTAEEKFQILESTVAQLETEKRTLKEKYMKAHKHKLKYEQEAEAMRSQIDGAES
RITRTDLKKKSLEGDIDRLRSQLHDAENEKHATKQRLEILQRTCKDLELKSSSLQLTVDRLNMSLSKSTD
QEHLLQSKVTELSAIISEKQSVVVSLQDKLQQTERIIVSNEQEKQAAFEKVENLKKQLLENKKLNDLQND
KILNLRNEIANLESTRINLESELRQVQTLLQKNKVGDEELLNKLEKFKEEKKNLQERLAQQQRLLAQGEQ
EKRDIEKNSVKVEKEKRALKATVEQLEKEKFLIDEELIQLKNDQTESDKIRSNYEQTINSLRIQLSQLQE
TLEEKELQHNKKLASMSARHRDDLMKEIERMKSSQQSLDRTYDARERAHKQRIRGLEDQINTLKDQFAKE
IQQKQSFINRTTQKNEEIQEIRQKINSSINEVARNADPRILDRESQKLDTTYDNYNDFSAVLTASTPYSN
RMKVSPFIIDKYSSMTPTTDPGLMSSRIDLPITTEARNLSNTTFVSPIRKNLGKK

>Amphimedon queenslandica_(XP_003384298)
MEEQETKAPPEEATKDTDSASGDGESKDVRVESTFEELEKEFEEVLQEMVGDGSLGKFKTEYEKLHKALI
KSHESEKRLMLQCQELNAELISNASKVTSALKITQDDKATISSQKQEIEKLWRAVDDAQDKETKTHETIH
QLKLENISLNKLIENGAGFGPAMQESMKALEKEKEELTVERDEQMEEISALRTELSELMEKRKEAENERE
SVELKVLQLQEEITAKNQEIDRDSRRKAKLEKDLRGVQAEVDNKVEEIKAKQAQLNKAHEDYAKAEQVLK
DTQKKYENSVKQNEALEVMLDKKKKEYDQEVNRNEELARQNSEQIMELKMKEESINEFKQEIMKINKIRE
GLQKKLREIEERKANVEANRDSLMQQIASIEKELEQERRQAESERRSFDELLRERDIISKSLRKSEGSAE
RLEHLMKMKEQTIKTLQHEITNFKEEAKKQRSLLLQLEKERDKYGKESTDATQKCLQQMEEVKRKELEML
QQKKLIAEAQNKLQQQKTLYEAASSDRSLYSKQLIEARDEITDMKRRIKILSHQIDQLKEEIAAKDGAMV
KVNADLMHLEREKDNLSAEISGRKQELEAARQLLENQKNEEKKLRKVLTEASTESARQKKELEQVINERD
ILGTQVIRRNDELSLLYEKIKIQQSTLDKGEIQYKSRVEDIRILKLEIKKLRREKGLLTKSVANIEDLKR
ELCQCQRELLRERTRCKAIEEELENPINIHRWRKLEASDLGTYEMIQKIHTLQKKLIQKTEEVVEKELLI
QEKEKLYMDLKQILQRQPGPEVAEQLQIYQQTLKEKTKQMKSMASELNMYETQVAEHKMDIDRLERELQE
VKKKYYLQKKKEHIQKERERMLTKGLAPPVIPKATTNMPRFTGGGFNLKPNVLA

Discs, large Drosophila Homolog-associated Protein-DLGAP

>Homo sapiens_DLGAP1(ENSP_316377)
MKGLSGSRSHHHGVTCDSACDSLSHHSDRKPYLLSPVEHHPADHPYYTQRNSFQAECVGP
FSDPLASSTFPRRHYTSQQELKDECALVPRTLATKANRIPANLLDQFERQLPLSRDGYHT
LQYKRTAVEHRSDSPGRIRHLVHSVQKLFTKSHSLEGPSKGSVNGGKASPDEAQAARYGK
RSKSKERRAEPKARPSTSPGWWSSDDNLDGDMCIYHAPSGVMTMGRCPDRSASQYFLEAY
NTISEQAVKASRSNNDVKCSTCANLPVSLDTPLLKKSAWSSTLTVSRAREVYQKASVNMD
QAMVKSESCQQERSCQYLQVPQDEWTGYTPRGKDDEIPCRRMRSGSYIKAMGDEDSGDSD
TSPKPSPKVAARRESYLKATQPSLTELTTLKISNEHSPKLQIRSHSYLRAVSEVSINRSL
DSLDPAGLLTSPKFRSRNESYMRAMSTISQVSEMEVNGQFESVCESVFSELESQAVEALD
LPMPGCFRMRSHSYVRAIEKGCSQDDECVSLRSSSPPRTTTTVRTIQSSTVSSCITTYKK
TPPPVPPRTTTKPFISITAQSSTESAQDAYMDGQGQRGDIISQSGLSNSTESLDSMKALT
AAIEAANAQIHGPASQHMGNNTATVTTTTTIATVTTEDRKKDHFKKNRCLSIGIQVDDAE
EPDKTGENKAPSKFQSVGVQVEEEKCFRRFTRSNSVTTAVQADLDFHDNLENSLESIEDN
SCPGPMARQFSRDASTSTVSIQGSGNHYHACAADDDFDTDFDPSILPPPDPWIDSITEDP
LEAVQRSVCHRDGHWFLKLLQAERDRMEGWCQQMEREEREENNLPEDILGKIRTAVGSAQL
LMAQKFYQFRELCEENLNPNAHPRPTSQDLAGFWDMLQLSIENISMKFDELHQLKANNWK
QMDPLDKKERRAPPPVPKKPAKGPAPLIRERSLESSQRQEARKRLMAAKRAASVRQNSAT
ESAESIEIYIPEAQTRL

>Pan troglodytes_DLGAP1(ENSPTRP_16750)
MKGLSGSRSHHHGVTCDSACDSLSHHSDRKPYLLSPVEHHPADHPYYTQRNSFQAECVGP
FSDPLASSTFPRRHYTSQQELKDECALVPRTLATKANRIPANLLDQFERQLPLSRDGYHT
LQYKRTAVEHRSDSPGRIRHLVHSVQKLFTKSHSLEGPSKGSVNGGKASPDEAQAARYGK
RSKSKERRAEPKARPSTSPGWWSSDDNLDGDMCIYHAPSGVMTMGRCPDRSASQYFMEAY
NTISEQAVKASRSNNDVKCSTCANLPVSLDTPLLKKSAWSSTLTVSRAREVYQKASVNMD
QAMVKSESCQQERSCQYLQVPQDEWTGYTPRGKDDEIPCRRMRSGSYIKAMGDEDSGDSD
TSPKPSPKVAARRESYLKATQPSLTELTTLKISNEHSPKLQIRSHSYLRAVSEVSINRSL
DSLDPAGLLTSPKFRSRNESYMRAMSTISQVSEMEVNGQFESVCESVFSELESQAVEALD
LPMPGCFRMRSHSYVRAIEKGCSQDDECVSLRSSSPPRTTTTVRTIQSSTVSSCITTYKK
TPPPVPPRTTTKPFISITAQSSTESAQDAYMDGQGQRGDIISQSGLSNSTESLDSMKALT
AAIEAANAQIHGPASQHMGNNTATVTTTTTIATVTTEDRKKDHFKKNRCLSIGIQVDDAE
EPDKTGENKAPSKFQSVGVQVEEEKCFRRFTRSNSVTTAVQADLDFHDNLENSLESIEDN
SCPGPMARQFSRDASTSTVSIQGSGNHYHACAADDDFDTDFDPSILPPPDPWIDSITEDP
LEAVQRSVCHRDGHWFLKLLQAERDRMEGWCQQMEREEREENNLPEDILGKIRTAVGSAQL
LMAQKFYQFRELCEENLNPNAHPRPTSQDLAGFWDMLQLSIENISMKFDELHQLKANNWK

QMDPLDKKERRAPPPVPKKPAKGPAPLIRERSLESSQRQEARKRLMAAKRAASVRQNSAT
ESAESIEIYIPEAQTRL

>Gorilla gorilla_DLGAP1(ENSGGOP_28607)
MKGLSGSRSHHHGVTCDSACDSLSHHSDRKPYLLSPVEHHPADHPYYTQRNSFQAECVGP
FSDPLASSTFPRRHYTSQQELKDECALVPRTLATKANRIPANLLDQFERQLPLSRDGYHT
LQYKRTAVEHRSDSPGRIRHLVHSVQKLFTKSHSLEGPSKGSVNGGKASPDEAQAARYGK
RSKSKERRAEPKARPSTSPGWWSSDDNLDGDMCIYHAPSGVMTMGRCPDRSASQYFMEAY
NTISEQAVKASRSNNDVKCSTCANLPVSLDTPLLKKSAWSSTLTVSRAREVYQKASVNMD
QAMVKSESCQQERSCQYLQVPQDEWTGYTPRGKDDEIPCRRMRSGSYIKAMGDEDSGDSD
TSPKPSPKVAARRESYLKATQPSLTELTTLKISNEHSPKLQIRSHSYLRAVSEVSINRSL
DSLDPAGLLTSPKFRSRNESYMRAMSTISQVSEMEVNGQFESVCESVFSELESQAVEALD
LPMPGCFRMRSHSYVRAIEKGCSQDDECVSLRSSSPPRTTTTVRTIQSSTVSSCITTYKK
TPPPVPPRTTTKPFISITAQSSTESAQDAYMDGQGQRGDIISQSGLSNSTESLDSMKALT
AAIEAANAQIHGPASQHMGNNTATVTTTTTIATVTTEDRKKDHFKKNRCLSIGIQVDDAE
EPDKTGENKAPSKFQSVGVQVEEEKCFRRFTRSNSVTTAVQADLDFHDNLENSLESIEDN
SCPGPMARQFSRDASTSTVSIQGSGNHYHACAADDDFDTDFDPSILPPPDPWIDSITEDP
LEAVQRSVCHRDGHWFLKLLQAERDRMEGWCQQMEREERENNLPEDILGKIRTAVGSAQL
LMAQKFYQFRELCEENLNPNAHPRPTSQDLAGFWDMLQLSIENISMKFDELHQLKANNWK
QMDPLDKKERRAPPPVPKKPAKGPAPLIRERSLESSQRQEARKRLMAAKRAASVRQNSAT
ESAESIEIYIPEAQTRL

>Mus musculus_DLGAP1(ENSMUSP_52858)
MKGLSGSRSHHHGITCEAACDSLSHHSDHKPYLLSPVDHHPADHPYYTQRNSFQAECVGP
FSDPLASSTFPRRHYTSQQELKDESALVPRTLATKANRLPTNLLDQFERQLPLSRDGYHT
LQYKRTAVEHRSDSPGRIRHLVHSVQKLFTKSHSLEGPSKGSVNGGKASPDESQTLRYGK
RSKSKERRSESKARSNASNASPTSPSWWSSDDNLDGDMCLYHTPSGVMTMGRCPDRSASQ
YFMEAYNTISEQAVKASRSNNDIKCSTCANLPVTLDAPLLKKSAWSSTLTVSRAREVYQK
ASVNMDQAMVKSEACQQERSCQYLQVPQDEWSGYTPRGKDDEIPCRRMRSGSYIKAMGDE
DSGDSDTSPKPSPKVAARRESYLKATQPSLTELTTLKISNEHSPKLQIRSHSYLRAVSEV
SINRSLDSLDPAGLLTSPKFRSRNESYMRAMSTISQVSEMEVNGQFESVCESVFSELESQ
AVEALDLPLPGCFRMRSHSYVRAIEKGCSQDDECVSLRSSSPPRTTTTVRTIQSSTVSSC
ITTYKKTPPPVPPRTTTKPFISITAQSSTESAQDAYMDGQGQRGDMISQSGLSNSTESLD
SMKALTAAIEAANAQIHGPASQHMGSNAAAVTTTTTIATVTTEDRKKDFKKNRCLSIGIQ
VDDAEEPEKMAESKTSNKFQSVGVQVEEEKCFRRFTRSNSVTTAVQADLDFHDNLENSLE
SIEDNSCPGPMARQFSRDASTSTVSIQGSGNHYHACAADDDFDTDFDPSILPPPDPWIDS
ITEDPLEAVQRSVCHRDGHWFLKLLQAERDRMEGWCKLMEREERENNLPEDILGKIRTAV
GSAQLLMAQKFYQFRELCEENLNPNAHPRPTSQDLAGFWDMLQLSIENISMKFDELHQLK
ANNWKQMDPLDKKERRAPPPVPKKPAKGPAPLIRERSLESSQRQEARKRLMAAKRAASVR
QNSATESAESIEIYIPEAQTRL

>Rattus norvegicus_DLGAP1(ENSRNOP_73938)
MKGLSGSRSHHHGITCESACDSLSHHSDHKPYLLSPVDHHPADHPYYTQRNSFQAECVGP
FSDPLASSTFPRRHYTSQQELKDESALVPRTLATKANRLPTNLLDQFERQLPLSRDGYHT
LQYKRTAVEHRSDSPGRIRHLVHSVQKLFTKSHSLEGPSKGGVNGGKASPDGSQTVRYGK
RSKSKERRSEPKARSNASNASPTSPSWWSSDDNLDGDMCLYHTPSGVMTMGRCPDRSASQ
YFMEAYNTISEQAVKASRSNNDVKCSTCANLPVTLDAPLLKKSAWSSTLTVSRAREVYQK
ASVNMDQAVVKSEACQQERSCQYLQVPQDEWTGYTPRGKDDEIPCRRMRSGSYIKAMGDE
DSGDSDTSPKPSPKVAARRESYLKATQPSLTELTTLKISNEHSPKLQIRSHSYLRAVSEV
SINRSLDSLDPAGLLTSPKFRSRNESYMRAMSTISQVSEMEVNGQFESVCESVFSELESQ
AVEALDLPMPGCFRMRSHSYVRAIEKGCSQDDECVSLRSSSPPRTTTTVRTIQSSTGVIK
LSSAVEVSSCITTYKKTPPPVPPRTTTKPFISITAQSSTESAQDAYMDGQGQRGDMISQS
GLSNSTESLDSMKALTAAIEAANAQIHGPASQHMGSNAAAVTTTTTIATVTTEDRKKDFK
KNRCLSIGIQVDDAEESEKMAESKTSSKFQSVGVQVEEEKCFRRFTRSNSVTTAVQADLD
FHDNLENSLESIEDNSCPGPMARQFSRDASTSTVSIQGSGNHYHACAADDDFDTDFDPSI
LPPPDPWIDSITEDPLEAVQRSVCHRDGHWFLKLLQAERDRMEGWCKQMEREERENNLPE
DILGKIRTAVGSAQLLMAQKFYQFRELCEENLNPNAHPRPTSQDLAGFWDMLQLSIENIS
MKFDELHQLKANNWKQMDPLDKKERRAPPPVPKKPAKGPAPLIRERSLESSQRQEARKRL
MAAKRAASVRQNSATESAESIEIYIPEAQTRL

>Myotis lucifugus_DLGAP1(ENSMLUP_11070)

MKGLSSSRSHHHGVTCDSTCDSHHSDRKPYLLSPVEHHPADHPYYTQRNSFQAECVGPFS
DPLASSTFPRRHYNSQQELKDECALVPRTLATKANRLPPNLLDQFERQLPLSRDGYHTLQ
YKRTALEHRSDSPGRIRHLVHSVQKLFTKSHSLEGPSKGSVNGGKASPDETQTVRYGKRS
KSKERRPEPKARPNTSPNTSPGWWSSDDNLDSDMCIYHSPSGVMTMGRCPDRSASQYFME
TYNTISEQAVKASRSNNDVKCSTCAHLPVNLDAPLLKKSAWSSTLTVSRAREVYQKASVN
VDQAMVKSESCQQERSCQYLQVPQDEWTGYTPRGKDDEIPCRRMRSGSYIKAMGDEDSGD
SDTSPKPSPKVAARRESYLKATQPSLTELTTLKISNEHSPKLQIRSHSYLRAVSEVSINR
SLDSLDPAGLLTSPKFRSRNESYMRAMSTISQVSEMEVNGQFESVCESVFSELESQAVEA
LDLPVPGCFRMRSHSYVRAIEKGCSQDDECVSLRSSSPPRTTTTVRTIQSSTGVIKLSSA
VEVSSCITTYKKTPPPVPPRTTTKPFISITAQSSTESAQDAYMDGQGQRGDIISQSGLSN
STESLDSMKALTAAIEAANAQIHGPASQHMGNNPATITTTTTIATITTEDRKKDHFKKNR
CLSIGIQVDDAEEPDKTGENKAPSKFQSVGVQVEEEKCFRRFTRSNSVTTAVQADLDFHD
NLENSLESIEDNSCPGPAARQFSRDASTSTVSIQGSGNHYHACAADDDFDADFDPSILPP
PDPWIDSITEDPMEAVQRSVCHRDGHWFLKLLQAERDRMEGWCQQMEREERENNLPEDIL
GKIRTAVGSAQLLMAQKFYQFRELCEENLNPNAHPRPTSQDLAGFWDMLQLSIENISMKF
DELHQLKANNWKQMDPLDKKERRAPPPVPKKPAKGPAPLIRERSLESSQRQEARKRLMAA
RRRRVTRATESAESIEIYIPEAQTRL

>Monodelphis domestica_DLGAP1(ENSMODP_35344)
MKGLSSSRSHHHGVTCDSACDSLSHHSDRKPYLLSPVEPHPADHPYYTQRNSFQAECVVP
FNDPLASSTFPRRHYNSQQELKEECALVPRTLANKANRLPPNLLDQFERQLPLNRDGYHT
LQYKRTAVEHRSDSPGRIRHLVHSVQKLFTKSHSLEGPSKGSINGGKASPDDTQTMRYGK
RSKSKERRPEPKARSNASGWWSSDDNLDGDVCLYHAPSAVMTMGRCPDRSASQYFMEAYN
TINEHAVKASRSNNDVKCSTCANLPVTLDAPLLKKSAWSSTLTVSRAREVYQKASVNVDQ
ALVKAETCQQERSCQYLQVPQDEWSGYTPRGGKDDEIPCRRMRSGSYIKAMGDEDSGDSD
TSPKPSPKIAARRESYLKATQPSLTELTTLKISNEHSPKLQIRSHSYLRAVSEVSINRSL
DSLDPAGLLTSPKFRSRNESYMRAMSTISQVSEMEVNGQFESVCESVFSELESQAVEALD
LPMPGCFRMRSHSYVRAIEKGCSQDDECVSLRSSSPPRTTTTVRTIQSSTGIIKLSSAVE
VSSCITTYKKTPPPVPPRTTTKPFISITAQSSTESAQDAYMDGQGQRGDIISQSGLSNST
ESLDSMKALTAAIEAANAQIHGPASQHMGSNTATASTTTTTTATVTAEERKKDHFKKNRC
LSIGIQVDDVDEPVKAGENKVTSKFQSIGVQVEEEKCFRRFTRSNSVTAAVQADLDFHEN
LENSLESIEDNTCPGPMSRQFSRDASTSTAERDRMEGWCQQMEREERENNLPEDILGKIR
TAVGSAQLLMAQKFYQFRELCEENLNPNAHPRPTSQDLAGFWDMLQLSIENISMKFDELH
QLKANNWKQMDPLDKKERRVPPPVPKKPAKGHVPLIRERSLESSQRQEARKRLMAAKRAA
SVRQNSATESAESIEIYIPEAQTRL

>Gallus gallus_DLGAP1(ENSGALP_46967)
MKGLSSSRSHHHGVTCDPPCDSLSHHSDRKPYLLNPVDPHPADHPYYTQRNSFQAECMVP
YNDQIASSTFPRRHYNSHHELKDECALVPHGTANKTNRIPANLLDQFERQLPLNRDGYHT
LQYKRTAMEHRSDSPGRIRHLVHSVQKLFTKSHSLEGPSKGSVNGSKASPEESQTMRYGK
RSKSKERRSEGKPRSNASGWWSSDDNLDNDVCIYHGPSGVMTMGRCPDRSSSQYFMEAYN
TISEHTVKSSRSNNDVKCSTCANLPVNLDSQLMKKSSWSSTLTVSRAREVYQKASVNMDQ
TVVKAETCQQERSCQYLQVPQDEWSGYTPRGKDDEIPCRRMRSGSYIKAMGDDDSGDSDT
SPKPSPKIAARRESYLKATQPSLTELTTLKISSEHSPKLQIRSHSYLRAVSEVSINRSLD
SLDPAGLLTSPKFRSRNESYMRAMSTISQVSEMEVNGQFESVCESVFSELESQAVEALDL
PMPGCFRMRSHSYVRAIEKGCSQDDECISLRSSSPPRTTTTVRTIQSSTVSSCITTYKKT
PPPVPPRTTTKPFISITAQSSTESAQDAYMDGHGQRGDIISQSGLSNSTESLDSMKALTA
AIEAANAQIHGPASQHVGNNTATVTTTTTIATVAVEDRKKDHFKKNRCLSIGIQVDGAEE
TAKPGENKATSKFQSIGVQVEEEKCFRRFTRSNSVTTAVQADLDFHENFEIIDPQEDNLC
SGQISRQFSRDASTSTVSIQGSGNHYHACAVDDDFETDFDPSILPPPDPWIDSITEDPLE
AVQRSVCPRDGHWFLKLLQAERDRMEGWCQQMEREERENNLPEDILGKIRTAVGSAQLLM
AQKFYQFRELCEENLNPNAHPRPTSQDLAGFWDMLQLSIENISMKFDELHQLKANNWKQM
DPHDKKERRVPPPVPKKPSKGQVPLIRERSLESSQRQEARKRLMAAKRAASVRQNSATES
AESIEIYIPEAQTRL

>Anolis carolinensis_DLGAP1(ENSACAP_21368)
MMKGLSGSRSHHHGVVYEPPCDSLVHHSERKPYLLNPVDHHPSDHSYYTQRNSFQGECMV
PYNDQIASSTFPRRHYNSHNELKDECALVPHGSAGKGNRIPANLLDQFERQLPLNRDGYH
TLQYKRTAVEHRSDSPGRIRHLVHSVQKLFTKSHSLEGPSKGSINGGKASPEETQSMRYG
KRSKSKDRRSEGKSRSNASGWWSSDDNLDNDVCLYHGPSGVMTMGRCPDRSASQYFMEAY
NTLSEHAVKPSRSNNDVKCSTCANLPVTLDAPLMKKSSWSSTLTVSRAREVYQKASMNMD

QTMMKSETCQQERSCQYLQVPQDEWSGYTPRGKDDEIPCRRMRSGSYIKAMGDDDSGDSD
TSPKLSPKIAARRESYLKATQPSLTELTTLKISSEHSPKLQIRSHSYLRAVSEVSINRSL
DSLDPAGLLTSPKFRSRNESYMRAMSTISQVSEMEVNGQFESVCESVFSELESQAVEALD
LPMPGCFRMRSHSYVRAIEKGCSQDDECLSLRSSSPPRTTTTVRTIQSSTDLLDPVSHYD
CKMILTNHRPQNKDASRRKLVSSCITTYKKTPPPVPPRTTTKPFISITAQSSTESAQDAY
MDGHGQRGDIISQSGLSNSTESLDSMKALTAAIEAANAQIHGPASQHIGNNTTTVTTTTT
VATVTADDRKRDHYKKNRCLSIGIQVDGEDSSSSSETRTPSKFQSIGVQVEEEKCFRRFT
RSNSVTTAVQADMDFHENFEMSMDPQEDLSYPGQMSRQYSRDASTSTVSIQGSGNHYHAC
TVDDDFDTEFDPSILPPPDPWIDSIIEDPIEAVQRSVCPRDGHWFLKLLQAERERMEGWC
QQMEREERETHLPDEILGKIRTAVGSAQLLMAQKFYQFRELCEENLNPNAHPRPASQDLA
GFWDMLQLSIENISMKFDELHQLKANNWKQMDPHDKKERRAPPPVPKKPSKGQVPLIRER
SLESSQRQEARKRLMAAKRAASVRQNSATESAESIEIYIPEAQTRL

>Xenopus tropicalis_DLGAP1(ENSXETP_35507)
MKGLSSSRSHHHGVVCDSVCETLPHHTDRKPYLLNPVDPHPADHPYYAQRNSYPVDHMIS
YGDQIASSTFPRRHYSSHHDIKDDCALVPHGSVGKGNRLPSSLLDQFERQIPINRDGYHT
LQYKRSAVEHRSDSPGRIRHLVHSVQRLFTKSHSLEGPMKGNANGNKANSDEMQTVRYGK
RSKSKERRAEVKPRANIQGWWGSQDNLDNDVCIYHGPSTVMTMGRCPDRSTSQYFMEAYN
TINDHALKSSRSNNDVKCNTCTNLPVSLDSQMLKKSSWASSLTVSRAREVYQKPAINMDQ
ALVKSESMTQPQERPCQYLQVPQDEWSGYSPKSKDDEIPCRRMRSGSYIKAMGDDDSGDS
DTNSPKPSPKIAARRESYLKATQPSLTELTTLKISNEHSPKLQIRSHSYLRAVSEVSINR
SMDSLDPAALLTSPKFRSRNESYMRAMSTISQVSEMEVNGQFESVCESVFSELESQAVEA
LDLPMPGCFRMRSHSYVRAIEKGCSQDDDSLLLRPSSPPRTATTVRTIQSSTVSSCITTY
KKTPPPVPPRTTTKPFISITAQSSTESAQDAYMDGHGQRGDMISHSGLSNSTESLDSMKA
LTAAIEAANAQIHGPASQHMDNTTTVNSTATTTAIINEDDRRKNHFRKNLSIGIQVDGPE
EAARAEERQAAFRYQSVGIQVEEERALRRLTRSNSVTAAVQADLDLREHFECPVELADQE
FQFSRPLAKQYSRDACTSTVSIQGSGNHYHACAVEEDFDADFDPSILPPPDPWIDSITED
PMEAVHRSVCHRDGHWFLKLLQAEKDRMEGWCQQMKREEHENDLPDPILDKIRSAVGCAQ
LLMSQKFYQFRELCEENLNPNAHPRPTSQDLAGFWDMLQLSIENISMKFDELHQLKANNW
KKMDSLDKKDKRAPPPVPKKPSKGQAPLVRDRSLESSERQRHEARKRLMSAKRAASVRQN
SATESADSIEIYIPEAQTRL

>Danio rerio_DLGAP1(ENSDARP_113100)
MKGLSGSRSHHHVVTCDPSFESMGHHLDHKPYLISQIDNPHPVDHSYYSQRSPYQPDCVV
PYGTFPRRHYSSQHELKDEMAVVPYSGGGVPASKGQQRLPVALLDQFDRQLPVPRDGYHT
LQYKRAAAALEQRSDSPGRIRHLVHSVQKLFTKSHSLEGPHHGHGHGKGSINGGKASPDG
EPPAIRHRKRSKSRERCKSAEPKNRTPPSGYWSSDELEREACLFHHGPPGVMTMGRHPDK
SQSQYFLEAYNTINDQALKNSRSNNDLGKCSTCTSIPLSVDASQLVKKSSWSSSLTVSRA
RQVYQKASVNVDKALVKAEACQQERSCQFLQLWKCVFVELSVCLDADMHVWAKVCDVASL
LNRQVPQDEWGGFPLGKDEDIPCRRMRSGSYVKAMAEDDSGDSDGSPKPSPKMQARRASY
LKATQPSLTEMTTLKISQEHSPKLQIRSHSYLRAVSEVSINRSLDTLDPKALLASPQYRS
RNESYMRAMSTISQIEAMCESVFNEMESHAVDALDLPMPGCFRMRSHSYVRAIDQGCSQD
DDSPLLPASPPRTTTTVRTIQSSTVSSCITTYKKTPPPVPPRTSTKPFISITAQSSTESA
QDAYMDGPGTRGEPVLHSGLSNSTESIDSMKALTAAIEAANAQIHGPASQHVSNSTMTIS
TAPPSPLPLPVPVPVPIPIPALEDVRRDVLRKSRCLSIGIQSKFQSVGVQVEEEHGVTTA
VQADLDTPDYTPADSPVTDFPAAGCLSRQYSRDAATSTVSIQGSGNHYHACTSDDYEDVG
FDPSILPPPDPWIDSVIEDSLEVVGRSVCQRDGRWFLKLLQAETERMEGWCRQMEHDEKE
NKMPDEVLGKIRGAVGSAQLLMSQKFQQFRELCEENLNPNAHPRPVAQDLAGFWDMLQLS
IENISLKFDELHQLKANNWRPLDPPERKERRLPPPVPKKPPKAHPPLARDRSLESTEKQR
QEARKRLMAAKRAASVRQNSATESADSIEIYIPEAQTR

>Takifugu rubripes_DLGAP1(ENSTRUP_45775)
MKGLSSSRSHHHGVTCDPSYDSMTLGHPDRRPYFLNPGEAHPADHPFYTQHNSCPSESST
YPDLASCTFPRRHFSSHHELKEECGALVPYTGPTGSRSNNRVSSAVLEQFDRQAPVRREG
YHTLQYKRTAVEHQRSDSPGRIRHLVHSVQKLFTKSHSLEGPSGSGGGGSGRVNGCKSSP
DDPPSTSGTLKHSKRSKSKDRSKSEPKMRTAISGYWSSDDTLDRDACLYHHHHGGRSGGL
PSGVMTMGRHPDKSQSQYFMESYNTISAHSLKTSRSNNDVKCTTCSAGSSGSIPLAAGSM
DGQVQLKKSSWSSTLTVSRAREVYQKASVNLDKALVKAEQGRTCHFLQVPQDDWSGFSPL
GKDDEIPCRRMRSGSYIKAMAEEDSGDSDGSPKLSPKVQARRASYLKATQPSLTEMTTLK
ISTEHSPKLQIRSHSYLRAVSEVSINRSLDSLDPAGLLASPKFRSRNESYMRAMSTISQV
SEVEVNGQIEAVCESVFSEMESQAVDALDLPVPSCFRMRSHSYVRAIEKGCSQEEEEAGG

GAKVGNRRTGSPPRTTTTVRTIQSSTAIMKLKIFSEVSSCITTYKKTPPPVPPRTTPSKP
FISITAQSSTESAQDAYMDGGSGPRAGISSQPGLSNSTESIDSMKALTAAIEAANAQVHG
PPSQHVTNSSITITATATTSAVAHISADSKAQREALRKCLSIGIQVDPEEGGPTEEQSKF
QSIGIQVEDERCYRRMTRSNSVTAAVQADLDDGTDPPELPPRHFATMPRQHSRDAATSTV
SIQGSGNHYHACASDDYGDAGFDPSILPPPDPWMDGAGEAEAETPEAIARSVCPRDGRWF
LKLLQAEAERMEGWCRQMEQDERENELPDEILGKIRSAVGSAQLLMSQKFQQFRELCEEN
LNPNAHPRPISQDLAGFWDMLQLSIENISLKFDELHQLKANHWKPLTPPEIQERRMPPPV
PKKPLKGHPPLARDRSLESSERQRLEARKRLLAAKRAASVRQSSATESADSIEIYIPEAQ
TRL

>Tetraodon nigroviridis_DLGAP1a(ENSTNIP_15747)
MKSLSSSRSHHHGVTCDPSYDSMTLGHGDRRPYFLNPGEGHHTDHPCYTQHNSCQSESST
YPDLASCTFPRRHYSSHHELKEECGALVPYTGPTGSSKGDGGGVGSNNRISSTVLEQFDR
QAPVRREGYHTLQYKRTAVEHQRSDSPGRIRHLVHSVQKLFTKSHSLEGPSGSGGGGSGR
VNGCKSSPDDPPSSSGTLKHTKRSKSKDRSKSEPKMRTAISGYWSSDDTLDRDACLYHHH
HGARSGGLPSGVMTMGRHPDKSQSQYFMESYNTISAHSLKTSRSNNDVKCTTCSVGSGGS
IPLGAGSMDGQVQLKKSSWSSTLTVSRAREVYQKASVNLDKALVKAEQGRTCHFLQVPQD
DWSGFSPLGKDDEIPCRRMRSGSYIKAMAEEDSGDSDGSPKPSPKVQARRASYLKATQPS
LTEMTTLNRSLDSLDPAGLLASPKFRSRNESYMRAMSTISQKACFGSKYSKKIVSRDTSL
FGFGCKVSEVEVNGQIEAVCESVFSEMESQAVDALDLPVPSCFRMRSHSYVRAIEKGCSQ
EEEEAEDQSCRPPFTPSTGCIGSAVFLAVSSCITTYKKTPPPVPPRTTPSKPFISITAQS
STESAQDAYMDGGSGPRAGISSQTGLSNSTESIDSMKALTAAIEAANAQVHGPPSQHVTN
SSITITATATATASAVAHISADSKAQREALRKCLSIGIQVDPEEGGPTEEQSKFQSIGIQ
VEDERCYRRMTRSNSVTTAVQADLDDAADPPELPPRHFATMPRQHSRDAATSTVSIQGSG
NHYHACTSDDIADVDFDPSILPPPDPWMDGAGEAEAETSEAIGRLVCPRDGRWFLKLLQA
ETERMEGWCRRMEQDETENELPDEILGKIRSAVGSAQLLMSQKFQQFRELCEENLNPNAH
PRPVSQDLAGFWDMLQLSIENISLKFDELHQLKANNWKPLTPPEIQERRMPPPVPKKPLK
GHPPLARDRSLESSERQRLEARKRLLAAKRAVSVRQSSATESADSIEIYIPEAQTRL

>Tetraodon nigroviridis_DLGAP1b(ENSTNIP_8779)
MKGLSSSRSHHHVISYEPSYDPLGHHVDRKPYLISQNDIPVQIPVPHPAELPYYNAQRTS
YPPDSNSNIPYGTFPRRCHSTSSSHHPEVKDECMAIVPYDGGKTPTRVSATFADPLERQS
SFSRDGYHTLQYKRGMLAHSGGMVANNNDSPGRIRHLVHSVQKLFTKSHSLEGPHHTQCS
KGASNGSRASIEGETPPTGPSHRKRSKSRERCRSAEPKHRSHHHRHHQLHGSASAPGSGY
WSSDDNLERELCLYHPHHHQPPPSPSISPSPIAMTMGRYTSNISHQYFMMDPYTTLNEPA
LKASRSNNDVKYAVSSACVPPLGLVDGPLMKKGACSSSLTVSRARELYTNNSSHNQARAG
AGSGNLNVDRALVKSKGSQQQERSCHFLQVPQDDWSNFSPLGKEDDIPCRRMRSGSYVKA
MAEDDSGDSDGSPKPSPKIQARRASYLKATQPSLTEMTTLHIWPYHNKMLSSKSVYKKPG
TKVEMVKFFPMLPDRFVLKLLCFFCVSLCLFSFPCSSVQQLSEVGGKRAGLSRSTVRCNL
RPWGRSMDTMDTMDTLPMPGCFRMRSHSYVRAIDQGCAGKGRPLLLLPSSPPKTSATTVR
TIQSSTVSSCITTYKKTPPPVPPRTSTCSSKPYISITAQSSTESAQDAYMEEEGPRGDMT
LQSRLSNSTESIDSMKALTAAIEAANAQVHGPASQHVSSSTMTVSPPKPPVLCRRPVVED
YQDDYRKEAFRKGKCLSIGIQVDGPEEIPDPEDSSKFTSVGVQVEDHRGYRRCQRSNSVT
AAVQADLDIPDLLDTPLDSPVTDMEILPSGVLSRQYSRDAATSTVSIQGSGNHYHACASD
DYEDVGFDPSILPPPDPWIDSVTDDPMDVNVVQRSVCQRDGRWFLKLLQAETDRMEGWCR
QMELDQRENALPEDILGKMRSAVGSAQLLMSQKFQQFRELCEENLNPNAHPRPVASDLAG
FWDMLQLSIENISLKYDELHQLRANNWRPIDPPERKERPLPPPVAKKPPKGPHHHPPLVR
DRSLESSEKQRQEARKRLMAAKRAASVRQNSATESADSIEIYIPEAQTRL

>Gasterosteus aculeatus_DLGAP1a(ENSGACP_5055)
MKGLSSSRSHHHAVTCDPSYDSMTLGHSDRRSYFLNPGEAHPSDHPCYTQRNSFQAECSA
YPDLASCTFPRRHYSSHHELKGECGAVVPYVGPTGCGKGGGNNNNRVPPNLLEQFERQAP
VRREGYHTLQYKRTAVEHQRSDSPGRIRHLVHSVQKLFTKSHSLGMNGSKSSPDDPPSSS
GTLKHSKRSKSKDRSKSEPKMRGAISGYWSSDDTLDREMCLYHHHRHGGTGGGPPTGVMT
MGRHPEKSQSQYFMESYNTISAHSLKTSRSNNDVKCSTCSGSGSGGGGGVPLAGSQDGQVQ
LKKSSWSSTLTVSRAREVYQKASVNLDKALVKAEQGRTCHFLQKWETASPTFTFISVCEK
SGIHVWEKLQPVYLPSHLQVPQDDWGSFSPLGKDDEIPCRRMRSGSYIKAMAEEDSGDSD
CSPKPSPKVQARRASYLKATQPSLTEMTTLEISTEHSPKLQIRSHSYLRAVSEVSINRSL
DSLDPAGLLASPKFRSRNESYMRAMSTISQIEAVCESVFSEMESQAVDALDLPIPNCFRV
RSHSYVRAIEKGCSQEDEEEAAAAAGVSRTGSPPRTIQSSTVSSCITTYKKTPPPVPPRT

TPSKPFISITAQSSTESAQDAYMDGGSGPRTGISSQPGLSNSTESIDSMKALTAAIEAAN
AQVHGPASQHVTNSTITITATAVAHVPADSKAQREALRKCLSIGIQSKFQSIGIQVEDER
CVTTAVQADLDDPAGHCATMPRQLSRDASSSTVSIQGSGNHYHACAPDDFGEAGFDPSIL
PPPDPWMDSVAEPDSGAPEAVGRSVCPRDGRWFLKLLRAETERMEGWCRQMEQDEVENEL
PDEILGKIRSAVGSAQLLMSQKFQQFRELCEENLNPNAHPRPVSQDLAGFWDMLQLSIEN
ISLKFDELHQLKANNWKPLTPPEIQERRMPPPVPKKPPKGHHPPLARDRSLESSERQRLE
ARKRLLAAKRAASVRQSSATESADSIEIYIPEAQTR

>Gasterosteus aculeatus_DLGAP1b(ENSGACP_19506)
MKGLSGSRSHHHVVSYEPSYDLLGHHVDRKPYLISQNDMPVPIPMPHQAELSYYNAQRTP
YPSESNSIVPYGTFPRRCHSTSSSHHPEVKDECMAMVPYYGGKTPTRVPEPFERQPSFPR
DGYHTLQYKRGVLAHSGGVGAQHNNNDSPGRIRHLVHSVQKLFTKSHSLEGPHHPQNSKG
ANNGSGANPEGEIPPVGVRQRKRSKSRERCRSAEPKHRSHHHPHHQLHGSASAPGSGYWS
SDDNLERELSLYHPHHHQPPPSPSPSISPSPIAMTMGRYPGNSQSQQYFMMDPYGTLNEH
THTHAHTHAHHGHTHHHSALKVSRSNNDVKYAASSACVPSLGLVDGPLVKKGAWSSSLTV
SRAREVYTNNSSHNQPRASAGASNLNLDRALVKSKGSQLQERSCHFLQVWGAIFLYLEWA
IAPSVHIWYRTDCEICTLLSRQVPQDEWSGFSPLGKEDDIPCRRMRSGSYVKAMAEEDSG
DSDGSPKPSPKMQARRASYLKATQPSLTEMTTLQISSEHSPKLQIRSHSYLRAVSEVSIN
RSLDTLDPKTLLDPKSLLSSPQYRSRNESYMRAMSTISQIEQVCEQVYSAMQAQAMEAAI
DSMETMDTLPMPGCFRMRSHSYVRAIDQGCSGDEEGEGGRPLLLLPSSPPRTSATTVRTI
QSSTVSSCITTYKKTPPPVPPRTSTCSTASKPYISITAQSSTESAQDAYMEEEGPRGDMT
IQSGLSNSTESIDSMKALTAAIEAANAQVHGPASQHVSNSAMTVCTPTPSVHSRGAVIED
HRDVYRKEALRKGKCLSIGIQSKFTSIGVQVEDDRGVTTAVQADMDIPDAMDTPLDSPDT
DMEVLSSGALSRQYSRDAATSTVSIQGSGNHYHACASDDYDDVGFDPSILPPPDPWIDSV
TDDSLDVVQRSVCHRDGRWFLKLLQAETDRMEGWCRQMELDQQDNDLPDDVLGKMRSAVG
SAQLLMSQKFQQFRELCEENLNPNAHPRPVASDLAGFWDMLQLSIENISLKFDELHQIRA
NNWRPLDPPERKERRLPPPVPKKPPKAPHHHPPLARDRSLESSEKQRQEARRRLMAAKRA
ASVRQNSATESADSIEIYIPEAQTR

>Oryzias latipes_DLGAP1(ENSORLP_10417)
MKGLSGSKSHHHVVSYEPSYDPLGHHVDRKQLLISQMDIPVPVPMPAEHPYYNAQRTSYP
SDSNSIVPYGTFPRRCHSTSSSHHPEVKDECMAIVPYDGGKMQTQVPVNIVDQYEHQPPF
SRDGYHTLQYKRGVLAHTGGMGTNDNNNDSPGRIRHLVHSVQKLFTKSHSLEGPHHTQSS
KGAHNGSVNGGGGGGGGSRASPEGGNPSAGVRHRKRSKSRERCRSAEPKHRSYHHHHPQL
HGSASAPGSGYWSSDDNLERELCLYHPHHHQPPPSPSPSISPSPIAMTMGRCVDNNPDKF
HARHGHTHHHSALKASRSNNDLKYAASSACLPPVGLLDGPLVKKGAWSSSLTVSRAREVY
TNSSHNQPRASAGSGNLNLDRALVKSKGSQQQDPSCHFLQIWYYQSVQTAVYLPGLFVAW
AKSGSYANLNNFQVPQDEWSGFSPLGKEEDIPCRRMRSGSYVKAMAEDDSGDSEGSPKPS
PKVQARRASYLKATQPSLTEMTTLQISTEHSPKLQIRSHSYLRAVSEVSINRSLDTLDPK
SLLDPKSLLSSPQYRSRNESYMRAMSTISQIEQVCEQVYSEMQSQAMEAAMDSLETMDTL
PMPGCFRMRSHSYVRAIDQGCAGEEEGEGGRPLLLLPSSPPRTSATTVRTIQSSTVSSCI
TTYKKTPPPVPPRTSTCSTASKPYISITAQSSTESAQDAYMEEEGPRGDMTIQSGLSNST
ESIDSMKALTAAIEAANAQVHGPASQHVNNSTMTVSVPNSSALSRGYSIEDHRDEYRKEA
LRKGKCLSIGIQSKFTSVGVQVEDDRGVTTAVQADLDLPDMLDMPMDSPEPNRHILSPNA
LSRQYSRDAATSTVSIQGSGNHYHACASDEYDDVGFDPSILPPPDPWIDNVTDDPLCVAS
RSICQRDGRWFLKLLQAETDRMEGWCQQMELDQQENDLPDEVLGKMRSAMESAQHLMSQK
FQQFRELCEENLNPNIHPRPVASDLAGFWDMLQLSIENISLKFDELHQLRANNWRPLDPP
ERKERRLPPPVPRKPHKGPHQHPPLVRDRSLESSEKQRQEARKRLMAAKRAASVRQNSAT
ESADSIEIYIPEAQTR

>Homo sapiens_DLGAP2(ENSP_400258)
MKGLSGSRTQPPLCSGHTCGLAPPEDCEHLHHGPDARPPYLLSPADSCPGGRHRCSPRSS
VHSECVMMPVVLGDHVSSSTFPRMHYSSHYDTRDDCAVAHAGAKINRIPANLLDQFEKQL
PLHRDGFHTLQYQRTSAAAEQRSESPGRIRHLVHSVQKLFTKSHSLEGSSKSNANGTKAD
GRADDHHHAHHAKHSKRSKSKERKPEGKPRPGMSSWWSSDDNLDSDSTYRTPSVLNRHHL
GPVAHCYPDALQSPFGDLSLKTSKSNNDVKCSACEGLALTPDAKYLKRSSWSTLTVSQAK
EAYRKSSLNLDKPLLHQDAKPALRPCHYLQVPQDEWGGYPTGGKDEEIPCRRMRSGSYIK
AMGDEESGESDSSPKTSPKSAILPEPLLKSIGQRPLGEHQTQTYLQAASDVPVGHSLDPA
ANYNSPKFRSRNQSYMRAVSTLSQASCVSQVSEAEINGQFESVCESVFSEVESQAMDALD
LPGCFRTRSHSYLRAIQAGYSQDDECIPMMTPSDITSTIRSTAAVSYTNYKKTPPPVPPR
TTSKPLISVTAQSSTESTQDAYQDSRAQRMSPWPQDSRGLYNSTDSLDSNKAMNLALETA

AAQRHLPESQSSSVRTSDKAILVSKAEELLKSRCSSIGIQDSEFPEHQPYPRSDVETATD
SDTESRGLREYHSVGVQVEDEKRHGRFKRSNSVTAAVQADLELEGFPGHITTEDKGLQFG
SSFQRHSEPSTPTQYSAVRTVRTQGLFSYREDYRTQVDTSTLPPPDPWLEPAIDTVETGR
MSPCRRDGSWFLKLLHAETKRMEGWCKEMEREAEENDLSEEILGKIRSAVGSAQLLMSQK
FQQFYWLCQQNMDPSAMPRPTSQDLAGYWDMLQLSIEDVSMKFDELQRLRLNDWKMMESP
ERKEERKVPPPIPKKPPKGKFPITREKSLDLPDRQRQEARRRLMAAKRAASFRQNSASER
ADSIEIYIPEAQTRL

>Pan troglodytes_DLGAP2(ENSPTRP_42538)
MKGLSGSRTQPPLCSGHTCGLAPPEDCEHLHHGPDARPPYLLSPADSCPGGRHRCSPRSS
VHSECVMMPVVLGDHVSSSTFPRMHYSSHYDTRDDCAVAHAGAKINRIPANLLDQFEKQL
PLHRDGFHTLQYQRTSAAAEQRSESPGRIRHLVHSVQKLFTKSHSLESSSKSNANGTKAD
GRADDHHHAHHAKHSKRSKSKERKPEGKPRPGMSSWWSSDDNLDSDSTYRTPSLLNRHHL
GPVAHCYPDALQSPFGDLSLKTSKSNNDVKCSACEGLALTPDAKYLKRSSWSTLTVSQAK
EAYRKSSLNLDKPLLHQDAKPALRPCHYLQVPQDEWGGYPTGGKDEEIPCRRMRSGSYIK
AMGDEESGESDSSPKTSPKSAILPEPLLKSIGQRPLGEHQTQTYLQAASDVPVGHSLDPA
ANYNSPKFRSRNQSYMRAVSTLSQASCVSQVSEAEINGQFESVCESVFSEVESQAMDALD
LPGCFRTRSHSYLRAIQAGYSQDDECIPMMTPSDITSTIRSTAAVSYTNYKKTPPPVPPR
TTSKPLISVTAQSSTESTQDAYQDSRAQRMSPWPQDSRGLYNSTDSLDSNKAMNLALETA
AAQRHLPESQSSSVRTSDKAILVSKAEELLKSRCSSIGIQVAALGRPRRSLLNVETATDS
DTESRGLREYHSVGVQVEDEKRHGRFKRSNSVTAAVQADLELEGFPGHITMEDKGLQFGS
SFQRHSEPSTPTQYSAVRTVRTQGLFSYREDYRTPVDTSTLPPPDPWLEPAIDTVETGRM
SPCRRDGSWFLKLLHAETKRMEGWCKEMEREAEENDLSEEILGKIRSAVGSAQLLMSQKF
QQFYWLCQQNMDPSAMPRPTSQDLAGYWDMLQLSIEDVSMKFDELQRLRLNDWKMMESPE
RKEERKVPPPIPKKPPKGKFPITREKSLDLPDRQRQEARRRLMAAKRAASFRQNSATERA
DSIEIYIPEAQTRL

>Gorilla gorilla_DLGAP2(ENSGGOP_7425)
PQYSWSPTQHFNEERYSPAPRSMKGLSGSRTQPPLCSGHTCGLAPPEDCEHLHHGPDARP
PYLLSPADSCPGGRHRCSPRSSVHSECVMMPVVLGDHVSSSTFPRMHYSSHYDTRDDCAV
AHAGAKINRIPANLLDQFEKQLPLHRDGFHTLQYQRTSAAAEQRSESPGRIRHLVHSVQK
LFTKSHSLEGSSKSNANGTKADGRADDHHHAHHAKHSKRSKSKERKPEGKPRPGMSSWWS
SDDNLDSDSTYRTPSVLNRHHLGPVAHCYPDALQSPFGDLSLKTSKSNNDVKCSACEGLA
LTPDAKYLKRSSWSTLTVSQAKEAYRKSSLNLDKPLLHQDAKPALRPCHYLQVPQDEWGG
YPTGGKDEEIPCRRMRSGSYIKAMGDEESGESDSSPKTSPKSAILPEPLLKSIGQRPLGE
HQTQTYLQAASDVPVGHSLDPAANYNSPKFRSRNQSYMRAVSTLSQASCVSQVSEAEING
QFESVCESVFSEVESQAMDALDLPGCFRTRSHSYLRAIQAGYSQDDECIPMMTPSDITST
IRSTAAVSYTNYKKTPPPVPPRTTSKPLISVTAQSSTESTQDAYQDSRAQRMSPWPQDSR
GLYNSTDSLDSNKAMNLALETAAAQRHLPESQSSSVRTSDKAILVSKAEELLKSRCSSIG
IQDSEFPEHQPYPRSDVETATDSDTESRGLREYHSVGVQVEDEKRHGRFKRSNSVTAAVQ
ADLELEGFPGHITMEDKGLQFGSSFQRHSEPSTPTQYSAVRTVRTQGLFSYREDYRTQVD
TSTLPPPDPWLEPAIDTVETGRMSPCRRDGSWFLKLLHAETKRMEGWCKEMEREAEENDL
SEEILGKIRSAVGSAQLLMSQKFQQFYWLCQQNMDPSAMPRPTSQDLAGYWDMLQLSIED
VSMKFDELQRLRLNDWKMMESPERKEERKVPPPIPKKPPKGKFPITREKSLDLPDRQRQE
ARRRLMAAKRAASFRQNSATERADSIEIYIPEAQTRL

>Mus musculus_DLGAP2(ENSMUSP_39647)
MGTAQVLPGILQKHCCILPDRNTESQCTLCGEPEEEEGGDLAQPGLSFPGPAEEDIDQQY
SWSPTQHFNEERYSPAPRNMKGLTGSRNQPQLCAGHTCGLSPPDDCEHPHDHMHHGSDVR
QPYLLSPAESCPMDHHRCSPRSSVHSECMMMPVMLGDHVSSSTFPRMHYSSHYDTRDDCA
MSHTSTKVNRIPANLLDQFEKQLPLHRDGFHTLQYQRASAATEQRNESPGRIRHLVHSVQ
KLFTKSHSLEGSSKSNINGTKSDSRVDDHHQSHLSKHSKRSKSKERKPESKHKSGMSSWW
SSDDNLDSDSTYRTPSVAHRHHMDHIPHCYPEALQSPFGDLSLKTSKSNNDVKCSACEGL
ALTPDTRYMKRSSWSTLTVSQAKEAYRKSSLNLDKPLVHPEIKPSLRPCHYLQVPQDDWG
AYPTGGKEEEIPCRRMRSGSYIKAMGDEESGESDSSPKTSPTVAIRPEPLLKPIIQRPLG
DHQTQSYLQAATEVPVGHSLNPSINYNSPKFRSRNQSYMRAVSTLSQASCVSQMSEAEVN
GQFESVCESVFSEVESQAMDALDLPGCFRTRSHSYLRAIQAGYSQDDECIPVMTSSNMTS
TIRSTAAVSYTNYKKTPPPVPPRTTSKPLISVTAQSSTESTQDAYQDSRAQRMSPWPQDS
RGGLYNSMDSLDSNKAMNLALETAAAQRHAADTQSSSTRSIDKAVLASKAEELLKSRCSS
IGVQDSEFPDHQPYPRSDVETATDSDTESRGLREYHSVGVQVEDEKRHGRFKRSNSVTAA
VQADLELEGFPGHVSMEDKGLQFGSSFQRHSEPSTPTQYGALRTVRTQGLFSYREDYRTQ

VDTSTLPPPDPWLEPSLDTVETGRMSPCRRDGSWFLKLLHTETKRMEGWCKEMEREAEEN
DLLEDILGKIRSAVGSAQLLMSQKFQQFYWLCQQNMDPSAMPRPTSQDLAGYWDMLQLSV
EDVSMKFDELHQLKLNDWKIIESPERKEERKIPPPIPKKPPKGKFPITREKSLDLPDRQR
QEARRRLMAAKRAASFRQNSATERADSIEIYIPEAQTRL

>Rattus norvegicus_DLGAP2(ENSRNOP_54654)
DQQYSWSPTQHFNEERYSPAPRNMKGLTGSRNQPQLCVGHTCGLSPADECEHPHDHVRHG
PDVRQPYLLSPAESCPMDHHRCSPRSSVHSECMMMPVMLGDHVSSSTFPRMHYSSHYDTR
DDCATSHASTKVNRIPANLLDQFEKQLPLHRDGFHTLQYQRASAATEQRNESPGRIRHLV
HSVQKLFTKSHSLEGSSKSNINGTKSEGRMDDHHQSHLSKHSKRSKSKERKPESKHKSGM
SSWWSSDDNLDSDSTYRTPSVAHRHHMDHIPHCYPEALQSPFGDLSLKTSKSNSDVKCSA
CEGLALTPDTRYMKRSSWSTLTVSQAKEAYRKSSLNLDKPLVHPEIKPSLQPCHYLQVPQ
DDWGAYPTGGKEEEIPCRRMRSGSYIKAMGDEESGESDSSPKTSPTVALRPEPLLKSIIQ
RPLGDHQTQSYLQAATEVPVGHSLDPSVNYNSPKFRSRNQSYMRAVSTLSQASCVSQMSE
AEVNGQFESVCESVFSEVESQAMDALDLPGCFRTRSHSYLRAIQAGYSQDDECIPVMTPS
NMTSTIRSTAAVSYTNYKKTPPPVPPRTTSKPLISVTAQSSTESTQDAYQDSRAQRMSPW
PQDSRGGLYNSMDSLDSNKAMNLALESAAAQRHAADTQSSSTRSIDKAVLVSKAEELLKS
RCSSIGVQDSEFPDHQPYPRSDVETATDSDTESRGLREYHSVGVQVEDEKRHGRFKRSNS
VTAAVQADLELEGFPGHVSMEDKGLQFGSSFQRHSEPSTPTQYGALRTVRTQGLFSYRED
YRTQVDTSTLPPPDPWLEPSLDTVETGRMSPCRRDGSWFLKLLHTETKRMEGWCKEMERE
AEENDLSEEILGKIRSAVGSAQLLMSQKFQQFYWLCQQNMDPSAMPRPTSQDLAGYWDML
QLSVEDVSMKFDELHQLKLNDWKIMESPERKEERKIPPPIPKKPPKGKFPITREKSLDLP
DRQRQEARRRLMAAKRAASFRQNSATERADSIEIYIPEAQTRL

>Loxodonta africana_DLGAP2(ENSLAFP_6018)
EEEGGDLAQPGISFPGPAEEELDQRYSWSPTQHFNEERYSPAPRNMKGLSGSRNQPQLCS
GHTCGLTPPEDCEHTHDHIHHGQETRQSYLLSPTDSCPMDHHRCSPRSSIHSECMMMPVM
MGDHMSSSTFPRMHYSSHYDTRDDCAMSHASTKINRIPANLLDQFEKQLPLHRDGFHTLQ
YQRTSTAEQRSESPGRIRHLVHSVQKLFTKSHSLEGSSKSNVNGTKGDSRVDDHHHGHHS
KHSKRSKSKERKPESKHKSGMSSWWSSDDNLDSDSTYRTPSVINRHHVDHISHCYPETLQ
GPFGDLSLKTSKSNNDVKCSACEGLSMTPDTKYMKRSSWSTLTVSQAKEAYRKSSLNLDK
PLVHQEIKPSLRPCHYLQVPQDEWGGYPTGGKDEEIPCRRMRSGSYIKAMGDEESGESDS
SPKTSPKVAIRPEPLLKSIGQRPLGDHQTQSYLQAASDVPGGHNLDPSANYTSPKFRSRN
QSYMRAVSTLSQASCVSQMSEAEINGQFESVCESVFSEVESQAMDALDLPGCFRTRSHSY
LRAIQAGYSQDDECIPAMTSSDIASTIRSTAAISYTNYKKTPPPVPPRTTSKPLISVTAQ
SSTESTQDAYHDSRTQRISPWPQDGRGLYNSTDSLDSNKAMNLALESAAAQRQASESQGG
SARTSDKAILVSKAEEFLKSRRSSIGIQDSEFPEHQPYPRSDVETAADSDTESRGLREYH
SVGVQVEDEKRHGRFKRSNSVTAGVQADLELEGFPGHITMEDKGLQFGSSFQRHSEPSTP
TQYGAVRTVRTQGLFSYREDYRTQVDTSNLPPPDPWLEPSIETVETGRMSPCRRDGSWFL
KLLHTETKRMEGWCKEMEREAEENDLSEEILGKIRSAVGSAQLLMSQKFQQFYWLCQQNM
DPSAMPRPTSQDLAGYWDMLQLSIEDVSMKFDELHHLKLNDWKIIESPERKEERKVPPPI
PKKPPKGKFPITREKSLDLPDRQRQEARRRLMAAKRAASFRQNSATERADSIEIYIPEAQ
TRL

>Dasypus novemcinctus_DLGAP2(ENSDNOP_4883)
DQQYSWSPTQHFNEERYSPAPRNMKGLSGSRNQPQLCSGHTCGLTPPEDCEHSHDHLHHG
QETRQSYLLSPTDSCPMDHHRCSPRSSIHSECMMMPMMMGDHMSSSTFPRMHYSSHYDTR
DDCAMSHASTKINRIPANLLDQFEKQLPLHRDGFHTLQYQRTSTAAEQRSESPGRIRHLV
HSVQKLFTKSHSLEGSSKSNVNGTKGDSRMDDHHHGHHSKHSKRSKSKERKPESKHKSGM
SSWWSSDDNLDSDSTYRTPSVLNRHHVDHISHCYPETLQSPFGDLSLKTSKSNNDVKCSA
CEGLAMTPDTKYMKRSSWSTLTVSQAKEAYRKSSLNLDKPLIHQEIKPSLRSCHYLQVPQ
DEWGGYPTGGKDEEIPCRRMRSGSYIKAMGDEESGESDSSPKTSPKVATRPEPLLKSIAQ
RPLGDHQTQSYLQAASDVPGGLGLDPSANYNSPKFRSRNQSYMRAVSTLSQASCVSQMSE
AEINGQFESVCESVFSEVESQAMDALDLPGCFRTRSHSYLRAIQAGYSQDDECIPAMTSS
NITSTIRSTAAAPYTNYKKTPPPVPPRTTSKPLISVTAQSSTESTQDAYHDSRAQRGSPW
PQDARGLYNSTESLDSNKALNLALETAAAQRHAAEGQGGSARAGDKAVLGAKAEEFLRSR
RASVGIQDSEFPEHQPYPRSDVETATDSDTESRGLREYHSVGVQVEDEKRHGRFKRSNSV
TAAVQADLELEGFPGHVTTEDKGLQFGSSFQRHSEPSTPTQYGAVRTVRTQGLFSYREDY
RTQVDTPNLPPPDPWLEPSIETVETGRMSPCRRDGSWFLKLLHTETKRMEGWCKEMEREA
EENDLSEEILGKIRSAVGSAQLLMSQKFQQFYWLCQQNMDPSAMPRPTSQDLAGYWDMLQ
LSIEDVSMKFDELHQLKLNDWKMIESPERKEERKVPPPIPKKPPKGKFPITREKSLDLPD

RQRQEARRRLMAAKRAASFRQNSATERADSIEIYIPEAQTRL

>Monodelphis domestica_DLGAP2(ENSMODP_17783)
DQQYSWSPTQHFNEERYSPAPRNMKGLSSSRNQPQLCSGHTCGLTPPEDCEHTHDHLHHG
QEPRQSYLLSPTESCPMDHHRCSPRSSIHSECMMMPMMMGDHMSSSTFPRMHYSSHYDTR
DDCAMSHASTKINRIPANLLDQFEKQLPLHRDGFHTLQYQRTSNAAEQRSESPGRIRHLV
HSVQKLFTKSHSLEGSSKSNVNGTKSDSRGDDHHHGHPSKHSKRSKSKERKPESKHKSGM
SSWWSSDDNLDSDSTYRTPSVVNRHHVDHISHCYPDALQSHFGDLSLKTSKSNNDMKCSA
CEGLAMTPDGKYMKRSSWSTLTVSQAKEAYRKSSLNLDKPLVHQEIKPALRPCHYLQVPQ
DEWGGYPTGGKDEEIPCRRMRSGSYIKAMGDEESGESDSSPKTSPKVAVRTEALLKSIGQ
RPLGDHQTQSYLQAAGDVPGGHNLDPTANYNSPKFRSRNQSYMRAVSTLSQASCVSQMSE
AEINGQFESVCESVFSEVESQAMDALDLPGCFRTRSHSYLRAIQAGYSQDDECIPAMTSS
NITSTIRSTTAVSYTSYKKTPPPVPPRTTSKPLISVTAQSSTESTQDAYHDSRTQRIPPW
PQDGRGLYNSTDSLDSNKAMNLALETAAAQRHASESQSSSVRTSDKAVLVTKAEEFLKSR
RSSIGIQTEMFPTHSLPYHSSTVETATDSDTESRGLREYHSVGIQVEDDKRHGRFKRSNS
VTAAVQADLELEGFPGHITMEDKGLQFGSSFQRHSEPSTPTQYGAVRTVRTQGLFSYRED
YRTPVDTSNLPPPDPWLEPSIETVETGRMSPCRRDGTWFMKLLHTETKRMEGWCKEMERE
AEENDLSEEILGKIRSAVGSAQLLMSQKFQQFYWLCQQNLDPSAMPRPTSQDLAGFWDLL
QLSIEDVSMKFDELHQLKLNEWKLIESPERKEERKVPPPVPKKPPKGKFPITREKSLDLP
DRQRQEARRRLMAAKRAASFRQNSATERADSIEIYIPEAQTRL

>Gallus gallus_DLGAP2(ENSGALP_26331)
MGTIQVLPGILQKHCCILPDRNTESQCTLCGEPEDLDSLTGHAFPEDEGGDLLKSSFSFP
GTPEEEIDQQYSWSPSHHFNEERYSPASRNMKGLSGSRNQPQLCSAHTCGLASPEDCEHT
HDHIHHGQEPRQSYLLSPTESCPMDHHRCSPRSSIHSECMMMPMVMGDHMSSSTFPRMHY
SSHYDTRDDCAMSHGNPKINRIPANLLDQFEKQLPLHRDGFHTLQYQRTSTAAEQRSESP
GRIRHLVHSVQKLFTKSHSLEGSSKGNVNGTKGDSRGDDHHHGHHSKHGKRSKSKERKPE
TKHKSGMSSWWSSDDNLDSDSTYRTPSVVNRHHADHISHCYPEALQGHFGDLSLKTSKSN
NDVKCSACEGLAMTPDSKYMKRSSWSTLTVSQAKEAYRKSSLNLDKPLVHQEIKPSLRPC
HYLQVPQDEWGGYPAGGKDEEIPCRRMRSGSYIKAMGDEESGDSDTSPKTSPKLAVRPES
LLKSIGQRPLGDHQNQSYLQTASDVPGGHNLDPSANYNSPKFRSRNQSYMRAVSTLSQAS
CVSQMSEAEVNGQFESVCESVFSEVEAQAMDALDLPGCFRTRSHSYLRAIQAGYSQDDEC
IPAMASSNITSTIRSTTAVTYTSYKKTPPPVPPRTTSKPLISVTAQSSTESTQDAYHDSR
AQRMSPWPQDGRGMYNSTDSLDSNKAMNLALETAAAQRHVSESASIRTSDKAILVTKAEE
FLKSRRSSIGIQVETATDSDTESRGLREYHSVGIQVEDEKRHGRFKRSNSVTAAVQADLE
LEGFPGHITMEDKGLQFGSSFQQHSEPSTPTQYSAVRTVRTQGLFSYREDYRTPVDTSNL
PPPEPWLEPAIETVETGRMSPCRRDGSWFMKLLHTETKRMEGWCKEMEREAEENDLSEEI
LGKIRSAVGSAQLLMSQKFQQFYWLCQQNLDPSAMPRPTSQDLAGFWDLLQLSIEDVSMK
FDELHQLKLNEWKIIESPERKPTGWWMEERKVPPPVPKKPPKGKFPITREKSLDLPDRQR
QEARRRLMAAKRAASFRQNSATERADSIEIYIPEAQTRL

>Anolis carolinensis_DLGAP2(ENSACAP_5144)
MKGLSGSRNQSQLCSAHTCGLASPEDCEHTHDHIHHGQESRQSYLLSPTESCPMDHHRCS
PRSSIHSECMMMPMVMGDHMSSSTFPRMHYTSHYDTRDDCTMSHANPKVNRIPANLLDQF
EKQLPLHRDGFHTLQYQRTSTAAEQRSESPGRIRHLVHSVQKLFTKSHSLEGSSKSNVNG
TKGDGRGDDHHHGHHSKHGKRSKSKERKPETKHKTGMSSWWSSDDNLDSDSTYRTPSVVN
RHHVDHISHCYPEALQGHFGDLSLKTSKSNNDVKCSACEGLAMAPDGKYMKRSSWSTLTV
SQAKEAYRKSSLNLDKPLVHQEIKPSLRPCHYLQVPQDEWGGYPAGGKDEEIPCRRMRSG
SYIKAMGDEESGDSDTSPKTSPKVAVHPESLLKSIGQRALGDHQNQSYLQAASDVPGGHS
LDPAANYNSPKFRSRNQSYMRAVSTLSQASCISQMSEAEVNGQFESVCESVFSEVEAQAM
DALDLPGCFRTRSHSYLRAIQAGYSQDDECIPAMASSNITSTIRSTTAVTYTSYKKTPPP
VPPRTTSKPLISITAQSSTESTQDAYHDSRTQRISPWPQDSRGMYNSTDSLDSNKAMNLA
LETAAAQRHMSENQSTSVRTSDKAILVTKAEEFRKSRRSSIGIQVETATDSDTESKGLRE
YHSVGVQVENDKRHGRFKRSNSVTAAVQADLELEGFPGHITMEDKGLQFGGSFQRHSEPS
TPTQYGAVRTVRTQGLFSYREDYRTQVDTSNLPPPEPWLEPAIETVETGRMSPCRRDGSW
FMKLLHTETKRMEGWCKEMEREAEENDLSEEILGKIRSAVGSAQLLMSQKFQQFYWLCQQ
NLDPSAMPRPTSQDLAGFWDLLQLSIEDVSIKFDELHQLKLNEWKIIESPERKEERKVPP
PVPKKPPKGKFPITREKSLDLPDRQRQEARRRLMAAKRAASFRQNSATERADSIEIYIPE
AQTRL

>Danio rerio_DLGAP2(ENSDARP_139510)

IDQLDSWSPPPYFDEDSYPSPPRNMKGLSSGRPQLTFTSGHTCGLPDCDQSLDHLQHVGP
DSRSPYLLSPTESCPLDPHRCSPRSSIHSECMVMPVSMASDHVSSSTFPRMHYSSHQDSL
RDDGSTSHGSGKMNRIPANLLDQFEKQLPIHRDGFHTLQYQRTSATTTNSEQRNESPGRI
RHLVHSVQKLFTKSHSLEGSSKMNGTKGDSRSDSGHHHHHHHHHGHDHGSHKHSKRSKSK
ERKSDSKQRSGIAGWWSSDDNLDSDSTYRTPSIMSRHHGDHITHCYPDMGHGHFGDLSLK
TSKSNNDVKCSACESIAMAPEGKFMKRSSWSTLTVSQAKEAYRKSSLNLEKPTMPQDLKS
SMRPCHFLQVPQDEWGGRDEEIPCRRMRSSSYVKAMGDQDSGDSDGSPKNSPQKMVRPNA
LVKSVLQRQLSDQRPSQSYHLQTSREIHPSITLEPSPNYNSPKFRSRNQSYMRAVSTLSQ
ASCVSQVSETEVNGQFESVCESVFSEVESQAMDALDLPGCFRTRSHSYLRAIQAGFSQDD
DCVPSMTSSTVTSTIRSTTAVTYTPSTPTYKRTPPPVPPRSTSKPLISITAQSSTESTQD
AYHEGQLARGFWGSDGSSLSLGRALYNSTDSLDSAKAVTIAMEAAAMAMAGKRHPSTDSH
SSVMTCDKAVLVSKAEEYLKTPRSSIGIQSSVFVQSQKIGRRDVEAATDSECESKGSREY
QSVGIQVEDERRQGRFKRSNSVTAAVQADLELEGFPLMEDKGLQFGGGFQRHSEPSTPTQ
YGAVRTVRTQGLFSYREDYRTPTEPPSPQRSPEPWLEPSPREPIATVAVTVTPPQPSDSG
RASPSCRRDGSWFMQLLHAETKKMEGWCKELEGEAEENDLSEEILGKIRSAVGSAQLLMS
QKFQQFYWLCQQNLDPSAMPRPTSQDLAGFWDLLQLSIDDVTSKFNELQKIKSNDWRLID
SPVKKVRADYLPPPVPKKTLRKSVTREKSLDLPDRQRIEARRRLMAAKRAASFRQNSASE
RADSIEIYIPEAQTRL

>Takifugu rubripes_DLGAP2(ENSTRUP_27118)
VDQQYSWSPSQYFDEDSNSPPPRNMKGLSGGRPAQLQLTSPTSAYSCGLVECDPSLDHHL
HHGNVRSPSYLLSPTESCPLDSHRRCSPRSSIHSECMVMPVPMSTTDHSISSSTFPRMHY
GSASRDSGGNTSGGSKMNRIPANLLDQFEKQMPLHRDGFHTLQYQRTSTTTTSTEQRNES
PGRIRHLVHSVQKLFTKSHSLEGSSKINGTKSESHRDSSHHHHHHHQGHHKHSKRSKSKD
RKSDGGGKQRSGGWWSSDDNLDSDSTYRPPSVMSRHPVEHISHCYSDSVHGHLVGDMSLK
TSKSNNDVKCTACESINMAPEGKFMKRSSWSTLTVSQAKEAYRKSSLNLEKPMTPTDLKP
NLRPCHYLQVPQDDWGGYPGDGKDDEIPCRRMRSSSYVKAMGDEESGESDSSPKASPQKS
VRPEALVKAVIRPRDLLDSQSQSYHLQTTRNMHPSIALEPSTNYNSPKFRSRNQSYMRAV
STLSQASCVSQVSETEINGQFESVCESVFSEVESQAMEALDLPGCFRTRSHSYLRAIQAG
YSQDDDCIPPMASTVTSTIRSTTAITYTPNYKKTPPPVPPRTTSKPLISITAQSSTESTQ
DAYHEGRHPHSGMWNPESLGLGPGRGLYNSNDSLDSTKAVTIAMEAAGVMAGKRHPSTDS
HSSVLTCDKAVLVSKAEEYLKTPSSIGIQSFIDLPLPSHPPSHRLALVEAATDSESESKG
SREYQSVGIQVEDNTKGQGRFKRSNSVTAAVQADMELEGFPVMEDKGLQFGGGFQRHSEP
STPTQYGAVRTVRTQGLFSYRDDYRTLAEPPSPRETSREATWQLEPPSPRENTASSAESG
RASPSLRRDGSWFMQLLHTDTKRIEGWCKEMEAEAEENDLSEEILGKIRSAVGSAQLLMS
QKFQQFYWLCQQNLDPSAMPRPTSQDLAGFWDLLQLSIDDVTVKFDQLQQIKNNNWRLID
SPEKKPPAPVPKKTARQKSVITREKSLDLPDRHRQEARRRLMAAKRAASFRQNSGSERAD
SIEIYIPEAQTRL

>Tetraodon nigroviridis_DLGAP2a(ENSTNIP_13668)
MKGLSSSRFQHQIPLPYGLDYDPHVHDLHAHHGSESRHPSYLLSPTESCPTDFHHRYSPR
SSIHSDCMVMSPVMMPPPAASDHISSSTFPRMHYSSQYYDTATRDDCAAITASASVKSNR
LPANLLDQYEKQLPLHRDGFHTLQYQRTSTTTEQRSESPGRIRHLVHSVQKLFTKSHSLE
GSSKMNGTKGDIGGGGGAGYHHYGQHSTKHGSKRSKSKERKHRSGGWWSSDDNLDSDSTY
RTPSVMSRHHGEHASHCHPDSMHSHHFGDLSLKTTKSSNDVKCSACEALGPEGKFMKRSS
WSTLTVSQAKEAYRRSAFNLDKPMMHTDLKSSRTCHFLQVPQDDWGGYPGEKDEEIPCRR
MRSSSYVKAMGEVDDDSGDSDTSIKTSPQKTVRSDALVLKSAMQRPHFDPQRVLNTQHPL
PPILTFSQGYHLQTLRDMRPHPSVTLDPATVHPPPFRSRNQSYMRAVSTLSQVSETEING
QFESVCESVFSEVESQAMEALDLPGSFRTRSHSYLRAIQAGYSQDDDCLPSMTSSTVTST
LRSTTAQKNGTNSKVTDYKKTPPPVPPRTTTKPLISVTAQSSTESTQDAYQYQEGKHPRS
GLWTTDSLGRPIYSSTDSLDSTKAVTMAMESAAGKRHTSLGSHTSVQTCDKAVLVSKAEE
YLKTPRSSIGIQVETVTDSEPETKGLPQFHSIGIQVEDRKRHGRFKRSNSVATAVQADME
LEGFPSMEDKGFQFGGAFGRHSEPSTPTQYGAIRTVRTQGLFSYREDYRPSSRPSSPHPE
PWLVEPSLESTDTGRVSPIRRDGSWFMQLLHNETKRLEGWCKEMEREAEEHDLSEEILGK
IRSAVGSAQLLMSQKFQQFYWLCEQNLDPSAMPRPTSQDLAGFWDLLQLSVDDVTAKFDE
LHQIKDNHWQLVKSPEKKERKWPPPVPKRPARGRSGIMRERSLDLQDRQRQEARRRLMAA
KRAASFRQNSATERADSIEIYIPEAQTRL

>Tetraodon nigroviridis_DLGAP2b(ENSTNIP_20628)
MKGLSGVRPAQLQLTSPVSAHTCGLAECDPSLDHHLHHGNARSPSYLLSPTESCPLDSHR
RCSPRSSIHSECMVIPVSMSTTDHSISSSTFPRMHYGSASRDSGGNTSSGRNTRDDGGSS

SHGSGSKMNRIPANLLDQFEKQMPLHRDGFHTLQYQRTSTTTTTTEQRNESPGRIRHLVH
SVQKLFTKSHSLEGSSKMNGTKSDSHRDSKRSKSKDRKSDGSGKQRSGGWWSSDDNLDSD
STYRPPSVMSRHPVDHISHCFPDSVHGHLAGDMSLKTSKSNNDVKCTACDSINMAPEGKF
MKRSSWSTLTVSQAKEAYRKSSLNLEKPMTPTDLKANLRPCHYLQVPQDDWGGYPGDGKD
DEIPCRRMRSSSYVKAMGDEESGESDSSPKASPQKTVRPDALFVAIQPSRTEMYLPSFAN
SIKGVLSLFSQSYHLQAARNIHPSIALDPSANYNSPKFRSRNQSYMRAVSTLSQVSETEV
NGQFESVCESVFSEVESQAMEALDLPGCFRTRSHSYLRAIQAGYSQDDDCIPPMASTVTS
TIRSTTAITYTPNYKKNPPPVPPRTTSKPLISITAQSSTESMQDAYHEGRHPHGRMWTPE
GLSLGLGPGRGLYNSTDSLDSTKAVTIAMEAAGLMAGKRHPSTDSHSSVLTCDKAILVSK
AEEYLKTPRSSIGIQVEAATDSESESKGSREYQSVGIQVEDNKKGQGRFKRSNSVTAAVQ
ADLELEGFPVIESAASSAESGRASPSLRRDGSWFMQLLHTDTKRIEGWCKEMEAEAEEND
LPEETSPLGLEPPSPRESAASSAESGRASPSLRRDGSWFMQLLHTDTKRIEGWCKEMEAE
AEENDLPESVLGKIRSAVGSAQLLMSQKFQQFYWLCQQNLDPSAMPRPTSQDLAGFWDLL
QLSIDDVTMKFDQLQQIKNNSWRLIDSPEKKPPAPVPKKTARQKTVITREKSLDLPDRHR
QEARRRLMAAKRAASFRQNSASERADSIEIYIPEAQTRL

>Gasterosteus aculeatus_DLGAP2a(ENSGACP_10715)
ETDQKFSPWSPSPKFNEDRRLPPTRNMKGLSGSRSQHQIPLPYGLDYDPHVHDLHGHHGS
DSRHSSYLLSPTESCPMDFHHRYSPRSSIHSDCMMMAPVMVPQAAASEHISSSTFPRMHY
SSPYYDPAAREKSNRLPANLLDQFEKQLPLHRDGFHTLQYQRTSTTTEQRGESPGRIRHL
VHSVQKLFTKSHSLEGSSKMNGTKGEERVSHHAHSTSMAARGARAKERKHRSGGWRSSDD
NLDSDSTYRTPSVMSRHHGEHVGHCYPDSTHSHHFGDLSLKASKSNNDVKCSACESMAMA
PEGKFMKRSSWSTLTVSQAKEAYRKSSLHLEKPMMHSDLKHSSRPCHYLQVPQDEWSGYP
GDKDEEIPCRRMRSSSYVKAMGEVEDESGDSDSSPKISPQKAVRPDALVLKSAMQRPRLD
SHSQGYHMQTARDMRPQHSVTLDPAAFHPPPFRSRNQSYMRAVSTLSQASCVSQVSETEI
NGQFESVCESVFSEVESQAMEALDLPGSFRTRSHSYLRAIQAGYSQDDDCLPSMTSSTVT
STLRSTTAVSYPPSYKKTAPPVPPRGAPKPLISVTAQSSTESTQDAYQEGRHPRGGLWTT
DSLGRPIYSSADSLDSTKAVTMAMESAAGKRHASSGSHTSVQTCDKAVLVSKAEEYLKTP
RSSIGIQKLSQLQSTHQEHFQICQVETVTDSESESKGLPQFHSIGIQVEDHKRHGRFKRS
NSVTTAVQADMELEGFPSMEDKGLQFGGGFGRHSEPSTPTQYGAIRTVRTQGLFSYREDY
RPSSGPPSPQPEAWLAEPSLEGADTGRVSPLRRDGSWFMQLLHNETKRLEGWCKEMEREA
EEHDLTEEILGKIRSAVGSAQLLMSQKFQQFYWLCQQNLDPSAMPRPTSQDLAGFWDLLQ
LSIDDVTAKFDELQQIKSNRWQLVESPDKKKERKWPPPLAKRPSKGRGVMRERSLDLQDR
QRQEARRRLMAAKRAASFRQNSVTERADSIEIYIPEAQTRL

>Gasterosteus aculeatus_DLGAP2b(ENSGACP_8971)
DQLYSWSPSQYFDEDSCSLPPRNMKGLSGGRPAQLHLTSPTAHTCGLVECDHSLEHHLHH
GNSRPPSYLLSPTDSCPLESHHRCSPRSSIHSECMVVPVSMSATDHSISSSTFPRMHYGS
GARESGGNTSGGRDSRDDGGSSSHGGSGKMNRIPPNLLDQFEKQMPLHRDGFHTLQYQRT
STTTTTTEQRNESPGRIRHLVHSVQKLFTKSHSLEGSSKMNGTKGDPHRESSHHANHHHQ
GHHKHSKRSKSKERKSDGSGKQRSGGWWSSDDNLDSDSTYRTPSVMSRHPADHVGHCYPD
SMHGHLAGELSLKTSKSNNDVKCSACESISMAPEGKFMKRSSWSTLTVSQAKEAYRKSSL
NLEKPMTPTELRPNLRPCHYLQVPQDDWGGYPGDEKDDEIPCRRMRSSSYVKAMGDEESG
ESDSSPKTSPQKTVRPDAMVKAVIRPRELLDSQSEGSHFQAAGKRRPRFAGDPSAIYISP
NFGPGNKSYGQAFSPLIQPSRVTQVSETEMNGQFESVCESVFSEVESQAMEALDLPGCFR
TRSHSYLRAIQAGYSQDDDCIPPMGSTVTSTIRATTAITYTTNYKKAPPPVPPRTTSKPL
ISITAQSSTESTQDAYHEARHPRGGMWAPDGLGLGLNAGRVLYNSTDSLDSTKAVTIAME
AAGVMVGKRHPSTDSHSSVLTCDKAVLVSKAEEYLKTPRSSIGIQVSPLSPYLGQPSAEV
EAATDSESEGKGLRQYHSVGIQVEDNKKGQGRFKRSNSVTAAVQADMELEGFPTMEDKGL
QFGGGFQRHSEPSTPTQYGAVRTVRTQGLFSYREDYRATAEPSSPRETTSEASWQLEPPS
PRKSAASPGDSGRASPSLRRDGSWFMQLLHTETKRIEGWCKEMEAEAEENDLSEDILGKI
RSAVGSAQLLMSQKFQQFYWLCQQNMDPSAMPRPTSQDLAGFWDLLQLSIDDVTMKFDRL
QQIKKNNWRPVDSPEKKLFHLKPPAPVPKKTIRQRSLVTREKSLDLPDRGRQEARRRLMA
AKRAASFRQDSASERADSIEIYIPEAQTRL

>Oryzias latipes_DLGAP2a(ENSORLP_21608)
DQQYSWSPSQYFDEDSYTPPPRNMKGLSGGRPPQLQLTSPTSAHTCGLAECDHPLDHHFH
GDARSPSYLLSPTDSYPLESLHRCSPRSSIHSECMMMPMSLSATDHSISSSTFPRMHYGS
GSRDSGGNTSGGRDSRDDGSSSQGGSGKMNRIPANLLDQFEKQMPLHRDGFHTLQYQRTA
TTTTTTEHRNESPGRIRHLVHSVQKLFTKSHSLEGSSKMNGTKSDSHRDSSRHHHHHHQG
HHKHSKRSKSKDRKSEGGGKHRSGGWWSSDDNLDSDSTYRTPSIMSQHPVDHITHCYPDS

VHSHLAGDLSLKTSKSNNDVKCSVCESMSMAPEGKFMKRSSWSTLTVSQAKEAYRKSSLN
LEKPMIPSDLKPNLRPCSYLQVPQDEWGGYPGGGKDDEIPCRRMRSSSYVKAMGDEESGE
SDSSPKTSPQKSVRKDALVKAIIRPKIGCHQVTKNYHLQATREIHPSFALDPSTNYNSPK
FRSRNQSYMRAVSTLSQASCVSQVSETEVNGQFESVCESVFSEVESQAMEALDLPGCFRT
RSHSYLRAIQAGYSQDDDCIPPMASTVTSTIRSTTAITYTPSYKKAPPPVPPRTTSKPLI
SITAQSSTESTQDAYHDGRHPRGGVWTADGLSLGLSPGRALYNSNDSLDSAKAVTIALEA
AGVMAGKRHPSTDSHSSLLTCDKAILVSKAEEYLKTPRSSIGIQSITVPSRYAAPPADVE
TATDSEAESKGSPEYHSVGIQVEDDKKGQGRFKRSNSVTAAVQADLELEGFPTMEDKGLQ
FGGGFQRHSEPSTPTQYGAVRTVRTQGLFSYRDDYRTPAEPPSPRETTSENTWQLEPPSP
RESNSSPGESGRVSPSLRRDGSWFMQLLHNETKKMEGWCKEMEAEAEENDLSEEILGKIR
SAVGSAQLLMSQKFQQFYWLCQQNLDPSAMPRPTSQDLAGFWDLLQLSIDDVSIKFDQLQ
QIKNNGWRPVDSPEKKSLRRQTPAPIPKKTARQKSTITREKSLDLPDRHRQEARRRLMAA
KRAASFRQNSATERADSIEIYIPEAQTRL

>Oryzias latipes_DLGAP2b(ENSORLP_14160)
DQQSSTWSPPRKFNEGRHLPPSRNMKGLSGSRSQHHIPLSHGLDYDPHVHDLHAHHGSDT
RYLSYLLSPTESCPIDFHHRYSPRSSVHSDRMMMAPVTMPPPAVSDHISSSTFPRMHYSS
QYYDSVTRDDCAAIHSSTKSNRLPANLLDQFEKQLPLQRDGFHTLQYQRASTAAEQRSES
PGRIRHLVHSVQKLFTKSHSLEGSSKMNGTKGDIIGGGGGTGGYYHHSHHSTKHGSKRSK
SKERKHRSGGWWSSDDNLDSDSTYRPSSVMSRHHGEHVDHCYPDSMHSHHFGEMSLKGSK
SNNDVKCSACESMAMAPEGKYMKRSSWSTLTVSQAKEAYRKSSLNLDKPIMHTDLKISSR
TPHYLQVPQDDWGGYPGEKDEEIPCRRMRSSSYVKAMGEVDDESGESDTSPKMSPVSKTV
RPDALVLKSAMQRPHLESQSQGYHLQTRDMRSHPSVTLNPAPSFHPPPFRSRNQSYMRAV
STLSQASCVSQVSETEINGQFESVCESVFSEVESQAMEALDLPGSFRTRSHSYLRAIQAG
YSQDDDCLPSMTSSTVTSTLRSTTVVSYPPSYKKTPPPVPPRTTTKPLISVTAQSSTEST
QDAYQGGRHPRGGLWTTDSLGRPIYSSMDSLDSAKAVTMVMETGASKRHASLGSHTSVQT
CDKAVLVSKAEEYLKTPRSSIGIQKCLASSKKKYNCSLDISQVEPATDTEADKTGLPQFH
SIGIQVEDKKRRGRFKRSNSVTTAVQADLELEGFPSMEDKGLQFGGGFGRHSEPSTPTQY
GAIRTVRTQGLFSYREDHRPSSRPTSPQPEPWLVEPSLESTDTGRVSPLRRDGSWFMQLL
HNETKRLEGWCKDMEQEAEDNDLSEEILGKIRSAVGSAQLLMSQKFQQFYWLCQQNMDPS
AMPRPTSQDLAGFWDLLQLTIDDVTAKFDELQEIKNNQWNLLESPEKNEQERKWPPPLPK
RSAKGRGSVMRERSLDLQDRQRQEARRRLMAAKRAASFRQNSATERADSIEIYIPEAQTR
L

>Homo sapiens_DLGAP3(ENSP_235180)
MRGYHGDRGSHPRPARFADQQHMDVGPAARAPYLLGSREAFSTEPRFCAPRAGLGHISPE
GPLSLSEGPSVGPEGGPAGAGVGGGSSTFPRMYPGQGPFDTCEDCVGHPQGKGAPRLPPT
LLDQFEKQLPVQQDGFHTLPYQRGPAGAGPGPAPGTGTAPEPRSESPSRIRHLVHSVQKL
FAKSHSLEAPGKRDYNGPKAEGRGGSGGDSYPGPGSGGPHTSHHHHHHHHHHHQSRHGK
RSKSKDRKGDGRHQAKSTGWWSSDDNLDSDSGFLAGGRPPGEPGGPFCLEGPDGSYRDLS
FKGRSGGSEGRCLACTGMSMSLDGQSVKRSAWHTMMVSQGRDGYPGAGPGKGLLGPETKA
KARTYHYLQVPQDDWGGYPTGGKDGEIPCRRMRSGSYIKAMGDEESGDSDGSPKTSPKAV
ARRFTTRRSSSVDQARINCCVPPRIHPRSSIPGYSRSLTTGQLSDELNQQLEAVCGSVFG
ELESQAVDALDLPGCFRMRSHSYLRAIQAGCSQDDDCLPLLATPAAVSGRPGSSFNFRKA
PPPIPPGSQAPPRISITAQSSTDSAHESFTAAEGPARRCSSADGLDGPAMGARTLELAPV
PPRASPKPPTLIIKTIPGREELRSLARQRKWRPSIGVQVETISDSDTENRSRREFHSIGV
QVEEDKRRARFKRSNSVTAGVQADLELEGLAGLATVATEDKALQFGRSFQRHASEPQPGP
RAPTYSVFRTVHTQGQWAYREGYPLPYEPPATDGSPGPAPAPTPGPGAGRRDSWIERGSR
SLPDSGRASPCPRDGEWFIKMLRAEVEKLEHWCQQMEREAEDYELPEEILEKIRSAVGST
QLLLSQKVQQFFRLCQQSMDPTAFPVPTFQDLAGFWDLLQLSIEDVTLKFLELQQLKANS
WKLLEPKEEKKVPPPIPKKPLRGRGVPVKERSLDSVDRQRQEARKRLLAAKRAASFRHSS
ATESADSIEIYIPEAQTRL

>Gorilla gorilla_DLGAP3(ENSGGOP_13017)
MRGYHGDRGSHPRPARFADQQHMDVGPAARAPYLLGSGEAFSTEPRFCAPRAGLGHISPE
GPLSLSEGPSVGPEGGPVGAGVGGGSSTFPRMYPGQGPFDTCEDCVGHPQGKGAPRLPPT
LLDQFEKQLPVQQDGFHTLPYQRGPAGAGPGPAPGTGTAPEPRSESPSRIRHLVHSVQKL
FAKSHSLEAPGKRDYNGPKAEGRGGSGGDSYPGPGSGGPHTSHHHHHHHHHHHQSRHGK
RSKSKDRKGDGRHQAKSTGWWSSDDNLDSDSGFLAGGRPPGEPGGPFCLEGPDGSYRDLS
FKGRSGGSEGRCLACTGMSMSLDGQSVKRSAWHTMMVSQGRDGYPGAGPGKGLLGPETKA
KARTYHYLQVPQDDWGGYPTGGKDGEIPCRRMRSGSYIKAMGDEESGDSDGSPKTSPKAV

ARRFTTRRSSSVDQARINCCVPPRIHPRSSIPGYSRSLTTGQLSDELNQQLEAVCGSVFG
ELESQAVDALDLPGCFRMRSHSYLRAIQAGCSQDDDCLPLLATPAAVSGRPGSSFNFRKA
PPPIPPGSQAPPRISITAQSSTDSAHESFTAAEGPARRCSSADGLDGPAMGARTLELAPV
PPRASPKPPTLIIKTIPGREELRSLARQRKWRPSIGVQVETISDSDTENRSRREFHSIGV
QVEEDKRRARFKRSNSVTAGVQADLELEGLAGLATVATEDKALQFGRSFQRHASEPQPGP
RAPTYSVFRTVHTQGQWAYREGYPLPYEPPATDGSPGPAPAPTPGPGAGRRDSWIERGSR
SLPDSGRASPCPRDGEWFIKMLRAEVEKLEHWCQQMEREAEDYELPEEILEKIRSAVGST
QLLLSQKVQQFFRLCQQSMDPTAFPVPTFQDLAGFWDLLQLSIEDVTLKFLELQQLKANS
WKLLEPKEEKKVPPPIPKKPLRGRGVPVKERSLDSVDRQRQEARKRLLAAKRAASFRHSS
ATESADSIEIYIPEAQTRLh

>Pongo abelii_DLGAP3(ENSPPYP_1806)
MRGYHGDRGSHPRPARFADQQHMDVGPAARAPYLLGSREAFSTEPRFCPRAGGHISPEPL
SLSEGPHTSHHHHHHHHHHHHQSRHGKRSKSKDRKGDGRHQAKSTGWWSSDDNLDSDSGF
LAGGRPPGEPGGPFCLEGPDGSYRDLSFKGRSGGSEGRCLACTGMSMSLDGQSVKRSAWH
TMMVSQGRDGYPGAGPGKGLLGPETKAKARTYHYLQVPQDDWGGYPTGGKDGEIPCRRMR
SGSYIKAMGDEESGDSDGSPKTSPKAVARRFTTRRSSSVDQARINCCVPPRIHPRSSIPG
YSRSLTTGQLSDELNQQLEAVCGSVFGELESQAVDALDLPGCFRMRSHSYLRAIQAGCSQ
DDDCLPLLATPAAVSGRPGSSFNFRKAPPPIPPGSQAPPRISITAQSSTDSAHESFTAAE
GPARRCSSADGLDGPAMGARTLELAPVPPRASPKPPTLIIKTIPGREELRSLARQRKWRP
SIGVQVETISDSDTENRSRREFHSIGVQVEEDKRRARFKRSNSVTAGVQADLELEGLAGL
ATVATEDKALQFGRSFQRHASEPQPGPRAPTYSVFRTVHTQGQWAYREGYPLPYEPPATD
GSPGPAPAPTPGPGAGRRDSWIERGSRSLPDSGRASPCPRDGEWFIKMLRAEVEKLEHWC
QQMEREAEDYELPEEILEKIRSAVGSTQLLLSQKVQQFFRLCQQSMDPTAFPVPTFQDLA
GFWDLLQLSIEDVTLKFLELQQLKANSWKLLEPKEEKKVPPPIPKKPLRGRGVPVKERSL
DSVDRQRQEARKRLLAAKRAASFRHS

>Mus musculus_DLGAP3(ENSMUSP_101700)
MRGYHGDRGSHPRPARFADQQHMDVGPAARAPYLLGSREAFSTEPRFCAPRAGLGHLSPE
GPLSLSEGPSSVGPEGGPGGVGAGGGSSTFPRMYPGQGPFDTCEDCVGHPQGKGATRLPP
TLLDQFEKQLPVQQDGFHTLPYQRGPAGPGPGPGSGAAPEARSESPSRIRHLVHSVQKLF
AKSHSLEAPGKRDYNGPKADGRGSSGGDSYSGPGSGGTPTSHHHHHHHHHHHHQSRHGKR
SKSKDRKGDGRHQTKATGWWSSDDNLDSDSGFLGGRPPGEPGGPFCLDAPDGSYRDLSFK
GRSGGSEGRCLACTGMSMSLDGQSVKRSAWHTMMVSQGRDGYPGAGPGKGLLGPETKAKA
RTYHYLQVPQDDWGGYPTGGKDGEIPCRRMRSGSYIKAMGDEESGDSDGSPKTSPKALAR
RFASRRSSSVDTARINCCVPPRIHPRSSIPGYSRSLTTGQLSEEFNQQLEAVCGSVFGEL
ESQAVDALDLPGCFRMRSHSYLRAIQAGCSQDDDCLPLLAAPASVSGRPGSSFNFRKAPP
PIPPGSQAPPRISITAQSSTDSAHESFTAAEGPARRCSSADGLDGPTMGARTLELAPVPP
RASPKPPTLIIKTIPGREELRSLARQRKWRPSIGVQVETISDSDTENRSRREFHSIGVQV
EEDKRRARFKRSNSVTAGVQADLELEGLAGLATVATEDKALQFGRSFQRHASEPQPGPRA
PTYSVFRTVHTQGQWAYREGYPLPYEPPATDGSPGPTPVPAPGPGSGRRDSWMERGSRSL
PDSGRTSPCPRDGEWFIKMLRAEVEKLEHWCQQMEREAEDYELPEEILEKIRSAVGSTQL
LLSQKVQQFFRLCQQSLDPTAFPVPTFQDLAGFWDLLQLSIEDVTLKFLELQQLKANSWK
LLEPKEEKKVPPPIPKKPSRGRGVPVKERSLDSVDRQRQEARKRLLAAKRAASFRHSSAT
ESADSIEIYIPEAQTRL

>Rattus norvegicus_DLGAP3(ENSRNOP_19214)
MRGYHGDRGSHPRPARFADQQHMDVGPAARAPYLLGSREAFSTEPRFCAPRAGLGHLSPE
GPLSLSEGPSSVGPEGGPGGVGAGGGSSTFPRMYPGQGPFDTCEDCVGHPQGKGATRLPP
TLLDQFEKQLPVQQDGFHTLPYQRGPAGPGPGPGSGAAPEARSESPSRIRHLVHSVQKLF
AKSHSLEAPGKRDYNGPKAEGRSSSGGDSYSGPGSGGPPTSHHHHHHHHHHHHQSRHGKR
SKSKDRKGDGRHQTKATGWWSSDDNLDSDSGFLGGRPPGEPGGPFCLDAPDGSYRDLSFK
GRSGGSEGRCLACTGMSMSLDGQSVKRSAWHTMMVSQGRDGYPGAGPGKGLLGPETKAKA
RTYHYLQVPQDDWGGYPTGGKDGEIPCRRMRSGSYIKAMGDEESGDSDGSPKTSPKALAR
RFASRRSSSVDTARINCCVPPRIHPRSSIPGYSRSLTTGQLSEEFNQQLEAVCGSVFGEL
ESQAVDALDLPGCFRMRSHSYLRAIQAGCSQDDDCLPLLAAPASVSGRPGSSFNFRKAPP
PIPPGSQAPPRISITAQSSTDSAHESFTAAEGPARRCSSADGLDGPTMGARTLELAPVPP
RASPKPPTLIIKTIPGREELRSLARQRKWRPSIGVQVETISDSDTENRSRREFHSIGVQV
EEDKRRARFKRSNSVTAGVQADLELEGLAGLATVATEDKALQFGRSFQRHASEPQPGPRA
PTYSVFRTVHTQGQWAYREGYPLPYEPPATDGSPGPPPVPAPGPGSGRRDSWMERGSRSL
PDSGRTSPCPRDGEWFIKMLRAEVEKLEHWCQQMEREAEDYELPEEILEKIRSAVGSTQL

LLSQKVQQFFRLCQQSLDLAGFWDLLQLSIEDVTLKFLELQQLKANSWKLLEPKEEKKVP
PPIPKKPSRGRGVPVKERSLDSVDRQRQEARKRLLAAKRAASFRHSSATESADSIEIYIP
EAQTRL

>Equus caballus_DLGAP3(ENSECAP_16420)
MRGYHGDRGSHPRPARFADQQHMDVGPAARAPYLLGSGEAFSTEPRFCAPRAGLGHLSPE
GPLSLSEGPSVGPEGGPGGAGVGGGSSTFPRMYPGQGPFDTCEDCVGHPQGKGAPRLPPT
LLDQFEKQLPVQQDGFHTLPYQRGPAGAGPGPGPESGTAPEARSESPSRIRHLVHSVQKL
FAKSHSLEAPGKRDYNGPKAEGRGGSGGDSFPGPGSGGPHTSHHHHHHHHHHHHQSRHGK
RSKSKDRKGDGRHQAKAAGWWSSDDNLDSDSGFLAGGRPPGEPGGPFCLEAPDGSYRDLS
FKGRSGGSEGRCLACTGMSMSLDGQSVKRSAWHTMMVSQGRDGYPGAGPGKGLLGPETKT
KARTYHYLQVPQDDWGGYPTGGKDGEIPCRRMRSGSYIKAMGDEESGDSDGSPKTSPKAV
ARRFTSRRSSSVDQARINCCVPPRIHPRSSIPGYTRSLTTGQLSEELNQQLEAVCGSVFG
ELESQAVDALDLPGCFRMRSHSYLRAIQAGCSQDDDCLPLLAAPASVSGRPGSSFNFRKA
PPPIPPGSQAPPRISITAQSSTDSAHESFTAAEGPARRCSSADGLDGPAMGARTLELAPV
PPRASPKPPTLIIKTIPGREELRSLARQRKWRPSIGVQVETISDSDTENRSRREFHSIGV
QVEEDKRRARFKRSNSVTAGVQADLELEGLAGLATVATEDKALQFGRSFQRHASEPQPGP
RAPTYSVFRTVHTQGQWAYREGYPLPYEPPATDGSPGPAPASTPGPGAGRRDSWMERGSR
SLPDSGRTSPCPRDGEWFIKMLRAEVEKLEHWCQQMEREAEDYELPEEILEKIRSAVGST
QLLLSQKVQQFFRLCQQSMDPTAFPVPTFQDLAGFWDLLQLSIEDVTLKFLELQQLKANS
WKLLEPKEEKKVPPPIPKKPSRGRGVPVKERSLDSVDRQRQEARKRLLAAKRAASFRHSS
ATESADSIEIYIPEAQTRL

>Loxodonta africana_DLGAP3(ENSLAFP_10976)
MRGYHGDRGSHPRPARFADQQHMDVGPAARAPYLLGSGEAFSTEPRFCAPRAGLGHLSPE
GPLSLSEGPSVGPEGGPGGAGVGGGSSTFPRMYPGQGPFDTCEDCVGHPQGKGAPRLPPT
LLDQFEKQLPVQQDGFHTLPYQRGPAVAGPGPGPGSGSAPEARSESPSRIRHLVHSVQKL
FAKSHSLEAPGKRDYNGPKAEGRGGSGGDSYPGPGSGGHHTSHHHHHHHHHHHHQSRHGK
RSKSKDRKGDGRHQAKAAGWWSSDDNLDSDSGFLVGGQPSGEPGGPFCLEAPDGSYRDLS
FKGRSGGSEGRCLACTGMSMSLDGQSVKRSAWHTMMVSQGRDGYPGAGPGKGLLGPETKA
KARTYHYLQVPQDDWGGYPTGGKDGEIPCRRMRSGSYIKAMGDEESGDSDGSPKTSPKAV
ARRFTSRRSSSVDQARINCCVPPRIHPRSSIPGYTRSLTTGQLSEELNQQLEAVCGSVFG
ELESQAVDALDLPGCFRMRSHSYLRAIQAGCSQDDDCLPLLAAPASVSGRPGSSFNFRKA
PPPIPPGSQAPPRISITAQSSTDSAHESFTAAEGPARRCSSADGLDGPAMGARTLELAPV
PPRASPKPPTLIIKTIPGREELRSLARQRKWRPSIGVQVETISDSDTENRSRREFHSIGV
QVEDDKRRARFKRSNSVTAGVQADLELEGLAGLATVATEDKALQFGRSFQRHASEPQPGP
RAPTYSVFRTVHTQGQWAYREGYPLPYEPPATDGAPGPAPTPTPGPGAGRRDSWIERGSR
SLPDSGRTSPCPRDGEWFIKMLRVEVEKLEHWCQQMEREAEDYELPEEILEKIRSAVGST
QLLLSQKVQQFFRLCQQSMDPTAFPVPTFQDLAGFWDLLQLSIEDVTLKFLELQQLKANS
WKLLEPKEEKKVPPPIPKKPSRGRGVPVKERSLDSVDRQRQEARKRLLAAKRAASFRHSS
ATESADSIEIYIPEAQTRL

>Myotis lucifugus_DLGAP3(ENSMLUP_12457)
MRGYHGDRGSHPRPTRFADQQHMDVGPAARAPYLLGSGEAFSTEPRFCAPRAGLGHLSPE
GPLSLSEGPSVGPEGGPGGTGIGGGSSTFPRMYPGQGPFDTCEDCVGHPQGKGAPRLPPT
LLDQFEKQLPVQQDGFHTLPYQRGPAGAGPGPGPGSGSAPEARSESPSRIRHLVHSVQKL
FAKSHSLEAPGKRDYNGPKADGRGGSGGDSYTGPGSGGPHTSHHHHHHHHHHHHQSRHGK
RSKSKDRKGDGRHQAKAGGWWSSDDNLDSDSGFLAGGRPPGEPGGPFCLEAPDGSYRDLS
FKGRSGGSEGRCLACTGMSMSLDGQSVKRNAWHTMMVSQGRDGYPGAGPGKGLLGPEAKA
KTRTYHYLQVPQDDWGGYPTGGKDGEIPCRRMRSGSYIKAMGDEESGDSDGSPKTSPKAL
ARRFASRRSSSVDQARINCCVPPRIHPRSSIPGYSRSLTTGQFSSHQISQWLAGPCGIGF
GVLLSLSCLNLDLPGCFRMREPQLTPGPSRPGCSQRRRLPAPSFAGPCLCLRGGPGSLLH
FKIPRPHPPGNPAPPAFLPGPEHHRYSLENFTPANSPPTLQSADGLDGPAMGARTLELAP
VPPRASPKPPTLIIKTIPGREELRSLARQRKWRPSIGVQVETISDSDTENRSRREFHSIG
VQVEEDKRRARFKRSNSVTAGVQADLELEGLAGLASVATEDKALQFGRSFQRHASEPQPG
PRAPTYSVFRTVHTQGQWAYREGYPLPYEPPATDGSPGPAPAPTPGPGAGRRDSWMERGS
RSLPDSGRTSPCPRDGEWFIKMLRAEVEKLEHWCQQMEREAEDYELPEEILEKIRSAVGS
TQLLLSQKVQQFFRLCQQSMDPTAFPVPTFQDLAGFWDLLQLSIEDVTLKFLELQQLKAN
SWKLLEPKEEKKVPPPIPKKPSRGRGVPVKERSLDSVDRQRQEARKRLLAAKRAASFRHS
SATESADSIEIYIPEAQTRL

>Monodelphis domestica_DLGAP3(ENSMODP_29570)
MRGYHGDRGSQPRPARLPDSPMDYGPSAPGSRPPYLLGPGDPYPTDPRYCPSRAGPGHLS
TDGPLSLSEPPSAGPDGGLGPGPGPGGAGSSTFPRMFPGQAPFDGCEDCVGHPPGKVAAR
LPPTLLDQFEKQLPLQQDGFHTLPYPRGVAGGSGGPGPGPGAGGAAPEPRSESPSRIRHL
VHSVQKLFAKSHSLEAPGKRDYNGPKAESRPGAGSGSEGYAGHHSPHGPPHHHHHHHHHH
HHHHQSRHGKRSKSKDRKANGRHTPKSMGWWSSDDNLDSDSGFLAGGRPPGDAGAPFCLE
APDGTFRDLSFKGLPGGPEGRCLACTGMSMSLDGQTVKRSAWHTMMVSQGRDGFQGSGPS
KGLLGPETKAKDRSYHYLQVPHDEWGGYPTGGKDGEIPCRRMRSGSYIKAMGDEDSGDSD
GSPKMSPKGAARRYAYRRSSSVDQARINCCVPPRIHPRTGAPGYGRSLTTGQLSEELSQQ
FEAVCGSVFGEPESQAVDALDLPGCFRMRSHSYLRAIQAGCSQDDDCLPLLTAPMGAGPS
SVLKTSTSFNFRKAPPPIPPGRQTPPRISITAQSSTDSAHESYPPPEGPAWAPEPTRRCS
SADGLDGPTTGTRTLELAPVPARASPKPPTLIIKTIPGREELRSLARQRKWRPSIGVQVE
TISDSDTETRSRREFHSIGVQVEEDKRRARFKRSNSVTAGVQADLELEGLAGLATVATED
KALQFGRSFQRHASEPQPGPRGPAYSVFRTVHTQGQWAYREGYPLPYEPPAPDGPPGSGP
APAPTPAPTHGRRDSWMERGSRSLPDSGRASPCPRDGEWFIKMLQAEVEKMEHWCQQMER
EAEDYDLPEEILEKIRSAVGSAQLLMSQKVQQFFRLCQQSMDPASFPVPTSQDLAGFWDL
LQLAIEDVSLKFLELQQLKANSWKPLEPEEEKKVPPPIPKKPSRGRGVPVKDRSLDSVDR
QRQEARKRLLAAKRAASFRHSSATESADSIEIYIPEAQTRL

>Gallus gallus_DLGAP3(ENSGALP_3970)
MKGYHGERSQAQHSSGHRCRCIPEDCEHPSDYIPHGPDGRPPYLLSPSEPCSLEHPFCPV
RSPSVAGECGPLSDPPSGSASSTFPRMHHAQQQQPYDSCDECMATAHPSSKINRLPPTLL
DQFEKQLPLHHDGFHTLQYPRAGGTEPRSESPSRIRHLVHSVQKLFAKSHSLEAPAKRDY
NGTKMDGRGDGYHHHHHHHHHHHHQSRHGKRSKSKDRKVDSRHRSKMMGWWSSDDNLDSD
SSYMVSGRHAVDQGTQYCVDAPESAFRDLTLKSLKSGGEGKCLACAGMSMSLDGQTVKRS
AWHTMTVSQAREAYPSASGTEKTLMLQETKAKDRAYQYLQVPQDEWSGYPAVGKDGEIPC
RRMRSGSYIKAMGDEDSADSDASAKVSPRAATRRDSYRRSSSADQARSKFASRHYSDSYI
CNCPSCGVPPRMLPRGQGYGRSFTTGQINDELNHQFEAVCESVFGEVESQAVEALDLPGC
FRMRSHSYLRAIQAGCSQDDDCLSLFSMSAPAGPPITSSILKPGTSFSYRKAPPPIPPGT
KAKPLISVTAQSSTESAHESYLPSEVTRGPAWSKDAAARCNSAESLESSKVTAVSLELPP
VQPRTAPKPSTLIIKAIPGREELRSLARQRKWRPSIGVQVEAVSDSDTESRSQREFHSIG
VQVEEDKRRARFKRSNSVTAGVQADLELEGFAGLAVATEDKALQFGRPFQRHSSEPESGR
QYAVYKTVHTQGQWAYREDYQMQYDAVEVPRRDAWVERGSRSLPDSGRASPCHRDGEWFI
KLLQAEVEKMEGWCQQMEREAEDYDLPEEILEKIRSAVGSAQLLMSQKVQQFYRLCQQNM
DPNAFPVPTFQDLAGFWDLLQLSIEDVSMKFAELQQLKANGWKIIEPKEEKKVPPPIPKK
PPRSKVHPVKERSLDSVDRQRQEARKRLLAAKRAASFRQNSATESADSIEIYIPEAQTRL

>Anolis carolinensis_DLGAP3(ENSACAP_13190)
MRSYHGERSQSREHPCRCVPEACDQPMEYVHHSQESRQPYLLSPSEPCSLDHYCPSRSSA
EAPLSLTEPPSAASSSSSTFPRMHHAPQPYDSCDECMAAAHPSSKINRLPPTLLDQFEKQ
QLPLHHDGFHTLQYQRASSTEQPRSESPSRIRHLVHSVQKLFAKSHSLEAPSKREFNGTK
ISHHHHRQSRHGKRSKSKDRKMDSSSSSRHRNKMMGWWSSDDNLDSDSSYMVSGRQHTVD
QATQFCVDAPESAFRDLTLKSLKSGGGEGKCLACAGMSMSLDGQTVKRSSWHTMTVSQAR
EAYPSSGGGPEKPLMLQEAKAKERSYHYLQVRRSSSWTSHAPLSRGSKIRCLGLYSGQLI
LEYIRSPPHRPRDASPSPEPGGMPELSSFLKSSFHCIFFPRTLCSAEDHVPKLVTESGFS
SMKEQWELSISHMAETLCPRNVNCCTSPRIRPRGQGYGRSFTTGQINDELNHQFEAVCES
VFGEVESQAVEALDLPGCFRMRSHSYLRAIQAGCSQDDDCLSLNTQSPSMIQGVMVQLVH
NAGCTFSYRKAPPALPSGIKAKPLIAVTTQSSSESAHDSFLPREVSRSPSSWSKEAAKRC
NSTESLESTKITAMSLDLPSLQARTAPKPSTLIIRTIPGREELRSLARQRKWRPSIGVQV
ETISDSDTESRSHREVHSIGVQVEEDKRRARFKRSNSVTAGVQADLELEGLAGLAVATED
KALQFGRSFQRHSSEPESTRQYSVYKTVHTQGQWAYREDYQMQYDTMEVPRQDSWMDRGS
RSLPDSGRASPCHRDGEWFIKLLQAEVEKMEGWCQQMELEAEDYDLPEEILEKIRSAVGS
AQLLMNQKFQQFYRLCQQNMDPNAFPMPTFQDLAGFWDLLQLSIEDVSIKFAELHQLKAN
GWKPIEPKLSFPQEDKKVPPPIPKKPPRSRLHPVKDRSLDSVDRQRQEARKRLLAAKRAA
SFRQSSATESADSIEIYIPEAQTRL

>Danio rerio_DLGAP3(ENSDARP_93925)
MKGYQVSRSLSQHSSGPCYCMPEDCDTARDYMHHPDHYQGHNDHHYYPPGGPPEPLAMVP
SGGGTFPRSHPSQPQHFDSTCEECLSSAGHGHGHGGKMHRIPPNLLDQFEKQLPFHPDGF
HTLQYQRTASGEQRSESPSRIRHLVNSVQRLFAKSHSLEAPSKREFNGTRGGEYRERERG
HRSGGEESGGHHYSGTHQSRSARRNKSRERSKSGDSRHESRSSRHRSKTAGWWSSDDNLD

SDSSFLVGAGRGVRGYPTGHETLDAAIQELTLRKSKGPPPGECLACTNIAMTGDSGHSLK
RSTWSSMTVSQAREVYPSSRGGGGGPGGGGGGYEKALVPLDPKMKERSLHHLQVPSDDWC
GGGPSDSGGEIPCRRMRSGSYIKAMGDDDSVDSDTSPKASPKSALIAQREAFRRSVSMDQ
RSSTTSKYSCKQCTDAYPNSRTTPKGHARSRSYTRSLTSAQLGDTLNLFGEMESQAVEAL
DLPGCFRTRSHSYVRAIQAGCSQDDDCLSVFSMSVPQASMKGEAVFPYRKGPPPLPPRMS
KPMISVTAQSSTESTQDAYFQSTGQPALVRPRQHSNSVDLTENSGGRTSRGGHYLGGGTA
RVRQSSNSAESLNGRALQSIAYHSGPGPMKPKHSSSADNLLEGPRGSRDRVGGSLGKSAS
LPQNSMSLAKALTVGEEFKQEGRGRKWRPSIAVQVDSSETLSDSDPEGKALREVHSIGVQ
VEDDKRRARFKRSNSVTASVQADLETEGFGGLTVPTQDKGLQFGCSFQRHSSEPESASQF
AEYRTVHTQGQWAYREDYHSGYTTEPGPPELRALPRSHSPLPLAPERGVGGWSEGPRSLP
DSGRASPCLRDGEFFLRLLQTEVERMEGWCQNMEREAETNELPEEVLELIRGAVGSAQML
MSQRVQQFFRMCQQSVDPSSYPQPTTQDLASFWDLLQLAIEDVRVKFQDLQRLKDSGWRL
PPEKKEDKKVPPPLPKKPIGGVTGSLRADSTAESGGMLVVSRGRSLPEREKSLDVGDRQK
LETRRRLLQTKRSASFRQNSATESADSIEIYITEAQTRL

>Takifugu rubripes_DLGAP3a(ENSTRUP_38091)
GGGTFPRSHPSQHPSLHSFDSCDECLPSGHSGKMHRIPPNMTDQFEKQVPFHPDGFHTLQ
YQRSTSGGAEQRSESPSRIRHLVNSVQRLFAKSHSLEAPSKREYNGTRGSSDYRSERGGG
HRSGGEDGGGHYSGHQPRSTRRNKSRERSKSGDSRHESGRRHRSRTAGWWSSDDNLDSDS
SFLVGGGRRGYPSGHESLDAAIQELTMKRPKERMAAPGPGDCVACTTMALAGNEGGGHHG
HHGHSLKRSTWSAMTVSQAREVYPSTRGGGGYDKALVPVDPKMKERTFHYLQVPSEDWAG
GYGGGDAVDSGGEIPCRRMRSGSYIKAMGDDDSVDSDTSPKASPKSTLIAQRDAFKRSAS
VDQRLNARTTPKSHTRSRSYTRSLTSTQLGDTLNRQFESVCETMFGEVESQAVEALDLPG
VFRTRSHSYVRAIQAGCSQDDDCLSVFSMSGPQGSIKSGAVFPYRKGAPPPLPPRMSKSS
LSVRAQSSTESTQDAYFLLEGPTRPARERDGRVGGSLGKSVSLPQNSMVLSKAGGHDDVR
GGRKLRPSIAVQVDSSETLSDSDAEGKALTEVHSIGVQVEDDKRRARFKRSNSVTASVQA
DLGPEGFPGLSIAVPMQDKSLQFGCSFQRHSSEPESASQYTDCHRTVHTQGQWAYRETDL
RRQHPHLPPRSHSPLPITSERAWAGTPSLEGPRSLPDSGRASPCMRDGEFFLRLLQTEVE
RMEGWCQNMEREAEENDLPEEILEPIRSAVGGAQMLMSQKVQQFFRLCQQSLDPSAYPQP
TSQDLANFWDLLQLNIEDVRIKFQDLQRVKESGWRLPPEKKEDKKLPPPLPKKPAGGGHT
LPIREKSLDLGDRQRTEARRRLLQTKRAASFRQNSATESADSIEIYIPEAQTRL

>Takifugu rubripes_DLGAP3b(ENSTRUP_38088)
HSGGGTFPRSHPSQHPSLHSFDSCDECLPSGHSGKMHRIPPNMTDQFEKQVPFHPDGFHT
LQYQRSTSGGAEQRSESPSRIRHLVNSVQRLFAKSHSLEAPSKREYNGTRGSSDYRSERG
HYSGHQPRSTRRNKSRERSKSGDSRHESGRRHRSRTAGWWSSDDNLDSDSSFLVGGGRRG
YPSGHESLDAAIQELTMKRPKERMAAPGPGDCVACTTMALAGNEGGGHHGHHGHSLKRST
WSAMTVSQAREVYPSTRGGGGYDKALVPVDPKMKERTFHYLQVPSEDWAGGYGGGDAVDS
GGEIPCRRMRSGSYIKAMGDDDSVDSDTSPKASPKSTLIAQRDAFKRSASVDQRLNATNS
GSRSVHRRIPNSRTTPKSHTRSRSYTRSLTSTQFVSPVTQLGDTLNRQFESVCETMFGEV
ESQAVEALDLPGVFRTRSHSYVRAIQAGCSQDDDCLSVFSMSGPQGSIKSGAVFPYRKGA
PPPLPPRMSKSSLSVRAQSSTESTQDAYFQNSGQLASGPGRTKQHSNSVDLGGSEGPSGR
SSRGGYYTAVGPGRSRQYSNSAESLDGNSMVLSKAGGHDDVRGGRKLRPSIAVQVRHEHR
ANDVDSSETLSDSDAEGKALTEVHSIGVQVEDDKRRARFKRSNSVTASVQADLGPEGFPG
LSIAVPMQDKSLQFGCSFQRHSSEPESASQYTDCHRTVHTQGQWAYREVFHKTKADLKMT
HQSTPSLEGPRSLPDSGRASPCMRDGEFFLRLLQTEVERMEGWCQNMEREAEENDLPEEI
LEPIRSAVGGAQMLMSQKVQQFFRLCQQSLDPSAYPQPTSQDLANFWDLLQLNIEDVRIK
FQDLQRVKESGWRLPPEKKEDKKLPPPLPKKPAGGTLPIREKSLDLGDRQRTEARRRLLQ
TKRAASFRQNSATESADSIEIYIPEAQTRL

>Tetraodon nigroviridis_DLGAP3(ENSTNIP_12089)
GGGTFPRSHPSQHPSLHSFDSCEECLPPGHSGKMHRMPPNMTDQFEKQVPFHPDGFHTLQ
YQRSTSGGAEQRSESPSRIRHLVNSVQRLFAKSHSLEAPSKREYNGTRGSGDYRSERGGG
HRSGGEDGGGHYSGHQPRSTRRNKSRERSKSGDSRHEAGRRHRSRTAGWWSSDDNLDSDS
SFLVGGGRRGYPSGHESLDAAIQELTMKRPKDRMVAPGPGECVACTTMALAGTEGGGHHG
HHGHSLKRSTWSAMTVSQAREVYPSTRGGGGYDKALVPVDPKMKERTFHYLQVPSEDWGG
GYGGGDSVDSGGEIPCRRMRSGSYIKAMGDDDSADSDTSPKASPKSTLIAQRDAFKRSAS
MDQRQCTDAYPNSRTTPKTHTRSRSYTRSLTSTQLGDTLNRQFESVCETMFGEVESQAVE
ALDLPGVFRTRSHSYVRAIQAGCSQDDDCLSVFSMSGPQGSIKSGAVFPYRKGAPPPLPP
RMSKSSLSVRAQSSTESTQDAYFQALGAPSHTATRWIWEGLKAPPVAPREEGTTPPRALD
APNSMALSKTGGHDDARGGRKLRPSIAVQVDSSETLSDSDAEGKALTEVHSIGVQVEDDK

RRARFKRSNSVTASVQADLGPEGFPGLSIAVPTQDKSLQFGCSFQRHSSEPESASQYTEC
HRTVHTQGQWAYRESGYTTETDLRRHQHPHLPPRSHSPLPITSERAWAGTPSLEGPRSLP
DSGRASPCMRDGEFFLRLLQTEVERMEGWCQNMEREAEENDLPEEILEPIRSAVGSAQML
MSQKVQQFFRLCQQSLDPAAYPQPSSQDLANFWDLLQLNIEDVRIKFQDLQRMKDSGWRL
PPEKKEDKKLPPPLPKKPAGGHTIPIREKSLDLGDRQRTEARRRLLQTKRAASFRQNSAT
ESADSIEIYIPEAQTRL

>Gasterosteus aculeatus_DLGAP3(ENSGACP_9867)
GGGTFPRSHPNQHPTHQSLDSCEECLSSVHGGKMHRIPPNVVEHFDKQVPFHPDGFHTLQ
YQRSASGGAEPRSESPSRIRHLVNSVQRLFAKSHSLEAPSKREYNGTSGGHRSGGEDGGG
GHYSGHQPRSTRRSKSRERSKSGDSRHESGRRHRSRTAGWWSSDDNLDSDSSFLVSGGRR
GYPSGHESLDAAIQELTMKRPKERGGGGPVPGECMACTTMALAGSEGGGSQGHLGHSLKR
STWSAMTVSQAREVYPSTRGASYEKALVPLESKLKERTFHYLQVPSDDWGGGYGGGDGGG
EIPCRRMRSGSYIKAMGDDDSVDSDTSPKTSPKSTLVAQRDAFRRSISMDQSRTTPKTQT
RSRSYTRSLTSSQLGDTLNRQFEAVCETMFGEVESQAVEALDLPGVFRTRSHSYVRAIQA
GCSQDDDCLSVFSMSGPQGSIKGGAGEVFPYRKGAPPPLPPRMSKSSLSVRAQSSTESNQ
DAYYQSAGQAKHSSSADSLLEGPPRPARERDGRVAGSLGKSVSLPQNSIVLSKAGGQDGR
KWRPSIAVQVDSSETLSDSDAEGKALTEVHSIGVQVEDDKRRARFKRSNSVTASVQADLD
PEGFPGLSIAVPTQDKSLQFGCSFQRHSSEPESASQYTDCHRTVHTQGQWAYRESGYTTQ
ACQADPRPHQHPHLPPRSHSPLPITSERAWAGTPSLEGPRSLPDSGRASPCMRDGEFFLR
LLQTEVERMEGWCQNMEREAEENELPEEILELIRNAVGSAQMLMSQKVQQFFRLCQQSVD
PSAYPQPTSQDLAGFWDLLQLNIEDVRIKFQDLQRLKDSGWRLPPEKKDKKLPPPLPKKS
AGGGHTLPIREKSLDLGDRQRTEARRRLLQTKRTASFRQNSATESADSIEIYIPEAQTRL

>Homo sapiens_DLGAP4(ENSP_384954)
MKGLGDSRPRHLSDSLDPPHEPLFAGTDRNPYLLSPTEAFAREARFPGQNTLPGDGLFPL
NNQLPPPSSTFPRIHYNSHFEVPEESPFPSHAQATKINRLPANLLDQFEKQLPIHRDGFS
TLQFPRGEAKARGESPGRIRHLVHSVQRLFFTKAPSLEGTAGKVGGNGSKKGGMEDGKGR
RAKSKERAKAGEPKRRSRSNISGWWSSDDNLDGEAGAFRSSGPASGLMTLGRQAERSQPR
YFMHAYNTISGHMLKTTKNNTTELTAPPPPPAPPATCPSLGVGTDTNYVKRGSWSTLTLS
HAHEVCQKTSATLDKSLLKSKSCHQGLAYHYLQVPGGGGEWSTTLLSPRETDAAAEGPIP
CRRMRSGSYIKAMGDEDSDESGGSPKPSPKTAARRQSYLRATQQSLGEQSNPRRSLDRLD
SVDMLLPSKCPSWEEDYTPVSDSLNDSSCISQIFGQASLIPQLFGHEQQVREAELSDQYE
AACESACSEAESTAAETLDLPLPSYFRSRSHSYLRAIQAGCSQEEDSVSLQSLSPPPSTG
SLSNSRTLPSSSCLVAYKKTPPPVPPRTTSKPFISVTVQSSTESAQDTYLDSQDHKSEVT
SQSGLSNSSDSLDSSTRPPSVTRGGVAPAPEAPEPPPKHAALKSEQGTLTSSESHPEAAP
KRKLSSIGIQERTRRNGSHLSEDNGPKAIDVMAPSSESSVPSHSMSSRRDTDSDTQDAND
SSCKSSERSLPDCTPHPNSISIDAGPRQAPKIAQIKRNLSYGDNSDPALEASSLPPPDPW
LETSSSSPAEPAQPGACRRDGYWFLKLLQAETERLEGWCCQMDKETKENNLSEEVLGKVL
SAVGSAQLLMSQKFQQFRGLCEQNLNPDANPRPTAQDLAGFWDLLQLSIEDISMKFDELY
HLKANSWQLVETPEKRKEEKKPPPPVPKKPAKSKPAVSRDKASDASDKQRQEARKRLLAA
KRAASVRQNSATESADSIEIYVPEAQTRL

>Pan troglodytes_DLGAP4(ENSPTRP_46919)
MKGLGDSRPRHLSDSLDPPHEPLFAGTDRNPYLLSPTEAFAREARFPGQNTLPGDGLFPL
NNQLPPPSSTFPRIHYNSHFEVPEESPFPSHAQATKINRLPANLLDQFEKQLPIHRDGFS
TLQFPRGEAKARGESPGRIRHLVHSVQRLFFTKAPSLEGTAGKVGGNGSKKGGMEDGKGR
RAKSKERAKAGEPKRRSRSNISGWWSSDDNLDGEAGAFRSSGPASGLMTLGRQAERSQPR
YFMHAYNTISGHMLKTTKNNTTELTAPPPPPAPPATCPSLGVGTDTNYVKRGSWSTLTLS
HAHEVCQKTSATLDKSLLKSKSCHQGLAYHYLQVPGGGGEWSTTLLSPRETDAAAEGPIP
CRRMRSGSYIKAMGDEDSDESGGSPKPSPKTAARRQSYLRATQQSLGEQSNPRRSLDRLD
SVDMLLPSKCPSWEEDYTPVSDSLNDSSCISQIFGQASLIPQLFGHEQQVREAELSDQYE
AACESACSEAESTAAETLDLPLPSYFRSRSHSYLRAIQAGCSQEEDSVSLQSLSPPPSTG
SLSNSRTLPSSSCLVAYKKTPPPVPPRTTSKPFISVTVQSSTESAQDTYLDSQDHKSEVT
SQSGLSNSSDSLDSSTRPPSVTRGGVATAPEAPEPPPKHAALKSEQGTLTSSESHPEATP
KRKLSSIGIQVDCIQPVPKEEPSPATKFQSIGVQVEDDWRSSVPSHSMSSRRDTDSDTQD
ANDSSCKSSERSLPDCTPHPNSISIDAGPRQAPKIAQIKRNLSYGDNSDPALEASSLPPP
DPWLETSSSSPAEPAQPGACRRDGYWFLKLLQAETERLEGWCCQMDKETKENNLSEEVLG
KVLSAVGSAQLLMSQKFQQFRGLCEQNLNPDANPRPTAQDLAGFWDLLQLSIEDISMKFD
ELYHLKANSWQLVETPEKRKEEKKPPPPVPKKPAKSKPAVSRDKASDASDKQRQEARKRL
LAAKRAASVRQNSATESADSIEIYVPEAQTRL

>Mus musculus_DLGAP4(ENSMUSP_105195)
MKGLGDSRPRHLSDSLDPPHEPLFAGPDRNPYLLSPTEAFAREARFPGQNTLPGDGLFPL
NNQLPPPSSTFPRIHYNSHFEVPEESPFPSHAQATKINRLPANLLDQFEKQLPIHRDGFS
TLQFPRGEAKARGESPGRIRHLVHSVQRLFFTKAPSMEGTAGKVGGNGSKKGGLEDGKGR
RAKSKERAKAGEPKRRSRSNISGWWSSDDNLDGEGGAFRSGPASGLMTLGRQQERTQPRY
FMHAYNTISGHMLKTTKNTTTELTAPPPPPAPPATCPSLGVGTDTNYVKRGSWSTLTLSH
AHEVCQKTSATLDKSLLKSKSCHQGLAYHYLQVPGGGGEWSTTLLSPRDMDSTAEGPIPC
RRMRSGSYIKAMGDEDSDESGGGSPKPSPKTAARRQSYLRATQQSLGEQSNPRRSLDRLD
SVDMLLPSKCPSWEDDYNPISDSLNDSSCISQVFGQASLIPQLFGHDQQVREADLSDQYE
AACESACSEAESTTAEALDLPLPSYFRSRSHSYLRAIQAGCSQEEDSVSLQSLSPPPSTG
SLSNSRTLPSSSCLVAYKKTPPPVPPRTTSKPFISVTVQSSTESAQDTYLDSQDHKSEVT
SQSGLSNSSDSLDSSTRPPSVTRGGITPGPEAPEPPPKHAALKSEQGTLTSSESHSEAIP
KRKLSSIGIQERTRRSGSHLSEDNGPKAIDVMAPSSESSAPSHSMSSRRDTDSDTQDAND
SSCKSSERSLPDCTSHPNSISIDAGPRQAPKIAQIKRNLSYGDNSDPALEASSLPPPDPW
LETSSSSPAEPAQPGACRRDGYWFLKLLQAETERLEGWCCQMDKETKENNLSEEVLGKVL
SAVGSAQLLMSQKFQQFRGLCEQNLNPDANPRPTAQDLAGFWDLLQLSIEDISMKFDELY
HLKANSWQLVETPEKRKEEKKPPPPVPKKPAKSKAAVSRDKASDAGDKQRQEARKRLLAA
KRAASVRQNSATESADSIEIYVPEAQTRL

>Rattus norvegicus_DLGAP4(ENSRNOP_34166)
MKGLGDSRPRHLSDSLDPPHEPLFAGPDRNPYLLSPTEAFAREARFPGQNTLPGDGLFPL
NNQLPPPSSTFPRIHYNSHFEVPEESPFPSHAQATKINRLPANLLDQFEKQLPIHRDGFS
TLQFPRGEAKARGESPGRIRHLVHSVQRLFFTKAPSMEGTTGKVGGNGGKKGVLEDGKGR
RAKSKERAKAGEPKRRSRSNISGWWSSDDNLDGEGGAFRSGPASGLMTLGRQPERTQPRY
FMHAYNTISGHMLKTTKNTTTELTAPPPPPAPPATCPSLGVGTDTNYVKRGSWSTLTLSH
AHEVCQKTSATLDKSLLKSKSCHQGLAYHYLQVPGGGGEWSTTLLSPRDMDSTAEGPIPC
RRMRSGSYIKAMGDEDSDESGGGSPKPSPKTAARRQSYLRATQQSLGEQSNPRRSLDRLD
SVDMPLPSKYPSWEEDYNPISDSLNDSGCISQVFGQASLIPQLFGHDQQVREADLSDQYE
AACESACSEAESTAAEALDLSLPSYFRSRSHSYLRAIQAGCSQEEDSVSLQSLSPPPSTG
SLSNSRTLPSSSCLVAYKKTPPPVPPRTTSKPFISVTVQSSTESAQDTYLDSQDHKSEVT
SQSGLSNSSDSLDSSTRPPSVTRGGITPGPEAPEPPPKHAALKSEHGTLTSSESHSEAVP
KRKLSSIGIQVDCIQPVPKEEPSPATKFQSIGVQVEDDWRSSAPSHSMSSRRDTDSDTQD
ANDSSCKSSERSLPDCTSHPNSISIDAGPRQAPKIAQIKRNLSYGDNSDPALEASSLPPP
DPWLETSSSSPAEPAQPGACRRDGYWFLKLLQAETERLEGWCCQMDKETKENNLSEEVLG
KVLSAVGSAQLLMSQKFQQFRGLCEQNLNPDANPRPTAQDLAGFWDLLQLSIEDISMKFD
ELYHLKANSWQLVETPEKRKEEKKPPPPVPKKPAKSKAAVSRDKASDAGDKQRQEARKRL
LAAKRAASVRQNSATESADSIEIYVPEAQTRL

>Canis familiaris_DLGAP4(ENSCAFP_12515)
MKGLGDSRPRHLSDSLDPPHEPLFAGPDRNPYLLSPTEAFAREARFPGQNALPGDGLFPL
NNQLPPPSSTFPRIHYNSHFEVPEESPFPSHAQATKINRLPANLLDQFEKQLPIHRDGFS
TLQFPRGEAKARGESPGRIRHLVHSVQRLFFTKAPSLEGTAGKVSGNGSKKGGLEDGKGR
RAKSKERAKAGEPKRRSRSNISGWWSSDDNLDGEGGAFRSSGPASGLMTLGRQAERSQPR
YFMHAYNTISGHMLKAAKSNAAELTAPPPPPAPPAACPSLGVGTDTNYVKRGSWSTLTLS
HAHEVCQKTSATLDKSLLKSKSCHQGLAYHYLQVPGGGEWSTALLSPREADAAAEGPIPC
RRMRSGSYIKAMGDEDSDESGGSPKPSPKTAARRQSYLRATQQSLGEQSNPRRSLDRLDS
VDMLLPSKCPSWEEDYNPVSDSLNDSSCISQIFGQASLIPQLFGHEQQVREADLSDQYEA
ACESACSEAESAAAEALDLPLPSYFRSRSHSYLRAIQAGCSQEEDSVSLQSLSPPPSTGS
LSNSRTLPSSSCLVAYKKTPPPVPPRTTSKPFISVTVQSSTESAQDTYLDSQDHKSEVTS
QSGLSNSSDSLDSSTRPPSVTRGGVTPAPEAPEPPPKHAALKSEQGTLTSSESHPEAIPK
RKLSSIGIQVDCIQPVPKEEPSPATKFQSIGVQVEDDWRSSAPSHSMSSRRDTDSDTQDA
NDSSCKSSERSLPDCTPHPNSISIDAGPRQAPKIAQIKRNLSYGDNSDPALEASSLPPPD
PWLETTSSSPAEPAQPGACRRDGYWFLKLLQAETERLEGWCCQMDKETKENNLSEEVLGK
VLSAVGSAQLLMSQKFQQFRGLCEQNLNPEANPRPTAQDLAGFWDLLQLSIEDISMKFDE
LYHLKANSWQLVETPEKRKEEKKPPPPVPKKPAKSKPAVSRDKASDAGDKQRQEARKRLL
AAKRAASVRQNSATESADSIEIYVPEAQTRL

>Monodelphis domestica_DLGAP4(ENSMODP_1726)
MKGLSGDSRGRHLSDSLEPPPPDALFPGSDRNPYLLSPTATVTADPFSRESGFPGASTGV
AGDTLFPLPSPLPTSGTFPRLHYNSHFEVPEEGPFPSHAQATKINRLPANLLDQFEKQLP

VHRDGFSTLQFHRAEPRPRGESPGRIRHLVHSVQKLFFAKAQSLEGGGLKGSGNGRKGGA
GGGETGKGRRAKGKDSGKTAEPKRRSRSNISGWWSSDDNLDSGNSGDCGYRPSAGLMTLG
RQPERAQPRYFVYNTFSEHMLKAAKNNSELPRAGAGGGGGAPPASLPASTEPSYGKRGSW
STLTLSHAREVCQKASATIDKALLKSKSCHQDLAYHYLQVPPSEWNSSLSRTGGDSEIPC
RRMRSGSYIKAMGDEDSEDSDTSPKPSPKTAARRQSYLKATQQSLSEQSTTHRSLDRLDS
VDMLLPSKFPSWEEEYNPIGNSITESSCINQIFGQASFVPQIFSHEEQVREAELSDQYEA
ACESAYSEAESATADVLDLPLPSYFRSRSHSYLRAIQAGCSQDEDSISLQSLSPPPTGSS
LSSSRTLPSSSCLVAYKKTPPPVPPRTTSKPFISVTVQSSTESAQDTYLDSQDHKSEVNS
QSGLSNSSDSLDSTRPPSVTQGSSGGTQAREAPELPQKNATLKNDKGTLTSEEPKAETAP
KRKLSSIGIQVDCIQPVPKEEPPPATKFQSIGVQVEDEWRYSGASHSMSSKRDTDSDTQE
ANDSSCKSSERSLSDCPPHHNSINIDTNPRPSVKASQIKRNLSYGDNSDPALETSSLPPP
DPWLETSSSSPLEPSQPGACRRDGYWFLKLLQAETERLEGWCCQMDKEAKENNLSEEVLG
KVLSAVGSAQLLMSQKFQQFRGLCDQNLNPDSNPRPTAQDLAGFWDLLQLSIEDISMKFD
ELYHLKANSWQLADPPEKKALLSQEAKMSSKRAKAKTTKKRPQRATSNVFAMFDQSQIQE
FKEAFNMIDQNRDGFIDKEDLHDMLASLGKNPTDEYLEGMMSEAPGPINFTMFLTMFGEK
LNGTDPEDVIRNAFACFDEEASGVIHEDHLRELLTTMGDRFTDEEVDEMYREAPIDKKGN
FNYVEFTRILKHGAKDKDD

>Gallus gallus_DLGAP4(ENSGALP_1565)
MKGLGDNRSRHLSDNLDSPQDSLYAPQDRNPYLLSPTDSFTMDPRFPAQNALHGDCLLPL
NNQISNSSTFPRIHYNSHFEVPDENPFPSHAQATKINRLPANLLDQFEKQLPIHRDGFST
LQFQRSDAKHRSESPGRIRHLVHSVQKLFAKSQSLEGPTKGNVNGKKGADDPKQNRRSKS
KDRAKTSEPKRRTRSNISGWWSSDDNLDSDTCSYRNPMGLMTLGRQPDHSQPKYFMHAYN
TISEHMLKSSKSNNDLKVTPCTNIPINNDSNYIKRGSWSTLTLSHAREVCQKASATIDKA
LLKSKSCHQDLAYHYLQVPQGEWNNTLSRDKDSEIPCRRMRSGSYIKAMGDDDSEDSETS
PKPSPKTAARRQSYLKATQQSLSEQSTTQRSLDRLDSVEILLPPKFPTWEEEYNQISDSV
NESSCINQIFGPPSLIPQIFTHGEQAREPELGEQYEAVCDSTYSEAESAAAEVLDLPLPS
YFRSRSHSYLRAIQAGCSQDEDTSSVRSISPPPAGSLSGSRTLPTNSSSCLVAYKKTPPP
VPPRTTSKPFISVTVQSSTESAQDTYLDSQDHKSEVTSQSGLSNSSDSLDSTRTPSVTRG
SIVTPARDTPELPQKNATLKSDKGTLTSEEPKVENVPKRKLSSIGIQVDCLQPVVKEEPP
PPVTKFQSIGVQVEDEWRNSHSRSMSSKQDTDSDTQEPNNSSCKSSERSIAECPQHNNSV
GVDTSTRQPAKPSQIKRNLSYGDNTDPALEPSSLPPPDPWLETSSASPSEPTQPGACRRD
GFWFLKLLQAETERLEGWCRQMDQETKENNLSEEVLGKVLSAVGSAQLLMSQKFQQFHGL
CEQNLNPNANPRPTAQDLAGFWDLLQLSIEDISMKFDELYHLKANSWQLAETPERKEEKK
PPPPVPKKPAKSKSQLNRDKASDSDKQRQEARKRLMAAKRAASVRQNSATESADSIEIYV
PEAQTRL

>Pelodiscus sinensis_DLGAP4(ENSPSIP_18720)
MKGLGDNRSRHLSDNLDSPQDSLYASQDRNPYLLSPTDPYPMDPRFPAQNSLHSDCLLPL
NNQISNSSTFPRIHYNSHFEVPDESPFPSHAQATKINRLPANLLDQFEKQLPIHRDGFST
LQFQRSDSKHRSESPGRIRHLVHSVQKLFAKSQSLEGPTKGSVNGKKGTEDPKQNRRSKS
KDRAKTTETKRRPRSNISGWWSSDDNLDSDTCSYRNPMGLMTLGRQPDHSQPRYFMHAYN
TISEHMLKSSKSNNDLKVTPCTNIPINNESNYIKRGSWSTLTLSHAREVCQKASATIDKA
LLKSKSCQQDLAYQYLQVPQGEWNNTLSRDKDNEIPCRRMRSGSYIKAMGDDDSEDSETS
PKPSPKTAARRQSYLKATQQSLSEQSTTHRSLDRLDSVEILLPPKFPSWEEEYNQLPDNI
NESSCISQIFGQSSFIPQIFTHGEQTREAELCDQYEAVCESAYSEAESAAVEVLDLPLPS
YFRSRSHSYLRAIQAGCSQEEDTASVRSISPPPASSIGGSRTLPSNSSSSCLVAYKKTPP
PVPPRTTSKPFISVTVQSSTESAQDTYLDSQDHKSEVNSQSGLSNSSDSLDSTRTPSVTR
GSLVTQARDAPELPQKNATLKSDKGTLTSEEPKVENIPKRKLSSIGIQVDCLQPVAKEEP
PTPTTKFQSIGVQVEDEWRNNHSRSMSSKQDTDSEAQEPNHSSSKSSERSLADCPPRHNS
VSVDASTRQPVKPSQFKRNLSYGDSSDPALEPSSLPPPDPWLETSSNSPSEPTQPGTCRR
DGYWFLKLLQAETERLEGWCRQMDQETKENNLSEEVLGKVLSAVGSAQLLMSQKFQQFRG
LCEQNLNPNANPRPTAQDLAGFWDLLQLSIEDISMKFDELYHLKANSWQLAETPEKKEEK
KPPPPVPKKPAKPKTPLNRDKASDSVDKQRQEARKRLMAAKRAASVRQNSATESADSIEI
YVPEAQTRL

>Xenopus tropicalis_DLGAP4(ENSXETP_37684)
MKGLGDARVRHLSGTLDSHPDSLYIPQERTPYLISSTESYPPEPRYTPENSLHPDCLYPL
NNQLSNSSTFPRIHFNSHYEPPEDTPPYPTPHVAPAKINRLPASLLDQFEKQLPIHKDGF
STLQFQRGVPEGKDRSESPGRIRHLVNSVQKLFNKSQSLEGGGRASVNGKKGSSEDSKGT
RRSKSKERGKGDGKGQSKGSAVSGWWSSDDNLDSDTGGYRNPAGMMTLGRRPEHAARHFI

SGYNTISESMLKASKSNNDLKFTPFQPPSLTLSPLPAPQDVLPVSSTSTLKRGSWSTLTL
SHAREVCQKASATIDKAFLKSKSCHQDLSYQYLQVPSGDWSGPPIKEDDIPCRRMRSGSY
IKAMGDADSDESEGSPQPSPKTAARRQSYLKATQLSLSESTTQSAINKHTKRLVNNEHAT
LLYVSLCYVPRSLDRVDSLDILSPPKFQSWEEDYNQMTESVAEESKEEQSDRFPMIPSVF
EESGSLRHEVFDLNLPNYFRSRSHRYARNIHKGLRHEVCCSSGNSSMEPTTTKLVTVNNI
GDSTGSSCLVGYKKTPPPVPPRTTSKPFISVTVQSSTESAQDSYLDAQEGQSEGNSQSGL
STSTESLESTRAPSVTRIGPPTPIRQVPPEIPPKNASVKSNKEEPRAPEPLPKRKLSSIG
IQVDSIQPVPIPELPPPARFQSIGVQVEEEWRTKGYTKNLNTLLSLIRIPSLSYKPEITF
LLTLCLQASNGSGPKKPQLSNLFSAFRESIRSYCRVFISFLIFKEEFNIRFYIFPPIRSI
IIEELGGPTIIIKTIKLLLHFLIIQRVDCSCSACSSVSVQDPNTSNLPELSVIGKVLSAV
GSAQLLMSQKFQQFRGLCEQNLNPNSNPRPSAQDLAGFWDLLQLSIEDISMKFDELFQLK
ANGWALSETPDRNEMKRAPPPVPKKPARPKPPLSRDKAPGPSNSDKQRQEARKRLMAAKR
AASVRQNSATESADSIEIYVPEAQTRL

>Danio rerio_DLGAP4(ENSDARP_2935)
MKAFTPQRTRHLSDCEPPRSPAHLGSVYPPSHTPYLLPSAMDHYGALDPHHFSPAPGPGG
LPPDCLMPLNNQLSSSSTFPRIHYNSHFDQAPSSSLPSERARTRKMNRLPPNLLDQFEKQ
LPGQQDGFSTLQFHRSTAVTTTKQQQQQRTDSPGKIRYLVHSVQKLFAKSQSLENSAIKA
NMNGRNSRSSSSDDKHHRSKSKDRAKSEGTAKRRPRSNMSGYWSSDDLDSDVSNYRNPMA
MMTLGRQGPDSQVASRYYMQGYSTISEHTLKSSKSNSDLKHQPLPALPAPGGIGGGRAQM
FDGNFSKAGPWSTLTLGPTRQVCQKGSATLDRTLLKSKSVQQDLGCHFLQMPSTGEWSGT
LGRSGPMGEIPCRRMRSGSYVKAMADLEDSEDSDGSPKPSPKSAARRQSYLRATQQSLSD
QFPSRKNRSLDNLDCIGGGSPFPNWDDDDFSQGCSTLGRGSCINQMRDMEMSQHYADELE
DIHPHPRCSDPPDVPMPTCFRSRSHSYLRAIQAGCSQDDDTASMDSDSPPPTTTTTVRTY
SNSTVSTCMTTCKKIAPPPVPPRTTSKPFISVTVQSSTESAQDCYPDRKSDGNSQSGRSN
SSDSLDSTRANSLAKGTRPQIPVAAPREPQIPAAVVISPNALRETHNEPLKGETLAADEP
PLEPVPRRKLSSIGIQVDCIQEIQAKVETPPPLARFQSIGVQVEDGWTFSRSSSMASRQE
TDSDTQDLSLTSLTFQSGSKSAEQKVMDSDPPVSSSGPSRQILTHRSTTQSSSSSFSESL
DPALDPSALPPPDPWLESGNGTGNGCSNQPLSSGTACRRDGHWFLKLLQAETGRMEGWCC
QMEKETTEHQLSEEGTGKVRSAVGSAQLLMNQKFQQFRGLCEQNLNVNANPRPTAQDLAG
FWDLLQLSIEDISLKFDELYHLKSNDWKLNGESPEKQGFKENQKQAPPVPKKPGKSKPTL
GREKSTESVDKQRQEARKRLMAAKRAQSVKQNSATESADSIEIYVPEAQTRL

>Takifugu rubripes_DLGAP4(ENSTRUP_24240)
MKGLGANRGRHLSDSCEPTGCPQKPLCPMSSDPHSSFLLSPTFNHYGTLDPHLHQYTPTS
PTSLTPDCLMPSSQMTNSSTFPRLHFTSQAEPGDCSLPCMAGAAAKINRLPSNLLDQIER
HLPLQRDGFSTLQFHRGRMSKQRSESPGRIRHLMHSVQKLFAKSHSLENSAVKGNLNGRS
AGGTAGSTGAGEDGGRPSRRSKSKDRAKTEGMRQIPRPNALGLWSSDDALDDVTKAGTIA
VGYRNPLSMMTLGRAVSDSQARHIPQGYNTISAHTTLKASKSSSDLKFLACPATQIGSKE
DEARTRGSKDDTMVKRGTWSTLTLHQARQVLQKGSATVNRTLLKSKSCHQDLAQQFLQVP
VGDWATLGRGRAKGTEIPCRRMRSGSYVKAMGDVEDSEDSEGSPKPSPKSAARRHSYLRA
TQRSLSEQQPPPPPHRSLDNLDCLVSPLEAPLRHRNNGFSQCSSTLGRSTQLGVFDVMCS
FSTARFRPHQHVMPSVLSMQGFGSSAPYGEVESQAVEALDLPAPTCFRSRSHSYLRAIQA
GCSQDEDTASVDSDSLPPISTGYSSNTRKTIPDISLTVSSRKAPPPVPPRGTSKPLISVT
AQSSTESAQDTYLDQQDRGSEANSQSGRSNSSDSLCSIRTGGLAQGSKPSPAPFPEVAPP
PAAPSVPPVPAPRELPPTITVTTDQPPTAPRRKLSSIGVQQDQERHQEFKRWIHDHPASS
TADLSPSDADRLSLSRDNSMASRQNTETELKESQDTTPTEKNTTNCTSQTSNNTAPSKPE
KPTDQQPIKNASAASRISASPADSLDPALDPSSLPPPDPSLDAGNCSTHGDGVQATPACP
RDGNWFLKLLRAETARMEGWCQQMEQETKDNKLSEEVLGMTRSAVGSAQLLISQKFEQFR
GLCNENLNINANPRPTAQDLAGFWDLLQLSIEDISNKFDELYHLKTNNWQLPEKLQKKDE
NKQLPSSVPKKPSKPKLSTGKDRSVDSAVDKQRQEARKRLMAAKRAASVRQNSATESADS
IEIYVPEAQTRL

>Tetraodon nigroviridis_DLGAP4(ENSTNIP_484)
MKGLGANRSRHLSDSCEPTGCPQRPLCPLSSDPHSPFLLSPTFNHYGTLDPHLHQCTPTS
PTSLTPDCLLPSSQMTNSSTFPRLHFTSQAEPGDCSLPCMAAAAKINRLPSNLLDQIERH
LPLQRDGFSTLQFHRGRMSKQRSESPGRIRHLMHSVQKLFAKSHSLENSAVKGNLNGRSA
GAGDDGGRPSRRSKSKDRAKTEGTRQTPRPNALGLWSSDDALDDATKAGTIAVGYRNPLS
MMTLGRAVSDSQARHIPQGYNTISAHPTLKASKSSSDLKFLACPATQICSREEEARSRGS
KDDTMVKRGTWSTLTLHQARQVLQKGSATVNRTLLKSKSCHQDLAQQFLQVPVGDWATLG
RGRAKGTEIPCRRMRSGSYVKAMGDVEDSEDSEGSPKPSPKSAARRHSYLKATQRSLKLS

TNRSLDNLDCLVSPLEAPPHRRNDFSHCSSTLGRGAQSLSQLLAERSVFAECHSGIKQGE
TQTLSVRPHEACFAFCHQHESQAVEALDLPAPTCFRSRSHSYLRAIQAGCSQDEDTASVD
SDSLPPISAGYTSNTITVGTFSTAVSSRKAPPPVPPRGTSKPLISITAQSSTESAQDTYL
DQQDRGSEANSQSGRSNSSDSLCSITPPPAAASVPPVPAPRDLPPTITVTTATTSTLPQN
DSSSRGVTLDQPPTAPRRKLSSIGVQVDCVLPVLKEEAQPLTAPLKFQSIGVQVENGRPL
SRDNSMASRQNTETELKETQDSTATDKTTTNCNRPTFSNTDPSKPEKPMEQQPVKNTCAA
SRISTCPAETLDPALDPSSLPPPDPSLDTGNCSAHGDGVQITPACPRDGNWFLKLLRAET
ARMEGWCQQMEQETKDNKLSEEVLGTIRSAVGSAQLLISQKFEQFRGLCNENLNINANPR
PTAQDLAGFWDLLQLSIEDISMKFDELYHLKANNWQLCEKPQKKDENKQLPSSVPKKSSK
PRLSTGKDRSVDSAVDKQRQEARKRLMAAKRAASVRQNSATESADSIEIYVPEAQTRL

>Gasterosteus aculeatus_DLGAP4a(ENSGACP_10781)
MKGLGTSRNRHFTDSCEPSSGHPEALYSQKTSTLPRSPYLLSPTMDHYGTMDPHLYPSAN
PGSLPPDCMLPLNNQMSNSSTFPRIHYNSYDQSDFSPPGDSIGGHMTKNQAPTSLLEFDK
QLPGGHDGFSTLQFHRTSAVAGGKQHTDSPGRIRYMLHSVQKLFAKSQSLESQNMKGNFN
GRSTGSGGGSSGTEDGGKQNRRSKSKDRGTKSEATSKRRPRSNMSGYWSSDDLDSSDLSS
YHNTMAMMTLGRPTGHDSQGAQTRYIQSGYNTISSSKSSNDMKYQALTGPGGGGGHGLVG
AGRTVTNDNDYMKGGSWSTLTMGQPRHVIQKGSATLDRSMLKSKSCQPELTCNYLQVGRR
GDWSSTLGRSGGANEIPCRRMRSGSYVKAMGDMEDSDDSDGSPKPSPKSAARRQSYLRAT
QQSLSDQLPPRKELSNNRSLDNLDCIGGTVSSSPQWDDDDFSQACSTLGRRSCLGQVSGN
LNRSKSYIAGHVRNLRDLEMSHQYEDRSSDSTFRDSRSHSQDNPEPLDLPMPTCFRSRSH
SYLRAIQAGCSQDDDTASMDSGCSPPHIDTTVRTYNTSTVSSCITTCKKAAPPPVPPRTT
SKPYKSVTVQSSTESAQDNYLDQQDRRSEVNSQSSHAHSNSSDSLDSTRANSLARGIRRP
PHIIPTPIAIPREPIAPTTSNASTETIEEPQVAPVPRRKLSSIGIQVDCIQEVPQEETPP
LAKFQSIGVQVEDGWQRRLSRSSSMASKQETDSDTQDIPVVSHYNNSKQYNGDTPGDAPS
PPPPRQILKRSTTRSSSSSFSESLDPALDPSCLPPPEPWLESGNGSNGSVPQSGGGGTLC
RRDGHWFLKLLQAETGRMEGWCHQMEQETKDNQLSEEVLGKVRSAVGCAQLLMAQKFQQF
RGLCEQNLNVNANPRATAQDLAGFWDLLQLSIEDISLKFDELYHLKSNDWQPMPSAAAQS
PPGRKDEEKESATKKPGKGRPSLGREKSTDSSSASSTASAEKQRHEARKRLLAAKKAASF
RQNSATESADSIEIYVPEAQTRL

>Gasterosteus aculeatus_DLGAP4b(ENSGACP_4213)
MKGFGANRSRHMSDSCEPARCPQKPLCPLTSDPHGAFLLTPTINHYGTLDPHLHLCSPTS
PTSLNPDCLLPFSQMSNSSTFPRLHYSSQAEQGSATISSSSAAAAKMNRLPSNLLDQLER
HLPLQRDGFSTLQFHRGRMSKQRSESPGRIRHLMHSVQKLFAKSQSLENSAIKGNVNGRS
AGGTAGGAAGAGEDGGRPTRRSKSKDRAKTEGTKQRQRPNALGLWSSDDTLDTDTTTAGT
IAVGYRNPLSMMSLGRAVSDSQAGPRHIPQGYHTISAHTLKTSKSSSDLKFLACQATQVV
SKEEEGRIRGSKDDLLVKRGSWSTLTLSQARQVLQKGSATVNRTLLKSKSCHQDMAQQFL
QVPLGDWAGTLGHGRPRGTEIPCRRMRSGSYVKAMGDLEDSEDSEGSPKPSPKATARRQS
YLRATQHSLSEQQPPPPQRNLDNLDCLVSPLEPPPRYRNSDFSRCSGTLGRGSATQVSQS
ALCPQRVSLHTFFQVYGQGCGHSLPYCQGESQAVEALDLPAPTCFRSRSHSYLRAIQAGC
SQDEDTASVDSESPPHTGAGYSYSSNTRSAQYLFSNRKAPPPVPPRTTSKPLISVTVQSS
TESAQDVYIDQQERGSEANSQSGRSNSSDSLCSIRTAGLAKGSRPLPVPVPAAAPGPAAG
SVHPVPAPRDLQSQNDTPSIGFTLDPVPVATKRKLSSIGIQVDCLLPVLREEPLPLTAPL
KFQSIGVQVENGRPLSRETSMASRQNTETEPQDATENNTANCTNSQTADANAPNGQDKAM
EQQPLKHTHAPSRISSPPQVTLDPALDPSSLPPPDPSLESGNCCSHGDAGQPGPPACLRD
GNWFMKLLQAETGRMEGWCQQMEQETKDNKLSEEVLGTIRSAVGSAQLLMTKKFEQFRGL
CKENLNLNANPRPTAQDLAGFWDLLQLSIEDISMKFDELFQLKANNWQLPEKSEKDENKQ
LPSSVPKKQTKPKLSAGKDRSVDSAVDKQRQEARKRLIAAKRAASVRQNSATESADSVEI
YVPEAQTRL

>Oryzias latipes_DLGAP4(ENSORLP_20789)
MKGLGTNRNRHLSDSCDPSSGHSEAFYTEKTSTLPRSPYLLSPTMDHYGSMDPHFYPSTN
PGSLPADCMLPLNNQLSNSSTFPRIHYNSYDQSDFSPPGDSIGGNQMPPHLLEFEKQLPG
GHDGFSTLQFHRTSAAAAAKQRTDSPGRIRYMLHSVQKLFAKSQSMETHNMKGNVNGRST
GSGGGSSGTEDGGRHQRRSKSKDRGSGTKSEATAKRRPRSNMSGYWSSDDLDSSDLSSYH
NTMGMMTQGRPTGHDTQVGQTRYVHSGYNTISSSKSTNDMKYPALPASGGRAGTADMLMN
DGDYMKGGSWSTLTMGQPRQVIQKGSATLDRSMLKSKSCQQDLTCNYLQVGRRGDWSSTL
GRSGGPNEIPCRRMRSGSYVKAMGDMEDSDDSDGSPKPSPKTAARRQSYLRATQQSLSDQ
LPPRNVQHTRRLKTQVKAFHILTSLKEMSSNRSLDNLDCIGGAGPLVPWEDDDFSQACST
LGRRSCLGQLRDLERSHAYEDRSSESTFRDSRSQSQDNSEPPDLPMPTCFRSRSHSYLRA

IQAGCSQDDDSASVDSGCSPPPSDSTVRTYSTSTELCGVKRWSAAPEVSTCITTCKKTAP
PPVPPRTTSKPFISVTVQSSTESAQDNYLDQQDRRSEVNSQSSHAHSNSSDSLDSTRANS
LARGIPRLPHIIATPVATTREPAASRTANASTETSDSGVQSEFLKRKLSSIGIQVDCIQE
VPREETPPPAKFQSIGVQVEDGWHWSRFRLSRSSSMASKQETDSDTKDSLDNGEPSGDAS
PPPPPRQILNRSTTRSSSSSFSESLDPALDPSSLPPPDPWLDSGTDGHSGLPQGGGGGTL
CRRDGHWFLKLLQAETGRMEGWCQQMEQETKDNQLSEEVLGKVRSAVGSAQLLMSQKFQQ
FRGLCEQNL

>Homo sapiens_DLGAP5(ENSP_247191)
MSSSHFASRHRKDISTEMIRTKIAHRKSLSQKENRHKEYERNRHFGLKDVNIPTLEGRIL
VELDETSQGLVPEKTNVKPRAMKTILGDQRKQMLQKYKEEKQLQKLKEQREKAKRGIFKV
GRYRPDMPCFLLSNQNAVKAEPKKAIPSSVRITRSKAKDQMEQTKIDNESDVRAIRPGPR
QTSEKKVSDKEKKVVQPVMPTSLRMTRSATQAAKQVPRTVSSTTARKPVTRAANENEPEG
KVPSKGRPAKNVETKPDKGISCKVDSEENTLNSQTNATSGMNPDGVLSKMENLPEINTAK
IKGKNSFAPKDFMFQPLDGLKTYQVTPMTPRSANAFLTPSYTWTPLKTEVDESQATKEIL
AQKCKTYSTKTIQQDSNKLPCPLGPLTVWHEEHVLNKNEATTKNLNGLPIKEVPSLERNE
GRIAQPHHGVPYFRNILQSETEKLTSHCFEWDRKLELDIPDDAKDLIRTAVGQTRLLMKE
RFKQFEGLVDDCEYKRGIKETTCTDLDGFWDMVSFQIEDVIHKFNNLIKLEESGWQVNNN
MNHNMNKNVFRKKVVSGIASKPKQDDAGRIAARNRLAAIKNAMRERIRQEECAETAVSVI
PKEVDKIVFDAGFFRVESPVKLFSGLSVSSEGPSQRLGTPKSVNKAVSQSRNEMGIPQQT
TSPENAGPQNTKSEHVKKTLFLSIPESRSSIEDAQCPGLPDLIEENHVVNKTDLKVDCLS
SERMSLPLLAGGVADDINTNKKEGISDVVEGMELNSSITSQDVLMSSPEKNTASQNSILE
EGETKISQSELFDNKSLTTECHLLDSPGLNCSNPFTQLERRHQEHARHISFGGNLITFSP
LQPGEF

>Pan troglodytes_DLGAP5(ENSPTRP_10806)
MSSSHFASRHRKDISTEMIRTKIAHRKSLSQKENRHKEYERNRHFGLKDVNIPTLEGRIL
VELDETSQELVPEKTNVKPRAMKTILGDQRKQMLQKYKEEKQLQKLKEQREKAKRGIFKV
GRYRPDMPCFLLSNQNAVKAEPKKAIPSSIRITRSKAKDQMEQTKIDNESDVRAIRPGPR
QTSEKKVSDKEKKVVQPVMPTSLRMTRSATQAAKQVPRTVSSTTAKKPVTRAANENEPER
KVPSKGRPAKNVETKPDKGISCKVDSEENTLNSQTNATSGMDPDGVLSKMENLREINTAK
IKGKNSFAPKDFMFQPLDGLKTYQVTPMTPRSANAFLTPSYTWTPLKTEVDESQATKEIL
AQKCKTYSTKTIQQDSNKLPCSLGPLTVWHEEHVLNKNEATTKNLNGLPIKEVPSLERNE
GRIAQPHHGVPYFRNILQSETEKLTSHCLEWDRKLELDIPDDAKDLIRTAVGQTRLLMKE
RFKQFEGLVDDCEYKRGIKETTCTDLDGFWDMVSFQIEDVIHKFNNLIKLEESGWQVNNN
MNHNMNKNVFRKKVVSGIASKPKQDDAGRIAARNRLAAIKNAMRERIRQEECAETAVSVI
PKEVDKIVFDAGFFRVESPVKSFSGLSVSSEGPSQRLGTPKSVSKAVSQSRNEMGIPQQT
TSPENAGPQNTKSEHVKKTLFLSIPESRSSIEDAQCPGLPNLIEENHVVNKTDLKVDCLP
SERMSLPLLAGGVADDINTNKKEGISDVVEGMELNSSITSQDVLMSSPEKNTASQNSILE
EGETKISQSELFDNKSLTTECHLLDSPGLNCSNPFTQLERRHQEHARHISFGGNLISFSP
LQPGEF

>Gorilla gorilla_DLGAP5(ENSGGOP_3409)
MSSSHFASRHRKDISTEMIRTKIAHRKSLSQKENRHKEYERNRHFGLKDVNIPTLEGRIL
VELDETSQELVPEKTNVKPRAMKTILGDQRKQMLQKYKEEKQLQKLKEQREKAKRGIFKV
GRYRPDMPCFLLSNQNAVKAEPKKAIPTSIRITRSKAKDQMEQTKIDNESDVRAIRPGPR
QTSEKKVSDKEKKVVQPVMPTSLRMTRSATQAAKQVPRTVSSTTARKPVTRAANENEPER
KVPSKGRPAKNVETKPDKGISCKVDSEENTLNSQTNATSGMDPDGVLSKMENLPEINTAK
IKGKNSFAPKDFMFQPLDGLKTYQVTPMTPRSANAFLTPSYTWTPLKTEVDESQATKEIL
AQKCKTYSTKTIQQDSNKLPCPLGPLAVWHEEHVLNKNEATTKNLNGLPIKEVPLLERNE
GRIAQPHHGVPYFRNILQSETEKLTSHCLEWDRKLELDIPDDAKDLIRTAVGQTRLLMKE
RFKQFEGLVDDCEYKRGIKETTCTDLDGFWDMVSFQIEDVIHKFNNLMKLEESGWQVNNN
MNHNMNKNVFRKKVVSGIASKPKQDDAGRIAARNRLAAIKNAMRERIRQEECAETAASVI
PKEVDKIVFDAGFFRVESPVKSFSGLSVSSEGPSQRLGTPKSVSKAVSQSRNEMGIPQQT
TSPENAGPQDTKSERVKKTLFLSIPESRNSIEDAQCPGLPDLIEENHVVNKTDLKVDCLP
SERMSLPLLAGAVADDINTNKKEGISDVVEGMELNSSVTSQDVLMSSPEKNTASQYSILE
EGETKISQSELFDNKSLTTECHLLDSPGLNCSNPFTQLERRHQEHARHISFGGNLITFSP
LQPGEF

>Mus musculus_DLGAP5(ENSMUSP_40416)
MLVSRFASRFRKDSSTEMVRTNLAHRKSLSQKENRHRVYERNRHFGLKDVNIPLEGRELG

NIHETSQDLSPEKASSKTRSVKMVLSDQRKQLLQKYKEEKQLQKLKEQREKAKRGVFKVG
LYRPAAPGFLVTDQRGAKAEPEKAFPHTGRITRSKTKEYMEQTKIGSRNVPKATQSDQRQ
TSEKQPLDRERKVMQPVLFTSGKGTESAATQRAKLMARTVSSTTRKPVTRATNEKGSERM
RPSGGRPAKKPEGKPDKVIPSKVERDEKHLDSQTRETSEMGPLGVFREVESLPATAPAQG
KERKSFAPKHCVFQPPCGLKSYQVAPLSPRSANAFLTPNCDWNQLRPEVFSTTTQDKANE
ILVQQGLESLTDRSKEHVLNQKGASTSDSNHASVKGVPCSEASEGQTSQPPHDVPYFRKI
LQSETDRLTSHCQEWEGKLDLDIPDEAKGLIRTTVGQTRLLIKERFRQFEGLVDNCEYKR
GEKETTCTDLDGFWDMVSFQVDDVNQKFNNLIKLEASGWKDSNNPSKKVLRKKIVPGRTS
KAKQDDDGRAAARSRLAAIKNAMKGRPQQEVQAHAAAPENTKEVDKIVFDAGFFRIESPV
KSFSVLSSERRSQRFGTPLSASKVVPEGRAAGDLLRQKMPLKKPDPQSSKSEHVDRTFSD
GLESRCHVEDTPCPGEQDSSDIEHDVNKINVKMDCFSVETNLPLPAGDANTNQKEAISAV
EGASSAVTSQDLLMSNPETNTSSQSNTSQEEAEASQSVLLHKSLTSECHLLEPPGLSCTS
PCTREETRQPDRSRQFSFGGDLILFSPL

>Rattus norvegicus_DLGAP5(ENSRNOP_15553)
MPVSRFAGRLRKDSSIEMIRTSLAHRKSLSQKENRHRVYERNRHFGLRDVNVPLEGRDLV
NIHEMSQDLTLENASSKTKSVKMVLSDQRKQLLQKYKEEKQLQKLKEQREKAKVFKVGLY
RPAAPGFLVADQRGVKAEPQKAFPYTGRITRSKARDHMEQSKTGSRNIPQAAQFGQRQIS
EKKPLDREKKVMQPMGPTSGKAAGSVAATQRAKQMAGTVPSTTRKPVTRATNENGSERRG
PSRGRPAGKAEGELDKIISSQGIALGSEEKHLQSQSSVTNEVESLQETVPAKGRERESFA
PERCAFQPPRGLKTYQVAPLSPRSANAFLTPSCDWNHLRPEVATAQDTANEVLVQQESNK
SQFGLDSQTDSRKERGLNQKDASTPNSDHLSVKGITSSEAGDGQTGQPQHDVPYFRKILQ
SETDRLNSHCLDWEKKLELDIPDDAKDLIRTAVGQTRLLIMERFRQFEGLVDNCEYKRGE
KETTCTDLDGFWDMVSFQVDDVTQKFNNLMKLEESGWKDNNNPSKKVIRKKIVPGKTSKA
KQDEDGRTAARSRLAAIKNAMKDRLRQEAQAQAAVPEIPTAADKIVFDAGFFRIESPVKS
FSVLSSERRSQFGAHVSASRAVPEGRAAGDLLRQKTPLKRPDPQSSTIEHVDRTFSDGLE
SRCSQVEDILCPVQQDSSDVERDVNKIKVKMESFSVATMNLPLPAGEVAGDANSNQKDAV
SAAAVEGVGSAVLSQDVLMSHPETNAFSQRDSLQEGAELSQSVLLHKSLTSECHLLGPPD
LSCTSPCTREDTRQQDRGRQFSFGGNLIVFSPLRP

>Canis familiaris_DLGAP5(ENSCAFP_40149)
MSSHFVSRHRKDLSVNMIRTKIAHRKSLSQKESRYKEYERNRHFGLKDVNIPTLESKILL
ELDETSQNLVPEKAKVKPRSKKTILGDQRKQMLQKYKEEKQLQKLKEQREKAKRGVFKVG
LYRPDMPCFLLSNQSTVKAEPKKAVSSFVRITRSKAKDQMEHTKINNGSDVRAIQPGQRQ
TSERKVLDKEEKVVQPVMTTSVRLTRSATQAAKQIARTVSSTTARKPIIRATNDHEMERQ
RPNQRRPTKKTETKPDKVVSFKVDSEDNTLDLQIGATNGMDPDGVLPKMENLPKNTAKTK
GKNSFAPQDFVFQPLDGLKTYQVKPMTPRSANAFLTPSYTWTPLKTEVDKTQEVTKEILT
QECKTYSAKMIHQDSDKLKCPLGSLTVWNKEHVLNKDETTTKNSNGLTIKEVPSLEIIED
QISQPHHDVLYFRNSLRLETEKLTSHCLEWDRKLELDIPDDAKDLIRTAVGQTRLLMKER
FKQFEGLVDNCEYKRGEKETTCTDLDGFWDMVSFQIEDVNQKFNNLTKLEESGWQNNNNI
SKKVPQKKVVSGVASKPKQDDDGRIAARNRLAAIKHAMREKMKQKEPVEAAASVMPKEVD
KIVFDAGFFRIESPIKSFSGLSISSECLSQRLRTPKSVSKTVSESRAEMDHLRQTMSPQN
PDPQSTKSEHVDKETLLSTIPKTRNSTEDAQCPGLQGLIEVNDNANKINFEMACLPSERM
NLPFLAGEVADDINTNKKKEIPDVIEGLELHSSVTAQDILMSSPEKNIPSQNNILQEEKT
KISQSVFDTRSLTTERHLLDSPDLNCSTTFTWVERKHQDHIRHISFGGNLIAFSPLRPFS
DKQPEEF

>Felis catus_DLGAP5(ENSFCAP_15423)
MSSSHFASRHRKDLSIDMIRTKIAHRKSLSQKENRHKEYERNRHFGLKDVNIPTLESRVL
LELDETCQNLVPEKASVKPRSTKTALGDQRKQMLQKYKEEKQLQKLKEQREKAKRGVFKV
GLYRPDMPCFLLSNQSTEPKKAVPSSVRITRSKAKDQMEQIKIDNGSDVRAIRPGQRQTS
ERKVLDKERKAVQPVTTTSVRMTRSATQAAKQNARTVSSTTTRKPVTRANDHEIERKAPN
QGRPAKNIETKPEKVISFKLDSEENTLDSQASTTNGMDPDGVLSKLENLPKTNTAKTKGK
NSFAPQDFMFQPLDGLRTYQVTPMTPRSANAFLTPSYTWTPLETEIAKTQEPTKEILAQK
CKTSSAKIVHQDANKLQCPLGSLTVWNKEHVLNKDETTKNSNGLPTKEVPSLEINEDQVS
QPQHDVPYFRNILQSETEKLTSHCLEWDRKLELDIPDDAKDLIRTAVGQTRLLMKERFKQ
FEGLVDNCEYKRGEKETTCTDLDGFWDMVSFQIEDVNQKFNNLTKLEESGWQNNNNTNKK
VFRKKVVSGVPSKSKEDDGRIAARNRLAAIKNAMRERMKKEEHVEAAASVMPKEVDTIVF
DAGFFRIESPVKSFSGLSISSERLSQRLRTPKSVSKAVSENRSKMGVLRQTVSPQNPDPS
STTCGHVDKETLLSTIPKNRNSIEDTPCPGLQDLIEVNYGTNKISFEMDRLSSERMSLPL
PAGEVRDDINDNKKEEISDDVEGMELNSSVTAQDVLMSSPEKNIASQNNILQEEETKISQ

SVLFDTKSLTTECHLLDSPGLNCGNTFTQVERRHPDHGRHISFGGNLITFSPLRPLSEEH
PEEF

>Myotis lucifugus_DLGAP5(ENSMLUP_9522)
MSSSHFASRRRKDLSTDMIRTKIAHRKSLSQKEHRHKEYERNRHFGLKDVNIPTLEGRYL
LELDETSQELVPEKANVKTKSVKTLLHEQRKQMLQKYKEEKQLQKLKEQREKAKRGVFKV
GLYRPDVPSFLFSNRNTTKAEPIKAVSSFVRMTRSKTKDQMEQTKITIGNDVQATRPGQR
QTSEKKVLNKEKKVDQPEMPTSIRMTRSATLAAKQMARTVSSTTRKPVTRATNDNEAERK
ATNQGRPAKNVETKPDKVISFQVDSEEDNLDSQTSATNDGVLSKMENLPNTNTVKMKGKN
SFAPKDFMFQPLDGLKTYQVTPMTPRSANAFLTPSFTWTPLKTEVDKTADATKEILAQKC
KIDSAKTVHQDSNKLQYPLDSLTVWNKENVLNTNDTTKNSSGLLIKEVPSLEINEDQLSQ
PQHDVPYFRNILQSETEKLTSHCLEWDRKLELDIPDDAKDLIRTTVGQTRLLMKERFKQF
EGLVDDCEYKRGEKETTCTDLDGFWDMVSFQIEDVNQKFNNLTKLEDSGWQNNNNNNISK
KVIRKKVVSGIASKPKQDDGGRIAARNRLAAIKNAMRERIKQEEHAEAAASVVPKEVDKI
VFDAGFFRVESPVKSFSGLSISSERLSQRLRTPTSVSKAVSDSRAALGLLSQTASPEHPG
PQSTRREQADEKTLLSNIPENRNSIVEDAQCPRLPDLIEVNHDISKINFKMDCLPSERMS
LPLPAGEVACDINTNKTEEISDVVEGMELNSSMTTQDVLMSSPEKDIPSQHNISQEEETK
IFQSVLFDNKGLTTECHLLESTDLNCSNLFTPVERRRREHARHISFGGNLITFSPLRALS
GEQPEEL

>Monodelphis domestica_DLGAP5(ENSMODP_17488)
MASLSVPNHYRRDASTEMIRTKVAHRKSITWKENRHKKYEQNRHFGLLDVNTTSLEERNL
PQLDETIDNILEKDNVKPSDLRELLSNQRKEMLQQYKERKQLQKMKEQRDRMRGGVFKVG
LYKPDPPQFLRGLPGQKTVKPEPKKVFQTSVRITRSKVKDKLEQPKVNNGHNMEAVQAGQ
KPTSEHQSSKERKVVKPDMRVTRSATIAAKQIPKPTSSVAMRRPIPKLISENQLERKAST
EERPPPKTEVKVDKGKILPSKAGGEENSSKDALTEGPSHSFKKSPSEEIAEFLGKEYIPE
KNVASQVRKKYSFAPENFRFQPPDSLVNYRIKLISPQRECVAPSCFWSPLEVKDYHSQES
KRETLKENKVDCNASAQPDLIDLQSPPKPLEILHKDCLSNKNGDTTTISDNLPLNTQIPL
LEINEHQTAPPQHDVQYFRNILQSETERLTGFCHDWEKKLTLDIPDDAQDLIRTTIGQTR
LLISERFKQFEGLVNDCDYNCGQKKITCMDLDGFWDMVNFQVEDVNKKFEKLSKLQESEW
QEKNTINQKVIRKKAVPVEKCKSKQDDAARTAARNRLAAIKASMKEKIKQEEPSLEIAVP
GVSKAKEIDKIVFDAGFFRVESPVKSFSAISASKEAVHSKSDDPAEYKTPGNKKRNLIEG
NWTKLCSPSYQ

>Xenopus tropicalis_DLGAP5(ENSXETP_34368)
METRTQFTGRYKKDLSTENLRAKVARRKSITQKENRHNEFKKCRGLSVADANISLLKECE
LPPLEETDECSILKAQETTVDIVGKSKEYTKQRRDMLQRFKEEKELRKLKEQREKAAKGV
FKCGIYKPDVTIPPVWGTQHTAKVKPKEKLAPPVVSRVTRSMAKAEPAGSKANRTQHVKA
GVLNTSKISAERGITKGCGQNSALKKPETKVAPSQRGVRTALSTTTKPALRNNVVAKSQN
KTTKETKAKENVKKSPVSEPPKVKEAEPLPKEAEPLPKEAEPLPKEAEPLPKEAEPLPKE
AEPLPKEAEPLPKEAEPLPKEAEPLPKEAAVETGTSSIEAHQPPHERKPSFAPQNFMFQP
MDGLSPYKFKPMTPSRANAFLTPCFTWSPVRLERNHLPASPKFNKEQTAVEQVLPVEQVL
PVEQVLPVEQVLPVEQVLPVEQVLPVEQVLPVEQVLPVEQVPAPQSSELEMGIHPAEQQK
ESATDVCLAPTDAPGSPSSSVSNPQKENEKEAPKEPEHDVPYFRDTLKSEIARLKQLCCE
WDKRIELDIPEDAAKDLIRTTVGQTRLLMCERFKQFEGLVDNCEFKRGEKETTCTDLDGF
WDMIYFQIEDVGKKFINLGKMEENSWQQNTAPTKKVVKKKIGPAATSKSTQGDSGRAAAR
SRLAAIKAALKNKGKQEEPTVEAPALPTRFDEVVFDAGFFRVESPAKGTNSVRAKCNSSW
SSPTTQPSTNRIQQSKNHNGEQITEVPNSPGTVNSPIRKSLFDNSEEVSVQSDEPHPIPS
VVETDKAACAVHVADLTKYLVPTETSSIQLCDSPAPEDIGMAPARALFVEETAGISGSFV
DDVFMCSPQTNGPAPQPISPTRESDAVCGNRDLNALNDQLDFLGSCTPINMVEK

>Danio rerio_DLGAP5(ENSDARP_66410)
MESRFAHLYKRDSSTSMIRLKMSRRRSQSQKENREKIQNMRRHLDRLPEIDLSMDTSTLM
ATQDKAPKTKTASDSSDAAEERKKMLARYKENKILQKEKEKREKEKKGVFKVGLYKPQPL
GYLPSNTAAPGRAKATEMNQSTRITRSMVQNQKLAVERHAVPKKVETLSTARAPAKDNRK
PLTSTKTRATTAEPPVRAQTTRSAAKTAPDAVVKPVADTRGPKTRSAVKYPAAPSSGRGK
VIQGNMKANVENKPAEIKDTAVDKVEEPKAAPASFAPQGFVFQPPVGLRSFEPVPLSPSS
ANAFLSPSFNSEVSMEPAFPSPPKPVVSFSSPPPSVSAYMPDHPISPPSVSSPLQAQSFP
QPSTDAAPSPSSAPMEPQHDVAYFRAAMASETERLTGLSELWESRFDDASIPEEMRDRMR
TAVGQARLLMKERFGQFNGLVDDCDFKRGEKITTCTDLQGFWDMVYFQVEDVNKKFSALK
EAEARDWKEEVKPVTKKRVVKKPPVVAGKAAAGGGNAAAKSRLAAIKAAMKAKQAEQKA

TETSDKPQEDSSSVSVAPVQSVVFHGGFFQVESPVKTPAAVRRSCRGVVSSPSVTKFSTP
VRQCRSTAISSASPLPCMSTTPPRPLQPEVLPICTPLRSAEPLPESPRAPQMTPDHMQAP
HTDLCSNSQPQFDSSSTCTQQTQPDFRNEAENSPAGQSESPVECSEAQIEDCIPESMLSP
STQAEDNVVSEESLHVSAGVDALSQCSFHEAEPSVCAPASPAMMPSTPTKDLNASSALSN
ELVLSACSSSPTSADVEMTGPQDAEGATGLDFERYLLPSVRDVSPEQSLAADRFSLGVMD
AEMESPVSQPGESPVDVLASPTAVPRMVFSHRISQMPPSLLLFTPEQRERVRQSVCERDL
MTFTPPTSK

>Takifugu rubripes_DLGAP5(ENSTRUP_43035)
EILLLISTETSCYFAFCYSHVSRSLSMDSKFSHLFRRDTSVSMLRVKMSRRRSQTQKENR
ERTVNTRRKLDKLMELDASCLDASTAVVNMSVAHERTLHGTAIAKKTEAAVEERVKQLER
WREHKALQREKEKREKERKGVFKTGLYHPKDTLVVFSQPWASSASDKVKEPVVQRLTRTR
AAAVKSACVQTKTKTDEPVSSRSAIRPPVTKTPAAKDKAKEKPAGIKFLSLEARTTRSRA
VVGVVVPFSVAHKKHKGVIIEPANTTQPAVPTVSISASQVMEPERKTCPSSPLPCCETTE
RTMDQPQDDCVPAGEADEAPSFAPKDFIFQAPVGLATYRFEPLTPRSADAFLRPRFEILQ
KVFSFKKNLRSLLPLLFHSISFSLLPAPVLDHEPQHVSNNPPPPTNPQCSPPGASPAEAA
PTPTCSMEAQHDVPYFRAEIANGTNRLMSLCVHWEAKVEDESIPEEMRDRMRTAVGQARL
LMKERFKQFSGLVDDCEFSRGEKITTCTDLQGFWDMVYYQVEDVNKKFDALKEAESRAWV
EDRKPPPRPKKAVMKHGALSQKPSGAPVKPTGSKAAAKSRLAAVKAAMKARQQAAETIKE
EPQTQTASQPAESVVFDGGFFQVESPAR

>Tetraodon nigroviridis_DLGAP5(ENSTNIP_15859)
RMESKFSHLSQRDNSVSMLRIKMSRRRSQTQKENRERTLNARRRLEKLPELDASSADASA
LTTNTSVLHQRTLNETALAKKAAVEERMKQLERWKERKAVQKEKEKREKERRGVFKTGLY
HPKDSLVVFSGPAASTRGKEIKEHTAPSHHTRVTRSMKQQLHLQTVLQFFTCLRMFSNVQ
NPLKTQEPNTMAIKGNYPLSQPTQRPTRTQAAPVKSATSQTKAKTGKKPKIQALYTHGSF
LAEPPSTRSASRPPVFRKPVENDKPKEPAGVQECGEGAGPPSKPSVTEQPRADSVPAQGA
PETPSFAPKDFTFQAPAGLTLFKFEPLTPQSADAFLTPSLSFSLPPAPVLDFGPQDVPGT
PPQPKTPPCPPPAEAPPPPACAAEPQHNVPYFRAEVATETDRLTSLCVHWEAKIEDGSIP
EEMRDRMRTAVGQARLLMKERFKQFTGLVDDCEFARGEKVTTCTDLQGFWDMVYYQVEDV
NKKFAALKEAESRSWVEERKPPPRAKRPVKKPSDAPAKPTGSKAAAKSRLAAVKAAMRAR
QQAAGNTDSGAEEAQAGSQPSEPVVFDGGFFHVESPAKPPGPSVAGSLRRSSRLG

>Gasterosteus aculeatus_DLGAP5(ENSGACP_12507)
MDSAFAHLRQRDTSVSMLRVKMSRRRSQSQKENRERVVNTRRQLAELPELEMSSLDASIA
TVNISNIQGKTLNNGKSAKDVAGEERLKQLARWKERKALEKEKEKRERKGVFKTGMYHPK
DTLTIPSLPVVPAASTRAKESVRVTRSMKQQQQTQAALDRTTRSRVAPVKRAPAPATAKI
HDPAAHSASRLPTEVEPAVRALRSKSASRPAVTAAPVVAKDKPMEKTTVKVSYSAAVEAD
VTMESQETPRPPGPAPSSEEQNVAVEAPAVAVEEASSFAPKGFMFKAPVGLSSFKFEPLT
PRSADAFLTPSLNAMLLLFLRSPSFSLPEVPAFNLQPPGEPSDSSPSKSPRRSPPCEAPA
TTGSPLESKRGVPYFRLEIVSETEGLMTLCADWEPKVEDESIPEEMRDRMRTAVGQARLL
MKERFKQFSGLVEDCELGRGEKITTCTDLQGFWDMVYYQVEDVHKKFNALKVAEGRGWVE
EHEPPPPRQRKGVKKPPSAAPAKATGTNQAARSRLAAVKAAMKAKQQAAEAEKAAKDAGA
AVEDAGPSSEESESRAEPPATVVFDGGFFQVESPAKPPGSLRRAGRPSAAALPQTPPCSN
YLSLRRSTRRCLALPQTPLLTAASPARPRHTPARLTLDQAPASPRGTPRASRHRASNSSL
CFSPVRDAAAVQGGAAPALSAPAAEHSLPSICVAEEPAGVCVDLPRSPCRTPQRSLSPSF
SLSPSVTPSRPPTSSPPAVLSPEEAQESVCLTPGTLVVEVNTLCSAVNKVLCFCCCFQEI
PGLDFERYLLPSQRSSLSPGGLDVEMESPRGQTEDLLTLPEHGVRLVSYHNNTNLKSTML
LFTPDLKDRIRQSVCPSDLMVFTPPL

>Oryzias latipes_DLGAP5(ENSORLP_4278)
MESRFASLRQRDTSVSMLRVKMSRRRSQTQKENRQRAMNTRRQLDKLPELEMSSLDASMA
RSNVSALQERETNAKPEKIKAQEERLKQLERWKERKALEREKEKREKQQKGVFKTGLYHP
KDICTGPAVPPVSAKHKEKKVTVAPSQSTRVTRSMKQQQETHLMNSSVSDCITTAGPAVQ
PLSSRSAKRPPVPKTSAVKDKPKEKVADVRTTRAKAALSSVAPSSGHTKSSNGQITGAAH
LVINPIHPTQVSTALSENAKAAPPSFAPRDFIFQAPTGLSSFKFEPLTPRSADAFLTPRL
VYVPLLLSSPTLILPPVSSFTLHPQAELSKPSPPKTSHQSPRGTSAAVTAPPPCSPLESK
HDVPYFRSEIAKETVRLTTFCHHWEPKVEDESIPEEMRDRMRTAVGQARLLMKERFKQFS
GLVDDCDLGRGEKVTTCTDLQGFWDMVYYQVEDVNKKFDALKEAEGRSWVEECKPPPRQR
KTVKKPSAAPAKATGTNAAAKSRLAAVKAAMKAKQQAAEAEKASQDSTSGDLPSQKPGPQ
AEALKLDAVVFDGGFFKVESPAKSSSEGGKPTVLCFICSFYKCVFRRSSRLSAAVLPLAS

PCSSYRTPRRAAQRPLALERTPIGSRASPVQLSRPTLEQAPTPKCQPDIQGIDGPANASL
CFSPVKELPSSEETSDGSPAQKAGGASPPTVSVERNSAEPTEGTPLSAAHCIPSSKSPSP
APHVPEPPSGLSFVLSPCAPLTSAAAEAPQAVYQTPNSSVVEEVPGLDFDRYFQPSQRCS
LSPREPVAEETLSPMATDVEMEGPRGQAEELPALSAVSSVFSPRSPQVQSAKAALLLFTP
DLKDRIRQSVCPTDLMSFTPPNF

>Anopheles gambiae_(XP_319732)
MDRSVDSIGSCSLDVDADSTDFSDTSGELNFLTPISGKDITREFTAGIKERCMLPVINCQAVTSVLDPVT
GHISTVLTTTTAPNGQQQQQQALQPLLSGTGDSSPTAATNKKPSYLNLACCVNGYSNLTTYDSKLRQDIN
KSREVSPSRPIIATLHYNRTETVNGGAAGRMRGMNGSILSPPSGGADMTDNVGMFNGNGSFAGSMRTKVI
SSQSQISSHARDEATAQLLMTPKKSFIQQRVEKLYGASEVVVLNKQNYSSSERKYLNNIANDNTSGGAPG
KLIHHGSTAQAGQQQQHHHHPMGNGTARNGADVESLPVLRHLRPEFRAQLQIFSPKKMPKPAAPPANQQD
HRTTVTNRSTMVVKNARISSHTTTTSSSTNGRTSNDSSFSSSNNNIVLDGNHFLRILKTEQNRLKAMADE
VEQEINLLKADGVEFGEDINGYILVAVGKARLLCSQKMKQFEDLCYTNLNQSPDEKFQTTGEDLRGFWDM
VMLQVNNVDASFAEIDSFRKNGWRKPAPKSPPPPARNGTAGATRTKLVKRPYKTGGTANGTSVTDGSNGV
EAKKASAALAAAKREAQRKQLMEMKRKQKLA

>Capitella teleta_(ELT90804)
MVIMADPVAIVTEDATSGYVRKMVSRFSESLQSEILNNGSEPSLVNHAPSSTPASKRTLTSHDPQPANGS
VDNLDHSVDSEAKSPSYVKLSCAVSGYSSNKYNAFNSEDKVKKTKPPLPHKPAPLRGASRTNGSDATSVS
LVSVTVDTDETDSCRVNGTSPRTPLVNGQRPQQHDNDVPTPQKLGICVLCPLCLFDVIKFDAGKRVSQQE
KPMSGAHVLHFERGLRQTLIMDGSHFLVVVQKESDRLHELCAKAELYLEAEDLPEEASGRIRATIGKANL
LTSKKFKQFRGLCQKNLEQADDEPFQTTAQDLAGFWDMVLTQVDDLDCKFAEIESLRSNGWISRNASSAS
LNNIEKSSNKAKHSGTKNKSCRQPSVACTPKSSLSASARAHRDEARRRLLQAKRAGRQRKVSQSDNKDDV
EVEIFVPQ

>Octopus bimaculoides_(XP_014770553)
MASSGPPSVLDRRSPHPDSKLVVDRITRRRSPVRANKGPCNDEVHSRIAQPYRTSFSAVVSLPRVRKTSN
EVLAANLALERSRSDQGSVSSKSSRRGISPRHVRYDDNVRVRHSDTEDSLMTSLSGQESSYSPLESPRDS
SDILSPISAMSSESDKSCPQLIVPSTGTLTNKPIHQETLRGIRKGCENNTLHPGDRLTPNNSVSFLPSES
SVHGGDSGVSTDVDSQREKTSKLNINKDYVYDRTISYRMAIQAQYSDEVKRLAEAKVVEKAPEEEAEVSN
PELKKSEVCAQSSLPSSQSNDSQKSTSQGAEPKRNDAHFLSHAKKSLSTSMGNFFRRLSPNVRRRTKHHK
RNSSTEGSAQSLNTSDSCSIESDPSGHPVHRSNSKSRRSFMKFFGKIHHRNKSHSLRSVGDRKPSKDDLE
CSQSQDIKQFDPSTQRILKSIEQNSMSDKTIYQKFKAKQPSNHTADAGNKSQAVRPLQATSKTLNNSPSG
ANQKNKENELKAMSLPLKPSLAEENELEANVSPQSNASTAGAASSVYATSSLSDQNQSIDDFSLAVHTPG
SEGTFATSPGSHTNNAAKTHLINNDSPGSLDLGSQSAPDSFIDIPSRKPSYLKLSCSVSGYGKYSVYSSY
KDIERRSPFSSSSSLKAESRSTSCEMPAIQSDNKEVTKSIDDSPSKDKELKADMDGSVPASSNGIDGIPE
SSVINITSTESSHVDGDYFLKLTLKEEEKLSKLCAETANDLDKHHLPDDVSGKIRAAIGKANLLQNKKFK
QFRELCQNYMTPVSSEEDHPTKLEDLQGFWEMVQIQIDDVYDMFAEIDLLRQNGWKEVHFESRRSSSSSQ
KSASVNSTPSHTPGNHITNSLPPKISPDGHLMVMDATGKIT

>Amphimedon queenslandica_(XP_003387885)
MSQARETQYKYKGRSKGVESRLKGKALDSKRQREELLSGRRRAALLEINEEIMAEEEIQEKGTGATGKKK
TSDDRKEELLKWKKERDRKKQKEQKKANAKPAFVVGKANISSNRKVPLPVEPSSTATVKEGPSKKGGKRV
TEPPGNAKREELGRKRVTGSRQPDKVQKPATRSSVRLQTAKSKETKAVKQSTKKQLTAESSKSTNSKREI
TKELSPHPPPCIDKSDLQRDSVLSSAWIPGAPPTPQCASQNARPNFEQFFSKQVFSPFQFKGQTGGETNT
GKAQSIFTFRKEFREEDIIKRIEDDSISNIEEEKREEKEAAVPATDDTGRGKSAKKEVKGGGRKRNLKKR
QNERTEKESDDEEDLETIDTGTKIGREEEPVDELPTNVRMSDTSIEAINVSVAHNNNYTNQQLEEERQSS
TDPPASAHTQDLSPPVTNFAPPTTDVTPPSEGLATDATGNDSGFDLVPFYKRHTDVTQRFNALCAEWDDK
LSQLEDDGEKEEDAGRPSDEVIGDIRTTVCQAKLLMRQKLKQYLELVQQAEDPAAEHKTMAEDLQGFWDM
VYFQVESIYKKFSSFSEMESNSWKTVVTPAEDGDSTTCKPRRSKRGASKAKRGGSKTGASSAISERLKAA
RAKREAERLKIREMRQAFAAASKTKEEEEVPPAPVTPEKKDNIAESPFPDLISFSPAPSSLRPPTTPKSL
LKTPQSASKSKSSLKKRLSSIPDPVFEVDKSHETEGAFMTKLVQINASKKMIKDFDTNLIMSPARRSKRL
STRRRSELIVSSISEIPNDRNNVAYLPNEALID

E2F Transcription Factor-E2F

>Homo sapiens_E2F1(ENSP_345571)
MALAGAPAGGPCAPALEALLGAGALRLLDSSQIVIISAAQDASAPPAPTGPAAPAAGPCD
PDLLLFATPQAPRPTPSAPRPALGRPPVKRRLDLETDHQYLAESSGPARGRGRHPGKGVK
SPGEKSRYETSLNLTTKRFLELLSHSADGVVDLNWAAEVLKVQKRRIYDITNVLEGIQLI

AKKSKNHIQWLGSHTTVGVGGRLEGLTQDLRQLQESEQQLDHLMNICTTQLRLLSEDTDS
QRLAYVTCQDLRSIADPAEQMVMVIKAPPETQLQAVDSSENFQISLKSKQGPIDVFLCPE
ETVGGISPGKTPSQEVTSEEENRATDSATIVSPPPSSPPSSLTTDPSQSLLSLEQEPLLS
RMGSLRAPVDEDRLSPLVAADSLLEHVREDFSGLLPEEFISLSPPHEALDYHFGLEEGEG
IRDLFDCDFGDLTPLDF

>Gorilla gorilla_E2F1(ENSGGOP_3528)
MALAGAPAGGPCAPALEALLGAGALRLLDSSQIVIISAAQDASAPPAPTGPAAPAAGPCD
PDLLLFATPQAPRPTASAPRPALGRPPVKRRLDLETDHQYLAESSGPARGRGRHPGKGVK
SPGEKSRYETSLNLTTKRFLELLSHSADGVVDLNWAAEVLKVQKRRIYDITNVLEGIQLI
AKKSKNHIQWLGSHTTVGVGGRLEGLTQDLRQLQESEQQLDHLMNICTTQLRLLSEDTDS
QRLAYVTCQDLRSIADPAEQMVMVIKAPPETQLQAVDSSETFQISLKSKQGPIDVFLCPE
ETVGGISPGKTPSQEATSEEENRATDSATIVSPPPSSPPSSLTTDPSQSLLSLEQEPLLS
RMGSLRAPVDEDRLSPLVAADSLLEHVREDFSGLLPEEFISLSPPHEALDYHFGLEEGEG
IRDLFDCDFGDLTPLDF

>Macaca mulatta_E2F1(ENSMMUP_30996)
MALAGAPAGGPCAPALEALLGAGALRLLDSSQIVIISAAQDASAPPAPTGPAAPAAGPCD
PDLLLFATPQAPRPTPSAPRPALGRPPVKRRLDLETDHQYLAESSGPARGRGRHPGKGVK
SPGEKSRYETSLNLTTKRFLELLSRSADGVVDLNWAAEVLKVQKRRIYDITNVLEGIQLI
AKKSKNHIQWLGSHTTVGVSGRLEGLTEDLRQLQESEQQLDHLMNICTTQLRLLSEDTDS
QRYPWIGRRDLRSIADPAEQMVMVIKAPPETQLQAVDSSETFQISLKSKQGPIDVFLCPE
ETVGGISPGKTPSQEATSEEENRATDSATIVSPPPSSPPSSLTTDPSQSLLSLEQEPLLS
RMGSLRAPVDEDRLSPLVAADSLLEHVREDFSGLLPEEFISLSPPHEALDYHFGLEEGEG
IRDLFDCDFGDLTPLDF

>Mus musculus_E2F1(ENSMUSP_99434)
MAVAPAGGQHAPALEALLGAGALRLLDSSQIVIISTAPDVGAPQLPAAPPTGPRDSDVLL
FATPQAPRPAPSAPRPALGRPPVKRRLDLETDHQYLAGSSGPFRGRGRHPGKGVKSPGEK
SRYETSLNLTTKRFLELLSRSADGVVDLNWAAEVLKVQKRRIYDITNVLEGIQLIAKKSK
NHIQWLGSHTMVGIGKRLEGLTQDLQQLQESEQQLDHLMHICTTQLQLLSEDSDTQRLAY
VTCQDLRSIADPAEQMVIVIKAPPETQLQAVDSSETFQISLKSKQGPIDVFLCPEESADG
ISPGKTSCQETSSGEDRTADSGPAGPPPSPPSTSPALDPSQSLLGLEQEAVLPRMGHLRV
PMEEDQLSPLVAADSLLEHVKEDFSGLLPGEFISLSPPHEALDYHFGLEEGEGIRDLFDC
DFGDLTPLDF

>Rattus norvegicus_E2F1(ENSRNOP_22428)
MAVAPAGGQHAPALEALLGAGALRLLDSSQIVIISTAPDVGAPQVPTGPAAPPAGPRDPD
VLLFATPQAPRPAPSAPRPALGRPPVKRRLDLETDHQYLAGSSGPFRGRGRHPGKGVKSP
GEKSRYETSLNLTTKRFLELLSHSADGVVDLNWAAEVLKVQKRRIYDITNVLEGIQLIAK
KSKNHIQWLGSRTMVGIGQRLEGLTQDLQQLQESEQQLDHLMHICTTQLQLLSEDSDIQR
LAYVTCQDLRSIADPAEQMVIVIKAPPETQLQAVDSAETFQISLKSKQGPIDVFLCPEES
AEGISPGRTSYQETSGEDRNADSGTAGPPPSPPSTSPTLDPSQSLLGLEQEAVLPRIGNL
RAPMEEDRLSPLVAADSLLEHVKEDFSGLLPGEFISLSPPHEAVDYHFGLEEGEGIRDLF
DCDFGDLTPLDF

>Felis catus_E2F1(ENSFCAP_4643)
LPQVKRRLDLETDHQYLAESSGPARGRGRHPGKGVKSPGEKSRYETSLNLTTKRFLELLS
RSADGVVDLNWAAEVLKVQKRRIYDITNVLEGIQLIAKKSKNHIQWLGSHAAVGIGGRLE
GLTQDLRQLQESERQLDHLIHICTAQLRLLSEDSDSQRSTLAYVTCQDLRSIADPAEQMV
MVIKAPPETQLQAIDSSETFQISLKSKQGPIDVFLCPEESAGGISPGKTSSQGTASGEED
RAVDPATTVPPPSSPSSSPAADPSQSLLSLEQEPLLSRMGGLRAPVDEDRLSPLVAADSL
LEHVREDFSGLLPEEFISLSPPHEALDYHFGLEEGEGIKDLFDCDFGDLTPLDF

>Equus caballus_E2F1(ENSECAP_21291)
AAPPRSAAPPVKRRLDLETDHQYLAESSGPTRGRGRHPGKGVKSPGEKSRYETSLNLTTK
RFLELLSRSADGVVDLNWAAEVLKVQKRRIYDITNVLEGIQLIAKKSKNHIQWLGSHAAV
GIGGRLEGLTQDLRQLQESERQLDHLIHICTTQLRLLSEDTDSQRYPCTAWRAADLRSIA
DPAEQMVMVIKAPPETQLQAVDSSETFQISLKSKQGPIDVFLCPEESAGGISPGKTLSQG
TASGEEDRAANPATTVPPPSSPSSSPATDPSQSLLSLEQEPLLSRMGGLRAPVDEDRLSP
LVAADSLLEHVREDFSSLLPEEFISLSPPHEALDYHFGLEEGEGIRDLFDCDFGDLTPLD

F

>Myotis lucifugus_E2F1(ENSMLUP_14006)
ATGVCGGVGGQALRACLGRARGGLLDSWQIVIISIAQDTSAPLPPAPAVRAASPRDPDLL
LFATPQAPRPTPSPRPALGRPPVKRRLDLETDHQYLAESSGLARGRGRHPGKGVKSPGEK
SRYETSLNLTTKRFLELLSRSADGVVDLNWAAEVLKVQKRRIYDITNVLEGIQLIAKKSK
NHIQWLGNHATVGIGGQLEGLTKDLQQLQENERQLDHLIHVCTTQLQLLSEDADSQRSTL
AYVTCQDLRSIADPAEQMVMVIKAPPETQLQAMDSSEAFQISLKSKQGPIDVFLCPEESA
GGISPRKTPSQGATSGEEDRAADPTPTVPPSSSLPSTPDVDPSQPLLSLEQEPLLSRMGG
LRAAMDEDHLSPLVAADSLLEHVREDFSSLLPEEFISLSPPHGALDYHFGLEEGEGIRDL
FDCDFGDLTPLDF

>Erinaceus europaeus_E2F1(ENSEEUP_9096)
VKRRLDLETDHQYLAESSGPPRGRGRHPGKGVKSPGEKSRYETSLNLTTKRFLELLSHSS
DGVVDLNWAAEVLKVQKRRIYDITNVLEGIQLITKKSKNHIQWLGNHAAVGIGGRLEELT
QDLQQLQESERQLDHLIHICTTQLQLLSEDSDSQRYPGLDLRSIADPAEQTVMVIKAPPE
TQLQAVDSAETFQISLKSKQGPIDVFLCPEETVGGASPGKTPSQGTASGEEDRTADAATT
VPQPPSPAGDPSPSLLSLEQEPLVPRMGCLRAPVDEDRLSPLVAADSLLEHVREDFSGLP
EEFISLSPPLEALDYHFGLEEGEGIKDLFDCDFGDLTPLDF

>Gallus gallus_E2F1(ENSGALP_4806)
PQVKRKLNLETDHQYIAESLPAARGRARIPGRGAKSPGEKSRYETSLNLTTKRFLELLSQ
SPDGVVDLNWAAEVLKVQKRRIYDITNVLEGIQLITKKSKNNIQWLGSQVAAGASSRQRL
LEKELRDLQAAERQLDDLIQTCTVQLRLLTEDPSNQSFAYVTCQDLRSIVDPSEQMVMVI
KAPPETQLQVSDPGEAFQVSVRSTQGPIDVFLCPEDSSGVCSPVKSPFKAPAEELSPGSS
QQRASPLLHSAQDVNMLLPEALLPGTALPTKCPTEDVSLSPLASMDTLLEHGKDDFPGFL
ADEFIALSPPQPQDYHFGLEEGEGISELFDCDFGDFTHLDF

>Anolis carolinensis_E2F1(ENSACAP_8599)
FQELQFIGWKILLFDFKVAISRNWLKSTHPLERVKRKLNLETDHQYIAESFQGNRGRARN
AVKGVKSPGEKSRYETSLNLTTKRFLELLSQSPDGVVDLNWAADVLKVQKRRIYDITNVL
EGIQLITKKSKNHIQWLGSRSTVGGPSNCHGLMKELQDLQDAEQQLDTLIQMCTTQFKLL
TEDLENKRKAYVTCQDLRSVVDPSEQLVMVIKAPPETQMQVSDPAEAFQIAVKSTQGPID
VFLCPEESSEVCSPTKSPQKVAVGQRTPESSQAAAVVTTAATPVLLHPSQAEPLLSGENT
LPLKCPGDDVSLSPLASVDTLLQQGKDDFSSFLPAEFIALSPPQTQDYHFGLEEGEGISE
LFDCDFGDFTSLDF

>Xenopus tropicalis_E2F1(ENSXETP_46332)
ALWEAGTFQVAEQQIVIISTPDVPAEAAGNPGLSEAELMLFTTPQGPSTSYTAQRPDLGR
PPVKRKLDLETVSRYNQEALQTPRGKGKRPLKAVKSPGERSRYDTSLHLTTKRFLELLSQ
SSDGVVDLNWAAQVLNVQKRRIYDITNVLEGIHLITKKSKNHIQWLGYTSYAEYNSRYQS
TLKDCQKLEDQEKQLDKLIHMANTQLKLFKEEILDTQQCTFGYVTCQDLRSIADPSERML
MVIRYPPDTDMCVSDPAEAFQMSLKSTQAPIDVFLCPDDSSGVCSPVTSPTKTSQAEPSS
INQAEPLPSVQPKEESPASIPMLDIGLGLLSDMQEPFLPPENEIPLDSTLNCLPLSPSNL
LLDYRDAMPDFLPNDFISLSPASSQEYSFGLQTCEGAAELFNFDCDFSGLTALDL

>Danio rerio_E2F1(ENSDARP_137549)
MSEGFISGQTSEDLLADFESLLNNGSIDLSEDHQIVIISTPGTATTSTATGDILLFATPH
HTSTGTTSLPDHRRPILGRPPVKRKLDLDSDHQYICTSRTASNGPTPPATPAPPRVPKLA
VEKSRYDTSLNLTTKRFLDLLAQSPDGVVDLNWASQVLDVQKRRIYDITNVLEGIHLISK
KSKNNIQWLGNRIDGASLARFQELQKEVSELTEAEEKLDELINKCSLQLRLLTEDTHNKK
YPLRYVRCQDLRNTVDPPDQIIMVIRAPAETAMQVSEPSEGYQISLKSSKGPIDVFLCPE
DSSGVCSPVTDCSPAKSSNQSPAKSDPASQEVTSTPTTLPSSSPLPLHTDSESFLDSDPF
SGICAMPDFDLSPLDSDFLLERSGAADGLGLPLDSFICLSPPHSQDYHFGLEDHEGVSEL
FDCDFSDLVPLEF

>Takifugu rubripes_E2F1(ENSTRUP_28243)
ETLLNSGSIDLSQDHQIVIITSPSNEGLHPGSAPTSTGEILLFATPQGPADVGLHDKRRP
ALGRPPVKRKLDLDSDHQYVSTTRPCIGQAPSTPAPPRVPRNTTEKSRYDTSLNLTTKRF
LNLLSQSADGVVDLNWASQVLDVQKRRIYDITNVLEGIHLISKKSKNNIQWLGNRVDTAL
VSRHKELQKEVCDLTEAEEQLDQLISKCSLQLRLLTEDPQNNTWGYVRCQDLKRSFDSPD

QLLMVIRAPPETQMQVSEPSKGFEMSLKSTQGPIDVFLCPEDSSGACSPVSGSSPTKTNT
EPSLVPPVAQPTDQSNASGSTATLQLHHWPVSSAGPSLHAPVPDMPDFDLSPLSAADFLN
GEGFSLPLDGFINLSPPHSHDYHFGLEDHEGISELFDCDFGDLTHVLG

>Tetraodon nigroviridis_E2F1(ENSTNIP_7715)
LITGQTSEDILADFETLLNSGSIDLGEDHQIVIITSPGNEGLHPGSAPTSTGEILLFATP
QGPADVGLQDKRRPALGRPPVKRKLDLDSDHQYVSTTRPCIGQAPPSTPAPPRVPRNLTE
KSRYDTSLNLTTKRFLNLLSQSADGVVDLNWASQVLDVQKRRIYDITNVLEGIHLISKKS
KNHIQWLGNRVDTALVSRHKELQKEVCDLAEAEEQLDQLVSKCSLQLRLLTEDPQNNTWG
YVRCQDLKKSVDSPDQLLMVIRAPPETQMQVSEPSKGFEMSLKSTQGPIDVFLCPEDSSG
ACSPVTGSSPAKATGPSLVPEQTTDQSKTRGSTALLEESSSSPASTSSTVTAASQQDSSS
LPMGNDSGYVSDMPNFDLSPLSAADFLNGEGFPLPLDGFINLSPPHSHDYHFGLEDHEGI
SELFDCDFGDLTHVLE

>Gasterosteus aculeatus_E2F1(ENSGACP_6233)
EILLFATPQGSVDVGVQDKRRPALGRPPVKRKLDLDSDHQYVSTTRPSIGQAPPSIPRTA
TEKSRYDTSLNLTTKRFLNLLSQSADGVVDLNWASQVLDVQKRRIYDITNVLEGIQLISK
KSKNNIQWLGNRVDAALVSRQKDLHGEVCDLKDAEEQLDQLISKCNLQLRLLTEDTQNNT
LGYVRCQDLKKSFDSPDQLVMVIRAPPETQMQVSEPSKSYQVSLKSTRGPIDVFLCPEES
SGVCSPVTGGSPSKPDPLLVPPPARPTDQSQARNSTAAQDETLLSPESLLGGDPFSGLGE
MPDFNLSPISSSDFLNADGIPLPLDGFINLSPPHSHDYHFGLEDHEGISELFNCDFDDLS
QVLG

>Oryzias latipes_E2F1(ENSORLP_1148)
CCRSPLGGRQCHDFETLLNSGSIDLSQDHQIVIITSPSNEGLHPLAAPSSTGEILLFATP
QSHADAGGQDRRRPALGRPPVKRKLDLDSDHQYVSTTRSSASQAPPSTPAPPRVPRSTAE
KSRYDTSLNLTTKRFLNLLSQSADGVVDLNWASQVLDVQKRRIYDITNVLEGIQLISKKS
KNHIQWLGNRIDASMVSRHKELQREACDLTEAEEQLDELIAKCNLQLRLLTEDPQNKKLG
YVRCQDLRQSFDSPDQLVMVIRAPPETQMQVSEPSEGYQVSLKSTRGQIDVFLCPEDSSG
VCSPVTGTSPSKTGADPSLASPLLPHPAEPPPQFPFPPTESLLGGDPFSSLGDMADFDLS
PLSSSDFLNGEGLPLALDNFINLSPPQSHDYHFGLEDHEGISELFDCDFDELSHVLG

>Homo sapiens_E2F2(ENSP_489612)
MLQGPRALASAAGQTPKVVPAMSPTELWPSGLSSPQLCPATATYYTPLYPQTAPPAAAPG
TCLDATPHGPEGQVVRCLPAGRLPAKRKLDLEGIGRPVVPEFPTPKGKCIRVDGLPSPKT
PKSPGEKTRYDTSLGLLTKKFIYLLSESEDGVLDLNWAAEVLDVQKRRIYDITNVLEGIQ
LIRKKAKNNIQWVGRGMFEDPTRPGKQQQLGQELKELMNTEQALDQLIQSCSLSFKHLTE
DKANKRLAYVTYQDIRAVGNFKEQTVIAVKAPPQTRLEVPDRTEDNLQIYLKSTQGPIEV
YLCPEEVQEPDSPSEEPLPSTSTLCPSPDSAQPSSSTDPSIMEPTASSVPAPAPTPQQAP
PPPSLVPLEATDSLLELPHPLLQQTEDQFLSPTLACSSPLISFSPSLDQDDYLWGLEAGE
GISDLFDSYDLGDLLIN

>Pongo abelii_E2F2(ENSPPYP_2014)
MLQGPRALASAAGQTPKVVPAMSPTELWPSGLSSPQLCPATATYYTPLYPQTAPPAAAPG
TCLDATPHRPEGQVVRCLPAGRLPAKRKLDLEGIGRPAVPEFPTPKGKCIRVDGLPSPKT
PKSPGEKTRYDTSLGLLTKKFIYLLSESEDGVLDLNWAAEVLDVQKRRIYDITNVLEGIQ
LIRKKAKNNIQWVGRGMFEDPTRPGKQQQLGQELKELMNTEQALDQLIQSCSLSFKHLTE
DKANKRLAYVTYQDIRAVGNFKEQTVIAVKAPPQTRLEVPDRTEDNLQIYLKSTQGPIEV
YLCPPAVLDKDSPSEEPLPSTSTICPSPDSAQPSSSTDPSIMEPTASSLPAPAPTPQQAP
PPPSLVPLEATDSMLELPHPLLQQTEDQFLSPTLACSSPLISFSPSLDQDDYLWGLEAGE
GISDLFDSYDLGDLLIN

>Macaca mulatta_E2F2(ENSMMUP_15528)
MLQGPRALVSAAGQPPKVVPAMSPTELWPSGLSSPQLCPATTTYYTPLYPQTAPPAVAPG
TCLDATPHGPEGQVVRCLPAGRLPAKRKLDLEGIGRPAVPEFPTPKGKCIRVDGLPSPKT
PKSPGEKTRYDTSLGLLTKKFIYLLSESEDGVLDLNWAAEVLDVQKRRIYDITNVLEGIQ
LIRKKAKNNIQWVGRGMFEDPTRPGKQQQLGQELKELMNIEEALDQLIQSCSLSFKHLTE
DKANKRLAYVTYQDIRAVGNFKEQTVIAVKAPPQTRLEVPDRTEDNLQIYLKSTQGPIEV
YLCPEEVQEPDSPSEEPLPSTSTLCPSPDSAQPSSSTDPSIMEPTASSVPAPAPTPQQAP
PPPSLVPLEATDSMLELPHPLLQQTEDQFLSPTLACSSPLISFSPSLDQDDYLWGLEAGE
GISDLFDSYDLGDLLIN

>Mus musculus_E2F2(ENSMUSP_50047)
MLRAPRTLAPATAQPTKSLPALNPTELWPSGLSSPQLCPATTATTYYTSLYTQTVPSSVA
LGTCLDATPHGPEGQIVRCAPAGRLPAKRKLDLEGIGRPTVPEFRTPKGKCIRVDGLPSP
KTPKSPGEKTRYDTSLGLLTKKFIYLLSESEDGVLDLNWAAEVLDVQKRRIYDITNVLEG
IQLIRKKSKNNIQWVGRELFEDPTRPSRQQQLGQELKELMNAEQTLDQLIQSCSLSFKHL
TEDNANKKLAYVTYQDIRAVGNFKEQTVIAVKAPPQTRLEVPDRAEENLQIYLKSTQGPI
EVYLCPEEGQEPDSPAKEALPSTSALSPIPDCAQPGCSTDSGIAETIEPSVLIPQPIPPP
PPPPLPPAPSLVPLEATDNMLELSHPLLQQTEDQFLSPILAANSPLISFSPPLDQDEYLW
GMDEGEGISDLFDSYDLGDLLIN

>Rattus norvegicus_E2F2(ENSRNOP_67839)
MGCGLVSPAGVSQAWRERGGAAGPQLLVLKTLGKDSLCLSKERMTSSPFRGPWILELEPK
RGGGVFVLFPNVGTSLGCGTEPGSPLGLGGCRSHPDKGQLWGCDGHQAKRKLDLEGLGRP
TVPEFRTPKGKCIRVDGLPSPKTPKSPGEKTRYDTSLGLLTKKFIYLLSESEDGVLDLNW
AAEVLDVQKRRIYDITNVLEGIQLIRKKSKNNIQWVGRGIFEDPTRPAKEQQLGQELKEL
MNAEQTLDQLIQSCTLSFKHLTEDNANKKLAYVTYQDIRAVGNFKEQTVIAVKAPPQTRL
EVPDRAEENLQIYLKSTQGPIEVYLCPEEGQEADSPTKEALPSTSTLSPVPDCAQPGCST
DSGLAETIESSVLMPQPVPPPLPPPPPAPSLVPLEATDNMLELSHPLLQQTEDQFLSPIL
TASSPLISFSPPLDQEEYLWGMDEGEGISDLFDSYDLGDLLIN

>Canis familiaris_E2F2(ENSCAFP_19556)
MLRGPRAPAAAAGPPSKALPAMSPTEPWPPGLSSPQLCPATATYCTSLYPQTVPPPAAAG
TCLDATPHGPEGQAVRCVPAGRLPAKRKLDLEGIGRPAIPEFRTPKGKCIRVEGLPSPRT
PKSPGEKTRYDTSLGLLTKKFIYLLSESKDGVLDLNWAAEVLDVQKRRIYDITNVLEGIQ
LIRKKAKNNIQWVGRGMFEDPTRPGKQQQLGQELKELMSMEQALDQLIQSCSLNFKHLTE
DKANKRLAYVTYQDIRAVGNFKEQTVIAVKAPPQTRLEVPDRNEENLQIYLKSTQGPIEV
YLCPEEVQDPDSPAKERLPPASTLGPSPDSTQPSSSTDPGMTDPVASSASHPPPAPPPPP
PPPPLVPLEATDSMLELPHPLLQQTEDQFLSPTLPCSSPLISFSPPLDQDDYLWGLDGGE
GISDLFDSYDLGDLLIN

>Felis catus_E2F2(ENSFCAP_10812)
MLRGPRALAPAAGPPSKGLPAMSPTELWPPGLSSPQLCPATTTYYTSLYPQTVPPTAAPG
TCLDATPHGPEGQAVRCVPAGRLPAKRKLDLEGIGKPAVPEFRTPKGKCIRLDGLPSPKT
PKSPGEKTRYDTSLGLLTKKFIYLLSESKDGVLDLNWAAEVLDVQKRRIYDITNVLEGIQ
LIRKKAKNNIQWVGRGMFEDPTRPGKRQQLGQELKELMSTEQALDQLIQTCSLNFKHLTE
DKANKRLAYVTYQDIRAVGSFKEQTVIVVKAPPQTRLEVPDRSEENLQIYLKSTQGPIEV
YLCPEEVQEPDSPTKDPFPSPSPLGPIPDSTQPSSSTDPGMTEPMASPGPALTPQQALQL
PAPPPPPLPLVPLEATDSMLELPHPLLQQTEDQFLSPTLPCSSPLISFSPPLDQDDYLWG
LDGGEGISDLFDSYDLGDLLIN

>Myotis lucifugus_E2F2(ENSMLUP_8054)
MLRGPRAPAPAAALPLKGLPALSPTELWPPGLGSPQLCPVTTAYYTSLYPQTVPPATVPG
TCLDATPHGPEGQAVRCVPAGRLPAKRKLDLEGIGRPAVLEFQTPKGKLIRVDGLPSPKT
PKSPGEKTRYDTSLGLLTKKFIYLLSESKDGVLDLNWAAEVLDVQKRRIYDITNVLEGIQ
LIRKKAKNNIQWVGRGALEDPTRPGKQQQLGQELKELMSMEQALDQLIQSCSLNFKHLTE
DKANKKLAYVTYQDIRAIGTFKEQTVIAVKAPPQTRLEVPDRNEENLQIYLKSTQGPIEV
YLCPEEMQEPDSPAKEPVPSTSTLSPTPDSAQPCSSTNPGILEPTASPDLSPAPSLTSQQ
ALLPPPSLVPLEATDSMLELPQPLLQQTEDQFLTDSSPLISFSPPLDQDDYLWGLDGGEG
ISDLFDSYDFGDLLIN

>Monodelphis domestica_E2F2(ENSMODP_15986)
MLRIPKGVSPAPGGSAPAAPQKPPQPLQIKKALPVVSPVWTPDVRSPQGGPVPASHFTPL
YPPMVAAVAPAAPGSCLDATPQGPEIRASRASAGRLPAKRKLDLEGMGRQGLSEFRTPKG
KCGGGEGLPSPKTPKSPGEKTRYDTSLGLLTKKFIYLLSESEDGVLDLNWAAEVLEVQKR
RIYDITNVLEGIQLIRKKAKNNIQWVGRGMFEDPAGPRKQQSLGQELKDLSDTERVLDQL
IQSCTSDLKHLTEDETNQRYPSWRRGPRMGGDIRAIGNFKDQTVIAVKAPPETRLEVPDL
REENLQIYLKSTNGPIEVYLCPEDNREAGGSSQEAPPSDPGPFPGRSSSPSSSCSLDLIA
PPGRCLAIGAFFLSVWA

>Pelodiscus sinensis_E2F2(ENSPSIP_17158)

MLRVPKGVSSAPQQQKKIMPVMSSADLVTSSLSSPQMHSIPASYFMQVYPQTVAAAPPPR
GSCLYATPHGPEIKAIRSASAGRLPAKRKLDLEGTGQPGLPEFRTPKGKGRMVTRIPSPR
TTKAPKSPGEKTRYDTSLGLLTKKFIHLLSESEDGVLDLNRAAEVLEVQKRRIYDITNVL
EGIQLIRKKSKNNIQWMGTGIFEDTSVTVKQQSLRQELAELAKTERKVDELIQDCTLQLK
HLTEDETNQRLAYVTYQDIRAIGNFQEQTVIAVKAPPETKLEVPDFSEENVQLHLKSTNG
PIEVYLCPEEILDDSPAKESSFLTPSLQDSSDGTDPSPGKGKGGLWPGLAWSPLPSPVLW
GLTFPFVAAAAPLMEVEDGLLDVPHHLLQQTEDQLPYGDPAPFVSFSPPLDQDDYLWGLE
GGEGVSDLFEAYDLGDLLKN

>Takifugu rubripes_E2F2(ENSTRUP_29627)
AKRKLDLEDPLYMPEFRTPKGKCNIAARLTSPRTPKSPGERTRYDTSLGLLTKKFVGLIA
ESPDGVLDLNWATEVLEVQKRRIYDITNVLEGVQLIRKKSKNHIQWLVGDVFEGGTGGGQ
KACTLRKEIGDLEQAEKCLDELILSSTGQLKQLTEHGDNQRLGYVTYHDIRSIGSLQDQT
VIAVKAPADTKLEVPDTEGESLQIYLKSKNGPIEVYLCPEDGLEDASPVKSVSTPKKEFP
PTVCTSHTTSTAPPSCSIKEEPVEFTIRIPAHIPAAAAASSSSILDVEGLLGLPPSLLQI
TEDQLPSATFTPDPNTPFVSFSPPLDHDDYLWSLDDGEGVSDFFDTYDLGDLLSN

>Tetraodon nigroviridis_E2F2(ENSTNIP_20402)
MMRMPKGVSAASGRPAAGLSCSQTKMKLLSAGVKTEFFSAGLSSPLMNAVPAGYFTQICN
TTAAEQRANSLYSTPHGPEAKPIRASSGRMPAKRKLDLEDPLYMPEFRTPKGKCNIAARL
TSPRTPKSPGERTRYDTSLGLLTKKFVGLIAESPDGVLDLNWATEVLEVQKRRIYDITNV
LEGVQLIRKKSKNHIQWLVGDVFEGGAGGGQKAGALRKELGDLERAEKCLDELILSSTAQ
LKQLTEYEDNQRLGYVTYQDIRSIGSFQDQTVIAVKAPADTKLEVPDTEGQGSLQIYLKS
KNGPIEVYLCPEEALEDASPVKSASTPKKDILQTICPPAPATSSTAPPSCSIKEEPVESK
PSSAHVAAASSASILDVEGLLGLPPSLLQITEDQLPSASFAPDPNTPFVSFSPPLDHDDY
LWSLDDGEGVSDFFDSYDLGDLLSN

>Gasterosteus aculeatus_E2F2(ENSGACP_12624)
MMRMPKGVSPAPGRQKIKVVSTAGGKHEFFSTGLSSPPMSTVPPGYFTQICNTAAGEQRA
NSLYSTPHGPEAKPIRSSCGRLPAKRKLDLEDPLYLPEFRTPKGKCSIAARIPSPRTPKS
PGERTRYDTSLGLLTKKFVGLIAESPDGVLDLNWATEVLEVQKRRIYDITNVLEGVQLIR
KKSKNNIQWLVGDVFEGGASGGEKACALRKELGELERVERSLDDLVQSSTAQLKQLTEYE
DNKRYPLRCTLGYVTYQDIRSIGTLRDQTVIAVKAPADTKLEVPDTAGQGSLQIYLKSKN
GPIEVYLCPEEGLEDASPGKSAATPIKEFSKPLDPPTATAMAPPSYSAKEEPAEPTLLLE
AVDSLCPKELINGLLGLPPSLLQLTDDQLPCTSFAPDPNAPFVSFSPPLDHDDYLWSLED
SEGVSDFFDTYDLGELLKS

>Homo sapiens_E2F5(ENSP_398124)
MAAAEPASSGQQAPAGQGQGQRPPPQPPQAQAPQPPPPPQLGGAGGGSSRHEKSLGLLTT
KFVSLLQEAKDGVLDLKAAADTLAVRQKRRIYDITNVLEGIDLIEKKSKNSIQWKGVGAG
CNTKEVIDRLRYLKAEIEDLELKERELDQQKLWLQQSIKNVMDDSINNRFSYVTHEDICN
CFNGDTLLAIQAPSGTQLEVPIPEMGQNGQKKYQINLKSHSGPIHVLLINKESSSSKPVV
FPVPPPDDLTQPSSQSLTPVTPQKSSMATQNLPEQHVSERSQALQQTSATDISSAGSISG
DIIDELMSSDVFPLLRLSPTPADDYNFNLDDNEGVCDLFDVQILNY

>Gorilla gorilla_E2F5(ENSGGOP_9511)
MAAAEPASSGQQAPAGQGQGQRPPPQPPQAQAPQPPPPPPLGGAGGGSSRHEKSLGLLTT
KFVSLLQEAKDGVLDLKAAADTLAVRQKRRIYDITNVLEGIDLIEKKSKNSIQWKGVGAG
CNTKEVIDRLRYLKAEIEDLELKERELDQQKLWLQQSIKNVMDDSINNSTFSYVTHEDIC
NCFNGDTLLAIQAPSGTQLEVPIPEMGQNGQKKYQINLKSHSGPIHVLLINKESSSSKPV
VFPVPPPDDLTQPSSQSLTPVTPQKSSMATQNLPEQHVSERSQALQQTSATDISSAGSIS
GDIIDELMSSDVFPLLRLSPTPADDYNFNLDDNEGVCDLFDVQILNY

>Mus musculus_E2F5(ENSMUSP_29069)
MAAAEPTSSAQPTPQAQAQPPPHGAPSSQPSAALAGGSSRHEKSLGLLTTKFVSLLQEAQ
DGVLDLKAAADTLAVRQKRRIYDITNVLEGIDLIEKKSKNSIQWKGVGAGCNTKEVIDRL
RCLKAEIEDLELKERELDQQKLWLQQSIKNVMEDSINNRFSYVTHEDICNCFHGDTLLAI
QAPSGTQLEVPIPEMGQNGQKKYQINLKSHSGPIHVLLINKESSSSKPVVFPVPPPDDLT
QPSSQSSTSVTPQKSTMAAQNLPEQHVSERSQTFQQTPAAEVSSGSISGDIIDELMSSDV
FPLLRLSPTPADDYNFNLDDNEGVCDLFDVQILNY

>Rattus norvegicus_E2F5(ENSRNOP_44464)
MAAAEPTSSAQPTPQAQAQPPPPPPHGAPSSHQSAALAGGSSRHEKSLGLLTTKFVSLLQ
EAQDGVLDLKEAADTLAVRQKRRIYDITNVLEGIDLIEKKSKNSIQWKGVGAGCNTKEVI
DRLRCLKAEIEDLELKERELDQQKLWLQQSIKNVMEDSINNRFSYVTHEDICSCFNGDTL
LAIQAPSGTQLEVPIPEMGQNGQKKYQINLKSHSGPIHVLLINKESNSSKPVVFPVPPPD
DLTQPSSQSSTSVTPPKSTMAAQNLPEQHVSERSQNFQQTPATEISSGSISGDIIDELMS
SDVFPLLRLSPTPADDYNFNLDDNEGVCDLFDVQILNY

>Myotis lucifugus_E2F5(ENSMLUP_5617)
MAAAEPASSGQSAPQGQAQVQWAPPASPAPPPQLGARGGARGRRRGCGPRCTRLFIRQLQ
GAGMGVPDAGEAADTLAVRQKRRIYDITNVLEGIDLIEKKSKNSIQWKGVGAGCNTKEVL
ERLRDLRAEVEDLDLKERELDQQKSWLQQSIRNVMDDTINSRRRTFSYVTHEDICNCFHG
DTLLAIQAPAGTQLEVPLPEMGQNGQKKYQINLKSHSGPIHVLLINKESSSSQPVVFPVP
PPDDLTQPPSQPSTPQTPHKPSPTPPSPPGPPASERSPSLQHIPATDSASGSVTGDLIDE
LMSSDVFPLLRLSPTPADDYNFNLDDSEGVCDLFDVQILNY

>Monodelphis domestica_E2F5(ENSMODP_7364)
MAAAAELGSSGQQQPPGQGQPPQQPPAAPPGNGGGGSSSRHEKSLGLLTTKFVSLLQEAK
DGVLDLKAAADTLAVRQKRRIYDITNVLEGIDLIEKKSKNSIQWKGVGAGCNTKEVIDRL
RYLKAEIEDLELKERELDQQKLWLQQSIKNVMDDSINNRYPFISISVTFSYVTHEDICNS
FNGDTLLAIRAPSGTQLEVPIPEMGQNGQKKYQINLKSHTGPIHVLLINKDSSSSKPVVF
PVPPPDDLTQPPSQVSTPVSPQKPNTETQNLPEQHTLTIGQHLQQTPVSDTPSGSISGDI
IDELMSSDVFPLLRLSPTPADDYNFNLDENEGVCDLFDVQILNY

>Gallus gallus_E2F5(ENSGALP_30213)
MAAAAEAAGGGGSSRHEKSLGLLTTKFVSLLQEAKDGVLDLKVAADALAVRQKRRIYDIT
NVLEGIDLIEKKSKNSIQWKGVGAGCNTKEVVDRLRYLEAEIEDLELKEKELDQQKLWLQ
QSIKNVMDDSTNHQFSYVTHEDICNCFNGDTLLAIQAPCGTQLEVPIPEMGQNGQKKYQI
NLKSSSGPIHVLLINKESNSSKPMVFPVPPPDDLAQTPSQPVAPVTPLKPTPASENPPEH
GLNQGQQLPETTVDTPSESSLQQNSTTPATPYSSLPDSVLYPSLSGDVTQATTSSNDYQA
LLPLDVNCILKPNSFDIAKMEEPAGNIGGDIIDELMSSDGKYLASLLAVIKKVVAWFLYE
QHSW

>Anolis carolinensis_E2F5(ENSACAP_13864)
ASPFALLCSLSLSFFYQAADTLAVRQKRRIYDITNVLEGIDLIEKKSKNSIQWKGVGAGC
NTKEIVDRLRYLEAEIEDLELKEKELDQQKIWLQQSIKNVKEDSENKHFSYITHEDICDC
FNGDTLLAIQAPSGTQLEVPVPQVGQKKYQINLKSNSGPIHVLLINKESSSSKPVVFPVP
PPDDLLHLPSQSAIPLIPSKMDAEPQTLQESGTSNQELSLSQTPVADMATDINLQDIPTP
ETFCVSVPDSELYESLVTETTPSSNTCQPFIPLDVNSILKPNTFDVAKMDESGGIINSDI
IDELMSSDIFPLLRLSPTPEDDYSFNLDDNEGICDLFDVQILNY

>Danio rerio_E2F5(ENSDARP_56678)
MAESNSASFPHSTPNGSSRHEKSLGLLTVKFVTLLQEAKDGVLDLKVAADSLAVKQKRRI
YDITNVLEGIGLIEKKTKNTIQWKGESTGCQPQEVLEQVELLKANIADLELQERELDMQK
ACLQQSIKQLNEDPYSCRYSYVMHEDICDAFSGDTLLAVMAPSGTQLEVPVPEMGHNGQK
KYQVNLRSHSAPIQVMLINRETSCSKPVVVSVPPIDDISSMPTPPSTPAGLQRFPISSID
LCDQKHGLLKSPAAEHQLTPSSTSPDVHMECNPESPASQCLLMQSSLGGPEEQQRELGGQ
DLQSMLEEMRDEREGVSNLIDELMSSDVFPLLRLSPNPGVDYSFNLDDNEGVCDLFDVQI
LNY

>Takifugu rubripes_E2F5(ENSTRUP_5420)
RAAPSRHEKSLGVLTMKFVRLLQQAEDGVLDLKVAASSLAVGQKRRIYDITNVLEGVGLI
EKKNKNIIQWRGENSHNRTREVMEQVQHLKAQNSELEAREKELDNQKAWLEENIKFLSHD
PNSTTYPFVTHEDICNAFRGETLLAVLAPAGTQLEVPLPEKGQSGQKKYQVNLRSHFSPI
QVLLINRDSDSSVPVVFPVPPTEIICPTPPSTPASLQRFPLSSSDHQMVLPEHDDLLTPS
STPPDAQTGKGPVDLIEELMSTDYYILITRFPPYTGIDYSFNLDDNEGVCDLFDVQILNY

>Oryzias latipes_E2F5(ENSORLP_22426)
FPTAAARSTDVRLNLQAADSLAVKQKRRIYDITNVLEGVGLIEKKNKNIIQWRGQRSVCS
QTKEVQEQVGLLKAQISQLEALEEELDQQKVCLEESIQALSHGPRRSTYTFVTHEDICRA
FEGETLLAIVAPAETQLEVPVPDTGGGGQKSYQVNLRSRRAPIRVLLIDRDLDSSAPVVF

PVPPSDDFCPMPTPPSTPGGLQRFTLVTPGGSAASPLCSQDPTCSDQQMAPVDLSGRAAS
SSDLASDQNPMDLEVQDFQAALGVGSLLKMSDGGESLKDSREGEAELMEELISAEAPPLP
RLPALTGLDYSFSLDAGGGGCDLFDVSILNY

>Homo sapiens_E2F6(ENSP_370936)
MSQQRPARKLPSLLLDPTEETVRRRCRDPINVEGLLPSKIRINLEDNVQYVSMRKALKVK
RPRFDVSLVYLTRKFMDLVRSAPGGILDLNKVATKLGVRKRRVYDITNVLDGIDLVEKKS
KNHIRWIGSDLSNFGAVPQQKKLQEELSDLSAMEDALDELIKDCAQQLFELTDDKENERL
AYVTYQDIHSIQAFHEQIVIAVKAPAETRLDVPAPREDSITVHIRSTNGPIDVYLCEVEQ
GQTSNKRSEGVGTSSSESTHPEGPEEEENPQQSEELLEVSN

>Pongo abelii_E2F6(ENSPPYP_14118)
MSQQRPARKLPSLLLDPTEETVRRRCRDPINVEGLLPSKIRINLEDNVQYVSMRKALKVK
RPRFDVSLVYLTRKFMDLVRSAPGGILDLNKVATKLGVRKRRVYDITNVLDGIDLVEKKS
KNHIRWIGSDLSNFGAVPQQKKLQEELSDLSAMEDALDELIKDCAQQLFELTDDKENERL
AYDSITVHIRSTNGPIDVYLCEVEQGQTSNKRSEGVGTSSSESTHPEGPEEEKNPQQSEE
LLEVSN

>Macaca mulatta_E2F6(ENSMMUP_30569)
MSQQRPARKLPSLLLDPAEETVRRRCRDPINVEGLLPSKIRINLEDNVQYVSMRKALKVK
RPRFDVSLVYLTRKFMDLVRSAPGGILDLNKVATKLGVRKRRVYDITNVLDGIDLVEKKS
KNHIRWIGSDLSNFGAVPQQKKLQEELSDLSAMEDALDELIKDCAQQLFELTDDKENERY
PLTYVTYQDIHSIQAFHEQIVIAVKAPAETRLDVPAPREDSITVHIRSTNGPIDVYLCEV
EQGQTGSKRSEGVGTSSSESTHAEGPEEEENPQQSEELLEVSN

>Mus musculus_E2F6(ENSMUSP_20908)
MSQQRTARRQPSLLVDPAQETVRRRCRDPINVENLLPSKIRINLEENVQYVSMRKALKVK
RPRFDVSLVYLTRKFMDLVRSAPGGILDLNKVATKLGVRKRRVYDITNVLDGIELVEKKS
KNHIRWIGSDLNNFGAAPQQKKLQAELSDLSAMEDALDELIKDCAQQLLELTDDKENERL
AYVTYQDIHGIQAFHEQIVIAVKAPEETRLDVPAPREDSITVHIRSTKGPIDVYLCEVEQ
NHSNGKTNDGIGASPSKSSHPQCPEKEDEPPQ

>Rattus norvegicus_E2F6(ENSRNOP_57241)
MSQQRTARKLPSLLVDPAQETVRRRCRDPINVENLLPSKIRINLEENVQYVSMRKALKVK
RPRFDVSLVYLTRKFMDLVRSAPGGILDLNKVATKLGVRKRRVYDITNVLDGIELVEKKS
KNHIRWIGSDLNNFGAAPQQKKLQAELSDLSAMEDALDELIKDCAQQLLELTDDKENERL
AYVTYQDIHGIQAFHEQIVIAVKAPEETRLDVPAPREDSITVHIRSTKGPIDVYLCEVEQ
NHSNGKTADGVGASPSDSKHLQSPEKEDKPLQ

>Canis familiaris_E2F6(ENSCAFP_5307)
MSQQRPARKLPSLLVDPAEETVRRRCRDPINVEGLLPSKIRINLEDNVQYVSMRKALKVK
RPRFDVSLVYLTRKFMDLVRSAPGGILDLNKVATKLGVRKRRVYDITNVLDGIDLVEKKS
KNHIRWIGSDLSNFGAVPQQKKLQEELSDLSAMEDALDELIKDCAQQLFELTDDKENERL
AYVTYQDIHSIQAFHEQIVIAVKAPAETRLDVPAPREDSITVHIRSTRGPIDVYLCEVEQ
GHSSNKSSEGAGTSSSKNKHPEPPNKEENPPQQSEELLEVSN

>Felis catus_E2F6(ENSFCAP_11090)
MLFICIFFICIIVFKLLIYISVFSFFQPSKIRINLEDNVQYVSMRKALKVKRPRFDVSLV
YLTRKFMDLVRSAPGGILDLNKVATKLGVRKRRVYDITNVLDGIDLVEKKSKNHIRWIGS
DLSNFGAVPQQKKLQEELSDLSAMEDALDELIKDCAQQLFELTDDKENERLAYVTYQDIH
SIQAFHEQIVIAVKAPAETRLDVPAPREDSITVHIRSTKGPIDVYLCEVEHDHSGNKVSE
GAGTSSSKNKHPEHPDKEEKPPQQSEELLEVSN

>Myotis lucifugus_E2F6(ENSMLUP_3074)
APLALSPGPEGGRCAGALPRSVRACLPSKIMINLEDNVQYVSMRKTLKVKTPRFDVSLVY
LTRKFMDLVRNAPGGILDLNKVATKLGVRKRRVYDITNVLDGIDLVEKKSKNHIRWIGSD
LNNFGAVPQQKKLREELSDLSAMEEALDELIKDCAQQLFELTDDKDNERLTLAYVTYQDI
HSIRAFHEQIVIAVRAPAETRLDVPAPREDSITVHIRSTKGPIDVYLCEVEQGRSGRKGS
EGAGPSASKKSKHSETPDKEENPPQQSEQVCDVSV

>Monodelphis domestica_E2F6(ENSMODP_13925)

PSKIKINLEENVQYMAMRKALRVAKPRYDVSLVYLTRKFMELIKSAPGGVLDLNEVATTL
GVRKRRVYDITNVLDGIDLIQKRSKNHIQWIGSDLGGIGSKVPQQKKIRDELTDLTAMEK
ALDELIKDCAQQLFELTDDKENERYPLSLAITYVTYQDIHSIQAFHEQIVIAVKAPEETK
LEVPTPKEDCITVHIKSTKGPIDVYLCEVEQDNISTKTFEEMSTALSES

>Gallus gallus_E2F6(ENSGALP_30960)
QKQKMQNKKEEAELLGATEEDKARNCRSILSHSNLRTPMLDLNLDDDVPVVKKTLKVKRP
RFDASLVYLTRKFMDLVKRAPDGVLDLNDVATALGVQKRRVYDITSVLDGIDLIQKRSKN
HIQWVGSNLDQVVEMAAQRQNLKDELSDLSAMEEALDELIKDCAHQLFDLTDDKENAKYP
YVTYQDIRSIQAFQKQIVIAIKAPEETRLEIPIPKEDCIKVHVKSTKGPIDVYLCEVEQD
KPADKNSEDKEAVTSETEPSVPPDEE

>Anolis carolinensis_E2F6(ENSACAP_242)
KTRYDGSLVHLTQRFMELLRTAPQGVLDLNEVSRKLGVRKRRVYDITNVLDGIHLIQKRS
KNHIEWVGSDIKNITRRTPEQQKLRDEIYDLTIMEEALDELIKDCAHQLFDLTDDKENSR
LAYVTYQDIRSIQAFQDQIIIAIKAPEETRMEVPPPKMNCIEVCIKSTKGPIDVFLCEV

>Tetraodon nigroviridis_E2F6(ENSTNIP_8765)
MDRGESRRTGDRLLPGDTCARGAPPDALPVIRFESSDEEGISSEPEGRSKARRSSDPQEH
TEGAMMKKFSRSRASLQRLTRRFLQLMQEAPGCCVDLSSASTRLRTKRRRLYDITNALYG
VQVIEKESRNKVRWIGKSPISVFLLNKKKELEKLRQMEATLDGLIRRCAQQLFDLTDDDR
HSACSLTWAYVTHQDLGLLQTFQEQTVIAVRAPEETKMEVPVPTEDSVQIHLKATQGPIT
VLSCEPGPGRTAEATACFLALEESRIRTNVHAPDRGSSAQDSW

>Gasterosteus aculeatus_E2F6(ENSGACP_15833)
MVKCVVSGCQSRVTSVNRGISNRTQKRFFSFPGDTARVKVWLAALRETDKQDSTEQHWIC
EDHFLPEDISSAGINSDAIPIMPPCLDGPMGLISPWGPQSSEDEEEDRWAPGGGAWFIGL
IKENDYDSHVSFKLWRFIQISNNDELQQLQRWQPETLRPSVSPPECQNPADKPTSGAATA
GDPDVSLWMLTQRFLELLLASPDGSVDLRHVTAVLQTRRRRVYDITNVLEGISLIKKKSA
NKFKWIGSCQISSFLKIHQQKFQRELDNLKLVEDTLDTLIKSCAQQLFDMTDDEQNASSS
CTAAYVTYEDVGRLAAFREQTVLVVKAPEETKLEVPRNTLKQDNIQVHLKGGRGPITVLT
CDIDTSSAESRCFLTLEESRIKTAPLLPASGSPLSAVHSA

>Oryzias latipes_E2F6(ENSORLP_22707)
MVKCVVSGCPNRQAAGSRAVFNRPRKRFFDFPKDPARVKVWLAALRETDKQDSTEQHLIC
EDHFLPEDISKNGVSNEAIPIMPHCLDGPLVSPWGAESSEDEDQWAAGGDDDCEGEDPQK
QSHTNPQKQSQHNNPRKQSHTNPQKQSQHNNPRKIFQTSDGLPAFLVCEQYKKELNMSQS
EYVSLGLLAQRFLDLLQNTPDGALDLRDVTTSLNTRRRRVYDITNVLEGISLLERQSANK
FKWIGKLPVSSFLGVFKIQKEVNNLKLVEDTLDSLIKSCAQQLFDLTDDLQNSLAFVTHE
DISRLQVFQEQTVMVVKAPEETKLEVPPPKEDSIQVHLKADRGPIMVMTCGVGAGQQSSC
FMALEESGIKLAPLPTELKSTGAATQKN

>Homo sapiens_E2F3(ENSP_262904)
MRKGIQPALEQYLVTAGGGEGAAVVAAAAAASMDKRALLASPGFAAAAAAAAAPGAYIQI
LTTNTSTTSCSSSLQSGAVAAGPLLPSAPGAEQTAGSLLYTTPHGPSSRAGLLQQPPALG
RGGSGGGGGPPAKRRLELGESGHQYLSDGLKTPKGKGRAALRSPDSPKTPKSPSEKTRYD
TSLGLLTKKFIQLLSQSPDGVLDLNKAAEVLKVQKRRIYDITNVLEGIHLIKKKSKNNVQ
WMGCSLSEDGGMLAQCQGLSKEVTELSQEEKKLDELIQSCTLDLKLLTEDSENQRLAYVT
YQDIRKISGLKDQTVIVVKAPPETRLEVPDSIESLQIHLASTQGPIEVYLCPEETETHSP
MKTNNQDHNGNIPKPASKDLASTNSGHSDCSVSMGNLSPLASPANLLQQTEDQIPSNLEG
PFVNLLPPLLQEDYLLSLGEEEGISDLFDAYDLEKLPLVEDFMCS

>Macaca mulatta_E2F3(ENSMMUP_46108)
MRKGIQPALEQYLVTAGGGEGAAVVAAAAAASMDKRALLASPGFAAAAAAAAAPGAYIQI
LTTNTSTTSCSSSLQSGAVAAGPLLPSAPGAEQTAGSLLYTTPHGPSSRAGLLQQPPALG
RGGSGGGGGPPAKRRLELGESGHQYLSDGLKTPKGKGRAALRSPDSPKTPKSPSEKTRYD
TSLGLLTKKFIQLLSQSPDGVLDLNKAAEVLKVQKRRIYDITNVLEGIHLIKKKSKNNVQ
WMGCSLSEDGGMLAQCQGLSKEVTELSQEEKKLDELIQSCTLDLKLLTEDSENQRYPLSY
VTYQDIRKISGLKDQTVIVVKAPPETRLEVPDSIESLQIHLASTQGPIEVYLCPEETETH
SPMKTNNQDHNGNIPKPTSKDLASTNSGHSDCSISMGNLSPLASPANLLQQTEDQIPSNL
EGPFVNLLPPLLQEDYLLSLGEEEGISDLFDAYDLEKLPLVEDFMCS

>Oryctolagus cuniculus_E2F3(ENSOCUP_12342)
MRKGIQPALEQYLVTAGGGEGAAVVAAAAAAAMDKRALLASPGFAAAAAAPGAYIQILTT
NTSTSSCSSSLQSGAVAAGPLLPSAPGAEPTAGSLLYTTPHGPSSRAGLLQQAPAPGRGG
SGGGGGPPAKRRLELGESGHQYLSDGLKTPKGKGRAALRSPDSPKTPKSPSEKTRYDTSL
GLLTKKFIQLLSQSPDGVLDLNKAAEVLKVQKRRIYDITNVLEGIHLIKKKSKNNVQWMG
CSLSEDGGMLAQCQGLSKEVTELSQEEKKLDELIQSCTLDLKLLTEDSENQRLAYVTYQD
IRKISGLKDQTVIVVKAPPETRLEVPDSIESLQIHLASTQGPIEVYLCSEETETHSPVKT
NNQDHNGNIPKPTSKDLASTNSGHSDCSISMANLSPLASPANLLQQTEDQIPSNLEGPFV
NLLPPLLQEDYLLSLGEDEGISDLFDAYDLEKLPLVEDFMCS

>Felis catus_E2F3(ENSFCAP_6648)
LQAKRRLELGESGHQYLSDGLKTPKGKGRAALRSPDSPKTPKSPSEKTRYDTSLGLLTKK
FIQLLSQSPDGVLDLNKAAEVLKVQKRRIYDITNVLEGIHLIKKKSKNNVQWMGCSLSED
GGMLAQCQGLSKEVTELSQEEKKLDELIQTCTLDLKLLTEDSENQRLAYVTYQDIRKISG
LKDQTVIVVKAPPETRLEVPDPIESLQIHLASTQGPIEVYLCPEETETHSPMKTNNQDHN
GNIPKPPSKEVVTEFPPSLPDPSVMGTRHDLASTNSGHSDCSISMANLSPLASPANLLQQ
TEDQIPSNLEGPFVNLLPPLLQEDYLLSLGEEEGISDLFDAYDLEKLPLV

>Dasypus novemcinctus_E2F3(ENSDNOP_15351)
PSFVLQAKRRLELGESGHQYLSDGLKTPKGKGRAALRSPDSPKTPKSPSEKTRYDTSLGL
LTKKFIQLLSQSPDGVLDLNKAAEVLKVQKRRIYDITNVLEGIHLIKKKSKNNVQWMGCS
LSEDGGMLAQCQGLSKEVTELSQEEKRLDELIQSCTLDLKLLTEDSENQRLAYVTYQDIR
KISGLKDQTVIVVKAPPETRLEVPDPLESLQIHLSSTQGPIEVYLCPEETETHSPMKTNN
QDHNGNIPKPTSKAQRSEFHFFLLQPPNIAARIGVIIEIPISDLASTNSGHSDCSVSMAN
LSPLASPANLLQQTEDQIPSNLEGPFVNLLPPLLQEDYLLSLGEEEGISDLFDAYDLEKL
PLV

>Monodelphis domestica_E2F3(ENSMODP_14171)
MRKGIQPALEQFLVTAGGGEGAAAAAAAAAASMDKRALLASPGFPAAAPGTYIQILTTNT
TTTSCSSSLQSGGAAAGPQHTNVPGVEQTAGSLLYSTPHGPSSRAGLLQQQPALGRGGGG
GPPAKRRLELGESGHQFLSDGLKTPKGKGRAAVRSPDSPKKKTRYDTSLGLLTKKFIQLL
SQSPDGVLDLNKAAEVLKVQKRRIYDITNVLEGIHLIKKKSKNNVQWMGCSLSDDGGMLA
QCQGLSKEVTELSQEEKRLDELIQSCTLDLKLLTEDSENQKLAYVTYQDIRKISGLKDQT
VIVVKAPPETRLEVPDPLESLQIHLSSNQGPIEVYLCPEENETHSPVKSYNQDHNGNIPK
PSSKDVASTNSGHADCSISMANLSPLASPTNLLQQTEDQIPSNLEGPFVNLLPPLIQEDY
LLSLGEEEGISDLFDAYDLEKLPLVEDFMCS

>Taeniopygia guttata_E2F3(ENSTGUP_6514)
AKRRLELGGSGHQYLAEGLKTPKGKGRAATRSPDSPKTPKSPSEKTRYDTSLGLLTKKFI
QLLSQSPDGVLDLNRAAEVLKVQKRRIYDITNVLEGIHLIKKKSKNNIQWMGCSLSEDGG
MLAQRQGLTKEVTELTQEEKKLDELIQSCTLDLKLLTEDSENQRYPFSQNLQTYVTYQDI
RKISGLKDQTVIVVKAPPETRLEVPDPVESALIHLSSTQGPIEVYLCPEENDALSPMKTY
SQDHNGNISKTISKEVASANSGQGDCSVNMATISPLASPANLLQQTEDQIPSNFEGPFLN
LLPPLLQEDYLLSLGDEEGISDLFDAYDLEKLPSLMDEFIYS

>Xenopus tropicalis_E2F3(ENSXETP_28)
MRKGTQGGHEKLLLPGLQGTDKGSGLAGGFIAGGGGVVGAGGGGYVQIISSPGPPLHHHG
LCIPEQAASFIYSTPHGPATRAGQLQPALGRPPAKRRLELGDGSHQYLSDGLKTPKGKGR
AVTHCPDSPKTPKSPLEKTRYDTSLGLLTKKFIQLLSQSSDGVVDLNRAAEVLKVQKRRI
YDITNVLEGIHLIKKKSKNNIQWMGCTLPDDGGNLAKSQELSKELSELAQEENKLDELIK
NCTLDLKHLTENAENQRYPFSYVTYQDIRKISGLKEQTVIVIRAPPETRLEVPDPVESLQ
IHLSSSQGAIEVYLCPEESESSSPVKQCNQDHNGNVSKPKPHSKDSLSPNMENVNCSVEG
ISPLTSPTNLLQQTEDQISLNMDAPFVNLLPPLMQEDYLLSLGDEEGISDLFDAYELEKL
PSLDDFMCS

>Danio rerio_E2F3(ENSDARP_109781)
MRKGGSSVPGQVMLTGVVRAEQHGVFTAVSDRRCAVYSNATRIQINTPPSTNNGCVPEAS
ADSLYTTPLQGSTHGTGLRPTLGRPPAKRRLELDITDHQYSEPAKTLRSRKGALKLKIPK
APKTPPEKTRYDTSLGFLTKKFCQLLAQSSDGVLDLNKAAIVLNVQKRRLYDITNVLEGV
RLIKKKSKNNIQWLGSSLPSDGGLPSPAMQSHSLAREMLALTQEERRLDELIQTCTRNVQ

QMTEEIHSQKYAYVTYQDIRRIKSLKDQTVIAIKAPSETKLEVPDPKESLQVHLSSSKGP
IDVFLCTDGGDSGSPLQNGLDVNGNHPAFLKVSQEAASDGPADNVKMNGNSNASVNGFGP
VSGNAPMSPLSSSLSSILQQPEEAIPFVPLSPALLSDEYMLGLGDEQGISDLFDSCDLDT
LALDDLLRI

>Takifugu rubripes_E2F3(ENSTRUP_21138)
MRRGISSASEKVILTGVGGSSLDSNIILTTLSDRLNPGQSNATFIQIITTPPPCNVTQTN
VCLSEPQINSIYTTPQQGAANGARQRPALGRPPAKRRLALDDSDHQHQPEPIRTPRGKGP
TANGTRIKTPKTPKSPPEKTRYDTSLGLLTKKFVELLGQSSDGVLDLNLAAETLQVQKRR
LYDITNVLEGIHLIKKKSKNNIQWMGCSLLEEEGSLSQRQRLTDEVSALGEEEQRLEQLI
QRCSTDMRHMSELSSNQKYAYITYQDIKQLGNLRDQTVIVVKAPTDTKLEVTDPDESLSI
HLTSTQGPIDVLLCPDDDSHPTSPVKNGGTDINGNSPFLKVLQAPSPGAPPAAAAVSVTT
LSPISSPYTSLLQQTEDQIPTSLGPFLNLAPPLLEQDDYLLGLGDDQGISDLFDACDFDK
IRSLGLDDLLCS

>Gasterosteus aculeatus_E2F3(ENSGACP_8788)
MRRGISSAPDKVILTGVGGSLDNNIILTALTDRLNAGQSNATYIQIITTPPPCNITQTSN
VCLSEPQINNIYTTPQQAGANGTGQRPTLGRPPAKRRLALDDSDHQYQSEPVRTRGRVGT
AVANGARPKTPRTPKSPPEKTRYDTSLGLLTKKFVDLLAQSSDGVLDLNLAAETLQVQKR
RLYDITNVLEGIHLIKKKSKNNIQWMGCSLLEVEGALSQRQRLTAEVSAMGEEEQRLEQL
IQGCSLDMRRMSELQSNQKYAYVTYQDIKQLGNLRDQTVIVVKAPTDTKLEVADPDENLS
IHLTSTKGPIEVLLCPDEENDPRSPVKDGGMDINGNSPFLKVLQGPTCSSASTIQSLAPP
SSSSAVSVTTLSPISSPYTSLLQQTEDQIASSLGPFLNLGLPLLGQEDYTLDLGDEHGIS
DLFDACDFDKMPSLGLDDLLCS

>Oryzias latipes_E2F3(ENSORLP_17999)
MMRVPKGVSPASVRPAVALASQQKMKVLSTGLSSPPMHAVPIGYFTQIGNTAAAEQRANS
LYSTPHGPEAKPIRSSSGRLPAKRKLDLEDPECLTEFRTPKDKGITAARIPSPRTPKSPG
ERTRYDTSLGLLTKKFVGLIAESPDGVLDLNWATEVLEVQKRRIYDITNVLEGVQLIRKK
SKNNIQWLVGDVFRGGASGGEKACSLRRELVDLDRAERSLDDLIQSSTTKLKQLTEYKDS
QLTLGYVTYQDIRSIASLRDQTVIAVKAPAETKLEVPETAAGSLQIYLKSKNGPIEVYLC
PEEGLEDPSPVKSILNPSKASPHSLGQPGAAPAEPLSCSIKKEPDDCKPQPALARPATPT
SPAASLLDHPALLNTVEKAFNLLQITEDQLPGTSFTSDANTPFVSFSPPLDHDDYLWSLE
DGEGVSDFFDSYDLGDLLRS

>Homo sapiens_E2F4(ENSP_368686)
MAEAGPQAPPPPGTPSRHEKSLGLLTTKFVSLLQEAKDGVLDLKLAADTLAVRQKRRIYD
ITNVLEGIGLIEKKSKNSIQWKGVGPGCNTREIADKLIELKAEIEELQQREQELDQHKVW
VQQSIRNVTEDVQNSCLAYVTHEDICRCFAGDTLLAIRAPSGTSLEVPIPEGLNGQKKYQ
IHLKSVSGPIEVLLVNKEAWSSPPVAVPVPPPEDLLQSPSAVSTPPPLPKPALAQSQEAS
RPNSPQLTPTAVPGSAEVQGMAGPAAEITVSGGPGTDSKDSGELSSLPLGPTTLDTRPLQ
SSALLDSSSSSSSSSSSSSNSNSSSSSGPNPSTSFEPIKADPTGVLELPKELSEIFDPTR
ECMSSELLEELMSSEVFAPLLRLSPPPGDHDYIYNLDESEGVCDLFDVPVLNL

>Pan troglodytes_E2F4(ENSPTRP_53494)
MAEAPQAPPPPGTPSRHEKSLGLLTTKFVSLLQEAKDGVLDLKLAADTLAVRQKRRIYDI
TNVLEGIGLIEKKSKNSIQWKGVGPGCNTREIADKLIELKAEIEELQQREQELDQHKVWV
QQSIRNVTEDVQNSCLAYVTHEDICRCFAGDTLLAIRAPSGTSLEVPIPEGLNGQKKYQI
HLKSVSGPIEVLLVNKEAWSSPPVAVPVPPPEDLLQSPSAVSTPPPLPKPALAQSQEASR
PNSPQLTPTAVPGSAEVQGMAGPAAEITVSGGPGTDSKDSGELSSLPLGPTTLDTWPLQS
SALLDSSSSSSSNSNSSSSSGPNPSTSFEPIKADPTGVLELPKELSEIFDPTRECMSSEL
LEELMSSEVFAPLLRLSPPPGDHDYIYNLDDSEGVCDLFDVPVLNL

>Mus musculus_E2F4(ENSMUSP_15003)
MAEAGPQAPPPPGTPSRHEKSLGLLTTKFVSLLQEAKDGVLDLKLAADTLAVRQKRRIYD
ITNVLEGIGLIEKKSKNSIQWKGVGPGCNTREIADKLIELKAEIEELQQREQELDQHKVW
VQQSIRNVTEDVQNSCLAYVTHEDICRCFAGDTLLAIRAPSGTSLEVPIPEGLNGQKKYQ
IHLKSMSGPIEVLLVNKEAWSSPPVAVPVPPPDDLLQSPPAVSTPPPLPKPALAQPQESS
PPSSPQLTTPTPVLGSTQVSEVACQTSEIAVSGSPGTENKDSGEVSSLPLGLTALDTRPL
QSSALLDSSSSSSSSSSSSSSSSGPNPSTSFEPIKADPTGVLDLPKELSEIFDPTRECM
SSELLEELMSSEVFAPLLRLSPPPGDHDYIYNLDESEGVCDLFDVPVLKL

>Rattus norvegicus_E2F4(ENSRNOP_21145)
MAEAGPQAPPPPGTPSRHEKSLGLLTTKFVSLLQEAKDGVLDLKLAADTLAVRQKRRIYD
ITNVLEGIGLIEKKSKNSIQWKGVGPGCNTREIADKLIELKAEIEELQQREQELDQHKVW
VQQSIRNVAEDVQNSCLAYVTHEDICRCFAGDTLLAIRAPSGTSLEVPIPEGLNGQKKYQ
IHLKSMSGPIEVLLVNKEAWSSPPVAVPVPPPDDLLQSPPAVSTPPPLPKPALAHPQETS
RPSSPQITTPTPVLGSTEVSDVAGQTAEIAVSGSPGTENKGSGELSSLPLGLTALDTRPL
QSSALLDSSSSSSSSSSSSSSSSSSSGPNPSTSFEPIKADPTGVLDLPKELSEIFDPTRE
CMSSELLEELMSSEVFAPLLRLSPPPGDHDYIYNLDESEGVCDLFDVPVLKL

>Canis familiaris_E2F4(ENSCAFP_30221)
MAEAGPQAPPPPGTPSRHEKSLGLLTTKFVSLLQEAKDGVLDLKLAADTLAVRQKRRIYD
ITNVLEGIGLIEKKSKNSIQWKGVGPGCNTREIADKLIELKAEIEELQQREQELDKHKVW
VQQSIRNVTEDVQNSPHTLAYVTHEDICRCFAGDTLLAIRAPSGTSLEVPIPEGLNGQKK
YQIHLKSVSGPIEVLLVNKEAWSSPPVAVPVPPPEDLLQSPPAVSTPPPLPKPTLAQPHD
TSRPSSPQLTTPTPVTGITEAQGVPGPAAEIAVSGGPGTDSKDGGELGSLPPGLAALDTR
PLQSSALLDSSSSSSSSSSSNSSSSGPNPSTTCHPVAPAPIEFLELPKELSEIFDPTREC
MSSELLEELMSSEVFAPLLRLSPPPGDHDYIYNLDESEGVCDLFDVPVLNL

>Myotis lucifugus_E2F4(ENSMLUP_18522)
MAEAGPQAPPPPGTPSRHEKSLGLLTTKFVSLLQEAKDGVLDLKLAADTLAVRQKRRIYD
ITNVLEGIGLIEKKSKNSIQWKGVGPGCNTREIADKLIELKAEIEELQQREQELDQHKVW
VQQSIRNVTEDVQNSPHTLAYVTHEDICRCFAGDTLLAIRAPSGTSLEVPIPEGLNGQKK
YQIHLKSVSGPIEVLLVNKEAWSSPPVAVPVPPPEDLLQSPSALSTPPPLPKPAVALPQD
ASHPSSPQVTSTTTICVPAGVEAQGMAIPAAEIIVSGGPGSDSKDSGELSSLSLGPAALD
TRPLQSSALLDSSSSSSSSSSSSSSSSSGPNPSTSFEPIKVDPTGVLELPKELSEIFDPT
PECMSSELLEELMSSEVFAPLLRLSPPPGDHDYIYNLDESEGVCDLFDVPVLNL

>Monodelphis domestica_E2F4(ENSMODP_7623)
PPGTPSRHEKSLGLLTTKFVSLLQEAKDGVLDLKLAADTLAVRQKRRIYDITNVLEGIGL
IEKKSKNSIQWKGVGPGCNTREIADKLIELKAEIEELQQREQELDQHKVWVQQSIRNVTE
DTFCLFSLTPATVAYVTHEDICKCFTGDTLLAIRAPSGTSLEVPVPEGLNVQKKYQIHLR
SATGPIEVLLVNKDAWSSPPVVVPVPPPEDLLQFQAAIPSPPQPRPAPSQHPETSIPSSP
CPSTPTSTQDQSAIGQPTASPVAGSSSGPGADSKDASVLGTLNTASPAALDTRPLQSSAS
LDSSSTLPNPSTSFEPIKPDPTGILELPKELSEMFDPTRECMSSGLLEELMSSEVFAPLL
RLSPPPGDHDYIYNLDESEGVCDLFDVPVLNL

>Gallus gallus_E2F4(ENSGALP_2638)
MAECGPQPPGAGGGGGGGGGGGSGGAGAPSRHEKSLGLLTTKFVSLLQEAKDGVLDLKLA
ADTLAVRQKRRIYDITNVLEGIGLIEKKSKNSIQWKGVGPGCNTREIAHKLIELKADIED
LEQQEQELEKQKMWVQQSIKNVTEDVQNNWLAYVTHEDICKCFTGDTLLAIRAPSGTRLE
VPIPEGPSRQKKYQIHLKSTSGPIDVLLVNKDAWSSPPVVLPVPPPEDLIQCQAVAPSKP
QIPPLAHFQEASVPSSTQPSTPTPSSTQDHSPPEQKDTSTECSISVVESKSSSSDLDPLS
NVSAPSGHTARLDTQLLQSSASLDSSSVLPSPSASFEPIKPDPAGMLELPKELSEMFDPT
RECMNSELLEELMSSEVFAPLLRLSPPPGDHDYIYNLDESEGVCDLFDVPVLNL

>Anolis carolinensis_E2F4(ENSACAP_22297)
MAADSGAQPPGGGPSRHEKSLGLLTTKFVSLLQEAKDGVLDLKLAADTLAVRQKRRIYDI
TNVLEGIGLIEKKSKNSIQWKGVGPGCNTREIAHKLIELKAEIEDLEQRERELEQQKIWV
QQSIKNVTDDVQNSRLAYITDEDLCKCFPGDTLLAIRAPSGTQLEVPVPEGLNGQKKYQI
HLKSTSGPIDVLLVNKDAWSSPPVVLPVPPPEDLIQCQLATPSKPHRPALHSVPSRSQPS
APVPSSTQDHVCATLKTAGATESDISITEPKSVGELDPLTPPAAPIILPAGLDTQPLQSS
ASLDSGSVLPSPSTSFQLIKPDPTEILELPKELSEMFEPDRECMNSELLEELMSSEVFAP
LLRLSPPPGDHDYIYNLDESEGVCDLFDVPILNL

>Takifugu rubripes_E2F4(ENSTRUP_24511)
LGAVGDSLQPQTPSRHEKSLGLLTTKFVTLLQEAKDGVLDLKAAADTLAVRQKRRIYDIT
NVLEGIGLIEKKSKNSIQWKGVGPGCNTREIADKLIDLKAELDDLALREHELDQQRVWVQ
QSIKNVTDDSNNSPLTLAYVKHEDLCGAFKGDTLLAIRAPIGTQLEVPIPESVGYIPNGQ
RKYQIHLKSSTGPIEVLLVNKDPSSASPVVLPVPPPDEILQSLPTPALTPASQPSVSQVL
KSAAAASVKPAPVAAAIPPVPSTESMMVPSILISSWSSDGTTVQVSDAPSSSPLAPATET

PVAATQQLLSSASLDGSASSSASAVFEPVKSDPSELLDFPKELSDMFDPTKEISGDLLED
LMSSEVFSPLLRLSPPPSDHDYIYNLDETEGLCDLFDVPILNL

>Gasterosteus aculeatus_E2F4(ENSGACP_18258)
STQPVRLTLPQTPSRHEKSLGLLTTKFVTLLQEAKDGVLDLKAAADTLAVRQKRRIYDIT
NVLEGIGLIEKKSKNSIQWKGVGPGCNTREIADKLIDLKAELDDLALRENELDQQRVWVQ
QSIKNVTDDSNNSPYPSLQHEDLCGAFKGETLLAIRAPIGTQLEVPIPESVVLNGQRKYQ
IRLKSSSGAIEVLLVNKDPSSSSPVVLPVPPPDDILQSLPVPTPTSQPPTAVPQYRLLSS
FTSSFRPSVLQNCSSNDCKTRPGRNNSCSSHRDNPSISTQQLQSSASLDGSASSSASAGF
QPIKSDPSELLDFPKELSDMFDPTKEIISGDLLEDLMSSEVFSPLLRLSPPPSDHDYIYN
LDETEGLCDLFDVPILNL

>Ciona intestinalis_(ENSCINP_30840)
MDNSRVFSTTTAREIFTNNLASVSPQCSSKSLPSNELHLTPSQDDTHQWLYGGHDMHGAT
DMSIVTVESQNNRNTAQVKRKLELQSSSSGSMNSYLMSESQIQQLQTQNFVNNYNKPPSF
KSPLKRAKSVSERKVLATNPPSRKRKMSTAGSVASGSPSEKSRYDTSLGLLTKRFTQLMR
NSSDGILDLNQAADILAVQKRRIYDITNVLEGIGLIEKRSKNNVQWVACPNTESDHSSEI
EKQETQNEVDALRNKEEELDQLIRKRQMELERLSESNTEHSYVTYQDIRGIKSFKEQIVI
CIKAPQDTKLEVPDPGEKIQMLLKSTKGEIDVFLCPDPQDHISPKKEPVEGDSGLLESTS
VSDGSQRLSGSQYEENRPLFMQTEDQMPSDEVDSAFIDEDSFIALSPAMNPDDYLFSLDD
SEGISELFDMNATSLSGARTS

>Ciona savignyi_(ENSCSAVP_5210)
MDNSRVFSTTTAREMFTNNLASISPHCSNKTFEFHASPSLEHTHQWLPGGHDLAGVSDIS
IMSVESQSNRTSNQVKRKLELPSHSPGQSPYYMSESQLQQLHNQQLTNYNKPSSFKSPLK
RGKSAMERKVLATNPPIRKRKNSSTNAAMAGSPSEKSRYDTSLGLLTKRFIQLMKSSSDG
ILDLNHAAEILSVQKRRIYDITNVLEGIGLIEKRSKNNVQWVDAQKCDDEQVNEEEKRES
ELELASLRKQEEQLDTLIRKRQLELERLSESNNEYPFYFSQLLDIYIRGIKSFKDQIVIC
IKAPQDTKLEVPDPGEKIQMLLKSTKGEIDVFLCPDPHDNMSPKKSQSDSCLGDTLEEDR
SQRISPNGKYEDTKPLLMQTDDCIPSDEVDSAFIDEDSFIALSPSVNPDEYLFTLDDSEG
ISELFDINSTSLSAPPSS

>Branchiostoma floridae_(XP_002590245)
KSPLEKTRYDTSLGLLTKKFVGLLGSSPDGIVDLNQAAEVLNVQKRRIYDITNVLEGINLIKKKSKNHIE
WRVQSTMEKDKERLNSAIGLFKTVDTEHIRENLLDELIRHSSTQLKHLTEDSENKKYPFQHGEHYCKCRN
DIRSIKTFEEQTVIAIKAPPETRLEVPDPRESIQIWLKSSKGPIEVYLCPEEQNTEDAGSSSSDAASSEG
CSSTSGSPCSVKGSELKTNEMARSLLPQTEDQDQEIQDFVALSPPLMEDDYLFSLSPGEGISDLFDAYDL

>Saccoglossus kowalevskii_(XP_002733979)
MPRGQPMQTVSRPAIPTLTITKSTVANTTYGEVSRGITIKGVTQIQELEPIYTVKALQQHQEILSQVGCT
PDLQTKVIKPTIGRPPQAKRKLDLDATAAMLDDGFKSPRGGRKKPRPSPKGSAQSPAERTRYDTSLGLLT
KKFVGLLRSAPEGVLDLNYAAEVLEVQKRRIYDITNVLEGINLIAKKAKNNIEWKGSSNSIASNGQEGSV
CASSVDLNAEIFDLEAKENRLDELIKSCTLQLKMLTEDANNGKYPL

>Octopus bimaculoides_(XP_014790584)
MSIITTEDLRTPKRGRKTRTLPDCSPKVRSPGEKTRYDTSLGLLTKKFVGLLRNAPDGVVDLNKAAESLE
VQKRRIYDITNVLEGINLISKKSKNHIQWKGCISSIAANPVPLQLTTKLMDLHSEMAIYEAKENEMDKLI
EHCTKQLKILTEDPEKSKYAYVTYHDIRNIRSLDDQTVMAVKAPPETRLELPDISKIWLKSNSQAIEVYL
CPDDVKKDVKHTPATEGHNSSSSISEEQLSEDSTSCDSFKTQVSLKNALLEEQDISPDASINLLQQTEDQ
NLDSTLTHLEPPIYMEDYMFNLNDGEGITDFFGGVF

>Hydra magnipapillata_(XP_012561724)
MQPLQPIIFTTFKTPSKVFMANSQTKQIIRSAPQPIAPATFKTKLNSPALEEKLAVQKSPACERRYETSL
GILTKRFVSLLRNSVSGILDLNQAAELLDVQKRRIYDITNVLEGIGVIEKNSKNNIKWVGAKHLENQNDN
IADVENQEEAILATNLVDLHQDIEDLKLSEAKLDELIQQCQNEMKQCSGAKHYNKHSYVTYQDIRGIKDF
NNKTVIAIKAPPETKLEVPDPNESIQIWLKSSNGPIDVYLCPENKENVPPSNDRLDECAYSDSTSSVSEF
SPFNNENLCSPGNLSQSSGYFDECQFSSYDANTTLSSMNCDALHINKLDSEDFIAYNESCYQNELSISPL
ISLEPNFKAEDYLFSLDCNEGISDLFDVDTFL

Family with Sequence Similarity 110-FAM110

>Homo sapiens_FAM110D(ENSP_363386)
MLLAPPSTPSRGRTPSAVERLEADKAKYVKTHQVIARRQEPALRGSPGPLTPHPCNELGP
PASPRTPRPVRRGSGRRLPRPDSLIFYRQKRDCKASVNKENAKGQGLVRRLFLGAPRDAA
PSSPASTERPAASGGWAAPQDAPEAAGKRALCPTCSLPLSEKERFFNYCGLERALVEVLG
AERFSPQSWGADASPQAGTSPPPGSGDASDWTSSDRGVDSPGGAGGGGGSEAAGSARDRR
PPVSVVERNARVIQWLYGCQRARGPPRESEV

>Pan troglodytes_FAM110D(ENSPTRP_39919)
MLLAPPSTPSRGRTPSAVERLEADKAKYVKTHQVIARRQEPALRGSPGPLTPHPCNELGP
PASPRTPRPVRRGSGRRLPRPDSLIFYRQKRDCKASVNKENAKGQGLVRRLFLGAPRDAA
PSSPASTERPAASGGWAAPQDAPEAAGKRALCPTCSLPLSEKERFFNYCGLERALVEVLG
AERFSPQSWGADASPQAGTSPPPGSGDASDWTSSDRGVDSPGGASGGGGSEAAGSARDGR
PPVSVVERNARVIQWLYGCQRARGPPRESEV

>Gorilla gorilla_FAM110D(ENSGGOP_23325)
MLLAPPSTPSRGRTPSAVERLEADKAKYVKTHQVIARRQEPALRGSPGPLTPHPCNELGP
PASPRTPRPVRRGSGRRLPRPDSLIFYRQKRDCKASVNKENAKGQGLVRRLFLGAPRDAA
PSSPASTERPAASGGWAAPQDAPEAAGKRALCPTCSLPLSEKERFFNYCGLERALVEVLG
AERFSPQSWGADASPQAGTSPPPGSGDASDWTSSDRGVDSPGGAGGGGGSEAAGSARDGR
PPVSVVERNARVIQWLYGCQRARGPPRESEV

>Pongo abelii_FAM110D(ENSPPYP_1957)
MLLAPPSTPSRGRTPSAVERLEADKAKYVKTHQVIARRQEPALRGSPGPLTPHPCNELGP
PASPRTPRPARRGSGRRLPRPDSLIFYRQKRDCKASVNKENAKGQGLVRRLFLGAPRDAA
PSSPASTERPAASGGWAAPQDAPEAAGKRALCPTCSLPLSEKERFFNYCGLERALVEVLG
AERFSPQSWGADASPQAGTSPPPGSGDASDWTSSDRGVDSPDGAGGGGGSEAAGSARDGR
PPVSVVERNARVIQWLYGCQRARGPPRESEV

>Mus musculus_FAM110D(ENSMUSP_60143)
MLLSSPTTPSRGRTPSAVERLEADKAKYVKTHQVIVRRQEPALRGGPGPLTPHPCNELGA
SASPRTPGPARRGSGRRQPRPDSLIFYRQKRDCKASVNKENAKGQGLVRRLFLGATRDAA
PSSPAPTERPGAPAGWAGSPDTPEATGKRAVCPTCSLPLSEKERFFNYCGLERALVEVLG
AERFSPQSWGAEHGPQVATSPPPGSGDTSDWTSSDRDAGSPDCAGGGGGSEAAGSARDGR
PTVSVVERNARVIQWLYGCQRARAPPRESEV

>Rattus norvegicus_FAM110D(ENSRNOP_22130)
MLLASPTTPSRGRTPSAVERLEADKAKYVKTHQVIVRRQEPALRGGSGPLTPHPCNELGA
SASPRTPGPARRGSGRRQPRPDSLIFYRQKRDCKASVNKENAKGQGLVRRLFLGATRDAA
PSSPAPTERPGAPAGWAGSPDTPEATGKRALCPTCSLPLSEKERFFNYCGLERALVEVLG
AERFSPQSWGAEHSPQVATSPPPGSGDTSDWTSSDRDAGSPDCAGGGGGSEAAGSARDGR
PTVSVVERNARVIQWLYGCQRARAPPRESEV

>Canis familiaris_FAM110D(ENSCAFP_18618)
MILASPSTPSRGRTPSAVERLEADKAKYVKTHQVIARRQEPALRGGPGPLTPHACNELGP
PASPRTPRPARRSSGRRLPRPDSLIFYRQKRDCKASVNKENAKGQGLVRRLFLGAPRDAP
SSSPGPTERPAAPGVWAAPQDAPEAAGKRALCPTCSLPLSEKERFFNYCGLERALVEVLG
AERFSPQSWGADASPQPGASPPPGSGDTSDWTSSDGDTDRPDGADRGGGGGGGSEAAGSA
RDGRPPVSVVERNARVIQWLYGCQRARGPPRESEV

>Bos taurus_FAM110D(ENSBTAP_17456)
MLLASPSTPSRGRTPSAVERLEADKAKYVKTHQVIARRQEPALRGGPGPLTPHTCNELGP
PPSPRTPRPARRGSGRRLPRPDSLIFYRQKRDCKASVNKENAKGQGLVRRLFLGSPRDVA
SSSAGSSERPAAPGGWTAPQDAPEAAGKRALCPTCSLPLSEKERFFNYCGLERALVEVLG
AERFSPQSWGADASPQPGTSPPPGSGDASDWTSSEGGADRRDGAEGGGSAAAGSERDGRP
QVSVVERNARVIQWLYGCQRARGPPRESEV

>Gallus gallus_FAM110D(ENSGALP_2206)
MVPLGSTPRAVCTSGRDVSPLRLLNRGPESLRAQLAGGSGGRTPSAVERLEADKVKYVKS
QQVINSRQEPALRGCSPCCSPRGRRLLALQRCHELCHGSDLSCDGSRKAPCPSSPVPRRG
GGSRRLLRPDSLVIYRQKRDCPVINKENTKGYGLVRRLFQGPLRDKPPSSPSAQGSDKAP
ESPMLWVSMEQAEDVRTPGAGGSSSSTVPMPDSPVPQPPEAPSLAQPPASPSPSKPALGL

RLSLPLSEKERFFNYCGLDRAMVEGLGKERFVPAGWDAASARLHGSCEWEPSQASGCSGE
DAGPGEEEPDTRLCSAISVVERNARVIKWLYGCQRAWAAAKESTV

>Anolis carolinensis_FAM110D(ENSACAP_17512)
YLRKQLEGTAGMRTPSAVERLEADKAKYVKSQQVIDRRQEPVLLCYSLQSSPCCRRPLAR
HQRLVVTKDGLGPKKLPPTPPSPQSPIARRGGGRRLLRPDSLVIYRQKRDCSAVNKENTK
SYGIVRRLFQSPLRDGHNSRVVSTEGVEHPQRDPGPTDSKRKLCLSSSLPLSEKERFFNY
CGLDRNLVQVLGAERFKPGTWDARSSGRFPGSVASASSLQHGSGEEGPAEEPSEKPSTSI
SLVERNARVIKWLYSCQQAQTAAKESTV

>Danio rerio_FAM110D(ENSDARP_84963)
MKPLTPIGSPSPLRLLNKGPDYLRRQMDAGSRGRSISAVERLEADKAKYVKTQQVINTKQ
EPVLVPCATPPPVPRRNLTTSTPSTPVLLPRNQMVPFSAPSFSRDENEDDSRKENCRNES
DVETNNNANKPPVLSPRTPLVAPHSAPIMRRSNGKRMLRPDSLVIYRQKKECKSPGENIN
GNIEVKGYSFVRRLFQGSMREKSSVNDVAQKTVINEEKASSSRDGDSRMSWSNDKDTVDG
GVETRRSSKSEREHSSAEIQNNNEEKQPKICLRNSTRNSNNNDMDPWKPVIPWKRTNMKR
SKSELRLRCSLAISEQERFFDYCGLDFDMVERLGPENFLAGASSVDTLSLLLRSVGGGGS
EPSEFSRHSGEGIFQEELAEQIPTGVSIIERNARVIKWLYGCRSAAQEGPKESTV

>Takifugu rubripes_FAM110D(ENSTRUP_18192)
LPGDSKRINMKPLTPIGSPSPLRLLNKGPDYLRRQIDGGGHGRTISAVERLEADKAKYVK
SQQVINTKQEPVLVPCATPPPQPRRGVSLPGNVTPRLPPRSSTTPFSTTSGSLTPKENRR
TSVDIEVHNRSNVSFGMPPIPRTPGGNSLVAPHSAPVLRRSSGKRMMRPDSLVIYRQKKE
CKSPSGGTVGENNNMESRRSSKTERERSPESLPSHAFGSTLERTRNGFRNGANEGAKTVA
TNGNHSSNPDDDADDDPWKRASPQPAARRKFGELRRSKSDLRLRCSVALSEQDYFFDFCG
LDMDMIQRLGRENFLSGASSIDTLSLALRSVVGDGCGGSEPSEFSRHSGDGLFEEELAEQ
LPTGVSIIERNARVIKWLYGCKNAAEEGPKESTV

>Gasterosteus aculeatus_FAM110D(ENSGACP_17970)
MKPLTPVGSPSPLRLLNKGPDYLRRQMDGGGHGRSVSAVERLEADKAKYVKSQQVINAKQ
EPALVPCATPPPQPRRAASIPGSPAPGLAPRRPSNAASSALSGSFASRDENENYDSRKEN
RRTSVDVEARNRSNANGALPPVPRTPGVGSLVAPHSAPVLRRSTGKRMLRPDSLLIYRQK
KECKSPHGAAAGDNSNTEGSTREKAAGGEGRIQKMLRRSESDLRLRCSAALSEQELFFAF
CGLDVDMVDRLGRTNFLSAASSLGTLSLALRSVDGDDRSEPSEFSQHSGDGLFQEELAEQ
PPTGVSIIERNARVIKWLYGCRNAAREGPKESTV

>Oryzias latipes_FAM110D(ENSORLP_18703)
SPSPLRLLNKGPDYLRRQIDGGGHGRSISAVERLEADKAKYVKSQQVINTKQEPVLVPCA
TPPPHTRRAFSTPGSLTPRLPPRRLSNTPFSISSGSFPSRDENENDDSRKENRRTSVDIE
VHNRSNVNYVTPPSPRTPGTNKLVAPHSAPVLRKSTGKRMLRPDSLVIYRQKKECKSLSD
AAAGENNNVDVKGQSFVRRLFQGSMREKNGGGEGRIQKMVIGLGPTTKMLQTAQEAVKPT
KNEVLSVYPALDPDSDLKTQATELQRSKSDLLLHCSVAFSEQEHFFDFCGLDMDMIERLG
RQNFLSGASSTDTLSLALRSVSEDSGGASEPSEFSRHSGDGLFQEELAEQLPTGVSIIER
NARVIKWLYGCKNAAQEGPKESTV

>Homo sapiens_FAM110C(ENSP_328347)
MRALAALSAPPNERLLPRDPAATRDPDAARPARRSAVERLAADRAKYVRGRPGTGRGVAS
EGSGPGAIKCPGNDPGPPARAPAPVARRAIARKPLRPDSLIIYRQKCEFVRGSGADGPRA
SLVKKLFQGPGKDKAPVPRTGDEGKAGNPETVPTTPGPAADPAIPETPAPAARSAAPSSV
PAAPPGPEPRVVRRRGLQRSQSDLSSRYSAALAESDTFFQYCGLDPEVVEALGRENFTAG
SDCVTLKVRSVSVATSGSGFSRHSGGDDEGLQEEELIEQVPSTTSVIERNARIIKWLYTC
KKAKETPSQEQSRTRGSKPSR

>Gorilla gorilla_FAM110C(ENSGGOP_16511)
SGADGPRASLVKKLFQGPGKDKAPVPRTEDEGKAGNPETVPTTPGPAADPATPETPAPAA
RSAAPSSVPAAPPGPEPRVVRRRGLQRSQSDLSSRYSAALAESDTFFQYCGLDPEVVEAL
GRENFTAGSDCVTLKVRSVSVATSGSGFSRHSGGDDDGLQEEELIEQVPSTTSVIERNAR
IIKWLYTCKKAKETPSQEQSRTRGSKPSR

>Macaca mulatta_FAM110C(ENSMMUP_251)
LAAQPEGESAVERLAADRAKYVRGRPGTGRGVASEGSGPGAIKCPGNDRAIARKPLRPDS

LIIYRQKCEFMRGAGADGPRASLVKKLFQGPGKDKVPVPRTGDEGKTGNPETVPTKPGAA
ADLAPPETPAPAARSAAPSSVPAAPPGPEPRVLRRRGLQRSQSDLSSRYSAALAESDTFF
QYCGLDPEVVEALGRENFTAGSDRVTLKVRSVSVATSGSGFSRHSGDDDGLQEEELIEQV
PSTTSVIERNARIIKWLYTCKKAKETPSQEQSRTR

>Mus musculus_FAM110C(ENSMUSP_41563)
MRALPTLDSLARMRPPLGDPRAAEDTLTPRPANKSAVERLAADRAKYVRSTLGSSRGPVS
EHRVPEAPGVQHRNPIPSALAPAPVARRAIARKPLRPDSLVIYRQKCEFVRGSDADCSRV
GLMKKFFQGSGKDKMAVAPETTRVADEDKTTKETEATWTKSSQAAAARPASMLPPPTPVV
AVKSPAEKTRVANEDKTTKETEATWTKSSQAAAARPASMLPPPTPVVAVKSPAEKTRVAN
EDKTTKETEATWTKSSQAAATRPASMLPPPTPVVAVKSPALPFEVAPRVPVGCSGVQLRV
SRSKGLQRSQSDLSSRYSIAKAESDTFFKYCGLDPDVVEALGRENFSAGSDCVTLKVRSV
SMAASDSSFSRHSEDGLQEEELLEQVPSTTSVVERNARIIKWLFTCKKAKETPSQKLQGP
A

>Rattus norvegicus_FAM110C(ENSRNOP_7466)
MRALPTLDSLARTRPSLGDSRAAEGTLTRQPANKSAVERLAADRAKYVRSTLGSSRGPVS
ENRVPEAPGVQHRNPIPSTLAPAPVARRAVARKPLRPDSLVIYRQKCEFVRGSDADSSRV
GLMKKFFQGSGKDKMSVTPETTRVADEYKTKETEATWTKASQAAAAKPASMLPPPAPVVA
MESPALPFEVAPRVPVRRSGVELRASRSKGLQRSQSDLSSRYSTARAESDTFFQYCGLDP
EVVESLGRENFSAASDCVTPKMRSVSMATSDSSFSRHSEDGLQEEELLEQVPSTTSVVER
NARIIKWLFTCKKAKETPSQKLQEPA

>Canis familiaris_FAM110C(ENSCAFP_34814)
MRALPGLDAPQRQRPPAQQPEGPRGRDAAVPARRSAAERLAADRAKYLRGPRGAGPAPEG
SSAGPSAGAAGAPGPPAPAPAPGAVARRTVARRPLRPDSLVIYRQKCDFVRGQGADGPRA
GLVRKLFPGPGRDRAAACPGTARGDDELRARREGAVVSRPGSVGAPAAPGTPRGAPPAPP
APPAPPASSAPPAPPGTPRRAPAAPPAAPGTPRGAPAAPPAPPAPPTPPASCAAPRTPAS
SAPPGPQPRGSRRGGLRRSQSDLSSRYSASLAEFDTFFQFCGLDPEVLDALGRENFSAGS
DRVTPQVRSVSVATSDSGFSRRSGGQEGLQEEELTEQVPSTTSVVERNARIIKWLYTCKR
AKETPGQGLQGPA

>Felis catus_FAM110C(ENSFCAP_24329)
PAGGEGRAPELQRSQSDLSSRCSASLAEFDTFFQFCGLEPEVVEALGRENFSAGSDRVTL
KVRSVSVATSDSGFSRHSGGEEGLQEEELTEQVPSTTSVVDRNARIIKWLYTCKKARETP
GQGLQ

>Xenopus tropicalis_FAM110C(ENSXETP_36569)
MPTDIMHAVRMHPIAELQHPSPAVPLRLLTRGPDYLRRQMEALGSPRANLSAVERLAADK
AKYVKTPQQQQQGVCVGGSSSAGGSRGDPRGSSASDSSSGSSRGSQQEGTRGQQGTAPVR
RGSSKRSNRPDSLVIYRLKSRGEGQQAEPARVGLVRRLFLKEKPPGSAIPVPPQEHSTPC
PSERLSPEKEKQDSRSRTGLHRSQSDISSRYSRSFSDFDSFFQYCGLEPEVVEELGRDRF
SPAREERLELLAQSRIRSVSVATSESGMSWRSEGEGSLGGLQEDEPLSGQLPGANSTTSV
IERNARIIKWLYGCKRAKETRGGSPD

>Danio rerio_FAM110C(ENSDARP_132007)
SRILVKGPEFLRKQMERENEANKGRVSAVERLAATKPQYVKCQQEVGSSQEPVISTESAS
GSSRGSSKEDDSVKNTVNVDYKELPEEDSNVVRRSSSKKRDSILLYRQKCELLRGAPVLR
RRLNRKPLLTVRDKGGESDRATSKECGKESSQVTITASGTNEVQRATPHRKVLETKEQER
KSRTGVARSSSDISSRYSKNFADFDVFFKYCGLDGDIIESLGKENFSARSDDLSSNIRSI
SVSTSDDGFSSCSDGLQEEELQETVRQGSSVIERNARIIKWLYSCRNAAESGKTLRDLD

>Takifugu rubripes_FAM110C(ENSTRUP_38131)
SAVERLAASKVKYVKSQEVVNMGSSDQNSRSSGNAAAGDNTAQPAFGAQPQSVGQVRRSS
SKKRPDSLLLYRQKCELLRGPGSDRKHRVRCKKLISSVHKDVPLLEVTAQQHDSMEKEKM
AMLEGPVKECSSSANGYQSERGRIKESVGKKSTGLLGIPEIERRSGKGVSRSHSDISSRY
SKNFADFDAFFKYCGLSGEVIESLGKENFSARSDELAIKIRSVSISTSDSGFTRSSGDSD
GLLEDTLQAKIRQGTSVIERNARIIKWLYSCKNAKE

>Tetraodon nigroviridis_FAM110C(ENSTNIP_15203)
IKILEKGPEYLRKQMELETETKRHLSAVERLAASKPKYVKSQEVVNATPEPVISLTELSV

SGLESSDQSSSLIGNVAAGDNSALPAFGAQHRSAGEVRRSSSKKRPDSLLLYRQKCELLR
GDRKHHVRHKPLLHSVHKVTDKDCESVEKEKMATLKGPAKECSSSVGRSVEKKSTCHLMI
PEIERRFGKGVSRSHSDISSRYSKNFADFDAFFKYCGLSGEVIESLGKENFSARSDEMAI
KLRSVSISTSDSGFTRSSGESDGLLEDDLQSKIRQGTSIIERNARIIKWLYSCKNAKEAG
KKLRD

>Oryzias latipes_FAM110C(ENSORLP_17369)
FPVESVSLDARKTTMETNSDATKILGKGPEYFRKQMERDNKGHMSAVERLAASKPIYVKS
QQVVNSCQEPVINLSPFTVSSSGSSNLPLKHGGNLVTGNSPTSVAQDSSPGQERRSSSKK
RPDSLLIYRQKCESLRGPANNRKHPMSRKLLLGPVNGNVPLLENASKECESKGNKEKLLL
AQGAAENCSSRVFVSQSEKRSNRAVEEHSTESSNDSGTKKCAGGLLEVPQTERRSGKGVS
RSHSDISSRYSTNFADFDVFFKYCGLDSDIIDSLGRENFSTCSDEISMSIIRSVSVSTSD
DGFSRDSGDSDGLLEKELEKKENQGTSVIERNARIIKWLYSCKNAAETGKMLRD

>Homo sapiens_FAM110B(ENSP_355204)
MPTETLQTGSMVKPVSPAGTFTSAVPLRILNKGPDYFRRQAEPNPKRLSAVERLEADKAK
YVKSQEVINAKQEPVKPAVLAKPPVCPAAKRALGSPTLKVFGNHAKTESGVQRENLKLEI
LKNIINSSEGSSSGSGHKHSSRNWPPHRSEATDLHRHSFAESLKVYPTQGRRSPQEGGSH
VGRRLLEQSAESFLHVSHSSSDIRKVTSVKPLKAIPCSSSAPPLPPKPKIAAIASMKSPE
ADPVEPACGVSRRPSLQRSKSDLSDRYFRVDADVERFFNYCGLDPEELENLGMENFARAN
SDIISLNFRSASMISSDCEQSQDSNSDLRNDDSANDRVPYGISAIERNARIIKWLYSIKQ
ARESQKVSHV

>Pan troglodytes_FAM110B(ENSPTRP_34709)
MPTETLQTGSMVKPVSPAGTFTSAVPLRILNKGPDYFRRQAEPNPKRLSAVERLEADKAK
YVKSQEVINAKQEPVKPAVLAKPPVCPAAKRALGSPTLKVFGNHAKTESGVQRENLKLEI
LKNIINSSEGSSSGSGHKHSSRNWPPHRSEATDLHRHSFAESLKVYPTQGRSSPQEGGSH
VGRRLLEQSAESFLHVSHSSSDIRKVTSVKPLKAIPCSSSAPPLPPKPKIAAIASMKSPE
ADPVEPACGVSRRPSLQRSKSDLSDRYFRVDADVERFFNYCGLDPEELENLGMENFARAN
SDIISLNFRSASMISSDCEQSQDSNSDLRNDDSANDRVPYGISAIERNARIIKWLYSIKQ
ARESQKVSHV

>Mus musculus_FAM110B(ENSMUSP_127942)
MPTETLQTGSMVKPVSPAGTFTSAVPLRILNKGPDYFRRQAEPNPKRLSAVERLEADKAK
YVKSQEVINAKQEPVKPAVLAKPPVCPGTKRALGSPTLKVFGNHAKTESGVQRETLKLEI
LKNIINSSEGSSSGSGHKHSSRNWPPHRDTTDLHRHSFAESLKVYPTPGHGSPQESSSHV
SRRLLEQSAETFLHVSHSSSDIRKVTSVKPLKAIPCSSSAPPLPPKPKVAAMKSPEADQV
EPACGVSRRPSLQRSKSDLSDRYFRVDADVERFFNYCGLDPEELENLGMENFARANSDII
SLNFRSASMISSDCEQSQDSNSDLRNDDSANDRVPYGISAIERNARIIKWLYSIKQARES
QKVSHV

>Rattus norvegicus_FAM110B(ENSRNOP_12067)
MPTETLQTGSMVKPVSPAGTFTSAVPLRILNKGPDYFRRQAEPNPKRLSAVERLEADKAK
YVKSQEVINAKQEPVKPAVLAKPPVCPGAKRVLGSPTLKVFGNHAKTESGVQRETLKLEI
LKNIINSSEGSSSGSGHKHSSRNWPPHRDTTELHRHSFAESLKVYPTPGRGSPQESSSHV
SRRLLEQSAESFLHVSHSSSDIRKVTSVKPLKAIPCSSSAPPLPPKPKVAAMKSPDADQV
EPACGVSRRPSLQRSKSDLSDRYFRVDADVERFFNYCGLDPEELENLGMENFARANSDII
SLNFRSASMISSDCEQSQDSNSDLRNDDSANDRVPYGISAIERNARIIKWLYSIKQARES
QKVSHV

>Bos taurus_FAM110B(ENSBTAP_15832)
MPTESLQTGSMVKPVSPAGTFTSAVPLRILNKGPDYFRRQAEPNPKRLSAVERLEADKAK
YVKSQEVINAKQEPVKPAVLAKPPVCPAAKRALGSPTLKGFGGGGGGAKSEGGAPRETLK
LEILKNILNSSEGSSTGSGHKHSARNWADAFLHVSHSSSDIRQGPGARPLKAILPCSSSA
PPLPPKPKVAAPAAVKSPEAEAAEPAGGVGRRPSLQRSKSDLSDRYFRVDADVERFFNYC
GLDPEELENLGMENFARANSDIISLNFRSASMISSDCEQSQDSNSDLRNDDSANDRVPYG
ISAIERNARIIKWLYSIKQARESQKVSHV

>Monodelphis domestica_FAM110B(ENSMODP_11099)
PPNMPTETLQTGSMVKPVSPAVTFTSAVPLRILNKGPDYFRRQAEPNPKRLSAVERLEAD
KAKYVKSQEVINAKQEPVKPAVLAKPPVCPAAKRALGSPTLKVFNNNAKTESGMQRENLK

LEILKNIINSSEGSSSGSGHKHSTRNWPPHRSDSAELNRHSFAESLKVYPTQGRSSPQES
NSNVSRRLLEHSAESFLHVSHSSSDIRKVTSVKPLKAIPCSSSAPPLPPKPKVAAIATMK
APEIDPIEPSSCGVSRRPSLQRSKSDLSDRYFRVDADVERFFNYCGLDPEELENLGMENF
ARANSDIISLNFRSASMISSDCEQSQDSNSDLRNDDSANDRVPYGISAIERNARIIKWLY
SIKQARESQKVSHV

>Gallus gallus_FAM110B(ENSGALP_24834)
MPTETLPTGSMVKPVSPAVTFTSAVPLRILNKGPDYFRRQAEPNPKRLSAVERLEADKAK
YVKSQEVINAKQEPVKPAVLAKPPVCPAAKRALGSPTLKVFSNNAKTESGVQRENLKLEI
LKNIINSSEGSSSGSGHKHGPRNWPPHRADSTELNRHSFAESLKVYPTQGRSSPQESSSN
VSRRLLDQSAETFLHVSHSSSDIRKVTSAKPLKAIPCSSSAPPLPPKPKIAAIATLKSPE
IEAVESGCGVSRRPSLQRSKSDLSDRYFRVDADVERFFNYCGLDPEELENLGMENFARAN
SDIISLNFRSASMISSDCEQSQDSNSDLRNDDSANDRVPYGISAIERNARIIKWLYSIKQ
ARESQKVSHV

>Anolis carolinensis_FAM110B(ENSACAP_5176)
MPTETLQTGSMVKPVSPAITFTSAVPLRILNKGPDYFRRQAEPNPKRLSAVERLEADKAK
YVKSQEVINAKQEPVKPAVLPKPPVCPASKRSLGSPTLKVFSNNVKTESGVQRENLKLEI
LKNIINSSEGSSSGSGHKHSSRNWPPHKSDSGDLNRHSFAESLKVYPTQGRGSPQDSNSS
VGRRLLEQSTEAFLHVSHSSSDIRKVSSGKPLKAIPCSSSAPPLPPKPKIAALTTLKSPE
IDAVETSCGVSRRPSLQRSKSDLSDRYFRVDADVERFFNYCGLDPEELENLGMENFARAN
SDIISLNFRSASMISSDCEQSQDSNSDLRNDDSANDRVPYGISAIERNARIIKWLYSIKQ
ARESQKVSHV

>Xenopus tropicalis_FAM110B(ENSXETP_12484)
MPTETLQTGSMVKPVSPAVTFTSAVPLRILNKGPDYFRRQSEPNPKRLSAVERLEADKAK
YVKSQEVINAKQEPVKPAVLAKPPICPGAKRALSSPTMKMFNNNAKNESCVQRENLKLEI
LKNIINSSESSSTGSAHKQSSRNWPPNRSDSADLNRHSFAESLKVYPTQTHSSPQDSNSN
ISRRFLEKTTNTFLHVSHSSSDIRKVTSVKQIKAIPCSSSAPPLPPKPKLAPLSTLKSPE
NDNLDSGCGVSRRPSLQRSKSDLSDRYFRVDADVERFFNYCGLDPEELEKLGMENFTRAN
SDIISLNFRSASMISSDCEQSQDSNSDLRNDDSANDRVPYGISAIERNARIIKWLYSIKQ
ARESQKVSHV

>Danio rerio_FAM110B(ENSDARP_138309)
MPTETLQADSKPVGQGGAFASAVPLRILNKGPDYFRRQTEPNPKRLSAVERLEADKAKYV
KSQEVINAKQEPIKPQVLAKPPVCPVPLKRGGGVGSGLKASNNNAKSDTCATTTKRENLN
LEILKNILNSSSSSEGPAKGTSHKVGARSWAPHHSSELSEPGCRSFTDSLKVPPSTQGRH
SPQGGNLNLSRRLLEERSCEVDGERSPLHASHSSSDIRRMCNGKPLRAARSSSSSAPPLP
PKPSAKALAACDNAPCSPERETLTSTAAELELGSSVARRPSLHRSKSDLSDRYARAGADV
ERFFNYCGLDPEELESVGVESFARANSDIVSLNFRSASMISSDCEQSRHSNDDLTDEEDD
AGERVPYGISAVERNARIIKWLYSIKQARESQKVSHV

>Takifugu rubripes_FAM110B(ENSTRUP_18808)
MPTETLAPALPDSKAPGPATPFSSAVPLRILNKGPDYFRRQVEPNPKRLSAVERLEADKA
KYVKSQEVINAKQEPIKPPVLAKPHSSHSLVPKRGSGIDEGGNGRGLPFKASNNNAKSES
CSTSSGNSKRENLNLEILKNLLNSSSSSGAASEGLEGGAKRGMSRSWTPSGPSTSHSLRS
FSHSLKVPPISSGVCHSPHQGGNLNLSRRVLNERTDGAVLDRSHSPLPPLLTSHSSSDLL
RLCNGKPLRTARSSSSSAPPLPPKPNPASLPPPPDANLEIELGSSAARRSSLHRSKSDLS
DRYARAGADVERFFNYCGLDPEELEAVGPENFARASSDIVSLNFRSASMISSDCDRSRRS
SNGDEDEEEDGGERVPYGISAVERNARVIKWLYSIKQARETQKVSHV

>Tetraodon nigroviridis_FAM110B(ENSTNIP_5998)
AMPTETLAPALPDSKAPGPATPFSSTVPLRILNKGPDYFRRQVEPNPKRLSAVERLEADK
AKYVKSQEVINAKQEPIKPPVLAKPHSSHSLVPKRGSGIDEGGPGRGFPFKASNNNAKSE
SCATSSGNSKRENLNLEILKNLLNSSSSSGAASEGLAGGAKRGMSRSWTPSGSPSTSHSL
RSFSHSLKVPPVGGGGRRSPHQGGNLNLSRRVLDERSRSPLPPLLTSHSSSDLLRLCNGK
PLRTARSSSSSAPPLPPKPNPASLPPPTSADANLEIELGSSAARRSSLHRSKSDLSDRYA
RAGADVERFFNYCGLDPEELEAVGPENFARASSDIVSLNFRSASMISSDCDRSRRSSNEG
LSDGGERVPYGISAVERNARVIKWLYSIKQARETQKVSHV

>Gasterosteus aculeatus_FAM110B(ENSGACP_19484)

MPTETLPPALPDSKTAGPATAFSSAVPLRILNKGPDYFRRQVESNPKRLSAVERLEADKA
KYVKSQEVINAKQEPIKPPVLAKPPAGHTLLSKRSLGIGGGANGGGMPFKASNNNTKSDT
CATSSGSSKRGNLNLEILKNLLNSSSSSGAGSEGLGAGAKSDSAPSPSTSHSLRSFSHSL
KVPPINSGGRRSPLQGGNLNLSRRGEGVLDRSRSPLPPLLTSHSSSDLLRLCNGKPLRTA
RSSSSSAPPLPPKPNPATIPLCDNTAPQSLPCDFGEPSTDANPELGSLVARRSSLHRSKS
DLSDRYARAGADVERFFNYCGLDPEELEAVGPENFARANSDIVSLNFRSASMISSDCDRS
RRSSNDVLSDGDEEEEEAGERVPYGISAVERNARVIKWLYSIRQARETQKVSHV

>Oryzias latipes_FAM110B(ENSORLP_10358)
MPTETLAPNLPDSKATGPATPLSATVPLRILNKGPDYFRRQVDPNPKRLSAVERLEADKA
KYVKSQEVINAKQEPIKPPILAKPAGHAFLLKRGSGTVGGGNGGGTPFKASNNNAKSDSC
ATSSGGTKRENLNLEILKNLLNSSSSSGAGSEGLASGSKSAVLMRSSDSAPSPSTSHSLR
SFPHSLKVPLTNSWGRCSPQLGGNLNLSRRVQDERGYEGIVVERSRSPLPPLLSSHSSSD
LLRLCNGKPLRTARSSSSSAPPLPPKPNPLSLAPCDNSTPQSLPCHLGDPPDASGDANME
LEQGPNVALRSSLHRSKSDLSDRYARAGADVERFFNYCGLDPEELEVVGLENFARANSDI
VSLNFRSASMISSDCDQSRRSSNDGVSEGDEAEDEEEEAGERVPYGISAVERNARVIKWL
YSIKQARETQKVSHV

>Homo sapiens_FAM110A(ENSP_445228)
MPVHTLSPGAPSAPALPCRLRTRVPGYLLRGPADGGARKPSAVERLEADKAKYVKSLHVA
NTRQEPVQPLLSKQPLFSPETRRTVLTPSRRALPGPCRRPQLDLDILSSLIDLCDSPVSP
AEASRTPGRAEGAGRPPPATPPRPPPSTSAVRRVDVRPLPASPARPCPSPGPAAASSPAR
PPGLQRSKSDLSERFSRAAADLERFFNFCGLDPEEARGLGVAHLARASSDIVSLAGPSAG
PGSSEGGCSRRSSVTVEERARERVPYGVSVVERNARVIKWLYGLRQARESPAAEG

>Pan troglodytes_FAM110A(ENSPTRP_22513)
MPVDTLSPGAPSAPALPCRLRTKVPGYLLRRPADGGVRKPSAVERLEADKAKYVKSLHVA
NTRQEPVQPLLSKQPLFSPETRRTVLTPSRRALPGPCRRPQLDLDILSSLINLCDSPVSP
AEASRTPGRAEGAGRPPPATPPRPPPSTSAVRRVDVRPLPASPARPCPSPGPAAASSPAR
PPGLQRSKSDLSERFSRAAADLERFFNFCGLDPEEARGLGVAHLARASSDIVSLAGPSAG
PGSSEGGCSRRSSVTVEERARERVPYGVSVVERNARVIKWLYGLRQARESPAAEG

>Pongo abelii_FAM110A(ENSPPYP_12132)
MPVDTLSPGAPSAPALPCRLRTKIPGYLLRRPADGGVRKPSAVERLEADKAKYVKSLHVA
NTRQEPVQPLLSKQPLFSPETRRTVLTPSRRALPGPCRRPQLDLGILSSLINLCDSPVSP
AEASRTPGRAEGAGHPPPATPPRPPPSTSAVRRVDVRPLPASPARPCPSPGPATAFSPAR
PPGLQRSKSDLSERFSRAAADLERFFNFCGLDPEEARGLGVAHLARASSDIVSLAGPSAG
PGSSEGGCSRRSSVTVEERARERVPYGVSVVERNARVIKWLYGLRQARESPAAEG

>Mus musculus_FAM110A(ENSMUSP_105489)
MPVDTLSPGAPATPALPFRLRTKVPGYLLPRPADGGARKPSAVERLEADKAKYVKSLRVA
NTRQEPVQPPLVRQPLFSPGPRGPVLTPSRRVLPCSGRRPQLDLDILSSLINLCDSPVSP
SEASRTPGRPEGSAHKVPPATPPRPPPSTVAVRRVDVRPLPASPARPYPSPGTTTTSSPG
RPPGLQRSKSDLSERFSRAAADLERFFNFCGLDPEEARGLGVAHLARASSDIVSLAGPSA
GPCSSEGGCSRRSSATVEERSLDRVPYGVSVIERNARVIKWLYGLRQARDPPTTEG

>Rattus norvegicus_FAM110A(ENSRNOP_66725)
MENLSGAPYPAANFVPVSVFSPLPSGYVTAMPVDTLSPGAPTTPALPFRLRTKVPGYLLP
RPADGGARKPSAVERLEADKAKYVKSLRVANTRQEPVQPPLARQPLFSPGSRGPVLTPSR
RVLPCSGRRPQLDLDILSSLINLCDSPVSPSEANRTPGRPEGSAHKVPPATPPRPPPSTV
AVRRVDVRPLPASPARPYPSPGAATTSSPGRPPGLQRSKSDLSERFSRAAADLERFFNFC
GLDPEEARGLGVAHLARASSDIVSLAGPSAGPCSSEGGCSRRSSATVEERTLERVPYGVS
VIERNARVIKWLYGLRQARDPPTTEG

>Canis familiaris_FAM110A(ENSCAFP_34783)
MPVDTLTPGARDTPALPFRLRTKVPGYLLRRPADGGARKPSAVERLEADKAKYVKSLHVA
NTRQEPVQPLLSKQPLFSPGTRRTVLTPSRRVLPGPGRRPQLDLDILSNLIDLCDSPASP
TEASRTPARTEGAHQAPPATPPRPPPSTAAVRRVDVRPLPASPARPCPSPGTATASSPAR
PPGLQRSKSDLSERFSRAAADLERFFNFCGLDPEEARGLGVAHLARASSDIVSLAGPSAG
PGSSEGDCSRRSSATVEERARERVPYGVSVVERNARVIKWLYGLRQARDTPAAEG

>Felis catus_FAM110A(ENSFCAP_10149)
MPVDTLTPGARDTPALPFRLRTKVPGYLLRRPADGGARKPSAVERLEADKAKYVKSLHVA
NTRQEPVQPLLSKQPLFSPGTRRTVLTPSRRVLPGPGRRPQLDLDILSSLIDLCDSPASP
AEAGRTPGRTEGAHQAPPATPPRPPPSTAAVRRVDVRPLPASPAQPCPSPGTTTASSPAR
PPGLQRSKSDLSERFSRAAADLERFFNFCGLDPEEARGLGVAHLARASSDIVSLAGPSAG
PGSSEGDCSRRSSATVEERARERERVPYGVSVVERNARVIKWLYGLRQARDTPAAEG

>Myotis lucifugus_FAM110A(ENSMLUP_9453)
MPVDMLSPGARDTPALPFRLRTKVPGYLLRRPADGGARKPSAVERLEADKAKYVKSLQVA
NTRQQPVQPLLSKQPLFSPGTRRTVLTPSRRALPGPCRRPQLDLDILSSLIDLCDSPVSP
AEASRTPGQAEGAHQDPPATPPRPPPSTAAVRRVDVRPLPASPAQPCPSPDTAASSPARP
PGLQRSKSDLSERFSRAAADLERFFNFCGLDPEEARGLGVAHLARASSDIVSLAGPSAGP
GSSEGGCSHRSSATIEERARERVPYGVSVIERNARVIKWLYGLRQARETPAVEG

>Gallus gallus_FAM110A(ENSGALP_40717)
MPVEALHAGDAMKGMAARTPFTSAMPIRILNKGPEYFRRRAEPAARKPSAVERLEADKAK
YVKSQQVASTRQEPVKPPLLKQPLFVPAVRRAVLTPSRKTTPGPRSAGRAKTSLNLEILN
HLINGCDSPKAESPRGGERHGAAEALSAGRVGKAPGSPAAPRPPGTVAVRRVDVRPCGAL
PPAVPGIRVPSSPSPCRPPAAPALSSPNRPESARRQTLLHRSKSDLSDRFSRATADLERF
FNYCGLDPEEVQDMGAEHFARASSDIVSVKFHSVSTASSEGGRSPRSAATPEGRPAERMP
YGISVVERNARVIKWLYGLRQAREPQQVSNV

>Anolis carolinensis_FAM110A(ENSACAP_10891)
MPVETLQASHAMKGISVTAAPFTSAMPFRILNKGPEYFRRQAAKAPPAAKKLSAVERLEA
DKAKYVKSQQVATTKQEPVRPLLLKQPLFTPGVRRVMLTPSRKTPQGSRCGAKTSLNLDI
LNNLINFCDSPLSPFPKADKSPLESKWRPEDRSRSPGYLPSTPKTKGGPSEGMSKLSQSS
ASSSSKPPSTVAVRRVDVRPCGPMRARVTPAIQLTPVPTSPAQARILSASPLNSPSPCGE
LRPDSAKRQTCLHRSKSDLSDRYSRATADLERFFNYCGLDPEDMGDMEVERFTRASSDIV
SIKFHSVSTASSEGTRSRRSAVTLEERQAERMPYGISIVERNARVIKWLYGLRQARECQK
VSNV

>Xenopus tropicalis_FAM110A(ENSXETP_42515)
MSVGTIRPSGVGAPLPPVMPLRIRNKGPEYFRRPAGGAGGKPSAVERLEADKLKYVKSKQ
VASTKQEPVRPALSKQPLFSPGVQRVLLTPNRRMPPAALRLSSPNNSLNLDVLTKLINLC
DSPLKSPRGEVAPWQAKKEPPNPSSTPNSPRTPSTAAVRRVDVKPREALPPAPGTPLSPS
SPLCSPSDSGKRRSQLQRSKSDLSDRFSRVSANLERFFNYCGLDPGEVGNIGAEHFACAS
SDIAKFHSVSSSGSECAQSQFSETAGEETPAARLPYGISVIERNARVIKWLYGLKQTIES
QKLPPTPG

>Apis mellifera_(XP_006557558)
MMTAMARLPCKQTSPSQQPQVNNFGNSRNCYWNMQTRHPAPPYVRNHQRQTDHTGKRKSAVELLQETKAF
YVKSETVLDRKQELKNSGHLQVSQLSAPGAPPRLLRKCGSASACSMQPTSPSQLPPAPMTPPCCWASCHE
QDRPDGILSPQQTLPPALPPKSPRLVPVPQRRTISGPPSSTGGDQLQTKLRRLLNTDSKENVFFPDSPTQ
TIVQTNGIPQEEKFRYSPGSLSPNFREEDRVKLGVVRIVRVRKKIDHCTQDVRFKFGRSNSHSHTSGRSG
RKSTSSQSVENTVCHKSLPDLHTSTRRRASLSPRASTKSSKPSGHHQSSSNCPDCETSSDYSEHSFKRDA
VCYRSSRHIRRSLGSGGGDASSVLSAGGRTQKSSGSSKLSHGATARDSGGSSGHCTPRSEPPPDCWSHIR
RDSGASTQHSTEKDRSRRGSYANGTPTSVVRRYSPESESSNSQTDYSPTCNSRFSDGWKEGRGRPILRSK
SDISDRYWRQDNGRSNKVPARPPRSITQLENFFDCLGLDSDNYQRITKPDSKSSSPVFFDSVSSVDSALG
LYSWAGNNQTNQSSQWQNNDCSVGMGNDDSNQRVNDPPSIVERNARIIKWLCQCRKVQFGFS

>Anopheles gambiae_(XP_001688662)
MAAVVYGNSPRHSSRHHQGHLLSQHSPYHTQSSNAGQHHSLPPPPGHHSSHHTLSSVSPPSQIITPPTSY
LSTATTAISSQQQQQSSVQQQSQPAYYHHHHSQSVSLPPQHAQTLSSYHQLRSSKRKSAVELLAESKPFY
VKSETVLDRQQQLLNKRSSAGGGSGSGGGSGGSSGGGGGGSQQSRSDGAQCGSTGTLSGSYMVSPSRTLP
SSAVSQALQQQVQQQQQQQQQRSTNRRSASSGSDLLQTKLRKLLNAADSKETIMPPASDVGLINSKYGQP
LETSYIGTGMVGGGGGGGSLDDIGYIQPPKNYQQQLTVHPEQPSDVSVFFPSAFLSPQSLPPHPGGDDDL
YLYGGLHDSLVLTDDYRAISPPAEYAEQQQQQQQQQHGGSPEGKHSMGGGHSRYNYQRSYSHSQAVVPG
SGHHPTDESDYSPTQSYNINSHKSLPDLHSQSSRHSPHSEALSCCSRGNRSNRSGSSFNRDSGGSSGHYT
HHSEPCCKQQQQQQQQQQHSQQHQSSHHQLSHLPHSQMMMQQQQQQHHSQQQHYQQQHHSQQHTLSSQGD
RQDSMMMLGDYRRDSGSSTQHSGNSYYAYGIPPLRYDCIECRAKIREEADCLLNFTTPEVPEAFQDGYSE
KQHQLQQQQQQQQQQHQQQQQHHQQQLSKSQHAKYYDERGRKYYKNPPMSPLSPVSPLSPPEQAQLDLSP

PLGTFKRQKCLRFKNRSGRQSAVSNPSPSSGGRCGPGGGGPGSSASAGAVAGGPMVDDDRRPILRSKSDI
SDRYWNRSQPERTTLLPVAAADVRDHHRERRADRERERDRERDRERDREQQLEKQQRSRSDSLTQLERFF
DRLGLDEETYDRYIAPKGGSLQRRSSGSYHHHSTAHHHNSASSDIHRLSDDCSTGGGGTPGGGGSGGGAD
SDESSAVFFSDVSTIDSTKLPDSTETANGPGAGGAAPASQLLVGAVGAVGQPGVGGQADGGVPLLGGAGP
APLYRTSEPPSIIERNARIIKWLCNCKKMQLA

>Nematostella vectensis_(XP_001629519)
MADSLCIILFGLKRNIQFRVSNDFLLLKDSRNIPKDLSSLQQKFTPLSGFTVRSIRSMAACCPTISDLPK
VLREKLAHILDESNTPTWRELIHVMPPDMYRAHQVESFAMATLKCRSPTQELFDDLARRSVTLDELSGWV
QKLPQSNKKQSMIQLLYGHPVVTRQPGNIMVAVGANAVLSCGATGHQPLNYQWFKSKKALEGQTGSLLRV
KNVSKLDEGHYICRVANSNGDYVFTEWAKLSLQNPSETHQFSRPIITSQPEYPEVHTVLGGTLRLYCDAL
GDPAPTFQWYKNDLPLPDGDQREYCKRDICADDQGRYMCVASNSSGSTESRPTRVFVRHQVPRGQMEAND
KVALLIGNKDYKMAQHLNTLFHPLNDVCDLAGVLRSIGFKVVSLVNLTLKEMEFALDEFYKLLVKDTYAL
FYFAGHGFEINGQGYLMPIDATERYHSQENLPAWKVLETMQNSEAKLNIIVLDCCRTTPNDKPIETIPVD
NGCYQPIVTKENVLVIHGCLPQNSVFESEDERNGFIAKHLIKLIGQTKPLSKIKLEFSKAIHDEKIYDPE
DRKQQVVHIHTTEIDDMCLADNTAVHMQGACAVSGSEGWKAAHGRYTQGFRYFRSSGGGGGPEIPEDPVT
VHERDGVVVELVFMAEFSNALLVTARCRPDNYHVTFDMPECVSGCLVTKVTGKESTIPQRFSRAESAIRI
ADLQRLQVFLCLNMDRQEAWGLGRAHIVQPSP

>Hydra magnipapillata_(XP_004205687)
MEECEFKEHFRVNRNTFNFLVYELHPLLGKTTTTFREPISVVKRVAVALHYLASCEEYRGVSSLFGIGKS
IANLIVHEFINAVNDILLPKYVKFPLSVENLIKHSRDFEAILGFQKCIGAVDGCHIPIFAPKDQAISYYN
YKGWYSIVLFSVVDCRYRFIYTSVGSPGRNNDSYIFQNSSLKAILESSLFDKCCKELGDSLVPLCLIGDS
AFPLTRHLLKPYPKNLELSEI

>Amphimedon queenslandica_(XP_011409799)
MDNSVFIGARPGDSEQALSSVVNYSTSKSSFFIVCEIFAIVLQRQTSLLTLTTLAHISSELSIFIDLLPK
ELLGAVRALCIPNAIYKACATGYSQLAQKSNVLSTPGMHKNFVTFIGLLLAFNCFTIHDIITYIIKPMFK
VYLLHPQQSSQANQGTLSLQFVSLLLRHILVDKYIRGEVNDDGVNIVYPPFVSHYLLAKSKELSFDVVLN
LLKELLKLSKDTLRSSRGTTWGGKTGMSILVESFPSGSQGNEAALIKLRETMELIAEQDWVREQCSKQDS
KQLLHVEKLGDASLSPAQAQYLLHLLIPYCPEDTVKRINESSGSLPPFMTHPEQTVDRILKNLDQWTLHN
ALLHLNLNINSSLHLHSIDHLSQSVIQVFEDSCTEEWTEDSSSQVDGAYCPSIWLVTPLVSELPKSVLGK
ILKSAGNVLGKGQWWNLDRQQERRRSTRASITPTTPNPKTGLQWQQPFLELVLACMNSNHSNHEELLGPL
QKQLTTFLTAFEKDSFPADEQNKSMLYTALKLRLGLVGSMLPSVCSVCESNVLDWVSLLIPLICSGAVDR
QPDNSELFTNCLDMLSALLQALSPDFHMCVFSGGEEGKKSYIMCIKKMKSDLASSKSTCKQEIQQLFPLP
QKLYEVTVVKPPSAFSQQFRIPEKVQGLRVQEVKEISPWEIIEGIKNSGPFQLSWFGATRIKRKPLKYLE
QEKQIQHHTHQHFLSNSGHLLGGSGPSTSRHSVFLPDSNDSTNGAIGGGGGGGGVSSDSGDNAIPPGAAS
TAGGTRASVGGTGEGSGLDNKAEILLQINKMNREQQPSLTTQPGPLPPTFHTTGQVGVSNTPHQLTQQQP
MQRQIPHGMMQQHSAAQMMRPPPHFLQRQLEYMTPEQRNHFMSLPNEEKRVYLSNLQRTFRERRMLEQQQ
QQQQQQQLMFRHPMHPRPMQVAPPMQMTQVLQQRMMPRYGPQHMGPPQMHHSGGYMMPGHHPAMFRQPPP
GPMYTMHRPPMHHNLPPHHQ

Grainyhead like Transcription factor-GRHL

>Homo sapiens_GRHL3(ENSP_288955)
MSNELDFRSVRLLKNDPVNLQKFSYTSEDEAWKTYLENPLTAATKAMMRVNGDDDSVAAL
SFLYDYYMGPKEKRILSSSTGGRNDQGKRYYHGMEYETDLTPLESPTHLMKFLTENVSGT
PEYPDLLKKNNLMSLEGALPTPGKAAPLPAGPSKLEAGSVDSYLLPTTDMYDNGSLNSLF
ESIHGVPPTQRWQPDSTFKDDPQESMLFPDILKTSPEPPCPEDYPSLKSDFEYTLGSPKA
IHIKSGESPMAYLNKGQFYPVTLRTPAGGKGLALSSNKVKSVVMVVFDNEKVPVEQLRFW
KHWHSRQPTAKQRVIDVADCKENFNTVEHIEEVAYNALSFVWNVNEEAKVFIGVNCLSTD
FSSQKGVKGVPLNLQIDTYDCGLGTERLVHRAVCQIKIFCDKGAERKMRDDERKQFRRKV
KCPDSSNSGVKGCLLSGFRGNETTYLRPETDLETPPVLFIPNVHFSSLQRSGGAAPSAGP
SSSNRLPLKRTCSPFTEEFEPLPSKQAKEGDLQRVLLYVRRETEEVFDALMLKTPDLKGL
RNAISEKYGFPEENIYKVYKKCKRGETSLLHPRLSRHPPPDCLECSHPVTQVRNMGFGDG
FWRQRDLDSNPSPTTVNSLHFTVNSE

>Gorilla gorilla_GRHL3(ENSGGOP_1766)
MSNELDFRSVRLLKNDPVNLQKFSYTSEDEAWKTYLENPLTAATKAMMRVNGDDDSVAAL
SFLYDYYMGPKEKRILSSSTGGRNDQGKRYYHGMEYETDLTPLESPTHLMKFLTENVSGT
PEYPDLLKKNNLMSLEGALPTPGKAAPLPAGPSKLEAGSVDSYLLPTSDMYDNGSLNSLF

ESIHGVPPTQRWQPDSTFKDDPQESMLFPDILKTSPEPPCPEDYPSLKSDFEYTLGSPKA
IHIKSGESPMAYLNKGQFYPVTLRTPAGGKGLALSSNKVKSVVMVVFDNEKVPVEQLRFW
KHWHSRQPTAKQRVIDVADCKENFNTVEHIEEVAYNALSFVWNVNEEAKVFIGVNCLSTD
FSSQKGVKGVPLNLQIDTYDCGLGTERLVHRAVCQIKIFCDKGAERKMRDDERKQFRRKV
KCPDSSNSGVKGCLLSGFRGNETTYLRPETDLETPPVLFIPNVHFSSLQRSGGAAPSAGP
SSSNRLPLKRTCSPFTEEFEPLPSKQAKEDDLQRVLLYVRRETEEVFDALMLKTPDLKGL
RNAISEKYGFPEENIYKVYKKCKRGETSLLHPRLSHHPPPDCLECSHPVTQVRNMGFGDG
FWRQRDLDSNPSPTTVNSLHFTVNSE

>Mus musculus_GRHL3(ENSMUSP_101481)
MSNELDFRSVRLLKNDPVSFQKFPYSNEDEAWKTYLENPLTAATKAMMRVNGDEESVAAL
SFLYDYYMGPKEKRILSSSTGGRNDQGKKFYHSMDYEPDLAPLESPTHLMKFLTENVSGS
PDYTDQLKKNNLLGLEGVLPTPGKTNTVPPGPSKLEASSMDSYLLPASDIYDNGSLNSLF
ESIHGVPPTQRWQPDSTFKDDPQESLLFPDILKTSPDPPCPEDYPGLKSDFEYTLGSPKA
IHIKAGESPMAYLNKGQFYPVTLRTPAGGKGLALSSSKVKSVVMVVFDNDKVPVEQLRFW
RHWHSRQPTAKQRVIDVADCKENFNTVQHIEEVAYNALSFVWNVNEEAKVFIGVNCLSTD
FSSQKGVKGVPLNLQIDTYDCGAGTERLVHRAVCQIKIFCDKGAERKMRDDERKQFRRKV
KCPDSSNNAGIKGCLLSGFRGNETTYLRPETDLETQPVLFIPNLHFSSLQRPGGVVPSAG
HSSSDRLPLKRTCSPFAEEFEPLPSKQAKEDDLQRVLLYVRRETEEVFDALMLKTPDLKG
LRNAISEKYGLPEENICKVYKKCKRGILVNMDNNIIQHYSNHVAFLLDMGELDGKIQIIL
KEL

>Rattus norvegicus_GRHL3(ENSRNOP_61035)
MSNELDFRSVRLLKNDPVSFQKFPYNNEDEAWKTYLENPLTAATKAIMRVNGDDESVAAL
SFLYDYYMGPKEKRILSSSTGGRNDQGKKYYHSMDYEPDLAPLESPTHLMKFLTENVSGS
PDYTDQLKKNNLLGLEGALPTPGKTSTLPPGPSKLEAGSMDSYLLPTSDMYDNSSLNSLF
ESIHGVPPTHDFEYTLGSPKAIHIKAGESPMAYLNKGQFYPVTLRTPAGGKGLSLSSSKV
KSVVMIVFDNDKVPVEQLRFWRHWHSRQPTAKQRVIDVADCKENFNTVQHIEEVAYNALS
FVWNVNEEAKVFIGVNCLSTDFSSQKGVKGVPLNLQIDTYDCGAGTERLGAERKMRDDER
KQFRRKVKCPDSSNNGIKGCLLSGFRGNETTYLRPEADLETQPVLFIPNLHFSSLQRPGG
VVPSAGHSSSDRLPLKRTCSPFAEEFEPLPSKQAKEDDLQRVLLYVRRETEEVFDALMLK
TPDLKGLRNAISEKYGFPEENICKVYKKCKRGILVNMDNNIIQHYSNHVAFLLDMGELDG
KIQIILKEL

>Bos taurus_GRHL3(ENSBTAP_26370)
SFSFRSVRLLKNDPVNFQKFPYTNEDEAWKTYLENPLTAATKAMMRVNGDDDSVAALSFL
YDYYMGPKEKRLLSSSTGGRNDQGKRYYHGMEYEMDLTPLESPTHLMKFLTENVSGTPEY
PDVLKKNNLMSVEGAAPTPGKAALLPAGPSKLETGSVDSYLLPTSDVYDNGSLSSLFESI
HGVPPTQRWQPDSTFKDDPQESLLFPDILKTSPEPPGPEDYPNPKSDFEYTLGSPKAIHI
KSGESPMAYLNKGQFYPVTLRTPAGGKGLALSSNKVKSVVMVVFDNEKGPVEQLRFWKHW
HSRQPTAKQRVIDVADCKENFNTVQHIEEVAYNALSFVWNVHEEAKVFVGVNCLSTDFSS
QKGVKGVPLNLQIDTYDCGSGTERLVHRAVCQIKIFCDKGAERKMRDDERKQFRRKVKCP
DSSNSGIKGSLLAGFRGNETTYLRPEADLETPPVLFIPNVHFSSLQRPGGLILSKKNNVD
ELALKRTCSPFTEEFEPLPSKQAKEDDLQRVLLYVRRETEEVFDALMLKTPDLKGLRSAI
SEKYGFPEENIYKVYKKCKRGILVNMDNNIIRHYSNHVAFLLDMGELDGKIQIILKEL

>Monodelphis domestica_GRHL3(ENSMODP_16354)
MSNELDFRSVQVLKNDAVNFQKFSCTNEDEAWKTYLGNPLTAATKAMMRVNGDEDSVAAL
SFLYDYYMGPKEKRILSSSTGARSDLGKRYHHGMEYEVDFPPLESPTHLMKFLTENVSGS
QEYPDLTKKNNLMNLEGTASTKSGLLPPGPSKLEDSPADGSYLLPTGDMYDSSLSNLFET
IHGPPPQQRWQPDSTFKDDGQESLLFPDILKSPSEPQCPEDYPGTKSDFEYTLGSPKAIH
IKSGESPMAYLNKGQFYPVTLRTPGGGRCLHLSSNKVKSMIMVVFDNEKIPVEQLRFWKH
WHSRQPTAKQRVIDVADCKENFNTVQHIEEVAYNALSFVWNVNEEAKVFIGVNCLSTDFS
TQKGVKGVPLNLQIDTYDCGTGTDRLVHRAVCQIKIFCDKGAERKMRDDERKQFRRKAKC
PDSSNNGLKGCPLSNFRGNETTYLRPETDLESQPVLFIPNIHFSNLQRNGVAGSPTIHNG
TNRLPLKRTCSPFSDDFDLLPPKQAKEDDLQRVLLYVRRETEEVFDALMLKTPDLKGLRN
AISEKYGFPEESIYKVYKKCKRGILVNMDNNIIQHYSNHVAFLLDMGELDGKIQIILKEL

>Gallus gallus_GRHL3(ENSGALP_6748)
MRCCLDMRIHLKQKGEQKQQNSPHSFRSVRLMKNDTMNFQKFSYTNEDEAWKTYLENPLT
AATKAMMRVNGDDDSVAALSLLYDYYMVPKEKRILPSGMMGRNELGKRQYHGMEYDSDIT

SFDGSAHLMKLLSENTSMTQEYCEPQKKNGLSLEGIPTPHKPAMLPPGTSKLDATPDTYM
VPPGDLYDNSALNSLFETNPVATPQQRWQPDSTFKEDPQESLLFSDILKPQPEPPCSESY
PTDGVKSDFEYTLGSPKAIHIKSGDSPMAYLNKGQFYPITLRAAGDSKCLHLSSNKVKSV
VMIVFDNEKIPTEQLKFWKHWHSRQPTAKQRVIDVADCKENFNTVQNIEELAYNALSFVW
NIHEEAKVFIGVNCLSTDFSSQKGVKGVPLNLQIDTYDYGNGTNHLVHRAVCQIKIFCDK
GAERKMRDDERKQFRRKGKCLDSNNNGLKGCLLSGFRGNEITFLRPETDLETQPVLFIPN
LHFSNQQRCGTALPPAVPNSANRLPLKRSGTSFTDEFDPVPPKQTKEEDPQRVLLYVRRE
SEEVFDALMLKTPDLQGLRMAISEKYGLPEESIYKVYKKCKRGILVNMDNNIIQHYSNHM
AFLLDMVEAEDKIQVILKEL

>Anolis carolinensis_GRHL3(ENSACAP_16996)
IALLRSFRSVRLLKNDGMGFQKFSCMNEDEAWKTYLENPLTAATKAMMRVNGDDESVAAL
SLLCDYYMIPKEKRYVPSGNTGRNDIGKRHCHVMDYEPDISSFENSAQLMKLLAENVSVS
QEYTDLPKKNGLVLDGLPAPHKPPALPPGPSKMEANPAESYMIPSGDVYDNGSLHSLFDA
IHVAPPQQRWQPDSTFKEDPQESLIFNDILKTQPEVPCSEGYSLNDAKSDFEYTLGSPKA
IHIKSGESPMAYLNKGQFYPVSLKTVGDTKGLHLSSDKVKSVVMVVFDNEKIPMEQLKFW
KHWHSRQPTAKQRVIDVADCKENFNTVQHIEEVAYNALSFVWNVAEEAKVFVGVNCLSTD
FSSQKGVKGVPLNLQIDTYDFGTGTNRLVHRAICQIKIFCDKGAERKMRDDERKQFRRKG
KCLDANNNSLKSCLVSGFRGNEITYLKPQADLETQPVLFIPNVHFSNLQRCATVLPASGV
PHDPSSALLLLCQFLLEISPGPPLISALLLHLPVLLYVRRESEEVFDALMLKTPDLKGLR
NAISEKYGLPEESIYKVYKKCKRGILVNMDNNIIQHYSNHMAFLLDVVEAEEKFQVTLKE
L

>Xenopus tropicalis_GRHL3(ENSXETP_8467)
MSNELDYRPVMLLQNDINLKYSSPNDDEAWSKYLENPMTAATKAMMRANGDDDGVAALSL
LYDYYRVPKEKRIITQGSTGRCDQVKRSCIEYDSDISTYDSTQLMRFLNDNNSSTHEYSE
THKKNSYLSLDCLINPSKLPLSSGKLDNNAHDDFMATSCDVYEKNPLTTLFDPIHVPPPQ
QRWQPDSTFKEDTPEPLIFNDILRSQAESTCSEDYIPGEANRDFECTLESPKAIHIKSGE
SPMAYLNKGQFYPVNLRTAETRKCVHLTSNKVKSVVMVVFDNEKNPEEQLKRWKHWHSRQ
PTAKQRVIDVADYKENCNTVENIEEVAYNALSFVWNINEEAKIFIGINCLSTDFSSQKGV
KGVPLNLQIDTYDFETGVKRLIHRAVCQIKIFCDKGAERKMRDEERKQFRRKGKSADQNN
KDIKASVLPGYRGSDFTYLRPVTDMETHPVLFIPNIHYSNLQRCGVVLQSAADNSDRLSL
KRSSQSFPESFEAPPSKQQTNEDPQRVLLYVRRETEEVFDALMLKTPDLKGLRNAISEKY
ELPEERICRVYKKCKRGILVNMDNNIIQHYSNHVAFLLDLTDVDGKIQVTLKEL

>Homo sapiens_TFCP2L1(ENSP_263707)
MLFWHTQPEHYNQHNSGSYLRDVLALPIFKQEEPQLSPENEARLPPLQYVLCAATSPAVK
LHEETLTYLNQGQSYEIRLLENRKLGDFQDLNTKYVKSIIRVVFHDRRLQYTEHQQLEGW
RWSRPGDRILDIDIPLSVGILDPRASPTQLNAVEFLWDPAKRASAFIQVHCISTEFTPRK
HGGEKGVPFRVQIDTFKQNENGEYTEHLHSASCQIKVFKPKGADRKQKTDREKMEKRTAQ
EKEKYQPSYETTILTECSPWPDVAYQVNSAPSPSYNGSPNSFGLGEGNASPTHPVEALPV
GSDHLLPSASIQDAQQWLHRNRFSQFCRLFASFSGADLLKMSRDDLVQICGPADGIRLFN
AIKGRNVRPKMTIYVCQELEQNRVPLQQKRDGSGDSNLSVYHAIFLEELTTLELIEKIAN
LYSISPQHIHRVYRQGPTGIHVVVSNEMVQNFQDESCFVLSTIKAESNDGYHIILKCGL

>Macaca mulatta_TFCP2L1(ENSMMUP_55543)
MLFWHTQPEHYNQHNSGSYLRDVLALPIFKQEEPQLSPENEARLPPLQYVLCAATSPAVK
LHEETLTYLNQGQSYEIRLLENRKLGDFQDLNTKYVKSIIRVVFHDRRLQYTEHQQLEGW
RWSRPGDRILDIDIPLSVGILDPRASPTQLNAVEFLWDPAKRASAFIQVHCISTEFTPRK
HGGEKGVPFRVQIDTFKQNENGEYTEHLHSASCQIKVFKPKGADRKQKTDREKMEKRTAQ
EKEKYQPSYETTILTECSPWPDVAYQVNSAPSPSYNGSPNSFGLGEGNTSPTHPVEALPV
GSDHLLPSASIQDAQQWLHRNRFSQFCRLFASFSGADLLKMSRDDLVQICGPADGIRLFN
AIKGRNVRPKMTIYVCQELEQNRVPLQQKREGSGDSNLCVYHAIFLEELTTLELIEKIAN
LYSISPQHIHRVYRQGPTGIHVVVSNEMVQNFQDESCFVLSTIKAESNDGYHIILKCGL

>Mus musculus_TFCP2L1(ENSMUSP_27629)
MLFWHTQPEHYNQHNSGSYLRDVLALPIFKQEEPQLSPENGARLPPLQYVLCAATSPAVK
LHEETLTYLNQGQSYEIRLLENRKLGDFQDLNTKYVKSIIRVVFHDRRLQYTEYQQLEGW
RWSRPGDRILDIDIPLSVGILDPRASPTQLNAVEFLWDPSKRASAFIQVHCISTEFTPRK
HGGEKGVPFRVQIDTFKQNESGDYSEHLHSASCQIKVFKPKGADRKQKTDREKMEKRTAQ
EKEKYQPSYETTILTECSPWPDVPYQANNTPSPSYNGSPNSFGLREGNSSPNHPVEPLPL

GSDHLLPSASIQDAQQWLHRNRFSQFCWLFASFSGADLLKMSRDDLVQVCGPADGIRLFN
AIKGRNVRPKMTIYVCQELEQNQLPLPQKQDDSGDNSLCVYHAIFLEELTTLELTEKIAS
LYSIPPQHIHRVYRQGPAGIHVVVSNEMVQNFQDESCFILSTLKAESNDGYHIILKCGL

>Canis familiaris_TFCP2L1(ENSCAFP_7168)
XYNQHSSGSYLRDVLALPIFKQEEPQLSPENEARLPPLQYVLCAATSPAVKLHEETLTYL
NQGQSYEIRLLENRKLGDFQDLNTKYVKSIIRVVFHDRRLQYTEHQQLEGWRWSRPGDRI
LDIDIPLSVGILDPRASPTQLNAVEFLWDPSKRASAFIQVHCISTEFTPRKHGGEKGVPF
RVQIDTFKQNENGEYTEHLHSASCQIKVFKPKGADRKQKTDREKMEKRTAQEKEKYQPSY
ETTILTECSPWPDMAYQVNSAPSPSYNGSPNSFGLGEGNSSPTHPVETLPLSNDHLLPSA
SIQDAQQWLHRNRFSQFCRLFASFSGADLLKMSRDDLVQICGPADGIRLFNAIKGRNVRP
KMTIYVCQELEQNRAPLQQKRDGGDSSLCVYHAIFLEELTTLELVEKIASLYSISPQHIH
RVYRQGPTGIHVVVSNEMVQNFQDESCFILSTLKAESNDGYHIILKCGL

>Felis catus_TFCP2L1(ENSFCAP_11266)
FITRDTQREEGDQDMSGGFVQSDVLALPIFKQEEPQLSPESEARLPPLQYVLCAATSPAV
KLHEETLTYLNQGQSYEIRLLENRKLGDFQDLNTKYVKSIIRVVFHDRRLQYTEHQQLEG
WRWSRPGDRILDIDIPLSVGILDPRASPTQLNAVEFLWEPSKRASAFIQVHCISTEFTPR
KHGGEKGVPFRVQIDTFKQNDNGEYTEHLHSASCQIKVFKPKGADRKQKTDREKMEKRTA
QEKEKYQPSYETTILTECSPWPDMAYQVNSAPSPSYNGSPNSFGLGEGNSSPTHPVETLP
LGNDHLLPSASIQDAQQWLHRNRFSQFCRLFASFSGADLLKMSRDDLVQICGPADGIRLF
NAIKGRNVRPKMTIYVCQELEQNRAPLQQKRDGGGDSSLCVYHAIFLEELTTLELVEKIA
NLYSISPQHIHRVYRQGPTGIHVVVSNEMVQNFQDESCFILSTLKAESNDGYHVILKCGL

>Homo sapiens_GRHL1(ENSP_324693)
MTQEYDNKRPVLVLQNEALYPQRRSYTSEDEAWKSFLENPLTAATKAMMSINGDEDSAAA
LGLLYDYYKVPRERRSSTAKPEVEHPEPDHSKRNSIPIVTEQPLISAGENRVQVLKNVPF
NIVLPHGNQLGIDKRGHLTAPDTTVTVSIATMPTHSIKTETQPHGFAVGIPPAVYHPEPT
ERVVVFDRNLNTDQFSSGAQAPNAQRRTPDSTFSETFKEGVQEVFFPSDLSLRMPGMNSE
DYVFDSVSGNNFEYTLEASKSLRQKPGDSTMTYLNKGQFYPITLKEVSSSEGIHHPISKV
RSVIMVVFAEDKSREDQLRHWKYWHSRQHTAKQRCIDIADYKESFNTISNIEEIAYNAIS
FTWDINDEAKVFISVNCLSTDFSSQKGVKGLPLNIQVDTYSYNNRSNKPVHRAYCQIKVF
CDKGAERKIRDEERKQSKRKVSDVKVPLLPSHKRMDITVFKPFIDLDTQPVLFIPDVHFA
NLQRGTHVLPIASEELEGEGSVLKRGPYGTEDDFAVPPSTKLARIEEPKRVLLYVRKESE
EVFDALMLKTPSLKGLMEAISDKYDVPHDKIGKIFKKCKKGILVNMDDNIVKHYSNEDTF
QLQIEEAGGSYKLTLTEI

>Callithrix jacchus_GRHL1(ENSCJAP_11987)
MTQEYDNKRPVLVLQNEALYPQRRSYTSEDEAWKSFLENPLTAATKAMMSINGDEDSAAA
LGLLYDYYKVPRERRSSTAKPEVEHPEPDHSKRNSIPIVTEQPLISAGENRVQVLKNVPF
NIVLPHGNQLGIDKRGHLTAPDTTVTVSIATMPTHSIKTETQPHGFAVGIPPAVYHPEPT
ERVVVFDRNLNTDQFSSGAQAPNAQRRTPDSTFSETFKEGIQEVFFPSDLSLRMPGMNSE
DYVFDSVSGNNFEYTLEASKSLRQKPGDSTMTYLNKGQFYPITLKEVSSNEGVHHPISKV
RSVIMVVFAEDKSREDQLRHWKYWHSRQHTAKQRCIDIADYKESFNTISNIEEIAYNAIS
FTWDINDEAKVFISVNCLSTDFSSQKGVKGLPLNIQIDTYSYNNRSNKPVHRAYCQIKVF
CDKGAERKIRDEERKQSKRKVSDVKVPLLPSHKRMDITVFKPFLDLDTQPVLFIPDVHFA
NLQRGTHVLPIASEELEGEGSALKRGPYGTEDDFSVPPSTKLSRIEEPKRVLLYVRKESE
EVFDALMLKTPSLKGLMEAISDKYDVPHDKIGKIFKKCKKGILVNMDDNIVKHYSNEDTF
QLQIEEAGGSYKLTLTEI

>Mus musculus_GRHL1(ENSMUSP_82689)
MTQEYDNKRPVLVLQNEALYPQRRSYTSEDEAWKSFLENPLTAATKAMMSINGDEDSAAA
LGLLYDYYKVPRERRSSAVKPEGEHPEPEHSKRNSIPNVTEQPLISAGENRVQVLKNVPF
NIVLPHSNQLGIDKRGHLTAPDTTVTVSIATMPTHSIKTEIQPHGFAVGIPPAVYHSEPT
ERVVVFDRSLSTDQFSSGTQPPNAQRRTPDSTFSETFKEGVQEVFFPSELSLRMPGMNSE
DYVFDNVSGNNFEYTLEASKSLRQKQGDSTMTYLNKGQFYPVTLKEGSSNEGIHHPISKV
RSVIMVVFAEDKSREDQLRHWKYWHSRQHTAKQRCIDIADYKESFNTISNIEEIAYNAIS
FTWDINDEAKVFISVNCLSTDFSSQKGVKGLPLNIQIDTYSYNNRSNKPVHRAYCQIKVF
CDKGAERKIRDEERKQSKRKVSDVKVQLLPSHKRTDITVFKPFLDLDTQPVLFIPDVHFT
NLQRGSHVLSLPSEELEGEGSVLKRGPFGTEDDFGVPPPAKLTRTEEPKRVLLYVRKESE
EVFDALMLKTPSLKGLMEAISDKYDVPHDKIGKIFKKCKKGILVNMDDNIVKHYSNEDTF

QLQIEEAGGSYKLTLTEI

>Rattus norvegicus_GRHL1(ENSRNOP_68887)
NSIPNVTEQPLISAGENRVQVLKNVPFNIVLPHSNQLGIDKRGHLTAPDTTVTVSIATMP
THSIKTEIQPHGFTVGIPPAVYHSEPTERVVVFDRSLSTDQFSSGPQPPNAQRRTPDSTF
SETFKEGVQEVFFPSELSLRMPGMTSEDYVFDSVSGNNFEYTLEASKSLRQKQGDSTMTY
LNKGQFYPVTLKEVSSNEGIHHPISKVRSVIMVVFAEDKSREDQLRHWKYWHSRQHTAKQ
RCIDIGEWPRSPPTSRRLQCPASQVLLLLAIINFLLKVFISVNCLSTDFSSQKGVKGLPL
NIQIDTYSYNNRSNKPVHRAYCQIKVFCDKGAERKIRDEERKQSKRKVSDVKVQLLPSHK
RTDITVFKPFLDLDTQPVLFIPDVHFTNLQRGSHVLSLPTEELEGDGSVLKRGPFGTEDD
FGVPPPAKLARTEEPKRVLLYVRKESEEVFDALMLKTPSLKGLMEAISDKYDVPHDKIGK
IFKKCKKGILVNMDDNIVKHYSNEDTFQLQIEEAGGSYKLTLTEI

>Bos taurus_GRHL1(ENSBTAP_9846)
MTQEYDNKRPVLVLQNEALYSQRRSYTSEDEAWKSFLENPLTAATKAMMSINGDEDSAAA
LGLLYDYYKVPRERRSSAAKADVEHAEPDHSKRNVPNVTEQPLISAGENRVQVLKNVPFN
IVLPHGNQLGIDKRGHPTAPDTTVTVSISAMPTHAIKTESQPHGFTVGIPPAVYHPEPTE
RVVVFDRNLNADQFGSGAQPPNAQRRTPDSTFSETFKEGVQEVFFPSDLSLRMPGMNSED
YVFDSVSGNNFEYTLEASKSLRQKPGDSTMTYLNKGQFYPVTLKEVSGSEAVHHPIGKVR
SVIMVVFAEDKSREDQLRHWKYWHSRQHTAKQRCIDIADYKESFNTISNIEEIAYNAISF
TWDINDEAKVFISVNCLSTDFSSQKGVKGLPLNIQIDTYSYNNRSNKPVHRAYCQIKVFC
DKGAERKIRDEERKQSKRKGKCTDPSSQLNAFSDVKVPLLPSHKRMDITVFKPFIDLDTQ
PVLFIPDVHFASLQRGAHVLPIASEELEGEGSSLKRGPYSAEDDFVIPPPAKLTRVEEPK
RVLLYVRKESEEVFDALMLKTPSLKGLMEAISDKYDVPQEKIGKIFKKCKKGILVNMDDN
IVKHYSNEDTFQLQMEESGGSFKLTLTEI

>Xenopus tropicalis_GRHL1(ENSXETP_15285)
MTQDYDNKRPVLVLQNDGLYQQRRSYTSEDEAWKSFLENPLTAATKAMMSINGDEDSAAA
LGLLYDYYKVPRERRPSAAKQEHDHADQEHNKRNGLPQINEQALLSENRVQVLKTVPFNI
VVPLANQVDKRGHLTTPDTTAAVSITSLPAHPIKTESQSHCFSVGLQSVFHTEPTERIVT
FDRTVPSDHFTSNSQPPNSQRRTPDSTFSETYKEDVPEVFFPPDLSLRMGSMNSEDYVFD
SVAGNNFEYTLEASKSLRPKPGDSTMTYLNKGQFYPITLKEIGGNKGIHHPISKVRSVIM
VVFADDKSREDQLRHWKYWHSRQHTAKQRCIDIADYKESFNTISNIEEIAYNAISFTWDL
NDEGKVFISVNCLSTDFSSQKGVKGLPLNLQIDTYSYNNRSNKPVHRAYCQIKVFCDKGA
ERKIRDEERKQSKRKVQDVKVGLPPSHKRTDITVFKPMMDLDTQPVLFIPDVHFANLQRT
THVLPIAPEDMEGELSSPGMKRVPFSPEEDFTAPPAKLPRVDEPKRVLLYVRRETEEVFD
ALMLKTPTLKGLMEAVSDKYEVPIEKIGKIFKKCKKGILVNMDDNIIKHYSNEDTFQLQI
EESGGSYKLTLTEI

>Danio rerio_GRHL1(ENSDARP_124180)
MSQEHENKRAVLVLPNDPAYNQRRPYTSEDEAWKSFLENPLTAATKAMMSINGDEDSAAA
LGLLYDYYKVPRDKRTISQQKTDVLGSDVDPNKRNMLTPLQETSMQLGDNRIQVLKGVPL
NIVLPGNQHVQDKRGLFPSPDTTVTVSIAPVASNSVKTEGPSHGFSVTVPNPHCAEPDSH
TVVFDRQLPHNQFSPNTQPRTPDSTFPENPDVFSFPGDLQLRMGPITQDDYGTFDTVSGN
NFEYILEASKSLRQKSGDGTMTYLNKGQFYPITLRETDNGKLLQGPICKVRSVVMVVFGE
EKSRDDQLKHWKYWHSRQHTAKQRCIDIADYKESFNTISNIEEISYNAISFTWDISEEAK
IFISVNCLSTDFSSQKGVKGLPLNIQIDTYSYNNRSNKPIHRAYCQIKVFCDKGAERKIR
DEERKQSRRKGKCPDVNPPLGNFGADVKVPLLHKRTDMTVFRTLTDFETQPVLFIPDIHF
STFQRHAFTAEDSEEGSAMKRLPYTEEEFGSPPNKLARMDEPKRVLLYVRRETEEVFDAL
MLKTPTLKGLVEAISEKYEVSLEKIGKVYKKCKKGILVNMDDNIIKHYSNEDTFQIQMEE
MGGMIKLTLTEIE

>Takifugu rubripes_GRHL1(ENSTRUP_34820)
MSQEHDNKRAVLVLQNEPGYGGQRRSFTTEDEAWKSFLENPLTAATKAMMSINGDEDSAA
ALGLLYDYYKVPREKRTIPQNKPEALICDVDPNKRHQLQAGHSITSLHDVGMENKIQVVK
GPFNILQLAQDKRSQFSSPDTTVTVSIATVPNYPPIKTEVPSHSFSVTMPNNCAETDGHA
GVFERQLAHNGVQFSPSTQSCTPPDSTFPESFKDSSQEVFSFPGELQLRMSSIASEEYPQ
FDAAPGNNFEYTLEASKSLRQKTGDGTMTYLNKGQFYPITLREIDNKGIQQPITKVRSVV
MVVFGEEKCRDDQLKHWKYWHSRQHTAKQRCLDIADYKESFNTIGNIEEISYNAISFTWD
TSDEAKIFISVNCLSTDFSSQKGVKGLPLNLQIDTYSYNNRSNKPIHRAYCQIKVFCDKG
AERKIRDEERKQSRRKVADVKVPILQKRNDVTIFKMMTDFETQPILFIPDIHFSTFQRHG

QYTLKAASETEEGEESSSGMKRLPFSDDEFASPPNKMAREEEPKKVLLYVRKETEEVFDA
VMLKDPSLKGLVEAISEKYELPLEKIGKVYKKCKKGILVHMDDNIIRHYSNEDTFQITME
ELGGMYKLTLTEI

>Tetraodon nigroviridis_GRHL1(ENSTNIP_20455)
MSQEHDNKRAVLVLQNEPGYGGQRRSFTTEDEAWKSFLENPLTAATKAMMSINGDEDSAA
ALGLLYDYYKVPREKRTIPQNKPEALICDVDPNKRHQIQAGHSIVPVHDAGVENKIQVVK
GPFNILQLAQDKRGQFSSPDTTVTVSIAAVPNYQPIKTEGPSHSFSVSIPNSCAETDGHV
GVFDRQLAHNGVPFSPGAQSRTPPDSTFPESFKDGSQEVFSFPGDLQLRMSSIASEEYPP
FDAVPGNNFEYTLEASKSLRQKTGDGTMTYLNKGQFYPITLREIDNKTVQQPITKVRSVV
MVVFGEEKCRDDQLKHWKYWHSRQHTAKQRCLDIADYKESFNTIGNIEEISYNAISFTWD
TNDEAKIFISVNCLSTDFSSQKGVKGLPLNLQIDTYSYNNRSNKPIHRAYCQIKVFCDKG
AERKIRDEERKQSRRKVVDVKVPILQKRNDVTVFKMMTDFETQPILFIPDIHFSTFQRHA
VPVSAQSSAHCSSGIKRLPFSDDEFASPPSKMAREEEPKKATVLLYVRKEAEEVFDAVML
KNPTLKGLVEAISEKYELPLEKMGKVYKKCKKGILVHMDDNIIKHYSNEDTFQITVEELG
GMYKLTLTEI

>Gasterosteus aculeatus_GRHL1(ENSGACP_11669)
MSQEHDNKRAVLVLQNEPGYAGQRRSFTTEDEAWKSFLENPLTAATKAMMSINGDEDSAA
ALGLLYDYYKVPRDKRTITQSKPEALVCDVDPNKRHQIQLGHPIPLQEGGLENRIQVLKG
PFNILQLAQDKRAQFASPDTTVTVSIAAVPNYTTVKTEGPAHGFSVTVPNNCAETAGHVG
VYDRQLAHNVVQFSPGTHTRTPPDSTFSESYKDGSQEVYSFPGDIQLRMAMSPEDYTPFN
TVLGNNFEYTLEASKSLRQKSGDGTMTYLNKGQFYPITLREADHKAMQQPITKVRSVVMV
VFGEEKCRDDQLKHWKYWHSRQHTAKQRCLDIADYKESFNTIGNIEEISYNAISFTWDTN
EEAKIFISVNCLSTDFSSQKGVKGLPLNLQIDTYSYNNRSNKPIHRAYSHIKVFCDKGAE
RKIRDEERKQTRRKVADAKVPLLQKRNDVTFFKTMSDHETQPVLFIPDIHFSTFQRQFPM
QPFPTEDVEESSGMKRLPFSDEEFGSPPNKMARGGEPKKVLLYVRKEAEEVFDALMLKNP
TLKGLVEAISEKYELPLDKVGKVYKKCKKGILVHMDDNIIKHYSNEDTFQITMEEMGGMY
KLTLTEI

>Oryzias latipes_GRHL1(ENSORLP_19084)
MSQEHDNFIPSKRAVVVLQNKQGYGNQRRSFTTEDEAWKSFLENPLTAATKAMMSINGDE
DSAAALGFLYDYYEVPRDKRTIHQPKTELLASDVDPNKRQGKSSLEICLQEANMDNRVQV
LKTPINILQLTSDKRTHFPSPDTTVTVSIATVPNYQSVKTEGPSHGFSVTVPNNCNDAGV
FDRQLNQNAIQFSPSTQSRTPPDSTFPESFKDGSQEEVFKHPPPQVKMFAFDQNCSNNFE
YSLEASKSLRQKTGDGTMTYLNKGQFYPITLREVDNKGIQQPITKVRSVVMVVFGEEKCR
EDQLKHWKYWHSRQHTAKQRCLDIADYKESFNTIDNIEEISYNAISFTWDTNEEAKIFIS
VNCLSTDFSSQKGVKGLPLNLQIDTYSYNNRSNKPIHRAYCQIKVFCDKGAERKIRDEER
KQSRRKGKVPDLSCNPPFADMKVPILQKRNDVTIFKMMNDLETQPVLFIPDIHFSALQRH
PMKSYNPFSAEEGEDRYVSHLLYISDDEFAPPPNKMPRVDEPKKVLLYVRKETEEVFDAL
MLKNPTLKGLMEAISEKYELPLDKIGKVYKKCKKGILVHMDDNIIKHYSNEDTFQITMED
QGGTYKLTLTEI

>Homo sapiens_GRHL2(ENSP_251808)
MSQESDNNKRLVALVPMPSDPPFNTRRAYTSEDEAWKSYLENPLTAATKAMMSINGDEDS
AAALGLLYDYYKVPRDKRLLSVSKASDSQEDQEKRNCLGTSEAQSNLSGGENRVQVLKTV
PVNLSLNQDHLENSKREQYSISFPESSAIIPVSGITVVKAEDFTPVFMAPPVHYPRGDGE
EQRVVIFEQTQYDVPSLATHSAYLKDDQRSTPDSTYSESFKDAATEKFRSASVGAEEYMY
DQTSSGTFQYTLEATKSLRQKQGEGPMTYLNKGQFYAITLSETGDNKCFRHPISKVRSVV
MVVFSEDKNRDEQLKYWKYWHSRQHTAKQRVLDIADYKESFNTIGNIEEIAYNAVSFTWD
VNEEAKIFITVNCLSTDFSSQKGVKGLPLMIQIDTYSYNNRSNKPIHRAYCQIKVFCDKG
AERKIRDEERKQNRKKGKGQASQTQCNSSSDGKLAAIPLQKKSDITYFKTMPDLHSQPVL
FIPDVHFANLQRTGQVYYNTDDEREGGSVLVKRMFRPMEEEFGPVPSKQMKEEGTKRVLL
YVRKETDDVFDALMLKSPTVKGLMEAISEKYGLPVEKIAKLYKKSKKGILVNMDDNIIEH
YSNEDTFILNMESMVEGFKVTLMEI

>Gorilla gorilla_GRHL2(ENSGGOP_27105)
MSQESDNNKRLVALVPMPSDPPFNTRRAYTSEDEAWKSYLENPLTAATKAMMSINGDEDS
AAALGLLYDYYKVPRDKRLLSVSKASDSQEDQEKRNCLGTSEAQSNLSGGENRVQVLKTV
PVNLSLNQDHLENSKREQYSISFPESSAIIPVSGITVVKAEDFTPVFMAPPVHYPRGDGE
EQRVVIFEQTQYDVPSLATHSAYLKDDQRSTPDSTYSESFKDATTEKFRSASVGAEEYMY

DQTSSGTFQYTLEATKSLRQKQGEGPMTYLNKGQFYAITLSETGDNKCFRHPISKVRSVV
MVVFSEDKNRDEQLKYWKYWHSRQHTAKQRVLDIADYKESFNTIGNIEEIAYNAVSFTWD
VNEEAKIFITVNCLSTDFSSQKGVKGLPLMIQIDTYSYNNRSNKPIHRAYCQIKVFCDKG
AERKIRDEERKQNRKKGKGQASQTQCNSSSDGKLAAIPLQKKSDITYFKTMPDLHSQPVL
FIPDVHFANLQRTGQVYYNTDDEREGGSVLVKRMFRPMEEEFGPVPSKQMKEEGTKRVLL
YVRKETDDVFDALMLKSPTVKGLMEAISEKYGLPVEKIAKLYKKSKKGILVNMDDNIIEH
YSNEDTFILNMESMVEGFKVTLMEI

>Mus musculus_GRHL2(ENSMUSP_22895)
MSQESDNNKRLVALVPMPSDPPFNTRRAYTSEDEAWKSYLENPLTAATKAMMSINGDEDS
AAALGLLYDYYKVPRDKRLLSVSKASDSQEDQDKRNCLGTSEAQINLSGGENRVQVLKTV
PVNLCLSQDHMENSKREQYSVSITESSAVIPVSGITVVKAEDFTPVFMAPPVHYPRADSE
EQRVVIFEQTQYDLPSIASHSSYLKDDQRSTPDSTYSESFKDGASEKFRSTSVGADEYTY
DQTGSGTFQYTLEATKSLRQKQGEGPMTYLNKGQFYAITLSETGDNKCFRHPISKVRSVV
MVVFSEDKNRDEQLKYWKYWHSRQHTAKQRVLDIADYKESFNTIGNIEEIAYNAVSFTWD
VNEEAKIFITVNCLSTDFSSQKGVKGLPLMIQIDTYSYNNRSNKPIHRAYCQIKVFCDKG
AERKIRDEERKQNRKKGKGQASQAQCNNSSDGKMAAIPLQKKSDITYFKTMPDLHSQPVL
FIPDVHFANLQRTGQVYYNTDDEREGSSVLVKRMFRPMEEEFGPTPSKQIKEENVKRVLL
YVRKENDDVFDALMLKSPTVKGLMEALSEKYGLPVEKITKLYKKSKKGILVNMDDNIIEH
YSNEDTFILNMESMVEGFKITLMEI

>Felis catus_GRHL2(ENSFCAP_4592)
LNKEQSSTKRLVALVPMPSDPPFNTRRAYTSEDEAWKSYLENPLTAATKAMMSINGDEDS
AAALGLLYDYYKVPRDKRLLSVSKASDSQEEPDKRNCLGTTEAQNNLTGGENRVQVLKTV
PVNLSLNQDHLENSKREQYSTNISESPAIIPVSGITVVKAEDFTPVFMAPPVHCPRGDGE
EQRVLIFEQTQYGVPSMASHSTYLKDDQRSTPDSTYSESFKDGTAEKFRSASVGAEEYMY
DQASSGTFQYTLEATKSLRQKQGEGPMTYLNKGQFYAITLSETGDNKCFRHPISKVRSVV
MVVFSEDKNRDEQLKYWKYWHSRQHTAKQRVLDIADYKESFNTIGNIEEIAYNAVSFTWD
VNEEAKIFITVNCLSTDFSSQKGVKGLPLMIQIDTYSYNNRSNKPIHRAYCQIKVFCDKG
AERKIRDEERKQNRKKGKGQPSQTQCNNSSDGKLAAIPLQKKSDITYFKTMPDLHSQPVL
FIPDVHFANLQRTGQVYYNTDDEREGSSVLVKRMFRPMEEEFGPAPSKQMKEEGMKRVLL
YVRKETDDVFDALMLKSPTVKGLMEAISEKYGLPVEKIAKLYKKSKKGILVNMDDNIIEH
YSNEDTFILNMESMVEGFKITLMEI

>Xenopus tropicalis_GRHL2(ENSXETP_14534)
MSQETDNKRLVVLVPNDAVFNPRRAYTSEDEAWKSYLENPLTAATKAMMSINGDEESAAA
IGLLYDYYKVPREKRLLSLNKINEGHEDQDKRNCLPANETPSNLSTGENRVQVLKTVPVN
LSLNNDTVESSNREKYTTSLSESPQPAPASAVTVVKAEEFTPVFMAPSVPYRSDGEEPRG
VIFEQTHFGVHSITTHSDYLKDDQRSTPDSTYNESFKETTEIFVPSQKYRTPSVGTEEFL
YEQTASSTFQYTLEATKSLRQKQGEGPMTYLNKGQFYAITLSETGANKCFRHPISKVRSV
IMVVFSEDKNRDEQLKYWKYWHSRQHTAKQRVLDIADYKESFNTIGNIEEIAYNAVSFTW
DVNEEAKIFITVNCLSTDFSSQKGVKGLPLMIQIDTYSYNNRSNKPIHRAYCQIKVFCDK
GAERKIRDEERKQNRKKGKSQAAQAQCNNSADGKLSAVPLQKKSDITFFKTMTDLDVQPV
LFIPDVHFGNLQRTGQVFYNTDDDIEGSGVLVKRLLRPVDEDYGPPAPKQMKEGSRKVLL
YVRKETDEVFDALMLKYPTVKGLLEAISEKYGIPVEKIVKIYKKSKKGILVNMDDNIIEH
YSNEDTFILNVESLAEQGYKITLTEI

>Takifugu rubripes_GRHL2(ENSTRUP_4868)
MSQETDNKRLVVVVPNESSYPSVRRAYTSEDEAWRSYLENPLTAATKAMMSINGDEDSAN
ALGLLYDYYKVPKEKRLLPVSKVSEITEEQEKRSTLTSNPSSQSEAESVDNHVQVLKSVP
VNLSLSTEPQEAKREPFTGSSGPAGAAGDGVSSTLTVVKAEMYAPVFMAGTGLHYRMEEE
EPASRVIYEHNPYDVTTVGHSSYVKEDQRSPPDSPYEEERDGTDDFLFPQERICRIIKSF
GFNSVVGACSDSFQYTLDATRSLRQKQGEVPMTYLNKGQFYAVTLNELSANKRLRQPISK
VRSVIMVVFSEDKNRDEQLKYWKYWHARQHTAKQRVLDIADYKESFNTISNIEEIAYNAI
SFTWDVNEEAKIFITVNCLSTDFSSQKGVKGLPLMIQIDTYSYNNRSNKPLHRAYSQIKV
FCDKGAERKIRDEERKLLRKKSKEGKTNPTVQLRMFVSSGKDGGVGVITIPKKSDTTFFK
TLTNLEAQPVLFIPDVHFGNLQRAGQVFAFNTEEIERDGGVLVKRMFRPSDEDLCPSPHK
QLKEDTQKRVLLYVRKETDEVFDALMLKSPTLGGLMDAISEKYGVPTERIAKVYKKSKKG
ILVNMDDNIIEHYSNEDTFILDIDSYADALKITLTEI

>Tetraodon nigroviridis_GRHL2(ENSTNIP_12011)

MSQETDSKRLVVVVPNESSFPAVRRAYTSEDEAWRSYLENPLTAATKAMMSINGDEDSAN
ALGLLYDYYKVVPKEKRLLPVSKVSETTDEQTKRSALASNPSGQSVAEAVDNHVQVLKSV
PVNLSLSTEPQEAKREPFTALTVVKAEMYAPVFMTGTGLHYRMEKEEEQASRVIYEQNPF
EATTGSHTSYVKEDQRSPPDSPFQEEQDGSAQKYHPPSALVTEDFLFPQEGVDSFQYTLD
ATRSLRQKQGEGPMTYLNKGQFYAVTLNELSANKRLRQPISKVRSVIMVVFSEDKNRDEQ
LKYWKYWHARQHTAKQRVLDIADYKESFNTISNIEEIAYNAISFTWDVNEEAKIFITVNC
LSTDFSSQKGVKGLPLMIQMDTYSYNNRSNRPLHRAYSQIKVFCDKGAERKIRDEERKLL
RKKSKGLGAEARADVGKDGGVGVMAVPKKSDTTFFKTMTNLEAQPVLFIPDVHFGNLQRA
GQVFAFNTEEMEREGGLLVKRMFRPSDEDLCPSPHKQLKEETQKRVLLYVRKEADEVFDA
LMLKSPTLRGLMDAISEKYGVPTERIAKVYKKSKKGILVNMDDNIIEHYSNEDTFILDID
SYADALKITLTEI

>Gasterosteus aculeatus_GRHL2(ENSGACP_4851)
MSQETDNNNNKRLVVVVPNESSYPAVRRAYTSEDEAWRSYLENPLTAATKAMMSINGDED
SANALGLLYDYYKVPKEKRLLPVPKVLEITEEQQKRSALPGNPDSQSEAETVDNRVQVLK
SVPVNLSLNTEPQEHKREHFSSLTEAAPGNGGSAAAVKAEAYAPVFMTGAGLHYRGEGEE
SSRVIYEQAAYEVTTVSHSSYGKEDQLSPPDSPYAEERDGMTIKKFHAPSSLATEDFLLH
QEGVDGFQYTLDATRSLRQKQGEGPMTYLNKGQFYGVTLNELSASKRLRHPISKVRSVVM
VVFSEDKNRDEQLKYWKYWHSRQHTAKQRVLDIADYKESFNTIGNIEEIAYNAISFTWDV
NEEAKIFITVNCLSTDFSSQKGVKGLPLMIQIDTYSYNNRSNKPLHRAYSQIKVFCDKGA
ERKIRDEERKLFRKKSKDIAMSCFCNNSKDGGVGVITVPKKADTTYFKTMSDLEAQPVLF
IPDVHFGNLQRAGQVFTFNTEEIEREGSVLVKRMFRRSDEDVCPSPHKQIKEETHKRVLL
YVRKETDEVFDALMLKSPTLRGLMDAISEKYGVATERIANVYKKSKKGILVNMDDNIIEH
YSNEDTFILSIDSYADAYKVTLMEI

>Oryzias latipes_GRHL2(ENSORLP_3636)
MSQETDKFSDSKRLVVVVPNENSFPAMRRAYTSEDEAWRSYLENPLTAATKAMMSINGDE
DSANALGLLYDYYKVPKEKRLLQIPKVAEVIEEQEKRSMLPSNSSNQSEPETLDNRVQVL
KSLPVNLSVNTEPQEAKREPFSTSTEPGSADAGSPPHAMVKAEGFPSVFMPGAGLHYRGE
GEEPTRVVFEQSPYEATTVSHSTYLKEDQKSPPDSPYDEDTDGHSKQKYHSSSSLAPDDF
LFHEAGVDSFQYTLDATRSLRQKQGEGPMTYLNKGQFYAVTLNELSANKRLRHPISKVRS
VIMVVFSQDKNRDEQLKYWKYWHSRQHTAKQRVLDIADYKESFNTINNIEEIAYNAISFT
WDVNEEAKLFFIVFLFLSSSSVSSGVSHLPHHNRSGEFAGQSNAVRPLHRLFCHLSVFFP
SQGAERKIRDEERKLFRKKSKGMTPSVSYINESKDSNVGVITVPKKSDTTYFKTMTDLEA
QPVLFIPDVHFGTLQRAGQVFAFNTEEMDREGGVLVKRMFRPSDEDLCPSPLKQIKEETH
KRVLLYVRKETEEVFDALMLKSPTLRGLMDAISEKYGVPTERIAKVYKKSKKGILVNMDD
NIIEHYSNEDTFILNIESCADSFKIILTEI

>Homo sapiens_TFCP2(ENSP_257915)
MAWALKLPLADEVIESGLVQDFDASLSGIGQELGAGAYSMSDVLALPIFKQEESSLPPDN
ENKILPFQYVLCAATSPAVKLHDETLTYLNQGQSYEIRMLDNRKLGELPEINGKLVKSIF
RVVFHDRRLQYTEHQQLEGWRWNRPGDRILDIDIPMSVGIIDPRANPTQLNTVEFLWDPA
KRTSVFIQVHCISTEFTMRKHGGEKGVPFRVQIDTFKENENGEYTEHLHSASCQIKVFKP
KGADRKQKTDREKMEKRTPHEKEKYQPSYETTILTECSPWPEITYVNNSPSPGFNSSHSS
FSLGEGNGSPNHQPEPPPPVTDNLLPTTTPQEAQQWLHRNRFSTFTRLFTNFSGADLLKL
TRDDVIQICGPADGIRLFNALKGRMVRPRLTIYVCQESLQLREQQQQQQQQQKHEDGDS
NGTFFVYHAIYLEELTAVELTEKIAQLFSISPCQISQIYKQGPTGIHVLISDEMIQNFQE
EACFILDTMKAETNDSYHIILK

>Callithrix jacchus_TFCP2(ENSCJAP_32500)
MAWALKLPLADEVIESGLVQDFDASLSGIGQELGAGAYSMSDVLALPIFKQEESSLPPDN
ENKILPFQYVLCAATSPAVKLHDETLTYLNQGQSYEIRMLDNRKLGELPEINGKLVKSIF
RVVFHDRRLQYTEHQQLEGWRWNRPGDRILDIDIPMSVGIIDPRANPTQLNTVEFLWDPA
KRTSVFIQVHCISTEFTMRKHGGEKGVPFRVQIDTFKENENGEYTEHLHSASCQIKVFKP
KGADRKQKTDREKMEKRTPHEKEKYQPSYETTILTECSPWPEITYVNNSPSPGFNSSHSS
FSLGEGNGSPNHQPEPPPPVTDNLLPTTTPQEAQQWLHRNRFSTFTRLFTNFSGADLLKL
TRDDVIQICGPADGIRLFNALKGRMVRPRLTIYVCQESLQLREQQQQQQQQQKHEDGDS
NGTFFVYHAIYLEELTAVELTEKIAQLFSISPCQISQIYKQGPTGIHVLISDEMIQNFQE
EACFILDTMKAETNDSYHIILK

>Bos taurus_TFCP2(ENSBTAP_25713)

MAWALKLPLADEVIESGLVQDFDASLSGIGQELGAGAYSMSDVLALPIFKQEESSLPPDN
ENKILPFQYVLCAATSPAVKLHDETLTYLNQGQSYEIRMLDNRKLGELPEINGKLVKSIF
RVVFHDRRLQYTEHQQLEGWRWNRPGDRILDIDIPMSVGIIDPRANPNQLNTVEFLWDPA
KRTSVFIQVHCISTEFTMRKHGGEKGVPFRVQIDTFKENENGEYTEHLHSASCQIKVFKP
KGADRKQKTDREKMEKRTPHEKEKYQPSYETTILTECSPWPEITYVNNSPSPGFNSSHSS
FSLGEGNGSPNHQPEPPPPVTDNLLPTTTPQEAQQWLHRNRFSTFTRLFTNFSGADLLKL
TRDDVIQICGPADGIRLFNALKGRMVRPRLTIYVCQESLQLREQQQQQNQQKHEDGDSN
GTFFVYHAIYLEELTAVELTEKIAQLFSISPCQISQIYKQGPTGIHVLISDEMIQNFQEE
ACFILDTMKETNDSYHIILK

>Gallus gallus_TFCP2(ENSGALP_54348)
MAWALKLPLADEVIESGLVQDFDASLSGIGQELGAGAYSMSDVLALPIFKQEESSLPPEN
ENKILPFQYVLCAATSPAVKLHDETLTYLNQGQSYEIRMLDNRKIGELPEINGKLVKSIF
RVVFHDRRLQYTEHQQLEGWRWNRPGDRILDIDIPMSVGIIDPRANPTQLNTVEFLWDPS
KRTSVFIQVHCISTEFTMRKHGGEKGVPFRVQIDTFKENENGEYTEHLHSASCQIKVFKP
KGADRKQKTDREKMEKRTPHEKEKYQPSYETTILTECSPWPEITYVNNAPSPGFNSSHSS
FSIGEGNGSPNHQPEPPPPIADNLLPTSTPQEAQQWLHRNRFSTFSRLFRNFSGADLLKL
TREDVIQICGPADGIRLFNALKGRMVRPRLTIYVCQESQQLQELQQKHEDGDAVTSTFFV
YHAIYLEELTAVELTEKLAQLFSISSQQISQIYKQGPTGIHVLISDEMIQNFQDESCFVL
DTMKAETNDSYHIILK

>Anolis carolinensis_TFCP2(ENSACAP_12164)
MAWALKLPLADEVIESGLVQDFDASLSGIGQELGAGAYSMSDVLALPIFKQEESNLPPDN
ENKILPFQYVLCAATSPAVKLHDETLTYLNQGQSYEIRMLDNRKMGELPELNGKLVKSIF
RVVFHDRRLQYTEHQQLEGWRWNRPGDRILDIDIPMSVGIIDPRANPTQLNTVEFLWDPS
KRTSVFIQVHCISTEFTMRKHGGEKGVPFRVQIDTFKENENGEYTEHLHSASCQIKVFKP
KGADRKQKTDREKMEKRTPHEKEKYQPSYETTILTECSPWPEITYVNNVPSPGFSSSHSS
FSISEGNGSPNHQPEPPAPIADNLLPTSTPQEAQQWLHRNRFSAFSRLFTNFSGADLLKL
TREDVIQICGPADGIRLFNALKGRMVRPRLTIYVCQESQQLSEQQKHEDGDAANSTFFVY
HAIYLEELTAVELTEKIAQLFSISAQQISQIYKQGPTGIHVLISDEMIQNFQDESCFILD
TMKAETNDSYHIILK

>Xenopus tropicalis_TFCP2(ENSXETP_21218)
MAWALKLPLADEVIESGLVQDFDASLSGIGQELGAGAYSMSDVLALPIFKQEESSLPSEN
ENKILPFQYVLCTATSPAVKLHEETLTYLNQGQSYEIRMLDNRKMGELPELNGKLVKSIF
RVVFHDRRLQYTEHQQLEGWRWNRPGDRILDIDIPMSVGIIDPRANPTQLNTVEFLWDPA
KRTSVFIQVHCISTEFTMRKHGGEKGVPFRVQIDTFKENENGEYTEHLHSASCQIKVFKP
KGADRKQKTDREKMEKRTPQEKEKYQPSYETTILTECSPWPEITYVSNAPSPGFNSSHNS
FPIGEGNGSPNHQPEPPAPIVDVSAGLTPIQNLLPTSTPQEAQQWLHRNRFAAFSRLFTN
FSGADLLKLTRDDVIQICGPADGIRLFNALKGRMVRPRLTIYVCQESQQLREQQQQHKHE
NGEAGNSAFYVYHAIYLEEMTAIELTEKIAQLFSISPHQINQIYKQGPTGIHVLISDEIM
FNHLICFVLPLAETNDSYHIILK

>Danio rerio_TFCP2(ENSDARP_121618)
MAWALKLPLADEVIESGLVQDFDASLSGIGQELGAGAYSMSDVLALPIFKQEESNLPPES
DNKILPFQYVLCAATSPAVKLHDETLTYLNQGQSYEIRMLDNRKMGELPEINGKMVKSII
RVVFHDRRLQYTEHQQLEGWRWNRPGDRILDLDIPMSVGVVDPRANPTQLNTVEFLWDPS
KRTSVFIQVHCISTEFTMRKHGGEKGVPFRIQIDTFKENEAGEYTEHLHSASCQIKVFKP
KGADRKQKTDREKMEKRAPQEKEKYQPSYETTILTECSPWPEATYVNNSPSPGFNSTHNS
FPVTEGNGSPSHQPEPVAQLADVSLSCHLLRRESESNQNLLPTATPQEAQQWLHRNRFSP
FCRLFTNFSGADLLKLTREDVIQICGPADGIRLFNALKGRVVRPRLTIYVCQESQQAREQ
HQKHENGDGTNNTFFVYHAIYLEDLTAVELTEKLAQLFSISPRQISQIFKQGPTGIHVLV
SDEMIQNFQDEVCFVLDTMKAETNDGYHIILK

>Takifugu rubripes_TFCP2(ENSTRUP_38080)
MAWALKLPLTDEVIESGLAQDFDASLSGIGQELGAGAYSMSDVLALPIFKQEESSLPSEN
ENMILPFQYVLCAPTSPAVKLHDETLTYLNQGQSYEIRMLDNRKIGELPEITGKMVKSII
RVVFHDRRLQYTEHQQLEGWRWNRPGDRILDLDIPMSVGIIDPRANPTQLNTVEFLWDPS
KRTSVFIQVHCISTEFTMRKHGGEKGVPFRVQIDTFKENENEEYTEHLHSASCQIKVFKP
KGADRKQKTDREKMEKRAPQEKEKYQPSYETTILTECSPWPEATYVNNSPSPGFNNTHNS
FPVSEGNGSPNHQPEPVVQVIDISKVSFCLQNLLPSATPQDAQQWLLRNRFSPFCRLFTN

FSGADLLKLTREDVIQICGPADGIRLFNALKGRVVRPRLTIYVCQESQQTREQQVKHENG
DAAINTFFVYHAIYLEEPTAAELTEKLAQLFNISPIQISQIFKQGPTGIHVLVSDEMIQN
FQDEVCFVLDTMKDDTNDGYHIILK

>Tetraodon nigroviridis_TFCP2(ENSTNIP_15497)
MAWALKLPLTDEVIESGLAQDFDASLSGIGQELGAGAYSMSDVLALPIFKQEESSLPSEN
ENMILPFQYVLCAPTSPAVKLHDETLTYLNQGQSYEIRMLDNRKIGELPEITGKMVKSII
RVVFHDRRLQYTEHQQLEGWRWNRPGDRILDLDIPMSVGIVDPRANPTQLNTVEFLWDPS
KRTSVFIQVHCISTEFTMRKHGGEKGVPFRVQIDTFKENENEEYTEHLHSASCQIKVFKP
KGADRKQKTDREKMEKRAPQEKEKYQPSYETTILTECSPWPEATYVNNSPSPGFNNTHNS
FPVAEGNGSPNHQPEPVVPAIDNLLPSATPQDAQQWLLRNRFSPFCRLFTNFSGADLLKL
TREDVIQICGPADGIRLFNALKGRVVRPRLTIYVCQESQQTREQQVKHENGDAAINTFFV
YHAIYLEELTAAELTEKLAQLFNISPRQISQIFKQGPTGIHVLVSDEMIQNFQDEVCFVL
DTMKDDTNDGYHIILK

>Gasterosteus aculeatus_TFCP2(ENSGACP_14466)
MAWALKLPLTDEVIESGLVQDFDASLSGIGQELGAGAYSMSDVLALPIFKQEESNLPPDN
ENKILPFQYVLCAATSPAVKLHDETLTYLNQGQSYEIRMLDNRKIGELPEINDKMAKSII
RVVFHDRRLQYTEHQQLEGWRWNRPGDRILDLDIPMSVGIIDPRANPTQLNTVEFLWDPS
KRTSVFIQVHCISTEFTMRKHGGEKGVPFRIQIDTFKENENEEYTEHLHSASCQFKVFKP
KGADRKQKTDREKMEKRTPQEKEKYQPSYETTILTECCPWPEVTYVGNSPSPGFNSTPNS
FPVPEGNGSPNHQPEPVVQVADNLLPTATPQDAQQWLLRNRFSPYCRLFTNFSAGADLLK
LTREDVIQICGPADGIRLFNALKGRVVRPRLTIYVCQESQQPREQQPKHENGDAAANTFF
VYHAIYLEELTAAELTEKLAQLFNISPRQINQIFKQGPTGIHVLVSDEMIQNYQDEVCFV
LDTMKDDTNDGYHIILK

>Oryzias latipes_TFCP2(ENSORLP_19587)
MAWALKLPLTDEVIESGLVQDFDASLSGIGQELGAGAYSMSDVLALPIFKQEESNLPADN
ENKILPFQYVLCAATSPAIKLHDETLTYLNQGQSYEIRMLDNRKIGELPEITGKMVKSII
RVVFHDRRLQYTEHQQLEGWRWNRPGDRILALDIPMSVGIIDPRANPTQLNTVEFLWDPS
KRTSVFIQVHCISTEFTMRKHGGEKGVPFRIQIDTFKENESGEYTEHLHSASCQVKVFKP
KGADRKQKTDREKMEKRTPQEKEKYQPSYETTILTECSPWPEVTYVNNSPSPGFNSSHNS
FPVAEGNGSPSHQPEPVVPVADNLLGTATPQDTQQWLLRNRFSPFCRLFTNFSVGADLLK
LTREDVIQICGPADGIRLFNALKGRAVRPRLTIYVCQESQQAREQQPKHENGDAASTFYV
YHAIYLEELTSTELTEKLAQLFNISPRQINQIFKQGPTGIHVLVSDEMIQNFQDEVCFVL
DTMKDETSDAYHIILK

>Homo sapiens_UBP1(ENSP_283628)
MAWVLKMDEVIESGLVHDFDASLSGIGQELGAGAYSMSDVLALPIFKQEDSSLPLDGETE
HPPFQYVMCAATSPAVKLHDETLTYLNQGQSYEIRMLDNRKMGDMPEINGKLVKSIIRVV
FHDRRLQYTEHQQLEGWKWNRPGDRLLDLDIPMSVGIIDTRTNPSQLNAVEFLWDPAKRT
SAFIQVHCISTEFTPRKHGGEKGVPFRIQVDTFKQNENGEYTDHLHSASCQIKVFKPKGA
DRKQKTDREKMEKRTAHEKEKYQPSYDTTILTEMRLEPIIEDAVEHEQKKSSKRTLPADY
GDSLAKRGSCSPWPDAPTAYVNNSPSPAPTFTSPQQSTCSVPDSNSSSPNHQGDGASQTS
GEQIQPSATIQETQQWLLKNRFSSYTRLFSNFSGADLLKLTKEDLVQICGAADGIRLYNS
LKSRSVRPRLTIYVCREQPSSTVLQGQQQAASSASENGSGAPYVYHAIYLEEMIASEVAR
KLALVFNIPLHQINQVYRQGPTGIHILVSDQMVQNFQDESCFLFSTVKAESSDGIHIILK

>Pan troglodytes_UBP1(ENSPTRP_25410)
MAWVLKMDEVIESGLVHDFDASLSGIGQELGAGAYSMSDVLALPIFKQEDSSLPLDGETE
HPPFQYVMCAATSPAVKLHDETLTYLNQGQSYEIRMLDNRKMGDMPEISGKLVKSIIRVV
FHDRRLQYTEHQQLEGWKWNRPGDRLLDLDIPMSVGIIDTRTNPSQLNAVEFLWDPAKRT
SAFIQVHCISTEFTPRKHGGEKGVPFRIQVDTFKQNENGEYTDHLHSASCQIKVFKPKGA
DRKQKTDREKMEKRTAHEKEKYQPSYDTTILTEMRLEPIIEDAVEHEQKKSSKRTLPADY
GDSLAKRGSCSPWPDAPTAYVNNSPSPAPTFTSPQQSTCSVPDSNSSSPNHQGDGASQAS
GEQIQPSATIQETQQWLLKNRFSSYTRLFSNFSGADLLKLTKEDLVQICGAADGIRLYNS
LKSRSVRPRLTIYVCREQPSSTVLQGQQQAASSASENGSGAPYVYHAIYLEEMIASEVAR
KLALVFNIPLHQINQVYRQGPTGIHILVSDQMVQNFQDESCFLFSTVKAENSDGIHIILK

>Mus musculus_UBP1(ENSMUSP_81946)
MAWVLSMDEVIESGLVHDFDSSLSGIGQELGAGAYSMSDVLALPIFKQEDSSLSLEDEAK

HPPFQYVMCAATSPAVKLHDETLTYLNQGQSYEIRMLDNRKMGDMPELSGKLVKSIIRVV
FHDRRLQYTEHQQLEGWKWNRPGDRLLDLDIPMSVGIIDTRTNPSQLNAVEFLWDPAKRT
SAFIQVHCISTEFTPRKHGGEKGVPFRIQVDTFKQNENGEYTDHLHSASCQIKVFKPKGA
DRKQKNDREKMEKRTAHEKEKYQPSYDTTILTEMRLEPIIEDAVEHEQKKSSKRTLPADY
GDSLAKRGSCSPWPDTPTAYVNNSPSPAPTFTSSQPSTCSVPDSNSSSPNHQGDGAAQAS
GEQIQPSATTQETQQWLLKNRFSSYTRLFSNFSGADLLKLTKEDLVQICGAADGIRLYNS
LKSRSVRPRLTIYVCQEQPSSTALQGQPQAAGSGGESGGGTPSVYHAIYLEEMVASEVAR
KLASVFNIPFHQINQVYRQGPTGIHILVSDQMVQNFQDETCFLFSTVKAENNDGIHIILK

>Canis familiaris_UBP1(ENSCAFP_6873)
MAWVLKMDEVIESGLVHDFDASLSGIGQELGAGAYSMSDVLALPIFKQEDSSLPLDGETK
HPPFQYVMCAATSPAVKLHDETLTYLNQGQSYEIRMLDNRKMGDMPEITGKLVKSIIRVV
FHDRRLQYTEHQQLEGWKWNRPGDRLLDLDIPMSVGIIDTRTNPSQLNAVEFLWDPAKRT
SAFIQVHCISTEFTPRKHGGEKGVPFRIQVDTFKQNENGEYTDHLHSASCQIKVFKPKGA
DRKQKTDREKMEKRTAHEKEKYQPSYDTTILTEMRLEPIIEDAVEHEQKKSSKRTLPADY
GDSLAKRGSCSPWPDTPTAYVNSSPSTAPAFTSPQQGTCSVPDSNSSSPNHQGDGVSQAC
SEQLQPSATIQETQQWLLKNRFSSYTRLFSNFSGADLLKLTKEDLVQICGAADGIRLYNS
LKSRSVRPRLTIYVCQEQPSSSPLQGQQPAGSGGDSGSGTPYVYHAIYLEEMVASEVARK
LAMVFNIPFHQINQVYRQGPTGIHILVSDQMVQNFQDESCFLFFTVKAENGDGIHIVLK

>Ciona intestinalis_(ENSCINP_23332)
YTSEDEAWRSYLENPLTAATTAMMSIHGDEDSAAALGILYDYYKVPREKRILTYQPNTGN
RDKQTRFKLCSKTSGVQQNLVPNPINLNLLRYLKDCYIKRDSCQKQSLFCRSISTYSKCV
SSQEGNISSTGLTSEDVSAMTPVISAASTAVHGDSSLEITVRDHSTEEVNGLGEYLNIGR
VQEDIRQLTPDSAYSEAKDSPPQMSQVYNFALTCFFNVLYSREYFEYAMEAPKSLKQKDG
EPTMSYINKGQFYCISLRECAGRPWRYKNTRVTSVVQIVFGDGKPEDEQLRHWKYWHARQ
HTAKQRIIDIADYKESCMISDIDEFAHNAISFNWDVNDVAKIFVSCNCLSTDFSAQKGIK
GLPLLLQIDTYMDNRRGAAPAHRGMCQLKVFCDKGAERKIRDEERKAMRKRQRSGMSPSI
TPPPTSAAPAGGAPGKRQEVVYFKTVVDGITPSVYFVPEIFVRNNSMTQHFYLDFSDPLR
GSTKRSLSMLSSDEGEFPSNKVLKRSEDTRVLLYVRRETDDVYDGIMLCDPTLEGLKRAL
EEKYGIPVQKMSKVLKKSRKGILVNVDDNIVRHYSNEDTFIIAVETETIDNDVTHKITLS
VV

>Ciona savignyi_(ENSCSAVP_18633)
PPINPLYTSEDEAWRSYLENPLTAASTAMMSIHGDEDSAAALGILYDYYKVPREKRILAY
QPSNKEKQNCRTTSINPQSENVNSGNQIRILTFLQTWSQINVRDHTPDELTSPLLKNFPH
SDVITLSTVRPELSALCEVSAHLFIYMFAVLHQVNGLGEYLNIGRTQDIKQLTPDSTYSE
AKESPPQITQPFGSPSSVATLPSMFSPTSEKYFEYAMEAPKSLKQKDGEPTMSYINKGQF
YCISLRECSGRPWRYQNSRVTSVVQIVFGDGKPEEEQLSHWKYWHARQHTAKQRIIDIAD
YKESCMISDIDEFAHNAISFSWDVNDVAKIFVSCNCLSTDFSAQKGIKGLPLLLQIDTYI
DNRRGVAPAHRGMCQLKVFCDKGAERKIRDEERKAIRKRQRSAQAPLRSPSITPPPAAGA
PGGTPGKRLDVVYFRTVFDYITPSVYFVPEVYRRILNNWYRKFRVHYRVTDDISPSFILL
KSELTLQIKLPMPKLSNNDDRVLLYVRKENDEVYDGIMLRESTVKGLKQAIEEKYGIPVQ
NISKVLKKSRKGILVNIDDNIVRHYSNEDTFIIDVETETLGNDVTHKITLSIV

>Branchiostoma floridae_(XP_002586459)
MTQEVDNSKRSVVVLQNEALYRNTYSSRDEDEAWRSYLENPLTAATTAMLNINGDEDSAAALGLLYDYYK
VPKEKRMNANYVTRTPMGPPETDQPSRDLTMLRNVPLADSITSSGFDGHHAHSSPVTTQPPATSPLTVRA
DHHIHIHKPDHSPMIVAKPDEMDRTPDSAFSEATFKDHQQEHDVLGDDMSAFQVFNYNISQVSLAAAHVQ
RRQQPELLQQVITETQIMPPPEPINGVAFEYILEAPKSLRQKPGEASMSYVNKGQFYAVTLTEAGSSPWR
HHSTKVRSVIQVVFGDGRSEEEQLKHWRYWHARQHTARQRIIDMADYKESTNVIENIDEIAHNAIAFSWD
VRETGKVFISVNCLSTDFSSQKGIKIDTYTDYFKGATPVHRAYVQIKVFCDKGAERKIRDEVRKISKKKQ
SEEIKSQIPSHKRNELAYFKPMADLKTPPVLFVPDFIPAARAPPQITQPVTFGLTPPFDRGMKRSFSEDS
AYSPESSPTPAAKVANKSVLLYVRKEEDDVYDALMLREPSVQGLLSAVEEKYKVPNDKVSKVFKKSKKGI
LVNIDDNIIEHYCNEDTFIIELQEETDGNIRVILTEVQDTP

>Drosophila melanogaster_(FBpp0312214)
MALSFLSQNSGLLDLQSIFDPQYTHHQQQQQLLHLSHHPQHHIQQHQNQQLQQQAEQIQ
QHQQERTLSTKFDLNLFNELDQMEFNNLNRSQYQNNNNNNSISNNQNSNNNTNTTNGISE
NLNQIQNRHFISGYHHQHIGSDYEQVINFVDSPPNSEESWTDGQSKDSPGPQIIDVQTIY
LNSGSRKRRMDWDSLDIGQSENSPTASQSGDLPSKVAHQEKEKHKREKHSGRSSWSDDIG

FDLNAEFNSNSYLNNENFLSFSPTLTTLKQEPQTDQIKPSPKVSLDNASASPSIAIAKLD
EVQNSPSQAISGQDSANGSGSAANGKHDVNSGLACGCGSPQGSPLANAEYELNEKGKPQQ
LSVLDPAKIEIGSANGATHAEDHKFQYILAAATSIATKNNEETLTYLNQGQSYEIKLKKI
GDLSLYRDKILKSVIKICFHERRLQFMEREQMQQWQQSRPGERIIEVDVPLSYGLCHVSQ
PLSSGSLNTVEIFWDPLKEVGVYIKVNCISTEFTPKKHGGEKGVPFRLQIETYIENTNSA
TASGSGGSNNSAIASGSGSSGSAAPASPERTPSAGSNGKQAVHAAACQIKVFKLKGADRK
HKQDREKIQKRPQSEQEKFQPSYECTIMNDISLDLVMSATTGCYSPEYMKLWPNSPVHI
PKYDGMLPFAPSAASPATSSSPIAINSVTSTNSPTLKLMDATNMVSPQHVPADMDDYSQN
IMPESTPSQVTQWLTNHRLTAYLSTFAQFSGADIMRMSKEDLIQICGLADGIRMFNILRA
KTIAPRLTLYASVDGCSYNAIYLLSNTAKELQQKLFKMPGFYEFMAKASAQENGAGGAAT
AAAAALFNNWGMHSKYSGSGSNIFNDANKSCVYISGPSGILVTITDEVLNNEIKDGSLYA
LEVQAGKVILKLINKQDNN

>Drosophila melanogaster_(FBpp0289766)
MSTSTATTSVITSNELSLSGHAHGHGHAHQLHQHTHSRLGVGVGVGILSDASLSPIQQGS
GGHSGGGNTNSSPLAPNGVPLLTTMHRSPDSPQPELATMTNVNVLDLHTDNSKLYDKEAV
FIYETPKVVMPADGGGGNNSDEGHAIDARIAAQMGNQAQQQQQQQQTEHQPLAKIEFDE
NQIIRVVGPNGEQQQIISREIINGEHHILSRNEAGEHILTRIVSDPSKLMPNDNAVATAM
YNQAQKMNNDHGQAVYQTSPLPLDASVLHYSGGNDSNVIKTEADIYEDHKKHAAAAAAAA
GGGSIIYTTSDPNGVNVKQLPHLTVPQKLDPDLYQADKHIDLIYNDGSKTVIYSTTDQKS
LEIYSGGDIGSLVSDGQVVVQAGLPYATTTGAGGQPVYIVADGALPAGVEEHLQSGKLNG
QTTPIDVSGLSQNEIQGFLLGSHPSSSATVSTTGVVSTTTISHHQQQQQQQQQQQQQQQQ
QHQQQQQHPGDIVSAAGVGSTGSIVSSAAQQQQQQQLISIKREPEDLRKDPKNGNIAGAA
TANGPGSVITQKILHVDAPTASEADRPSTPSSSINSTENTESDSQSVSGSESGSPGARTT
ATLEMYATTGGTQIYLQTSHPSTASGAGGGAGPAGAAGGGGVSMQAQSPSPGPYITANDY
GMYTASRLPPGPPPTSTTTFIAEPSYYREYFAPDGQGGYVPASTRSLYGDVDVSVSQPGG
VVTYEGRFAGSVPPPATTTVLTSVHHHQQQQQQQQQHQQQQQQQQHHQQQQHHSQDGKSN
GGATPLYAKAITAAGLTVDLPSPDSGIGTDAITPRDQTNIQQSFDYTELCQPGTLIDANG
SIPVSVNSIQQRTAVHGSQNSPTTSLVDTSTNGSTRSRPWHDFGRQNDADKIQIPKIFTN
VGFRYHLESPISSSQRREDDRITYINKGQFYGITLEYVHDAEKPIKNTTVKSVIMLMFRE
EKSPEDEIKAWQFWHSRQHSVKQRILDADTKNSVGLVGCIEEVSHNAIAVYWNPLESSAK
INIAVQCLSTDFSSQKGVKGLPLHVQIDTFEDPRDTAVFHRGYCQIKVFCDKGAERKTRD
EERRAAKRKMTATGRKKLDELYHPVTDRSEFYGMQDFAKPPVLFSPAEDMEKSFYGHETD
SPDLKGASPFLLHGQKVATPTLKFHNHFPPDMQTDKKDHILDQNMLTSTPLTDFGPPMKR
GRMTPPTSERVMLYVRQENEEVYTPLHVVPPTTIGLLNAIENKYKISTTSINNIYRTNKK
GITAKIDDDMISFYCNEDIFLLEVQQIEDDLYDVTLTELPNQ

>Apis mellifera_(XP_006558550)
MELQFIMDGSADALNGTIWFPNTPSPPYEQLSPVRQNRCDPIATQHAMGYHSAENSLRQDKYNSPKDSPE
DGVQIRSSSESPQHLSPSSDNSPNCQQSTLKRRAGEPLSQITELEKKHAKRDGSVGSNGSGQGWSTQVEE
LAEHLAADFDGSLSALAASDLATAVASYNMSEALLALPSLTVFKQEAPSPENQQNSNLNHVSQRTINSAP
TNNVNNSVEADSNNNGQTAASLHQLLYPSNEEYPPTSSSGNHVSSSQQGSELNEDCRFQYVLAAATSIAT
KVNEETLTYLNQGQSYEIKLKKLGDLSAYRGKILKSTIRICFHERRLQYTEREQMLAWQRARPGERLLEV
DVPLSYGMVDVCQPSPSNNSVEFMWDPTKEVGVYIKVNCISTEFTPKKHGGEKGVPFRIQVETRLPGGPR
LHAASCQVKVFKLKGADRKHKQDRDKILRRPPHEQDKYQPSYDCTVLSDIPLDSLSSSNVITQSGGSPYA
STEAISPQSRSTISGNTSPVVAPLALSVSNPGIVPLVPASDAVKENVGTAPALPSPAAILPDQSCIETER
SSCLVQLSPDASAAQTTTWLRASRFNAFESTFASFSASDILRLSRDDLIQICGLADGIRLFNALHSKAPT
PKLTLYFSLEDNSSLWRVAYLDSLTSSALINKLLNTLSLSHDRLHSVLFLGPQGIHVLVTDELVANMKDE
SMYFVETIKDNASERYKLLLKPSSRS

>Caenorhabditis elegans_(Y48G8AR)
MSFQLDQSTSVIKTAPPGRLEFKNEPIDIASSNIQDLAKMQTLDGYYQQTSLQLPQSQQY
HRMYENVYNNQQILYAQTQNFQYPSTLLTNGSLAQTEWRGQQMDTSAYSNTGYSRSSTSQ
QPQYTQQVTENERIVYKPVDMPSPVDSGIGGDISILNPKEEFFTSADGGSMLERSSERTL
SHRDSPLVIPKLYNNLGFQYVLEAPISTSVRRDDDRMTYVNKGQFYTVSLEYTPDLNKCL
KSQTVKSQLMVVFREDKTYEEEIKTWQSWHARQHVSKQRILEIDSKNSSGMIGQIEEIGN
NAVQFYWNPSDPSGVRISIAVQCLSTDFSTQKGVKGLPLHVQIDTYDGENDKVPFHRGYC
QIKVFCDKGAERKLRDEDKRAQKRKVQEYTAGALPGGRKKSDGEYHDQCERSEFYHMREL
DKPAALFIAPEEFEPRYVDSTSLSFDMSEIEPIPTKRPRTSERIMLYVRKRDEQIYQPLH
VVPASLSGLALAIANKFGADPDKMSGVYKRCAKGITVKVDDEMLRLYCNEDTFIIDVEHA
TDGSTAATLIEVAPTNPNSYSNS

>Nematostella vectensis_(XP_001636486)
MGFFGLFNRYTFILEAPTSIVQRRGDDTLTYLNKGQFYAIDFEGNFDPPSTEEDIIRVKSVVHLVFRDEK
DPRAELEHWNYWHSQQPNPQQRAFDIDRKSCQNIDENITDQAYNAAGFTWSPHLNAKIVIRINCLSTDFS
PQKGVKGIPLHLQIDTYEDVDNPDAEPVHRAFCQIKVFRDKGAERKNKDESKSAERR

Inhibitor of DNA Binding protein-ID

>Homo sapiens_ID1(ENSP_365280)
MKVASGSTATAAAGPSCALKAGKTASGAGEVVRCLSEQSVAISRCAGGAGARLPALLDEQ
QVNVLLYDMNGCYSRLKELVPTLPQNRKVSKVEILQHVIDYIRDLQLELNSESEVGTPGG
RGLPVRAPLSTLNGEISALTAEAACVPADDRILCR

>Pan troglodytes_ID1(ENSPTRP_59507)
MKVASGSTATAAAGPSCALKAGKTASGAGEVVRCLSEQSVAISRCAGGAGARLPALLDEQ
QVNVLLYDMNGCYSRLKELVPTLPQNRKVSKVEILQHVIDYIRDLQLELNSESEVGTPGG
RGLPVRAPLSTLNGEISALTAEAACVPADDRILCR

>Gorilla gorilla_ID1(ENSGGOP_15723)
MKVASGSTATAAAGPSCALKAGKTASGAGEVVRCLSEQSVAISRCAGGAGARLPALLDEQ
QVNVLLYDMNGCYSRLKELVPTLPQNRKVSKVEILQHVIDYIRDLQLELNSESEVGTPGG
RGLPVRAPLSTLNGEISALTAEAACVPADDRILCR

>Mus musculus_ID1(ENSMUSP_92019)
MKVASGSAAAAAGPSCSLKAGRTAGEVVLGLSEQSVAISRCAGTRLPALLDEQQVNVLLY
DMNGCYSRLKELVPTLPQNRKVSKVEILQHVIDYIRDLQLELNSESEVGTTGGRGLPVRA
PLSTLNGEISALAAEAACVPADDRILCR

>Rattus norvegicus_ID1(ENSRNOP_52261)
MKVASSSAAATAGPSCSLKAGRTAGEVVLGLSEQSVAISRCAGTRLPALLDEQQVNVLLY
DMNGCYSRLKELVPTLPQNRKVSKVEILQHVIDYIRDLQLELNSESEVATAGGRGLPVRA
PLSTLNGEISALAAEAACVPADDRILCR

>Bos taurus_ID1(ENSBTAP_21521)
MKVASGSAAAAAGPSCALKAGKAAGGAGEVVRCLSEQSVAISRCAGGPGARLPALLEEQP
VNVLLYDMNGCYSRLKELVPTLPQNRKVSRVEILQHVIDYIWDLELELNSESHVGTPGGR
GLPARAPLSTLNGEIGALAAEAACVPADDRILCR

>Loxodonta africana_ID1(ENSLAFP_17611)
MKVASGSASAAAGPSCALKAGKAAGGAGEVVRCLSEQSVAISRCAGGAGGRLPALLDEQQ
VNVLLYDMNGCYSRLKELVPTLPQNRKVSKVEILQHVIDYIWDLQLELDSQSAVGVPGSR
GLPARAPLSTLNGEMSALAAEAACVPADDRILCR

>Myotis lucifugus_ID1(ENSMLUP_19722)
MKVASGNAAAAAGPSCALKAGKTAGEVVRCLSEQSVAISRCAGGPGARLPALLDEQQVNV
LLYDMNGCYSRLKELVPTLPQNRKVSRVEILQHVIDYIWDLELELNSESQVGTPGARGLP
ARAPLSTLNGEISALAAEAACVPADDRILCR

>Monodelphis domestica_ID1(ENSMODP_24318)
MKVASGSAAAGPSCALKPGKVSGGAGEVVRCLSEQSVAISRCGGAGTRLPALLDEQQVNV
LLYDMNGCYSRLKELVPTLPQNRKVSKVEILQHVIDYIWDLQLELDSQAAGTPGARGLPA
RAPLSTLNGEMSALAAEAACVGADDRILCR

>Danio rerio_ID1(ENSDARP_59731)
MKVVGPTCALKSSKVGGEDVVRCLSDQSLAISKCKIPLLDEQMTMFLQDMNSCYSKLKEL
VPTLPTNKKASKMEILQHVIDYIWDLQVELESKKNQSSAPRTPLTTLNAELASISVENGC
SDDRIMCR

>Takifugu rubripes_ID1(ENSTRUP_24736)
PTVEATLTTMKVVGSTCALKSKVGSEEMVRCLSDQSLTISKCKIPLLDEQMAVLLQDMNS
CYSKLKELVPTLPTNKKASKVEILQHVIDYIWDLQVELDEPEKSRQLAGSVPRTPLTTLN

AELASIAVENGCSDDRIMCR

>Tetraodon nigroviridis_ID1(ENSTNIP_3643)
STVEATRATMKVVGSTCALKSKVGSEDVVRCLSEQSLAISKCKIPLLDEQMAVLLQDMNS
CYSKLKELVPTLPTNKKASKVEILQHVIDYIWDLQVELDEPEMSRQLSAASVPRTPLTTL
NAELASITVENGCSDDRIMCR

>Gasterosteus aculeatus_ID1(ENSGACP_13273)
MKVVGSTCALKSKVGGEEMVRCLSEQSLSIAKCKIPLLDEQMSAFLHDMNSCYSKLKELV
PTLPTNKKASKVEILQHVIDYIWDLQVELDEPEKSRHHSAGGVTRTPLTTLNAEIASIAV
ENGCSDDRIMCR

>Oryzias latipes_ID1(ENSORLP_17208)
ASVEATLLKMKVVGSTCALKTKVSGEMGLSDQSLAISKCKLPLLDEQMSVFLQDMNSCYS
KLKELVPTLPTNKKASKVEILQHVIDYIWDLQIELDEPEKSRQLSAASGQRTPLTTLNAE
LASIAVENGCSDERIMCR

>Homo sapiens_ID2(ENSP_379585)
MKAFSPVRSVRKNSLSDHSLGISRSKTPVDDPMSLLYNMNDCYSKLKELVPSIPQNKKVS
KMEILQHVIDYILDLQIALDSHPTIVSLHHQRPGQNQASRTPLTTLNTDISILSLQASEF
PSELMSNDSKALCG

>Macaca mulatta_ID2(ENSMMUP_4318)
MKAFSPVRSVRKNSLSDHSLGISRSKTPVDDPMSLLYNMNDCYSKLKELVPSIPQNKKVS
KMEILQHVIDYILDLQIALDSHPTIVSLHHQRPGQNQASRTPLTTLNTDISILSLQASEF
PSELMSNDSKALCG

>Callithrix jacchus_ID2(ENSCJAP_11113)
MKAFSPVRSVRKSSLSDHSLGISRSKTPVDDPMSLLYNMNDCYSKLKELVPSIPQNKKVS
KMEILQHVIDYILDLQIALDSHPTIVSLHHQRPGQSQTSRTPLTTLNTDISILSLQASEF
PSELMSNDSKALCG

>Mus musculus_ID2(ENSMUSP_20974)
MKAFSPVRSVRKNSLSDHSLGISRSKTPVDDPMSLLYNMNDCYSKLKELVPSIPQNKKVT
KMEILQHVIDYILDLQIALDSHPTIVSLHHQRPGQNQASRTPLTTLNTDISILSLQASEF
PSELMSNDSKVLCG

>Rattus norvegicus_ID2(ENSRNOP_9491)
MKAFSPVRSVRKNSLSDHSLGISRSKTPVDDPMSLLYNMNDCYSKLKELVPSIPQNKKVT
KMEILQHVIDYILDLQIALDSHPTIVSLHHQRPGQNQTSRTPLTTLNTDISILSLQASEF
PSELMSNDSKVLCG

>Felis catus_ID2(ENSFCAP_21760)
MKAFSPVRSVRKNSLSDHSLGISRSKTPVDDPMSLLYNMNDCYSKLKELVPSIPQNKKVS
KMEILQHVIDYILDLQIALDSHPTIVSLHHQRPGQSQASRTPLTTLNTDISILSLQASEF
PSELMSNDSKALCG

>Bos taurus_ID2(ENSBTAP_28235)
MKAFSPVRSVRKNSLSDHGLGISRSKTPVDDPMSLLYNMNDCYSKLKELVPSIPQNKKVS
KMEILQHVIDYILDLQIALDSHPTIVSLHHQRPGQSQASRTPLTTLNTDISILSLQASEF
PSELMSNDSKALCG

>Monodelphis domestica_ID2(ENSMODP_18263)
MKAFSPVRSVRKNSLSDHNLGISRSKTPVDDPMSLLYNMNDCYSKLKELVPSIPQNKKVS
KMEILQHVIDYILDLQIALDSHPSIVSLHHQRPGQNPSSRTPLTTLNTDISILSLQASEF
PSELMSNESKALCG

>Gallus gallus_ID2(ENSGALP_36138)
MKAFSPVRSVRKNGLSEHNLGISRSKTPVDDPMSLLYNMNDCYSKLKELVPSIPQNKKVS
KMEILQHVIDYILDLQIALDSHPSIVSLHHQRPGQNAASRTPLTTLNTDISILSLQAAEF
PSELMASDSKALCG

>Pelodiscus sinensis_ID2(ENSPSIP_4744)
MKAFSPVRSVRKNSLSEHTLGISRSKTPVDDPMSLLYNMNDCYSKLKELVPSIPQNKKVS
KMEILQHVIDYILDLQIALDSHPSIVSFHHQRPGPNPSSRTPLTTLNTDISILSLQASEF
PSELMSNDSKALCG

>Danio rerio_ID2(ENSDARP_72092)
MKAISPVRSFRKSSASVTTTEHSLGISRSKTPVDDPLSLLYNMNDCYSKLKELVPSIPQN
KNVSKMEILQHVIDYILDLQIALDSNSAITSLHHPRAGQGTSRTPLTTLNTDISILSLQT
PEFPSDLITEDSRTLYR

>Takifugu rubripes_ID2(ENSTRUP_37209)
MKAISPVRSFRKNNANFSEHSLGISRSKTPVDDPLSLLYNMNDCYSKLKELVPSIPQNKN
VSKMEILQHVIDYILDLQIALDSSVALNSRLHHPTRPGQTPSRTPLTTLNTDISILSLQS
PELPSELMTDDSRTLHR

>Tetraodon nigroviridis_ID2(ENSTNIP_20446)
MKAISPVRSFRKNNANFSEHSLGISRSKTPVDDPLSLLYNMNDCYSKLELVPSIPQNKNV
SKMEILQHVIDYILDLQIALDSNVALNSRLHHPARPGQTPSRTPLTTLNTDISILSLQSP
ELPSELMTDDSRTLHR

>Gasterosteus aculeatus_ID2(ENSGACP_11916)
MKAISPVRSFRKNNANISEHSLGISRSKTPVDDPLSLLYNMNDCYSKLKELVPSIPQNKN
VSKMEILQHVIDYILDLQIALDSSVALTSLHHPARPGQAPSRTPLTTLNTDISILSLQAT
YEQHRTPCQ

>Oryzias latipes_ID2(ENSORLP_18816)
MKAISPVRSFRKNAVNLTEHSLGISRSKTPVDDPLSLLYNMNDCYSKLKELVPSIPQNKN
VSKMEILQHVIDYILDLQIALDSSVALSSLHHPARPGQTGASRPPLTTLNTDISILSLQS
PELPSELMTDDSRTLHR

>Homo sapiens_ID3(ENSP_489102)
MKALSPVRGCYEAVCCLSERSLAIARGRGKGPAAEEPLSLLDDMNHCYSRLRELVPGVPR
GTQLSQVEILQRVIDYILDLQVVLAEPAPGPPDGPHLPIQTAELTPELVISNDKRSFCH

>Pongo abelii_ID3(ENSPPYP_2013)
MKALSPVRGCYEAVCCLSERSLAIARGRGKGPAAEEPLSLLDDMNHCYSRLRELVPGVPR
GTQLSQVEILQRVIDYILDLQVVLAEPAPGPPDGPHLPIQTTELSPELVISNDKRSFCH

>Mus musculus_ID3(ENSMUSP_8016)
MKALSPVRGCYEAVCCLSERSLAIARGRGKSPSTEEPLSLLDDMNHCYSRLRELVPGVPR
GTQLSQVEILQRVIDYILDLQVVLAEPAPGPPDGPHLPIQTAELTPELVISKDKRSFCH

>Rattus norvegicus_ID3(ENSRNOP_35270)
MKALSPVRGCYEAVCCLSERSLAIARGRGKSPSAEEPLSLLDDMNHCYSRLRELVPGVPR
GTQLSQVEILQRVIDYILDLQVVLAEPAPGPPDGPHLPIQTAELTPELVISKDKRSFCH

>Canis familiaris_ID3(ENSCAFP_19544)
SGAAAGVRALPAAVGAPWLSCSSRHPASLCNMKALSPVRGCYEAVCCLSERSLAIARGRG
KGPAAEEPLSLLDDMNHCYSRLRELVPGVPRGTQLSQVEILQRVIDYILDLQVVLAEPAP
GPPDGPHLPIQTAELAPELVISNDKRSFCH

>Felis catus_ID3(ENSFCAP_23941)
MKALSPVRGCYEAVCCLSERSLAIARGRGKGPAAEEPLSLLDDMNHCYSRLRELVPGVPR
GTQLSQVEILQRVIDYILDLQVVLAEPAPGPPDGPHLPIQTAELAPELVIANDKRSFCH

>Pteropus vampyrus_ID3(ENSPVAP_8330)
MKALSPVRGCYEAVCCLSERSLAIARGRGKGPAAEEPLSLLDDMNHCYSRLRELVPGVPR
GTQLSQVEILQRVIDYILDLQVVLAEPAPGPPDGPHLPIQTAELSPELVISNDKRSFCH

>Ornithorhynchus anatinus_ID3(ENSOANP_19268)

MKAISPVRSVRGCYEAVCCLSEQSLAIARGGGGKRPALEEPLGLLYDMNGCYSRLRELVP
GIPRGTTLSQVEILQHVIDYILDLQGALEEPAAGAGPPTGPLLSLKAAELASELYSGDER
RLC

>Gallus gallus_ID3(ENSGALP_42729)
MKAISPVRSVRSCYEAVCCLSEQSLAIARSSNNKSPALEEPMNLLYDMNDCYSKLRELVP
GIPQGTKLSQVEILQHVIDYIFDLQIVLEEGAKGRDPSSEATLLSLKAAELASGLCSKDE
RSLCH

>Taeniopygia guttata_ID3(ENSTGUP_927)
MKAISPVRSVRSCYEAVCCLSEQSLAIARGSHNKSPALEEPMNLLYDMNDCYSKLRELVP
GIPQGTKVSQVEILQHVIDYIFDLQIVLEEGSKGRDPSSEATLLSLKAAELAS

>Pelodiscus sinensis_ID3(ENSPSIP_18126)
MKAISPVRSVRSCYEAVCCLSDQSLAIARGSHNKSPALEEPMSLLYDMNDCYSKLRELVP
GIPQGSKLSQVEILQHVIDYIFDLQIVLEEQAKGQDSSSDTSLLSRKAAEFASELCSKDE
RRLCH

>Danio rerio_ID3(ENSDARP_71555)
MKAISPVRSVRSCYEAVCCISEQSLAISRCKSPSEELSDMNMNDCYSKLKELVPSIPQNK
SVSQVEILQHVIDYIFDLQIALENETDTQNTPDIFLSMKNSEMSRNFSKEDGAMCH

>Takifugu rubripes_ID3(ENSTRUP_29951)
MKAISPVRSARSCYKAVCCISEQSLVIGRNKHSCLEEPVSALHDMNDCYSRLKQLVPSIP
QNESVSQVEILQHVIDYIFDLQIALQAEEAAAPKMVLSIKTKDLTHNFSKEGERLYH

>Tetraodon nigroviridis_ID3(ENSTNIP_1755)
MKAISPVRSARSCYKAVCCISEQSLVISRNKHSCVEEPVGALRDMNDCYSRLKELVPSIP
QDQSVSQVEILQHVIDYIFDLQIALQAEEAAAPKMVLSIKVRTDGACVSADISWDSNRVC
VCS

>Gasterosteus aculeatus_ID3(ENSGACP_12641)
MKAISPVRSVRSCYKAVCCISEQSLAINRNKHSCLEEPLGALCDMNDCYSKLKELVPSIP
QNKSVSQVEILQHVIDYIFDLQIALEAEDAATPEMVLSIKAADLARNFSKEEGRLCH

>Oryzias latipes_ID3(ENSORLP_17983)
MKAISPVRSVRNCYKAVCCISEQSLAINRTNKHCCEDEPVSALCDMNDCYSRLKELVPSI
PQNKSVSQVEILQHVIDYIFDLQIALEAVDPSAPEMVLSIKTSDITRNFSKEEGRLCH

>Homo sapiens_ID4(ENSP_367972)
MKAVSPVRPSGRKAPSGCGGGELALRCLAEHGHSLGGSAAAAAAAAAARCKAAEAAADEP
ALCLQCDMNDCYSRLRRLVPTIPPNKKVSKVEILQHVIDYILDLQLALETHPALLRQPPP
PAPPHHPAGTCPAAPPRTPLTALNTDPAGAVNKQGDSILCR

>Pongo abelii_ID4(ENSPPYP_23841)
MKAVSPVRPSGRKAPSGCGGGELALRCLAEHGHSLGGSAAAAAAAAAARCKAAEAAADEP
ALCLQCDMNDCYSRLRRLVPTIPPNKKVSKVEILQHVIDYILDLQLALETHPALLRQPPP
PAPPHHPAGTCPAAPPRTPLTALNTDPAGAVNKQGDSILCR

>Macaca mulatta_ID4(ENSMMUP_19275)
MKAVSPVRPSGRKAPSGCGGGELALRCLAEHGHSLGGSAAAAAAAAAARCKAAEAAADEP
ALCLQCDMNDCYSRLRRLVPTIPPNKKVSKVEILQHVIDYILDLQLALETHPALLRQPPP
PAPPHHPAGTCPAAPPRTPLTALNTDPAGAVNKQGDSILCR

>Mus musculus_ID4(ENSMUSP_21810)
MKAVSPVRPSGRKAPSGCGGGELALRCLAEHGHSLGGSAAAAAAAAAARCKAAEAAADEP
ALCLQCDMNDCYSRLRRLVPTIPPNKKVSKVEILQHVIDYILDLQLALETHPALLRQPPP
PAPPLHPAGACPVAPPRTPLTALNTDPAGAVNKQGDSILCR

>Equus caballus_ID4(ENSECAP_11106)
PALCLQCDMNDCYSRLRALVPTIPPNKKSKVEILQHVIDYILDLQLALETHPALLRQPPP

PAPPHHPAGTCPSAPPRTPLTALNTDPAGAVNKQGDSILCR

>Monodelphis domestica_ID4(ENSMODP_14146)
MKAVSPVRPSGRKAPSGCGGELALRCLAEHSHSLGGSAAAVAAAAAARCKAAEAAAEEPS
LCLQCDMNDCYSRLKKLVPTIPPNKKVSKVEILQHVIDYILDLQLALETHPALLRQQPPP
PPPLHHPSSGSCPAGPPRTPLTALNTDPAGAVNKQGDSILCR

>Gallus gallus_ID4(ENSGALP_50623)
MKAVSPVRHPGRRAQPAGAGGGGGHPALRCLAEHSGCKGGEEPAALCLQCDMNDCYSRLR
RLVPTIPPNKRVSKVEILQHVIDYILDLQLALETHPALLRQPPPTLHPGGCPAGTPRTPL
TALNTDPVRGWLC

>Anolis carolinensis_ID4(ENSACAP_3085)
DEPAALCLQCDMNDCYSRLRKLVPTIPPHKRVSKVEILQHVIDYILDLQLALETHPALLR
QPPQQQQQQLLAPHQAPRTPLTALNVDPVR

>Xenopus tropicalis_ID4(ENSXETP_54646)
APVAAAMKAVSPVRPQSRKAQVPAVCGELALHCLSEHSLGVARYKMEEEETLCLQYDMND
CYSRLKRLVPTIPPNKKVSKVEILQHVIDYILDLQLALDTHPVLLRQQPPTRTPLTDLNT
DPAALVVNKQGDSILCR

>Danio rerio_ID4(ENSDARP_66371)
MKASVPVRPHKLPSSCSQLSLRYLSESSRCKMEDEDLFCLQYDMNDCYSRLKRLVPTIPQ
DKKVSKVEILQHVIDYILDLQLALETHPALLKQTSPPASTRTPLTQINTEQRTTGVNKED
SLFLCR

>Takifugu rubripes_ID4(ENSTRUP_30900)
MKAVTPVRPQDSSSGSNQLSLHCLTKQSYNIARCRMEEEDLFCLQYDMNDCYSRLKRLVP
TIPQDKKVSKVEILQHVIDYILDLQLALETHPSLHKQQSGTCPPPASKPSRTPLTVLNVD
HHQGPGRKNTCPV

>Gasterosteus aculeatus_ID4(ENSGACP_4826)
MKAVTPVHPQDSSSGGSPLSLHYLSKESLNIARCRVEDEDLFCLQYDMNDCYSRLKRLVP
TIPQDKKVSKVEILQHVIDYILDLQLALETHPSLHKQQPQRTGTCPAPASNPSRTPLTPL
NVDHHQRTSIVKKPEDSILCR

>Oryzias latipes_ID4(ENSORLP_3198)
MKAVSPVRPQDPSSSSSQLSLHYLSKQSLNIARCRMEEEDLFCLQYDMNDCYSRLKRLVP
TIPQDKKVSKVEILQHVIDYILDLQLALETHPALHIQQRLGTCPPAASNPNRTPLTVLNV
DHQRTSVVKKPEDSVLCR

>Saccoglossus kowalevskii_(NP_001161570)
MKERQGNRATKLHNSTSALRHVSKESVETCSRGNMVAAAAAVLAAGSMSEYLDGILTYSMSDCYSKLKDL
VPTIPPNKKVSKVELLQHVIDYIQDLQVELEAHPAMRNTPPPPLEKQIPTNRTPLGTIPPENTAIAADQV
SHLEKSASCSWDIDSCSPRSC

>Ptychodera flava_(AJS19026)
ETKDSRPSKLHNSTLSLRHVSKDSHSISSCKGNTAAAAAALAAVGPMSSYLDGILTYSMQDCYSKLKDLV
PSIPPNEKVSKVELLQHVIDYIQDLQTALESHPALRNSPPPPLMEKQNSNRTPLGTIPPENTAILSDQVS
HLEKTTNNTNWEDSCSPRSC

>Strongylocentrotus purpuratus_(XP_780755)
MKQPPQMQTAVQHQPYPAALHHTLPQHAGGEKKQKRAGVSNFTMSDCYEKLKQLVPTIPKNRKVTRVEIL
QHVIDYIQDLQTALEGKNIDVKMMGSVNALTGMITDSMAMPAPSPAASNRMPFTTIVPQSTQQQSHQQMT
CPPATPLGVHTQSNVRQQQQQQCYPQPQHTSLFHHQGYSDYHQRQQMHMNMLQQHPGSSSKVLPHPYETP
ESSPESRPCSC

>Nematostella vectensis_(XP_001636690)
MKPQSKPTDSKFEEALRATIRSASSITRASIFSEFERDSNDNMSECYERLKNMVPNVPVGRRISKVEILQ
YVIDYILDLQTALENQTANRRRRRSNRVNRSPLSTISLNEDIEKLRNDIAFFKIAENK

>Hydra magnipapillata_(XP_012554514)
MVVMTPNMTDKKGLKNTSNEETKKKVELEIKNENLKPEVFETFLPTTISYHSNCLPDAIFLTTSNGQLIT
TSTINYSIFLTDITSSSVTNKQETKKDKDEEHLIAAAATPSASQLCSSATSKEENIFSPKFPFLTTSSQS
DNNITKTFAPVFSLSNPLLAPNLAIPIYNTQPTTTGVGDSFKNIFVTTPQAKPNPSKSKESGNSESDSHN
MATSRGRKRTAQSRLPAKLKEPAAVARRNARERRRVKMVNDGFLRLRRHVPTDPKNKKLSKVKTLRLAIE
YIHHLQHLLQVDNCSQQTLQLVANISQVSSFDDIDGGNAWLNTEAMNRVNREMDEDIFQTIDPARLRTNY
TDYITR

>Trichoplax adhaerens_(XP_002110381)
MNPLSVDSISPVDSDSNLSQYSFTNSSIPSISSSKTSTTITMNQSDPCLDTSSGYLPNHQSNQFVLSSYQ
NDEEETTVHLNNQSKRKHLKKSKSKESVTAKRRNERERRRVQLVNDGFSELRKHVPTDSENRKLSKVKTL
RSAIEYIKQLQSLLEKQTVPEAPRRKVYARQSMLKKSNVKGKTNLGYINFEVLLENISDSVNERTYELAD
VELDYLCHLRIIIYISGNRAWIQ

>Amphimedon queenslandica_(XP_003387453)
MKRHLSDSETEMNEEYEECSSPDTAQVVSRKKRRGIIEKRRRDRINNCLMELRRLVPAAFEKQGSAKLEK
AEILQMTVDHLRHLHQTRDPRGFTDPLSAYSNTRAFLDYRVMGFRECVAEVARYMTNVEGLDMKDPMRVR
VLNHLENYLTQRELAINAAVAASAQMGLPKLSPPATLPPAGIAIQHATGPFIPIPAHRASPDGNLSPPTT
FALPTPVISFAPTTATVPVFGAAHIATPNAHPPIISLAKIEGSVQATPISPTKKFCSSTSSSPSATKQPF
RPWADPTDDD

Maestro Heat-like Repeat-containing Protein Family-MROH

>Homo sapiens_MROH5(ENSP_481783)
MDRQCSERPYSCTPTGRVSSAVSQNSRISPPVSTSMKDSSCMKVHQDSARRDRWSHPTTI
LLHKSQSSQATLMLQEHRMFMGEAYSAATGFKMLQDMNSADPFHLKYIIKKIKNMAHGSP
KLVMETIHDYFIDNPEISSRHKFRLFQTLEMVIGASDVLEETWEKTFTRLALENMTKATE
LEDIYQDAASNMLVAICRHSWRVVAQHLETELLTGVFPHRSLLYVMGVLSSSEELFSQED
KACWEEQLIQMAIKSVPFLSTDVWSKELLWTLTTPSWTQQEQSPEKAFLFTYYGLILQAE
KNGATVRRHLQALLETSHQWPKQREGMALTLGLAATRHLDDVWAVLDQFGRSRPIRWSLP
SSSPKNSEDLRWKWASSTILLAYGQVAAKARAHILPWVDNIVSRMVFYFHYSSWDETLKQ
SFLTATLMLMGAVSRSEGAHSYEFFQTSELLQCLMVLMEKEPQDTLCTRSRQQAMHIASS
LCKLRPPIDLERKSQLLSTCFRSVFALPLLDALEKHTCLFLEPPNIQLWPVARERAGWTH
QGWGPRAVLHCSEHLQSLYSRTMEALDFMLQSLIMQNPTADELHFLLSHLYIWLASEKAH
ERQRAVHSCMILLKFLNHNGYLDPKEDFKRIGQLVGILGMLCQDPDRATQRCSLEGASHL
YQLLMCHKTGEALQAESQAPKELSQAHSDGAPLWNSRDQKATPLGPQEMAKNHIFQLCSF
QVIKDIMQQLTLAELSDLIWTAIDGLGSTSPFRVQAASEMLLTAVQEHGAKLEIVSSMAQ
AIRLRLCSVHIPQAKEKTLHAITLLARSHTCELVATFLNISIPLDSHTFQLWRALGAGQP
TSHLVLTTLLACLQERPLPTGASDSSPCPKEKTYLRLLAAMNMLHELQFAREFKQAVQEG
YPKLFLALLTQMHYVLELNLPSEPPKQQAQEAAVPSPQSCSTSLEALKSLLSTTGHWHDF
AHLELQGSWELFTTIHTYPKGVGLLARAMVQNHCRQIPAVLRQLLPSLQSPQERERKVAI
LILTKFLYSPVLLEVLPKQAALTVLAQGLHDPSPEVRVLSLQGLSNILFHPDKGSLLQGQ
LRPLLDGFFQSSDQVIVCIMGTVSDTLHRLGAQGTGSQSLGVAISTRSFFNDERDGIRAA
AMALFGDLVAAMADRELSGLRTQVHQSMVPLLLHLKDQCPAVATQAKFTFYRCAVLLRWR
LLHTLFCTLAWERGLSARHFLWTCLMTRSQEEFSIHLSQALSYLHSHSCHIKTWVTLFIG
HTICYHPQAVFQMLNAVDTNLLFRTFEHLRSDPEPSIREFATSQLSFLQKVSARPKQ

>Macaca mulatta_MROH5(ENSMMUP_31900)
MDGQCSERPHSHTPTGRMSSAVSQNSRISLPVSTSTKDSSCMKAPQDSACRDRWSHPTTI
LLHKSQSSQAALMPQEHGMFMAEAYSAAICLKMLQDMNGADLFHLKYIIKKIKNMARGSP
KLVMETIHNYFIDNPEISSRHKFRLFQTLEMVIRASDVLEETWEKTFMQLALENMTKATE
LEDTYQDAASNVLVAICRHSWQAVAQHLETELLTGIFPHRSLLYVMGVLSSSEELFSQED
KACWEEQLIQMAIKSVPFLSMDVWSKELLWTLTTPSRTQQEQSPEKAFLFTYYGLILQAE
KNSATVRRHLQALLETSHQWPKQREGMALTLGLAATRHLDDVWAVLDQFGRSRPIRWSLH
SSSPKNSEDLRWKWASSTILLAYGQMAAKARAHILPWVDNIVSRMVFYFHYSSWDETLKQ
SFLTATLMLMGAVSRNEGAHSYEFSQTSELLECLMVLMEKEPQDTLYTRSRQQTMHIASS
LCKLRPPIDLERKSQLLSTCFRSVFALPLLDALEKHTCLFLEPPNIQVWPMAREWAGWMH
QGWGPGAVLHCSELLQNLYSRTMEALDLMLQSLIMQNPTTDELHFLLSHLYIWLASEKAH
ERQRAVHSCMILLKFLNHKGNLDPKENFKRIGQLVGILGMLCQDPDRAIQRCSLEGASHL
YQLLMCHKGEFCGAPTSTPTATSGRPTPSPLTSTLPQFAPKPNPDLNHNPGRGEAWPPGP
GISMEYECGKEPGPWFALTDVDVCNPFRVQAASEMLLIAVQEHGDKLKIVSGMAQAICLH

LCSVRIPQAKEKTLHAITLLARSHTRELVTTFLDISIPLDSHTFQLWRALGAEQPTSHLV
LTMLLAWLQERPLPTSASDSSPCPKEKTYLRSLAAMNMLHELQFARECCQACQRGLPLPC
SSTNKAFSLETFPAKPGPSHQPQEASASPEPPLLSCSTSLEALKSLLSTTGHWRDFAHLE
LQGSWELFTTIHTYPKGVGLLARTVGPGQNHCRQIRAVLRQLLPSLQSPEERERKVAILI
LTEFLYSPALLEVLPKQAALTVLTQGLRDPSPEVRVLSLQGLSNILFHPDKGSLLRGQLR
PFLDGFFQSSEQVIVCIMDTVSDMLHRLGAQGAGSQSLSVAISARSFFNDERDRIRAAAM
ALFGDLVAAMADRELSGLRTQVHQSMVPLLLHLKDQCPAVTTQAKFTFYRCAVLLRWRLR
HTLFCTLAWERGLSARHFLWTCLVRGLGSVGRNRAKEWGPGLRLPIHSRHTICCHPQAVF
QMLNAGDINLLLCTFEHLKSDPEPSIREFATSQQSFLQKVSARPKQ

>Canis familiaris_MROH5(ENSCAFP_1794)
MSQNPRAAQRQAGTRGNPEGDAPCPASHRISSPISRSMKDLSCMKVPQDPAHRDKQAHPT
TILLCKSRSSQAALMPKEHKMFMEEAYNAAICFKMLRDLNGSDPLHLKYIIKKIKGMAYR
APNLVLETIHDYLVDNPEISSRHKFRLFHVLEAVIEASNSLEEAWQKSFMQLALENMTKS
TELEEVYQDAASDVLLAICRHSWRMVAQHLETEVLTGVFPHRSLLYVMGILTSNEAIFNE
GEKACWEEQLAQLAVKSVQFLNTDMWSKELLLALTKPDRTHQEQPPEKAFLFIYYGLILQ
AEDNSTTVRTHLRTLLDTSHQWPKQREGIALTIGLAAARHLDHVWAVLEQFGRSTPIKWS
LHSFCLKNAEDLQWKWASSTILLSYGQMAVRAKAHILPWVDNILSRMVFYFHYSSWDETL
KQSFLSAVLMLVGAVSRNEGAHSYEFSQVSELLDCLMVLMEKEPRDTLCTPTRQQTIHII
SSLCKLRPPLDLEKKSQLLSTCLHSVLALPLLDVLEKHTCLFLEPPNIQAGPLAPYRRGA
DAGAWRPPDLPHVPTQTLYNKTSSALDQLLQSFIMQSPTTDELHLLLSHLYPWLASEKAQ
ERQRAVDRSTALLRFLSHSHSLDPKEDFRRIGQLVGMLGILCQDPDRAIQQSSVEGLGHL
YQLLISQTARAAPMVNAQAPKQLSPASVDGAPVWSSGDQKTAPPTPPEMTFPKDHIFQLD
SSQVIKEVMKHLTKAELTDLIWTAIEGLGSASPFRVQAAADMLLTTIREHGAKLETVANL
GRAIHLQLCSVCIPQAKEKALHAITLLAQNHTPELVATFLDFSVPLDSHAFCLWRALGAE
QPVSRQVLAVLLAWLQERALPPGPRDGSPQPKDKPCPRSLAAMNMLHELQFAREFKKAVQ
EAFPQLLLALLAQLHYTLELHLPQEPHLPQERQGWSVTPPSHCSTSLEALKSLLSTTGHW
QDFAHLELQGGWKLFSTIDTYPQGVSLLARAMVQNHCKQIKAVTSQLLPNLQSKEARERK
VAILILTEFLYSPTLLEVLPRQAALIVLARSLRDPSPEVRVLSLQGLGNILFHPEKESLL
RRQLPPFLDGFFQNNEQVVVQVMGTVSDVLHRLGAHSAAAQSLGVAINARSFFDDERDGI
RAAAMALFGDLVAAMAGKELSSLRTQVHQSMVPLFLHLKDHCPGVVTQAKFTLYRCAVLL
RWRLRHTLFCTLAWERGLSARHFLWTCLMTHSQEEFGIHLAQALSYLHSRHRHIKTWAAL
FIGYTICYHPQAVSQMVSSVDTNLLFSTFEDLKKDPEPSIRVFATRQLYFLQKVAARP

>Felis catus_MROH5(ENSFCAP_24435)
MDEQNSEKSHSHASADRTSSAVSQNPRTSSHVSKSMKELSYLKVPQDPSRKDRQGHPTTI
ILCKSQSSQVAVEPKEHSMFMEETYSAAICFKMLQDLNGSDTRGLKYIIKKIKNMAHEAP
NLVLETIHDYFVNNPEMSCRHRFWLFQVLEAAIEASDSLEEAWEKTFMKLALENMTKSTE
LEEVYQDAASNVLLAICRHSWQVVAQHLETEVLTGVFPHRSLLYVMGILTSNESIFQKGE
KACWEKQLAQMAGKSVQFLHMEKWSKELLSALTKPDQTHQEQPPEKAFLFVYYGLILQNE
DNATTVRTHLRTLLDTSHQWPKQREGIALTVGLAATRHLDHVWAVLEQFGRSTPVKWSLH
SFCLKKSEDPKWRWASSTMLLAYGQMAVKAKAHILPWVDNILSRMVFYFHYSSWDETLKQ
SFLTAIFMLVGAISRNEGAHSYEFSQISELLECLMVLMEKEPQDTLCTSTRQQTIHIISS
LCKLRPPLDLKRKSRLLSVCLRSVFALPLMDVLKKHTCLFLEPPNIQLAPSMSTPGEPSS
KHWGSRDLLHPPAQTLYNKTLEALDQMLQSLIMQNPTADELHFLLSHMYTWLASEKTHER
QRAVHSCVALLGFLSHDLYLDTKEDFRRTGQLVGVLGILCQDPDKATRHSSLEGVGHLYQ
LLMRQRARELPTVEAQAPKQLSQASEDGAPIWSIGDQQTAPPTPREMAFPKDHIFQLGSS
QVIKEVMKHLTVAELTDLIWTAVDGLGSASSFRVQAAADMLLLAIQEHGAELETVAKLGR
AIHLQLCSVHIPQAKEVALHAITLLARNHTSELVATFLDPSIPLDSHTFQLWRALGAERP
TSRLVVATLLAWLQERPLPPGARDGSPPPEDKTYLPSLTAMNLLHELQFAREFEKAVQEA
YPELLLALLTQTHYISELNLPEEPQPGQEAQPLATPSPQRTSLEALKGLLSTTGHWQDFA
HLELRGAWELFATTRTYPQGVGLLARAMVQNRCGQIKAVTSRLLSSLRSEEKRERKTAVL
ILVEFLYSPMLLEVLPRQAVLTVLARSLRDPSPEVRALSLQGLGNILFHPEKGSLLQRQM
PAFLNGFFQNSEQVVVRVMGTVSDVLHRLGANGAAAQSLRVAINARSFFDDERDGIRAAA
MALFGDLVAAAAGRELIGLRAQVHQSLVPLLLHLKDRCPAVVTVSARCRAFPLAGLRVCR
WRQGAALLSGSSLALGQQVWPPALVSVTEEFSIHLAQALSYLHSRHHHIKTWAALFIGYN
ICYHPQAVSQVVNSMDTNLLLCTFEDLKKDPEPGIREFASRQLFFLQEMAARPQ

>Loxodonta africana_MROH5(ENSLAFP_25258)
QAFLFYYYGLILQATEDSATVRAHLQSLLETSHQWPKQREGMALTMGLAAVRHLEDVWAV
LDQFGRSAPIKCSPHIFSPKDDTLKQSFLKGVLLLVRAISRNEGAHSYEFSQAPELLDCL

MILLEKESPDTLCTSIRQQAIHIISSLCKLRPPPDLERKSRLLSVCFRSVFALPLLEVLE
KHTCLFLEPPNIQALYDRTAEALDQLLQTFIRQTPTAEELHFLLSHMYFWLASEKAHERQ
RAVHSCTALLKFLSQNPHLDPKEEFRWTGKLVGMLGILCQDPDRATQLSSLEGMGHLYQL
LMRQKDATASRPQIYKQLQDFSNVPSRKVPLKCYHLKEVMKRLTMQELTDLIWTAIDGLG
SHSPFRVQAAAEMLLAAIQDRGAKLDTVARLGRAIHLQLCSVRIPQAKEHALQAITLLAR
SHTSQLVAAFLDFSIPLDRCPAPDWIKLSAGARVPVEIVELVYVLKHKQFAPPRDILLST
GDSRLLARSEAMNTVHELQFTREFKVAVQEAYPELLLALLAQVHYILELDLPREPQPHSE
IQEAAVTSPQSTSLEALKSLLSTTGHWHDFAHLELQGSWELFTTLPTYPQGVSLLARAMV
QNHCQQIKAVLGQLLPSLQSREERERKVAMLILTEFLYSPALLEVLPKQAALAVLARGLR
DTSPKVRVWSLQGLGNVLFQSGKVSLLRGQLPPFLDGFFQDSEPVVVRVMGTVSDVLRRL
GALGAGAQSLGVAINARSFFDDERDGIRAAAMALFGDLVAAMEGKELGRLRAQVYQSMVP
LLLHLKDHCPAVVTQAKFTFYRCTVLLHWRLRHTLFCTLAWEQGLSARHFLWTCLMTHSQ
EEFHVHLTQALSYLHSCHHHIKTWAALFIG

>Ornithorhynchus anatinus_MROH5(ENSOANP_10714)
ELKDAYQNAASNILVALYAHSKQLVTHKLEEEFLTGTFPHRSLLYTMGTISSSEHLQDTP
EKKEYWEEQLTRVGTKSTGFLMVDDWAQQLMWAITLSDRDCPEPTPEKAFLFTYYGMTLR
ASNNSALVRKHLRSLLGVSHQSPAHREGVALTLGLVANRHLTDAWAVLDEFGRMGPQRKC
WSHLSHQVPDDSQDVQLKWMGSTILLSYGQIAAKAKEMILPWVDNIASRMVNYFHCSIWD
DSLKQSFLVATLMLVRAISRTEGAQSYAFHQAQELTRCLVTLVEKEPLDSLCTSARQQAM
LIIADLSKLRPALEPKKKSHLLFTCFRSVFTLPPMEMLEKHTCLLANPPDVQVNHPATPS
QPTVLSRALAPSGVFSEHLWALFEETREALDKILQSFLSENPCPEELHCLVGHMYSWLAS
ERALERQRAVKSCVSLLRFTYENLCFDSSSEFRRIGHLVGMLGLLCRDPDRVTRHLDLEG
IGYLWLLLLRHKGEKELQAVTSGPEHRHNCPCHGGPCHSEKKKKTSPSAPVSLLGHLLHI
CQTTTLSTMMKVMGLIQLRDLIWTVLDGLRGSVHFRVEAAADMLLALLEDFGAKLEKVAD
IGRTLYLQLASIHSVAIKTSVLKALTLLARDHTWELVSAFLEFSLPPDSQAFDLWRAVGT
EQHVSPKVLKVLLAKLQERPLPEESKGAIEEKTHFKSFAAMSTLHEVLFAHEFKAAVQEA
YPELFLALVTQLHYVFELGPPGQASLPGPQRSETNFSSPGSSTALEAVKTLLSTSGHWKD
LAHVELQGGWDLFTSLNTYQKGLTLLASRAMVQNRCPQLRAVLKLTVPGLESKHERERKV
AILIFTEFLHSPTILQDLPKRTILSLLHKALADPSPTVRVISLQGLGNVLFHPGKGALLQ
AQLGGFLNGLYQDKEQVVTGVMDTMSNVLYRLAQQGAGPQSLDIALTLRPFFDDERDQVR
AAAISLFGDLVTVMEGKDRSTLKTQVCRSLVSLLLHLKDDSPAVVTKSKFAFFRCAVFLK
FRLCHTLFCTLAWEQGLSARHFIWTCLMSNGRDEFHIHLSQALGYLHSPLQHLRMAAATF
IG

>Homo sapiens_MROH6(ENSP_479227)
MAGGVWGRSRAREAPVGALTLTALTEGIRARQGQPQGPPSAGPQPKSWEVKPEAEPQTQA
LTAPSEAEPGRGATVPEAGSEPCSLNSALEPAPEGPHQVPQSSWEEGVLADLALYTAACL
EEAGFAGTQATVLTLSSALEARGERLEDQVHALVRGLLAQVPSLAEGRPWRAALRVLSAL
ALEHARDVVCALLPRSLPADRVAAELWRSLSRNQRVNGQVLVQLLWALKGASGPEPQALA
ATRALGEMLAVSGCVGATRGFYPHLLLALVTQLHKLARSPCSPDMPKIWVLSHRGPPHSH
ASCAVEALKALLTGDGGRMVVTCMEQAGGWRRLVGAHTHLEGVLLLASAMVAHADHHLRG
LFADLLPRLRSADDPQRLTAMAFFTGLLQSRPTARLLREEVILERLLTWQGDPEPTVRWL
GLLGLGHLALNRRKVRHVSTLLPALLGALGEGDARLVGAALGALRRLLLRPRAPVRLLSA
ELGPRLPPLLDDTRDSIRASAVGLLGTLVRRGRGGLRLGLRGPLRKLVLQSLVPLLLRLH
DPSRDAAESSEWTLARCDHAFCWGLLEELVTVAHYDSPEALSHLCCRLVQRYPGHVPNFL
SQTQGYLRSPQDPLRRAAAVLIGFLVHHASPGCVNQDLLDSLFQDLGRLQSDPKPAVAAA
AHVSAQQVAMLARARGCPRGPRLLRIAPRPARPPPVFADSPFQRRSVAGRWGCSGPRRA

>Macaca mulatta_MROH6(ENSMMUP_2188)
PVLSTARLSLCCPVKATRALGEMLAVSGCVGATRGFYPHLLLALVTQLHKLACSPRSPDM
PKIWVLSHRGPPHSHASCAVEALKALLTGDGGRMVVTCMEQAGGWRRLVGAHTHLEGVLL
LASAMVAHADHHLRGLFADLLPRLRSADDLQRLTAMAFFTGLLQSRPTARLLREEVILER
LLTWQGDPEPTVRWLGLLGLGHLALNRRKVRHVSTLLPALLGALGEGDSRLVGAALGALR
RLLLRPRAPVRLLSVELGPRLPPLLDDIRASAVGLLGSLVRRGRGGLRLGLRGPLRKLVL
QSLVPLLLRLHDPSRDAAESSEWTLARCDQAFCWGLLEEMVTVAHYDSPEALSHLCRRLV
QRYPGHVPNFLSQTQGYLRSPQDPLRRAAAVLIG

>Callithrix jacchus_MROH6(ENSCJAP_39882)
MARGVWGWVPEMPMGALTLTAVAEGIRARQGRSREPPSTGPQPEPWELKPEAEPQAQAIA
THSETESGCGATIPEAGSEPRSPERPHQVPQSSWEEGVLADLALYTAACLEEAGFAGTQA

TALTLSSALEARGERLEDQVHALVRGLLAQVPSLAEGRPRRAALRVLSALALEHARDVVC
ALLPRSLPPDRAAAELWRLSCNQRVNGQVLVQLLWALKGASGPEPQTLAATRALGEMLAV
SGCVGATRGFYSHLLLALVTQLHKLARSPRSPDMPKIWVLSHRGPPHSHASCAVEALKAL
LTGDGGRMVVTCMEQAGGWRRLVGAHTHLEGVLLLASAMVAHADHHLRGLFADLLPRLRS
ADDPQRLTAMAFFTGLLQSRPTARLLREEVILERLLTWQGDPEPTVRWLGLLGLGHLALN
RRKVRHVSALLPALLGALGEGDARLVGAALGALKRLLLRPRAPVRLLSSELGPRLRPLLD
HTRDSVRASAVGLLGTLVRRGRGMLRLGLRGPLRKLVLQSLVPLLLRLHDPSPDAAESSE
WTLARCDQAFCWGMLEEMVTVARYDSPEALSHVCRRLVQRYPGHVPNFLSQTQGYLRSPH
DPLRRAAAVLIGFLVHHASPGCVNQDLLDSLFHDLGRLQNDPEPAVAAAAHVSAQQVALL
ARSRGRPRPVFANSPFQRRSLAGRWGCSGPCRA

>Mus musculus_MROH6(ENSMUSP_139029)
MAGGVWGRGRDGRDGPVGSLTLTALAEGIRASQGQPVGPSSTGPQSDPQELGPEPEPYIQ
TLTPAFEAQPGNEVVAPTAGSEIYSPHNAQEPAPERPYQASQYSWEEGTLADLALYTAAC
LEEAGYAGTQATALTLSSALEARGQRLEDQVHGLVRGLLAQVPSLAEGRPRRAALRVLSA
LALEHAQDVVCALLPSSLPPDRAASELWRSLSRNQRVNGQVLVQLLWALKGVAAPESEAL
AATRALGEMLAVSGCVGATRGFYPHLLLALVTQLHELAQGAHSPDSPKVWEPSHRGPPHS
HASCTVEALKALLTGDGGRMVVTCMEQAGGWRRLVGAHTHLEGVLLLASAMVAHADHHLR
GLFADLLPRLRSANNPQRLTAMAFFTGLLQSQSTARLLREEVILERLRTWQGDPEPTVRW
LGLLALGNLAMNRGKIRHVNMLLPVLLRALGEGDVRLVGAALGALRRLLLQPGAPVRLLS
SELRPRLPPLLDDARDSVRASAVGLLGTLVRRGQRGLRLGLRGPLRKLVLQSLVPLLLRL
HDSSQDAAESSEWTLARCDQALRWGLLEEMVTVAHYDSPEALSRVCHRLVQRYPSHVQYF
LSQTQGYLRSPQDTLRRAATVLIGFLVYHTRPKGISQDLLDSLFQDLGQLQGDPESAVSS
AAHVSAQQVALLARAQVQTRSPNLFRFIRRHHSYPPRPHPLYDDSPFLRRSLAGRWSCLE
PH

>Rattus norvegicus_MROH6(ENSRNOP_42442)
MAGGVWGRGRDGPVGSLTLTALAEGIRASQGQPVGPSSTGPQAEPEELGPEAEPYIQTLT
PASEAQPGNEVVAPTASSEIGSPPNVQEPAPERPYQVSQYSWEEGTLADLALYTAACLEE
AGYAGTQATALTLSSALEARGQRLEDQVHDLVRGLLAQVPSLAEGRPRRAALRVLSALAL
EHAQDVVCALLPSSLPPDRAASELWRSLSRNQRVNGQVLVQLLWALKGAAGPESEALAAT
RALGEMLAVSGCVGATRGFYPHLLLALVTQLHDLAQGAHSPDSPKVWEPSHRGPPHSHAS
CTVEALKALLTGDGGRMVVTCMEQAGGWRRLVGAHTHLEGVLLLASAMVAHADHHLRGLF
ADLLPRLRSANNSQRLTAMAFFTGLLQSQPTAQLLREEVILERLRTWQGDPEPTVRWLGL
LGLGHLAMNRGKIRHVNTLLPVLLRALGEGDVRLVGAALGALRRILLQPGAPVRLLSSEL
RPRLPPLLDDARDSVRASAVGLLGTLVRRGQRGLRLGLRGPLRKLVLQSLVPLLLRLHDS
SQDAAESSEWTLVRCDRALRWGLLEEMVTVAHYDSPEALSRVCHRLVHRYPSHVQHFLSQ
TQGYLRSPQDTLRRAATVLIGFLAYHTSPKGVSQDLLDSLFQDLGQLQGDPESAVSSAAH
VSAQQVALLARAQVQTRSSNLFRFIRHRHRSYPTRPHPLYDDSPFLRRSLAGRWSCLEPR

>Bos taurus MROH6_(ENSBTAP_45971)
MAGGVWGPARGAPVGALTLTALAEGIRASRGQPMGPPAPGPQPQPELEPAVEEPGRAATT
PTAGGEPPSPPPPQEPAPEGPQQTPRSSWEEGALADLAVYTAACLEEAGFVGTQATALTL
SSALEAQGHRLEDQVHGLVRGLLAQVPHLAEGRPRRAALRVLSSLALEHSRDVVFALLRC
SPAPDRVAAELWRSLSRNQRVNGQVLVQLLWALKGSAGSQLEALAATRALGEMLSVSGCV
GATRGFYPNLLLVLVTQLHELARDAHSPDTPKVWVPSHRGPPHSHASCTVEALKALLTGD
GGRMVVTCMEQAGGWRRLVGAGTHLEGVLLLASALVAHADHHLRGLFADLLPRLRSTDDA
QRLTAMAFFTGLLQSRPTARLLREEVILERLRAWQGDPEPTVRWLGLLGLGHLALNHGKV
RHATTLLPALLGALGEGDPRLVDAALGSLRRVLLQPRAPVRLLSAELRPRLPPLLDDTRD
SVRASAVGLLGTLVRRGRGGLRVGLRRPLRKLVLQSLVPLLLRLHDPSPDTAESSEWTLA
RCDQALHWGLLEEMVTVAHYDSPEALSRICHRMVQWYPSHVPSFLSQTQGYLRSPQGPLR
RAAALLTGFLVHHSSPSHVNQDLLDSLFQDLGRLRSDPEPAVAAAARVSGQQVALLARAQ
PQPCGRSLWGLRHLPCPGRPRPRPARPPPVYADSPFQHRSHPGRWGCSGPG

>Pteropus vampyrus_MROH6(ENSPVAP_5655)
MAGGVWGRARGAPVGALTLTALAEGIRASQGQPVRPPPKGPQPEPEPTEAEPGNITASPE
PCSPPREQEPASERPHQVPQSSWEEGPLADLALYTAACVEEAGFMGTQATALTLSSALEA
RGERLEDQVRALVLGLLAQVPSLAEGRPRRAALRVLSALALEHAQDVVRALLPCSLPPDR
AAAELWSSLGRNQRVNGQVLLQLLWVLKGTAGLELEALAATCALGEMLAVSGCVGATRGF
YPHLLLTLVTQLHRLARGAHSPDTPKIWAPSQRGPPHSHASCTVDALKALLTGDGARMVV
TCMEQAGGWRRLAAAHTHLEGVLLLASAMVAHADHHLRGLFAELLSRLCSPDEAQRLTAM

AFFTGLLQSGPTARLLREEGILERLRAWQHDPEPTVRWLGLLGLDHLALNNGKARHVSAL
LPALLGALGEADARLVGAALGALRRVLLQPRAPVRLLSTELGPRLAPLLDDARDSVRASA
IGLPGTLVRQGRGGLRVGL

>Monodelphis domestica_MROH6(ENSMODP_14116)
MARGRRGWGGGEPRGQAGPLGALTLAALAEGIQISRGATGPSQGQPPQEPKEPENPTEAV
TTPRTEGLEPDPATGDLKNEPCSSKASSSESEPQPWEPEQHPQCLLAPGSLADFALQTVV
CLEDAGISGTHALAFSLSSALEAQGGQLPEKVREVVQGLHGQMAQLVEGRARRVALRALC
SLAAEHTQEVVQTLLPHSLPCDRAAAELWRGLSRNQRVNGLVLVHLLQEVKGPPQSQGLS
PSSSPGPSRQDAKPHTQALAATRALGEMLSVSGCVAATRGFYPQVLIALVTQLHQLAWGV
HSPQAQVKVRGPPSNHASCAVEALKALLSGDGGRMVVTCMEQSGGWRRLMGSGTHLEGVL
LLASALVAHADHHLRGLFADLLPRLQSPDKAPRLTAMAFFTGLLQSQPTARLLRDEAIVQ
RLTAWQGDPEPTVRWLGLLGLGHLALNHRKARHVGTLLRALLDALGEADTRLVGAALGAL
RRLLLRPRARARVSAYCASLGTRLRPLLDHCQDSVRASAIGLLGTLVGRCSLRRARMVRE
LVLGSLVTLLLRLQDPSLDAAQSAEWTLARCDRFLHWKLLEEISTMAHYDSPEALSRTCG
RLVQWYPRRASDFLSQAQGYLRSPEVPIRRVAAMFIGFLIHHMDPDCVNPGLVDSLLHDL
GEMQWDAETCIRDVSHITMHQVRLVCQDQDSPGHGNFSPWQLFCCWGHHQERERPVYEDS
PFKPRSRAGLWGCGRPS

>Anolis carolinensis_MROH6(ENSACAP_3363)
MNSFYVSVIIFLVIQLHYLVDCSDIDYGQEEILQTSSYISCTMETLKALVKRETTSRACF
ASLTGSWDLLSSPENYLEGVLLLARALVKHHRGLDYAVFTKVIPLLHHGDDKQKLTAMAF
FTALLSSESTYTVLQKHYSLGLLKNWLADSSATYRWLSLHGMGNVAHHLQSKKEFTTLIL
SILPSFDDSDEKVALTAMEVITKVMVQQKINVNLYVKITKQLQPFLADGRNKVSCAANKL
FQDILKNIDAKDKYAMQDQVLTAIVTLMVNLQELNLDVAKESGSRWLLVVTTSFSCWLQE
SFNGWTTNDNEDSWDMVCKHLVKEHPGKLRRLLNQAQHYRQSPQKSSRSAATIFIG

>Homo sapiens_MROH7(ENSP_396622)
MALSPGANLVFHEDPKMTPSPPSCGAPGLGSGTIPQPHPDMAQVPMLNLLPSPGLALVPD
LNDSLSPVSGEASGLVSENTPRPDDSRAIAPASLQITSSCSGEALDLDSKDVSRPDSQGR
LCPASNPILSPSSTEAPRLSSGNHPQSNSEDAFKCLSSKIFKLGQRNSNPSRHELNPFIR
HHSREGLVLGHCISRPSSKALLIPTSNSSLDLDSNPLLNMGSRNTSKLNLNVAPDSHGTL
IPDTNETITLASHNISESVSKGAFSTTWSTSSKETMNVASSGHSRSDLSVTITQASYVTL
IPGSSYGISLHSSTHEPNSTISPPSCMTLILGSNETLSLDSSLLFSDTSTLTLSSQQDDA
KDNSIHTVPLEENLESWSEMASIKVGQFPLGFPISNPAGKDAVTLQGIPEGAFDEVTSCL
VKVPEKTEGGNNMALVENVTTLQKSQDLLEAEGEKKTMIKKIMRQIQEEPLDSLSSSVRK
QAMEILTQLSHTQPTLGMRERSELVNVCVHSVFSLPSVQAMQEKDEAKAETIQALYHQTL
EALQTLLKALFIEDPTPAGLKSILEALGPWMNSGKAHERARAVNTNVSVLNHMLLTLPFF
MPLGFPALGLLLGRLILHIGDPDEEIGCEALDGIIILYTILELQKRARDKEETNKKELYE
SNKHFLGPYNPVSPCQNILRVIEEFGDFLGPQQIKDLLLAALEGLKGSSEAPGKDSREMM
QLASEVMLSSVLEWYRHRALEVIPEIMQGIYMQLSHIQEPRARQVALLPVSLLASSFMTE
VVVALLMCPLPLNSNGAEMWRQLILCKPSCDVRDLLDLLLGSLKEKPVTKEGRASIVPLA
AASGLCELLSVNSCMGRVRRIYPQLLLALLIQVHYHIGLNLPGCVAPPKDTKKGAQPSPF
VPVRWVVKVVKTLLLRMGCSYETTFLEDQGGWELMEQVESHHRGVALLARAMVQYSCQEL
CRILYLLIPLLERGDEKHRITATAFFVELLQMEQVRRIPEEYSLGRMAEGLSHHDPIMKV
LSIRGLVILARRSEKTAKVKALLPSMVKGLKNMDGMLVVEAVHNLKAVFKGRDQKLMDSA
VYVEMLQILLPHFSDAREVVRSSCINLYGKVVQKLRAPRTQAMEEQLVSTLVPLLLTMQE
GNSKVSQKCVKTLLRCSYFMAWELPKRAYSRKPWDNQQQTVAKICKCLVNTHRDSAFIFL
SQSLEYAKNSRASLRKCSVMFIGSLVPCMESIMTEDRLNEVKAALDNLRHDPEASVCIYA
AQVQDHILASCWQNSWLPHGNSWVCYSATTHRWSPSCENLPTSHQRRSWIMQALGSWKMS
LKK

>Pan troglodytes_MROH7(ENSPTRP_1342)
MALSPGANLVFHEDPKMTPSLPSCGAPGLGSGTIPWPHPDTAHVPMLNLLPSPGLALIPD
LNDSLSPVSGEASGLVSENTPRPDDSRAIAPASLQITSSCSGEALDLDSKDVSRPDSQGR
LCPASNPILSPSSTEAPRLSSGNHPQSNSEDAFKCLSSKIFELGQRNSNPSRHELNPFIR
HHSREGLVLGHCLSKPSSKALLIPTSNSSLDLDSNPLLNMGSRNTSKLNLNVAPDSHGTL
IPDTNETITLASHNISESVSKGAFSTTWSTSSKETMNVASNGHPRSDLNVTITQASYVTL
IPGSSDGISLHSSTHGPNSTISPPSCMTLILGSNETLSLDSSLLFSDTSTLTLSSQQDDA
KDNSIHTVPLEENLESWSEMASIKVGQFPLGFPTSNPAGKDAVTLQGIPEGAFDEVTSCL
VKVPEKTEGGNNMALVENVTTLQKSQDLLEAEGEKKTMIKKIMRQIQEEPLDSLSSSVRK

QAMEILTQLSHTQPTLGMRERSELVNVCVHSVFSLPSVQAMQEKDEAKAETIQTLYHQTL
EALQTLLKALFIEDPTPAGLKSILEALGPWMNSGKAHERARAVNTNVSVLNHMLLTLPFF
MPLGFPALGLLLGRLILRIGDPDEEIGCEALDGIIILYTTLELQKRARDKEETNKKELYE
SNKHFLGPYNPVSPCQNILRVIEEFGDFLGPQQIKDLLLAALEGLKGSSEAPGKDSREMM
QLASEVMLSSVLEWYRHRALEVIPEIMQGIYMQLSHIQEPRARQVALLPVSLLASSFMTE
VVVALLMCPLPLNSNGAEMWRQLILCKPSCDVRDLLDLLLGSLKEKPITKEGRASIVPLA
AASGLCELLSINSCMGRVRRIYPQLLLALLIQVHYHIGLNLPGCVAPPKDTKKGAQPSPF
VPVRWVVKVVKTLLLRMGCSYETTFLEDQGGWELMEQVESHHRGVALLASCQELCRILYL
LLPLLERGDEKHRITATAFFVELLQMEQVRRIPEEYSLGRMAEGLSHHDPIMKVLSIRGL
VILARRSEKTAKVKALLPSMVKGLKNMDGMLVVEAVHNLKAVFKGRDQKLMDSAVYVEML
QILLPHFSDAREDVRSSCINLYGKVVQKLRAPRTEAMEEQLVSTLVPLLLTMQEGNSKVS
QKCVKTLLRCSYFMAWELPKRAYSRKPWDNQQQTVAKICKCLVNTHRDSAFIFLSQSLEY
AKNSRASLRKCSVMFIGSLVPCMESIMTEDRLNEVKAALDNLRHDPEASVCIYAAQVQDH
ILASCWQNSWLPHGNSWVCYSATTHRWSPSCENLPTSHQRRSWIMQALGSWKMSLKK

>Pongo abelii_MROH7(ENSPPYP_1530)
MALSPGANLVFHEDPKMTPSLPSCGALGIGSGTIPQPHPDMAHVPMLNLLPSPGLALVPD
LNASPSPVSGEASGLVSENTPRPDDSRAIAPASLQITSSCSGEALDLDSKDVSRLDSQGH
LCPASNPILSPSSTEAPGLSSGNHPQSNSEDAFKCLSSKIFELGQRNSNPSRHELNPFIR
HHSREGLVLGHCISRPSSKALLIPASNSSLDLDSNPLLNMGSRNTSKLNLNGAPDSRGTL
IPDTNKAFTLASHNISGSVSKGAFSTTWSTSSKETMNVASNDHPRSDLNVTITQASYMTL
IPGSSDGISLHSSTHGPNSTISPPSCMTLILGSNETLSLDSSLLFSDTSTLTLSSQQDDA
KDNSIHTVALEENLESWSEMASIKMGQFPLGFLTSNSAGKDAVTLQGIPEGAFDEMTSCL
VKVPEKTEGGNNMALVENVTTLQKSQDLLEAEGEKKTMIKKIMRQIQEEPLDSLSSSVRK
QAMEMLTQLSHTQPTLGMRERSELVNMCVQSVFSLPSVQAMQEKDEAKAETIQTLYHQTL
EALQTLLKALFIEDPTLAGLKSILEALGPWMNSGKAHERARAVNSNVSVLNHMLLTLPFF
MPLGFPALGLLLGRLILRIGDPDEEIGCEALDGIIILYTILELQKRARDKEETNKKELYE
SNKHFLGPYNPVSPCQNILRVIAEFGDFLGPQQIKYLLLAALEGLKGSSETPGKDSREMM
QLASEVMLSSVLEWYRHRALEVIPEIMQGIYTQLSHIQEPRARQVALLPVSLLASSFMTE
VVVALLMCPLPLNSNGAEMWRQLILCKPSCDVRDLLDLLLGSLKEKPVTREGRASIMPLA
AASGLCELLSVNSCMGRVRRIYPQLLLALLIQVHYHIGLNLPGCVAPPKDTKKGAQPSPF
VPVRWMVKVVKTLLLRMGCSYETTFLEDQGGWELMEQVESHHRGVALLARAMVQYSCQEL
CRILYLLIPLLERGDEKHRITATAFFVEMEQVRRIPEEYSLGRMAEGLSHHDPIMKVLSI
RGLVILARRSEKTAKVKALLPSMVKALKNMDGMLVVEAVHNLKAVFKGRDRKLMDSAVYV
EMLQILLPHFSDAREDVRSSCINLYGKVVQKLRAPRTQAMEEQLVSTLVPLLLTMQEGNA
KVSQKCVKTLLRCSYFMAWELPKRAYSRKPWDNQQQTVAKICKCLVNTHQDSAFIFLSQS
LEYAKNSRASLRKCSVMFIGSLVPCMESIMTEDRLNEVKAALDNLRHDPEASVCIYAAQV
QDHILANCWQNSWLPHGDSWVCYSATTHRWSPSCENLPTSHQRRSWIMQALGSWKMSLKQ

>Mus musculus_MROH7(ENSMUSP_102382)
MALSRGTSLILHEDPEKIPSPNSCEVPGIMSNTTPRPTPDLALAPPPEHALALTPALHPA
LSPDPEGVSGPVSNDIPSHNASGATTPSSTQINTVDTADQGLNHTSGPDAAGTLCPDSQP
ARIPSSTQANVLSPENSSRPCSEDVSKSFSSKVFGLGQSNSNPSRPEPNLYIKALSREAL
VRSHNISRQGSQVPLLLPSNTSLDRLHSGNISKVNLGIAPNSNEAITLTSHTIFASISKE
ALSAPWNTGSKGSINGTSTIQPRSGLNVTVTHASHVSMIPGSSEGLSLQSSARVPNSTLS
PSSCMTLIMDSESPSMDSSFLVTDTSTLTLSSHRDYSEDNSIRTMPLEENLGKWDSLQGV
TALQSPPEGTSEDVKVNEAEKRNHDNKAALVANIITYQKNQEMVEMKEEKEATVKMMMRQ
IQEEPLDSLLSPARRQAMEILAQLSHTKPILSVRERVELVNTCVRSVFSLPSVQAMQEKD
ESKAEVIQILYYQTLDSLQKLLNALFIEDPTPTGLKSILEPLGPWMNSGKAHERARAVNS
NVSVLNHTLVTLPFLISSGFPTLGLLLGRLLLRIGDPDEEIGREALDGITILYTILDLQK
RTKNKEDTNKKELYENNKRFLGPYNPVSPCQNILRVIAEFGDFLGPQQVRDLLLAALEGL
KGISETQGKDSGEMMQLASEVMLSSVLEWYRHRALEVIPEIMQGIYMQLTHIQEPRAREV
ALLPISFLASSFMTEVVVALLMCPLPLDSNGAEMWRQLILRKPSCDVRDLLDLLLTSLKE
KPVTKKGRASIVPLAAASGLCELLSVNSCVGRVRRIYPQLLLALLIQVHYHIGLNLPSRM
APRKDSKDDTQPPLFIPVRWMVKVVKTLLLKMGCSYESAFLEEQGGWELMGQAESHYRGV
SLLARAMVHYSCQELCRILYLLIPLLERGDERHKITATAFFVELFRMEQVRRIPEEYSLG
RMVEGLSHRDPIMNVLSIRGLVILACKSEKMAKVQSLLPSMVKSLKNMDGMLVVEAVHDL
KRIFKGQGKKLTDSAVYVEMLQILLPHFTDAREMVRASCINVYGKVVKKLQTPRTQAMEE
QLTSTLMPLLFIIQEGNAKVSQKCVKTLVCCSSFMNWELPKKAYSQKPWDNQQLTVTKIC
KYLVSSHRDNVFTFLNQSLEYAKNSRASLRKSSVIFIGSLVPCMENMMTEERLNEVKATL
EILRHDPEASVCICAAQAQDQIMATCWRNSWPLLYGDSWVCDPSSMHRWSPSCENLPTSH

QRRSWIMQALASWKMSLKQ

>Rattus norvegicus_MROH7(ENSRNOP_72770)
MALSRGTSLILHEDPEKTPSPNSCEVPGIMSNTTPRPTPDLTLAPLPAHGVALAPALHPA
LSPDPEGVSGPVPNDISNPNASGAATPSSIQINTADTDDQSLDHTPKPNLAGTLCPDSQP
VPSLSSTQATVLSPENSIKPLSEDIPKSFSSKVFGLGQSNSNPSRPEPNLYIRALSREAL
VRSHTISRQSSQTPLLLPSNTSLDRLHSGNISKVNLGRAQNSNEAITLTSHTIFASVSKE
ALSASWNTGSKGSVNVTSNSQPRSGLNVTVTHASHVSLIPGSSEGLSLHSSAQVPNSTLS
PSSCMTLIMGSESLSMDSSFVVTDTSTLTLSSHRDYSEDNSIRTMPLEENLGKWDSLQGV
TAFRSPPEGTSEDAKANESEKRDHDNDTALVENIISYQKNQDEVEVTGEKETTVKMMMRK
IQEEPLDSLLSPARRQAMEILAQLSHTKPILSVRERVELVNTCVRSVFSLPSVQAMQEKD
ESKAEVIQTLYHQTLDSLQKLLNALFIEDPTPAGLKSILEPLGPWMNSGKVHERARAVNS
NVSVLNYTLVTLPFFVSSGFPTLGLLLGRLLLRIGDPDEEIGREALDGITILYTILDLQK
RTKNKEEINKKELYETNKRFLGPYNPASPCQNILRVIAEFGDFLGPQQVRDLLLAALEGL
KDISETQGKDSIEMMQLASEVMLSSVLEWYRHRALEVIPEIMQGIYMQLSHIQEPRAREV
ALLPISFLASSFMTEVVVALLMCPLPLDSNGAEMWRQLILRKPSCDVRDLLDLLLTSLKE
KPVTKKGRASIVPLAAASGLCELLSVNSCVGRVRRIYPQLLLALLIQVHYHIGLNLPSRV
APRKDSKDDIQPPLFVPVRWMVKVVKTLLLKMGCSYESAFLEEQGGWELMGQAESHYRAV
SLLARAMVHYSCQELCRILYLLIPLLERGDERHKITATAFFVELFRMEQVRRIPEEYSLG
RMVEGLSHRDPIMKVLSIRGLVILARRSEKMTKVQGLLPSMVKSLKNMDGVLVMEAVHDL
KTIFKGQAKKLTDNSVYIEMLQTLLPHFIDARETVRTSCINTYGKVVKKLRMPRTQAMEE
QLTSTLMPLLFIIQEGNAKVSQHFPLEFHTAGIFFLLVCRNV

>Bos taurus_MROH7(ENSBTAP_53490)
METLTQLSHSQPVLGLQERSELVNTCVKSVFSLPTIQAMQEKDQAKADALQILYCQTLDA
LQTLLNALFVEDPYVSLLSSRLSPLGPWMNSGKAHERARAVKSNVSVLNHALLTPPFLMS
SGFPALGLLLGRLIIRIGDPDEEIGREALDGITILYTILELQKRARDKEETNKKELYESN
KRFLGPYNPVSPCQNILRVIAEFGDFLGPQQVKDLLLAALEGLKGGSEAREKDLGEMMQL
ASEVTLSSVLEWYRHRALEVIPEIMHGIYAHLSHIQEPRAREVALLPVSLLASSFMTEVV
VALLMCPLPLDSNGAEMWRQLILRKPSCDVRDLLDLLLTSLKEKPVTKKGRASIVPLAAA
SGLCELLSVNSCMGRVRRIYPQLLLALLIQVHYHIGLSLPGRVASRKDAKKDAQTPGFIP
VRWVVKVVKTLLLKMGCSYEANFLEEQGGWELMGQAENHHRGVSLLARAMVHYSCQELCR
ILYLLIPLLERGDEKHKITATAFFVELLRMEQVRRIPEEYSLGRMAEGLSHRDPIMKVLS
IRGLVILAQRTEKAAKVQALLPSMVKGLKSVDGLLVVEAVHNLKTIFKGQDRKLMDNSVY
VEILQILLPHFSDSREDVRTSCINLYGKVVQKLRSPRTPAVEEQLISTLVPLLLTMQEGN
AKVSQKCVKTLFRCSCFMAWELPKKAYSRKPWDNQQQTVAKICKYLVDTHRDSAFTFLSQ
GLEYAKSPRASLRKSSVMFIGSLVPCMESSMTEDRMNEVKAALENLRHDPEASVCICAAQ
AQDNILASCWRNSWPLPHGDSWVCDPATTHRWSPSCENLPTSHQQRSWIMQVLGSWKISL
KQ

>Myotis lucifugus_MROH7(ENSMLUP_15399)
MALSQRASLVLCEDPKKTPSFSSTGAPGLGSGTISGPNPGTDLVPALNVPPSPGLALVPD
LHPILSPVSEEAPGLVSDSTPSPDESGAVAPAALQTTPPPSGEALGLELNHISRPNSQEA
LHPASPLVPSPDSAEAQGPSSDNPSGLNSEEAFNPHSSKAFELGQNNSNPSRSESNPFLR
TFSREALVLGPCLSRPCSKALLIPASGSMDSPSNHLLSVGSRTISKLDLKGAPGPCGTLS
PSTNETIKLVSQDISGSVSKGSFGAAWKTSSKGNINVALNSNSRSDVNVTVTQASCLTVI
PGFQDDISLRSSTRRPNSVPSPTSCMTLIMGSKETLSLSSSLIFSDTSTLTLSSQQDDSE
DNTIHTVPLEESQSWSEMANSEMSRFPLVFPTCNTADKGTAAFHSVPERTFSEETSCLVN
APGEREDGEDGNKMTLVENVITLQKSQDVPEGDGEKKTMMKKMMEQIQEEPLDSLSSSVR
RGMETLTQLSHTQPALGVRERSELVNTCVRSVFSLPSVQAMQEKDEAKAESIQILYRQTL
DALQTLLNALFVEDPTPAGLKSILEPPGPWMNSGKAHERAPPCEQQCLSLFLILLRLPFV
SPMPTRMISFSLLGSVCLRGRNSVDQVSTEAKPTLLVEKTLTTCSKGARDKSETDKRELY
ESNKRFLGPYNPASPCQNILRVIAEFGDFLGPQQVKDLLLAALEGLTGGSEAEAQGRDSG
EMMQLASEVTLSSVLEWYRHRALEVIPEITQAIYVQLSHIQEPRAREVALLPISLLASSF
MTEVVVALLMCPLPLDSNGAEMWRQLILGKPSCEVRDLLDLLLTSLKEKPVTKKGRASIV
PLAAASGLCELLSVNSCLGRVRRLYPQLLLALLIQVHYHIGLNLPRRLPSQPDRKDAKKD
AQPSVFEPVRWVVKVVKTLLLKMGCSYEATFLDDQGGWELMGQEENHHRGVSLLARAMVQ
YSCQERCRILYLLIPLLERGDEKHKITATAFFVELFQMEQVRRIPEEYSLGRMAEGLNHQ
NPIMKVLSIRGLVILARKTEKTAKVQALLPSMVKGLKSVDGLLVMEAGHNLKTIFKGQDR
KLNDSSVYVELLQILLPHFSDAREDVRSFCINLYGKVVQKLRSPCTPAMEQQLVSTLVPL
LLTMQEGNTKVGQLCVKTLFRCSCFMAWELPKRAYSRKPWDSQQQAVTKICNYLVNTHRD

SALTFLSQSLEYSNNSRASLRKSSVLLIGSLVPFMDSIMTEDRLNEVKAALENLRHDPEA
SVCICAAQAQDSILTSCWRTSWPMTHADLWVCDPAATHRWGSSCENLPTSHQPRSWIMQA
LGSWTTSLRQ

>Monodelphis domestica_MROH7(ENSMODP_4784)
MALNPDKSAHHSKSSDMTPSPSMVLSPTLGISLSPSTSETLVPNLGSLPRLASTAALAPF
FGLAPTLMVSDAESASSDLALVPASITYQIPTSSKAVSMGANGGPNSGSSIILGPASSTI
LIPGLNDSSDPGPNQISKPHSNAAMAPGPSPSLLAGKEADSPNPSSTVLLQPALSSAFLV
SRSPSTHRALGLNSNSTPKSSSTGFDKAVKLSQSPSLTSGKALCLSFDTSHLMGPAGTHL
MVTDPSPNFQKNPDVESVSRPSSNLTLSPFSGLTSSPSSRKALGVGMGNVSRSSSSVALT
PTLSRPSSPASKKASNAETDNSNGTPDSAANTEALSDPNVSRPNSLMALAPIACMALVVD
HSQDKKNDSGCMLTDASCVTLSSSQDSPDNFSIVLESNSKTTELKRKLESSQSVMLQTLS
RSTSEDSRLSQAKSDENKNVPEKSQEQLEPEGETKQLLRKMMKQIQEEPIDVLSNSNRQQ
AMITLAHLSQIPPRLHAKEKSELVTVCVRSVFSLPSVQTMQEQDSPNADFIKILYSQTLD
ALQALLKSLFLEDPTPASLTSILEVSRSLRDGSGPGLGQVRGNGWTREKPLGPWITSAKA
HERARAVNNNVSLLNCTLQTQSFFTSHIEHDSNTPMEPQNKEKLSIEQNRRNYYLVPGFP
MFGLLLGRLILRICDPDEEIGSEALDGVIILYTILELQKRNLDKEETNELELYESNKRFL
GPYNPVSPCQNILRVIAEFGDFLGPQQVKDLLLAALEGLRDDQETPTKDTAEMMQLASEV
TLSSVLEWYRHRVLEVIPEIMNGIYVHLHGIHEPRAREVALLPISLLASSFMTEVVIALL
MCPLPLDSNGAEMWRQLILRKPSCDIRDLLELLLTSLKEKPVTNKGRASIVPLAAASGLC
ELLSVNSCVTRVRRIYPQLLLALLVQVHYHIGLNLPGQGSCFRNSLSATFMPVRWVVKVV
KTLLLKMGCSYEATLLESQGGWELMGQAEHHHRGVSLLARAMVHYSCQELCRMLYLLIPL
LERGDEKHKITATAFFVELLRMEQVKRIPEEYSLGRMSEGLSSEDPIMKVLCIRGLVILA
HRTEKSNKIQALLPSMMKGLQWVDGLLAVEAVSNVKTILRSQDKKSSDISIYLDILQVLQ
PHFDDNREEVRLAAITLFGKVVQLLRSPCTPLTEQQLVNSLVPLILQLQEGNPVVGKRCK
KTLSYCCYHMAWVLPKMMYSKKPWDPSQQSVAKICKYLVNTHRDSALVFLTQSLEYTKSP
RAALRRSAVTFIGSLVPCMEGMLTEELLSEVKSEQAKTYKDEGNDYFKDKDYKKAVLSYT
EGLRKKCSDPDLNAVLLTNRAAAQFHLGNLRSALNDVTAARKLKPNHLKAIIRGAACHLE
LKNFREATRWCDEGLRLDSQERKLLETRAKAERLKRVEERDIRKAKVKEKKEEAQKGALL
RAIQARNIRLAAAAPGDDDEPSDDADDPDGASPHAGLGAENAAGARMCLGDDGRLRWPTL
LLYPEHGQTDFISAFHEDSRFIDHFLVMFGELPPWDLERKYHPDNLQLYFEDDAQEELYQ
LSPESTLLEALQHPRYVVKASTPAFLVLAKQSPFCKSFLRGRKVHRLK

>Homo sapiens_MROH8(ENSP_339971)
XVAGTPTSSQPSFGPSIRERPSQSLCPCPCPAGEGDPAWPALERRLVTSRGRQPSPQSHA
QLLTRRRHSSEQVPPESEPRADFRSGKWLQEPATGDARDSRQALRARMSSKHRICSQEEV
VIPCAYDSDSESVDLELSNLEIIKKGSSSIELTDLDIPDIPGLHCEPLSHSPRHLTQQDP
LSEAIVEKLIQSIQKVFNGELKGELEKLKFLGDLSSLSQALPYDETAKSFIHSHIADIVH
TLNVLVQEERPHSLSSSMRQEVFVTIADLSYQDVHLLLGSEDRAELFSLTIKSIITLPSV
RTLTQIQEIMPNGTCNTECLYRQTFQAFSEMLQSLVVKDPHLENLDTIIKHLVPWLQSVK
DHERERATASMAQVLKCLSKHLNLKLPLRFQRLGHLVALMALLCGDPQEKVAEEAAEGIH
SLLHITLRLKYITHDKKDQQNLKRALTKCREFLELHSSAAKCFYNCPFRIAQVFEGFLDS
NELCQFIMTTFDTLKTLKHPCIQRSAGELLLTLAKNTESQFEKVPEIMGVICAQLSIISQ
PRVRQQIINTVSLFISRPKYTDIVLSFLLCHPVPYNRHLAEVWRMLSVELPSTTWILWRL
LRKLQKCHNEPAQEKMAYVAVAATDALYEVFLGNRLRAATFRLFPQLLMTLLIQIHHSIG
LTMSDVDIPSGLYTEQEVPSEVTPLCFAMQATKTLLLRTCCLQEFNIMEKNKGWALLGGK
DGHLQGLFLLANALLERNQLLAQKVMYLLVPLLNRGNDKHKLTSAGFFVELLRSPVAKRL
PSIYSVARFKDWLQDGNHLFRILGLRGLYNLVGHQEMREDIKSLLPYIVDSLRETDEKIV
LSAIQILLQLVRTMDFTTLAAMMRTLFSLFGDVRSDVHRFSVTLFGAAIKSVKNPDKKSI
ENQVLDSLVPLLLYSQDENDAVAEESRQVLTICAQFLKWKLPQEVYSKDPWHIKPTEAGT
ICRFFEKKCKGKINILEQTLMYSKNPKLPIRRSAVLFVGLLSKYMDHNELRRMGTDWIED
DLRDLLCDPEPSLCIIASQTLLLVQMARAEPKPKQRVNWLQKLMGRSSA

>Pongo abelii_MROH8(ENSPPYP_12264)
MPLSRRGRGSRLAGSRAATSSLPRATALSPKSTGRGALSISHAQLLTRRRHSSEQVPPAS
EPQADFRSGKWLQEPATGDARDSRQALRARMSSKHRICSQEEVQEEVVIPCAYDSDSQSV
DLELSNFETIKKGSSSIELTDLDIPDIPGLHCEPLSHSPRHLTQQDPLSEAIVEKLIQSI
QKVFNGELKGELEKLKFLGNLSSLSQALPYDETAKSFIHSHIADIVHTSNVLVQEERPHS
LSSPVRQEVFVTIADLSYQDVHLLFGSEDRAELFSLIIKSIITLPSVRTLTQIQEIMPNG
TCNTECLYRQTFQAFSEMLQSLVVKDPHLENLDTIIKLPLRFQRLGHLVALMALLCGDPQ
EKVAEEAAEGIHSLLHITLRLKYITHDKKNQQSLKRALTKCREFLELYSSAAKCFYSCPF

RIAQVFEGFLDSDELCQFIMTTFDTLKNLKHPCIQRSAGELLLTLAKNAESQFEKVPEIM
GVICAQLSIISQPRVRQQIINTVSLFISRPKYTDIVLSFLLCHPVPYNRHLAEVWRMLAV
ELPSTTWILWRLLRKLQKCHNEPAQEKMAYVAVAATDALYEVFLGNRLRAATFRLFPQLL
MTLLIQIHHSIGLTMSDVDIPSGLYTEQEVPSEVTPLCFAMQATKTLLLRTCCLQEFNIM
EKNKGWALLGGKDHHLQGVFLLANALLERNRLLAQKVMYLLVPLLNRGNDKHKLTSAGFF
VELLRSPMAKRLPSIYSVARFKDWLQDGNHLFRILGLRGLYNLVGHQEMREDIKSLLPNI
VDSLRETDEKIVLSAIQILLQLVRTMDFTTLAAMMRTLFSLFGDVRSDVHRFSVTLFGAA
IKSVKNPDKKSIENQVLDSLVPLLLYSQDENDAVAEESRRVLTICAQFLKWKLPQEVYSK
DPWHIKPTEAGTICRFFEKKCKGKINILEQTLMYSKNPKLPIRRSAVLFVGLLSKYVDHN
ELRRMGTDWIEDDLRDLLCDPEPSLCIIASQTLLLVQMARAKPKPKQRVNWLQKLMGRSS
A

>Macaca mulatta_MROH8(ENSMMUP_18889)
MPLSRRGRGSRLAGSRAATSSLPRATALSPKSAGRGALSISHAQLLTRRRHSSEQVPPES
EPQADFRSGKWLQEPATGDARDSRQALRARMSSKHRICSQEEVVIPYAYDNDSGSVDLEL
SNLETIKKGSSSIELTDLDIPDIPGLHCEPLSDSPRHLTRQDPLNEAIVDKLIQSIQRGF
SGELKGELEKLTFLGSLSSLSRALPYDETTESFIHSHIADIVHTLNVLVQEERPHSLSSL
VRQEVFVTIADLSYQDVHLLFGSEDRAELFSLVIKSIITLPSVRTLTQIQEIMPNGTCNT
ECLYRQTFQAFSEMLQSLVVKDPYLENLDTIIKHLDPWLQSVKDHERERATASMTQVLKC
LSRHLSLKLPLRFQRLGHLVALMALLCGDPQENVAEEAAESIHYLLHITLRLKYIIHDKK
NQQNLKRALTKCREFLELYSSAAKCFYSCPFRIAQVFEGFLDSNELCQFIMTTFDTLKNL
NHPCIQRSAGELLLTLAKNAESRFEKVPEIMGVICAQLSIISQPRVRQQIINTVSLFISR
PKYTDIVLSFLLCHPVPYNRHLAEVWRMLAVELPNTTWILWRLLRKLQKCHNEPAQEKMA
YVAVAATDALYEVFLGNRLRAATFRLFPQLLMTLLIQIHHSIGLTMSDVDIPSGLYTEQE
VSSEVTPLCFAMQATKTLLLRTCCLQEFNIMEKNKGWALLGGKDCHLQGVFLLANALLER
NRLLAQKVMYLLVPFLNRGNDKHKLTSAGFFVELLRSPVAKRLPSIYSVARFKDWLQDGN
HLFRILGLRGLYNLVGHQEMREDIKSLLPYIVDSLRETDKKIVLSAIQILLQLVRTMDFT
TLAAMMRTLFSLFGDVRPDVHRFSMTLFGAAIKSVKNPDKKSVEKQVLDSLVPLLLYSQD
ENDSVAEESRRVLTICAQFLKWKLPQEVYSKDPWHIKPTEAGTICRFFEKKCKGKINILE
QTLIYSKNPKLSVRRSAVLFVGLLSKYMDHNELRRMGTDRIEDDLRDLLCDPEPSLCIIT
SQTLLLVQMASAEPKPKQRVNWLQKLMGRSSA

>Mus musculus_MROH8(ENSMUSP_124362)
MPLPRRGGEPRLAGARVVRTSLPRATPRSPKRVGRGAPLLSHAQRLTRRRHSSEQVPPEP
EQADFRSGKWLQERAGGDARDPRHTPLARMGSRQRASGTEEVVIPCASDSDSGSVDLQLS
NLDDVQKGGASVEFTDSDIADIPTLPQESLPDNLRLLTLQEPLSETSIERLVQSIQEVLH
GELKGETQKLKFLRSLSSLSQTIPYDENTESFIHRHTAEIVHVLSVLVQEEPLHSLTSMV
RQEIFVTITDLSFQNVYLLFGSEDREDLLGLITKSIISLPATSTPIQLLKIVSNQAIEQE
CLYRETFEAFSVMLQSLVIKDPHLEFFDSIFKHIDPWLQSDRDHERERATTVMAQVLKCL
SSYLSLKLPLQFQRLGNLVAVMALLCGDPQKEVAEEAAEGTHYLLHITLRLKYIMQDRDN
NSKNLRETLKKCRLLLEQYSLKRFYCCPYKIVQVFEVFLDSRELGQFIMTILDSLKNLPH
PCIQQTAGELLITLVKSAEARFERVPEIVGVICARLSIITLTSVRQQIINTVSLFVSRPK
YTDVVLSHLLCHPVPYDRHLAELWRSLEVELSNTTWVLWRLLRKLQKCHEGPSQEKMAYV
AVAVTDALYEVLMGNKLRAAAFRLFPQLLMTLLIQIHHSIGLTMSDVSIPSGLYAEQEVP
MEVTPLCFAIQATKTLLLRTLCWQEFSIMEKSGGWTLLKGEGHLQGVFLLANALMERSHL
FAYKILYLLVPLINRGNDKHKLTSAGFFVELLKSPMARRLPKVYSTIRLKAWLNEESKLF
RILALRGLRNLVRHQEMREDIKSLLPCILSSLHETDERIILLAIQILLQLVRMLDSTTLA
TMMKTLLSLFGDVRQDVHCFSMTLFGASIKAVKHTEKKSVEDQVLESLVPLLLFSQDEND
EIAKESRRVLTLCARFLNWKLPREVYCKDPLYIKPNEVRRVCKFFGEKSKGKIDILDQTL
PYSKDPKLPIRRTAVLFVGLLSHYVDTSELKRKGTDWIEKDLRELLQDPEPSLRILASQA
LFRIQRVGSTSEAETSVSWLRKLLCCLHT

>Rattus norvegicus_MROH8(ENSRNOP_52093)
MPLPRRGGEPRLAGARVVRTSLPRATPRSPKRVGRGAPLLSHAQRLTRRRHSSEQVPPEP
EHADFRSGKWLQERAGGDARDLRHTPLARMGSRQRVSGTEEVVIPCASDSDSGSVDLQLS
NLDDIHKGGPSVEFADSDISDIPILPQESLPDNLRSPTLQEPLSEASVERLVQSIQEVLH
GDLKGELQKLKFLRSLSSLSQTIPYDESTESFIHRHTAEIVHILTVLVQEEPLHSLNSLV
RQEVFVTITDLSYRDVHLLFGSEDREDLLGLITKSMMSLPAICTPIQLPRIVSNQAIQQE
CLYRGTFETFSVMLQSLVVKDPHLEIFDSIFKHIDPWLQSDRDHERERATFVMAKVLKCL
SSYLSPKLPLQFHRLGHLVAVMALLCGDPHKEVAEEAAEGTHYLLHITLRLKYITQDRDS
GKNLRETVKKCRELLEQYNLKGFYCCPFKIVQVFEVFLDSKELGQFIMTVLDSLKSLPHP

YIQQTAGELLITLVKSAEARFEKVPEIIGVICARLSIITLASVRQQIINTVSLFVSRPKY
TDIVLSHLLCHPVPYDRHLAELWRTLEVELSSTTWVLWRLLRKLQKCHDGPTQEKMAYVA
VAVTDALYEVLMGNKLRAAAFRLFPQLLMTLLIQIHHSIGLTMSDVSIPSGLYAEQEIPT
EVTPLCFAIQATKTLLLRTLCWQEFSIMEKNGGWTLLKGEGHLQGVFLLANALMERSHLF
AYKIMYLLVPLINRGNDKHKITSAGFFVELLRSPMARRLPRVYSTIRLKTWLNEESKLFR
ILALRGLRNLVGHREMREDIKSLLPCILNSLYETDERIILLAIQILLQLVRTLDSSTLAT
MMKTLLSLFGDVRQDVHRFSMTLFGASIKAVKHTEKKSVEDQVLESLVPLLLFSQDENDA
IAKESRRVLTLCARFLNWKLPREVYCKDPLYIKPNEVRRVCKFFGERSKGKIDILDQTLT
YSKDPKLPIRRTAVLFVGLLSNYVDASELKRKGTDWIENDLRELLQDPEPSLRIIASQAL
FRIQRVGNIPETEISVSWMRKLLGCLHT

>Canis familiaris_MROH8(ENSCAFP_12816)
MALYWMNHHSLFYCWICSQEEVVIPCDSDNDSGSVDLQLSNLEDIKKDPSSLELTDSDIF
DITGLSQESFIDSFRHLTQQGPVSEANVERLIQSIQEVFNGELQSEFEKLTFLRSLSSLS
QTLPYDETTESFIHSHITDIVHVLNMLIEEEPLHSLFSSVRQEVFITIANLSYQDIHLLF
GSEDRADLFSLITKSIITLPSVETLIQLQEIMPSGSYNTECLYRQTFQAFSEMLQSLVVK
DPHLENLDTIFKQMDPWLQSTKDHERERATASMAQVLKCLSRHLSLKLPLRFQRLGHFVA
LMALLCGDSVTEVAEEAAEGMHCLLHVTLRLKCTIYDKKNHPNLRRAMKKCQELLELYSI
KQFYSCPFKIAQIFEAFLNSNELFQFVMTTLDGLENLKHPCTQKSAGELLITMVKNAESR
FEKVPEIMGVICARLSIISQPRVRRQIINTVSLFISRPKYTDIVIGHLLCHPVPYDRHLA
ELWRTLEVELPSTTWILWRLLRKLQKCHNEPTHEKMAYVAVAATDALYEVFVGNRLQAAT
FRLFPQLLMTLLIQIHHSIGLTMSDVAVPSGLYTGQEMSSEVTPLCFAIQATKSLLLRTL
CWQEFNIMEKNKGWTLLEGKECHLQGVSLLANALLERNHLLAQKVMYLLVPLLNRGNDKH
KLTSAGFFVELLQSPVARRLPSIYSIVRLKDWLHHGNRVFKFLALRGLCYLIKHQDMRED
IRSLLPCILDLLCETDEKIVLLAIQILLQLVGTMDFTTLTAMMKTLFTLFGDVRSDVHRL
SMTLFGASIKSVKYTDKKGVENQVLDSLVPLLLYSQDENDAVAEESRQVLTICAQFLKWK
LPQEVYCKDPWYISPSEAGTVCKFFGKKCQGKVNLLAQTLMFSKNAKLPIRRAAVLFVGL
LLKYVDYSELRTKGTDWIENDLKALLHDPEPSLCIIASQALFRVQKVGAEPDSRQSIPG

>Felis catus_MROH8(ENSFCAP_11066)
PKKSPSTCPFPTGEEAPAWPAPEWRALASRGLRPTRPASHAQRLTRRRHFSEQVPPGPEP
QADFRSGKWRQERAGGDARDSRRSARAGMGSRHRICSQEEIVIPCASDNDSGSVDLQLSN
LEDVKNDASSLELTDSDISDIAGLPQESLTDSFGHLTQQGPLSEPIVERLIQSIQEFFNG
DLQSELEKLTFLRSLSSLSRALPYDETIGAFIHSHIEDIVHILNMLVEEELLHSLFSPVH
QEVFVTIADLSYQDVHLLLGSEDQADFFSLLTKSIITLPPVKTLTQLEEIVPSGSYNTEC
VCELTFTLFLLPLIFVNQHSSHIGFLSFILVQMDPWLQSTKDHERERAAVSMAEVLKCSS
RHLSLKLPLQFQRLGRLVALMALLCGDPVKEVAEEAAEGTHYLLHITLRLKYVTHDKKNH
PSLRRAMKKCQELLELYSIKQFYSCPFRIAQVFEVFLNSNELCQFVMTILDGLKNLKHPC
TQKSAGELLITLVENAESRFEKVPEIMGVICAQLSIISQPRVRQQIINTVSLFISRPKYT
DIVISHLLCHPVPYDRHLAELWRTLDVKLPSTTWILWRLLRKLQKCHNAPTHEKMAYMAV
AATDALYEVFVGNRLRAATFRLFPQLLMTLLIQIHHSIGLTMSDVAIPSGLYTEQEMSSQ
VTPLCFAIQATKTLLLRTLCWQEFNIMEKNKGWTLLEGKDGHLQGISLLANALLERNHLF
ANKIMYLLVPLLNRGNDKHKLTSAGFFVELLQSPMARRLPSIYSIARLKDWLHHGNNVFK
TLALKGLHYLTRHQEMREGIKSLLPCILDLLCETDEKIVLLAIQILLQLVETMDFTTLTA
MMKTLFSLFGDVRPDVHRFSMTLFGASIKSVKYTDKKGIENQVLDSLVPLLLYSQDENDA
VAEESRQVLTICAQFLKWKLPQEVYCKDPWYIRCGKVGKVCKFFGKKCKGKVNLVAQTLI
FSKNAKLPIRRAAVLFVGFLLKYVDHSELRMKGTDWIEKGHIDLRDLLCDPEPSLRIIAS
QALFQVREVGTEQDPRQTVCGLRKLMGCLST

>Myotis lucifugus_MROH8(ENSMLUP_2058)
TPTPPQVPCIPPARQPSLRLWTPERRSPCPSPAEEGDPAWPAPEWRTLASRGRWPSRRGH
AQHLTRRRHSSEQVQEGSAPPADFRSEKWLPERAGGDAGNSRQAPRAEMASRDRICSWDV
VVPYASDSDSGSVDLQLSNLEDIIKGPSSLEFTGFRLRKDRPFISKSHCTQLGSDKFRYL
THDDAFSEANIENLIESIQEFFHGELKIQSEHEKLTYLRSLSSLSRILPYDETTESFIHG
HIADIVHILYILVEEEPLHSLFSPVRQEVFVTITDLSYQDVHLLFGSEDRVNLFSLIIKS
TITLPSEKTLTQLQEIKPSGSCKSECLYRQTFQVFSEMLQSLVVRDPHLENFDIILKHMD
PWLQSIKDHERERAAASIAQVLKCLCMHLNLKLPLRFQRLGHLVALMVLLCGDPLNEVAE
EAAESTYYLLHITLRLKYITEDKKNRPILRGAMKNCQELLEFYSIKQFYACPFKIAKIFE
VLLDSNELCQFVVTILDGLKNLEHSCTHQLAGELLMSLVENVEFRFEEVPEIVGVICAQL
SMISQPSVRQQVITIVSLLISRPKYTDTVISYLLYHPVPYDRNLAEMWRTLKVEVPSTTG
ILSRLLRKLQTCHNEPTQEKMAYVAVAATDALYEVFMGNKLQEAMFQLFPQLLMTLLIQI

HHSIDLTMSEVAIPPGLYKEQEMSSKVTPLCFAIQATKTLLLRTLCFQEFNIMEKNEGWA
LLAGKDCHLQGVSLLANALLERNHVLAHKILYLLVPLLNRGNAKHKLTSAGFFVELLQSP
VARRLPSIYSIARLKDWLHHGNNIFKILALTGLYYIIRHQKMREDIQSLLPCILDLLCES
DEKIVLLAIQILLLLVRTMDFSTLAAMMRKMFSLFGDVRPNVHCFSMTLFGASVKSVKYA
DKKGVENQVLDSLLPLLLYSQDENDAVAEESRRILTICAQFLKWKLPQQVYYKDPWYINP
SEVETICKFFGKKCQEKVNILAQALTFFKNAKLPIRRAAVLFVGIFSKYLDRNELKTKGT
KWIEDDLRHLLRDPEPSMHIVVSQALLQIQKVGAEPEPEQTVCGLRKLMCCLPT

>Monodelphis domestica_MROH8(ENSMODP_400)
SRFSPLEEMVFPFVPENDSGSVDLQLSKLEGIKKDANSIDFTVDSDFELPGTSKKNASED
VTPLLIEGPPGLIIVENLIDSVQDFLNGDRKGDAEKLTFLRCLSSLSQTLPYDETAESFI
HDRIEQIVNALNMIIQQEPLHSLFTPTRQEVFTAITDLSSQEINLLFGSKEKLDLFRNLI
RSMIALPSTKILNILQTVMPTGQYNTESLYRQTFQAFCEMLQSLVIQDPHLENLDQILEY
MEPWLQSAKDQERERAAASMAQMLRSLSRHVNLQPPLHFQNLGKLVALMSLLCGDPVEEV
TQEAAEGTHHLLRITLRIKCLTYDKKSHQNFKGAVKKCKELLEIYNIRQFYSNPFKIAQV
FEVFLSPNELSQYIITTLDGLKNPKHPCIQQSAGILLVTMVKCAETRFEKVSDIIRAICG
RLPIIGQFIARQHAMNIVSLLISKPTYTDIVLDQLLLHPIPFDRHLAELWRTLDVDLPNT
TWILWRLLRKLQKCHSMVEDKMAFMAVAATDALYEIFVGNKLQAATYRLFPQLLMTLLIQ
MHHSIGLTMSDVVFPSGMYIEEEVVITCTPLSFAIQATKALLLRTTCWFEFYTMEKKKGW
NLLELESDHIQGVSLLANVLLEKHVVLAQRILYLLIPLLNRGNEKHRLTSAAFFVELLRS
PLARRIPKRYTIVRLEEWLHHENYQFRVLSLKGIDNLLCYHQGMEEIDGMIPAILSNLSE
ANERIVLLSISVLRNLLNTMDFTTLKNTMHALFSLFSDVRPKIHLLAVTLFGDSIKMVKS
KDKKEIEGRILESLIPLLLHLQDEDDDVAKESSRVLSIICHFLKWKIPREVYHKDLWHGR
SGIEAICKFFEQKCRGKCDMLIQAAKYIQSDQVNLRREAIFITGALAKFMDRNELRTKGT
EWLEEILRPQLYDPEPSVRIIAAQALIRIQRAVLAQPEPTPVLSRLRKLFNFIYH

>Homo sapiens_MROH9(ENSP_356733)
MLTRNPKTKSSLQILQDSVKWHHMAHKVNSLLDAYSGLLSNESMILAVNSSFVDPLLQFE
SQLKIIESSFGMLVVMPSLDKVKEMGSSYEYIEDMENLYHNILNIYENILTSLVSKDLYK
LQILKEMLVWMSKDSSYLQERIMVIINKVLRFTVTKVRKYISVDAPCLGLLAAELSLLCS
HEDPSIVKQASLGMCHLLYIARCQNDIGTNKPTNGKSHSLQFPSSDVEFLPKEFQQDESK
IAQRVGQTLLPPLLTDFVQSLLMKLSSPDDKIASDAASILIFTLEFHAEKVTMVSKIVDA
IYRQLCDNNCMKDVMLQVITLLTCTSPKKVIFQLMDYPVPADDTLIQMWKAACSQASVAP
HVLKTILLILKGKPGEMEDTVTEGKRFSLDITNLMPLAACQALCTFLPLGSYRKAVAQYF
PQLLTTLMFQVFYNSELKPILKDRALYAQDALRVLLNCSGLQQVDITLMKENFWDQLSED
LCYYHGVCFIAKTLSEYNFPQFPETLSYLYKLSVEGPRRSEDTVIVLIFLTELLNNFFKD
PLPEEFLVLFINWINDSNPVVSRLILHRIVHMSPIINKTENVSSILIAILDAFLSKDDNV
VLQALLTLRRLLNELDKVTYSLGTRIGSSYCTLMDHINGGIRSMAIRHFGQLVRDMRQYT
WMVNDVVLEGLVPLILFLEDDDKRVAEACKYTLKICTSQLKWSTSRLLKDENYSFEMVVL
NICNNLIISHRNYITDLTSDTLRFLWSPRTYLKRASVILIGYLAKSGGHLLLRDEIEVML
DVIERLLRDEDPMIKQLAEITYDIFKKKAHKLTSAPLKQNFQKLLKLFYIKKLKPLYNYN
SPNGQIDSPTDSKDVKNDKAL

>Gorilla gorilla_MROH9(ENSGGOP_9013)
MLTRNPKTKSSLQILQDSVKRHHMAHKVSSLLDAYAGLLSNESMILAVNSSFVDPLLQFE
SQLKIIESSFGMLVVMPSLDKVKEMGSSYEYIEDMENLYHNILNIYENILTSLVSKDLYK
LQILKEMLVWMSKDSSYLQERIMVIIKKVLRFTVTKVRKYISVDAPCLGLLAAELSLLCS
HEDPSIVQQASLGMCHLLYIARCQNDIGTNKPTNGKSHSLQFPSSDVEFLPKEFQQDESK
IAQRVGQTLLPSLLTDFVQSLLMKLSSPDDKIASDAASILIFTLEFHAEKVTMVSKIVDA
IYRQLCDNNCMKDVMLQVITLLTCTSPKKVIFQLMDYPVPADDTLIQMWQAAGSQASVAP
HVLKTILLILKGKPGEMEDTLTEGKRFSLDITNLMPLAACQALCTFLPLGSYRKAVAQYF
PQLLTTLMFQVFYNSELKPILKDRALYAQDALRVLLNCSGLQQVDITLMKENFWDQLSED
LCYYHGVCFIAKTLSEYNFPQFPETLSYLYKLSVEGPRRSEDTVIVLIFLTEVSFVDCEQ
LCSHFLFLPKFKSKFQFLVSLPLNVGSYQDLRS

>Pongo abelii_MROH9(ENSPPYP_602)
MLTRNPKTKSSLQILQNSVKWHHMAHKVSSLFDAYSGLLSNESVILAVNSSFVDPLLQFE
SQLKIIESSFGMLVVMPSLDKVKEMGSNYEYIEDVENLYHNILNIYENILTTLVSKDLYK
LQILKEMLVWMSKDSSYLQERIMVIINKVLRFTVRKVRKYISVDAPCLGLLAAELSLLCS
HEDPSIVQQASLGMCYLLYIAQCQNDIGTNKPTNGKSHSLQFPSCDVEFLPKEFQQDKSK
IAQRVGQTLLPSLLTDFVRSLLMKVSSPDDKIASDAASILIFTLEFHAEKVTMVSKIVDA

IYRQLCDNNCMKHVMLQVITLLTCTSPKKVIFQLMDYSVPADDTLIQMWQAAGSEVSVAP
HVLKTILLILKGKPGEMEDTLTEGKRFSLDGTNLMPLAYAQDALRVLLNCSGLQQVDITL
MKENFWDQLSEDLCYYHGVCFIALSL

>Mus musculus_MROH9(ENSMUSP_94365)
MIQETTNTMTRLQTLQSSVTWYHVEFKAEILLDTYSGLLSEQSIILAMNSSFVDPLVQFE
SQLQIIELSFRKVFSMPSLSTVKAMGRTSEDTEDLENLYQNIFNIFEDTLLILVSKDFYK
IQILKEMATWMSEDSQYQQERAMMVITRVLIFASKRVKKYRSIDAPCLGLLAAELSLFCS
HNDPEIIQQAMAGMNYVLYIALCQNSNVVKFRSSKPSEEGSNIKVMTSGVESLAKMTQGE
KTKIAQSVGQILLPQLLTDFVWTLLKKLSSPNNKMAIEAASLLKLTLEFHAQKITRVSKI
VEDIYEQLCGNPSPMMKSVMLHVISVLTRASPKKVIFQLMEFPVPADKTLLLLWQAASME
SSVASEVLKTILLILKGKPGEIEETLGEKRRFSLDATNMMPVAASQALCTFLPVTSYEKV
VVKLFPEFLMALLLQLSYCSHHLKNTARNRPLYVRDALKALLNCSGLEEVDKALEKKNFW
DQFSQVMDHQYGIHLIAKTLSECNFPQFPETLHYVYKIAVEGPRRSEDSIVTIIFFAELL
NNFFKESLPEEFLVLFRNWINDSNPSVGKMSLQKIASMAPVFSEIESVCNLLLPVLDAFL
SKEHTVIIRAMLTLRNILSKLDKKIYSTVCIRIASSYSPLMDHVNSGIQCMAIRHFGELL
MDMNHYNWMLKHIVLGSLVPLILFLESKETRIVKACRYTLTICVTQLKWSTASLLKEETY
NFESVVLDICNNLLSSYESYITDLISDTLGFLGSSRDFLKKGAIILVGYLGKSGEHLLLR
DEIDIMIEVIERLTRVEDALIKELAEKTRKLFKEIANRMSSSTIKQTFRKWVKMFRTKKL
KLIYDVASIRTLEGNEIEIIKDSLIESNLEKSEKPYRVTKENYFNESLYTV

>Rattus norvegicus_MROH9(ENSRNOP_4931)
MATWMNEDTLYQQERAMMVITRVLIFASKRVKKYPSIDAPCLGILAAELSLFCSHDDPEI
IKQAITGMNYVLYIALCQNSNAITFRSLKTNEKESNPKIITSDVELLAKITQGEKTKLVQ
SVGQILLPQLLTDFVWTLLKKLSSPYNNTAKDAASLLKLTLEFHAQKITRVSKIVNDIYE
QLSENSSYMMQNAMLPVISLLTRASPKKVIFQLMEFPVPADKVLLLMWQAASVESSVASE
VLKTILLILKGKPGEMEETMVERRRFSLDATNMMPVAASQALCTFLPVTSYRKVVVKFFP
EFLMALLLQLFYCSQHLKDTAQDRPLYVRDALKALLNCSGLEEVDKALERKNFWDQFSQV
MDHQYGIHLIAKTLSECNFPQFPETLHYVYKVAVEGPRRSEDSIVTIIFFAELLNNFFKE
SLPEEFLVLFRNWINDSNPSVGKMSLQKIASMAPVFSEIESVCNLLLPVLDAFLSKEHTV
IIRAMLTLRNILGKLDKVIYSKVCIRIASSYCPLMDHVNGGIQCMAIRHFGELLMDMNQY
NWMLHHIVLGSLVPLILFLESKETRIVNACRYTLTICTTQLKWSTAHLLKEETYNFESVV
LDVCNNLLSSYEGYITDLISDTLGFLGSSRAFLKKGAIILVGYLGKSGEHLLLRDEIDIM
IEVIERLTREEDPLIKELVEKTRKLFKEMANRMASSTVKQTFRKWAKMFRTKKLKLIYDA
ANMISPKEGNTESIKEDNLIRPNLENSKELHRVINEDKLVKPNVKKSGYFP

>Canis familiaris_MROH9(ENSCAFP_22081)
MKTRKNLRHSKDKETGLQTLQSSVKWHHIVNSAPSLWDAISRLLSDQAMVLSLNSSFVDP
LLQFESQLNIIESSFKMVFAIPSLEKVREMGTSEDDKEDLENLYQSILNMFEETLLILVS
KDFYKLQILKDMIIWMNEDTSYLQERVMMIISSVLSFASKKVKRYASIDAPCLGVLAAEL
SLLCFHNDPLITHQASLGMCYLLWIAKCQNDIEEIKSTMCNSGNHGLLPVPSDTEFLLMD
LQQDKTKITQYVGQTLKPSLLTDFVWSLLMRLSSSNCVTAFEASTMLKMTLEYHAHKVTM
VSKIVDAIYKQLCEHSSHVMRHTLLRVITLLIRSSPKKVIFQLMDYPIPADNTLLLMWQA
AGSESRMAPHVLKTILLILKGKPGEMQDSLMERRRFSLDTTNMMPVAASQALCTLLPISS
YKRAVAEFFPQLLMALMLQLFYSSDLRLMIKDRLFYARDALRVLLNCSGLQKVDMALKKK
NCWNQFSQVLSHHHRVYLVAKTLSDYNFPQFPETLHYLYKLSVEGPRRSEDSVIIVIFFT
ELLNNFFKDPFPEEFLVLFRNWVNDPNPAVSKLSLQKIASMAPVINKVENVRSLLISILD
AFFSKDKTVIIWALLTLRKLLGKLNKVTYSSLSTRIASSYYPLMDHSNGGIRSMAIYHFG
ELLKDMSQYPWILNDMVTGGLVPLILFLEDTEIRVAKACKYTLKICASELNWSTSYLLKD
KHHNFELVVLNICKNLLISHESYITNLISDTLGFLRSSRIYLRRASVILIGYLTKLGSHL
LLREEIEVMLDAIDPVLRDEDPVIRELAEITYRDFKEMSLKLTSSNFKQIFRRLFSLIYL
KKLKPLYNWPDNLADSL

>Felis catus_MROH9(ENSFCAP_2773)
LHLLSSLLNNCTFFLISFVDPSLQFESQLKIIESSFKKVFSIPSLEKVRQMGNSEDDKED
IENLYQNILNMYEDTLLILVSKDLYKLQILKDMAMWMNEDSSYLQERAIVVISRVLSFAS
RKVRGYASVDAPCLGVLAAELSLLCCHNDVSITQQASLGMHHLLCIAKCQNEDIEKTKST
KYEKHGNHCLLSPSSVQEFVPKVLQRDRAKIAQSVGQTLMPSLLTDFVWSLLMTLSSPNC
VTAFKASTILKLTLEYHAHKVTMVSKIVDAIYKKLRENSSHVMRQAMLRVTTLLTRTSPK
KVIFQLMDYPVPADDTLILMWHAAGSESSVAPHVLKTILLILKGKPGEVHASLPERRRFS
LDTTNMMPVAASQALCTLLPVGSYKKAVAQFFPQLLMALMLQVFYISELRLVTEDRPFYA

RDALRVLLNCSGLQQVDIALKKKNCWNQFSEELFHHQGVYLIAKTLSEYNFPQFPETLQY
LYKLSVEGPRRSEDTVITVIFFTEVSFIRRLNNQVLFPYANLRQHSNLLSPSLNIDSYQG
LRQ

>Myotis lucifugus_MROH9(ENSMLUP_5298)
VTNLQILQSVVKWQHLIQEYTATSLWDTLATIFNHQALILAVNSSFVDPLLQFETQLSII
ESSFKVVFAMPTLKEVRDIGNNESEELDLEELYQTILNIFEDTLLILVSKDLYKLQILKE
MAVWMNEDNSYLQHRVLVIICRVLNFASRKAKGYGSVDAPCLGDLAAELSLLCSHTDGSI
TEQASFGIYHLLCIANCENVNIAEGKLTKCDKTGSYNWLPSLLDTEYLPDILRGDKTKIA
QSIGHTLEPSLLTDFVWNLFMRLNSPNPVMVKEAANMLKLTLEFHANKVTMVSKIVNTIY
EQLSGNSTHIMKQTMLKVITLLTRTSPKKVIFQLMDYPVPADNTLILMWHAAGNESSVAP
YVLKTILLILKGKPGEIQDSFLERRRSSLDATNMMPVAASQALCTLLPVSSYKKAVAQFF
PQLLMALMFQLCYSSNTQMGSEDNLMGPSYARDALRVLLNCSGLQQVDLSLKRKNFWKQA
SQVLFHLHGTYLVAKTLAEHNFPQFPETLHYLYKLSVEGPRRTEEGVITVIFLTELLNKF
FRDPFPEEFLVLFRNWVKDSNPAVSKLSLQKIATMAPVINQIESGCSLVLSILDAFYSKD
KTVVIRALFTLRRLLNKLDKVTYSSLCTRIATSYFPLMDHTDAGIQSMAIRHFGELLRDM
NQYTWMLNTVVLESLVPLILFLEDIETRVVMACKHTLAICALELKWPAFPQFMEHYSFEM
VVLNICNNIFISHGSYITDLLSDSLGFLRSSRVYLRRAALILIGYLAVSGRHLLLRDDIE
VMLEAIERVLQDEDPLVEELAKITYGLFKKIAHKMTSSVIKQSFRRLFNFIYIKKVKLLY
NYHSPGSSVEIKETDNYEEDVEDDQKPKFIKLTNKFEKSHKYSI

>Monodelphis domestica_MROH9(ENSMODP_4394)
MDPLEQFEIQLQTIEECFRNVFSMPNLSKLLERQKNSDDQEDIEHLYKSILCIFEDTLRI
LVAKDLYKLQILREMIIWMNANNRYLQERVMTIISKVIKFSRRKIQGYKTVDIPCMGHLA
AELAVLCSHKNLQIVIQASIAMYHLLCIIKFQNVDVPSDTSAEVENCLYFVPFDKEFTPK
LLQKNNEAIAKYVGDYLKSSLLDDFVFSLLKKLPKYPSHTSTEASALLKLILEMKGDQVI
RVARIVDFIHTQLNANLPIDLKQTLLQIIALLTRSVPQKVVFQLLDYMLPVDRSVLKMWK
AVSTESKSSCLVLKTILIVLKGKPGEVEESSKIRKRFSLDTANMMPVVAAQALCILLPVP
EYKKAIAQFFPQLLIDLILQLHYNCEQNYKKNDNTQRFPQNALRILLRSSGLENVDSALN
KRDCWNQFSSLTFHHYGVYLIARTLSEFNFPQLPETLHYLYKLSVEGPRRKEDSVTTVIF
LIELLNNFFKDPFPEEFLALFKIWVNDPNSTVSKLSLQKISTMAPVINQIKNIKELLLCI
VDALPSKDKTVIIQAMTTLRKLLSVLDKVTYSIICMRIASSYYSLMDHETASIRAMAFRH
FGDLLKDMKQYTWMLKPAVIKCLVPLLLFLEDSELRVVKACKYTLNICAPYFKWKVIPLL
QDNLYNYEIVVLNICNCLYNNFGSFISDIIFTTLGFLKSDRGFLRKAAIIFLGYMAKMGS
NVLVKNEIWNMIDDGETESQRDLRLHRY

>Drosophila melanogaster_(FBpp0071261)
MDGRGVGGGGSGAATGGGSANNKSAAGNSAATGNADASTATAAPNDKPTILEGVLHNIFD
GLMDKEEQVRIAMQQAIVKILETHPERAAEILSEHRSQQPKMTDQTVAMLLDCIRRVVGA
ETPLPPAANQKLIDLALQELTKNPEHLPLIQNPAQRILVAIGRSSPESCEQVMEALQEKG
TSTEGVAHFMLMQCLGLLATENPAGVVPHIKAILSRSQPHLVGIRQDHIKQAHAYAIGRF
SEALLEESGKKEEENCSTEISVAYDVLFNQWLHSREPKVCVEILQALSSMYPLLPKDRIQ
DQAARLVPQILALYRRSVDRNAVTQFLCSVLKTNLALNATVLDGIIDVLVTHLFDLVCVY
PDYEKPQTVKGHYEVLRCFHLLAGHQPYSTRIMDTLLIHLRNNSERERMKSLLILTHLLN
SCAGNIENRIPATIECLKQLILSERGIKMKLTLLKTIVALAQKSHIRDKEFVWFVVRHSC
RFSKPSQEHGSQEEHANLVLSCENTLYMLASTVGTLDELLKRELLNYLILLDYTDICGNL
AKCLASLFAKSPHIEYDIAGDDAATEQATPESMGAAGEDRSVIKRGKVMVPGAETIYARC
LALLGNQQCIKRCSNILSFLRYYHPQVNPALEELWERKIPDLLLQINRESAYRQLLHDFV
LETNEFLGGLDENFAQRLASKLADQMYLYPMQLPHSEWHLPDLSAERGMLLQAVALTLLQ
VTDVACIHTKIDLIVTTARQERLDKHVKHADYEKRIEPCARALGYISRQHLGHLIKKLTE
LAQVGGRKHSTGFFSNLHFIKDTHKELENYKSNLLVVKAFGRIMDEADPLQSIQHLDEDD
TLLGFLIQQLAVHKDQTIMSAILQTLLSICNQLIATKEQLPAPLRHRKQIMETVFNIPIE
APFHDLPLLPTILKLGTDFIRIGGPDTEECVDGGVIFEIACRNFFGCAQQLKMKFDSQEE
DERNSFLAKHLNESLPQLNALVRAIVELDASPATLDLIIGILEGWTRDRNSEVRICASHV
FNNTLDVYIKSMKIGCEAPSKFNQTGQMLGKIVPRCIDSNGTVRQVSVEILQKTLEIACI
YETLTIASIDSTADWLKEIETIKEHIITDEPKQIYNLAGDIAKIIALRISSFQYLQFCKT
LLHSLRDPEQSSTIGASVVLKFFIQQKGSELFHAIPDLVRDSLLALRVCEVPRAKSGVLK
ALVALTKHHPKLVCAEMLSQPLPYDPNLVEYWHLVCNDPELTGLTLDNFLQLLSGASLQE
GGQDASLSERQKLASGQPFAIFCALHEMLPCKDIKGQLETRFADMFCMLLTSLSSYTNLA
APNPINNASLSPNQTQSGKTKFGFVPNKELIKLNPCQIALETFQAFLTNLEMEQIASVLT
VNTQLASSADWHNYIELLTPMAIGLGQQLQLGSPQMRQLVNSLSKYVASPYDGQRVAAVG

IFSRLVPLKPTGELAASILLHLGAALSDPNAVVRGLSIQGMGYVGQLGEKEAKRYSETAI
GALLKGVDDPVGDCLINIPLESMRGLSGILRALPSERVEPFHVSLAIRIRPFLGNYALEM
REAAIQLFGDICEGKHDDGSSSPTSSMEALREQLIANLFPLLLHLSESEVAIASACRGTL
QRVCRLLTAPRVVEMAEQQLGEERGHQLNYSSFVLEFVKTIALELTDHIQDFIDSCLPQL
RSQWPEVRGSAAIVIGILHNFLSERNVQTETVASKIAVLLKDEQALVRMRAATALGYFFG
DI

>Anopheles gambiae_(XP_001237319)
MHISVAVVTSLLDSLNDKEELLRTTTEASLIRIAKRRHDEIVEVFCDYRKKHPKLGDTHTLIILRVISYI
ATNLIDVIDPNTASKCIQYSMAEIMKLTEQNASVQNPCREILVAVGRRYCKEIMEIFVKHLEQNQLGNFM
IVQSVGALATANTLDTVPYIKPILALMLPTLSAVRQDFLRQAYAYAIGRFCEAFSEYQEYQRQNPNEETP
MKTGVRYEIQDEVSIVYDHFHAQWLASREPKLTGEVLNAFAYMYPLLPVERVSDNLAKTVPSVLALYRKS
LDRSAVTQYLASIIKTVLQLDSKLLAPMADTLVGCLFDLVCVSPDYDKPQTVKGHFEVLRCFDLLAPEYG
EKIIEILLIHLRNNSERERIKSLLVVTHLTNTSEPVVRDKLPEITAILRGLLATEKPVKVRIVLLRTVVA
FMQKNFVRDREFVAFLIRCSCRPAKLNLDHGSAEEHQEFQRACHDTMHILSSTVGTVDRMLRAELLQAYL
RYEYTDICGTIGKCLANLYAKDPELGLHGAAAAGAAPDRHSDNPFDESAGGGGVELGDEAQPGAPPPPRP
IAVFVRTLALLGNFGEQGRIQHLLAFLKSYAGSLYRHLVPLWGELLGALQAELAGGIDEQRYFALLFAFV
QETIRDVDEYTFVEGVIAEMFHQLPLYQPASGGGSGGSAGGGGGGGSGGGGAGQAAEFRVPPLDQERRML
VKVFGVCLCHTRDEHLIENKLDLIVALAKGEKADKHAPTEEYERLMQDYAEALGYASVEHLDRVMGKLTA
LVIDDGAVKKSSSFFANLNFIKDSARELEAYKLKVLALQSLHVIVGRAPKDAIAQHYTDAVVRYLIAQFD
SKELFVRQLVLKTLLALTAVLSPSDGDERLASERNRADLHRICMTITADSANEYLPLLPSIIQLATVLVQ
LSAEEQNLDVNGLLNAICFYFFTTAQNLKSRLDTTEDDARTSYLARFLNRSLPEVNQFIRTVLVQQNASP
ACLDDVHSILEKWLKDRNGEVRICACHVYNSTLEVYMRSMKIGCEAPSKFNQTGSMLGKMVPRCIDSNAT
VRQTAVDVLKKILEIACVYETLTVADSSVEWVGELDRIRDEIVTDDAKDIYRLAAELARIIAQRLSSYQY
VQFSKCLLYSVSDPEPSSVIGASYVLKFFMHVKGSEMFHAIPELVKECLYAVKICEVPRAKTTILKSILA
LTKHHPKLVCNEILTQCLPLEEHVIEYWKTLTMDADLSGIILDNFIGAVTSTCLYEQPQQQQQQPDPTD
DAARTATLHPFAIVCVLREMFACAELKSEMQSRFPEIFCMLLSTLASYITLLPPYSVLAQPNAAGNVTIP
KGGSRRGAKANGAPGGTGKEAAAAKLSPCQIVLDAFQTFLDTLGMQQISLVLAVCPDLAASTDLNSFIEI
LTPLGVATASEVGINSALMRQLVTTMSRYVSSPYDTQRIASTGFYAHLVPLQPYGETASVIMLSLESSLN
DPNPLVRGLSIRGLAYVCSLTRHDIDKYATMCLTSLLKGIEDYNERCFINIPLDSMRGLSRVLQAIEPAK
FEPFQVSSAIRIRPFFEKSSTELRESAILLFGDLCGLKLKQLPAGEAGQHDGADVSESLVEQLRANLCSL
LLHLCEQNSMIARACKITLKNVCALLGTAKMNALAQNHLLEHGQLQCAVFLKDFVKLIGEELQEWINDFI
DACLPLLRSQWPEIRGNAAILIGLLHCQNANVKCQHMEQIGHKISLLLKDECTTVRVSASEALGYIYGEM

>Capitella teleta_(ELT91566)
MALIDAQLDKDSNVRDVVGASLRDLGSKQTSLVLTSCLNYLSKHVKLPVAHKIAILNCMADVVRANLEKV
DKELAIAIVKEAATQMTQTKDAVPEWQAAASHVLVAVGCQWILESNTELIKRFNPGQLPDFFIVETMGNL
AKENVYGMVPYIKAGLSTMLSVIGIAKQDNIRWIFAYTISRFCEAILDYCANMDNFDSDPNVNIAFYSDE
IDVIHEVLLNNWIGTRDAKIREQVIEAVGQMSYVVSKPKLQERLPKLLQTFSNLYKKHPEPFHVSKGLYN
ILDANSSDILHPHVESILQAIFPQVCHAVDYSVPMTIKNHNELLRCLSVLVRVDSMLVVMFLMEKLENSN
ERVRIGVLSIMRHLINSAGEEMADKKDIFISGLKKVTGDPNNKVKKILAQTIIAMAHHGYLQLVGGQEMI
EFIVNQCALPTETPGRRPTDPEYVSNDALRSMCDNVLQLLTTTVDNMGKVLWPFLLECVIPEKYTMAQST
ICRCIAHTAAKKKEENDDSFYVDFDASANVPKPQALLARFFVLCGLPVQSKHRGLHALTALRWLAPLIHE
NLIEMWDIVIPKLIKHLEDHQDLDDWSQNNWEDLMLKFVSKSLEQVGDEEWICSMGDAMAEQISLYDNLH
DEKNFLYKCLGIIMRISSKKDFVSHHLDTLFSTVKHANQVEREGCAVGLGYAAASHLDIVTTKLDLIAKR
KSDSTRKSGILSFIKDFGSGSELDDQTKATILLCYGYVALYAPPHLIISRLQTQILVAINPHLTNAKLLL
LSPPLPSQDPDVKQNLIRTIELIGRSMHPEHLNARHDFSHKNKLLQHLQDYIKAESPTQVTGETRYLAFK
AMCVLVQLTPRLMSSELAALLQNVLDASMQLPPLPPNADAKDSELLLSNTLKSLQQLLLDILSTEMTTKG
LDIICTALQPWRVSSHSHERERAMELSLKIYHHFLQGVDVVSVSSLLAFCSQGSCLGRIVPRCSDPQDDV
RESAFNCIYTLLKIKKHAYFSFPRKRPDYSDPFVESVNELKHKIDVEDSAGLFEVISQLAEVLRQKLSSD
QLMPFLQVLMEGLVDAEPFCSSGACVVMKSFMESRGAELHSQVATLQTGLHAKLSVITHPQTRTGTLRAV
RALGKHHMLTMLEHLMQRTLPLDAATLDTWKVIGQKASLTAAFMETALEMLRQALPYEERSDKERVIRSA
TQHSMALTVVLTELCKGEEAAPVVASNYARLLASMMTMVSSMVDVRPPQQQAKDANGKPAKHSSALTTAR
IDPSEVSIDGLRQVLIASQCPAIMQALDSVEIWGKFRNIEQVADGFALLTKCLVEFQPHCIGHLVSNLNP
LLGASVYDPQRMMAAAVYAQVISERSLIEEGCAGDSNLVDLLVNGLLARLVDSNHRVRMLCIRGLGNVAS
AGSEQLRKYSMSVVSAMIAGMDDKEDVDDYITLEAMEGLSRILAEIDEEQIRGVLINVSLRIRPCFEKNN
DRVRAVAIGLFGDLARFGDGPSKDPFVEQIHSNLVTLLLHLNDPSPQVRLACKKTLRSIGPFVGSEAINE
MFQKHLLESASLIYGEFMNDLSKAINKDFVDKLNFYTMGNVSFFRSSWSEIRSNAAMFVGFLLGNMDSSS
ASNLSKEHICGALILLLKDLSPDVRIKAAEAMSYLHCI

>Hydra magnipapillata_(XP_012564350)

MASELLGGNKYVSSSVVIPIVCHLSREMVIQDDYPRYITLFKEKFVNEMERRFKDFLNDTSNERSNIWQK
LSELLESVLDIRPVSIQQKPKVKMDSYTKDYSYSSIRRKAKKQAELDLNTTTDFNLQSNFSSETIRDLNF
ANDNVNDIDSEENNYVSSSSDNQDENFDGEINNILRDWSLQYNITHVALASLLCILKPYFPTLPQHPRTL
LKTPTILEVKKLKSGGDYCHIGLENGLKNCINDLFIPENTNIHLQINIDGLPLFKSSGVSVWPILCIVKN
FSMKEPFVVGIYSGKEKPPSAAEFLEEFVEETKHLVDNGFLFNNKRYSVKIHSFICDAPARAFVKGIKGH
SGYNSCEKCATNGEYDGKLIYPETNAPLRTDLSFFHMIDKKHYLWECPLKLLNVGFVSQFGLDYMHLVCL
GVMKRLILYWKGPKGPLHVRIGTRAMDQLSNSLNSLSQNIPSEFSRKCRSAHEVLRWKATKFRLFLLYLG
PLVLSNVLSLPLYNHFMLLNVAITILVDPVFSKDYSEYAHELLVLFVNEGKSLYGKGFLVYNVHCLIHLS
KDVQTLGPLDNFSSFPFENLLGQLKKMIRKPQFPIQQLVRRLGERKLSSNLKNDNNLDELNLLKGEHFNG
PLPNGFEHAKQFKKVQLEKWFFSTSNKDNCFITNDGVPCIIRNMLLINNDITFVCEKFLTVYESFMFPIS
STKLGICKVS

>Trichoplax adhaerens_(XP_002108256)
MSASGKQWLSEYGSEYSTGGQIDEMVLALIESCADKSESVRATVADSLTSIGKKKPALVLNFCKVFLEKR
TKLDKGHRVLLLGVIKRIVTETIDTLPSQVGCDLVRLASSEMTASKEVIVDWQTAASEVLVALGSRFAKE
VITSLLDKFGAGAIPHYFVVQTLANLASKNAYCVVPHLKDTFGRLLPMLGMAKSDNMKYIFSYALNRFCE
SISEYVANIDKAPDKKISSGMYFGEVSAGYDVLFGVWLQSKDSRLRSVVAEAIGQMALILPRDKIEEQST
KLISVIVSLYRKHSPNDHLLITECLCNILAAHLQVGCQIADSLADNVMATLHNQACAFADFGSTSSIKNH
NEVLRCFAVIGPITSNRLIQFLLNKLENPNDKIRIGTLNILKHIINASGIPQDKMALIISGLKILLNETN
NKVKQTLAQVISAMAHHEYLHLEGGQLLVEFIVRQCALTDNLQKTTKPDSEVSNASLRVMCGNVLQLVTT
TVAHMQNVLWPTLIEFIVPMKYNNAAGVICRSAAFLANKKQSEESSDYYLDYACQVNLPKPVALLARLLV
LAGLPKRSCGQGLHVLNFLLAMSSNINEAFADMWDSVLPRMITYLEDCITLEHSAGDDWNQKAWEDLVLK
LLTKSLEEVEDENWTCELGSEMSSQIVMYESFPEEKASKNFLYKCIGVILRKTTNKAYVEKQLNIMFSSV
NHTNQTEREGCAMGMGFASYAHLDAVLQKLEDVPKREMDKSEADVERIKSTLILSYGYVTYYSPVNLITS
RIEVNILRVINPQFGNVKDSTVKQNLIRSVALIGHALHKDHLKFSFTFNKRDDLITHMLAEELAEKTLRS
FNLMLCEILENGSPSNDFFMLYKVHKKIYSPHLENWIYSSHERERDRSLQSLAAILEWYLEHAESENKDG
SSKKFGAVIARLAPRVADPLASVRESTITCTQLIFRIEMQFQAIGNDELVDAMDLLIERCRKEESTVITS
VVNDYGKILAKKIREVELVEFIYLLLDGLVDCQLQSAVGTSAIINAVLRSRAGGLREEKLREKLEEIDDD
KCIKGVRKSFRVLASYHLLPVLDVLLNFKLPYDRYVVGLWKTLAEEEEMLGSVCSHFMDLLKKSFPFQEQ
GRKEIIKIATEQPIAIACGFNELFKVPTAVSIAKRDYPQLFSTLLLRIGTCIKVQQVKEKSSKAHGVSPM
RTAISAFQGFLTLSEEFEVLDEIDKEDGWDKIRHDKAYSDAIVAITSALCKSILLRPHIAKVIAYLMPAL
NGPYDNQRLIVAAFFAEIINQRCLGELGQADIIINCLLSRLVDDNLKVRMFCVRGLGNVASVGREHFNAY
STTVLSALMSGIDEKDDSDCDITLEAMSGLSRIIAELDETHVRSILINISLKIRPCFEKSEEKVRARAFT
LFGHLSKFGDGPSKVPFLEQIQANMVSLLLHLNENEASVVKACKFTLRSIGPLLNNSEIDYMFQKYLLDD
ATLYYGEFLNNLTRLLISEYKEKTSLYVTGATRFFKSEWEEIRANAATLIGYYLGNLPNELRETISKEHV
CNALKMLLKDTSPMVRSGCATATSLLYEY

Myelin Transcription Factor-MYT

>Homo sapiens_MYT1L(ENSP_382114)
MEVDTEEKRHRTRSKGVRVPVEPAIQELFSCPTPGCDGSGHVSGKYARHRSVYGCPLAKK
RKTQDKQPQEPAPKRKPFAVKADSSSVDECDDSDGTEDMDEKEEDEGEEYSEDNDEPGDE
DEEDEEGDREEEEEIEEEDEDDDEDGEDVEDEEEEEEEEEEEEEEEEENEDHQMNCHNTRI
MQDTEKDDNNNDEYDNYDELVAKSLLNLGKIAEDAAYRARTESEMNSNTSNSLEDDSDKN
ENLGRKSELSLDLDSDVVRETVDSLKLLAQGHGVVLSENMNDRNYADSMSQQDSRNMNYV
MLGKPMNNGLMEKMVEESDEEVCLSSLECLRNQCFDLARKLSETNPQERNPQQNMNIRQH
VRPEEDFPGRTPDRNYSDMLNLMRLEEQLSPRSRVFASCAKEDGCHERDDDTTSVNSDRS
EEVFDMTKGNLTLLEKAIALETERAKAMREKMAMEAGRRDNMRSYEDQSPRQLPGEDRKP
KSSDSHVKKPYYGKDPSRTEKKESKCPTPGCDGTGHVTGLYPHHRSLSGCPHKDRVPPEI
LAMHESVLKCPTPGCTGRGHVNSNRNSHRSLSGCPIAAAEKLAKAQEKHQSCDVSKSSQA
SDRVLRPMCFVKQLEIPQYGYRNNVPTTTPRSNLAKELEKYSKTSFEYNSYDNHTYGKRA
IAPKVQTRDISPKGYDDAKRYCKDPSPSSSSTSSYAPSSSSNLSCGGGSSASSTCSKSSF
DYTHDMEAAHMAATAILNLSTRCREMPQNLSTKPQDLCATRNPDMEVDENGTLDLSMNKQ
RPRDSCCPILTPLEPMSPQQQAVMNNRCFQLGEGDCWDLPVDYTKMKPRRIDEDESKDIT
PEDLDPFQEALEERRYPGEVTIPSPKPKYPQCKESKKDLITLSGCPLADKSIRSMLATSS
QELKCPTPGCDGSGHITGNYASHRSLSGCPRAKKSGIRIAQSKEDKEDQEPIRCPVPGCD
GQGHITGKYASHRSASGCPLAAKRQKDGYLNGSQFSWKSVKTEGMSCPTPGCDGSGHVSG
SFLTHRSLSGCPRATSAMKKAKLSGEQMLTIKQRASNGIENDEEIKQLDEEIKELNESNS
QMEADMIKLRTQITTMESNLKTIEEENKVIEQQNESLLHELANLSQSLIHSLANIQLPHM
DPINEQNFDAYVTTLTEMYTNQDRYQSPENKALLENIKQAVRGIQV

>Pan troglodytes_MYT1L(ENSPTRP_55706)
MEVDTEEKRHRTRSKGVRVPVEPAIQELFSCPTPGCDGSGHVSGKYARHRSVYGCPLAKK
RKTQDKQPQEPAPKRKPFAVKADSSSVDECDDSDGTEDMDEKEEDEGEEYSEDNDEPGDE
DEEDEEGDREEEEEIEEEDEDDDEDGEDVEDEEEEEEEEEEEEEEENEDHQMNCHNTRIM
QDTEKDDNNNDEYDNYDELVAKSLLNLGKIAEDAAYRARTESEMNSNTSNSLEDDSDKNE
NLGRKSELSLDLDSDVVRETVDSLKLLAQGHGVVLSENMNDRNYADSMSQQDSRNMNYVM
LGKPMNNGLMEKMVEESDEEVCLSSLECLRNQCFDLARKLSETNPQERNPQQNMNIRQHV
RPEEDFPGRTPDRNYSDMLNLMRLEEQLSPRSRVFASCAKEDGCHERDDDTTSVNSDRSE
EVFDMTKGNLTLLEKAIALETERAKAMREKMAMEAGRRDNMRSYEDQSPRQLPGEDRKPK
SSDSHVKKPYYDPSRTEKKESKCPTPGCDGTGHVTGLYPHHRSLSGCPHKDRVPPEILAM
HESVLKCPTPGCTGRGHVNSNRNSHRSLSGCPIAAAEKLAKAQEKHQSCDVSKSSQASDR
VLRPMCFVKQLEIPQYGYRNNVPTTTPRSNLAKELEKYSKTSFEYNSYDNHTYGKRAIAP
KVQTRDISPKGYDDAKRYCKDPSPSSSSTSSYAPSSSSNLSCGGGSSASSTCSKSSFDYT
HDMEAAHMAATAILNLSTRCREMPQNLSTKPQDLCATRNPDMEVDENGTLDLSMNKQRPR
DSCCPILTPLEPMSPQQQAVMNNRCFQLGEGDCWDLPVDYTKMKPRRIDEDESKDITPED
LDPFQEALEERRYPGEVTIPSPKPKYPQCKESKKDLITLSGCPLADKSIRSMLATSSQEL
KCPTPGCDGSGHITGNYASHRSLSGCPRAKKSGIRIAQSKEDKEDQEPIRCPVPGCDGQG
HITGKYASHRSASGCPLAAKRQKDGYLNGSQFSWKSVKTEGMSCPTPGCDGSGHVSGSFL
THRSLSGCPRATSAMKKAKLSGEQMLTIKQRASNGIENDEEIKQLDEEIKELNESNSQME
ADMIKLRTQITTMESNLKTIEEENKVIEQQNESLLHELANLSQSLIHSLANIQLPHMDPI
NEQNFDAYVTTLTEMYTNQDRYQSPENKALLENIKQAVRGIQV

>Mus musculus_MYT1L(ENSMUSP_58264)
MDVDSEEKRHRTRSKGVRVPVEPAIQELFSCPTPGCDGSGHVSGKYARHRSVYGCPLAKK
RKTQDKQPQEPAPKRKPFAVKADSSSVDECYESDGTEDMDDKEEDDDEEFSEDNDEQGDD
DDEDEVDREDEEEIEEEDDEEDDDDEDGDDVEEEEEDDDEEEEEEEEEEEENEDHQMSCTR
IMQDTDKDDNNNDEYDNYDELVAKSLLNLGKIAEDAAYRARTESEMNSNTSNSLEDDSDK
NENLGRKSELSLDLDSDVVRETVDSLKLLAQGHGVVLSENISDRSYAEGMSQQDSRNMNY
VMLGKPMNNGLMEKMVEESDEEVCLSSLECLRNQCFDLARKLSETNPQDRSQPPNMSVRQ
HVRQEDDFPGRTPDRSYSDMMNLMRLEEQLSPRSRTFSSCAKEDGCHERDDDTTSVNSDR
SEEVFDMTKGNLTLLEKAIALETERAKAMREKMAMDAGRRDNLRSYEDQSPRQLAGEDRK
SKSSDSHVKKPYYGKDPSRTEKRESKCPTPGCDGTGHVTGLYPHHRSLSGCPHKDRVPPE
ILAMHENVLKCPTPGCTGRGHVNSNRNSHRSLSGCPIAAAEKLAKAQEKHQSCDVSKSNQ
ASDRVLRPMCFVKQLEIPQYGYRNNVPTTTPRSNLAKELEKYSKTSFEYNSYDNHTYGKR
AIAPKVQTRDISPKGYDDAKRYCKNASPSSSTTSSYAPSSSSNLSCGGGSSASSTCSKSS
FDYTHDMEAAHMAATAILNLSTRCREMPQNLSTKPQDLCTARNPDMEVDENGTLDLSMNK
QRPRDSCCPVLTPLEPMSPQQQAVMSSRCFQLSEGDCWDLPVDYTKMKPRRVDEDEPKEI
TPEDLDPFQEALEERRYPGEVTIPSPKPKYPQCKESKKDLITLSGCPLADKSIRSMLATS
SQELKCPTPGCDGSGHITGNYASHRSLSGCPRAKKSGIRIAQSKEDKEDQEPIRCPVPGC
DGQGHITGKYASHRSASGCPLAAKRQKDGYLNGSQFSWKSVKTEGMSCPTPGCDGSGHVS
GSFLTHRSLSGCPRATSAMKKAKLSGEQMLTIKQRASNGIENDEEIKQLDEEIKELNESN
SQMEADMIKLRTQITTMESNLKTIEEENKVIEQQNESLLHELANLSQSLIHSLANIQLPH
MDPINEQNFDAYVTTLTEMYTNQDRYQSPENKALLENIKQAVRGIQV

>Equus caballus_MYT1L(ENSECAP_12726)
MEVDAEEKRHRTRSKGVRVPVEPAIQELFSCPTPGCDGSGHVSGKYARHRSVYGCPLAKK
RKTQDKQPQEPAPKRKPFAVKADSSSVDECYESDGTEEDMDEKEEDEEEEYSEDDDEQRD
DDDEEEEVDREEEEEIEEEDDDDDEDGEDVDEEEEEEEEEEEEEEEEEENEDHQMNCHNTR
IMQDAEKDDNNNDEYDNYDELVAKSLLNLGKIAEDAAYRARTESEMNSNTSNSLEDDSDK
NENLGRKSELSLDLDSDVVRETVDSLKLLAQGHGVVLAENMNERNYADTMSQQDNRNMNY
VMLGKPMNNGLMEKMVEESDEEVCLSSLECLRNQCFDLARKLSETNPQERNQQQNMNIRQ
HVRQEDDFPGRTPDRNYSDMMNLMRLEEQLSPRSRTFSSCAKEDGYHERDDDTTSVNSDR
SEEVFDMTKGNLTLLEKAIALETERAKAMREKMAMEAGRRDNMRSYEEQSPRQLPGEERK
PKSSDSHVKKPYYGKDPSRAEKKESKCPTPGCDGTGHVTGLYPHHRSLSGCPHKDRVPPE
ILAMHENVLKCPTPGCTGRGHVNSNRNSHRSLSGCPIAAAEKLAKAQEKHQSCDVSKSNQ
ASDRVLRPMCFVKQLEIPHYGYRNNVPTTTPRSNLAKELEKYSKTSFEYNSYDSHTYGKR
AIAPKVQTRDISPKGYDAKRYCKNASPSSSTTSSYAPSSSSNLSCGGGSSASSTCSKSSF
DYTHDMEAAHMAATAILNLSTRCREMPQNLSTKPQDLCTARNPDMEVDENGTLDLSMNKQ
RPREGCCPILTPLEPMSPQQQAVMNSRCFQLSEGDCWDLPVDYTKMKPRRIDEDDPKEIT
PEDLDPFQEALEERRYPGEVTIPSPKPKYPQCKESKKDLITLSGCPLADKSIRSMLATSS
QELKCPTPGCDGSGHITGNYASHRSLSGCPRAKKSGIRIAQSKEDKEDQEPIRCPVPGCD

GQGHITGKYASHRSASGCPLAAKRQKDGYLNGSQFSWKSVKTEGMSCPTPGCDGSGHVSG
SFLTHRSLSGCPRATSAMKKAKLSGEQMLTIRQRASNGIENDEEIKQLDEEIKELNESNS
QMEADMIKLRTQITTMESNLKTIEEENKVIEQQNESLLHELANLSQSLIHSLANIQLPHM
DPINEQNFDAYVTTLTDMYTNQDRYQSPENKALLENIKQAVRGIQV

>Anolis carolinensis_MYT1L(ENSACAP_5659)
MEVDADEKRHRTRSKGVRVSISLQFPWKPAIQELFSCPTPGCDGSGHVSGKYARHRSVYG
CPLAKKRKTQEKQPQEPAPKRKPFVVKTDNSSVDECYETDGTEEMDDKEEEEEEEEEEDY
SDDNDDHGDDEEEEEEEVEDREEEEIEEEEEEEEEEEEADEVEDEEEEEEEEEEEEEEKET
KAEDHQMNCHNTRMQDAEKDDNNNDEYDNYDELVAKSLLNLGKIAEDAAYRARTESEMNS
NTSNSLEDDSDKNENVGRKSELSLDLDSDVVRETVDSLKLLAQGHGVVLSENINDRNYTD
NMSQHENRNMNYGMLGKPTNNGLTEKMVEESDEEVCLSSLECLRNQCFDLARKLSETTPQ
ERNQQQNMNSRQHARQEEDSQGRTPERNYSDMMNLMRLEEQLSPRSRTFPSCAKEGGCHE
RDDDTTSITSDRSEEVFDMTKGNLTLLEKAIALETERAKAMREKMAMEAGRRDNMRSYEE
HSPRHLSGDDRKSKSSDGHVKKPYYGKDPSRTEKKESKCPTPGCDGTGHVTGLYPHHRSL
SGCPHKDRVPPEILAMHENVLKCPTPGCTGRGHVNSNRNSHRSLSGCPIAAAEKLAKAQE
KHQSCDASKSNQASDRVLRPMCFVKQLEIPQYGYRNNVPTTTPRSNLAKELEKYSKTTFE
YNSYDNHAYGKRAIAPKVQSRDISPKGYDVAAKRYCKNSSPTSSTTSSYAPSSSSNMSCG
GGSSASSTCSKSSFDYTHDMEAAHMAATAILNLSTRCREMPQNLSTKPQDLCSRKNQNPD
MEVDENGTLDLSLNKQRQRDGCCAILTPLEPMSPQQQAVMNNRCYQLNESDCWDLPVDYT
KMKPNTIDEDDSKEMNPEDLDPFQEALEERRYPGEVTIPSPKPKYPQCKENKKDLITLSG
CPLADKSIRSMLATSSQELKCPTPGCDGSGHITGNYASHRSLSGCPRAKKSGIRIVQSKE
DKEDQEPIRCPVPGCDGQGHITGKYASHRSASGCPLATKRQKDGYLNGSQFSWKSVKTEG
MSCPTPGCDGSGHVSGSFLTHRSLSGCPRATSAMKRAKLSGEQMLTIKQRASNGIENDEE
IKQLDEEIKELNESNTQMEADMIKLRTQITTMESNLKTIEEENKVIEQQNESLLHELANL
SQSLIHSLANIQLPHMEPINEQNFDAYVTTLTDMYTNQDRYQSPENKALLENIKQAVRGI
QV

>Xenopus tropicalis_MYT1L(ENSXETP_25129)
MEVESDEKRHRTRSKGVRVPVEPAIQELFSCPTPGCDGSGHVSGKYARHRSVYGCPLAKK
RKTQDNKPQEPAPKRKPFPGKADNSSVDECYETDGTEDMDDSQTRSEEQFHSNHHEQNDL
VKSPIPRKLSDSILEPDFEHETCPIHNQDMERKSENKQKSRTQNEDLGRMSLHPMDCHSS
RILHDTEKDDNNNDEYDNYDELVAKSLLNLGKIAEDAAYRARTESEMNSNTSNSMEDDSD
KNEHVGRKTELSLDLDSDVVRETVDSLKLLAQGHGVVLSDNLNERHFVDNAMQEDTRNMN
YVMLGKPTNNGHAEKMIEESDEEVCLSSLECLRNQCFDLARKLSETNSHDRSQQQHMNMR
MHRQEEDYSDHTHDRNYSDMMNLMRLEEQLSPRSRSFSSCAKEDECIERDDDTTSITSDR
SEEVFDMTKGNLTLLEKAIALESERAKAMRDKMAMEAGRRESMRDCDDHSVRHLSGEDRR
LKTNDGHVKKPCYTKDPSRTEKKESKCPTPGCDGTGHVTGLYPHHRSLSGCPHKDRVPPE
ILAMHENVLKCPTPGCTGRGHVNSNRNSHRSLSGCPIAAAEKLAKAQEKHQSCDASKTNQ
ASDRVLRPMCFVKQLEIPQYGYRNNVSTTTPRSNLAKELEKYSKTTFDYNNYENHAYGKR
AIAPKVQSRDISPKGYDAKRYCKNSSPTSSTTSSYAFDYTHDMEAAHMAATAILNLSTRC
REMPQNLSTKPQDLCSRNPDIDVDENGTLDLSMNKQSHRDGCCTILTPLEPMSPQRQAVM
NSQCYQMNEGDCWDLPVDYTKMKPRRIDEDDSKEINHEDLDPFPDPLEERRYPGEVTIPS
PKPKFPQCKESKKDLITLSGCPLADKSIRSMMATSSQELKCPTPGCDGSGHITGNYASHR
SLSGCPRAKKSGIKIAQSKEDKEDQEPIRCPVPGCDGQGHVTGKYASHRSASGCPLAAKR
QKDGYLNGSQFSWKSGKTEGMSCPTPGCDGSGHISGSFLTHRSLSGCPRATSAMKKARLS
GEEMLTIKQRATNGIENDEEIKQLDEEIKELNESNSQMESDMIKLRTQITTMESNLKTIE
EENKAIEQQNESLLHELANLSQSLINSLANIQLPHMDPINEQNFDAYVTTLTDMYTNQDR
YQSPENKALLENIKQAVRGIQV

>Danio rerio_MYT1L(ENSDARP_127218)
MEVDAAEKRHRTRSKVAVEAAQELFSCPTPGCDGSGHVSGKYARHRSVYGCPLAKKRKTQ
EKPLQEPASKRRPFLTMADGEHAMYENYEAEAMEEGEEREQEEIEEVEEDEEEGEDEEEE
CSDDNEDLGEEEEEEEEGEDDGEVEREDVEEIEQMEEEVEEDEEVVEEDGDDEEQEEDDDE
EEDDDEHDEEEYYGNLSHQQDDNYSSGQSKSNHMIEKDNNNNNNNTNNEEYENYDELVAK
SLLNLGKIAEDAAYQAMTESEMNSSSSNSAGEDDDEDEDDGSEQGCHKGELSVDLDSDVV
RETVDSLKLLAQGHGAVLPDEGYADAGSGDACQANGRGQAEESEEEVCLSSLECLRNQCF
DLARKLSETQPSERPLMEHHMQHHSEQQQPQHLHPHPHPHHPPMPRYDSCQMDQHRPLER
NYSDMVNLMKLEEQLSPASRGYPSSCAQDGDEDTTSVASDRSDEAFDMTKGNLSLLEKAI
ALESERAKVMRDRMASEQMVARRDNQRLHGEHSPRQSSSAEERKARLHHDGAKRAYYPKD
SRGEKKESKCPTPGCDGTGHVTGLYPHHRSLSGCPHKDRVPPEILAMYENVLKCPTPGCS

GRGHVNSNRNSHRSLSGCPIAAAEKMAKVQEKHHSCDGPKSNQASDRVLRPMCFVKQLEI
PEYGYKNNVPTSTPRSNLAKELEKYSKASFDYSNTYENQPHAYGKRSLIPKSHGRDTSPK
GYDAKRYCKTSSPASSTTSSYAPSSSSSLSCGGGGGGGGGGGGGSSASSTCSKSSFDYTH
DMEAAHMAATAILNLSTRCREMPQSLAAKGPELLAQSPDDRDGDDSRALDVGRVGGMGEG
SGGTVLTPLQPMSPQRQALLNSHCYQMSTADCWDLPVDYTKIKRLDDDHKEDDLDPFQDL
LEDQQYSGEVSLPSPKHKYAPCKESKKELLTLSSCQLADKGMRGMMTSNSQDLKCPTPGC
DGSGHITGNYASHRSLSGCPRAKKSGIKIMHSKEDKDDQEPIRCPVPGCDGQGHVTGKYA
SHRSASGCPLAAKRQKDGYVNGAPFSWKSGKTDGMTCPTPGCDGSGHVSGSFLTHRSLSG
CPRATSAMKKARMTGVEMLTIKQRASKGIENDEEIKQLDEEIKDLNESNNQVESDMIKLR
TQITTMETNLKSIEEENKVIEQQNDSLLHELANLSQSLINSLANIQLPHMKPMPLKEAPV
KNYCCLQMPHREPMNEQNFDTYVSTLTDMYTHQDQYQSPENKALLENIKQAVQGIQV

>Takifugu rubripes_MYT1L(ENSTRUP_7999)
SVNGYPLAKKRKRVDRQTPDNYPKRSSYLDDMHNSTMEEWYDTDGTEDMDDREEEEVERV
IEEEDEVERGHEDSEEDYEEEQIEVEQEESEEERVDEEEETVEEVKYDEGEEEEKQQSHG
QEDDEMSSAERVKANGEARSSTGKSGPLTEKDDNNNDEYDNYDELVAKSLLNLGKIAEDA
ANRAMTESEMNSNLSNTEEDEEDDEDEEEDDDDLRLNADSDVVRETVDSLKLLAQGHGAM
LAENVDGQEFADDAAKTVNKNDYAHNGANSHTVGNKQVMSICSGQIEKSDEEVCLSSLEC
LRNQCFDLARKLSETKTSGDSNQPTQHSSGDYQHSQTNGRSLNRSYSDMDNLMKLEEQLS
PRSKAFTSCAQDGRCHKNFDEDTASVASDRSEEMFDMTKGNLSLLEKAIALESERAKAMR
DKMAADPTRRDRGRYSHGDNGSRQSYGVDRKSRFPDGMKKPFYLKDASRVDKKESRCPTP
GCDGTGHVTGLYPHHRSLSGCPHKDRVPPENKVVAMYENVLKCPTPGCTGRGHVNSNRNS
HRSLSGCPIAAAEKLAKTKEKHQTCDGTKSNQGSDGVLRPTCFAYVVFKQVDYPKYDYKN
NVSTSTPRSNLAKELEKYSKTNFDYCAFDGSTNHQAYGKRAIAPKVDGQRDTSPKGYNER
IIFAWNISLSGPDNASKQQPEISSSLSCGGGRGASSTCSKSSFDYSHDMEAAHMAATAIL
NLSTRCREMPQSLGGKPQDLCSQAKSPGLDVDENGTLDLSLSKRLCSGGSGSSGAGDSVL
MPLEPMSPLRQATLLGSRCYGMGNAASCWDLPVDYSKIKHVDEDENEPTGYDDLDAFKDL
LEDRSYTTDVRIPSPKPKFGQCKENKKDLITLSGCPLADKSIRSMLANNAQELKPRCPTP
GCDGSGHITGNYASHRSLSGCPRARKNGIKIIHSKENKEDQEPIRCPVPGCDGQGHVTGK
YVSHRSASGCPIAAKRQKDGYLNGIQFTWKSGKTEGMSCPTPGCDGSGHVSGSFLTHRSL
SGCPRATSAMRKARLSGVEMLTIKQQCASNGLEHEDDVKQLDEEIKDLSESNPHVEADMI
KLRTQITTMESNLKSIEEENKVIEQQNESLLHELANLSHSLINSLANAQLPHMEPISEQN
FEDYVTTLTDIYTNQDQYQIPENKALLENINQTVQGIQA

>Tetraodon nigroviridis_MYT1La(ENSTNIP_13634)
MEMNVAKRRHRTRSKGLRAAAEAATQELFSCPTPGCDGSGHVSGKYARHRSVYGCPLAKK
RKALEKQPLEPAAKRRPFLTSSLDVGAYASYEGEVMEVGSSDKEPREVEEERDGEDEMED
EFSEDKEQRDAEKRGSEDERKRRCFDQPPTPVCDVTEGAEAEQSGRNNVGSPQTWKVSDL
FLLSWQQKTTTTTADNPSSSRLLVAKSLLNLGKIAEDAAYQAMTESEMNSNSSNSTGEDE
DEDEDEDGSDQGDGKGELSVDLDSDVVRETVDSLKLLAQGHGAVLPEDGYPEGSVVEEGR
SNGRDAEEDVCLSGLECLRSQCFDLAQKLSQSQAPERGAVHHQLQLPSEQQMLHHLNSYD
GCHQGEPRSLERNFSDMANLMKLEEQLSPASRGYPTSCSQEGDEDTTSVASDRSDETFDM
AKGNLSLLEKAIALESERAKVMRDRMSPENSFLRRDHHQHRGHGEHSPRVNASEERRSRL
HQDGLKRAYYPKEYSRGEKKESKCPTPGCDGTGHVTGLYPHHRSLSGCPHKDRVPPEIVA
MYENVLKCPTPGCSGRGHVNSNRNSHRSLSGCPIAAAEKMAKVHEKSQTVEVGSKSSQTS
DRVLRPMCFVKQLEIPQNGYKNNIPTSTPRSNLAKELEKYSKTSFDYSAYDGQHAYGKRA
PAAKAPHGREPSPKAYEGAKRYCKTSSPASSTTSSYAPSSSSLSCGGGAGSAGGGGGSSA
SSTCSKSSFDYTHDMEAAHMAATAILNLSTRCRELPHALGGKPQELLPQSPVGDLDDSGP
AEPQPGGPEGSGTVLTPLQPMSPQRQALLSSRCYQLSEADCWDLPVDYTKIKRLGEEPEH
KEVTGDELDPFRQLLEEQRYPAEVPMPSPKHKYPACKEGKKELLTLSSCQLADKSIRGMM
TPNSQELKCPTPGCDGSGHITGNYASHRSLSGCPRAKKSGIKILHKEDKEDQEPIKCPVP
GCDGQGHVTGKYASHRSASGCPLAAKRQKDSYVNGSQYVWKSGKTDGMSCPTPGCDGSGH
VSGSFLTHRSLSGCPRATTAMKKARMSSVDMLSIKQRASKGIENDEEIKQLDEEIKENES
NSQVESDMIKLRTQITTMESNLKSMEEENKVIEQQNDSLLHELANLSQSLINSLNIQLPH
MEPMNEQNFDTYVSTLTDMYTHQDQYQSPENKALLENIRQAVQGIQV

>Tetraodon nigroviridis_MYT1Lb(ENSTNIP_11126)
SVYGCPQAKKRKRVDRQTPENYPKRSTYLDDMHSSTMEECYETDGTEDMDDREEEELERV
LEGYMDYNGHVVQQKKPNVEEWLAMEVEPLHGMYHCQTEKVEEDDEMSSTEEVETTSEAW
GSAAKSGPLTEKDDNNNDEYDNYDELVAKSLLNLGKIAEDAANRAMTESEMNSNRENEEE
ENRDEDETQRSGPRLNADSDVVRETVDSLKLLAQGHGAVLAENEDGQEIMDDAAEAAIKN

DCAHSGAISHTVGNKQVKSACNGQIEKSDEEVCLSSLECLRNQCFDLAPEKNHVSCGDSN
QPPQHSYGDYQHGQENGRSLNRNYSDMDNLMKLEEQLSPCSKAFSCAQDGRCHQDFDEDT
ASVTSDRSEEVFDMTKGNLSLLEKAIALESERAKAMRDKMSADPTRRDRVRYNHGDNGPR
HAYGVERKARFPDGMKKPFYLKDASRVDKKESRCPTPGCDGTGHVTGLYPHHRSLSGCPH
KDRVPPETLWFVGVSLACSVKFCCEEKNCNLENVIFLNLSGCPIAAAEKLAKTKEKHQTC
DGSKSNQASDRVLRSTVCQKQVDCLRYGYKNNVSTSTPRSNLAKELEKYSKTNFDYSAFD
GSSNHQVYGKRAIAPKVDGQRDASPKGYDGTTKRYCKNSSSASSTTSTYASCGGGAGGTG
GRGGGSSSASSTCSKSSFDYSHDMEAAHMAATAILNLSTRCREMPQSMGGKPQELCSQSP
GLDVDENGTLDLSLSKRLCGGGSGSSGGGDSVLTPLEPMSPLRQASLLGSRCYGMGNAAN
CWDLPVDYTKIKHIDEDENEIKECSPQDDLDAFNDLLEDRSYTADVGMPSPKSKYGQCKE
SKKDLITLSGCPLADKSIRSMLANNAQELKCPTPGCDGSGHITGNYASHRSLSGCPRARK
NGIKIIHSKENKEDQEPIRCPVPGCDGQGHVTGKYVSHRSASGCPIAAKRQKDGYLNGIQ
FTWKSGKTEGMSCPTPGCDGSGHVSGSFLTHRRFTGTLQQDQVLHRQKFKLRILVNSNNQ
TSAGLEHEDDVKQLDEEMKDLSESNPHVEADMIKLRTQITTMESNLKSIQEENKVIEQQN
ESLLHELANLSQSLINSLANAQLPHMEPISEQNFEDYVTTLTDIYTNQEQYQIPENKTLL
ENINQAVQGIQV

>Gasterosteus aculeatus_MYT1La(ENSGACP_15823)
MEVNAARKRHRTRSKGVRAAVEAATQELFSCPTPGCDGGGHVSGSYPGPRSVYGCPLAKK
RKALEKQPLEPAAKRRPFLTPCNGGEEDAGAYARYQNRALRWGERVRQWEESDLKTEESL
YNTKRARMQWMGPLISPLGTASSGSAESQNQMDGASLGPMSRPASSSEPAVGEEEVMRAA
ETKYRAVRRSCIWMSERFLSLFPPTAENHYKPAGQSKLLLIQKDDNNNHSCTIGAGNAPG
EDYENYDELVAKSLLNLGKIAEDAAYQALTESEMNSNSSNSGGEDEDDEEYKGELSVDLD
SDVVRETVDSLKLLAQGHGAMLPEDGYPDGGMLGDAGHHANGRPGVGVRVQADESEEEVC
LSSLECLRNQCFDLARKLSETQACDRPALHALHHQLQMPADHQVVHHLNSHFWSHRRLRY
DSCQQSAQEEHRSLERSYSDMVNLMKLEEQLSPGSRGYPTSCSQEGDEDTTSVASDRSDE
TFDMAKGNLSLLEKAIALESERAKVMRDRIASEHSLPRRRGHGEHSSRLSCAGEERKSRM
HHDGLKRAFYPKDSSRGDKKESKCPTPGCDGTGHVTGLYPHHRSLSGCPHKDRVPPEILA
MYENVLKCPTPGCSGRGHVNSNRNSHRSLSGCPIAAADKMSKVHEKSIPTESGSKTNQTS
DRVLRPMCFVKQLEIPQYGYKNNVPTSTPRSNLAKELEKYSKTSYDYGGYDGQHGGGYGK
RGLATKLPHGRDTSPKGYDAKRYCKTSSPASSTTSSYAPSSSSSLSCSASSTCSKSSFDY
THDMEAAHMAATAILNLSTRCRELPHALGGKPQDLLTQSPVGDLEDGGPLDVGGQPGGPE
GSGTVLTPLQPMSPQRQALLSSRCYQLSEADCWDLPVDYTKIKRLGGEEEHKEADQLDPF
QELLEEQRYPAEAPVHSPKHHKYPACKEGKKELLTLSSCQLADKNIRGIMTTNSQDLRCP
TPGCDGSGHITGNYASHRSLSGCPRAKKSGIKILHSKEDKDDQEPIKCPVPGCDGQGHVT
GKYASHRSASGCPLAAKRQKDNYVNGSQFVWKSGKTDGMSCPTPGCDGSGHVSGSFLTHR
SLSGCPRATSAMKKARMSGVEMLTIKQRASKGIENDEEIKQLDEEIKDLNESNTQVESDM
IKLRTQITTMETNLKSIEEENKVIEQQNDSLLHELANLSQSLINSLANIQLPHMKPAAQK
EAPVKHSCCVQMPPREPMNEQNFDTYVSTLTDMYTHQDQYQSPENKALLENIKQAVQGIQ
V

>Gasterosteus aculeatus_MYT1Lb(ENSGACP_17522)
DFDEDTASVTSDRSEEVFDMTKGNLSLLEKAIALESERTKAMRDKMAAEAARRDGGYGVE
RKARLPDGMKKPFYHKDASRADKKESRCPTPGCDGTGHVTGLYPHHRSLSGCPHKDRVPP
EIVAMYENVLKCPTPGCTGRGHVNSNRNSHRSLSGCPIAAAEKLAKAQEKHQGCDVPKTS
QASDRVLSLMCSVKQLDCPQYGYKNNVSSSTPRSNLAKELEKYSKTSFDYSAFDGSSNHP
AYGKRAIAPKVHGQRDTSPKGYDDSDFQRTRDVRALFISSFALCSLASGAGIPFSPFVCS
FHPSPPLLIIISLKNNLTQTHPPQHTSTHTRKPHPTVQSPGLDVDENGTLDLSMSKRLCS
GGDSVLTPLEPMSPQRQAALLGSRCYGMGDAADCWDLPVDYTKIKHIDEDEKESASQPAL
HAPTRSQADDLDPFHDLLEERSYTADVSIPSPKPKYVHCKEGKKDLITCPTSRCDGSGHM
TSNLAFDFLSSCLSGCPLADKSIRSMLANNAQELKCPTPGCDGSGHITGNYASHRSLSGC
PRARKSGIKIIHSKENKEDQEPIRCPVPGCDGQGHVTGKYASHRSASGCPIAAKRQKDGY
LNGIQFTWKSGKTEGMSCPTPGCDGSGHVSGSFLTHRSLSGCPRATSAMRKARLSGVELL
TIKQQCAGNGLEHEEDIKQLDADIKDLSESNSQVEADMIKLRTQITTMESSMKSIEEENK
VIEQQNESLLHKLSHLSQSLTNSLANVQLPHMEPISEQNFDAYVTTLSDMYSNQDPYQSP
ENKVLLDNIKQAVQGIQV

>Oryzias latipes_MYT1La(ENSORLP_22342)
SVYGCPLAKKRKSLDIEPLETSPKRSTLLDDMDNSTMEECYDTDGTKSLDTPSHSNEWED
KSWSYVKIGGKSQVDEGGNAKEKLEVLQKPEINDWWLASVASIPADFEKDNQLSPGDSTS
EGGGGDGYSKLGPAMEKDDNNNDEYENYDELVAKSLLNLGKIAEDAANRAMTESEMNSNS

PNSAEGELQRGDLSLDVESDVVRETVDSLKLLAQGHGAVLSDSLDGQDFEGSARPGDQTN
YSHNGGNGQMKTTNNGKIEGSDEEVCLSSLECLRNQCFDLARKLSETKSIDRNGPSQQNH
YSCGDPSQQPQHHGYGDFHNSQDSRRTLDRNYSDMDNLMKLEEQLSPRSKAFSSCAQDER
YHTSHRDFDEDTASITSDRSEEVFDMTKGNLSLLEKAIALESERAKAMRDKLAAEAARRD
GVRYAHEDHGPRHGYGVERKARLPDGMKKPFFHKGADPSRADKKESRCPTPGCDGTGHVT
GLYPHHRSLSGCPHKDRVPPEIVAMYENVLKCPTPGCTGRGHVNSNRNSHRSLSGCPIAA
AEKLAKAQEKHQSCDGPKSNQSSDRVLRPMCFVKQLDYPQYGYKNNVSTSTPRSNLAKEL
EKYSKTSFDYSAFDSTNNHHVYGKRAIAPKAHGQRDASPKGHDAFAAKRYCKNSSSASST
TSTYAPKSSFDYSHDMEAAHMAATAILNLSTRCREMPHGIGGKPQDLCSQSPGLDVDENG
TLDLSMSKRLCGGGAGDAVLTPLEPMSPQRQAALLGSRCYGMGDAADCWDLPVDYTKIKH
IDEDEKEKYLQTDDLDPFQDLLDDRSYTTDVNMPSPKPKYVQCKETKKDLITLSGCPLAD
KSIRSMLANNAQELKCPTPGCDGSGHITGNYASHRSLSGCPRARKSGIKIIHSKENKEDQ
EPIRCPVPGCDGQGHVTGKYASHRSASGCPIAAKRQKDGYLNGIQFTWKSGKTEGMSCPT
PGCDGSGHVSGSFLTHRSLSGCPRATSAMRKARLSGVEMLTIKQQHASNVLDHEEEIKQL
DEEIKDLSESNSQVEADMIKLRTQITTMESSLKSLEEENKVIEQQNESLLHELANLSQSL
INSLVNAHLPHMESLSEQNFDAYVTTLTDMYTNQDQYQSVENKTLLENIKQAVQGIQV

>Oryzias latipes_MYT1Lb(ENSORLP_22706)
MEGNVTEKRHRTRSKGVRVAVEAVTQELFSCPTLGCDGSGHVSGKYARHRSIYGCPLAKK
RKTLDKQPLEPAAKRRPFLSPRAGQEIDENAGSFSSYQNDAMKTGRQREQMNCQQSKTEQ
NDGESHEDVCSVLNILMGLHDQGRDSESIGRRSRGLEEDQRRFLDAVKENMKVADVRGED
AEDRDETLLTRDAEAKYSFRFFFSVFLSAVKQFKPNGQSKLGPVEKDNNNNSCGPGNSTG
EDYENYDELVAKSLLNLGKIAADAAYQAMTESEMNSNSSNSAGEDDEDDASEQGDRKGEL
SVDLDSDVVRETVDSLKLLAQGHGAILPEDEYPHAAMMNDGKHANGAPCMRVKAQDSGEE
VCLNSLECLRNQCFDLARKLSETQPSDRPILHSLQNQSINPADQQGVHHLNSSPEIFLFP
FIRYDSSHQNALEEPRLLDRRYSDMVNLMKLEEQLSPASRGYPTSQDGDEDTTSIASDHS
DETFDMAKGNLSLLEKAIALESERAKVMRDRMASEHSMPRRDQHYHRSHGEHSPRLSSSA
EERKSRMQQDGLKRAYYPKDSSRGEKKESKCPTPGCDGTGHVTGLYPHHRSLSGCPHKDR
VPPEIQVLAMYENVLKCPTPGCSGRGHVNSNRNSHRSLSGCPIAAAEKMAKVHEKSHSAD
SSKTNQTSDRVLRPMCFVKQLEIPQYGYKNNVPTSTPRSNLTKELEKYSKTSYDYGGYDC
QQGSYGKRGSTTKSQHGRDTSPKGYDAVKFVLTSRLRWSKMSQLSEDSLNASVRDGRNAA
AKLTGTGSASTHPLQFPPSYFDYTHDMEAAHMAATAILNLSTRCRELPNALGGKPQDLLS
QSPVGDLDDGSELDLEGQPGGPEGSGTVLTPLQPMSPQRHALLSSRCYQLSDSDCWDLPV
DYTKMKRLGDEHKESDELDPFQELLEEQRYPAEVTTPSSKHHKYSPCKESKKELITLSSC
QLIDKNIRNMLPTNSQELRCPTPGCDGSGHITGNYASHRSLSGCPRAKKSGIKILQSKED
KDEQEPIKCPVPGCDGQGHVTGKYASHRSASGCPLAAKRQKDSYVNGSQFVWKSGKTDGM
TCPTPGCDGSGHVSGSFLTHRSLSGCPRATSAMKKSRMSGVEMLTIKQRASKGIENDEEI
KQLDEEIKDLNESNNQVESDMIKLRTQITTMETNLKSMEEENKAVEQQNDSLLHELANLS
QSLINSLANIQLPHMRPLPQKEAPVKHSCCLQMPPREPMNEQNFDTYVSTLTDMYTHQEM
VKTSVCVSLLQNPGLSLEGLLC

>Homo sapiens_ST18(ENSP_276480)
MDAEAEDKTLRTRSKGTEVPMDSLIQELSVAYDCSMAKKRTAEDQALGVPVNKRKSLLMK
PRHYSPKADCQEDRSDRTEDDGPLETHGHSTAEEIMIKPMDESLLSTAQENSSRKEDRYS
CYQELMVKSLMHLGKFEKNVSVQTVSENLNDSGIQSLKAESDEADECFLIHSDDGRDKID
DSQPPFCSSDDNESNSESAENGWDSGSNFSEETKPPRVPKYVLTDHKKDLLEVPEIKTEG
DKFIPCENRCDSETERKDPQNALAEPLDGNAQPSFPDVEEEDSESLAVMTEEGSDLEKAK
GNLSLLEQAIALQAERGCVFHNTYKELDRFLLEHLAGERRQTKVIDMGGRQIFNNKHSPR
PEKRETKCPIPGCDGTGHVTGLYPHHRSLSGCPHKVRVPLEILAMHENVLKCPTPGCTGR
GHVNSNRNTHRSLSGCPIAAAEKLAMSQDKNQLDSPQTGQCPDQAHRTSLVKQIEFNFPS
QAITSPRATVSKEQEKFGKVPFDYASFDAQVFGKRPLIQTVQGRKTPPFPESKHFPNPVK
FPNRLPSAGAHTQSPGRASSYSYGQCSEDTHIAAAAAILNLSTRCREATDILSNKPQSLH
AKGAEIEVDENGTLDLSMKKNRILDKSAPLTSSNTSIPTPSSSPFKTSSILVNAAFYQAL
CDQEGWDTPINYSKTHGKTEEEKEKDPVSSLENLEEKKFPGEASIPSPKPKLHARDLKKE
LITCPTPGCDGSGHVTGNYASHRSVSGCPLADKTLKSLMAANSQELKCPTPGCDGSGHVT
GNYASHRSLSGCPRARKGGVKMTPTKEEKEDPELKCPVIGCDGQGHISGKYTSHRTASGC
PLAAKRQKENPLNGASLSWKLNKQELPHCPLPGCNGLGHVNNVFVTHRSLSGCPLNAQVI
KKGKVSEELMTIKLKATGGIESDEEIRHLDEEIKELNESNLKIEADMMKLQTQITSMESN
LKTIEEENKLIEQNNESLLKELAGLSQALISSLADIQLPQMGPISEQNFEAYVNTLTDMY
SNLERDYSPECKALLESIKQAVKGIHV

>Gorilla gorilla_ST18(ENSGGOP_1207)
MDAEAEDKTLRTRSKGTEVPMDSLIQELSVAYDCSMAKKRRAEDQALGIPVNKRKSLLMK
PRHYSPNADCQEDRSDRTEDDGPLETHDHSTAEEIMIKPMDESLLSTAQENSSRKEDRYS
CYQELMVKSLMHLGKFEKNVSVQTISENLNDSGIQSLKAESDEADECFLIHSDDGRDKID
DSQPPFCSSDDNESNSESAENGWDSGSNFSEETKPPRVPKYVLTDHKKDLLEVPEIKTEG
DKFIPCENRCDSETERNDPQNALAEPLDGNAQPSFPDVEEEDSESLAVMTEEGSDLEKAK
GNLSLLEQAIALQAERGCVFHNTYKELDRFLLEHLAGERRQTKVIDMGGRQIFNNKHSPR
PEKRETKCPIPGCDGTGHVTGLYPHHRSLSGCPHKVRVPLEILAMHENVLKCPTPGCTGR
GHVNSNRNTHRSLSGCPIAAAEKLAMSQDKNQLDSPQTGQCPDQAHRTSLVKQIEFNFPS
QAITSPRATVSKEQEKFGKVPFDYASFDAQVFGKRPLIQTVQGRKTPPFPESKHFSNPVK
FPNRLPSAGAHTQSPGRASSYSYGQCSEDTHIAAAAAILNLSTRCREATDILSNKPQSLH
AKGAEIEVDENGTLDLSMKKNRILDKSAPLTSSNTSIPTPSSSPFKTSSILVNAAFYQAL
CDQEGWDTPINYSKTHGKTEEDKEKDPVSSLENLEEKKFPGEASIPSPKPKLHARDLKKE
LITCPTPGCDGSGHVTGNYASHRSVSGCPLADKTLKSLMAANSQELKCPTPGCDGSGHVT
GNYASHRSLSGCPRARKGGVKMTPTKEEKEDPELKCPVIGCDGQGHISGKYTSHRTASGC
PLAAKRQKENPLNGASLSWKLNKQELPHCPLPGCNGLGHVNNVFVTHRSLSGCPLNAQVI
KKGKVSEELMTIKLKATGGIESDEEIRHLDEEIKELNESNLKIEADMMKLQTQITSMESN
LKTIEEENKLIEQNNESLLKELAGLSQALISSLADIQLPQMGPISEQNFEAYVNTLTDMY
SNLERDYSPECKALLESIKQAVKGIHV

>Mus musculus_ST18(ENSMUSP_131417)
MDAEVEDKTLHTLSKGTEVPMDSLIPELRVPYDCSMAKKRRAEEQASGVPINKRKSLLMK
PRHYSPDMGCKESPDNRNEDDGLLETNDHATADEIMVKSMDETLHLPAQDSSLQKKDQYT
CYPELMVKSLVHLGKFEESESVQTTCENLNGSSIQSLKAESDEAHEGSMVHSDNGRDKVH
HSQPPFCSSGDSESDSDSAENGWGNGSNSSEDTDTHKGPKHKLTYNRKDLLEVPEIKAED
DKFIPCENRCDSDTDGRDPQNSHMEPLVVKAQPSFPEVEEGESLATVTEEPAEVEKAKGN
LSLLEQAIALQAERGSVFHHTYKELDRFFLDHLARERRQPRVTDANGRQIFTNKHSPRPE
RREAKCPIPGCDGTGHVTGLYPHHRSLSGCPHKVRVPLEILAMHENVLKCPTPGCTGRGH
VNSNRNTHRSLSGCPIAAAEKLAMTQDKSQLDSSQTGQCPEQAHRVNLVKQIEFNFRSHA
ITSPRASASKEQEKFGKVPFDYASFDAQVFGKRPLLQTGQGQKAPPFPESKHFSNPVKFP
NGLPSAGAHTQSTVRASSYGHGQYSEDTHIAAAAAILNLSTRCREATDILSNKPQSLRAK
GAEIEVDENGTLDLSMKKNRIHDKSIPPTSSPTTITTPSSSPFNASSLLVNAAFYQALSD
QEGWNVPINYSKSHGKTEEEKEKDPVNFLENLEEKKFAGEASIPSPKPKLHTRDLKKELI
TCPTPGCDGSGHVTGNYASHRSVSGCPLADKTLKSLMAANSQELKCPTPGCDGSGHVTGN
YASHRSLSGCPRARKGGIKMTPTKEEKEDSELRCPVIGCDGQGHISGKYTSHRTASGCPL
AAKRQKENPLNGAPLSWKLNKQELPHCPLPGCNGLGHVNNVFVTHRSLSGCPLNAQAIKK
VKVSEELMTIKLKATGGIDGDEEIRHLDEEIKELNESNLKIEADMMKLQTQITSMESNLK
TIEEENKLIEQSNESLLKELAGLSQALISSLADIQLPQMGPINEQNFEAYVNTLTDMYSN
LEQDYSPECKALLESIKQAVKGIHV

>Rattus norvegicus_ST18(ENSRNOP_56380)
MDAEVEDKALHTLSKGTEVPMDSLIPELRVAYDCSMAKKRRAEEQALGVPVNKRKSLLMK
PRHYSPDMDCKENPDNRNEDDGLETNDHSTADEIVVKPMDKTLHLPAQESSLPKEDQYAC
YPELMVKSLMHLGKFEESESVQTVGENLNGNGIQSLKAECDEANECFMVHSDDGRDKVHH
SQPPFCSSGDSESDSDSTENGWGSGSNSSEDTDTHKGPKRKLTYNRKDLLEVPEIKAEDD
KFIPCENRCDSDTSGRDPQNSHMEPLAVKVQPSFPEVEESESLATVIAESAEVEKAKGSL
SLLEQAIALQAERGSVFHHTYKELDRFLLDHLARQRRQPKVTDASGRQIFNNKHSPRPER
REAKCPIPGCDGTGHVTGLYPHHRSLSGCPHKVRVPLEILAMHENVLKCPTPGCTGRGHV
NSNRNTHRSLSGCPIAAAEKLAMTQDKSQLDSSQTGQGPEQAHRVNLVKQIEFNFRSQAI
TSPRASASKEQEKFGKVPFDYASFDAQVFGKRPLLQTGQGQKAPPFPESKHFSNPVKFSN
GLPSAGAHTQSTVRASSYGHGQYSEDTHIAAAAAILNLSTRCREATDILSNKPQSLRAKG
AEIEVDENGTLDLSMKKHRILDKSIPPTSSHTTIATPSSSPFKASSLLVNAAFYQALCDQ
EGWNVPINYSKSHGKTEEEKEKDPVNSLENLEEKKFAGEASIPSPKPKLHTRDLKKELIT
CPTPGCDGSGHVTGNYASHRSVSGCPLADKTLKSLMAANSQELKCPTPGCDGSGHVTGNY
ASHRSLSGCPRARKGGIKMTPTKEEKEDSELRCPVIGCDGQGHISGKYTSHRTASGCPLA
AKRQKENPLNGTPLSWKLNKQELPHCPLPGCNGLGHVNNVFVTHRSLSGCPLNAQAIKKV
KVSEELMTIKLKATGGIEGDEEIRHLDEEIKELNESNLKIEADMMKLQTQITSMESNLKT
IEEENKLIEQSNESLLKELAGLSQALISSLADIQLPQMGPINEQNFEAYVNTLTDMYSNL
ERDYSPECKALLESIKQAVKGIHV

>Felis catus_ST18(ENSFCAP_7935)

LNHYSAAYDCSMAKKRRAAEQALGVPVNKRKSLLMKPRHYSPNVDCREECDDRTEAEEED
GLPEASDHTASEETMIKPMDEHLHSTAQENSNGKEDRYGCYRELVVKSLMHLGKFENHVS
VQTVSENLNGSGLQCAQAESDEADEHFVVHSDDGKEKINDPQLRFCSSDDSESNSESAEN
GWDSGSNLSEEAKPHRVPKYILVDDGKDLLGAPEIKTEGDKFISCENTCDPETDRRDPQN
TRVEPLDGTGQPSCAGVEGDGNGSLAAMREESLDLEKAKGSLSVLEQAIALQAERGCVFH
NTYKELDRFLLEHLAGERRQTKVIDMGGRQILNSKHSPRPEKRETKCPIPGCDGTGHVTG
LYPHHRSLSGCPHKVRVPLEILAMHENVLKCPTPGCTGRGHVNSNRNTHRSLSGCPIAAA
EKLAMSQDKSQLDSPKSGQCPEQVHRTSLVKQIEFNFRSQAITSPRATVSKEQEKFGKVP
FDYASFDAQVFGKRPLVQTGQGRKTPPFPESKHFSNPGKFPNRLPSAGAHTQSPCRASSY
GYGQCSEDTHIAATAAILNLSTRCRETADILSNKPQSLHAKGAEIEVDENGTLDLSLKKN
RILDKAAPLTSSNTSIPTPSSSPFKTSSILVNAAFYQALCDQEGWDTPINYSKTHGKTEE
EKEKDPASSLENLEEKKFPGEASIPSPKPKLHARDLKKELITCPTPGCDGSGHVTGNYAS
HRSVSGCPLADKTLKSLMAANSQELKCPTPGCDGSGHVTGNYASHRSLSGCPRARKGGVK
MTPTKEEKEDPELKCPVIGCDGQGHISGKYTSHRTASGCPLAAKRQKENPVNGAPLSWKL
NKQELPHCPLPGCNGLGHVNNVFVTHRSLSGCPLNAQAIKKGKVSEELMTIKLKATGGIE
SDEEIRHLDEEIKELNESNLKIEADMMKLQTQITSMESNLKTIEEENRLIEQNNESLLKE
LAGLSQALISSLADIQLPQMGPISEQNFEAYVNTLTDMYSNLERDSSPECKALLESIKQA
VKGIHV

>Dasypus novemcinctus_ST18(ENSDNOP_7514)
SKQLFLKPCDLSSLDTFAVYDCSMAKKRRMDEQALGVPVNKRKSLLMKPQHYSPNMDCRE
ENDDRAEAEDSGLLATNENGTREEIMIKPMDESLHSAAQEISSRKEGRYASYQELVVKSL
MHVGKFEKDVSIQTTNENLNDSGIQSLKAESDEADECFMIHSDEGTVKVSDGGLRRCSSA
DNDNDSNSGSAETGWDSGSNFSEETKPPRVPKYILVDDRKDLVEVPEIKTEGEKFVPCKN
MCDSETERRDLQNAPVEPLGSQGQSSFSENEDRGREGLSAVTEEAVDVGKAKGNLSLLEQ
AIALQAERGRAFHHTYKELDRFLLEHLAGERRHAKVIDMGGRQIFTSKHSPRPEKRETKC
PIPGCDGTGHVTGLYPHHRSLSGCPHKVRVPLEILAMHENVLKCPTPGCTGRGHVNSNRN
THRRNSLSGCPIAAAEKLAMSQDKNLLDSSKTGQCPDQVHRTSLVKQIEFNFRSQAITPP
RATMSKEQEKFGKVPFDYASFDAQVFGKRPLTQTGQGRKTPPFPESKHFSSPVKCPHRLP
GPGAHAPSPCRASSYGHGQCSEDAHIAAAAAILNLSTRCREAAEILSNKPQSLPAKGADI
EVDENGTLDLSMKKNRILDKAAPLTSSNTSIPTLSSSPFKTSSLLVNAAFYQALCVQEGW
DVPVNFSKAHGKAEEEKEKDSVNSPENLEEKKFPGEASIPSPKPKLHARDLKKELITCPT
PGCDGSGHVTGNYASHRSVSGCPLADKTLKSLMAANSQELKCPTPGCDGSGHVTGNYASH
RSLSGCPRARKGGVKMTPTKEEKEDPELKCPVMGCDGQGHISGKYTSHRTASGCPLAAKR
QKESPLNGAPLAWKLNKQELPHCPLPGCNGLGHVNNVFVTHRSLSGCPLNAQAIKKGKVS
EELMTIKLKATGGIESDEEIRHLDEEIKELNESNLKIEADMMKLQTQITSMESNLKTIEE
ENKLIEQNNESLLKELAGLSQALISSLADIQLPQMGPISEQNFEAYVNTLTEMYSNLERD
YSPECKALLESIKQAVKGIHV

>Monodelphis domestica_ST18(ENSMODP_12044)
MSYHGNYLSKTQLLVHSSAHKGRETYKFWKQKKMDADAEDKRLHTRSKGTKVQVDSLVQE
LSAAYGCPMAKKRKTEEPALGVPVNKRKSLLMKPRHYNPSVECKEENEDQSETEEDDCLL
EMNGHPTAEENLVKPTEGILHSSGKESFNRNKDSYTNYQDLVVKSLMHLGKLDQDASIQT
LTENLNDSGIQSLKTESDEVDDCFMIHSAECKAKINDSNLRYCSSDDNESHSEIAENGWD
SGSNFSEETPEKSHVTQTYIVSNNQEESLEAPEIKTEVENFVPCEKVCDSEMEIRESQTS
PMESLESRAQNPLPEGDEDDTECLSITTEESVDLDKTKGHLSLLEQAIALQAEQGHVFHS
TYKELDRFLLEHLAGERRHTKVIDMGGRQVFNSKHSPRPEKRETKCPIPGCDGTGHVTGL
YPHHRSLSGCPHKVRVPLEILAMHENVLKCPTPGCTGRGHVNSNRNTHRSLSGCPIAAAE
KLAMSQDKNQLDSPKTGQCPDQVHRTSLVKQLEFSQFSFRSSQTVTSPRATITKEQEKFG
KVPFDYASFDAQVFGKRTIVPTIQGQNPSQLPESKHFSNPVKLSNRLPSASAHTQSPCRA
SSYSYGQCSEDTHMAAAAAILNLSTRCREAAEILSNKPQSLSAKVAEIQVDENGTLDLSM
KKNRILDKSTPLNSPHISIPTSSASPFKTSSLLVNAAFYQALCEQEGWDTPINYSKTHGK
KEEEKEKDPVSSPENLEEKKYPGEVSIPSPKPKLHARELKKELITCPTPGCDGSGHVTGN
YASHRSVSGCPLADKTLKSLMAANSQELKCPTPGCDGSGHVTGNYASHRSLSGCPRARKG
GVKVTPTKEDKEDPELKCPVLGCDGQGHISGKYTSHRTASGCPLAAKRQKENPLNGSSLS
WKLNKQELPHCPLPGCNGLGHVNNVFVTHRSLSGCPLNAQAIKKGKVSEELMTIKLKASG
GLESDEEIRHLDEEIKELNESNLKIEADMMKLQTQITSMESNLKTIEEENKLIEQNNESL
LKELAGLSQALISSLADIQLPQMGPISEQNFEAYVNTLTDMYSNLERDYSPECKALLESI
KQAVKGIHV

>Gallus gallus_ST18(ENSGALP_24567)

SSSLKISHLFSRQSYKSAYKCSMAKRKAEEQISGVPVNKRKSLLMKPRHYSPSLECKEEN
EDSIDHDGLRSNHPFSYAEERTAKPCEETPNSGGQENSSQRKDNYTSYQDAMAKSLMNSG
KIAKDAPSQAMAENLNDSGIQSLKTESDDADECYMIDSAEGKEKTSISDPKYCSSEENES
NSEMMDNEWDSSSNFSEETSVKPHLTQKYTVECNQPSILEVPEIKKEEVEFDPCETLCDS
EAELRAPQESHPDPFEKRIQNVFPESEEDDNECLSETTEMSVELDKAKGNLSLLEEAIAL
QAEQGHVFHSTYKELDRFLLEHLAGERRQTKVIDVGGRQLFSNKHSPRPEKRETKCPIPG
CDGTGHVTGLYPHHRSLSGCPHKVRVPLEILAMHENVLKCPTPGCTGRGHVNSNRNTHRS
LSGCPIAAAEKLAMSQEKNQLESPKAGQCHDQTHSTNLTKQLEVVQYNYRTSQPVTSSRT
TLTKEKEKFGKVPFDYASFDAQVFGKRTIAPMVQGRKTPQFLESKHFSNPVKFSNRLPSA
SAHTQSPCHANSYSYGQCSEDTHIAAAAAILNLSTRCREAAEILSNKPQSLSAKGAEIEV
DENGTLDLSMKKNRSQDKVTPLTSSSTVLPTPSSSPFKTSSILVNAAFYQALCEQEGWDT
PINYSKTHGRKEEKEKDPVSSPENMEEKKYPGEVSIPSPKPKLHSRDLKKELITCPTPGC
DGSGHVTGNYASHRSVSGCPLADKTLKSLMAANSQELKCPTPGCDGSGHVTGNYASHRSL
SGCPRARKGGIKVTPTKDEKEDPELKCPVIGCDGQGHISGKYTSHRTASGCPLAAKRQKE
SPVNGSSLSWKLNKQELPHCPLPGCNGLGHVNNVFVTHRSLSGCPLNAQAIKKGKISEEL
MTIKLKASGGIESDEEIRHLDEEIKELNESNLKIEADMMKLQTQITSMETNLKTIEEENK
LIEQNNESLLKELAGLSQALISSLADIQLPQMGPISEQNFEAYVNTLTDMYSNLERDYSP
ECKALLESIKQAVKGIHV

>Xenopus tropicalis_ST18(ENSXETP_12512)
SQEAKIKYYVLLDIVPSVTSAYNCTMSKKRKLLEPCSTAPVNKRKSLLMKPQHYDPREDF
DEDNEDKSDFPSFKLLKKKCGNTVEKSACEKSEKTLHFLIVTVKIPILSNYCCCYQDIMS
NASILRNAAKETIMNPLNDIINDSGTQSVKADTDDAQESYCDQPSKHKDHQAFSADDTNS
NCEITENGWDSSSSFSEEMNANDHSPDEYSKKMKINPTVDFKKRSTDKGDVCETQSNSVI
QATEQEDRKTQHSKLENEEDDKCLSALKEESVDLNNTKGSLTLLEQAIALQAEQGHMVHS
TYKELDMFLLEHLARERRQTKIIDITGRQIYSNKNSPRPEKRETKCPIPGCDGSGHVTGL
YPHHRSLSGCPHKVRVPIEILAMHENVLKCPTPGCNGRGHVNSNRNTHRSLSGCPIAAAE
KLSKTQEKQLTSNCGSNIEPIHRIQVLSPVLHNFQLYGATQPLTTARATIIKEHDRFGKV
PFDYASFDAQVFGKHTIATVVHSQDSNFSGAKQHTSPAKSSNRLHSAHTQSPCHASSYNY
GQCSEDTHIAAAAAILNLSTRCREAAAEILSNKPLSLSAKATDIEVDENGTLDLSMKKNR
FHETAANFGITKPILSTPSLSPFKTSSILVNAAFYKALCEKEGWEAPVNYSKVHGTIGED
KEPDLGDYSDNTDETKYSGDSSLPSPKSKFHSRDMKKDLITCPTPGCDGSGHVTGNYASH
RSVSGCPLADKTLKSLMAANSQELKCPTPGCDGSGHVTGNYASHRSLSGCPRARKNGVKL
FASKEDKEEQELKCPVVGCDGQGHISGKYTSHRSASGCPLAAKRQKESILNGSSLPWKQN
KQELPHCPLPGCNGLGHVNNVFVTHRSLSGCPLNAQALKKGKTSEELMSVKTKSSNGTEN
EDDIRNLDEEIKELNESNLKIEADMMKLQTQISSMETNLKTIEEENKLIEQNNESLLKEL
AGLSQALISSLADIQLPQMEPISEHNFEAYVNTLTEMYSTMEHDYPPECKALLENIKQAV
KGIHV

>Takifugu rubripes_ST18a(ENSTRUP_14767)
MDSEGEEQTLKTYHGSTGYRFNNPTLELSIFPYGPVARKRSAQEGAQGAPVNKRKSLLMK
PRHYSPSEACEEESEDLAAPQDKEEPRNIDTPAGVASPPRDEQCDLIRTVHPLLSRPDPR
FRCCCCLLQPPLTLSSLSARSRLVHFLDLYETLDDNMLQLLLLSQIHATNGSPQCEPDAT
ERLVDEDEEELLNNEEEHQSQDRMSGASSPPSQYDEEREVEWEPLSLAAKVNGSLPETPE
DLTGGNGHIKEEPPSDLSGLMGRAGVFQVESFRYRPLPAEEDSKGEAERPPRLDGWALGL
HEMSRGRVNFSLLEQAIALQTEQRQALHHAYREMDRFLMEQMSHERRHQRMMEMDGRLSY
HGGKESPRADKKDVKCPTPGCDGTGHVTGLYPHHRSLSGCPHKVRVPPEILAMHENVLKC
PTPGCTGRGHVNSNRSTHRSLSGCPIAAAVKVSKPQEESLKCRQTPDRSPRAVGLMKKLE
MNQLNYRLSHPAAALQSTLAKDMDKYSRIRFDYASFDAQVFGKQPLMGANPEQEMSPFSD
SPQQYPGFLAAPGGCLPTSGHHSPSSQFKKDDGLQASAATAAILNLSTRYQKNTEAVAGR
PLAAPAKDMLIEVDENGTLDLSMKKCKENVKTQARTPPGDSLGPSEAAAAKGGSLLIGPA
FYQPLCDRDSWESPLNFSKAHILHDPEEDYDGVSLEDQHYKGDGSMLSPKAKALLRDSKK
ELLSCPTPGCDGSGHVTGNYASHRSVSGCPLADKTLKSLMAANAQELKCPTPGCDGSGHV
TGNYASHRSLSGCPRARKGGLKLTLNKENNTSPLVTCPVMGCDGQGHISGKYTSHRSAGS
CPLAAKRQKESSINGLPFAWKASKQELPHCPLPGCNGLGHANNVFATHRSLSGCPLNAHN
IKKKHSEEEMTIKLKASSGVENKEDIKHLDHEIEDLNESNMKIEADMMQLQTQQISSMEC
NLKTIEEENKMIEQHNDSLLKELARLSQALINSLTDIQLPQMGPISEHNFEAYVKTLTDM
YNNSEHEYSPECKALLDNIKQAIKGIHV

>Takifugu rubripes_ST18b(ENSTRUP_14768)
EMSRGRVNFSLLEQAIALQTEQRQALHHAYREMDRFLMEQMSHERRHQRMMEMDGRLSYH

GGKESPRADKKDVKCPTPGCDGTGHVTGLYPHHRSLSGCPHKVRVPPEILAMHENVLKCP
TPGCTGRGHVNSNRSTHRSLSGCPIAAAVKVSKPQEESLKCRQTPDRSPRAVGLMKKLEM
NQLNYRLSHPAAALQSTLAKDMDKYSRIRFDYASFDAQVFGKQPLMGANPEQEMSPFSDS
PQQYPGFLAAPGGCLPTSGHHSPSSQFKKDDGLQASAATAAILNLSTRYQKNTEAVAGRP
LAAPAKDMLIEVDENGTLDLSMKKCKENVKTQARTPPGDSLGPSEAAAAKGGSLLIGPAF
YQPLCDRDSWESPLNFSKAHILHDPEEDYDGVSLEDQHYKGDGSMLSPKAKALLRDSKKE
LLSCPTPGCDGSGHVTGNYASHRSVSGCPLADKTLKSLMAANAQELKCPTPGCDGSGHVT
GNYASHRSLSGCPRARKGGLKLTRSVPPPQRHPLFARCPVMGCDGQGHISGKYTSHRSAG
SCPLAAKRQKESSINGLPFAWKASKQELPHCPLPGCNGLGHANNVFATHRSLSGCPLNAH
NIKKKHSEEEMTIKLKASSGVENKEDIKHLDHEIEDLNESNMKIEADMMQLQTQQISSME
CNLKTIEEENKMIEQHNDSLLKELARLSQALINSLTDIQLPQMGPISEHNFEAYVKTLTD
MYNNSEHEYSPECKALLDNIKQAIKGIHV

>Tetraodon nigroviridis_ST18(ENSTNIP_11341)
EMSRGRVNFSLLEQAIALQTEQRQALHHAYREMDRFLMEQMTNERRHQRMMEMDGRLTYH
GGKDSPRTDKKDVKCPTPGCDGTGHVTGLYPHHRSLSGCPHKVRVPPEILAMHENVLKCP
TPGCTGRGHVNSNRSTHRSLSGCPIAAAVKVSKPQDESLKCRQTPDRSPRAVGLIKKLEM
NQLNYRLSHPVASLQTNLAKDMDKYSRIRFDYASFDAQVFGKQPLMGVNPEQEMSPFSDS
PQQYPGFLAAPGGRLPTSGHHSPSSQFKKEDSLQASAATAAILNLSTRYRKNMEAVAGRP
LATSTKDMLIEVDENGTLDLSMKKCKENVTAQAKMPPEDSLTPSEAAAAKGGSMLIGPAF
YQPLCERESWESPINFSKAHVLHDPEEDYDHVSLEEQHYKGDGSMLSPKAKALLRDSKKE
LLSCPTPGCDGSGHVTGNYASHRSVSGCPLADKTLKSLMAANAQELKHRCPTPGCDGSGH
VTGNYASHRSLSGCPRARKGGLKLTPNKDDKDEQELKCPVMGCDGQGHISGKYTSHRSAG
SCPLAAKRQKESSISGLPFAWKASKQELPHCPLPGCNGLGHANNVFATHRSLSGCPLNAH
NIKKKHSEEEMTIKLKASSGVENKEDIKHLDHEIKDLNESNMKIEADMMQLQTQQISSME
CNLKTIEEENKMIEQHNDSLLKELARLSQALINSLTDIQLPQMGPISEHNFEAYVKTLTD
MYNDSEHEYSPECKALLDNIKQAIKGIHV

>Gasterosteus aculeatus_ST18(ENSGACP_23711)
MDSEGEEQTLRTRNGDTGVPLTAVVKLRSVPYGSVARKRSAQEGVQGAPVNKRKSLLMKP
RHYSPSEACEEESEDLAPPQDKEEPRNIDTPAVIVRFGGHFLLKKKQKLDEQTGGRRIKF
SCFSQKVTQNLKPLKALWRITVDIQFLSRVLEGSQKNGYDAESSDSLGCSEVTSDGEPDA
GDEELPDSERDDQTEDLMSGASSSVLTETPGRPSVSRRVCPAAAPPPPRKTRRGGSGPRE
RRSPARRGAAEVQAAAAEEDGEGEAEAERPPQPDGWGLGLHERGLEMGRGRVNFSLLEQA
IALQTEQRQVLHHAYREMDRFLMEQMTSERRHHRMMDMDGRLSFHGGKDSPRTDKKDIKC
PTPGCDGTGHVTGLYPHHRSLSGCPHKVRVPPEXMHENVLKCTRPCTRRGHVNSNRSTHR
SLSGCPIAAAVKVSKPQEESPKCRQTPDCSPRAIGLMKKFEMNQLNYRLSHPVAALKTSL
AKDMDKYSRIRFDYASFDAQVFGKQPLMGASQDQEMPHFPDSPQRYHAFLGAPGGGLPAS
GQRSPPSQFRKEDGLQAAAATAAILNLSTRYRKNMEAVAGRPLATSTKDVPIEVDENGTL
DLSMKKSKDSGKGPLDDSLSLAEAAKGGGVLVGPAFYQPLCERDGWDGPLPLHFNKAHVS
PDAEVQTDARHNRRPKNFLNNGSVMSPQAKLLLLRDAKKEPLNCPTPGCDGSGHVTGNYAS
HRSVSGCPLADKTLKSLMAANSQELKCPTPGCDGSGHVTGNYASHRSLSGCPRARKGGLK
LTPDKDDKDEQELKCPVMGCDGQGHTSGKYTSHRSAGSCPLAAKRQKESSVSGLPFAWKA
NKQELPHCPLPGCNGLGHANNVFATHRSLSGCPLNAQSIKKKHSEEEMMTIKLKTSSGVE
NKEDIQHLDHEIQELNESNMKIEADMMQLQTQQISSMECNLRTIEEENRMIEQHNDGLLK
ELARLSQALINSLTDIQLPQMGPISEQNFEAYVNTLTDMYNNSEHEYSPECKALLDNIKQ
AIKGIHV

>Oryzias latipes_ST18(ENSORLP_21882)
FLQKMDSEGEEQTLKTCHSGSGLAECITETHSPYASMARKRSAQEGVQGAPVNKRKSLLM
KPRHYSPSEACEEESEDLAPPQDKEELRNIDTPAESHLLHSCFPPSAISQQPQSLCSGIK
TDILRAVFLGKVKRKLSVFFSCSNLSYGNFQRINGVADGGYPCAGAKSCSPLGWSEVTSN
GSPQCEVDSSEAALAEEEDDLNNDEEDQNLDRMSGTSSPQSPYRNMAKWEPLSLSAKDNS
PSEDLSGRSDHLRQELLPEAEALRYKLLSAEEDSEGEAEAERPPRLDSWALGLRERGLDM
SRGSVNLSLLEQAIALQTEQRQVLHHAYREMDRFLMEQMTSERRTHRMMDMDCRLRHTHT
HCRLLSKLMSKWLLPFALGRGRGAGLLRAISSRHKSDLPYQVLAMHENVLKCPTPGCTGR
GHVNSNRSTHRSLSGCPIAAAVKVSRPQEESLKCRQTPDRSPRAIGLIKKFEMNQLNYRL
SHPVATLQTSLTKDLDKYSRIRFDYASFDAQVFGKQPLLGTNPEQEMPLYPDCKFHSSPQ
QYPGFLGAPGGRLPASGQHSPLSPFKKEDDPQATAATAAILNLSTRYRKNVEAVAGQPLA
TTTKDMAIEVDENGTLDLSMKRCKENGKAQSRTPPEAPLSPPESAVAKGGSVLIGPAFYQ
PLCERDSWESSLPVHFSKAHILQDKEDDYEQISLEDQHYLGDSSMLSPKAKLLLRDSKKE

LLSCPTPGCDGSGHVTGNYASHRSVSGCPLADKTLKSLMAANSQELKCPTPGCDGSGHVT
GNYASHRSLSGCPRARKGGLKLTPNKDDKDEQELKCPVIGCDGQGHISGKYTSHRSAGSC
PLAAKRQKESSINGLPFAWKTNKQELPHCPLPGCNGLGHANNVFATHRSLSGCPLNAQSI
KKKHTEEEMTIKLKASSGIENKDDISHLDHEIKELNNSNMKIEADMMQLQTQQISSMECN
LKTIEEENRMIEQHNDSLLKELARLSQALINSLTDIQLPQMGPISEHNFEAYVNTLTDMY
NNSEHEYSPECKVLLDNIKQAIKGIHV

>Homo sapiens_MYT1(ENSP_477771)
MSLENEDKRARTRSKALRGPPETTAADLSCPTPGCTGSGHVRGKYSRHRSLQSCPLAKKR
KLEGAEAEHLVSKRKSHPLKLALDEGYGVDSDGSEDTEVKDASVSDESEGTLEGAEAETS
GQDEIHRPETAEGRSPVKSHFGSNPIGSATASSKGSYSSYQGIIATSLLNLGQIAEETLV
EEDLGQAAKPGPGIVHLLQEAAEGAASEEGEKGLFIQPEDAEEVVEVTTERSQDLCPQSL
EDAASEESSKQKGILSHEEEDEEEEEEEEEEEEDEEEEEEEEEEEEEEEEEEEEEEEEEE
EEEEEEAAPDVIFQEDTSHTSAQKAPELRGPESPSPKPEYSVIVEVRSDDDKDEDTHSRK
STVTDESEMQDMMTRGNLGLLEQAIALKAEQVRTVCEPGCPPAEQSQLGLGEPGKAAKPL
DTVRKSYYSKDPSRAEKREIKCPTPGCDGTGHVTGLYPHHRSLSGCPHKDRIPPEILAMH
ENVLKCPTPGCTGQGHVNSNRNTHRSLSGCPIAAAEKLAKSHEKQQPQTGDPSKSSSNSD
RILRPMCFVKQLEVPPYGSYRPNVAPATPRANLAKELEKFSKVTFDYASFDAQVFGKRML
APKIQTSETSPKAFQCFDYSQDAEAAHMAATAILNLSTRCWEMPENLSTKPQDLPSKSVD
IEVDENGTLDLSMHKHRKRENAFPSSSSCSSSPGVKSPDASQRHSSTSAPSSSMTSPQSS
QASRQDEWDRPLDYTKPSRLREEEPEESEPAAHSFASSEADDQEVSEENFEERKYPGEVT
LTNFKLKFLSKDIKKELLTCPTPGCDGSGHITGNYASHRSLSGCPLADKSLRNLMAAHSA
DLKCPTPGCDGSGHITGNYASHRSLSGCPRAKKSGVKVAPTKDDKEDPELMKCPVPGCVG
LGHISGKYASHRSASGCPLAARRQKEGSLNGSSFSWKSLKNEGPTCPTPGCDGSGHANGS
FLTHRSLSGCPRATFAGKKGKLSGDEVLSPKFKTSDVLENDEEIKQLNQEIRDLNESNSE
MEAAMVQLQSQISSMEKNLKNIEEENKLIEEQNEALFLELSGLSQALIQSLANIRLPHME
PICEQNFDAYVSTLTDMYSNQDPENKDLLESIKQAVRGIQV

>Gorilla gorilla_MYT1(ENSGGOP_6203)
MSLENEDKRARTRSKALRGPPETTAADLSCPTPGCTGSGHVRGKYSRHRSLQSCPLAKKR
KLEGAEAEHLVSKRKSHPLKLALDEGYGVDSDGSEDTEVKDASVSDESEGTLEGAEAETS
GQDEIHRPETAEEEEEEEAAPDVIFQEDTSHTSAQKAPELRGPESPSPKPEYSVIVEVRS
DDDKDEDTHSQKSTVTDESEMHDMMTRGNLGLLEQAIALKAEQVRTVCEPGCPPAEQSQL
GLGEPGKAAKPLDTVRKSYYSKDPSRAEKREIKCPTPGCDGTGHVTGLYPHHRSLSGCPH
KDRIPPEILAMHENVLKCPTPGCTGQGHVNSNRNTHRSLSGCPIAAAEKLAKSHEKQQPQ
TGDPSKSSSNSDRILRPMCFVKQLEVPPYGSYRPNVAPATPRANLAKELEKFSKVTFDYA
SFDAQVFGKRMLAPKIQTSETSPKAFQCFDYSHDAEAAHMAATAILNLSTRCWEMPENLS
TKPQDLPSKSVDIEVDENGTLDLSMHKHRKRENAFPSSSSCSSSPGVKSPDASQRHSSTS
APSSSMTSPQSSQASRQDEWDRPLDYTKPSRLREEEPEESEPAAHSFASSEADDQEVSEE
NFEERKYPGEVTLTNFKLKFLSKDIKKELLTCPTPGCDGSGHITGNYASHRSLSGCPLAD
KSLRNLMAAHSADLKCPTPGCDGSGHITGNYASHRSLSGCPRAKKSGVKVAPTKDDKEDP
ELMKCPVPGCVGLGHISGKYASHRSASGCPLAARRQKEGSLNGSSFSWKSLKNEGPTCPT
PGCDGSGHANGSFLTHRSLSGCPRATFAGKKGKLSGDEVLSPKFKTSDVLENDEEIKQLN
QEIRDLNESNSEMEAAMVQLQSQISSMEKNLKNIEEENKLIEEQNEALFLELSGLSQALI
QSLANIRLPHMEPICEQNFDAYVSTLTDMYSNRDPENKDLLESIKQAVRGIQV

>Mus musculus_MYT1(ENSMUSP_104387)
MSSESDDKRARTRSKTLRGPPETTGADLSCPTPGCTGSGHVRGKYSRHRSLQSCPLAKKR
KLEDAETEHLVSKRKSHPLRLALDEGYRMDSDGSEDAEVKDVSVSDESEGPLEEAEAEMS
GQEEIHHPQTAEGKSLIKPHFDSNPTSSPSGFSKSSYSSYQGIIATSLLNLGQIAEEALV
KEDSVSVAKLSPTVVHQLQDEAAMGVNSDEGEKDLFIQPEDVEEVIEVTSERSQEPCPQS
LKDMVSEESSKQKGVLGHEEEGEEEEDEEEEDEEEEEEGEEGEEEEEEEEEEEEEEEDEE
EEEEEEEAAPNVIFGEDTSHTSVQKASPEFRGPELSSPKPEYSVIVEVRSDDDKDEDSRS
QKSAVTDESEMYDMMTRGNLGLLEQAIALKAEQVRAVCESGCPPAEQGHLGPGEPGKMAK
PLDVVRKSCYSKDPSRVEKREIKCPTPGCDGTGHVTGLYPHHRSLSGCPHKDRIPPEILA
MHENVLKCPTPGCTGQGHVNSNRNTHRSLSGCPIAAAEKLAKSHEKQQLQTGDPPKNNSN
SDRILRPMCFVKQLEVPPYGSYRPNVAPATPRANLAKELEKFSKVTFDYASFDAQVFGKR
MLAPKIQTSETSPKAFQCFDYSHDAEAAHMAATAILNLSTRCWEMPENLSTKPQDLPSKA
VDIEVDENGTLDLSMHKHRKRENTFPSSSSCSSSPGVKSPDVSQRQSSTSAPSSSMTSPQ
SSQASRQDEWDRPLDYTKPSRLREEEPEESEPAAHSFASSEADDQEVSEENFEERKYPGE
VTLTNFKLKFLSKDIKKELLTCPTPGCDGSGHITGNYASHRSLSGCPLADKSLRNLMAAH

SADLKCPTPGCDGSGHITGNYASHRSLSGCPRAKKSGLKVAPTKDDKEDPELMKCPVPGC
VGLGHISGKYASHRSASGCPLAARRQKEGALNGSSFSWKSLKNEGPTCPTPGCDGSGHAN
GSFLTHRSLSGCPRATFAGKKGKLSGDEILSPKFKTSDVLENDEEIKQLNQEIRDLNESN
SEMEAAMVQLQSQISSMEKNLKNIEEENKLIEEQNEALFLELSGLSQALIQSLANIRLPH
MEPICEQNFDAYVNTLTDMYSNQDCYQNPENKGLLETIKQAVRGIQV

>Rattus norvegicus_MYT1(ENSRNOP_32420)
MVANSDEGEKDLFIQPEDVEEVIEVTSERSQEPCPQSLKDMVNEESSKQKGVLGHEEEEE
EEEEEEEEEEGEEGEEEEEEEEEEDEEDEEEDEEEEEEEEEEAASNVLFGDDTTHTSVQK
ASSEFRGPELSSPKPEYSVIVEVRSDDDKDEDSHSQKSAVTDESEMYDMMTRGNLGLLEQ
AIALKAEQVRAVCESGCPPAEQGHLGPGEPGKMAKPLDVVRKSCYNKDPSRVEKREIKCP
TPGCDGTGHVTGLYPHHRSLSGCPHKDRIPPEILAMHENVLKCPTPGCTGQGHVNSNRNT
HRSLSGCPIAAAEKLAKSHEKQQLQTGDPPKNNSNSDRILRPMCFVKQLEVPPYGSYRPN
VAPTTPRANLAKELEKFSKVTFDYASFDAQVFGKRMLAPKIQTSETSPKAFQCFDYSHDA
EAAHMAATAILNLSTRCWEMPENLSTKPQDLPSKAVDIEVDENGTLDLSMHKHRKRENTF
PSSSSCSSSPGVKSPDVSQRQSSTSAPSSSMTSPQSSQASRQDEWDRPLDYTKPSRLREE
EPEESEPAAHSFASSEADDQEVSEENFEERKYPGEVTLTNFKLKFLSKDIKKELLTCPTP
GCDGSGHITGNYASHRSLSGCPLADKSLRNLMAAHSADLKCPTPGCDGSGHITGNYASHR
SLSGCPRAKKSGLKVAPTKDDKEDPELMKCPVPGCVGLGHISGKYASHRSASGCPLAARR
QKEGALNGSSFSWKSLKNEGPTCPTPGCDGSGHANGSFLTHRSLSGCPRATFAGKKGKLS
GDEILSPKFKTSDVLENDEEIKQLNQEIRDLNESNLEMEAAMVQLQSQISSMEKNLKNIE
EENKLIEEQNEALFLELSGLSQALIQSLANIRLPHLEPICEQNFDAYVNTLTDMYSNQDC
YQNPENKGLLETIKQAVRGIQV

>Canis familiaris_MYT1(ENSCAFP_19510)
MSLDNEDKRARTRSKAVRVPPEPTGADLSCPTPGCTGSGHIRGKYSRHRSLQSCPLAKKR
KLEGAQAEHLVSKRKSHPLKLALDEGYSVDTDGSEDAEVKDASVSDESEGTLEEDEAEMS
GQEEIHRPEPAEGRSPVKSHFASKPIGSSAGPAKGSYSSYQEIIATSLLNLGQIAVETLV
GEDLGQVAKPGPAVVHLLQEEAEEEATSDEGEKDVFIRPDDTEDVVEVSRECSQEPGPQS
VEGVAREESSKMKVTMGNEEEEEEEEEEEEEEEEEEEEEEEEEEEEEDEEEEEEEEVEEE
EEEEVPAPDVICQEDAPHAPTQKAPELRHPASPSPKPEYSVIVEVRSDDGKDEDSHSQKS
AVTDESEMYDMMTRGNLGLLEQAIALKAEQVQAVCEPGCPPTEQGQLGPGEPGKAARPLD
ASRKNYCSKDPSRAEKREIKCPTPGCDGTGHVTGLYPHHRSLSGCPHKDRIPPEILAMHE
NVLKCPTPGCTGQGHVNSNRNTHRSLSGCPIAAAEKLAKSHEKQQPQTGDPSKNSSNSDR
ILRPMCFVKQLEVPPYGSYRPSVAPATPRANLAKELEKFSKVTFDYASFDAQVFGKRMLA
PKIQTSETSPKAFKCFDYAHDAEAAHMAATAILNLSTRCWEMPENLSTKPQDLPSKAADI
EVDENGTLDLSMHKHRKRESAFPSSSSSSSSPGVKSPEASQRQSSTSAPSSSMTSPQSSQ
ASRQDEWDRPLDYTKPSRQREEEPEESEPAAHSFASSEADDQEVSEESFEERKYPGEVTL
TNFKMKFLSKDIKKELLTCPTPGCDGSGHITGNYASHRSLSGCPLADKSLRNLMAAHSAD
LKCPTPGCDGSGHITGNYASHRRTSLSGCPRAKKSGARVTPTKDDKEDPELMKCPVPGCV
GLGHISGKYASHRSASGCPLAARRQKEGSLNGSPFSWKSLKNEGPTCPTPGCDGSGHANG
SFLTHRSLSGCPRATFAGKKGKVSGDEVLSTKFKTSDGAVLENDEEIKQLNQEIRDLNES
NSEMEAAMVQLQSQISSMEKSLKNIEEENKLIEEQNEALFLELSGLSQALIQSLANIRLP
HMEPICEQNFDAYVSTLTDMYSNQECYQNPENKDLLESIKRAVRGIQV

>Felis catus_MYT1(ENSFCAP_1104)
MSLEHEDKRARTRSKALRVPPEPTGADLSCPTPGCTGSGHIRGKYSRHRSLQSCPLAKKR
KLEGAQPEHLVSKRKSHPLKLALDEGYSVDTDGSEDAEVKDASVSDESEGTLEEDEAEMS
GQEEIHRPEPAEGRSPVKPHFGSNPISSPTGPTKGSYSSYQEIIATSLLNLGQIAVETLV
GEDLGQVAKPGPGVVHLVQEEAEEGATSDEGEKDVFIRPEDTEEVMEVTRERSQELGPQS
GEGMVSEESSKMKVXEEEEEEEEEEEEEVAAPDVICQEDAPHAPTQKAPEPRHPASPSPKP
EYSVIVEVRSDDGKDEDSRSQKSAVTDESEMYDMMTRGNLGLLEQAIALKAEQVQAVCEP
GCPPTEQGQPGPGEPGKVVRPLDAARKNYCSKDPSRAEKREIKCPTPGCDGTGHVTGLYP
HHRSLSGCPHKDRIPPEILAMHENVLKCPTPGCTGQGHVNSNRNTHRSLSGCPIAAAEKL
AKSHEKQQPQGGDPSKNSSNSDRILRPMCFVKQLEVPPYGSYRPSVAPATPRANLAKELE
KFSKVTFDYASFDAQVFGKRMLAPKIQTSETSPKAFKCFDYTHDAEAAHMAATAILNLST
RCWEMPENLSTKPQDLPGKAADIEVDENGTLDLSMRKHRKRDSAFPSSGSSGSSPGVKSP
DASQRHSSTSAPSGSMTSLPSSQASRQDEWDRPLDYTKPSRQREEEPEESEPAAHSFASS
EADDQEVSEESFEERKYPGEVTLANFKMKFLSKDIKKELLTCPTPGCDGSGHITGNYASH
RSLSGCPLADKSLRNLMAAHSADLKCPTPGCDGSGHITGNYASHRRTSLSGCPRAKKSGA
RVTPTKDDKEDPELMKCPVPGCVGLGHISGKYASHRSASGCPLAARRQKEGSLNGSPFSW

KSLKNEGPTCPTPGCDGSGHANGSFLTHRSLSGCPRATFAGKKGKVSGDEVLSTKFKTSD
GAVLENDEEIKQLNQEIRDLNESNSEMEAAMVQLQSQISSMEKSLKNIEEENKLIEEQNE
ALFLELSGLSQALIQSLANIRLPHMEPICEQNFDAYVSTLTDMYSNQECYQNPENKDLLE
SIKRAVRGIQV

>Myotis lucifugus_MYT1(ENSMLUP_6981)
MSLENEEMRARTRSKALRAPLEPTGAELSCPTPGCSGSGHIRGKYSRHRSLQSCPLAKKR
KLEGPEAQHLVSKRKSPPLKLALDEGYGVDSEGSEDAEVKDASVSEESEGALEEAEAELA
GQEGARHPPPAEGRSPIQSQFSSNPAGTPIGSSKGSYSSFQEAIATSLLDLGHVAEEPLV
REGSGQQAKPGPGVLHLVQEEAEGGAASDRGEKEVFIQPADTEEVIEVTSEYTQGPGPQF
LEGMASEALHQQTVSGDHEEKEEEEAEEEEEEEEEEEEEEEEEETEDEEDEEDEEEITPD
VTCREGAPRSPPEQAPEPHSRKPEYSVLVELRSDDGKDEDSSSQKSEVTDESEMHDMMTR
GNLGLLEQAIALKAEQVRAACEPGELGLGEPVKVARPLDATRKTYCSKDSSRAEKREIKC
PTPGCDGTGHVTGLYPHHRSLSGCPHKDRIPPEILAMHENVLKCPTPGCTGQGHVNSNRN
THRSLSGCPIAAAEKLAKSHEKQQPQTGDPSKTDSSSERILRPMCFVKQLEVPPYGSYRP
SVAPSTPRANLAKELEKFSKVTFDYASFDAQVFGKRMLAPKIQTSETSPKAFKCKFHYAH
DAKRVHIVANVHLKLTALCAEPEERLRGCAHLFPSEAVAIEVDEHGTLDLSMRRHRRRET
FPGSGGPEPGVKAPEASQRQSSTSAPGSSMTPQSSQASHQDEWDRPLDYTKPSRQREEEP
EESEPAAHSFASSEADDQEVSEENLEERKYPGDVTLTNFKLKFLSKDIKKELLTCPTPGC
DGSGHITGNYASHRSLSGCPLADKSLRNLMAAHSADLKCPTPGCDGSGHITGNYASHRSL
SGCPRAKKSGTKVTPTKDDKEDPELMKCPVPGCVGLGHVSGKYASHRSASGCPLAARRQK
EGSLNGPSFWKSPRNEGPTCPTPGCDGSGHANGGFLTHRSLSGCPRATFAGKKGKLSGDD
VLGTKFKTSDGAVLENDEEIKQLNQEIRDLNESNSEMEAAMVQLQSQISSMEKSLRTIEQ
ENKLIEEQNEALFLELSGLSQALIQSLANIRLPHMEPICEQNFDAYVSTLTDMCSSRESY
QNPEHRALLESIKQAVRGIQV

>Gallus gallus_MYT1(ENSGALP_9531)
MKLCGTFLENEFRGELLNQRWTCGEKISSCEGAASLHGTCIKMSLENDDKRARTRSKSVR
VPTELISQEVSCPTPGCNGSGHIRGKYARHRSLQSCPLAKKRKLQDAEAEHLVSKRKSHP
LKLALDEGYNVDSDGSEEAEVKEESGSEESEGTLEEDEAEVVEQEETRSPEPAEERSPAK
SAHYGQKPATSTSGPSKANYSNYQEIIANSLLNLGQIAEETLAIEGQLPEAELQVSKPPS
NVVHLVHEDAGEEIVEEECDKEIIIQTEDAEEVIEVTSERSSELCPEHQDDTNCEESCKL
QKAMQTEAEEEDEEDEDEDEDEDEEEEEEEEEEEEEEEEEEEEEMAPEVICEEAPHTSQD
TQKSHCEGQFSPKPEYSVIVEVRSDDDKDDDAHSQKSAVTDESEMYDMMTRGNLGLLEQA
IALKAEQVKIVREPSRLPGEHVKHFQVDEKQSKPLDSIRKSYYGKDPSRPEKREIKCPTP
GCDGTGHVTGLYPHHRSLSGCPHKDRIPPEILAMHENVLKCPTPGCTGQGHVNSNRNTHR
SLSGCPIAAAEKLAKSHEKQQTQSGDPSKPSSNSDRILRPMCFVKQLEIPQYGSYRPNMV
PATPRANLAKELEKYSKVTFDYASFDAQVFGKRMLAPKIQTSETSPKAFKSKPFPKASSP
SHSPSSSYVKSTSSSSTGFDYSHDAEAAHMAATAILNLSTRCWEMPENLSTKQQEAPGKS
MDIEVDENGTLDLSMNKNRKRESTFPSSSSCSSSPSVKSPDVSQRQNSTSATSSTMTSPQ
SSQTSRQDEWDGPIDYTKPNRQREEEPEESEPAAHSFASSEADEQEVSEENFEERKYPGE
VTLTNFKLKFLSKDIKKELLTCPTPGCDGSGHITGNYASHRSLSGCPLADKSLRNLMAAH
SADLKCPTPGCDGSGHITGNYASHRSLSGCPRAKKSGIKITPTKDDKEDPELMKCPVPGC
VGLGHISGKYASHRSASGCPLAARRQKEGSLNGSSFSWKSLKNEGPTCPTPGCDGSGHAN
GSFLTHRSLSGCPRATFAGKKGKLSGDEILNTKFKTSDVLENDEEIKQLNKEISELNESN
SEMEAAMVKLQSQISSMEKNLKNIEEENKIIEEQNEALFLELSGLSQALIQSLANIRLPH
MEPISEQNFDAYVNTLTDMYTNQECYQNPENKDLLESIKQAVKGIQV

>Pelodiscus sinensis_MYT1(ENSPSIP_11346)
MKLCGTFLENEFRGKLLNQRWTCGEKLSSCETAKSLHSRHNKMSLENDDKRARTRSKSIR
VPTELIGQEVSCPTPGCNGSGHIRGKYARHRSLQSCPLAKKRKLQDAEAEHLVSKRKSHP
LKLALDEGYNVDSDGSEETEVKEESVSDESEGTLEEEEAETIEQEETHSPEPAEVRSPAK
SSHYGHNAATNTPGPSKANYSSYQEIIANSLLNLGQIAEETLAIEGQLPENELQVAKPPV
NVVHLVHEGIEEEVVEEAGDKEIIIQTEDAEEVIEVASERSSELCPEHQDDMSCEESCKL
EKGIQAEAEEEEEEEEEEEEEEEEEEDEDEEDEDEEEDEEEEMAPEVICEETPQTSQDS
HKTPPEYSVIVEVRSDDDKDDDARSQKSAVTDESEMYDMMTRGNLGLLEQAIALKAEQVK
ITREPSRLPGEHTKHFQGDEKQNKPLDTIRKNYYSKDPSRPEKREIKCPTPGCDGTGHVT
GLYPHHRSLSGCPHKDRIPPEILAMHENVLKCPTPGCTGQGHVNSNRNTHRSLSGCPIAA
AEKLAKSHEKQQPQAGDPSKSSSNSDRILRPMCFVKQLEIPQYGSYRPNMVPATPRANLA
KELEKYSKVTFDYASFDAQVFGKRMLAPKIQTSETSPKAFKSKPFPKASSPSHSPSSSYV
KSTSSSSTGFDYSHDAEAAHMAATAILNLSTRCWEMPENLSTKQQDFPSKSMDIEVDENG

TLDLSMNKHRKRESAFPSSSSCSSSPSVKSPDVSQRQNSTSATSSTMTSPQSSQTSRQDE
WDGPIDYTKPNRQREEEPEESEPAAHSFASSEADEQEASEENFEERKYPGEVTLTNFKLK
FLPKDIKKELLTGSGGTNNRGYKTSCFVGHQKRESQDHHHGQTMSCPTPGCDGSGHITGN
YASHRSLSGCPLADKSLRNLMAAHSADLKCPTPGCDGSGHITGNYASHRSLSGCPRAKKS
GIKITPTKDDKEDPELMKCPVPGCVGLGHISGKYASHRSASGCPLAARRQKEGSLNGSSF
SWKSLKNEGPTCPTPGCDGSGHANGSFLTHRSLSGCPRATFAGKKGKLSGDEILNTKFKT
SDVLENDEEIKQLNREITELNESNSEMEAAMVKLQSQISSMEKNLRNIEEENKIIEEQNE
ALFLELSGLSQALIQSLANIRLPHMEPISEQNFDAYVNTLTDMYTNQECYQNPENRDLLE
SIKQAVKGIQV

>Xenopus tropicalis_MYT1(ENSXETP_25128)
MNVDNVNKGTRTRSKAIRVIPLDLIEQEVSLQSCPLSRKRKLQESEQEHPPSKRKSHPLK
LALDEGFNVDSDGSEEAEVKEEDSGTEESEAIIEDIEEDTEPTEPKEEPSSPTVEAESTA
KVDTEETETKTESSPPAKVNYSSYQEIIANSLLNLGQVAKEALVSEGHLQESELNEEKPS
SVKSNEEEVEQLMVEEACEKEIIIQTEDAEEVIEVTSEPSSESGTEPRDEVNCEDTGKLQ
KDMEEHSIEMANQDLPHASQDSPKLHCEGQFSPKPEYSVIVEVRSDDDKDDDSHSQKSAV
TDESEMYDMMTRGNLGLLEQAIALKAEQVKVIREPSRSSLDNMKNFSADEKQNRPIDTMR
KNFYDAARPEKREIKCPTPGCDGTGHVTGLYPHHRSLSGCPHKDRIPPEILAMHENVLKC
PTPGCTGQGHVNSNRNTHRSLSGCPIAAAEKLARSHEKQQQPGDLSKGSSNSDRILRPMC
FVKQLEIPQYGSYRPNMAPATPRANLAKELEKYSKVTFDYASFDAQVFGKRLIAPKIPSS
ETSPKAFKSKPFPKASSPSHSPSSSYIKSTSSSSSSGFDYTHDAEAAHMAATAILNLSTR
CWEMPENLSTKQQDTPGKSSEMKVDENGTLDLSMNKHRKRESTFPSSSSCSSSPSMKSPD
QSRGQNCTSATSSNMTSPQSSQTSRQDDWDGPIDYTKPNRQREEEPEEMEPAAASFASSE
ADDQEMQEESYEERKYPGDVTLTNFKLKFLSKDSKKELLTCPTPGCDGSGHITGNYASHR
SLSGCPLADKSLRNLMAAHSADLKCPTPGCDGSGHITGNYASHRSLSGCPRAKKSGLKIT
PTKDDKDDPELMKCPVPGCDGLGHISGKYASHRSASGCPLAARRQKEGALNGSAFSWKSL
KTEGPTCPTPGCDGSGHANGSFLTHRSLSGCPRASFAGKKGKISGDEFLGTKFKTSDVLE
NDEEIKQLNKEINELNESNSEMEADMVNLQSQITSMEKNLKNIEDENKVIEEQNDALFVE
LSGLSQALIRSLTNIRLPQMEPISEQNFDAYVNTLTDMYTNQECYQNPENKALLESIKQA
VKGIKV

>Danio rerio_MYT1a(ENSDARP_136943)
MKLCGTFLENEYRGKLLNQRWTRGDKISRCRPSLRLQSRPIKMSLDTDDKRTRTRSKAGR
VPSEIVGQELSCPTPGCNGTGHISGKYARHRSALSCPLARKRRLQEAESEQPSSKRKSHP
LKLAMDEGFNAESEGSGEETELREEEEEEDEECTQQKLGDQEDEQQAKQNTESTNTEEEG
DEEECIIIESSSTEKPKPSPSEDLSSYQHIVANSMTHLNNHNNGAMSPQQPPVIMETQEK
SPFRETEEEKYGTEETRSQVGVVVCKENGLKLEDEEADSESQKESDHQYFTEEMQTQQDD
EEEEEDDDDDEGLRSEIQKGETYHNELNDIKHEEEEEEEERETVCSVITATQGSHIREDYI
SHKLALPQSYSSNKAPTASAMLIDVQSEGSERDADDDDDDDDDDDDDDGDEGDDDSLSQR
STVADESEMFDIMRGNLGLLEQAIALKAQQVKAHRGLSRIPEHHRFFALDDRAGKHMEHI
RKSCFVKESSRSEKREIKCPTPGCDGTGHVTGLYPHHRSLSGCPHKDRIPPEILAMHENV
LKCPTPGCTGQGHVNSNRNTHRSLSGCPIAAAEKLSKSHDKQHLPQPMGEHIKGSPNSDR
VLRPMCFVKQLEIPPHGSYRPSLVPATPRANLAKELEKYSKVSFDYASFDVQVFGKRMLA
PKMHTSETSPKAFKSKPPFPRVSPPSPSLHGVYGKSSSSGYDYSHDAEAAHMAATAILNL
STRCWERPENLSIRHQDKSMEIEVDENGTLDLSMKKPIKREVGMSCTSPEVRSPDLSSSS
SSSSTSLHHGNSSISPHSLHTYKQEEWEGPLDYTKPNRQREEELEEMDHSAPSFASSDPE
DCDMMQDGLEDRKYPGEVTTPSFKVRFQPKDNKKDLLMCPTPGCDGSGHITGNYASHRSL
SGCPLADKSLRSLMAAHTAELKCPTPGCDGSGHITGNYSSHRSLSGCPRAKKGGIKTTPI
KDDKEDSELLKCPVPGCDSLGHISGKYATHRSAYGCPLAARRQKEGLLNGSPFSWKAFKT
EGPTCPTPGCDGSGHANGSFLTHRSLSGCPRASLNKKKAKFPGEEYLSTKFRASDVLDND
EDIKQLNKEINELNESNNEMEADMVNLQTQITSMEKNLKNIEQENKMIEEQNEALFMELS
GLSQALIRSLANIRLPHMQEPITEQNFESYVSTLTDMYTNKDCYQNPENKALLETINKAV
KGIKV

>Danio rerio_MYT1b(ENSDARP_102049)
MKLCKTFLENEFRGQLLSQRWTRGDRVSSCESKRSRRQKMSQDADDKRTRTRSKGIRVPL
ELVGQEYSCPTPGCDGLGHVSGKYARHRSALGCPLAKKRKLQEAEENLPAPKKKPNLLKL
AMDDGFNADSDTNSETETKDEGVESEEDTADKVMEVKEKSPASEAMTEESSQVNLEEKET
PIRQTSPVPEIEIEKEEANIESTPIQQITMDTEAEEELQVAEEIQAREEEVEEEAEEITE
EERVTQQLKETDEPVTTVEEEEEDDEEDEEGEEETQCLSQKNNSDHQYFSGDFGKMQTKE
TGELERDSEDEDEEGDEEEEEEQSQVEPSSLMAPVPTVSAQELDVMTHGNINLDHHLGED

EEEEEDEDERDDASDKDSPTVVIELGSDGEEEEEEEEEEDDGEEEEEEIDEADDDDLDSMS
QRSEVTDDSETYDMTRGNLGLLEQAIALKAEQTGHSSEESYSPEQQPYHSLDDRTSKVMD
IAARRAYYGKDGSRLEKKEVKCPTPGCDGTGHVTGLYPHHRSLSGCPHKDRIPPEILAMH
ENVLKCPTPGCTGQGHVNSNRNTHRSLSGCPIAAAGKHKSHDKQSQPQTSVIDPASVGSN
SDRVLRPMCFVKQLEIPQYGSYRTNAMPATPRANLAKELEKYSKVSFDYASFDVQVFGKR
MLAPKMPTSETSPKAFKSPSPSHSLSGGYTKSSSSSTSSGYDYTHDAEAAHMAATAILNL
STRCWERPENLSTRPPDQAGKDMDIEVDENGTLDLSMKKPKREGVRSPDPSSSSSSSSQH
HGVTSPHSSHTYKQEEWEGPLDYTKSNRQKEEEPEEVEYTTHSYASSDGEDDDGTQEGAE
DRTYPGEVTTSSYKVKFQPKDLKKEILLCPTPGCDGSGHITGNYASHRSLSGCPLADKSL
RTLMAAHTAELKCPTPGCDGSGHITGNYASHRSLSGCPRAKKGGIKTTPTKDDKEDSELI
KCPVPGCDSLGHISGKYATHRSAYGCPLAARRQKEGMLNGTPFSWKAFKTEGPTCPTPGC
DGSGHANGSFLTHRSLSGCPRASANKKRARFPGDDYISKKFRASDVLDNDEDIKQLNKEI
NELNESNSEMEADMANLHTQISSMEKNLKSMEEENKLIEEKNEALFMELSGLSQALIRSL
ANIRLPQMQEPITEQNFDAYVDTLTHMFTDKECYQNPENRALLESINQAVKGIEV

>Takifugu rubripes_MYT1(ENSTRUP_17361)
MSQDVTETRTRTRSKGIRASSELAAQEMNSCPTPGCDGKGHVSGRYSRHRSVLGCPIVKK
RKLEEAEESQSAPKKRNQPVKQAAGEDFTHDTSDEEEEEEEGEEEGEEEAEEEEELREKK
KPTSKIRAEATKESSPAGAPPDSEVCSADDGATSGNQCENKDVSQEVAAEDQVSEQLEEE
MTVINLTKTKEEEKNEEDVTKTTEEEHRERREVPEEATVVERPMINQENSDHQYSSGDYK
HQAKEVAEEQQQEEEKEEEEEEEEEPEEEEEEEEEEDEEELEEEQEEERREEEEHGHEKE
EGELMIEEDEDDQDSIKASPATVVIEVRSEEEDEEEDDDEEDEGSGITDDSENWDMSRGN
LGLLEKAIALKAGEVKGGEEVQSSPEYHPYSSSSRSSQGAGAARRSAYYGKDKKEVKCPT
PGCDGTGHVTGLYPHHRSLSGCPHKDRVPPEILAMHENVLKCPTPGCTGQGHVHSNRNTX
HSLSGCPIAAAAKLNKTQDKQVHLQPPASEASSNSDRVLRPMCFVKQLEIPQYGSFRPNT
VTTTPRANLAKELEKYSKVSFDYASFDVQVFGKRTLIPKTPSSTETSPKAFKSYDYSQDA
EAAHMAATAILNLSTRCWERPESLCSVSREPCSKESDIEVDENGTLDLSMKKTKREGLQA
SEPPSSSSSSSQLTGATLSQGHSQPEWEEPLDFTTQTGVKEEDHEEVEYAAPSYTSSDAE
EEDQDNLEDRKYPGEVTTSSFKVKFQAKDSKKELLVCPTPGCDGSGHITGNYASHRSLSG
CPLADKSLRTLMAAHTAELKCPTPGCDGSGHITGNYASHRSLSGCPRAKKSGVKATPTKD
DKEDSELLRCPVPGCDSLGHISGKYATHRSAYGCPLAARRQKEGLLNGTPFSWKAFKAEG
PTCPTPGCDGSGHANGSFLTHRSLSGCPRASANKKKAKLPGDEFITAKFRASDVLDNDED
IKQLNTEISELNESNSEMEADMMNLHTQISSMEKNLKNMEEENKQIEERNEALFLELSGL
SQALIRSLANIRLPTMEPMSEQNFDTYVEALTHMFTNKDCYQNPENRALLESINQAVKGI
EV

>Tetraodon nigroviridis_MYT1(ENSTNIP_5677)
TSVDVDEKRTRTRSKGIRVPVELLGQEQSCPTPGCSGSGHVSGRYSRHRSVLSCPIARKH
RLEEEEQDAEKPPSKRKAHPLKLALDEGFSAESDTSSEKDEDEAGGAVVSEETQGHNGDE
ATPTQDGHMELEAQLDSPEEDKETEMEEEAAAAAEGEAAQLSGQSRSWAGLKVTGMLARQ
PVAPRLIFIDRTGDAAADPGCLCSTEEECVMVGPSPRSSQRPHQAPDSLLHLGRVSGDPS
PSTAPQPPVTMETQEDNSTAAAEAKDEEQEYGDEDEGPDQSNPLIQIHHPDGEHEEMTED
ENAKGGETDGRREDHLANVQENYATHRASSPTSPDVIEVHSDRSEDRDFEDVDGDDDKDS
LSQRSTMTDESEIFDISRGNLGLLEQAIALKAEQVQPAGQRERFCSPDVHQQRYITVEDR
PKHLDALRRSYFNKESGRSEKKEVKCPTPGCDGTGHVTGLYPHHRSLSGCPHKDRIPPEI
LAMHENVLKCPTPGCTGQGHVNSNRNTHRSLSGCPIAAAEKMSKGHDKPHPPAPGGESLK
ASPNDRVLRPMCFVKQLEIPQYGSFRPSAAPSTPRANLAKELEKYSKVSFDHASFDAQVF
GKRVLAPKMPTSETSPKAFKIYDYSQDAEAAHMAATAILNLSTRCWEKPENLSTRAQDKS
AGEMDIEVDENGTLDLSMKKPIKREGSVSGASPGVRSPDPSSSSSVHQSGSSGVTSPNLL
AYKQEEWEGPLDYTKPNRQREEDMEEMEQTGQAFVSSDSEDCDLTQDGLEDRRYPGEVTS
ASFKVKFQPKDTKKELLACPTPGCDGSGHITGNYASHRSLSGCPLADKSLRSLMAAHAPE
LKCPTPGCDGSGHITGNYASHRSLSGCPRAKKSGIKTPTKDNQEDSELLKCPIPGCDSLG
HISGKYATHRSAYGCPLAQRRQKEGLLNGTPFNWKAFKAEGPTCPTPGCDGSGHANGSFL
THRSLSGCPRALYAKKKAKFPSDEYPSSKFRASHVLDNDEDIKQLDKEISDISESNNEME
ADMVNLQTQISSMEKNLKNIEHENKMIEEQNEALFVELSGLSRALIRSLANIRLPHMEPI
TEQNFDSYVSTLTDMYTNKERFQSPENKALLESINKAVKGIKV

>Gasterosteus aculeatus_MYT1a(ENSGACP_17495)
MSVESDTKRTRTRSKGIRVPIELVGQELSCPTPGCNGSGHISGRYSRHRSILGCPVARKR
RLEEAEAEQEQDAEQPAAKRKSPPLRMALDEGFSAESDAGSEAEGEGQRSGETAEIAADE
AERKLCRCSSLEHTLLNEECVIVGPRLGPAPPARRECRSASQSAEEVADSLLHLGRVSDS

FAPAAAVHQPVAVDTEEEVTVAAKRGEDAKDEQEGNMTEASEEAAEVEEEEVEDKKGECP
DSSSHVIQENHQHLTQDVRHQQIKGDEEEEEDMAAPSAEEEKEEERHKENYTAGPPLSYG
LNRVSPLEDHFPNLRGENYKVHRAVSSTSPDVIEVRSDRSEERDFEDADGDDERDDDDSL
SQRSTVTDESEMFDITRGNLGLLEQAIALKAEQVKPAGPGELLRTPDIHQQRYFTLDDRP
KHLDVIRKSYFSKESSRPEKREIKCPTPGCDGTGHVTGLYPHHRSLSGCPHKDRIPPEIL
AMHENVLKCPTPGCTGQGHVNSNRNTHRSLSGCPIAAAEKLSKGLDKQHLSQPGSEQLKG
SLNDRVLRPMCFVKQLEVPQYGSYRPNMAPATPRANLAKELEKYSKVSFDYASFDAQVFG
KRTLAPKMPASETPPKAFKTYDYSHDAEAAHMAATAILNLSTRCWEKPENLSTKAPSKEL
AIEVDENGTLDLSMKKPIKREGSSPGVRSPDLRHGGSSGVTSPHLHTYKQEEWEGPLDYT
KSSRHREEEPDEMEPSSDAEDCDMTQDCLEDRKYPGEVTTPSFKVKFQPKDSKKELLSCP
TPGCDGSGHITGNYASHRSLSGCPLADKSLRSLMAAHTPELKCPTPGCDGSGHITGNYTS
HRSISGCPRAKKSGIKTPTKDNQEDSELLKCPVPGCDSLGHISGKYATHRSAYGCPLAAR
RQKEGLLNGTPFNWKAFKTEGPTCPTPGCDGSGHANGSFLTHRSLSGCPRALFAKKKAKF
PTEDYLSTKFRASDVLDNDEDIKQLNKEINDLNESNNEMEADMVNLQTQVDSKFVCLKTI
EHENKMIEENEALLPKLSGLSRALIRSLGNISLPHMQEPITEQNFDSYVSTLTHMYTNKD
RFQSAENKALLETINRAVKGIKV

>Gasterosteus aculeatus_MYT1b(ENSGACP_16935)
SVVLAMHENVLKCPTPGCTGQGHVNSNRNTHRSLSGCPIAAATKLNKGQDKQVILQASPS
EPSSNSDRVLRPMCFVKQLEIPQYGSYRPNTVTTTPRANLAKELEKYSKVSFDYASFDVQ
VFGKRMLIPKTPSSAETSPKAFKCNSYDYSQDAEAAHMAATAILNLSTRCWERPETISTK
PREPCSKESDIEVDENGTLDLSMKKTKREGAQPPEPSSSSSSSSQHIGATLSQSHTQPEW
EGPLDFTKSSGVKEEDHEEVEYTAPSYTSSDGEEGDQENLEERKYPGEVTTGSFKVKFPP
KESKKEILICPTPGCDGSGHITGNYASHRSLSGCPLADKSLRTLMAAHTAELKCPTPGCD
GSGHITGNYASHRSLSGCPRAKKGGVKSTPTKDDKEDSELLRCPVPGCDSLGHISGKYAT
HRSAYGCPLAARRQKEGLLNGTPFSWKAFKTEGPTCPTPGCDGSGHANGSFLTHRSLSGC
PRATANKKRAKFPGDEYITAKFRASDVLDNDEDIKQLNKEINELSESNSEMEADMMNLHT
QISSMENNLKSMEEENKHIEERNEALFLELSGLSQALIRSLTNIRLPALEPPSEQNFDSY
VDTLTHMFTNKDCYQNPENRALLESINQAVKGIEV

>Oryzias latipes_MYT1a(ENSORLP_24992)
DDDEAEEQAEEDEEGGRTEPEAAVPATQGTDSEILLRDQTVISLVASKQGYDEGDEHEHA
SVGSGITDDSENWDMTRGNLGLLEQAIALKAEEVQGGSESFSSSDYQPHGSSIKASESAA
AARRSSYFNKGKKKLKKKEVKCPTPGCDGTGHVTGLYPHHRSLSGCPHKDRIPPEILAMH
ENVLKCPTPGCSGQGHVNSNRNTHRSLSGCPIAASSKLIKQQDKPVHLQAAASEPSSNSD
RVLRPMCFVKQLEIPQYGNYRPNTATTTPRANLAKELEKYSKVSFDYASFDVQVFGKRMI
IPKTVGITDTSAKASKASLPKASSPTHAVSESFPKTALSSSGYDYSQEAEAAHMAATAIL
NLSTRCWERPETLSTKPQNPYAKESDIEVDENGTLDLSMKKAKTDDTPSPEPSSSSYTSS
QPVGVTSQEHTHDDWEGPLDFTKSTEVKEEDEEEMDYTALSYSSSEGEEDDQESLGDRKY
PGEVTTTNFKVKFQPKDSKKDIIVCPTPGCDGSGHITGNYASHRSLSGCPLADKSLRTLM
AAHSAELKCPTPGCDGSGHITGNYASHRSLSGCPRAKKGGVKTTPTKDDKDDSELLKCPV
PGCDSLGHISGKYATHRSAYGCPLAARRQKEGLLNGTPFSWKAFKTEGPTCPTPGCDGSG
HANGSFLTHRSLSGCPRASANKKKTKLPGDEYITAKFRASDVLDNDEDIKQLNKEISELN
ESNSEMEADMLHLHTQISSMEKNLKSLEEENEQMEEKNEALFLELSGLSQALIHSLANIH
LPTMEPMTEQNFDSYVDTLTQMFTNKDCYQNPENRALLESINQAVKGIEV

>Oryzias latipes_MYT1b(ENSORLP_2798)
MSVESDDKRTRTRSKGIRVPIELVGQELRICPTPGCNGSGHISGRYSRHRSILGCPIARK
RRLEEAEQEQEQEAERPASKKSDASSIKLCFENSVAMEADDPAAEWEEEVKDEQEENLME
EEEAQRLQKLEESPALQEEANDQNDEDDEGSDCHVFLHEQIKHEQEAEEEEEESEEEMVV
SVQETRTEEEEEHRGHSISTSDAPTAVRTITSTAAAQASSSSASPDIIEVRSEKDFDLDG
DVDHDDEDSLSQRSAFTDESEMFDMTRGNLGLLEQAIALKAEQVKPGGPRELLRAPDIHH
QRYFTLEDRPKHLDIIRKSYFRNDILIFINVLQHLRNVTRRCQKQHFHLSGSLQNKDLKR
LHEEHGSKSPSSSYVSQMNLLKTMEEVQINPAKNISKLNSCKEPQRTSVYIKSDKRSSLF
EVSLGETPNPTLLLPGGSSSRQRRIRLNRRKVLQKDLRQKTRAFDGSKPVLKMSNKDRIS
SSLSTPPPPPPSIMSDAESSRPEKREIKCPTPGCDGTGHVTGLYPHHRSLSGCPHKDRIP
PEILAMHENVLKCPTPGCTGQGHVNSNRNTHRSLSGCPIAAAEKLSKTHDKPAVAPPTSE
LLKVSPNDRVLRPMCFVKQLDVPQYGSYRPNMAPSTPRANLAKELEKYSKVSFDYASFDA
QVFGKRTLAPKISTSETSPKAFKTYFEYCKNKYRFANCTNKTKPPSSPSLSLHSYGKSSS
LAYDYSHDAEAAHMAATAILNLSTRCWEKPENLSTKPPNKEMDIEVDENGTLDLSMKKPI
KREGSVSGTSPGVRSPDPSSSSSSSLHHGGNSGMTSPNLQTYKQEDWEGPLDFTKPNRQR

EEEADESYVSSDPEDCDMMQDGLEERKYPGEVTTPSFKVKFQPKDSKKELLSLSGCPLAD
KSLRSLMAAHTPELKCPTPGCDGSGHITGNYASHRSLSGCPRAKKSGIKTPTKDNQEDSE
LLKCPVPGCDSLGHISGKYATHRSAYGCPLAARRQKEGLLNGTPFNWKAFKTEGPTCPTP
GCDGSGHANGSFLTHRSLSGCPRALYAKKKAKFPSEDYLSTKFRPTDVLDNDEDIKQLNK
EISDLNDSNNEMEADMVNLQTQISSMEKNLKSIEHENKMIEEQNEALFVELSGLSRALIR
SLANIRLPHMEPITEQNFDRYVSTLTDMYTNKDCFQSPENKALLESINKAVKGIKV

>Trichoplax adhaerens_(XP_002116647)
MSEVDDKPFQCPIEGCKESFTNMDHLEVHQKRHTSNLKIQCANRASSDTPASALPDQTPTPSRFLRAFEE
AGLEDALQNPFEATFAKAAVDRADPSAAIASGVLATMKLRDSSQHDVAAALTSLSQQPSPVPISPTSIAT
LLPSKSTIKPVIESIGNTTSASTATVVANPIATTATQQSISHSIPTITKTVTAQLSQQPVNNGALNSITV
VSAKERLQHSLRVKAIKTSASTASSKTPQVTKTKTLDIVNKVTKAPTAVVKAVSTNDTPVRKSEAVTNGP
HRTKRARRSQEDLDPEEKRQRFLERNRAAATRCREKKKTWISGLDKKVKELSDKNSSLQAEAAKLRSEVA
HLKSLLIAYQNGSIAPRKEHLSDSVSDAETVASSVLTHLAMAPSTDITSNGGAKITASTVVLDN

>Amphimedon queenslandica_(XP_011403471)
MSGEVGSGTTSANASTQSTPTPPNIGRRGRRLRKCATPIKSAKKDERGTFPQTGSLSLVLPPGTNSSNSD
VITPSSSTATTPAKIENETKATPIRAPLPSPSTLPGTSNLIKKRKRIPYTLVEATKGIRCPTSGCYGLGH
VTGLYAMHYAVSGCPIAAQAKKELEKDVIKPPRPPPPHPSFRKKKRRFRSYKKKVPAQNGASKTGVTPPI
NEPHAIGETETLSKAPTTRLSSSKQTDSNPQSSSVKPPSSAESTSKDSSVKKRKRIPYTLVEAVKGNRCP
TPGCGGIGHITGLYSMHYAVSGCPLAHGKTPEECKSRRDELNKSRAQASQTLESESALSPPPRKNPRLMA
SRNLSATLDSVAIGDPLPPNNTPSFKTLPPNDNKKRFRMPEYYFRPEPKLEGLTSSQDLQLFREAQAKMR
EQLVNVEGKHTDYKLSKIIFGCYEIDTWYSSPYPPEYTELSQIYVCEFCLRYYKTVVTYNNHTSCCVQRR
PPGREIYRKNTLSVFEVDGENHKEYSQNLCLLAKLFLDHKTLYFNVEPFLFYIVTRYDDTGCHIVGYFSK
EKHSPQGFNVSCILVMPPHMRRGYGHLLIDFSYLLTRHQEKVGSPERPLSDLGLLCYRSYWKDKVLSYLT
KHNSQSISMKEISLETGISADDLISSLQYYRILKYWKGKHIIIKKKELLEEHSCKVERHKSSDLTIDASC
LKWMPTTYPVYP

Nuclear Receptor Coactivator-NCOA

>Homo sapiens_NCOA1(ENSP_320940)
MSGLGDSSSDPANPDSHKRKGSPCDTLASSTEKRRREQENKYLEELAELLSANISDIDSL
SVKPDKCKILKKTVDQIQLMKRMEQEKSTTDDDVQKSDISSSSQGVIEKESLGPLLLEAL
DGFFFVVNCEGRIVFVSENVTSYLGYNQEELMNTSVYSILHVGDHAEFVKNLLPKSLVNG
VPWPQEATRRNSHTFNCRMLIHPPDEPGTENQEACQRYEVMQCFTVSQPKSIQEDGEDFQ
SCLICIARRLPRPPAITGVESFMTKQDTTGKIISIDTSSLRAAGRTGWEDLVRKCIYAFF
QPQGREPSYARQLFQEVMTRGTASSPSYRFILNDGTMLSAHTKCKLCYPQSPDMQPFIMG
IHIIDREHSGLSPQDDTNSGMSIPRVNPSVNPSISPAHGVARSSTLPPSNSNMVSTRINR
QQSSDLHSSSHSNSSNSQGSFGCSPGSQIVANVALNQGQASSQSSNPSLNLNNSPMEGTG
ISLAQFMSPRRQVTSGLATRPRMPNNSFPPNISTLSSPVGMTSSACNNNNRSYSNIPVTS
LQGMNEGPNNSVGFSASSPVLRQMSSQNSPSRLNIQPAKAESKDNKEIASILNEMIQSDN
SSSDGKPLDSGLLHNNDRLSDGDSKYSQTSHKLVQLLTTTAEQQLRHADIDTSCKDVLSC
TGTSNSASANSSGGSCPSSHSSLTERHKILHRLLQEGSPSDITTLSVEPDKKDSASTSVS
VTGQVQGNSSIKLELDASKKKESKDHQLLRYLLDKDEKDLRSTPNLSLDDVKVKVEKKEQ
MDPCNTNPTPMTKPTPEEIKLEAQSQFTADLDQFDQLLPTLEKAAQLPGLCETDRMDGAV
TSVTIKSEILPASLQSATARPTSRLNRLPELELEAIDNQFGQPGTGDQIPWTNNTVTAIN
QSKSEDQCISSQLDELLCPPTTVEGRNDEKALLEQLVSFLSGKDETELAELDRALGIDKL
VQGGGLDVLSERFPPQQATPPLIMEERPNLYSQPYSSPSPTANLPSPFQGMVRQKPSLGT
MPVQVTPPRGAFSPGMGMQPRQTLNRPPAAPNQLRLQLQQRLQGQQQLIHQNRQAILNQF
AATAPVGINMRSGMQQQITPQPPLNAQMLAQRQRELYSQQHRQRQLIQQQRAMLMRQQSF
GNNLPPSSGLPVQMGNPRLPQGAPQQFPYPPNYGTNPGTPPASTSPFSQLAANPEASLAN
RNSMVSRGMTGNIGGQFGTGINPQMQQNVFQYPGAGMVPQGEANFAPSLSPGSSMVPMPI
PPPQSSLLQQTPPASGYQSPDMKAWQQGAIGNNNVFSQAVQNQPTPAQPGVYNNMSITVS
MAGGNTNVQNMNPMMAQMQMSSLQMPGMNTVCPEQINDPALRHTGLYCNQLSSTDLLKTE
ADGTQQVQQVQVFADVQCTVNLVGGDPYLNQPGPLGTQKPTSGPQTPQAQQKSLLQQLLT
E

>Gorilla gorilla_NCOA1(ENSGGOP_11007)
MSGLGDSSSDPANPDSHKRKGSPCDTLASSTEKRRREQENKYLEELAELLSANISDIDSL
SVKPDKCKILKKTVDQIQLMKRMEQEKSTTDDDVQKSDISSSSQGVIEKESLGPLLLEAL
DGFFFVVNCEGRIVFVSENVTSYLGYNQEELMNTSVYSILHVGDHAEFVKNLLPKSLVNG

VPWPQEATRRNSHTFNCRMLIHPPDEPGTENQEACQRYEVMQCFTVSQPKSIQEDGEDFQ
SCLICIARRLPRPPAITGVESFMTKQDTTGKIISIDTSSLRAAGRTGWEDLVRKCIYAFF
QPQGREPSYARQLFQEVMTRGTASSPSYRFILNDGTMLSAHTKCKLCYPQSPDMQPFIMG
IHIIDREHSGLSPQDDTNSGMSIPRVNPSVNPSISPAHGVARSSTLPPSNSNMVSTRINR
QQSSDLHSSSHSNSSNSQGSFGCSPGSQIVANVALNQGQASSQSSNPSLNLNNSPMEGTG
ISLAQFMSPRRQVTSGLATRPRMPNNSFPPNISTLSSPVGMTSSACNNNNRSYSNIPVTS
LQGMNEGPNNSVGFSASSPVLRQMSSQNSPSRLNIQPAKAESKDNKEIASILNEMIQSDN
SSSDGKPLDSGLLHNNDRLSDGDSKYSQTSHKLVQLLTTTAEQQLRHADIDTSCKDVLSC
TGTSNSASANSSGGSCPSSHSSLTERHKILHRLLQEGSPSDITTLSVEPDKKDSASTSVS
VTGQVQGNSSIKLELDASKKKESKDHQLLRYLLDKDEKDLRSTPNLSLDDVKVKVEKKEQ
MDPCNTNPTPMTKPTPEEIKLEAQSQFTADLDQFDQLLPTLEKAAQLPGLCETDRMDGAV
TSVTIKSEILPASLQSATARPTSRLNRLPELELEAIDNQFGQPGTGDQIPWTNNTVTAIN
QSKSEDQCISSQLDELLCPPTTVEGRNDEKALLEQLVSFLSGKDETELAELDRALGIDKL
VQGGGLDVLSERFPPQQATPPLIMEERPNLYSQPYSSPSPTTNLPSPFQGMVRQKPSLGT
MPVQVTPPRGAFSPGMGMQPRQTLNRPPAAPNQLRLQLQQRLQGQQQLIHQNRQAILNQF
AATAPVGISMRSGMQQQITPQPPLNAQMLAQRQRELYSQQHRQRQLIQQQRAMLMRQQSF
GNNLPPSSGLPVQMGNPRLPQGAPQQFPYPPNYGTNPGTPPASTSPFSQLAANPEASLAN
RNSMVSRGMTGNMGGQFGTGINPQMQQNVFQYPGAGMVPQGEANFAPSLSPGSSMVPMPI
PPPQSSLLQQTPPASGYQSPDMKAWQQGAIGNNNVFSQAVQNQPTPAQPGVYNNMSITVS
MAGGNTNVQNMNPMMAQMQMSSLQMPGMNTVCPEQINDPALRHTGLYCNQLSSTDLLKTE
ADGTQQVQQVQVFADVQCTVNLVGGDPYLNQPGPLGTQKPTSGPQTPQAQQKSLLQQLLT
E

>Mus musculus_NCOA1(ENSMUSP_82971)
MSGLGDSSSDPANPDSHKRKGSPCDTLASSTEKRRREQENKYLEELAELLSANISDIDSL
SVKPDKCKILKKTVDQIQLMKRMEQEKSTTDDDVQKSDISSSSQGVIEKESLGPLLLEAL
DGFFFVVNCEGRIVFVSENVTSYLGYNQEELMNTSVYSILHVGDHAEFVKNLLPKSLVNG
VPWPQEATRRNSHTFNCRMLIHPPEDPGTENQEACQRYEVMQCFTVSQPKSIQEDGEDFQ
SCLICIARRLPRPPAITGVESFMTKQDTTGKIISIDTSSLRAAGRTGWEDLVRKCIYAFF
QPQGREPSYARQLFQEVMTRGTASSPSYRFILNDGTMLSAHTKCKLCYPQSPDMQPFIMG
IHIIDREHSGLSPQDDSNSGMSIPRINPSVNPGISPAHGVTRSSTLPPSNNNMVSARVNR
QQSSDLNSSSSHTNSSNNQGNFGCSPGNQIVANVALNQGQAGSQSSNPSLNLNNSPMEGT
GIALSQFMSPRRQANSGLATRARMSNNSFPPNIPTLSSPVGITSGACNNNNRSYSNIPVT
SLQGMNEGPNNSVGFSAGSPVLRQMSSQNSPSRLSMQPAKAESKDSKEIASILNEMIQSD
NSDNSANEGKPLDSGLLHNNDRLSEGDSKYSQTSHKLVQLLTTTAEQQLRHADIDTSCKD
VLSCTGTSSSASSNPSGGTCPSSHSSLTERHKILHRLLQEGSPSDITTLSVEPEKKDSVP
ASTAVSVSGQSQGSASIKLELDAAKKKESKDHQLLRYLLDKDEKDLRSTPNLCLDDVKVK
VEKKEQMDPCNTNPTPMTKPAPEEVKLESQSQFTADLDQFDQLLPTLEKAAQLPSLCETD
RMDGAVTGVSIKAEVLPASLQPTTARAAPRLSRLPELELEAIDNQFGQPGAGDQIPWANN
TLTTINQNKPEDQCISSQLDELLCPPTTVEGRNDEKALLEQLVSFLSGKDETELAELDRA
LGIDKLVQGGGLDVLSERFPPQQATPPLMMEDRPTLYSQPYSSPSPTAGLSGPFQGMVRQ
KPSLGAMPVQVTPPRGTFSPNMGMQPRQTLNRPPAAPNQLRLQLQQRLQGQQQLMHQNRQ
AILNQFAANAPVGMNMRSGMQQQITPQPPLNAQMLAQRQRELYSQQHRQRQIIQQQRAML
MRHQSFGNNIPPSSGLPVQMGTPRLPQGAPQQFPYPPNYGTNPGTPPASTSPFSQLAANP
EASLATRSSMVNRGMAGNMGGQFGAGISPQMQQNVFQYPGPGLVPQGEATFAPSLSPGSS
MVPMPVPPPQSSLLQQTPPTSGYQSPDMKAWQQGTMGNNNVFSQAVQSQPAPAQPGVYNN
MSITVSMAGGNANIQNMNPMMGQMQMSSLQMPGMNTVCSEQMNDPALRHTGLYCNQLSST
DLLKTDADGNQDKKTEEFFSVVTTD

>Rattus norvegicus_NCOA1(ENSRNOP_71037)
MSGLGDSSSDPANPDSHKRKGSPCDTLASSTEKRRREQENKYLEELAELLSANISDIDSL
SVKPDKCKILKKTVDQIQLMKRMEQEKSTTDDDVQKSDISSSSQGVIEKESLGPLLLEAL
DGFFFVVNCEGRIVFVSENVTSYLGYNQEELMNTSVYSILHVGDHAEFVKNLLPKSLVNG
VPWPQEATRRNSHTFNCRMLIHPPDEPGTENQEACQRYEVMQCFTVSQPKSIQEDGEDFQ
SCLICIARRLPRPPAITGVESFMTKQDTTGKIISIDTSSLRAAGRTGWEDLVRKCIYAFF
QPQGREPSYARQLFQEVMTRGTASSPSYRFILNDGTMLSAHTKCKLCYPQSPDMQPFIMG
IHIIDREHSGLSPQDDTNSGMSIPRINPSVNPGISPAHGVTRSSTLPPSNNNMVSARANR
QQSTDLNSGSSHSNSSSSQGSFGCSPGNQIVASVALSQGQAGSQSSNPSLNLNNSPMEGT
GISLAQFMSPRRQANSGLATRARMSNNSFPPNIPTLSSPVGITSGACSNNNRSYSNIPVT
SLQGMNEGPNNSVGFSAGSPVLRQMSSQNSPSRLSMQPAKAESKDNKEIASILNEMIQSD
NSANEGKPLDSGLLHNNDRLSDGDSKYSQTSHKLVQLLTTTAEQQLRHADIDTSCKDVLS

CTGTSSSASSNPSGGTCPSSHSSLTERHKILHRLLQEGSPSDITTLSVEPEKKDSASAST
AMSVSGQAQGSASIKLELDASKKKESKDHQLLRYLLDKDEKDLRSTPNLSLDDVKVKVEK
KEQMDPCNTNPAPVTKPAPEEVKLESQSQFSADLDQFDQLLPTLEKAAQLPGLCETDRMD
GAVPGVNIKSEILPASLQPTPARSAPRLNRLPELELEAIDNQFGQPGAGDQIPWANNTVT
AINQSKPEDQCISSQLDELLCPPTTVEGRNDEKALLEQLVSFLSGKDETELAELDRALGI
DKLVQGGGLDVLSERFPPQQATPPLMMEDRPTLYPQPYSSPSPTAGLSGPFQGMVRQKPS
LGTMPVQVTPPRGTFSPNMGMQPRQTLNRPPAAPNQLRLQLQQRLQGQQQLMHQNRQAIL
NQFAANAPVGMNMRSGMQQQITPQPPLNAQMLAQRQRELYSQQHRQRQIIQQQRAMLMRH
QSFGNNIPPSSGLPVQMGNPRLPQGAPQQFPYPPNYGTNPGTPPASTSPFSQLAANPEAS
LATRSSMVNRGMAGNMGGQFGTGISPQMQQNVFQYPGSGLVPQAEANFAPSLSPGSSMVP
MPIPPQSSLLQQTPPTSGYQSPDMKAWQQGTMGNNNVFSQAVQSQPAPAQPGVYNNMSIT
VSMAGGNANVQNMNPMMGQMQMSSLQMPGMNTVCSEQMNDPALRHTGLYCNQLSSTDLLK
TEADGNQDKKTEEFFSVVTTD

>Canis familiaris_NCOA1(ENSCAFP_6049)
MSGLGDSSSDPANPDSHKRKGSPCDTLASSTEKRRREQENKYLEELAELLSANISDIDSL
SVKPDKCKILKKTVDQIQLMKRMEQEKSTTDDEVQKSDISSSSQGVIEKESLGPLLLEAL
DGFFFVVNCEGRIVFVSENVTSYLGYNQEELMNTSVYSILHVGDHAEFVKNLLPKSLVNG
VPWPQEATRRNSHTFNCRMLIHPPDEPGTENQEACQRYEVMQCFTVSQPKSIQEDGEDFQ
SCLICIARRLPRPPAITGVESFMTKQDTTGKIISIDTSSLRAAGRTGWEDLVRKCIYAFF
QPQGREPSYARQLFQEVMTRGTASSPSYRFILNDGTMLSAHTKCKLCYPQSPDMQPFIMG
IHIIDREHSGLSPQDDTNSGMSIPRVNPSVNSSISPAHGVARSSTLPPSSNNMVSPRVNR
QQSSDLHSSSHSNSSNSQGSFGCSPGNQIVASVALNQGQASSQSTNPSLNLNNSPMEGTG
ISLAQFMSPRRQVSSGLAPRPRMPNNSFPPNIPTLSSPVSMTGTACNNSNRSYSNIPVTS
LQSMNEGPNNSVGFSASSPVLRQMSSQNSPSRLNIQPAKAESKDNKEIASILNEMIQSDN
SSTDGKPLDSGLLHNNDRLSEGDNKYSQTSHKLVQLLTTTAEQQLRHADIDTSCKEVLSC
SGTSNSASANSSSGSCPSSHSSLTERHKILHRLLQEGSPSDITTLSVEPDKKDGASTSVS
VTGQVQGNSSVKLELDASKKKESKDHQLLRYLLDKDEKDLRSTPNLSLDDVKVKVEKKEQ
MDPCTTNPTPMTKPAPEEIKLESQSQFTADLDQFDQLLPTLEKAAQLPGLCEPERMDGAV
TNVTIKSETLPASLQSTTARPTSRLNRLPELELEAIDNQFGQPGTGDQIPWANNTVTAVN
QNKPEDQCISSQLDELLCPPTTVEGRNDEKALLEQLVSFLSGKDETELAELDRALGIDKL
VQGGGLDVLSERFPPQQATPPLMMEERPNLYSQPYSSPSPTANLSSPFQGMVRQKPSLGT
MPVQVTPPRGAFSPGMGMQPRQTLNRPPAAPNQLRLQLQQRLQGQQQLIHQNRQAILNQF
AANAPVGINMRSGMQQQITPQPPLNAQMLAQRQRELYSQQHRQRQLIQQQRAMLMRQQSF
GNNLPPSSGLPVQMGNPRLPQGAPQQFPYPPSYGTNPGTPPASTSPFSQLAANPESTLAN
RNSMVNRGMAGNMGGQFGTGINPQMQQNVFQYPGSGMVPQGEANFAPSLSPGNSMVPMPI
PPPQSSLLQPTPPSSGYQSPDMKAWQPGAMGNNSVFSQAVQNQPTPAQPGVYNNMSITVS
MAGGNTNVQNMNPMMGQMQMSSLQMPGMNTVCPEQINDPALRHTGLYCNQLSSTDLLKTE
TDGTQVQQVQVFADVQCTVNLVGGDPYLNQPGPLGTQKPTAGPQTPQAQQKSLLQQLLTE

>Felis catus_NCOA1(ENSFCAP_22579)
MSGLGDSSSDPANPDSHKRKGSPCDTLASSVTNSTEKRRREQENKYLEELAELLSANISD
IDSLSVKPDKCKILKKTVDQIQLMKRMEQEKSTTDDEVQKSDISSSSQGVIEKESLGPLL
LEALDGFFFVVNCEGRIVFVSENVTSYLGYNQEELMNTSVYSILHVGDHAEFVKNLLPKS
LVNGVPWPQEATRRNSHTFNCRMLIHPPDEPGTENQEACQRYEVMQCFTVSQPKSIQEDG
EDFQSCLICIARRLPRPPAITGVESFMTKQDTTGKIISIDTSSLRAAGRTGWEDLVRKCI
YAFFQPQGREPSYARQLFQEVMTRGTASSPSYRFILNDGTMLSAHTKCKLCYPQSPDMQP
FIMGIHIIDREHSGLSPQDDTNSGMSISRVNPSVNSSISPAHGVARSSTLPPSSNNMVSP
RVNRQQSSDLHSSSHSNSSNSQGSFGCSPGNQIVASVALNQGQASSQSTNPSLNLNNSPM
EGTGISLAQFMSPRRQVSSGLATRPRMPNNSFPPNIPTLSSPVGMTGTTCNNSNRSYSNI
PVTSLQSMNEGPNNSVGFSASSPVLRQMSSQNSPSRLNIQPTKAESKDNKEIASILNEMI
QSDNSSSDGKPLDSGLLHNNDRLSDGDNKYSQTSHKLVQLLTTTAEQQLRHADIDTSCKE
VLSCSGTSGSASANSSSGSCPSSHSSLTERHKILHRLLQEGSPSDITTLSVEPDKKDSAS
TSVSVTGQVQGNSSVKLELDASKKKESKDHQLLRYLLDKDEKDIRSTPNLSLDDVKVKVE
KKEQMDPCNTNPTPMTKPAPEEIKLESQSQVGSDVSEMGEKCEAVDKTVSSGLLCYSISF
EAFSEAVFLLSLQPLSEEGLRVFCTNRKSRLPELELEAIDNQFGQPGTGDQIPWANNTVT
TVNQNKPEDQCISSQLDELLCPPTTVEGRNDEKALLEQLVSFLSGKDETELAELDRALGI
DKLVQGGGLDVLSERFPPQQATPPLMMDERPNLYSQPYSSPSPTANLSSPFQGMVRQKPS
LGTMPVQVTPPRGAFSPSMGMQPRQTLNRPPAAPNQLRLQLQQRLQGQQQLIHQNRQAIL
NQFAANTPVGINMRSGMQQQITPQPPLNAQMLAQRQRELYSQQHRQRQLIQQQRAMLMRQ
QSFGNNLPPSSGLPVQMGNPRLPQGAPQQFPYPPNYGTNPGTPPASTSPFSQLAANPEST

LANRNSMVNRGMTGNMGGQFGTGINPQMQQNVFQYPGSGMVPQGEASFAPSLSPGSSMVP
MPIPPPQSSLLQQTPPTSGYQSPDMKAWQQGAMGNNNVFSQAVQSQPTPAQPGVYNNMSI
TVSMAGGNTNVQNMNPMMGQMQMSSLQMPGMNTVCPEQINDPALRHTGLYCNQLSSTDLL
KTETDGTQQVQQVQVFADVQCTVNLVGGDPYLNQPGPLGTQKPTAGPQTPQAQQKSLLQQ
LLTE

>Monodelphis domestica_NCOA1(ENSMODP_29006)
MSGLGDSSTDPSNPDSHKRKGSPCDTLASSTEKRRREQENKYLEELAELLSANIGDIDSL
SVKPDKCKILKKTVDQIQQMKRMEQEKTATDDDVQKSDISSSSQGMIEKESLGPLLLEAL
DGFFFVVNREGRIVFVSENVTSYLGYNQEELMNTSVYSILHVGDHAEFVKNLLPKSLVNG
VPWPQEATRRNSHTFNCRMLIRPPDEPGTENQEACQRYEVMQCFTVSQPKSIQEEGEDFQ
SCLICIARRLPRPAAITGSESFMTKQDTTGKIISIDTSSLRAAGRTGWEDLVRKCIYAFF
QPQGREPSYARQLFQEVMTRGTAFSPSYRFILNDGTMLSAHTKCKLCYPQSPDMQPFIMG
IHIIDREHNVLSPQDNTNSGMSLPRINPSVNPSVSPAQSLARSSTLPPSSNNMVTPRVNH
QQSPDLHSNSSNTNSNNSQGSFGCSAGNQVVASIALNQGQAGSQGSNHSLSLNNSPMDST
GITLAQFMSPRRQVNSGLVTRPRMPNNSFSPNISSLSNPIGMVGSACGNPNRTYSNISAA
SLQGINEGPSNSIGFSATSPVLRQLSSQNSPSRMNMLPTKAESKESKEITSILNEMVQSD
NSSNDVKPLDSGLLHSTDKLSEGDNKYSQTSHKLVQLLATTAEQQLRHTDADTSGKDSLS
CSGTSTATSINSSSSGNTCPSSHSSLTERHKILHRLLQEGSPSDITTLSVEHEKKDNGSG
SVNNQVPGNANIKLELEASKKKESKDHQLLRYLLDKDEKDLRAAPTLSLDDVKVKVEKKE
QMDPCNTTPTTLTKPTPEEIKLETQNQFPADLDQFDPLLPTLEKASQLPGLCESERMEGG
GTGVAIKSEILPTALQSTPVRPPPRLNRLPELELSGLDHQFGQPTIDDQVPWTNNTMTAM
NQGPLNKPEDQWVKKVLGEGTFTETWIAGSSREAVLPALRVAVVSDKPALCCGELRGHLF
YNREKRGGGFEGRRKRLSFKDLFSFFTSPNSTSIPTHASRGPQIDKMRDKRQLCLLRAKP
LQPPQSSDSAIFKETIQVPSPQFSHLPITLFHFLPVFLQLIHQNRQAILNQFAASAPVGI
NMRSGMQQQITPQPPLNAQMLAQRQRELYSQQHRQRQLIQQQRALLMRQQSFGNSLPPSS
GLPVQMGTSRLPQGAPQQFPYPSSYGTNPGTPPTSTSPFSQLASNPEASLASRNNMVNRG
ITGNVGGQFGTGINPQMQQSVFQYPGSGLGQQSEPNFTPSLSPGSPMMSLQIPPSQSPML
QQTQTQPTSGYQSPDMKTWQQGAMGNNNMFSQTQPAPAQPGVYNNMSITVSMAGGNTNVQ
NMNPMMGQMQMSSLQMPGMNTVCSEQINDPALRHTGLYCNQLSSADILKTESDSGAQQVQ
QVQVFADVQCTVNLVGGDPYLNQPGPLGSQKPTSGPQNPQAQQKSLLQQLLTE

>Gallus gallus_NCOA1(ENSGALP_26767)
MSGLGDSSTDPANPDSRKRKGSPCDTAQSNEKRRREQENKYLEELAELLSANIGDIDTLS
VKPDKCKILKKTVDQIQQMKRLEQEKAADDEVQKSDISSSSQGVIEKESLGPLLLEALDG
FFFVVNREGRIVFVSENVTSYLGYNQEELMNTSVYSILHVGDHTEFVKNLLPKSLVNGVP
WPQEATRRNSHTFSCRMLIRPPDEPGTENQEARQRYEVMQCFTVSQPKSFKEEGEDFQSC
LICIARRLPRPAAVTPSESFVTKQDTTGKIISIDTSSLRAAGRTGWEDLVRKCIYAFFQP
QGREPSYAKQLFQEVMTRGTAFSPSYRFTLSDGTVLSAHTKCKLCYPSSPEVQPFIMGIH
IIDRDHGMLSPQENTNSAMALPRVSPSLNPNISPGQGMALPPSIPPSNGSMLATPGNQQP
GAGLHSSNSSTSQTSFGCSPGNQIGANVALSQGQAGPPNSNHNLNLSSSPMNSPGITPPQ
FMSPRHRVSPGLVPRPRGPSNPFSPTMPTMHSPVGMANSGGGGNGGGGGGGSRSFPTAPI
NSLQGLSEGASTPVGYSTPPPVLRQLGSQSSPGRLGMQPMKAESKDSKEIASILSEMIQS
DGSAGDGKPLDSGVLHASERLTEGESKCSQATSHKLVQLLASTAEQQLRHADADTSCKDS
LACAGTTGTLGASNPPSSTCPSSHSSLTERHKILHRLLQEGSPSDITTLAMEHDKKDNVP
NPATTPTAPSQLPGTQDIKLESDAMKKKDVKDHQLLRYLLDKDEKELAAAPALSLDDVKV
KVEKTEQMEPCNTTPVPLAKPPAAEEVKLESQGQFAAELEQLDQLLPSLEKAAQLPGLCG
PERSESGVAVKSEMLAAPLQPSTAPRAPTGLNPLSSGQGMQAARAPFEFCDPLEPAQLRP
VAFPTTNGSSPRPRAPAEQGRGTVPGPNPFPPDTGMSELEMGVPDHQFGQPGMGEQMPWT
DNSMAPMNRGTLSKPEDPCITSQLDELLCPPTTPEGRNDEKALLEQLVSFLSGKDETELA
ELDRALGIDKLVQGGGLEALTERFQPQQATPPLMMEQKPGMYPQPYSSASPTTNLPAAFP
GMVRQKPSFGPMPVQVPPPRGAFPTTMAMQPRQALSRPPTAPNQLRLQLQQRLQGQQQLI
HQNRQAILNQFAANSPVGINMRAGMQQQMTPQPPLNAQMLAQRQRELYSQQHRQRQLMQQ
RAILMRQQSFGNNLPPSAGLPVQMGAARLPQAPPQQFPYPPNYGTSPGNPPTSTSPFSQL
ASSPEAALANRGGMVTRGMMGSVGGQFGTGMTPQMQQNIFQYSGSGMGQQNEPTFAPSLS
PSSPLMSPQLPPSQSPMLQPAPPAPGYQSPDMKTWQQGAMGNSNVFSQTGQTQPAPAQQG
MYNNMSITVSMAGGNTNVQNMNPMAGQMQMNSLQMPGMNSMCSEQVNDPALRPTGLYCNQ
LSSTDLLKTEADGAQQVQQVQVFADVQCTVNLVGDPYLNPPAPLGAPKAAAVPPTPQGQQ
KSLLQQLLTE

>Anolis carolinensis_NCOA1(ENSACAP_770)

MSGLGNNSMDPANPDSQKRKGSPCDTLAQSNEKRRREQENKYLEELADLLSANIGEIDSL
SVKPDKCKILKKTVDQIQQMKRLEQEKTADDDVQKSDISSSSQGVIEKESLGPLLLEALD
GFFFVVNREGRIVFVSENVTSYLGYNQDELMNTSVYSILHVGDHAEFVKNLLPKSLVNGV
PWPQESTRRNSHTFNCRMLTRPPDESAAENQEARQRYEVMQCFTVSQPKLIKEEGEDLQS
CLICIARRLPRSTVPTTSESFMTKQDTTGKIISIDTSSLRAAGRTGWEDLVRKCIYAFFQ
PQGREPSYAKQLFQEVMTRGTAFSPSYRFTLSDGTILSAHTKCRLCCPQSPEMQPFIMGI
HIIEREHGPLSPQDNTNSGMPVPRVNPALNPNISPGQGMPLSTPLPPTNNGNMLPSNPGH
PPGPGLHANSGGAAATGPAGFGCSPGSQMGPNVALSQGQAAPQNSSHAMNLSSSPMNSPG
VTPPQFMSPRHRVSPGLVHRPRVPSNPFSPTMPTTHSPVGMMGGCGVASAVGNRPYPGTP
LNSMQGLREGPSPPVGYSTPSPVLRQLSNQNSPSRMSMPPMKTEPKDSKEMGSIMCGMAH
SDSVSGDSKPLDSSLLHGGDRLSEGDSKCLQATSQKLVQLLATTAQEQLRHADADTSCKE
PLSCTGTSGTPSSGNPVGSACPSSHSSLTERHKILHRLLQEGSPSDINSLAMEHDKKDGV
AGGNTAAVAAQNQGPGTPDMRLEADAMKKKESKDHQLLRYLLDKDEKELASNPTLSLDDV
KVKENPEQLESCSITPKQEDVKLQPQGQFASDLDQILPSLEKAAQLPPGLCGSETAESGG
ISIKSEILTPSLQPTTTARTPSGMSSVNGGQGMQAGRSPYEICDPLEPVQPRTAPFPPMN
GSPHPGLPPELARGAMSGPTPYQPDTGLPELELGVTDHQFGQPTMGEQLPWADNAMTSIN
QGPISKPDDHCITSQLDELLCPPTTPEGRNDEKALLEQLVSFLSGKDENELAELDRALGI
DKLVQGGGLEALTERFQPQQAAPAPPPMAMDQKPSLYPQPYPSASPNANLAGPFQGMVRQ
KQSFGPMPVQVPPPRGGFPTNMGMQPRQALSRPPTAPNQLRLQLQQRLQGQQQLIHQNRQ
AILNQFAGNPPGSMGMRPSMQQQIAGQPPLNAQMLAQRQRELYSQQHRQRQLMQQRAMLM
RQQQSFGSSLPPSAGVPVPMGAGRLPQAPPQQFPFPPNYGTNPGNPPASTSPFSQLAASN
PEAALASRNNMVNRGMMGSVGGQFGTNMSPQMQQNIFQYSGSGMGQQGEAAFAPSLSPGS
PLMSPQIPPSQSPMLQSAPPTPGYQSPDMKTWQQGAMGNNSVFGQAGQSQPSPAQQGMYN
NMSITVSMAGGNANVQNMNPMAGQMPMSSLQMPGMNQLCPEQVNDPSLRPSGLYCNQLSS
DLLKTEADGAQGKKTEEFFFSVVTTG

>Xenopus tropicalis_NCOA1(ENSXETP_45770)
MSGLGDSSADPASQDRKRKALPCDTAQSTEKRRREQENKYLEELAELLSANIGDIDSLSV
KPDKCKILIKRMDQIQQLKKKKQEKALGADDDVQKSDISSSSGGVIEKESLGPLLLEALD
GFFFVVNQEGRIVFVSENVTNYLGYKQEELMKDSIYSILHVGDHEEFVKNLLPKSLVNGV
PWSQEASRRNSHTLNCRMLIRPPDDAGVENQEARQRYEVMQCFTVSQPKSFKEEGEDFQS
CLICIARRLPRPAPITSLESFITKQDATGKIISIDTSSLRASGSLGWEDLVRKCIYAFFQ
AQGTEPSYAKQLFQEVMTRGTACSPSYKFTLSDGTELSAHTKCKLCYPQSPDVQPYIMGI
HIIERDQSSLCTQENTNSGMSLPRLSPSVNLTITPTQGMPFSSTTSPSSGGMLSSGINQH
QVAGLHNSNSSNTSQANFGCSPGSQIGTNVALSLGQAGPQNSNNLNLNNSPMSSPGISPP
QFMSPRPRASPGLVSRSRASGNPFSPPMPAMHSPVGISNSICNNNNNRPFSNVPLNSMPV
VNDPNTPLGFSTPSPGMTNNVSTTNNGRLFSNTPMNTVQGINEGPNPTVGYSTTSPVLRQ
LNSQNSPGRLNMQPIKMEPKDNSGNSDRTVEGETKPSLATSSNKLVQLLATTAEQQLRDV
DTNCRDPLTCHVVTNTSCGSSSSGTCPSSHSSLTERHKILHRLLQEGSPSDISSLSIDHE
KKNTGSANNQTPGGPPEVKMESESDKKKDSKDHQLLRYLLDKDEKEVKSSSALTLEDVKV
KVEKEQVDQCSSTPTTLIKAGQEDIKLETQNQFADDFDQLDQLLPTLDKAAQLPGMVPDR
TDNSVPLKTEMLPSSLQTGTVARTPTRLNNFNGMNTNQSPFGFCDPMDPVQLDLNPFPAP
SDTSPRSGLTPDQGRGVTHDATAFQSDPVLPSMLPLILLQAPPFNIKALGLAVHWRILLA
KLLLGVIIFLSRIDDFLCPPTTAEGRNDEKALLEQLVSFLSGKDENELAELDRALGIDKL
VQGGGLDVVNDRFPQQSIAPVIMEQKPGLYPQQYPSASPNPGLPFFNMVRQKAAFGSVPV
QVQPPQARGTYPNTMGMQPRQALARSVTTSNQLRLQLQQRLQGQQQVLHQNRQANLNQFG
VVNTVAMGMRPGMQQAISTQQPPLNAQMLAQRQRELYSQQHRQRQLMQQRSILMRQQGFG
NSLAPAGSLPVTMGAGRLPQGPPQQFPYPHSYGTNPGNPPPSTSPFSPLATTPEATLANR
GGGNGNGIVNRGMMGNVGGQFGSGINPQMQQNVFQYSATGISQQGDPTFAPSLSPTSPLM
SPRITPSQSPMLQQTPSTPGYQSPDLKTWQQSPLGNSGVFSQGGQTQPPPGQQGMYNNMS
ITVSMGGGNTGVQNVNQMTGQMQMSSLPMSNMNAMCTEQQVSDPALRPSGLYCNQLSSTD
LLKTEPETGQVQQVQVFADVQCTVNLVGGDPYINQPGSITSQKATTGPQTPQAQQKSLLQ
QLLTE

>Danio rerio_NCOA1(ENSDARP_45947)
MLKMSAAGENALDPNILESRKRKGSPCDTSGPSVEKRRRELECRYIDELAELLSANMSDI
ASLNVKPDKCHILKSTVDQIQQIKRREQENAALMSPNDEVQQSDISSSSHGMVEKEALGP
LLLEALDGFFFVVNREGRIVFVSENVTGYLGYTQEELMTSSVYSILHVGDHNEFVRILLP
KSLVNGVPWSQEQGRRNSHTFNCRLLKRPPDEVDSENQEARQQYELMQCFTVSQPKALQE
EGEDLQSCLICIACQIPRPPQIPVSTESFITKQDPTGKIISIETSALRATGRPGWEDLVR
KCIYAFFQPQGNEPSHAKKLLHEVMTHGMAISPLYHFTLSDGTSLSAQTRCKFCCPQNPD

VQPFIMGFHTIDREHNTASSQENTTPSLSSPAAPPSRSPALQPSSDLSIHLNNTNGTCAT
PGPLTPTPPGYLTPSRVGPSQQVSSPSPLGSPLTATPTSFMSPRPPRGSPGLASSPRVPG
HPFSPSTPSIHSPAGGLTRQQSGGDGVGFSLPSPLPPRQTPTPSSSPARQPPAKPPEASN
DEPKSNQVGNPKLSQLLDGASAPEQDSQPRPSPTSQCPASHSSLTERHKILHRLLQDNSP
ADAGKEPDIKKEPPTSPNSALIARGLGKEPQDHQLLRFLLDTDEKDLEDLPPPATLSLQT
VKVKAEKKGSRENVSCAAALANNAMPGSPQSSGKASPQFPSTDLDTLNQLLPTLRASVGG
KTVEEPSEDSLSIGVNVKRESPGTSSQAFPDGENLSSQTSFDFCDPSTPNQGQSQVQVSD
LGQTELFQPPKESSSPFASPTSHNSFNTNTGLAKLELPDSQQFQALPLVEPMTFDNSLAN
QTQAPLSSPQEQCVPCPLDEMLCPPTTPEGRNDEKALLEQLVTFLSHTDESELAELDRAL
GIDKIVQSGCLEPVTQTPFPQSQPPTPVPLDPKLPSYPSQFPTSPSQFPAEMATAQGMSF
GAPRMPFPGNVVQAGMNRAPGVSNQLRLPPNQLRLQLQQRLQGPQQLQNRLAAMGSFPQG
GHVAMGMRQSIQQPQMPSQPPLNAQMMAQRQREVYSYQHRQRQLLQQKVMLMRQGINTGP
LGAQRLPKGPQQQQQPPPQQQQQFGFPPGYNAVPGNTPTSPSHFNPMGGPLDPKLSVRAG
LGNQGIMGAIQGQFGGPVNTPVQQGLFQQFGGSGMIQQADPSFAPELSPTSPLLSPQNST
SQSPLLQQTQPPPGYQSPDLKTWQQGTVANNGMYNQVGPPVSQSFGQQGVYNNMSITVSM
AGGSGAVNTLPPIGPSVGMGNNNLGNVTTMCNEQVQQVQVFADVQCTVNLVGSDSYLNQS
GPIGSQKGPSGTPSAQSQQKSLLQQLLTE

>Takifugu rubripes_NCOA1a(ENSTRUP_16274)
MSAVGENPLDPATSEARKRKGSPCDTSGPSLEKRRRELECRYIEELAELLSSNMGDIASL
SVKPDKCHILKSTVDQIQQMKRRKQEKAALLSPDDDVQKSDVSSSSQGLVEKEALGPMLL
EALDGFFFVVNREGRIVFVSENVTSYLGYPQEELMTSSVYSILHVGDHSEFVRNLLPKSL
VNGVPWPQEPGRRNSHTFNCRMLKRPPDEVDSENPEARQQYEIMQCFTVSQPQSMQEEGE
DLQTCLICIACRIPRPQPFNTESFITRQDPTGKIISIETSALRATGRPGWEDLVRKCIYA
FFQPQGKEPSLAKKLLHEVMTHGSAVSPLYHFTLSDGTPLSAQTRCKFCCPPNPDVQPFI
MGIHTIDREHNTASSQENTNPSLPQNLGSLGQTPSRSPSLPPGSNWTQGSGVATSGLNPN
SSNGSSQGQNSSTPAGYLTPNRTCPQQLNSPSPLSSPLTAAPTSFISSRMPQVGPGLGES
PRITGNPFSPSTPGLHSPAGGTNSGGSLNRQHSGGDGTSSASSGSSGSFLSSPVHQRQTS
TPTSSSTRPPSVKPSEEGDEDSVKAPLSSASQVGNPRPSQFHDSNGTGSESSNSIHSTSL
PHPPAPTPQCPASHSTLTERHKILHRLLQDSSPNDSSANNEEGISQNPAEIKKEPPPSPA
MTSPIPKQDHQLLRFLLDTDEKDLGDLPPPSALSLQSIRAKVEKRTSIDSAPCQTVMTPK
SPPYALQVLPQMSGGPVDMDTITQLLPCLRGPTGARQGSEDSSTSGGGPQLQSPVPQPSA
QLQSPVSQSLPQLQSPSPQLAPPSPQLKLQSPTQLQPAEASTPCGVNQPPHSKQCVPCTL
DDVLGPSNIPEARNDEKALIEQLVNFLSGTDESELAELDKALGIDKLVQSGCFDPSGQSF
LTQQPAVSPVAMDTKLPSYPSQFTPASQAQLPPELATVGPQGQGFGVPRAAFPGGATGVG
LRQGVMRPQGPQLRLSPNQLRLQLQQRLQAPQQLQNRMASVNQFPGGVQHVNMGIRQGVQ
QPQLPTQQPPLNAQMLAQRQREIYSIQHRQRQLFQQKVMLMRQNMAGAPAGSVGSIGTAQ
APKVAPTTPQQPPQQQFSFPQVYNPLTGKPATSPSPFSPMTGGPLDNKVTVRVPLNNQTL
LGGVQGQFSTVNSSIQQGLFQQFSGSAMAQQEPPFPPEMSPTSPLLSPQNSTPQTPLLQQ
APPPGYQAADTKSWPPTGITSNSLFGQSGQGAGPAFGQQGVYNNMSITVSMAGGSSGVSS
LPPMGQPVAMSNSNLSSVSVVCSDQQSSNFSLLKYCVHQRNVTLTCVMMDLVQQVQQVQV
FADVQCTVNLVGSDSYMNQGSGGTASSQKGPGTQGSQNNPAQQKSLLQQLLTE

>Takifugu rubripes_NCOA1b(ENSTRUP_16273)
MSAVGENPLDPATSEARKRKGSPCDTSGPSLEKRRRELECRYIEELAELLSSNMGDIASL
SVKPDKCHILKSTVDQIQQMKRRKQEKAALLSPDDDVQKSDVSSSSQGLVEKEALGPMLL
EALDGFFFVVNREGRIVFVSENVTSYLGYPQEELMTSSVYSILHVGDHSEFVRNLLPKSL
VNGVPWPQEPGRRNSHTFNCRMLKRPPDEVDSENPEARQQYEIMQCFTVSQPQSMQEEGE
DLQTCLICIACRIPRPQPFNTESFITRQDPTGKIISIETSALRATGRPGWEDLVRKCIYA
FFQPQGKEPSLAKKLLHEVMTHGSAVSPLYHFTLSDGTPLSAQTRCKFCCPPNPDVQPFI
MGIHTIDREHNTASSQENTNPSLPQNLGSLGQTPSRSPSLPPGSNWTQGSGVATSGLNPN
SSNGSSQGQNSSTPAGYLTPNRTCPQQLNSPSPLSSPLTAAPTSFISSRMPQVGPGLGES
PRITGNPFSPSTPGLHSPAGGTNSGGSLNRQHSGGDGTSSASSGSSGSFLSSPVHQRQTS
TPTSSSTRPPSVKPSEEGDEDSVKAPLSSASQVGNPRPSQFHDSNGTGSESSNSIHSTSL
PHPPAPTPQCPASHSTLTERHKILHRLLQDSSPNDSSANNEEGISQNPAEIKKEPPPSPA
MTSPIPKQDHQLLRFLLDTDEKDLGDLPPPSALSLQSIRAKVEKRTSIDSAPCQTVMTPK
SPPYALQVLPQMSGGPVDMDTITQLLPCLRGPTGARQGSEDSSTSGGGPQLQSPVPQPSA
QLQSPVSQSLPQLQSPSPQLAPPSPQLKLQSPTQLQPAEASTPCGVNSENSPAVQCVPCT
LDDVLGPSNIPEARNDEKALIEQLVNFLSGTDESELAELDKALGIDKLVQSGCFDPSGQS
FLTQQPAVSPVAMDTKLPSYPSQFTPASQAQLPPELATVGPQGQGFGVPRAAFPGGATGV
GLRQGVMRPQGPQLRLSPNQLRLQLQQRLQAPQQLQNRMASVNQFPGGVQHVNMGIRQGV

QQPQLPTQQPPLNAQMLAQRQREIYSIQHRQRQLFQQKVMLMRQNMAGAPAGSVGSIGTA
QAPKVAPTTPQQPPQQQFSFPQVYNPLTGKPATSPSPFSPMTGGPLDNKVTVRVPLNNQT
LLGGVQGQFSTVNSSIQQGLFQQFSGSAMAQQEPPFPPEMSPTSPLLSPQNSTPQTPLLQ
QAPPPGYQAADTKSWPPTGITSNSLFGQSGQGAGPAFGQQGVYNNMSITVSMAGGSSGVS
SLPPMGQPVAMSNSNLSSVSVVCSDQQSSNFSLLKYCVHQRNDGSPSSCLQVQQVQQVQV
FADVQCTVNLVGSDSYMNQGSGGTASSQKGPGTQGSQNNPAQQKSLLQQLLTE

>Tetraodon nigroviridis_NCOA1(ENSTNIP_22339)
MSAVGENPLDAATSESRKRKGSPCDTSGQSEKQRSSHEGKWTEETKSFLVAAVGGIFQFX
VKPDKCHILKSTDQIQQMKRREQEKAALLSDDDVQKSDISSSSQGLVEKEALGPMLLEAL
DGFFFVVNREGRIVFVSENVTSYLGYPQEELMTSSVYSILHVGDHSEFVRNLLPKSLVNG
VPWPQEPGRRNSHTFNCRMLKRPPDEVDSENPEARQQYEIMQCFTVSQPQSMQEEGEDLQ
TCLICIACRIPRAQPFNTESFITRQDPTGKIISIETSALRATGRPGWEDLVRKCIYAFFQ
PQAKSPPTSCLIPTVMTHGSAVSPLYHFTLSDGTPLSAQTRCKFCCPPNPDVQPFIMGIH
TIDREHNTASSQENTIPSLPPNLGSLGQTPSRSPSLPPGSNWTQGSGVAASGLNPNSSNG
SSQGQNSSTPPGYLTPNRTQQLNSPSPLSSPLTATPTSFMPSRMPPAGPGLGGSPRITGN
PFSPSTPGLHSPAGGVNSGSSLSRQHSGGDGTSSTSTGSSGSFLSSPVHQRQTSTPTSSS
TRPPSVKPSEGGGDEGEEDSVKAPLSSASQMGNPRPSQFHDSNGTGSESSNSIHSTSLPH
PPNPSPGPQCPASHSTLMERHKILHRLLQDSSPNDSSAVIDEGLDKNPAEIKKEPPPSPA
MTSPIPKQDHQLLRFLLDTDEKDLGDLPPPAALSLQTIRAKVEKRTSIDTAPCTGSAAAA
TCKPAANFGRSSSISSGPVDLDPLAQLLPCLRGPTGARQGSEDSSTPGASTPRSVNVKRE
PPGTPNRGLNDAGPSSGCSVQSQSSFDFCSPPTPSQTPGQGDSFQTPKHTSPFSETDILN
PFSSNTGLNKMDVGDSQFQPLALSDTLSFDALGPSLQAPLASPQEQCVPCTLDDVLGPSS
LPEARNDEKALIEQLVTFLSGTDESELAELDKALGIDKLVQSGCFDSLGQSFPTQQPTVS
PVPMDPKLPSYPSQFTPASQAQLPPELATVGPQGQGFGAPQAAFPVGTTGMGLRPGPMRA
QGPQLRLSPNQLRLQLQQRLQGPQQLQNRMAGVNQFPGVVQHVNIGIRQGVQQPQQPPLN
AQMLAQRQREIYSIQHRQRQLFQQKVMLMRQNMAGAPAGSVGPVGTARAPKVPPATPQQP
PQQQFSFPQAYNPMAGKAPTSPSHFSPMTGAPLDNKLTVRVPLNNQTLLGGVQGQFSSSQ
QGTVSADECLYLFKAMVQQEPPFPPEMSPTSPLLSPQKSTSQTPLLQQAPAPGYQSSDTK
SWQQTGITSNSLFSQPGQGAGQAFGQQGVYNNMSITVSMAGGSGGVSSLPPMGQPVAMST
SNLSNVSSVCSDQQVGHSDSRVVRFRSAFISHIFQQVQVFADVQCTVNLVGSDSYISQGS
VGTAASQKAPGAQGSQNNQAQQKSLLQQLLTE

>Homo sapiens_NCOA2(ENSP_399968)
MSGMGENTSDPSRAETRKRKECPDQLGPSPKRNTEKRNREQENKYIEELAELIFANFNDI
DNFNFKPDKCAILKETVKQIRQIKEQEKAAAANIDEVQKSDVSSTGQGVIDKDALGPMML
EALDGFFFVVNLEGNVVFVSENVTQYLRYNQEELMNKSVYSILHVGDHTEFVKNLLPKSI
VNGGSWSGEPPRRNSHTFNCRMLVKPLPDSEEEGHDNQEAHQKYETMQCFAVSQPKSIKE
EGEDLQSCLICVARRVPMKERPVLPSSESFTTRQDLQGKITSLDTSTMRAAMKPGWEDLV
RRCIQKFHAQHEGESVSYAKRHHHEVLRQGLAFSQIYRFSLSDGTLVAAQTKSKLIRSQT
TNEPQLVISLHMLHREQNVCVMNPDLTGQTMGKPLNPISSNSPAHQALCSGNPGQDMTLS
SNINFPINGPKEQMGMPMGRFGGSGGMNHVSGMQATTPQGSNYALKMNSPSQSSPGMNPG
QPTSMLSPRHRMSPGVAGSPRIPPSQFSPAGSLHSPVGVCSSTGNSHSYTNSSLNALQAL
SEGHGVSLGSSLASPDLKMGNLQNSPVNMNPPPLSKMGSLDSKDCFGLYGEPSEGTTGQA
ESSCHPGEQKETNDPNLPPAVSSERADGQSRLHDSKGQTKLLQLLTTKSDQMEPSPLASS
LSDTNKDSTGSLPGSGSTHGTSLKEKHKILHRLLQDSSSPVDLAKLTAEATGKDLSQESS
STAPGSEVTIKQEPVSPKKKENALLRYLLDKDDTKDIGLPEITPKLERLDSKTDPASNTK
LIAMKTEKEEMSFEPGDQPGSELDNLEEILDDLQNSQLPQLFPDTRPGAPAGSVDKQAII
NDLMQLTAENSPVTPVGAQKTALRISQSTFNNPRPGQLGRLLPNQNLPLDITLQSPTGAG
PFPPIRNSSPYSVIPQPGMMGNQGMIGNQGNLGNSSTGMIGNSASRPTMPSGEWAPQSSA
VRVTCAATTSAMNRPVQGGMIRNPAASIPMRPSSQPGQRQTLQSQVMNIGPSELEMNMGG
PQYSQQQAPPNQTAPWPESILPIDQASFASQNRQPFGSSPDDLLCPHPAAESPSDEGALL
DQLYLALRNFDGLEEIDRALGIPELVSQSQAVDPEQFSSQDSNIMLEQKAPVFPQQYASQ
AQMAQGSYSPMQDPNFHTMGQRPSYATLRMQPRPGLRPTGLVQNQPNQLRLQLQHRLQAQ
QNRQPLMNQISNVSNVNLTLRPGVPTQAPINAQMLAQRQREILNQHLRQRQMHQQQQVQQ
RTLMMRGQGLNMTPSMVAPSGMPATMSNPRIPQANAQQFPFPPNYGISQQPDPGFTGATT
PQSPLMSPRMAHTQSPMMQQSQANPAYQAPSDINGWAQGNMGGNSMFSQQSPPHFGQQAN
TSMYSNNMNINVSMATNTGGMSSMNQMTGQISMTSVTSVPTSGLSSMGPEQVNDPALRGG
NLFPNQLPGMDMIKQEGDTTRKYC

>Pongo abelii_NCOA2(ENSPPYP_24008)

MSGMGENTSDPSRAETRKRKECPDQLGPSPKRNTEKRNREQENKYIEELAELIFANFNDI
DNFNFKPDKCAILKETVKQIRQIKEQEKAAAANIDEVQKSDVSSTGQGVIDKDALGPMML
EALDGFFFVVNLEGNVVFVSENVTQYLRYNQEELMNKSVYSILHVGDHTEFVKNLLPKSI
VNGGSWSGEPPRRNSHTFNCRMLVKPLPDSEEEGHDNQEAHQKYETMQCFAVSQPKSIKE
EGEDLQSCLICVARRVPMKERPVLPSSESFTTRQDLQGKITSLDTSTMRAAMKPGWEDLV
RRCIQKFHAQHEGESVSYAKRHHHEVLRQGLAFSQIYRFSLSDGTLVAAQTKSKLIRSQT
TNEPQLVISLHMLHREQNVCVMNPDLTGQTMGKPLNPISSNSPAHQALCSGNPGQDMTLS
SNINFPINGPKEQMGMPMGRFGGSGGMNHVSGMQATTPQGSNYALKMNSPSQSSPGMNPG
QPTSMLSPRHRMSPGVAGSPRIPPSQFSPAGSLHSPVGVCSSTGNSHSYTNSSLNALQAL
SEGHGVSLGSSLASPDLKMGNLQNSPVNMNPPPLSKMGSLDSKDCFGLYGEPSEGTTGQA
ESSCHPGEQKETNDPNLPPAVSSERADGQSRLHDSKGQTKLLQLLTTKSDQMEPSPLASS
LSDTNKDSTGSLPGSGSTHGTSLKEKHKILHRLLQDSSSPVDLAKLTAEATGKDLSQESS
STAPGSEVTIKQEPVSPKKKENALLRYLLDKDDTKDIGLPEITPKLERLDSKTDPASNTK
LIAMKTEKEEMSFEPGDQPGSELDNLEEILDDLQNSQLPQLFPDTRPGAPAGSVDKQAII
NDLMQLTAENSPVTPIGAQKTALRISQSTFNNPRPGQLGRLLPNQNLPLDITLQSPTGAG
PFPPIRNSSPYSVIPQPGMMGNQGMIGNQGNLGNSSTGMIGSSASRPTMPSGEWAPQSSA
VRVTCAATTSAMNRPVQGGMIRNPAASIPMRPSSQPGQRQTLQSQVMNIGPSELEMNMGG
PQYSQQQAPPNQTAPWPESILPIDQASFAGQNRQPFGSSPDDLLCPHPAAESPSDEGALL
DQLYLALRNFDGLEEIDRALGIPELVSQSQAVDPEQFSSQDSNIMLEQKAPVFPQQYASQ
AQMAQGSYSPMQDPNFHTMGQRPSYATLRMQPRPGLRPTGLVQNQPNQLRLQLQHRLQAQ
QNRQPLMNQISNVSNVNLTLRPGVPTQAPINAQMLAQRQREILNQHLRQRQMHQQQQVQQ
RTLMMRGQGLNMTPSMVAPSGMPATMSNPRIPQANAQQFPFPPNYGISQQPDPGFTGATT
PQSPLMSPRMAHTQSPMMQQSQANPAYQAPSDMNGWAQGNMGGNSMFSQQSPPHFGQQAN
TSMYSNNMNINVSMATNTGGMSSMNQMTGQISMTSVTSVPTSGLSSMGPEQVNDPALRGG
NLFPNQLPGMDMIKQEGDTTRKYC

>Macaca mulatta_NCOA2(ENSMMUP_21016)
MSGMGENTSDPSRAETRKRKECPDQLGPSPKRNTEKRNREQENKYIEELAELIFANFNDI
DNFNFKPDKCAILKETVKQIRQIKEQEKAAAANIDEVQKSDVSSTGQGVIDKDALGPMML
EALDGFFFVVNLEGNVVFVSENVTQYLRYNQEELMNKSVYSILHVGDHTEFVKNLLPKSI
VNGGSWSGEPPRRNSHTFNCRMLVKPLPDSEEEGHDNQEAHQKYETMQCFAVSQPKSIKE
EGEDLQSCLICVARRVPMKERPVLPSSESFTTRQDLQGKITSLDTSTMRAAMKPGWEDLV
RRCIQKFHAQHEGESLSYAKRHHHEVLRQGLAFSQIYRFSLSDGTLVAAQTKSKLIRSQT
TNEPQLVISLHMLHREQNVCVMNPDLTGQTMGKPLNPISSSSPAHQALCSGNPGQDMTLS
SNINFPINGPKEQMGMPMGRFGGSGGMNHVSGMQPTTPQGSNYALKMNSPSQSSPGMNPG
QPTSMLSPRHRMSPGVAGSPRIPPSQFSPAGSLHSPVGVCSSTGNSHSYTNSSLNALQAL
SEGHGVSLGSSLASPDLKMGNLQNSPVNMNPPPLSKMGSLDSKDCFGLYGEPSEGTTGQA
ESSCHPGEQKETNDPNLPPTVSGERADGQSRLHDSKGQTKLLQLLTTKSDQMEPSPLASS
LSDTNKDSTGSLPGSGSTHGTSLKEKHKILHRLLQDSSSPVDLAKLTAEATGKDLSQESS
STAPGSEVTIKQEPVSPKKKENALLRYLLDKDDTKDIGLPEITPKLERLDSKTDSASNTK
LIAMKTEKEEMSFEPGDQPGSELDNLEEILDDLQNSQLPQLFPDTRPGAPAGSVDKQAII
NDLMQLTAENSPVTPVGAQKTALRISQSTFNNPRPGQLGRLLPNQNLPLDITLQSPTGAG
PFPPIRNSSPYSVIPQPGMMGNQGMIGNQGNLGNNSTGMIGSSASRPTMPSGEWAPQSSA
VRVTCAATTSAMNRPVQGGMIRNPAASIPMRPSSQPGQRQTLQSQVMNIGPSELEMNMGG
PQYSQQQAPPNQTAPWPESILPIDQASFASQNRQPFGSSPDDLLCPHPAAESPSDEGALL
DQLYLALRNFDGLEEIDRALGIPELVSQSQAVDPEQFSSQDSNIMLEQKAPVFPQQYASQ
AQMAQGSYSPMQDPNFHTMGQRPSYATLRMQPRPGLRPTGLVQNQPNQLRLQLQHRLQAQ
QNRQPLMNQISNVSNVNLTLRPGVPTQAPINAQMLAQRQREILNQHLRQRQMHQQQQVQQ
RTLMMRGQGLNITPSMVAPSGMPATMSNPRIPQANAQQFPFPPNYGISQQPDPGFTGATT
PQSPLMSPRMAHTQSPMMQQSQANPAYQAPSDMNGWAQGSMGGNSMFSQQSPPHFGQQAN
TSMYSNNMNISVSMATNAGGMSSMNQMTGQISMTSVTSVPTSGLSSMGPEQVNDPALRGG
NLFPNQLPGMDMIKQEGDTTRIYC

>Mus musculus_NCOA2(ENSMUSP_6037)
MSGMGENTSDPSRAETRKRKECPDQLGPSPKRSTEKRNREQENKYIEELAELIFANFNDI
DNFNFKPDKCAILKETVKQIRQIKEQEKAAAANIDEVQKSDVSSTGQGVIDKDALGPMML
EALDGFFFVVNLEGSVVFVSENVTQYLRYNQEELMNKSVYSILHVGDHTEFVKNLLPKSM
VNGGSWSGEPPRRSSHTFNCRMLVKPLPDSEEEGHDSQEAHQKYEAMQCFAVSQPKSIKE
EGEDLQSCLICVARRVPMKERPTLPSSESFTTRQDLQGKITSLDTSTMRAAMKPGWEDLV
RRCIQKFHTQHEGESLSYAKRHHHEVLRQGLAFSQIYRFSLSDGTLVAAQTKSKLIRSQT
TNEPQLVISLHMLHREQNVCVMNPDLTGQAMGKPLNPISSSSPAHQALCSGNPGQDMTLG

SNINFPMNGPKEQMGMPMGRFGGSGGMNHVSGMQATTPQGSNYALKMNSPSQSSPGMNPG
QASSVLSPRQRMSPGVAGSPRIPPSQFSPAGSLHSPVGVCSSTGNSHSYTNSSLNALQAL
SEGHGVSLGSSLASPDLKMGNLQNSPVNMNPPPLSKMGSLDSKDCFGLYGEPSEGTTGQA
EASCHPEEQKGPNDSSMPQAASGDRAEGHSRLHDSKGQTKLLQLLTTKSDQMEPSPLPSS
LSDTNKDSTGSLPGPGSTHGTSLKEKHKILHRLLQDSSSPVDLAKLTAEATGKELSQESS
STAPGSEVTVKQEPASPKKKENALLRYLLDKDDTKDIGLPEITPKLERLDSKTDPASNTK
LIAMKTVKEEVSFEPSDQPGSELDNLEEILDDLQNSQLPQLFPDTRPGAPTGSVDKQAII
NDLMQLTADSSPVPPAGAQKAALRMSQSTFNNPRPGQLGRLLPNQNLPLDITLQSPTGAG
PFPPIRNSSPYSVIPQPGMMGNQGMLGSQGNLGNNSTGMIGSSTSRPSMPSGEWAPQSPA
VRVTCAATTGAMNRPVQGGMIRNPTASIPMRANSQPGQRQMLQSQVMNIGPSELEMNMGG
PQYNQQQAPPNQTAPWPESILPIDQASFASQNRQPFGSSPDDLLCPHPAAESPSDEGALL
DQLYLALRNFDGLEEIDRALGIPELVSQSQAVDAEQFSSQESSIMLEQKPPVFPQQYASQ
AQMAQGGYNPMQDPNFHTMGQRPNYTTLRMQPRPGLRPTGIVQNQPNQLRLQLQHRLQAQ
QNRQPLMNQISSVSNVNLTLRPGVPTQAPINAQMLAQRQREILNQHLRQRQMQQQVQQRT
LMMRGQGLNVTPSMVAPAGLPAAMSNPRIPQANAQQFPFPPNYGISQQPDPGFTGATTPQ
SPLMSPRMAHTQSPMMQQSQANPAYQPTSDMNGWAQGSMGGNSMFSQQSPPHFGQQANTS
MYSNNMNISVSMATNTGGLSSMNQMTGQMSMTSVTSVPTSGLPSMGPEQVNDPALRGGNL
FPNQLPGMDMIKQEGDASRKYC

>Rattus norvegicus_NCOA2(ENSRNOP_11026)
MSGMGENTSDPSRAETRKRKECPDQLGPSPKRSTEKRNREQENKYIEELAELIFANFNDI
DNFNFKPDKCAILKETVKQIRQIKEQEKAAAANIDEVQKSDVSSTGQGVIDKDALGPMML
EALDGFFFVVNLEGNVVFVSENVTQYLRYNQEELMNKSVYSILHVGDHTEFVKNLLPKSM
VNGGSWTGEPPRRNSHTFNCRMLVKPLPDSEEEGHDNQEAHQKYETMQCFAVSQPKSIKE
EGEDLQSCLICVARRVPMKERPALPSSESFTTRQDLQGKITSLDTSTMRAAMKPGWEDLV
RRCIQKFHTQHEGESLSYAKRHHHEVLRQGLAFSQIYRFSLSDGTLVAAQTKSKLIRSQT
TNEPQLVISLHMLHREQNVCVMNPDLTGQAMGKPLSPMSSSSPARQAMCSGNPGQDVALG
SNMNFPMNGPREQMSMPMGRFGGSGGMNHVSGMQATTPQGSNYALKMNSPSQSSPGLNPG
QPSSVLSPRHRMSPGVAGSPRVPPSQFSPAGSLHSPAGVCSSTGNSHSYTNSSLNALQAL
SEGHGVSLGPSLASPDLKMGNSQNSPVNMNPPPLSKMGSLDSKDCFGLYGEPSEGTTGQA
EASCHPEEQKRPNDSSMPQAASGDRAEGHSRLHESKGQTKLLQLLTTKSDQMEPSPLPSS
LSDTNKDSTGSLPGPGSTHGTSLKEKHKILHRLLQDSSSPVDLAKLTAEATGKELNQESS
GTAPGSEVTVKQEPASPKKKENALLRYLLDKDDTKDIGLPEITPKLERLDSKTDPASNTK
LIAMKTVKEEVSFEPSDQPGSELDNLEEILDDLQNSQLPQLFPDTRPGAPTGSVDKQAII
NDLMQLTADSSPVTPVGAQKAALRMSQSTFNNPRPGQLGRLLPNQNLPLDITLQSPTGAG
PFPPIRNSSPYSVIPQPGMMGNQGMLGSQGNLGNNSTGMIGSSTSRSSMPSGEWAPQSPA
VRVTCAATTGAMNRPIQGGMIRNPTASIPMRANSQPGQRQMLQPQVMNIGPSELEMNMGG
PQYNQQQAPPNQTAPWPESILPIDQASFGSQNRHPFGSSPDDLLCPHPAAESPSDEGALL
DQLYLALRNFDGLEEIDRALGIPELVSQSQAVDPEQFSSQESSMMLEQKPPVFPQQYASQ
TQMAQGSYNPMQDPNFHTMGQRPNYTTLRMQPRPGLRPTGIVQNQPNQLRLQLQHRLQAQ
QNRQPLMNQISGVSNVNLTLRPGVPTQAPINAQMLAQRQREILNQHLRQRQMHQQQQVQQ
RTLMMRGQGLNMTPSMVAPTGLPAAMSNPRIPQANAQQFPFPPNYGISQQPDPGFTGATT
PQSPLMSPRMAHTQSPMMQQSQANPAYQPASDMNGWAQGSMGGNSMFSQQSPPHFGQQAN
TSMYNNNMNINVSMATNTAGLSNMNQMTGQMSMTSVTSVPTSGLSSMGPEQVNDPALRGS
SLFTTNQLPGMDMIKQEGDGSRKYC

>Canis familiaris_NCOA2(ENSCAFP_11619)
MSGMGENTSDPSRAETRKRKECPDQLGPSPKRSTEKRNREQENKYIEELAELIFANFNDI
DNFNFKPDKCAILKETVKQIRQIKEQEKAAAANIDEVQKSDVSSTGQGVIDKDALGPMML
EALDGFFFVVNLEGNVVFVSENVTQYLRYNQEELMNKSVYGILHVGDHTEFVKNLLPKSI
VNGGSWSGEPPRRNSHTFNCRMLVKPLPDSEEEGHDNQEAHQKYETMQCFAVSQPKSIKE
EGEDLQSCLICVARRVPMKERPVLPSSESFTTRQDLQGKITSLDTSTMRAAMKPGWEDLV
RRCIQKFHAQHEGESVSYAKRHHHEVLRQGLAFSQIYRFSLSDGTLVAAQTKSKLIRSQT
TNEPQLVISLHMLHREQNVCVMNPDLTGQAMGKPLNPISSSSPAHQAMCSGNPGQDMTLS
SNINFPINGPKEQMGMPMGRFGGSGGMNHVSSMQATTPQGSNYALKMNSPSQSSPGMNPG
QPNSMLSPRHRMSPGVAGSPRIPPSQFSPAGSLHSPVGVCSSTGNSHSYTNSSLNALQAL
SEGHGVSLGSSLASPDLKMGNLQNSPVNMNPPPLSKMGSLDSKDCFGLYGEPSEGTTGPA
ESSCHPGEQKETNDSNMPPAVSSERADGQNRLHDSKGQTKLLQLLTTKSDQMEPSPLSSS
LSDANKDSTAGLPGSGSTHGTSLKEKHKILHRLLQDSSSPVDLAKLTAEATGKELSQEAS
STAPGSEVTIKQEPVSPKKKENALLRYLLDKDDTKDIGLPEITPKLERLDSKTDPASNTK
LIAMKTEKEEMSFEPSDQPGSELDNLEEILDDLQNSQLPQLFPDTRPGAPAGSVDKQAII

NDLMQLTAENSPVTPVGAQKTALRISQSTFNNPRPGQLGRLLPNQNLPLDITLQSPTGAG
PFPPIRNNSPYSVIPQPGMMGNQGMIGNQGNLGNSSTGMIGSNASRPTMPSGEWAPQSSA
VRVTCAAATTSAMNRPIQGGMIRNPTASIPMRPNSQPGQRQMLQSQVMNIGPSELEMNMG
GPQYSQQQAPPNQTAPWPESILPIDQASFANQNRQPFGSSPDDLLCPHPAAESPSDEGAL
LDQLYLALRNFDGLEEIDRALGIPELVSQSQAVDPEQFSSQDSNIMLEQKAAVFPQQYAS
QAQMAQGSYPPMQDPNFHTMGQRPSYATLRMQPRPGLRPTGLVQNQPNQLRLQLQHRLQA
QQNRQPLMNQISNVSNVNLTLRPGVPTQAPINAQMLAQRQREILNQHLRQRQMHQQQQVQ
QRTLMMRGQGLNMTSSMVAPGGLPATLSNPRMPQANAQQFPFPPNYGISQQPDPGFTGAT
TPQSPLMSPRMAHTQSPMMQQSQANPAYQASSDMNGWAQGNMGGNSMFSQQSPPHFGQQA
NTSMYNNNMNINVSMATNTGGMNNMNQMTGQISMTSVTSVPTSGLSSMGPEQVNDPALRG
GNLFPNQLPGMDMIKQEGDASRKYC

>Bos taurus_NCOA2(ENSBTAP_27071)
MSGMGENTPDPSRAETRKRKECPDQLGPSPKRSTEKRNREQENKYIEELAELIFANFNDI
DNFNFKPDKCAILKETVKQIRQIKEQEKAAAANIDEVQKSDVSSTGQGVIDKDALGPMML
EALDGFFFVVNLEGNVVFVSENVTQYLRYNQEELMNKSVYSILHVGDHTEFVKNLLPKSI
VNGGAWSGEPPRRNSHTFNCRMLVKPLPESEEEGHDNQEAHQKYETMQCFAVSQPKSIKE
EGEDLQSCLICVARRVPMKERPALPSSESFTTRQDLQGKITSLDTSTMRAAMKPGWEDLV
RRCIQKFHAQHEGESVSYAKRHHHEVLRQGLAFSQIYRFSLSDGTLVAAQTKSKLIRSQT
TNEPQLVISLHVLHREQNVCVMNPDLTGQAMGKPLNPISSSSPAHPAMCSGNPGQDMTLS
SNINFPMNGPKEQMGMPMGRFGGSGGMNHVSSMQATTPQGSNYALKMNSPSQSSPGLNPG
QPSSMLSPRHRMSPGVAGSPRIPPSQFSPAGSLHSPVGVCSSTGNSHSYTNSSLNALQAL
SEGHGVSLGSSLASPDLKMGNLQNSPVNMNPPQLSKMGSLDSKDCFGLYGEPPEGPAGPA
ESSCHPGEPKDPSEAGLPPAGSNERPDGQSRLLDNKGQTKLLQLLTTKSDQIEPSPLPSS
LSDTNKDSTGSLPGPGSTHGTSLKEKHKILHRLLQDSSSPVDLAKLTAEATGKELSQEAS
STAPGSEATIKQEPVSPKKKENALLRYLLDKDDTKDIGLPEITPKLERLDSKTDPASNTK
LIALKTEKEEMSFEPSDQPGSELDNLEEILDDLQNSQLPQLFPDTRPGAPAGSVDKQAII
NDLMQLTAESSPVTPVGAQKTAMRIPQSTFNNPRPGQLGRLLPNQNFPLDITLQSPTGAG
PFPPIRNSSPYPVIPQPGMMGNQGMIGNQGNLGNSSTGMIGSNAARPTMPSGEWAPQSPA
VRVTCAATSNAMNRPIQGGMIRNPTASIPMRPNSQPGQRQMLQPQVMNMGPSELEMNMGG
PQYSQQQAPPNQTAPWPESILPIDQAPFASQNRQPFGSSPDDLLCPHPAAESPSDEGALL
DQLYMALRNFDGLEEIDRALGIPELVSQSQGVDPEQFSSQDSSLMLEQKTPVFPQQYASQ
AQMAQGSYTPMQDPNFHTMGQRPSYATLRMQPRPGLRPTGLVQSQPNQLRLQLQHRLQAQ
QNRQPLMNQISNVSNVNLTLRPGVPTQAPINAQMLAQRQREILNQHLRQRQMHHQQQQVQ
QRTLMMRGQGLNMTPSVVAPSGVPAAMSNPRIPQANAQQFPFPPNYGISQQPDPGFTGAA
APQSPLISPRLAHTQSPMLQQSQANPAYQASSDMNGWAQGSMGANSMFSQQSPPHFAQQA
NTSMYNNNMNINVSMATNTGGMNNMNQMTGQISMTSVTSVPTSGLSSMGPEQVNDPALRG
GGLFPSQLPGMDMIKQEGDASRKYC

>Monodelphis domestica_NCOA2(ENSMODP_9152)
MSGMGENTSDPSRAETRKRKECPDQLGPSPKRSTEKRNREQENKYIEELAELIFANFNDI
DNFNFKPDKCAILKETVKQIRQIKEQEKAAAANVDEVQKSDVSSTGQSVIDKDALGPMML
EALDGFFFVVNLEGNVVFVSENVTQYLRYNQEELMNTSVYSILHVGDHAEFVKNLLPKSL
VNGGPWSNEAPRRNSHTFNCRMLVKPLTDSEEESHDSQEAHQKYETMQCFAVSQPKSIKE
EGEDLHSCLICVARRVPMKERPVLPSSESFTTRQDLQGKITSLDTSTMRAAMKPGWEDLV
RRCIQKFHAQHEGESVSYAKRHHHEVLRQGLAFSQIYRFSLSDGTLVAAQTKSKLIRSQT
TNEPQLVISLHMLHREQNVCVMNQDLTGQAMGKPLNPISSSSPAHQAMCSGNPGQDMTIS
SNINFAINGPKEQMGMPVGRFGGSGGMNHVSSMQASTPQGSNYALKMNSPSQSSPGMNPG
QPNSMLSPRHRMSPGVAGSPRIPPSQFSPAGSLHSPVGVCSSTGNSHSYTNSSLNALQAL
SEGHGVSLGSSLASPDLKMGNLQNSPVNMNPPPLSKMGSLDSKDCFGLYGEPSEGTTGQA
ESSCHSGEQKENSDTNIPQVVSGERSDGQNRLHDSKGQTKLLQLLTTKSDQMEPSPLSGS
LGETNKDSTGGLAGSVSTHGTSLKEKHKILHRLLQDSSSPVDLAKLTAEATGKELNQDSN
STGPGSEVTIKQEPVSPKKKENALLRYLLDKDDTKDIGLPEITPKLERLDSKTDPASSTK
LIAMKSEKEEMTFEPNEQPGSELDNLEEILDDLQNSQLPQLFPDTRPGAPTGSVDKQAII
NDLMQLTAENSPVAPAQKPALRISQSTFNNPRPGQLGRLLPNQNLPLDITLQSPTGAGPF
PPIRNNSPYSVIPQPGMMGNQGMLGNQGNLGNNSTGMIGGNTSRPAIPSGEWGSPGSAVR
VTCPATTSAMNRPIQGGMIRNPTASIPMRPSSQPGQRQMLQSQVMNMGPAELEMNMGGPQ
YSQQQAPPNQTAPWPESILPIEQASFAGQNRQPFGTSPDDLLCPHPASESPSDEGTLLDQ
LYLALRNFDGLEEIDRALGIPELVSQSQAVDPEPFSSQESSLIIEQKPPVFPQQYASQAQ
MAPGSYTPMQDPNFHTLGQRPSYATLRMQPRPGLRPTGIVQNQPNQLRLQLQHRLQAQQN
RQPLMNQISNVSNMNLTLRPGVPTQGPINAQMLAQRQREILNQHLRQRQMHQQQQVQQRT

LMMRGQGLNMAPSMVASGGIPATMSNPRIPQANAQQFPFPPNYGISQQPDPGFTGATTPQ
SPLMSPRMAHTQSPMIQQSQANPAYQSSSEMNGWAQGNMGGNSMFSQQSPPHFGQQANTS
MYNNNMNISVSMATNTNGMNNMNQMTGQISMTSVTSVPTSGLSSMGPEQVNDPAMRGGNL
FPNQLPGMDMIKQEGDASRKYC

>Taeniopygia guttata_NCOA2(ENSTGUP_11821)
MSGMGENTSDPSRAESRKRKDPDQVGPSPKRSTEKRNREQENKYIEELAELIFANFNDID
NFNFKPDKCAILKETVKQIRQIKEQEKAAAANEDEVQKADVSSTGQSVIDKDALGPMMLE
ALDGFFFVVNPEGNVVFVSENVTQYLRYNQEELMNTSIYSILHVGDHNEFVKILLPKSLV
NGGSWSGDTPRRNSHTFNCRMLVKPLADSEEGHDNQEAHQKYETMQCFAVSQPNSFKEDG
EDLQSCLICVARRAPMKERPLLPSSESFTTRQDLQGKITSLDTSTMRAAMKPGWEDLVRR
CIQRFHSQHEGEISFAKRHHQEVLRQGLAFSPVYRFSLSDGTIVSAQTKSKLIRSQTTSE
PQLVISLHMLHREQNVCGMNQDLTGQGMGKILNPISSSSPAHQAMCSGNPGQDMTISSNI
NFAINGPKEQVGMPAGRFGGSGGLNHVSSIQASTPQGSNYALKMNSPSQSSPGMNPGQPN
SMLSPRHRVSPGVAGSPRIPPSQFSPAGSLHSPVGVCSSTGNSHNYTNSSLNALQALSEG
HGVSLGSSLASPDLKMGNLQNSPVNMNPPQLSKMGSLDSKDCFGIYGEQSEGTTGQAESS
CHSGEQKDNSDSNMPPVVSGERPDGQNKLHDGKSQTKLLQLLTTKSDQMEPSPLSSTMGD
ISKDSTGGLPGSGSAHGTSLKEKHKILHRLLQDSSSPVDLAKLTAEATGKELNQESSSTA
PGSEVTVKQEPASPKKNNNALLRYLLDKDDTKDIGLPDIPPKLERLDSKTDPSGSTKLIA
MRTEKEEISFEPNEQPGSELDNLEEILDDLQNSQLPQLFPDSRPGVPAGSVDKQAIINDL
MQLTSDSSPGAAVATQKPAMRISQSTFNNPRAGQLGRLLPNQNLPLDITLQNPTGAGTFP
PIRTSSPYSVIPQPGLMGRNEGMIGSQGTLGNSSAGMIGGNAPRPAMASGDWGSQAPAVR
VTCAPTAGTMNRPVQAGMIRNPTASIPLRPSNQPGPRQMLPSQVMNMGPSELEMNIGGPQ
YSQQQAPPNQTAPWPDSILPIEQATFGNQNRQPFGSSPDDLLCNHPSSESPSDEGALLDQ
LYMALRNFDGLEEIDRALGIPELVSQSQAVDPETFPSQESSMLMEQKAPVFSQQYAAQAQ
MAQGSYAPMQDPSFHAMGQRPGYTALRMQPRPGLRPAGIVQNQPNQLRLQLQHRLQAQQN
RQPLINQISSVSNMNLSLRTGVPTQGPINAQMLAQRQREILSQHLRQRQMQQQQQQVQQR
TLMMRGQGLSMTPSMVATGGIPATMSNPRIPQANAQQFPFPPNYGISQQPDPGFTGATTP
QSPLMSPRLAHTQSPMIQQSQANPAYQSSTEMNGWAQGNMGGNSMFSQQSPPHFGQQAST
SMYNNNMNISVSMATNTSGMNNMNQMTGQISMTSATSVPTSGLSSMGPEQVNDPAMRGGN
LFANQLPGMDMIKQDGDAAR

>Anolis carolinensis_NCOA2(ENSACAP_14888)
MSGIGENSSDPARAESRKRKECPDQLGPSPKRSTEKRNREQENKYIEELAELIFANFNDI
DNFNFKPDKCAILKETVKQIRQIKEQEKAAAANEDEVQKADVSSTGQSVIDKDALGPMML
EALDGFFFVVNLEGNVVFVSENVTQYLRYNQEELMNTSVYSILHVGDHNEFVRNLLPKSL
VNGGPWSGEAPRRNSHTFNCRMLVKPLTDSEEEGHDNQEAHQKYETMQCFAVSQPKSIKE
EGEDLQSCLICVARRVPMKERPLPSSETFSTRQDLQGKITSLDTSIMRAAMKPGWEDVVR
RCIQRFYAQQEGDLSYARKHHQDVLRQGLAFSPVYRFSLSDGTMVAAQTKSKLICNQTTN
EPQLVISLHMLHREQNVCGMSQDLTGQAMGKPLNPISSSSPAHQTMCSGNPGQDMTISSN
MNFAINGPKEQMGMPASRFGGSGGMNHVSSIQASTPQGSNYALKMNSPSQNSPGMAPGQP
NSMLSPRHRVSPGVAGSPRIPPSQFSPAGSLHSPVGVCSSTGNSHSYTNSSLNALQALSE
GHGVSLGSSLASPDVKMGNLQNSPVNMNPPQLSKMGSLDSKDGFGLHGEQSEGTTGQAEN
VCHSGDQKESNDNMPQVVGGERPDGQNRLHDGKSQTKLLQLLTTKSDQMEPSPLSNAMGD
ANKDSMGGLSGSGSAHGTSLKEKHKILHRLLQDSSSPVDLAKLTAEATGKELSQETGSTA
PGSEVTVKQEPVSPKKKENALLRYLLDKDDTKDIGLPDITPKLERSDSKTDPSSSTKPVA
MKTEKEEMRFEPIEQPGSELDNLEEILDDLQNSQLSQLFPDTRPVASAGSVDKQAIINDL
MQIAGENSPGTPVGAPKPAMRMSQSTFTNARQAQLGRMLPNQSLQLDITLQNTSGAGTFT
PIRNNSPYSVIPQPGMMGSQGMMGNQANLGNNSPGMIGGNPPRSSMPSGDWGSQGAAVRV
TCAATTNTMNRPIQGGMIRNPTSSLPLRPNSQAGPRQMLQSQVMNMGASELEMNMGGPQY
TQPQAPPNQTAPWPDSILPIDQASFSNQNRQPFGSSPDDLLCQHPVTESPSDEGTLLDQL
YMALRNFDGLEEIDRALGIPELVSQSQAVDPEPFSSQESNIILEQKAPVFTQQYASQAQM
AQGNYPPIQDPNFHPMAQRPGYAALRMQPRPGLRPAGIVQSQPNQLRLQLQHRLQAQQNR
QPLINQISNVSNMNLSLRPGVPTQGPINSQMLAQRQREILSQHLRHRQMQQQVQQRTLMM
RGQTMNMTPSMVASGGIPATMTNPRIPQANTQQFPFPPNYGINQQPDPGFTGATTPQSPL
MSPRMAHSQSPMIQQSQANPAYQSSTEMNGWAQGNIGANSMFSQQSPPHFGQQANTSMYN
NNMNINVSMVTNTSGMNNMNQMTGQISMTSATSVPTSGLSSMGPEQVNDPAMRGGNLFPN
QLPGMDMIKQEGDASRKYC

>Xenopus tropicalis_NCOA2(ENSXETP_34772)
MGENPSDPSRAEPRKRKESIDQLGPSPKRSTEKRNREQENKYIEELAELIFANFNDIDNL

NFKPDKCAILKETVKQIRQIKEHEKTAAANEDEVQKADVSSTGQSVIDKDALGPMMLEAL
DGFFFVVNREGNVVFVSENVTQYLRYNQEELMNTSVYSILHVGDHSEFIKNLLPKSIVNG
GPRRNSHTFNCRMLVKPMMECEEEGHDGQETHQKYETMQCFAVSQPKSIKEEGEDFQSCL
ICVARRVPMKERPVPPPSESFTTRQDLQGKITSLDTTNMRALMRPGWEDLVRRCIQRFHS
QHDGEISYSKRHHQEVLRQGHATSPFYRFALSDGTTVLAHTKSKLMRSQTTNEPQLVLSL
HVLQREQNMCGLNQDLAGQAMGKTLNPVQSSSPAHQAMYGGNPGQDTTISSNMNYAITGP
KEQMGMAAGRFVGSGGMNHISSLQATTPQGNNYALKINSPSQGSPGMGQGQPNSMLSPRH
RVSPGVAGSPRIAPSQFSPAGSLHSPVSVCSSTGNSHSYTNSSLNALQALSEGHGVPLAP
PLSSPDLKVGNVQHSPVNMNPPQLRKMGSIDSKESFGLYGEQPESAAGQGESGCHSNEQK
DCSENLSSVGDQTEGQSRLLDSKGQQKLLKLLTTKSDQMEPSPLPSNTLGDMNKDSLSNF
ASNSMSASAHGTSLKEKHKILHRLLQDSSSPVDLAKLTAEATGKELSQESNSTGPGSEVT
IKQEPVSPKKKEHALLRYLLDKDDTKDNVADITPKLERPDVKVEPTSCPKLLSVKAEKEE
PSFGHSDQLMPPGSDLDNLDEILDDLQNSQLSQLFPDARHDGSNSADKQAIMNDLMQLAG
DNSTGLPAGAHKQRMIRIQQNNGFSSQLAAQLGRLPNQSLPLDINLQSQVSAGSFPPMRN
SAPYTTVSQSVVMNNQAMMGSQGNVSNSSPGIIGVNGPRPALKPGDWGSQASAVRPACPT
PSTAINRPIQDLTRSPTASIPMRPGGQVCPRQVLQSPVINMGSSELDMNIGGPQYTQQQA
PPNQTAPWPDSILPIDQPSFNNQNRQPFGSPADDLICQPIASQSPTDDGNLLDQLYMALR
NFDGLEEIDRALGIPEMVSQGQAVEQESFVSPESNLMMEQKPPLYNHAYANQGQMAQNSY
TPMQDPGFNPMGQRPSYGILRMQNRPGLRPTGMVQNQPNQLRLQLQHRLQAQNRQPIMNP
INNVSNMNLAMRPGVPGQLREQGPINAQMLAQRQRELLSQHLRQKQVQQHRAMMMRGQGL
AMPPNMVGSGGIPASINSPRIPQGNTQQFPFPPNYGTGIPSPPPFTSPFSPVPPSPGSQS
LSHNSLHGSQMNLANQGIMGSMGGQYGPVMNPQMQHNAFQFANSGMSQQSDPGFTGATTP
QSPIMSPRMGHIQSPMMQQSQASPAYQSELNGWAQGNPAGNSMFSQQSPPHFGQQSGTSM
YNSNNMNISVSMAANGNGMNSMNQMTGQINMTSVTSVPTSGLSSMGPEQKYC

>Danio rerio_NCOA2(ENSDARP_109306)
MSAVGENSSDPARSEAQKRKEGPSDLLGPSPKRSTEKRNREHENKYIEELAELIFANFND
IDNFNVKPDKCAILKETVKQIRQIKEQEKAAAANEDEVQKADVSSTGQSVIDKDALGPMM
LEALDGFFFVVNMEGNIVFVSENVTQYLRYNQEELMNTSVYSILHVGDHAEFIKNLLPKS
HVNGVPWSSENPRRNSHTFNCRMLVNPHSEAEETQDHEAQQKYETMQCFAVSEPKSIKEE
GEDFQSCLICVARRVPMKERPMLPTQESFTTRQDLQGKITSLDTSLLRASMKPGWEDLVR
RCIQRFHLQNDGDISFAKRHQQEVIRHGQAFSPIYRFSLSDGTIVSAHTKSKLVRSSSTN
EPQLYMSLHILQREQPVCGMASDLGNAQTMGKPMNPMSSPNTAGSSCTPQGQDATISSNT
STFPSPGAQKEPGAMHRFGCPGTMSHSATMQAATPQGSGYPLKLSSPSQGSPGMLSPRHR
ASPGVAGSPRLPPPQFSPAGSLHSPASMCTSSTGGNGGAGANHGYTSSSLNALQALSECH
GVSHGQSLGSPDRKLGSPAAHSVAVSHHLINKMSVPESFGEQNQSEQGTPGQDEGIDLPR
DEKGNLGQFGNLDGSDPQSRLRDNKSHTKLLQLLTTKTEPIESTSPPPMAGGEPGCKDGG
AGNGGMGSQGGNGSHATSLKEKHKILHRLLQNSSSPVDLAKLTAEATGKELCQDSAGGTA
GVPELAIKQEPVSPKKKDNALLRYLLDKDDNVLKGKGIKMEPGEIKEEGGKMTGIKTEKT
DGGYDRVEPSSELDDILDDLQNSQPGLFTDSRPVSLPSAVDKQSIINDILQMTGESGANM
SPQQQRALQTAVSQQNFPAPRPGQPGRAPPVRSVSLDMNMPPKGAQGQYPMMRNNNPYSI
MQQQGMMGNHAVMPNQPNMVNAGVMGNNGPRVGMQQDGWGAQGTVGSAASPATAAPAGQH
SLPQGALHSRMVPNPATGMPMRPNSQPGPRPMLQPQMMANAQSNMDMGMAGHQFPQQQAP
PNQTAPWPDSVMPIEPVPFGNQSSSMYGGSQEDVLCPPASEGPADEGALLSQLYSALKDF
DGLEEIDRALGIPALVGQAQPVEQDQFPGQDPSMMMDQKPPMYGQQQYASSPANMQQAGY
TPMQDATFHGLQGQMGQRPGYPMLRMQARPGLRPGGVVPNQPNTLRLQLQHRLQAQQNRQ
PMMNQMGGVSNMNLPLRPNVPNQGTINAQMLAQRQRELLSNHLRQRQLDQQRQQQQQQQQ
QRSLAMRAQGLTMPPNMAAAAAGMPGAMSNPRIPQANPQQFPYPPNYGMSQQSDGGFGGA
ATPQSPLMSPRMGHAQSPMMQQAQGNSSFQSSPDMNGWPQGNINTNSMFTQQSPPQFSQQ
ANNNMYNGNGMNLNVSMAANSSNMGQMGNQMSMSSMNSGPGSGMANMGPEQQKYC

>Takifugu rubripes_NCOA2(ENSTRUP_29760)
MSGVGETPSDPARAEPRKRKDCSSELLGPSPKRSNEKRNREQENKYIEELAELIFANFND
IDNFNVKPDKCAILKETVKQIRQIKEQAEKAAAANEEEVQKADVSSTGQSVIDKDALGPM
MLEALDGFFFVVNMEGNIVFVSENVTQYLHYNQEELMNTSVYSVLHVGDHAEFIKNLLPK
SLVNGVPWSSEGPRRNSHTFNCRMLVNPHSENPEESHEHEPQQQKYETMQCFAVSEPKSI
KEEGDDLQSCLICVARRVPMKERPILPTHESFTTRQDLQGKITSLDTSLLRASMKPGWED
LVRRCIQRFHLQNDGEMSFARRHHQEVMRHGQAFSPIYRFSLSDGTIVSAHTKSKLVRSP
ATNEPQLYMSLHILQREQTVCRMGQDMGPGSQATGMAPKPINSSTPSMTPPTPGSLPGSG
PQGQDTSFNSNSTPFSPPGPTGPREPAAMGGHMQGYRFGCPPPHNHATGMQPPQQGPNWR
MNSPSRASPSPAGGPHPPQQNSMLSPRHRGSPGGAGSPRVTGLQSHSPVGMCGGPGSAGS

GGTTGTHANSYTSSSLSALQALSECHGVGHGGPHAHTLGSPDRKIGSPAGVTQGSGVNAQ
LMSKHVGGVVAAPNSGDSFGPHSEVGQGQQQGQTEGSLTEPKEEREGPSEGPDAHSRLHD
KSHTKLLQLLTRKPEPLETPLSPGSRGEGKDQPGTGARAGHTGGNPHASSLKEKHKILHQ
LLQNNTSPGDLAKLTAEATGKELNQEQGAGGTAPGAEITPKQEPLSPKKKDNALLRYLLD
KDDTVMKDKVPKLEPGEVKVEGGKHALVKTEKQDAGYDQAEQESPASESSELDDILNDLQ
NSQSQLFCELRPVTLPTPMDKQNIINDILQMSESNPALAAQQQHRGIAAGMGPSRMMAAG
GQRPGMQQQLQEGWGASASAPPLGQSGPTQQGVMPGRIGANTAPMRATSQPRPMLQSPLM
ANAQPDMDIGMAGHNFTQQQAPPNQTAPWPESMMPIDQTFANQNRWPVHAAPQDDLLCNT
ELNSGDGTGVDEGALMSQLYTALKDFDGLEELDRALGIPALVEQNQSLELDHFSQDPSMM
IDQKPPMYTQQFGPPASHMAHRGYPGSAMQEQCFHPMSGQMGPRPGYPMMRMQARPGLRP
SGVVPNQPNTLRMQLQHRLQSQQNRQPMITQMSSVSNVNLPLRASAPNQGTINAQMLAQR
QREMLSNHLRQRQQQQVQQQRIAMRAPGLNLPPNMATGGISNPRIPQANPQQFPYPPSYG
TSMSQQADGGFGGTGTPQSPLLSPRMAHAQSPMMQQGQGNASFQGSPDMNGWPQGNMAGN
SMFSQQQTSPQFTQQNNNMYNSNGMNLNSVSMATNMATSSMGQMAGQMNVTSMASGPSPG
LPSMGQEQVNTRAIR

>Tetraodon nigroviridis_NCOA2(ENSTNIP_11019)
MSGVGETPSDPARAESRKRKDCSSELLGPSPKRSNEKRNREQENKYIEELAELIFANFND
IDNFNVKPDKCAILKETVKQIRQIKEQEKAAAANEEEVQKADVSSTGQSVIDKDALGPMM
LEALDGFFFVVNMEGNIVFVSENVTQYLHYNQEELMNTSVYSVLHVGDHAEFIKNLLPKS
LVNGVPWSSEGPRRNSHTFNCRMLVNPHSENQEEPHEHEPQQQKYETMQCFAVSEPKSIK
EEGDDLQSCLICVARRVPMKERPMLPTHESFTTRQDLQGKITSLDTSLLRASMKPGWEDL
VRRCIQRFHLQNDGEMSFAKRHQQEVLRHGQAFSPIYRFSLSDGTVVSAHTKSKLVRSPA
TNEPQLYMSLHILQREQTVCGMGQDMGPGSQATGMAPKPINSSTPSMTPPTPGSLPGSGP
QGQDAAFSSNSTPFSPPGPTGPREPAAIGGHMQGYRFGCPPPHSHATGMQPPQQGPNWRM
NSPSRASPSPAGGPHPPQQNSMLSPRHRGSPGAGSPRVTGGLQSPSPVGMCGGGPGSAGS
SSTTNSHANSYTSSSLSALQALSECHGVGHGGPHAHTLGSPDRKIGSPAGVTQVSGVNAQ
LMSKHAGGSGGAGNSGDSFGPHSDVGQGQPQGPTEGSLSEPKEEREGPSEGHDAHSRLHD
KSHTKLLQLLTRKPEPLEMPLSPGSRGEGKDQPGSGVRAGHNGGNAHATSLKEKHKILHQ
LLQNNTSPVDLAKLTAEATGKELNQEQGAGGTAAGEVTPKQEPLSPKKKENALLRYLLDK
DDTVMKDKVPKLEPGEVKVEGVKHTLVKTEKQDAGYDQAEQENPASEGRELDDILNDLQN
AQPQLFCELRPVSLPSPMDKQNIINDILQMSESNPALAAQQQHRGIAAGMGPSNFGNIRP
SQPGRGLPVRSGSLDMNMSPQQPSLQYPIRATSPYTVKGMVGNHTMMANQTGMVNAVVAT
LGMMAAGGPRPGVQQQLQEGWVASASAPTMGQSGPAQQGVMQGRIGANTAPMRATSQPRP
MLHSPMMATAQPDVDIGMTGHNFSQQQAPPNQTAPWPESMMPIDQTFANQNRYSSNLMQQ
SMIHTNSPSDEGALMSQLYTALKDFNGMEELDRALGIPALVEQSQSLELDHLSQDPSMMI
DQKPPMYAQQFGPPAPHMAHRGYPASAMQEQCFHPMSGQMAPRPGYPMMRMQARPGLRPS
GVVPNQPNTLRMQLQHRLQSQQNRQPMITQMSSVSNVNLPLRASAPNQGTINAQMVAQRQ
RELLSNHLRQRQQLQVQQQQRMAMRGPGLNLPPNMASGGMSNPRIPQANPQQFPYPPSYG
MSQQADGGFGGTGTPQSPLLSPRMAHTQSPMMQQGQGNASFQGSPDVNGWPQGNMATGNG
MFPQQNNNNMYNSNGMNLNNVSMATNMATSSMSSMGQMAAQMSVTSMASGPSPGLPSMGQ
EQVDPGAKNRYLSDLLKPKQPLMSQKDGGRGSNRFC

>Gasterosteus aculeatus_NCOA2(ENSGACP_3702)
MSGVGENPSDPARAESRKRKECSAELLGPSPKRSNEKRNREHENKYIEELAELIFANFND
IDNFNVKPDKCAILKETVKQIRQIKEQAEKAAAANEEEVQKADVSSTGQSVIDKDALGPM
MLEALDGFFFVVNMEGNIVFVSENVTQYLRYNQEELMNTSVYSVLHVGDHAEFIKNLLPK
SLVNGVPWSSEGPRRNSHTFNCRMLVNPHGDNQDELHEPQQQKYETMQCFAVSEPKSIKE
EGDADFQSCLICVARRVPMKERPMHPTHESFTTRQDLQGKITSLDTSLLRASMKPGWEDL
VRRCIQRFHLQNDGEMSFAKRHQQEVLRHGQAFSPIYRFSLSDGTIVSAHTKSKLVRSPA
TNEPQLYMSLHILQREQMVCGMGQDMGPGSQATGMAPKPMNSSTPSMTPPTPGSLPGSGP
QGQDTSISGNSTPFSPQGPAGPREPAAMGGHMQGYRFGCPPPHIHTGGMQPPQQGPNWRM
NSPSRASPSPAGGPHPPQQNSMLSPRHRGSPGGAGSPRVAGGLHSPSPVGMCGGGGGGAC
GSGAASTHANSYTSSSLSALQALSECHGVGHGHGAPHAHALGSPDRKIGSPVGVTQGSGV
NPHLMSKLGGGGGTGGVTGDSFRPHAEVGPGQTESNLAEPKEEREGPPEGHDSHSRLHDN
KGHTKLLQLLTTKPEPLEMPLSPGAGGEGKDQAGAGVRCGLAGVNTHATSLKDKHKILHQ
LLQNSTSPVDLAKLTAEATGKELGQDPTQGAGGAASGAELSLKQEPLSPKKKDNALLRYL
LDKDDTGVKDKVPKLEPGEVKVEGGKHPLVKTEKQDTGYDRAEQVRGGQSSELDDILNDL
QNAQPQLFCEPRPVSLPSPMDKQNIINDILQMSETNPAMVAQQQHRGIGTGMGPTNFAGV
RPGQPGRGLPVRSVSLDMNVSPQPPSLQYPIRATSPYTLMQQQQQHGMVGGHGMMAHQAG
MVGAESPFLTLCVALCSSPVTSWHSDSLPVTAVCSSRNDCSGRSTAGGWVGSASAPPLAP

SGPGQQGPMQGRIGPNGAPMRPNGQLGPRPMLQSSMMAGGESPVCPENVAQVFKSGNEKY
ESVDIKAPFLYPATHFIALLLIVKGCPPVADQSAALNKDMFSRDNQSCILLPTIRLHLVP
QMRRRPFRFFPTEQPSIHTSPYNLCLSLCQRVDASNCSRLGLAIDVCLCVLKVLCPVQNG
VLAVHSFSTRGRQERNNQSKLSRGHPEKPDSFRVSWQRHDQGCTLHRSTIYANYSMIILI
CMMTYSMAPQSDSGTSVCAGALMSTYLDVSPTGMSQQADGGFGGPGTPQSPLLSPRMAHA
QSPMMQQSQGNAAFQGSSDMNGWPQGNIGGNSMFPQQQASQQFPQQSNSNMYNGNAMNLN
NVNMAASSMGQMAGQMSVTSMASGPSPSLPSMGPEQVN

>Oryzias latipes_NCOA2(ENSORLP_10164)
MSGVGENSSDPARAESRKRKECSVDLIGPSPKRSNEKRNREHENKYIEELAELIFANFND
IDNFNVKPDKCAILKETVKQIRQIKEQEKAAAANEEEVQKADVSSTGQSVIDKDALGPMM
LEALDGFFFVVNMEGNIVFVSENVTQYLRYNQEELMNTSVYSVLHVGDHAEFIKNLLPKS
LVNGVPWSSEGPRRNSHTFNCRMLVNPRSESQDETHEHEPQQQKYETMQCFAVSEPKSIK
EEGDDFQSCLICVARRVPTKERPLPPTHESFTTRQDLQGKITSLDTSLLRASMKPGWEDL
VRRCIQKFHLQSDGEMSFAKRHQQEVLRHGQAFSPIYRFSLSDGTMVSAHTKSKLMRSPG
TNEPQLYMSLHILQREPAVCGMGQDMGPGMQATGMAPKPMNSSTPSMTPPTPGSLPGLGP
QGQDTTISSNSTPFSPPGPAGPREPAAMGGHMQSYRFGCPLPHNHTGGMQPPQQGPNWRM
NSPSRASPSPAGGPHPPQQNSMLSPRHRGSPGGASSPRVAGGLHSPSPVGMCGGAGNGTN
SAHPNSYTSSSLSALQALSECHGVGHGHVPPHAHTLGSPDRKIGSPSGVTPGSGVNTHMM
SKLGGSTSSSGEFGPFPDGSQGPQAQTDGSQTDPKDESHIQENKCHTKLLQLLTTKPEPL
ETPLSPGADGECKDQAGSGVRAGHTGGSTHATSLKEKHKILHQLLQNSTSPVDLAKLTAK
ATGKELGQDTTQSAGSTASGAEITPKQEPLSPKKKDNALLRYLLDKDDTVMKDKVPKLEP
GEVKIEDCKQPQVKIEKQDAGYDRAEQPSELDDILNALQNTQPQLFVESRPVSLPSPMDK
QNIINDILQMSDNNPSMAPQQQHRCLGPGMGPSRMMGVSGPQPGMQQQESWGGSASAPPL
GAPGQQSAMQGRMGPGSAPMRPSSQPGPRPIMQSAMMAGAQPDLDIGMVGPHFSQQQAPP
NQTAPWPESMIPIDQTSFINQNRPAPQEDLLPTAGDGSTVDEGALMSQLYTALKDFDGLE
EIDRALGIPALVEQSQSLEPDHFQQDPSLMMDQKPPMYAHIGPPSAHMAQRGYAGTPMQE
PGFHPMPGQMGPRPGYPIMRMQPRPGIRAPGLVPNQPNTLRLQLQHRLQSQQNRQPMMTQ
MSSVSNVNLPPRANAPNQGTINAQMVAQRQREMLSNHLRQKQQQQVQQAQQQRSMALRAQ
GLSMAPNMTARNPQAGPQQFPYPPNYGMSQQADGSFGGPGTPQSPLISPRMAHTQSPMMQ
QTQQNTAFQGSPDMNGWPQGNMGANSMFSQQQASPQFSQQNNIYNGNGMSLNSVPMTTNM
ATSSMSSMGQIAGQMSSSSMRSGPSPGLPPIGQEQV

>Homo sapiens_NCOA3(ENSP_361066)
MSGLGENLDPLASDSRKRKLPCDTPGQGLTCSGEKRRREQESKYIEELAELISANLSDID
NFNVKPDKCAILKETVRQIRQIKEQGKTISNDDDVQKADVSSTGQGVIDKDSLGPLLLQA
LDGFLFVVNRDGNIVFVSENVTQYLQYKQEDLVNTSVYNILHEEDRKDFLKNLPKSTVNG
VSWTNETQRQKSHTFNCRMLMKTPHDILEDINASPEMRQRYETMQCFALSQPRAMMEEGE
DLQSCMICVARRITTGERTFPSNPESFITRHDLSGKVVNIDTNSLRSSMRPGFEDIIRRC
IQRFFSLNDGQSWSQKRHYQEAYLNGHAETPVYRFSLADGTIVTAQTKSKLFRNPVTNDR
HGFVSTHFLQREQNGYRPNPNPVGQGIRPPMAGCNSSVGGMSMSPNQGLQMPSSRAYGLA
DPSTTGQMSGARYGGSSNIASLTPGPGMQSPSSYQNNNYGLNMSSPPHGSPGLAPNQQNI
MISPRNRGSPKIASHQFSPVAGVHSPMASSGNTGNHSFSSSSLSALQAISEGVGTSLLST
LSSPGPKLDNSPNMNITQPSKVSNQDSKSPLGFYCDQNPVESSMCQSNSRDHLSDKESKE
SSVEGAENQRGPLESKGHKKLLQLLTCSSDDRGHSSLTNSPLDSSCKESSVSVTSPSGVS
SSTSGGVSSTSNMHGSLLQEKHRILHKLLQNGNSPAEVAKITAEATGKDTSSITSCGDGN
VVKQEQLSPKKKENNALLRYLLDRDDPSDALSKELQPQVEGVDNKMSQCTSSTIPSSSQE
KDPKIKTETSEEGSGDLDNLDAILGDLTSSDFYNNSISSNGSHLGTKQQVFQGTNSLGLK
SSQSVQSIRPPYNRAVSLDSPVSVGSSPPVKNISAFPMLPKQPMLGGNPRMMDSQENYGS
SMGGPNRNVTVTQTPSSGDWGLPNSKAGRMEPMNSNSMGRPGGDYNTSLPRPALGGSIPT
LPLRSNSIPGARPVLQQQQQMLQMRPGEIPMGMGANPYGQAAASNQLGSWPDGMLSMEQV
SHGTQNRPLLRNSLDDLVGPPSNLEGQSDERALLDQLHTLLSNTDATGLEEIDRALGIPE
LVNQGQALEPKQDAFQGQEAAVMMDQKAGLYGQTYPAQGPPMQGGFHLQGQSPSFNSMMN
QMNQQGNFPLQGMHPRANIMRPRTNTPKQLRMQLQQRLQGQQFLNQSRQALELKMENPTA
GGAAVMRPMMQPQVSSQQGFLNAQMVAQRSRELLSHHFRQQRVAMMMQQQQQQQQQQQQQ
QQQQQQQQQQQQQQQQTQAFSPPPNVTASPSMDGLLAGPTMPQAPPQQFPYQPNYGMGQQ
PDPAFGRVSSPPNAMMSSRMGPSQNPMMQHPQAASIYQSSEMKGWPSGNLARNSSFSQQQ
FAHQGNPAVYSMVHMNGSSGHMGQMNMNPMPMSGMPMGPDQKYC

>Gorilla gorilla_NCOA3(ENSGGOP_19561)
MSGLGENLDPLATDSRKRKLPCDTPGQGLTCSGEKRRREQESKYIEELAELISANLSDID

NFNVKPDKCAILKETVRQIRQIKEQGKTISNDDDVQKADVSSTGQGVIDKDSLGPLLLQA
LDGFLFVVNRDGNIVFVSENVTQYLQYKQEDLVNTSVYNILHEEDRKDFLKNLPKSTVNG
VSWTNETQRQKSHTFNCRMLMKTPHDILEDINASPEMRQRYETMQCFALSQPRAMMEEGE
DLQSCMICVARRITTGERAFPSNPESFITRHDLSGKVVNIDTNSLRSSMRPGFEDIIRRC
IQRFFSLNDGQSWSQKRHYQEAYLNGHAETPVYRFSLADGTIVTAQTKSKLFRNPVTNDR
HGFVSTHFLQREQNGYRPNPNPVGQGIRPPMAGCNSSVGGMSMSPNQGLQMPSSRAYGLA
DPSTTGQMSGARYGGSSNIASLTPGPGMQSPSSYQNNNYGLNMSSPPHGSPGLAPNQQNI
MISPRNRGSPKIASHQFSPVAGVHSPMASSGNTGNHSFSSSSLSALQAISEGVGTSLLST
LSSPGPKLDNSPNMNITQPSKVSNQDSKSPLGFYCDQNPVESSMCQSNSRDHLSDKESKE
SSVEGAENQRGPLESKGHKKLLQLLTCSSDDRGHSSLTNSPLDSSCKESSVSVTSPSGVS
SSTSGGVSSTSNMHGSLLQEKHRILHKLLQNGNSPAEVAKITAEATGKDTSSITSCGDGN
VVKQEQLSPKKKENNALLRYLLDRDDPSDALSKELQPQVEGVDNKMSQCTSSTIPSSSQE
KDPKIKTETSEEGSGDLDNLDAILGDLTSSDFYNNSISSNGSHLGTKQQVFQGTNSLGLK
SSQSVQSIRPPYNRAVSLDSPVSVGSSPPVKNISAFPMLPKQPMLGGNPRMMDSQENYGS
SMGGPNRNVTVTQTPSSGDWGLPNSKASRMEPMNSNSMGRPGGDYNTSLPRPALGGSIPT
LPLRSNSIPGARPVLQPQQQQQQMLQMRPGEIPMGMGANPYGQAAASNQLGSWPDGMLSV
EQVPHGTQNRPLLRNSLDDLVGPPSNLEGQSDERALLDQLHTLLSNTDATGLEEIDRALG
IPELVNQGQALEPKQDAFQGQEAAVMMDQKAGLYGQTYPAQGPPMQGGFHLQGQSPSFNS
MMNQMNQQGNFPLQGMHPRANIMRPRTNTPKQLRMQLQQRLQGQQFLNQSRQALELKMEN
PTAGGAAVMRPMMQPQVSSQQGFLNAQMVAQRSRELLSHHFRQQRVAMMMQQQQQQQQQQ
QQQQQQQQQQQQQTQAFSPPPNVTASPSMDALLAGPTMPQAPPQQFPYQPNYGMGQQPDP
AFGRVSSPPNAIMSSRMGPSQNPMMQHPQAASIYQSSEMKGWPSGNLARNSSFSQQQFAH
QGNPAVYSMVHMNGSSGHMGQMNMNPMPMSGMPMGPDQKYC

>Macaca mulatta_NCOA3(ENSMMUP_15773)
MSGLGENLDPLATDSRKHKLPCDAPGQGLTCSGEKRRREQESKYIEELAELISANLSDID
NFNVKPDKCAILKETVRQIRQIKEQGKTISNDDDVQKADVSSTGQGVIDKDSLGPLLLQA
RALDGFLFVVNRDGNIVFVSENVTQYLQYKQEDLVNTSVYNILHEEDRKDFLKNLPKSTV
NGVSWTNETQRQKSHTFNCRMLMKTPHDILEDINASPEMRQRYETMQCFALSQPRAMMEE
GEDLQSCMICVARRITTGERAFPSNPESFITRHDLSGKVVNIDTNSLRSSMRPGFEDIIR
RCIQRFFSLNDGQSWSQKRHYQEAYLNGHAETPVYRFSLADGTIVTAQTKSKLFRNPVTN
DRHGFVSTHFLQREQNGYRPNPNPVGQGIRPPMAGCNSSVGSMSMSPNQGLQMPSSRAYG
LADPSTTGQMSGARYGGSSNIASLTPGPGMQSPSSYQNNNYGLSMSSPPHGSPGLAPNQQ
NIMISPRNRGSPKIASHQFSPVAGVHSPMGSSGNSGNHSFSSSSLSALQAISEGVGGTSL
LSTLSSPGPKLDNSPNMNITQPSKVSNQDSKSPLGFYCDQNPVESSMCQSNSRDHLSDKE
SKESSVEGSENQRGPLESKGHKKLLQLLTCSSDDRGHSSLTNSPLDSSCKESSVSVTSPS
GVSSSTSGGVSSTSNMHGSLLQEKHRILHKLLQNGNSPAEVAKITAEATGKDTSSITSCG
DGNVVKQEQLSPKKKENNALLRYLLDRDDPSDALSKELQPQVEGVDNKISQCTSSTIPSS
SQEKDPKIKTETSEEGSGDLDNLDAILGDLTSSDFYNNSISSNGSHLGTKQQVFQGTNSV
GLKSPQSVQSIRPPYNRAVSLDSPVSVGSSPPVKNISAFPMLPKQPMLGGNPRMMDSQEN
YGSSMGGPNRNVTVTQTPSSGDWGLPNSKASRMEPMNSNSMGRPGGDYNTSLPRPAVGGS
MPTLPLRSNSIPGARPLLQPQQQQQMLQMRPGELPMGMGANPYGQAAASNQLGSWPDGML
SIEQVPHGTQNRPLLRNSLDDLVGPPSNLEGQSDERALLDQLHTLLSNTDATGLEEIDRA
LGIPELVNQGQALEPKQDAFQGQEAAVMMDQKAGLYGQTYPAQGPPMQGGFHLQGQSPSF
NSMMNQMNQQGNFPLQGMHPRANIMRPRTNTPKQLRMQLQQRLQGQQFLNQSRQALELKM
ENPTAGGAAVMRPMMQPQVKIAQGFLNAQMVAQRSRELLSHHFRQQRVAMMMQQQQQQQQ
QQQQQQQQQQQQQQQQQQQQTQAFSPPPNVTASPSMDGLLAGPAMPQAPPQQFPYQPNYG
MGQPPDPAFGRVSSPPSAMISSRMGPSQNPMMQHPQAASIYQSSEMKGWPSGNLARNSSF
SQQQFAHQGNPAVYSMVHMNGSSGHMGQMNMNPMPMSGMPMGPDQKYC

>Mus musculus_NCOA3(ENSMUSP_85416)
MSGLGESSLDPLAAESRKRKLPCDAPGQGLVYSGEKWRREQESKYIEELAELISANLSDI
DNFNVKPDKCAILKETVRQIRQIKEQGKTISSDDDVQKADVSSTGQGVIDKDSLGPLLLQ
ALDGFLFVVNRDGNIVFVSENVTQYLQYKQEDLVNTSVYSILHEQDRKDFLKHLPKSTVN
GVSWTNENQRQKSHTFNCRMLMKTHDILEDVNASPETRQRYETMQCFALSQPRAMLEEGE
DLQCCMICVARRVTAPFPSSPESFITRHDLSGKVVNIDTNSLRSSMRPGFEDIIRRCIQR
FFSLNDGQSWSQKRHYQEAYVHGHAETPVYRFSLADGTIVSAQTKSKLFRNPVTNDRHGF
ISTHFLQREQNGYRPNPNPAGQGIRPPAAGCGVSMSPNQNVQMMGSRTYGVPDPSNTGQM
GGARYGASSSVASLTPGQSLQSPSSYQNSSYGLSMSSPPHGSPGLGPNQQNIMISPRNRG
SPKMASHQFSPAAGAHSPMGPSGNTGSHSFSSSSLSALQAISEGVGTSLLSTLSSPGPKL
DNSPNMNISQPSKVSGQDSKSPLGLYCEQNPVESSVCQSNSRDHPSEKESKESSGEVSET

PRGPLESKGHKKLLQLLTCSSDDRGHSSLTNSPLDPNCKDSSVSVTSPSGVSSSTSGTVS
STSNVHGSLLQEKHRILHKLLQNGNSPAEVAKITAEATGKDTSSTASCGEGTTRQEQLSP
KKKENNALLRYLLDRDDPSDVLAKELQPQADSGDSKLSQCSCSTNPSSGQEKDPKIKTET
NEEVSGDLDNLDAILGDLTSSDFYNNPTNGGHPGAKQQMFAGPSSLGLRSPQPVQSVRPP
YNRAVSLDSPVSVGSGPPVKNVSAFPGLPKQPILAGNPRMMDSQENYGANMGPNRNVPVN
PTSSPGDWGLANSRASRMEPLASSPLGRTGADYSATLPRPAMGGSVPTLPLRSNRLPGAR
PSLQQQQQQQQQQQQQQQQQQQQQQQQQQQQMLQMRTGEIPMGMVNPYSPAVPSNQPGS
WPEGMLSMEQGPHGSQNRPLLRNSLDDLLGPPSNAEGQSDERALLDQLHTLLSNTDATGL
EEIDRALGIPELVNQGQALESKQDVFQGQEAAVMMDQKAALYGQTYPAQGPPLQGGFNLQ
GQSPSFNSMMGQISQQGSFPLQGMHPRAGLVRPRTNTPKQLRMQLQQRLQGQQFLNQSRQ
ALEMKMENPAGTAVMRPMMPQQAFFNAQMAAQQKRELMSHHLQQQRMAMMMSQPQPQAFS
PPPNVTASPSMDGVLAGSAMPQAPPQQFPYPANYGMGQPPEPAFGRGSSPPSAMMSSRMG
PSQNAMVQHPQPTPMYQPSDMKGWPSGNLARNGSFPQQQFAPQGNPAAYNMVHMNSSGGH
LGQMAMTPMPMSGMPMGPDQKYC

>Rattus norvegicus_NCOA3(ENSRNOP_7768)
MSGLGESSLDPLATESRKRKLPCDAPGQGLVYSGEKWRREQESKYIEELAELISANLSDI
DNFNVKPDKCAILKETVRQIRQIKEQGKTISSDDDVQKADVSSTGQGVIDKDSLGPLLLQ
ALDGFLFVVNRDGNIVFVSENVTQYLQYKQEDLVNTSVYSILHEQDRKDFLKHVPKSTVN
GVSWTNENQRQKSHTFNCRMLMKTHDILEDTNASPETRQRYETMQCFALSQPRAMLEEGE
DLQCCMICVARRVTTDRPFPSSPESFITRHDLSGKVVNIDTNSLRSSMRPGFEDTIRRCI
QRFFSLNDGQSWSQKRHYQEAYIHGHAETPVYRFSLADGTIVSAQTKSKLFRNPVTNDRH
GFVSTHFLQREQNGCRPNPNPVGQGIRPPAAGCGMSLSPSQSVQMLGSRTYGVADPSNTG
QMAGARYGASSSVASLTPGQSLQSPSSYQSNSYGLNMSSPPHGSPGLGPNQQNIMISPRN
RGSPKMASHQFSPAAGVHSPMGSSGNTGSHSFSSSSLSALQAISEGVGTSLLSTLSSPGP
KLDNSPNMNINQPSKASSQDSKSPLGLYCEQNPVESSVCPSNSRDHPSDKENKENSGEAS
ETPRGPLESKGHKKLLQLLTCSSDDRGHSSLTNSPLDSNCKDSSISVTSPSGVSSSTSGA
VSSTSNMHGSLLQEKHRILHKLLQNGNSPAEVAKITAEATGKDTSSTASGGEGSVRQEQL
SPKKKENNALLRYLLDRDDPSDVLAKELQPQADGGDSKLSQCSCSTNPSSGQEKDPKIKT
EASEEVSGDLDNLDAILGDLTSSDFYNSPTNGSHPGAKQQMFAGPSSLGLRSPQPVQSVR
PPYNRALSLDSPVSVGSVPPVKNVSAFPVLPKQPILAGNPRMMDSQENYGANMGGPNRNV
PVNPTSSSGDWGLANSRASRMEPLASSPLGRAGGDYSAALPRPALGSSGPTLPLRSNRLP
GARPTLQPQPQPQQQQQQQQQQQQQQQMLQMRAGEVPMGMGVSPYSPAVPSNQPGSWPEG
MLSMEQGPHGAQNRPLLRNSLDELLGPPSNPEGQSDERALLDQLHTLLSNTDATGLEEID
RALGIPELVSQGQALESKQDVFQGQEAAVMMDQKAALYGQTYPAQGPPLQGGFHLQGQSP
SLNSMMSQISQQGSFPLQGLHPRASMVRPRTNTPKQLRMQLQQRLQGQQFLNQSRQALEM
KMESPTGAAVMRPMLQSQAFFNAQMAAQQKRELMNHHLQQQRMAMMMSQPQPQAFSPPPN
VTASPSMDGVLAGSAMPQAPPQQFPYATNYGMGQPPEPAFGRGSSPPSAMMSSRMGPSQN
AMVQHPQTAPMYQSSEMKGWPSGNLARNGSFPQQQFAPQANPAAYNMVHMNSSGSHLGQM
TMTPMPMSGMPMGPDQKYC

>Canis familiaris_NCOA3(ENSCAFP_15958)
MSGLGENSLDPLAPDSRKRKLPCDTPGQGLTCSGEKWRREQESKYIEELAELISANLSDI
DNFNVKPDKCAILKETVRQIRQIKEQGKTISNDDDVQKADVSSTGQGVIDKDSLGPLLLQ
ALDGFLFVVNRDGNIVFVSENVTQYLQYKQEDLVNTSVYSILHEEDRKDFLKNLPKSTVN
GVAWTNETQRQKSHTFNCRMLMKTPHDILEDINTSPEMRQRYETMQCFALSQPRAMMEEG
EDLQSCMICVARRITTGERAFPSNPESFITRHDLSGKVVNIDTNSLRSSMRPGFEDIIRR
CIQRFFSLNDGQSWSQKRHYQEAYIHGHAETPVYRFSLADGTIVTAQTKSKLFRNPVTND
RHGFVSTHFLQREQNGYRPNTNPVGSGIRPPTAGCNSSVGGMSMSPNQGLQMLSSRPYGL
ADPSTTGQMSGARYGASNPIASMNPGPGMQSPSSYQNSNYGLNMSSPPHGSPGLGSSQQN
IMISPRNRGSPKMASHQFSPVAGVHSPMGSSGNTGSHSFSSSSLSALQAISEGVGTSLLS
TLSSPGPKLDNSPNMNISQPSKVNSQDSKSPLGLYCDQNPVESSMCQSNSRDHLSEKESK
ENIVEGSENQRGPLESKGHKKLLQLLTCSSDDRGHSSLTNSPLDSSCKDSSISVTSPSGV
SSSTSGGVSSTSNMHGSLLQEKHRILHKLLQNGNSPAEVAKITAEATGKDTSSTVSCVEG
NVKQEQLSPKKKENNALLRYLLDRDDPSDTLSKELQPKVEGVDNKMSQCSSSTIPSSSQE
KDSKIKTETNEEGSGDLDNLDAILGDLTSSDFYNNSISTNGSNLGTKQQMFQGTNSLGLR
SPQSVQAIRPPYNRAVSLDSPVSVGSSPPVKNISAFPMLTKQPMVGGNPRMMDGQENYGS
TMGGPNRNVTMNQTPSSGDWGLQNSKASRMEPMSTNSMGRPGADYNASLPRPAVGGSMPT
MPLRANSIPGARPVLQQQQQQQQQQQMLQMRPGEIPMGMGVSPYGQAAPSNQPGSWPDGM
LSMEQGPHGTQNRPLLRNSLDDLLGPPSNLDGQSDERALLDQLHTLLSNTDATGLEEIDR
ALGIPELVNQGQALESKQDAFQGQEAAVMMDQKAALYGQTYPAQGPPVQGGFNLQGQSPS

FNSMMNQMNQQGNFPLQGMHPRANMMRPRTNTPKQLRMQLQQRLQGQQFLNQSRQALEMK
MDSPTAGSAAVMRPMMQPQVTSQQGFLNAQMVAQRSRELLSHHFRQQRVAVMMQQQQQQQ
QQQQQQQQPQAFSPPPNVTASPSMDGVLAGPVMPQVPPQQFPYPPNYGMGQQPDPAFGRV
SSPPNAMMPSRMGPSQNPMMQHPQTAPMYQSSEIKGWPSGNLARNSSFPQQQFAHQGNPA
AYSMVHMNGSSGPMGQMNMSSMPMAGMPMGPDQKYC

>Felis catus_NCOA3(ENSFCAP_13944)
MSGLGENSLDPLAPDSRKRKLPCDTPGQGLTCSGEKWRREQESKYIEELAELISANLSDI
DNFNVKPDKCAILKETVRQIRQIKEQGKAISNDDDVQKADVSSTGQGVIDKDSLGPLLLQ
ALDGFLFVVNRDGNIVFVSENVTQYLQYKQEDLVNTSVYSILHEEDRKDFLKNLPKSTVN
GVAWTNETQRQKSHTFNCRMLMKTPHDILEDINTNPEMRQRYETMQCFALSQPRAMMEEG
EDLQSCMICVARRITTGERAFPSNPESFITRHDLSGKVVNIDTNSLRSSMRPGFEDIIRR
CIQRFFSLNDGQSWSQKRHYQEAYIHGHAETPVYRFSLADGTIVTAQTKSKLFRNPVTND
RHGFVSTHFLQREQNGYRPNANPVGQGIRPPAAGCNSSVGGMSMSPNQGLQMLSGRPYGL
ADPSSTGQMSGPRYGASSTIASMNAGPGMQSPSSYQNSTYGLNMSSPPHGSPGLGSNQQN
IMISPRNRGSPKMASHQFSPVAGVHSPMGSSGNTGSHSFSSSSLSALQAISEGVGTSLLS
TLSSPGPKLDNSPNMNISQPSKVNSQDSKSPLGLYCDQNPVESSMCQSNSRDHLSDKESK
ESSIEGSENQRGPLESKGHKKLLQLLTCSSDDRGHSSLTNSPLDSSCKDSSISVTSPSGV
SSSTSGGVSSTSNMHGSLLQEKHRILHKLLQNGNSPAEVAKITAEATGKDTGSAASGVEG
SVKQEQLSPKKKENNALLRYLLDRDDPSDPLSKELQPKVEGVDNKGSQCSSSAVPSSSQE
KDAKIKTEANEEGSGDLDNLDAILGDLTSSDFYNNSVSTNGSNLGTKQQMFQGTNSLGLR
SPQSVQSIRPPYNRAVSLDSPVSVGSSPPVKNISAFPMLPKQPMLGGNPRMMDSQENYGS
TMGGPNRNVTVNQTASSGDWGLPSSKTSRMEPLSSNSMGRPGADYNASLPRPAVSGSMPT
LPLRSAGIPGARPALPPQQPPQQQMLQMRPGEIPMGVGVSPYGQAAPSNQPGSWPDGMLS
VEQGSHGTQNRPLLRNSLDDLLGPPSNLEGQSDERALLDQLHTLLSNTDATGLEEIDRAL
GIPELVNQGQALESKQDAFQGQEAAVMMDQKAALYGQTYAAQGPPMQGGFNLQGQSPSFN
SMMNQMSQQGSFPLQGMHPRANIMRPRTNTPKQLRMQLQQRLQGQQFLNQSRQALEMKME
NPTAGGAAVMRPMMQPQVNPQQGFLNAQMVAQRSRELLSHHFRQQRVAMMMQQQQQQQQQ
QQQTQAFSPPPNVTASPSMDGVLAGPVMPQAPPQQFPYPPNYGMGQQPDAAFGRVSSPPN
AMMPSRMGPSQNPMMQHPQTAPMYQSSEIKGWPSGNLARNSSFPQQPFAHQGNPAAYGMV
HMNGSSGPMGQVNVNPMPMSGMPMGPDQ

>Myotis lucifugus_NCOA3(ENSMLUP_383)
MSGLGENSLDPLVPDSRKRKLPCDTPGQGLTCSGEKWRREQESKYIEELAELISANLSDI
DNFNVKPDKCAILKETVRQIRQIKEQGKAISNDDDVQKADVSSTGQGVIDKDSLGPLLLQ
ALDGFLFVVNRDGNIVFVSENVTQYLQYKQEDLVNTSVYNILHEEDRKDFLKNLPKSTVN
GVTWTNETQRQKSHTFNCRMLMKTPHDIVEDMNTSPEMRQRYETMQCFALSQPRAMMEEG
EDLQSCMICVARRITTGERAFPSNPESFITRHDLSGKLVNIDTNSLRSSMRPGFEDIIRR
CIQRFFSLNDGQSWSQKRHYQEAYIHGHAETPVYRFSLADGTIVTAQTKSKLFRNPVTND
RHGFVSTHFLQSREQNGYRQNPNPVGQGIRPPAAGCNSVGGMSMSPNQGLQMLNSRAYGL
ADPGTTGQMSGARYGASSNIATMNPGPAMQSPSSYQNNNYGLNMSSPPHGSPGLGSNQQN
IMISPRNRGSPKMASHQFSPVADPGVHSPIGSSGNTGSHSFSSSSLSALQAISEGVGTSL
LSTLSSPGPKLDNSPNMNITQPSKVNSQDSKSPLSLYCDQNPVESSMCQSNSRDHLNDKD
SKESSVEGSENQRGPLESKGHKKLLQLLTCSSDDRGHSSLTNSPLDSSCKDSSISVTSPS
GVSSSTSGGVSSTSSMHGSLLQEKHRILHKLLQNGNSPAEVAKITAEATGKDTSSATFCV
EGNVKQEQLSPKKKENNALLRYLLDRDDPSDTLSKEVQPKVEGVDNKMSQCSSSTVPSSS
QEKDAKIKTEANEEGSGDLDNLDAILGDLTSSDFYSNSVSTNGSNLGTKQQMFQGTNSLG
LRSPQSVQSIRPPYNRAVSLDSPVSVGSSPPVKNITAFPMLPKQPMLGGNPRMMDSQENY
GSNMGGPNRNVTMNQTPSSGDWGLSNSKASRMESMSSNSMGRPGADFNASLPRPTMGGSM
PTLPLRSNSIPGARPMLQQQQQQQQQRPGEIPMGMGVSPYGPAAPSNQSGSWPDGMLSME
QGPHGTQNRPLFRNSLDDLLGPPSNLEGQNDERALLDQLHTLLSNTDATGLEEIDRALGI
PELVNQGQALEPKQDVFQGQEATVMMDQKAAFYGQMYPAQGTPMQGGFNLQGQSPSFNSM
MSQMNQQGNFPLQGMHPRANLMRPRTNTPKQLRMQLQQRLQGQQFLNQSRQALEMKMENP
TAGGAAMMRPMMQPHILYKQGFLNAQMVAQRSRELLSHHFRQQRVAAMMMQQQQQQQQQQ
QTQAFSPPPNVTASPSMDGVLAGPAMPQAPPQQFPYQPNYGMGQQPDPAFGRVSSPPNAM
MSSRMGPSQNPMMQHPHTAPMYQSSEMKGWPSGNLARNSSFPQQQFAHQGNPAYNMVHMN
GSSGPMGQMNMNSMPMSGMPMGPEQKYC

>Monodelphis domestica_NCOA3(ENSMODP_20305)
MSGLGENSLDPLTTDSRKRKLPCDTPVQGLTSSGEKWRREQESKYLEELAELISANLSDI
DSFNVKPDKCAILKETVRQIRQIKEQGKAASIDDDVQKADVSSTGQGVIDKDSLGPLLLQ

ALDGFLFVVNRDGTIVFVSENVTQYLQYKQEDLVNTSVYSILHEDDRKDFLKNLPKSAVN
GDPWTSETPRQKSHTFNCRMLVKTVHDILDDVGNSPDMRQRYETMQCFALSQPRAMMEEG
EDLQSCMICVARRLTAVERVFPSNPESFITKHDLSGKVITIDTNSLRSSMRPGFEDIIRR
CIQRFFSLNDGQSWSQKRHYQEAYIQGHAETPVYRFSLADGTIVSAQTKSKLFRNPVTND
RHGFLSTHFLQREQNGYRPTSNPVGQNIRAPTVGCSSSVGGMNMSPNQSLPIQNRPYSLG
DPNAVGQMSGIRYGAPNNLNSINLGPGMQSSPYQNNNYGLTMNSPPHGSPGLGSNQQNIM
ISPRNRGSPKMPSHQFSPVPGMHSPMGSTSNTGGNSFSSSSLSALQAISEGVGTSLLSTL
SSPGPKLDNSPNMNINQQSKVGSQDSKSPVGLYCEQSQVESSICQVNSRDHPCDKDSKET
GVEGPENQRGPLESKGHKKLLQLLTCSSDDRGHSTLTNSPLDPGCKDSASSVTSPSGVSS
STSGGVSSTSNMHGSLLQEKHRILHKLLQNGNSPAEVAKITAEATGKDTSSAASSEGIVK
QEQLSPKKKENNALLRYLLDRDDPNDSISKDMKPKVEGVDNKMGQCNSSSLPISNQEKEI
KVKTEQNEEVSGDLDNLDAILGDLTSSDFYNNAMSTNGNNLANKQQMYQGSTPLGLRSPQ
SLPSARPPFNRAVSLDSPIPVGSSPPVRNVNTFPMLPKQPVVSGNSRMMDTQENFTSNMV
LGGRNKNFFLTLLPPGEGVFPIFKVAGRDQTPVSLGGPGPVYNFFFPSPPRGGIFPVLPF
RPNGIPGPGPFLPRQMFQRGPGEIPMGMGVNAYGQTGPSITPGSWPDNMLTMDQGPRGTQ
NRALVRNSLDDLLCPPPNIEGQSDERALLDQLHTLLSNTDATGLEEIDRALGIPDLVNQG
QALEAKQESFQSQESAVMIDQKSALYGQSYQAQSAVIQGSFSSLQGQQQSFNTMMNQMSQ
QGNFPLQGIHPRANMMRPRTNTPKQLRMQLQQRLQGQQFLNQSRQALEMKMETPPTSGAT
VMRPVMQAQVNSQQGFLNAQMVAQRSRELISHHIRQHRMAVMMQQQQQQQTQQQQQTQA
FSPPPNVTASASIDGALAGTPMPQAPPQQFSYPPNYGMSQQSDPAFSRVSSPPSAMMSAR
IGASQNPMMQHPQATPMYQSPEMKGWPSGTLSRNSSFPQQQFPPQGSPAPYSMMHMNGNS
GHIGQMNMNSMSMSGMPMGPDQKYC

>Gallus gallus_NCOA3(ENSGALP_7263)
MVRMSGLGESSLDSLTNESRKRKPLPCDTPGASLTCSGEKRRREQESKYIEELAELISAN
LSDIDNFNVKPDKCAILKETVRQIRQIKEQGKAVSNDDEVQKADVSSTGQGVIDKDSLGP
LLLQALDGFLFVVNRDGNIVFVSENVTQYLQYKQEDLVNTSVYGILHEEDRKDFLKNLPK
SSVNGVAWTNDAPRQKSHTFNCRMMVKTSHDLLEDVGSHQDTRQRYETMQCFALSQPRAM
MEEGEDLQSCMICVARRIATAERVFPTNPESFITRHDLSGKLVNIDTNSLRSSMRPGFED
TIRRCIQRFLCHNDGQSWSNKRHYHEAYTQGHAETPLYRFSLADGTIVTAQTKSKVFRNP
VTNDRHGFVSTHFLQREQNGYRPNPNTVGQNIRTTVAGNTNSVGLMNMSPGQGMPMQNRN
YGMGDPSTVGQLNSSRYGGPGNMGPVNSGPGMQSSAYQSNSYGLNISSPPHGSPGLTSNQ
QNLMISTRNRGSPKMNSHQFSPVTGMHSPMGSASNTSNSTFSSSSLSALQAISEGVGTSL
LSTLSSPGPKLDSSPNVSIAQQNKANNQDSKSPSGLYCEQNQVESSICQSNSRDVLSEKD
SKDGSLDASESQRGQSESKGHKKLLQLLTCSSDERGHSTASNSPLDSNCKESSTNVTSPS
GVSSSTSGGVSSSSNVHGSLLQEKHRILHKLLQNGNSPAEVAKITAEATGKDTYHDTSNT
VPCGESTVKQEQLSPKKKENNALLRYLLDKDDVKDPLSKELKPKVENVDNKMGQCSSSTI
PTSSQEKEMKIKTEPTEEMSGDLDNLDAILGDLTSSDFYNNAMSTSGNNFGMKQALFQGN
ASLTGMRSPQSVQAARPPFNRAMSLDSPVGSGASVRNVNAFSMLQKQNLMGGSPRMTENQ
ESFGANIASTPNRGVSMNQHQSGDWGLPNSKVNRLEPTSSTSMLRQGAEFSASLPRPTAG
GSMPGLPVRSNSIPGTRPMLQQQMMHMRSNEINIGMGGNPYGQTGPTSQPGSWPDSMLSM
EQASRGLQNRQLVRNSLDDLLSAAPSVEGQNDERALLDQLHTLLSNTDVTSLEEIDRALG
IPDLVNQGQTLEPKQDSFQGQESTVMMDQKPSLYGQPYQGQGTALPGGFNNIQGQQPSFN
SVMNQMSQQNNFPLQSMHPRANAMRPRTNTPKQLRMQLQQRLQGQQFMNQTRQALEMKME
NPGSSNSSVMRPVMQPQVGSQQQGFLNAQMVAQRNRELISHHFRQQRMAMMMQQQQPQAF
SPPPNVTASGSMDSGLTGPPMAQVPPQQFSYPPNYGISQQPDPAYSRVSSPPNPMMASRI
GPSQNPMMQHPQTAPMYQSPEMKGWPSGSMARSSSFPQQQFSHQGNPATYSMMHMNGSSG
HIGQMNITTVPMSGMPMGPDQKYC

>Anolis carolinensis_NCOA3(ENSACAP_16172)
MIRMSGLGENSLDSMTSDSRKRKPLPCDTPGPGLICSGEKRRREQESKYIEELAELISAN
LSDIDNFNVKPDKCAILKETVRQIRQIKEQGKAASADDDVQKADVSSTGQGVIDKDSLGP
LLLQALDGFLFVVNRDGNIVFVSENVTQYLQYKQEDLVNTSIYSILHEEDRKDFLKNLPK
SAVNGVSWTNEAPRQKSHTFNCRMMVKTIHDLLEDVGSNQDTHQRFETMQCFALSQPRAM
LEEGEDLQSCMICVARRISAMERVFSPNTESFVTRHDLSGKLVNIDTNSLRSMKPGCEDT
IRRCIQRFLCQNEGQPWSNKRHYQEAYIQGHSETPFYRFSLADGTIVIAQTKSKLFRNPV
TNDRHGFISTHFLQREQNGYRPNPGSMGQNIRAPTTGGTNSGGGVMNMLPGQNMPLQNRT
YGMGDPNLMGQMSSARYGAHGNMGSMNAAPGMQSSPYQSNYGLNMSSPPHGSPGLNSSQQ
NLMISPRNRGSPKVNSHQYSPVTGVHSPMGPASNTSNNFSSSSLSALQAISEGVGTSLLS
TLSSPGPRLDNSPNMNVSQQSKMGNQDSKSPPGIFPEQNQVESSMCQSNSRDSLGNKDSK
DESLEGSDQRGPAESKGHKKLLQLLTCSSEDRGHSTLSNSPLDSTCKDSSTNATSPSSGV

SSSTSGGVSSSNMQEKHRILHKLLRNGNSPAEVAKITAEATGKDTYHDSSTVSCGEGMVK
QEQMSPKKKENHALLRYLLDKDDAKDSLSKDIKPKIECLDSKMGQCCNSAIPTSSQEKEM
KIKTEPTEEMSGDLVDNLDAILGDLTNSDFCSNPISTNGSNIGNKQPLCQGTVPLGMRSP
QPVQNTRPPFNRAMSLDSPISVGSSAPVRGINAFSMLQKQSMMGGSPRITENQENFATNM
GGSSRNMPMNQHSASDWSVQNSKVSRIEPTSAGSMTRPGADCNLSLSRSSVGGSMPGLPM
RSNSMPGTRTMLQQQMIHMRPNEINMGMGGNPYVHPGPSNQPNAWPDSMLSVEQGPRGSQ
NRQLVRNSLDDLLYSSPTVDGQSDERALLDQLHTLLSSTDVTSLEEIDRALGIPDLVNQG
QPLEPKQEPFHGQEPAVMIEQKPPMYSQPYQGQGAAIPGGFSSMQGQQAAFNSVMNQMNQ
QSNFPMQNMHPRANVMRPRTNTPKQLRMQLQQRLQGQQFLNQTRQTLDIKMENPNPGNAS
VMRPVMPPQMGSQQQGFLNAQMVAQRNRELISHHIRQQRMAMMMQQQQQQSQTFSPPPNV
TASGSMDGAVAGPPMAPVPPQQFSYPSNYGMNQPPDPAFSRVSSPPNPLMAQRMGPSQNP
MMQHPQATPMYSSPEMKGWPSGSMARSSSFPQQQFSHPGNPAAYNMLHMNSNGGHLGPMN
INTMPMSGMPMGPDQKYC

>Xenopus tropicalis_NCOA3(ENSXETP_7533)
CVFTMSGLGENSLDPLAAETRKRKPSSCDTPGPGLTCSGEKRRREQESKYIEELAELISA
NLSDIDNFNVKPDKCAILKETVRQIRQIKEQGKASSNDDDVQKADVSSTGQGVIDKDSLG
PLLLQALDGFLFVVNREGSIVFVSENVTQYLQYKQEDLVNTSVYSILHEEDRKDFLKNLP
KSTVNGVPWSSETPRQKSHTFNCRMLVKTSHDHLEDGSGNLDTRQRYETMQCFALSQPRA
MIEEGEDLQSCMICVARRITTVERVFSANPESFITRHDLTGKVVNIDANSLRSSMRPGFE
DTIRRCIQRFLCHSDGQPWTYKRHYQEAYVHGHSETPLYRFSLADGTIVTAQTKSKLFRI
PVTNDPHGFVSTHFLQREQNGYRPNPNPMAQNIRPPVNPNPPNTMNAMPPQAMQQQNRNY
GMGDPNSMAQMPGMRYKSPGNMAPVNQPPGVQQSPYQNNSNYGLNMNSPPHGSPGMNSNQ
PNLMVSPRNRASPKMASNQFSPVPGMNSPMGSSGSAGGGSFSSSSLSALHAISEGVGSSL
LSSLSSPGQKVENNTNMNMPQQGKIGNQDCKSPSGLYCEQGQVESSVCQSSGREHLGEKD
VKENILEASDSQRSQAESKGHKKLLQLLTGSSEDRGPSLMSSSSIDCKDSSTNVTSPSGV
SSSTSTGVSSTSNLHGSMLQEKHRILHKLLQNGNSPAEVAKITAEATGKETVSSAPCTEA
TVKREQLSPKKKENNALLRHLLDKDDWKDPLSKDIKPKVEHMDAKMGPCSSSSVPTSSQD
KEVKIKTEPADEVPGDLDNLDAILGDLAGSDFYNNSMSSRASDHGPKQPVFQDSPTLGMR
SPDSVHGSRPPFNRAISLDSTMPGNSTPPVRNVNSFPMLPKQGMMGSPRMMDGQDNFGVS
GSGPNRSMNQHPGGEWAMQNAAVNRLEPPNAGNVGRPGPDYSTAMPRPAMGGNMPGLPAR
SNSIPGSRPVLQQQMLPMMLGPNEMAMGMGSNPYGQQAPSNPPGSWPDGMMIDQGHGGTQ
ARQLGRNSLDDLLCPPSTVEGQTDEIALLDQLHTLLSNTDATGLEEIDRALGIPDLVSQG
QALEPQQDSYQPQGSPVMIDQKPPMYGQPYAGQGTAMAAAGFNAMQGQHPPFNSVMGQMN
QQQGNFPLQGMHPRANLMRPRNNIPKQLRMQLQQRLQGQQFLNQNRQALEMKVETMNPGG
AGVMRPVMQTPVSQQGFLNAQMVAQRNRELISHQIRQHRMAMMMQQQQGQPQTFSPPPN
VTATASMDNPLGGPPMPQAPPQQFSYPPNYGMNQQADPTFGRVSSPPNAIMSSRMAPSQN
PHPQTAQMYPSPDMKGWPSGNMTRPNSFPQQQYSHQANPATYNMMHMNGNGSHMGQINMN
SMPMSGMPMGPDQKYC

>Danio rerio_NCOA3a(ENSDARP_102117)
MSGVGENSLEPLCSDRKRKLSTCDTPGQGCDKRRREQESKYIEELAELISANLSDIDSFN
VKPDKCAILKETVRQIRQIKEQGKSSCGDDDVQKADVSSTGQGVIDKDHLGPLLLQALDG
FLFVVSREGSIVFVSDNVTQYLQYKQEELINTSIYNILHEEDREELHKNLPKSNGPNSVS
WGGDSSRQKSHTFNCRMLVKFGHGGPGGEEGAGGPRYETMQCFALTQPKAMMEEGEDLQS
CMICVARRVTAVERTERFSTRHELSGKLIEIEQQSLLHTTMRPGWEDLVRRCMQMFLHRS
EGQPWSYKRHYHEAYVHGRAETPPYRFSLSDGTPVTAQTRSELCRNPVTSDPHTFLSTHL
LQREQNGYRPNQGGVMRSVMGNANPNAQMGMGNTRGYGMNEQGHMGPRGGAMYGPGNRMN
QMNPMHQMNQMNQMNSMNQMNMNQMNCMNQMGSMNQMNQMNQMNSMNQMGHPGMNQHQQG
QFHGGGGYGMVMTSPSQGSPGMNPGQQNLMGSPRIRGSPKMGASPFSPGGMHSPMGPVGM
GGSSGSSGPSVGNTFSSSSLNALQAISEGVGSSLPSALNSPAHKSDSSPNINSTQSSQQG
PIGNNKPITSDSKSPSSAHSTGLDQQQASSTENTDKPDSQSNKDGSAGGEVNRRVPDGKG
HKKLLQLLTSPTEEIGIGTATSGPVPGRASTPMGSDPKETGSGMMSPSSTGVSSSSGSSS
GNNVNQQSSGGVSSTACPSGHHSTHTLQEKHKILHKLLQNGNTPDDVARITAEATGKSSL
SSEHPGAEGRNLELKQEQHSPKEGKKPQALLHYLLNKDDSKEPGATEVKPKLEELEARGA
SGGQGAGVTTSSSSNQESKVKLEQPDELESLESILGGLRNPGPGMFVDSGSGGSEVGNKQ
GNGPNSTAHDREGVGMGSGQRVPFQRAVSVDGKPMSGPGMTGRKSAPSSLVKQEHTDSQI
GMGGPNRLMGMPNRPPMAGAGDWMSSSGSPAGSVGHPGMGRPGMDHNPKSMMGGPMVSRS
NSVPATRSMLQQQLMEMGPYDVGMGMNSFRGQAPPPQSPSWPDSGMGMEGPPSANRLPIN
SPLDELLAPPSTSEGQSDERALLDQLDSLLNNTDVIALEEIDRALGIPDLINQGHSSDQP
PPSDPFSGPCLGPDSSMGMEPPKAMYGQGYPGPPSMGMQGGYGPGPNPGAGPGPGPMQGQ

SGPGFNPMMSQMNQQGGFPGMGGMGPMHPRANMMRNRVPQAKPLRLQLQQRLQGQQFLNQ
SRQAMGMRPDASPAGNPGMRPSMQPGMGGQPGFLNAQMMAQRSREMLNLQTEMRRQRMMM
MMQQQQQQQQQVAAGGFSPPPNVTAPTGMDNPMAGPPMSQPGQQAFNYGGNYGIGQQGDP
SFVGTGSSPPNNMMSGRMGGPQNPMMQQHPQSNPMYQSAEMKGWGQGPMPMNNSYPQQQQ
YPQQGAPGQYGGMMMNGSMQGGVNGNGAGQMPPMQGQMGMNTMGMGRMPMGPDQKYC

>Danio rerio_NCOA3b(ENSDARP_142017)
MSGVGENSLEPLCSDRKRKLSTCDTPGQGCDKRRREQESKYIEELAELISANLSDIDSFN
VKPDKCAILKETVRQIRQIKEQGKSSCGDDDVQKADVSSTGQGVIDKDHLGPLLLQALDG
FLFVVSREGSIVFVSDNVTQYLQYKQEELINTSIYNILHEEDREELHKNLPKSNGPNSVS
WGGDSSRQKSHTFNCRMLVKFGHGGPGGEEGAGGPRYETMQCFALTQPKAMMEEGEDLQS
CMICVARRVTAVERTERFSTRHELSGKLIEIEQQSLLHTTMRPGWEDLVRRCMQMFLHRS
EGQPWSYKRHYHEAYVHGRAETPPYRFSLSDGTPVTAQTRSELCRNPVTSDPHTFLSTHL
LQREQNGYRPNQGGVMRSVMGNANPNAQMGMGNTRGYGMNEQGHMGPRGGAMYGPGNRMN
QMNPMHQMNQMNQMNSMNQMNMNQMNCMNQMGSMNQMNQMNQMNSMNQMGHPGMNQHQQG
QFHGGGGYGMVMTSPSQGSPGMNPGQQNLMGSPRIRGSPKMGASPFSPGGMHSPMGPVGM
GGSSGSSGPSVGNTFSSSSLNALQAISEGVGSSLPSALNSPAHKSDSSPNINSTQSSQQG
PIGNNKPITSDSKSPSSAHSTGLDQQQASSTENTDKPDSQSNKDGSAGGEVNRRVPDGKG
HKKLLQLLTSPTEEIGIGTATSGPVPGRASTPMGSDPKETGSGMMSPSSTGVSSSSGSSS
GNNVNQQSSGGVSSTACPSGHHSTHTLQEKHKILHKLLQNGNTPDDVARITAEATGKSSL
SSEHPGAEGRNLELKQEQHSPKEGKKPQALLHYLLNKDDSKEPGATEVKPKLEELEARGA
SGGQGAGVTTSSSSNQESKVKLEQPDELESLESILGGLRNPGPGMFVDSGSGGSEVGNKQ
GNGPNSTAHDREGVGMGSGQRVPFQRAVSVDGKPMSGPGMTGRKSAPSSLVKQEHTDSQI
GMGGPNRLMGMPNRPPMAGAGDWMSSSGSPAGSVGHPGMGRPGMDHNPKSMMGGPMVSRS
NSVPATRSMLQQQLMEMGPYDVGMGMNSFRGQAPPPQSPSWPDSGMGMEGPPSANRLPIN
SPLDELLAPPSTSEGQSDERALLDQLDSLLNNTDVIALEEIDRALGIPDLINQGHSSDQP
PPSDPFSGPCLGPDSSMGMEPPKAMYGQGYPGPPSMGMQGGYGPGPNPGAGPGPGPMQGQ
SGPGFNPMMSQMNQQGGFPGMGGMGPMHPRANMMRNRVPQAKPLRLQLQQRLQGQQFLNQ
SRQAMGMRPDASPAGNPGMRPSMQPGMGGQPGFLNAQMMAQRSREMLNLQTEMRRQRMMM
MMQQQQQQQQQVAAGGFSPPPNVTAPTGMDNPMAGPPMSQPGQQAFNYGGNYGIGQQGDP
SFVGTGSSPPNNMMSGRMGGPQNPMMQQHPQSNPMYQSAEMKGWGQGPMPMNNHPETVMK
MCEYNFIPPATAISPAGGARTVWWNDDERLHAGGCEWQRCRTDAPNAGPDGHEHHGHGTH
ANGT

>Takifugu rubripes_NCOA3a(ENSTRUP_10584)
MSGLGENSMEPLSSENRKRKLSTCDTPGLGCERKRREQESKYIEELAELISANLSDIDSF
NVKPDKCAILRETVRQIRQIKEQGKASSSDDDVQKSDVSSTGQGVIDKDHLGPLLLQALD
GFLFVVNREGSIVFVSDNVTQYLQYKQEELINTSVYNILHEDDREELHKNLPKTNLNGVS
WGGETARQKSHTFTCRMLVKFGHGSMEEGPGGPRYETMQCFALTQPKAMIEEGEDLQSCM
ICVARRVTAMERTESFSTRHELSGKLIQIDQNSVRASMRPGWEDLLRRCIQMFLQHSDGQ
PWSHKRHYHEAFLQGHAETPLYRFSMSDGTPVTAQTKSKLYRHPMSNEPQGFISTHLLQR
EPNGYRSAQSGNMMPNTMRQQGMGGPQMNPQMSMNPGGGMGGMGGMSRGFGMSEQGHMGH
MGPMGGGSMNQMGPMNQQHPQQPPFQSSGGFGLSGMNSPSGSPRMSGPQQGLLMSPRNRG
SPKMGANQFSPGAGLHSPMSGIGSGLGSGGSGTTFSSSSLNALQAISEGVGSSLPSTLTS
PSPVLKPDSSPSIHSSSSSCPGDHHHPVHHHHHLQHPQAESSGDRPDSQATAAPKESGEG
SSEAGLASSEPQRRVPDSKGHKKLLQLLTSPTEELGIGGSGPTAPPLQPPPTSSNTPASS
DSKDPTGGMTSPSSTGVSSSSAIVNQGQASAVASLSSAHYTGSLQEKHKILHKLLQNGNT
PDEVAKITAEATGKVTLGSQEGGEGGAAGTGGGAGPGMTETKQEQHSPKKEKTHALLHYL
LNKDDSKEPADVKPKMEELDGKVAPGAVGGPVRAGPGSGPSSVPDHPESKIKSEPPDDLH
NLESILGVLSGSGSDLFPDQTGGGGANDAGNKPQGCLDDNLRVFRPGSPGIGPGPQGPFQ
RSMSMDGKPPGGPGGAGRRPMLIKQENMMGSPDGYPGNMGVCRSPMGRGMVPQRSPMGGS
GDWGMPRSSASPVGSMMRPGLEYTNNKAMMSGPMVGRSNSVPGTSSMLQRQMNRLWGLGG
SADMGMGMSPFNQQGQPSQSPSWSDPMMGMEGNRRQFGNTLDDLLVPPTTSEGQSDERAL
LDQLDSLLNNTDGIALEEIDRALGIPDLVSQTQGAEPPIESFPGQDPAMVLDQKPIYGQG
YPGPPAMPMQPGYGGNPMQGQAPQGGFGPMLSQMGQGGSYPGMGAMGGMGHPRANMMRPR
MMAANKPMRLQLQQRLQGQQFMNQTRQGMAMKMENMGNPGMRPAMQPGMGGQPGFLNAQM
MAQRSREMVTMQMRRQRMMMLMQQQQQAGAAAGGFSPPPNVTAPGGMDNPMGGPSMGQPG
PQQFGYGGNYGMGQQGDPSFGPSGGSPPNAMMPGRMGPQNPMMQQHPQGGPMYQGTDMKG
WGQGGMARNSSYAQQQFGQQGNPGQYGGMMMNGGMPPNGGGGHMGQMGGQMGMNPMAMGR
MPMGPDQKYC

>Takifugu rubripes_NCOA3b(ENSTRUP_10582)
MSGLGENSMEPLSSENRKRKLSTCDTPGLGCERKRREQESKYIEELAELISANLSDIDSF
NVKPDKCAILRETVRQIRQIKEQGKASSSDDDVQKSDVSSTGQGVIDKDHLGPLLLQALD
GFLFVVNREGSIVFVSDNVTQYLQYKQEELINTSVYNILHEDDREELHKNLPKTNLNGVS
WGGETARQKSHTFTCRMLVKFGHGSMEEGPGGPRYETMQCFALTQPKAMIEEGEDLQSCM
ICVARRVTAMERTESFSTRHELSGKLIQIDQNSVRASMRPGWEDLLRRCIQMFLQHSDGQ
PWSHKRHYHEAFLQGHAETPLYRFSMSDGTPVTAQTKSKLYRHPMSNEPQGFISTHLLQR
EPNGYRSAQSGNMMPNTMRQQGMGGPQMNPQMSMNPGGGMGGMGGMSRGFGMSEQGHMGH
MGPMGGGSMYGGGGSGGGGNRMMQMNQMGPMNQSSGGFGLSGMNSPSGSPRMSGPQQGLL
MSPRNRGSPKMGANQFSPGGLHSPMSGIGSGLGSGGSGTTFSSSSLNALQAISEGVGSSL
PSTLTSPSPVLKPDSSPSIHSSSSSSQLTQTAKGGPDGSKSPAGGLGPESSGDRPDSQAT
AAPKESGEGSSEAGLASSEPQRRVPDSKGHKKLLQLLTSPTEELGIATGVSSSSAIVNQG
QASAVASLSSAHYTGSLQEKHKILHKLLQNGNTPDEVAKITAEATGKVTLGSQEGGEGGA
AGTGGGAGPGMTETKQEQHSPKKEKTHALLHYLLNKDDSKEPADVKPKMEELDGKVAPGA
VGGPVRAGPGSGPSSVPDHPESKIKSEPPDDLHNLESILGVLSGSGSDLFPDQTGGGGAN
DAGNKPQGCLDDNLREKASKGPEIRNLIQLMSDHFYLIFRPGSPGIGPGPQGPFQRSMSM
DGKPPGGPGGAGRRPMLIKQENMMGSPDGYPGNMGPMGRGMVPQRSPMGGSGDWGMPRSS
ASPVGSMMRPGLEYTNNKAMMSGPMVGRSNSVPGTSSMLQRQLIGGGSADMGMGMSPFNQ
QGQPSQSPSWSDPMMGMEGNRRQFGNTLDDLLVPPTTSEGQSDERALLDQLDSLLNNTDG
IALEEIDRALGIPDLVSQTQGAEPPIESFPGQDPAMVLDQKPIYGQGYPGPPAMPMQPGY
GGNPMQGQAPQGGFGPMLSQMGQGGSYPGMGAMGGMGHPRANMMRPRMMAANKPMRLQLQ
QRLQGQQNSLALLNPHSSVMASVHANLQPGFLNAQMMAQRSREMVTMQMRRQRMMMLMQQ
QQQAGAAAGGFSPPPNVTAPGGMDNPMGGPSMGQPGPQQFGYGGNYGMGQQGDPSFGPSG
GSPPNAMMPGRMGPQNPMMQQHPQGGPMYQGTDMKGWGQGGMARNSSYAQQQFGQQGNPG
QYGGMMMNGGMPPNGGGGHMGQMGGQMGMNPMAMGRMPMGPDQVCNTVLIPRCFPNNLGV
FRVNKGHERTKRRHC

>Tetraodon nigroviridis_NCOA3a(ENSTNIP_15572)
ARTMSGLGENSMEPLSSETRKRKLSTCDTPGLGSSCRCERKRREQESKYIEELAELISAN
LSDIDSFNVKPDKCAILRETVRQIRQIKEQGKASSSDDDVQKSDVSSTGQGVIDKDHLGP
LLLQALDGFLFVVNRDGSIVFVSDNVTQYLQYKQEELINTSVYNILHEDDREELHKNLPK
TTLNGVSWGGETARQKSHTFTCRMLVKFGHGPAEEGPGGPRYETMQCFALTQPKAMIEEG
EDLQSCMICVARRVTATERTESFSTRHELSGKLIQIDQNSVRASMRPGWEDLLRRCIQMF
LQHSDGQPWSHKRHYHEAFLQGHAETPLYRFSLSDGTPVTAQTKSKLYRHPMSNEPQGFI
STHLLQREPNGFRSAPNAMRQPAMGGPAINMTPGGGMGGMSRGFGMNEQAHVGPMGGAAM
SHPGQAMQPHPQPHPQQPPFQSSGGFGLGGMNSPSGSPRMSGPQQGLLMSPRNRGSPKMG
ANQFSPGAGIHSPMSGIGSGLGSGGGGTTFSSSSLNALQAISEGVGSSLPSTLTSPSPVL
KPDSSPSIHSSSSLSQLTQTAKGGLDGSKSPAGGLGPGPGPQAESSGDRPDSQATASVKE
PGEGGSDAGVAGSEPQRRVPDSKGHKKLLQLLTSPTEELGMGGGSTTPAGSDSKDPAGGM
TSPSSGVSSSSSSGVNPGPASAAASLASAHYTGSLQEKHKILHKLLQNGNTPDEVAKITA
EATGKVTLGGQEGGEAGVAGTGAGSAPGMPETKQEQHSPKKEKPHALLHYLLNKDDSKEA
ADVKPKLEELEGKAAPGGPVRPGPGPGSAPDHPEIKIKSEPPDDLHNLESILGVLSGSGP
DLFPDQTGGGGANEGGNKPQGCLDDNLRGSPGIGPGPQGPFQRSMSMEGKPPGGPGGAGR
RPMLVKQENMMGSPDGYHGNMGPMNRGMVPQRSPMGGSGDWGMARSSASPLGSMMRPGLE
YSSSKSMMVGRSNSVPGTTSRLQQQLALATLCGGSADMGMGMGPFSQQGQPSQSPSWDPM
MAMEGNRRQFGNTLDDLLVPPTTSEGQSDERALLDQLDSLLNNTDGIALEEIDRALGIPD
LVSQTQGAEPPIESFPGQDPAMVLDQKPLYGQGYPGPPAMPMQSGYGGNPMQGQAQQGGF
GPMLSQMGQGGNFPGMGGLGGMPRANMMRPRMMTANKPMRLQLQQRLQGQQYMNQTRQNM
SMKMENMGNPAMRPGMQPGMGAQPGFLNAQMMAQRSREMVAMQMRRQRMMMLRAAAGGFS
PPPNVTAPGGMDNPMAGPNMGQPGPQQFGYGGSYGMGQQGDPSFGPSGASPPNAMMPARM
GPQNPMMQQHPQGGAMYQSADMKAWGQGGMARNSSYAQQQFGQQGNPGQYGAMMMNGGMP
ANGAGGHMGQMGGQMGMNPMTMGRMPMGPDQKYC

>Tetraodon nigroviridis_NCOA3b(ENSTNIP_10107)
AVKMSGIGDNSLEPLCSDRKRKLSTCDTPGLGCDKRRREQESKYIEELAELISANLSNID
SFNVKPDKCAILKETVRQIRQIKEQGKSACSDDDVQKADVSSTGQGVIDKDHLGPLLLQA
LDGFLFVVNREGSIVFVSDNVTQYLQYKQEELINTSVYNIIHDEDREEFHKNLPKSNVVS
NGAWGGETPRQKSHTFNCRMLVNTVHGQNHGLTEERPPGGQRYETMQCFALTQPRAMMEE
GEDLHSCMICVARRIIVVERTERFSTRHELSGKLVEIEQQASLHTTMRPGWEDLVRRCMQ
MFLHRTEGQSWSYKRHYQEAFHNGHAETPLYRFSLSDGTPVTAQTRTDLCRNPSNNEPHS
FLSTHFLQRDQNNYRGNQGGAMRPQNMAVNNPNQQMSVGGAMGMNRGYNMADQGNVSQRA

MPSYTGGNRMNPMNPMQQMNPMGGPVAGNYGMGMTSPTQASPAMNAPPHNVLSSPRVRGS
PKMGASPFSPLSGLNSPMSSSHPGNAGGGSTFSSSSLNALQAISEGVGNPMPSPLTSPPP
QRPDSSPSINSTNVPTGPCKLPTYADSKSPGSSLGGGAEHPTQQHLHTPTGEVPPEKPDS
QASREVGVSGGESSRRVPDSKSHKKLLQLLTSPTDELVPPNHPPTSGPSSTPEAKDGPPT
VTSPSPSTGVSSSTGGNHSTVSSSGGAGQSSSQSLQEKHKILHKLLQNGNTPDEVARITA
EATGKCALDSAAQEPAAVGTRGTELKQEQLSPKKEKPNALLHYLLNKDDSKEGSDIKPKL
DELEARGVQAAGVTSSDPHCINGKVKMEPPDEAETLETILGVSRSNSAFYPEPDSRTGKE
VGHKPDSLHGVEFKSPFPYLIHISSHHLSVLRAAYQRALSLDSKPMGGEGPAAAGGLAGR
RNVACPTLVKQENVEAPIRFGSVPNGFPGGGGGMGRGMAVPQQRPPVSGPGDWGMTRPAS
TPVVRPGHPGMGRANPMAGPMISRSNSLPGNTRSMLQQKLMDMAANETSMSRFGGPGPPP
QSPSWQSSPLGVDGPQRNTSSRDQFGNPLDELLGPLTSSEGQSDERALLDQLDSLLNTAD
VGALQEIDRALGIPEIVGQQGHAQCPSAETFPGQDTPLSMDQKPMYNQGYPGPPGPPAVN
MQPGYGGSGMQGQPPGGFSPMMNQMGQTGSFAGMAGMSSPNANMMRPRMMTATKPLRLQL
QQRLQGQQFMNQTRQGVKMEHNPGGNPAMHPGVQPGMGAQPGFLNPQMVAQRNREMMTIS
QMRRQRMMMLMQQQGQGAAGGFNPPPNVTAPGGMDGPVGAPAMNQTGQQGFNYGANYGMS
QQGEPPFMAPGSSPPGNMMQGRMGGPQNSMMSGMQATPQGGSVYPSGDMKGWPQGNMPRS
SSYSQAQFPQQGNQNQFGNMMMNNSRQRPGQMGMNPMGMGRMPMGPDQKYC

>Gasterosteus aculeatus_NCOA3a(ENSGACP_13262)
MSGVGDNLLEPLCADRKRKLSTCDTPGLGCDKRRREQESKYIEELAELISANLSNIDSFN
VKPDKCAILKETVRQIRQIKEQGKSSCSDDDVQKADVSSTGQGVIDKDHLGPLLLQALDG
FLFVVNREGSIVFVSDNVTQYLQYKQEELINTSVYNIIHDEDREEFHKNLPKSNAPNGAS
WAGEAPRQKSHTFNCRMLVNFVHGPGHGLSEERPGGHRYETMQCFALTQPRAMMEEGEDL
QSCMICVARRIIAVERTERFSTRHELSGKLIEIEQQTSLSTTMRPGWEDLVRRCMQMFLH
RSEGQPWSYKRHYQDAFHNGHAETPLYRFSLGDGTPVTAQTRSDLCRNPNTNEPHSFLST
HLLQREQNSYRGNQVGSMRPQNMAVNNPNQPMNMGPGGGMTMNRGYGMAEHGNMPQRGVP
PYAGANRMNPMNHMNPMHQGQFHGGGGGPGGGGYGMGMTSPPLASPGINGPPHNVMGSPR
VRGSPKMGASPFSPGAGMNSPMGSGHPGNSGGGTTFSSSSLNALQAISEGVGNPMPSPLT
SPPAHKPDSSPSINSTNQAAGGPCKPSLPAYADSKSPGSSLAGGGEPQSQQNPRTPTSEG
PPDKPDSQAGREAGPAGGESNRRVADTKSHKKLLQLLISPTDELVPPNHPPGSTPEAKDG
TAGVTSPSSSTGVSSSTGAVSSSGGTGPLTSQSLQEKHKILHKLLQNGNTPDEVARITAE
ATGKSSLDPGAPEPGPAAAGAVRGSESKQEEQSPKKEKPHALLHYLLNKDDSKESADVKP
KQEELEGRGAQGAGVTSSDPHCMDGKVKMEPSDEAETLETILGVPRNNPGFYPEPDSRAG
KDAGNKQGNGPDNLRDGERGPVPPAQRAPYQRALSMDAKPPAGGEGPFPNGFPGGTAVGS
PRGHPTRGMGRGMGMPQRPPMSAPPDWGMPRSGGSPVAGQVHPGMGRSGPMGGPMINRSN
SVPGKTRSMLQQQLMDMGTNEANMGMSPFGGQGPPPRSPSWPDSAMGLDRPKAKSNRDQF
GHPLEELLGPRGHSEGPSDERALLDQLDSLLNTTDVIALQEIDRALGIPEIVGQGRCPPS
EPFTGSDSSIGMDQKPGYNQGYPGPPGPSSIGMQPGYGGNTMQGQSPGGFNPMMNQAGQA
AGFPGMAGMGGMGNPRANMMRPRMMSATKPLRLQLQQRLQGQQFMNQTRQGMKMEHAPGG
NPAMQPGMQPGMQPGMQPGFLNAQMIAQRSREMMTMQMRRQRMMMMRGFSPPPNVTAPGG
MDASIGGPPMNQPGQQGFSYGGNYAGMNQQGDPSFMAPVSSPPGNMMQTTMMPGMQGNPQ
GGPMYQSGDMKGWPQGGMPRGNTYPQQQFPQQGNQGQFGPMMMNKQGQMQGQLGMNPMAM
GRMPMGPDQKYC

>Gasterosteus aculeatus_NCOA3b(ENSGACP_20073)
CTLPPPLCRCERKRREQESKYIEELAELISANLGDIDSFNVKPDKCAILKETVRQIRQIK
EQGEWTRRAGGPREVKTEMTRAFRHVSCSLPVYPLLLYSLSVALSASLHVRASQSFAVFL
FSRVKQTVRGYNDERIMNECVFTCLCQLKDCIFKKVLPVCVFNSPPAASRARVFSRGASC
TCQKETSGQLKAPDKALRQPWRKVGCLSEVCPPFLALPGSLPLKGDPLPLCVTVCVPLPP
AMRKLGTRPVRRLDFSNALTGSHSHSLCKSMPPLLFNNSLLYSPKSLIQTSQQRLPPTRA
PRWAPTFLQGHAETPLYRFSLSDGTPVTAQTKSKLYRHPMSNEPQGFISTHLLQREPNGY
RSAQGGSMMPNTMRQQGMGGPNPNAQMNMNTGGGMGNVGRGFGMSEQGHLGHMGPMGGGS
IPPQHPPQHPQQHPQQHPLQHPFQSSGGFGLSGMNSPSGSPRMGGPQQGLLMSPRNRGSP
KMAANQFSPGVIFSPGMHSPMSGIVSGGGSGGGTTFSSSSLNALQAISEGVGSSLPSTLT
SPSPAHKPDSSPSVHSSSALSSQPAKPGQDGSKSPAGGLGPGPGQAESSGDRPDSQATTA
TKESGEGGNEAGVASSEPSRRLPDSKGHKKLLQLLTSPTEELGMGGSGSSGPPVPPSTTS
ISTPAGSDTKDPSGAMSSAHYTGSLQEKHKILHKLLQNGNTPDEVAKITAEATGKVTLGG
QEGGEAGSGGSGGGPGLVAEPKQEQQSPKKEKTHALLHYLLNKDDSKEPVDVKPKLEELE
GKAVTGSVRGGPGSAPEHPESKIKSEPPDDLHNLESILGDLRGSTSDFYPDQAGGGGAND
TGGKAQGCLDDSLRAGSPGMGPGPRGPFQRALSMDGKPPVGPAGPGRRPMLIKQENMMGS
PDGYPGNMGPMNRGMVPQRSPMGGSGDWGMSRSSGSPVGHPSMMRQGMEYGNSKGMMSGP

MVGRSNSVPGSRSMLQQQLMDMGTSRGSADMGMGMSPFSQQGQPNQSPSWPDTMMGMEGN
RRQFGNTLDDLLVPPTTSEGQSDERALLDQLDSLLNNTDGIALEEIDRALGIPDLVSQNQ
GSEQQMEPFPGQDQSMVLDQKPLYGQGYPGPPSMPLQSGYGGNPMQGQAQPGGFGPMLSQ
MGQGGSFPGMGGMGSMGHPRANMMRPRMMTANKPMRLQLQQRLQGQQFMNQTRQGMGMKM
ENPGGNPGMRPGMQPGMGGQPGFLNAQMMAQRSREMVTMQMRRQRMMMLMQQQQQAAAAA
AGGFSPPPNVTAPGGMENPMGGPNMGQPGPQQFGYGGNYGLAQQGDPSFGPSGGSPPGAM
IPGRLGPQNPMMQQHPQGGPMYQGADMKGWSQGGMARNSSYPQQQFGPQGNPGQYGGMMI
NGGMPASGGGGHMGQMGGQMGMNPMVMGRMQMGPDQKYC

>Oryzias latipes_NCOA3a(ENSORLP_15195)
MSGLGENSMEPLSSENRKRKLSTCDTPALGFVCSSERKRREQESKYIEELAELISANLSD
IDSFNVKPDKCAILKETVRQIRQIKEQGKSSSNDDDVQKSDVSSTGQGVIDKDHLGPLLL
QQALDGFLFVVNREGSIVFVSDNVTQYLQYKQEELINTNVYNILHEDDREEFHKNLPKSS
INGVSWGNEPVRQKSHTFTCRMLVKFGHAHGPMEEGPGGPRYETMQCFALTQPKAMIEEG
EDLQSCMICVARRVTAMERTESFNTRHELSGKLIQIDQNSLRASMRPGWEDLLRRCIQMF
LQHSDGQPWSHKRHYHEAFLQGHAETPLYRFSLSDGTPVTAQTKSKLYRHPMSNEPQGFI
STHLLQREPNGYRSAPGGGMMSNSMRQQGMGGPSPNTQMNVNPGGGMGMGGMGGMNRGFS
MNEQGHMAHMAPMGGGSMYGGSGGAGGGGGGMGNRVMQMNQNSGGFGLSGMNSPSGSPRI
GGPQQGFLMSPRNRGSPKMGANQFSPGAPGMHSPMGSIGSGGAGGGGTSTFSSSSLNALQ
AISEGVGSSLSSTLTSPPPAHKPDSSPSIHSSSSSSQPNQQGKPSQDGCKSPTGGLGPGD
HHLSTHHHHHQQHPAESSADRPDSQAATSTKESGEGSGEAGVTNSESQRRLPDSKGHKKL
LQLLTSPTEELGIGGSGTSGPPVPPPNSSRNDVGLDCKDASGGMTSPSSTGVSSSSAASS
QAAGQGSLQEKHKILHKLLQNGNTPDEVAKITAEATGKVSLGGQESSEADSKQEQHSPKK
EKTHALLHYLLNKDDSKEPVDVKPKLEELEGKGPPGPTGGPLPEHPESKIKSEPSDDVRF
FQLHNLESILGDLRGSSSDFYPDLAGGGVANRAGNKPQGCLDDGLQGSPVLGPGARGPFQ
RTLSMDGKPSIAPGGAGRRPMLIKQEKMMGSPDSYPGNLGPINRGVVPQRSPMGGSGDWG
MPCSNASAAGSAGHPSMMRPGMEYNSGKSMMPGPMVGRSNSVPGSRSMLQQQLMDMGKGG
YSKPKQRQQFQRSYISFGILRGSSDMGIGMSPFSQQGQPTPSPSWPDSMMSMEGNRLRRQ
FGNSLDDLLVPPTTSEGQSDERALLDQLDSLLNNTDGIALEEIDRALGIPDLVSQNQGPE
TLEPFPGQDQAMLLDQKPLYGQGYPGPPSMPMQSGYGGNPMQGQGQQGGFGPMLSQMGQG
GNFPGMAGMGGMGNPRANMMRPRMMSPNKPMRLQLQQRLQGQQFMSQTRQGMGMKMENPG
GNPGMRPVPTVQPGFLNAQMMAQRSREVVTMQMRRQRMMMFTGGFSPPPNVTAPGGMDSP
MGGPNMAQPGPQQFGYSSSYGMAQQGDPSFGPSGGSPPNAMMPGRLGPQNPMMQQHSQGG
PMYQGPDMKAWSQGGVGRNSASYPQQQFGQQGNPAQYGGMMMNGAMPSSGGGGHMGQMGG
QMGMNPMVMGRMPMGPDQVC

>Oryzias latipes_NCOA3b(ENSORLP_17196)
MSGIGDNSLEPLRSDRKRKLSTCDTPGLGPAKSMMCDKRLREQESKYIEELAELISANLS
NIDSFNVKPDKCAILKETVRQIRQIKEQGPSSCSDDDVQKADVSSTGQGVIDKDHLGPLL
LQALDGFLFVVNREGSIVFVSDNVTQYLQYKQEELINTSVYNIIHDDNREEFHKNLPKPN
GPNGQWGGEAPRQKSHTFNCRMLVNLAHGASHGLSEDRPGGQRYETMQCFALTQPRAMME
EGDDLQSCMICVARRITAVERTERFTTRHELSGKLIEIEQQSSLHNTMRPGWEDLVRRCM
QMFLNRSDGQPWSYKRHYQDAFHTGRAETPLYRFSLSDGTLVTAQTRTDLCRNPSTNEPH
SFLSTHFLQSNMPQRGMASYSGGGRMNQMNHMNPMHQMNNMGQMNPMHPMNHMGSMNQMG
HHGMHQQHTHTQMGQMHGGPGGGGFGMGMTSPPQSSPGINGPPHNVLGSPRVRGSPKTGA
SPFSPGGMHSPMSSTHTGGSGGGGGTTFSSSSLNALQAISEGLGNPMPSPLTSPLSHKPD
SSPSINSTNQETGGKSALPPYSESKSPGSSLRAGGEQQSQQHPQTPTSEGPPDKPDSQAG
LGGGEANRRVPDKCNKKLLQLLTSPTDDLVPSNHPQSSGSSSIPDSKDGSGGHGAVSSSG
SSGHFPSQSLQEKHKILHKLLKDGNTPDEVAQITLLATGKSSLDSAAPEAGLSSTGGEKG
SETKKEQHSPKKEKSHALLHYLLNKDDSKEGGDMKPKLEDLDGRGAPGAGVTSSDPHGMD
GKVKLEPSDEAETLETILGVRQNNSSFYPELDSRATKEVANKQGAVSESLHENIQGGATG
SPPRSILIHPLLRRERGPMLPAQRAAYQRALSVDSKPMGGDMPVGGGLASRRNVPCPTLV
KQESMEAPVRPGTMPNGFPGGMGVCPPRGNPTRGIGRGMGAHQRPPMAGPGEWGMPRSGG
SAAAGPGHPVMGRPGQMGGPMISRSISAPENMRSMLQQQLMDMGANESNVSMSPFGGQRP
PPQSPSWPDSAMGMDRPQTSTNREQFPHPLEELLGPLPSSEGPGDDRALLDQLDSLLNNA
DVITLHEIDKALGIPEIVGQAHGPEAEAFSAMDSSAGMDQKAMYSQGYPGPPTMGMQSAY
AGNTGPPPGGFNPMMNQMGPTGAFPGMGPMGNPRANMTRPRMMTATKPLRLQLQQRLQGQ
QQGIQPGIQPGMAPAGMNVQLKDYICGQPGFVNAQMLAQRQREAMTTMQMRRQRMMMLMQ
QQQGQAAAGGFSPPPNVTAPAGMENPIGSPPITQPGQQGFNYGGSYGQLTESALHSFVKL
SFGVLTVAVVFAGMNQQGESFIPPGSSPPGHMMQGRMGGPQQNTMMPGMQGNPQGGHVYS
SGDMKGWLQGNMPRNNLYSQQQFPQQGNQGQFGSMMMNTSMGGTGPGSGSSVGQMTQMPG

QMQGQMGMNPMGMGRMPMGPDQQKYC

>Saccoglossus kowalevskii_(XP_006811320)
MNNPQVSKLLNKTDSDRTQSNKSLNERRRREQENIYIEELAELISASITNMDSCNVKPDKCAILQETVKQ
IKTIKRQQVAESNGAVQQCHVSSSKPALLANDVLGPLLLEALDGFLFVVNSQGRIEFVTENVSTYLKFGK
DELVGSSIYSLIHAGDHAQLTECLLPLTNGIVWPSENPNKLSKSRTFNCRMYIGSQDEHGDDGGSQESKL
EHMQCSAVLQPHPDDKQPNADGSASVDGPAYLVCVARRMPQEDRSLIEQFVTKQDLSGKIISADISSLTL
PFHSKSDLVGHSLQEYCHSSDMQQLIKHHQEVLSNITDTCTTAMYKFRATDHCWLFVQTRSKLFLRNGQP
EFIMSTHSIYRETPNTPSTDASTSASTNQVSSIPSTPKSDHSGPLPSLSNLLRSGNSASASSNSPIEMLQ
NSSLSSLLKSTNLKPYMSHLQTKLPTTRINTSSDVMRPPVPGSSYSRSETSYKTSSPLCCSSQSPVSAST
SGLVSPDSVQGRVEKPASTSSLKRPSQDSAQGGKFKKNRLLQKLLMEDDDTDYAAQGAKACGEAVEFTSN
PPSNSSTQSEASVRMKEAILQRLLEQSPNPLSVESKDMDADKKDFSDSEDENKILSKLLDGDCSVKFGSE
LYPLEQLRRMQNHSGGDLFPRIPSQDTSKILDHHRTLIPDACPPGKQSKNGAPQKQPKNVLLQKLLMEDD
SSVLSHSTLFGKTFGAGFRKHEIVPNVNESDSIVSSSSSSSSSSNSNNNSNNVALASAVTSGENTASTA
KTPVTSQQQQNQLQKPEQQQTQSPQTSALPALENFNIYNLDEEETNTLIQQLFHVANSLPEEALTGTQNS
QSVVQELLAGCDGNQVTQMETSSLQKPMVPIDNNNKQNSNPENPLLQQLLMEGSASDQLKLQQQQSVPPD
STVQSPPFTPIPNTAPIGFGALLNRQSNQQLRRVSTWGQQPSQLGRSSGGIQRRNTIGSVHSISNQGLSA
TGMTNPMPFSHPLQSTQKSSIQVMTNLNNLSGNTSQQSPSSIMSDDSSASLSDDAILQQLEQVLNNSNLS
LCEIDNALGIEQLLSTPHSDEQLTSDLLNSALSGGASGRNTPSSSSVNSMNTIATSSMNTLANSMGGSIA
SNTITNTYPHSTITDSLSSNSGLLNTGNLGLNTNFMDSTMRQQSQQPVYSMQQDLPDIINPRFSQGHSMN
SGPRFPGSGQMPQSFQQQQVQARLRHQQQLQTVRQQRSHLRQTLLMRQQQQRLQQQQQQQQQQQQHHELQ
QQKLQQMLQQQIMMQQQHNPLLQQQQKHLVMLQQQQQQQSFIGSQINHQQLIETQETNFPPDTLADMLAQ
TSNITAPNVTIQKSPGAFKPQMSPHSARPPTPGTAVGPRLSQAASRPLQSGTRNSTGLDFSLSPSFSQRP
PQQTPPVSHFKPSQNIFQFPDLNTTAPPNNTVTSSMSQNNFHMQQTNSSGSNSITTNPMTPQTSATGTNS
IFTNTIMHLTGISSSSTITANLVLQQGCSAASSGSAQLNNATRPSTTMVNSMISQTSSTVASSAVANQII
AMSPQTANQMMSPSRMLQFTQSPANTIASSNRQSPAWGRQMSEPGSLLLAQLNAKSSPVDTQEIMGSASQ
LQRSLSTDQQQLRPSSREVHSSHLLSASSQGTSATSSPLASITSPPSVLNDTPSSMSETLTPKKEMDKSL
KASPCSRPSNNSEQFNVAVDIVDQGNVIASSTTMYHHPYPSFDETEDNESMMLDFPFDDLTGDDSKPAAV
ENTEPPDSEDSARNDIQEDELADRLTMVMENHTREEAHKRFQKFCALANVKPPSDPSMKAATLFRNQLLN
PDTKAPPLEISTDDSKPIVQPEKKGKSSLLQKLLLE

>Ptychodera flava_(BAC06837)
MALIAIFTLTLVSLQQLSTDAMPFSHLKIPCHIMPGSEITRLNHKGQQYSLHEESDPEVASLFGVTEDGI
IRSMEELTDYANRMFSVVVEQFLDDDAWKDSFNIYITKSGDNSEHLKFTQDRYEGSVLENSPAGTVVYGF
ESISACSECDLVEYSLLGTGEEKFNLENKDGIVTIQTNEVLDRESRASYNLTLVAASASLETETTDLLIT
VEDVNDNAPEFDPETCEVTVPEISPADAIVAYVHAVDPDAGKNGKVMYYTDTNDMTFSVHPDTGGVIIGG
PLQAKEYTLTVFARDRGKPSLEASTPGRVIITVTPVDQSLKFSSILGPGPVILQDDDEELPEIKVSLMED
APKGSVVTTIKAPSNDKSSQYRFLMNNPMNPRFHVQYYTGDIVLTGRLDIDAHPTEEINVGITKDFNDPL
REAPKEQKVIITIIPSETDKPAPPNQVVKPRIRRASKPSFDQPSYEGTVEEVDGLASPVPAFVPREILTV
RANGDPSNIQYRIQQAEFREDFAIGLTSGIISSRKHLDREDNKGQVREFTVTASNSAGTTSVEVLIKIKD
VNDNAPQFPYPPYIGSFPENGAPGVTVMTIQAEDLDDPNEGGNALVRYSILRNAQNSVGQAIFAIDSESA
VITTQVGNLDREEIPSYDIVVKAVDGGAHTGTVTATIYVSDENDNPPEFPQAKSFVEISENVPVDSSVFK
VIAIDRDVGINSYSIISGNVGEKFRIETDPDSGYAIVKVAKKIDFELNESKFVLTIEVDDSKNTATTELD
IRVMDANDEVPSFSQPRYLAEHPEDFAKWTTIGTVAATDRDKSEKFSEIEFEVVRVPEFPDSSFFTVGSA
NGSVMAIKDLDREPYEDPDQIRENYIFTVTATDNVRGQSGERRNTATVTFTVSITDVNDNAPEFLPGYTL
LVPENTPAGYEAGVVEIIDRDDDSNGPPFTHRVIDDDGILSFFDVSSPIGVEDQLRVVSLEEFDREATPF
YYIPVVLSDKHGVSATQTLTVTIGDVNDNPPSPATKTILVYSYKGQIPRSAIGEVYAADEDIVKGDDISY
TVTTPIPQFSVDKDTGQIIIEENTKAGTYDFKVEVDEKPWPPVICTVIVEVKDIPEEAVFSSGSVRLLTS
AEKFIRGTPSMKDRFHDILVDVLGAKKENIDIFSVINVPGEENQCDVRWSAHGSPYYRPEKMNTQLLLAK
DVIESQLGVSFGMVPIDMCLDEGICESSCTNYFNAITDPTIIDDGADTFVSVTTIQRAECVCGAKLAPRG
PCASNPCLNGGQCIDTPSGYVCQCDEKYGGPNCEDISRGFQSNSFAWYPPLSQCEETKTSIEFLTKSRDG
IILYNGPIVPIDLGQPQDFMAIELVGGKPKLYMDLGSGMLPLTIDSPPLNDEKWHTLEIIRKSKHVEFVL
DHCENAVITETERTSVSDESNCKATDTTPGTNRFLNVNTPLQLGGIDMDSTYTYPNTFNFAPGGDFVGCI
RNVIQDGKVYDLGTPGREKNSEQGCPQTDLLCYVDGVYVCKNGLCVTESASVYTCVCDPGWGGEACDEEL
PSYDFLSDSYVIYDLEASLVSDKYMSDYQIMVGNTQEEDALLMHLQNVDGEEYITMKMKEGNAFIEYNLG
DPGMKYSLRVHNYTINDGNYHTVRLLRYGNIFRFEVDGGGGVRTVEKAQGIFKEFIPDPKSVIVGANVSL
SEMRVLGDDYSGCAKDARISNSYIGFDGEIDGTIAVPNRVEKDCNICLLSPCIAPLVCVPTPSFYYCSCP
GDLVFRGGACVKEGGPAPQTGAAGLSLAALIAILLCIFILLVLILAFIVYKRHAQKDKGALVLDVDEDDL
HENVVVYDDEGGGEEDYDAYDINTLRKPVDPALESPVIPKKKQPVSETPRTRPNLPPGAELPDIGDFLDK
RQNDADDDPEAPPYDTLRVYNFEGEGSTAGSLSSLNTSGSGESEQNFDYLKDLGPPFKKLADMYGGEED

Na+/K+ Transporting ATPase Interacting Protein-NKAIN

>Homo sapiens_NKAIN1(ENSP_362841)
MGKCSGRCTLVAFCCLQLVAALERQIFDFLGYQWAPILANFLHIMAVILGIFGTVQYRSR
YLILYAAWLVLWVGWNAFIICFYLEVGQLSQDRDFIMTFNTSLHRSWWMENGPGCLVTPV
LNSRLALEDHHVISVTGCLLDYPYIEALSSALQIFLALFGFVFACYVSKVFLEEEDSFDF
IGGFDSYGYQAPQKTSHLQLQPLYTSG

>Pan troglodytes_NKAIN1(ENSPTRP_793)
MAVILGIFGTVQYRSRYLILYAAWLVLWVGWNAFIICFYLEVGQLSQDRDFIMTFNTSLH
RSWWMENGPGCLVTPVLNSRLALEDHHVISVTGCLLDYPYIEALSSALQIFLALFGFVFA
CYVSKVFLEEEDSFDFIGGFDSYGYQAPQKTSHLQLQPLYTSG

>Gorilla gorilla_NKAIN1(ENSGGOP_19669)
MGKCSGRCTLVAFCCLQLVAALERQIFDFLGYQWAPILANFLHIMAVILGIFGTVQYRSR
YLILYAAWLVLWVGWNAFIICFYLEVGQLSQDRDFIMTFNTSLHRSWWMENGPGCLVTPV
LNSRLALEDHHVISVTGCLLDYPYIEALSSALQIFLALFGFVFACYVSKVFLEEEDSFDF
IGGFDSYGYQAPQKTSHLQLQPLYTSG

>Mus musculus_NKAIN1(ENSMUSP_144007)
MGKCSGRCTLVAFCCLQLVAALQRQIFDFLGYQWAPILANFLHIMAVILGIFGTVQYRSR
YLILYAAWLVLWVGWNAFIICFYLEVGQLSQDRDFIMTFNTSLHRSWWMENGPGCLVTPV
LNSRLALEDHHVISVTGCLLDYPYIEALSSALQIFLALFGFVFACYVSKVFLEEEDSFDF
IGGFDSYGYQAPQKTSHLQLQPLYTSG

>Canis familiaris_NKAIN1(ENSCAFP_16446)
MAVILGIFGTVQYRSRYLILYAAWLVLWVGWNAFIICFYLEVGQLSQDRDFIMTFNTSLH
RSWWMENGPGCLVTPVLNSRLALEDHHVISVTGCLLDYPYIEALSSALQIFLALFGFVFA
CYVSKVFLEEEDSFDFIGGFDSYGYQAPQKTSHLQLQPLYTSG

>Monodelphis domestica_NKAIN1(ENSMODP_21301)
MSKCNGRCTLVGFCCLQLVAALERQVFDFLGYQWAPILANFLHIMAVILGIFGTVQYRSR
YLIMYAGWLVLWVGWNAFIICFYLEVGHLSQDRDFIMTFNTSLHRSWWMENGPGCLVTPV
LNSRLALEDHHVITVTGCLLDYPYIEVLSSALQIFLALFGFVYACYVSKVFLEEEDSFDF
IGGFDSYGYQAPQKTSHLQLQPLY

>Xenopus tropicalis_NKAIN1(ENSXETP_28231)
MGRCSGRCTLVGICCLQLAAALQRQIFDFLGYQWAPILANFLHIMVVILGILGTLHYRSR
YLILYSIWLALWVAWNAFIICFYLEVGHFSQHRDLIMNFNTSMHRSWWMENGPGCLVTPV
RGPPLPLADHHMVTVTGCLLDYPYIEALSSALQIFLALFGFVYACYVSKVFMDEEDSFDF
IGSYDSYGYQAPMKTSHLQLQPLYKPG

>Takifugu rubripes_NKAIN1(ENSTRUP_33429)
MGKCDGRCTLLVICSLQLVAALQRQVFDFLGYQWAPILANFLHIMAVILGMFGTVQFRFR
YLIFYAVWLVVWVGWNAFTICFYLEVGNLSQERDFLMTFNTSLHRSWWMEHGPGCLVTPV
RDSLIAPDGHHVITVSSCLLDYQYIEVLSSAVQILLALFGFVYACYVSKVFQDDEDSFDF
IGGFDSYGYQPPQKSSHLQLQPLYTAG

>Tetraodon nigroviridis_NKAIN1(ENSTNIP_20105)
MGRCDGRCTLLVICSLQLVAALQRQVFDFLGYQWAPILANFLHIMAVILGMFGTVQFRFR
YLIFYAVWLVLWVGWNSFIICFYLEVGNLSQDRDFLMTFNTSLHRSWWMEHGPGCLVTPV
VDSLIAPDDHHVITVSGCLLDYQYIEVLSSAIQILLALFGFVYACYVSKVFQDDEDSFDF
IGGFDSYGYQPPQKSSHLQLQPLYTAG

>Gasterosteus aculeatus_NKAIN1(ENSGACP_7528)
MGKCDGRCTLLVICSLQLVAALQRQVFDFLGYQWAPILANFLHIMAVILGMFGTVQFRFR
YLIFYAVWLVLWVGWNSFIICFYLEVGNLSQDRDFLMTFNTSLHRSWWMEHGPGCLVTAV
LDSRMAPDDHHVITVSGCLLDYQYIEVLSSAIQVLLALFGFVYACYVSKVFQDDEDSFDF
IGGFDSYGYQPPQKSSHLQLQPLYTAG

>Homo sapiens_NKAIN3(ENSP_429073)

MGCCTGRCSLICLCALQLVSALERQIFDFLGFQWAPILGNFLHIIVVILGLFGTIQYRPR
YIMVYTVWTALWVTWNVFIICFYLEVGGLSKDTDLMTFNISVHRSWWREHGPGCVRRVLP
PSAHGMMDDYTYVSVTGCIVDFQYLEVIHSAVQILLSLVGFVYACYVISISMEEEDTYSC
DLQVCKHLFIQMLQIIE

>Pan troglodytes_NKAIN3(ENSPTRP_34735)
MGCCTGRCSLICLCALQLVSALERQIFDFLGFQWAPILGNFLHIIVVILGLFGTIQYRPR
YIMVYTVWTALWVTWNVFIICFYLEVGGLSKDTDLMTFNISVHRSWWREHGPGCVRRVLP
PSAHGMMDDYTYVSVTGCIVDFQYLEVIHSAVQILLSLVGFVYACYVISISMEEEDTYSC
DLQVCKHLFIQMLQIIE

>Gorilla gorilla_NKAIN3(ENSGGOP_28708)
VSALERQIFDFLGFQWAPILGNFLHIIVVILGLFGTIQYRPRYIMVYTVWTALWVTWNVF
IICFYLEVGGLSKDTDLMTFNISVHRSWWREHGPGCVRRVLPPSAHGMMDDYTYVSVTGC
IVDFQYLEVIHSAVQILLSLVGFVYACYVISISMEEEDTYSCDLQVCKHLFIQMLQIIE

>Mus musculus_NKAIN3(ENSMUSP_113113)
MGCCTGRCSLVCLCALQLLSALERQIFDFLGFQWAPILGNFLHIIVVILGLFGTIQYRPR
YIMVYTVWTALWVTWNVFIICFYLEVGGLSKDTDLMTFNISVHRSWWREHGPGCVRRVLP
PSAHGMMDDYTYVSVTGCVVDFQYLEVIHSAVQILLSLVGFVYACYVISISMEEEDTCRN
K

>Rattus norvegicus_NKAIN3(ENSRNOP_17479)
MGCCTGRCSLVCICICMYLSALERQIFDFLGFQWAPILGNFLHIIVVILGLFGTIQYRPR
YIMVYTVWTALWVTWNVFIICFYLEVGGLSKDTDLMTFNISVHRSWWREHGPGCVRRVLP
PSAHDMMDDYTYVSVTGCVVDFQYLEVIHSAVQILLSLVGFVYACYVISISMEEEDTCK

>Equus caballus_NKAIN3(ENSECAP_18083)
LSALERQIFDFLGFQWAPILGNFLHIIVVILGLFGTIQYRPRYIVVYTVWTALWVTWNVF
IICFYLEVGGLSKDTDLMTFNISVHRSWWREHGPGCVRRVLPPSAHGMMDDYTYVSVTGC
IVDFQYLEVIHSAVQILLSLVGFVYACYVISIFMEEEDT

>Monodelphis domestica_NKAIN3(ENSMODP_10310)
ISALERQIFDFLGFQWAPILGNFLHIIVVILGLFGTIQFRPRYIVVYTVWTALWVTWNVF
IICFYLEVGGLSKDTDLMTFNISVHRSWWREHGPGCVRRVLPPSAHGMMDDYTYVSVTGC
VIEFQYLEVIHSAIQILLSLIGFVYACYVISVFMEEEDS

>Taeniopygia guttata_NKAIN3(ENSTGUP_11578)
LAALERQIFDFLGFQWAPILGNFLQIIVVILGLFGTIQFRPRYIVVYTVWTALWVTWNVF
IICFYLEVGGLSKETDLMTFNISMHRSWWREHGPGCIRRVVRPSSPGILEDYSYVSVTGC
IIDFQYLEVIHSAIQILLSLIGFVYACYVISIFMEEEDS

>Anolis carolinensis_NKAIN3(ENSACAP_23034)
MGCCTGRCTLVFLCTLQLLAALERQIFDFLGFQWAPILGNFLHIIVVILGLFGTLQYRPR
YIVVYSVWTALWVTWNVFIICFYLEVGGLSKETDLMTFNISMHRSWWREHGPGCIRRVVR
PSAPGILEDFSYVSVTGCIMDFQYLEVIHSAIQILLSLVGFVYACYVISVFMEEEDSCKY

>Xenopus tropicalis_NKAIN3(ENSXETP_46789)
ARCTFSSCCLIYICVIQLVALERQIFDFLGYQWAPILGNFLHIIVVILGLFGTIQYRPRY
IVAYTIWTAFWVAWNVFIICFYLEVGGLSKDTDLMTFNISIHRSWWREHGPGCVWRLVPA
PPSKNLGDHSFISVTGCIIEFQYIEVIHSAVQILLSLIGFVYACYVISVITDEEDSCRHK

>Homo sapiens_NKAIN4(ENSP_359340)
MGSCSGRCALVVLCAFQLVAALERQVFDFLGYQWAPILANFVHIIIVILGLFGTIQYRLR
YVMVYTLWAAVWVTWNVFIICFYLEVGGLLKDSELLTFSLSRHRSWWRERWPGCLHEEVP
AVGLGAPHGQALVSGAGCALEPSYVEALHSCLQILIALLGFVCGCQVVSVFTEEEDSFDF
IGGFDPFPLYHVNEKPSSLLSKQVYLPA

>Pongo abelii_NKAIN4(ENSPPYP_12544)
MGSCSGRCALIVLCAFQLVAALERQVFDFLGYQWAPILANFVHIIVVILGLFGTIQYRPR
YVMVYTVWAAVWVTWNIFIICFYLEVGGLLQDSELLTFSLSRHRSWWREHWPGCLHEEVP

AVGLGAPHGQALVSGAGCALEPSYVEALHSGLQILIALLGFVCEEESFDFIGGFDPFPLY
HVNEKPPSLLSKQAYLPA

>Macaca mulatta_NKAIN4(ENSMMUP_12099)
MGSCSGRCALIVLCTFQLVTALERQVFDFLGYQWVPILANFAHIVVIILGLFGTIQFRPR
YVVVYTVWTAIWVTWNIFIICFYLEVGGLSQDSELLTFSLSQHRSWWHEHWPGCLHEEVP
AAGLGAPHGQALVSGAICALEPSSVEALHSGLQILIALLGFVCGCYVVSVFTEEEDSFDF
IGGFDPFPLYHVNEKPSSLLSKQAYLPA

>Mus musculus_NKAIN4(ENSMUSP_99342)
MGFCSGRCTLLALCALQLVTALERQVFDFLGYQWAPILANFTHIIVVILGLFGTIQYRPR
YIVVYVVWAAVWVTWNVFIICFYLEVGGLSKDSELLTFNLSGHRSWWEEHGPGCLHEEEA
TAGLGALHGQSLVVGAGCAMVHSYVEALHSGLQILLALLGFVYGCYVVSVLTEEEDSFDF
IGGFDPFPLYHVNEKPSSLLSKQAYLPA

>Rattus norvegicus_NKAIN4(ENSRNOP_75472)
ACRELSDSPLTPGSPTVSIYAVFLRLLKVTQLGFFHTLVTVLERQVFDFLGYQWAPILAN
FAHIVVVILGLFGTIQYRPRYIVVYVVWAAVWVTWNIFIICFYLEVGGLSKDSELLTFSL
SRHRSWWEEYGPDCLHEKAATAGLGALHGQSVVVGPGCGMVHSYVEALHSGLQILLALLG
FVYGCYVVSVLTEEDDSFDFIGGFDPFPLYHVNEKPSGLLSKQAYLPA

>Canis familiaris_NKAIN4(ENSCAFP_18827)
MGCCSGRCALLFICSFQLVTALERQVFDFLGYQWAPILATFVHIVMVILGLLGTIQYRPR
YVVVYAVWEAVWVTWNVFIICFYLEVGGLSKDSKLLTFNLSPHRSWWREHGPGCLHDEPE
AGLGPLGGQALVAGVGCALEHGYTEALHSALQILLALVGFVCACCVVSVFTEEEDSFDFI
GGFDPFPRYHVSEKPSNLLLKQVYVPA

>Loxodonta africana_NKAIN4(ENSLAFP_11277)
VRETPWRCAAFTRLLLPQVTALERQVIDFLGYQWAPILANFLHIVLVILGLFGTVQYRPR
YVVTYAVWAPIWVTWNVFIICFYLEVGGLSKDSELLTFHFSQHRSWWREHGLGCVHEEAL
ATSLRPLDGQALVSGSGCTLEYHYVEALHSVLQALVALVGFVYACYVVSALTEEKDSFDF
IGGFDPFPLYHVNEKLSHLLFKQAFMPG

>Gallus gallus_NKAIN4(ENSGALP_33347)
MGCCTGRCTLIFLCTLQLLAALERQVFDFLGYQWAPILANFLHIIIVILGLFGTIQYRPR
YIVVYAIWTAVWVTWNIFIICFYLEVGGLSKDSELLTFNISRHQSWWSENGPGCVRKEAS
MSGIAGLDSHSYISVIGCALEYRYIEVMHSSFQILIALVGFVYACYVVSVFTEEEDSFDF
IGGFDPFPLYHVNEKPTNLLFKQTYLPA

>Anolis carolinensis_NKAIN4(ENSACAP_20361)
MGCCTGRCTLILLCTLQLLAALERQVFDFLGYQWAPILANFLHIIIVILGLFGTIQYRPR
YIVVYAIWTAIWVTWNIFIICFYLEAGGLSKDSELLTFNISRHQSWWSENGPGCVRKETP
MVGLKGLDSHSYVSVIGCALEYRYIEVMHSALQILVALVGFVYACYVVSVFTEEEDSFDF
IGGFDPFPLYHVNEKPSNLLFKQTYLPA

>Takifugu rubripes_NKAIN4(ENSTRUP_11648)
MGCCSGRCTLIFICTLQLVLALERQVFDFLGYQWAPILANFFHIIIVILGLFGTIQYRPR
YIVVYTVWAALWVAWNVFIICFYLDVGGLSKDSDLLTFNISAHHSWWGEHGPGCVRREMH
QAPGVRTTDSQSYITVMGCLLGYQYIEVAHSGAQILVALLGFVYACYVVSAITEEEDSFD
FIGGFDPFPLYHINEKSSDLLLKPMYPPG

>Gasterosteus aculeatus_NKAIN4(ENSGACP_3416)
MGCCSGRCTLIFICTLQLVLALERQVFDFLGYQWAPILANFFHIIAVILGLFGTIQYRPR
YIVAYTVWAALWVAWNVFIICFYLDVGGLSKDSDLLTFNISAHHSWWSEHGPGCVRGEMP
QAAGGRTTESHSYITVMGCFMDYQYIEVLHSAAQILVALLGFVYGCYVVSAITEEEDSFD
FIGGFEPFPLYHVNEKPSHLLLRPV

>Oryzias latipes_NKAIN4(ENSORLP_12189)
VVLSSFRRSACLERQVFDFLGYQWAPILANFFHIIVVILGLFGTIQYRPRYIIVYIVWAA
VWVAWNVFVICFYLDVGGLTKDSDLLTFNISAHHSWWSEHGPGCIRREMPEAAGVRSTES
HSYITVMGCHMEYQYIEVLHSSTQILVALLGFVYACYVVSAMTEEEDSFDFIGGFEPFPL

YHVNEKSSHLLLKPMYL

>Homo sapiens_NKAIN2(ENSP_357402)
MGYCSGRCTLIFICGMQLVCVLERQIFDFLGYQWAPILANFVHIIIVILGLFGTIQYRPR
YITGYAVWLVLWVTWNVFVICFYLEAGDLSKETDLILTFNISMHRSWWMENGPGCTVTSV
TPAPDWAPEDHRYITVSGCLLEYQYIEVAHSSLQIVLALAGFIYACYVVKCITEEEDSFD
FIGGFDSYGYQGPQKTSHLQLQPMYMSK

>Pan troglodytes_NKAIN2(ENSPTRP_31701)
MGYCSGRCTLIFICGMQLVCVLERQIFDFLGYQWAPILANFVHIIIVILGLFGTIQYRPR
YITGYAVWLVLWVTWNVFVICFYLEAGDLSKETDLILTFNISMHRSWWMENGPGCTVTSV
TPAPDWAPEDHRYITVSGCLLEYQYIEVAHSSLQIVLALAGFIYACYVVKCITEEEDSFD
FIGGFDSYGYQGPQKTSHLQLQPMYMSK

>Macaca mulatta_NKAIN2(ENSMMUP_17512)
MGYCSGRGTLIFICGMQLVCVLERQIFDFLGYQWAPILANFVHIIIVILGLFGTIQYRPR
YITGYAVWLVLWVTWNVFVICFYLEAGDLSKETDLILTFNISMHRSWWMENGPGCTVTSV
TPAPDWAPEDHRYITVSGCLLEYQYIEVAHSSLQIVLALAGFIYACYVVKCITEEEDSFD
FIGGFDSYGYQGPQKTSHLQLQPMYMSK

>Mus musculus_NKAIN2(ENSMUSP_140463)
MGYCSGRCTLIFICGMQLVCVLERQIFDFLGYQWAPILANFVHIIIVILGLFGTIQYRPR
YVTGYAVWLVLWVTWNVFVICFYLEAGDLSKETDLILTFNISMHRSWWMENGPGCMVTSV
TPAPDWAPEDHRYITVSGCFLDYQYIEVAHSSLQIVLALAGFIYACYVVRCITEEEDSFD
FIGGFDSYGYQGPQKTSHLQLQPMYMSK

>Felis catus_NKAIN2(ENSFCAP_823)
VCVLERQIFDFLGYQWAPILANFVHIIIVILGLFGTIQYRPRYITGYAVWLVLWVTWNVF
VICFYLEAGDLSKYTLLILTFNISMHRSWWMENGPGCTVTSVTPAPDWAPEDHRYITVSG
CFLEYQYIEVAHSSLQIVLALAGFIYACYVVKCITEEEDSFDFIGGFDSYGYQGPQKTSH
LQLQPMYM

>Danio rerio_NKAIN2(ENSDARP_112659)
MGCCSGRCTLAFICGMQLISILERQIFDFLGYQWAPILTNFIHIITVILGLFGTVQFRPR
YVTGYAVWLVLWVAWNVFIICFYLEVGDLSKDTDLIMTFNISMHRSWWMENGPGCRVTPV
TPPPSWAPEDHRYISIAGCLLEYQFVEVAHSSLQIILA

>Takifugu rubripes_NKAIN2(ENSTRUP_37282)
TDRCLVDLQCIKLVSVLERQVIDFLGYQWAPILTNFLHIIVVILGLFGTIQFRPRYVTGY
AAWLVMWMTWNVFIICFYLEVGDLSRDSDLVLTFNLSMHRSWWMENGPGCRVTPISPPPS
WAPEDHRYISISGCLMDFQFIEVAHSSLQILLALMGFIYACYVVKLISEEEDSFDFIGGF
DSYGYQGPQKTSHLQLQPMYIPC

>Tetraodon nigroviridis_NKAIN2(ENSTNIP_13874)
LGNGAGRPFTPSVACMWGVSVLERQVIDFLGYQWAPILTNFLHIIVVILGLFGTIQFRPR
YVTGYAAWLVMWMTWNVFIICFYLEVGDLSRDSDLVLTFNLSMHRSWWMENGPGCRVTPV
TPPPSWAPEDHRYISISGCLMDFQFIEVAHSSLQILLALMGFIYACYVVKLISEEEDQLR
VDFIGGFDSYGYQGPQKTSHLQLQPMYMKG

>Gasterosteus aculeatus_NKAIN2(ENSGACP_17511)
FSFLELLLSGCSLPEVAVVSVLERQVVDFLGYQWAPLLTNFLHIIVVILGLFGTIQFRPR
YAAWLVVWMTWNVFIICFYLEVGDLSRDSDLVLTFNLSMHRSWWMENGPGCRVTPVTPPP
SWAPEDHRYISISGCLMDFQFIEVAHSSLQILLALMGFIYACYVVKLISEEEDSFDFIGG
FDSYGYQGPQKTSHLQLQPMYM

>Oryzias latipes_NKAIN2(ENSORLP_21348)
VNYCGGLCKCLGPFLSSMYKVSVLERQVIDFLGYQWAPILTNFFHIIIVILGLFGTIQFR
PRYVTGYAAWLVVWMTWNVFIICFYLEVGDLSRNSDLVLTFNLSMHRSWWMENGPGCRVT
PISPPPSWAPADHRYISISDCLMDFQFIEVAHSSLQILLALAGFIYACYVVKLISEEEDS
FDFIGGFDSYGYQGPQKTSHLQLQPMYIKK

>Branchiostoma floridae_(XP_002603157)
MSCCSGRCTLIALCFLQLIATVERQVFDFLGYMWAPIIGNFFQIICVILGLFGTYQYRPRYIVVYCTWGV
LWVAWNIFIICVYLELGSLTVETPALTLGAPQSISWWREHGIGCQASNATATSTNPVVTGCLLDFTYVEM
IHAAIQIVLAIVGFIYGIYVVYMFTEEDDSFDFIGGFDSYSAYQAPQKTSHMQLQPMYV

>Capitella teleta_(ELT97184)
MKEYCCHGNRLTLTLGVNVQQIATVERQVFDFLGYMWAPIIGNFLQIIFIIVGFFGALQYRPKIVIVYAV
WSLLWIGWNIFVICFYFEVGLLKRDSPILNIDTGNRSWWFDNGIGCQTPNKTQLDTGLDYVEGCLLQYYY
VEIIHAAIQCLFSLLGFCVGCYVVYVFSEEDDSFDFIGGFDTFSSAFQSPAKSSHVQLQPVYVATGAR

>Octopus bimaculoides_(XP_014782962)
MGSGRCSLSPTNTAIMVIFVLQLLSTVERLIFDFLGYMWAPIMGNFLQIICVIIGIFGVCQYRPCLVAVY
SIWSLLWIGWNIFVICLYLEIGILSRTRELYILNIGTKNKSWWLEHGIGCQVTNNTWEKNYHGETSRPIP
PEEFVEGCLVEYYYVEFIHAAVQCLFSFFGFIISCITIYMYTEEDESFDFIGGFDSYTSYQSQGKGSHMQ
LQPLYSTYLEQLLSLWIKPSNFYLAYQCE

>Trichoplax adhaerens_(XP_002117095)
MACGTLRTWTMAILILQLISLLERITFDFMGAVWALIIGNFVAILITVVGTFGAYQFRPRYLIMYLIWSF
AWIACNVAVMLTYLEISIFKQGGTILTINTESASWWKNNGIGCTVEYESSSQTGSTSSAIIAKVTGCILE
YKYVEVIHAGIQCFFSLLGIIIGSWLTHRLYNPKAIDQSANSASYRSTNFHLQNV

Potassium Voltage-Gated Channel subfamily Q-KCNQ

>Homo sapiens_KCNQ4(ENSP_262916)
MAEAPPRRLGLGPPPGDAPRAELVALTAVQSEQGEAGGGGSPRRLGLLGSPLPPGAPLPG
PGSGSGSACGQRSSAAHKRYRRLQNWVYNVLERPRGWAFVYHVFIFLLVFSCLVLSVLST
IQEHQELANECLLILEFVMIVVFGLEYIVRVWSAGCCCRYRGWQGRFRFARKPFCVIDFI
VFVASVAVIAAGTQGNIFATSALRSMRFLQILRMVRMDRRGGTWKLLGSVVYAHSKELIT
AWYIGFLVLIFASFLVYLAEKDANSDFSSYADSLWWGTITLTTIGYGDKTPHTWLGRVLA
AGFALLGISFFALPAGILGSGFALKVQEQHRQKHFEKRRMPAANLIQAAWRLYSTDMSRA
YLTATWYYYDSILPSFRELALLFEHVQRARNGGLRPLEVRRAPVPDGAPSRYPPVATCHR
PGSTSFCPGESSRMGIKDRIRMGSSQRRTGPSKQHLAPPTMPTSPSSEQVGEATSPTKVQ
KSWSFNDRTRFRASLRLKPRTSAEDAPSEEVAEEKSYQCELTVDDIMPAVKTVIRSIRIL
KFLVAKRKFKETLRPYDVKDVIEQYSAGHLDMLGRIKSLQTRVDQIVGRGPGDRKAREKG
DKGPSDAEVVDEISMMGRVVKVEKQVQSIEHKLDLLLGFYSRCLRSGTSASLGAVQVPLF
DPDITSDYHSPVDHEDISVSAQTLSISRSVSTNMD

>Pan troglodytes_KCNQ4(ENSPTRP_1033)
MAEAPPRRLGLGPPPGDAPRAELVALTAVQSEQGEAGGGGSPRRLGLLGSPLPPGAPLPG
PGSGSGSACGQRSSAAHKRYRRLQNWVYNVLERPRGWAFVYHVFIFLLVFSCLVLSVLST
IQEHQELANECLLILEFVMIVVFGLEYIVRVWSAGCCCRYRGWQGRFRFARKPFCVIDFI
VFVASVAVIAAGTQGNIFATSALRSMRFLQILRMVRMDRRGGTWKLLGSVVYAHSKELIT
AWYIGFLVLIFASFLVYLAEKDANSDFSSYADSLWWGTITLTTIGYGDKTPHTWLGRVLA
AGFALLGISFFALPAGILGSGFALKVQEQHRQKHFEKRRMPAANLIQAAWRLYSTDMSRA
YLTATWYYYDSILPSFRELALLFEHVQRARNGGLRPLEVRRAPVPDGAPSRYPPVATCHR
PGSTSFCPGESSRMGIKDRIRMGSSQRRTGPSKQHLAPPTMPTSPSSEQVGEATSPTKVQ
KSWSFNDRTRFRASLRLKPRTSAEDAPSEEVAEEKSYQCELTVDDIMPAVKTVIRSVRIL
KFLVAKRKFKETLRPYDVKDVIEQYSAGHLDMLGRIKSLQTRVDQIVGRGPGDRKAREKG
DKGPSDAEVVDEISMMGRVVKVEKQVQSIEHKLDLLLGFYSRCLRSGTSASLGAVQVPLF
DPDITSDYHSPVDHEDISVSAQTLSISRSVSTNMD

>Gorilla gorilla_KCNQ4(ENSGGOP_21390)
FLLVFSCLVLSVLSTIQEHQELANECLLILEFVMIVVFGLEYIVRVWSAGCCCRYRGWQG
RFRFARKPFCVIGNEXEELRKKSELGLREMGDLYPFPVWKSPTSPRQAQRSELITAWYIG
FLVLIFASFLVYLAEKDANSDFSSYADSLWWGTITLTTIGYGDKTPHTWLGRVLAAGFAL
LGISFFALPAGILGSGFALKVQEQHRQKHFEKRRMPAANLIQAAWRLYSTDMSRAYLTAT
WYYYDSILPSFRELALLFEHVQRARNGGLRPLEVRRAPVPDGAPSRYPPVATCHRPGSTS
FCPGESSRMGIKDRIRMGSSQRRTGPSKQHLAPPTMPTSPSSEQVGEATSPTKVQKSWSF
NDRTRFRASLRLKPRTSAEDAPSEEVAEEKSYQCELTVDDVMPAVKTVIRSVRILKFLVA
KRKFKETLRPYDVKDVIEQYSAGHLDMLGRIKSLQTRVDQIVGRGPGDRKAREKGDKGPS
DAEVVDEISMMGRVVKVEKQVQSIEHKLDLLLGFYSRCLRSGTSASLGAVQVPLFDPDIT

SDYHSPVDHEDISVSAQTLSISRSVSTNMD

>Mus musculus_KCNQ4(ENSMUSP_30376)
MAEAPPRRLGLGPPPGDAPRAELVALTAVQSEQGEAGGGGSPRRLGLLGSPLPPGAPLPG
PGSGSGSACGGQRSSAAQKRYRRLQNWVYNVLERPRGWAFVYHVFIFLLVFSCLVLSVLS
TIQEHQELANECLLILEFVMIVVFGLEYIIRVWSAGCCCRYRGWQGRFRFARKPFCVIDF
IVFVASVAVIAAGTQGNIFATSALRSMRFLQILRMVRMDRRGGTWKLLGSVVYAHSKELI
TAWYIGFLVLIFASFLVYLAEKDANSDFSSYADSLWWGTITLTTIGYGDKTPHTWLGRVL
AAGFALLGISFFALPAGILGSGFALKVQEQHRQKHFEKRRMPAANLIQAAWRLYSTDTSR
AYLTATWYYYDSILPSFRELALLFEHIQRARNGGLRPLEVRRAPVPDGAPSRYPPVATCH
RPGSASFCPGESSRMGIKDRIRISSSQKRTGPSKQHLAPPPIPTSPSSEQVGEASSPSKV
QKSWSFNDRTRFRASLRLKPRCSAEEGPSEEVAEEKSYQCELTVDDVMPAVKTVIRSVRI
LKFLVAKRKFKETLRPYDVKDVIEQYSAGHLDMLGRIKSLQARVDQIVGRGPGDRKTREK
GDKGPSDTEAVDEISMMGRVVKVEKQVQSIEHKLDLLLGFYSRCLRSGTSASLGTVQVPL
FDPDITSDYHSPVDHEDISVSAQTLSISRSVSTNMD

>Dasypus novemcinctus_KCNQ4(ENSDNOP_17886)
MAEAPPRRLGLAPPPGDAPRAELVALTAVQSEQGEAGGGGSPRRHGLLGSPLPPGAPLPG
PGSGSGSACGQRSSAAHKRYRRLQNWVYNVLERPRGWAFVYHVFIFLLVFSCLVLSVLST
IQEHQELANECLLILEFVMIVVFGLEYIVRVWSAGCCCRYRGWQGRFRFARKPFCVIDFI
VFVASVAVIAAGTQGNIFATSALRSMRFLQILRMVRMDRRGGTWKLLGSVVYAHSKELIT
AWYIGFLVLIFASFLVYLAEKDANSDFSSYADSLWWGTITLTTIGYGDKTPHTWLGRVLA
AGFALLGISFFALPAGILGSGFALKVQEQHRQKHFEKRRMPAANLIQAAWRLYSTDTSRA
YLTATWYYYDSILPSFRELALLFEHVQRARNGGLRPLEVRRVPVPDGAPSRYPPVATCHR
PGSTSFCPGESSRMGIKDRIRMGSSQRRTGPSKQHLAPPPIPTSPSSEQMGEATSPTKVQ
KSWSFNDRTRFRASLRLKPRTSAEEGPSEDVAEEKSYQCELTVDDVMPAVKTVIRSVRIL
KFLVAKRKFKETLRPYDVKDVIEQYSAGHLDMLGRIKSLQARVDQIVGRGTGDRKAREKG
DKGPSDPEAVDEISMMGRVVKVEKQVQSIEHKLDLLLGFYSRCLRSGTSASLGTVQVPLF
DPDITSDYHSPVDHEDISVSAQTLSISRSVSTNMD

>Xenopus tropicalis_KCNQ4(ENSXETP_49800)
MAVGTQPQADNGRVPSLSLAPISGQESGMEFVALTAVQTDSGNPRRLGLLGTPIPGAPRS
SSSHKKYRRLQNYLYNVLERPRGWAFIYHAFIFFLVFSCLVLSVFSTIPEYHHLANKGLF
ILEFVMIVVFGMEYVIRIWSAGCCCRYRGWKGRLRFARKPFCVIDFIVLVASLAVIAAGT
QGNIFATSALRSMRFLQILRMVRMDRRGGTWKLLGSVVYAHSKELITAWYIGFLVLIFAS
FLVYLAEKDANKEFSTYADSLWWGTITLTTIGYGDKTPHTWLGRVLAAGFALLGISFFAL
PAGILGSGFALKVQEQHRQKHFEKRRTPAANLIQAAWRLYSTDVSRSYLTATWYYYDSIL
PSFRELLLLFDTWHRTCSRNGGVRLCQGSPRYQPVPSTPKPATPPFCTGESSKLGLKDRM
RGTVAPKQTTRSKQYLAPPERASPGTEPAAETPSPTKVQKSWSFNDRTRFRASLRLKSRP
QADEGPGDDINDEKRLPNDLTMEDVMPTIKTLIRAFRILKFLVAKRKFKETLRPYDVKDV
IEQYSAGHLDMLGRIKSLQGRVDQIIGRGPVPVEKKPRDKGEKVSTMGPEVEAADELSMM
GRVVKVERQVQSIEDKLDILLGFYSQCIRKGPASSFTLTSMQMPLIDQEMTSDYQSPVDP
VDFSVSAQTLNISHSASTNME

>Takifugu rubripes_KCNQ4(ENSTRUP_36608)
MLGSPSNNGGIRMLAPPAPDDDRRVEFVALTAVHTERSEPSTPERGHPSHRTGLLGTPLP
GPPGPRANTSASSKRFRKLQNFLYNVLERPRGFAFIYHAFNFLLVFSCLVLSVFSTIHEH
QKFANQDSSSWWLLHEFVMIVVFGLEYFIRIWAAGCCCRYRGWQGRLRFARKPFCVIDLI
VFVPSLAVIAAGTQGNIFATSALRSMRFLQILRMVRMDRRGGTWKLLGSVVYAHSKELIT
AWYIGFLVLIFASFFVYLVEKDVNTDFNTYADSLWWGTITLTTIGYGDKTPRTWQGRLLA
AGFALLGVSFFALPAGILGSGFALKVQEQHRQKHFEKRRMPAANLIQAAWRLYSTDASHS
YLTATWYFYDSMLPSFRELTLLFSHLQRQRNKQLLPLHDTYAQIHAGRKQRYPRLRACAR
GSADCFVPSRGEGSKIGFRDRIRMNNSRSSQTIRSKASPLPPGSNVRRSPSQENVPDPTS
PGKVQKSWSFNDRTRFRTSLRLKPRPPADVEGVGEDSTEDKPYCDMAMEEVIPAVKTLIR
AVRILKFLVAKRKFKETLRPYDVKDVIEQYSAGHLDMLGRIKSLQTRVDQIIGRGAVQPD
KKARPEKGEKTPPELDSLDELSMMGRVVKVEKQVQSIENKLDLLLNLYSHCLKKGSSNYT
LSTLLDPDLTSDYHSPTDQRDLFPSANTLNISTSESGNLE

>Tetraodon nigroviridis_KCNQ4(ENSTNIP_5762)
MLGSPSNNGGIRMLAPPAPDDERRVEFVALTAVHTERSEPSTPERGHPSHRTGLLGTPLP
GPPGPRASASASSKRFRKLQNFLYNVLERPRGFAFIYHAFIFLLVFSCLVLSVFSTIPEH

QKFANQGLFILEFVMIVVFGLEYFIRIWAAGCCCRYRGWQGRLRFARKPFCVIDLIVFVA
SLAVIAAGTQGNIFATSALRSMRFLQILRMVRMDRRGGTWKLLGSVVYAHSKELITAWYI
GFLVLIFASFLVYLAEKDINTDFSTYADSLWWGTITLTTIGYGDKTPRTWQGRLLAAGFA
LLGVSFFALPAGILGSGFALKVQEQHRQKHFEKRRMPAANLIQAAWRLYSTDASHSYLTA
TWYFYDSMLPSFRELTLLFSHLQRRTPGALWSIETSHLTPPPCSQERVPDLKPCPPRFHS
FFFAVSSKIGFRDRIRMNNSRSSQTIRSKASPLPPGSNGRRSPSQENMADSTSPGKVQKS
WSFNDRTRFRTSLRLKPRPPADAEGVGEDSVEDKPYCDVAMEEVIPAVKTLIRAVRILKF
LVAKRKFKETLRPYDVKDVIEQYSAGHLDMLGRIKSLQTRVDQIIGRGAVQPDKKVRPEK
GEKTPPELDSLDELSMMGRVVKVEKQQVQSIENKLDLLLDLYSHCLKKGSSNFTLSTLLD
PDLTSDYHSPTDQRDLFPSANTLNISTSESGNLE

>Gasterosteus aculeatus_KCNQ4(ENSGACP_6436)
MPESPSNNGGARMLAPPTSSDDRRVEFVALTAVHTERSEPSSPERGHPSNRTGLLGTPHT
SASNKRYRKLQNCLYNVLERPRGWAFIYHAFIFLLVFSCLVLSVFSTIPDHQRFANQGLF
ILEFVMIVVFGLEYFIRIWAAGCCCRYRGWQGRLRFARKPFCVIDFIVFVASLAVIAAGT
QGNIFATSALRSMRFLQILRMVRMDRRGGTWKLLGSVVYAHSKELITAWYIGFLVLIFAS
FLVYLAEKDINSDFNTYADSLWWGTITLTTIGYGDKTPRTWQGRLLAAGFALLGVSFFCP
CLLTPQGILGSGFALKVQEQHRQKHFEKRRMPAANLIQAAWRLYSTDAKHSYLTATWYFY
DSMLPSFSARGGVRELTLLFSHLQRQRNTKKVLPSSYHTLLSGLRPYSLPYLSGDSSKMG
FRDRIRMNNSRSSQTIRSKGSPLPPSSGVRGSPSQENVPEAISPGKVQKSWSFNDRTRFR
TSLRLKTRPPVDGSVEGVGEDSVEDKPYCDVSMEEVIPAVKTLIRAVRILKFLVAKRKFK
ETLRPYDVKDVIEQYSAGHLDMLGRIKSLQMRVDQIIGRGAVQPDKKARTEKGEKTPPEL
DPLDELSMMGRVVKVEKQVQSIENKLDLLLSFYSQCLKKGSSHFTLSSLLDPELTSDYHS
PTDQRDLFPSANTLNISESGNLE

>Oryzias latipes_KCNQ4(ENSORLP_15074)
PGPNDDRRVEFVALTAVQTERSEPSTPERGPPSHRTGLLGTPLPGPPGPRASASASSKRF
RKLQNFLYNVLERPRGWAFIYHAFMWEVRMSSVLFLCALQEFVMIVVFGMEYFIRIWAAG
CCCRYRGWQGRLRFARKPFCVIDFIVFVASLAVIAAGTQGNIFATSALRSMRFLQILRMV
RMDRRGGTWKLLGSVVYAHSKELITAWYIGFLVLIFASFLVYLAEKDINSDFGTYADSLW
WGTITLTTIGYGDKTPRTWQGRLLAAGFALLGVSFFALPAGILGSGFALKVQEQHRQKHF
EKRRMPAANLIQAAWRLYSTDAKHSYLTATWYFYDSMLPSFRELTLLFSHLQRQRSTKKV
LQNSYHTLLSGLRPNSSPYLSGDSSKMGFRDRIRMNNSRSSQAIRNKASPLPPGSGVRRS
PSQENIPEATSPGKVQKSWSFNDRTRFRTSLRLKPRPPVDAEGLGEDSVEDKPYCDVAMD
EVIPAVKTLIRAVRILKFLVAKRKFKETLRPYDVKDVIEQYSAGHLDMLGRIKSLQMRVD
LIIGRGPVQPDKKTRPEKGEKTPPDLDPLDELSMMGRVVKVEKQVQSIEGKLDLLLSFYS
QCIKKGSSHVTLSSLLDPDLTSDYHSPTDHRDLFPSANTLNISRSESDNLE

>Homo sapiens_KCNQ3(ENSP_373648)
MGLKARRAAGAAGGGGDGGGGGGGAANPAGGDAAAAGDEERKVGLAPGDVEQVTLALGAG
ADKDGTLLLEGGGRDEGQRRTPQGIGLLAKTPLSRPVKRNNAKYRRIQTLIYDALERPRG
WALLYHALVFLIVLGCLILAVLTTFKEYETVSGDWLLLLETFAIFIFGAEFALRIWAAGC
CCRYKGWRGRLKFARKPLCMLDIFVLIASVPVVAVGNQGNVLATSLRSLRFLQILRMLRM
DRRGGTWKLLGSAICAHSKELITAWYIGFLTLILSSFLVYLVEKDVPEVDAQGEEMKEEF
ETYADALWWGLITLATIGYGDKTPKTWEGRLIAATFSLIGVSFFALPAGILGSGLALKVQ
EQHRQKHFEKRRKPAAELIQAAWRYYATNPNRIDLVATWRFYESVVSFPFFRKEQLEAAS
SQKLGLLDRVRLSNPRGSNTKGKLFTPLNVDAIEESPSKEPKPVGLNNKERFRTAFRMKA
YAFWQSSEDAGTGDPMAEDRGYGNDFPIEDMIPTLKAAIRAVRILQFRLYKKKFKETLRP
YDVKDVIEQYSAGHLDMLSRIKYLQTRIDMIFTPGPPSTPKHKKSQKGSAFTFPSQQSPR
NEPYVARPSTSEIEDQSMMGKFVKVERQVQDMGKKLDFLVDMHMQHMERLQVQVTEYYPT
KGTSSPAEAEKKEDNRYSDLKTIICNYSETGPPEPPYSFHQVTIDKVSPYGFFAHDPVNL
PRGGPSSGKVQATPPSSATTYVERPTVLPILTLLDSRVSCHSQADLQGPYSDRISPRQRR
SITRDSDTPLSLMSVNHEELERSPSGFSISQDRDDYVFGPNGGSSWMREKRYLAEGETDT
DTDPFTPSGSMPLSSTGDGISDSVWTPSNKPI

>Macaca mulatta_KCNQ3(ENSMMUP_11684)
XGDAAAAGDEERKVGLAPGDVEQVTLALGAGADKDGTLLLEGGGRDEGQRRTPQGIGLLA
KTPLTRPVKRNNAKYRRIQTLIYDALERPRGWALLYHALVFLIVLGCLILAVLTTFKEYE
TVSGDWLLLLETFAIFIFGAEFALRIWAAGCCCRYKGWRGRLKFARKPLCMLDIFVLIAS
VPVVAVGNQGNVLATSLRSLRFLQILRMLRMDRRGGTWKLLGSAICAHSKELITAWYIGF
LTLILSSFLVYLVEKDVPEVDAQGEEMKEEFETYADALWWGLITLATIGYGDKTPKTWEG

RLIAATFSLIGVSFFALPAGILGSGLALKVQEQHRQKHFEKRRKPAAELIQAAWRYYATN
PNRIDLVATWRFYESVVSFPFFRQLEAASSQKLGLLDRVRLSNPRGSNTKGKLFTPLNVD
AIEESPSKEPKPVGLNNKERFRTAFRMKAYAFWQSSEDAGTGDPTAEDRGYGNDFPIEDM
IPTLKAAIRAVRILQFRLYKKKFKETLRPYDVKDVIEQYSAGHLDMLSRIKYLQTRIDMI
FTPGPPSTPKHKKSQKGSAFTFPSQQSPRNEPYVARPSTSEIEDQSMMGKFVKVERQVQD
MGKKLDFLVDMHMQHMERLQVQVTEYYPTKGTSSPAEAEKKEDNRYSDLKTIICNYSETG
PPEPPYSFHQVPIDKVSPYGFFAHDPVNLPRGGPSTGKVQAAPPSSATTYAERPTVLPIL
TLLDSRVSCHSQADLQGPYSDRISPQQRRSITRDSDTPLSLMSVNHEELERSPSGFSISQ
DRDDYVFGPNGGSSWMREKRYLAEGETDTDTDPFTPSGSMPLSSTGDGISDSVWTPSNKP
I

>Mus musculus_KCNQ3(ENSMUSP_63380)
MGLKARRAAGAAGGGGGEGGGGGGGAANPAGGDSAVAGDEERKVGLAPGDVEQVTLALGA
GADKDGTLLLEGGGREEGQRRTPQGIGLLAKTPLSRPVKRNNAKYRRIQTLIYDALERPR
GWALLYHALVFLIVLGCLILAVLTTFKEYETVSGDWLLLLETFAIFIFGAEFALRIWAAG
CCCRYKGWRGRLKFARKPLCMLDIFVLIASVPVVAVGNQGNVLATSLRSLRFLQILRMLR
MDRRGGTWKLLGSAICAHSKELITAWYIGFLTLILSSFLVYLVEKDVPEMDAQGEEMKEE
FETYADALWWGLITLATIGYGDKTPKTWEGRLIAATFSLIGVSFFALPAGILGSGLALKV
QEQHRQKHFEKRRKPAAELIQAAWRYYATNPNRLDLVATWRFYESVVSFPFFRKEQLEAA
ASQKLGLLDRVRLSNPRGSNTKGKLFTPLNVDAIEESPSKEPKPVGLNNKERFRTAFRMK
AYAFWQSSEDAGTGDPMAEDRGYGNDFLIEDMIPTLKAAIRAVRILQFRLYKKKFKETLR
PYDVKDVIEQYSAGHLDMLSRIKYLQTRIDMIFTPGPPSTPKHKKSQKGSAFTYPSQQSP
RNEPYVARAATSETEDQSMMGKFVKVERQVHDMGKKLDFLVDMHMQHMERLQVHVTEYYP
TKGASSPAEGEKKEDNRYSDLKTIICNYSETGPPDPPYSFHQVPIDRVGPYGFFAHDPVK
LTRGGPSSTKAQANLPSSGSTYAERPTVLPILTLLDSCVSYHSQTELQGPYSDHISPRQR
RSITRDSDTPLSLMSVNHEELERSPSGFSISQDRDDYVFGPSGGSSWMREKRYLAEGETD
TDTDPFTPSGSMPMSSTGDGISDSIWTPSNKPT

>Bos taurus_KCNQ3(ENSBTAP_27546)
MGLKARRPAGAAGGGGDGGGGGGGAANPAGGDAAAAGDEERKVGLAPGDVEQVTLALGAG
ADKDGTLLLEGGGRDEGQRRTPQGIGLLAKTPLSRPVKRNNAKYRRIQTLIYDALERPRG
WALLYHALVFLIVLGCLILAVLTTFREYETVSGDWLLLLETFAIFIFGAEFALRIWAAGC
CCRYKGWRGRLKFARKPLCMLDIFVLIASVPVVAVGNQGNVLATSLRSLRFLQILRMLRM
DRRGGTWKLLGSAICAHSKELITAWYIGFLTLILSSFLVYLVEKDVPEVDAQGEEMKEEF
ETYADALWWGLITLATIGYGDKTPKTWEGRLIAATFSLIGVSFFALPAGILGSGLALKVQ
EQHRQKHFEKRRKPAAELIQAAWRYYATNPNRIDLVATWRFYESVVSFPFFRKEQLDPAA
SQKLGLLDRVRLSNPRGSNTKGKLFTPLNVDAIEESPSKEPKPVGSNNKERFRTAFRMKA
YAFWQSSEDAGTGDPTAEDRGYGNDFLIEDMIPTLKAAIRAVRILQFRLYKKKFKETLRP
YDVKDVIEQYSAGHLDMLSRIKYLQTRIDMIFTPGPPSTPKHKKSQRGAAFTYPSQQSPR
NEPYVARPSTSETEDQSMMGKFVKVERQVHDMGKKLDFLVDMHLQHMERLQVHVAGFSPS
KGASSPAEAEQKEDRRDADLKTIICNYSETGAPDAPYSFHQVPVDKVGPYGFFAHDPVNL
PLGGPSSGKGHATPYAERPTVLPILTLLDSRGSYRSQVELHGPCSDRVSPRQRRSITRDS
DTPLSLMSVNHEELERSPSGFSISQDRDDYAFGPSGGSSWMREKRYLAEGETDTDTEPFT
PSGSLPLSSTGDGISDSIWTPSGKPT

>Myotis lucifugus_KCNQ3(ENSMLUP_14023)
QTPSSFPPRFLIVLGCLILAVLTTFKEYETVSGDWLLLLETFAIFVFGAEFALRIWAAGC
CCRYKGWRGRLKFARKPLCMLDIFVLIASVPVVAVGNQGNVLATSLRSLRFLQILRMLRM
DRRGGTWKLLGSAICAHSKELITAWYIGFLTLILSSFLVYLVEKDVPEVDAQGEEMKEEF
ETYADALWWGLITLATIGYGDKTPKTWEGRLIAATFSLIGVSFFALPAGILGSGLALKVQ
EQHRQKHFEKRRKPAAELIQAAWRYYATNPNRIDLVATWRFYESVVSFPFFRKEQLEAAA
SQKLGLLDRVRLSNPRGSNTKGKLFTPLNVDAIEESPSKEPKPVGLNNKERFRTAFRMKA
YAFWQSSEDAGTGDPMAEDRDYGNDFIEDMIPTLKAVIRAVRILQFRLYKKKFKETLRPY
DVKDVIEQYSAGHLDMLSRIKYLQTRIDMIFTPGPPSTPKHKKSQKGAAFTYPSQQSPRN
EPYLARASTSEMEDQSMMGKFVKVERQVQDMGRKLDFLVDMHMQHMERLQAHVPYYDPAK
GTSPAAEEERGDPRDSEMKTILCNYSETGLPDPSFSFHQVPIDKVSPYRFFAHDPVGLPR
GGPGSGRVPGTLSSSTPAYMERPTVLPILTLLPSPTDPHGLRQRRSFTRDSDTPLSLMSV
DHEELERSPSGFSISQDRDDVFGLGGGSSWMREKRYLAEGETDTDTDPFTPSSSMPLSST
GDGISDSIWTPSNKPV

>Monodelphis domestica_KCNQ3(ENSMODP_1454)

FLIVLGCLILAVLTTFKEYETVSGDWLLLLETFAIFIFGAEFALRIWAAGCCCRYKGWRG
RLKFARKPLCMLDIFVLIASVPVVAVGNQGNVLATSLRSLRFLQILRMLRMDRRGGTWKL
LGSAICAHSKELITAWYIGFLTLILSSFLVYLVEKDVPEIDAQGVEMKEEFETYADALWW
GLITLATIGYGDKTPKTWEGRLIAATFSLIGVSFFALPAGILGSGLALKVQEQHRQKHFE
KRRKPAAELIQAAWRYYATNPNRIDLVATWRFYESIVSFPYFRQQLEAAASQKLGLLDRV
RLSNPRGTNTKGKLFTPLNVDAIEESPSKEPKNVGLNNKERFRTAFRMKAYAFWQSSEDA
GTGEPMAEERGYSNEFLIEDMIPTLKSAIRAVRPAVFALFCTVTGLASTKYSEILFSYFF
RSFRVDMIFTPGPPSTPKHKKSQRGGAFTYPSQQSPRNDPYVAKASSSEIEDQSMMGKFV
KVERQVHDMGKKLDFLVDMHMQHMEQLQVQVTEYYPTKKATATPAEAEKKEENKYSELKT
IIYNYSEPCTQESPYSFHQVPVNKVSPYAFFAHDPEKLPRTVPSSVPYAERPTVLPILTL
LDSRVSYHSQAELHSPYQDRISPRQRRSFTRDSDTPLSLLSVNHEELERSPSGFSISQDR
DDYLFGPNGGSSWMREKRYLAEGETDTDTDPFTPSGSMPLSSTGDGISDSIWTPSNKPV

>Gallus gallus_KCNQ3(ENSGALP_56287)
MGLKARRGAAGGSGAGGAAEGRRAAGGGGGDPEQGSLALGSGADRDGTLLLEGGGKEEGG
KRSPPGLGLLAKTPLSRPVKRNHAKYRRIQTLIYDALERPRGWALLYHGFVFLIVLGCLI
LAVLTTFKEYETVSGDWLLLLETFAIFIFGAEFALRIWAAGCCCRYKGWRGRLKFARKPL
CMLDIFVLIASVPVVAVGNQGNVLATSLRSLRFLQILRMLRMDRRGGTWKLLGSAICAHS
KELITAWYIGFLTLILSSFLVYLVEKDVPEKDANGVEMKEEFETYADALWWGLITLATIG
YGDKTPKTWEGRLIAATFSLIGVSFFALPAGILGSGLALKVQEQHRQKHFEKRRKPAAEL
IQAAWRYYATNPNRIDLVATWRFYESIVSFPFFRKEQLDGTASQKLGLLDRVRGTNTKGK
LFTPLNVDAIEESPSKEPKNVVLNNKERFRTAFRMKAYAFWQSSEDAGTGEPGGEERGDS
SDFLMEDMIPTFKTAIRAVRILQFRLYKKKFKETLRPYDVKDVIEQYSAGHLDMLSRIKY
LQARVDMIFTPGPPSTPKHKKSQKGGAFSYPSQQSPRNDPYVAKASTVDTEDQSMMGKFV
KVERQVNDMGKKLDFLVDMHMHHMEQLQIQVAEISPLKETASPAKEKREENKYSELKTII
YKYTDQCTHETPYNFQQVPVNKVSPYGFSAHGHMTHSQPLSTGGQNSGKLQATPSSASYA
ERPTVLPIFTLMDSQVSYHSQGDMQSPYSDKVSPRQRRSLTRDSDTPLSLLSVNHEELER
SPSGFSISQDRDDFPFGPNGGCAWMREKRYLAEGETDTDTDPFTPSGSMPLSSTGDGISD
SIWTPSNKPV

>Anolis carolinensis_KCNQ3(ENSACAP_10760)
MGLKARKAAAAGAKAAPSSSLAAPPPRPRGPGAEPGEGPKGADLEQGGGGGAGADKDGTL
LLGRPTREEEDDDEEEGLPQRRSAGQGLGLLAKTPLSRHPVKRHNLKYRRSQTLIYDALE
RPRGWALLYHAFVFLIVLGCLILAVLTTFKDYEKASEKWLLLLETFAIFIFGAEFALRIW
AAGCCCRYKGWRGRLKFARKPLCMLDIFVLIASVPVVAVGNQGNVLATSLRSLRFLQILR
MLRMDRRGGTWKLLGSAICAHSKELITAWYIGFLTLILSSFLVYLVEKDVAETNEQGQET
RDFETYADALWWGLITLATIGYGDKTPKTWEGRLIAATFSLVGVSFFALPAGILGSGLAL
KVQEQHRQKHFEKRRKPAAELIQAAWRYYATNPNRLDLVATWRFYESVVSFPYFRKEQVD
GTSQKLGLLDRVRGTNTKGKLFTPLNVDAIEESPSKEPKSVVVLNNRERFRTAFRMKAYA
FWQSSEDAGTGDPLAEDRGYSNELITEDMIPTLKAVIRAVRILQFRLYKKRFKETLRPYD
VKDVIEQYSAGHLDMLSRIKYLQTRIDMIFAPGPPSTPKHKKSQKGGTFSYPSQQSPRNE
PYIAKASTVDSEDQSMMGKFVRVERQVHDMGKKLDFLVDMHMQHMEQLQVQVTEVCPSKE
ASSPAEDNRYSELKSIIYKYSEPSIHEKPPSFHHVPVNKVPPFGFTTQGQSCGPFPSPFG
GQNSSRWPAMPFSTSYAERPTVLPILTFIDPHISYQSPPGDFQSPHSERIPSRQKHSVTR
DSDTPLSLLSVNHEELERSPSGFSISQDRDDFLFGPNGGSSWMKEKRYLAEGETDTDTDP
FTPSGSMPLSSTGDGLSDSIWTPSSKPV

>Xenopus tropicalis_KCNQ3(ENSXETP_49802)
WAANWATLMFLIVLGCLILAILTTFKEYEDASGDWLLLLETFALVIFGVEFALRIWAAGC
CCRYKGWRGRLKFARKPLCMLDIYIYIPAFSLLTIGHNNQICCRLVNSLQWVASHLKGTM
SYSTWENLNLHYMAANRELITAWYIGFLTLILSSFLVYLVEKDVPERNEKGELVEEEFET
YADALWWGLITLATIGYGDKTPKTWEGRLIAATFSLIGVSFFALPAGILGSGLALKVQEQ
HRQKHFEKRRKPAAELIQSAWRFYATNLSRIDLIATWRYYETTVQFPYFRLRKEQMDGTN
RLVIHFGLFVERIGSEKGLYGKYILFLPLGVTVAAPMNSKGDRGPGLHHCNKFHNDFQAK
IQSLWSKNDAGAAGEPIAESGYGAEAMLEDLIPTIKAAIHASRILQFLLHKKRFKETLRP
YDVKDVIEQYSAGHLDMLSRIKYLQTRIDMVFAPGPLSTPKHKKSQKGTGGLGGGFGYSS
QQSPRHDPYSVKVPEIEDQSMMGKFVKVERQVQDMEKKLDFLVDMHMQHMDQLQVHITEY
YPLRETLAEDEGVDSRYSEVEGLIYNYTESCAHTSPYHHLHQVSFNKTNQYGFFENYPRN
FSAPLPFNGENCPRVATPPSSSYTERPTVLPIQTLIDTRVEFQDSGGASNPYLKEASPTQ
RRSFRDSDTPLSLLSVNHDELQRSPSGFSISQEKEDFSFGNNGSNWMREKRFLAEGETDT
DTDPFTPSGPITLSSTGDGISESAWTPSKPV

>Tetraodon nigroviridis_KCNQ3(ENSTNIP_8726)
GFSLVFHVFRFLIVLGCLILAVLTTFKEHEKVSAHWLVILETFAIFIFGAEFALRIWAAG
CRCRYKGWRGRLQFARKAALRAGDIFVLIASVPVVAVRNQGNVLATLRSLRFLQILRMLR
MDRRGGTWKLLGSAIYAHSKELITAWYIGFLSLILASFLVYLVEKDDVSVDASDPESPTA
QPRTQDFDTYADALWWGAGRSDGPHSCEQRTVGALVGDRRNLPHADGSSWNQTNNLSGLI
RNLLCCPSSQKLRLLERVRLPNAPTGSSDGAEESPSKPAGFSNRERLRTAFRMRAYTLRQ
SSEDPAAPTEAAVEERGFPPDILLEEMIPTLKLVIRAVRILQFLLNKKRFKETLRPYDVK
DVIEQYSAGHLDMLCRIKYLQTRIDMILAPGPPLTPKQKKSQKPPFACPSSQSPRHESFL
PKAASLPDAEDQSMMGRFVRVERQVEDMEKKLDFLVDMHIQHTEHLQVDSVGTAHMTLEA
CHPTGNGEIRRVFLNYAEGFPHMAYQVPVGGASRTREGQRLPAAVPTYTERPTVLPISSL
QDQAGGVARTTGGRGADTPLSMLSVNHEELERSPSGFSISGERDGEDGGGPSAGATVRTR
ARPTHLAEGETDTDTDPFTPSGGPLPLSSTGEGFGESVWATPP

>Gasterosteus aculeatus_KCNQ3(ENSGACP_20863)
ASGSEEQKQTCGAPPGDLDQGTGGADRDGALLLAAPGREDFKRGSQGIGIGLLAKTPLNY
TGPAKRNNNRRRRIQNLLYDALERPRGWALLYHAFVFLIVLGCLILAILTTFREHEKVSA
HWLVILETFAIFIFGAEFGLRVWAAGCCCRYKGWRGEAQVRKLLCILGKHVSNVGIGSSL
KEHSRVNAACRGGTQVTEVTEVTVSHLQITLTTIGYGDKTPKTWAGRLLAGTFALIGVSF
FALPAGILGSGLALKVQEQHRQKHFEKRRHPAAGLIQSAWRYYSTNPIREDLIATWRFYE
TIISLPCFRKDHLEAIASQKLSLLERVRLPNARPSIGTGKRLTGHTDSIEESPSKEPKPA
GFSNRERFRTAFRMKAYTLRQSSEDGGALAEPALEERGFPPDILMEEMIPTLKLVIRAVR
IMQFLLNKKRFKETLRPYDVKDVIEQYSAGHLDMLCRIKYLQTRIDMILAPGPLLTPKQK
KTPKAPFNYPANQSPRHESYLAKATSIPDVEDLSMMGRFVRVERQVEDMEKKLDFLVDMH
IQHSEHLQVDSAGAAHMTLEACNPTGAGEIRRVFFNYAESFPHLSYQMPVGKMSGGSGGG
GGHQPPPPLLHQRRPPAAVPTYAERPTVLPISSLQDLSAGPGRTTGARGGDSPLSMLSVH
DELERSSGFSISGSRMKPAGRDPDPSYLAEGETDTDTDPFTPSGGALPLSSPGEGFGDAV
WNTPP

>Oryzias latipes_KCNQ3(ENSORLP_8236)
FLIVLGCLILAILTTFKEHENTSAHWLVILETFAIFIFGAEFGLRIWAAGCCCRYKGWRG
RLKFARKPLCILDIFVLIASVPVVVVRNQGNVLATSLRSLRFLQILRMLRMDRRGGTWKL
LGSAIYAHSKELITAWYIGFLSLILASFLVYLVEKDDVSVDGSDREDPTVQPKQQDFDTY
ADALWWGLITLTTIGYGDKTPKTWAGRLLAGTFALIGVSFFALPAGILGSGLALKVQEQH
RQKHFEKRRHPAAGLIQSAWRYYSTNPVRDDLIATWRFYETIISLPCFRQDTFEVMVSQK
LSLLERVRLSNTRPSVGMGKRLMGNADSVEDSPSKESKPAGFSNRERFRTAFRMKAYTLR
QSSEDAGALADPALEERGFPPDILMEDMIPTLKLVIRATRIMQFLLNKKRFKETLRPYDV
KDVIEQYSAGHLDMLCRIKYLQTRIDMILAPGPLLTPKSKKTQKTPFSYPANQSPRQESY
LPKTTAMPDTDDQSMMGRFVRVERQVEDMEKKLDFLVDMHIQHTEHLQVDSAGTAHMTLE
TCGPGANGEIRRVFLNYSEAFPHMSYQVAIGKAGRGRGDQECRRGAGLARGHPERPAMGT
VPTYIERPTVLPISSLQDLSAGPGRTTGRQGGDSPLSMLSVNHEELERSPSGFSISVDRE
GDERVPTEADMATGEVSWTRPRPSYLAEGETDTDTDPFTPSGGALPLSSTGEGFCDAEWN
APL

>Homo sapiens_KCNQ2(ENSP_486706)
MVQKSRNGGVYPGPSGEKKLKVGFVGLDPGAPDSTRDGALLIAGSEAPKRGSILSKPRAG
GAGAGKPPKRNAFYRKLQNFLYNVLERPRGWAFIYHAYVFLLVFSCLVLSVFSTIKEYEK
SSEGALYILEIVTIVVFGVEYFVRIWAAGCCCRYRGWRGRLKFARKPFCVIDIMVLIASI
AVLAAGSQGNVFATSALRSLRFLQILRMIRMDRRGGTWKLLGSVVYAHSKELVTAWYIGF
LCLILASFLVYLAEKGENDHFDTYADALWWGLITLTTIGYGDKYPQTWNGRLLAATFTLI
GVSFFALPAGILGSGFALKVQEQHRQKHFEKRRNPAAGLIQSAWRFYATNLSRTDLHSTW
QYYERTVTVPMYSSQTQTYGASRLIPPLNQLELLRNLKSKSGLAFRKDPPPEPSPSQKVS
LKDRVFSSPRGVAAKGKGSPQAQTVRRSPSADQSLEDSPSKVPKSWSFGDRSRARQAFRI
KGAASRQNSEEASLPGEDIVDDKSCPCEFVTEDLTPGLKVSIRAVCVMRFLVSKRKFKES
LRPYDVMDVIEQYSAGHLDMLSRIKSLQSRVDQIVGRGPAITDKDRTKGPAEAELPEDPS
MMGRLGKVEKQVLSMEKKLDFLVNIYMQRMGIPPTETEAYFGAKEPEPAPPYHSPEDSRE
HVDRHGCIVKIVRSSSSTGQKNFSAPPAAPPVQCPPSTSWQPQSHPRQGHGTSPVGDHGS
LVRIPPPPAHERSLSAYGGGNRASMEFLRQEDTPGCRPPEGNLRDSDTSISIPSVDHEEL
ERSFSGFSISQSKENLDALNSCYAAVAPCAKVRPYIAEGESDTDSDLCTPCGPPPRSATG
EGPFGDVGWAGPRK

>Pan troglodytes_KCNQ2(ENSPTRP_23658)
MVQKSRNGGVYPGPSGEKKLKVGFVGLDPGAPDSTRDGALLIAGSEAPKRGSILSKPRAG
GAGAGKPPKRNAFYRKLQNFLYNVLERPRGWAFIYHAYVFLLVFSWLEIVTIVVFGVEYF
VRIWAAGCCCRYRGWRGRLKFARKPFCVIDIMVLIASIAVLAAGSQGNVFATSALRSLRF
LQILRMIRMDRRGGTWKLLGSVVYAHSKELVTAWYIGFLCLILASFLVYLAEKGENDHFD
TYADALWWGLITLTTIGYGDKYPQTWNGRLLAATFTLIGVSFFALPAGILGSGFALKVQE
QHRQKHFEKRRNPAAGLIQSAWRFYATNLSRTDLHSTWQYYERTVTVPMYSSQTQTYGAS
RLIPPLNQLELLRNLKSKSGLAFRKDPPPEPSPSKGSPCRGPLCGCCPGRSSQKVSLKDR
VFSSPRGVAAKGKGSPQAQTVRRSPSADQSLEDSPSKVPKSWSFGDRSRARQAFRIKGAA
SRQNSEEASLPGEDIVDDKSCPCEFVTEDLTPGLKVSIRAVVDQIVGRGPAITDKDRTKG
PAEAELPEDPSMMGRLGKVEKQVLSMEKKLDFLVNIYMQRMGIPPTETEAYFGAKEPEPA
PPYHSPEDSREHVDRHGCIVKIVRSSSSTGQKNFSAPPAAPPVQCPPSTSWQPQSHPRQG
HGTSPVGDHGSLVRIPPPPAHERSLSAYGGGNRASMEFLRQEDTPGCRPPEGTLRDSDTS
ISIPSVDHEELERSFSGFSISQSKENLDALNSCYAAVAPCAKVRPYIAEGESDTDSDLCT
PCGPPPRSATGEGPFGDVGWAGPRK

>Mus musculus_KCNQ2(ENSMUSP_99340)
MVQKSRNGGVYPGTSGEKKLKVGFVGLDPGAPDSTRDGALLIAGSEAPKRGSVLSKPRTG
GAGAGKPPKRNAFYRKLQNFLYNVLERPRGWAFIYHAYVFLLVFSCLVLSVFSTIKEYEK
SSEGALYILEIVTIVVFGVEYFVRIWAAGCCCRYRGWRGRLKFARKPFCVIDIMVLIASI
AVLAAGSQGNVFATSALRSLRFLQILRMIRMDRRGGTWKLLGSVVYAHSKELVTAWYIGF
LCLILASFLVYLAEKGENDHFDTYADALWWGLITLTTIGYGDKYPQTWNGRLLAATFTLI
GVSFFALPAGILGSGFALKVQEQHRQKHFEKRRNPAAGLIQSAWRFYATNLSRTDLHSTW
QYYERTVTVPMYSSQTQTYGASRLIPPLNQLELLRNLKSKSGLTFRKEPQPEPSPSQKVS
LKDRVFSSPRGMAAKGKGSPQAQTVRRSPSADQSLDDSPSKVPKSWSFGDRSRTRQAFRI
KGAASRQNSEEASLPGEDIVEDNKSCNCEFVTEDLTPGLKVSIRAVCVMRFLVSKRKFKE
SLRPYDVMDVIEQYSAGHLDMLSRIKSLQSRVDQIVGRGPTITDKDRTKGPAETELPEDP
SMMGRLGKVEKQVLSMEKKLDFLVSIYTQRMGIPPAETEAYFGAKEPEPAPPYHSPEDSR
DHADKHGCIIKIVRSTSSTGQRNYAAPPAIPPAQCPPSTSWQQSHQRHGTSPVGDHGSLV
RIPPPPAHERSLSAYGGGNRASTEFLRLEGTPACRPSEAALRDSDTSISIPSVDHEELER
SFSGFSISQSKENLDALGSCYAAVAPCAKVRPYIAEGESDTDSDLCTPCGPPPRSATGEG
PFGDVAWAGPRK

>Rattus norvegicus_KCNQ2(ENSRNOP_40830)
MVQKSRNGGVYPGTSGEKKLKVGFVGLDPGAPDSTRDGALLIAGSEAPKRGSVLSKPRTG
GAGAGKPPKRNAFYRKLQNFLYNVLERPRGWAFIYHAYVFLLVFSCLVLSVFSTIKEYEK
SSEGALYILEIVTIVVFGVEYFVRIWAAGCCCRYRGWRGRLKFARKPFCVIDIMVLIASI
AVLAAGSQGNVFATSALRSLRFLQILRMIRMDRRGGTWKLLGSVVYAHSKELVTAWYIGF
LCLILASFLVYLAEKGENDHFDTYADALWWGLITLTTIGYGDKYPQTWNGRLLAATFTLI
GVSFFALPAGILGSGFALKVQEQHRQKHFEKRRNPAAGLIQSAWRFYATNLSRTDLHSTW
QYYERTVTVPMYSSQTQTYGASRLIPPLNQLELLRNLKSKSGLTFRKEPQPEPSPSQKVS
LKDRVFSSPRGVAAKGKGSPQAQTVRRSPSADQSLDDSPSKVPKSWSFGDRSRARQAFRI
KGAASRQNSEEASLPGEDIVEDNKSCNCEFVTEDLTPGLKVSIRAVCVMRFLVSKRKFKE
SLRPYDVMDVIEQYSAGHLDMLSRIKSLQSRVDQIVGRGPTITDKDRTKGPAETELPEDP
SMMGRLGKVEKQVLSMEKKLDFLVSIYTQRMGIPPAETEAYFGAKEPEPAPPYHSPEDSR
DHADKHGCIIKIVRSTSSTGQRNYAAPPVMPPAQCPPSTSWQQSHQRHGTSPVGDHGSLV
RIPPPPAHERSLSAYSGGNRASTEFLRLEGTPACRPSEAALRDSDTSISIPSVDHEELER
SFSGFSISQSKENLDALASCYAAVAPCAKVRPYIAEGESDTDSDLCTPCGPPPRSATGEG
PFGDVAWAGPRK

>Gallus gallus_KCNQ2(ENSGALP_9339)
MVQKSRNGGVYPGPAAEKKLKVGFVGLDPGAPDSSRDGALLIAGSESTKRGSILSKPRSG
VSGSGKPPKRNAFYRKLQNFLYNVLERPRGWAFIYHAYVFLLVFSCLVLSVFSTIDEYQN
SSEGALYILEIVTIVVFGVEYFVRIWAAGCCCRYRGWRGRLKFARKPFCVIDIMVLIASI
AVLAAGSQGNVFATSALRSLRFLQILRMIRMDRRGGTWKLLGSVVYAHSKELITAWYIGF
LCLILASFLVYLAEKGENEHFDTYADALWWGLITLTTIGYGDKYPQTWNGRLLAATFTLI
GVSFFALPAGILGSGFALKVQEQHRQKHFEKRRNPAAGLIQAAWRFYATNLSRTDLHSTW
QYYERTVTVPMYSTQTQTYGASRLIPPLNQLELLRNLKSKSGLTFRKEQQPEPSPSQKVS
LKDRVFSSPRGAGTKGKGSPQAQGIRRSPSADQSIEDSPSKVPKSWSFGDRSRARQAFRI
KGAASRQNSEEASLPGEDIPDENKSCNCEFVTEDLTPGLKVSIRAVCIMRFLVSKRKFKE
SLRPYDVMDVIEQYSAGHLDMLSRIKNLQSRVDQIVGRGSSMTNKDRTKGPAEGELPEDP

SMMGRLGKVEKQVQSMEKKLDFLVNIYMQRMGIPPTETEAYFGSKEPDPAPPYHSPEDSR
EHIDKNGCIIKIIRSTSSMGQKNFSAPPAPTNSCPPSTSCQQQPYQRQSHGSSPVGDPGS
LVRIPPPPSHERSLSAYSGGHRASAEYLRNEDITTKHHESALRDSDTSISIPSVDHEELE
RSFSGFSISQSKENLDILNNGCYAAVAKIRPYIAEGESDTDSDLCTPCGPPPRSATGEGP
FSDMGWSAPRQ

>Anolis carolinensis_KCNQ2(ENSACAP_5855)
MSFLFLFCLFLLPSSFLLVFSCLVLSVFSTIDDYEASSEGALYILEIVTIVVFGVEYFVR
IWAAGCCCRYRGWRGRLKFARKPFCVIDIMVLIASIAVLAAGSQGNVFATSALRSLRFLQ
ILRMIRMDRRGGTWKLLGSVVYAHSKELITAWYIGFLCLILASFLVYLAEKGENEHFDTY
ADALWWGLITLTTIGYGDKYPQTWNGRLLAATFTLIGVSFFALPAGILGSGFALKVQEQH
RQKHFEKRRNPAAGLIQAAWRFYATNLSRTDLHSTWNYYERTVTVPMYSSQTQSYGASRL
IPPLNQLELLRNLKSKSGLTFRKEQQPEPSPSQKVSLKDRVFSSPRGAATKGKGSPQAPG
IRRSPSADQSIDDSPSKVPKSWSFGDRSRARQAFRIKGASSRQNSEDAGLPGEDMGDPDN
KSCNCEFVTEDLTPGLKLSIRAVCVMRFLVSKRKFKESLRPYDVMDVIEQYSAGHLDMLS
RIKNLQSRIDMIVGPPPPSTPRHKKYSTKGSSPQYSPRVDQIVGRGPSMTDKDRTKGSAE
GELQEDPSMMGRLGKVEKQVQSMEKKLDFLVNIYMQRMGIPPTETEAYFGSKEPEPAPPY
HSPEDSREHIDKNGCIIKIIRSTSSMGQKNFSAPPATTVPNNSCPPSTSWQPYPRQSHGV
SPVGEPGSLVRIPPPPSHERSSSAYSGGHRASAEYAKNEDVTAKHPESALRDSDTSISIP
SVDHEELERSFSGFSISQSKENLDVLNNGCYTAVAKIRPYIAEGESDTDSDLCTPCGPPP
RSATGEGPFSDMGWSTPRQ

>Xenopus tropicalis_KCNQ2(ENSXETP_49801)
MVQKSRNGGVYPGPTGEKKLKVGFVGLDAGPPDTSRDGALLIDGSDKRSSILIKQRSGVS
GTGKPPKRNAFYRKLQNFLYNVLERPRGWAFIYHAYVLLITWNSLNLVLFSTIKEYESSS
EGALYILEIITIVVFGVEYFVRIWAAGCCCRYRGWRGRLKFARKPFCVIDIMVLIASIAV
LSAGSQGNVFATSALRSLRFLQILRMIRMDRRGGTWKLLGSVVYAHSKELITAWYIGFLC
LILASFLVYLAEKEDNDMFDTYADALWWGLITLTTIGYGDKYPKTWNGRLLAATFTLIGV
SFFALPAGILGSGFALKVQEQHRQKHFEKRRNPAAGLIQCAWRFYATNLSRMDLHSTWQY
YERTVTVPMYSWVSSSQTYGASRLIPPLNQLDLLRNLKSKSGLTFRKEQQAEPPSSQKVS
LKERVFSSPRGVATKGKGSPQSQGMRRSPSADQSLEESPSKVPKSWSFGDRSRTRQAFRI
KGSASRQNSEVSLPEEEVAVPEENRSCNCEFLAEDLTPGLKASIRAVCIMRFLVAKRKFK
ESLRPYDVMDVIEQYSAGHLDMLARIKNLQSRVDQIVGRGGSITDKDRAKGGAETEIPDD
PSMMGRLGKVEKQVLSMEKKLDFLVNIYMQRMGIPPSETEAYFGAKDREPAPPYHSPEDS
RDPVDKSCITKIIRSSSSMGQKNFSAPPAQLTPTTHCPPSTSWQQQQPYHRHSHGSSPVG
DPGSLVRIPPPPSHERSLSAYGVSRMVGVEEGSGKHRDPAALRDSDTSISIPSVDHEELE
RSFSGFSISQSKENLEILNNGAVAGPGPCHKVRPYIAEGESDTDSDLCTPCGPPARSTTG
EGPYGDMGWPSSKK

>Danio rerio_KCNQ2(ENSDARP_122368)
MVKKSANGEVYLPPAGEKKPKVGFVGLDPGAAETSRDGALLIAGSESTKRGSILCRPRSS
ISRGSIAHKRNARYRRLQNFLYNALERPRGWAFIYHAYVFVLVFSCLVLSVFATIKEYKK
SSESALYILEIVTIVVFGVEYIVRIWAAGCCCRYRGWRGRLRFARKPFCIIDIMVLFASV
SVLAAGSQGNVFATSAIRSLRFLQILRMLRMDRRGGTWKLLGSVVYAHSKELITAWYIGF
LCLILASFLVYSVEKDDNAEMFETYADALWWGLVTLTTIGYGDKFPVTWNGRLIAATFSL
IGVAFFALPAGILGSGFALKVQEQHRQKHFEKRRNPAAGLIQAAWRFHATNLSRTDLFST
WDFYAQTVSVPMYSRNTLLSNLWQLIPPVNQLDILRSLKGKSAFRKDSQIDVTPSSEISH
KDTLCGCCPRTNSRKPSVKEKGSPSKSTGGKEARTDGVKESPSKVTKSLSFTDRNKAKHA
FKMKDGASRQNSEEASLPEDLGDDRGYNCEFLPHLSPGLKVTIRSICVMRFLLSKRRFKE
SLRPYDVMDVIEQYSAGHLDMLSRIKNLQIDLIVGPPPPSTPRHKKYVNIHQWPSQTRWL
YYWTALQVDQIVGKGASAGEKDKPKSDTEVPEDPSMMGRLNKMEKEVGAMDLKLNFLVSI
YTAQMGIPRSETEALLGFKIAYPAPPYHSPDEKSEKVPDEDENKGKSPSPINPGPNGTLT
ESQCRPSTSWHQQDEHPLSVPLWNNSRGVSPIGTDDPSLYRLPPPPFHESMDNSRSRRSR
RPVQQQAAVESDTSLSIPSVDHEELDRSFSGFSISQAREEDYVPPVSLGPFGGGGGTLCT
RIRPYLAEGESDTDSDLYTPGAPSPLSFTGEGTFGDRMWPGMK

>Takifugu rubripes_KCNQ2a(ENSTRUP_24561)
MVQKSLNGGVYPAEDGEKKHRVGFVGLDPGAPESSRDGALLIAGPDGAHKRSSILCRRGS
SISTGRPRPTKKNASYRKLQNFLYNALERPRGWAFVYHAYVFLLVFSCLILSVFATIKEF
KNSSESALYILEIVTIVVFGVEYIVRIWSAGCCCRYRGWRGRLKFARKPFCVIDIMVLIA
SVSVLAAGSQGNIFATSAIRSLRFLQILRMLRMDRRGGTWKLLGSVVYAHSKELITAWYI

GFLCLILASFLVYSVEKESNEEFETYADALWWGLITLTTIGYGDKVPKTWNGRMLGAAFS
MIGVAFFALPAGILGSGFALKVQEQHRQKHFEKRRNPAAGLIQGAWRFYATNLSRTDLVC
TWDFYEQTVSVPMYRIDQCQILPPPRFIPPLNQLDLLRNLKGKSFRKESQTEMSPSNENA
HKERLCGCCPRAISRKASLKDKVFPSPSKAPFAKGKAQSPGAEEGVEASPYKVAKSWSFT
DKNRGTKNSFKARGSTSRQNSEEASLPGEEMGDDKSCHCEFLTQELPSSLKVTIRAICIM
KFLVSKRKFKESLRPYDVMDVIEQYSAGHLDMLCRIKNLQTRVDQIVGKSPKADGDPGDD
LSMMGRLVKVEKQVLCMDRKLDFLVNVYVQRMGITHSETEAYFNSKEPYPAPPYHSPDDS
KLEKDNKEQMNSLEQKDPLVGRSGARSVGSPSASQSRPSTSWQHQLQHPLSQPAWNSSVA
NTPSPVCGDQSPNLFRLPPPPAPAHDRSSGSSSHSRRRRRRQTCQQDGTAAESDTSLSIP
SVDHEELERSFSGFSISQAKEDFFGPSFFAGTAGCPRVRPFIAEGESDTDSDLYAPSPVS
FTGDTSCGERPWPGLK

>Takifugu rubripes_KCNQ2b(ENSTRUP_24560)
MVQKSLNGGVYPAEDGEKKHRVGFVGLDPGAPESSRDGALLIAGPDGAHKRSSILCRRGS
SISTGRPRPTKKNASYRKLQNFLYNALERPRGWAFVYHAYVFLLVFSCLILSVFATIKEF
KNSSESALYILEIVTIVVFGVEYIVRIWSAGCCCRYRGWRGRLKFARKPFCVIDIMVLIA
SVSVLAAGSQGNIFATSAIRSLRFLQILRMLRMDRRGGTWKLLGSVVYAHSKELITAWYI
GFLCLILASFLVYSVEKESNEEFETYADALWWGLITLTTIGYGDKVPKTWNGRMLGAAFS
MIGVAFFALPAGILGSGFALKVQEQHRQKHFEKRRNPAAGLIQGAWRFYATNLSRTDLVC
TWDFYEQTVSVPMYRFIPPLNQLDLLRNLKGKSFRKESQTEMSPSRKASLKDKVFPSPSK
APFAKGKAQSPGAEEGVEASPYKVAKSWSFTDKNRGTKNSFKARGSTSRQNSEEASLPGE
EMGDDKSCHCEFLTQELPSSLKVTIRAICIMKFLVSKRKFKESLRPYDVMDVIEQYSAGH
LDMLCRIKNLQTRIDMIVGPPPPSTPRHKKSTEGLDLSPITSPIYLKFNARVDQIVGKSP
KADGDPGDDLSMMGRLVKVEKQVLCMDRKLDFLVNVYVQRMGITHSETEAYFNSKEPYPA
PPYHSPDDSKLEKDNKEQMNSLEQKDPLVGRSGARSVGSPSASQSRPSTSWQHQLQHPLS
QPAWNSSVANTPSPVCGDQSPNLFRLPPPPAPAHDRSSGSSSHSRRRRRRQTCQQDGTAA
ESDTSLSIPSVDHEELERSFSGFSISQAKEDFFGPSFFAGTAGCPRVRPFIAEGESDTDS
DLYAPSPVSFTGDTSCGERPWPGLK

>Tetraodon nigroviridis_KCNQ2(ENSTNIP_13799)
MVQKSLNGGVYPEEEGEKKHKVGFVGLDPGAPESSRDGALLIAGPDGAHKRSSILCRRGS
GVSTGRPRPSKKNASYRKLQNFLYNALERPRGWAFVYHAYVGLLVLLCDFLDVSATIKEF
KNSSESALYILEIVTIVVFGVEYIVRIWSAGCCCRYRGWRGRLKFARKPFCVIDIMVLIA
SVSVLAAGSQGNIFATSAIRSLRFLQILRMLRMDRRAGTWKLLGSVVYAHSKELITAWYI
GFLCLILASFLVYSVEKESNEEFETYADALWWGLITLTTIGYGDKVPKTWNGRMLGAAFS
MIGVAFFALPAGILGSGFALKVQEQHRQKHFEKRRNPAACLIQGAWRFYATNLSRTDLLC
TWDFYEQTVSVPMYRGFSFSFFVFRFIPPLNQLDLLRNLKGKSFRKESQTELSPSRKASL
KDKVFPSPSKAPFAKGKAPSPGAEDGVETSPYKVAKSWSFNEKNRGAKNSLKARGSSSRQ
NSEEASLPGEEVGDDKSCHCDFLTQELPSSLKVTIRAVCIMKFLVSKRKFKESLRPYDVM
DVIEQYSAGHLDMLCRIKNLQTRIDMIVGPPPPSTPRHKKSTEGPRDGEAGDDLSMMGRL
VKVEKQVLCMDRKLDFLVNVYVQRMGITHSETEAYFNSKEPYPAPPYHSPDESKLEKDPK
EQMNSLEQKDPLLGRSVARSLGSPSASQSRPSTSWQHQLQHPLSQPAWNSSVANTPSPVC
GDRSPNLFRLPPPPAPAHDRSSGSSSQSRRRRRRQTCQQAESDTSLSIPSVDHEELERSF
SGFSISQAKEDFFGASFFAGPPVCPRDRPFIAEGESDTDSDLYAPSPVSFTGDIFCGEKP
WPGLK

>Gasterosteus aculeatus_KCNQ2(ENSGACP_6133)
FLLVFSCLILSVFATIREFKNSSESALYILEIVTIVVFGVEYIVRIWAAGCCCRYRGWRG
RLKFARKPFCVIDIMVLIASVSVLAAGSQGNVFATSAIRSLRFLQILRMLRMDRRGGTWK
LLGSVVYAHSKELITAWYIGFLCLILASFLVYSVEKESNDEFETYADALWWGLITLTTIG
YGDKVPKTWNGRLLAATFSMIGVAFFALPAGILGSGFALKVQEQHRQKHFEKRRNPAAGL
IQATWRFYATNLSRTDLLYTWDFYEQTVAVPMYRLIPPLNQLDLLRNLKGKTFRKESQTE
TSPSRKASLKDKVFPSPSKAPLAKGKSQSPDGAEASPGKVDKSWSFADKNRGPKHSFRAR
GSTSRQNSEEASLPGEEIGDDKSCHCEFLSQELPPGLRVTIRAICVMKFLVSKRRFKESL
RPYDVMDVIEQYSAGHLDMLSRIKNLQSRQDRYDCGAPAPSTPRHKKSTEGPSIMLFTPQ
PKYRSKVDQIVGKGPKSEGDSSDDQSMMGRLGKVEKQVICMDRKLDFLVHVYVQRMGIPR
SETEAFFHSKEPYPAPPYHSPEESKLQTCSVPAIPCSDGSLEKKDPLLGRCVPSHSRPST
SWQHQLQHPLSQPAWNSSVANTPSPVAGSADHSPTLFRLPPPPAPVHDRPSRGNGRESGS
RSRRRHRRRQRDAVALESDTSLSIPSVDHEELERSFSGFSISQAKEDFFGPSFFAGSGGG
AGTEAAAGPALCARVRPFIAEGESDTDSDLYAPSPLSFTGEVSCGDPAWPGRK

>Oryzias latipes_KCNQ2(ENSORLP_1054)
MVQKPLNGGVYPAQAEEKKHKVGFVGLDPGAPESSRDGALLIAGSESSKRGSILGRPRSS
VSTGRPRPSKKNASYRKLQNFLYNALERPRGWAFIYHAYVFLLVFSCLILSVFATIREFK
DSSENALYILEIVTIVVFGVEYIVRIWAAGCCCRYRGWRGRLKFARKPFCVIDIMVLIAS
VSVLAAGSQGNVFATSAIRSLRFLQILRMLRMDRRGGTWKLLGSVVYAHSKELITAWYIG
FLCLILASFLVYSVEKESNEEFETYADALWWGLITLTTIGYGDKVPKTWNGRLLGAAFSM
IGVAFFALPAGILGSGFALKVQEQHRQKHFEKRRNPAAGFIQAAWRFYATNLTRTDLLCT
WEFYEQTVALPMYRIIPPLNQMDLLRNLKGKSFRKESQTETSPRFLLSRKPSLKEKVFPS
PSKAPMGKGAEDGAEASPSKPWGFTDKNRGPKHAFRVRSGTGRQNSEEASLPGEEIGDDK
SCNCEFLAQELPPGLKVTIRAICVMKFLVSKRRFKESLRPYDVMDVIEQYSAGHLDMLCR
IKNLQSRIDMIVGPPPPTTPRHKKSTEGPRLDGRETPNSCCRVDQIVGKSPKVDGEVGDD
QSMMGRLVKVEKQVVCMDRKLDFLVNVYAQRMGIAHSETEAFFNSKEPYPAPPYQSLLDI
KEEKNGKEEVKELVKVKTYSAPDVPCSDGPLESNSRPSTSWKHQLQHPLSQPAWGSSMAN
TPSPVGLSGDRSPNPFRLPPPPAPSHDWSSSGHGGGSSRDNASRSRRRHRRQRCQQDAAS
AAAVESDTSLSIPSVDHEELERSFSGFSISQAKEDFFGPPYFTGSGGGGAAGTEAGTGHS
LCARVRPFIAEGESDTDSDLYAPSPLSFTGEVPCGEKGWPGLK

>Homo sapiens_KCNQ5(ENSP_345055)
MPRHHAGGEEGGAAGLWVKSGAAAAAAGGGRLGSGMKDVESGRGRVLLNSAAARGDGLLL
LGTRAATLGGGGGGLRESRRGKQGARMSLLGKPLSYTSSQSCRRNVKYRRVQNYLYNVLE
RPRGWAFIYHAFVFLLVFGCLILSVFSTIPEHTKLASSCLLILEFVMIVVFGLEFIIRIW
SAGCCCRYRGWQGRLRFARKPFCVIDTIVLIASIAVVSAKTQGNIFATSALRSLRFLQIL
RMVRMDRRGGTWKLLGSVVYAHSKELITAWYIGFLVLIFSSFLVYLVEKDANKEFSTYAD
ALWWGTITLTTIGYGDKTPLTWLGRLLSAGFALLGISFFALPAGILGSGFALKVQEQHRQ
KHFEKRRNPAANLIQCVWRSYAADEKSVSIATWKPHLKALHTCSPTKKEQGEASSSKFCS
NKQKLFRMYTSRKQSQKLSFKERVRMASPRGQSIKSRQASVGDRRSPSTDITAEGSPTKV
QKSWSFNDRTRFRPSLRLKSSQPKPVIDADTALGTDDVYDEKGCQCDVSVEDLTPPLKTV
IRAIRIMKFHVAKRKFKETLRPYDVKDVIEQYSAGHLDMLCRIKSLQTRVDQILGKGQIT
SDKKSREKITAEHETTDDLSMLGRVVKVEKQVQSIESKLDCLLDIYQQVLRKGSASALAL
ASFQIPPFECEQTSDYQSPVDSKDLSGSAQNSGCLSRSTSANISRGLQFILTPNEFSAQT
FYALSPTMHSQATQVPISQSDGSAVAATNTIANQINTAPKPAAPTTLQIPPPLPAIKHLP
RPETLHPNPAGLQESISDVTTCLVASKENVQVAQSNLTKDRSMRKSFDMGGETLLSVCPM
VPKDLGKSLSVQNLIRSTEELNIQLSGSESSGSRGSQDFYPKWRESKLFITDEEVGPEET
ETDTFDAAPQPAREAAFASDSLRTGRSRSSQSICKAGESTDALSLPHVKLK

>Gorilla gorilla_KCNQ5(ENSGGOP_11126)
YSFLLVFGCLILSVFSTIPEHTKLASSCLLILEFVMIVVFGLEFIIRIWSAGCCCRYRGW
QGRLRFARKPFCVIDTIVLIASIAVVSAKTQGNIFATSALRSLRFLQILRMVRMDRRGGT
WKLLGSVVYAHSKELITAWYIGFLVLIFSSFLVYLVEKDANKEFSTYADALWWGTITLTT
IGYGDKTPLTWLGRLLSAGFALLGISFFALPAGILGSGFALKVQEQHRQKHFEKRRNPAA
NLIQCVWRSYAADEKSVSIATWKPHLKALHTCSPTKKEQGEASSSKFCSNKQKLFRMYTS
RKQSQKLSFKERVRMASPRGQSIKSRQASVGDRRSPSTDITAEGSPTKVQKSWSFNDRTR
FRPSLRLKSSQPKPVIDADTALGTDDVYDEKGCQCDVSVEDLTPPLKTVIRAIRIMKFHV
AKRKFKETLRPYDVKDVIEQYSAGHLDMLCRIKSLQTRVDQILGKGQITSDKKSREKITA
EHETTDDLSMLGRVVKVEKQVQSIESKLDCLLDIYQQVLRKGSASALALASFQIPPFECE
QTSDYQSPVDSKDLSGSAQNSGCLSRSTSANISRGLQFILTPNEFSAQTFYALSPTMHSQ
ATQVPISQSDGSAVAATNTIANQINTAPKPAAPTTLQIPPPLPVIKHLPRPETLHPNPAG
LQESISDVTTCLVASKENVQVAQSNLTKDRSMRKSFDMGGETLLSVCPMVPKDLGKSLSV
QNLIRSTEELNIQLSGSESSGSRGSQDFYPKWRESKLFITDEEVGPEETETDTFDAAPQP
AREAAFASDSLRTGRSRSSQSICKAGESTDALSLPHVKLK

>Mus musculus_KCNQ5(ENSMUSP_110955)
MPRHHAGGEEGGAAGLWVRSGAAAAAGAGGGRPGSGMKDVESGRGRVLLNSAAARGDGLL
LLGTRAAALGGGGGGLRESRRGKQGARMSLLGKPLSYTSSQSCRRNVKYRRVQNYLYNVL
ERPRGWAFVYHAFVFLLVFGCLILSVFSTIPEHTKLASSCLLILEFVMIVVFGLEFIIRI
WSAGCCCRYRGWQGRLRFARKPFCVIDTIVLIASIAVVSAKTQGNIFATSALRSLRFLQI
LRMVRMDRRGGTWKLLGSVVYAHSKELITAWYIGFLVLIFSSFLVYLVEKDANKEFSTYA
DALWWGTITLTTIGYGDKTPLTWLGRLLSAGFALLGISFFALPAGILGSGFALKVQEQHR
QKHFEKRRNPAANLIQCVWRSYAADEKSVSIATWKPHLKALHTCSPTKKEQGEASSSKFC
SNKQKFFRVYTSRKQSQKLSFKERVRMASPRGQSIKSRQASVGDRRSPSTDITAEGSPTK
VQKSWSFNDRTRFRPSLRLKSSQPKPVIDADTALGIDDVYDEKGCQCDVSVEDLTPPLKT

VIRAIRIMKFHVAKRKFKETLRPYDVKDVIEQYSAGHLDMLCRIKSLQTRVDQILGKGQM
TSDKKSREKITAEHETTDDPSMLARVVKVEKQVQSIESKLDCLLDIYQQVLRKGSASALT
LASFQIPPFECEQTSDYQSPVDSKDLSGSAQNSGCLTRSASANISRGLQFILTPNEFSAQ
TFYALSPTMHSQATQVPMSQNDGSSVVATNNIANQISAAPKPAAPTTLQIPPPLSAIKHL
SRPEPLLSNPTGLQESISDVTTCLVASKESVQFAQSNLTKDRSLRKSFDMGGETLLSVRP
MVPKDLGKSLSVQNLIRSTEELNLQFSGSESSGSRGSQDFYPKWRESKLFITDEEVGAEE
TETDTFDGTPPPAGEAAFSSDSLRTGRSRSSQNICKTGDSTDALSLPHVKLN

>Bos taurus_KCNQ5(ENSBTAP_10251)
CLMFQEFVMIVVFGLEFIIRIWSAGCCCRYRGWQGRLRFARKPFCVIDTIVLIASIAVVS
AKTQGNIFATSALRSLRFLQILRMVRMDRRGGTWKLLGSVVYAHSKELITAWYIGFLVLI
FSSFLVYLVEKDANKEFSTYADALWWGTITLTTIGYGDKTPLTWLGRLLSAGFALLGISF
FALPAGILGSGFALKVQEQHRQKHFEKRRNPAANLIQCVWRSYAADEKSVSVATWKPHLK
ALHTCSPTKKEQGEASSSQKLSFKERVRMASPRGQSVKSRQASLGDRRSPSTDITAEGSP
TKVQKSWSFNDRTRFRPSLRLKSSQPKPVVDADTALGTDDVYDDKGCQCDVSVEDLTPPL
KTVIRAIRIMKFHVAKRKFKETLRPYDVKDVIEQYSAGHLDMLCRIKSLQTRVDQILGKG
QITSDKKSREKITAEHEATDDLSMLGRVVKVEKQVQSIEAKLDCLLDIYQQVLRKGSAPA
LALAPFPSPPFECEQTSDYQSPGDGRDLSGPAPGGGLPRSATRERALRQSLDLGGAALLS
VRPAVPQDPGRSVSAQNLARSTEDLDVQLSGSESSGSRGSQEFYPKWRESKLFISDEEAA
SPEDTGTDALDAVPPAREPAPAPDSLRTGRSRSSQSSCPAAGGADALGRPHVRLK

>Gallus gallus_KCNQ5(ENSGALP_49052)
MSLLGKPLSYSSGPSCRRNAKYRRLQNYLYNVLERPRGWAFVYHAFVFLLVFGCLILSVF
STIPEHTKLASGCLFILEFVMIVVFGLEFIIRIWSAGCCCRYRGWQGRLRFARKPFCVID
IIVLIASIAVVSAKTQGNIFATSALRSLRFLQILRMVRMDRRGGTWKLLGSVVYAHSKEL
ITAWYIGFLVLIFSSFLVYLVEKDANNQFSTYADALWWGTITLTTIGYGDKTPLTWLGRL
LSAGFALLGISFFALPAGILGSGFALKVQEQHRQKHFEKRRNPAASLIQCVWRSYAADEK
SVSIATWKPHLKALHTCSPTKKEQGEASSSKLCSNKQKLFRMYTPRKHSQKLSFKERVRM
ASPRGQSIKNRQSSVGDRRSPSADIATDGSPTKVQKSWSFNDRTRFRPSLRLKSSQPKPV
VDADTSLGTDDVYDDKGCQCDVSVEDLTPALKTVIRAIRIMKFHVAKRKFKETLRPYDVK
DVIEQYSAGHLDMLCRIKSLQSRVDQILGKGQITADKRSREKNTAENETTDDLSMLGRVV
KVEKQVQSIESKLDCLLDIYQQVLRKGSASALTLASFQMPAFECEQTSDYQSPSDSKDLS
NSAQISGCVSRSASANLPRGLQLILTPNEFSTQAYYAISPAMHSQATQVPNDGHTTVNNT
SNQINQATKPATPTTLQIPPFSSVKHINRPEPVHIIPVTTQENISDVTACLVGSKDNGQV
AQPSATKDLTWRKGLDTEGEPLLSVKPVVQMDLGKSLSIQNLIQSTEELNVQITGSDSSG
SRGSQDVYSKWRESKLFITDEELETEAGTETTEAISRPLVETTLSTDSLRTGISRSSQSI
CKPGDGTEALSLLHVKLK

>Taeniopygia guttata_KCNQ5(ENSTGUP_13065)
FSFLLVFGCLILSVFSTIPEHTKLASGCLFILEFVMIVVFGLEFIIRIWSAGCCCRYRGW
QGRLRFARKPFCVIDIIVLIASIAVVSAKTQGNIFATSALRSLRFLQILRMVRMDRRGGT
WKLLGSVVYAHSKELITAWYIGFLVLIFSSFLVYLVEKDANTEFSTYADALWWGTITLTT
IGYGDKTPLTWLGRLLSAGFALLGISFFALPAGILGSGFALKVQEQHRQKHFEKRRNPAA
SLIQCVWRSYAADEKSVSIATWKPHLKALHTCSPTKKEQGESSSSQKLSFKERVRMASPR
GQSIKSRQSSVGDRRSPSADIATEGSPTKVQKSWSFNDRTRFRPSLRLKSSQPKPMVDAD
TSLGTDDVYDDKGCQCDVSVEDLTPALKTVIRAIRIMKFHVAKRKFKETLRPYDVKDVIE
QYSAGHLDMLCRIKSLQSRVDQILGKGQITADKRSREKNPAEHETADDVSMLGRVVKVEK
QVQSIESKLDCLLDIYQQVLRKGSASALTLASLQMPAFECEQTSDYQSPSDSKDLSNSAQ
LSGCVSRSASANLPRGLQLILTPNEFSTQAYYAFSPAMHSQATQVPNDGPATVNNTSNQI
NQTTKPATPTTLQIPPFSSMKHINRPEPIHIIPVTTQENISNVTACLVASKDNGQVAQPS
ATKDLAWRKSLDTEGEPLLSVKPVVQMDLGKSLSVQNLIQSTEELNIQIAGSDSSSSRGS
QDVYSKWRESKLFITDEELETESGTETTEAISRPLVETTLSADSLRTGISRSSQNICKPG
DGTEALNLLHVKLK

>Danio rerio_KCNQ5a(ENSDARP_118905)
MPRNHSGDEGGAGLWMKTSQHYGMKDVEAGRGTMNNATRNADSLLSAPGTTGAGGSENQR
RNQGARLSLLGKPLAYSAQSGRRNARYRKLQNYLYNVLERPRAWAFVYHAFVFTLVFGCL
VLSVFSTIPAHMDLSNHCLLILEFVMIVVFGLEYIIRIWSAGCCCRYRGWQGRLRFARKP
FCVIDIIVLIASIAVVSAGSQGNIFATSALRSLRFLQILRMVRMDRRGGTWKLLGSVVYA
HSKELVTAWYIGFLVLIFSSFLVYLVEKEFNKQFATYADALWWGTITLTTIGYGDKTPQT
WTGRLLSAGFALLGISFFALPAGILGSGFALKVQEQHRQKHFEKRRNPAASLIQCVWRSY

AADENSVSIATWKPHLKALHTCSPTNQKLSFRERVRMASPRGQSVKSRQMSVNDRRSPGT
EVAVEGSSPAKVQKSWSFNDRTRFRPSLRLKSQSRTTTEADTTLGPDDAFDDKACHCDVT
VEDLSAPLKAVIRAIRIMKFHVAKKKFKETLRPYDVKDVIEQYSAGHLDMLCRIKSLQTR
VDQILGKGQISVDRKGREKILPEGESLEHDMSMLGRVCKVERQVQSIESKLDSLLDIYRQ
VLQKGSSSMLGLSALPLFELDQTSDYQSSLHSKESSSQLTGSGVSRSCSSNLHRGLHLAL
APSELNLGTGYPHSASSFSPSPLLNNQPSSPDSFYPSSPPPILTPNNLSRSHQRFSELTR
PVPTVHSTSTTLQLPPMVPAPPGRPTSLIPETLQESRAECPTNCLLGTQNEVESDKEAES
IPSTVQLRDKPERNSKEDGSWRRHLSLDIDPLMLMSSTAAASLQVERGLGKSLSAQNLML
PTAADCHPALSTRSSGSSNNESSDREPLTDWGETELFISDKEMDFLHQQSNDTNFSSELL
RTGASGTSPSQAGPRDVLESHNLPHVCLE

>Danio rerio_KCNQ5b(ENSDARP_79805)
MPRNHSGDEAGSGLWMNTSPGHHAESYGLHNVECDNRMKNNCSRPGDGLLSASHAGTGAS
GTERDRGKQGARLSLLGKPLVYGTQSGRRNARYRRIQNYLYNVLERPRSWAFIYHAFVFV
LVFGCLVLSVFSTIPDHQEMASQSLLILEFVMIVVFGLEYIIRIWSAGCCCRYRGWQGRL
RFARKPFCVIDIIVLIASVAVVSAGSQSNIFATSALRSLRFLQILRMVRMDRRGGTWKLL
GSVVYAHSKELVTAWYIGFLVLIFSSFLVYLVEKEFNKDFATYADALWWGTITLTTIGYG
DKTPKTWTGRMLSAGFALLGISFFTLPAGILGSGFALKVQEQHRQKHFEKRRNPAACLIQ
CVWRSYAADENSVSVATWKPHLKALHTCSPTKKDQGESATSQKLSFKDRVRMASPRGQSI
KSRQTSVTDRRSPGAEISTDGSSPAKVQKSWSFNDRTRFRPSLRLKSQSRSTPEGEANVT
VDEAFDEKGCHCDMSVEDLPSALKTVIRAVRIMKFHVAKKKFKETLRPYDVKDVIEQYSA
GHLDMLCRIKSLQTRVDQILGRGQMPVDKKVREKLLSDGDILEDVSMLGRVCKVERQVQS
IESKLDSLLDIYRQVLQKGSSTALTFSSLPPFGLEENSDYQSTVLSKDLSCASQVSQSIC
GNHPRALQLILAPNELMNLNQNNSTTSSPGLNPASASPFNLSTFQVPSTPSLECPSAQGS
PSQILNANSFHCSIGHFPSVGQPPPPLSSTTTSKYQLSTVPSQAGHARSMGPTRSDPADY
AKSCFRGVGIDLGLNSSMICKLQQKNRPSAKEDSSWRRHISLDSEVEMDPLAPVSPLAPV
QGQCSGDRGLGKSLSVQDLIQPALEGVGVDTHSSQASFSTSISSSQDSSAGGIEMGAGGW
GEADLFISDRDLDTSTKAHNQGFDFLSQAPIDASYSSELLRTSTAAGASHSLASGHTSNT
GSNETINMPHVRLK

>Takifugu rubripes_KCNQ5a(ENSTRUP_31899)
AGDAMPRSHSGDQSGGPGLWLKTSTGRRGQEYPLKDVECGRTTMNNAVRVGDGVLSAAAA
GALGVDRKQGARLSLLGKPLTYSAQSGRRNARYRRLQNYLYNVLERPREWAFVYHAFVFI
LVFGCLVLSVFSTIPAHQEFSSHCLLILEFVMIVVFGLEYIIRIWSAGCCCRYRGWQGRL
RFARKPFCVIDIIVLIASVAVVSAGSQGNIFATSALRSLRFLQILRMVRMDRRGGTWKLL
GSVVYAHSKELVTAWYIGFLVLIFSSFLVYLVEKEFNKDFATYADALWWGTITLTTIGYG
DKTPQTWTGRLLSAGFALLGISFFALPAGILGSGFALKVQEQHRQKHFEKRRNPAASLIQ
CVWRSYAADENSVSIATWKPHLKALHTCSPAKKEQGETTYSQKLSFKERVRMASPRGQSM
KNRQLSINDRQRCSPGNDVMGGAELMGGSPAKVQKSWSFNDRTRFRPSLRLKSQTRAAVS
DVDAGMSTEDSLDERGCHCDVTLEDLSAPLKAVIRAVRIMKFHVAKKKFKETLRPYDVKD
VIEQYSAGHLDMLCRIKSLQTRVDQILGKGQIPIDKKIRDKLNPDGDSLEGMSMLGRVCK
VERQVSSIESKLDSLLDVYRQVLQKGPSALTLSSLPLFELHNHQRHSSDYQTSILSRDLP
SPQEGDPMGGENNGGINHSLSVNTRGLRLILAPADDDDPPDSAPPSYPPSTASLSPSPLL
PPDSPYPNLATPNISKRAHFPDLPPPPSSGGGFTLQLPPILHPSQPHCPIEPTSNNQGTM
GPSVVVRSPVDGDTEPGSAQEEAQQCEKPGLKSEGVWRRHINLEVNPLLPPSSSPDWEEC
LGKSLSAHNLCQPSTNRAQSHSSDLHNSSSSHSSKCVNAHGGLSNWDEAELFISDRKLNR
E

>Takifugu rubripes_KCNQ5b(ENSTRUP_20763)
MPRNHNGDEGGTGLWMRTSQGHRSEGSYGLKDVESGQRMLNNAGDGLLSASAAAGAVGSE
SLRVKQGARLSLLGKPLIYSAQSGRRNVRYRRLQNYLYNVLERPRAWAFVYHAFVFILVF
SCLVLSVFSTIPPHQDFSSYCLLILEFVMIVVFGLEYIIRIWSAGCCCRYRGWPGRLRFA
RKPFCVIDIIVLIASVAVVSAGSQGNIFATSVLRSLRFLQILRMVRMDRRGGTWKLLGSV
VYAHSKELVTAWYIGFLVLIFSSFLVYLVENKFNKEFATYADALWWGTITLTTIGYGDKT
PKTWTGRMLSAGFALLGISFFALPAGILGSGFALKVQEQHRQKHFEKRRNPAAYLIQCVW
RSYAADENSVSIATWKPHLKALHTCSPTKKEQGESTTSQKLSFKERVRMASPRGQTIKNR
QTSSGNDRRSPVTDINTEGTSPAKVQKSWSFNDRTRFRPSLRLKSQSRSTTDATDSNVAG
EDGFDEKGCNCEISVDDLLPAVKSVIRAVRIMKFYVAKKKFKETLRPYDVKDVIEQYSAG
HLDMLCRIKSLQTRVDQILGKGQIPLDKKIREKLLSDGDILEDMSMLGRVCKVERQVQSI
ESKLDSLLDIYRQLLHKGSSSALTLSSLPLFELEQTSDYPSSVFSKDLSCSSQVSGSGAP
HPGGCVSHSSTNSRLSQGGLRLILAPSSDSSST

>Tetraodon nigroviridis_KCNQ5(ENSTNIP_17165)
FRFILVFSCLVLSVFSTIPAHQDFSSDCLLILECVMIVVFGLEYIIRIWSAGCCCRYRGW
QGRLRFARKPFCVIDIIVLIASVAVVSAGSQGNILAASFLRSLRFLQILRMVRMDRRGGT
WKLLGSVVYAHSKELVTAWYIGFLVLIFSSFLVYLVENKGNKEFATYADALWWGTITLTT
IGYGDKTPKTWTGRMLSAGFALLGISFFALPAGILGSGFALKVQEQHRQKHFEKRRNPAA
YLIQAAWRYYSTDSTRPDLDATWRHYESLLPPHSQKLSFKERVRLASPRGQTLKNRQASS
VNDRRSPVTDISAEGTSPAKVQKSWSFNDRTRFRPSLRLKSQSRSTTDAAEDGFDEKGCN
CEISVEDLLPSVKSVIRAVRIMKFYVAKKKFKETLRPYDVKDVIEQYSAGHLDMLCRIKS
LQTSRVDQILGKGQIPLDKKIREKLLSDGDILEDMSMLGRVCKVERQVQSIESKLDSLLD
IYRQMLHKGSSSALTLSSLPLFELEQTSDYHSPVFAKDPSCSSQAPKTVSIEASTNME

>Gasterosteus aculeatus_KCNQ5a(ENSGACP_23908)
FRFVLVFSCLVLSVFSTIPAHQDFSSYCLLILEFVMIVVFGLEYIIRIWSAGCCCRYRGW
QGRLRFARKPFCVIGEILLIASVAVVSAGSQGNIFATSVLRSLRFLQILRMVRMDRRGGT
WKLLGSVVYAHSKELVTAWYIGFLVLIFSSFLVYLVENKFNKDFATYADALWWGTITLTT
IGYGDKTPKTWTGRMLSAGFALLGISFFALPAGILGSGFALKVQEQHRQKHFEKRRNPAA
YLIQCVWRSYAADENSVSIATWKPHLKALHTCSPTKKEAGESTTSQKLSFKERVRMASPR
GQSIKNRQTSSVNDRRSPVAEAGTDGTSPAKVQKSWSFNDRTRFRPSLRLKSQSRSTTDA
ADSNMAGEDSYDERGCHCEMSVQDLLPSVKSVIRALRIMKFHVAKKKFKETLRPYDVKDV
IEQYSAGHLDMLCRIKSLQTRVDQILGRGQIPLDKKIREKLLSDGDILEDMSMFGRVCKV
ERQVQSIESKLDSLLDIYRQVLRKGASSALTLSSLPLFELEQTSDYHSSVFGKDLSSSTQ
ASSNAALPPGSNSHLSQGGLHLILAP

>Gasterosteus aculeatus_KCNQ5b(ENSGACP_23911)
ELVNLVESLIVLPGFSVILYPLLEFVLVFSCLVLSVFSTIPAHQDFSSYCLLILEFVMIV
VFGLEYIIRIWSAGCCCRYRGWQGRLRFARKPFCVIDIIVLIASVAVVSAGSQGNIFATS
VLRSLRFLQILRMVRMDRRGGTWKLLGSVVYAHSKELVTAWYIGFLVLIFSSFLVYLVEN
KFNKDFATYADALWWGTQKPHSDPSGRDDEITLTTIGYGDKTPKTWTGRMLSAGFALLGI
SFFALPAGILGSGFALKVQEQHRQKHFEKRRNPAAYLIQAAWRCYSTDSTRPYLDATWRP
YKSLLTSYSQKLSFKERVRMASPRGQSIKNRQTSSVNDRRSPVAEAGTDGTSPAKVQKSW
SFNDRTRFRPSLRLKSQSRSTTDGPREDSYDERGCHCEMSVQDLLPSVKSVIRALRIMKF
HVAKKKFKETLRPYDVKDVIEQYSAGHLDMLCRIKSLQTRVDQILGRGQIPLDKKIREKL
LSDGDILEDMSMFGRVCKVERQVQSIESKLDSLLDIYRQVLRKGASSALTLSSLPLFELE
QTSDYHSSVFGKDLSSSTQASSNAALPPGSNS

>Oryzias latipes_KCNQ5a(ENSORLP_3087)
KIKMCFGNVISKKRGPPFPPKKVLKFILVFSCLVLSVFSTIPAHQEFSSYCLLILEFVMI
VVFGFEYIIRIWSAGCCCRYRGWQGRLRFARKPFCVIDIIVLIASVAVVSAGSQGNIFAT
SVLRSLRFLQILRMVRMDRRGGTWKLLGSVVYAHSKELVTAWYIGFLVLIFASFLVYLVE
NKFNKDFATYADALWWGTTFRTPCLYLQITLTTIGYGDKTPKTWTGRMLSAGFALLGISF
FALPAGILGSGFALKVQEQHRQKHFEKRRNPAAYLIQCVWRSYSADENSVSIATWKPHLK
AMHTCSPTNQKLSFKERVRMASPRGQSIKNRQTSSMNDRRSPVADGGAEGTSPAKVQKSW
SFNDRTRFRPSLRLKSASRSTTDVLWPGDDGFDDKGCHCDLSVEDLLPSVKAVIRAVRIM
KFHVAKKKFKETLRPYDVKDVIEQYSAGHLDMLCRIKSLQTRVDQILGKGQIPLDKKIRD
KLLSDGDLLEDMSMLGRVCKVERQVQSIESKLDSLLDIYRQVLRKGSSSGLTLSSLPLFE
LEQTSDYNSPVFSKDLSTPSQPITIATSSSSSHA

>Oryzias latipes_KCNQ5b(ENSORLP_2826)
FILVFGCLVLSVFSTIPAHQEFSSHCLLILEFVMIVVFGLEYIFRIWSAGCCCRYRGWQG
RLRFARKPFCVIDIIVLITSVAVVSAGSQGNVFATSALRSLRFLQILRMVRMDRRGGTWK
LLGSVVYAHSKELVTAWYIGFLVLIFSSFLVYLVEKEFNKDFDTYADALWWGTCPLWSSP
IQITLTTIGYGDKTPQTWTGRLLSAGFALLGISFFALPAGILGSGFALKVQEQHRQKHFE
KRRNPAASLIQQIEWRVKVVYQFNTHLLAFWELFELYDGLLLASPSQKLSFKERVRMASP
RGQSMKSRQTTIGDRQRCSPGSEVVGSNELMSGSPAKVQKSWSFNDRTRFRPSLRLKSQL
SGDGGAATEDSFERGCHCDVTVEDLSAPLKAVIRAVRIMKFHVAKKKFKETLRPYDVKDV
IEQYSAGHLDMLCRIKSLQTRVDQILGKGQIPVEKKPRDKLHPDGDSLEGMSMLGRVCKV
ERQFSGVLHRVQTRLLLDIYRQVLQKGSSGLSSLPLFELENHQRHGSDFPLSPHRGDAIR
SRDEGGGMQRSLSASPR

>Homo sapiens_KCNQ1(ENSP_155840)

MAAASSPPRAERKRWGWGRLPGARRGSAGLAKKCPFSLELAEGGPAGGALYAPIAPGAPG
PAPPASPAAPAAPPVASDLGPRPPVSLDPRVSIYSTRRPVLARTHVQGRVYNFLERPTGW
KCFVYHFAVFLIVLVCLIFSVLSTIEQYAALATGTLFWMEIVLVVFFGTEYVVRLWSAGC
RSKYVGLWGRLRFARKPISIIDLIVVVASMVVLCVGSKGQVFATSAIRGIRFLQILRMLH
VDRQGGTWRLLGSVVFIHRQELITTLYIGFLGLIFSSYFVYLAEKDAVNESGRVEFGSYA
DALWWGVVTVTTIGYGDKVPQTWVGKTIASCFSVFAISFFALPAGILGSGFALKVQQKQR
QKHFNRQIPAAASLIQTAWRCYAAENPDSSTWKIYIRKAPRSHTLLSPSPKPKKSVVVKK
KKFKLDKDNGVTPGEKMLTVPHITCDPPEERRLDHFSVDGYDSSVRKSPTLLEVSMPHFM
RTNSFAEDLDLEGETLLTPITHISQLREHHRATIKVIRRMQYFVAKKKFQQARKPYDVRD
VIEQYSQGHLNLMVRIKELQRRLDQSIGKPSLFISVSEKSKDRGSNTIGARLNRVEDKVT
QLDQRLALITDMLHQLLSLHGGSTPGSGGPPREGGAHITQPCGSGGSVDPELFLPSNTLP
TYEQLTVPRRGPDEGS

>Callithrix jacchus_KCNQ1(ENSCJAP_21515)
MAAASSPPRAERKRWGWGRLPGARRGSAGLAKKCPFSLELAEGGPAGGALYAPIAPGAPG
PAPPASPAAAPTVPSDLGPRPPVSLDPRVSIYSTRRPLLARTHVQGRVYNFLERPRGCKC
FIYHFAVFLIVLVCLIFSVLSTIEQYAALATGTLFWMEIVLVVFFGTEYMVRLWSAGCRS
KYVGLWGRLRFARKPISIIDLIVVVASMVVLCVGSKGQVFATSAIRGIRFLQILRMLHVD
RQGGTWRLLGSVVFIHRQELITTLYIGFLGLIFSSYFVYLAEKDAVNESGHVEFGSYADA
LWWGVVTVTTIGYGDKVPQTWVGKTIASCFSVFAISFFALPAGILGSGFALKVQQKQRQK
HFNRQIPAAASLIQTAWRCYAAENPDSSTWKIYVRKPPRSHALLSPSPKPKKSVVIKKKK
LKLDKDNGVSPGEKTLTVPQITCDPPEERRLDHFSVDSYDSSVRKSPTLLEVSMPHFMRT
NSFAEDLDLEGETLLTPITHISQLREHHRATIKVIRRMQYFVAKKKFQQARKPYDVRDVI
EQYSQGHLNLMVRIKELQRRLDQSIGKPSLFIAVSEKSKDRGSNTIGARLNRIEDKVTQL
DQRLVLITDMLHQLLSLHRGGPPGGSSPPREGGAHITPPCGSGGSIDPKLFLPSSALPTY
EQLTVPRRGPDEGS

>Mus musculus_KCNQ1(ENSMUSP_9689)
MDTASSPPSAERKRAGWSRLLGARRGSAVVKKCPFSLELAEGGPEGSTVYAPIAPTGAPG
LAPPMSTPVSPAPAPADLGPRPRVSLDPRVSIYSARRPLLARTHIQGRVYNFLERPTGWK
CFVYHFTVFLIVLVCLIFSVLSTIEQYAALATGTLFWMEIVLVVFFGTEYVVRLWSAGCR
SKYVGIWGRLRFARKPISIIDLIVVVASMVVLCVGSKGQVFATSAIRGIRFLQILRMLHV
DRQGGTWRLLGSVVFIHRQELITTLYIGFLGLIFSSYFVYLAEKDAVNESGRIEFGSYAD
ALWWGVVTVTTIGYGDKVPQTWVGKTIASCFSVFAISFFALPAGILGSGFALKVQQKQRQ
KHFNRQIPAAASLIQTAWRCYAAENPDSATWKIYVRKPARSHTLLSPSPKPKKSVMVKKK
KFKLDKDNGMSPGEKMFNVPHITYDPPEDRRPDHFSIDGYDSSVRKSPTLLEVSTPHFLR
TNSFAEDLDLEGETLLTPITHVSQLRDHHRATIKVIRRMQYFVAKKKFQQARKPYDVRDV
IEQYSQGHLNLMVRIKELQRRLDQSIGKPSLFIPISEKSKDRGSNTIGARLNRVEDKVTQ
LDQRLVIITDMLHQLLSMQQGGPTCNSRSQVVASNEGGSINPELFLPSNSLPTYEQLTVP
QTGPDEGS

>Rattus norvegicus_KCNQ1(ENSRNOP_27875)
MDTASSPPNAERKRAGWGRLLGARRGSAGLAKKCPFSLELAEGGPTGGTVYAPIAPTGAP
GLAPPMSPPVSPVPAPADLGPRPRVSLDPRVSIYSTRRPLLARTHIQGRVYNFLERPTGW
KCFVYHFTVFLIVLVCLIFSVLSTIEQYAALATGTLFWMEIVLVVFFGTEYVVRLWSAGC
RSKYVGIWGRLRFARKPISIIDLIVVVASMVVLCVGSKGQVFATSAIRGIRFLQILRMLH
VDRQGGTWRLLGSVVFIHRQELITTLYIGFLGLIFSSYFVYLAEKDAVNESGRIEFGSYA
DALWWGVVTVTTIGYGDKVPQTWVGKTIASCFSVFAISFFALPAGILGSGFALKVQQKQR
QKHFNRQIPAAASLIQTAWRCYAAENPDSSTWKIYVRKPARSHTLLSPSPKPKKSVMVKK
KKFKLDKDNGLSPGEKIFNVPHITCDPPEDRRPDHFSIDGYDSSVRKSPTLLEVSTPHFL
RTNSFAEDLDLEGETLLTPITHVSQLRDHHRATIKVIRRMQYFVAKKKFQQARKPYDVRD
VIEQYSQGHLNLMVRIKELQRRLDQSIGKPSLFIPISEKSKDRGSNTIGARLNRVEDKVT
QLDQRLVIITDMLHQLLSLQQGGPTCNNRSQVVASDEGGSINPELFLPSNSLPTYEQLTV
PQTGPDEGS

>Canis familiaris_KCNQ1(ENSCAFP_42679)
GHRRCPGLRRGSAGGAFRCALRPHQGQSRTLGGEGLHPRGPGAPGPGLPRPQAAPASPPD
LLGPFLPLPVTPNARPAAGPGVCPRPRRSYPSVRCWNFRVRLPTDRRVLPPHSFLIVLVC
LIFSVLSTIEQYVALATGTLFWMEIVLVVFFGTEYVVRLWSAGCRSKYVGIWGRLRFARK
PISIIDLIVVLASMVVLCVGSKGQVFATSAIRGIRFLQILRMLHVDRQGGTWRLLGSVVF
IHRQELITTLYIGFLGLIFSSYFVYLAEKDAVNDSGQVEFGSYADALWWGVVTVTTIGYG

DKVPQTWVGKTIASCFSVFAISFFALPAGILGSGFALKVQQKQRQKHFNRQIPAAASLIQ
TAWRCYAAENPDSSTWKIYVRKPARSHALLSPSPKPKKSVMVKRKKFKQDKDNGVSPGEK
MLTVPHITCEPVSEERRPDHFSVDNCDSSVKKSPMLLEVSTAHFMRTNSFAEDLDLEGET
LLAPITHVSQLREHHRATIKVIRRMQYFVAKKKFQQARKPYDVRDVIEQYSQGHLNLMVR
IKELQRRLDQSIGKPSLFISVSEKSKDRGSNTIGARLNRVEDKVTQLDQRLVLITDMLHQ
LLSLYHGGPPGGRPPSGSGVQVVQPCSSINPELFLPSNTLPTYEQLTVPRRGPEEGS

>Xenopus tropicalis_KCNQ1(ENSXETP_43172)
IFQLLKKPLSWKPFVYYTPKFLIVLICLIFSVLSTIQQYNNLATETLFWMEIVLVVFFGA
EYVVRLWSAGCRSKYVGIWGRLRFARKPISVIDLIVVVASIIVLCVGSNGQVFATSAIRG
IRFLQILRMLHVDRQGGTWRLLGSVVFIHRQELITTLYIGFLGLIFSSYFVYLAEKDAID
SSGEYQFGSYADALWWGVVTVTTIGYGDKVPQTWIGKTIASCFSVFAISFFALPAGILGS
GFALKVQQKQRQKHFNRQIPAAASLIQTAWRCYAAENPDSATWKIYIRKQSRNHHILSPS
PKPKKSAMVKKKKIRTDREEGSTDKMLNIPNITYDHVADDRKNDGYSVEAYENTVRKPFG
FLDPGTGPFIRTSSFTDDLDMEGDTLLTPITHISELKEHHRAAIKVIRRMQYFVAKKKFQ
QARKPYDVRDVIEQYSQGHLNLMVRIKELQRRLDQSLGKPSLFLSVSDKVKDKGSNTIGS
RLNRVEDKVTQMDHKLNLITDMLHHLVTNQQGTQSIRTPHRSNSLNSENHASRNTLPTYE
QLTVPRPGQDNVS

>Danio rerio_KCNQ1(ENSDARP_77949)
MTSPAHSTATEASWTAGAADLQMTEALHLRNDGVRFPSSVPVDPAVITQHPPSHLHPRMS
VYSATRPTLSRSFLQGRVYNFLERPTGWKCFVYHFTVFLIVLSCLILSVLSTIDEYQALA
NKTLFWMELVLVMFFGVEYVVRLWSAGCRSKYVGILGRLRFARKPISVIDLIVVVASIIV
LAFGSNGQVFATSAVRGVRFLQILRMLHVDRQGGTWRLLGSVVFIHRQELITTLYIGFLG
LIFSSYFVYLAEKDAVDDHGNSGFGSYADALWWGVVTVTTIGYGDKVPQTWIGKTIASCF
SVFAISFFALPAGILGSGFALKVQQKQRQKHFNRQIPAAASLIQASWRCFALLNPDSATY
KLFVKRNLSSSGSSPKLKVKMRRKMKISDRNNGQNSPAVPSITYDSFDDGRDSRQETLTS
LQQSVISDGTNTTRRYERAPSWNSLSSFQFSTPPATKPGVFEVSSRPTLQRSSSIADDME
TEPEREIVLIPVAHVSQLRDSHRAAIRVIQRMYYFVARKKFQQARKPYDVRDVIEQYSQG
HLNLMVRIKELQRRLDHSLGKQSLFQTSSERLKDKGTNTIGSRLNRMDEKITHMDRTLNS
IAESLNLMLARERRGDLARGKEQRSTMRRQSATFSLSVLDSSEQLSTTTAIHEDS

>Takifugu rubripes_KCNQ1(ENSTRUP_41806)
KPPLDLQLSEIGPGAPDVEPPARNDGRKMPADATANSEVPPAYLHPYSAHPRMSFRMSVY
STGVRPVLTRAYIQGRVYNFLERPSGWKCLLYHFTVFLIVLACLILSVLSTIDQYQSLSQ
TTLFWVEIVLVVFFGMEYFIRLWSAGCRSKYVGVWGRLRFARKPISIIDLIVVVASVIVL
SVGSKGQVFATSAVRGIRFLQILRMLHVDRQGGTWRLLGSVVYIHRQELITTLYIGFLSL
IFSSYFVYLAETYAISGGSTEFRNYADALWWGVVTVTTIGYGDKVPQTWIGKTIASCFSV
FAISFFALPAGILGSGFALKVQQKQRQKHFNRQIPAAAGLIQTSWRCFAVENLDSATYKM
FLRKRSNITASSLSSPKSKKSVKIKRKPKSADQDNGPTSPTVPSITYDSVFDSGRELSSD
VYSTTVRRSPGLLDVHGHDPLRRNASFANDTELESEHHNHLFPAASLSQLTESHRKAIRV
IQRMRYLVAKRNFQQARKPYDVRDVIEQYSQGHLNLMVRIKELQRRLDQSLGKMTLFQAG
SERAKDRGNNSIGSRLNRMEDKISHMDQTLGRISESLAVLLEQK

>Tetraodon nigroviridis_KCNQ1(ENSTNIP_12469)
PRMSFRMSVYSTGVRPVLTRAYIQGRAYNFLERPSGWKCLVYHFSVFLIVLACLILSVLS
TIDQYQSLSQTTLFWVEIVLVVFFGMEYFVRLWSAGCRSKYVGIWGRLRFARKPISIIDL
IVVIASVIVLSVGSKGQVFATSAVRGIRFLQILRMLHVDRQGGTWRLLGSVVYIHRQELI
TTLYIGFLSLIFSSYFVYLAETYSVSGGSTDFRNFADALWWGVVTVTTIGYGDKVPQTWI
GKTIASCFSVFAISFFALPAGILGSGFALKVQQKQRQKHFNRQIPAAACLIQTSWRCFAV
ENLDSATFKMFLRKRSNISASSLPSPKSKKSVKIKRKPKSVDKDSGPTSPTVPSITYDSV
FDSGRELSSVPACVQTERRAPGLLDVHSHDALRRNASFADDTELESEPHTHLFPAASLSQ
LTESHRKAIRVIQRMRYFVAKRNFQQARKPYDVRDVIEQYSQGHLNLMVRIKELQRRLDQ
SLGKMTFFQAGSERAKNRGNNSVGSRLNRMEDKITHMEQTLGCIADSLALLLKQRDAGEA
EGGGRLRPQRARHFVPSHDSLPSYDQLSSSPSSFSSNN

>Gasterosteus aculeatus_KCNQ1(ENSGACP_22656)
WCGVCSPRRGGVDRLRTAVAQFGTNNKTSERPATRGAPPDPLPGPRLHIPSTTRERMPED
GNAAATDGPNVYLHPYLSHPRMSVRMSVYSTGIRPVLTRAHIQGQVYNFLERPSGWKCFV
YHFTVFLIVLACLILSVLSTIDQYQALAHTTLFWVEIVLVVFFGVEYFVRLWSAGCRSKY
VGICGRLRFARKPISIIDLIVVVASIIVLSVGSKGQVFATSAVRLKFSDNVTTSHEKDPI

GQWIDLSVLYCVFQELITTLYIGFLSLIFSSYFVYLAEKDAVDSSGYSEFENYADALWWG
VVTVTTIGYGDKVPQTWIGKTIASCFSVFAISFFALPAGILGSGFALKVQQKQRQKHFNR
QIPAAACLIQTSWRCFAVENFDSVTFKKFLKKRAHIPASSLSTLKPKKSVKVKTRRKPRS
SDKDNGPTSPTVPSITYSNVFDSGREFIFLSNIKGFNSTFEASAGLLDVHSPGPLRRNCS
FADDLDAEAERDGELAPVTSLSQLTESHRRAIRVIQRMRYFVARRNFQQARKPYDVRDVI
EQYSQGHLNLMVRIKELQRRLDQSIGRQTLFQSGSDRAKDKGTNSIGSRLNRMEDKITHM
DHTLNRIAESLTSVLHRRDGPEEPRRPPQ

>Branchiostoma floridae_(XP_002588016)
MARTDAAYRPRKMSNISALSDADSPFSVLPRPAQLTVPETTSRMSMSDTETEFLFPPKLSISEAEDDISL
NMGDGAGRQEPPGRCSIGPDTDAETGAKEVTGLLTPEPRRNMGDGAGRQEPPGRCSIGPDTDAETGAKEV
TGLLTPEPRRSVGNCAPAGRRSVKQVEFIGLRQLTVEEEEYQAAVARKQGTVGRYALPRMSLLGKPIGYR
ATRRAARYRHFQMMVYNFLERPAGCKTLAYHIIIPDQPGAGGVTHDWGETPQQDSTRTPCDQEEWSNLLT
GREAVKTSSDWEDLAPTPKTTGAHCWKENIPSKVWFLRGKRKRKARKVQSATSAAPIPDITVTMAGKRVN
WEDSVSQETSNSAETISMEDMVSERVGGESRASPGSPPPGYDTLLAVKGPQAVEDVVSIGSHSSDQVFAD
SGLTDDVHLCNNVKVDVDVPETQLRRRGMLDAEGPGGPPRMTLLGKPIPHVYHSNKNRRTLYIKTQIFNF
FEVPQKQLLSKDSTRTPCDQEEWSNLLTGREAVKTSSDWEDLAPTPKTTGAHCWKENIPSKVWFLRGKRK
RKARKVQSATSAAPIPDITVTMAGKRVNWEDSVSQETSNSAETISMEDMVSERVDRVESRASPGSPPPGY
DTLLAVKGPQAVEDVVSIGSHSSDQVFADSGLTDDVHLCNNVKVDVPETQLRRRGMLDAEGPGGPPRMTL
LGKPIPHVYHSNKNRRTLYIKTQIFNFFEVPQKQLLSKCENGTNATDNSQHNCPKTEEQDKLPNQALYVM
CENGTNATDNSQHNCPKTEEQDKLPNQALYVMEIIIVIVFGMEYILRMWAAGCRSRYQTWRGRLRFARRP
FVIIVLSLGSHGNVFAASALRGLRFLQILRMVRMDRRGGTWKLLGSVVWAHRQELLTTAYIGFLALIFAS
FSLYLIEKDENPEDFGNFAKALYWGVITLLTVGYGDATPDTWLGKLVAVGFSLLGISFFMLPAITMTTVG
YGDATPDSWQGKLLTVVFGLCGISFFALPAGILGSGFALKCLWRCYAGDKNSRSIATWLPHLRAVPDRKD
AAAKKKSFKSQLSRFSTSSPLARPTVSRLQGLEVGDGVDQQFTTQVSAESSGSTGRQMSVADSGKPLSGK
KPAGVRWRETVNAVMTSYESPGSDKSGLRDKYRVRYTVEDMAGYALSEEEYHQPWGLARLSPAHKNAIRA
IRKIKYFVARRKFREALRPYDVKDVIEQYSAGHVDLLARVKHLQSRLDEILGSSSRSPGHAKDKYANDPK
MPLQNRVIRLEKQVHNIDQKLDVLINLMHQKPPDPGSHGDKEIGDHGNKANSDPSVHGDGANTACHSNQI
SLIPDSTRSKDCVELQQAPQSGRTRALPRQSATVISESDPSEGDAAETTSCQQDGKEAQDCVETGEETAG
NGHQTGNGRAKLSSDSDSSLRMLGRETDIV

>Saccoglossus kowalevskii_(XP_006820358)
MAVSVIIIAIGTSGQMFAFSALRGLRFLQILRLVRVDRRGGTWKLLGSVVWAHRQELITTWYIGFLSLSV
LSFLVYNAEMEKNKEDFETFADAMWWGLVTLTTVGYGDKVPVTWVGKLVAAVFAIVGISFFALPAGILGS
GFALKVQQQQRQKHFLRRRHPAAELIQGILGSGFALKVQQQQRQKHFLRRRHPAAQLIQCVWRCYAADPC
SMSVATWKPHLRPVNSPTTNRTNPSLISRLSTKRKIGSPMVRRHNPLNQHDALKKSHSHDLDVGDSLNKS
SLSLHDGNGPLSRNQDSLYSPKNRDDDSIKEDEPVVPEPQTTEWFVTQNIAALKRLLDDDETMGLTRLTE
PHKNAVRAIRKIKYFVARRKFKEAFRPYDVKDVIEQYSAGAAEMQFRMKAIQARLDEILGKTEGKNGIEL
YNTKVPMITRVIKVEKKVNMIDRKLDMLIELYREERQEKMKALQQNKHEHDDQNDSDKDTKDPPSRIRRH
GRARKLSRVKSEGHGAHVDTHEKSERRVEFRKLSDSQPSIPSSLNLDAPDATTVTVSTALSQPSITTTTE
SEQGLQLANAKGVGRGQNIPDYDSAIDDTRV

>Drosophila melanogaster_(FBpp0301793)
MDPDNDIYAFYDIRGYKGKCRPGRPNSERILQPRMSLLGKPLNYNRGTRRDVRYRRLQSR
LYNFLERPRGLHAIFYHVMVFLMVFTCLALSVFSTIKEYEEDAVYILFRMEILVVIWFTM
EFGARLWSSGCRSRYQGCLGRLKFVKRPFCIIDIVTILASIVVLGMGTSGQVFATSALRG
LRFFQILRMVRMDRRGGTWKLLGSVVYAHRQELITTMYIGFLGLIFASFLVYMWEKDVND
KFSNFAQALWWGVITLCTVGYGDMVPITWQGKLIASCCALLGISFFALPAGILGSGFALK
VQQQQRQKHMIRRRQPAATLIQAVWRCYAADEHSVSVATWNIHRVALPSPPASRASSSFK
HNTSFVARLPTIRRHKSQTIQTPGGGDGGGVSKPPGSSRASTRYTRTIRDINASVENLEV
VQNGKSMNPSFSEDSVAETTCLKNIKNSDDEEDEPRCTQLTNRHKTAIRFIRKLKYFVAR
RKFKEALKPYDVKDVMEQYAAGHVDLLGRVKMLHLRLDQILGKQGSKAKDVYASKISLAS
RVVKVERQVADIEEKLDILIKAYMEDRDRFLALPLPAKPKIHSISPSHKPLHHAHNLAMI
DVWKRTAALSVHPEQVTTTPLLNPSAPDSSELRSLTATQTPTTTTDAIATQTPMPPHVQH
TATNTKSSVLNSYQLGSEKQQHNDVFMTELENRTKKRVTLSLHRSTSEPYSKQEQRITIP
DEGADSLDSSAKPTPPDSSIILIDEYEDFEEEDLNCEGEMDHFPTWEIDSDIGVEVDVDA
DADGDCDESTEDTALLQCATRTAIVITPISPVSSAHNLQQLNDQTTTLNKSNLLPPDSG

>Apis mellifera_(XP_006565037)
MKDRPHRPYRDHHGQAMPLEEVQGLLLPPSRTGNRGPLTIVQVGKGNGGGGDGGSDLLKLEPPSDLRPTP
SPLSETSATLQSDNNDTFSGGVDPRLLLGANTGGDRNTWEGRYRVKEHRRAGKATFQGQVYNFLERPTGW

KCFLYHFSVFMVVLVCLIFSVLSTIEQYSNFANETLFWMEICLVVFFGVEYLVRLWSAGCRSKYMGCCGR
LRFIRKPICIIDLIVVVASMVVLSVGSNGQVFATSAIRGIRFLQILRMLHVDRQGGTWRLLGSVVFIHRQ
ELITTLYIGFLGLIFSSYFVYLAEKDVIGPDGKTDFSSYADALWWGVITVTTIGYGDTVPQTWMGKIVAS
CFSVFAISFFALPAGILGSGFALKVQQKQRQKHFNRQIPAAAMLIQCLWRCYAADKAINSVATWNIYIKD
PAPAGQPSTPLGKLICVKKMSLMIQVAKKASVLKRRKSRNRMDVQQQQQSLPPVTISGGMSSGGATGDQ
NDNQRMENEGDVIFYMEEPKAGTPNRARRDNRGSLFTSQTSSVTEATSDEIDIDIEEPQRVTTLTEAHRN
AIRAIRKIKYFVARRKFQQARKPYDVRDVIEQYSQGHLNMMVRIKELQRRLDQTLGKPGSYLAGIDRAGN
VKPMTIGARLYRVEQQLSVMDKKLDQLVYVLNAVAQKQQIPLRSIEDDV

>Hydra magnipapillata_(XP_012559369)
MKHQCEIELNNKEKMSFLNRNTLQKSKTIHKTRMSVFYFLEKPQTIFALLYHKVMFVLVLVSLSLSILIT
IESFARNTKIHSAIYYIEVSLAIVFTAEYSLRVWSCSIQSKYAGVSGRLRFIKRPYVILDMIVILATIFI
IYSHKIIRDDLHTTLFQFLNFLQILRILRLDRQQGAFKIMSLVVYEHRQELMTCWYLSFILLAACSFLVY
IAEKNDSIVSSNKIKNLGEGIYFGMITLLTIGYGDFSPNSWIAKIIVVVFAFIGTAFFALPAGILGSGFA
LKVAQNQKKKHFNRRRVPAALIIQYVWRNYSTQVNPSSNTWTNYRMIIKEKHQTLSMNNTPILSKQNNSN
KNVVGLHKKNQVDRKPFKETTCATIPSAVNFRNRPNSDGNLIVMFPTNIEEHQGTIVSLPASTDVDLKEF
LKSRINVENQRSCTRQLNRIEKIAVRFLIKIKIKVALRKFKVVCRPYDIKDVIEQYTSGQIEMFGFIRKF
QSRIENALGIQDGRLMIPSLDVRMDIFEKKIDTLSVKLDNVMELLNSNSQLT

>Trichoplax adhaerens_(XP_002117304)
ERVAINVSGLRFETYESTLIKHPNTLLGNRARRDRYYDYERKEYFFDRHRQSFEAILYYYQSEGRLNRPS
TVPWDIFYEELCFFQIEPEVLSHLRKVEGMEEDPAPLLPQNKIKRRIWLLFEYPDTSMMAKCVAFFSMAI
ILLSVAVFCLETVDGFNVNFRNKSNVTVEQNRTATRNADALYNVETMCVVWFTFELIIRFLACPSKWKFV
ISPMNIIDFLAIMPYYITLIMRTNGNPNVTTVEALRVLRLARVLRIFKLSRHSSGLQTLGKTFKSSMNEL
FMLGCILVICVILFSSFVYFFEYERNGKEFQSIPHAFWWAVITMTTVGYGDISPRTGLGQIIGSLCAVTG
VLCIALPVPIIVSNFTYYY

>Amphimedon queenslandica_(XP_011404311)
MADRVEHRISQQHSLPNSPLLRKRKKIRRRSRPEVYDQSAPVPASPSLTSLSCSSSDSECESNREEEEDP
LPDKDGQSVTVKQALEGLFYNKAVQLVHHHDCILYVAEAIYIDRLLKSADEEEFILGCFNQTWVNITEPS
YLNLLIIPKDYTLWSLQLFLALICLIGAFTVIYVHQEPYGSVPVLRHVFRYMTHSIVICVLIGLAFFVTL
LMPQCLHILWIPTFLFCWPARHNFMYIVQQKSEVSFFRSFHGFRTTAISLATFLLCFMFSCLCLIHHFER
LGKKISLFDTYWFVVVTFSTVGYGDISPAHWTGKVIITIFIIAALMYLPPKVDKLWRSFTLHRKHYKQYN
KANITKHVVLAAVDLKPLVLRDFLSELYSDPGQMDTQVVVLIKEEPADYIKAILLHPIWSQRVAILRGTA
LRPSDLARVKLRTADACFLHSSRTGRAADADQHTIMSAWTVKNYAPKTRLFVHVRQPESKFQLNIADEVI
CDGELTSAVLALNSVCPGLLTLSALLVHTFTYEGRRFNIPEYKRYEECSPVEIYHVKLGESRYFHRFAGK
NFLFTSVVAYRKFHVLLLGICPSGGTDILFNPGINHIMDRNDECYYISDTHEHNSDHQIIPASTFQSSLW
RTSATLGLLAMYISGIDPDQMFHQTQEYEERDSDNKGGFPRSASNDRLKKLSSPDSIPEIQVEGVASGLS
LTEEVGLPSIGEEEEVLDEQLAVEVTNWQHDVQHGLQLLKYHGEGELPERKPCVKLSVKSHFEGHHSPRS
HSLPVAATTCTVMEPHPLAHPLPSLGVIPEDEPDVIVIGEEEPHLLSHLQRVGDKQQHSSGGGGGKHRVH
SQPALFHFGFNKRQSESKHDDHLLKAKKHSLKLHDNTSMKSYVSSNASVKSDMSNHSIDDVVSLEVEDLS
DIEELEDLEEGRQHETEGGFISEAETESIDVPPIPYCGCEPLKCHLMKNARTYEDIIIDRSFIGEDITRQ
WYHHAIIVYSDVVSDGLFHFITPLRAAYLPPSSLKPIVFLLGNMPERHFISTISQLPMIYFMVGSISSTS
DLLRAGIMVAESLVVFSSNKMRQSDEGEHLADAEHITAIEKIARLFPHVKISCDIKYRFNIRFIRTTPFE
DLCMQRLRHMARKVDHLTYLFLPQFASSKAFSSSMLDTLLYQSYQKPYIIKLFRQLLGCTQVKGSGFLWK
VPVNNELAKLHTYVRVFQRLVASFSFTSMALYRTANVDHNIDPQYVKGYCEEELEKLRLFLISELHSLGI
KRNVEPLKRPSTQQFLYVNPPPDFPVIEGDIILGIRPPLSNKPPPPSDDVKTEPFSPPPASQISELVRAY
SASAFYGGGSPIPTAMPLGPGGVVLQDTRLHFGLMRKFSFTGSLPQQHQTNTGSCSADSCDM

Regulator of G-protein Signalling-RGS

>Homo sapiens_RGS13(ENSP_442837)
MSRRNCWICKMCRDESKRPPSNLTLEEVLQWAQSFENLMATKYGPVVYAAYLKMEHSDEN
IQFWMACETYKKIASRWSRISRAKKLYKIYIQPQSPREINIDSSTRETIIRNIQEPTETC
FEEAQKIVYMHMERDSYPRFLKSEMYQKLLKTMQSNNSF

>Macaca mulatta_RGS13(ENSMMUP_2239)
MSRRNCWICKVFRDESKRPPSNLTLEEVLQWAQSFENLMATKYGPVVYAAYLKMEHSDEN
IQFWMACETYKKIASRWSRTSRAKKLYRIYIQPQSPREINIDNSTRQTIIKNIQEPTETC
FEEAQKIVYMHMERDSYPRFLKSEMYQKLLKTMQSNNSF

>Mus musculus_RGS13(ENSMUSP_107572)
MSRHICWICKLCRDESKRLPSNLTLDEVLKWAQSLESLMATKYGPIVYTAYLKLEHSDEN
IKFWMACETYKKIASRRGRISRAKKLYNIYIQPQSPREINIDSTTREAIIKSIREPTQTC
FEEAQKIVYMHMEMDSYPRFLKSEMYQQLLKTVQSQSS

>Rattus norvegicus_RGS13(ENSRNOP_5163)
MSRHICWICKLCRHESKRLPSNLTLDEVLKWAQSLESLMATKYGPVVYTAYLKLEHSDEN
IKFWMACETYKKIASRRGRISRAKKLYNLYIQPQSPREINIDSTTREAIIKSIREPTQTC
FEEAQKIVYMHMEMDSYPRFLKSEMYQKLLKTVQSKSC

>Homo sapiens_RGS8(ENSP_258302)
MWNTLTRSLSDHPVGKDPQAMRTGQRQNKGMRTRLGCLSHKSDSCSDFTAILPDKPNRAL
KRLSTEEATRWADSFDVLLSHKYGVAAFRAFLKTEFSEENLEFWLACEEFKKTRSTAKLV
SKAHRIFEEFVDVQAPREVNIDFQTREATRKNLQEPSLTCFDQAQGKVHSLMEKDSYPRF
LRSKMYLDLLSQSQRRLS

>Callithrix jacchus_RGS8(ENSCJAP_8703)
MWSALTRSLSDHPVGKDPQAMRTGQRQNKGMRTRLGCLSHKSDSCSDFTAILPDKPNRAL
KRLSTEEATRWADSFDVLLSHKYGVAAFRAFLKTEFSEENLEFWLACEEFKKTRSTAKLV
SKAHRIFEEFVDVQAPREVNIDFQTREATRKNMQEPSLTCFDQAQGKVHSLMEKDSYPRF
LRSKMYLDLLSQSQRRLS

>Mus musculus_RGS8(ENSMUSP_107445)
MRTSQRQNKGMRTRLGCLSHKSDSCSDFTAILPDKPNRALKRLSTEEATRWAESFDVLLS
HKYGVAAFRAFLKTEFSEENLEFWLACEEFKKTRSTAKLVTKAHRIFEEFVDVQAPREVN
IDFQTREATRKNMQEPSLTCFDQAQGKVHSLMEKDSYPRFLRSKMYLDLLSQSQRRLS

>Gallus gallus_RGS8(ENSGALP_42229)
MAALLIPRRNRGMRTRLGCLSHKSDSYNDFTAILPDKPNRALKRLSTEEATRWADSFDVL
LSHKYGLAAFRAFLKTEFSEENLEFWLACEDFKKTRSTAKLAAKAQRIFEEFIDVQAPRE
VNIDFQTRELTRRNMQEPSLSCFDQAQGKVHSLMEKDSYPRFLRSKIYTDLLSQTQRRLS

>Anolis carolinensis_RGS8(ENSACAP_4874)
MKEDAPRCSDQSLCDHTLGKELQSTRTGQRQNRGMRTRLGCLSHKSDSCSDFTAILPDKP
NRALKRLSTEEATRWGDSFDVLLSHKYGVAAFRAFLKTEFSEENLEFWLACEEFKKTRST
AKLASKAQRIFEEFVDVQAPREVNIDFQTREVTRRNIQEPSSSCFDQAQGKIHSLMEKDS
YPRFLRSKIYTDLLSQTQRRLS

>Danio rerio_RGS8(ENSDARP_93238)
MKTRLGCLSNKSDSCSDFSEFLPPAQERTTRYLKLSNDEVTRWADSFDILLSNKYGLAAF
RTFLKTEFSDENIEFWLACEDYKKIKSPAKMMSKANKIYKEFIDVHAPREVNIDHRTREE
TKNRLLEPTPHSLNEVQAKVYSLMEKDSYPRFIRSKIYQDLLNRTQIYCQRKSV

>Takifugu rubripes_RGS8(ENSTRUP_11344)
SCIVWMPERTPTMKTRLGCLSKKSDSYSDFSEFLPPAHDTTARWLNRLSTDEVVRWSESF
DHLLSHKHGLAAFRTFLKSEFSDENIEFWMACEEYKKTKSSTKLVSKANKIYQEFLDIQA
PREVNIDHRTREKTKQSLEDPSPTSLNEVQAKVYSLMERDSYPRFLRSKMYKDLVSRAHA
RGQ

>Tetraodon nigroviridis_RGS8(ENSTNIP_11066)
MHSLYDPKQDPTMKTRLGCLSKKSDSYSDFSEFLPPAHETTARCLNRLSTDEVVRWSESF
EHLLSHKHGLAAFRTFLKSEFSDENIEFWMACEEYKKTKSSTKLVSKANKIYKEFIDTQA
PREVNIDHRTREKTKQSLEDPSPTSLNEVQAKIYSLMERDSYPRFLRSKMYQDMVSRAHA
QGQRRSV

>Gasterosteus aculeatus_RGS8(ENSGACP_7635)
MKTRLGCLSNKSDSYSDFSEFLPPAHETTARCLKVSTDEVVRWSESFDHLLSHKYGLAAF
RTFLKSEFSDENIEFWMACEEYKKIKSSTKLVSKANKIFQEFIDIQAPREVNIDYRTREK
TKQSLEDPSPTSLNEVQGKVYGLMEKDSYPRFLRSKMYQDMVNRANAHGQRRSV

>Oryzias latipes_RGS8(ENSORLP_15410)

LAALLQFGRLSTMKTRLGCLSKKSDSYSDFSEFLPPAHETTARCLNTSHAVTSEQLKQTF
RLSTDEVVRWSDSFEHLLSHKYGLAAFRTFLKSEFSDENIEFWMACEEYKKIKSSTKLLS
KANKIFKEFIEVQSPREVNIDYRTREKTKQSLADPTPTSLNEVQGKIYSLMEKDSYPRFL
RSKMYQDMINRAHAQGQRRSV

>Homo sapiens_RGS1(ENSP_356429)
MRAAAISTPKLDKMPGMFFSANPKELKGTTHSLLDDKMQKRRPKTFGMDMKAYLRSMIPH
LESGMKSSKSKDVLSAAEVMQWSQSLEKLLANQTGQNVFGSFLKSEFSEENIEFWLACED
YKKTESDLLPCKAEEIYKAFVHSDAAKQINIDFRTRESTAKKIKAPTPTCFDEAQKVIYT
LMEKDSYPRFLKSDIYLNLLNDLQANSLK

>Mus musculus_RGS1(ENSMUSP_140624)
MRAAAISMPRLNKMPGMFFSASPKDSKEHSHSLLDDKKQKKRPKTFGMDVKTYLRSMIPH
LESGMKSAKSKDILSAEEVMQWSQSLEKLLANQTGQNVFGRFLKSEFSEENIEFWLACED
YKKTETDLLHNKAENIYKAFVHSDAVKQINIDFHTRESTAKKIKTPTPTSFDEAQKVIYS
LMEKDSYPRFLKSNIYLNLLNDLQANTLK

>Monodelphis domestica_RGS1(ENSMODP_15110)
MRAAAISTPRLNKMPGIFFSSANTNELKGSDHLLLDDKTQKKKPKAFGTDLKAYLKSMLP
HLESGIKSSKSKDILSVTEVIQWSQSLEKLLTNQTGLAVFGNFLKSEFSEENIEFWLACE
DYKRTESDHLHCKAEKIYKAFVQSDATKQINIDFHTRESTAKKIQVPTPTSFDEAQKVIY
TLMERDSYPRFLKSEVYLKLLSELQGNSLK

>Xenopus tropicalis_RGS1(ENSXETP_12507)
AADINVNALYKMPGIFFSHANALKEVDNKPDEVMAQKKKNFAVDLKNYLKSMLPHLETMK
SSSSTSSSDSEKNKLTPDEIIQWTMSLEKLLISEDGQSVFREFLKSEFSEENIEFWLACE
DYKATNDSEELRCKANVIYQEFIQPNANKQINIDFSTRNSVTKDLLEPTITTFNDAQKMI
FILMERDSYPRFLKSEIFLRLAERHHGNNVRG

>Homo sapiens_RGS18(ENSP_356430)
METTLLFFSQINMCESKEKTFFKLIHGSGKEETSKEAKIRAKEKRNRLSLLVQKPEFHED
TRSSRSGHLAKETRVSPEEAVKWGESFDKLLSHRDGLEAFTRFLKTEFSEENIEFWIACE
DFKKSKGPQQIHLKAKAIYEKFIQTDAPKEVNLDFHTKEVITNSITQPTLHSFDAAQSRV
YQLMEQDSYTRFLKSDIYLDLMEGRPQRPTNLRRRSRSFTCNEFQDVQSDVAIWL

>Mus musculus_RGS18(ENSMUSP_27603)
MDMSLVFFSQLNMCESKEKTFFKLMHGSGKEETSIEAKIRAKEKRNRLSLLLQRPDFHGE
TQASRSALLAKETRVSPEEAVKWAESFDKLLSHRDGVDAFTRFLKTEFSEENIEFWVACE
DFKKCKEPQQIILKAKAIYEKFIQNDAPKEVNIDFHTKEVIAKSIAQPTLHSFDTAQSRV
YQLMEHDSYKRFLKSETYLHLIEGRPQRPTNLRRRSRSFTYNDFQDVKSDVAIWL

>Gallus gallus_RGS18(ENSGALP_3996)
MENPLLLFPQLNISASKDKSYYKVMKPTVKEEPNKASKPGAKQKRNRLSLLLQKSELHEA
EHLGKPTNLTKAPSVSPEEAMKWGESFDKLLSQKDGLDAFTRFLKTEFSEENIEFWIACE
DYKKSKTTNELFPKAKTIYEMFIRKDAPKEVNLDFKTKEVTSQNIEQPVITTFDAAQNTV
YRLMEQDSYPRFLRSDLYLNLVKGRNPGRPTLRRRSRSFTVNDFQGVRPDFTIWQ

>Anolis carolinensis_RGS18(ENSACAP_17931)
PDVIKKWGESFYNLISQKAGVDTFTQFLRSEFSEENIEFWMACEDYKKCKDASQLSLKAQ
AIYETFIEKEAPKEVNLDFNTKVCTSENMSHPTLNTFDAAQNKIYALMEQDSYPRFLKSD
LYLDMVHGQPRGHSALRRRSRSFTFNEFKDVQPDFAIWM

>Danio rerio_RGS18(ENSDARP_52418)
MRSEDEQLACRMKETHLRSKDKKGRLSLLLARSGSHENVSPDKKPQAKSNHIPPDVAMKW
SDSFEDLLSHSDGVEVFTRFLRTEFSEENIEFWLACEDFKTTESSTKLQTKAKQIHAIFI
DKEAPKEINIDHSTKAAIEKNILQPTNSCFDAAQHKIYGLMKRDCYPRFLASDIYLTLTK
RSGPPTMTRRRSRSFVFNERPEGTAAWL

>Takifugu rubripes_RGS18(ENSTRUP_25011)
METLLLLFPQFSFTAQKEEVYFRMLGPGATQAAKMKEDSSSHGRQKDKERTSRLSLFLAK
SGSHENVSPHKTTNSTANNVSPELALRWSDSFEELLSHSDGVEVFSQFLRKEFSKENIEF

WLACEEFKTIDSRTKMLSKAKYIYTVFCETDSPKEINIDYNTRMAIQRSMAKPTKGCFEA
AQQQVYNLMKKDSYPRFLLSDTYLNLTRRRGPGATMFRRRSRSCVFNERGEAAAEPSAW

>Tetraodon nigroviridis_RGS18(ENSTNIP_9731)
METLLLLFPQFSYTTQKQEAYCRMLAPGASRALKMKEDSSGQKDKERKSRLSLFLTKSGS
HENVSPHKKKNAPANNVSPESALQWSDSFEELLSHSDGVEVFSQFLRKEFSEENIEFWLA
CEEYKTIDSETKLLSKAKYIYTVFCEPDSPKEINIDYNTRMAIQRGVAKPTKGCFEAAQL
QVYNLMKKDSYPRFLLSDTYLRLTGRRGPRANMFRRRSRSCVFDERGEAAAERSAW

>Gasterosteus aculeatus_RGS18(ENSGACP_21268)
MKDDSSRQKDKERKSRLSLFLTKSGSHENVSPTKKTNTTTNISPETALQWSDSFEELLKH
SDGVESFSQFLRTEFSEENIEFWLACEEYKTIDSDTKLLSKAKCIYTVYIDSDAPKEINI
DYNTKAAIQKVIAKPTKSCFEAAQLIVYSLMKKDSYPRFLHSDIYLRLTRRKGPGTNMFR
RRSRSCVFNDRGEATTEPSAW

>Oryzias latipes_RGS18(ENSORLP_13691)
METLLFLFPQFTYMAPKEEAYFKMLGAEDAQAAKMKDDSSRQNEKERKSRFSLFLTKSGS
HENISPQKKTNTTLSNISPETALQWSDSFEDLLKHPDGVEAFTQFLRTEFSEENIEFWLV
CEEYKSIDSKSKLLSKAKYIYTVFIESEAPKEVNIDYNTKMAIKANMSHPTRSTFEAAQM
KVYSLMKKDCYPRFLHCDIYLRLTRRKGPGATMFRRRSRSCVFNERGEPTQPSAW

>Homo sapiens_RGS16(ENSP_356529)
MCRTLAAFPTTCLERAKEFKTRLGIFLHKSELGCDTGSTGKFEWGSKHSKENRNFSEDVL
GWRESFDLLLSSKNGVAAFHAFLKTEFSEENLEFWLACEEFKKIRSATKLASRAHQIFEE
FICSEAPKEVNIDHETHELTRMNLQTATATCFDAAQGKTRTLMEKDSYPRFLKSPAYRDL
AAQASAASATLSSCSLDEPSHT

>Pan troglodytes_RGS16(ENSPTRP_2932)
MCRTLAAFPTTCLERAKEFKTRLGIFLHKSELGCDTGSTGKFEWGSKHSKENRNFSEDVL
GWRESFDLLLSSKNGVAAFHAFLKTEFSEENLEFWLACEEFKKIRSATKLASRAHQIFEE
FICSEAPKEVNIDHETRELTRMNLQTATATCFDAAQGKTRTLMEKDSYPRFLKSPAYRDL
AAQASAASATLSSCSLAEPSHT

>Mus musculus_RGS16(ENSMUSP_27748)
MCRTLATFPNTCLERAKEFKTRLGIFLHKSELSSDTGGISKFEWASKHNKERSFSEDVLG
WRESFDLLLNSKNGVAAFHAFLKTEFSEENLEFWLACEEFKKIRSATKLASRAHHIFDEY
IRSEAPKEVNIDHETRELTKTNLQAATTSCFDVAQGKTRTLMEKDSYPRFLKSPAYRDLA
AQASATSTSAPSGSPAEPSHT

>Canis familiaris_RGS16(ENSCAFP_41741)
MCRTLAAFPTTCLERAKEFKTRLGIFLHKSELGSDTGNVGKFEWSSKHSKDGRNFSEDVL
GWKESFDSLLSSKNGVAAFHAFLKTEFSEENLEFWLACEEFKKLRSAAKLASRAHKIFEE
FIRSEAPKEVNIDHETRELTEKNLQAATATCFDVAQGKIRTLMEKDSYPRFLKSPAYRDL
AAQASAASASQSGCSPAEPLHT

>Myotis lucifugus_RGS16(ENSMLUP_10119)
MCRTLSAFPTTCLERAKEFKTRLGIFLHKSDLSSDTGNAGKSEWNNKHSKERRNFSEDVL
GWRESFDLLLSSKNGVAAFHAFLKTEFSQENLEFWLACEEFKKIRSASKLASRAHRIFEE
FICSEAPKEVNIDHETRELTRTNLQAATATCFDVAQGKTRTLMEKDSYPRFLKSPAYQDL
AAQASATNASPSSGSSAEPSHT

>Monodelphis domestica_RGS16(ENSMODP_8487)
MCRTLSAFPSTCLERAKEFKTRLGIFLHKSELGTESGNVSKFERGNKHSKEKLVPEDVLV
WKESFNQLLSSQNGVAAFRAFLKTEFSEENLEFWLACEEFKKIQSAAKLATRAHRIFEEF
ICSDAPKEVNIDHETRELTRKNLQEATTTCFDEAQGKTHTLMEKDSYPRFLKSAAYRDLV
EQALAASPSQSVRHVGECSHT

>Gallus gallus_RGS16(ENSGALP_51958)
MSCWMPRCPALSMCRGLAALPIACLERAKELKTRLGILLHKPELRHGIGAAGKLQLGSRR
RDSSREVLEWRESFDQLLKSKNGVTAFHTFLKTEFSEENLDFWLACEDFKKTRSKTKLAS
KANRIFEEFVQSEAPREVNIDHETREITRKNLTGATSACFNEAQAKTRTLMEKDSYPRFL

KSASYQDMTKQATSRSINKRLHT

>Anolis carolinensis_RGS16(ENSACAP_4866)
MCRGLAALPASCLERAKEFKSRLGILLHKPEFGCKFGNMGKATKHRYSLEEVLGWKDSFE
NLLRSKNGVAAFHEFLKTEFSEENLEFWLACEEYKRTRSKGKLSAKANRIFEDFVQSEAP
REVNLDHETREATRRNVIAATSDCFEEAQVKTHTLMEKDSYPRFLKSAYYRDLTEQAMSH
KMSKPVHT

>Homo sapiens_RGS21(ENSP_428343)
MPVKCCFYRSPTAETMTWSENMDTLLANQAGLDAFRIFLKSEFSEENVEFWLACEDFKKT
KNADKIASKAKMIYSEFIEADAPKEINIDFGTRDLISKNIAEPTLKCFDEAQKLIYCLMA
KDSFPRFLKSEIYKKLVNSQQVPNHKKWLPFL

>Pongo abelii_RGS21(ENSPPYP_457)
MPVKCCFYRSPTAETMTWSENMDTLLANQAGLDAFRTFLKSEFSEENVEFWLACEDFKKT
KNAEKIASKAKMIYSEFIEADAPKEINIDFSTRDLISKNIAEPTLKCFDEAQKLIYCLMA
KDSFPRFLKSEIYKKLVNSQQVPNHKKWLPFL

>Bos taurus_RGS21(ENSBTAP_50085)
MVKGRCCFHRSPTTETVAWSENMDTLLTNQAGLDAFRTFLKSEFSEENVEFWLACEDFKK
TESAEKIASKARMIYSEFIEADAPKEINIDFSTRDLISKNIAEPTLKCFDEAQKLIYSLM
ANDSFPRFLKSEIYKKLVNSKEIGSHKKWLPFL

>Monodelphis domestica_RGS21(ENSMODP_40198)
MPVKSTPTNAMAWSESMDTLLANQAGLDAFRTFLKSEFSEENVEFWLACEDYKKTESAEK
IASKAMMIYSEFIQADAPKEINIDFSTRDLISRNISQPTLKCFDEAQRLIYSLMAKDSFP
RFLKSEAYKKLVNSQQDGNHKRWLPFL

>Homo sapiens_RGS4(ENSP_397181)
MYNMMLLIQKRKGIGSQLLRAGEAEGDRGAGTAERSSDWLDGRSWAIKETPTGLAGRRSE
DSDNIFTGEEAKYAQSRSHSSSCRISFLLANSKLLNKMCKGLAGLPASCLRSAKDMKHRL
GFLLQKSDSCEHNSSHNKKDKVVICQRVSQEEVKKWAESLENLISHECGLAAFKAFLKSE
YSEENIDFWISCEEYKKIKSPSKLSPKAKKIYNEFISVQATKEVNLDSCTREETSRNMLE
PTITCFDEAQKKIFNLMEKDSYRRFLKSRFYLDLVNPSSCGAEKQKGAKSSADCASLVPQ
CA

>Canis familiaris_RGS4(ENSCAFP_40445)
MCKGLAGLPASCLRSAKDMKHRLGFLLQKSDSCEHNSSHSKKDRVVVCQRVSQEEVKKWA
ESLENLISHECGLAAFKAFLKSEYSEENIDFWISCEEYKKIKSPSKLSPKAKKIYNEFIS
VQATKEVNLDSCTREETSRNMLEPTITCFDEAQKRIFNLMEKDSYRRFLKSRFYLDLANS
SIAGSEKQKGAKSSADCPSLVPQYA

>Felis catus_RGS4(ENSFCAP_19941)
MCKGLAGLPASCLRSAKDMKHRLGFLLQKSDSCEHNSSHSKKDKVVVCQRVSQEEVKKWA
ESLENLISHECGLAAFKAFLKSEYSEENIDFWISCEEYKKIKSPSKLSPKAKKIYNEFIS
VQATKEVNLDSCTREETSRNMLEPTITCFDEAQKRIFNLMEKDSYRRFLKSRFYLDLADS
SSAGSEKQKGAKSPADCPSLVPQCA

>Gallus gallus_RGS4(ENSGALP_4041)
MCKGLAALPATCLKSAKDMKHRLGVLLQKSESCDYGSSQGKKEKVSPSQRVSQEEVKKWA
ESLENLIHHDRGLAAFRAFLKSEYSEENIEFWVSCEDYKKTKSPAKLSTKARKIYDEFIS
VQATKEVNLDSCTREKTSHNMLEPTLSCFDEAQRKIFTLMEKDSYRRFLKSPYYLDLVSP
PRAGCGPENCKRAHAHALDCNSNIISQCA

>Xenopus tropicalis_RGS4(ENSXETP_22973)
MCKGLAGVTAFPATCLKSAKDIKHRLGFLLQKADGCEHSPCVKKDKPGSQRLRQDEVKKW
AESLENLINNECGLAAFRAFLQSEYSEENIDFWLACENYKKVKTPEKLLEKAQKIYEDFI
SVEATREVNLDSATREETSNNIIQPTHSTFDEAQHRIFILMEKDSYRRFLKSKFYLDLVL
SSSGASTKKMKVLTTDSNPFIPQCA

>Danio rerio_RGS4(ENSDARP_93260)

MCKGLAALPATCLKSAKDIKHKIGFLLQKPDPPQEQKTLKEKEKEKDKEKVKDTVVNRIT
PAETEKWKTSFTNLIKNDDGRKAFASFLQSEYSQENIEFWVACEDFKQTPADKMNLKARN
IFERYIEADSPREVNLDSVTREQTRKNLEMCDVSCFDEAQSKIFTLMEKDSYRRFLRSRL
FLELSQPAMDNKPCGLEKKVKRQISDYSQCLPSYA

>Takifugu rubripes_RGS4(ENSTRUP_13149)
MCKGLATLPATCLKSAKDIKHKISFLLQKHFACQCNNSNKSTQRKIRLCFRVPAADEVKK
WNLSFSHVMNSEMGRQVFASFLRSEFSEENMDFWVACEEYRKMAPSDLAARAQQIYQLYI
ESDAPKEVNLDAVTREETRQNVDQAHPSCFDEAQKMIYILMEKDSYRRFLHSTLVQDL

>Homo sapiens_RGS2(ENSP_235382)
MQSAMFLAVQHDCRPMDKSAGSGHKSEEKREKMKRTLLKDWKTRLSYFLQNSSTPGKPKT
GKKSKQQAFIKPSPEEAQLWSEAFDELLASKYGLAAFRAFLKSEFCEENIEFWLACEDFK
KTKSPQKLSSKARKIYTDFIEKEAPKEINIDFQTKTLIAQNIQEATSGCFTTAQKRVYSL
MENNSYPRFLESEFYQDLCKKPQITTEPHAT

>Gorilla gorilla_RGS2(ENSGGOP_2712)
NSRGSSGRTIMQSAMFLAVQHDCRPMDKSAGSGHKSEEKREKMKRTLLKDWKTRLSYFLQ
NSSTPGKPKTGKKSKQQAFIKPSPEEAQLWSEAFDELLASKYGLAAFRAFLKSEFCEENI
EFWLACEDFKKTKSPQKLSSKARKIYTDFIEKEAPKEINIDFQTKTLIAQNIQEATSGCF
TTAQKRVYSLMENNSYPRFLESEFYQDLCKKPQITTEPHAT

>Equus caballus_RGS2(ENSECAP_9948)
MQSAMFLAVQHDCGPMDKSAGSGPKSEEKREKMKRTLLKDWKTRLSYFLQNSSSPGKPKT
GKKSKQQAFLKPSPEEAELWSEAFDELLASKYGLAAFRAFLKSEFCEENIEFWLACEDFK
KTKSPQKLSSKARKIYTDFIEKEAPKEINIDFQTKTLIAQNIQEVTSGCFTTAQKRVYSL
MENNSYPRFLESEFYQDLCKKPQITTEPHAT

>Pteropus vampyrus_RGS2(ENSPVAP_1109)
MQSAMFLAVQHDCGSMDKSSGSGPKSEEKREKMKRTLLKDWKTRLSYFLQHSSSPGKPKT
GKKSKQQTFIKPSPEEAQLWSEAFDELLASKYGLAAFRAFLKSEFCEENIEFWLACEDFK
KTKSPQKLSSKAKKIYTDFIEKEAPKEINIDFQTKTLIAQNIQEATSGCFTTAQKRVYSL
MENNSYPRFLESEFYQDLCKKPQITSEPHAT

>Taeniopygia guttata_RGS2(ENSTGUP_17249)
ARPGCRARMERGGRRRSEPEEADKGRMKRTIIKDWKTRLSYFLQNSSHPNKRKSKKAGKR
HSYFRPSAEEVQLWSEAFDELLASKYGLAAFRAFLKSEFCEENIEFWLACEDFKKTKSPQ
KLTSKAKKIYNDFIEKEAPKEINIDFQTKNMIAQNIQEATHTCFSVAQKRVYSLMENNSY
PRFLESEFYQELCKKMPINRAAQGT

>Xenopus tropicalis_RGS2(ENSXETP_12515)
SSGTNTNPLRDIMQSAMFLAIQHNCGQVERSSPGGCQKNEEKRGKMKKTILKDWKTRLSY
FLQNSQNTSNGSLNMGQNKRGKKNAYCRPTPEEAKSWSETFDDLLASKYGVAAFRAFLKS
EFSEENIEFWLACEDFKKTKSPHKLTAKAKKIYDEFIEKEAPKEINIDFQTRENIMQTMQ
EPSHSCFSAAQRRVYSLMVNNSYPRFIQSEFYQELCQPVPQMPSEPQAT

>Takifugu rubripes_RGS2(ENSTRUP_8753)
KDWRNRLGFFLKLNSSHSMFHLMKNRSYSSVLRPSVDDVNQWAQSLDTLLSNKYGKTAFC
VFLKSEFSEENIEFWTACEEFRAHTSQENLLSRASSIYEEFVKNEAPKEINLDFHTKNAI
MQSLHEPHANIFMAAQRKVYSLMENNAYPRFIHSDLYRELC

>Tetraodon nigroviridis_RGS2(ENSTNIP_15305)
LINLSYFRRKDWRNRLGFFSKLNSSHSMFHLLKNRPYRPTVDDVNQWAQSLDTLLSNKYG
KTAFCIFLKSEFSEENIEFWTACEDFRLHTSQEELLSRANSIYEEFVKSEAPKEINLDFH
TKNAIVQNLHQPNASIFLAAQRKVYSLMENNSYPRFIHSDLYRELCSAARRESRHPKP

>Gasterosteus aculeatus_RGS2(ENSGACP_7723)
LSQKSQVKRKNWRNRIGFLLKTHTSKSLLHLKKRSYRPTADDVQQWAQSFDKLLGHEYGK
RVFWAFLKTEFCEENIEFWTACEDFRNVTSSKALASKANSIYEEFISSEAPKEINLDFHT
KNTIVQNLREPTVTSFLAAEKKVYSLMENDSYPRFINSKLYKEMCAAARGEG

>Homo sapiens_RGS20(ENSP_297313)
MPQLSQDNQECLQKHFSRPSIWTQFLPLFRAQRYNTDIHQITENEGDLRAVPDIKSFPPA
QLPDSPAAPKLFGLLSSPLSSLARFFSHLLRRPPPEAPRRRLDFSPLLPALPAARLSRGH
EELPGRLSLLLGAALALPGRPSGGRPLRPPHPVAKPREEDATAGQSSPMPQMGSERMEMR
KRQMPAAQDTPGAAPGQPGAGSRGSNACCFCWCCCCSCSCLTVRNQEDQRPTIASHELRA
DLPTWEESPAPTLEEVNAWAQSFDKLMVTPAGRNAFREFLRTEFSEENMLFWMACEELKK
EANKNIIEEKARIIYEDYISILSPKEVSLDSRVREVINRNMVEPSQHIFDDAQLQIYTLM
HRDSYPRFMNSAVYKDLLQSLSEKSIEA

>Macaca mulatta_RGS20(ENSMMUP_20223)
FPPAQLPYSPAAPKLLGLLYSPLSSLTRFFSHLLRRPPPEAPRRRPNFSPLLPALPAARL
SRGHEELPGRLSLLLGAALALPGRPSGGRPLRPARPVGKPREEDATAGQSSPMPPMGSER
MEMRKRQMPLAQDAPGAAPGQPGAGSRGSNACCFCWCCCCSCSCDTFLMMKFQLTTTNSS
PMPLLFWSPRSPAPTLEEVNAWAQSFDKLMVTPAGRNAFREFLRTEFSEENMLFWMACEE
LKKEANKNIIEEKARIIYEDYISILSPKEVSLDSRVREVINRNMVEPSQHIFDDAQLQIY
TLMHRDSYPRFMNSAVYKDLLQSLSEKSIEA

>Xenopus tropicalis_RGS20(ENSXETP_61981)
MGSERMEMRKRQMSVHQEAVSATQAQQGVGNRGSNACCFCWCCCCSCSCLTVRNQDDERT
RRSSNEIRGHGIPNWEESPSPTLEDVYSWGQSFDKLMTTPAGRNAFREFLRTEFSEENML
FWMACEELKKEASKNVIEEKARLIYEDYISILSPKEVSLDSRVREVINRNMLEPSQRTFD
DAQLQIYTLMHRDSYPRFMNSAIYKNLLQTLSESPADS

>Danio rerio_RGS20(ENSDARP_56734)
MGSERMEMRKRQMSVQQESVAGTTAPAQNEQPGQGNRASNACCFCWCCCCSCSCLTVRNE
DRDERNRRASYDFKADGDADFEESPKPTAEEICLWGQSFDKLMNCPSGRSAFRQFLRTEF
SEENMLFWLACEEFRKEANKSVIEEKARIIYEDYISILSPKEVSLDSRVREVINRNMLEP
TSHTFDDAQLQIYTLMQRDSYPRFMNSPVYKNLLKTVSETQSVES

>Takifugu rubripes_RGS20(ENSTRUP_29421)
PMGSERMEMRKRQMSVQQESAAGGTAPTQQGQPGQQANPRGSNACCFCWCCCCSCSCRCV
DRNEDRDERSQKAAFDVKEGTADSEDCPKPTLEEVRTWGQSFDKLMCCPAGRNSFRQFLR
TEFSEENMLFWLACDEFRREANKSAIEEKARAIYEDYISILSPKEVSLDSRVREAINRNM
QEPNLHTFDDAQLQIYTLMQRDSYPRYMNSPVYKNLFNTLSEQSPES

>Gasterosteus aculeatus_RGS20(ENSGACP_22637)
MGSERMEMRKRQMSVQQESAAGGTAPAQQGQPGQANPRGSNAYFLHVACRPCSSLIIRNE
DRDERNRKASYDVKEETSDCEDCPKPTLEEVRTWGQSFDKLMCCPAGRNAFRQFLRTEFS
EENMLFWLACEEFSKDANKTPVEERARVIYEDYISILSPKEVSLDSRVRESINRNMQEPN
LQTFEDAQLQIYTLMQRDSYPRYMNSAAYKNLLNTLSEQSPES

>Homo sapiens_RGS19(ENSP_333194)
MPTPHEAEKQITGPEEADRPPSMSSHDTASPAAPSRNPCCLCWCCCCSCSWNQERRRAWQ
ASRESKLQPLPSCEVCATPSPEEVQSWAQSFDKLMHSPAGRSVFRAFLRTEYSEENMLFW
LACEELKAEANQHVVDEKARLIYEDYVSILSPKEVSLDSRVREGINKKMQEPSAHTFDDA
QLQIYTLMHRDSYPRFLSSPTYRALLLQGPSQSSSEA

>Pan troglodytes_RGS19(ENSPTRP_40391)
NQERRRAWQASRESKLQPLPSCEVCATPSPEEVQSWAQSFDKLMHSPAGRSVFRAFLRTE
YSEENMLFWLACEELKAEANQHVVDEKARLIYEDYVSILSPKEVSLDSRVREGINKKMQE
PSAHTFDDAQLQIYTLMHRDSYPRFLSSPTYRALLLQGPSQSSSEA

>Oryctolagus cuniculus_RGS19(ENSOCUP_22545)
MAIGMQILPHLPLQDEREELRISPRMVLVIQKPYSQTDLLLKPYSYSPLNPAPAQLRDVC
HAEPEEVESWAQSFDKLMHIPAGRSVSRAFLRTEYSEENMLFWLACEELKAVGNQHVVDE
KVRLIYEDYVSILSLKKVSLDSRVREGINRKMQEPSAHTFDGAQLQIYTLMHWDSYPRFL
SSPTYHAHLLR

>Canis familiaris_RGS19(ENSCAFP_38171)
MPTPHEAEKQHAGPEEAGRPPSMSSHDAAPPAAPSRNPCCLCWCCCCSCSWNEERRRAWR
ASRDNKLQPLPSCEACATPSPEEVRSWAQSFDKLMHSPAGRGVFREFLRTEYSEENMLFW

LACEELKAEANQHVVDEKARLIYEDYVSILSPKEVSLDSRVREGINKKMQEPSAHTFDDA
QLQIYTLMHRDSYPRFLSSPTYRALLLRGSSQSSHEA

>Homo sapiens_RGS17(ENSP_206262)
MRKRQQSQNEGTPAVSQAPGNQRPNNTCCFCWCCCCSCSCLTVRNEERGENAGRPTHTTK
MESIQVLEECQNPTAEEVLSWSQNFDKMMKAPAGRNLFREFLRTEYSEENLLFWLACEDL
KKEQNKKVIEEKARMIYEDYISILSPKEVSLDSRVREVINRNLLDPNPHMYEDAQLQIYT
LMHRDSFPRFLNSQIYKSFVESTAGSSSES

>Gorilla gorilla_RGS17(ENSGGOP_17405)
LGPEVAEMRKRQQSQNEGTPAVSQAPGNQRPNNTCCFCWCCCCSCSCLTVRNEERGENAG
RPTHTTKMESIQVLEECQNPTAEEVLSWSQNFDKMMKAPAGRNLFREFLRTEYSEENLLF
WLACEDLKKEQNKKVIEEKARMIYEDYISILSPKEVSLDSRVREVINRNLLDPNPHMYED
AQLQIYTLMHRDSFPRFLNSQIYKSFVESTAGSSSES

>Mus musculus_RGS17(ENSMUSP_113519)
MRKRQQSQNEGTQAVSQAPGNQRPNNTCCFCWCCCCSCSCLTVRNEERGDSSGRSPHTTK
MESIQVLEECQNPTADEVLSWSQNFDKMMKTPAGRNLFREFLRTEYSEENLLFWLACEDL
KKEQNKKAVEEKARMIYEDYISILSPKEVSLDSRVREVINRSLLDPSPHMYEDAQLQIYT
LMHRDSFPRFLNSQIYKAFVESTTSCTSES

>Felis catus_RGS17(ENSFCAP_12811)
LSSFSQLGTEAAEMRKRQQSQNEGTPAVSQAPGNQRPNNTCCFCWCCCCSCSCLTVRNEE
RGENAGRPTHTTKMESIQVLEESQNPTSDEVLSWSQNFDKMMKAPAGRNLFREFLRTEYS
EENLLFWLACEDLKKEQNKKVIEEKARMIYEDYISILSPKEVSLDSRVREVINRNLLDPN
PHMYEDAQLQIYTLMHRDSFPRFNSQIYKSFVESTASSTSES

>Xenopus tropicalis_RGS17(ENSXETP_18378)
FSLSVCCLFVATVDIAEMRKRQQAQNEEISTVSQAPGGNQRPNTCCFCWCCCCSCSCLTV
RNEERGEQAGRPTHTTKMENIQVEEECQTPTAEEILSWSHNFDKMMKSPAGRNLFREFLR
TEYSEENLLFWLACEDLKNEQNKKIVEDTARNIYEDYISILSPKEVSLDSRVREVINRNL
LDPTPHMYEDAQLQIYTLMHRDSFPRFLNSQVYKSLLESTEGSITES

>Danio rerio_RGS17(ENSDARP_107036)
MSPQSVSGVEMRKRQQGRIEGPPQAPGHPRPNTCCLCWCGCCKCLWNEDRRERSERQTCT
KMDSIEATEELHPTLDEVIAWSRSFEMMMRSPEGRDVFREFLRSEYSEENLLFWIACEDM
KKETNPSVVEEKARIIYEDYVSILSPKEVSLDSRVREGINQSLAEPSSTMYEEAQLQIYT
LMHRDSFPRFLNSSVYRDLLNSKRACLDT

>Takifugu rubripes_RGS17(ENSTRUP_12338)
ILFCSLHVLVTQPWSVSGVEMRKRQAAHIEPPPQAPGQPRPNTCCLCWCGCCKCLWNEDR
MERSERQTCTKMDSIEAAEEQRPSLEEVLSWSRSFELMLRSLEGREVFREFLRSEYSEDN
LLFWLACEDLKKETDSALVDEKARIIYEDYVSILSPKEVSLDSRVREGINQTLAEPSNLM
YEEAQFQIYTLMHRDSFPRFLNSSLYRDLLANRRRACLDP

>Tetraodon nigroviridis_RGS17(ENSTNIP_20133)
AEMRKRQAAHIEAPPQAPGQPRPNTCCLCWCGCCKCLWFRCRNEDRMERSERQSCTKMDS
IEAAEEQFARRPSLEEALSWSRSFELMLRSLEGREVFREFLRSEYSEDNLLFWLACEDLK
KETDSALVDEKARIIYEDYVSILSPKEVSLDSRVREGINQTLAEPSNLMYEEAQFQIYTL
MHRDSFPRFLNSSLYRDLLANRRRACLDP

>Gasterosteus aculeatus_RGS17(ENSGACP_13703)
MRKRQAAHIEAPPQAPGQPRPNTCCLCWCGCCKCLWNEDRIERSERQTCKMDSIEAVEEQ
QPSLDEVLSWARSFEMMLRSLEGREVFREFLRSEYSEDNLLFWLACEELKKETDPAVVEE
KSRIIYEDYVSILSPKEVSLDSRVREGINQNLAEPSNLMYEEAQFQIYTLMHRDSFPRFL
NSSVYRDLLANRRRACLDP

>Oryzias latipes_RGS17(ENSORLP_6183)
VVVRYLLNSIQLGSSVSGVEMRKRQAAHIEAPPQAPGQPRPNTCCLCWCGCCKCLCITVC
RNEERMERNERQTCTMMDSIDAADEHCRRPSLEELLSWARSFDMMLRSFEGREIFREFLR
SEYSEDNLLFWLACEELKKETNPSVVDEKARIIYEDYVSILSPKEVSLDSCVREGINQSL

AEPSNVMYEEAQLQIYTLMHRDSFPRFLNSSVYRDLLASRRRTCLDT

>Homo sapiens_RGS3(ENSP_259406)
MPVIPALWEVEMGRSQGQEIETILANRSHSDSTPLPNFLSGSHRPECCTCRLLTASGAQD
SLPFGRRLYSGPWRSCEEVCHVSVLSVLSTSCGLSLSLPIFPGWMEWLSPDIALPRRDEW
TQTSPARKRITHAKVQGAGQLRLSIDAQDRVLLLHIIEGKGLISKQPGTCDPYVKISLIP
EDSRLRHQKTQTVPDCRDPAFHEHFFFPVQEEDDQKRLLVTVWNRASQSRQSGLIGCMSF
GVKSLLTPDKEISGWYYLLGEHLGRTKHLKVARRRLRPLRDPLLRMPGGGDTENGKKLKI
TIPRGKDGFGFTICCDSPVRVQAVDSGGPAERAGLQQLDTVLQLNERPVEHWKCVELAHE
IRSCPSEIILLVWRMVPQVKPGPDGGVLRRASCKSTHDLQSPPNKREKNCTHGVQARPEQ
RHSCHLVCDSSDGLLLGGWERYTEVAKRGGQHTLPALSRATAPTDPNYIILAPLNPGSQL
LRPVYQEDTIPEESGSPSKGKSYTGLGKKSRLMKTVQTMKGHGNYQNCPVVRPHATHSSY
GTYVTLAPKVLVFPVFVQPLDLCNPARTLLLSEELLLYEGRNKAAEVTLFAYSDLLLFTK
EDEPGRCDVLRNPLYLQSVKLQEGSSEDLKFCVLYLAEKAECLFTLEAHSQEQKKRVCWC
LSENIAKQQQLAASPPDSKMFETEADEKREMALEEGKGPGAEDSPPSKEPSPGQELPPGQ
DLPPNKDSPSGQEPAPSQEPLSSKDSATSEGSPPGPDAPPSKDVPPCQEPPPAQDLSPCQ
DLPAGQEPLPHQDPLLTKDLPAIQESPTRDLPPCQDLPPSQVSLPAKALTEDTMSSGDLL
AATGDPPAAPRPAFVIPEVRLDSTYSQKAGAEQGCSGDEEDAEEAEEVEEGEEGEEDEDE
DTSDDNYGERSEAKRSSMIETGQGAEGGLSLRVQNSLRRRTHSEGSLLQEPRGPCFASDT
TLHCSDGEGAASTWGMPSPSTLKKELGRNGGSMHHLSLFFTGHRKMSGADTVGDDDEASR
KRKSKNLAKDMKNKLGIFRRRNESPGAPPAGKADKMMKSFKPTSEEALKWGESLEKLLVH
KYGLAVFQAFLRTEFSEENLEFWLACEDFKKVKSQSKMASKAKKIFAEYIAIQACKEVNL
DSYTREHTKDNLQSVTRGCFDLAQKRIFGLMEKDSYPRFLRSDLYLDLINQKKMSPPL

>Callithrix jacchus_RGS3(ENSCJAP_33218)
QSGLIGCMSFGMKSLLTPDKEISGWYYLLGEHLGRTKHLKVATRRLQLLRDPLLRMPGSG
DTENGEKLKITIPRGKDGFGFTICCDSPVRVQAVDSGGPAERAGLQQLDTVLQLNERPVE
HWKCVELAHEIRSCHSEIILLVWRMVPQVKPGPDGGVLRRASCKSSHDLLSPPNKREKNC
THGAQARPEQRHSCHLVCDSSDGLLLSGWERYTEVAKRGGQHTLPALSRATAPTDPNYII
LAPLNPGSQLLRPVYQEDTIPEESGSPSKGKSYTGLGKKSRLMKTVQTMKGHGNYQNCPV
MRPHATHSSYGTYVTLAPKVLVFPVFVQPLDLCNPARTLLLSEELLLYEGRNKASQVTLF
AYSDLLLFTKEDEPGRCNVLRNPLYLQSVKLQEGSSEDLKFCVLYLAEKAECLFTLEAHS
HEQKKRVCWCLSENITKQQQLAASPPDSKMFETEADEKREMSLEEGKGPGAEDSPPSKEP
SPGKEDLPPKKDSSSGQEPPPGQEPLSSNNSATCEESPPGPDALPSKDAPPCQEPPPAQD
LSPCQDLPAGQEPLPHQDPLLTKDLPAVQEPPTQDLPPCQDLPPSQVSLPVKALIEETTS
SGDLLAATGDPPAASRPAFVIPEVRLDSTYSQEAGAERGSSGDTSDDNYGERSEAKRSSM
IETGQGAEGGLSLRVQNSLRRRTHSEGSLLQEPRGTCFASDTTLHCSDGEGAASAWAMPS
PSTLKKELGRNGGSMHHLSLFFTGHRKISGADTAGDDDEASRKRKSKNLAKDMKNKLGLF
RRRNESPGAPPAGKADKMMKSFKPTSEEALKWGESLEKLLLHKYGLAVFQAFLRTEFSEE
NLEFWLACEDFKKVKSQSKMAAKAKKIFAEYIAIQACKEVNLDSYTREHTKDNLQSVTRG
CFDLAQKRIFGLMEKDSYPRFLRSDLYLDLINQKKMSPPL

>Rattus norvegicus_RGS3(ENSRNOP_71774)
MEWPHQDTSLSRKDACTQTYPPRRRIRHAQLQDAGQLKLSIGAQDRVLLLHVIEGKGLIS
KKPGICDPYVKISLIPEDSQLCCQTTQVIRDCRDPAFHEHFFFPVPEEGDQKRLLVTVWN
QASETRQHALIGCMSFGVRSLLTPDKEISGWYYLLGEDLGRTKHLKVARRRLQPLRDVLL
RMPGDGDPENGEKLQITIRRGKDGFGFTICCDSPVRVQAVDSGGPAERAGLQQLDTVLQL
NERPVEHWKCVELAHEIRSCPSEIILLVWRVVPQIKPGPDGGVLRRASCKSTHDLLSPPN
KREKNCTHGAPTRPEQRHSCHLVCDSSDGLLLGGWERYTEVGKRSGQHTLPALSRATTPT
DPNYIILAPLNPGSQLLRPVYQEDAIPEEPGTTTKGKSYTGLGKKSRLMKTVQTMKGHSN
YQDCSALRPHIPHSSYGTYVTLAPKVLVFPVFVQPLDLCNPARTLLLSEELLLYEGRNKT
SQVTLFAYSDLLLFTKEEEPGRCDVLRNPLYLQSVKLQEGSSEDLKFCVLYLAEKAECLF
TLEAHSQEQKKRVCWCLSENIAKQQQLAATPTERKMFETEADEKEMPLVEGKGPGAEERT
PSKDPSPSQELPPGQELPPSKDPSPSQELPPGQDLPPSKDPSPSQELPPGQELPPSKDPS
PSQELPAGQDLPPRKESFSGQEAAPGPESPSSEDIATCQNPPQSPETSTSKDSPPGQGSS
PTTEVPSCQGLPAGQESTSQDPLLSQEPPAIPESSASDQNVLPSQESPPSQGSLSEKALA
EQTISPGELPAATAGEPSASRPNFVIPEVRLDSAYSQQDGAHGGSSGEDEDAEEGEEGEE
GEEDEEDDTSDDNYGDRNEAKRSSLIETGQGAEGGLSLRVQNSLRRRTHSEGSLLQEARG
PCFASDTTLHCSDGEGTTSTWAIPSPRTLKKELGRNGGSMHHLSLFFTGHRKMSGTDLAD
DVEASRKRKSKNIAKDMKNKLAIFRRRNESPGAQPAGKADKTTKSFKPTSEEALKWSESL
EKLLLHKYGLEVFQAFLRTEFSEENLEFWLACEDFKKVKSQSKMAAKAKKIFAEFIAIQA

CKEVNLDSYTREHTKENLQSITRGCFDLAQKRIFGLMEKDSYPRFLRSDLYLDLINQKKM
SPPL

>Felis catus_RGS3(ENSFCAP_18970)
LSVLSESCGLSPRLPMFSGWKEWQGQHTTLHRKDAWTQTTPARRRIRHAQIQGAGQLKLS
IDAQDRVLLLHIIEGKGLMSKEPGICDPYVKISLIPEDNHRLRRQKTQTILDCREPVFHE
HFFFLVQEEDEQKRLLVTVWNRAGDSRQSGFIGCMSFGVKSLLTPDKEINGWYYLLGEHL
GRTKHLKVARRRLRPLRGPLQRIPGYGDTESGEKLKITIPRGKDGFGFTICCDSPVRVQA
VDSGGPAERAGLQQLDTVLQLNERPVEHWKCVELAHEIRSCPSEIILLVWRMVPQVKPGP
DGGVLRRASCKSTHDLQSPPNKREKNCTHGAQARPEQRHSCHLVCDSSDGLLLGGWERYT
EVAKRGGQHTLPALSRATASTDPNYIILAPLNPGSQLLRPLYQEDAIPEESGGPSKGKSY
TGLGKKSRLMKTVQTMKGHGNYQNCPVMRPHTPHSSYGTYVTLAPKVLVFPVFVQPLDLC
NPARTLLLSEELLLYEGRNKAVEVTLFAYSDLLLFTKEDEPGRCNVLRNPLYLQSVKLQE
GSSEDLKFCVLYLAEKAECLFTLEAHSQEQKKRVCWCLSENIAKQQQLAASPPESKMFEP
EADEKREMPLEEGKELGAEDPPPNKDPSSGQEPPSGQDFPPNKESSSVQEPSPGQEPLSS
KDSPSCKEPPPGQEPWPSKDSPPCQEPPALPPCQDLPTSQEPPPSQDPQVSQDLPVIRDP
PAQELEPCQDQVPLPAEETTSSGDPPAAPGDLPVASRPAFVIPEVRLDSAYSQKAGAEGG
GLGFEEDAEEAEEGDEGDEEEEEEDTSDDNYGEHHEAKRSSMIEAGQAAEGGLSLRVQNS
LRRRTHSEGSLLQEARGPCFASDTTLHCSDGEGTASTWAMPSPRTLKKELGRNGGSMHHL
SLFFTGHRKMSGADAVGDDDEASRKRKSKNLAKDMKNKLGIFRRRNESPGAQPGGKADKM
VKSFKPLEGKLGGQRAEVAGSASHKRPGLLLTDGLAVFQAFLRTEFSEENLEFWLACEDF
KKVKSQSKMTAKAKKIFAEYIAIQACKEVNLDSYTREHTKDNLQSVTRGCFDLAQKRIFG
LMEKDSYPRFLRSDLYLDLINQKKMSPPL

>Monodelphis domestica_RGS3(ENSMODP_29681)
MEHHSQVFPVLGGERVSELENTKKRISYSQLEGAGQLKLSIDKEGRVLLLHIMEARDLMN
KDSQPCDAYVKVSVVPDNDRKRRQKTQTVPDCKNPAFHERFFFPIQEADEQKRLLVAVWN
RKRDQRQSELIGCMSFGVKSLLVPDKEVSGWYYLLGQKLGRAKHLKVATRRLKQRRDLFL
KTPMLVLRQEDTENMQLKITIPRGRDGFGFTICCDSPVRVQAVDSGGPADRAGLQQLDTV
LQLNEQPVEHWKCVELAHEIRNCPGEIILLVWRIIPQIKPGPDGGVLRRASCKSTHDLQS
PPNKREKNSILGARPEQRHSCHLVCDSSDGLLVGGWERYSELGKQTQHTLPPLTRASAST
DPNYIILAPLNPGSQLLRPLYQEESIPEDIGNTSKGKSHTGLGKKSRLMKTVQTMKGYGN
YQNCTIMKPHTPHSSYGTYVTLAPKVLVFPVYVQPLDLCNTARTLLLAEELLLYEGRNKT
AKVTLFVYSDLLLLTKEDEPGRCNVLRNPLYLQSVKLQEGLPEDLKFCILYLAEKAECLL
TLEAYSQEQKKRVCWCLSENISKQPQQAAAASESKMLETDVDERRKMPSEEEVPPSTEGG
AGGAPEEPGPLESNPSQKEIFSGEATSLEMPLPAEGVANEASEEQSPPENHPSQGEALAG
EATNLEMPLPAEGVTNGAPEELGPPASDCPQVEVPTEEGPGSRASALTDKQPFVIPEVRL
DPTFSQSLGAEGIEQDGILDEDAEEEEEVEGEEGYEEEDDESDDSYLEHNEAKRCSMIET
SATEGTFMLTVQSSLRRRTHSEGSLLQETKSHCFTSDTTLNCSDGEGKKSGWALASPGTV
KKDLGRNGGSMHHLSLFFTGHRKLSGAEASCDEGAEASQKRSKKLAKDMKNRLGIFRRRN
DSPGGNGAGKTDKGMKSLKPTSEEALKWGESLEKLLLHKYGLAVFRAFLRTEFSEENLEF
WLACEDFKKVKSQSKMASKAKKIFAEYIAIQACKEVNLDSYTREHTKENLQNVTRACFDL
AQKRIYGLMEKDSYPRFLRSELYLDLINQKKPSSPL

>Danio rerio_RGS3(ENSDARP_50870)
MDYSKQNSHHVTIRERRKRLHRESLWLQDPVLPKGPSTKKPCHELKRPSDSRARRSSVRH
IRSVRDKRPLAASQEENVTRPAEGNWRKAVSRNSSAQVKVASVVKTTVKNDKQPATKKRR
KIHPSGFRGKGQLKLIVTPGRGWLYMQVLEARGLMGREYRPCDSYVKISIAPDVDHSKRW
KTRTVLDCKSPVFNEGFMLDVDEDDQKKRLLVTVWNRDRDSRRSDFLGCMSFGVHSLITS
SKEIRGWYYLLGEELGRSKHLKVASRRIRQTAEAAAPNYEAGPESSGPENMQSFTVTILR
GKDGFGFTICSDSPVRVQAVDPGGPAHQAGLQQLDTLLQLNGQPVEHWKCVDLAHAIRNC
RNEITVVVWRTVPIMKPYYEGLIHRPSYKPSGFESLSSTAKTQNKTPPLLSRPTNHKQGR
RKKGSSSDNAGSGVGESLWSWTGKREENDYKTRTQTLKGTRVTSSNGDNYIILSPVSPGN
QILQPVYSDSNGTLGTLGRIYQTSRGLQQSPDYLQDGGLQGQATLCRTMSSKTTTLPPSS
YRQSFANYQNCTIVQSHLPHSNYGTYVSLAPKILIFPVFVQPLDLCSPERVLMLSEEMIL
HDSKHIALKATVFIYTDLLLLTREDEPGRCNVLQSPLYLHQIQLQDVPADKLRLYISYWI
ERTECLFSLEAFSSEQKRRVVQCLRDNIDKQLALRERIGPDQMLEPKAAEQADLGLLGFS
PHSVSEPCSPSCPSASPILRRTNCTSPAQRLSYPDLPDANQTSPTSRDACKPSYTDTSEI
WKNRQKEVKESDLEKREQGEGESASETISVGARPTSSSSSSSPLVIPKLCLDRSFNTDAL
TSPSTDDDDDDEEEDEEDSDEGFLKRRSMVESSPCGGQQSGGLCVQRSLHRRTHSEGSLL
QEPRSPRFISDQAIDCIEAKRETPERWAVPSPQTLRKELTKNGGSVHQICLLFTGRRVCG

KPNCKCDMGKTAVKKKKSKNLAKDMKNRLTFLRKKTNDRHGSNPASKLDKVLKSDKPAPE
EALKWAESLDTLLSHKYGLAVFRSFLQTEFSEENLDFWLACEEFKKIKSLSKMTSRAKKI
FTEYISIQSCKEVNLDSYTREHIKENMENICADCFDLAQSRIFGLMERDSYPRFLRSDIY
MELTNQKRPGSAADPS

>Homo sapiens_RGS5(ENSP_433001)
MCKGLAALPHSCLERAKEIKIKLGILLQKPDSVGDLVIPYNEKPEKPAKTQKTSLDEALQ
WRDSLDKLLQNNYGLASFKSFLKSEFSEENLEFWIACEDYKKIKSPAKMAEKAKQIYEEF
IQTEAPKEVGLWVNIDHFTKDITMKNLVEPSLSSFDMAQKRIHALMEKDSLPRFVRSEFY
QELIK

>Macaca mulatta_RGS5(ENSMMUP_49829)
MCKGLAALPHSCLERAKEIKIKLGILLQKPDSVGDIVIPYNEKPEKPAKTHKPSLDEALQ
WRDSLDKLLQNNYGLASFKSFLKSEFSEENLEFWIACEDYKKIKSPAKMAEKAKQIYEEF
IQTEAPKEVNIDHFTKDITMKNLVEPSLSSFDMAQKRIHALMEKDSLPRFVRSEFYQELI
K

>Mus musculus_RGS5(ENSMUSP_27997)
MCKGLAALPHSCLERAKEIKIKLGILLQKPDSAVDLVIPYNEKPEKPAKAHKPSLEEVLQ
WRQSLDKLLQNSYGFASFKSFLKSEFSEENLEFWVACENYKKIKSPIKMAEKAKQIYEEF
IQTEAPKEVNIDHFTKDITMKNLVEPSPRSFDLAQKRIYALMEKDSLPRFVRSEFYKELI
K

>Bos taurus_RGS5(ENSBTAP_32130)
MCKGLAALPHSCLERAKEIKIKLGILLQKPESAIDLVIPYNEKPEKPAKTQKPSLDEALQ
WRDSLDKLLQNNYGLASFKSFLKSEFSEENLEFWMACEDYKKIKSPVKMAETAKKIYEEF
IQVEAPKEVNIDHFTKEITVKNLVEPSPSSFDVAQKRIHALMEKDSLPRFVRSEFYQEFI
K

>Myotis lucifugus_RGS5(ENSMLUP_14434)
SCTSAAAMAEEHVCTAKEIKIKLGTLLQKPDPAADLVIPYNEKPEKPAKTQKPSPEEALQ
WRDSLDKLLQSSYGLASFKSFLKSEFSEENLEFWMACEDFKKTKSATKMAEKAKKIYEEF
IQTEAPKEVNIDHFTKDITVRNLVEPSPSSFDVAQKRVHALMEKDSLPRFVRSEFYQEFI
K

>Gallus gallus_RGS5(ENSGALP_42833)
MCKGLAALPHTCLERAKEIKTKLGTLLQKPDSAIDFIIPYPEKPEKPAKALKPSPEEVLQ
WRDSLEKLLQNPYGLASFRSFLCSEFSEENVEFWVACEDYKKTKSPVKMEEKAKRIYEEF
IQTEAPKEVNIDHFTKAVTMKNLVEPSPSSFDMAQKRIFALMEKDSLPRFVRSEFYQELI
K

>Xenopus tropicalis_RGS5(ENSXETP_22975)
MCKGLAALPHTCLERAKEIKTKLGTLLQKPESAIDLIIPYPDKPEKTPKASKPSAEEAGQ
WRDSLDKLLQNSYGLSVFQGFLKSEFSEENIEFWMACEDYKKVKSPSKMATKAKKIYDEF
IQTEAPREVNIDHFTKAVTMKNLVEPSSSSFDLAQKKIFALMEKDSLPRFVRSEFYQELI
K

>Danio rerio_RGS5(ENSDARP_4766)
MCKGLAALPQTCLERAKEIKTKLGTLLQKPENSIDLIIPYQEKPEKKPEKLQKATPEEAA
QWRESLDKVLSNSYGLATFKSFLRSEFSEENIEFWEACEDFKKTKNPLKMATKAKKIYED
FIQTGGPKEVNIDHFTKDVTLRNLVDLSSSTFELAQSRIYTLMEKDSFGRFLRSDQYQEL
LK

>Takifugu rubripes_RGS5(ENSTRUP_12673)
MCKGLAALPQSCLERAKEIKSKLGVLLQKPENAIDLIIPYPEKTEKKPEKQQKPTPEEAA
QWRESLDRVLNNSYGLNAFRSFLQSEFSDENVEFWMSCEDFKKTKDPVKMAAKAKKIYED
FIQAEGPREVNIDHFTKDMTLRNLVKLSHMTFDLAQKRIYALMEKDSFGRFLRSELYQDL
LVN

>Tetraodon nigroviridis_RGS5a(ENSTNIP_4031)
MCKGLAALPQTCLERAKEIKTKLGVLLQKPENAIDLIIPYPEKTEKKPEKQQKTHLYHSL

EWKREEIDKLNLNIKNGLNAFRSFLQSEFSDENIEFWMSCEEFKKSKDPVKMAAKAKKIY
EDFIQAEGPREVNIDHFTKDVTLRNLVKLSPTTFDLAQKRIYALMEKDSFGRFLRSQLYQ
DLLV

>Tetraodon nigroviridis_RGS5b(ENSTNIP_1535)
MCKGLAALPQTCLERAKEIKTKLGVLLQKPENAIDLIIPYPEKTEKKPEKQQKYKQCEFI
HKREEIDKLNLNIKNGLNAFRSFLQSEFSDENIEFWMSCEEFKKSKDPVKMAAKAKKIYE
DFIQAEGPREVNIDHFTKDVTLRNLVKLSPTTFDLAQKRIYALMEKDSFGRFLRSQLYQD
LLV

>Gasterosteus aculeatus_RGS5(ENSGACP_7574)
MCKGLAALPQSCLERAKEIKSKLGVLLQKPENAIDLIIPYPEKAEKKPEKQQKPTPEEAA
QWREGLDRVLNNSYGLAAFRSFLQSEFSDENIEFWMTCEDFKKTKNPVKMATKAKKIYED
YIQSEGPREVNIDHFTKDVTLRNLVDLSPTTFDLAQKRIFALMEKDSFGRFLRSEQYQQL
LV

>Oryzias latipes_RGS5(ENSORLP_15473)
MCKGLAALPQTCLERAKEIKTKLGVFLNKPENAIDLIIPYPEKTEKKPEKLQKPTHEEAA
QWRESLDRVLNNNYGLTAFRSFLQSEFSDENIEFWMACEDFKKTKNPVKMAAKAKKIYED
FIQSEGPREVNIDHFTKDVTLRNLVDLSPSTFELAQKRIFALMEKDSFARFLRSEQYQEL
LVN

>Ciona savignyi_(ENSCSAVP_490)
MNIYRPRSMACLGRNLNSEYDGEFHKTRSGSTSTEGKISISSDGFRSMMKDIQVGLGNKL
NKFSSVSDLTGMEFGDFNDVSFVGKRTEKIGGIIGKITKKKKYQFGSVSNISVCSSLSHR
VKKLNVSNSASKLRGRFKRSGRQDKSDKLQHKFENIFAWLHRSGKKAGRYAMRRNRSPIP
MTKSDTRRKPLKLTLKEVLSKKETTNAFRAFLSRELSEENLDFWLDVETYKRAKASKRQK
MGIKIYEKYISPSSTHEINVESCVRQQTRRKLLNFDVDTFDEAQQHIFKLMAADSFRRFL
EVF

>Saccoglossus kowalevskii_(XP_006825374)
MTQRAHCHTSTRGHKRRSIELRTQSLTHGFEAMALETPPVHKRRKTGRFNQLPIQDFPEPQVYTPGQLKL
SIYRTRRHLMVNIIKARGISHIPGYTDGCYVTVVIVAPHGKKDSMATRVVTDATPHFQEIFPLSFLHLDL
LDRIVISLYRQSSTLNDLMGCMSFGAKHLIKGTKLIDGWYYLLNQELGVKKHLGVPYKRPKLSKEVKKKQ
QPVRIIKKVMTVVQGKHGYGFTILDTTPVRVGKVDSGGPSEKVGLQMGDVIIRINKHHVERSPCTDIIGI
VKNAPGHVRLVIERRAKTVIRRLSEDVERRPLNTLMSSNRCHNQKEYAKQLCGETEYTQHLCPDTDYARH
LRYDTPDSLYRSSTCSSTISGTPSEDDMMTGQVQITVVCNYHMQQLELRLAAYSSPAQKTKQELMTLGDI
YLAVLDGFISSYAVYQSCLRMAEENLAVKLQVPVWSDDEYFNTVRGFMYRPSEYLDKLCQLLQMLWTSTP
TEHPDHENFKHVLNGSVHIYFTDTRQFRLAELGRRYVFAGTLHNINQTNRDRSKKLWAIMCTDLLLLTKK
QDDDSFLIVDKLLLNSLFVQDIESSPNDFIVTVVTNIKQKENKVLRAPTEHLKKTWKKLLNKHSSGIYLM
TFDGSVKEIHIGDRKETKKGISRPFLGRRKISSPDLPSTAKTASLLSRKVSSPEIKITSFDNDDSQLNRN
AVITEELSDDTGIMMNSSKNRSKLAVELYKKSPSCEDCSKSESTQNSLEQLTTASAPGSPLFHISRSWNS
LTPTGTVSGGYPCKSNEDCAKTSKDTSSTKAKPKRKNIFQLFTGDNKNKDEIDGHDAPPHALFLKEPVEG
KSQETTSPRRTITRMRSRSVGSEDEAIEEELEDPGSGSDSPTDDALQLPSFNTDNDKRKRKKNLAKDMKE
KLRFLRRRHTDGCIKDAEKLKTLRPSYAQAIKWSESFDSLLADKYGLASFRAFLKTEFSDENIEFWLACE
DYKKVRPSKWSSRAQRIYNDYVAIQAPREVNLDSHSRLATVSNLSNPTKHAFDLAQKRIQSLMEKDSYPR
FLRSEFYQSLINDKKQVSNC

>Saccoglossus kowalevskii_(XP_006816283)
MKRDRQNTQSTPPPSPAEDKNKSSKACCFCWCCCCSCSWTHSLAVRDPSENEKQKSDTKMELKDKEIEKP
SRDEIKSWGESFDRLMKSAAGRDSFREFLKSEYSEENLLFWLACEELRHQTDQEIIEEKSRLIYEDYISI
LSPKEVSLDSRVREVINRNMVDPTQHTFDDAQQQIYILMHRDSYPRFINSQKYKQLLCKG

>Octopus bimaculoides_(XP_014787789)
MIEMTTMMRNQSPATNENDVRMRSNRPSKDNKENKETKDQPVQQQTPQNESPEEAVSPTSSPTHTRERRF
STTCCFCWCCTPPCFSNLGSGRSSKGDNSPNSNMVVKNDQLSQSRSKELDSNSDREELLSLEDMKNWSES
FDRLIRSNGGRRKFREFLRMEYSEENMLFWLACEELKREQNSELIEEKARLIYEDYISILSPKEVSLDSR
VREVINQNMLDPSPHTFDEAQLQIYTLMHQDPYPRFLNSQLYKKLIEEASS

>Nematostella vectensis_(XP_001629433)
MFVSRIAMAIKELMCEARAIKELMPSSEEINEWGRSFERLLSSKNGIRVFHEFLKSEYSEENLLFWLAVE

RLKKETEPASVKNLAQTIYNDYVSTESPKEVSIDHKTRQVIDDEISQPTKETFSKAQQHVYYVMYQDCYP
RFLSSSTFKTLQQECLNN

>Hydra magnipapillata_(XP_012555230)
MASTIIRTVNLKRSKKGLGFIISNQFPCIISCVNQNTPAFDAGLKSGDEILEINSIDVSQFKHNDVVKII
LCNEQPHILIKVRTFYSVSKDFLLHGFNQQVLDITHLSDHEKIKINFTHEKNSKDCSTVVSNSYCEERKA
NVQHSEMTENDVHYCFENISTKSSLKRSPSQPVSIYYPPIYNVLLLYYCSVDIPHDINLKISSDSTINDC
ISKIKKLNNKPVLVHLQISKMGIKLINPFGNEIVTYPLRTIIFSNIYAEDKRFFSFVTQNSLLDHSVTTS
LNSTKKFVKSKIVCLHACHVFMVESKLSVHSDHKEFADVFKIACTPSLKFDGCQEFPPSPSYVLQELNAF
LKERKNVRKGVQSFCFVSTEEFPSTPEEDVIYNTLEKIQKSSLNSPTRNTQKLMVSPPCSLQGINEDEKL
CLIKEIQQNNNYSQKISLTADYPMQKNMHSLLEIGGEKNISWIKGNEFSKKLFPSKRMEYKKPPVPRKIS
CATKSFGFTKIISSEQKGSNIKNQENKKLFGLFNSTEKCKEQVSSDIKKERSSSDSMVSENSTESFSDIN
SRKGTPPTNPKHSVKSPEKEKNVHNKKVELWSCSFESLLADIDGVKCFQEFLKKEFSEENLMFWKACNDF
LKLKDINKIKECAKEIFDNYLSSKAPFLVNVNLSICKRVEQQLDSPNTDLFVIPQQQVFQLMKFDSYPRF
IKSDWFKERVNNYNADNSSKENLKKLNQSFEQEEKNVVNFRTGKVKSPELKNSRAAIHKGINLIVSTLYL
YCVFR

>Trichoplax adhaerens_(XP_002109868)
MVMALLKVYVNSLNGIEAFARFLKSEFSDENIKFWLACEEFRKIDNKGEIESRAKWIYDTYVSRKAKTEI
NLDSKTRSHIRKRMESIDNNIFDQGQKCIKELMATDSYPRFIKSSKYRSLLA

Regulating Synaptic Membrane Exocytosis Protein-RIMS

>Homo sapiens_RIMS3(ENSP_361768)
MFNGEPGPASSGASRNVVRSSSISGEICGSQQAGGGAGTTTAKKRRSSLGAKMVAIVGLT
QWSKSTLQLPQPEGATKKLRSNIRRSTETGIAVEMRSRVTRQGSRESTDGSTNSNSSDGT
FIFPTTRLGAESQFSDFLDGLGPAQIVGRQTLATPPMGDVHIAIMDRSGQLEVEVIEARG
LTPKPGSKSLPATYIKVYLLENGACLAKKKTKMTKKTCDPLYQQALLFDEGPQGKVLQVI
VWGDYGRMDHKCFMGMAQIMLDELDLSAAVTGWYKLFPTSSVADSTLGSLTRRLSQSSLE
SATSPSCS

>Pan troglodytes_RIMS3(ENSPTRP_1025)
MFNGEPGPASSGASRNVVRSSSISGEICGSQQAGGGAGTTTAKKRRSSLGAKMVAIVGLT
QWSKSTLQLPQPEGATKKLRSNIRRSTETGIAVEMRSRVTRQGSRESTDGSTNSNSSDGT
FIFPTTRLGAESQFSDFLDGLGPAQIVGRQTLATPPMGDVHIAIMDRSGQLEVEVIEARG
LTPKPGSKSLPATYIKVYLLENGACLAKKKTKMTKKTCDPLYQQALLFDEGPQGKVLQVI
VWGDYGRMDHKCFMGMAQIMLDELDLSAAVTGWYKLFPTSSVADSTLGSLTRRLSQSSLE
SATSPSCS

>Mus musculus_RIMS3(ENSMUSP_130295)
MFNGEPGPASAGASRNVVRSSSISGEICGSQQAGGGAGTTTAKKRRSSLGAKMVAIVGLT
QWSKSTLQLPQPEGATKKLRSNIRRSTETGIAVEMRSRVTRQGSRESTDGSTNSNSSEGT
FIFPTRLGAESQFSDFLDGLGPAQIVGRQTLATPPMGDVHIAIMDRSGQLEVEVIEARGL
TPKPGSKSLPATYIKAYLLENGACVAKKKTKVAKKTCDPLYQQALLFDEGPQGKVLQVIV
WGDYGRMDHKCFMGMAQIMLDELDLSAAVTGWYKLFPTSSVADSTLGSLTRRLSQSSLES
ATSPSCS

>Bos taurus_RIMS3(ENSBTAP_24925)
MFNGESGPASAAASRNVVRSSSISGEICGSQQAGDGTGTSTAKKRRSSLGAKMVAIVGLT
QWSKGTLQLPQAEGATKKLRSNIRRSTETGVAVEMRSRVPRQGSRESTDGSTNSNSSDGT
FIFPTTRLGAESQFSDFLDGLGPAQIVGRQTLATPPMGDVHIAIMDRSGQLEVEVIEARG
LTPKPGSKSLPATYIKVYLLENGACLAKKKTKVAKKTCDPLYQQALLFDEGPQGKVLQVI
VWGDYGRMDHKCFMGMAQIMLDELDLSAAVTGWYKLFPTSSVADSTLGSLTRRLSQSSLE
SATSPSCS

>Monodelphis domestica_RIMS3(ENSMODP_21938)
MFDGEPGTGPAASSRNVVRSSSISGEVFGGGGEQAGGTAGASTAKKRRSSLGAKMAAIVG
LTQRSRSSLQLPQPEGGPKKLPSNIRRSTETGIAVEMRSRVTRQGSRESTDGSTNSNSSD
GTFIFPTARLGAESQFSDFLDGLGPAQIVGRQTLGTPPMGDVHIAMMDRSGQLEVEVIEA
RGLTPKLGSKSLPATYIKVYLLENGACLAKKKTRVAKKTCDPLYHQALLFDEGPQGKVLQ
VIVWGDYGRMDHKCFMGMAQIVLEELDLSNVVTGWYKLFPTSSVADSTLGPLTRRLSQSS

LESSTSPSCP

>Xenopus tropicalis_RIMS3(ENSXETP_38276)
MFNGETSSSVRNVIRSSSISGEMYNLEKGNSANPDAAGVTTTKKRRSSLGAKMVAIVGLS
QWSKSTLQLNQPDGTTKKLRSNIRRSTETGIAVEMRNRVTRQGSRESTDGSTNSNSSDGT
FIFPTTRLGAESQFSNFLDGLGPAQIVGRQTLATPPMGDVHIGMVDRNGQLEVEVIQARG
LTPKPGSKSIPAPYIKVYLIENGVCLSKKKTRTSKKTWDPVYQQALLFDEGPQGKVLQVI
VWGDYGRMDHKCFMGMAQILLEELDLSSCVTGWYKLFPTSSLADSSIAPLTRRLSQSSLE
SSTSPSCT

>Danio rerio_RIMS3(ENSDARP_94802)
MMLSSSVDVPSPGGMSAVAARNVVRSSSISGAMYSLEKSPSSTGPDGTSLTGNKKRRSSL
GAKMVAIVGLSQWSKSTLQLNQQDGGTKKLRSTIRRSTETGIAVEMRNRVTRQGSKDSTD
GSTNSNSSDGTFIFPTTRLGPESQFSDFLDGLGPAQIVGRQTLATPSMGDVYIGVVDRGG
QLEVEITQARSLTPKPGSKNIPATYIKVYVLENGMCLAKKKTKVVKKTLDPTFQQSLMFD
ESPQGKVLQVIVWGDYGRMDSKCFMGMAQILLDDLDLSSMVGGWYKLFPTSSLADPSIGP
LTRRLSQSSLESATSPSCT

>Takifugu rubripes_RIMS3(ENSTRUP_46461)
VEVPSPGGMSGLSVAARNVVRSSSISGAMYSLEKSPGGGGSDSTSLAGNKKRRSSLGAKM
VAIVGLSQWSKSTQQLNQQEGGTKKLRSTIRRSTETGIAVEMRNRVTRQGSKDSTDGSTN
SNSSDGTFVFPTTRLGPESQFSDFLDGLGPAQIVGRQTLATPPMASTGDVHIGIVDRGGQ
LEVEVNQARGLTPKPGSKNIPATYVKVYVLENGVCLAKKKTKVVKRNLDPTYQQALLFDE
SPQGKVLQVIVWGDYGRMDHKCFMGMAQILLEDLDLSATVSGWYKLFPTSSLADPSIGPL
TRRLSQSSLESATSPSCS

>Tetraodon nigroviridis_RIMS3(ENSTNIP_12061)
VEVPSPGGMSGLSVAARNVVRSSSISGAMYSLEKSPGGGGSDSTSLAGNKKRRSSLGAKM
VAIVGLSQWSKSTQQLNQQDGGTKKLRSTIRRSTETGIAVEMRNRVTRQGSKDSTDGSTN
SNSSDGTFVFPTTRLGPESQFSDFLDGLGPAQIVGRQTLATPPMGFTSTGDVHIGIVDRG
GQLEVEVNQARGLTPKPGSKNIPATYVKVYVLENGVCLAKKKTKVVKRNLDPTYQQALLF
DESPQGKVLQVIVWGDYGRMDKCFMGMAQILLEDLDLSATVSGWYKLFPTSSLADPSIGP
LTRRLSQSSLESATSPSCS

>Gasterosteus aculeatus_RIMS3(ENSGACP_9569)
MGGLSGLSAAAARNVVRSSSISGAMYGLEKSPGGGGPDSASLAGNKKRRSSLGAKMVAIV
GLSQWSKSTQQLHQQDGGTKKLRSTIRRSTETGIAVEMRNRVTRQGSKDSTDGSTNSNSS
DGTFVFPTTRLGPESQFSDFLDGLGPAQIVGRQTLATPSMGDVNVGMVDRGGQLEVEVNQ
ARGLTAKPGSKNMPATYVKVYVLENGLCLAKKKTRVVKKNLDPTYQQALLFDESPLGKVL
QVIVWGDYGRMDHKCFMGMAQILLEDLDLSATVSGWYKLFPTSSLADPSIGPLTRRLSQS
SLESATSPSCT

>Oryzias latipes_RIMS3(ENSORLP_6562)
QIKSKSEVQIRRTFWSLINAHYFDVDHFSEKNNGGTKKLRSTIRRSTETGIAVEMRNRVT
RQGSKDSTDGSTNSNSSDGTFVFPTTRLGPESQFSDFLDGLGPAQIVGRQTLATPSMGDV
HVAMVDRGGQLEVEVSQARGLTPKPGTRNIPATYVKVYVLENGVCLAKKKTKVVKKTLDP
TYQQALLFDESPQGKVLQVIVWGDYGRMDHKCFMGMAQILLEDLDLSATVSGWYKLFPTS
SLADPSIGPLTRRLSQSSLESATSPS

>Homo sapiens_RIMS2(ENSP_427018)
MSAPVGPRGRLAPIPAASQPPLQPEMPDLSHLTEEERKIILAVMDRQKKEEEKEQSVLKK
LHQQFEMYKEQVKKMGEESQQQQEQKGDAPTCGICHKTKFADGCGHNCSYCQTKFCARCG
GRVSLRSNKVMWVCNLCRKQQEILTKSGAWFYNSGSNTPQQPDQKVLRGLRNEEAPQEKK
PKLHEQTQFQGPSGDLSVPAVEKSRSHGLTRQHSIKNGSGVKHHIASDIASDRKRSPSVS
RDQNRRYDQREEREEYSQYATSDTAMPRSPSDYADRRSQHEPQFYEDSDHLSYRDSNRRS
HRHSKEYIVDDEDVESRDEYERQRREEEYQSRYRSDPNLARYPVKPQPYEEQMRIHAEVS
RARHERRHSDVSLANADLEDSRISMLRMDRPSRQRSISERRAAMENQRSYSMERTREAQG
PSSYAQRTTNHSPPTPRRSPLPIDRPDLRRTDSLRKQHHLDPSSAVRKTKREKMETMLRN
DSLSSDQSESVRPPPPKPHKSKKGGKMRQISLSSSEEELASTPEYTSCDDVEIESESVSE
KGDMDYNWLDHTSWHSSEASPMSLHPVTWQPSKDGDRLIGRILLNKRLKDGSVPRDSGAM
LGLKVVGGKMTESGRLCAFITKVKKGSLADTVGHLRPGDEVLEWNGRLLQGATFEEVYNI

ILESKPEPQVELVVSRPIGDIPRIPDSTHAQLESSSSSFESQKMDRPSISVTSPMSPGML
RDVPQFLSGQLSSQSLSRRTTPFVPRVQIKLWFDKVGHQLIVTILGAKDLPSREDGRPRN
PYVKIYFLPDRSDKNKRRTKTVKKTLEPKWNQTFIYSPVHRREFRERMLEITLWDQARVR
EEESEFLGEILIELETALLDDEPHWYKLQTHDVSSLPLPHPSPYMPRRQLHGESPTRRLQ
RSKRISDSEVSDYDCDDGIGVVSDYRHDGRDLQSSTLSVPEQVMSSNHCSPSGSPHRVDV
IGRTRSWSPSVPPPQSRNVEQGLRGTRTMTGHYNTISRMDRHRVMDDHYSPDRDRDCEAA
DRQPYHRSRSTEQRPLLERTTTRSRSTERPDTNLMRSMPSLMTGRSAPPSPALSRSHPRT
GSVQTSPSSTPVAGRRGRQLPQLPPKGTLDRKAGGKKLRSTVQRSTETGLAVEMRNWMTR
QASRESTDGSMNSYSSEGNLIFPGVRLASDSQFSDFLDGLGPAQLVGRQTLATPAMGDIQ
VGMMDKKGQLEVEIIRARGLVVKPGSKTLPAPYVKVYLLDNGVCIAKKKTKVARKTLEPL
YQQLLSFEESPQGKVLQIIVWGDYGRMDHKSFMGVAQILLDELELSNMVIGWFKLFPPSS
LVDPTLAPLTRRASQSSLESSTGPSYSRS

>Pan troglodytes_RIMS2(ENSPTRP_35048)
MQFETLRQVCNSVLSHFHGVFSSPPNILQNELFGQTLNNARKRSPSVSRDQNRRYDQREE
REEYSQYATSDTAMPRSPSDYADRRSQHEPQFYEDSDHLSYRDSNRRSHRHSKEYIVDDE
DVESRDEYERQRREEEYQSRYRSDPNLARYPVKPQPYEEQMRIHAEVSRARHERRHSDVS
LANADLEDSRISMLRMDRPSRQRSISERRAAMENQRSYSMERTREAQGPSSYAQRTTNHS
PPTPRRSPLPIDRPDLRRTDSLRKQHHLDPSSAVRKTKREKMETMLRNDSLSSDQSESVR
PPPPKPHKSKKGGKMRQVSLSSSEEELASTPEYTSCDDVEIESESVSEKGDMDYNWLDHT
SWHSSEASPMSLHPVTWQPSKDGDRLIGRILLNKRLKDGSVPRDSGAMLGLKVVGGKMTE
SGRLCAFITKVKKGSLADTVGHLRPGDEVLEWNGRLLQGATFEEVYNIILESKPEPQVEL
VVSRPIGDIPRIPDSTHAQLESSSSSFESQKMDRPSISVTSPMSPGMLRDVPQFLSGQLS
IKLWFDKVGHQLIVTILGAKDLPSREDGRPRNPYVKIYFLPDRSDKNKRRTKTVKKTLEP
KWNQTFIYSPVHRREFRERMLEITLWDQARVREEESEFLGEILIELETALLDDEPHWYKL
QTHDVSSLPLPHPSPYMPRRQLHGESPTRRLQRSKRISDSEVSDYDCDDGIGVVSDYRHD
GRDLQSSTLSVPEQVMSSNHCSPSGSPHRVDVIGRTRSWSPSVPPPQSRNVEQGLRGTRT
TTGHYNTISRMDRHRVMDDHYSPDRDSHFLTLPRSRYSQTIDHHHRDGRDCEAADRQPYH
RSRSTEQRPLLERTTTRSRSTERPDTNLMRSMPSLMTGRSAPPSPALSRSHPRTGSVQTS
PSSTPVAGRRGRQLPQLPPKGTLDR

>Mus musculus_RIMS2(ENSMUSP_48719)
MSAPLGPRGRPAPTPAASQPPPQPEMPDLSHLTEEERKIILAVMDRQKKEEEKEQSVLKK
LHQQFEMYKEQVKKMGEESQQQQEQKGDAPTCGICHKTKFADGCGHNCSYCQTKFCARCG
GRVSLRSNKVMWVCNLCRKQQEILTKSGAWFYNSGSNTLQQPDQKVPRGLRNEEAPQEKK
AKLHEQPQFQGAPGDLSVPAVEKGRAHGLTRQDTIKNGSGVKHQIASDMPSDRKRSPSVS
RDQNRRYEQSEEREDYSQYVPSDGTMPRSPSDYADRRSQREPQFYEEPGHLNYRDSNRRG
HRHSKEYIVDDEDVESRDEYERQRREEEYQARYRSDPNLARYPVKPQPYEEQMRIHAEVS
RARHERRHSDVSLANAELEDSRISLLRMDRPSRQRSVSERRAAMENQRSYSMERTREAQG
QSSYPQRTSNHSPPTPRRSPIPLDRPDMRRADSLRKQHHLDPSSAVRKTKREKMETMLRN
DSLSSDQSESVRPPPPRPHKSKKGGKMRQVSLSSSEEELASTPEYTSCDDVELESESVSE
KGDSQKGKRKTSEQGVLSDSNTRSERQKKRMYYGGHSLEEDLEWSEPQIKDSGVDTCSST
TLNEEHSHSDKHPVTWQPSKDGDRLIGRILLNKRLKDGSVPRDSGAMLGLKVVGGKMTES
GRLCAFITKVKKGSLADTVGHLRPGDEVLEWNGRLLQGATFEEVYNIILESKPEPQVELV
VSRPIGDIPRIPDSTHAQLESSSSSFESQKMDRPSISVTSPMSPGMLRDVPQFLSGQLSI
KLWFDKVGHQLIVTILGAKDLPSREDGRPRNPYVKIYFLPDRSDKNKRRTKTVKKTLEPK
WNQTFIYSPVHRREFRERMLEITLWDQARVREEESEFLGEILIELETALLDDEPHWYKLQ
THDVSSLPLPRPSPYLPRRQLHGESPTRRLQRSKRISDSEVSDYDCEDGVGVVSDYRHNG
RDLQSSTLSVPEQVMSSNHCSPSGSPHRVDVIGRTRSWSPSAPPPQRNVEQGHRGTRATG
HYNTISRMDRHRVMDDHYSSDRDSHFLTLPRSRHSQTIDHHHRDGRDCEAADRQPYHRSR
STEQRPLLERTTTRSRSSERPDTNLMRSMPSLMTGRSAPPSPALSRSHPRTGSVQTSPSS
TPGTGRRGRQLPQLPPKGTLERSAMDIEERNRQMKLNKYKQVAGSDPRLEQDYHSKYRSG
WDPHRGADTVSTKSSDSDVSDVSAVSRTSSASRFSSTSYMSVQSERPRGNRKISVFTSKM
QNRQMGVSGKNLTKSTSISGDMCSLEKNDGSQSDTAVGALGTSGKKRRSSIGAKMVAIVG
LSRKSRSASQLSQTEGGGKKLRSTVQRSTETGLAVEMRNWMTRQASRESTDGSMNSYSSE
GNLIFPGVRLASDSQFSDFLDGLGPAQLVGRQTLATPAMGDIQVGMMDKKGQLEVEIIRA
RGLVVKPGSKTLPAPYVKVYLLDNGVCIAKKKTKVARKTLEPLYQQLLSFEESPQGRVLQ
IIVWGDYGRMDHKSFMGVAQILLDELELSNMVIGWFKLFPPSSLVDPTLAPLTRRASQSS
LESSTGPSYSRS

>Equus caballus_RIMS2(ENSECAP_20682)

RVKEEHKPQPTQWFPFSGITELVNNVLQPQQKQQNEKEAQTKLHQQFELYKEQVKKMGEE
SQQQQEQKGDAPTCGICHKTKFADGCGHNCSYCQTKFCARCGGRVSLRSNKVMWVCNLCR
KQQEILTKSGAWFYNSGSNTPQQPDQKVLRGLRNEEAPQEKKAKLHEQAQFQGPSGDLSV
PAVEKSRSHGLTRQDSIKNGSGVTHQIASDIASDRKRSPSVSRDQNRRYDQREEREEYSQ
YATSDSAMPRSPSDYADRRSQHEPQFYEESDNINYRDSNRRSHRHSKEYIADDEDVESRD
EYERQRREEEYQARYRSDPNLARYPVKPQPYEEQMRIHAEVSRARHERRHSDVSLANAEL
EDSRISMLRMERPSRQRSISERRAALENQRSYSMERTREAQGPSSYPQRTTNHSPPTPRR
SPIPIDRPDLRRTDSLRKQHHLDPSSAINKRKRSILAQMLAQGPSSSSRKRKIGNRGKKE
RKEKKESKKRGNSLTFSEETGKSLPSFFFCESLISKVSLSNLWDSQKGKRKTREQAVLSD
SNTRSEKQKKMMYFGGHSLEEDLEWSEPQIKDSGVDTCSSTTLNEEHSHSDKHPVTWQPS
KDGDRLIGRILLNKRLKDGSVPRDSGAMLGLKVVGGKMTESGRLCAFITKVKKGSLADTV
GHLRPGDEVLEWNGRPLQGATFEEVYNIILESKPEPQVELVVSRPIGDIPRIPDSTHAQL
ESSSSSFESQKMDRPSISVTSPMSPGMLRDVPQFLSGQLSIKLWFDKVGHQLIVTILGAK
DLPSREDGRPRNPYVKIYFLPDRSDKNKRRTKTVKKTLEPKWNQTFIYSPVHRREFRERM
LEITLWDQARVREEESEFLGEILIELETALLDDEPHWYKLQTHDVSSLPLPHPSPYMPRR
QLHGESPTRRLQRSKRISDSEVSDYDCDDGIGVVSDYRHNGRDLQSSTLSVPEQVMSSNH
CSLSGSPHRVDVIGRTRSWSPSVPPPQSRNVEQGLRGTRSATGHYNTISRMDRHRVMDDH
YSPDRDRDCEAADRQPYHRSRSTEQRPLLERTTTRSRSTERPDTNLMRSMPSLMTGRSAP
PSPALSRSHPRTGSVQTSPSSTPVAGRRGRQLPQLPPKGTLERKAGGKKLRSTVQRSTET
GLAVEMRNWMTRQASRESTDGSMNSYSSEGNLIFPGVRLASDSQFSDFLDGLGPAQLVGR
QTLATPAMGDIQVGMMDKKGQLEVEIIRARGLVVKPGSKTLPAPYVKVYLLDNGVCIAKK
KTKVARKTLEPLYQQLLSFEESPQGKVLQIIVWGDYGRMDHKSFMGVAQILLDELELSNM
VIGWFKLFPPSSLVDPTLAPLTRRASQSSLESSTGPSYSRS

>Xenopus tropicalis_RIMS2(ENSXETP_8820)
MSAPLGPRGGPLPPAQPQAPMPEMPDLSHLTEDERKIIMAVMERQKKEEEKEQSVLKVKE
EQKAQPAQWFPFSGITELVNNVLQPQPKEQNEKEPEIKLHQQFEMYKEQVKKMGEEAQQQ
QEQKGEAPTCGICHKTKFADGCGHDCSYCQTKFCARCGGRVSLRSNKVMWVCNLCRKQQE
ILTKSGAWFYSSGSSTPLKPGEESQAGYQTEEAPQEKKAKLQEQSQYQGPSGDLLTPASD
KNRPQGIVRQDSIKNGSELKHPIPGDLASDSHRKRSPSIPRDQNRRYDTRDDIGEHSQHA
TSDSAMPRSPSDYSDRRLQRGSQLYDEPEHGDYRRSRRRSNEYSVDEEDIQDREEYERQR
REEEYQARYRSDPNLARYPVKPQPYEEQMRIHAEVSRARQRRHSDVSLAQTEFEESRMST
LRMERPSRQRSASERRAAMESQRSYSIERTRDVQGPGPGRQRTSNHSPPTPRRSPIPNDR
PDMRRTDSLRKQHHLDPSSAVRKTKREKMETMLRNDSLSSDQSESVRPPPPKPHKTKKGG
KMRQVSLSSSEEELASTPEYTSCDDVEIESESVSEKGDSQRGKRKTSEQAVLSDSNILSE
RQKKMVCFGGHSLEEDLEWSEPQIKDSGVDTCSSTTLNEEHSHSEKHPVTWQPSKDGDRL
IGRILLNKRLKDGSVPRDSGAMLGLKVVGGKMTESGRLCAFITKVKKGSLADTVGHLRPG
DEVLEWSGRILQGATFEEVYNIILESKPEAQVELLVSRPIGDIPRIPDSTHAQLESSSSS
FESQKMDRPSISVTSPMSPGMLRDVPQFLSGQLSIKLWYDKVGHQLIVTILGAKDLPSRE
DGRPRNPYVKIYFLPDRSDKNKRRTKTVKKTVEPKWNQTFIYSPVHRREFRERMLEITLW
DQARVREEESEFLGEILIELETALLDDEPHWYKLQTHDVSSLPLPHPSPFMQRRQLHGES
PTRRLQRSKRISDSEVSDYDCDDGVGVVSDFRHNGRDLQSSTLSVPEQVMSSNHCSRTGP
PHVDSIGRTRSWSPSVPPPQSRNLEQGPRGTRSGPAHYNTMSRVERHRVLDNHYSPDRDR
AHPPRTGSVQTSPSGTPLAGRRGRQLPQLPPKGTLERSAMDVEERTRQLKVKINKYKQVG
GSDSRLEKDYHPKYRTGRDPQRGSDNMSNKSSDSDVSDVSAVSRTSSASRFSSTSYMSVQ
SERPRGNMKIGALASKMQSRQMGASGKNMTKSTSIGGDMYTLEKNDGSQSDTAVGTVGAG
GKKRRSSIGAKMVAIVGLSRKSRSTSQLSQT

>Takifugu rubripes_RIMS2(ENSTRUP_19111)
MSAPHGPRGGPGPGPAAAAASAAGAVPDLPDLSHLTEEERKIILGVMERQKTEIKEEPKP
QPAQWFSGITELVNNVLQGPQKSQNEKEPEPDTKLHQQFESYKEQVKKMGEETKPAQDQK
SEAPTCGICHKTKFADGCGHLCSYCQTKFCARCGGRVSLRSNKVMWVCNLCRKQQEILTK
SGAWFYPSGPGQVRGVFEGGPQGKKAKLQDPSLYPYHGTSGDLTPASDRSRPHGGLLPRQ
ASLDNGSGLRYPGPGDAATDRKRSPSASRDPNQKYDQREGGDHAQYAPTDGSMPRSPSDY
GDGDPRRAPRGSHMYAEAARMGYRERRGPGRWHSQEYPLDQELDGPPSEYELQRQRQEEF
QARYRSDPNLARYPVKPQPYEEQMRMHAEVGRLRHERRHSDVSLAYTELDEPGPIRLSRQ
HLTERRPPMVGQRSYSVDRSSPGPMGPGLGGHRTSNHSPPTPHRSPVLGERSGDLRRGDL
GSDPMRRQQHHLDPSSAVRKTKREKMESMLRNDSLSSDQSESVRPPPPKPHKTKKGGKMR
QVSLSSSEEELATTPEYTSCEDVEIESESVSEKGESQRGKRKTFEQTFMSEPTHTLSERQ
KKMVRFGGHSFEEDLTWCEPQVKDSGVDTCSSTTLNEEHSHSEKHLVTWQPSKDGDRLIG
RILLNKRMKDGSVPRDSGALLGLKVVGGKMTESGRLCAFITKVRKGSLADTVGHLRPGDQ

VLEWNGKLLQGATFKEVYNIILESKPEPQVELVVSRPLGDIPRIPDSTHAQLESSSSSFE
SQKMDRPSISVTSPMSPSMLRDAPQYLSGQLSVKLWYDKVGHQLIVTILGAKDLPPREDG
RPRNPYVKIYFLPDRSDKSKRRTKTVKKSLEPKWNQTFMYSPVHRREFRERMLEITLWDQ
ARVREEESEFLGEILIELETALLDDEPHWYKLQTHDVSSIPLPRASPNSQRRQLHGESPT
RRLQRSQRISDSEISDYDCEDGVGLFVSEYRSNGRDFQSSTLSVPEQVMSSNHCSRSADI
NRARSRSPSVPPPSRRVEKCGPGSSDMSKMCLSLQRTWQHAGMARAYSEGAYTSDYDYER
NHGAMDRHEYHSRSRSADQRPTIERPTCRSRSTERPHDSSLMRSMPSLPSGRSAPPSPAL
TRAHPRSGSVQTSPTSTPMSGRRGRQLPQLPPTGKDRKAGGKKLRSTIQRSTETGLAVEM
RSRMTRQASRESTDGSMNSYSSEGNLIFPGVRLSSDAQFSDFLDGLGPAQLVGRQTLATP
PMGDIQIGMVEKKGALEVEVIRARGLVGKPGSKSLPAPYVKVYLLENGVCIAKKKTKVAR
KTLDPLYQQQLPFEESPGGKVLQVIVWGDYGRMDHKSFMGAVQILLDELDLSNMVIGWFK
LFPPSSLVDPTLAPLTRRASQSSLDSFSRS

>Tetraodon nigroviridis_RIMS2(ENSTNIP_11042)
AAAAAAAAASAAGAVPEMPDLSHLTEEERKIILGVMERQKNEEAKEQSMLNLGCKTNFAS
TMVGAGTRSEKEQMPSEKPLLIYGLCFRIKEEPKPQPAQPGRAQKLHQQFESYKEQVKKM
GEETKPAQDQKSEAPNCGICHKTKFADGCGHLCSYCQTKFCARCGGRVSLRSNKEDKVVM
WVCNLCRKQQEILTKSGAWFYGSGPGQVRGVFEGGPQGKKAKLQDPSRLRNPLSAVVSQN
TLSILSPKPRRLECGETSSTVEPSSAKAKKDRTEKKPIKTQRAPDLGVMNLGSGNNKSCR
KRSPSASRDPNQNSAVRKTKREKMESMLRNDSLSSDQSESVRPPPPKPHKTKKGGKMRQV
SLSSSEEELATTPEYTSCEDVEIESESVSEKGDLDGHWWDHSSWHSSEASPMSLHLVTWQ
PSKDGDRLIGRILLNKRMKDGSVPRDSGALLGLKVVGGKMTESGRLCAFITKVRKGSLAD
TVGHLRPGDQVLEWNGKLLQGATFKEVYNIILESKPEPQVELVVSRPLGDIPRIPDSTHA
QLESSSSSFESQKMDRPSISVTSPMSPSMLRDAPQYLSGQLSVKLWYDKVGHQLIVTILG
AKDLPPREDGRPRNPYVKIYLLPDRSDKSKRRTKTVKKSLEPKWNQTFMYSPVHRREFRE
RMLEITLWDQARVREEESEFLGEILIELETALLDDEPHWYKLQTHDVSSIPLPRASPNSQ
RRQLQSPTPRLQRSQRISDSEISDYDCEDGVGVITVISDYRSNGRDFQSSTLSVPEQVMS
SNHCSRSADINRPRSRSPSVPHQRPTIERPTCRSRSTERPHDSSLMRSMPSLPSGRSAPP
SPALTRAHPRSGSVQTSPTSTPMSGRRGRQLPQLPPMGKDRKKKSLDMEERTRQMKLKMN
KYKPGAGSDSRLEQEYHKHAQYTFFRDPRGSDNLSAKSSDSDVSDVSAVSRTSSASRFSS
TSYMSVQSERPQGNRKIRDFASKMKNRQMGSSSGMNMTKSTSISGDMCNLEKTDGSQSDT
AVGTVGTDDKKRRSSIGAKMQAMVGMSRKSQSTSQLSQTEAGGKKLRSTIQRSTETGLAV
EMRSRMTRQASRESTDGSMNSYSSEGNLIFPGVRLSSDAQFSDFLDGLGPAQLVGRQTLA
TPPMGDIQIGMVEKKGALEVEVIRALCALWGNQGSKSLPAPYVKVYLLENGVCIAKKKTK
VARKTLDPLYQQLLPFEESPGGKVLQVIVWGDYGRMDHKSFMGAVQILLDELDLSNMVIG
WFKLFPPSSLVDPTLAPLTRRASQSSLDSFSRS

>Gasterosteus aculeatus_RIMS2(ENSGACP_6381)
MSAPHGPRGGPSAAGAMPEMPDLSHLTEDERKIILGVMDRQKKEEAKEQTMLKRRRRERA
GSGQWPYPWSGRLLGWPALMQPDQRKNQRLQPAQKLHQQFESYKDQVKKMGEETKPTQEL
KSEAPTCGICHKTKFADGCGHLCSYCQTKFCARCGGRVSLRSNKVMWVCNLCRKQQEILT
KSGAWFFDPGPGQVRGVFEGGPQGKKAKLQDPSLYPYQGASGDLTDRSRPHGGLLPRQAS
LDNGAGLRYSAPGDAPIDRKQSPSASRDPNQKYDQREGVDHSQYAPTDGGMPRSPSDYGD
GDHRRAPRGPHMYPDAEAARMGYRGPGRWHSQEYPLDQELDGPPSEYDLQRQRQEEFQTR
YRSDPNLARYPVKPQPYEEQMRMHAEVGRVRHERRHSDVSLAYTEQDDPGPVRLSRQHLT
ERRPPMVGQRSYSVDRSSPGPMGPGLGGHRTSNHSPPTPHRSPVLGDRSGDLRRGDLGVG
GDSMRRQQHHLDPSSAVRKTKREKMESMLRNDSLSSDQSESVRPPPPKPHKTKKGGKMRQ
VSLSSSEEELATTPEYTSCEDVEIESESVSEKGDSQRGKRKTIEQTYMSEPTHTLSERQK
KMVRFGGHSFEEDLAWCEPQVKDSGVDTCSSTTLNEEHSHSEKHPVTWQPSKDGDRLIGR
ILLNKRMKDGSVPRDSGALLGLKVVGGKMTESGRLCAFITKVRKGSLADTVGHLRPGDQV
LEWNGKLLQGATFKEVYNIILESKPEPQVELVVSRPIGDIPRIPDSTHAQLESSSSSFES
QKMDRPSISVTSPMSPSMLRDAPQYLSGQLSVKLWYDKVGHQLIVTILGAKDLPAREDGR
PRNPYVKIYFLPDRSDKSKRRTKTVKKSLEPKWNQTFMYSPVHRREFRERMLEITLWDQA
RVREEESEFLGEILIELETALLDDEPHWYKLQTHDVSSIPLPRASPNTQRRQLHGESPTR
RLQRSQRISDSEISDYDCEDGVGVITDYRPNGRDFQSSTLSVPEQVMSSNHCSRSADINR
ARSRSPSVPPPSSRSLDQAFDLRPSQYSSSARVDHHSVSEDNYSPDSRGRAHPRSGSVQT
SPTSTPASNRRGRQLPQLPPTGKDRNSCMDMEERTRQMKLKMNKYKQGAGSDPRLEQDYH
KEYRSGRDPRASDNLSAKSSDSDVSDVSAVSRTSSASRFSSTSYMSVQSERPQGNRKIRD
FASKMKNRQMGSSGGVNMTKSTSVSGDMCNLEKTDGSQSDTAVGTVGTDDKKRRSSIGAK
MQAMVGMSRKSRSTSQLSQTETGGKKLRKEIHRSTETGLAMEMRSRMTRQASREYTDGSM
NSYSSEGNLIFPGVRLSSEAQFSDFLDGLGPAQLVGRQTLATPPMGDIQIGMVDKKGALE

VEIIRARGLVGKPGSKALPAPYVKVYLLENGVCIAKKKTKVARKTLDPLYQQQLPFEESP
GGKVLQVIVWGDYGRMDHKSFMGAVQILLDELDLSNMVIGWFKLFPPSSLVDPTLAPLTR
RASQSSLDSFSRS

>Oryzias latipes_RIMS2(ENSORLP_3004)
RFKEEPKPQPAQWFSGITELVNNVLQPQQKPQNDKEPEADTKLHQQFESYKDQVKKMGEE
TKPTQDQKSEAPTCGICHKTKFADGCGHLCSYCQTKFCARCGGRVSLRSNKVMWVCNLCR
KQQEILTKSGEWFYSSGPGQVRGVFEGGPQGKKAKLQDPSLYPHQGVSGDLRPSSDRSRP
HGGLLPRQASLDNDTGLRYSGSGDPSLDRKQSPSASRDPNQKYDQREGGNHPQYAPADGG
MPRSPSDYGDRNSRQTSRGPHMYPDVDAMRMGFRDRRGPGRWHSQEYPLDQELDGPPTEY
DLQRQRQEEYQARYRSDPNLARYPVKPQPYEEQMRMHAEVGRLRHERRHSDVSLAYTEQD
DPGPIRLSRQHFTERRPPMVGQRSYSVDRSSPGPMGPSLGSHRTSNHSPPTPRHSPVLGD
RQDQRRGDFGGDAMRRQQHHLDPSSAVRKTKREKMESMLRNDSLSSDQSESVRPPPPKPH
KTKKGGKMRQVSLSSSEEELATTPEYTSCEDVEIESESVSEKGESQRGKRKTIEQAFMSE
PTHTLSERQKKMVRFGGHSFEEDLAWCEPQVKDSGVDTCSSTTLNEEHSHSEKHPVTWQP
SKDGDRLIGRILLNKRMKDGSVPRDSGALLGLKVVGGKMTESGRLCAFITKVRKGSLADT
VGHLRPGDQVLEWSGRLLQGATFKEVYNIILESKPEPQVELVVSRPIGDIPRIPDSTHAQ
LESSSSSFESQKMDRPSISVTSPMSPRTLRDAPQYLSGQLSVKLWYDKVGHQLIVTILGA
KDLPPREDGRPRNPYVKIYFLPDRSDKSKRRTKTVKKSLEPKWNQTFMYSPVHRREFRER
MLEITLWDQARVREEESEFLGEILIELETALLDDEPHWYKLQTHDVSSIPLPRASPNSQR
RQLHRESPTRRLQRSQRISDSEISDFDCEEGVGVVTDYRSNGRDFQSSTLSVPEQVMSSN
HCSRSADIARARSRSPSVPPLSRRESEQANGFRDKTNHFLTLPRTRHTQLIDDPEKDPRN
HGAMDRHEYHSRSRSADQRPVTERLAPRSHSTERPHDSSIMRSMPSLPSGRSAPPSPAMT
RAHPRSGSVQISPTGTPMSSRRGRQLPQLPPTGMDRKAGGKKLRSTIQRSTETGLAVEMR
SRMTRQASRESTDGSMNSYSSEGNLIFPGVRLSSDAQFSDFLDGLGPAQLVGRQTLATPS
MGDIQIGMVDKKGALEVEIIRARSLVGKPGSKALPAPYVKVYLLENGVCIAKKKTKVARK
TLDPLYQQQLPFEESPGGKVLQVIVWGDYGRMDHKSFMGAVQILLDELDLSNMVIGWYKL
FPPSSLVDPTLAPLTRRASQSSLDSFSRS

>Homo sapiens_RIMS4(ENSP_439287)
MERSQSRLSLSASFEALAIYFPCMNSFDDEDAEGDSRRLKGAIQRSTETGLAVEMPSRTL
RQASHESIEDSMNSYGSEGNLNYGGVCLASDAQFSDFLGSMGPAQFVGRQTLATTPMGDV
EIGLQERNGQLEVDIIQARGLTAKPGSKTLPAAYIKAYLLENGICIAKKKTKVARKSLDP
LYNQVLLFPESPQGKVLQVIVWGNYGRMERKQFMGVARVLLEELDLTTLAVGWYKLFPTS
SMVDPATGPLLRQASQLSLESTVGPCGERS

>Macaca mulatta_RIMS4(ENSMMUP_13905)
MERSQSRLSLSASFEALAIYFPCMNSFDDEDAGDSRRLKGAIQRSTETGLAVEMPSRTLR
QASHESIEDSMNSYGSEGNLNYGGVCLASDAQFSDFLGSMGPAQFVGRQTLATTPMGDVE
IGLQERNGQLEVDIIQARGLTAKPGSKTLPAAYIKAYLLENGVCIAKKKTKVARKSLDPL
YNQVLLFPESPQGKVLQVIVWGNYGRMERKQFMGVARVLLEELDLTTLAVGWYKLFPTSS
MVDPATGPLLRQASQLSLESTVGPCGERS

>Mus musculus_RIMS4(ENSMUSP_45637)
MERSQSRLSLSASFEALAIYFPCMNSFDDEDAADSRRLKGAIQRSTETGLAVEMPSRTLR
QASHESIEDSMNSYGSEGNLNYGGVCLASDAQFSDFLGSMGPAQFVGRQTLATTPMGDVE
IGLQERNGQLEVDIIQARGLTAKPGSKTLPAAYIKAYLLENGVCIAKKKTKVARKSLDPL
YNQVLLFPESPQGKVLQVIVWGNYGRMERKQFMGVARVLLEELDLTTLAVGWYKLFPTSS
MVDPATGPLLRQASQLSLESTVGPCGERS

>Felis catus_RIMS4(ENSFCAP_10849)
EGDSRRLKGAIQRSTETGLAVEMPSRTLRQASHESIEDSMNSYGSEGNLNYGGVCLASDA
QFSDFLGSMGPAQFVGRQTLATTPMGDVEIGLQERNGQLEVDIIQARGLTAKPGSKTLPA
AYIKAYLLENGVCIAKKKTKVARKSLDPLYNQVLLFPESPQGKVLQVIVWGNYGRMERKQ
FMGVARVLLEELDLTTLAVGWYKLFPTSSMVDPAAGPLLRQASQLSLESTVGPCGERS

>Dasypus novemcinctus_RIMS4(ENSDNOP_20169)
MNSFDDEDAGDSRRLKGAIQRSTETGLAVEMPSRTLRQASHESIEDSMNSYGSEGNLNYG
GVCLASDAQFSDFLGSMGPAQFVGRQTLATTPMGDVEIGLQERNGQLEVDIIQARGLTAK
PGSKTLPAAYIKAYLLENGICVAKKKTKVARKSLDPLYNQVLLFPESPQGKVLQVIVWGN
YGRMERKQFMGVARVLLEELDLTTLAVGWYKLFPTSSMVDPAAGPLLRQASQLSLESTVG

PCGERS

>Gallus gallus_RIMS4(ENSGALP_6585)
MNSFDEEDADGDGRRLKGAIQRSTETGLAVEMPSRTVRQASHESIEDSMNSYGSEGNLNF
SGVHLASAGQFSDFLGSMGPAQFVGRQTLATTSMGDVEIGLQERNGQLEVDIIQARGLTP
KPGSKTLPAAYIKAYLLENGVCIAKKKTKVARKSLDPLYNQVLLFPESPQGKVLQVIVWG
NYGRMERKHFMGVARVLLEELDLSTLAIGWYKLFPTSSMVDPTTGPLLRQSSQLSLESTV
GPCCDRS

>Pelodiscus sinensis_RIMS4(ENSPSIP_14824)
MPSRTVRQASHESIEDSMNSYGSEGNLNFSGVHLASAGQFSDFLGSMGPAQFVGRQTLAT
TSMGDVEIGLQERNGQLEVDIIQARGLTPKPGSKTLPAAYIKAYLLENGVCIAKKKTKVA
RKSLDPLYNQVLLFAESPQGKVLQVIVWGNYGRMERKYFMGVARVLLEELDLSTLAIGWY
KLFPTSSMVDPTTGPLLRQSSQLSLESTVGPCCDRS

>Danio rerio_RIMS4(ENSDARP_77256)
MERSQSRLSLSASFEALAIYFPCMNSFDEDDGDPEGRRLKGIQRSTETGLAVEMPSRTVR
QASHESIEDSMNSYGSEGNLNYSGMCLASDAQFSDFLDGMGPAQFVGRQTLATTSMGDVE
ISLMERSGQLEVEVIQARGLIMKPGSKGPPAAYIKVYLLENGICIAKKKTKSVRKSLDPL
YNQVLVFSESPQGKVVQVIVWGNYGRMDRKCFMGVARILLEELDLSSMVIGWYKLFPTSS
MVDPTMAPLIRHSSQLSLESTVGPCCERS

>Takifugu rubripes_RIMS4(ENSTRUP_40546)
EGRRLKGAIQRSTETGLAVDMPSRTIRQASHESIEDSMNSYGSEGNLTYSGMCLASDAQF
SDFLDGMGPAQFVGRQTLATTSMGDVEIGLLERNGGLEVEVVQARGLTMKSGSKTPPAAY
IKVYLLENGVCVAKKKTKSVRKSLDPLYNQVLVFPESPQGKVVQLIVWGNYGRMDRKCFM
GVARILLEELDLSTMVIGWYKLFPTSSMVDPKMAPLIRHSSQMSLESTLGPCCERS

>Tetraodon nigroviridis_RIMS4(ENSTNIP_6738)
QPRLAKPADPGPTAGLYMERSQSRISLSASFEALAIYFPCMNSFDEDDGVDAEGRRLKGA
IQRSTETGLAVEMPSRTIRQASHESIEDSMNSYGSEGNLTYSGMCLASDAQFSDFLDGMG
PAQFVGRQTLATTSMDITGDVEIGLLERNGGLEVEVVQARGLTMKPGSKTPPAAYIKVYL
LENGICVAKKKTKSVRKSLDPLYNQVLVFSESPQGKVVQLIVWGNYGRMDRKCFMGVARI
LLEELDLSTMAIGWYKLFPTSSMVDPKMAPLIRHSSQMSLESTLGPCCER

>Gasterosteus aculeatus_RIMS4(ENSGACP_4606)
MERSQSRISLSASFEALAIYFPCMNSFDEDDGVDTEGRKLKGAIQRSTETGLAVEMPSRT
VRQASHESIEDSMNSYGSEGNLNYSGMCLASDAQFSDFLDGMGPAQFVGRQTLATTSMGD
VEIGLMERNGALEVEVVQARGLTMKAGSKGPPAAYIKVYLLENGTCVAKKKTKSVRKSLD
PLYNQVLVFSESPQGKVVQVIVWGNYGRMDRKCFMGVARILLEELDLSTMVIGWYKLFPT
SSMVDPAMAPLIRHSSQMSLESTIGPCCERS

>Oryzias latipes_RIMS4(ENSORLP_11935)
DGRKLKGGIQRSTETGLAVEMPSRTVRQASHESIEDSMNSYGSEGNLNYSGMCLASDAQF
SDFLDGMGPAQFVGRQTLATTSMGDVEIGLMERNGGLEVEVVQARGLTMKPGSKAPPAAY
IKVYLLENGVCVAKKKTKSVRKSLDPLYNQVLVFSESPQGKVVQVIVWGNYGRMDRKCFM
GVARILLEELDLTAMVIGWYKLFPPSSMVDPTMGPLIRHSSQMSLESTIGPCCDRS

>Homo sapiens_RIMS1(ENSP_428417)
MSSAVGPRGPRPPTVPPPMQELPDLSHLTEEERNIIMAVMDRQKEEEEKEEAMLKCVVRD
MAKPAACKTPRNAENQPHQPSPRLHQQFESYKEQVRKIGEEARRYQGEHKDDAPTCGICH
KTKFADGCGHLCSYCRTKFCARCGGRVSLRSNNEDKVVMWVCNLCRKQQEILTKSGAWFF
GSGPQQTSQDGTLSDTATGAGSEVPREKKARLQERSRSQTPLSTAAASSQDAAPPSAPPD
RSKGAEPSQQALGPEQKQASSRSRSEPPRERKKTPGLSEQNGKGALKSERKRVPKTSAQP
VEGAVEERERKERRESRRLEKGRSQDYPDTPEKRDEGKAADEEKQRKEEDYQTRYRSDPN
LARYPVKPPPEEQQMRMHARVSRARHERRHSDVALPRTEAGAALPEGKAGKRAPAAARAS
PPDSPRAYSAERTAETRAPGAKQLTNHSPPAPRHGPVPAEAPELKAQEPLRKQSRLDPSS
AVLMRKAKREKVETMLRNDSLSSDQSESVRPSPPKPHRSKRGGKKRQMSVSSSEEEGVST
PEYTSCEDVELESESVSEKGDLDYYWLDPATWHSRETSPISSHPVTWQPSKEGDRLIGRV
ILNKRTTMPKDSGALLGLKVVGGKMTDLGRLGAFITKVKKGSLADVVGHLRAGDEVLEWN
GKPLPGATNEEVYNIILESKSEPQVEIIVSRPIGDIPRIPESSHPPLESSSSSFESQKME

RPSISVISPTSPGALKDAPQVLPGQLSVKLWYDKVGHQLIVNVLQATDLPARVDGRPRNP
YVKMYFLPDRSDKSKRRTKTVKKILEPKWNQTFVYSHVHRRDFRERMLEITVWDQPRVQE
EESEFLGEILIELETALLDDEPHWYKLQTHDESSLPLPQPSPFMPRRHIHGESSSKKLQR
SQRISDSDISDYEVDDGIGVVPPVGYRSSARESKSTTLTVPEQQRTTHHRSRSVSPHRGN
DQGKPRSRLPNVPLQRSLDEIHPTRRSRSPTRHHDASRSPVDHRTRDVDSQYLSEQDSEL
LMLPRAKRGRSAECLHTTRHLVRHYKTLPPKMPLLQSSSHWNIYSSILPAHTKTKSVTRQ
DISLHHECFNSTVLRFTDEILVSELQPFLDRARSASTNCLRPDTSLHSPERERGRWSPSL
DRRRPPSPRIQIQHASPENDRHSRKSERSSIQKQTRKGTASDAERVLPTCLSRRGHAAPR
ATDQPVIRGKHPARSRSSEHSSIRTLCSMHHLVPGGSAPPSPLLTRMHRQRSPTQSPPAD
TSFSSRRGRQLPQVPVRSGSIEQASLVVEERTRQMKMKVHRFKQTTGSGSSQELDREQYS
KYNIHKDQYRSCDNVSAKSSDSDVSDVSAISRTSSASRLSSTSFMSEQSERPRGRISSFT
PKMQGRRMGTSGRSIMKSTSVSGEMYTLEHNDGSQSDTAVGTVGAGGKKRRSSLSAKVVA
IVSRRSRSTSQLSQTESGHKKLKSTIQRSTETGMAAEMRKMVRQPSRESTDGSINSYSSE
GNLIFPGVRLGADSQFSDFLDGLGPAQLVGRQTLATPAMGDIQIGMEDKKGQLEVEVIRA
RSLTQKPGSKSTPAPYVKVYLLENGACIAKKKTRIARKTLDPLYQQSLVFDESPQGKVLQ
VIVWGDYGRMDHKCFMGVAQILLEELDLSSMVIGWYKLFPPSSLVDPTLTPLTRRASQSS
LESSTGPPCIRS

>Pongo abelii_RIMS1(ENSPPYP_18764)
MSSAVGPRGPRPPTVPPPMQELPDLSHLTEEERNIIMAVMDRQKEEEEKEEAMLKCVVRD
MAKPAACKTPRNAENQPHQPSPRLHQQFESYKEQVRKIGEEARRYQGEHKDDAPTCGICH
KTKFADGCGHLCSYCRTKFCARCGGRVSLRSNNDKYVMWVCNLCRKQQEILTKSGAWFFG
SGPQQTSQDGTLSDTATGAGSEVPREKKARLQERSRSQTPLSTAAASSQDAAPPSAPPDR
SKGADPSQQALGPEQKQASSRSRSEPPRERKKTPGLSEQNGKGALKSERKRVPKTSAQPV
EGAVEERERKERRESRRLEKGLSQDYPDTPEKRDEGKAADEEKQRKEEDYQTRYRSDPNL
ARYPVKPPPEEQQMRMHARVSRARHERRHSDVALPRTEAGAALPEGKVGKRAPAAARASP
PDSPRAYSAERTAETRAPGAKQLTNHSPPAPRHGPVPAEAPELKAQEPLRKQSRLDPSSA
VLMLRNDSLSSDQSESVRPSPPKPHRSKRGGKKRQMSVSSSEEEGVSTPEYTSCEDVELE
SESVSEKGDLDYYWLDPATWHSRETSPISSHPVTWQPSKEGDRLIGRVILNKRTTMPKDS
GALLGLKVVGGKMTDLGRLGAFITKVKKGSLADVVGHLRAGDEVLEWNGKPLPGATNEEV
YNIILESKSEPQVEIIVSRPIGDIPRIPESSHPPLESSSSSFESQKMERPSISVISPTSP
GALKDAPQVLPGQLSVKLWYDKVGHQLIVNVLQATDLPARVDGRPRNPYVKMYFLPDRSD
KSKRRTKTVKKILEPKWNQTFVYSHVHRRDFRERMLEITVWDQPRVQEEESEFLGEILIE
LETALLDDEPHWYKLQTHDESSLPLPQPSPFMPRRHIHGESSSKKLQRSQRISDSDISDY
EVDDGIGVVPPVGYRSSARESKTTTLTVPEQQRTTHHRSRSVSPHRGNDQGKPRSRLPNV
PLQRSLDEIHPTRRSRSPTRHHDASRSPVDHRTRDVDSQYLSEQDSELLMLPRAKRGRSA
ECLHTTRHLVRHYKTLPPKMPLLQSSSHWNIYSSVLPAHTKTKSVTRQDISLHHECFNSR
VLRFTGRWSPSLDRRRPPSPRIQIQHASPENDRHSRKSERSSIQKQTRKGTASDAERVLP
TCLSRRGHAAPRATDQPVIRGKHPARSRSSEHSSIRTLCSMHHLVPGGSAPPSPLLTRMH
RQRSPTQSPPADTSFSSRRGRQLPQVPVRSSSIEQASLVVEERTRQMKMKVHRFKQTTGS
GSSQELDREQYSKYNIHKDQYRSCDNVSAKSSDSDVSDVSAISRTSSASRLSSTSFMSEQ
SERPRGRISSFTPKMQGRRMGTSGRSIMKSTSVSGEMYTLEHNDGSQSDTAVGTVGAGGK
KRRSSLSAKVVAIVSRRSRSTSQLSQTESGHKKLKSTIQRSTETGMAAEMRKMVRQPSRE
STDGSINSYSSEGNLIFPGVRLGADSQFSDFLDGLGPAQLVGRQTLATPAMGDIQIGMED
KKGQLEVEVIRARSLTQKPGSKSTPAPYVKVYLLENGACIAKKKTRIARKTLDPLYQQSL
VFDESPQGKVLQVIVWGDYGRMDHKCFMGVAQILLEELDLSSMVIGWYKLFPPSSLVDPT
LTPLTRRASQSSLESSTGPPCIRS

>Oryctolagus cuniculus_RIMS1(ENSOCUP_15419)
RKKAPGLSEQNGKGALKSERKRVPKTLAQPGEVAAEERERKERRESRRLEKGRSQDYPDM
PDKREEAKAAAKEEKQRKEEGLPGPGIAATRNLARTPVKPPARRGSRCXESKAGKRAPAV
TRASPPDSPRAYAAERTAETRAPGAKALTNHTPPAPRHGPGPAEAPELKAQEPLRKQSRL
DPSSAVLIRKAKREKVETMLRNDSLSSDQSESVRPSPPKPHRSKRGGKKRQMSVSSSEEE
GVSTPEYTSCEDVELESESVSEKGDLDYYWLDPATWHSRETSPISSHPVTWQPSKEGDRL
IGRVILNKRTTMPKESGALLGLKVVGGKMTDLGRLGAFITKVKKGSLADVVGHLRAVQGD
EVLEWNGKPPGATNEEVYNIILESKSEPQVEIIVSRPIGDIPRIPESSHPPLESSSSSFE
SQKMERPSISVISPTSPGALKDAPQVLPGQLSVKLWYDKVGHQLIVNVLQATDLPPRVDG
RPRNPYVKMYFLPDRSDKSKRRTKTVKKVLEPKWNQTFVYSHVHRRDFRERMLEITVWDQ
PRVQEEESEFLGEILIELETALLDDEPHWYKLQTHDESSLPLPQPSPFMPRRHIHGESSS
KKLQRSQRISDSDISDYEVDDGIGVVPPVSYRSSARESKSTTLTVPEQQRTTHHRSRSVS
PHRGDDQGRPRSRLPNVPLQRSLDEIHPTRRSRSPTRHHDASRSPVDHRSRDVDSQYLSE

QDSELLMLPRAKRGRSAECLHTTRHLVRHYKTLPPKMPLLQNGSHWNIYSSIMPTHSKTK
SVTRQDNPLHHEYFTSRVLLRFTDEILASELQPSLDRARSASTNCLRPDTSLHSPERERA
NRGRWSPSLDRRRPPSPRIQIQHASPENDRHSRKSERSSIQKQTRKGTASDTERVLPPCL
SRRGHAAPRTADQPGIRGKHPARSRSSEHSSVRTLCSMHHLAPGGSAPPSPLLTRMHRQG
SPTQSPPADVSFSNRRGRQLPQVPVRSSSIEQASLVVEERTRQMKMKVHRFKQTTGSGSS
QELDREQYSKYNIHKDQYRSCDNVSARSSDSDVSDVSAISRTSSASRLSSTSFMSEQSER
PRGRIRYSSFTPKMQGRRMGTSGRAITKSTSVSGEMYTLEHNDGSQSDTAVGTVGAGGKK
RRSSLSAKVVAIVSRRSRSTSQLSQTESGHKKLKSTIQRSTETGMAAEMRKMVRQPSRES
TDGSINSYSSEGNLIFPGVRLGADSQFSDFLDGLGPAQLVGRQTLATPAMGDIQIGMEDK
KGQLEVEVIRARSLTQKPGSKSTPAPYVKVYLLENGACIAKKKTRIARKTLDPLYQQSLV
FDESPQGKVLQVIVWGDYGRMDHKCFMGVAQILLEELDLSSMVIGWYKLFPPSSLVDPTL
TPLTRRASQSSLESSTGPPCIRS

>Canis familiaris_RIMS1(ENSCAFP_3850)
RLHQQFESYKEQVRKIGEEARRYQGEHKDDAPTCGICHKTKFADGCGHLCSYCRTKFCAR
CGGRVSLRSNNEDKVVMWVCNLCRKQQEILTKSGAWFFGSGPQQPSQDGTLSDTATGAGS
EVPREKKARLQERSRSQTPLSTAAASSQDTAPPSAQPDRSKGAESSQQAVGPEQKQVSSR
SRSEPPRERKKTAGLSEQNGKGAQKSERKRAPKSSVQPGEGAVEERERKERRESRRLEKG
RSQDFPDVPEKREDRKAAEDEKQRKEEEYQTRYRSDPNLARYPVKPPPEEQQMRMHARVS
RARHERRHSDVALPRTEAGAPPPDSKAGKRAPAAARASPPDSPRAYSAERTAEARAPGAK
QLTNHSPPAPRHGPVPAEALEPKAQEPLRKQSRLDPSSAVLIRKSKREKVETMLRNDSLS
SDQSESVRPSPPSRTGRSNGVRKRMICLVNSSESEIVSIYKFTKCQEIEHSEGEIVYGAG
DLDYYWLDPATWHSRETSPISSHPVTWQPSKEGDRLIGRVILNKRTTMPKESGALLGLKV
VGGKMTDLGRLGAFITKVKKGSLADVVGHLRAVQGDEVLEWNGKPLPGATNEEVYNIILE
SKSEPQVEIIVSRPIGDIPRIPESSHPPLESSSSSFESQKMERPSISVISPTSPGALKDA
PQVLPGQLSVKLWYDKVGHQLIVNVLQATDLPTRVDGRPRNPYVKMYFLPDRSDKSKRRT
KTVKKVLEPKWNQTFVYSHVHCRDFRERMLEISVWDQPRVQEEESEFLGEILIELETALL
DDEPHWYKLQTHDESSLPLPQPSPFMPRRHIHGESSSKKLQRSQRISDSDISDYEVDDGI
GVVPPVGYRSSARESKSTTLTVPEQQRTTHHRSRSVSPHRGDDQGRPRSRLPNVPLQRSL
DEIHPTRRSRSPTRHHDASRSPVDHRSRDVDSQYLSEQDSELLMLPRAKQGISCMFVLPC
SRHFVRHYKTLPPKMPLLENGSHWNIYSSVLPAHTKTKSVTRQDFPLHHECFHSRVLRFT
DEILVSELQPSLDRARSASTNCLRPDTSLHSPERESLVHWANRGRWSPSLDRRRPPSPRI
QIQHASPENDRHSRKSDRSSIQKQTRKGTASDAERVLPPCLSRRGYAALRATDQPVLRGK
HPARSRSSEHSSVRALCSTHHLAPGGSAPPSPLLTRMHRQASPTHPPPSDTSFSSRRGRQ
LPQVPVRSSSIEQASLVVEERTRQMKMKVHRFKQTTGSGSSQELDREQYSKYSIHKDQYR
SCDNVSAKSSDSDVSDVSAISRTSSASRLSSTSFMSEQSEHPRGRIRYSSFTPKMQGRRM
GTSGRAITKSTSVSGEMYTLEHNDGSQSDTAVGTVGAGGKKRRSSLSAKVVAIVSRRSRS
TSQLSQTESGHKKLKSTIQRSTETGMAAEMRKMVRQPSRESTDGSINSYSSEGNLIFPGV
RLGADSQFSDFLDGLGPAQLVGRQTLATPAMGDIQIGMEDKKGQLEVEVIRARSLTQKPG
SKSTPAPYVKVYLLENGACIAKKKTRIARKTLDPLYQQSLVFDESPQGKVLQVIVWGDYG
RMDHKCFMGVAQILLEELDLSSMVIGWYKLFPPSSLVDPTLTPLTRRASQSSLESSTGPP
CIRS

>Ornithorhynchus anatinus_RIMS1(ENSOANP_2403)
RLHQQFESYKEQVRKIGEEARRYQGEHKDDAPTCGICLKTKFADGCGHLCSYCRTKFCAR
CGGRVSLRSNNEDKVVMWVCNLCRKQQEILTKSGAWFFGSGPQQPPSQDGTLSDTTTGVD
APREKKARLQERSRSQTPLSTAAVSAQEKIPSGAQPDRSKRAEPSQQIMGPEQKQASSRS
RSEPPRESRKKTAVGSEQNGKGALKSERKRVPKSSLQQGEAPLDERERKERRETRRLEKG
RSQDVPEKRDKGKGGDDEKQRKEEEYQTRYRSDPNLARYPVKPHPEEQQMRMHATVSKAR
HERRHSDVSLPHTEMEAAEAPENKLGKHSQVPGRISVSKQLTNHSPPAPRHGPAPSEHLE
SKSQESLKKQSRLDPSSAILVRKAKREKMETMLRNDSLSSDQSESVRPSPPKPHRSKRGG
KKRQMSVSSSEEEGASTPEYTSCEDVEIESESVSEKGDLDYYWLDPATWHSRETSPISSH
PVTWQPSKEGDRLIGRVILNKRTTMPKEAGALLGLKVVGGKMTDLGRLGAFITKVKKGSL
ADVVGHLRAGDEVLEWNGKPLPGATNEEVYNIILESKSEPQVEIIVSRPIGDIPRIPESS
HPPLESSSSSFESQKMERPSISVISPTSPGALKDAPQVLPGQLSVKLWYDKVGHQLIVNV
LQATDLPPRVDGRHRNPYVKMYFLPSVSDKSKRRTKTVKKILEPKWNQTFLYSHVHRRDF
RERMLEITVWDQPRVQEEESEFLGEILIELETALLDDEPHWYKLQTHDESSLPLPQPSPF
MPRRHVHPESSSKKLQRSQRISDSDISDYEIDDGIGVVPPVGFRSNTRESKSATLTVPEQ
QRATHHRSRSVSPHRGDDQGRPRSRLPNVPLQRSLDEIHQMRRSRSPTRHHDASRSPVDD
RSRDMDDQYLSEQESELLMLPRAKRGRSAECLHTTSELQPSLDRARSASTNCLRPDTSLH
SPERERIHQQGSQGQSPPADTSFSNRRGRQLPQLPVRSGSLEQASLVVEERTRQMKMKVH

RYKQTTGSGSSQELDREQYSKYNLHKEQYRSCDNVSAKSSDSDVSDVSAISRTSSASRLS
STSFMSEQSERPRGRVRYSSFTPKMQGRRMGTSGRTITKSTSVSGEMYKLEHNDGSQSDT
AVGTVGTGGKKRRSSLSAKVVAIVSRRSRSTSQLSQTERGHKVL

>Gallus gallus_RIMS1(ENSGALP_25647)
MSSSAGPRGPRPPTVPPPMQELPDLSHLTEEERNIIMAVMDRQKEEEEKEEAMLKRLHQQ
FESYKEQVRKIGEEARRYQGEHKDDAPTCGICHKTKFADGCGHLCSYCRTKFCARCGGRV
SLRSNNEDKVYRNTVPTHCGSPVPVRVMWVCNLCRKQQEILTKSGAWFFGSGPQPSPSQD
GTLSDTATGASSDAPREKKARLQERSRSQTPLSTAAASSQEISPSSVQSDRRKGAEVSQP
AMGLDQKQVSSRSRSEPPRERKKTLVSDQNGKVVKSERKRVPKTSLQKEGPADDRERKER
HENRRLEKGKSQDYPDLPEKLEEGKVPDDEKQKKEDEYHTRYRSDPNLARYPVKPHPEEQ
QMRMHAKVSKARHERRHSDVALPHTEMEEAEVPENNLGKRSQLQGTQDRKSLVETQRSYS
IDRTGDVRISVSKQLTNHSPPTPRHSPVPIEHVEYKNHDTFKKQSRLDPSSAILMRKAKR
EKMETMLRNDSLSSDQSESVRPSPPKPHRAKRGGKKRQMSVSSSEEEGASTPEYTSCEDV
EIESESVSEKGDLDYYWLDPATWHSRETSPISSHPVTWQPSKEGDRLIGRVILNKRTTMP
KESGALLGLKVVGGKMTELGRLGAFITKVKKGSLADVVGHLRAGDEVLEWNGKPLPGATN
EEVYNIILESKSEPQVEIIVSRPIGDIPRIPESSHPPLESSSSSFESQKMERPSISVISP
TSPGALRDAPQVLPGQLSVKLWYDKVGHQLIVNVLQATDLPPRVDGRPRNPYVKMYFLPD
RSDKSKRRTKTVKKSLEPKWNQTFLYSHVHRRDFRERMLEITVWDQPRVQEEESEFLGEI
LIELETALLDDEPHWYKLQTHDESSLPLPQPSPFMPRRHVHGGESSSKKLQRSRPISDSD
ISDYDVDDGIGVVPPGYRSSTRESRSTTLTVPEQQRTTHHRSRSVSPHRGDDQGRTRSRL
PNVPSQRSLDEIHQMRRSRSPTRHHEASRSPADYRSRDMDSQYLSDQESELLMLPRAKRG
RSAECLHTISELQPSLDRARSASTNCLRPDTSLHSPERERGRWSPSLERRRPTSPRIHIQ
HASPEDDRQSRKVERYSSQKQTRKGSAAETERIHQQGSPTQSPPADTSFSSRRGRQLPQV
PVRSGSIEQASLVVEERTRQMKMKVHRYNQTSGSGSSQEHEREQYTKYNIQTDQYRSCDN
VSAKSSDSDVSDVSAISRTSSASRLSSTSFMSEQSERPRGRISTFTPKMQGRRMGTSGRI
TKSTSVSGEMYKLEHNDGSQSDTAVGMVGTGGKKRRSSLSAKVVAIVSRRSRSTSQLSQT
EAGNKKLKSTIQRSTETGMAAEMRSRMVRQPSRESTDGSINSYSSEGNLIFPGVRLGADS
QFSDFLDGLGPAQLVGRQTLATPAMGDIQIGMVDKKGQLEVEVIRARGLTQKPGSKSTPA
PYVKVYLLENGACIAKKKTRIARKTLDPLYQQTLVFDESPQGKVLQVIVWGDYGRMDHKC
FMGVAQILLEELDLSSVVIGWYKLFPPSSLVDPTLTPLTRRASQSSLESSTGPPCIRS

>Anolis carolinensis_RIMS1(ENSACAP_4113)
MSSSSSSSCSVGLRAPRPPAVPPPMPELPDLSHLTEEERNIILAVMDRQKEEEEKEEAML
KRLHQQFESYKEQVRKIGEESRRSQGDHKDDAPTCGICHKTKFADGCGHLCSYCRTKFCA
RCGGRVSLRSNNEDKVYRNNVSAGYGPLLEGPETVMWVCNLCRKQQEILTKSGAWFFGSG
PQQPPSQDGTLSDTATGGSSESPREKKARLQERSRSQTPLSTAAATSQEFSSGVQTDRRK
GTEASQQAMGLEQKQTSSRSRSEPPRERKKTSAAPEQNGKGGLKGERKRVPKSAPQQGEG
QTDERESKERRDGRRLEKGRSQDYSDFPPNFEEGKAIDEEKQRKEDDYHTRYRSDPNLAR
YPVKPHPEEQQMRMHAKVSKVRHERRHSDVALPHTEVEEPEVPENKLGKHPQLPATQDMK
SHPDTQRAYAIERTGDVRISVSKQLANHSSPTPRHSMVPTEHLESKNHDSFKTQSRLDPS
SAILSRKAKREKMETMLRNDSLSSDQSESVRPSPPKPHRSKRGGKKRQMSVSSSEEEGAS
TPEYTSCDDVEIESESVSEKGDLDYYWLDPATWHSRETSPISSHPVTWQPSKEGDCLIGR
VILNKRTTMPKESGALLGLKVVGGKMTELGRLGAFITKVKKGSLADVVGHLRAGDEVLEW
NGKPLPGATNEEVYNIILESKSEPQVEIIVSRPIGDIPRIPETSHPPLESSSSSFESQKM
ERPSISVISPTSPGALKDAPQVLPGQLSVKLWYDKVGHQLIVNVLQATDLPPRIDGRPRN
PYVKMYFLPDRSDKSKRRTKTVKKSLEPKWNQTFLYSHVHRRDFRERMLEITVWDQPRVQ
EEESDFLGEILIELETALLDDEPHWYKLQTHDESSLPLPQPSPFMPRRHAHGGENSSKKL
QRSQRISDSDFSDYDVEDGIGVVPSVGYRSSTRENKSTTLTVPEQQRTTHHRSRSVSPHR
GDEQGRTRSRLPNVPLQRSLDEIHQMRRSRSPTRHHDASRSPIDRRSRDMDSQYLSDQES
ELLMLPRAKRGRSAECLHTASELQPSLDRARSASTNCLRPDTSLHSPEQERHRWSPSLDR
RRPTSPRIHIQHASPEDDRTTPPLESCIPKQDTLNFLSANPEETQRHSRKVERYSNQKQT
RKVSASETERMHQPGSPMQSSPADTPFSSRRGRQLPQVPVRSGSIEQEQESYSPSSKANL
VVEERTRQMKMKMHRYNQTTGSGSSQELEREQYSKYDIQADQYRSCDNVSAKSSDSDVSD
VSAISRTSSASRLSSTSFMSEQSERPRGRISTFTPKMQGRRMGTSGRIITKSTSVSGEVY
KLEHNDGSQSDTAVGTLGTGGKKRRSSLSAKVVAMVSKRSRSTSQLSQTETGSKKLKSTI
QRSTETGMAAEMRSRMVRQPSRESNDGSINSYSSEGNLIFPGVRLGADSQFSDFLDGLGP
AQLVGRQTLATPAMGDIQIGMVDKKGQLEVEVIRARGLTQKPGSKSTPAPYVKVYLLENG
ACVAKKKTRIARKTLDPLYQQTLVFEESPHGKVLQVIVWGDYGRMDHKCFMGVAQILLEE
LDLSSVVIGWYKLFPPSSLVDSTLTPLTRRASQSSLESSTGPPCIRS

>Xenopus tropicalis_RIMS1(ENSXETP_4933)
MLKRLHQQFESYKEQIRKIGEDTRRYPGEHKDDAPTCGICHKTKFADGCGHLCSYCRTKF
CARCGGRVSLRSNNVMWVCNLCRKQQEILTKSGDWFFGSGVQQPSSQDGTLSDTATGAST
DAPREKKARLQERSRSQTPLSTAAVAGSQGTPPSSLQSDRSKGSEPSLQSSVQEPKLSSR
SRSEPPKDRKKALAISEQNGKVLKSDRKRVQKCLNQQAEGHMDDREHKERRESRRLGKGR
SQDIPEKAEEDTNMEEDKQRTEDEYQTRYRSDPNLARYPVKPQPEEQQMRMHAKVSKARH
ERRHSDASLPHTELDEAEVPENKLGKNSQGSASQDKKSVLENKRSYSVDRNGEVLVSKPK
QSANHSLHSSQHSPVPPEHVDSMNHDSFKKQSRLDPSSALLTRKATREKMETMLRNDSLS
SDQSESVRPSPPKPHRSKRGGKKRQMSVSSSDDEGASTPEYTSCEDAEIESESVSEKGDL
DCFWLDPATWHSRETSPLGSHPVTWQPSKEGDRLIGRVILNKRTSMPKESGSLLGLKVVG
GKMSETGRLGAFITKVKKGSLADVVGHLRAGDEVLEWNGKPLPGATNEDVYNIILQSKSE
AQVEIIVSRPIGDIPRIPENSHPPLESSSSSFESQKMDRPSISVISPTSPGALKDAPQVL
PGQLSVKLWYDKVGHQLIVNVLQARDLPTRPDGRPRNPYVKMYFLPDRSDKSKRRTKTVK
KSSEPKWNQSFLYSHVHRRDFRERMLEITVWDQPRVQEEESEFLGEILIELETALLDDEP
HWYKLQTHDESSLPLPQPSPFMPRRHVHGENSSKKLQSSRSNARESRSTTLTVPEHQRPT
HHRSRSVSPHRVDDQGRTRSRLPNVPLQRSLDEIHQMRRSRSPTRHHDASRSPVDRRSRD
SDSQYLSEQESELLMLPRAKRGRSAECLHTIRNTVRHSKTLPPKMPLLEYGSHWRLYSEL
QPSLDRARSASTNCLRPDTSLHSPERERQPRKLERSSNQKQNRKSTVSEIERNHQLGSQS
SADSSFSSRRGRQLPQVPVRSNSTEQEPESFSSSSKANLSMEERTRQMKIKVQRYKQIAG
SSSSQEQEREQYTKYNTHRDQYRSCDNVSAKSSDSDVSDVSAISRTSSASRLSSTSFMSE
QSERPRRRLCSFTPRMQSRRMGTSGIAITKSTSVSGEIYKLEHNDGSQSDTAVGMVGTGG
KKRRSSLSAKVVAIVSRRSRSTSQLSQTDSGNKKLKSTIQRSTETGMAAEMRSRMVRQPS
RESTDGSINSYSSEGNLIFPGVRLGVDSQFSDFLDGLGPAQLVGRQTLATPAMGDIQIGM
VDKKGQLEVEVIRARGLIQKPGSKSTPAPYVKVYLLENGACVAKKKTRIARKTLDPLYQQ
TLVFEESPNGKVLQVIVWGDYGRMDHKCFMGVAQILLEELDLSSVVIGWYKLFPPSSLVD
PTLTPLTRRASQSSLESSTGPPSIRS

>Danio rerio_RIMS1(ENSDARP_135764)
MSASVGPQSGPRPPTVPAPMPDMPDLSHLTEEERKVIMAVMVRQREEEEKEQAMLKSILK
NKIKHTLCRVEVGISSQSHAPTVWLFLCVCNVQKPSKHHGGARKNKTLHQQFESYKEQVR
KIGAETKRQQAVHKDDAPTCGICRKTKFADGCGHLCSYCQTKFCARCGGRVSLRSNNEDK
VVMWVCNLCRKQQEILTKSGEWFSSGPGGRPLSVGSPLSGPELEKERKLRSRSQVPGAND
TLLPNAGPGDHPKGADTMPASRSRSEPPREKRRPVSMHEQNGKVIGRGERRRAPARLSSQ
ASEDRAPGENRDGRRLEKDHSQEDDPTHPERRRRQEEEERERQRRDSSEEEGGSTPEYTS
CEDVEIESVSEKGDWDCHPLDPTVWHLNMKHVMSAHHPVSWQPSKEGDHLIGRITLSKRS
TMPREAGSLLGLKVVGGKMTESGRLGAFITKVKKGSLADIVGHLRAGDEVLQWNGKALPG
ATKKEVYNIILESQSAPQVEIVVSRPIGETPRLPGTSHPPLESTGSSSIDESQKMDRPSI
SVMSPTSPGMLRDLPLVLPGQLSVKLWYDKVGHQLIVNVLQARELPPRPDGRPRNPYVKM
YFLPDRSDKSKRRTKTVKKSAEPKWNQTFLYSHVHRRDFRERMLEITVWDQPRAQEEESD
FLGEILIELETALLDDQAHWYKFQTHDVSSLPLPQPSPCLPRRHVHGDSPSKKLQIKFHI
PHHRIIETDYDDGITVVSSAAERTSRDRERSSTLTVPERQAAIQHRSRSVSPHREDQCRA
RSRPAHIPMQRSLDEIHQNRHHSYSPSHYHDSHQEHRSGDSDYEYSEDSNSEVLEMHRSI
RGGSAECLHTNSDLQPSLDRVRSASTTCLRPESSFHSPERHRGKYSNYLLIGQIFSPISF
VFRNSHFPFHRHSGNLDRPSCQIANKKTIDGDSFLLPLVFSAVAVFRLQPTSILRGSRRR
RETPLRPFGQTSLGGSCPNSPRADRDLPHGGHYSPAGTTSLGRRGRQLPQLPAKSSSIEQ
AALAVEERARQLQMRVHSYRPGSSANSAHDPEAELKNRRDMYKDQRRSCDNMSARSSDSD
MSDVSAISHASSASRLSATSYMSIQSERPHGRIRKNVSPKLNASDKCSNYFQLIKSTDLI
CSLYRLLSDNNAQFNEQMHRLGGTGSKEQSNCSIKPKCIVEHRIRATDTLKPPEASNKKS
KGSQGTIQRSQETGMAVELQRNMSRQPSRESNNGSTNSYNSEGSPIFPAVRVETQFSDFL
DGLGPAQLVGRQTLATPAIGDIQIGMVNKKGQLEVEVIRARGLIQKPGSKSLPAPYVKVY
LLHNGAYVAKKKTKIARKTLDPLYQQTLQFEESPQGKVLQVIVWGDYGRMDHKSFMGVAQ
ILLEELDLSSNVIGWYKLFPPSSLVDPTLASLTRRASQSSLDKDNSPAPTGVRS

>Takifugu rubripes_RIMS1(ENSTRUP_20109)
MSASVGPQGGPRPPTVPPSMPDLPDLSHLTEEERTIIMAVMARQKDEEEKEQAMLKCLIK
DVNVCSAAVKERVQFHLSSPPSRTLHQQFESYKQEVRRIGAETRRQQTQQKDDAPTCGIC
HKTKFANGCGHLCSYCQTKFCARCGGRVSLRSNNEDKVVMWVCNLCRKQQEILTKSGEWF
SGSGVRPGSQGASLNDLTTGGEAQPDRKLQRSRSQAPPTFNANAGLPDGTQPPAGVGAKG
ANNMLGSRSQSEPPREKKRPVSLHEQNGKVCVGRGSGRRHAGKLQSQSSLDEQVVSGDRH
MGVGRRLEKVHSHGFEDAQDNLGAPETHRRRPKDEEQRERQCREEEYQNRYRSDPNLARY
PVKPQKEEQEMRIHAKVSKVRHERRHSDLAINEVGLGPGDGGGGGTKGEVNENRLSRKAG

GGDGDRKLLENNRPYSVERNVGTGRGAPIRSGHQTGGPGLNKGHTESYTTRTPRDRGDDN
MLRKDSQSSDQSESLRPPPPRSYKSKGGVNKRQMSISSSEEEGGSTPEYTSCEDVDMESV
SEKGDWDYHCLDPAVWHSTSCFPLSLQHPVSWQPSKEGDHLIGRITLSKRSAVPREAGSL
LGLKVVGGKMTETGRLGAFITKVKKGSLADVVGHLRAGDEVLQWNGKSLPGATKKEVYNI
ILESKPEPQVELVVSRPIGDIPRIPESTHPPLESTGSSSFESQKMDRPSISVMSPTSPGA
LRELPLLLPGQLSVKLWYDKVGHQLIVNVLQAIDLPPRPDGRPRNPYVKMYFLPDRSDKS
KRRTKTVKKSAEPKWNQTFIYSHVHRRDFRERMLEITVWDQPRVQEEESEFLGEILIELE
TALLDDIPHWYKLQTHDVSSIPLPQPSPYLPRRHLHKDSPSKKLQRSHRIIDTEFDDGLM
NEDCSVAPCPQGTERNSRERDRGSTLAVPEQKRAAQHRSRSVSPHREDCCRARSRPAHVP
MQRSLDEIHQNRHQSNSPSHYHDSHLEHQRSGDSDYEYSEDSHGNGSEVLEMHRSIRGGS
AECLHTNSRGTARYSNTLPPKMPLLVNGIHKDIYSSTLPACLKSKPAPRQEGGSSEPRSI
LTHRVLRFTDEVPVRWRSDLQPSLDRVRSASTTCLRPEPNFHFLDRDRYELLLKWTILQE
RVSLPHCSTCSLCCNVTGYWQLIKPESRQLQNLKSDGQCEEIKRRIAACLSRPCNAAPEW
MTYFPVSIGAPSVCPLSSALLPVQHMCKMHSTALVGSCPNSPRLDRVHLHGGPSPAGTPL
PGRRGRQLPQLPAKSSSIEQALAGEERARLHMKVHSCRPSVSRNPEIDLKTKREVGSHPF
EQRRSSDNMSARSSESDASDASALSPASSASHISCASYRSIQSERPRGRLRSVDEDMMIR
QIQSVGRSMLKSSSVSGEIYSQERTDGSQSDTALGTVCGGGKKRRSSLSARVVAIVGNRR
SRSTSQISGPEGKSKKEKGAPIQRSTETGMAVELTRNMSRQPSRESNNGSMNSYSSEGNL
IFSGVNLGASSQFSDFLDGLGPAQLVGRQTLATPAIGDIQIGMMEKKGQLEVEVIRARGL
VQKPGSKSLPAPYVKVYLLNNGAYVAKKKTKIARKTLDPLYQQALLFEESPHGKVLQVIV
WGDYGRMDHKSFMGVAQILLEELDLSSTVIGWYKLFPPSSLVDPTLASLTRRASQSSLDS
SSGPVGVRS

>Tetraodon nigroviridis_RIMS1(ENSTNIP_22973)
LHQQFESYKQEVRRIGAETRRQQTQQKDDAPTCGICHKTKFANGCGHLCSYCQTKFCARC
GGRVSLRSNNEDKVVMWVCNLCRKQQEILTKSGEWFPGSGRGSQGTSLNNLTTGGEAQPD
RKLQRPRSQAPPSINANAGLPDGTQRSTGVVAKGANNMPGSRSQSEPPREKKRPVSLHEQ
NGRCGLGRGSGRRAPGKLQSQSSLDEQIVSGERERHMGVGRRLEKIRSQDFEDGQVNLGP
PETHRRRPEDEEQRDSSEEEGGSTPEYTSCEDVDMESVSEKGDWDYHCLDPAVWHSRSHH
FLLPMSLQHPVSWQPSKEGDHLIGRITLSKRSAMPREAGSLLGLKVVGGKMTEAGRLGAF
ITKVKKGSLADVVGHLRAGDEVLQWNGKSLPGATKKEVYNIILESKPEPQVELVVSRPIG
DIPRIPESTHPPLESTGSSSFESQKMDRPSISVMSPTSPGALRDLPLLLPGQLSVKLWYD
KVGHQLIVNVLQAIDLPPRPDGRPRNPYVKMYFLPDRSDKSKRRTKTVKKSAEPKWNQTF
IYSHVHRRDFRERMLEITVWDQPRAQEEESEFLGEILIELETALLDDIPHWYKLQTHDVS
SIPLPQPSPYLPRRHIHKDSPSKKLQRSHRIIDTEFDDGLITSPQLSCTERNSRERDRGS
TLAVPEQKRPIQHRSRSVSPHREDCCMARSRPAHVPMQRSLDEIHQNRHHSNSPSHYHDP
HLEHQRSGDSDYEYPEDSEVLEMHRSIRGGSAECLHTNRPSDLQPSLDRVRSASTTCLRP
EPNFHFLDRDSYQSKQSRTNFLKQAIKTAWKEMAHLHGGPSPAGTPPSGRRGRQLPQLPA
KSSSIEQALAVEERARQLEMKVHSCRPSVSRNHEIDLKTKREMYAEQRRSSDNMSARSSD
SDRSDVSALSRASSASRLSSTSYMSIQSERPRGRLRSLIPDLMCRHLRSVGRSMLKSSSV
SGEIYSQERTDGSQSDTALGTVCGGSKKRRSSLSARVVAIVGNRRSRSTSQISGPEGKSK
KEKGAPIQRSTETGMAVELTRNMSRQPSRESNNGSINSYSSEGNLIFSGVNLGASSQFSD
FLDGLGPAQLVGRQTLATPAIGDIQISMVEKKGQLEVEVIRARGLVQKPGSKSLPATYVK
VYLLNNGAYVAKKKTKIARKTLDPLYQQALLFEESPHGKVLQVIVWGDYGRMDHKSFMGV
AQILLEELNLSRIVIGWYKLFPPSSLVDPTLASLTRRASQSSLDSSSGPVGVRS

>Gasterosteus aculeatus_RIMS1(ENSGACP_23917)
MSASVGPQGGPHPPTVPTTMPDLPDLSHLTEEERKIIMAVMARQKEEEDKEQAMLKKKVV
SGQNPPCCCKTEEESSAKRHQESGTLHQQFESYKQEVRRIGADTRRQPTQLKDDAPTCGI
CRKTKFADGCGHLCSYCQTKFCARCGGRVSLRSNNEDKVVMWVCNLCRKQQEILTKSGEW
FSESGVRPGSLGTSLNNPARGGETQRDRKLIRSRSQAPPSSTNSNAGQLDGTQPAAGVAA
AKGAETMPGSRSQSEPPKEKKRPVSLHEQNGKGGMGRGSGRRPAGKLPSQSSLDERVVPG
DRGERRLGDGRRLEKIHSQDYEDREGNLGRAETHRRRPEDEEQRERQRREEEFQNRYRSD
PNLARYPVKPQKEEQEMRMHAKVSKVRHERRHSDLAINEVGLGPGEGGGGGSGGGDVPEN
RLARRGGGGEGNHKALLENHHVFSVDRTAGVGGAAQPAGGGVRSGPQPEGPVAPDWGTNK
GRLEPGTTRTPRDKGGDSLPRKDSQSSDQSESLRPPPPRSYKSKRGVNKRQMSISSSEEE
GGSTPEYTSCEDVDMESVSEKGDWDCHSLDPTVWHHYILFPSLLSVHPVTWQPSKEGDHL
IGRITLSKRSAMPREAGSLLGLKVVGGKMTETGRLGAFITKVKKGSLADVVGHLRAGDEV
LQWNGKSLPGATKKEVYNIILESKAESQVEIVVSRPIGDIPRIPESSHPPLESRSSSFES
QKMERPSISVMSPTSPGTLRDLPLVLPGQLSVKLWYDKVGHQLIVNVLQAIDLPPRPDGR
PRNPYVKMYFLPDRSDKSKRRTKTVKKSVEPKWNQTFLYSHVHRRDFRERMLEITVWDQP

RVQEEESEFLGEILIELETALLDDIPHWYKLQAHDMSSIPLPQPSPYLPRRHVHGDSPSK
KLQRSHRIIDTEFDDGLMVVTKGAERNSRERERGSTLAVPEQQRPVQHRSRSVSPHREDS
CRARSRPAHVPMQRSLDEIHQNSHHSHSTLRYHDSRLEHQRSGDSDYEYSEDSHRHGNDS
EVLEMHRSIRGGSAECLHTNRGIGRYSNTLPPKMPLLVNGIHKDIYSSTLPACLKRKPPL
RQEGGSTMPRSILTHRVLRFTDEVTVSDLQPPLDRVRSASTTCLRPDTNFQSPDRDSSRC
SNSLPLKTPPSPRILVEHVAPEEDRYTRPAPQQHIYFLSKKGSKKFADGSRIQPTSILRG
SRGRGGPLRPFGQTALAGSGPNSPRLESQRYASTPLCADITLSVAMLYAPVSHLKESRAH
PHGSPSSPSGTPSSGRRGRQLPQLPAKSSSIEQALAVEERARQLQVKVHSYRPSASHDPE
MDLKTKREMYAEQRRSSDNMSARSSDSDMSDASALSRASSASRLSSTSYMSIQSERPGGR
LRYFGPKPLSRQMRSSAGRSMLKSSSVSGEIYTQERTDGSQSDTALGTVGGGSKKRRSSL
SARVVAIVGNRRSRSTSQISGPEGKSKKEKGAPIQRSTETGMAVELTRNMSRQPSRESNN
GSMNSCNSEGNLIFSGVNVGASSQFSDFLDGLGPAQLVGRQTLATPAIGDIQIGMMEKKG
QLEVEVIRARGLVQKPGSKSLPAPYVKVYLLNNGAYVAKKKTKIARKTLDPLYQQALLFE
ESPQGKVLQVIVWGDYGRMDHKSFMGVAQILMEELDLSSTVIGWYKLFPPSSLVDPTLAS
LTRRASQSSLDSSSGPPGVRS

>Ciona intestinalis_(ENSCINP_21734)
MMELPDLSHLTEEEQKQILSVIKRQQDEEKKEKAMINLGIEPPNDSSLPIHCFKIQRNLH
QQFETYKEQVKKASETYESKNTPGTNVDIGETCSLCHKTKMAEGSGHSCSYCSRKFCSRC
GGRVPVKSSKLMWVCNLCRKQQEMLTKSGAWCNANGSKSTLTLNLEPIPANGIQPAENKS
VNKPHRTNDIMTNSEKDIVKPKAKPRVVSSSTSSHLDTQPPRSSGSTPNMSGGKSSETIR
SQVGSDHRKEQDDRMRTSSSSPRMGNDARVHQSSRDVKHREIINHNIRDEYHGKRQTEPT
HKPGEIPSQPHSASESPRSTHRAATHHGSPNLPLKKNEDPTLLPSDPRSHSKPHEASPKR
VRSISVSQSSHFSGSPHSSPQIKRSDRSVGNFTSHSPESHGTQRRNHRMNKDRQSYLGES
MILTKYSPTNHNIDTLDTCKPFLNIIFSMFSRLSYSDVNLYDNHIECRNHHRKRMNRQDY
LDSNAMTIRRPHHHKVKRPDKYKRRNPSLSSTDEEREDFQTTPDYSSCEDEHQRHGDLNL
TSRNEHFDGRNRKKVHFTESVDKKYLQKYPGSLSDHSMSVSAENLSSGTRDSGIDTSSTN
THSDEKEVGIVQPVSWQPSPDGRFLIGRMILNKGLATGDSQNQEGAILGLKVVGGKMTES
GRLGAFITKVKHGSLADVVGHLQPGDEVVMWNDNCLQNASFDEVYEAVLASKKSKLVELT
VSRQIKEVPRIPDSLHRHPVTESSSSSFESQKREEGMNGRNGQRRTRVTPSKIQVRLWFD
NSTQQLYVTVLQAIDLLPREGGMLRNPYIKMYLLPDRSEASKRRSKTAKKTLNPSWHQSF
VYGPIRRQDLGSMALEVSLWDLDRSVESNNHFLGEIFIDLGMTRLDDTTQWFNLVMEEDT
VNHSVSHSISHYDYVPEPHQVVKGRRRVASEADFGFVFIKKQFLVIYF

>Ciona savignyi_(ENSCSAVP_17838)
TCSLCHKTKMAEGSGHSCSYCSRRFCSRCGGRVPVKSNKLMWVCNLCRKQQEIVAKSGSW
SNSNGSKSTLTLNLEPIPPNGKLTNKWLNEPRPVGSKITPKPKEQHQWTSVENMFKLQVL
CHSFSFGGGKTHNNKNYCQPKENGSKPSLNNEKENPKNKSDLRVTPTTQRDAQIPKTSGS
SLSLSSGRSSDVVRSLSECRWDGEEHAKSRPSSPNPRKSIGRCVTSSPDIQNRDVAPKQP
NDTKRPNDTKRTNDTKRPTPATSRTLDYHPTPEPTRTHRTSHSSAHLASSKLEESAPHMP
ANSKSHHKLPNDTSPKQTMRVSQSTNFNSPHYSSPHSSPHLKRNERTLDNTLSTSDPNRR
RNNKTTKDRKSYLGELICSLFKKYAIFKKYSIIKNLFDFSNNICLLSRGREPPPPPWKVR
NRAATRNPSLSSTDEEREDFHSTPDYSSCDDNNYYGFMTSPQFDGRLRKKVHFTESVEQK
FRSKHQSFSDQSVSVENLSCVTRDSGIDTGGSVTHTASTQPDRNEQPVSWQPSPDGKFLI
GRMILNKGLATGNTQNYDGPILGLKVVGGKMTESGRLGAFITKVKHGSLADVVGHLRPGD
EVVMWNEHCLQNASFDDVYEAVLASKFSKLVELMVSRKIEDTPRIPDPLQRHPVAESSSS
SFESQKRDEECNQRFHTRQQRRRAASGKIQVRLWLDPPTQQLYVTVLQATGLTYRDGGLL
RNPYIKMHLLPDRSESSKRRSKTAKKTLNPAWNQSFVYGPIRRQDLGSMALEITLWDLDR
SVETNNHFLGELLIDLGLARLDDTTQWFELSMESNYQAVQHSVSHYDYPMERHRVERRRI
SDAGFGLDCQSNLFSDRSYYEQDPSFPNHTHQSSPALVRHRVDPTLTHRYGNPPLPHPRD
LSPRRRGDSPGRRMGVSPSITSIHHSERSQGEFLPGGRRVLPPSPQNMRIIMTSQYVGSS
PRPRRSSVGAHSSESLDSSHLNEQLQLNGSTHPDDQPSMTSQSSRTSTNQRSEVATPNPG
DKISKSVSLGANLHEKNQSDQKLGNSPRSSEFGVQPELSREGSRGSASGSLPSLVSESST
VGKTLQDFVGGLGPGQVIGRQTLAASSLGDLELSFYETRGQLEVQVIRARNLVPKANSKV
LPAPYVKVYLMFGMQCISKKKTKIARRTLDPVFHQTMNFDPTQYGHCVQVIVWGDYGRMD
HKAFMGVAQVKLENLDLSRNTIGWYRLFPTTSLIDAASLGSTRPGSMTSLDSEATTMRN

>Anopheles gambiae_(XP_003435904)
MFTQTNRDIRFSSVANGDDNDDDGSSHHGLKMDDMPDLSHLTQEERAIIEGVMMRQKQEEERENEIMRRK
QDEVATLVDSIRQKSEQQKKAGVELEATCHICLKTKFADGIGHICHYCNIRCCAKCGGKVTLRNNKVIWV
CIVCRKKQELLSKTGQWMNKSTSPDGVIRRQEGDPRTLPQTMLDPHDPSDKRPKLERARSAAEKENNPMQ

RANSQLRRQYSQQDPPTRRLSQSDGSGMDMMMSPGHHQQQIGRMQQMGHGQVGGMYGGRPGAGQQGYGGG
PYGQHSQLPQPPMHHGGGGGGSGPYGGQHSMTPQVHITHAGGGGYGHDDPSYYQSEIEDLMRTHPHLVHP
RQQQHYAESLQSSSAHGSSGMGGGGGGVGSGGMHLPPEPTKSHKRPLGGPYLPQQRSFSSSDEDIRSTPE
FEAGHQRQYFDNHTRLNANLTDYRATKLDVQQRNSYNRTNHNRYQSPSQAAPHSSHPYHYQPSQDPLGRD
YHPQQPLPQVVVGDSTIAGYDSISHHHHQPHQPHQSLHHLSGTVAPSPVTIPQGLHGGSSGSSSSSQQHH
PYQQQPHQQQCNSSSSGGSSHSTTGHHPVAQNSHARYGTSPAGTEQPDPYWDEPADSRRFTERRKKTVRF
DGQDSDDWSRWESERQGSQDSATKDSGIDTSSTFTSSEDSNRGDGPKNPLSWQVVSSSDGGRPIGPAVQR
RPFDGEDVLGLKVRGGQVLPSDPRAALIELESAHIRPENYDRRDKPSVLVTSPGSPDLHSVRNYSSSRYS
NRLAPAGTVTQENSVGGRVQIKLGFEPSSLQLIVTILCANGLVPRGNGAARNPYVKICLLPDRSEKSKRR
TKTLALTNDPRWGQTFVYEGLRRADLNNRLFEVTVWDYVRYGANDFLGEVIIDLSTHPLDDEAEWYILQP
HQETLRDTVQPGNELDMILTPTDHLSPPSTTSRLSDSDTTSECDIDGLMTGRDGASVSSLGSSSSPPPEV
DLQDRRSRRDMSPQGRKRAAGMVAKDYRTVSGVGQGFHNQMSAEVCQSRQRERLHPSLTVYLCFLGTGIV
GVQAQRYDGVESAKPIGGTERQLSGRSAGLKRQLPQVPHMSRNAAIRERFIQDFEERSGGRFARHRGRQS
HHQPTYRSTGMGGWERHYSGLSDSDLTTHSLESRIRPRHSLSPDKDFMGDFGDSDMESVVSVTSSAFSTQ
SERPRGSKGIRRAAAWRACFGATVYLEGQHGCGKTHQNRRLQHRSLPTGWATTSLCALPSESYPCGGQQV
MADNSGILPVLPDVTQATIIPPKPEFELPPPACTVVGPSVVGNASTCSSFVSPLPVSIPIDSVLSEPIPI
SPAPPDKTISQMSDMAGSYGSVPVSHGGSMPEGYFNEQCITLQPERSTIRSISMNPGSDNVMSTSVNLSS
SQPGCQHPATSFEFQSGSLPTEHYFGRARAHPAGGVSMDPIMSSSMHAADGTGTVEYEQCAPSMGRRPSY
AMRSNTSEPNLNSMIMRRLSNRPIEQPHAFGYAQPRNQMISISNRYHPQPHAGMGGSDMGSKYAPPPVAT
NQIILKSSFSDQNLHNSIVYEPGGMSGGAGGQNICISNKNVYDPHTGYQNQTITCISNSRENLGESNFRY
TSNPEGTVCEINAPDVQFRSSRASNLYEEVAMPLDQARVPVKETLSAGRNIDCDEHEYGVSDLEMHPDHI
LNKPIEVLKSPSEYRYSAYPKKVTEDTYVVSSDVRGKPEKISRSHSLIAQDQIIDIEYDSDTGWKTKSVR
KNVFASSVEEKVYRKRTRSLGGGNSEEETKGRRDSQCETKKVTPVQGRKSRSGSKASLDRSNLTLNIVKN
VDGESSKNQSEKGKSKNIEKPTATGKQGEKEKCTAVAKKEKESSKVGKTSSSSKPGMVKSKSSTGSSTPK
KGTKSPAKSGSGSIKKAGSLKGKQTKTGGSTPSIASSSKVKVAEKPNFKEIDTKKSKSKLPCKKTDSKSR
VDEIKSESTVLYSSSESIKSIIDEVRMELLQKPKSTVYLSDESPTVLNRSHSFSDGELNYSKRIHDSYDK
YEFVDGRKHYLLKDERLKQRLTDRYGHERAVGYERSTDRRRGDDEYERRGCRMVPDGEEDDPRRRPEMFA
KCKSRSSSLVSNDAGEIPLRRKVYHSDSAEEEDDDDGREDEEEGDDEVFESSFSRGRNSSFNGKSSLKRR
ARYDTRRTSSLEGLDQFLANLQDRNRRGGRDGAGKNDSARHSSVSINEKPEYFEYTKSPSSPYCTSSSGR
ASASSRKPANYASILSSNKSSNGVALLQTTPKRPSMKKSSNMPPAVGDVVGRASDGTRSRSHDRHAATRS
GSPSTSAGRLPVDHQRRAHTTHSDYDVRGRSRYGGHNGGREAYRQRDRDRDRDRNSSDHERDRDLSDREQ
RESRERGELDQSLSNTEGTPEDKIDGSLSDTAVIQSLDARRKLDQRSPKSETPTRDRDRFGTGMGIKSNS
TSQLSATGRKRRMGFGKRGKNSFTVHRSEEVLPGEVRGGGGLSRGSSASSDGEGSADGDSTPRVASKRVD
RVTSGAPAHKCTMLPPHSDRLVHQILSSPSVAHSILLADQGVSPSSSSSTSQRKRASAGSIQRSVEVTPI
PAQSAAYRAGSLHSISSSEGSSWSPSLRVAGDTGPLSDFIDGLGPGQLVGRQVLGAPALGDIQLSMCYQK
GSLEVEVIRARGLQARPGSKVLPAPYVKVYLVSGKKCIEKAKTTTARKTLDPLYQQQLVFREPFAGCILQ
VTVWGDYGRMEGKKVFMGVAQIMLDNLNLSHIVIGWYKLFGTTSL

>Hydra magnipapillata_(XP_012564402)
MIMSQLSTGNDYYPASPPPIDLSHLTEAEKRKIKNVLDRQKELENETAFIQSGIVRELATYQVRYELKSN
QAPLSDANMCELCHKNKEFCVNNYKRYCKFCRSKLCDECSIQSGSGKMVTWSCLICKKKQDLLLKTGRWF
HGKEHKFPNPAAEIEQLLTVPTQQKKPLSPKIIKTGYPASSVTKAQLADKNCTKVLDQTNNESTELSNTK
KVTFGNKSKSNMLEEQQIKEMFQSLVSHHVHWSRPDDENKILGDILLSIGSNIIKKFPDYCTAFGIKLSM
GVSDEHGLLKTLVKSVVPGSIGDIIGKIKPGDELLEWNELSLVGCSKEEVEDVLANEDSLDLHLLLSRKC
HKEKILANIQSVPPSHHSPPSQPMGILKTIDGSFDKTDCQNGAVECDSSKSNVVQANQSRGTPILKANNI
SGQLQIKLKYEEDTSCLYVSIIGAQGLNHRQNMQLRNPYVKLYFLPDRSLQSKRRTKTVLRSLSPTWNQT
FMYTLKSQKFKGRYLEITLWDYERSESNEFLGEILISMLEAPLDNLCHWYPLQLHDQQNMLPQPTPTPTL
SPQCSKAQFILQDNGEREHPVENQTVSSFSPADINEKINEVGTVKTSFSYDSTGGTSTDENTPVPSPSPS
QVFEMNKELSQITSSKNSDNEKRQYLKPALVQESSVIRPSLLQRAQLFLQKQLDVLTSSSSSETQLTKNS
TSLWTSNPTNYESKLTTVTQTENQSPLSSLSVDTSSISSDMSTVNNIRYGKEVYGRFSSEKYPQIKRVDS
VDSSRKIEPRLTDYNLSLSQQGDRAGDKSAEEWRQSGNDNNYGVKFNNKPTLLRMDSMSGSSPKNFEKKA
ENYYREQHNYNEDKRFQPRSNHEISFMRSTDFDHNGYSAMRNVNRYRQNSYGSGHSSPVSSIGSAKSESS
SSIASSMMTLTSVGSSMSWIPPSLRLSGDVQLIDFVEGLGPAQVVGRQVLGSPLMGDVQLSLLDRRGVLD
VEIIRAKSLIIKPDSKTLPAPYVKVYLLCGKKCVEKRKTKTSSKRTLDPHYAQHLTFVQNYQGKVLQIMV
WGDYGRLDRKVFMGVCQILLDDLDLSQLVVGWYKLFTTTSLVHPPSNPSPNPSNPTTSSTNSDRF

>Amphimedon queenslandica_(XP_011405965)
MESKKRVVSNYRSSIIGPPVFQYTSIYAKISEAKNLASGLRDVYCYIKVDNEAIARTASVFNSSEPFWGE
EYRLHVPLEYQYVSVYLYDHDTLGVKVPIGKVRLDRDSIMGSPRGLDSWFPLKPISKDDEIQGELLAEIM
IDEYSDEMFRGVITLVEARDLAVRDINSKVDPYAVINYKGKELNTPTLKKTRFPRWNKTYELPINRPLEE
FDNRTVRITIYDSAKFHHDAFLGEVIIDLAELFLGQRYKTWYRLQQQDKETSAAMPSGGAKKDGKKEKDL

GSLRLKVQCHEERILDSQLYSPFVQLMISAVESPGPEGSPLLIMLEEVMTMDRNSLAQTLVRFYIAHNAI
LPLFDCLISREVENVSLSNVTTLFRGNSLASKCLDQFMKVVALPYLHETLKPVIDTIFNDKKLVELDPDQ
LKTRSSSTSKLIDTSVAQLTGYIQIATESIFESVERCPQVLRQSMRLLWDRVIVKFPDGDTPYSSVTGFI
FLRFFVPAILSPKLFALREEHADQRAERTLKLTAKLIQTMGNLQTAVDAKEQFMSPMATLLKEGVDKVKT
YINQLIDVQIVMNTPSATPVEVLSDGLGYLQHVVLNSGVLWKRVTSGTSGAVGSLLYRKRFFELTNIAFT
YFPTEKKTGEAKSYPVQWIRVVEKLDEAAFSRKHMFQIVIVPVGPSTAFSPSETVLYLQAADVNEQNRWV
SAIRKACLCNREMSPVHHTGVYLPRKKEWSCCRATAPAEPGCCPCHRGITIGDWRDPLDPYLDAQVLYSQ
YQQGRETLTKNYSAPAESENPENPDVPPPRPAKPGSDEARMQAAYGRLAEVLDELSYHHSQVPNV

R-Spondin Homolog-RSPO

>Homo sapiens_RSPO1(ENSP_383846)
MRLGLCVVALVLSWTHLTISSRGIKGKRQRRISAEGSQACAKGCELCSEVNGCLKCSPKL
FILLERNDIRQVGVCLPSCPPGYFDARNPDMNKCIKCKIEHCEACFSHNFCTKCKEGLYL
HKGRCYPACPEGSSAANGTMECSSPAQCEMSEWSPWGPCSKKQQLCGFRRGSEERTRRVL
HAPVGDHAACSDTKETRRCTVRRVPCPEGQKRRKGGQGRRENANRNLARKESKEAGAGSR
RRKGQQQQQQQGTVGPLTSAGPA

>Gorilla gorilla_RSPO1(ENSGGOP_7543)
MRLGLCVVALVLSWTHLTISSRGIKGKRQRRISAEGSQACAKGCELCSEVNGCLKCSPKL
FILLERNDIRQVGVCLPSCPPGYFDARNPDMNKCIKCKIEHCEACFSHNFCTKCKEGLYL
HKGRCYPACPEGSSAANGTMECSSPAQCEMSEWSPWGPCSKKKKLCGFRRGSEERTRRVL
HAPVGDHAACSDTKETRRCTVRRVPCPEGQKRRKGGQGRRENANRNLARKESKEAGAGSR
RRKGQQQQQQQGTVGPLTSAGPA

>Macaca mulatta_RSPO1(ENSMMUP_11037)
MRLGLCVVALVLSWTHLTIGSRGIKGKRQRRISVEGSQACAKGCELCSEVNGCLKCSPKL
FILLERNDIRQVGVCLPSCPPGYFDARNPDMNKCIKCKIEHCEACFSHNFCTKCKEGLYL
HKGRCYPACPEGSSAANGTMECSSPAQCEMSEWSPWGPCSKKKKLCGFRRGSEERTRRVL
NAPGGDHAACSDTKETRRCTVRRVPCPEGQKRRKGGQGRRENANRNLARKESKEAGTGSR
RRKGQQQQQQGTVGPLTSAGPA

>Mus musculus_RSPO1(ENSMUSP_30687)
MRLGLCVVALVLSWTHIAVGSRGIKGKRQRRISAEGSQACAKGCELCSEVNGCLKCSPKL
FILLERNDIRQVGVCLPSCPPGYFDARNPDMNKCIKCKIEHCEACFSHNFCTKCQEGLYL
HKGRCYPACPEGSTAANSTMECGSPAQCEMSEWSPWGPCSKKRKLCGFRKGSEERTRRVL
HAPGGDHTTCSDTKETRKCTVRRTPCPEGQKRRKGGQGRRENANRHPARKNSKEPGSNSR
RHKGQQQPQPGTTGPLTSVGPTWAQ

>Oryctolagus cuniculus_RSPO1(ENSOCUP_9364)
MRLGLCVVALTLSWVHLAAGSRGIRGKRQRRISAEGSQACAKGCELCSEVNGCLKCSPKL
FILLERSDIRQVGVCLPSCPPGYFDARNPDMNKCIKCKIEHCEACFSHNFCTKCREGLYL
HKGRCYPACPEGSTASNGTLECGSPAQCEMSEWSPWGPCSKKKKLCGFRRGSEERTRRVL
HAPAGDHTACSDTKEARRCTVRRTPCPEGQKRRKGGQGRRENTHRNLARKDSKETGAGSR
RRKGQQQQPGTARPFTTAGPT

>Bos taurus_RSPO1(ENSBTAP_5002)
MRLGLCVVALVLSWMHLAAGSRGIKGKRQRRISADGGQACAKGCELCSEVNGCLKCSPKL
FILLERNDIRQVGVCLPSCPPGYFDARNPDMNKCIKCKIEHCEACFSHNFCTKCQESLYL
HKGRCYPACPEGSAPADGTMECSSPAQCEMSEWSLWGPCSKKKKLCGFRRGLEERTRRVL
HAPGGDHAVCSDTKETRKCTVRRTPCPEGQKRRKGSQGRRENANRNPGRKESKEAGTGAR
RRKGQQQQQQGTEGPVTSLGPT

>Dasypus novemcinctus_RSPO1(ENSDNOP_20525)
MRLGLCVVVLVLSWMHLAAGSRGTKGKRQRRISVEGSQACAKGCELCSEVNGCLKCSPKL
FILLERNDIRQVGVCLPSCPPGYFDARNPDMNKCIKCKIEHCEACFSHNFCTKCKESLYL
HKGRCYPACPEGSAAANGTMECSSPAQCEMSEWSPWGPCSKKKKLCGFRRGSEERTRRVL
HVPAGDHAICSDTRETRRCTVQRTPCPEGQKRRKGGQGRREKVNRNPDRKESKEAGTGSR
RRQGLQQPQQQGTAGPVTSAGPT

>Myotis lucifugus_RSPO1((ENSMLUP_11140)

MRLGLCVVALVLSWMHLTAGSRGIKGKRQRRISAEGSQACAKGCELCSEVNGCLKCSPKL
FILLERNDIRQVGVCLPSCPPGYFDARNPDMNKCIKCKIEHCEACFSHNFCTKCKESLYL
HKGRCYPACPEGSAAANGTMECSSPAQCEMSEWSLWGPCTKRKKLCGFRRGSEERTRRVL
HAPGGDHAICSDTKEIRRCTVRRTPCPEGQKRRKGGQGRREKANRNPSRKESKEAGTGSR
RRKGQQQPQQQQQGTVGPLTSAGPT

>Ornithorhynchus anatinus_RSPO1(ENSOANP_3258)
MQLGLFAVLVILSWTDLAGGSKVAKGKRQRRISTEVSQGCAKGCELCSEFNGCLKCSPKL
FILLERNDIRQIGICLPSCPPGYYDARNPDMNKCIKCKIENCDACFSQNFCTKCKEGLYL
HKGRCYPDCPSGYSAANGTMECSSPAHCEMSEWSSWGPCSKKKKLCGFRKGTEERARRVL
NAPTGDRSGCPPAREVRRCTVQKSLCPEGPKRRKDEQGKRENGNGNHNRKEAKEARSGSR
RRKGQQQQQQQRGTVLPLTLAGPD

>Gallus gallus_RSPO1(ENSGALP_3008)
MQLGLFVVVVFLSSMDLTGGSKVVKGKRQRRISTELSQGCARGCDLCSEFNGCLRCSPKL
FILLERNDIRQIGICLPSCPLGYFGLRNTDMNKCIKCKIENCESCFSRNFCTKCKEGLYL
HKGRCYVTCPEGYSAANGTMECSSPAQCEMSEWGPWGPCSKKRKLCGFKKGNEDRTRRIL
QAPSGDVSLCPATTEVRRCTVQKSQCPEGKRKKKDEQGKQDNTNGNRNRKDTKDAKSGTK
KRKSKQRGAVAPTTSASPAQ

>Pelodiscus sinensis_RSPO1(ENSPSIP_8505)
MQLGLFLVVVFLSLMDLAGSSKVVKGKRQRRISSEVSQGCAKGCDLCSEFNGCLKCSPKL
FILLERNDIRQIGICLPSCPLGYFGVRNPDMNKCIKCKIENCEACFSRNFCTKCKEGLYL
HKGRCYSTCPEGYSAANGTMECSNPAQCEMSEWGPWGPCSKKRKLCGFKKGNEERSRKVL
LAPSGDVSVCPATTEVRKCTVQKNQCPEGKRKRKEEQEKRDNMNGNRNQKDTKEAKSGTK
KRKSQQRGTVVPMTLASPAQ

>Xenopus tropicalis_RSPO1(ENSXETP_63528)
MQFGLFAVLVLILMDIADSNKFVKGRRHRRRSTEVSMVCAKGCDLCSEFNGCMKCLPKLF
ILLERNDIRQTGICLQSCPQGYFDDRNRENNRCLKCKINNCETCFSKNFCTKCKEGFYSH
KGSCYSSCPEGFSAVNGTAECSSVQCEMSEWGAWSPCSRTGQCGRKKGTEERSRIVVKAP
LGDLALCPPTTERRKCTMPKIPCTNRSLGMLKNQNKQELSKRKKEKNSKTQIEQDGSLKR
NKSQQKVTSIPSTPSLPVQ

>Danio rerio_RSPO1(ENSDARP_135809)
MHLGLLALALVFFSSMGHADNLKASKARRQRRISTEVPPSCSNGCEHCSEYNGCLKCRPR
LFILLERNDIRQIGICLAACPVGYYGIRNRDMNKCTQCKIENCEACFSRNFCTKCKEGLY
SHRGRCFSSCPEGFTVNGTMECVVQCDLSEWSPWGPCMKKNKTCGFKKGNQTRTREPLQV
PSPATSTGAAPASGCVPEIQTQRCTVQKKIPCKGENKKNQQNRGENSKNRGRDSKERGGN
KKRKNTNRSTTVPTITTSMVT

>Takifugu rubripes_RSPO1(ENSTRUP_24334)
MQLGLVVLAMIFLSSMGHSDALKLSKARRQRRVSPEMPLSCPKGCDRCSEYNGCIKCKPK
LFIFLERNDIRQIGVCLASCPIGYFGMRNPEGNNRCTQCKIENCEACFNRNFCTKCKEGL
YSHSGRCYVSCPQDQSAVNETMECVDCELGEWSQWSSCMKKNKTCGFRKGSQSRSRVPLP
QVHSPDTSLALVPSQTCAPETERKKCTLSKKAPCGRERNGKGDQQGDTNRRERENGRGHG
RNKGGGSRRKKGQSRTTSAPSITTSSVS

>Gasterosteus aculeatus_RSPO1(ENSGACP_18089)
MQLGLVALAMVFVSSVGHGDALRPSRGRRQRRVSTEGPPSCPKGCDRCSEYNGCIKCRPK
LFIFLERNDIRQIGVCLASCPVGYFGMRNPEGNNRCTQCKIDNCEACFNRNFCTKCKEGL
YSHSGRCYVSCPPGQRTANESMECVGQRADECILGEWSQWGSCMKKNKTCGFKKGSQSRE
RVPSQVHSPDASPAPEPSQTCAPQVERRKCSVTKTPCARGKRKIH

>Oryzias latipes_RSPO1(ENSORLP_18367)
MQLGLVALAMIILSSMGHSDVVKLSKARRQKRVSTEGPPSCPKGCDRCSEYNGCVKCKPK
LFIFLERNDIRQVGVCLASCPVGYFGMRNPEGNNRCTQCKIDNCEACFNRNFCTKCKEGL
YSHSGRCYVSCPPGQRTANETMECVGQHALKCELGKWSQWSSCMKKNKTCGFKKGSQFRV
RVPLQQTYAADSSSTLEETCTSQSERRKCVVPKTPCVKDRKNKGDNTNRRENRRGLGREG
KDGSRGGGGKRRRGPSRTTTVPSITTSSVT

>Homo sapiens_RSPO2(ENSP_276659)
MQFRLFSFALIIILNCMDYSHCQGNRWRRSKRASYVSNPICKGCLSCSKDNGCSRCQQKLF
FFLRREGMRQYGECLHSCPSGYYGHRAPDMNRCARCRIENCDSCFSKDFCTKCKVGFYLH
RGRCFDECPDGFAPLEETMECVEGCEVGHWSEWGTCSRNNRTCGFKWGLETRTRQIVKKP
VKDTILCPTIAESRRCKMTMRHCPGGKRTPKAKEKRNKKKKRKLIERAQEQHSVFLATDR
ANQ

>Pongo abelii_RSPO2(ENSPPYP_21114)
MQFRLFSFALIIILNCMDYSHCQGNRWRRSKRASYVSNPICKGCLSCSKDNGCSRCQQKLF
FFLRREGMRQYGECLHSCPSGYYGHRAPDMNRCAKGCEVGHWSEWGTCSRNNRTCGFKWG
LETRTRQIVKKPAKDTIPCPTIAESRRCKMTVRHCPGGKRTPKAKEKRNKKKKRKLTERA
QEQHSVFLATDRANQ

>Mus musculus_RSPO2(ENSMUSP_67325)
MRFCLFSFALIIILNCMDYSQCQGNRWRRNKRASYVSNPICKGCLSCSKDNGCSRCQQKLF
FFLRREGMRQYGECLHSCPSGYYGHRAPDMNRCARCRIENCDSCFSKDFCTKCKVGFYLH
RGRCFDECPDGFAPLDETMECVEGCEVGHWSEWGTCSRNNRTCGFKWGLETRTRQIVKKP
AKDTIPCPTIAESRRCKMAMRHCPGGKRTPKAKEKRNKKKRRKLIERAQEQHSVFLATDR
VNQ

>Rattus norvegicus_RSPO2(ENSRNOP_71706)
MRFCLFSFALIIILNCMDYSQCQGNRWRRSKRASYVSNPICKGCLSCSKDNGCSRCQQKLF
FFLRREGMRQYGECLHSCPSGYYGHRAPDMNRCARCRIENCDSCFSKDFCTKCKAGFYLH
RGRCFDECPEGFAPLDETMECVEGCEVGHWSEWGTCSRNNRTCGFKWGLETRTRQIVKKP
AKDTIPCPTIAESRRCKMAMRHCPGGKRTPKAKEKRNKKKRRKLIERAQEQHSVFLATDR
VNQ

>Canis familiaris_RSPO2(ENSCAFP_995)
MQFRLFSFALIIILNCMDYSHCQGNRWRRSKRASYVSNPICKGCLSCSKDNGCSRCQQKLF
FFLRREGMRQYGECLHSCPSGYYGHRAPDMNRCARCRIENCDSCFSKDFCTKCKVGFYLH
RGRCFDECPDGFAPLDETMECVEGCEVGHWSEWGTCSRNNRTCGFKWGLETRTRQIVKKP
AKDTIPCPTIAESRRCKMAMRHCPGGKRAPKAKEKKNKKKKRKLIERAQEQHSVFLATDR
ANQ

>Bos taurus_RSPO2(ENSBTAP_18083)
MDYSHCQGNRWRRSKRASYVSNPICKGCLSCSKDNGCSRCQQKLFFFLRREGMRQYGECL
HSCPSGYYGHRAPDMNRCARCRIENCDSCFSKDFCTKCKVGFYLHRGRCFEECPDGFAAL
DETMECVEGCEVGHWSEWGTCSRNNRTCGFKWGLETRTRQIVKKPAKDTIPCPTIAESRR
CKMAMRHCPGGRKPDNNIGKK

>Pteropus vampyrus_RSPO2(ENSPVAP_402)
MQFRLFSFALIIILNCMDYSHCQGNRWRRSKRASYVSNPICKGCLSCSKDNGCSRCQQKLF
FFLRREGMRQYGECLHSCPSGFYGHRAPDMNRCARCRIENCDSCFSKDFCTKCKVGFYLH
RGRCFDECPDGFAPLDETMECVEGCEVGHWSEWGTCSRNNRTCGFKWGLETRTRQIVKKP
AKDTIPCPTIAESRRCKMAMRHCPGGRRAPKAKEKKNKKKRRKLTERAQEQHSVFLATDR
ADQ

>Ornithorhynchus anatinus_RSPO2(ENSOANP_23721)
ASYGANPICKGCLSCSKDNGCSRCQQKLFFFLRREGMRQYGECLHSCPLGYYGLRSPDMN
RCARCRIENCDSCFSRDFCTKCKAGSFLHRGRCFDECPDGFAPLEETMECVEGCEVGPWS
EWGVCSRNNRTCGFKWGLETRTRQIVKKPAKDTIPCPTIAESRRCKMALRHCPGGK

>Anolis carolinensis_RSPO2(ENSACAP_9991)
MQFRLFSFALIVLNCMDYSHCQGARWRRSKRANYGPNPICKGCLSCSKDNGCIRCQHKLF
FFLRREGMRQYGECLNSCPSGYYGLRAPDMNRCSRCRIENCDSCFSRDFCTKCKPGFYLY
RGRCFGECPDGFMPLEETMECVEGCEVGPWSEWGICSRNNKTCGFKWGLETRTRQIVKKP
AKDTLPCPTIAESRRCKMALRHCPGGKRTPKTKDKKKKKKNLMERAQKQHSLFLSTDRAG
Q

>Xenopus tropicalis_RSPO2(ENSXETP_63896)
MQFQLFSFVLIIILNCVDYSHCQANRWRRSKRASYGTNPICKGCLSCSKDNGCLRCQPKLF

FYLRREGMRQYGECLQSCPPGYYGVRGPDMNRCSRCRIENCDSCFSRDFCIKCKSGFYSH
KGQCFEECPEGFAPLDDTMVCVDGCEVGPWSEWGTCSRNNRTCGFKWGLETRTRQIVKKP
AKDTIPCPTIAESRRCKMAMRHCPGGTRTTKKKDKKNKKKKKKLLERAQEQHSVVLATDR
SSQ

>Danio rerio_RSPO2(ENSDARP_100941)
MQFRLFSFALIVLNCVDYSHCQTQQSHRWRRNKRASYGAYPICKGCSVCSKDNGCMNCQP
KLFLFLRRERMRQYGECLHACPSGYYGMRGTEINMCSRCRIDDCEACFSKDFCTKCKLGF
YLHKGRCFEKCPEGFAPLEDTMECGEGCEVGQWSEWGTCARRNKTCGFKWGLETRTRHIV
KKPPKDTIPCPTIAESRRCKMAMRHCRKGGSGKGHRTKDQKKKSNKQRLRLRAQNQHSDH
LASIRAGQ

>Takifugu rubripes_RSPO2(ENSTRUP_13116)
MQFRLFSFALIVLNCMEYSNCQAPSHRWRRNKRASYGAYPICKGCSMCSRDNGCVNCQPK
LFLFLRRERMRQYGECLHDCPAGYYGMRSPELNMCSRCRIENCESCFSKDFCTKCKSGFY
LHKGRCFDRCPEGFAPLDETMECGEGCEVGQWSEWGACTKRNKTCGYKWGLETRTRHIVK
KPPKDTIPCPTIAESRRCKMPTRHCSRGKLLEKTKNKHLLKQRRKYI

>Gasterosteus aculeatus_RSPO2(ENSGACP_7152)
GCSVCSRDNGCVNCQPKLFLFLRRERMRQYGECLLDCPAGYYGMRTLEINMCSRCRIENC
ESCFSKDFCTKCKSGFYLHKGRCFDKCPEGFAPLEDTMECGEGCEVGQWSEWGACNRRNK
TCGYKWGLETRTRHIVKKPPKDTIPCPTIAESRMCKMAIRHCRRGKDV

>Homo sapiens_RSPO4(ENSP_217260)
MRAPLCLLLLVAHAVDMLALNRRKKQVGTGLGGNCTGCIICSEENGCSTCQQRLFLFIRR
EGIRQYGKCLHDCPPGYFGIRGQEVNRCKKCGATCESCFSQDFCIRCKRQFYLYKGKCLP
TCPPGTLAHQNTRECQGECELGPWGGWSPCTHNGKTCGSAWGLESRVREAGRAGHEEAAT
CQVLSESRKCPIQRPCPGERSPGQKKGRKDRRPRKDRKLDRRLDVRPRQPGLQP

>Pan troglodytes_RSPO4(ENSPTRP_22516)
MRAPLYLLLLVAHADMLALARWGEQVGTGLGGNCTGCIICSEENGCSTCQQRLFLFIHRE
GIRQYGKCLHDCPPGYFGIRGQEVNRCKKCGATCESCFSQDFCIRCKRQFYLYKGKCLPT
CPPGTLAHQNTRECQGECELGPWGGWSPCTHNGKTCGSAWGLETRVREAGWAGHEETATC
QVLSESRKCPIQRPCPG

>Mus musculus_RSPO4(ENSMUSP_41578)
MRAPLCLLLLLAHAVDMLALYRRKKQAGTGLGGNCTGCVICSEENGCSTCQQRLFLFIRR
EGIRQYGKCVHDCPLGFFGIRGQEANRCKKCGATCESCFSQDFCIRCKRRFHLYKGKCLP
SCPPGTLTHQSTRECQEECEPSPWGSWSPCIHNGKTCGSGWGLETRVREAGPAKQEETAS
CRVLSESRKCPIKRLCPGERNPRQKNRKDRRQRKDRKLERRPHQRGSQ

>Rattus norvegicus_RSPO4(ENSRNOP_12951)
MRAPLCLLLLLAHAVDMLALYRRKKQAGTGLGGNCTGCVICSEENGCSTCQQRLFLFIRR
EGIRQYGKCVHDCPLGFFGIRGQEANRCKKCGATCESCFSQDFCIRCKRRFHLYKGKCLP
TCPPGTLTHQSTRECQEECEPSPWGSWSPCIHNGKTCGSGWGLETRVREAGPAKQEEMAS
CRVISESRKCPIKRPCPGERNPRQKNRKDRRQRKDRKLERRLHQHGSQ

>Canis familiaris_RSPO4(ENSCAFP_10193)
VGTGLGGNCTGCVICSEENGCSTCQQRLFLFIRREGIRQYGKCVHDCPPGYFGVRGQEVN
RCKKCGATCESCFSQDFCIQCKRRFYLYKGKCLPTCPLGTAAQPSTRECQEECELGPWGS
WSPCMHNGKTCGSAWGLETRVREAGRAGREEAPTCQVLSESRKCPIQRPCPAGAEKNPSQ
KKGRRDRRPRKDRKLDRTLDVRPRPPAA

>Bos taurus_RSPO4(ENSBTAP_29170)
VGTGPGGNCTGCVICSEENGCSTCQQRLFLFISREGIRQYGKCVHDCPPGYFGVRGQEVN
RCKKCGATCESCFSQDFCIQCKRRFYLYKGKCLPTCPPGTAAQQSTRECQEECELNPWGS
WSPCTHNGKTCGSAWGLETRVREAGRAGREEAATCQVLSESRKCPIRRPCPGERNPNRKK
GQRERRPRKDRKLDGRGDGRPRPAP

>Pteropus vampyrus_RSPO4(ENSPVAP_2129)
MRVPLCLLLLVAHAADMLPLNRRKKQVGIGLGGNCTGCVICSEENGCSTCQQRLFLFIRR

EGIRQYGKCVHDCPSGYFGVRGQEINRCKKCGATCQSCFSQDFCIQCKRRFYLYKGKCLP
TCPPGTTAQQSTQECQEECELGPWGSWSPCTHNGKTCGSAWGLETRVREAGRAGQEEGAT
CQVLSESRKCPIQRPCPGERNPSQKKGRRDRRPRKDRKLDRKLDVRPPRPGRQW

>Monodelphis domestica_RSPO4(ENSMODP_24370)
MPWILLILLLFGNAVEMFALNHSKKQVGVGLKDNCTGCLICSEENGCSTCQPRLFLHIRR
DGIRQYGKCVHDCPHGYFGVRGHEVNRCKKCGSTCESCFSQDFCIRCKKRFFLHKGKCAP
TCPPGTIAQQSTWECQEECEFGPWSLWSPCLQDGKTCGSAWGLETRVREVSRGSREEGTA
ACQALSESRNCAIRRHCPGEKNLTRRRGRKERRHRKERKMEHS

>Taeniopygia guttata_RSPO4(ENSTGUP_7065)
GCVLCSEDNGCITCHHRLFLLIWRDGIRQYGMCVHTCPPGYFGVRGLEVNRCTKCRSPSC
ESCFNRDFCMMCKDNFTCTMASAFGCCPAGTAAQTGTRECQETCEPGPWSEWSACTHESR
TCGCRWGVETRVREVPEAAREEGTACPALLETRRCRMKKHCPGGEHRGQG

>Anolis carolinensis_RSPO4(ENSACAP_10663)
NCTGCIMCSEDNGCISCQQKLFLIIWRDGMRQYGACVHACPPGYFGVRGQEVNRCIKCRS
PNCESCFSKDFCIKCKQQFYLHKGKCLSTCPLDTIAQPSTQECLSKPEKCEMGPWGNWSP
CTNQGRTCGWRWGSAARSREAIKSSKEEAEACPVLTESRKCRMKKRCPGAKSPNKQNIST
RYYRKSSLAQESRLASRSSD

>Danio rerio_RSPO4(ENSDARP_123862)
XLLVQMHWQLLALLSLFCQMLLLTLAVRKRRESWRQDCRSCVLCSRENGCLRCSERLFLF
LRRDGILHHGSCLHSCPAGHFGLRGKELNRCMKCKAPECERCFNKDFCTKCKIGFLLFKG
KCFKSCPEGTFAQSTDCVEGCVFSVFGFWSEWSSCQHNGLSCGVTWGLQSRTRELSRNTP
EEKETLCPPQTESRKCQMKRRCRKDKRKNEKRQERRNAQKKLKLYGNRTITNPPQGNLNF
RSFVPQ

>Homo sapiens_RSPO3(ENSP_349131)
MHLRLISWLFIILNFMEYIGSQNASRGRRQRRMHPNVSQGCQGGCATCSDYNGCLSCKPR
LFFALERIGMKQIGVCLSSCPSGYYGTRYPDINKCTKCKADCDTCFNKNFCTKCKSGFYL
HLGKCLDNCPEGLEANNHTMECVSIVHCEVSEWNPWSPCTKKGKTCGFKRGTETRVREII
QHPSAKGNLCPPTNETRKCTVQRKKCQKGERGKKGRERKRKKPNKGESKEAIPDSKSLES
SKEIPEQRENKQQQKKRKVQDKQKSVSVSTVH

>Pan troglodytes_RSPO3(ENSPTRP_31716)
MHLRLISWLFIILNFMEYIGSQNASRGRRQRRMHPNVSQGCQGGCATCSDYNGCLSCKPR
LFFALERIGMKQIGVCLSSCPSGYYGTRYPDINKCTKCKADCDTCFNKNFCTKCKSGFYL
HLGKCLDNCPEGLEANNHTMECVSIVHCEVSEWNPWSPCTKKGKTCGFKRGTETRVREII
QHPSVKGNLCPPTNETRKCTVQRKKCQKGERGKKGRERKRKKPNKGESKEAIPDSKSLES
SKEIPEQRENKQQQKKRKVQDKQKSGIEVTLAEGLTSVSQRTQPTPCRRRYL

>Callithrix jacchus_RSPO3(ENSCJAP_6657)
MHLRLISWFFIILNFMEYIGSQNASRGRRQRRTHPNVSQGCQGGCATCSDYNGCLSCKPR
LFFVLERIGMKQIGVCLSSCPSGYYGTRYPDINKCTKCKADCDTCFNKNFCTKCKSGFYL
HLGKCLDNCPEGLEAKNHTMECVNIVHCEVSEWNPWSPCTKKGKTCGFKRGTEIRVREII
QHPSAKGNLCPPPNETRKCTVQRKKCQKGERGKKGRERKRKKVNKGESKEAIPDSKDLES
SKETPEQRENKQQQKKRKVQDKQQKSVSVSTVH

>Mus musculus_RSPO3(ENSMUSP_90287)
MHLRLISCFFIILNFMEYIGSQNASRGRRQRRMHPNVSQGCQGGCATCSDYNGCLSCKPR
LFFVLERIGMKQIGVCLSSCPSGYYGTRYPDINKCTKCKVDCDTCFNKNFCTKCKSGFYL
HLGKCLDSCPEGLEANNHTMECVSIVHCEASEWSPWSPCMKKGKTCGFKRGTETRVRDIL
QHPSAKGNLCPPTSETRTCIVQRKKCSKGERGKKGRERKRKKLNKEERKETSSSSDSKGL
ESSIETPDQQENKERQQQQKRRARDKQQKSVSVSTVH

>Rattus norvegicus_RSPO3(ENSRNOP_15395)
MHLRLISWFFIILNFMEYIGSQNATRGRRQRRMHPNVSQGCQGGCATCSDYNGCLSCKPR
LFFVLERIGMKQIGVCLSSCPSGYYGTRYPDINKCTKCKADCDTCFNKNFCTKCKSGFYL
HLGKCLDSCPEGLEASNHTMECVSIVHCEASDWSSWSPCMKKGKTCGFKRGTETRVRDIL
QHPSAKGNLCPPTSETRTCIVQRKKCSKGERGKKGRERKRKKLNKEDKKETSSSDSKGLE

SSRETSEQHESKERQQQQKRRAREKQQKSVSVSTVH

>Canis familiaris_RSPO3(ENSCAFP_1500)
MLKNSPPVTSCQEWPVLESRQQLPWPTLHPNVSQGCQGGCATCSDYNGCLSCKPRLFFVL
ERIGMKQIGVCLSSCPSGYYGTRYPDINKCTKCKADCDTCFNKNFCTKCKSGFYLHLGKC
LDNCPEGLEANNHTMECVSIVHCEASEWSPWSPCMKKGKTCGFKRGTETRVREITQHPSA
KGNLCPPTSETRKCTVQRKKCQKGERGKKGRERKRKKPNKEESKDAVPDNKGLEPSRETP
EPRENKDKQQQKKRKVQDKQQKSSVDLFWGLKSS

>Loxodonta africana_RSPO3(ENSLAFP_2613)
MHLRLVPWFFIILNFMEYIGSQNASRGRRQRRMHPNVSQGCQGGCATCSDYNGCLSCKPR
LFFVLERIGMKQIGVCLSSCPSGYYGTRYPDINKCTKCKADCDTCFNKNFCTKCKSGFYL
HLGKCLDNCPEGLEANNHTMECVSIVHCEASEWSPWSPCMKKGKTCGFKRGTETRAREIV
QHPSAKGNLCPPTSETRKCTVQRKKCQKGERG

>Myotis lucifugus_RSPO3(ENSMLUP_3791)
MHLRLISWFFIILNFMEYIGSQNASRGRRQRRMHPNVSQGCQGGCATCSDYNGCLSCKPR
LFFVLERIGMKQIGVCLSSCPSGYYGTRYPDINKCTKCKADCDTCFNKNFCTKCKSGFYL
HLGKCLGNCPEGLEANNHTMECVSIVHCEASEWSAWSPCTKKGKTCGFKRGTETRVREIT
QHPSAKGNLCPPTSETRKCTVQRKKCQKGERGTK

>Monodelphis domestica_RSPO3(ENSMODP_22041)
MHLRLISWFFIILNFMEYIGSQNASRGRRQRRMHPNVSQGCQGGCATCSDYNGCLSCKPR
LFFVLERNGMKQIGVCLSSCPSGYYGTRSPDINKCTKCKADCDTCFNKNFCTKCKNGFYL
HLGKCLDTCPEGLEANNHTMECASIVHCEASEWSPWSPCTKKGKTCGFKRGTETRFREII
QLPSAKGNLCPPTSETRKCMVQRKKCPKGERGKKGRERKRKKLNKEENKETRPENKGQDP
SSESRDQRENKDKQQQKKRKVQDKQQKSSMDLFGGLTSP

>Gallus gallus_RSPO3(ENSGALP_48902)
MQLRLISWFFIALNFMEYIGSQHASRVRRQGRMHPNVSQGCQGGCATCSDYNGCLSCKPR
LFFVLERIGMKQIGVCLSSCPSGYYGTRYPDINKCAKCKADCDTCFTRNFCTKCKSGFYL
YSGKCLEKCPDGLEANNHTMECTSIVHCEASEWSPWSPCTKKGKTCGFKRGNEVRVREIV
QHPSARGNPCPATSESRKCMVQRKRCQKEGKGKKNREEKRKKSNKDESKESRQESKGRAA
RRQNREQRENNNKTQPKKRKAPSKEQDLAPVDTAH

>Xenopus tropicalis_RSPO3(ENSXETP_61149)
MQLRLLSLCFIILNFLEYIDSQQIPRGRRHRRMHPNVSQGCQGGCATCSDYNGCLTCKPQ
LFFALVRNGMKQIGVCRPSCPNGYFGTRSPEINKCTKCKADCETCFNKNFCTKCKSGFYK
HNGKCLDTCPEGFENNHNMECTSVVHCVVGEWSAWGPCTKRGKTCDIKRGNETRVREILQ
YPSPRGTPCPPTSETKKCVVKRKKCQDSQDRQRPRGNRDEIKKNKQRRKNGDAPRKQRQR
KQERNQREGKRGEGKV

>Danio rerio_RSPO3(ENSDARP_58577)
MQLQLISIVLILHFMEYTNCQHHGSRHRGNKQVSGVSSQGCQGGCQTCSVYNGCLTCKPK
LFIHLERDGMRQIGVCLASCPNGFYGTRSPDRNDCIKCGSECDSCFNRNFCLRCRAGSYL
HKGKCMESCPDGLVPSDTKKECVAACPALCDLCQNSDTCTRCVSGHFLHAGQCHHVCPDE
FEPNDSMECIPTVHCEVSEWSEWGTCSRSGKTCGFKWGEETRTRKVLQNPSPMGSPCPPT
SEKRECFVKKKRCKPPKGQRRGEKKKRFNLQEKVTAEARRERKREREKETIDREESESRN
KTEQRRRRDQSRDAGTV

>Takifugu rubripes_RSPO3(ENSTRUP_9205)
MQLHLISFVLIIILHCMDYTGCQQHSSSRHWQHKQISGVSSGCQQGGCLTCSDYNGCLSCK
PRFFMHLERIGMKQIGVCVTSCPPGFYGTRFPERSPCTKCRSECDSCFAKNFCTRCRAGF
YLHLGKCQETCPEGLVRNEAQRECAPISLNLVHCEVGEWSEWSPCSRSGRTCRFKRGQET
RTRQVLRYPSPFGNPCPEISEIKECLVKRRKCPDPGGSERGRGRERERERERERERERER
KKERKKERKKERKKERKKERKKERISVVLVI

>Tetraodon nigroviridis_RSPO3(ENSTNIP_22293)
MQLQLISFALIIILHCMDYTGCQPHSSSRHWQHKQISGVSSGCQQGGCLTCSDYNGCLSCK
PRFFMHLERIGMKQIGVCMTSCPPGFYGTRFPERSPCTKCRSECDSCFARNFCTRCRAGF
FLHLGKCQETCPEGLVRNEAQRECAPKCPADCESCVNSDTCTRCRQGLYLLSGRCHHVCP

EDYEPSDKLMECTPQVHCEVGEWSEWSPCSQSGRTCGFKRGQETRTRQVLRYPSPFGNPC
PDISEIKECLLKKRKCPGGRRKGERKERLNRNNRKDKENQTGEREDSDNRNKTEHRHRRG
HDPDLAWP

>Gasterosteus aculeatus_RSPO3(ENSGACP_8044)
MQLQLISFVLIILHGMDNTGCQQHSSPRNRQHKQISGVSSGCQQGGCLTCSDYNGCLSCK
PRFFMHLERIGMKQIGVCMTSCPLGFYGTRSPERNTCTKCRSECDSCFNKNFCTRCRAGF
YLHLGKCQENCPEGLVRSDTQRECVPGCPAECEACVNSETCARCRPGLYQLGGRCHHVCP
EDYEPSDKLMECTPQVHCDVGEWSEWSPCSRSGRTCGFKRGQETRTRQVLQHPSPFGNPC
AEVSEFKECLVKRRKCPERGQLGGQGTKNMNRGGRKKNERRERRNRNNRKDTGEREDLDN
RNKTEHRHRRGHDTDPASP

>Oryzias latipes_RSPO3(ENSORLP_7233)
MQLQVISFVLIILHCMDFTGCQPHSSSRHPQHKSISGVSSGCQQGGCLTCSDYNGCLSCK
PRFFMHLKRIGMKQIGVCMTSCPPGFYGTRSPERNTCTKCRSECDSCFDKNFCTRCRAGF
YLHLGKCQESCPDSMVHNDTQRECVLSCPAECELCVNSETCMRCRQGFYQLNGKCYHICP
EDYEPNDRLMECTPQVHCQVGEWSEWSPCIRSSKTCNRRGQEARMRQILQTPSQFGRPCP
LTTEVKDCLVKRKRCSGRRNPERKERRNRKNRKDKESDEGRREKKRGRERDRGTGERENS
ENRNKTEHRHRRGHNTETASP

>Strongylocentrotus purpuratus_(XP_796266)
MELEGFLFAITFFCGLLATMEGKHRGKGCPLGCSTCSVYNGCITCKPRYLFLLHRSGMKQVGICTHACPV
GFYPNSAGDYHRCSRCRIENCDTCYSRTFCSRCQPPYVGLYGKCVLQCPDSMYVNEMTSQCEVKVDCAVS
PWSPWSPCIKKGQTCGFKWGEHRRYREVVARPTPEAEPCPELFQGEKCRMHPRYCPGQLPRKPKDKGKRK
KEKGTTDNQEGTTRKNDGNKQKDRTNRRSKKKDRQKPSPRDERRQRQRKEEHLIDFPLSTRRTI

>Drosophila melanogaster_(FBpp0271811)
MLRHIVLLILLSSEVRGVCNRIPQGASGPRSPVDDNFKILIDGNPETYVPEHQYNVSLSC
PINMKFVSFTLVVEAEDPSAAFGGQDMTGHFELLGVGDTRFSTSCENMVENTNTNAKIYI
AVSWIAPRNPDSGCVLIKAGVVQHRDVWFLDDGFLTKRICPEEIDELNSLTPPLETCCAC
DEAKYEIVLERKWARNTHWKDFPSEDWRTRLGEVIGASHSHSYRYWAYGGRASKGMKELA
EHGATRTLENEIRENTQNGEVRTIIKAPGIPYRPKTFGTTLANARLDPVHHQISLAAKID
PSPDWILGVAGLELCLSNCTWLERKVLNLYPWDIGTDSGPSYMSSDEPQVPPDVVRRITS
SYPSDHRSPFYDDTGAPMKPLATLYLTRKKLYIRECEEVPQEGPLECAVHPWNEWTNCST
RCGQGYSHRQRSFKNPNLAANFNCDRRLEEFRQCQGTQCGAAEEELEGEEPGLGMENPPS
SGSMAECQLTNWGEWSPCTKTCGRGSSTRTRDYYNPQARQRCLSVMRIPLEETRECIGSD
CGGTIPDNGELDGFPEPDVFGETDHGGYMGKTTSWNSKQEHSSLGSGNQAWNRKPYNSWK
DKSLSNPRDNAQESPNLPFDQIENPQRPAYNPSDNMGGYDKGFPPTNSYSNDKLVGNRPG
YNDFNSDYNNKDAYNDYTTPSFTSRTPFGSRYPNQQETVEGNTNDINGNYNVVQDYCFEK
PYASTLPCFAKPVVESNYWFYDHEDHECKIFTTDNCDQNRNRFRTLTACEGTCLQPQINM
ERSENDAMDLYGRRSYDQKQTRGRPYDQTSGIAYGQTEGRSYDQKVGKSYDRTFGRSYKQ
TGGGSYDQPEDRSYDLSTGRSYVQPEDRSYDLSRGRSYDQPVGRSYDLAGGRSYGETSEA
GDIGEPMSQTRSRYDTSRRGRYGG

>Apis mellifera_(XP_016772377)
MPSSFSFDYPTFFDEFGPRPRQTTYHRQRNRGVPLQNLFTDPLFKEQWYLNGGAKDGYDMNLGPAWQKGY
TGKSVVVSILDDGIQTNHPDLALNYDHQASTDINDNDNDPMPRDNGDNKHGTRCAGEVAAVAFNQYCGVG
VAYNASIGGVRMLDGPVNDAVEARALGLNPDHIDIYSASWGPEDDGKTVDGPGPLARRAFIYGVTSGRKG
KGSIFVWASGNGGRHTDSCNCDGYTNSIFTLSISSATQGGYKPWYLEECSSTLASTYSSGTPGNDKSVAT
VDMDAKLRPDHICTVEHTGTSASAPLAAGIAALALEANPSLTWRDMQYLVVLTSRSTPLEKESGWILNGV
KRKVSHKFGYGLMDAGAMVNLAEQWTNVPPQHICKSDEINEERRIDPTYGYTLSVYMDVTGCAGSVNEVR
FLEHVQCKVSLRFFPRGNLRLLLTSPMGTTSTLLFERPRDVLSSSFDDWPFLSVHFWGEKADGRWTLQVN
NAGNRHVNSPGILKKWQLIFYGTATNPIRIRTRQFNPGYQGYPYVGAASNVQASVATLDGSSAPILTNRL
PGDTSSSSSFDSPPSSSQVGDESLDTARKILHDCDPQCDPQGCYGKGPTQCIACKNYRLDNTCVSQCPPR
SFPNQGGVCWPCHESCEICAGAGQDSCLSCAPAHLRVTDLAVCLQQCPEGYYENTENSTCVPCEANCASC
QDRPDKCTSCEHHLVMYENKCYAACPLYTYETQDYNCAPCHSTCETCNGTAENQCISCRPGLFSLNGACR
ASCPIGYGADKKRRECVPCPPGCATCDMSTCRSCDDGWSLNEEGLCAPEHRDRCKTTEFYENGHCKQCHA
SCKTCAGPGENNCISCQKPLLLQAKRCVYQCDDGYYSMAQPSGPVCVPCLHTCKTCVSRLNCTACQDGLQ
LQSGECRSSCAQGYYSDRGQCAKCYLACKTCSGPRRDQCVTCPGGWQLAAGECHPECPEGFFKSNFGCQK
CHHYCRTCKGRRNYRAGVTLLTPLLDGVNIERAIHTPGRDPCPDFIPETHRYDLHLSPFKFQSLNIHSIR
ISSPEKIQTMYINLSFI

>Trichoplax adhaerens_(XP_002110515)
MAEQLVKILLLFLTLIHIVRQESTEHYTNTWVIHVPGGLTNAKLVATTHASTILHQVGSLEDHYLIRHAG
TPHRSKRSNSYKSWNLRLDTRVLYVEQQISKLRIKRSFPNFNDPLWPKQWYLENTGQASGPPHLDHNIIP
AWKSGATGIGIVVSILDDGLYYEHPDLRRNYDPEASFDINGNDHDPTPRKTGNDENRHGTRCAGEVAAIA
NNSICTVGAAYNAKIGGIRMLDGDVTDAVEATSLSWKPQHIDIYSSSWGPEDDGRNIDGPGPLAKKAFID
GVNKGRHGLGSIFVWASGNGGSVHDNCNCDGYVISIYTISISAATDTGNVPWYTESCSSILATTFSSGNN
GQNKIITDDLHDRCTESHTGTSAAAPLAAGMFALALEANPRLTWRDLQHIVVITSKPDKLHTDDWTINGV
GRKISNWFGYGLLDAAALVSTARNWKTVPIQRSKSFKFKTKKNYIPPNGRLIVSINVEKSEAIVNYLEHV
QAVISLSHNQRGLITIQLISPAGTKSVLFDRRQQDTSRAGFKNWPLMTTHFWGEKSAGNWTLIIINKDSN
SGKLKGWKLVLFGTTSKPHDLYYSSCNPECERSSGCNGPGAADCRICRHFQAADGRCVKNCSNGQYGNLE
TKACINCPYGCDVCNNDSCITCKPNFANSNGSCVSSCPESTYQHSFNGNTKCLKCSSNCAACANETYCIS
CPGTAYEYRGSCIQQCPQGTYSNYVYGIRTCTSCKHNCLDCYGNEAGSCRVCQKGYQLVVDRSKCTKSCP
VGYYNAISGEQNICLACHHRCTSCLSAASSGCKSCSHGFHLYNGSCTDKCPDGTYTDKFTGECFPCFNTC
TTCNAGKSKSCLSCKSNLYLQNGECVSNCFKGYYQDDNLMRCRRCHHSCASCNGPSSNSCTSCKLSQSFL
HNNSCLMECPVGHYSDITMKACNPCSSHCYHCVNSSVCLSCNKNFILHHQKCAKSCPSGYILKERTCNPC
HPTCSNCSDDDIASCSGCKSHTHFLHQGICTKSCLFGYFGDTITQQCQPCHSSCASCFGEGNSSCLLCPL
GRFLQHGSCVTTCNNGFPGKNGTCSSCEGVGCRECLPNDPNQCLTCLDNYFNHKFNCVRECPVGTYTNAT
AGQCLICHDSCNTCSGKHIENCTDCKPGWFMHAGKCVSYCPSGSYANGNACSKCSHGCKLCISQNTCLEC
ESSLVKFNGSCLKDCPSNSYATDENTECQLCHKGCETCKIVNRESQCLSCINQYLILKAGKCLPNGNCSG
NMYFNGSNCSKCHSDCEACFDDDRISCLRCREDKYLNPVSAICEQYCPLGYFADNATRRCQQCHYSCLSC
SNASVCTQCRPGFYNSSGNCITKCASGEYFNNKTASCQLCGNNCKECNINSKTCITCKSGNYKYDGICLS
ACPTQTYASNGNCKACPSGCLECKADMTCTKCKNNYKLSQSACVLSLPNGCKNSSCLVCKVANCSRCSPS
DSTICNICIKPFILHQGSCIKQCPDGTYFDHVKKNCFDCNDSCSSCVGPKETDCVKCSSAKPYLLHYKCL
SKCPANYYIDNANAKCRQCHFSCKSCSGGSMNDCIICSDGFHYLKSEKQCVNKCPDLSYYRKNETDCGSC
YSGCASCYGNEKDKCISCISPMVNFNFECMEKCPLGSYNDSGICRKCHPSCGSCTGPHLNQCTSCSTGLF
LKNGECHGTCPSGYYEDMKSLKCLPCNPSCKTCVSDSAQCLTCNHGMVLQNSKCLRNCSSGYYHTDGGCR
PCHTSCLSCKGFTASDCLSCSKGWELLPHGICEPVCPSGQYYDIGLKSCGNCHSSCATCSSKQSASCLSC
LKGRLLDKQSSRCYPCCKIDSDSQCCNCSASLKVCVLLSDTGNNIVTTPSLVTVTHHDFGNTFKEEKPVQ
LILISGAISLLLFGVVVIIRCRRNIFGHRGYKKLTPSGYDHTTISSTRTAYPDYDNDEINQV

Solute Carrier Family-SLC

>Homo sapiens_SLC30A1(ENSP_355968)
MGCWGRNRGRLLCMLALTFMFMVLEVVVSRVTSSLAMLSDSFHMLSDVLALVVALVAERF
ARRTHATQKNTFGWIRAEVMGALVNAIFLTGLCFAILLEAIERFIEPHEMQQPLVVLGVG
VAGLLVNVLGLCLFHHHSGFSQDSGHGHSHGGHGHGHGLPKGPRVKSTRPGSSDINVAPG
EQGPDQEETNTLVANTSNSNGLKLDPADPENPRSGDTVEVQVNGNLVREPDHMELEEDRA
GQLNMRGVFLHVLGDALGSVIVVVNALVFYFSWKGCSEGDFCVNPCFPDPCKAFVEIINS
THASVYEAGPCWVLYLDPTLCVVMVCILLYTTYPLLKESALILLQTVPKQIDIRNLIKEL
RNVEGVEEVHELHVWQLAGSRIIATAHIKCEDPTSYMEVAKTIKDVFHNHGIHATTIQPE
FASVGSKSSVVPCELACRTQCALKQCCGTLPQAPSGKDAEKTPAVSISCLELSNNLEKKP
RRTKAENIPAVVIEIKNMPNKQPESSL

>Pan troglodytes_SLC30A1(ENSPTRP_3292)
MGCWGRNRGRLLCMLALTFMFMVLEVVVSRVTSSLAMLSDSFHMLSDVLALVVALVAERF
ARRTHATQKNTFGWIREEVMGALNAILGLCFAILLEAIERFIEPHEMQQPLVVLGVGVAG
LLVNVLGLCLFHHHSGFSQDSGHGHSHGRHGHGHGLPKGPRVKSTRPGSSDINVAPGEQG
PDQEETNTLVANTSNSNGLKLDPADPENPRSGDTVEVQVNGNLVREPDHMELEEDRAGQL
NMRGVFLHVLGDALGSVIVVVNALVFYFSWKGCSEGDFCVNPCSPDPCKAFVEIINSTHA
SVYEAGPCWVLYLDPTLCVVMVCILLYTTYPLLKESALILLQTVPKQIDIRNLIKELRNV
EGVEEVHELHVWQLAGSRIIATAHIKCEDPTSYMEVAKTIKDVFHNHGIHATTIQPEFAS
VGSKSSVVPCELACRTQCALKQCCGTLPQAPSGKDAEKTPTVSISCLELSNNLEKKPRRT
KAENIPAVVIEIKNMPNKQPESSL

>Pongo abelii_SLC30A1(ENSPPYP_251)
MGCWGRNRGRLLCMLALTFMFMVLEVVVSRVTSSLAMLSDSFHMLSDVLALVVALVAERF
ARRTHATQKNTFGWIRAEVMGALVNAIFLTGLCFAILLEAIERFIEPHEMQQPLVVLGVG
VAGLLVNVLGLCLFHHHSGFSQDSGHGHSHGGHGHGHGLPKGPRVKSSRPGSSDINVAPG
EQGPDQEETNTLVANTSNSNGLKLDPADPENPRSGDTVEVQVNGNLVRESDHMELEEDRA
GQLNMRGVFLHVLGDALGSVIVVVNALVFYFSWKGCSEGDFCVNPCFPDPCKAFVEIINS

THASVYEAGPCWVLYLDPTLCVVMVCILLYTTYPLLKESALILLQTVPKQIDIRNLIKEL
RNVEGVEEVHELHVWQLAGSRIIATAHIKCEDPTSYMEVAKTIKDVFHNHGIHATTIQPE
FASVGSKSSVVPCELACRTQCALKQCCGTLPQAPSGKDAEKTPTVSISCLELSNNLEKKP
RRTKAENIPAVVIEIKNTPNKQPESSL

>Mus musculus_SLC30A1(ENSMUSP_42410)
MGCWGRNRGRLLCMLLLTFMFMVLEVVVSRVTASLAMLSDSFHMLSDVLALVVALVAERF
ARRTHATQKNTFGWIRAEVMGALVNAIFLTGLCFAILLEAVERFIEPHEMQQPLVVLSVG
VAGLLVNVLGLCLFHHHSGEGQGAGHGHSHGHGHGHLAKGARKAGRAGVEAGAPPGRAPD
QEETNTLVANTSNSNGLKADQAEPEKLRSDDPVDVQVNGNLIQESDNLEAEDNRAGQLNM
RGVFLHVLGDALGSVIVVVNALVFYFNWKGCTEDDFCTNPCFPDPCKSSVEIINSTQAPM
RDAGPCWVLYLDPTLCIIMVCILLYTTYPLLKESALILLQTVPKQIDIKHLVKELRDVDG
VEEVHELHVWQLAGSRIIATAHIKCEDPASYMQVAKTIKDVFHNHGIHATTIQPEFASVG
SKSSVLPCELACRTQCALKQCCGTRPQVHSGKDAEKAPTVSISCLELSENLEKKARRTKA
EGSLPAVVIEIKNVPNKQPESSL

>Rattus norvegicus_SLC30A1(ENSRNOP_6340)
MGCWGRNRGRLLCMLLLTFMFMVLEVVVSRVTASLAMLSDSFHMLSDVLALVVALVAERF
ARRTHATQKNTFGWIRAEVMGALVNAIFLTGLCFAILLEAVERFIEPHEMQQPLVVLSVG
VAGLLVNVLGLCLFHHHSGEGQGAGHGHSHGHGHGHLAKGARKAGRAGGEAGAPPGRAPD
QEPDQEETNTLVANTSNSNGLKADQAEPEKLRSDDPVDVQVNGNLIQESDSLESEDNRAG
QLNMRGVFLHVLGDALGSVIVVVNALVFYFSWKGCTEDDFCVNPCFPDPCKSSVELMNST
QAPMHEAGPCWVLYLDPTLCIIMVCILLYTTYPLLKESALILLQTVPKQIDIKHLVKELR
DVEGVEEVHELHVWQLAGSRIIATAHIKCEDPASYMQVAKTIKDVFHNHGIHATTIQPEF
ASVGSKSSVVPCELACRTQCALKQCCGTRPQVHSGKEAEKAPTVSISCLELSENLEKKPR
RTKAEGSVPAVVIEIKNVPNKQPESSL

>Canis familiaris_SLC30A1(ENSCAFP_41421)
MLLLTFMFIVLEVVVSQVTSSLVMLSDSFHMLSDVLALVVALVAERFARRTHATQKNTFG
WIRAEVMGALVNAIFLTGLCFAILLEAIERFIEPHEMQQPLVVLGVGVAGLLVNVMGLCL
FHHHSGFGNDSGHGHSHGGHGHGLPKGARGKGGRAGAGDDSETPGEQRPDAEETNTLVSN
SSNSNGLKLERPDPEKSRNDAVEVQVNGNLIRESDHVELEDDDKAAQLNMRGVFLHVFGD
ALGSVIVVVNALVFYFSWRGCPEGEFCVNPCIPDPCKAFVEIINSTHATVHEAGPCWVLY
LDPTLCIVMVCILLYTTYPLLKESALILLQTVPKQIDIKNLIKELRDVEGVEEVHELHVW
QLAGSRIIATAHIKCEDPTSYMQVAKIIKDVFHNHGIHATTIQPEFASVGSKSSVVPCEL
ACRTQCALKQCCGTRPQAPSGKDAEKAPTVSISCLELSDNLEKKPRRTKVESIPAVVIEI
KKMPNKQPESSL

>Felis catus_SLC30A1(ENSFCAP_22351)
MLMLTFMFMVLEVVVSRVTSSLAMLSDSFHMLSDVLALVVALVAERFARRTHATQKNTFG
WIRAEVMGALVNAIFLTGLCFAILLEAIERFIEPHEMQQPLVVLGVGVAGLLVNVMGLCL
FHHHSGFGNDSGHGHSHGGHGHPKGARGKSNRTGSSDNSVTPGEQRPEQEETNTLVSNSS
SSNGLKLDRPDPEKSRNDAVEVQVNGNLIREPDHMELEDDNKAGQLNMRGVFLHVFGDAL
GSVIVVVNALVFYFSWKGCPKGEFCVNPCIPDPCKAFVEIINSTHATVYEAGPCWVLYLD
PTLCIVMVCILLYTTYPLLKESALILLQTVPKQIDIKNLIKELRDVEGVEEVHELHVWQL
AGSRIIATAHIKCEDPTSYMQVAKIIKDVFHNHGIHATTIQPEFASVGSKSSVVPCELAC
RTQCALKQCCGTRPQAPSGKDAEKAPTVSISCLELSDNLEKKPKRTKAESIPAVVIEIKK
MPNKQPESSL

>Myotis lucifugus_SLC30A1(ENSMLUP_14723)
MGCWSRNRGRLLCMLLLTFMFMVLEMVVSQVTSSLAMLSDSFHMLSDVLALVVALVAERF
AQRTHATQKNTFGWIRAEVMGALVNAIFLTGLCFAILLEAIERFIEPHEMQQPLMVLAVG
VAGLVVNVLGLCLFHHHSGFGNNSGHGHSHGGHGHTKGVRGKSSRPGDSDHRSPGGQRAD
QEETNTLVAGSSNSNGLKLGGTDPEKSRINATDVQMNGNVIRESDNTDLEDEADDAQKAG
QLNMRGVFLHVFGDALGSVIVVVNALVFYFYWDSCPEGRFCENPCIADPCKALVEIINST
QGTVFEAGPCWVLYLDPTLCIVMVCILLYTTYPLLKESALILLQTVPKQIDIRNLIKELR
NVEGVEEVHELHVWQLAGSRIIATAHIKCEDPTSYMQVAKTIKDVFHNHGIHATTIQPEF
ASVGSKSSVVPCELACRTQCALKQCCGTRPQAYSEKDTEKAPTVSISCLELSENLEKPRT
QAENMPAVVIDMKKMPNKQPESSL

>Monodelphis domestica_SLC30A1(ENSMODP_3320)

MGCWGRNRARLLCMLSLTFMFMVLEMVVSRLTSSLAMLSDSFHMLSDVLALIVALVAERF
ARRTQSTQKNTFGWIRAEVMGALVNAIFLTGLCFVILLEAIERFIEPHEIKQPLVVLGVG
VAGLVVNLLGLCLFHHNGFGQGGESGGGHGHSHGGHVHGHSHGKGVRGKSNRSGGNNSNL
TESERAADGEETNNLVISSSKSNGMKLEREEEQDGDRTMDVQVNGTLIVDDSDNLDSEDE
DNRGQLNMRGVFLHVFGDALGSVIVVINALVFFFSWKSCPEGEICKNPCVPDPCEAFVGI
INSTDPVGEVGPCWVLYLDPTLCLVMVGILFYTTYPLLKESALILLQTVPKQVNIRNLKM
ELRDVDGVEEVHELHVWQLAGSRIIATAHIKCRDPASYMVVAKSIKDVFHNHGIHATTIQ
PEFASVGSKSGVAPCELACRTQCALKQCCGTAQKNKETEKAPISISCLELSDNLEKQPRR
TKIENIPAVMIDIKKMPNKQPESSM

>Danio rerio_SLC30A1a(ENSDARP_22487)
MACAPNRVRLLCMLSLTFGFFIVEVVVSRITSSLAMLSDSFHMLSDVIALVVALVAVRFA
EQTQSTNKNTFGWIRAEVMGALVNAVFLTALCFTIILEAIERFTEPHEIEQPWVVIGVGA
LGLLVNLLGLCLFHGHAGGGGHGHSHGGHGHGHSHGGAKKHKNGKVRRSETLEDSRSAGEET
NNLVANHNSPNGVNAERKAADMCHSDSLDLQINGSAPYDELENGHDAASQLNMRGVFLHV
LGDALGSVIVVINAIIFVFVWKPCEPGTICENPCSGQHCADHALNISSPLTNGTLIKAGP
CWVLYLDPTLCIIMVCILLYTTYPLLKESALILLQTVPKQIDMHQLNARLRDLEGVLAVH
ELHIWQLAGSRIIATAHIKCHDPTSYMDVAKRIKDFFHDEGIHATTIQPEFVTVNSESRA
SLCELSCRTQCAPKLCCGAVDTAKPLEGSLESKKSCESKTPTLPSASVTSLEIISESVED
TSRPESVVIITREVESSL

>Danio rerio_SLC30A1b(ENSDARP_94784)
MGLGQNRMRLLCMLTLTLVFFVVEVVVSRITASLAMLSDSFHMLSDVIALTVALIAVSFA
ETTRSTSKNTYGWIRAEVMGALVNAVFLTALCFTIFLEAVERYTQPHEIENPRVVIWVGV
AGLLVNVLGLFLFHGHAGFGGHGHAHGGHSHAGHSHAHQTNHREKPKAHEEACTLMINSS
QGDHTCEQKSDSLNIRITAKGSLEDLDQSPESASQLNMRGVFLHVLGDALGSIVVVVNAL
IFTFVWTPCHSDQICYNPCRNNHLTDHHHVNTTVLSISKTPDRIPRTISVAGPCWVLYLD
PTLCLIMVCILLYTTFPLLKESALILLQTVPEQIDMPKLKGKLKSLDGVLAVHDLHIWQL
AGARIIATAHIKCTDPALYMDIAKRIKDVFHDEGIHATTVQPEFSAFSEGFGDPQCELSC
RSQCKPKLCCNPTKRAKPEEELAKCRHDKPHSHAVEWGDGKKVDDAVHTEMESSV

>Takifugu rubripes_SLC30A1(ENSTRUP_46128)
MACEPNRARLLCMLTLTFVFFIVEVVVSRMTASLSMLSDSFHMLSDVIALIVALVAVRFA
EKSHSTKKNTFGWIRAEVMGALVNAVFLTALCFTILLEAIERFTEPHEIESPEMVAGVGA
AGLLVNLLGLCLFHGHAGGGHGHSHGGKNKKLKLGKDGKGSPAAETSTLVDNHSSPSDTK
PKNEMICKDSEDLHMNGNTLYDELEEHASSSQLNMRGVFLHVLGDALGSVIVVLNALIFT
FVWKPCPPDEMCLNPCINGHSTDHRHINQTLVGITEGPSMSAMTFAGPCWVLYLDPTLCV
IMVGILLYTTYPLLKESALILLQTVPKQIDMHQLNERLQGLEGVLAIHDLHIWQLAGSRI
IATAHIKCHDPTSYMDVAKRIKDFFHNEGIHATTIQPEFVTFSSESRDSLCELSCRTQCA
PKLCCGTADKQNAGADKKTDAECKAAAAAAASTLEVITEVPEQVAAAQTLLQPSADAVAA
RELESSL

>Tetraodon nigroviridis_SLC30A1(ENSTNIP_13679)
MACEPNRARLLCMLTLTFVFFIVEVVVSRITSSLSMLSDSFHMLSDVIALIVALVAVRFA
EKTHSTSKNTFGWIRAEVMGALVNAVFLTALCFTIVLEAIERFTEPHEIESPEVVAGVGA
AGLLVNLLGLCLFHGHAGGGHGHSHGSKNKKLKMGEAGEGSPAAETSTLVDNHNSPTDPK
PKNGKSSEITCKDSEDLQMNGNTHYDELEDEQASSSQLNMRGVFLHVLGDALGSVIVVVN
ALIFTFVWRPCGSEETCVNPCISGHSTDHQHVNHTLVDLSEGPSVWSATRAGPCWVLYLD
PTLCVIMVGILLYTTYPLLKESALILLQTVPKQIDMHRLNERLLGLDGVLAIHELHIWQL
AGSRIIATAHIKCHDPTSYMDVAKRIKDFFHDEGIHATTIQPEFVTFSSESRDSLCELSC
RTQCAPKLCCGSADKANAGPDKKSGGECRASAASALEVISEVPEEKAAAQTLLQHGAEAV
RELESSL

>Gasterosteus aculeatus_SLC30A1(ENSGACP_13084)
MTCEPNRVRLLCMLSLTFAFFIVEVVVSRMTSSLSMLSDSFHMLSDVIALVVALVAVRFA
EKTHATDKNTFGWIRAEVMGALVNGLFLTALCFTIVLEAVERFTEPHEIETPLVVAGVGA
AGLLINLLGLCLFRGHAGGGHGHSHGGHSHGNKNKRGKTGKSQKAGNGSSGEETNNLVAS
HNSPGDARPRNASEISSTDSADVQMNGSPHFEEMEHDAASQLNMRGVFLHVLGDALGSVI
VVVNSLIFTFVWQPCGKEEMCVNPCINSHISDHQHVNHTLVNLLEGTTMSAMKYAGPCWV
LYLDPTLCIIMVCILLYTTYPLLKESALILLQTVPKQINMNRLNQRLLGLEGVLAIHELH
IWQLAGSRIIATAHIKCHDPTSYMEVAKRIKDFFHDEGIHATTIQPEFVTFNSESRDSLC

ELSCRTQCAPKLCCGSADKPNAGSDKKASSYCKAASASGLEVIVETPEQAAGVEAGTRSE
AAITVAREVESSL

>Oryzias latipes_SLC30A1(ENSORLP_15324)
MACEPNRARLLCMLSLTFGFFLVEVVVSRITSSLSMLSDSFHMLSDVIALIVALVAVRFA
EKTQATNKNTFGWIRAEVMGALVNAVFLTALCFTIVLEAVERFTEPHEIDSPMVVAGVGA
AGLLINLLGLCLFHGHAGGGHGHSHGGHSHGGKGKKGKHGKSQKAGNGSAGEETNYLVGN
HTSNTKPSMAEVSCKDSTEVQMNGNTHFEEMESEADASSQLNMRGVFLHVLGDALGSVIV
VVNSVIFTFVWKPCEGPSCTNPCINSHKTDHRHINHTLVDLQDTDTKAGPCWVLYLDPTL
CIIMVGILLYTTYPLLKESALILLQTVPKQINMHRLNERLLSLDGVLAIHDLHIWQLAGS
RIIATAHIKCHDPTSYMDVAKRIKDFFHDEGIHATTIQPEFVTFNSETQGSQCELSCRTQ
CAPKLCCGQKSASDSKAAAASGLEVISEASEQVPSIQGGPRTEAEVAVTREVESSV

>Homo sapiens_SLC30A2(ENSP_363396)
MEAKEKQHLLDARPAIRSYTGSLWQEGAGWIPLPRPGLDLQAIELAAQSNHHCHAQKGPD
SHCDPKKGKAQRQLYVASAICLLFMIGEVVEILGALVSVLSIWVVTGVLVYLAVERLISG
DYEIDGGTMLITSGCAVAVNIIMGLTLHQSGHGHSHGTTNQQEENPSVRAAFIHVIGDFM
QSMGVLVAAYILYFKPEYKYVDPICTFVFSILVLGTTLTILRDVILVLMEGTPKGVDFTA
VRDLLLSVEGVEALHSLHIWALTVAQPVLSVHIAIAQNTDAQAVLKTASSRLQGKFHFHT
VTIQIEDYSEDMKDCQACQGPSD

>Pan troglodytes_SLC30A2(ENSPTRP_52651)
MEAKEKQHLLDARPAIRSYTGSLWQEGAGWIPLPRPGLDLQAIELAAQSNHHCHAQKGPD
SHCDPKKGKAQRQLYVASAICLLFMIGEVVGGYLAHSLAVMTDAAHLLTDFASMLISLFS
LWMSSRPATKTMNFGWQRAEILGALVSVLSIWVVTGVLVYLAVERLISGDYEIDGGTMLI
TSGCAVAVNITMGLTLHQSGHGHSHGTTNQQEENPSVRAAFIHVIGDFMQSMGVLVAAYI
LYFKPEYKYVDPICTFVFSILVLGTTLTILRDVILVLMEGTPKGVDFTAVRDLLLSVEGV
EALHSLHIWALTVAQPVLSVHIAIAQNTDAQAVLKTASSRLQGKFHFHTVTIQIEDYSED
MKDCQACQGPSD

>Mus musculus_SLC30A2(ENSMUSP_101499)
MQTMDKQNLLESTRGARSFLGSLWKSEASRIPPVDLPAVELAVQSNHYCHAQKDSGSHPD
PEKQRARRKLYVASAICLVFMIGEIIEILGALLSVLSIWVVTGVLVYLAVQRLISGDYEI
KGDTMLITSGCAVAVNLIMGLALHQSGHGHSHGNSRDDSSQQQNPSVRAAFIHVIGDLLQ
SVGVLVAAYIIYFKPEYKYVDPICTFLFSILVLGTTLTILRDVILVLMEGTPKGVDFTTV
KNLLLSVDGVEALHSLHIWALTVAQPVLSVHIAIAQNADAQAVLKVARDRLQGKFNFHTM
TIQIEKYSEDMKNCQACQGPLE

>Rattus norvegicus_SLC30A2(ENSRNOP_72908)
MQTMDKQNLLESTRGARSFFGALWKSEASRIPPVNLPSVELAVQSNHYCHAQKDSGSHPN
SEKQRARRKLYVASAICLVFMIGEIIGGYLAQSLAIMTDAAHLLTDFASMLISLFSLWVS
SRPATKTMNFGWQRAEILGALLSVLSIWVVTGVLVYLAVQRLISGDYEIKGDTMLITSGC
AVAVNIIMGLALHQSGHGHSHGHSHEDSSQQQQNPSVRAAFIHVVGDLLQSVGVLVAAYI
IYFKPEYKYVDPICTFLFSILVLGTTLTILRDVILVLMEGTPKGVDFTTVKNLLLSVDGV
EALHSLHIWALTVAQPVLSVHIAIAQNVDAQAVLKVARDRLQGKFNFHTMTIQIESYSED
MKSCQECQGPSE

>Canis familiaris_SLC30A2(ENSCAFP_18683)
MEPTEKQHLVDTKSGTRSYTGSLWQEGTGWIPLPSPGLDLQAIELVAESNHYCHAQKGPA
SHLEPKKERARRQLYVASAICLVFMIGEIVGGYLAHSLAIMTDAAHLLTDFASMLVSLFS
LWMSSRPATKTMNFGWQRAEILGALLSVLSIWVVTGVLVFLAVERLISGDYEIEGGTMLI
TSGCAVAVNIIMGLTLHQSSHGHSHDSSQQQENPSVRAAFIHVIGDFLQSVGILVAAYIL
YFKPEYKYVDPICTFLFSILVLGTTLTILRDVIVVLMEGTPKGVDFTAVRDLLLSVEGVE
ALHSLHIWALTVTQPVLSVHIAIAQNADGQAVLKAASTRLQGKFRFHTITIQIEDYSEDM
KDCQECQGPSD

>Felis catus_SLC30A2(ENSFCAP_11870)
XPTERQHLMDARPGARSYTGSLWQEGAGWIPLPAPGLDLQAIELAAESNHYCHAQKGPGS
HFDSKKEQARRRLYVASAICLVFMIGEVIGGYLAHSLAVMTDAAHLLTDFASMLVSLFSL
WMSSRPATKTMNFGWQRAEILGALLSVLSIWVVTGVLVFLAVERLISGDYEVEGGTMLIT
SGCAVAVNIIMGLTLHQSGHGHSHDTSQQQENPSVRAAFIHVVGDFLQSLGILVAAYILY

FKPEYKYVDPICTFLFSILVLGTTLTILRDVILVLMEGTPKGVDFTAVRDLLLSVEGVEA
LHSLHIWALTVSQPVLSVHIAIAQNADAQVVLKAASTCLQGKFRFHTMTIQIEDYSEDMK
DCQACQGPSD

>Myotis lucifugus_SLC30A2(ENSMLUP_14244)
HYCHSQKGPGSHGDPKKERARRQLYVASAICLVFMIGEVVGGYLAHSLAIMTDAAHLLTD
FASMLISLFSLWMSSRPATKTMNFGWQRAEILGALLSVLSIWVVTGVLVYLAAERLISGN
YEIEGKTMLITAGCAVAVNIIMGLTLHQSSHGHSHGQGHGHSHGQGHSHDTTQQQENPSV
RAAFIHVIGDLLQSLGVLVAAYILYFKPEYKYVERDPVLMKGNRAWGSLFALGSPALILH
FIFPILSPGTPKGVDFTAVRDLLLSVEGVEALHSLHIWALTVTQPVLSVHIAI

>Monodelphis domestica_SLC30A2(ENSMODP_17619)
MNLLRGHILELLGRMTSPIPLPETSVDLHAIGLDSQSNQHCHAQQGLSSPYNAQKERARR
QLYLASSICLVFIIGEVVGGYLAHSLAVMTDAAHLLTDFASMLISLFSLWMSSRPATKTM
NYGWHRAEILGALLSVLSIWVVTGVLVYLAVQRLISGDYEIEGGAMLITSGCAVAVNIIM
GFTLHQSGHGHGHGHGHGHNHGTSSKEEEQNPSVRAAFIHVVGDLLQSLGVLVAALILYY
KPEYKYVDPICTFLFSILVLGTTLTILRDVVLMLMEATPKGVDFTAVRDLLLSVEGVAAL
HSLHIWALTVAQPVLSVHIAIAQEADAQMVLKEARARLQGMFHFHTTTIQIEDYSEDMKD
CQECQGPSD

>Xenopus tropicalis_SLC30A2(ENSXETP_20184)
MEGETEKQLLLSGEKRSYSGIPGKDLHSQVFTYISDLNGIELGHQGNQHCHSNRELDDWN
AKEKKRARRKLYVASAVCLVFMIGEVIGGYLAHSLAIMTDAAHLLTDFASMMISLFALWM
SSRPATKTMNFGWHRAEILGALLSVLSIWVVTGVLVYLAVERIISGDYEIEGDAMLITSA
CAVAVNIIMGVTLHQTGHGHSHGDGNSHSHSHAAGDHKNPSVRAAFIHVVGDLLQSVGVL
IAAYVIYYKPEYKIIDPICTFLFSVLVLITTLTILRDVLLVLMEGTPKGVDFNLVKDTLL
SIDGVKALHSLHIWALTVTQPVLSVHIAINENADSQKVLKEASSQLQNKFHFHTTTIQIE
NYSDDMKDCQECQDPSD

>Homo sapiens_SLC30A3(ENSP_233535)
MEPSPAAGGLETTRLVSPRDRGGAGGSLRLKSLFTEPSEPLPEESKPVEMPFHHCHRDPL
PPPGLTPERLHARRQLYAACAVCFVFMAGEVVGGYLAHSLAIMTDAAHLLADVGSMMGSL
FSLWLSTRPATRTMTFGWHRSETLGALASVVSLWMVTGILLYLAFVRLLHSDYHIEGGAM
LLTASIAVCANLLMAFVLHQAGPPHSHGSRGAEYAPLEEGPEEPLPLGNTSVRAAFVHVL
GDLLQSFGVLAASILIYFKPQYKAADPISTFLFSICALGSTAPTLRDVLRILMEGTPRNV
GFEPVRDTLLSVPGVRATHELHLWALTLTYHVASAHLAIDSTADPEAVLAEASSRLYSRF
GFSSCTLQVEQYQPEMAQCLRCQEPPQA

>Pan troglodytes_SLC30A3(ENSPTRP_20201)
MEPSPAAGGLETTRLVSPRDRGGAGGSLRLKSLFTEPSEPLPEESKPVEMPFHHCHRDPL
PPPGLTPERLHARRQLYAACAVCFVFMAGEVVGGYLAHSLAIMTDAAHLLADVGSMMGSL
FSLWLSTRPATRTMTFGWHRSETLGALASVVSLWMVTGILLYLAFVRLLHSDYHIEGGAM
LLTASIAVCANLLMAFVLHQAGPPHSHGSRGAEYAPLEEGPEEPLPLGNTSVRAAFVHVL
GDLLQSFGVLAASILIYFKPQYKAADPISTFLFSICALGSTAPTLRDVLRILMEGTPRNV
GFEPVRDTLLSVPGVRATHELHLWALTLTYHVASAHLAIDSTADPEAVLAEASSRLYSRF
GFSSCTLQVEQYQPEMAQCLRCQEPPQA

>Mus musculus_SLC30A3(ENSMUSP_31037)
MEPSLATGGSETTRLVSARDRSSAGGGLRLKSLFTEPSEPLPEEPKLEGMAFHHCHKDPV
PQSGLSPERVQARRQLYAACAVCFIFMAGEVVGGYLAHSLAIMTDAAHLLADIGSMLASL
FSLWLSTRPATRTMTFGWHRSETLGALASVVSLWIVTGILLYLAFLRLLHSDYHIEAGAM
LLTASIAVCANLLMAFVLHQTGAPHSHGSTGAEYAPLEEGHGYPMSLGNTSVRAAFVHVL
GDLLQSFGVLAASILIYFKPQYKVADPISTFLFSICALGSTAPTLRDVLLVLMEGAPRSV
EFEPVRDTLLSVPGVRATHDLHLWALTLTYHVASAHLAIDSTADPEAVLAEASSRLYSRF
GFSSCTLQVEQYQPEMAQCLRCQEPSQA

>Rattus norvegicus_SLC30A3(ENSRNOP_8126)
MEPSPASGGSETTRLVSPRDRSSAGGGLRLKSLFTEPSEPLPEGPKLEGMAFHHCHKNRV
SQSGLSPERAQARRQLYAACVVCFIFMAGEVVGGYLAHSLAIMTDAAHLLADIGSMMASL
FSLWLSTRPATRTMTFGWHRSETLGALASVVSLWIVTGILLYLAFLRLLHSDYHIEAGAM
LLTASIAVCANMIMAFVLHQTGAPHSHGPRGAEYAPLEEGHGHPLSLGNTSVRAAFVHVL

GDLLQSLGVLAASILIYFKPQYKVADPISTFLFSICALGSTAPTLRDVLLVLMEGAPRSV
EFEPVRDTLLSVPGVRATHDLHLWALTLTYHVASAHLAIDSTADPEAILAEASSRLYSRF
GFSSCTLQVEKYRSEMAHCLRCREPPKA

>Canis familiaris_SLC30A3(ENSCAFP_7210)
MEPSPATGGSETTRLVSPRDRGGAGGGLRLKSLFTEPSEAVPEEPKSVELPFHHCHRDPL
PQPGLTPERLQAQRQLCAACAVCCVFMAGEVVGGYLAHSLAIMTDAAHLLADVGSMIGSL
FSLWLSTRPATRTMTFGWHRSETLGALASVVSLWMVTGILLYLAFIRLLHSDYHIEGGAM
LLTASIAVCANLLMAFVLHQAGPPHSHGSRGAEYAPLEEGPGEPLPLGNTSVRAAFVHVL
GDLLQSLGVLAASVLIYFKPQYKAADPISTFLFSICALGSTAPTLRDVLRVLMEGSPRSV
GFEPVRDTLMSVPGVRATHELHLWSLTLTYHVASAHLAIDSTADPEAVLAEATSQLHSRF
GFSSCTLQVEQYQPEMAQCLRCREPPQA

>Felis catus_SLC30A3(ENSFCAP_17271)
MEPSPATGGSETTRLVSPRDRGGAGGGLRLKSLFTEPSEPLPEEPKPEEMPFHHCHRDPL
PPPGLTPERLQAQRQLCAACAVCCVFMAGEVVGGYLAHSLAIMTDAAHLLADVGSMMGSL
FSLWLSTRPATRTMTFGWHRSETLGALASVVSLWMVTGILLYLAFIRLLHSDYHIEGGAM
LLTASIAVCANLLMAFVLHQAGPPHSHGSRGAEYAPLEEGPGEPLPLGNTSVRAAFVHVL
GDLLQSLGVLAASVLIYFKPQYKAADPISTFLFSICALGSTAPTLRDVLRVLMEGSPRSV
GFEPVRDTLLSVPGVRATHELHLWSLTLTYHVASAHLAIDSTADPEAVLAEATSQLQSRF
GFSSCTLQVEQYQPEMAQCLRCREPPQA

>Myotis lucifugus_SLC30A3(ENSMLUP_1312)
MEPAPAAGGSETTPWRAPRDRESTGEGVRLLSLFTEPMESTAEETTFHCHRDPLPPPGLT
PERLRAQRQLCVACAVCCVFMAGEVVGGYLAHSLAIMTDAAHLLADVGSMLGSLFSLWLS
TRPATRTMTFGWHRSETLGALASVISLWMVTGILLYLAFIRLLHSDYHIEGGAMLVTAGI
AVCANLLMAFVLHQAGPQHSHGSRGAEYAPLEEGPGEHLPLGNTSVRAAFVHVLGDLLQS
LGVLAASILIYFKPQCKAADPISTFLFSICALGSSAPTLRDVLRVLMEGAPHNVGFEPVR
DALLSVPGVQATHELHMWSLTLTYHIASAHLAIDSTADPDAVLAEATSRLYSRFGFSSCT
LQVEQYQSEMDQCLRCQEPPQA

>Ornithorhynchus anatinus_SLC30A3(ENSOANP_20322)
SLFSEPSEPLPAESAAPEQPRPQHCHGALAPQAGPSAERLRARKQHGAACAVCCLFMAGD
VAGGYLAHSLAIMTDAVHLLADVGSMAGSLFSLWLSARPATRTMTFGWHRSETLGALASV
LSLWLVTGFLLYLASLRLLHSDYHIEGGAMLFTAGIAVCANLLMAFVLHQASPTHSHGAG
GAGYMPLEEGQEAPLHLGNTSVRAAFVHVLGDLLQSFGVLVAATLIYFKPQYKAADPIST
FLFSACALGSTAPTLRDVLRVLMEGAPPNIGFEPVRDALLGVPGVRATHELHLWALTLTY
HVASAHLAIDEAADPEAVLAEASSRLHARFGFASCTFQVERYQPDMAQCPHCQEP

>Homo sapiens_SLC30A4(ENSP_261867)
MAGSGAWKRLKSMLRKDDAPLFLNDTSAFDFSDEAGDEGLSRFNKLRVVVADDGSEAPER
PVNGAHPTLQADDDSLLDQDLPLTNSQLSLKVDSCDNCSKQREILKQRKVKARLTIAAVL
YLLFMIGELVGGYIANSLAIMTDALHMLTDLSAIILTLLALWLSSKSPTKRFTFGFHRLE
VLSAMISVLLVYILMGFLLYEAVQRTIHMNYEINGDIMLITAAVGVAVNVIMGFLLNQSG
HRHSHSHSLPSNSPTRGSGCERNHGQDSLAVRAAFVHALGDLVQSVGVLIAAYIIRFKPE
YKIADPICTYVFSLLVAFTTFRIIWDTVVIILEGVPSHLNVDYIKEALMKIEDVYSVEDL
NIWSLTSGKSTAIVHIQLIPGSSSKWEEVQSKANHLLLNTFGMYRCTIQLQSYRQEVDRT
CANCQSSSP

>Pan troglodytes_SLC30A4(ENSPTRP_12029)
MAGSGAWKRLKSMLRKDDAPLFLNDTSAFDFSDEAGDEGLSRFNKLRVVVADDGSEAPER
PVNGTHPTLQADDDSLLDQDLPLTNSQLSLKVDSCDNCSKQREILKQRKVKTRLTIAAVL
YLLFMIGELVGGYIANSLAIMTDALHMLTDLSAIILTLLALWLSSKSPTKRFTFGFHRLE
VLSAMISVLLVYILMGFLLYEAVQRTIHMNYEINGDIMLITAAVGVAVNVIMGFLLNQSG
HRHSHSHSLPSNSPTRGSGCERNHGQDSLAVRAAFVHALGDLVQSVGVLIAAYIIRFKPE
YKIADPICTYVFSLLVAFTTFRIIWDTVVIILEGVPSHLNVDYIKEALMKIEDVYSVEDL
NIWSLTSGKSTAIVHIQLIPGSSSKWEEVQSKANHLLLSTFGMYRCTIQLQSYRQEVDRT
CANCQSSSP

>Mus musculus_SLC30A4(ENSMUSP_5952)
MAGPGAWKRLKSLLRKDDTPLFLNDTSAFDFSDEVSDEGLSRFNKLRVVVADDDSEAPER

PVNGAHPALQADDDSLLDQDLPLTNSQLSLKMDPCDNCSKRRELLKQRKVKTRLTIAAVL
YLLFMIGELVGGYMANSLAIMTDALHMLTDLSAIILTLLALWLSSKSPTRRFTFGFHRLE
VLSAMISVMLVYVLMGFLLYEAVQRTIHMNYEINGDVMLITAAVGVAVNVIMGFLLNQSG
HHHSHAHSHSLPSNSPSMVSSGHNHGQDSLAVRAAFVHALGDLVQSVGVLIAAYIIRFKP
EYKIADPICTYIFSLLVAFTTFRIIWDTVVIILEGVPSHLNVDYIKESLMKIEDVYSVED
LNIWSLTSGKSTAIVHMQLIPGSSSKWEEVQSKAKHLLLNTFGMYKCTIQLQSYRQEVIR
TCANCHSSST

>Rattus norvegicus_SLC30A4(ENSRNOP_184)
MAGPGAWKRLKSLLRKDDTPLFLNDTSAFDFSDEVSDEGLSRFNKLRVVVADDDSEAPER
PVNGAHPALQADDDSLLDQDLPLTNSQLSLKMDPCDNCSKRRELLKQRKVKTRLTIAAVL
YLLFMIGELVGGYMANSLAIMTDALHMLTDLSAIILTLLALWLSSKSPTRRFTFGFHRLE
VLSAMISVMLVYVLMGFLLYEAVQRTIHMNYEINGDVMLITAAVGVAVNVIMGFLLNQSG
HHHSHAHSHSLPSNSPSMVSSGHSHGQDSLAVRAAFVHALGDLVQSVGVLIAAYIIRFKP
EYKIADPICTYIFSLLVAFTTLRIIWDTVVIILEGVPSHLNVDYIKESLMKIEDVYSVED
LNIWSLTSGKATAIVHMQLIPGSSSKWEEVQSKAKHLLLNTFGMYKCTVQLQSYRQEATR
TCANCQSSST

>Canis familiaris_SLC30A4(ENSCAFP_32670)
MAGSSAWKRLKSLLRKDDAPLFLNDTSAFDFSDEVGDEGLSRFNKLRVVVADDGSEIPER
PINGAHPALQADDDSLLDQDLPLANSQLSLKVDPCDNCSRQRELLKQRKVKTRLTIAAVL
YLLFMIGELVGGYIANSLAIMTDALHMLTDLSAIILTLLALWLSSKSPTKRFTFGFHRLE
VLSAMISVLLVYILMGFLLYEAVQRTIHMKYEINGDIMLITAAVGVAVNVIMGFLLNQSG
HHAHSHSLPSNSPTIGSGCRHNHGQDSLAVRAAFVHALGDLVQSVGVLIAAYIIRFKPEY
KIADPICTYVFSLLVAFTTFRIIWDTIVIILEGVPSHLNVDYIKEALMKIEDVYSVEDLN
IWSLTSGKPTAIVHIQLIPGSSSKWEEVQSKAKHLLLNTFGMYKCTIQLQSYRQEVDRTC
AHCQSSSP

>Felis catus_SLC30A4(ENSFCAP_23880)
XGAWKRLKSLLRKDDAPLFLNDTSAFDFSDEVGDEGLSRFNKLRVVVADDGSEIPERPVN
GAHPALQADDDSLLDQDLPLTNSQLSLKVDPCDNCSRQRELLKQRKVKTRLTIAAVLYLL
FMIGELVGGYIANSLAIMTDALHMLTDLSAIILTLLALWLSSKSPTKRFTFGFHRLEVLS
AMISVLLVYILMGFLLYEAVQRTIHMKYEINGDIMLITAAVGVAVNVIMGFLLNQSGHHA
HSHSLPSNSPTVGSGCRHNHGQDSLAVRAAFVHALGDLVQSVGVLIAAYIIRFKPEYKIA
DPICTYVFSLLVAFTTFRIIWDTIVIILEGVPSHLNVDYIKEALMKIEDVYSVEDLNIWS
LTSGKPTAIVHIQLIPGSSSKWEEVQSKAKHVLLNTFGMYKCTIQLQSYRQEVDRTCAHC
QSSSS

>Myotis lucifugus_SLC30A4(ENSMLUP_5017)
VNGAHPALQDDDSLLDQDLPLTNSQLSLKVDPCDTCSRRRELLKQRKVKTRLTIAAVLYL
LFMIGELVGGYIANSLAVMTDALHMLTDLSAIILTLLALWLSSKSPTKRFTFGFHRLEVL
SAMISVLLVYILMAFLLYEAVQRTIHMNYEINGDIMLITAAVGVAVNVIMGFLLNQSGHH
HAHSHSLPSNSPTTGPGYGHNHGQDSLAVRAAFVHALGDLVQSVGVLIAAYIIRFKPEYK
IADPICTYVFSLLVAFTTFRIIWDTVVIILEGVPRHLHVDYIKEALMKIEDVHSVEDLNV
WSLTSGKPMAIAHIQLIPGSSSQWEEVQSKARHLLLNTFGMYKCTIQLQSYRQDVDRTCA
NCQNASS

>Monodelphis domestica_SLC30A4(ENSMODP_21986)
MARASVWNRLKSWWKKDDTPPFLNDTSAFDFSDEVGDEDFPRFNKLRVVVADDGLETPET
INGTHLAFQADDDSLLDQDLPLPNSQLGFKVDPCDSCRKQRELLKLKKVKTRLTIAGVLY
LLFMIGELVGGYIANSLAIMTDALHMLTDLSAIILTLLALWLSAKSPTKRFTFGFHRLEV
LSAIISVLLVYILMGFLLYEAVQRTIHMNYEINGDIMLITAAIGVAVNVIMGFLLNQSGH
HHSHSHSPPPNSPTTGPGHGHGHNQGNDSLAVRAAFVHALGDLVQSIGVLVAAYIIRFKP
EYKIADPICTYIFSLLVAFTTFRIIWDTIVIILEGVPSHLNVDFIKEALMKIEDVHSVED
INLWSLTSGKTTAIVHLQLAPGSSSEWEEVQSKARHLLINTFGIYKCSVQLQSYRRAANK
TCGGCRSASA

>Xenopus tropicalis_SLC30A4(ENSXETP_47719)
MWRPQAWWSSVRSAFQRSDQPPYLNDATGFDCTEEPALEGEEVLPRFNKLRVLVADEPVA
ANGVPMNMASDDESLLESWQNRVKTEPCEKCSNHRERRKQGKVKRKLLLAAALYLLFMVG
ELVGGYVANSLAIMTDALHMLSDLSSIILTLLALCLSSKPPNKRFTFGFHRLEVLSAIVS

VILVYILTGFLLYEAIQRTIHMDYNINGDVMLITAAVGVAVNLIMGILLSQSGHHHSHSH
GPAANSPSPHGHSHGSLAVRAAFVHALGDLAQSIGVLIAAYIIRFKPEYKIADPICTYIF
SVLVVFTTVRLVWDTVLIILEGVPRHLNVDRIKDDLMKIEDVYSVKDLNVWSLTTGKSAA
IIHLQLCPDASSKWEEVQNKARHVLLKTHGMYKCFIQIQSYGQEESTRCTDCSNA

>Danio rerio_SLC30A4(ENSDARP_136240)
MSGLIGRVRSAFRSRPDSWHLSDAAAFDFSEDLQEEESPRFSRLQVVVSGEAEERPAVNG
SARSTLTDDDDSLLGASTGSTGPEACDGCQRNREQQRRRRVMRKLGGAAVLYFLFMVGEL
IGGYVANSLAIMTDALHMLTDLIGIIVSLLALWLSAKPPTYRFTFGLHRLEVLSAGISVL
LIYILTGVLLNEAAQRTIHQDFTIDGDVMLITAAVGVAVNLLMGFLLNQSGHLHSHGHGH
GHSHGSAGHGSSDQGSSGQGQRGHGSLAVRAAFIHALGDLVQSVGVLIAAYIVRFKPEYK
LADPICTYLFSVLVLFSTFQIIRDTGIILLEGVPRHMDVSVIRAELLKLQHVECVEELKL
WALTADKTAAIVHLQLAPGIVGNWEEVQASARRLLMVSHGVTHCTQLQITPGENNTRIIT
CIQCEFQMKESTYCKDESR

>Takifugu rubripes_SLC30A4(ENSTRUP_43292)
MSAANLFNKVRSTFRRDPAGLDLNDTTPIDFSDELVEDEPPRFNHLKVVVSGETSGFAAG
ASANGVAVNTLVVTDDDDSLIESSSMGSSSGLNTHPCDNCTKKREMMKNKKVMKKLVIAA
VLYLLFMTAEIIGGYVSNSLAIMTDAVHMLTDVVGILFSLLALWLSNKPPTKRFTFGLHR
LEVISAVLSVALIYILTAVLVYEAVLRTVSQDFDIDGDVMLITAAVGVAVNIVMGFLLNQ
DGHLHSHGHGHSHAHGHGHSHSRGHGHSHGASGSPETTQKQHGSLAVRAAFIHALGDLFQ
SVGVLIAAYVVRFKPELKLADPICTYVFSILVLVTTVRIIRDTVVIVLEGVPRHLDTLQI
KEDLLKLDDVQAVVELNVWALTTDKTAALVHLQLRPSSGNKWEDVQSKARHLLMRTHGLS
RCTVQVQTYRERLEHSCDNCRQSVA

>Tetraodon nigroviridis_SLC30A4(ENSTNIP_12410)
MSAGNFFNKVRSAFRREPADLDLNDTTPIDFSNDLMEDEAPRFNHLRVMVSGETSGYSSR
STSNGVAVNTLVVTDDDDSLMESSSVSSSSSGPAVHPCEGCTRRKEMLKNKKIIKRLVIA
AGLYLFFMTAEIIGGYLSNSLAIMTDAVHMLTDVVGILFSLLALWLSNRPPTKRFTFGLH
RLEVISALLSVALIYILTAILVYEAVLRTISQEFDIDGDVMLIVAAIGVAVNIIMGFLLN
QDGHLHGHGHSHGHGHSHGHGHGHGHSHSRGHMAVRAAFIHALGDLFQSVGVLVAAYVVR
FKPELKLADPICTYVFSVLVLVTTVRIIRDAMVIVLEGVPRHLDSLQMKEDLLKLDDVQA
VDELNVWALTTDKTAALVHLQLRPSSGSKWEDVQAKARHLLMHIHGLSRCTVQVQTYRER
LENSCLNCRQSIA

>Gasterosteus aculeatus_SLC30A4(ENSGACP_22242)
KMSGGSLFSKVRSSFRRERNDWHLSDTAPFDFSDELAEDEAPKFNKLRVVVSGEMSDYSA
GAPSNGVAVSTLAVAAGDDDDDDCLLDSSVGPGSPGLNMDPCDSCTKKRDGVKHRRVMRR
LVFAALLYFLFMTAEIVGGYLSNSLAIMTDAVHMLTDVVGILFSLLALWLSTKPPTSRFT
FGLHRLEVVSAVLSVVLIYVLTAVLLYEAIQRTVHQEFDIDGDIMLITAAVGVAVNLIMG
FLLNQNGHLHSHGHGHAHAHGHSHHQGSLAVRAAFIHALGDLVQSVGVLIAAYIVRFKPE
LKLADPICTYIFSILVLFTTFRIMRDTAVIVLEGVPRHLDTLRIKEDLLKLEDVQSVDEL
NIWALTADKTAAIVHLQLAPSSSSSWEDVQAKARHLLLHTYGLTRCTVQVQTHRLRLVHS
CAHCQLS

>Oryzias latipes_SLC30A4(ENSORLP_3059)
PWRPLAYEGGNLLSKVRSAFRRERSDWDLSDTAPFDFSDELVEDETPKFNKLKVVVSGEM
TDYAAGAPSNGVTVSTLVADDDDSLLDSSSNSGCPGLNMDPCDNCTKKRERIKHRRVMKR
LVIAALLYFLFMTGEIIGGYVSNSLAIMTDAVHMLADVVGILFSLLALWLSTKPPTKRFT
FGLHRLEVVSAVLSVVLIYILTGVLLYEAVQRTVHQDFSIDGDVMLITAAVGVAVNLIMG
FLLNQGGHLHSHSHGHGHSHSSAAAAGSRPAGSGQPHGSLAVRAAFIHALGDLLQSVGVL
IAAYVVRFKPEFKLADPICTYIFSVLVLFTTIRIIRDTIVIVLEGVPRHLDTQRIREDLL
KLEDVQSIDELNVWALTADKTAAIVHLQLTPSSGHSWEDVQAKARHLLLHTYGLSRCTVQ
VQTHRERMVRSCANCLQP

>Homo sapiens_SLC30A8(ENSP_415011)
MEFLERTYLVNDKAAKMYAFTLESVELQQKPVNKDQCPRERPEELESGGMYHCHSGSKPT
EKGANEYAYAKWKLCSASAICFIFMIAEVVGGHIAGSLAVVTDAAHLLIDLTSFLLSLFS
LWLSSKPPSKRLTFGWHRAEILGALLSILCIWVVTGVLVYLACERLLYPDYQIQATVMII
VSSCAVAANIVLTVVLHQRCLGHNHKEVQANASVRAAFVHALGDLFQSISVLISALIIYF
KPEYKIADPICTFIFSILVLASTITILKDFSILLMEGVPKSLNYSGVKELILAVDGVLSV

HSLHIWSLTMNQVILSAHVATAASRDSQVVRREIAKALSKSFTMHSLTIQMESPVDQDPD
CLFCEDPCD

>Pan troglodytes_SLC30A8(ENSPTRP_35104)
MEFLERTYLVNDKAAKMYAFTLESVELQQKPVNKDQCPRERPEELESGGMYHCHSGSKPT
EKGANEYAYAKWKLCSASAICFIFMIAEVVGGHIAGSLAVVTDAAHLLIDLTSFLLSLVS
MWLSSKPPSKRLTFGWHRAEILGALLSILCIWVVTGVLVYLACERLLYPDYQIQATVMII
VSSCAVAANIVLTVVLHQRCLGHNHKEVQGNASVRAAFVHALGDLFQSISVLISALIIYF
KPEYKIADPICTFIFSILVLASTITILKDFSILLMEGVPKSLNYSGVKELILAVDGVVSV
HSLHIWSLTMNQVILSAHVATAASRDSQVVRREIAKALSKSFTMHSLTIQMESPVDQDPD
CLFCEDPCD

>Oryctolagus cuniculus_SLC30A8(ENSOCUP_7337)
MEFLERTYLVNDKATKMYACTLDSVELQQKPVNKDQCPGEGPGELEAGGIYHCHSNSKAT
ENRANEEVQAKWKLRAASAICFVFMIAEVVGGHIAGSLAIITDAAHLLIDLTSFLLSLFS
LWLSTKPPSKRLTFGWHRAEILGALLSILCIWLVTGVLVYLACERLLHPDYQIQGTVMII
VSGCAVVANIILTMVLHQRRLGHNHKEMQTNASVRAAFVHALGDLFQSISVLISALVIYF
KPDYKIADPICTFIFSILVLASTITILKDFSILLMEGVPKGLNYNGVKELIMAVDGVVSV
HSLHIWSLTMNQVILSAHVATAASWDGQVVRREIAKALSKSFAVHSLTIQMESPADQDPD
CIFCEAPQD

>Canis familiaris_SLC30A8(ENSCAFP_1179)
MEFLERTYLVNDRATKMYAFNLDSVELQQKSLNKDQCPGEKPEELESGAIYHCHSNSKAT
ENRANEQVYAKWKLYAASGVCFIFMIAEVVGGHIAGSLAVITDAAHLLIDLTSFLLSLFS
LWLSSKPPSKQLTFGWHRAEILGALLSILCVWVVTGVLVYLACERLLYPDYQIQGTVMIL
VSGCAVAANIMLSVILHQKHPGHNHKEVQANASVRAAFVHALGDLFQSISVLTSALIIYF
KPDYKMADPICTFVFSILVLASTITVLKDFSILLMEGVPKNLNYSDVKELILAVDGVVSV
HSLHIWSLAMNQVILSAHVAAAASRDSQVVRREIVKALSNSYTVHSLTIQMESPADQDPN
CFFCEDPRD

>Felis catus_SLC30A8(ENSFCAP_6576)
MEFLERTYLVNDKATKMYAFTLDSVELQQKSLNKNQCPGEKPEELDSGAIYHCHSNSKAT
ENRANEQVYAKWKLCAASGICFVFMIAEAVGGHIAGSLAVVTDAAHLLIDLTSFLLSLFS
LWLSSKPPSKQLTFGWHRAEILGALLSILCVWVVTAVLVYLACERLLYPDYQIQATVMII
ISGCAVAANIILSVILHQRHAGHNHKEVQANASVRAAFVHALGDLFQSISVLTSALIIYF
KPDYKMADPICTFVFSILVLASTITVLKDFSILLMEGVPKNLNYDDVKELILAVDGVVSV
HSLHIWSLAMNQVILSAHVAAAASRDSQVVRRAIVKVLNDSFTVHSLTIQMESPADQDPD
CFFCEEPRD

>Myotis lucifugus_SLC30A8(ENSMLUP_2351)
MEFPERTYLVTDKATKMYAFTLDSVDRQQKPVTKDHCSGEKPEVLEPGAIYHCHSNAKAT
ENRANEQVYAKWKLWAASGICFVFMIAEVVGGHIAGSLAVVTDAAHLLIDLTSFQLSLFS
LWLSSKPPSKRLTFGWHRAEILGALLSMLCVWAVTGVLVYLACERLLYPDYQIQATVMII
VSGCAVAANIVLSVILHQRPPGHSHKEVQANASVRAAFVHALGDLFQSISVLTSALIIYF
KPDYKMADSICTFVFSVLVLASTVTVLKDFSILLMEGVPKNLNYNAVKELILAVDGVVSV
HSLHIWSLTTNQVILSAHVAAAASRDSQSVRREIAKVLSNSFPVHSLTIQMESPADQDPG
CLFCEEPQD

>Gallus gallus_SLC30A8(ENSGALP_25920)
MAAKKGPERACLVSERDTKKYSLALDRVSWHQKNFNEERQQQEGENAEANQAYHCHGYSQ
AYEDRKMEQHQARRKLYTASVICIIFMVAEITGGQIAGSLAVVTDAAHILVDLTSFLISL
FSLWLTSKPPTKHLTFGWHRAEILGALMSMIIIWMVTGVLTYLASMRLLHTDYDIDAAVM
LITSACAVFANILLSLILHQSGHGHSHGAQARETTLAPLEKPVLSNASLRAAFVHAIGDL
FQSVSVLISALIIFFKPEYKIADPICTFVFSIFVLATTITILRDILIVLMEGTEKGLSYD
AVKARILAVEKVESVHNLHLWSLTMNQTILSAHVATADTVDSQKMLKDITQALFEHYSFH
SITIQIESGGDQEQNCVFCQEPRD

>Anolis carolinensis_SLC30A8(ENSACAP_10676)
MASKKSLERACLVNDRDTKRYSLTLSSNGSNSDQENHHEEPVGTGIHAVHHCHSYSKGYE
RRKREQHEARLRLCVATVICIIIMVAEIIGGHVAGSLAVITDAAHVLVDLMSFLISILSL
WLSSKSPSKRLTYGWHRAEILGALFSMIIIWVVTGVLTYLAILRLLHPQYEIEATVMLIT

SGCAVIANIILSLMLHHKGGGNLYNVHQSSDGRSDPERTLASSNASVRAAFIHAVGDLFQ
SISVLISALIIFLKPEYKMADPICTFVFSLLVLGTTCAMLRDILLVLMEGMPKGMSYKAV
KERILTVDKVESVHSLHVWSLTMNQPVLSAHIVTDTANKQQILNEITELLIDTYNFYTVT
LQIEPKADQQPDCVFCQDPRD

>Danio rerio_SLC30A8(ENSDARP_133246)
MFTKKPEKIHLVSENSAKMYTIMSESMPSVDMKDGKHCHDNNRALEDREREKKIAKKRLY
IVSAVCLVFMVGEILGGYFAGSLAVMTDAAHLLVDLTSFIISLCSLWLSSRPATRTLNYG
WHRAEILGALLSIFTIWLVTGVLVYLAVERIINDNFTIEGTVMLITSGCAVLANIIMALT
LHQSGHGHSHGGLSAGHGHSHDHGKENGHSHSDGSHHDVEEQRPGKTQANASVRAAFVHV
IGDLLQSISVLVSALIIFFKPEYKIADPICTFLFSLFVLGTTFTIMRDIVIVLMEGTPAG
VNYNEVRQLLLGVKGVKAVHNLHIWALTMNQAVLSAHVATDETVDPQEVLREMTQVCFTK
YSFHSVTIQLEPQADQRPDCSLCQDPAK

>Takifugu rubripes_SLC30A8(ENSTRUP_7845)
MFKQKSSERRRLLSGEENSYSAAGRSFRMSKLSDSASQEEPLKDGALSIKHCHDNSHSRE
DREQEKKVARRRLYVASVVCVIFMTGEILGGYFAGSLAVMTDAAHLLVDFLSFIISLLSL
WLSSRPPTHKLNYGWHRAEILGALLSVFTIWVVTGVLVYLAVQRLISNDYTIEGTIMLIT
SACAVLANIIMALTLHQSGHGHSHGGLGSKGHSHKTPERSRRRTQQANASVRAAFVHVVG
DLLQSISVLISAIIIFFKPEYKMADPICTFLFSVFVLFTTFTIIRDILIVLMEGAPAGLK
YSDVRDGLLAVNGVTAVHNLHIWALTMNQAVLTAHVAIDETADAHSVLREMTQACFSSYN
FHSVTIQMEKQSDLKPGCDLCEDPQL

>Tetraodon nigroviridis_SLC30A8(ENSTNIP_7337)
GGYFAGSLAVMTDAAHLLVDFLSFIISLLSLWLSSRPATHRLNYGWHRAEILGALLSVFT
IWVVTAVLVYLAVERLINNDYTIEGKIMLITSGCAVLANIIMALTLHQSGHGHRHRTQQA
NASVRAAFVHVVGDLLQSISVLISAIIIFVKPEYKIADPICTFLFSIFVLFTTVTIIRDI
VIVLMEGAPVGVKYSDVRDGLLAVKGVRAVHNLHIWALTMNQAVLTAHVAIDETVDAQSV
LREMTQACFSSYNFHSVTIQMEKTADLKPGCNLCEDPKL

>Gasterosteus aculeatus_SLC30A8(ENSGACP_3931)
MFKKKDPERTGLVSDLRSSYTALARCASSVPNQMALQIDGTGGIKHCHDNSSAQEDRERE
KKVAQRRLYVVSVICLLFMIGEIIGGYLAGSLAVMTDAAHLLADFISFLISLLSLWLSSR
PATHKLSYGWHRAEILGSLLSVFTIWLVTGVLVYLAVERLVSNDYTIEGTIMLITSGCAL
LANIVMALILHQSGHGHSHGGVGSHGHSHKGGRSQQDNASVRAAFVHVLGDLLQSVSVLL
SAIIIFFKPEYKIADPICTFLFSIFVLCTTFTILRDILVVLMNGTPAGVKYGEVRDSLLE
VKGVKAVHNLHIWALTMNQTVLTAHVAIDETVDAQTVLREMTQACISSYNFHSVTIQMER
QADLKPGCTLCEEPKM

>Oryzias latipes_SLC30A8(ENSORLP_2202)
NFHKTTYLLTIKAANFFTFYKKSIIMTFFPKTKNQITFKIPPNLEGLQGGAEHCHDNSRA
QEDRETEKKVAKRRLYVVSVICLVFMVAEIVGGYLAGSLAVMTDAAHLLTDLTSFLISLF
SLWLSSKPATQKLSFGWHRAEILGALLSVFTIWLVTGVLVYLAVERLVTDDFTIEGSIML
ITSGCAVVANIIMAVTLHQSGHGHSHGGRTQQANASVRAAFVHVIGDLLQSISVLISAIV
IFFRPEYKIADPICTFLFSILVLCTTFTIMRDILLVLMEGTPSGVKYSEVRDRLLAVKGV
TAVHNLHIWALTMNQAMLSAHVAIDDSADAQMVLREMTQACFSSYNFHSVTIQMERQADL
KPGCSLCENPKK

>Homo sapiens_SLC30A10(ENSP_355893)
MGRYSGKTCRLLFMLVLTVAFFVAELVSGYLGNSIALLSDSFNMLSDLISLCVGLSAGYI
ARRPTRGFSATYGYARAEVVGALSNAVFLTALCFTIFVEAVLRLARPERIDDPELVLIVG
VLGLLVNVVGLLIFQDCAAWFACCLRGRSRRLQQRQQLAEGCVPGAFGGPQGAEDPRRAA
DPTAPGSDSAVTLRGTSVERKREKGATVFANVAGDSFNTQNEPEDMMKKEKKSEALNIRG
VLLHVMGDALGSVVVVITAIIFYVLPLKSEDPCNWQCYIDPSLTVLMVIIILSSAFPLIK
ETAAILLQMVPKGVNMEELMSKLSAVPGISSVHEVHIWELVSGKIIATLHIKYPKDRGYQ
DASTKIREIFHHAGIHNVTIQFENVDLKEPLEQKDLLLLCNSPCISKGCAKQLCCPPGAL
PLAHVNGCAEHNGGPSLDTYGSDGLSRRDAREVAIEVSLDSCLSDHGQSLNKTQEDQCYV
NRTHF

>Pan troglodytes_SLC30A10(ENSPTRP_3342)
MGRYSGKTCRLLFMLVLTVAFFVAELVSGYLGNSIALLSDSFNMLSDLISLCVGLSAGYI

ARRPTRGFSATYGYARAEVVGALSNAVFLTALCFTIFVEAVLRLARPERIDDPELVLIVG
VLGLLVNVVGLLIFQDCAAWFACCLRGRSRRLQQRQQLAEGCAPGAFGGPQGAEDPRRAA
DPTAPGSDSAVTLRGTSVERKREKGATVFANVAGDSFNTQNEPEEMMKKEKKSEALNIRG
VLLHVMGDALGSVVVVITAIIFYVLPLKSEDPCNWQCYIDPSLTVLMVIIILSSAFPLIK
ETAAILLQMVPKGVNMEELMSKLSAVPGISSVHEVHIWELVSGKIIATLHIKYPKDRGYQ
DASTKIREIFHRAGIHNVTIQFENVDLKESLEQKDLLLLCNSPCISKGCAKQLCCPPGAL
PLAHVNGCAEHNGGPSLDTYGSDGLSRRDAREVAIEVSLDGCLSDHGQSLNKTQEDQCYV
NSTHF

>Mus musculus_SLC30A10(ENSMUSP_53181)
MGRYSGKTCRLLFMLVLTAAFFVAELVSGYLGNSIALLSDSFNMLSDLISLCVGLGSGYI
ARRGPRGSSATYGYVRAEVVGALSNAVFLTALCFTIFVEAVLRLARPERIDDPELVLIVG
ALGLAVNVVGLLIFQDCGACFSRCTRGRRTRPSQQPSQGDPRGALGCPQEAATATAPGSG
TAVTLRGSSAGRKQQEGATVFSNVAGDSLNTENEPEETTKKEKKSEALNIRGVLLHVMGD
ALGSVVVVITAIIFYVQPLRREDPCNWQCYIDPSLTVVMVIIILSSAFPLIKETAVILLQ
MVPKGVNMEELMSQLSTVPGISSVHEVHIWELISGKIIATLHIKHQKGTEYQDASRKIRE
IFHHAGIHNVTIQFETLDLKEALEQKDFLLTCSAPCITQSCAKKLCCPPGTLPLALVNGC
AEHNGRSSRESYRSIEAPEVAIDVDGCPREQGQTLSKTQERQHYENSTHF

>Rattus norvegicus_SLC30A10(ENSRNOP_50013)
MGRYSGKTCRLLFMLVLTAAFFVAELVSGYLGNSIALLSDSFNMLSDLISLCVGLGSGYI
ARRGPRGSSATYGYVRAEVVGALSNAVFLTALCFTIFVEAVLRLARPERIDDPELVLIVG
ALGLAVNVVGLLIFQDCGACFSRCTRGHRTRPSQPPRQGDTGGALGCPREAATATDPGSD
SAVTLRGASAGRKQQEGATVFSNVAGDSLNTQNEPEETTKKEKKSEALNIRGVLLHVMGD
ALGSVIVVVTAIIFYVRPLRHEDPCNWQCYIDPSLTVVMVIIILSSAFPLIKETAVILLQ
MVPKGVNMEELMSQLSMVPGVSSVHEVHIWELISGKVIATLHIKHQEGTEYQDASRKVRE
IFHHAGIHNVTIQFETLDLKEALEQKDFLPTCSAPCISQSCAKKLCCPPGTLPLALVNGC
AEHNGGPNLESYRSIEAPEVVIDVALDGCPRKQGQVHSKTQERQHCENSTHF

>Canis familiaris_SLC30A10(ENSCAFP_15955)
MGRYSGRSFRLILMCIVSILLFVMELVIANIGNSLSLASDAFAVLSHFLSMIIALFGVRV
SSIKQHRKSTFGFLRADVVGAFGNSIFAVALMFSILVEAVKRYINPQKTEEPLLILSAGI
IGLLFNILKVNYYQICLSFFTLCLELANSRQSTRVLVVGGDCPGAEGARPDAYKMPRVAC
RPVVPCSTRIVDLRGKMKKKKRKKEINLFLNNAGDSLNTQNEPEETMKKEKKSEALNIRG
VLLHVMGDALGSVVVVITAIIFYVRPLQPEDPCNWQCYIDPSLTIIMVIIILSSAFPLIK
ETAAILLQMVPKGVNMEELMSKLSAVPGISSVHEVHIWELISGKIIATLHIKYQKDRDYQ
DASRKIREIFHSVGIHNVTIQFEQVDLKDPVEQKDLLLLCSSPCISKGCEKQLCCPPGAL
PLAHVNGCAEHNGCPPLDTYPSDGLRRRETTEVAIEVSLDSCLSEHGQAPSKSQEDPHYV
NSTHF

>Felis catus_SLC30A10(ENSFCAP_14129)
MGRYSGKTCRLLFMLVLTVAFFVAELVSGYLGNSIALLSDSFNMLSDLISLCVGLGAGYV
ARRPRGGLGATYGYARAEVLGALSNAVFLTALCFTISVEAVLRLARPERIDDPELVLIVA
SFSFLASFPFPLFYLLWSIDCLENAVCTLFSPSPFAHSSVPLGVTGFGDGGRLPLASFFF
LAKMYHSGICVRVCWGRGGRGRGSDELVGNAGDSLNTQNEPEETMKKEKKSEALNIRGVL
LHVMGDALGSVVVVVTAIIFYVRPLKGEDPCNWQCYIDPSLTVIMVAIILSSAFPLIKET
AAILLQMVPKGVNTEELMSKLSAVPGVSSVHEVHIWELISGKIIATLHIKYRKDRGYQDA
SMKIREIFHNAGIHNVTIQFEHVDLKETMEQKDLLLLCSSPCISKGCEKQLCCPPGALPL
AHVNGCAEHNGCPPLDTYPSDGHRGQVTREVAIEVSLDSCLSDHGQALSKTQEDQHYVNS
THF

>Monodelphis domestica_SLC30A10(ENSMODP_5329)
MGRYSGKTCRLIFMLVLTAGFFVAELVSGYLGNSIALISDSFNMLSDLISLCVGISAGFI
ARRHTRGARATYGYSRAEVVGALSNAVFLTALCFTIFVEAILRLVRPERIDDPKLVLIVG
ALGLAVNVVGLLIFQDCSWFTCRGRRRRRRLDDSKREPSAPLDAVRTQQLQPSGSPAVVP
GSDSALTLRVASSPPKEKAAALQPSEAGDSLNIQNEPEENIKKEKKSEALNIRGVLLHVM
GDALGSVVVVIAAIIFYVLPLDVNAPCNWQCYIDPSLTIIMVIIILSSAFPLIKETASIL
LQMVPKGINLEELVSKLSNVAGVNSIHEVHVWELVSGKNIATLHIKCQKGKDYRDANFKM
REIFHSVGIHNVTIQPEYVDQKESLEQEWMLFCNSPCISKACVKQLCCPPETQPLTHVNG
CTEPNGFPPTATYRSDGLSINETAAIVIEADGLSDSGQAAKKVQGDLVFVNSTHF

>Xenopus tropicalis_SLC30A10(ENSXETP_5883)
PACPPAMGRYTGKTCRLIFMLVLTVIFFVAELVSGYLGNSIALISDSFNMLSDLISLCVG
ITASQISRRKSRGPRATYGYPRAEVVGALCNAIFLTALCFTILVDSVLRLAQPQRIDNVT
LVLIVGTLGLLVNIVGLLVFQDYGSYRSHSYKGGMWGVPLPGQTRNTQVSVAHCLTVTPS
KAVGRELHLVGNPLLSSSLKVNGEPVMCHGDAMSPPEDEKSEEKKKEEKKAATLNIRGVM
LHVVGDALGSVVVVVTAVIFYVLPLDANAPCNWQCYIDPSLTVVMVAIILYSAFPLIKET
AYILLQMVPQGVQVGEIGQKLALVPGVNSVHEIHIWELASGKNIATLHVKFQDFASHATA
SQEIRRIFHEEEIHAVTIQAEFPNEKDLTLACSAPCISEKCDPYLCC

>Danio rerio_SLC30A10(ENSDARP_132768)
MGRYSGKTCRLILMLVITVIFFVAEIVAGYMGNSVALVSDSFNMLSDILSLCVGLTAARV
SRRAGSGRFSFGLGRAEVVGALANAVFLIALCFSISMESLKRLAMPQAIDDAPLVLIVGS
LGLAVNLVGLVIFQDCGRLCGRRGKEKKREEHREDREQELEQVETGLQEEKTEKDGAPLN
IRGVLLHVLNDALGSVVVVVASALFYVWPLEPDQPCNWQCYVDPSLTLVMVIIILSSAAP
LLKETTTILLQMSPEDLPVSAILESVCRLPGVSSVHEAHVWELAKGRNVASLHVKLQAPA
GALWAQVSLQQQITQIFHRAGVHSLTLQLELADSDADSSCSAPCLSSSCQKLSCCPAGPV
CVCAPPRDSSTEKHTPQACTHSSKL

>Ciona intestinalis_(ENSCINP_14617)
LSSMLGLIIVYFLAEAVVGHLTSSLTLIADSFHMLSDALSLVVALVAVRLSKREQAYFNT
FGWVRFEVVGALINSTFLFALCISITMEAIEKFYDPGLISQPELVLAVGGCGLLINVIGL
VLFGGHAHAGHNHSHGHDHGHNHGHDNHHHNHLQVSSMHVFLLGNLFFRPTIFIVNNNGST
EQHMNMKAVFLHVLGDALGSVIVMISATIIYLVPEYIYCNKVCQSDPNLVLIELKNATAV
NVVINVNEWIMYIDPAMSIVLVLIMIFTTYPLFKESSLVLLQTVPKHIKLQHLKEKIKTI
EGVQEIQNFHLWQLTGEKLVATVHVWCNDAISFLRIAEEIKQRLNDAGIHSTTIQPEFYR
SPKPYC

>Ciona intestinali_(ENSCINP_3497)
SSAPSEVESDHCHDSSDEKTQRENKKRKKAMRKLLIATGLSFLFMVGEIVGGFLANSLAI
MTDASHLLTDVSSFLISIIALKMAARPISKKMTFGWHRAEVIGALISVLAIWIVTGVLVY
LAVMRLIHNNYEVNGKTMLITASLAVGFNILMGLVLHEHSHTHGHSHGHSHAGHSHDHND
HDHEESGNVNVRAAFIHVIGDLLQSLGVLTAAFVIYFKPEYKIADPICTFLFSIFVLITT
ITILRDTIIVIMEGSPVGVEYSVLKEKLLEIEGVTAVHDLRIWSLTLNQTVMSAHLAIDK
GTDPNLVLKRATRRLKSLYDFHSLTIQVEYYSNDMDECKKCQPA

>Ciona savignyi_(ENSCSAVP_8066)
MDLTNSEERLKEQDSLMDNEHEHGGICKKLLRTKTSRLASMLSLIVIYFFAEVIVGHLTS
SLTLIADSFHMLSDALSLIVALFAVRMSKRDAQQSLTPWPSKQAYFNTFGWVRFEVVGAL
INATFLLALCISILMEAIEKFYDPSLISQPELVLAVGGGGLLINLIGLVLFGGHAHAGHD
HDHGHGHGHSHGHGHGHSHNHNHDHSDHKHNNNITNEEHMNMKAVFLHVLGDALGSVIVM
ISASIVYFVPYEETSTASTTNATNASSVIVTTHVNSWIMYLDPAMSIVLVVIMITTTYPL
FKQSSLVLLQTVPKHIQLQSVKDKIKTIDGVQEIHDFHIWQLTGEKLVATVHVQCSDSQV
YLRIAKEIKQKLHDEGIHSSTVQPEFHRSPTAQCDMACETDACKTKRCCSVSDVPGLAKN
NSKTVVNSATLAEIDETDPIRPSVQPNSSDA

>Ciona savignyi_(ENSCSAVP_10119)
HCHDSSDEKTQRENKKRQKAMRKLLIATGLSFLFMVGEIVEVIGALVSVVAIWIVTGVLV
YLAVMRLINNKYEVHGKTMLITASLAVGFNILMGLVLHEHSHTHGDASDTGNVNVRAAFI
HVIGDLLQSLGVLTAAFVIYFKPEYKIADPICTFLFSVFVLITTITILRDTIIVIMEGSP
VGVEYTVVKEKLLGIEGVAAVHDLRIWSLTLNQTVMSAHLAIDKSTDPNIVLKRATRRLK
SLYDFHSLTIQVEYYSNDMDECKKCQPA

>Caenorhabditis elegans_(C15B12)
MEVTMEDRSVKADKADRDDNNTTSTELLGKMRQTKVIDNCSHGTHTLEHDLRLKGEPIGK
SESVKGVSRSLIIQIGMTVIFCALEFITGVVCSSIAMLADSYHMAADVMALIVAFTCIKI
ATRPSTRLGYGWVRAETLGGFFNGIFMCTVCVLVFQEAVGRIINVHMITHPLQVLVIGFI
GLLINLFGMFNLSGHGHSHGGGSHGHSHGGSHGHSHNNKKTKKNDGHGHSHANGHGHSHD
GKSDCNGEEEPDHTRLNGKFRSASAMANSDANVRLLDNDDNSNDIIERRLSGVNSQNTII
ATVDRQMTPYGTHMASEVLNVSSNNLDKSAQKTEQKKDKNVNIHGVWLHLLSDAFGSVIV
MISAGFVYFLPTWKIAAYLDPILSISLASIMGFTAVVLVKTSGEKLLKQTPEGLDLEKVK
KDLCSIVGVSKVEKLSVWTLCGQRIIAAAHVNICHPAVFPEAAYKIKNYFHDLGVHSTTI

EPTFEDTCMQSMRIMVKKVVDGKSIEEPVSVSTENEITE

>Capitella teleta_(ELT87571)
MHCHTDKQEDRLDRRARNKLIIASMLCVLFMIAEAVGGVLANSLA
VATDAAHLLTDFASFMISLFSLYVA
SRPSTKKMSFGYYRAEVIGALVSVLLIWVVTAVLVYLAVDRVISGDYEIGGATMLITAGCAVAFNIFMGL
TLHDTHGHSHGGGSHGHSHGENHTQHQPNINVRAAFIHVIGDFLQSLGVMIAAIVVYFKPEYKIVDPICT
FLFSILVLFTTITILRDTLNVLMEGTPKGIDFQDVRQALCTIPGVRELHNLRIWSLTVSKVALSVHLAVE
PSTSHQQTLAIAARLMRERFGVHECTVQIEDYVDEMDDCTQCQEPKD

Syntrophin, Gamma-SNT

>Homo sapiens_SNTA1(ENSP_217381)
MASGRRAPRTGLLELRAGAGSGAGGERWQRVLLSLAEDVLTVSPADGDPGPEPGAPREQE
PAQLNGAAEPGAGPPQLPEALLLQRRRVTVRKADAGGLGISIKGGRENKMPILISKIFKG
LAADQTEALFVGDAILSVNGEDLSSATHDEAVQVLKKTGKEVVLEVKYMKDVSPYFKNST
GGTSVGWDSPPASPLQRQPSSPGPTPRNFSEAKHMSLKMAYVSKRCTPNDPEPRYLEICS
ADGQDTLFLRAKDEASARSWATAIQAQVNTLTPRVKDELQALLAATSTAGSQDIKQIGWL
TEQLPSGGTAPTLALLTEKELLLYLSLPETREALSRPARTAPLIATRLVHSGPSKGSVPY
DAELSFALRTGTRHGVDTHLFSVESPQELAAWTRQLVDGCHRAAEGVQEVSTACTWNGRP
CSLSVHIDKGFTLWAAEPGAARAVLLRQPFEKLQMSSDDGASLLFLDFGGAEGEIQLDLH
SCPKTIVFIIHSFLSAKVTRLGLLA

>Pan troglodytes_SNTA1(ENSPTRP_22970)
MASGRRAPRTGLLELRAGAGSGAGGERWQRVLLSLAEDVLTVSPADGDPGPEPGAPREQE
PAQLNGAAEPGAGPPQLPEALLLQRRRVTVRKADAGGLGISIKGGRENKMPILISKIFKG
LAADQTEALFVGDAILSVNGEDLSSATHDEAVQVLKKTGKEVVLEVKYMKDVSPYFKNST
GGTSVGWDSPPASPLQRQPSSPGPTPRNLSEAKHVSLKMAYVSKRCTPNDPEPRYLEICS
ADGQDTLFLRAKDEASARSWATAIQAQVNALTPRVKDELQALLAATSTAGSQDIKQIGWL
TEQLPSGGTAPTLALLTEKELLLYLSLPETREALSRPARTAPLIATRLVHSGPSKGSVPY
DAELSFALRTGTRHGVDTHLFSVESPQELAAWTRQLVDGCHRAAEGVQEVSTACTWNGRP
CSLSVHIDKGFTLWAAEPGAARAVLLRQPFEKLQMSSDDGASLLFLDFGGAEGEIQLDLH
SCPKTIVFIIHSFLSAKVTRLGLLA

>Pongo abelii_SNTA1(ENSPPYP_12200)
MASSRRAPRTGLLELRAGAGSGAGGERWQRVLLSLAEDVLTVSPADGDPGPEPGAPREPE
PAQLNGAAEPGAGPPQLPEALLLQRRRVTVRKADAGGLGISIKGGRENKMPILISKIFKG
LAADQTEALFVGDAILSVNGEDLSSATHDEAVQALKKTGKEVVLEVKYMKDVSPYFKNSA
GGTSVGWDSPPASPLQRQPSSPGPTPRNLSEAKHVSLKMAYVSKRCTPTDPEPRYLEICS
ADGQDTLFVRAKDEASARSWATAIQAQVNALMPRVKDEVQALLAATSTAGSQDIKQIGWL
TEQLPSGGTAPTLALLTEKELLLYSSLPETREALSRPARTAPLIATRLVHSGPSKGSVPY
DAELSFALRTGTRHGVETHLFSVESPQELAAWTRQLVDGCHRAAEGVQEVSTACTWNGRP
CSLSVHIDKGFTLWAAEPGAARAVLLRQPFEKLQMSSDDGASLLFLDFGGAEGEIQLDLH
SCPKTIVFIIHSFLSAKVTRLGLLA

>Mus musculus_SNTA1(ENSMUSP_105350)
MASGRRAPRTGLLELRCGAGSGAGGERWQRVLLSLAEDALTVSPADGEPGPEPEPAQLNG
AAEPGAAPPQLPEALLLQRRRVTVRKADAGGLGISIKGGRENKMPILISKIFKGLAADQT
EALFVGDAILSVNGEDLSSATHDEAVQALKKTGKEVVLEVKYMKEVSPYFKNSAGGTSVG
WDSPPASPLQRQPSSPGPQPRNLSEAKHVSLKMAYVSRRCTPTDPEPRYLEICAADGQDA
VFLRAKDEASARSWAGAIQAQIGTFIPWVKDELQALLTATGTAGSQDIKQIGWLTEQLPS
GGTAPTLALLTEKELLFYCSLPQSREALSRPTRTAPLIATRLVHSGPSKGSVPYDAELSF
ALRTGTRHGVDTHLFSVESPQELAAWTRQLVDGCHRAAEGIQEVSTACTWNGRPCSLSVH
IDKGFTLWAAEPGAARAMLLRQPFEKLQMSSDDGTSLLFLDFGGAEGEIQLDLHSCPKTM
VFIIHSFLSAKVTRLGLLA

>Rattus norvegicus_SNTA1(ENSRNOP_21715)
MASGRRAPRTGLLELRSGAGSGAGGERWQRVLLTLAEDALTVSPADGEPGPEPEPAQLNG
AAEPGAASPQLPEALLLQRRRVTVRKADAGGLGISIKGGRENKMPILISKIFKGLAADQT
EALFVGDAILSVNGEDLSSATHDEAVQALKKTGKEVVLEVKYMKEVSPYFKNSAGGTSVG

WDSPPASPLQRQPSSPGPQPRNLNEAKHVSLKMAYVSRRCTPTDPEPRYLEICAADGQDT
LFLRAKDEASARSWAGAIQAQISTFIPWVKDELQALLTATGTAGSQDIKQIGWLTEQLPS
GGTAPTLALLTEKELLLYCSLPQTREALSRPARTAPLIATRLVHSGPSKGSVPYDAELSF
ALRTGTRHGVDTHLFSVESPQELAAWTRQLVDGCHRAAEGVQEVSTACTWNGRPCNLSVH
IDKGFTLWAAEPGATRAVLLRQPFEKLQMSSDDGMSLLFLDFGGAEGEIQLDLHSCPKTM
VFIIHSFLSAKVTRLGLLA

>Canis familiaris_SNTA1(ENSCAFP_11007)
MPILISKIFKGLAADQTEALFVGDAILSVNGEDLSSATHDEAVQVLKKTGKEVVLEVKYM
KEVSPYFKNSAGGTSVGWDSPPASPLPRQPSSPGPSPRDLSDAKHMSLKMAYVSRRCTST
DPEHRYLEICSADGQDTLFLRAKDEASAKSWAAAIQAQVNALMPWVKDELQALLAATSTT
GSQDIKQIGWLTEQLPNGGTAPTLALLTEKELLLYCHLPQTREALSQPSRTAPLIATRLV
HSGPSKGSVPYDAELSFALRTGTRHGVDTHLFSVESPQELATWTRQLVDGCHRAAEGVQE
VSTACTWNGRPCSLSVHIDKGFTLWAAEPGAARAVLLRQPFEKLQMSSDDGASLLFLDFG
GAEGEIQLDLHSCPKTMVFIIHSFLSAKVTRLGLLA

>Felis catus_SNTA1(ENSFCAP_21236)
GGRENKMPILISKIFKGLAADQTEALFVGDAILSVNGEDLSSATHDEAVQVLKKTGKEVV
LEVKYMKEVSPYFKNSAGGTSVGWDSPPASPLQRQLSSPGPPPRDISDAKHISLKMAYVS
RRCTPTDPEPRYLEICSADGQDTLFLRAKDEASAKSWAAAIQAQVNALMPWVKDELQALL
AATSTAGSQDIKQIGWLTEQLPGGGTAPTLALLTEKELLLYCHLPQTREALSQPARTAPL
IATRLVHSGPSKGSVPYDAELSFALRTGTRHGVDTHLFSVESPQELAAWTRQLVDGCHRA
AEGVQEVSTACTWNGRPCTLSVHIDKGFTLWAAEPGAARAVLLRQPFEKLQMSSDDGASL
LFLDFGGAEGEIQLDLHSCPKTMVFIIHSFLSAKVTRLGLLA

>Monodelphis domestica_SNTA1(ENSMODP_3704)
MAAGRRSPKSGLLELRAPGSAGPGPWLRVLVSLAADVVTVSAAPASGEPEPGPGPVSELV
SASLPQLNGADPGAPAAPGAPPTPPLPHALLNQKRTVRVVKQEAGGLGISIKGGQENKMP
ILISKIFKGLAADQTEALFVGDAILSVNGGDLSTATHDEAVQVLKKTGKEVVLEVKYMKE
ISPYFKNSSSGATVGWESPTASSPTLCPRDLKEGKNVSLKMAYVSRRCIPADAERRYLEL
RSADGRDILFLRAKDEASAQAWLAALQANIGVLVPWVTDELRALLAAAGGVSGSTEIKRI
GWLTEQLSGGGTEPVLAVLTEKDLLFYSSLPQNRDALSAPARSSPLIATRLVHSGPSKGS
VLYDSELSFALRSGTHQGVETHLFSVESPRDLSSWTRLLVDGCHNAAETVQEVSTACTWN
GRSCSLSVHIDKGFTLWAVEPGLAKTLLLQEPFEKLQMSSDDGSSLLFLDFGGTEGEIQL
DLHSCPKTIVFIIHSFLSAKVTRLGLA

>Anolis carolinensis_SNTA1(ENSACAP_8363)
MLWNQGSCGMVKVREIKVRESTARSLSRSKRCQPALVPPCGGAGREAPGALLGGRRGGGG
GGAGAWVRPPKAAESERARKGRAKEGRQEGRNALKMAAGRRAPKSGLLELRAPGEQWLRV
LLALSEDFLSVSPGGGGGGGGGKGPEETPPSSPGQLNGGDPPPALVPDALTNIKRTVRIV
KQDVGGLGISIKGGRENKMPILISKIFKGLAADQTEALFVGDAILSVNGADLSDATHDEA
VQALKKTGKEVVLEVKYMKEISPYFKNSSTGATVSWDSSPAAFQKQASPSLSLRDLNEGK
NIPLRMCYISRKCLPSDPENRYLEVCSADGRIPLFLRAKDEASAQAWFNAIHSNISALLP
RVREELRALQSGAGIAGSRDIKHVGWLTEQLPGDGTKNILAILTEKELLFYSCLPQSRDA
LGKPTHSFPLIATRLVHSGPSKGSLLYDSELSFALRTGTKKGVETHLFSVETHRDLAMWT
RMLVDGCHNAAECVQEVSTACTWNGRDCTLSIHIDKGFTISALEPGLAKVILLQQPFERL
QMSSDDGAKMLYLDFGSSEGEIQLDLHSCPKTIVFIIHSFLSAKVTRMGLLA

>Xenopus tropicalis_SNTA1(ENSXETP_46345)
MASPGTAHRSGLLELRCPGERWVRVLLLLGEETVTFTPEPEPGAAEEKALNGAEPAGGVP
ESLINLRRTVRVLKQDAGGLGVSIKGGRENKMPILISKIFKGLAADLTGSLYVGDAILSV
NGVDLSEATHDDAVQALKKTGKEVVLEVKYMKEISPYFKGSPPSGSPAAPLQKRGSPALP
PKEYKDGKSIPLKMCYVSRRFLPTDTEPRYLEICSADGRDVLFFRAKDEGTAQSWFNAIH
TNASALAPRVREDVKQLLGKDIKQMGWLTEQLPQDGKRNLLALLTEKDLLLYNSLPHSRE
GFSKPYCHPLIATRLVHSGPSKSSPLYDTELAFALRSGTQKGVETHLFSVETQRELATWT
RVLVDGCHSAAELIKEVTTACTWGGNECALSIHIDQGFTLFTEEPGLNKVIHFHQPFEKL
RMSSDDGVRILYLEFGNPEAELQLDLHSCPKTIVFIIHSFLSAKVSRLGLFT

>Takifugu rubripes_SNTA1(ENSTRUP_31180)
MAAAMKAQKTGLLELRVTVDRWRRVLATLTEDTLTVNPSEASEEPAKAAPTPVGALNGDP
PNLSSSPVPETITNVKRTVRVTKQDVGGLGISIKGGKENKMPILISKIFKGLAADQTEAL

YVGDAILSVNSYDLREATHDEAVQALKKTGKEVVLEVKYIKEMSAFFKSSGSPGGALPWD
SPPSTPQRTSEPLPAEVKEPRSIPLKMCQVSRKQCPPDTENRYFEVISATRKNSVFLRAK
DPAMAQSWYNAIQNSIANLLPRMKDEMKVMQPGMEVKHVGWITEQVIQGPEKPVLAVLTD
RDLLLYPSLPESKEGLKSPTKSHPLITTRLVHSGPGKSSPLLDSDLSFGLRTGTKQGVET
HVFRVDSAKDLSTWTHLLVEGCHNAAELIKEVTTACSWNGKECTLGVHIDEGFTLFTEEM
GVRKRILLQHPFEHLKMSSDDGVRMMFLDFGGPEAEIQLDLHCCPKTLVFIIHSFLSAKV
KRLGLLA

>Tetraodon nigroviridis_SNTA1(ENSTNIP_9832)
MAAAMKAQKTGLLELRVTVDRWMRVLATLTEDTLTVNPGEATEEPAKAAPTPAGAINGDP
PNLSSSPVPETITNVKRTVRVTKQDVGGLGISIKGGKENKMPILISKIFKGLAADQTEAL
YVGDAILSVNSYDLREATHDEAVQALKKTGKEVILEVKYIKEMSAFFKSSGSPGGALPWD
SPPSSPQRTSEPLPAEVKEPRSIPLKMCQVSRKQCPPDTENRYFEVVSSTRKNSMFLRAK
DPAMAQSWYNAIQAGIANLLPKVKDEMKVMQPGMEVKHLGWITEQVTQGPERPVLAVLTD
RDLLLYPSLPDSKESLNSPTRSHPLITTQLVHSGPGKSSPLLDSDLSFGLRSGTKQGVET
HVFRVDSAKDLSAWTHLLVEGCHNAAELIKEVTTACSWNGKECTLGVHIDEGFTLFTEEM
GVRKRVLLQHPFERLKMSSDDGVRMMFLDFGGPEAEIQLDLHCCPKTLVFIIHSFLSAKV
KRLGLLA

>Gasterosteus aculeatus_SNTA1(ENSGACP_8047)
GGKENKMPILISKIFKGLAADQTEALYVGDAILSVNGYDLREATHDEAVQALKKTGKEVI
LEVKYIKEMSAFFKNSGSPGATLPWDSPPSTPHTGTELSPAEVKEPRSIPLKMCQVSRKQ
CPPDTENRYFEVISSSRRNSVFLRAKDPAVAQSWYNAVQAGAASLLPRVKEEMRSMQQGV
EVKHLGWITEQVCKNADVHEKPVLAMLTDRDLLLYPSLPESKESLGNPAKSHPLIATRLV
HSGPGKSSPLLDSDLSFGLRSGTKQGVETHVFRVDSAKELSTWTHLVVEGCHNAAELVKE
VTAACSWNGKECTLGVHIDEGFTLFTQEMGVRKSILLQQPFERLRMSSDDGVRMMFLDFG
GQEAEIQLDLHSCPKTIVFIIHSFLSAKVKRLGLLA

>Oryzias latipes_SNTA1(ENSORLP_7508)
MAAAMKTQKTGLLELRVTVDRWVRVLATLTEDALTLNPGDSADEPAKPGHQSPAGAVNGD
PPNLSSSPVPETITNVKRTVRVTKQDVGGLGISIKGGKENKMPILISKIFKGLAADQTEA
LYVGDAILSVNGYDLREATHDEAVQALKKTGKEVILEVKYIKEMSAFFKSSGSPGASLPW
DSPPSTPQRDPELSPAEGKEPRSVPLKMCQVSRKQCPPDTEDRYFEVTSFNRKNSVFLRA
KDPAMAQSWYNAIQAAAGSLLPQVKEELKIMQPGVQIKHVGWMTEQKKYLKIDMRQGPEK
AVLAVLTEKDLLLFPSLPENKQSLSSPTKSHPLITTRLVHSGPGKSCLHADSELSFGLRS
GTRQGVETHVFRVDSAKELSAWTHLLVEGCHNAAELIKEVTTGTEPSRKRAVQFLRDSTP
EVFSADPIGLLFFLYKDFYSILAFETLDFFLCIYPNQGFSSPQQLDLHCCPKTLVFIIHS
FLSAKVRRLGLLA

>Homo sapiens_SNTB1(ENSP_431124)
MAVAAAAAAAGPAGAGGGRAQRSGLLEVLVRDRWHKVLVNLSEDALVLSSEEGAAAYNGI
GTATNGSFCRGAGAGHPGAGGAQPPDSPAGVRTAFTDLPEQVPESISNQKRGVKVLKQEL
GGLGISIKGGKENKMPILISKIFKGLAADQTQALYVGDAILSVNGADLRDATHDEAVQAL
KRAGKEVLLEVKYMREATPYVKKGSPVSEIGWETPPPESPRLGGSTSDPPSSQSFSFHRD
RKSIPLKMCYVTRSMALADPENRQLEIHSPDAKHTVILRSKDSATAQAWFSAIHSNVNDL
LTRVIAEVREQLGKTGIAGSREIRHLGWLAEKVPGESKKQWKPALVVLTEKDLLIYDSMP
RRKEAWFSPVHTYPLLATRLVHSGPGKGSPQAGVDLSFATRTGTRQGIETHLFRAETSRD
LSHWTRSIVQGCHNSAELIAEISTACTYKNQECRLTIHYENGFSITTEPQEGAFPKTIIQ
SPYEKLKMSSDDGIRMLYLDFGGKDGEIQLDLHSCPKPIVFIIHSFLSAKITRLGLVA

>Pan troglodytes_SNTB1(ENSPTRP_35135)
MAVAAAAAAAGPAGAGGGRAQRSGLLEVLVRDRWHKVLVNLSEDALVLSSEEGAAAYNGI
GTATNGSFCRGAGAGHPGAGGAQPPDSPAGVRTAFTDLPEQVPESISNQKRGVKVLKQEL
GGLGISIKGGKENKMPILISKIFKGLAADQTQALYVGDAILSVNGADLRDATHDEAVQAL
KRAGKEVLLEVKYMREATPYVKKGSPVSEIGWETPPPESPRLGGSTSDPPSSQSFSFHRD
RKSIPLKMCYVTRSMALADPENRQLEIHSPDAKHTVILRSKDSATAQAWFSAIHSNVNDL
LTRVIAEVREQLGKTGIAGSREIRHLGWLAEKVPGESEKQWKPALVVLTEKDLLIYDSMP
RRKEAWFSPVHTYPLLATRLVHSGPGKGSPQAGVDLSFATRTGTRQGIETHLFRAETSRD
LSHWTRSIVQGCHNSAELIAEISTACTYKNQECRLTIHYENGFSITTEPQEGAFPKTIIQ
SPYEKLKMSSDDGIRMLYLDFGGKDGEIQLDLHSCPKPIVFIIHSFLSAKITRLGLVA

>Mus musculus_SNTB1(ENSMUSP_41294)
MAVAAAAVAAPAGGGGARAQRSGLLEVLVRDRWHKVLVNLSEDALVLSCEEGAAAYNGIG
AATNGSFCRGSGTGHPVPGVAQAPDSPAGVRTAFTDLPEQVPESISNQKRGVKVLKQELG
GLGISIKGGKENKMPILISKIFKGLAADQTQALYVGDAILSVNGADLRDATHDEAVQALK
RAGKEVLLEVKYMREATPYVKKGSPVSEIGWETPPPESPRLGGGSAEPLSSQSFSFHRDR
KSIPLKMCYVTRNMTLADPENRQLEIHSPDAKHTVILRSKDSATAQAWFSAIHSNAGDLL
TRVVADIREQLGKTGIAGSREIRHLGWLAEKVPGESEKQWKPALVVLTEKDLLIYDSMPR
RKEAWFSPVHSYPLLATRLVHSGPGKGSPQAGMDLSFATRTGTKQGIETHLFRAEISRDL
SHWTRSIVQGCHNSAELTAEITTACTYRNQECRLTIHYDNGFSISTEPQDGAFPKTIIQS
PYEKLKMSSDDGIRMLYLDFGGKEGEIQLDLHSCPKPIVFIIHSFLSAKITRLGLVA

>Felis catus_SNTB1(ENSFCAP_23633)
VNLSEDALVLSCEEGAAAYNGIGTATNGSFCRGAGGAQPPDSPAGVRTAFTDLPEQVPES
ISNQKRGVKVLKQELGGLGISIKGGKENKMPILISKIFKGLAADQTQALYVGDAILSVNG
ADLRDATHDEAVQALKRAGKEVLLEVKYMREATPYVKKGSPVSEIGWETPPPESPRLGGG
SSDPLSSQPFSFHRDRKSIPLKMCYVTRSMASADPENRQLEIHSPDAKHTVILRSKDSAT
AQAWFSAIHSNVSDLLTQVIAEVREQLGKTGIAGSREIRHLGWLADKVPGESEKQWKPAL
VVLTEKDLLIYDSMPRRKEAWFSPVHTYPLLATRLVHSGPGKGSPQAGVDLSFATRTGTR
QGIETHVFRAETGRDLSHWTRSIVQGCHNSAELVTEVTTACTYKNQACCLTIHYENGFSI
TTEPQEGAFPKTILQSPYEKLKMSSDDGIRMLYLDFGGKDGEIQLDLHSCPKPIVFIIHS
FLSAKITRLGLVA

>Erinaceus europaeus_SNTB1(ENSEEUP_6243)
MAVAAAAAVAAAPAGGGGGRAQRSGLLEVLVRDRWHKVLVNLGEDALVLSCEEGAAAYNG
FGAATNGSFCRAGASGPSGTQSPDSPAGVRTAFTDLPEQVPEAISNQKRGVKVLKQEMGG
LGISIKGGKENKMPILISKIFKGLAADQTQALYVGDAILSVNGADLRDATHDEAVQALKR
AGKEVLLEVKYMREATPYVKKGSPVSEIGWETPPPESPKLGGSSSDPLSSQSFSFHRDRK
NIPLKMCYVTRSMALADPENRQLEIHSPDAKHTVILRSKDSATAQAWFSAIHSNVSDLLT
RVITNVRDQLGKTGIAGSREIRHLGWLAEKVPGESEKQWKPALVVLTEKDLLIYDSMPRR
KEAWFSPVHTYPLLATRLVHSGPGKGSPQTGVDLSFATRTGTRQGIETHLFRAEISRDLS
HWTRSIVQGCHNSAELITEITTACTYKNQECRLTIHYENGFSITTEPQEGAFSKTIIQSP
YEKLKMSSDDGIRMLYLDFGGKDGEIQLDLHTCPKPIVFTIHSFLSAKITRLGLVA

>Gallus gallus_SNTB1(ENSGALP_26399)
MAVAAAGGGGGGGRAQRSGWLEVLVRERWHKVLANLGGEALVLSGEERPDGAAHNGLGGD
GAACRGAEGGGGGSAVRTAFTDPPEQVPEAVSNKKRCVKVLKQELGGLGISIKGGKENKM
PILISKIFKGLAADQTQALYVGDAILAVNGTDLRDATHDEAVQALKRAGKEVLLEVKYMR
EATPYVKKGSPVSEIGWETPPPESPRLGCVSADPLSQLSLSIHRDKKTIPLKMCYVTRNM
TVSDPENRLIEVHSPDAKHTVVLRSKDSATAQAWFNAIHSSVNDLIPRVIAEVRDQLGKA
GIAGSREIRHLGWLAEKVPGDNEKHWKPVLVVLTEKDLLIYESMPRMKEAWFSPLHTYPL
LATRLVHSGPGKGSPQSGVDLSFATRTGTRQGIETHLFRTETSRDLSLWTRSIVQGCHNS
AELITEITTSCTYKSQECRLTIHYEHGFSLTTEPQDGAFSKTIAQYPYEKLKMSSDDGIR
MLYLDFGGKDGEIQLDLHSCPKPIVFIIHSFLSAKITRLGLVA

>Pelodiscus sinensis_SNTB1(ENSPSIP_8557)
MPILISKIFKGLAADQTQALYVGDAILAVNGTDLRDATHDEAVQALKRAGKEVLLEVKYM
REATPYVKKGSPVSEIGWETPPPESPRLGGVSSDPLSQLSLSIHRDRKTIPLKMCYVTRN
MAVSDPENRLLEVHSPDAKHTVVLRSKDSATAQAWFNAIHSSVSDLIPRVIAEVKDQLGK
TGIAGSREIRHLGWLAEKVPGENEKHWKPALVVLTEKDLLMYDSMPRMKEAWFSPLHTYP
LLATRLVHSGPTKGSPQSGVDLSFATRTGTRQGIETHLFRTETSRDLSLWTRNIVQGCHN
SAELITEIKTSCTYKNQECRLTIHYEHGFSLTVEPRDGALSKTIVQCPYEKLKMSSDDGI
RMLYLDFGGKDGEFQLDLHSCPKPIVFIIHSFLSAKITRLGLVA

>Takifugu rubripes_SNTB1(ENSTRUP_25844)
APKMAVVIAKGVSGACGVRKSGVLEVLVRERWHKVSVNLNEEALTLSCEEDSGNDADDSV
NFNCNGSYLDNNNANSNNGHQHVRPACTERVPEAIANRKRCVKVAKQEIGGLGISIKGGK
ENKMPILISKIFKGLAADQTQALYVGDAILSVNEMNLRDATHDEAVQALKRAGREVTLEV
KYMREATPYVKRGSPVSEIGWETLPPESPRLGTPPITSSPISNPPSPPTQPFLSPQGDRR
CIPLKMCYVTRAMTTADPENRQLELHSPDTRHTVVLRCPDQPSALSWFAAMHSVTSSLAQ
WALAEVVQNTARMGIAGSKEIRHLGWLAGKVPQTESEKQSWKPVLVVVTEKDLLLYDSLP
RSKEAWHCPAHIYPLLATRLVHSGPDRGSPHSGTELFFATRTGTRLGIEAHLFRVETTKD

LSLWTRHIVNGCHASAEMIKEVTTSCLYRGQECRLVIHYEQGFSVLADPTPLGDRENGEG
RKAAAAAPPQVLLSYPYEKLKMSSDDGIRMLLLDFGGKEGEIQLDLHSCPKPIVFILHSF
LSAKISRLGLVA

>Tetraodon nigroviridis_SNTB1(ENSTNIP_12507)
MAVVIAKGVSGACGVRKSGVVEVLVRGRWHKVSVHLNEEALTLSCEGERVGNADDGVSLS
CNGSYLDNNNANSNNGQQHVRPACTERVPEAIANRKRCVKVAKQEIGGLGISIKGGKENK
MPILISKIFKGLAADQTQALYVGDAILSVNGMNLRDATHDEAVQTLKRAGKEVTLEVKYM
REATPYVKKGSPVSEIGWETLPPESPRLGTPPISSPTHLSTPPSPSAQPFLSPQGDRRCI
PLKMCYVTRAMTTADPENRQLELHSPDTRHTVVLRCPDQPSALSWFAALHSVTSSLAQRA
LAEVVQNTARMGIAGSKEIRHLGWLAGKTESEKQSWKPVLVVVTEKDLLLYDSLPRSKEA
WHSPAHIYPLLATRLVHSGPDRGSPHSGTELFFATRTGTRLGIEAHLFRVETTKDLSSWT
RHIVNGCHASAEMIKEVTTSCLYRGQECRLVIHYEQGFSVLADPTPLGGRENGEEREAAN
AAKPQVLLSYPYEKLKMSSDDGIRMLFLDFGGREGEIQLDLHSCPKPIVFILHSFLSAKI
SRLGLVA

>Gasterosteus aculeatus_SNTB1(ENSGACP_9186)
APKMAVVITTGVPGVSGVQKSGFLDVLVRERWHKVLVNLNEEALTLSCEEEDCGPDRDSV
TPNGVTNGSYLDNNNANSNNGPHAARTAFTDLPERVPEAIANRKRCVKVTKQEIGGLGVS
IKGGKENKMPILISKIFKGLAADQTQALYVGDAILSVNGVNLRDATHDEAVQALKRAGKE
VTLEVKYMREATPYVKKGSPVSEIGWETPPPESPRLGSPTVLSSPTSFSFSGDRRCIPLK
MCCVTRALTTPDPENRQLELHSPDARHTVVLRCPDQPSALSWFSAMHSVTSKLAKRALSE
VIHSTARTDVAGSREIRHLGWLAGKVGTESEKQCWKPVLVVVTEKDLLLYESLPRGKEAW
QSPAHQYPLLATRLVHSGSERGSPHSGTELLFATRTGTRLGIETHLFRAETAKDLSLWTR
QLVNGCHASAEMIKEVTTSCLYQSQECRLVIHYEQGFSVLADPKLGDGPQVLLSYPYEKL
KMSSDDGVRMLFLDFGGEEGEIQLDLHSCPKPIVFILHSFLSAKIYRLGLVA

>Homo sapiens_SNTB2(ENSP_338191)
MRVAAATAAAGAGPAMAVWTRATKAGLVELLLRERWVRVVAELSGESLSLTGDAAAAELE
PALGPAAAAFNGLPNGGGAGDSLPGSPSRGLGPPSPPAPPRGPAGEAGASPPVRRVRVVK
QEAGGLGISIKGGRENRMPILISKIFPGLAADQSRALRLGDAILSVNGTDLRQATHDQAV
QALKRAGKEVLLEVKFIREVTPYIKKPSLVSDLPWEGAAPQSPSFSGSEDSGSPKHQNST
KDRKIIPLKMCFAARNLSMPDLENRLIELHSPDSRNTLILRCKDTATAHSWFVAIHTNIM
ALLPQVLAELNAMLGATSTAGGSKEVKHIAWLAEQAKLDGGRQQWRPVLMAVTEKDLLLY
DCMPWTRDAWASPCHSYPLVATRLVHSGSGCRSPSLGSDLTFATRTGSRQGIEMHLFRVE
THRDLSSWTRILVQGCHAAAELIKEVSLGCMLNGQEVRLTIHYENGFTISRENGGSSSIL
YRYPFERLKMSADDGIRNLYLDFGGPEGELTMDLHSCPKPIVFVLHTFLSAKVTRMGLLV

>Pan troglodytes_SNTB2(ENSPTRP_14133)
MRVAAATAAAGAGPAMAVWTRATKAGLVELLLRERWVRVVAELSGESLSLTGDASAAELE
PALGPAAAAFNGLPNGGGAGDSLPGSPSRGLGPPSPPAPPRGPAGEAGASPPVRRVRVVK
QEAGGLGISIKGGRENPMPILISKIFRGLAADQSRALRLGDAILSVNGTDLRQATHDQAV
QALKRAGKEVLLEVKFIREVTPYIKKPSLVSDLPWEGAAPQSPSFSGSEDSGSPKHQNST
KDRKIIPLKMCFAARNLSMPDLENRLIELHSPDSRNTLILRCKDTATAHSWFVAIHTNIM
ALLPQVLAELNAMLGATSTAGGSKEVKHIAWLAEQAKLDGGRQQWRPVLMAVTEKDLLLY
DCMPWTRDAWASPCHSYPLVATRLVHSGSGCRSPSLGSDLTFATRTGSRQGIEMHLFRVE
THRDLSSWTRILVQGCHAAAELIKEVSLGCMLNGQEVRLTIHYENGFTISRENGGSSSIL
YRYPFERLKMSADDGIRNLYLDFGGPEGELTMDLHSCPKPIVFVLHTFLSAKVTRMGLLV

>Pongo abelii_SNTB2(ENSPPYP_8467)
MRVAAAAGAGPAMAVWTRATKAGLVELLLRERWVRVVAELSGESLSLTGDAAAAELEPAL
GPAAASFNGLPNGGGAGDSLPGSPSRGLGPPSPPAPPRGPAGEAGASPPVRRVRVVKQEA
GGLGISIKGGRENRMPILISKIFPGLAADQSRALRLGDAILSVNGTDLRQATHDQAVQAL
KRAGKEVLLEVKFIREVTPYIKKPSLVSDLPWEGAAPQSPSFSGSEDSGSPKHQNSTKDR
KIIPLKMCFAARNLSMPDLENRLIELHSPDSRNTLILRCKDTATAHSWFVAIHTNIMALL
PQVLAELNAMLGATSTAGGSKEVKHIAWLAEQAKLDGGRQQWRPVLMAVTEKDLLLYDCM
PWTRDAWASPCHSYPLVATRLVHSGSGCRSPSLGSDLTFATRTGSRQGIEMHLFRVETHR
DLSSWTRILVQGCHAAAELIKEVSLGCMLNGQEVRLTIHYENGFTISRENGGSSSILYRY
PFERLKMSADDGIRNLYLDFGGPEGELTMDLHSCPKPIVFVLHTFLSAKVTRMGLLV

>Mus musculus_SNTB2(ENSMUSP_148684)

MAVWTRATKAGLVELLLRERWVRVVAELSGESLSLTGDAAAVEPEPPAAAFNGLPNGGGG
ESLPGSPNRGLGPPSPPAPPRGPAGEASASPPVRRVRVVKQEAGGLGISIKGGRENRMPI
LISKIFPGLAADQSRALRLGDAILSVNGTDLRQATHDQAVQALKRAGKEVLLEVKFIREV
TPYIKKPSLVSDLPWEGASPQSPSFSGSEDSGSPKHQNTTKDRKVIPLKMCFAARNLSMP
DLENRLIELHSPDSRNTLILRCKDTATAHSWFVAIHTNIMALLPQVLAELNAMLGATSTA
GGSKEVKHIAWLAEQAKLDGGRQQWRPVLMAVTEKDLLLYDCMPWTRDAWASPCHSYPLV
ATRLVHSGSGCRSPSLGSDLTFATRTGSRQGIEMHLFRVETHRDLSTWTRILVQGCHAAA
ELIKEVSLGCTLSGQEVRFTVHYEHGFTITRDNGGASSILYRYPFERLKMSADDGIRNLY
LDFGGPEGELTMDLHSCPKPIVFVLHTFLSAKVTRMGLLV

>Rattus norvegicus_SNTB2(ENSRNOP_27561)
MAVWTRATKAGLVELLLRERWVRVVAELSGESLSLTGDAAAVEPEPPAVAFNGLPNGGGG
GGDSLPGSPNRGLGPPSPPAPPRGPAGEASASPPVRRVRVVKQEAGGLGISIKGGRENRM
PILISKIFPGLAADQSRALRLGDAILSVNGTDLRQATHDQAVQALKRAGKEVLLEVKFIR
EVTPYIKKPSLVSDLPWEGASPQSPSFSGSEDSGSPKHQNTTKDRKVIPLKMCFAARNLS
MPDLENRLIELHSPDSRNTLILRCKDTATAHSWFVAIHTNIMALLPQVLAELNAMLGATS
TAGGSKEVKHIAWLAEQAKLDGGRQQWRPVLMAVTEKDLLLYDCMPWTRDAWASPCHSYP
LVATRLVHSGSGCRSPSLGSDLTFATRTGSRQGIEMHLFRVETHRDLSTWTRILVQGCHA
AAELIKEVSLGCTLSGQDVRFTVHYEHGFTITRDNGGTSSILYRYPFERLKMSADDGIRN
LYLDFGGPEGELTMDLHSCPKPIVFVLHTFLSAKVTRMGLLV

>Canis familiaris_SNTB2(ENSCAFP_30087)
MNNETPRGERPAPPPRAGRGKRRGCPTGMRVSAAAAAAGAGPAMAVWTRATKAGLVELLL
RERWVRVVAELSGESLSLTGDAAAAEPEAALGPAAAAFNGLPNGGGAGDSLPGSPSRGLG
PPSPPAPPRGPAGEAGASPPVRRVRVVKQEAGGLGISIKGGRENRMPILISKIFPGLAAD
QSRALRLGDAILSVNGTDLRQATHDQAVQALKRAGKEVLLEVKFIREVTPYIKKPSLVSD
LPWEGASPQSPSFSGSEDSGSPKHQNSTKDRKVIPLKMCFAARNLSMPDLENRLIELHSP
DSRNTLILRCKDTATAHSWFVAIYTNIMALLPQVLAELNAMLGATSTAGGSKEVKHIAWL
AEQAKLDGGRQQWRPVLMAVTEKDLLLYDCMPWTRDAWASPCHSYPLVATRLVHSGSGCR
SPSLGSDLTFATRTGSRQGIEMHLFRVETHRDLSTWTRILVQGCHAAAELIKEVSLGCTL
NGQEVRLTVHYENGFTISKENGGSSSILYRYPFERLKMSADDGIRNLYLDFGGPEGELTM
DLHSCPKPIVFVLHTFLSAKVTRMGLLV

>Gallus gallus_SNTB2(ENSGALP_43176)
MGTREAPSAPPLAALTANSVPPCGRGARPRVGARGGAERRGGGGGMAVWTRASKAGLLEL
LLRERWVRVAAELSGEALSLTAEPGEPTPGLNGVANGGGGGEWGGAEAAVPGCVRRVRVV
KAEAGGLGISIKGGRENRMPVLISRIFPGLAAERSGALRLGDAILAVNGVDLRDATHDQA
VQALKRAGREVILEVKFMREVTPYMKKPSLVSDLPWEGAAPQSPSLSGSEDSGSPQHHGA
RDRKVIPLKMCFAARNLSMPDLENRLIELHSPDSRNTLILRCKDTATAHSWFTALHANIM
ALLPQVLAELNAALGGSAGGREVKHVAWLAEQARLDGGRQQWRPVLMAVTEKDLLLYDAM
PWTRDAWASPCHSYPLVATRYRDGDSCGSHPHTWDAAPTSPPLPRLVHSGSGRRSPSLGS
ELTFATRTGSRQGIEMHVFRVETHRDLSCWTRVLVQGCHAAAELIKEVSVGCTLGGQEVK
LCIHYEGGFTICRDEAGGSVLYRYPYEKLKMSADDGIRTLYLDFGGPEGELALDLHSCPK
PIVFVLHTFLSAKVTRMGLLV

>Danio rerio_SNTB2(ENSDARP_67932)
MMAVWTRAEKQGHLDLLLSDRWVRVSAELTRDNLTLTAEGEPGGPAGYPEHNSHLKNGVS
NGNEQNRDVFTNRVQYDNSPGRVNGTGSDSESSGYCDSEGVRRVRIVKQESGGLGISIKG
GRENRMPILISKIFPGLAADQSRALRVGDAILSVNGSDLRDATHDAAVQALKKAGKEVTL
EVKYIREVSPLFKKSSMSADLSWDGRLQSPNLSSSDESPKSSVSRDRKVIPLKMSFISRN
LTMPDPENRLLELHSPDGQHTVVLRCKDGATAHSWFTAIHTNVAALLPQTLSYINTFLNN
ANTHCTLKHIGWLVEQLDMDGSRHQYKRVIMALTEKDILLFQAVPWTYESWTTPVLTHPL
LATRLVHSGSVRSSPALGGDLLFATRTGTGRGIESHVFRVETHWDLSTWTRALVQGGHSA
AELIKEVSIGCVLNRQNVRLILHYERGFTVTRESGDSVLFHFPYERLKMSADDGVRNLFL
DFGAPEGEMVFELHSGPKPVVFVLHSFLSAKLARLGLLT

>Takifugu rubripes_SNTB2(ENSTRUP_6306)
QAVPILEGSSEAVRKVRVVKQESGGLGISIKGGRENRMPILISKIFPGLAADQSRALRVG
DAILSVNGSDLREATHDLAVQALKKAGKEVTLEVKYIREVSPLFKKPSLVADLPWDGVRP
QSPSYSGSEDSGSPKHSSSSSSKDRKVISLKMCFISRNLTMPDLENRLLELHSPDGQHTV
VLRCKDAPTANSWFTAVHTNIAALLPQVLAHINAYLGASSSAPTHPHLKHIGWLAEQIQL

DGGRQQYKPVVMALTEKDILLFEAVPWSRESWSLPLLTHPLLATRLVHSGSARGSPAQGS
ELVFATRTGTGRGIESHVFRVETHWDLSSWTRALVQGAHAAAELIKEVSIGCTLNRQDVR
LTLHYEKGFTVTREPADLTGGAVLYRYPYEKLKMSADDGIRNLYLDFGGPEGEMVFDLHS
GPKPVVFVLHSFLSAKLTRMGLLT

>Tetraodon nigroviridis_SNTB2(ENSTNIP_11695)
MAVWTRADKNGQLDLLLRDRWIRVAAELTRETLTLTAEAEAGGHHHCANSPGKYPNNGSN
SDFGSPGSSYGSPGSSFGSRHGDFPLALEGSSEAVRKVRVVKQESGGLGISIKGGRENRM
PILISKIFPGLAADQSRALRVGDAILSVNGNDLREATHDLAVQALKKAGKEVTLEVKYIR
EVSPLFKKPSLVADLPWDGVRPQSPSYSGSEDSGSPKHGGSSPSKDRKVISLKMCFISRN
LTMPDLENRLLELHSPDGQHTVVLRCKDAPTANSWFTAMHTNIAALLPQVLAHINAYLGA
SVSASTHPHLKHIGWLAEQIQLEGGRQQYKPVVMALTEKDILLFESVPWSRESWSLPLLT
HPLLATRLVHSGSARGSPAQGSELVFATRTGTGRGIESHVFRVETHWDLSSWTRALVQGA
HAAAELIKEVSIGCTLNRQEVRLTLHYEKGFTVTREPADLTGGAVLFRYPYEKLKMSADD
GIRNLYLDFGGPEGEMVFDLHSGPKPVVFVLHSFLSAKLTRMGLLT

>Gasterosteus aculeatus_SNTB2(ENSGACP_2773)
PRAPPAASAGVGMAVWTRADKRGQLDLLLRDRWIRVAAELTRETLTLTAEAEATGPGGNH
WDYNGSSAAPRNGVPNGNEPGASPGNPARVPPSGPEQLQSQNQGPDGSSEAVRKVRVVKQ
ESGGLGISIKGGRENRMPILISKIFPGLAADQSRALRVGDAILSVNGSDLREATHDLAVQ
ALKKAGKEVTLEVKYIREVSPLFKKPSLVADLPWDGARAQSPSCSGSEDSGSPKHCGASP
RDRKVMSLKMCFISRNLTMPDLENRLLELHSPDGQHTVVLRCKDGATANSWFTAVHTNVA
ALLPHTLAHVNAYLGASSPAHPHLQHIGWLAEQVQLEGGRQQHKPVVMALTEKDILLFES
VPWSRESWSAPLLTHPLLATRLVQCRGARGSPPQGSDTAFVTRTGTGRGIESHVFRVETQ
WDLSTWTRALVQGAHAAAELIKEVSIGCTLNRQDVRLTLHYEKGFTVTREPADPPGGAVL
FRYPYEKLKMSADDGMRNLYLDFGGPEGEMVFDLHSGPKPVVFVLHSFLSAKLTRMGLLT

>Homo sapiens_SNTG1(ENSP_483474)
MDFRTACEETKTGICLLQDGNQEPFKVRLHLAKDILMIQEQDVICVSGEPFYSGERTVTI
RRQTVGGFGLSIKGGAEHNIPVVVSKISKEQRAELSGLLFIGDAILQINGINVRKCRHEE
VVQVLRNAGEEVTLTVSFLKRAPAFLKLPLNEDCACAPSDQSSGTSSPLCDSGLHLNYHP
NNTDTLSCSSWPTSPGLRWEKRWCDLRLIPLLHSRFSQYVPGTDLSRQNAFQVIAVDGVC
TGIIQCLSAEDCVDWLQAIATNISNLTKHNIKKINRNFPVNQQIVYMGWCEAREQDPLQD
RVYSPTFLALRGSCLYKFLAPPVTTWDWTRAEKTFSVYEIMCKILKDSDLLDRRKQCFTV
QSESGEDLYFSVELESDLAQWERAFQTATFLEVERIQCKTYACVLESHLMGLTIDFSTGF
ICFDAATKAVLWRYKFSQLKGSSDDGKSKIKFLFQNPDTKQIEAKELEFSNLFAVLHCIH
SFFAAKVACLDPLFLGNQATASTAASSATTSKAKYTT

>Pan troglodytes_SNTG1(ENSPTRP_34657)
MDFRTACEETKTGICLLQDGNQEPFKVRLHLAKDILMIQEQDVICVSGEPFYSGERTVTI
RRQTVGGFGLSIKGGAEHNIPVVVSKISKEQRAELSGLLFIGDAILQINGINVRKCRHEE
VVQVLRNAGEEVTLTVSFLKRAPAFLKLPLNEDCACAPSDQSSGTSSPLCDSGLHLNYHP
NNTDTLSCSSWPTSPGLRWEKRWCDLRLIPLLHSRFSQYVPGTDLSRQNAFQVIAVDGVC
SGIIQCLSAEDCVDWLQAIATNISNLAKHNIKKINRNFPVNQQIVYMGWCEAREQDPLQD
RVYSPTFLALRGSCLYKFLAPPVTTWDWTRAEKTFSVYEIMCKILKDSDLLDRRKHCFTV
QSESGEDLYFSVELESDLAQWERAFQTATFLEVERIQCKTYACVLESHLMGLTIDFSTGF
ICFDAATKAVLWRYKFSQLKGSSDDGKSKIKFLFQNPDTKQIEAKELEFSNLFAVLHCIH
SFFAAKVACLDPLFLGNQATASAAASSATTSKAKYTT

>Mus musculus_SNTG1(ENSMUSP_118101)
MDFRTTCEETKTGVCLLQDGNQEPFKVRLHLARDLLMLQEQDVLCVSGEPFYSGERTVTI
RRQTVGGFGLSIKGGAEHNIPVVISKISKEQRAELSGLLFIGDAILQINGINVRKCRHEE
VVQVLRNAGEEVTLTVSFLKRAPAFLKLPVNEDCACAPSDQSSGTSSPLCDSGLHLNYHP
NNTDTLSCSSWPTSPGLRWEKRWCDLRLIPLLHARFSQYVPGTDLSRQNAFQVVAVDGVC
SGILQCLSAEDCMDWLQAIASNISNLTKHNIKKINRNFPVNQQIVYMGWCEAREQESLQD
RVYTPVFLALRGSCLYRFLSPPVTTWDWTRAEKTFSVCEIMCKVLKDSDLLDRRKHCFTM
QSECGEDLYFSVELESDLAQWERAFQTATFLEVERIQCKTYACVLESHLMGLTIDFSTGF
ICFDAATKAVLWRYKFSQLKGSSDDGKSKIKFLFQNPDTKQIEAKELEFSNLFAVLHCIH
SFFAAKVACLDPLFLGNQAATTAAVSSASTSKAKHLA

>Canis familiaris_SNTG1(ENSCAFP_10009)

MDFRTACEETKTGICLLQDGNQEPFKVRLHLAKDILMIQEQDVICVSGEPFYSGERTVTI
RRQTVGGFGLSIKGGAEHNIPVVISKISKEQRAELSGLLFIGDAILQINGINVRKCRHEE
VVQVLRNAGEEVTLTVSFLKRAPAFLKLPLNEDCACAPSDQSSGTSSPLCDSGLHLNYHP
NNTDTLSCSSWPTSPGLRWEKRWCDLRLIPLLHSRFSQYVPGTDLSRQNAFQVIAVDGVC
SGIIQCLSAEDCIDWLQAIATNISNLTKHNIKKINRNFPVNQQIVYMGWCEAREQDPLQD
RAYSPTFLALRGSCLYKFLAPPVTTWDWTRAEKTFSVYEIMCKILKDSDLLDRRKHCFTV
QSESGEDLYFSVELDSDLAQWERAFQTATFLEVERIQCKTYACVLESHLMGLTIDFSTGF
ICFDAATKAILWRYKFSQLKGSSDDGKSKIKFLFQNPDTKQIEAKELEFSNLFAVLHCIH
SFFAAKVACLDPLYLGNQTAASTAASSSTTSRAKYTT

>Myotis lucifugus_SNTG1(ENSMLUP_10385)
MDFRTACEETKTGICLLQDGNQEPFKVRLHLAKDILMIQEQDVICVSGEPFYSGERTVTI
RRQTVGGFGLSIKGGAEHNIPVVISKISKEQRAELSGLLFIGDAILQINGINVRKCRHEE
VVQVLRNAGEEVTLTVSFLKRAPAFLKLPLNEDCACAPSDQSSGTSSPLCDSGLHLNYHP
NNTDTLSCSSWPTSPGLRWEKRWCDLRLLPLLHSRFSQYVPGTDLSRQNAFQVIAVDGVC
SGIIQCLSADDCIDWLQAIATNISNLTKHNIKKINRNFPVNQQIVYMGWCEAREQDPLQD
SVYSPIFLALRGSCLYKFLAPPVTTWDWTRAEKTFSVYEIMCKILKDSDLLDQRRHCFTV
QSESGEDLYFSVELDCDLAQWERAFQTATFLEVERIQCKTYACVLESHLMGLTIDFSTGF
ICFDAATKAVLWRYKFSHLKGSSDDGKSKIKFLFQNPDTKQIEAKELEFSNLFAVLHCIH
SFFAAKVACLDPLFLGHQATASAAASSATMSKAKYTT

>Monodelphis domestica_SNTG1(ENSMODP_12397)
MDFRTSCEETKTGICLLQDGNQEPFKVRLHLAKDILMIQEQDVICVSGEPFYSGERTVTI
RRQTVGGFGLSIKGGAEHNIPVVISKISKEQRAELSGLLFIGDAILQINGINVRKCRHEE
VVQVLRNAGEEVTLTVSFLKRAPAFLKLLLNEDCACAPSDQSSGTSSPLCDSGLHLNYHP
TNTDTLSCSSWPTSPGLRWEKRWCDLRLIPLLHSRFSQYLPGSDLCRQNAFQVIAVDGVC
SGIIQCLSAEDCIDWLQAIATNISNLTKHNIKKINRNFPVNQQIVYMGWTEEREQDSLQD
RKYTPKFLALRGSSLYKFLAPPVTTWDWTRAEKTFSVYEIMFKIFKDSDLLDRRKHCFGV
QSETGEDLYFSVELECDLAQWEKAFQTATFLEVERIQCKTYACVLESHLMGLTIDFSMGF
VCFDAATKAVLWRYKFSQLKGSSDDGKSKIKFLFQNPDTKQIEAKELEFSNLFAVLHCIH
SFFAAKVACLDPLYLGNQASASASTSASSAATGKVKYTT

>Taeniopygia guttata_SNTG1(ENSTGUP_11271)
MDFRTTCEETKTGVCLLQDGNQEPFKVRLHLAKDILMIQEQDVICVSGEPFYSGERTVTI
RRQTVGGFGLSIKGGAEHNIPVVISKISKEQRAELSGLLFIGDAILQINGINVRKCRHEE
VVQVLRNAGDEVTLTVSFLKRAPAFLKLPLNEDCACAPSDQSSGTSSPLCDSGLHLNYHP
NNTDTLSCSSWPASPGLRWEKRWCDLRLIPLLHSRFSQYLPGSDLCRQNAFQVIAVDGVC
SGIIQCLSAEDCIDWLQAIASNISNLTKHNIKKINRNFPVNQQIVYMGWTEEREQDSLQD
RMYTPKFLALRGASLYKFIAPPVTTWDWTRAEKTFSVYEIMCKILKDSDLLDRRKHCFSV
QSESGEDLYFSVELECDLTLWEKAFQTATFLEVERIQCKTYACVLESHLMGLTIDFSMGF
VCFDAATKAVLWRYKFSQLKGSSDDGKSKIKFLFQNQDTKQIEAKELEFSNLFAVLHCIH
SFFAAKVACLDPLYVGSQATASAAPTTSGTGKLKYST

>Anolis carolinensis_SNTG1(ENSACAP_12130)
MDFRTACEETKTGICLLQDGNQEPFKVRLHLAKDILTIQEQDVICVSGEPFYSGERTVTI
RRQTVGGFGLSIKGGAEHNIPVVVSKISKEQRAELSGLLFIGDAILQINGINVRKCRHEE
VVQVLRNAGEEVTLTVSFLKRAPAFLKLPLNEDCACIPSDQSSGTSSPLCDSGLHLNYHP
NNTDTLSCSSWPTSPGLRWEKRWCDLRLIPLLHSRFSQYLPGSDMCRQNAFQVIAVDGIC
SGIIQCLSGEDCIDWLQAIATNISNLTKHNIKKINRNFPVNQQIVYMGWTEEREQDSLQD
RMYTPKFLALRGSSLYKFIAPPVTTWDWTRAERTFSVYEIMYKILKDSDLLDRRKHGFGV
QSEMGEDLYFSVELESDLILWEKAFHSATFLEVERIQCKTYACVLESHLMGLTIDFGMGF
VCFDAATKAVLWRYKFSQLKGSSDDGKSKIKFLFQNPDTKQIEAKELEFSNLFAVLHCIH
SFFAAKVACLDPQYVGSQAAASTSVSAASTAAAAAAAGTSTLATSSGSSKLKYTT

>Xenopus tropicalis_SNTG1(ENSXETP_12501)
MEFNLCICAKTGICLLQDGNQEPFKVRLHLAKDILMIQEQDVICVSGEPFYSGERTVAIR
RQTVGGFGLSIKGGSEHNIPVVISKISKEQRAELSGLLFIGDAILQINGINVRKCKHEEV
VQVLRNAGEEVTLTVSFLKRSPAFLKLPLNEDCSCVPCDQSSGTSSPLCDSGLHLNYHPN
NTDSLSCSSWPTSPGLRWEKRWCDLRLIPLLHSRLSQHITGTDLCRQNAFQVTAVDGVGS
GIIQCLLAEECIDWLQSISANISNLTKHNIKKINRNFPVNQQIVFLGWVEEREQDTLKDY
VYSPKFLALRGSSLYKFNSPPVTTWDWMRAEKKFTVYEIMCKIFKDSDLLDRRKHCFGVQ

SETGEDLYFSVELECDLLQWEKAFQTATFLEVERIQCKTYACVIESHLMGLTIDFGMGFI
CFDAATKAILWKYKFSQLKGSSDDGKSKIKFLFQNQDTKQIEAKELEFSNLFAVLHCIHS
FFAAKVACLDPRFVDSQGAASAAVITTSCATAGVCKMKYST

>Danio rerio_SNTG1(ENSDARP_134700)
QTKTGIYLLQDGSEEPFNIRLHLAKDILTIQEQDVICVSGEPFYSSERTVTIRRQTIGGF
GLSIKGGAEHKIPVVISKISKEQKAELSGLLFIGDGILQINGINVRSYRHEEVVQVLRNA
GEEVTLTVSFLKRTPAFLKLPLCEDCSCIPSDQSSGTSSPLCDSGLHLNYHPNNTDTLSC
SSWPMSPGLRWEKRWVDLRLIPLLHARLSQYVPGTDICRQNAFQVIAVDGVCSGVIQCPG
TDECTDWLQAISANISALTKHNVHLTLKCRLPGDTIIYMGWVDEKEQDSVQDRMYTPKFL
ALRGSSLFKFSAPPVTTWDWTRAEKSFTVYEIMFKILKFYLISSYFQVYFIYVRVQDSEL
LDKRKHCFSVQTDSGEDIFFSVELECELLLWEKAFQMATFFEVERIQCKTYACIVDSHLM
GLTIDFSMGFVCFDAASKAVLWRYKFSQLKGSSDDGKSKIKFLFQNQDTKLIEAKELEFS
NLFAVLHCIHAFFAAKVACLDPMFVSSRNAAMAPVVSA

>Takifugu rubripes_SNTG1(ENSTRUP_16051)
MDFKTSNEETKTGIYLMQEGNEEPFNIRLHLAKDILTIQEQDVICVSGEPFYSSERTVTI
RRQTIGGFGLSIKGGAEHKIPVVISKISKEQKAELSGLLFIGDGILQINGINVRSYRHEE
VVQVLRNAGEEVTLTVSFLKKTPAFLKLPLCEDCTCIPSDQSSGTSSPLCDSGLHLNYHP
NNTDTLSCSSWPTSPALRWEKRWVDLRLIPLLHSRLSRYSPGSDSCRKNAFQVIAVDGVC
SGVVQFPTAEDCLDWLQAISGNISSLTKHNVKKINRNFPVNQQIIYMGWVDEKEQDSVQD
RMYSPKFLALRGSSLFKFSAPPVTTWDWTRAEKSFTLYEIMFKILKDNDLLDKRRHCFSV
QTESGEDMFFSVELDCELLIWEKAFQTATFLEVERIQCKTYACIMESHLMGLTVDFGMGF
VCFDAASKAVLWRYKFSQLKGSSDDGKSKIKFLFQNQDSKSIEAKELEFSNLFAVLHCIH
AFFAAKVACLDPMFVESQGTATTAGFPSLSAISSKHLS

>Tetraodon nigroviridis_SNTG1(ENSTNIP_11343)
MDFKTSNEETKTGIYLMQEGNEEPFNIRLHLARDILTIQEQDVICLSGEPFYSSERTVTI
RRQTIGGFGLSIKGGAEHKIPVVISKISKEQKAELSGLLFIGDGILQINGINVRSYRHEE
VVQVLRNAGEEVTLTVSFLKKTPAFLKLPLCEDCTCIPSDQSSGTSSPLCDSGLHLNYHP
NNTDTLSCSSWPTSPALRWEKRWVDLRLIPLLHSRLSRYSPGSDSCRKNAFQVIAMDGVC
SGVVQFATAEDCLDWLQAISSNISSLTKHNVKKINRNFPVNQQIIYMGWADEKEQDSVQD
RMYSPKFLALRGSSLFKFSAPPVTTWDWTRAEKSFTVYEIMFKILKDNDLLDKRKHCFSV
QTESGEDMFFSVELDCELLIWEKAFQTATFLEVERIQCKTYACIMESHLMGLTVDFTMGF
VCFDAASKAVLWRYKFSQLKGSSDDGKSKIKFLFQNQDSKSIEAKELEFSNLFAVLHCIH
AFFAAKVACLDPMFVESQGTATTAGFPSLSAISCHSQT

>Gasterosteus aculeatus_SNTG1(ENSGACP_23700)
MDFKTSNEETKTGIYLMQEGNEEPFNIRLHLARDILTIQEQDVICVSGEPFYSSERTVTI
RRQTIGGFGLSIKGGAEHKIPVVISKISKEQKAELSGLLFIGDGILQINGINVRSYRHEE
VVQVLRNAGEEVTLTVSFLKKTPAFLKLPLCEDCTCVPSDQSSGTSSPLCDSGLHLNYHP
NNTDTLSCSSWPACPGLRWEKRWVDLRLIPLLHSRLSQYNPGSDVCRKNAFQVIAVDGVC
SGVLQFLTADDCLDWLQALASNISSLTKHNVKKINRNFPVNQQIIYMGWVDEKEQDSVQD
RMYSPKFLALRGSSLFKFSAPPVTTWDWTRAEKSFTVYEIMCKILKDNDLLDKRKHCFGV
QTESGEDLFFSVELECELLIWEKAFQMATFLEVERIQCKTYACIMESHLMGLTVDFGAGF
VCFDAASKAVLWRYKFSQLKGSSDDGKSKIKFLFQNQDSKSIEAKELEFSNLFAVLHCIH
AFFAAKVACLDPIFVESQGNATPAGFPSLCSISCNSQTPKLKYAT

>Oryzias latipes_SNTG1(ENSORLP_21863)
TSFFPTWNCSQTKTGIYLMQEGNEEPFNVRLHLAKDILTIQEQDVICVSGEPFYSSERTV
TIRRQTIGGFGLSIKGGAEHKIPVVISKISKEQKAELSGLLFIGDGILQINGINVRSYRH
EEVVQVLRNAGEEVTLTVSFLKKTPAFLKLPLCEDCTCIPSDQSSGTSSPLCDSGLHLNY
HPNNTDTLSCSSWPTSTGLRWEKRWVDLRLIPLLHSHLSQYMPGSDTCRKNAFQVIAVDG
VCSGVLQFPTAEDCLDWLHAIASNISSLTKHNVKKINRNFPVNQQIIYMGWVDEKEQDSV
QDRMYTPKFLALRGSSLFKFTAPPVTTWDWTRAEKSFTVYEIMSKILKVRLSLCVIIQFP
PHFKERLSQENDLLDKRRHCFSVQTESGEDMFFSVELECDLLVWEKAFQMATFLEVERIQ
SKTYACIMESHLMGLTIDFSMGFVCFDAASKAVLWRYKFSQLKGSSDDGKSKIKFLFQNQ
ESKSIEAKELEFSNLFAVLHCIHAFFAAKVACLDPVFLESQGTAANAGIPS

>Homo sapiens_SNTG2(ENSP_311837)
MGTEGPPPPAASRGRQGCLLVPARTKTTIALLYDEESENAYDIRLKLTKEVLTIQKQDVV

CVGGSHQGRNRRTVTLRRQPVGGLGLSIKGGSEHNVPVVISKIFEDQAADQTGMLFVGDA
VLQVNGIHVENATHEEVVHLLRNAGDEVTITVEYLREAPAFLKLPLGSPGPSSDHSSGAS
SPLFDSGLHLNGNSSTTAPSSPSSPIAKDPRYEKRWLDTLSVPLSMARISRYKAGTEKLR
WNAFEVLALDGVSSGILRFYTAQDGTDWLRAVSANIRELTLQNMKMANKCCSPSDQVVHM
GWVNEKLQGADSSQTFRPKFLALKGPSFYVFSTPPVSTFDWVRAERTYHLCEVLFKVHKF
WLTEDCWLQANLYLGLQDFDFEDQRPYCFSIVAGHGKSHVFNVELGSELAMWEKSFQRAT
FMEVQRTGSRTYMCSWQGEMLCFTVDFALGFTCFESKTKNVLWRFKFSQLKGSSDDGKTR
VKLLFQNLDTKQIETKELEFQDLRAVLHCIHSFIAAKVASVDPGFMDSQSLARKYMYSS

>Pan troglodytes_SNTG2(ENSPTRP_19957)
MGTEGPPPPAASRGRQGCLLVPARTKTTIALLYDEESENAYDIRLKLTKEVLTIQKQDVV
CVGGSHQGRNHRTVRLRRQPVGGLGLSIKGGSEHNVPVVISKIFKDQAADQTGMLFVGDA
VLQVNGIHVENATHEEVVHLLRNAGDEVTITVEYLREAPAFLKLPLGSPGPSSDHSSGAS
SPLFDSGLHLNGNSSTTAPSSPSSPIAKDPRYEKRWLDTLSVPLSMARISRYKAGTEKLR
WNAFEVLALDGVSSGILQFYTAQDGTDWLRAVSANIRELTLQNMKMANKCCSPSDQVVHM
GWVNEKLQGADSSQTFRPKFLALKGPSFYVFSTPPVSTFDWVRAERTYHLCEVLFKVHKF
WLTEDCWLQANLYLGLQDFDFEDQRPYCFSVVAGHGKSHVFNVELGSELAVWEKSFQRAT
FMEVQRTGSRTYMCSWQGEMLCFTVDFALGFTCFESQTKNVLWRFKFSQLKGSSDDGKTR
VKLLFQNLDTKQIETKELEFQDLMAVLHCIHSFIAAKVASVDPGFMDSQSLARKYMYSS

>Callithrix jacchus_SNTG2(ENSCJAP_43085)
PAAPRGRQGCLLVLARLKTAVALLYSEEYHSAYDIRLKLTKEMLTIQKQDVICVGGSHQG
RSHRTVTLRRQPVGGLGLSIKGGSEHNVPVVISKIIKGQAADQTGMLFVGDAVLQVNGIN
VENATHEEVVHLLRNAGDEVTITVEYLREAPAFLKLPLGSPGPSSDHSSGASSPLFDSAL
HLNGNSRNTAPSSPSSPIAKDPRYEKRWLDALSVPLSMARISRYKAGTEKFRWNAFEVLA
LDGVSSGILQFYTAQDGADWLRAVSANIRELTLQNMKLANRCCSASDQVVHMGWVHEKLH
GADSSQTCRPKFLALKGPSFYVFSSPPVSTFDWVRAERTYYLCEVLFKVHKFWLTDDCWL
QANLYLGLQDGDFEDQRPYCFSVVAGHGKSHVFSVELGSELAVWEKSFQRAMFMEVQRSG
SKTYACSWQGEMLCFTVDFALGFTCFESKTKNVLWRFKFSQLKGSSDDGKTRVKLLFQHL
DTKQIETKELEFQDLTAVLHCIHAFIAAKVASVDPGFVDSQSLAKKYMYSN

>Mus musculus_SNTG2(ENSMUSP_21004)
MSAEGSQSLAAPRGRPSHLLVPARTKTALALLYDEGLENAYDVRLKLTKEVLTIQKQDVV
CIGGAPPGANHRTVTLRRQPVGGLGLSIKGGAEHGVPVVISKIFKDQAADQTEMLFIGDA
VLQVNGINVENATHEEVVHLLRNAGDDVTITVEYLREAPSFLKLPLGSPGPSSDHSSRAS
SPLFDSGLHLNGHCSHTAPSSPSSPIANEPKYEKRWLDTLSVPLSMARISRYKAGTEKLR
SSALEVLALDGASTGVLQFSTAQDCADWLRSISTNISDLTLQHMKMANKCCSPCDQVVHM
GWVNERLQGADNSQNFRPKFLALRGSSFYIFGAPPVSTLDWGRAERAYNLCEVLFKVHKF
WLSDNYWLQANLYLGLQDFDCEDPRSYCFSVLANHGKSHIFSVELGSELAVWEKAFQRAT
FMEVQRTGSKTYLCSWQGETLCFTVDFALGFTCFDGKTKNVLWRFKFSQLKGSSDDGKTR
VKLLFQNLDTKQIETKELEFQDLTAVLHCIHSFIAAKVASLDPVFMDSQSMARRYLCSS

>Felis catus_SNTG2(ENSFCAP_12755)
ARGRGPVVAPTSLRSRPWPVSALPPQTRTGLALLHHEGSGSPCDVRLKLTREVLTIQKQD
VVCVRGSSHGAHHRTVTLRRQPVGGLGLSVKGGAEHRVPVVISKIFRGHAADQTGMLFVG
DAVLQVNGINVEHATHEEVVHLLRNAGDEVAITVDYLREAPAFLKLPLGSPGPAGDHSSG
ASSPLFDSGLHLNSNPSSTDSPSPSSPAADAPQYEKRWLDTLSVPLSMARISRCKAGTGK
CRSGAFQVLAVDGVRTGVLQFHTARESADWLRAVSATISDLTLESMKMVNEGCSPCDQVV
HMGWVRERLEGTSSRETFRSKFLALKGSLLYVFSTAPVSTLDWVRAEKTYNLCEVLFKLH
KLWLADDGWLHEACHLGPPGFAPQRPFSFSVLVGHGQSRVFSVDLGSELAAWERSFQRAT
FKEVQRSRSKTYTCSWQGEMLCFTVDFALGFTCSDSKTKNVLWRFKFSQLKGSSDDGNAR
VKLLFQHPDTKQIEVKELEFPDLTAVLHCVHAFLAAKVASVDPAFMDSQSFARKSASAE

>Monodelphis domestica_SNTG2(ENSMODP_17858)
SQGPPPPASFRLLIGCILVPLLTKTGIALLYDEGSENAFDIRLKLTKEVLTIQKQDVVCV
SGSNHSTNHRTVTLHRQPVGGLGLSIKGGAEHKVPVVISKIFKDQAADQTGMLFIGDAVI
QVNGINVENATHEEVVHLLRNAGDEVTITVQYLREAPSFLKLPLGSPGPSSDHSSGASSP
LFDSGLHLNGNSSNTAPSSPSSPIANEPKYEKRWLDTLSVPLSMARISRYKAGTDKLRSN
AFEVLALDGVSTGIIQFYTSQESADWLRAVSTNIGDLTLQNMKMANKCCSPCDQVVHMGW
VNERIQGIDSSQPYKYKFLALKGSSFYIFSTPPVSTLDWVRAEKIYNLCEVLFKVHKLWL
ADDCWLQANFYLGLHQDYDLEDQRPYCFSVMVGQGKSHFFNVELGSELAVWEKSFQRAIF

MEVQRTGSKTYMCSWQGEALCFTVDFALGFTCFDSKTKNVLWRFKFSQLKGSSDDGKTRV
KLLFQNLDTKQIEMKELEFQDLTAILHCIHSFIAAKVASVDPMFIDSQSIARRYMYSN

>Gallus gallus_SNTG2(ENSGALP_26350)
VMYMGITGLSLERAEPHELLIPDRTKTGIALLYDEVSENAYDIRLKLTKEVLTIQKQDVI
CVSGSDRSANHRTVTLHRQPVGGLGLSIKGGAEHKVPVVISKMFKDQAADQTGMLFIGDA
ITQVNGINVESATHEEVVHLLRNAGDEVTITVQYLREAPSFLKLPLGSPGPSSDHSSGAS
SPLFDSGLHLNGNSANTAPSSPSSPIANEPKYEKRWLDTLSVPLSMARISRYKTGTDKLR
SNAFEVLALDGVSTGILQFYTAQESADWLRAISTNISDLTLQNMKMANKCSSPSDQVVHM
GWVHERLEGTDSSQLYKFKFLALKGSSFYIFSTPPVSTLDWVRAEKMYNLCEVLFKIHKL
WLADDCWLQANLYFGIHQDFDFEDHRPYCFSVMVGHGKSHCFNVELGSELAVWEKSFQRA
IFLEVQRTGSKTYMCSWQGDTLCFTVDFALGFTCFDSKTKNVLWRFKFSQLKGSSDDGKT
KVKLLFQNPDTKQIETKELEFQDLTAVLHCIHSFIAAKVASVDPVFIDSQSIARKYMYSN

>Pelodiscus sinensis_SNTG2(ENSPSIP_6229)
QTKTGIALLYDEGSENAYDIQLKLTKEVLTIQKQDVICVSGSDHSTNHRTVTLHRQPVGG
LGLSIKGGAEHKVPVVISKIFKDKAADQTGMLFIGDALLQVNGINVESATHEEVVHLLRN
AGDEVTITVQYLREAPSFLKLPLGSPGPSSDHSSGASSPLFDSGLHLNGNTTNTAPSSPS
SPIANEPKYEKRWLDTLSVPLSMARISRYKAGTDKLRSNAFEVLALDGVSTGILQFYTAQ
ESADWLRALATNISDLTLQNMKMANKCCSLSDQVIHMGWVNERLEGTDSSQLYKYKFLAL
KGSSFYIFSTPPVSTLDWIRAEKIYHLCEVLFKVHKLWLADDCWLQANLYLGIHQDYAFE
DQRPYCFSVMVGHGKSHSFNVELGSELVAWEKSFQRAIFLEVQRTGSKTYMCSWQGVALC
FTVDFALGFTCFDSKTKNVLWRFKFSQLKGSSDDGKTKVKLLFQNIDTKQIEMKELEFQD
LTAVLHCIHSFIAAKVASLDPVFIDSQSIARKYMYSN

>Danio rerio_SNTG2(ENSDARP_130115)
MNAAAKHQPDLLERPASLLVPTWTKTGLALLYDEHTSNTYDVRLKLTKDLLIIQKQDVVC
ASGIESHLNHRTVVLRRQATGGLGLSIKGGAEHKVPVVISKIFKDQVADQTGKLFIGDAV
LQVNGINVEKCTHEEVVHLLRTAGDEVSITVRYLREAPAFLKLPLGSPGPSSDHSSRASS
PLFDSGLHLNGNSSNTAPSSPSSPSANEPKYEKRWLDAVTLPLFMARISRRKAGTDKLRS
NSFEVIALDGVCTHVLQFCTAGESTDWLQAISTNINDLTQDSIKTVNKYCSADDQVDCPH
GLGVRADSRRRCRQRRRRRSLLQIPGAQRISSAYLQDSTYWTQAEASFHLCEVLFKVHKL
WIAEDCWIQTKLYMGLQQDGELRDSEPFCFSVLLGHGQSFCFSVELGSDLVLWEKAFQRA
VFMEVQRVRSKSYMCSSQGKILCFTIDFESGFSCSDTLSKKIIWKYKFSQLKGSSDDGKT
RVKLLFQNSENKQIEMKELEFANLTAVLHCIHSFIAAKVATVDPVFVSNQSLTQKYMCSS

>Tetraodon nigroviridis_SNTG2(ENSTNIP_8351)
AAQITFSFADPRHDPSHRVKLLTIKTKSGIALLYKEETNNSYDVCLKLTKEVLTIQKLDV
VCTNGPETQVNHRTVVLRRQANGGLGLSIKGGAEHNVPVVISKIFKDQVADQTGKLFVGD
AVLQVNGIHVELCTHKEVVHLLRTAGDEVTITVRYLREVPSFLKLPLGSPGPSTDHSRVS
SPLFDSGLHLNGNGNSTAPSPPLPSANEPKYEKRWLDAVSLPLLMARVCRYKAATDQLRS
NCFEVFALDGASTNILQFCTAAESTDWLQAISTNINELTQENIKIINKYCSSDDQIVHMG
WVCECPEGGAAGRSPAFKFLALRGPHFYIFRHPPVSAAEWGQAETSYNLYEVLFKVHKLW
MAEDCWLQARLYLGLEHSPDQQDNESLCFSILVGHGQSHTFRVELATDLAIWEKSFQRAV
FLEVQRIRSKSYMCSCQGAVLCFTIDFGSGFTCSEGSSKTVLWRYKFSQLKGSSDDGKTR
VKLLFKNAESQQIEMKELEFANLTAVLHCIHSFIAAKVASMDPLFMYSQSFHGNHVNS

>Gasterosteus aculeatus_SNTG2(ENSGACP_7480)
MNVTGKQQPELIERPASLPVPTSTKSGIALLYNEETNNTYDVCLKLTKELLTIQKLDVVC
TSGSESPANHRTVVLRRQAAGGLGLSIKGGAEHNVPVVISKIFKDQVADRTGKLFVGDAV
LQVNGINVEPCTHKEVVHLLRTAGDEVTVTVRYLREVPSFLKLPLGSPGPSTDHSRVSSP
LFDSGVHLNGNGNNTAPSPPPPADKPRYEKRWLDAVPLPLLMARVSRCKAATDQLRSNC
LDVFALDGASTNILQFCTAAESTDWLQAISTNINELTQENIKMINKYCPSDDQTVHMGWT
CGSPEGGAAGRASAFKFLALRGPHFYIFRSPPISAADWGQAETTYNLYEVLFKVHKLWMA
EDCWLQARLYLGLEHSPEQRDNESLCFSILVGHGQSHTFRVELASDLATWEKSFQRAVFW
EVQRIRSKSYMCSSQGSVLSFTVDFGSGFSCAEGSSKTALWRYKFSQLKGSSDDGKTRVK
LLFKNAESNQIEMKELEFANLTAVLHCIHSFIAAKVASVDPLFVCSHGL

>Oryzias latipes_SNTG2(ENSORLP_22013)
CSFQRQQTKSGIALLYNEDTNNTYDVCLKLTKEALTIQKLDVICTSGTESQVNHRTVVLR
RQATGGLGLSIKGGAEHNVPVVISKIFKDQVADQTGKLFIGDAVLQVNGINVESCTHEEV

VHLLRTAGDEVTITVRYLREVPSFLKLPLGSPGLDHSRGSSPLFDSGLHLNGNENNAAPS
PPSPSANEPKYEKRWLDDVCMPLLMARVSRYKAGTDKLRSNCFQVFASGGTCTNILQFFT
AAESTDWLQAISANINGLTQENIKIINKHSPAENQIVHMGWACESLEDSTVGRSSTFKFL
ALRGPYFYIFKRPPISSADWGKSEITYNLYEVLFKVHKLWMAEDCWLKARLYLGLENSPE
QQDNEPLCFSILVGHGQSHTFRVELASDLAIWEKSFQRAVFTEMNKTVSKSYMCSSHGTV
LCFTIDFGSGFSCSEASSSTILWRYKFSQLKGSSDDGKTRVKLLFKNAESKQIEMKELEF
ANLTAVLHCIHSFIAAKVASMDPLFVCSPSFSGNCLNS

>Homo sapiens_GOPC(ENSP_52569)
MSAGGPCPAAAGGGPGGASCSVGAPGGVSMFRWLEVLEKEFDKAFVDVDLLLGEIDPDQA
DITYEGRQKMTSLSSCFAQLCHKAQSVSQINHKLEAQLVDLKSELTETQAEKVVLEKEVH
DQLLQLHSIQLQLHAKTGQSADSGTIKAKLERELEANKKEKMKEAQLEAEVKLLRKENEA
LRRHIAVLQAEVYGARLAAKYLDKELAGRVQQIQLLGRDMKGPAHDKLWNQLEAEIHLHR
HKTVIRACRGRNDLKRPMQAPPGHDQDSLKKSQGVGPIRKVLLLKEDHEGLGISITGGKE
HGVPILISEIHPGQPADRCGGLHVGDAILAVNGVNLRDTKHKEAVTILSQQRGEIEFEVV
YVAPEVDSDDENVEYEDESGHRYRLYLDELEGGGNPGASCKDTSGEIKVLQGFNKKAVTD
THENGDLGTASETPLDDGASKLDDLHTLYHKKSY

>Gorilla gorilla_GOPC(ENSGGOP_13221)
MSAGGPCPAAAGGGPGGASCSVGAPGGVSMFRWLEVLEKEFDKAFVDVDLLLGEIDPDQA
DITYEGRQKMTSLSSCFAQLCHKAQSVSQINHKLEAQLVDLKSELTETQAEKVVLEKEVH
DQLLQLHSIQLQLHAKTGQSVDSGTIKAKLLKSGPSVEELERELEANKKEKMKEAQLEAE
VKLLRKENEALRRHIAVLQAEVYGARLAAKYLDKELAGRVQQIQLLGRDMKGPAHDKLWN
QLEAEIHLHRHKTVIRACRGRNDLKRPMQAPPGHDQDSLKKSQGVGPIRKVLLLKEDHEG
LGISITGGKEHGVPILISEIHPGQPADRCGGLHVGDAILAVNGVNLRDTKHKEAVTILSQ
QRGEIEFEVVYVAPEVDSDDENVEYEDESGHRYRLYLDELEGGGNPGASCKDTSGEIKVL
QGFNKKAVTDTHENGDLGTASETPLDDGASKLDDLHTLYHKKSY

>Pongo abelii_GOPC(ENSPPYP_18990)
MAAGGPCPAAAGGGPGGASCSVGAPGGVSMFRWLEVLEKEFDKAFVDVDLLLGEIDPDQA
DITYEGRQKMTSLSSCFAQLCHKAQSVSQINHKLEAQLVDLKSELTETQAEKVVLEKEVH
DQLLQLHSIQLQLHAKTGQSVDSGTIKAKLSGPSVEELERELEANKKEKMKEAQLEAEVK
LLRKENEALRRHIAVLQAEVYGARLAAKYLDKELAGRVQQIQLLGRDMKGPAHDKLWNQL
EAEIHLHRHKTVIRACRGRNDLKRPMQAPPGHDQDSLKKSQGVGPIRKVLLLKEDHEGLG
ISITGGKEHGVPILISEIHPGQPADRCGGLHVGDAILAVNGVNLRDTKHKEAVTILSQQD
DENVEYEDESGHRYRLYLDELEGGGNPGASCKDPSGEIKVLQGFNKKAVTDTHENGDLGT
ASETPLDDGASKLDDLHTLYHKKSY

>Oryctolagus cuniculus_GOPC(ENSOCUP_18266)
MFRWLEVLEKEFDKAFVDVDLLLGEIDPDQADITYEGRQKMTSLSSCFAQLCHKAQTVSQ
INHKLEAQLVDLKSELTETQAEKVVLEKEVHDQLLQLHSIQLQLHAKTGQSVDSGTIKAK
LERELEANKKEKVKEAQLEAEVKLLRKENEALRRHIAVLQAEVYGARLAAKYLDKELAGR
VQQIQLLGRDMKGPAHDKLWNQLEAEIHLHRHKTVIRACRGRNDLKRPMQAPPGHDQDSL
KKSQGVGPIRKVLLLKEDHEGLGISITGGKEHGVPILISEIHPGQPADRCGGLHVGDAIL
AVNGVNLRDTKHKEAVTILSQQRGEIEFEVVYVAPEVDSDDENVEYEDESGHRYRLYLDE
LEGGGNPGASSKDASGEIKVLQEYNKKPVTDTHENGDLGSSSETPLDDSASKLDDLHSLY
HKKSY

>Felis catus_GOPC(ENSFCAP_22118)
AQLVDLKSELTETQAEKVVLEKEVHDQLLQLHSIQLQLHAQTGQSVDSGTIKAKLERELE
ANKKEKMKEAQLEAEVKLLRKENEALRRHIAVLQAEVYGARLAAKYLDKELAGRVQQIQL
LGRDMKGPAHDKLWNQLEAEIHLHRHKTVIRACRGRNDLKRPMQAPPGHDQDSLKKSQGV
GPIRKVLLLKEDHEGLGISITGGKEHGVPILISEIHPGQPADRCGGLHVGDAILAVNGVN
LRDTKHKEAVTILSQQRGEIEFEVVYVAPEVDSDDENVEYEDESGHRYRLYLDELEGGSN
PGASCKDTSGEIKVLQGFNKKAVTDGHENGDLGTSSEPPVEDSASKLDDLHSLYHKKSY

>Myotis lucifugus_GOPC(ENSMLUP_6884)
MSAGGPCPAGAGGGPGGASCAVGVSPGGVSMFRWLEVLEKEFDKAFVDVDLLLGEIDPDQ
ADITYEGRQKMTSLSSCFAQLCHKVQTVSQINHKLEAQLVDLKSELTETQAEKVVLEKEV
HDQLLQLHSVQLQLHAKTGQSVDSGTIKAKLLKSVPSVEELERELEANKKEKMKEAQLEA
EVKLLRKENEALRRHIAVLQAEVYGARLAAKYLDKELAGRVQQIQLLGRDMKGPAHDKLW

NQLEAEIHLHRHKTVIRACRGRNDLKRPMQAPPGHDQDSLKKSQGVGPIRKVLLLKEDYE
GLGISITGGKEHGVPILISEIQPGQPADRCGGLHVGDAILAVNGVNLRDTKHKEAVTILS
QQRGEIEFEVVYVAPEVDSDDENVEYEDESGHRYRLYLDELEGGSNPGATCKDTSGEVKV
LQGFNKKSVTDGHENGDLGTSRETPLEDSAAKLDDLHSLYQRKSC

>Monodelphis domestica_GOPC(ENSMODP_22195)
MSAGGPCSAGPGGVSGGASCASGVPPGGVSMFRWLEVLEKEFDKAFVDVDLLLGEIDPDQ
ADITYEGRQKMTSLSSCFAQLCHKAQTVSQINHKLEAQLVDLKSELTETQAEKVVLEKEV
HDQLLQLHSVQLQLHAKTGQSVDSGTIKAKLSVPSVEELERELEANKKEKMKEAQLEAEV
KLLRKENEALRRHIAVLQAEVYGARLAAKYLDKELAGRVQQIQLLGRDMKGPAHDKLWNQ
LEAEIHLHRHKTVIRACRGRNDLKRSMQAPPGHDQDSLKKSQGVGPIRKVLLLKEDHEGL
GISITGGKEHGVPILISEIHPGQPADRCGGLHVGDAILAVNGVNLRDAKHKEAVTILSQQ
RGEIEFEVVYVAPEVDSDDENVEYEDESGHRYRLYLDELEGSGNPVAHRKGASGDIKLLQ
GFNKKPVIDGHENGDLGTSSETPLNDSASKLAESAESLS

>Gallus gallus_GOPC(ENSGALP_23990)
MRAGTFRSPGCVAAPGEPGPPRGRRGTAEAGGGASRALCEARPGRAVAGGMSAAGGVGSC
GPGGAACGGGVGGAASAPGGGVSMFRWLEVLEKEFDKAFVDVDLLLGEIDPDQADITYEG
RQKMTSLSSCFAQLCHKAQTVSQINHKLEAQLVDLKSELTETQAEKAVLEKEVHDQLLQL
HAVQLQLHAQTGQNVDSGAIKAKLERELEANKKEKVKEAQLEAEVKLLRKENEALRRHIA
VLQAEVYGARLAAKYLDKELAGRVQQIQLLGRDMKGPAHDKLWNQLEAEIHLHRHKTVIR
ACRGRNDLKRPMQAPPGHDPDALKKSQGVGPIRKVLLVKEDHEGLGISITGGKEHGVPIL
ISEIHPGQPADRCGGLHVGDAILAVNGVNLRDAKHKEAVTILSQQRGEIEFEVVYVAPEV
DSDDENVEYEDESGHRYRLYLDELEEGANSGSNRKEVNVDVKPSQVFDKKPSIDGHENGD
LGNSVEPSLEDAAPKLTRSAESLS

>Anolis carolinensis_GOPC(ENSACAP_14036)
MSGGGSCAGPGPGACGGAGAASSSASSPSGAGGLSMFRWLEVLEKEFDKAFVDVDLLLGE
IDPDQADITYEGRQKMTSLSSCFAQLCHKAQTVSQINHKLEAQLVDLKSELTETRAEKAV
LEKEVHDQLLQLHAIQLQLHAKTGQSVDSGAIKAKLSVPSVEDLERELEANKKEKVKEAQ
LEAEVKLLRKENEALRRHIAVLQAEVYGARLAAKYLDKELAGRVQQIQLLGRDMKGPAHD
KLWNQLEAEIHLHRHKTVIRACRGRNDLKRPMQAPPGHNTDSLKKSQGVGPIRKVLLVKE
DHEGLGISITGGKEHGVPILISEIHPGQPADRCGGLHVGDAILAVNGVNLRDAKHKEAVT
ILSQQRGEIEFEVVYVAPEVDSDDENVEYEDESGHRYRLYLDELEEGGNSASSRKDGSGD
VKSLQVFEKKASIDGHENGDLGNSVDTEETVSKLAQSAESLA

>Xenopus tropicalis_GOPC(ENSXETP_28166)
MSAAPCSTSASGPVPGMSMFRWLEVLEKEFDKAFVDVDLLLGEIDPDQADITYEGRQKMT
TLSSCFAQLCHKAQTVSQINHKLEAQLVDMKSELTEVQAEKAVLDKEVHEQLLQLHAVQL
QLHSKTGQSVDSGAIKAKLSVPSVEAMEKELEANKKEKVKEAQLEAEVKLLRKENEALRR
HIAVLQAEVYGARLAAKYLDKELAGRVQQIQLLGRDMKGPAHDKLWNQLEAEIHLHRHKT
VIRACRGRNDLKRPLPVPPGHDPDCLKKSQGVGPIRKVILAKEDHEGLGISITGGKEHGV
PILISEIHPAQPADRCGGLHVGDAILAVNGINLRDAKHKEAVTILSQQRGEIEFEVVYVA
PEIDSDDENVEYEDESGHRYRLYLDELEEAGANPGVSTKDGSADTKPLQAFEQKLHIDGN
ENGDLETASESLSGGDTASKLAYSKSFGRRQAY

>Takifugu rubripes_GOPCa(ENSTRUP_8920)
MSMFRWLEVLEKEFDKAFVDVDLLLGEIDPDQVDITYEGRQKMTTLSSCFAQLCHKAQTV
FQLNHKLEAQLVDQRSELTEVKAEREVMEREVHDQLLQLHSLQLQLYAKQGQAEDSDTIK
DRLKSDLRRRKVK

>Takifugu rubripes_GOPCb(ENSTRUP_8919)
PVGQSSGSGMSMFRWLEVLEKEFDKAFVDVDLLLGEIDPDQVDITYEGRQKMTTLSSCFA
QLCHKAQTVFQLNHKLEAQLVDQRSELTEVKAEREVMEREVHDQLLQLHSLQLQLYAKQG
QAEDSDTIKDRLVADTSKGSHCFVPGE

>Tetraodon nigroviridis_GOPC(ENSTNIP_10059)
LHQWAPGPGSLGPGTSSLPGQSSGSGMSMFRWLEVLEKEFDKAFDVDLLLGEIDPDQVDI
TYEGRQKMTTLSSCFAQLCHKAQTVFQLNHKLEAQLVDQRSELTEVKAEREVLEREVHDQ
LLQLHSLQLQLYAKEGQAEDSDTIKDRLPAPTLEQMEHELETKKKKHLSEAKLEMEVRLY
KKETEALRRHIAVLQAEVYGARLAAKYLDKELAGRVQQIQLLGRDMKGPAHDKLWNQLEA

EIHLHRHKTVIRACRGRNDAKKPLPSPADHVPEMLKKTQGVGPIRKVMLVKDDHEGLGIS
ITGGKEHGVPILISEIHVSQPAERCGGLHVGDAILAVNGVNLRDAKHKEAVTILSQQQGQ
IEFEVVYVAPELDSDDENVEYEDDNGHPYRLYLEELDPSSSAAASNSAA

>Gasterosteus aculeatus_GOPC(ENSGACP_7682)
MSASAGCSPVAQSSGLGPGMSMFRWLEVLEKEFDKAFVDVDLLLGEIDPDQVDITYEGRQ
KMTSLSSCFAQLCHKTQTVFQLNHKLEAQLVDLRSELTEAKAERKVIEREVHDQLLQLHA
LQLQLHPKQGQAEDSDSIRDRLPAPSLEKMEQELKVSKKEKFEDARLEAEVRLFKKENEA
LRRHMAVLQAEVYGARLAAKYLDKELAGRVQQIQLLGRDMKGPAHDKLWNQLEAEIHLHR
HKTVIRACRGRNDPKKPLLSPVGHDPDMLKKSQGVGPIRKVVLVKDDHEGLGISITGGKE
HGVPILISEIHPSQPADRCGGLHVGDAILAVNSINLRDAKHKEAVTILSQQQGQIEFEVV
YVAPEVDSDDENVEYEDDSGHRYRLYLDEVDDCSTAPPSSGSAALHALEKMSLSNGAENR
DTGISSETTAEETPSKPPETDCSS

>Oryzias latipes_GOPC(ENSORLP_19672)
CSAPSSSDMSSSAGCSPSGQSSGLGPALSMFRWLEVLEKEFDKAFVDVDLLLGEIDPDQV
DITYEGRQKMTSLSSCFAQLCHKSQTVFQLNHKLEAQLVDLRSELTEAKAERVVAEREVH
ELLLQLHALQLQLRAQQGQTEDSDTIKDRLPAPTLEVMEQELMAGKKEKLAEARLEKEVH
LFRKENEALRRHIAVLQAEVYGARLAAKYLDKELAGRVQQIQLLGRDMKGPAHDKLWNQL
EAEIHLHRHKTVIRACRGRIDPKKPLPSPVGHEHDLLKKTQGVGPIRKVALVKEDHEGLG
ISITGGKEHGVPILISEIHPGQPADRCRGLHVGDAILAVNSINLRDAKHKEAVTILSQQR
GQIEFEVVYVAPEVDSDDENVEYEDDSGHRYRLYLDELEDSSSGGGAPPNNNAASLQALE
NMTLNSGAENGNPGSCCESRPDEEGAPKPPESDASS

>Branchiostoma floridae_(XP_002594112)
MNEKSLVRPGSAPPPSGMATWSRDARVPAHGTPVGANMALTAAASAMTRSGLLEVYVRDRWCRVLVSLEE
ETLTISLEDSSVGGGDQNGDAIRNSEFDHDQLPESIANHKRVVKVIKQDVGGLGISIKGGKENKMPILIS
KIFKGLAADQTESLYVGDAILSVNGEDLRDATHDEAVRALKRAGKEVVLEVKYLREVTPYFRRSTVIGEV
GWENASPYVIKEPGADNKASTNNFTEMKTIPLKMCYVTRNLTMPDPEQRTFELHSPDGRSSCILRCQDAS
SASQWFAAIHANTSMLTASAMAEANSKLVSGRGSAGDREIKHMGWLCEQAHGEGGSSSWKPVFAALTPTD
LLLYDTVPWSREEWAVPVQSHPLLATRCSAYFDSRVGHTGTTPSRSPSGGASGRDLAFGTRTGSRVETQR
DLAAWTRLIVQLCHQAAEDMKEVIQACFWQGRECKLMVHYESGFTLMDNSNPNESQSDVLWNFPFERMRM
SADDGVGRLWLDFGGAEGEQQLDMCTGPKPIVFIIHNFLSAKVTRMGLFA

>Drosophila melanogaster_(FBpp0302599)
MVESSSLGRTPPAAQQPIPQQHSPSPSGLRCGNMETRVRGAWYRVLVTLETDFLAVSLDE
SCEAAQPSNDGQSTTLNGTLGSNHSGGGGGGAGGGGTGGGGQNGTLPNSASLQGMNIQDT
ELDGSASNDNGDRDPCLNNNNNAGDGGGMDMCDVPDHVANQKRHVRIIKSDNNGLGISIK
GGRENRMPILISKIFRGMAADQAKGLYVGDAILTVNGEELRDATHDEAVRALKRSGRVVD
LEVKFLREVTPYFRKASIISEVGWELQRAFLCPLGPGVPTSPPAPKTTPRADTRYIPLQL
THLARNLKYIDPENRCFELHSPDGVHSCILRAADSAEALVWFNALHSAMGTSTQRALAEA
NRALTNLIGELKHIGWLSKRMSGGGSSGSAGGGAASGGSGTSNSVVAGELPSGRSSSESS
DESDKWLPIFVAVTEREFRIYESAPWSVEAWSRPLEIYALATTRLAGAGNNSSLNGQQTT
VFCVRCGTARGVLVYWLRSETHRDMAAWARSLVQGSHQAVNYQREFSFRCLFQGRQCQLV
VHINRGFFLYDCGGFAPTTPTVATAKTQLWQFAFDKLKGSADDGARMLYLDFGEDGEIEL
DMECCPKPVVFVVHNCLSAKVHNIA

>Anopheles gambiae_(XP_320202)
GIAEICDVPDSVANQKRHVRVIKSDNNGLGISIKGGRENRMPILISKIFRGMAADNAKGLYVGDAILSVN
GEDLRDATHEEAVRALKRAGRVVDLEVKFLREVTPYFRKASIISEVGWELQRAFLCPLGPNASSPPGVAP
RSPPRADTRYIPLQLTHLARNLKYHDPENRCIELHSPDGIHSCILRAMDPQEATTWFNALHSAIGKSTQK
ALLDANRALVSIIGELKHIGWLSRRSGGGEYTHTHTHRSTAETGPNSQALAALVRLNGRSSSESSDELDK
WQSIFVAVTDRELRLYESAPWSVEAWSRPFESCPLVATRLAGAGNTSTVGSNSSTHSSVFCIRCGTTRGV
VSHWLRSETNRDMAAWARVLVQGCHNAIICQREFSFRCLFQGRPCQLIVHLDRGFTLLDSGLGPASKALW
TFPFDKLKGSADDGNKLLYLDFSGTEDGAELELDMECCPKPVVFVLHNCLSAKVHSLA

>Nematostella vectensis_(XP_001638712)
MAVAVSMFRWLDLLEKEFDKAFVDLDLLLGEIDPDQSEITYDGRQKMTQLSSAFAQLVHKSQTIFQSNAK
LEAELVALRHDVVEEQATKRVLEKEVNNLLLQLHAVQLQLHTTSGTHLESDNIRSKLLNDHSPFHIELEI
SKYKADAMKEAKLDCQVRQLEKENAALRKHIFSLQGEVYGARLAAKYLDKELAGRIQQIQLLGRDMRGAE
HDKLWNQIEAEIHLHRHKTVIRACRGRKDPRNKPPQPPPPEPTENDEDADSISRKRRGIGEPRTVIVNKD

KTEGLGISITGGKEHGVPILISEIHDGMPAARCGGLYVGDAILAVNGIDLQDAKHNDAVKILSSIHGEIT
MEVLYVAPDDSSDEEEDAWEEDDTRR

>Hydra magnipapillata_(XP_002167025)
MSLAVSMFRWLDMLEKEFDKAFVELDSILGYIDYDDGNFIDDGRKKLAILSSVFSQLSHKSQTIFENNAK
YEAELIGLRTELSEAKATVTILEKEVKNSLLQLHSVQLQLHSKGENSADLQIIKTKLENDLTQFRQNAIK
ESQVTEEIKIIKKENDALRKYITQIQGEVCGARLAAKYLEKELAGRIQQIQLLGRNLKLQEHEMLWNQIE
SEIFLHRHKTVIRACKKNPPKKSSSSSHINKEKQERVNIAKHSGDKIVVKFYREKSEGLGISITGGKEHG
IPVIISEIHEGMLASRCEDLQTGDAILAVNNINLENVSHEEAVSILSALSGNVTMEVWRVIDTSSEDEEW
ETNYNSRYSTVGLLDDPNEISLDSDINMTNSPAPNSNKKNVNISSLRSSLAELKLAQSLRKINKDMSPAD
EASPQPTHI

Sorting Nexin Family-SNX

>Homo sapiens_SNX27(ENSP_357836)
MADEDGEGIHPSAPHRNGGGGGGGGSGLHCAGNGGGGGGGPRVVRIVKSESGYGFNVRGQ
VSEGGQLRSINGELYAPLQHVSAVLPGGAADRAGVRKGDRILEVNHVNVEGATHKQVVDL
IRAGEKELILTVLSVPPHEADNLDPSDDSLGQSFYDYTEKQAVPISVPRYKHVEQNGEKF
VVYNVYMAGRQLCSKRYREFAILHQNLKREFANFTFPRLPGKWPFSLSEQQLDARRRGLE
EYLEKVCSIRVIGESDIMQEFLSESDENYNGVSDVELRVALPDGTTVTVRVKKNSTTDQV
YQAIAAKVGMDSTTVNYFALFEVISHSFVRKLAPNEFPHKLYIQNYTSAVPGTCLTIRKW
LFTTEEEILLNDNDLAVTYFFHQAVDDVKKGYIKAEEKSYQLQKLYEQRKMVMYLNMLRT
CEGYNEIIFPHCACDSRRKGHVITAISITHFKLHACTEEGQLENQVIAFEWDEMQRWDTD
EEGMAFCFEYARGEKKPRWVKIFTPYFNYMHECFERVFCELKWRKEEY

>Pan troglodytes_SNX27(ENSPTRP_2202)
MADEDGEGIHPSAPHRNGGGGGGGGSGLHCAGNGGGGGGGPRVVRIVKSESGYGFNVRGQ
VSEGGQLRSINGELYAPLQHVSAVLPGGAADRAGVRKGDRILEVNHVNVEGATHKQVVDL
IRAGEKELILTVLSVPPHEADNLDPSDDSLGQSFYDYTEKQAVPISVPRYKHVEQNGEKF
VVYNVYMAGRQLCSKRYREFAILHQNLKREFANFTFPRLPGKWPFSLSEQQLDARRRGLE
EYLEKVCSIRVIGESDIMQEFLSESDENYNGVSDVELRVALPDGTTVTVRVKKNSTTDQV
YQAIAAKVGMDSTTVNYFALFEVISHSFVRKLAPNEFPHKLYIQNYTSAVPGTCLTIRKW
LFTTEEEILLNDNDLAVTYFFHQAVDDVKKGYIKAEEKSYQLQKLYEQRKMVMYLNMLRT
CEGYNEIIFPHCACDSRRKGHVITAISITHFKLHACTEEGQLENQVIAFEWDEMQRWDTD
EEGMAFCFEYARGEKKPRWVKIFTPYFNYMHECFERVFCELKWRKEEY

>Mus musculus_SNX27(ENSMUSP_29783)
MADEDGEGIHPSAPHRNGGGGGGSGLHCAGNGGGGGGGPRVVRIVKSESGYGFNVRGQVS
EGGQLRSINGELYAPLQHVSAVLPGGAADRAGVRKGDRILEVNGVNVEGATHKQVVDLIR
AGEKELILTVLSVPPHEADNLDPSDDSLGQSFYDYTEKQAVPISVPTYKHVEQNGEKFVV
YNVYMAGRQLCSKRYREFAILHQNLKREFANFTFPRLPGKWPFSLSEQQLDARRRGLEEY
LEKVCSIRVIGESDIMQEFLSESDENYNGVSDVELRVALPDGTTVTVRVKKNSTTDQVYQ
AIAAKVGMDSTTVNYFALFEVINHSFVRKLAPNEFPHKLYVQNYTSAVPGTCLTIRKWLF
TTEEEVLLNDNDLAVTYFFHQAVDDVKKGYIKAEEKSYQLQKLHEQRKMVMYLNMLRTCE
GYNEIIFPHCACDSRRKGHVITAISITHFKLHACTEEGQLENQVIAFEWDEMQRWDTDEE
GMAFCFEYARGEKKPRWVKIFTPYFNYMHECFERVFCELKWRKEEY

>Felis catus_SNX27(ENSFCAP_18486)
NGVNVEGATHKQVVDLIRAGEKELILTVLSVPPHEADNLDPSEDSLGQSFYDYTEKQAVP
ISVPTYKHVEQNGEKFVVYNVYMAGRQLCSKRYREFAILHQNLKREFANFTFPRLPGKWP
FSLSEQQLDARRRGLEEYLEKVCSIRVIGESDIMQEFLSESDENYNGVSDVELRVALPDG
TTVTVRVKKNSTTDQVYQAIAAKVGMDSTTVNYFALFEVINHSFVRKLAPNEFPHKLYVQ
NYTSAVPGTCLTIRKWLFTTEEEILLNDNDLAVTYFFHQAVDDVKKGYIKAEEKSYQLQK
LYEQRKMVMYLNMLRTCEGYNEIIFPHCACDSRRKGHVITAISITHFKLHACTEEGQLEN
QVIAFEWDEMQRWDTDEEGMAFCFEYARGEKKPRWVKIFTPYFNYMHECFERVFCELKWR
KEEVVAEVEASEVESFSHASVCLSEAGLQKNIFRMARSQQRDVAT

>Bos taurus_SNX27(ENSBTAP_33754)
MADEDGEGIHPAAPHRNGGGGGGGGSGLHCAGNGGGGGGGPRVVRIVKSESGYGFNVRGQ
VSEGGQLRSINGELYAPLQHVSAVLPGGAADRAGVRKGDRILEVNGVNVEGATHKQVVDL
IRAGEKELILTVLSVPPHEADNLDPSDDSLGQSFYDYTEKQAVPISVPTYKHVEQNGEKF

VVYNVYMAGRQLCSKRYREFAILHQNLKREFANFTFPRLPGKWPFSLSEQQLDARRRGLE
EYLEKVCSIRVIGESDIMQEFLSESDENYNGVSDVELRVALPDGTTVTVRVKKNSTTDQV
YQAIAAKVGMDSTTVNYFALFEVINHSFVRKLAPNEFPHKLYVQNYTSAVPGTCLTIRKW
LFTTEEEILLNDNDLAVTYFFHQAVDDVKKGYIKAEEKSYQLQKLYEQRKMVMYLNMLRT
CEGYNEIIFPHCACDSRRKGHVITAISITHFKLHACTEEGQLENQVIAFEWDEMQRWDTD
EEGMAFCFEYARGEKKPRWVKIFTPYFNYMHECFERVFCELKWRKENIFQMARSQQRDVA
T

>Monodelphis domestica_SNX27(ENSMODP_23571)
MADEDGEGIHPSAPQRNGGGGGGGGSGLLGAGNGGGGGPRVVRIVKSESGYGFNVRGQVS
EGGQLRSINGELYAPLQHVSAVLPGGAADRAGVRKGDRILEVNGVNVEGATHKQVVDLIR
AGEKELILTVLSVPPHEADNLDPSDDSLGQSFYDYTEKQAVPISVPTYKHVEQNGEKFVV
YNVYMAGRQLCSKRYREFAILHQNLKREFANFTFPRLPGKWPFSLSEQQLDARRRGLEEY
LEKVCSIRVIGESDIMQEFLSESDENYNGVSDVELRVALPDATTVTVRVKKNSTTDQVYQ
AIAAKVGMDSTTVNYFALFEVINHSFVRKLAPNEFPHKLYVQNYTSAVPGTCLTVRKWLF
TTEEEALLNDNDLAVTYFFHQAVDDVKKGYIKAEEKSYQLQKLCEQRKMVMYLNMLRTCE
GYNEIIFPHCSCDSRRKGHVITAISITHFKLHACTEEGQLENQVIAFEWDEMQRWDTDEE
GMAFCFEYARGEKKPRWVKIFTPYFNYMHECFERVFCELKWRKENIFQMARSQQRDMAT

>Gallus gallus_SNX27(ENSGALP_1411)
MADEEGEGPPRSAAAPQRNGGDEGAPGGPRVVRIVKSESGYGFNVRGQVSEGGQLRSING
ELYAPLQHVSAVLGGGAADRAGVRKGDRILEVNGVNVEGATHKQVVDLIRAGEKELILTV
LSVPPHEADNLDPSDDSLGQSFYDYTEKQAVPISIPTYKHVEQNGEKFVVYNVYMAGRQL
CSKRYREFAILHQNLKREFANFTFPRLPGKWPFSLSEQQLDARRRGLEEYLEKVCSIRVI
GESDIMQEFLSESDENYNGVSDVELRVALPDITTVTVRVKKNSTTDQVYQAVAAKVGMDS
VTANYFALFEVINHSFVRKLAPNEFPHKLYVQNYTSAVPGTCLTIRKWLFTTEEEALLND
NDLAVTYFFHQAVDDVKKGYIKAEEKSYQLQKLCEQRKMVMYLNMLRTCEGYNEIIFPHC
SCDSRRKGHVITAISIKHFKLHACTEEGQLENQVIAFEWDEMQRWDTDEEGMAFCFEYAR
GEKKPRWVKIFTPYFNYMHECFERVFCELKWRKEVEEEATDKDNQNCSTDTVCSKNIFRT
VRSQQRDAAT

>Anolis carolinensis_SNX27(ENSACAP_8596)
LLSRNGVNVEGATHKQVVDLIRAGEKELVLTVLSVPPHEADNLDPSDDSLGQSFYDYTEK
QAVPISIPTYKHVEQNGEKFVVYNVYMAGRQLCSKRYREFAILHQNLKREFANFTFPRLP
GKWPFSLSEQQLDARRRGLEEYLEKGSTVCSIRVIGESDIMQEFLSESDENYNGVSDVEL
RVALPDITTVTVRVKKNSTTDQVYQAVAAKVGMDSTTANYFALFEVINHSFVRKLAPNEF
PHKLYVQNYTSAVPGTCLTIRKWLFTTEEEVLLNDNDLAVTYFFHQAVDDVKKGYIKAEE
KSYQLQKLCEQRKMVMVRGVLFSDGGLRKKEASGCKVSWRHKGPLLPTCNNYKLRGCDEE
KKIENQVIAFEWDEMQRWDTDEEGMAFCFEYARGEKKPRWVKIFTPYFNYMHECFERVFC
ELKWRKENIFQVMRTQHRDITT

>Danio rerio_SNX27(ENSDARP_11221)
MADVVEGDGTRSAAPSAIHSAARNGSGMSAGTCAGGLVATTVTSGPRLVRIVKSDSGYGF
NVRGQVSEGGQLRSINGELYAPLQHVSAVLPGGAADRAGISKGDRILEVNGVNVEGATHK
QVVDLIRAGEKELVLAVLSVPQPETDSLDPGDDGSSQSCYDYSDKQAVPISVPTYKHVEQ
SGEKFVVYNVYMAGRQLCSKRYREFAILHQNLKREFANFAFPKLPGKWPFSLSEQQLDAR
RRGLEEYLEKVCSVRVIGESDIMQEFLSESDENYNGVSDVELRIAMPDKTTVTVRVRKNC
TTDQVYQAVVTKIGMDSITASYFALFEVINHTFARKLAPNEFPHKLYVQNYTSAVPGTCL
TLRKWLFTTEEEILLNDNELAINYCFHQALDDVKKGFIKVGEKSYQLQKLTEQRKMTMYL
SILRTCEGYNEITFPHCSCDSRRKGHVITAISIRHFKLHACTEDGTLENQVIAFDWSEMQ
RWDTDEEGMAFCFEYARGEKKPRWVKIFTPYFNYMHECFERVFCELKWRKEVEEEATDKD
NKNCSKDEYLPAVETQKGWRHMNEEIISS

>Takifugu rubripes_SNX27a(ENSTRUP_18906)
MADVEGQGICSLPPPLPHPSTLTCSSSGNVTPGTGSSCQTATTVTSGPRLVRIVKSDSGY
GFNVRGQVSEGGQLRSINGELYAPLQHVSAVLPGGAADRAGISKGDRILEVNGVNVEGAT
HKQVVDLIRAGERELVLAVLSVPPQEADCLDPGDDVSAQSCYDYSDKQAVPISVPSYKHT
ELNQEKFVVYNVYMAGRQLCSKRYREFVILHQNLKREFDNFTFPKLPGKWPFSLSEQQLD
ARRRGLEEYLEKVCSVRVIGESDIMQEFLSDSDENYNGVSDVELRIAMPDKTTLNVRVRK
NATTDQVYQAVVMKLGMDSVTSSYFALFEVINHTFVRKLAPNEFPHKLYVQNYTSAVPGT
CLTMRKWLFTTEEEVLLNDNQLAVSYFFHQAVEDVKKGSVKSEQKFYQLQKLAEQKKMAA

YLSLLRGCEGYNEIIFPHCSCDSRRKGHVIAAISIHHFKLHACTEEGTLENQVIAFDWAE
MQRWDTDEEGMAFCFEYARGEKKPRWVKIFTPYFNYMHECFERVFCELKWRKENIFQLLR
HRRDGST

>Takifugu rubripes_SNX27b(ENSTRUP_18905)
MADVEGQGICSLPPPLPHPSTLTCSSSGNVTPGTGSSCQTATTVTSGPRLVRIVKSDSGY
GFNVRGQVSEGGQLRSINGELYAPLQHVSAVLPGGAADRAGISKGDRILEVNGVNVEGAT
HKQVVDLIRAGERELVLAVLSVPPQEADCLDPGDDVSAQSCYDYSDKQAVPISVPSYKHT
ELNQEKFVVYNVYMAGRQLCSKRYREFVILHQNLKREFDNFTFPKLPGKWPFSLSEQQLD
ARRRGLEEYLEKVCSVRVIGESDIMQEFLSDSDENYNGVSDVELRIAMPDKTTLNVRVRK
NATTDQVYQAVVMKLGMDSVTSSYFALFEVINHTFVRKLAPNEFPHKLYVQNYTSAVPGT
CLTMRKWLFTTEEEVLLNDNQLAVSYFFHQAVEDVKKGSVKSEQKFYQLQKLAEQKKMAA
YLSLLRGCEGYNEIIFPHCSCDSRRKGHVIAAISIHHFKLHACTEEGTLENQVIAFDWAE
MQRWDTDEEGMAFCFEYARGEKKPRWVKIFTPYFNYMHECFERVFCELKWRKEIEEEATD
KDNKNCSKDEYFPTPEAQKGWKHLGREIATS

>Tetraodon nigroviridis_SNX27a(ENSTNIP_21490)
MTDVDGQGTCSPPSPLPHPSTLTCSSSGNVTPGTGSSSCQTATTVTSGPRLVRIVKSDSG
YGFNVRGQVSEGGQLRSINGELYAPLQHVSAVLPGGAADRAGISKGDRILEVNGVNVEGA
THKQVVDLIRAGERELVLAVLSVPPQEADCLDPGDDVSAQSCYDYSDKQAVPISVPSYKH
TELNQEKFVVYNVYMAGRQLCSKRYREFVILHQNLKREFDNFAFPKLPGKWPFSLSEQQL
DARRRGLEEYLEKVCSVRVIGESDIMQEFLSDSDENYNGVSDVELRIALPDKTALTVRVR
KNATTDQVYQAVVTKLGMDSVTSSYFALFEVINHTFVRKLAPNEFPHKLYVQNYTSAIPG
TCLTMRKWLFTTEEEVLLNDNQLAVSYFFHQAVDDVKKGSVKSEQKSYQLQKLVEQKKMA
AYLSLLRGCEGYNEIVFPHCSCDSRRKGHIIAAISIHHFKLHACTEEGTLENQVIAFDWA
EMQRWDTDEEGMAFCFEYARGEKKPRWVKIFTPYFNYMHECFERVFCELKWRKEIEEEAT
DKDNKNCSKDEYFPAAEAQKGWRHLGQEIITS

>Tetraodon nigroviridis_SNX27b(ENSTNIP_21489)
MTDVDGQGTCSPPSPLPHPSTLTCSSSGNVTPGTGSSSCQTATTVTSGPRLVRIVKSDSG
YGFNVRGQVSEGGQLRSINGELYAPLQHVSAVLPGGAADRAGISKGDRILEVNGVNVEGA
THKQVVDLIRAGERELVLAVLSVPPQEADCLDPGDDVSAQSCYDYSDKQAVPISVPSYKH
TELNQEKFVVYNVYMAGRQLCSKRYREFVILHQNLKREFDNFAFPKLPGKWPFSLSEQQL
DARRRGLEEYLEKVCSVRVIGESDIMQEFLSDSDENYNGVSDVELRIALPDKTALTVRVR
KNATTDQVYQAVVTKLGMDSVTSSYFALFEVINHTFVRKLAPNEFPHKLYVQNYTSAIPG
TCLTMRKWLFTTEEEVLLNDNQLAVSYFFHQAVDDVKKGSVKSEQKSYQLQKLVEQKKMA
AYLSLLRGCEGYNEIVFPHCSCDSRRKGHIIAAISIHHFKLHACTEEGTLENQVIAFDWA
EMQRWDTDEEGMAFCFEYARGEKKPRWVKIFTPYFNYMHECFERVFCELKWRKENIFQLL
RHRRDGGT

>Gasterosteus aculeatus_SNX27(ENSGACP_15830)
MADVEGQGICSTVPELPHPSSRNGSGSGNVTPGGTGGSCQTATTVTSGPRLVRVVKSDSG
YGFNVRGQVSEGGQLRSINGELYPPLQHVSAVLPGGAADRAGISKGDRILEVNGVNVEGA
THKQVVDLIRAGERELALAVLSVPPQETDCLDPGDDISTQSCYDYSDKQAVPISVPSYKH
TELNQEKFVVYNVYMAGRQLCSKRYREFVILHQNLKREFAIFTFPKLPGKWPFSLSEQQL
DARRRGLEEYLEKVCSVRIIGESDIMQEFLSESDENYNGVSDVELRIAMPDKSPLTVRVC
KNSTTDQVYQAVVMKLGMDSVTASYFALFEVINHTFVRKLAPNEFPHKLYVQNYTSAIAG
TCLTLRKWLFTTEEEILLNDNQLAVNYFFHQAVDDVKKGFIKAEQKSYQLQKLAEQKKLS
MYMSLLRDCEGYNEIIFPHCSCDSRRKGHVITAISIRHFKLHACTEEGTLENQVIAFDWG
EMQRWDTDEEGMAFCFEYARGEKKPRWVKIFTPYFNYMHECFERVFCELKWRKEVEEEAT
DKDNKNCSKDEYFPAAEAQKGWRHLGREIVTS

>Homo sapiens_SNX17(ENSP_233575)
MHFSIPETESRSGDSGGSAYVAYNIHVNGVLHCRVRYSQLLGLHEQLRKEYGANVLPAFP
PKKLFSLTPAEVEQRREQLEKYMQAVRQDPLLGSSETFNSFLRRAQQETQQVPTEEVSLE
VLLSNGQKVLVNVLTSDQTEDVLEAVAAKLDLPDDLIGYFSLFLVREKEDGAFSFVRKLQ
EFELPYVSVTSLRSQEYKIVLRKSYWDSAYDDDVMENRVGLNLLYAQTVSDIERGWILVT
KEQHRQLKSLQEKVSKKEFLRLAQTLRHYGYLRFDACVADFPEKDCPVVVSAGNSELSLQ
LRLPGQQLREGSFRVTRMRCWRVTSSVPLPSGSTSSPGRGRGEVRLELAFEYLMSKDRLQ
WVTITSPQAIMMSICLQSMVDELMVKKSGGSIRKMLRRRVGGTLRRSDSQQAVKSPPLLE
SPDATRESMVKLSSKLSAVSLRGIGSPSTDASASDVHGNFAFEGIGDEDL

>Pongo abelii_SNX17(ENSPPYP_14016)
MHFSIPETESRSGDSGGSAYVAYNIHVNGVLHCRVRYSQLLGLHEQLRKEYGANVLPAFP
PKKLFSLTPAEVEQRREQLEKYMQAVRQDPLLGSSETFNSFLRRAQQETQQVPTEEVSLE
VLLSNGQKVLVNVLTSDQTEDVLEAVAAKLDLPDDLIGYFSLFLVREKEDGAFSFVRKLQ
EFELPYVSVTSLRSQEYKIVLRKSYWDSAYDDDVMENRVGLNLLYAQTVSDIERGWILVT
KEQHRQLKSLQEKVSKKEFLRLAQTLRHYGYLRFDACVADFPEKDCPVVVSAGNSELSLQ
LRLPGQQLREGSFRVTRMRCWRVTSSVPLPSGSTSSPGRGRGEVRLELAFEYLMSKDRLQ
WVTITSPQAIMMSICLQSMVDELMVKKSGGSIRKMLRRRVGGTLRRSDSQQAVKSPPLLE
SPDATRESMVKLSSKLSAVSLRGIGSPSTDASASDVHGNFAFEGIGDEDL

>Callithrix jacchus_SNX17(ENSCJAP_47034)
MHFSIPETESRSGDSGGSAYVAYNIHVNGVLHCRVRYSQLLGLHEQLRKEYGANVLPAFP
PKKLFSLTPAEVEQRREQLEKYMQAVRQDPLLGSSETFNSFLRRAQQETQQVPTEEVSLE
VLLSNGQKVLVNVLTSDQTEDVLEAVAAKLDLPDDLIGYFSLFLVREKEDGAFSFVRKLQ
EFELPYVSVTSLRSQEYKIVLRKSYWDSAYDDDVMENRVGLNLLYAQTVSDIERGWILVT
KEQHRQLKSLQEKVSKKEFLRLAQTLRHYGYLRFDACVADFPEKDCPVVVSAGNSELSLQ
LRLPGQQLREGSFRVTRMRCWRVTSSVPLPSGGTSSPGRGRGEVRLELAFEYLMSKDRLQ
WVTITSPQAIMMSICLQSMVDELMVKKSGGSIRKMLRRRVGGTLRRSDSQQAVKSPPLLE
SPDATRESMVKLSSKLSAVSLRGIGSPSTDASASDVHGNFAFEGIGDEDL

>Mus musculus_SNX17(ENSMUSP_31029)
MHFSIPETESRSGDSGGSAYVAYNIHVNGVLHCRVRYSQLLGLHEQLRKEYGANVVPAFP
PKKLFSLTPAEVEQRREQLEKYMQAVRQDPLLGSSETFNSFLRRAQQETQQVPTEEVSLE
VLLSNGQKVLVTVLTSDQTEDVLEAVAAKLDLPDDLIGYFSLFLVREKEDGAFSFVRKLQ
EFELPYVSVTSLRSQEYKIVLRKSYWDSAYDDDVMENRVGLNLLYAQTVSDIEHGWILVT
KEQHRQLKSLQEKVSKKEFLRLAQTLRHYGYLRFDACVADFPEKDCPVVVSAGNSELSLQ
LRLPGQQLREGSFRVTRMRCWRVTSSVPLPSGGTSSPSRGRGEVRLELAFEYLMSKDRLQ
WVTITSPQAIMMSICLQSMVDELMVKKSGGSIRKMLRRRVGGTLRRSDSQQAVKSPPLLE
SPDASRESMVKLSSKLSAVSLRGIGSPSTDASASAVHGNFAFEGIGDEDL

>Monodelphis domestica_SNX17(ENSMODP_19547)
MHFSIPETESRSGDSGGSNYVAYNIHVNGVLHCRVRYSQLLGLHEQLRKEYGANVLPAFP
PKKLFTLTPAEVEQRREQLEKYMQAVRQDPLLGGSETFNSFLRRAQQETQQVPTEEVSLE
VLLSNGQKVLVTILTSDQTEDVLEAVAAKLDLPDELIGYFSLFLVREQEDGAFSFVRKLQ
EFELPYVSVTSLRSQEYKIVLRKSYWDSAYDDDVMENRVGLNLLYAQTVSDIEHGWILVT
KEQHRQLKSLQEKVSKKEFLRLAQTLRHYGYLRFEPCVADFPEKGCPVVVSAGNSELSLQ
LRLPGQQLREGSFRVTRMRCWRVTSSVPLPSGNTSSPNRGRGEVRLELAFEYLMSKDQLQ
WVTITSPQAIMMSICLQSMVDELMVKKSGGSIRKMLRRRAGGTLRRSDSQQAVKSPPLLD
SPDATRESVVKLSSKLSAVSLRGISSPSPDTNTSDVHGNYAFEGIGDEDL

>Erinaceus europaeus_SNX17(ENSEEUP_7993)
MHFSIPETESRSGDSGGSAYVAYNIHVNGVLHCRVRYSQLLGLHEQLRKEYGANVLPAFP
PKKLFSLTPAEVEQRREQLEKYMQAVRQDPLLGSSETFNSFLRRAQQETQQVPTEEVSLE
VLLSNGQKVLVSVLTSDQTEDVLEAVAAKLELPDDLIGYFSLFLVREKEDGAFSFVRKLQ
EFELPYVSVTSLRSQEYKIVLRKSYWDSAYDEDVMENRVGLNLLYAQTVSDIERGWILVT
KEQHRQLKSLQEKVSKKEFLRLAQTLRHYGYLRFDACVADFPEKNCPVVVSAGNSELSLQ
LRLPGQQLREGSFRVTRMRCWRVTSSVPLPSGGTSSPGRGRGEVRLELAFEYLMSKDRLQ
WVTITSPQAIMLSICLQSMVDELMVKKSGGSIRKMLRRRVGGTLRRSDSQQAVKSPPLLE
SPDASRESMVKLSSKLSAVSLRGIGSPGTDASASDVHGNFAFEGIGDEDL

>Gallus gallus_SNX17(ENSGALP_26600)
MQAVRQDPTLGGSETFNSFLRKAQQETQQIPTEEVVLEVLLSNGQKVKVTILTSDQTEDV
LEAVASKLDLPDDLVGYFSLFLVRETKDGAFSFVRKLQEFELPYVSVTSLHNPEFRIILR
KSYWDSSYDDDVMEHHVGLNLLYAQTVSDIEHGWILVNKEQHRQLKSLQEKVSKKEFIRL
AQTLKYYGYLKFDPCVTDFPEKGCHVVVSAGNNELNFQVRLPSEQIKEGSFKVTRMRCWR
VTSSVPMSNGPSGSSPGKSEVKLELAFEYLMSKDRLQWVTITSPQAIMLSICLQSMVDEL
MVKKSGGSIRKMFRRRANGALRRSDSQQAVKSPPLLDSPDASREPMAKLSSKLTSVSLRG
ISHSSSANDVGANDFHGNYAFEGIGDEDL

>Taeniopygia guttata_SNX17(ENSTGUP_15174)

AYNIHVNGVLHCRVRYSQLLGLHEQLRKEYGANVVPAFPPKKIFTLTPAEVEQRREQLEK
YMQAVRQDPTLGGSETFNSFLRKAQQETQQIPTEEVVLEVLLSNGQKVKVTILTSDQTED
VLEAVASKLDLPDDLVGYFSLFLVRETKDGAFSFVRKLQEFELPYVSVTSLHNPEFQIIL
RKSYWDSAYDDDVMEHRVGLNLLYAQTVSDIEHGWILVNKEQHRQLKSLQEKVSKKEFIR
LAQTLKYYGYLKFDPCVTDFPEKGCHVVVSAGNNELNFQVRLPNEQIKEGSFKVTRMRCW
RVTSSVPMNNGPSGSSPGKSEVKLELAFEYLMSKDRLQWVTITSPQAIMLSICLQSMVDE
LMVKKSGGSIRKMFRRRVNGALRRSDSQQAVKSPPLLDSPDASWEPMDKLSSKITSVSLR
GISHSSSANDVGANDFHGNYAFEGIGDEDL

>Takifugu rubripes_SNX17(ENSTRUP_17937)
MHFSIPETEVCSGENGSTYVAYNIHVNGVLHCRVRYSQLLSLHEQIKKEYGSNVVPAFPP
KKIFTLTPAEVEQRREQLEKYMQAVRQDPLLGSSEMFNSFLRKAQQETQQIPTEDVALDI
CLSNGQKISVNILTSDQTEDVLDAVATNLDLPEDLIGYFSLFLIRESVDGGVTFVRKLQE
FELPYVSISSLQSSDFHIVLRKSYWDTAYDGDVMDNRVGLSLLYAQTVSDIERGWILVNK
DQHRQLKSLQEKGSKKEFIHLGQTLKYYGYIKFDPCITDFPEKGCQVIVSAGNNELNFHV
KLPNEQMKEGSFKVTRMRCWRVTSSQVPLANGTTNTCSSSKCEVKLELAFEYLMSKDRLQ
WVTITSQQAIMMSICLQSMVDELMVKKSGGSIKKQMLRKRHNGSIYRADGQQAVKSPPLL
DSPNTNREQIVKLSTKLSSMSLLGISASNSANDISGNDFHGNYAFEGIGDDDL

>Tetraodon nigroviridis_SNX17(ENSTNIP_19436)
MHFSIPETEVCSGENGSSYVAYNIHVNGVLHCRVRYSQLLSLHEQIKKEYGSNVVPAFPP
KKIFTLTPAEVEQRREQLEKYMQAVRQDPLLGSSEMFNSFLRKAQQETQQIPTEEVALDI
SLSNGQKITANILTSDQTEDVLDAVAAKLDLPEDLIGYFSLFLIRELVDGGVTFVRKLQE
FELPYVSISSLQSSDFHILLRKSYWDTAYDGDVMDNRVGLNLLYAQTVSDIERGWILVNK
DQHRQLKSLQEKGSKKEFIHLGQTLKYYGYIKFDPCVTDFPEKGCQVIVSAGNNELNFHV
KLPNEQMKEGSFKVTRMRCWRVTSSQVPLANGTTSTCSSKCEVKLELAFEYLMSKDRLQW
VTITSQQAIMMSICLQSMVDELMVKKSGGSIKKMLRKRHNGSIYRADSQQAVKSPPLLDS
PDTNREQIVKLSTKLSSVSLLGISGSNSANDISGNDFHGNYAFEGIGDDDL

>Gasterosteus aculeatus_SNX17(ENSGACP_14244)
MHFSIPETEVRSGENGSSYVAYNIHVNGVLHCRVRYSQLLGLHEQIKKEYGSNVVPAFPP
KKIFTLTPAEVEQRREQLEKYMQAVRQDPLLGANEMFNSFLRKAQQETQQIPTEDVSLDI
CLSNGQKVTVNILTSDQTEDVLDAVATKLDLPDDLVGYFSLFLIREGADGGLTFVRKLQE
FELPYVSITSLRNSEFHILLRKSYWDMAYDGDVMDNRVGLNLLYAQQTVSDVERGWILVN
KDQHRQLKSLQEKGSKKEFIHLGQTLKYYGYIKFDPCVTDFPEKGCQVIVSAGSNELNFH
VKLPSEQMKEGSFKVTRMRCWRVTSCQVPTANGTTNPCSASKCEVKLELSFEYLMSKDRL
QWVTITSQQAIMMSICLQSMVDELMVKKSGGSIKKMLRKRHNSIQRSDSQQAVKSPPLLD
SPDPNREQIVKLSTKLGSVSLRGISASNSANDISGNDFHGNYAFEGIGDDDL

>Oryzias latipes_SNX17(ENSORLP_20418)
MHFSIPETEVRSGDNGSTYVAYNIHVNGVLHCRVRYSQLLGLHEQIKKEYGSNVVPNFPP
KKIFTLTPAEVEQRREQLEKYMQAVRQHPLLGASDLFNSFLRKAQQETQQIPTEEAALDI
CLCSGPKLTVHILTSDQTEDVLEAVGTKLDLPDDLVGYFSLFLVHEGVDGSLTFVRKLQE
FELPYVSITSLQSSEFHIVLRKSYWDMAYDQDVMDNRVGLNLLYAQTVSDIERGWILVNK
DQQRQLKSLQEKGSKKEFIHLAQTLKYYGYIKFDPCITDFPEKGCQVIVSAGNNELNFHV
KLPNEQMKEGSFKVTRMRCWRVTSSQVPVTNGTTNPCGSAKCDVKLELAFEYLMSKDRLQ
WVTITSQQAIMMSICLQSMVDELMVKKSGGSIKKVLKKRQESLHRADSQQAVKSPPLLES
PDPNREQIVKLSTKLNSVSLRGISTSNSANDISSNDFHGNYAFEGIGDDDL

>Homo sapiens_SNX31(ENSP_312368)
MKMHFCIPVSQQRSDALGGRYVLYSVHLDGFLFCRVRYSQLHGWNEQLRRVFGNCLPPFP
PKYYLAMTTAMADERRDQLEQYLQNVTMDPNVLRSDVFVEFLKLAQLNTFDIATKKAYLD
IFLPNEQSIRIEIITSDTAERVLEVVSHKIGLCRELLGYFGLFLIRFGKEGKLSVVKKLA
DFELPYVSLGSSEVENCKVGLRKWYMAPSLDSVLMDCRVAVDLLYMQAIQDIEKGWAKPT
QAQRQKLEAFQKEDSQTKFLELAREVRHYGYLQLDPCTCDYPESGSGAVLSVGNNEISCC
ITLPDSQTQDIVFQMSRVKCWQVTFLGTLLDTDGPQRTLNQNLELRFQYSEDSCWQWFVI
YTKQAFLLSSCLKKMISEKMVKLAAENTEMQIEVPEQSKSKKYHIQQSQQKDYSSFLSRK
SKIKIAKDDCVFGNIKEEDL

>Gorilla gorilla_SNX31(ENSGGOP_28414)
MKMHFCIPVSQQRSDALGGRYVLYSVHLDGFLFCRVRYSQLHGWNEQLRRVFGNCLPPFP

PKYYLAMTTAMADERRDQLEQYLQNVTMDPNVLRSDVFVEFLKLAQLNTFDIATKKAYLD
ILLPNEQSIRIEIITSDTAERVLEVVSHKTGLCRELLGYFGLFLIRFGKEGKLSVVKKLA
DFELPYVSLGSSEVENCKVGLRKWYMDPSLDSVLMDCRVAVDLLYMQAIQDIEKGWAKPT
QAQRQKLEAFQKEDNQTKFLELAREVRHYGYLQLDPCTCDYPESGSGAVLSVGNNEISCC
ITLPDSQTQDIVFQMSRVKCWQVTFLGTLLDTDGPQRTLNQNLELRFQYSEDSCWQWFVI
YTKQAFLLSSCLKKMISEKMVKLAAENTEMQIEVPEQSKSKKYHIQQSQQKDYSSFLSRK
SKIKIAKDDCVFGNIKEEDL

>Macaca mulatta_SNX31(ENSMMUP_13018)
MKMHFCIPVSQQRSDALGGRYVLYSVYLDGFLFCRVRYSQLHDWNEQLRRVFGNCLPPFP
PKYYLAMTTAMADERRNQLEQYLQNVTMDPNVLRSDVFVEFLKLAQLNTFDITPKKAYLD
IFLPNEESINIEIITSDTAERVLEVASHKIGLCRELLGYFGLFLIRFGKEGKLSVVKKLA
DFELPYASLGSSEVEHCKVGLRKWYMDPSLDSTLMDCRAALDLLYMQAIQDIEKEWAKPT
QAQRQKLEAFQKEDNQTKFLELAREVQHYGYLQLDPCTCDYPEPGSGVVLSVGNNEISCC
ITLPDSQTQDVIFQMSRVKCWQVTFLGTLLDMDGPQRAFNQNLELRFQYSEDSCWQWFVI
YTKQAFLLSSCLKKMISEKMVKLAAKNPEMQIEVPEQSKSKKYHIQQSQQKDYSSFLSRK
SKIKIAKDDCVFGNIKEEDL

>Mus musculus_SNX31(ENSMUSP_124063)
MKMHFCIPVSQQRPDALGGRYVLYSVYLDGFLFCKVRYSQLHRWDEQLRRVFGNCLPPFP
PKYYLAMTTAMAEERRDQLERYLQNVTADPRVTRSDVFTEFLTLVQLHTLNITIQNVELA
VFLPDGRSIKVEGLTSDTAERVLEVMAHKLGLQPDLVGYFGLFLIQCFPEGKLSVVKKLA
DFELPYTSLQSSEMENCKIGLRKWYLDPALDSMLMDCRAAGDLLYMQAVQDIEKEWMKPT
QAQREELKALQKKENQTKFLELSQEVRHYGYVQLDPCTCNHPEPGCGAQLSIGNNEISCC
ITLPNGQIQDIAFQMSRVKCWQVTFLGTLLDTDGPQRTLNQNLELRFQYSEDSCQQWFVI
YTKQAFFLSSCLKKMISERMTKLTEQSPEMQIEVPEQGRSKKHPSQPSQQKVYFNFLRKG
KMKRSEGDYVWDTLMEEGL

>Rattus norvegicus_SNX31(ENSRNOP_37890)
MKMHFCIPVSQQQPDTLGGRYVLYSVYLDGFLFCKVRYSQLHRWDEQLRRVFGNCLPPFP
PKYYLAMTTTMVEERRGQLECYLQNVTADPRVTRSDVFTEFLTLVQLQTLNITTKNVVLA
VFLPDGRSINVGSLTSDTAERVLEVVAHKLGLHPELVGYFGLFLIQCFPEGKLSVVKKLA
DFELPYTSLQSSEVENCKIGLRKWYLDPAMDSVLMDCRAAGDLLYMQAVQDVEIEWMKPT
QAQREELKALQKQENQTKFLELSQKVQHYGNIQLDPCTCNHPEPGCGVLLSFGNNEISCH
ITLPDGQTQDIVFQMSRVRCWQVTSLGTLLDTDGPQRTLNQNLELRFQYSEDSHQQWFVI
YSKQAFFLSSCLKKMVSEKMAKLTEESPEMQIEVPDQGRSKKHPGQPSQQKDYFNFLRKG
KMKKSEGDYVWGTLTEEGL

>Canis familiaris_SNX31(ENSCAFP_797)
MKMHFCIPVSQQRLDRLGGRYVLYSVYLDGYLFCRVRYSQLHRWNEQLRRVFGNCLPPFP
PKYYLAMTASMANERRDQLEQYLQNVTVDPNMLRSDVFVDFLKLVQLDTFSITTKTASLD
IFLPDGRNIQVEILTSDTAERVLEVVSHKIGLCQELLGYFGLFLIQCCKEGKFSVMKKLV
DFELPYVSLQSAKVEDCKVGLRKWYMDPSLDSVLMDSAAAVALIYMQAVQDMEKEWAKPT
QVQRQKLEALQKEDNQTKFLELSQEVRHYGFLQLDPCTCDYPEPGCRAVLSVGNNEISCC
ITLPGNQTQEIIFQMNRVKCWQVTFLGSLLDMDGPQRTLNQNLELRFQYSEDSRWQWFVI
YTKQAFLLSSCLKKMISEKMVKLAAENPEMQIEVPEQGKSKKIPHSTKPEQKDSCFLPRN
KIEIIKVDCVFGTIMEEDL

>Felis catus_SNX31(ENSFCAP_1733)
MKMHFCIPVSQQRPDALGGRYVLYSVYLDGYLFCRVRYSQLHRWNEQLRRVFGNSLPPFP
PKYYLAMTASMADERRDQLEQYLQKVTMDPNMLRSDVFIDFLKLVQLDTFSITMKTAALD
IFLPDGRNIQIEILTSDTAERVLEVVSHKIGLCQELLGYFGLFLIRFRKEGKLSVMKKLA
DFELPYVSLRSAGMENCKVGLRKWYMDPSLDSMLMDCTAAVDLIHMQALQDIEQEWAKPT
QAQRQKLEALQKEDNQTKFLELSQEVRHYGYLQLDPCTCDYPEPGCGAVLSVGNDEISCC
ITLPDNQTQEIIFQMSRVKCWKVTFLGTLLDVDGPQRTLNQNLELRFQYSEDSRWQWFVI
YTKQAFLLSSCLKKMISEKTVKLAAENPEMQIEVPEQGKSKKCHIQQSQQKESSRFPPRK
SKIEIAKADCVFGTIKEEDL

>Myotis lucifugus_SNX31(ENSMLUP_13398)
MKMHFCIPVSQQRPDALGGRYTLYSVYLDGLLFCRVRYSQLHRWNEQLRRVFGNCLPPFP
PKYYLAMTTSMANERRDQLEHYLQNVTVDPNMLRSDVFVEFLKRVQLDTFSITTKKAALD

VLLPDGRSMEVEILTSDTAERVLEVVSHKIGLCRELLGYFGLFLIRFCKEGKLSVMKKLA
DFELPYVSLRSSELENCKVGLRKWYMDPSLDSLLMDCRAAVNLIYMQAIQDIQEGWAKPT
QAQRQELEALQEEGNQTKFLELSREVRHYGCVQLDPCTCDYPEPGCGAVLSVGNNEISCC
VTLPGSQTQQVVFPMSRVKCWQVTFLGTLLDMDGPQRTLNQNLELRFQYSEESRWQWFVL
YTKQAFLLSSCLKKMISEKMAKRAAENPEMQIEVPEQGKSKKYHIQQSQQKDYSSFLPRT
SKIKTAEGDCVSRTPEEE

>Monodelphis domestica_SNX31(ENSMODP_7764)
MQRQPLAPRSWVLHGVQEKRRNTSPSLTPPQPSPQSTMKMHFSIPVSQQRLDTLGGRYVL
YSVYLEGFLFCKVRYSQLHRWNEQLKRVFGNNLPPFPPKYYLAMTTSMADERRDQEQYLQ
NVPMDQNILRSEVFIRFFKLLQQDTFKITTKKVSLDVFLPDGRSIRIEILTSDTAERVLE
IVSYKMGLSRELIGYFSLFLIQFQSDNVLSVMKKMAEFELPYVSLQSTEVENCKVGLRKW
YMDPSLDSMLMDCQTAINLLYEQAVQEIEKKWAKPTEEQRQELEALRKAGNQTKFLELAQ
KVQHYGYMQLDSCACDYPEPGCVATLAVGNNEISCYFKLPSNQTQEVNFKMSRIKSWQVT
FLGTLLEEEESPQQTLNQNLELRFQYSEGNNSWQWFTIYSKQAFLLSSCLKKMITEKMMK
LAAENPEMQIEVPLEQGKRKKSYHQQSQTNSFDFPLRKGKISFKSTEEDCVFETIREEDL

>Anolis carolinensis_SNX31(ENSACAP_16180)
MKMHFSIPLSEELLEKFGGRYVLYSVYLEGFLFCKVRYSQLHRWNEQLRRVFGRGIPSFP
PKFYLAMTKSMADERRANLEQYLQNVSIDPSITNSEVFISFFRKLQQDTFKIQTQRAFLD
VYLPDGRYLRIDVQTSDTAERVLEVVSYKIELSRELIGYFSLFLIQDHNNGELSVLKKLA
EFELPYVSLRSMKEPSCKIGIRKWYMDPSLDKMLVDCRTAVNLLYMQVVQEIEKKWLRPS
DGQMQKLQIFQKAPNKMKFLELVQEVQHYGYIQLDPCSCDYPEVGCTAAIHVGNNEISCC
IKLPSNQIKEVSFKINRVRCWQVTFLGSVTGQGLDQFLELRFEYNDSDTWRWIVFNTKQA
FLLSSCLKKIISEQLMTATKSDQEMQIELPQPDKAKKASIHPSQIFHSGFKSRKKITTSR
SNEDDCVFEKISEEDL

>Xenopus tropicalis_SNX31(ENSXETP_38721)
MHICIPVTEELQDTLGCRFVLYSVYLEGFLLCKVRYKDLHLWNEQIYRVFGNRLPKFPPK
YYLAMTKAMTNERRLQLEQYLQQIVSDPVVTSSEIFMEYFKKLQMDTFNMPTVQLILRIY
MPDGAAVELDVQTSDSAERVLEAALFKLGVSRELGEYFSLFITHKEAKGPFTVVKRIAGF
ELPFLTIWNLEDDQFQIEVRKWYMNPSNDAMLMGSTEAIDLLYLQAVQEFQMEWTRPTKD
QQQKLQHCLKQQNKLKFLELMKTVEYYGYLHITSCTSDYPECDSEVTIWVGNNEMSCHFY
SPGGHAENLRLSIKDLICWNVTLLPKKQEVMSPNHQHLELKFDYQQGSSLKCITIHTEQA
FLLSSCLKKMLSERPVHRCKEELEIQVDKATCKSNIRPEQNGVHAKKLKDKREYCVVDNI
SDLDL

>Branchiostoma floridae_(XP_002609888)
MAESGGEEDLPSPEQVSTKPRVVTIYKTDTGFGFNVRGQVSEGGQLRSINGQLYAPLQHVSAVLEGGAAH
KAGIQRGDRILEVNNENVEGATHKRVVDLIKSGGDSLTLKVVSVSRKEAARLDAQGDDNSASYACVDYSE
KRALPISVPDYRTVETSAEKFVVYNIYMAGRQLCARRYREFSNLHQTLKREFPEFPFPKMPGKWPFTLSE
QQLDARRRGLEQYLEKVCSIRVIAESDVMQEFLADITEKDVNSGTAEVDLRVLLPDRSTLTVTIRRNNNT
DQVYDAVVDKLGLDEELLPYFGLFEIMDTNFERKLVPTEFPHNLYIQNYTTAASTCIALRKWLFDLQREL
QLTHLDMANTFFYWQTLKREFPEFPFPKMPGKWPFSLSEQQLDARRRGLEQYLEKVCSIRVIAESDVMQE
FLADITEKDRL

>Saccoglossus kowalevskii_(XP_006817773)
SQHSTLASSPLFNGFFLSAQQETQRQEAEDVSVDVYLLNGHKITIKIMSTDQTDDVLETVASQINLPDDF
VYYFALYLVKKDSDGSFSIVRKMQDFESPFISLKTAGSKHKIVLRKNFWDATYDDDLLDNRVSMNLLYVQ
AVSDIDRGWTLANKEQMARLEVLKSKGSKKEYLRLAQTLKYYGFIQFKPCTSDFPVKNTKVLVSAGNREL
SFRVSDPDGQVKEGSFKVTRMRCWRITTTIAETNGESDVSNTTSELELSFEYLVAKDKLQWIAVQSDQAI
LMSICLQGMVDELIMKKEGSRMKRPQDRIRGTRTDFKKRDAPNSIKMSSSSSSHSVSSPDSPSEETASAA
QKAKESVKKISVSKNKINLFSNNTTILLCCFASKLLTLIHAELGSLISSANHERQLNFLYIHMSIPVK

>Strongylocentrotus purpuratus_(XP_011660429)
MHFSIPDTQECKDEQGSSYRVYNVHVNGVFHCSVRYSQLHDFNEQVKKEFSNSFTGYQKFPPKKFLSLTP
SQREERREKLEKYIQIVSQDPRIANSDIFNGFLLTSQKETRQEEPVDVQLEVYMMNGSRVKVDIKSTDQT
DDVLERVTSEIGLHEQFTYYFALFLVIKDKDEEAGTIIRRIQDFESPYISLKAATGTCKIVLRKNYWDPS
FDSDLLEDRVALQLLYVQTLSDVSRGWVVASEDQMARLQSLKAKNSKKEYIKFAQTLKYYGTLQFKQCTV
NYPEPETKTVINTGNKELYFRVELPDKSTKEGVFRITRMRCWRITNSADKEARRLSGAEPIVVDNSPKKK
DSQLELSFEYLVAKNKLQWVTIRSQQAILISLCLQSMVDELLLQKKGGHIKRVGFFQRENVALSSLPVAH

YGLGN

>Octopus bimaculoides_(XP_014780947)
MADSDEEGSTPSTPERMMSDHSGPRVVTITKTETGFGFNVRGQVSEGGVLKSINGVLYAPLQHVSAVLEG
GAAHRAGIRKGDRILEVNGVNVEGATHRQVVDLIKSGRDNLTLTVISVPERVAERLEPSDDSSGPSLIDY
SERRSLPISIPDYQSVENNGEKYVVFNIYMAGRHLCSRRYREFDCLHSQLKREFTDFNFPKLPGKKLFTL
SDQQLDARRRGLEQYLEKVCAVRVIGESETVQEFLAASDSHMENGGCNEVELKVSLPDHSLCIVTIRRTD
NTEKVYQAVCNKLQIDNTSSKFFYLFETVDYNFERKLQPQEFPHNIYIQNYSTATATCIALRKWVFSLAL
EMQLSYSEYVSNLLYWQAIDDVSRSLIKAGDRLYELKALQEAAKVNEYLKMARRLDSYGEIVFPHCACDS
RKEGHVMASIGIRAFRLQACKEDGSIEPQVVEIKWSDIKNYDIDDETMSFIFEYFRPSRKSRLVRIYSTY
FVYMYDSFERIFEELDWQKEPFKCGLEMNQTSAESEDSDRVIDGH

>Trichoplax adhaerens_(XP_002110491)
MSDGEETVDSPKTTNGDRHTESDAQSLSVENSKSVTIRKSQKGFGFNVRGQVSEGGQLRAINGKLYPPLQ
HVSAVIDGGSAKQAGVRVGDRILAVNGISVEGSSHQAVVDLIKQNSQVCELLLLPVTDDEAKRLDGRGRG
SAHHDYPPDYTEKNALPITIPDWTNIDRNGERYTRPYNYTCLLPNNEVILLKISLFILKPLILQIGVQCL
HDLKESTENTTFQNFPASGHLAYLNHKSIKEDAVWSPTYVKHRFERFSFCFLEVTSVCAIYDSKTIQEFL
CINDESKKTNTEPTDDNNTSIEISLPDAKTIAISIRKSDIVEDVYQIAAKKAGIGKSNMKYFAMFEHISE
NFWRKLQDNEMPYAVIYRSSSNRNLAIKKWTFSEDKEKTLVSDDTVLNYIYHEAANEIKSGKLSSQEKIN
DLKAAQNSGKKALFVELAQTLRGYNTVVFPHCASNVRKNGNVIVSISKSGLALTACTNTGELEEQEHKFN
WDSIIEWETDEDEASFNFRYNRENMPPRLVKLYTDYDMYMKECFDRVIAELEWEREQIDVKPTENVAAVT
ASEKPSAKKLTKPSALADIGDGDL

>Amphimedon queenslandica_(XP_003386341)
MRNLGYSTIKKGVASTKLEEKEMHIHIPSISDALDSKGSKFKVFDIYVNGLYHGSVRYSQLLDLHNEVKV
KYSSATIVDFPAKVFMISEAQLHERRRMLERYVHCLAQDRNIGSSDMFQDFMLSCQQISSGEEDKEVEID
VYLSNEKSKKIKFSSFDRSPHVLEATCKSIGVSEDLTHYFGLFMEYSIPGDKWKTLHCLSDFQCPYLSLC
AANRSEDYKFRLTIKKWFFSPKFLTDCLSDSTALNMIYIETIRDIDTETLTVPDSIKDKLVSLKKSGKRK
EFIETLSSQPTCLFGYNYCEGLLFNDQSTSSALAGGTKIVLKDIEKVYPISKMRCWKVGVLRGNREHFFS
FEYEYSHQKFVWVVLKGVSTILLAQSITRAVEEFLVLSKNKSIRKPSENLFSRRPERPFPSFELTLGPPA
GEDIHMAAAASDSQSPSIVRTESQSSSKDSEVSTDSEENNTSNTRADIHVTTPTVTPTTSNESPSLGGGG
GGTYVTSGSSKKTKSSGNKKSLVPNYVKSKKKDEEEIAFNPTFLSDADL

Stathmin-STMN

>Homo sapiens_STMN1(ENSP_410452)
MASSDIQVKELEKRASGQAFELILSPRSKESVPEFPLSPPKKKDLSLEEIQKKLEAAEER
RKSHEAEVLKQLAEKREHEKEVLQKAIEENNNFSKMAEEKLTHKMEANKENREAQMAAKL
ERLREKMYFWTHGPGAHPAQISAEQSCLHSVPALCPALGLQSALITWSDLSHHH

>Pan troglodytes_STMN1(ENSPTRP_653)
MASSDIQVKELEKRASGQAFELILSPRSKESVPEFPLSPPKKKDLSLEEIQKKLEAAEER
RKSHEAEVLKQLAEKREHEKEVLQKAIEENNNFSKMAEEKLTHKMEANKENREAQMAAKL
ERLREKMYFWTHGPGPHPAQISAEQSCLHSVPALCPALGLQSALITWSDLSHH

>Gorilla gorilla_STMN1(ENSGGOP_18067)
MASSDIQVKELEKRASGQAFELILSPRSKESVPEFPLSPPKKKDLSLEEIQKKLEAAEER
RKSHEAEVLKQLAEKREHEKEVLQKAIEENNNFSKMAEEKLTHKMEANKENREAQMAAKL
ERLREKMYFWTHGPGPHPAQISAEQSCLRSVPALCPALGLQSALITWSDLSHHH

>Mus musculus_STMN1(ENSMUSP_101493)
MASSDIQVKELEKRASGQAFELILSPRSKESVPDFPLSPPKKKDLSLEEIQKKLEAAEER
RKSHEAEVLKQLAEKREHEKEVLQKAIEENNNFSKMAEEKLTHKMEANKENREAQMAAKL
ERLREKFLPGQARGRGAEEQRIQRPRG

>Rattus norvegicus_STMN1(ENSRNOP_22574)
MASSDIQVKELEKRASGQAFELILSPRSKESVPEFPLSPPKKKDLSLEEIQKKLEAAEER
RKSHEAEVLKQLAEKREHEKEVLQKAIEENNNFSKMAEEKLTHKMEANKENREAQMAAKL
ERLREKDKHVEEVRKNKESKDPADETEAD

>Felis catus_STMN1(ENSFCAP_18356)

MASSDIQVKELEKRASGQAFELILSPRSKESVPEFPLSPPKKKDLSLEEIQKKLEAAEER
RKSHEAEVLKQLAEKREHEKEVLQKAIEENNNFSKMAEEKLTHKMEANKENREAQMAAKL
ERLREKDKHIEEVRKNKESKDPADETEAD

>Equus caballus_STMN1(ENSECAP_19130)
MASSDIQVKELEKRASGQAFELILSPRSKESVPEFPLSPPKKKDLSLEEIQKKLEAAEER
RKSHEAEVLKQLAEKREHEKEVLQKAIEENNNFSKMAEEKLTHKMEANKENREAQMAAKL
ERLREKDKHIEEVRKNKESKDPADETEAD

>Myotis lucifugus_STMN1(ENSMLUP_9804)
MASSDIQVKELEKRASGQAFELILSPRSKESVPEFPLSPPKKKDLSLEEIQKKLEAAEER
RKSHEAEVLKQLAEKREHEKEVLQKAIEENNNFSKMAEEKLTHKMEANKENREAQMAAKL
ERLREKDKHIEEVRKNKESKDPADETEAD

>Gallus gallus_STMN1(ENSGALP_2235)
MATSDIQVKELEKRASGQAFELILGPRSKEAAPEFPLSPPKKKDLSLEEIQKKLEAAEER
RKSHEAEVLKQLAEKREHEKEVLQKAIEENNNFSKMAEEKLTHKMEANKENREAQMAAKL
ERLREKDKHIEEVRKNKEGKDPGEAETN

>Anolis carolinensis_STMN1(ENSACAP_17541)
MASSDIQVKELEKRASGQAFELILGPPSKDAVPEFPLSPPKKKDLSLEEIQRKLEAAEER
RKSHEAEVLKQLAEKREHEKEVLQKAIEENNNFSKMAEEKLTHKMEANKENREAQLAAKM
ERLREKEKHLEEVRKNKEGGKDSGDNESD

>Danio rerio_STMN1a(ENSDARP_41006)
MASSGADIQVKELDKRASGQAFEVILSPTAPDAKGEFPLSTPKKKEVSLDEIQKKLDAAE
ERRKNHEAEVLKHLAEKREHEKEVLQKAMEENNNFSKMAEEKLNQKMEANKENRTARMAA
MNEKFKEKDKKLEEVRKNKETKEGGEDEN

>Danio rerio_STMN1b(ENSDARP_133564)
MAATSDIQVKELDKRASGQAFEVILGSPASDVKNEFLLSPPKKKDLSLVEIQKKLEAAEE
RRKSHEAEVLKHLAEKREHEKEVLQKALEENNNFSKMAEEKLNQKMEANKENRTAIMAAM
NEKFKEKDKKIEEVRKNKETKEHNAFSFSDTFITSGLGGDLCA

>Takifugu rubripes_STMN1a(ENSTRUP_28187)
MAASEDIKVKELDKRASGQAFEVILATPDPKGDFPLSPPKKKDVSLEEIQRKLDAAEERR
KNHEAEVLKHLAEKREHEKEVLQKAMEENNNFSKMAEEKLNQKMEANKENRTALKAAMDE
KFKEKDKKLEEVRKNKETKEGTSED

>Takifugu rubripes_STMN1b(ENSTRUP_28188)
IRLFISDIKVKELDKRASGQAFEVILATPDPKGDFPLSPPKKKDVSLEEIQRKLDAAEER
RKNHEAEVLKHLAEKREHEKEVLQKAMEENNNFSKMAEEKLNQKMEANKENRTALKAAMD
EKFKEKDKKLEEVRKNKETKEGTSED

>Tetraodon nigroviridis_STMN1a(ENSTNIP_529)
SRSGGSDIQVKELDKRASGQAFEVILAAPDPKGDFPLSPPKKKDVSLEEIQRKLDAAEER
RKNHEAEVLKHLAEKREHEKEVLQKAMEENNNFSKMTEEKLNQKMEANKENRTALKAAMN
EKFKEKDKKYEEVRKNKETKDGASED

>Tetraodon nigroviridis_STMN1b(ENSTNIP_15665)
MAAAEDIQVKELDKRASGQAFEVILAAPDPKGDFPLSPPKKKDVSLEEIQRKLDAAEERR
KNHEAEVLKHLAEKREHEKEVLQKAMEENNNFSKMTEEKLNQKMEANKENRTALKAAMNE
KFKEKDKKYEEVRKNKETKDGASED

>Gasterosteus aculeatus_STMN1(ENSGACP_10298)
MATSEEILVKELDKRASGQAFEVILAAPAPDAKPEFPLTPPKKKDVSLEEIQRKLDAAEE
RRKNHEAEVLRHLAEKREHEKEVLQKAMEENNNFSKMAEEKLNQKMEANKENRTALMAAM
NEKFKEKDKKLEEVRKNKETKEGSED

>Homo sapiens_STMN2(ENSP_429243)
MAKTAMAYKEKMKELSMLSLICSCFYPEPRNINIYTYDDMEVKQINKRASGQAFELILKP

PSPISEAPRTLASPKKKDLSLEEIQKKLEAAEERRKSQEAQVLKQLAEKREHEREVLQKA
LEENNNFSKMAEEKLILKMEQIKENREANLAAIIERLQEKLVKFISSELKESIESQFLEL
QREGEKQ

>Pan troglodytes_STMN2(ENSPTRP_34842)
MAKTAMAYKEKMKELSMLSLICSCFYPEPRNINIYTYDDMEVKQINKRASGQAFELILKP
PSPISEAPRTLASPKKKDLSLEEIQKKLEAAEERRKSQEAQVLKQLAEKREHEREVLQKA
LEENNNFSKMAEEKLILKMEQIKENREANLAAIIERLQEKLVKFISSELKESIESQFLEL
QREGEKQ

>Pongo abelii_STMN2(ENSPPYP_20967)
MAKTAMAYKEKMKELSMLSLICSCFYPEPRNINIYTYDDMEVKQINKRASGQAFELILKP
PSPISEAPRTLASPKKKDLSLEEIQKKLEAAEERRKSQEAQVLKQLAEKREHEREVLQKA
LEENNNFSKMAEEKLILKMEQIKENREANLAAIIERLQEKERHAAEVRRNKELQVELSG

>Mus musculus_STMN2(ENSMUSP_29002)
MAKTAMAYKEKMKELSMLSLICSCFYPEPRNINIYTYDDMEVKQINKRASGQAFELILKP
PSPISEAPRTLASPKKKDLSLEEIQKKLEAAEERRKSQEAQVLKQLAEKREHEREVLQKA
LEENNNFSKMAEEKLILKMEQIKENREANLAAIIERLQEKERHAAEVRRNKELQVELSG

>Canis familiaris_STMN2(ENSCAFP_12328)
MAKTAMAYKEKMKELSMLSLICSCFYPEPRNINIYTYDDMEVKQINKRASGQAFELILKP
PSPISEAPRTLASPKKKDLSLEEIQKKLEAAEERRKSQEAQVLKQLAEKREHEREVLQKA
LEENNNFSKMAEEKLILKMEQIKENREANLAAIIERLQEKERHAAEVRRNKELQVELSG

>Bos taurus_STMN2(ENSBTAP_26776)
MAKTAMAYKEKMKELSMLSLICSCFYPEPRNINIYTYDDMEVKQINKRASGQAFELILKP
PSPISEAPRTLASPKKKDLSLEEIQKKLEAAEERRKSQEAQVLKQLAEKREHEREVLQKA
LEENNNFSKMAEEKLILKMEQIKENREANLAAIIERLQEKERHAAEVRRNKELQVELSG

>Myotis lucifugus_STMN2(ENSMLUP_14224)
TVPFVPAYKEKMKELSMLSLICSCFYPEPRNISIYTYDDMEVKQINKRASGQAFELILKP
PSPISEAPRTLASPKKKDLSLEEIQKKLEAAEERRKSQEAQVLKQLAEKREHEREVLQKA
LEENNNFSKMAEEKLILKMEQIKENREANLAAIIERLQERERHAAEVRRNKELQVELSG

>Gallus gallus_STMN2(ENSGALP_25330)
LTRFLVSAYKEKMKELSMLSLICSCFYPEPRNMNIYKYDDMEVKQINKRASGQAFELILK
PPSPVSEAPRTLASPKKKELSLEEIQKKLEAAEERRKSQEAQVLKHLAEKREHEREVLQK
ALEENNNFSKMAEEKLILKMEQIKENREANLAALIERLQEKERHAAEVRRNKELQVELSG

>Anolis carolinensis_STMN2(ENSACAP_13160)
MAKTAMAYKEKMKELSMLSLICSCFYPEPRNLNIYTYDDMEVKQINKRASGQAFELILKP
PSPVSEAPRTLASPKKKDLSLDEIQKKLEAAEERRKSQEAQVLKQLAEKREHEREVLQKA
LEENNNFSKMAEEKLVMKMEQIKENREANLAALVERLQEKERHAAEVRRNKELQVELSG

>Danio rerio_STMN2a(ENSDARP_94591)
MAKTAIAYKEKMKELSMLSLICSCFSPQTRNNIVCEFEDMEVKPINKRASGQAFEVILKP
PSPVSDAAHSITSPPKKRDMSLDDIQKKLEAAEDRRRSQEAQVLKALAEKREHERDVLLK
AMEENSNFSKMAEEKLILKMEQNKENREAHLAAMIDRLHEKERHAAIVRRNKELREQIVE

>Danio rerio_STMN2b(ENSDARP_72761)
MAKTAIAYKEKMKELSLVSLICSCLYPEARKNIMGEFEDMEVKAINKRASGQAFEVILKP
PSPTTEGGYSITSPPKKRDMSLEDIQKKLEAAEDRRRSQEAQVLKALAEKREHERDVLLK
AMEENSNFSRMAEEKLILKMEQIKENRDAHLAAMMDRLHEKEKHAQLVRRNKELREELTA

>Takifugu rubripes_STMN2(ENSTRUP_34816)
MAKTATAYKEKMKELSVLSLICSCFYPEARNKFVHEFEDMEVKAIKKRTSGQAFEVILKP
PSPVSDAVHNFPSPPKRDISLEDIEKKLEAAEDRRRYQEAQVMRALAEKREHERDVLLKA
MEENSNFSKMAEEKLQMKMDQIKENRDAHLAAMLERLQEKVRKPQNSVPRPSVLLLPHPD
RGGSVTVTFQERHAALVRKNKELREELT

>Tetraodon nigroviridis_STMN2a(ENSTNIP_21571)
MAKTASAYKEKMKELSVLSLICSCFYPEARNKLLHEFEDMEVKPIKKRASGQAFEVILKP
PSPVSDAVHNFPPPPKRDISLEDIEKKLEAAEDRRRYQEAQVLRALAEKRDHERDVLLKA
MEDNSNFSKMAEEKLQMKMDQIKENRDAHLAAMLERLQEKERHAALVRKNKELREELTA

>Tetraodon nigroviridis_STMN2b(ENSTNIP_20938)
AASAPSAYKEKMKEISVLSLICSCLYPESRKNILGDFEDLDIKPINKRASGQAFEVLLKP
SSPVTDAAHCVTSPPKRDLSLEDIQKKLEAAEDRRRSQEAQVLQALAEKREHERDVLLRA
MEENSNFSRMAEEKLQLKMEQIEENRMAYLAAMMERLQEKEKHAQEVRRNKELREALTA

>Gasterosteus aculeatus_STMN2a(ENSGACP_11495)
MSKTATAYKEKMKELSVFSLICSCFYPEAHNKRVCEFEVADMEVKAINKRASGQAFEVIL
KPLSPVSDVAHNLPTPPKRDISLEDIEKKLEAAEDRRKYQEAQVLRALAEKRDHEREVLI
KAMEENSNFSKMAEEKLLMKMEQIKENREAHLAAMIERLQEKVRNAEILLWCARTRELRE
ELTD

>Gasterosteus aculeatus_STMN2b(ENSGACP_4486)
MAKTAIAYKEKMKEISVLSFICSCLYPETHKNIMGDFEDMDIKPINKRASGQAFEVILKP
TSPVSDAAHCVTTPPKRDVSLEDIQKKLEAAEERRRSQEAQVLRALAEKRDHERDVLLKA
MEENSAFSRMAEEKLQVKMEHIEENRQAYLAAIMERLQERERHAQEVRRNKELREEVTA

>Oryzias latipes_STMN2a(ENSORLP_10355)
MAKTATAYKEKMKELSVLSLICSCFYPESRNKLVCEFEDMEVKPIKKRTSGQAFEVILKP
LSPVSEVSHSLPSPPKRDISLEDIEKKLEAAEERRKYQEAQVLRALAEKREHERDVLLKA
MEENSNFSKMAEEKLHMKMEQIKENREAHMAAMMERLLEKEKRAVVVRRNKELREELTA

>Oryzias latipes_STMN2b(ENSORLP_16214)
MEKTAIAYKEKMKEMSVFSLICSCLYPESRKNIMGDFEGDLDIKPLNKRASGQAFEVILK
PPSPVSDVAHCISSPPKRDISLEDIQKKLEAAEDRRRSQEAQILRILAEKREHERDVLLK
AMEENSNFSRMAEEKLQMKMEQIEENRQAYLAAMMERLQERERHAQVVRRNKELREELLP

>Homo sapiens_STMN4(ENSP_342538)
MTLAAYKEKMKELPLVSLFCSCFLADPLNKSSYKYEGWCGRQCRRKDESQRKDSADWRER
RAQADTVDLNWCVISDMEVIELNKCTSGQSFEVILKPPSFDGVPEFNASLPRRRDPSLEE
IQKKLEAAEERRKYQEAELLKHLAEKREHEREVIQKAIEENNNFIKMAKEKLAQKMESNK
ENREAHLAAMLERLQEKDKHAEEVRKNKELKEEASR

>Pan troglodytes_STMN4(ENSPTRP_53212)
MTLAAYKEKMKELPLVSLFCSCFLADPLNKSSYKYEGWCGRHCRRKDESHRIDSADRETI
PHADTVDLNWCVISDMEVIELNKCTSGQSFEVILKPPSFDGVPEFNASLPRRRDPSLEEI
QKKLEAAEERRKYQEAELLKHLAEKREHEREVIQKAIEENNNFIKMAKEKLAQKMESNKE
NREAHLAAMLERLQEKVRGPWLERRFLGHAVFLLQQVCRDLLIISGRKQPRAKIRIFFLG
RILREFRRMIREVRGKK

>Gorilla gorilla_STMN4(ENSGGOP_3284)
MTLAAYKEKMKELPLVSLFCSCFLADPLNKSSYKYEGWCGRQCRRKDESQRKDSADWRER
RAQADTVDLNWCVISDMEVIELNKCTSGQSFEVILKPPSFDGVPEFNASLPRRRDPSLEE
IQKKLEAAEERRKYQEAELLKHLAEKREHEREVIQKAIEENNNFIKMAKEKLAQKMESNK
ENREAHLAAMLERLQEKDKHAEEVRKNKELKEEASR

>Mus musculus_STMN4(ENSMUSP_113759)
MTLAAYKEKMKELPLVSLFCSCFLSDPLNKSSYKYEGWCGRQCRRKGQSQRKGSADWRER
REQADTVDLNWCVISDMEVIELNKCTSGQSFEVILKPPSFDGVPEFNASLPRRRDPSLEE
IQKKLEAAEERRKYQEAELLKHLAEKREHEREVIQKAIEENNNFIKMAKEKLAQKMESNK
ENREAHLAAMLERLQEKDKHAEEVRKNKELKEEASR

>Rattus norvegicus_STMN4(ENSRNOP_73294)
MTLAAYKEKMKELPLVSLFCSCFLSDPLNKSSYKYEGWCGRQCRRKGQSQRKGSADWRER
REQADTVDLNWCVISDMEVIELNKCTSGQSFEVILKPPSFDGVPEFNASLPRRRDPSLEE
IQKKLEAAEERRKYQEAELLKHLAEKREHEREVIQKAIEENNNFIKMAKEKLAQKMESNK
ENREAHLAAMLERLQEKDKHAEEVRKNKELKEEASR

>Felis catus_STMN4(ENSFCAP_13372)
AAYKEKMKELPLVSLFCSCFLADPLNKSSYKYEGWCGRQCRRKDQSQRKDSADWRERREQ
ADTVDLNWCVISDMEVIELNKCTSGQSFEVILKPPSFDGVPEFNASLPRRRDPSLEEIQK
KLEAAEERRKYQEAELLKHLAEKREHEREVIQKAIEENNNFIKMAKEKLAQKMESNKENR
EAHLAAMLERLQEKDKHAEEVRKNKELKEEASR

>Bos taurus_STMN4(ENSBTAP_23448)
MTLAAYKEKMKELPLVSLFCSCFLADPLNKSSYKYEADTVDLNWCVISDMEVIELNKCTS
GQSFEVILKPPSFDGVPEFNASLPRRRDPSLEEIQKKLEAAEERRKYQEAELLKHLAEKR
EHEREVIQKAIEENNNFIKMAKEKLAQKMESNKENREAHLAAMLERLQEKDKHAEEVRKN
KELKEEASR

>Myotis lucifugus_STMN4(ENSMLUP_4034)
MTLAAYKEKMKELPLVSLFCSCFLADPLNKSSYKYEADTVDLNWCVISDMEVIELNKCTS
GQSFEVILKPPSFDGVPEFNASLPRRRDPSLEEIQKKLEAAEERRKYQEAELLKHLAEKR
EHEREVIQKAIEENNNFIKMAKEKLAQKMESNKENREAHLAAMLERLQEKDKHAEEVRKN
KELKEEASR

>Gallus gallus_STMN4(ENSGALP_26738)
MTLAAYKEKMKELPLVSLFCSCFLSDPLNKPAYTYEADTVDLTWCVISDMEVIELNKRTS
GQSFEVILKPPSFDGIPEFNASLPRRRDPSLEEIQKKLEAAEERRKYQEAELLKHLAEKR
EHEREVIQKAIEENNNFIKMAKEKLAQKMESNKENREAHLAAMLERLQEKDKHAEEVRKN
KELKEEASR

>Xenopus tropicalis_STMN4(ENSXETP_38062)
MTLAAYKEKVKELPLVSIFCSCFLSDPLKNQTYKYDADTVDLTWCAISDMEVIELNKRAS
GHAFEVILKPPSFDAIPEITATLPQKRDPSLEEIQKKLEAAEERRKYQESELLKHLAEKR
EHEREVIQKAIEENNNFSKMAKVKLAQRMEVNKENREAHLAAMLERLQEKDKHAEEVRKN
KELKEEASR

>Danio rerio_STMN4(ENSDARP_113219)
MTLAAYRDKVKELPLVSLLCSCINSNRAENPTYKAEDAVDLNWCVIKEMEVIELNKRTSG
QAFEVILKPPSFDVAPELNTSMPQRKDPSLEEIQKKLEAAEERRKFQEAEMLKHLAEKRE
HEREVIQKAFEENNNFIKNAKEKLEQRMEANKENREALLAAMLERLQEKDKHAEEVRKNK
ELKEEACR

>Takifugu rubripes_STMN4(ENSTRUP_3348)
MTLAAYKEKVKELPLVSLFCACLNPRTIENPTYKAEDAVDLGWCVMKDVEVIELNKRASG
QAFEVILKPPSFDGVPDLNTSMPQRRDPSLEEIQKKLEAAEERRKCQEAELLKHLAEKRE
HEREVIQKAFEENNNFIKNAKEKLEQKMEAIKENREALLAAMLERLQEKDKHAEEVRKNK
EMKEEACR

>Tetraodon nigroviridis_STMN4(ENSTNIP_22371)
MTLAAYKEKVKELPLVSLFCACLNPQTLENPTYKAEADAVDLGWCVIKDVEVIELNKRAS
GQAFEVILKPPSFDGVPELNTSMPQRRDPSLEEIQKKLEAAEERRKCQEAELLKHLAEKR
EHEREVIQKAFEENNNFIKNAKEKLEQKMEAIKENREALLAAMLERLQEKDKHAEEVRKN
KEMKEEACR

>Gasterosteus aculeatus_STMN4(ENSGACP_5935)
MTLAAYKEKVKELPLVSLFCACFNPQRADKPTYKAEDAVDLGWCVIKDVEVIELNKRTSG
QAFEVILKPPSFDGVPELNGSMPQRRDPSLEEIQKKLEAAEERRKCQEAELLKHLAEKRE
HQREVIQKAFEENNNFIKNAKEKLEQKMEANKENREALLAAMLERLQEKDKHAEEVRKNK
EMKEEACR

>Oryzias latipes_STMN4(ENSORLP_20225)
MTLAAYKEKVKELPLVSLFCACLNPQTAEKTPYKAEDPTDAVDLGWCVMKDVEVIELNKR
TSGQAFEVILKPPSFDALPELNTSMPQRRDPSLEEIQKKLEAAEERRKCQEAELLKHLAE
KREHEREVIQKAYAENNNFIKNAKEKLEQKMEANKENREALLAAMLERLQEKVQHKKILK
KKKKIKNESNG

>Homo sapiens_STMN3(ENSP_359070)
MASTISAYKEKMKELSVLSLICSCFYTQPHPNTVYQYGDMEVKQLDKRASGQSFEVILKS
PSDLSPESPMLSSPPKKKDTSLEELQKRLEAAEERRKTQEAQVLKQLAERREHEREVLHK
ALEENNNFSRQAEEKLNYKMELSKEIREAHLAALRERLREKELHAAEVRRNKEQREEMSG

>Pan troglodytes_STMN3(ENSPTRP_51938)
MASTISAYKEKMKELSVLSLICSCFYTQPHPNTVYQYGDMEVKQLDKRASGQSFEVILKS
PSDLSPESPMLSSPPKKKDTSLEELQKRLEAAEERRKTQEAQVLKQLAERREHEREVLHK
ALEENNNFSRQAEEKLNYKMELSKEIREAHLAALRERLREKELHAAEVRRNKEQREEMSG

>Gorilla gorilla_STMN3(ENSGGOP_4679)
MASTISAYKEKMKELSVLSLICSCFYTQPHPNTVYQYGDMEVKQLDKRASGQSFEVILKS
PSDLSPESPMLSSPPKKKDTSLEELQKRLEAAEERRKTQEAQVLKQLAERREHEREVLHK
ALEENNNFSRQAEEKLNYKMELSKEIREAHLAALRERLREKELHAAEVRRNKEQREEMSG

>Mus musculus_STMN3(ENSMUSP_99334)
MASTVSAYKEKMKELSVLSLICSCFYSQPHPNTIYQYGDMEVKQLDKRASGQSFEVILKS
PSDLSPESPVLSSPPKRKDASLEELQKRLEAAEERRKTQEAQVLKQLAERREHEREVLHK
ALEENNNFSRLAEEKLNYKMELSKEIREAHLAALRERLREKELHAAEVRRNKEQREEMSG

>Rattus norvegicus_STMN3(ENSRNOP_18697)
MASTVSAYKEKMKELSVLSLICSCFYSQPHPNTIYQYGDMEVKQLDKRASGQSFEVILKS
PSDLSPESPVLSSPPKRKDASLEELQKRLEAAEERRKTQEAQVLKQLAERREHEREVLHK
ALEENNNFSRLAEEKLNYKMELSKEIREAHLAALRERLREKELHAAEVRRNKEQREEMSG

>Bos taurus_STMN3(ENSBTAP_48555)
MASTISAYKEKMKELSVLSLICSCFYSQPHPNTVYQYGDMEVKQLDKRASGQSFEVILKS
PSDLSPESPMLSSPPKRKDTSLEELQKRLEAAEERRKTQEAQVLKQLAERREHEREVLHK
ALEENNNFSRLAEEKLNYKMELSKEIREAHLAALRERLREKELHAAEVRRNKEQREEMSG

>Myotis lucifugus_STMN3(ENSMLUP_19179)
PGLPPTAYKEKMKELSVLSLICSCFYSQPHPNTIYQYGDMEVKQLDKRASGQSFEVILKS
PSDLSPESPVLSSPPKRKDTSLEELQKRLEAAEERRKTQEAQVLKQLAERREHEREVLHK
ALEENNNFSRLAEEKLNYKMELSKEIREAHLAALRERLREKELHAAEVRRNKEQREEMSG

>Monodelphis domestica_STMN3(ENSMODP_21178)
MASTMSAYKEKMKELSVLSLICSCFYTQPHPNTIYQYGDMEVKQLDKRASGQSFEVILKS
PSDLSPESPILSSPPKKKDISLEELQKRLEAAEERRKTQEAQVLKQLAEKREHEREVLHK
ALEENNNFSRLAEEKLNYKMELSKEIREAHLAALRERLREKELHAAEVRRNKEQREEISG

>Gallus gallus_STMN3(ENSGALP_9879)
MASTVSAYKEKMKELSLLSLICSCFHTQPHPNTIYQYGDMEVKQLDKRASGQSFEVILKS
PSDLSPESPILSSPPKKKDLSLEELQRRLEAAEERRKTQEAQVLKQLAEKREHEREVLHK
ALEENNNFSRLAEEKLNYKMELSREIREAHLAALRERLREKELHAAEVRRNKEQREEISG

>Xenopus tropicalis_STMN3(ENSXETP_7299)
SSSSLAAYKEKMKELSMIDMICSCLYSQPLPKTVYQFEDMEVKQLNKRASGQSFEVILKP
PTDLSPESPPLTTSPTKKDISLEELQKRLEAAEERRKIQEAQVLKQLSEKREHERNVLHR
AIEDNNSFSRIAEEKLNYKMELSQEIRDANLAALKERLREK

>Danio rerio_STMN3(ENSDARP_128691)
MDSTLSAYSDKLREMSVLSLICSCLYNQPHPNTFGQFEDMEVKSLNKRASGQAFEVILKS
PTDPSPERPQTLALPPQRKEPSLGELQRRLEAAEARRRSQEKQVLKQLAEKREREKEVLT
KAQEVNNNYSKKTEEKLNHKMEMITENRMARLNALKQRLREKEIHAEEVRKNKELNTDLS
G

>Saccoglossus kowalevskii_(XP_006814464)
MRSFICPLSFRALDTFSRPFACCFYSEISMPSSTLVKECAEVKTTGGVAYDVILSEPKTETVLKRPVSPP
KKSEKSLTDIASKLAAAEERRKSMESQLLQRLAEERALAQEKASRVMKDNNNFQEVTKKQLDEKMQVFEE
NRNARFQALQKRLKERRYMAEKYATTKGPSAKKMQKMAIEH

>Drosophila melanogaster_(FBpp0078829)
MLIGLVRDSVMQCFCHTCRAPGILPAVSRRSAPIKKNNKIRSKQPRLSKKVKFITTEIRC
QEKSRGGLSYEVILAEPAPNVAVPKRPVTPGKNVSVEEIEQKLKAAEERRISLEAKKMAD
ISTKLAKVEEATRKKDEITNEFITQTKEQLESKMELHVEKREAIISDMKEKLKIHAQDIE
KTRETLEQQKANEQKAIEEKLKIAQSLRDENIKKMLDRLKEHERRAELVRQNKAQAQDLD
GQQSAIASSG

Serine/Threonine-Protein Kinase-STK

>Homo sapien_STK3(ENSP_429744)
MLQLMDSGITICLRNGAASVFKKKEWSTQGEENKQDSKLKKLSEDSLTKQPEEVFDVLEK
LGEGSYGSVFKAIHKESGQVVAIKQVPVESDLQEIIKEISIMQQCDSPYVVKYYGSYFKN
TDLWIVMEYCGAGSVSDIIRLRNKTLIEDEIATILKSTLKGLEYLHFMRKIHRDIKAGNI
LLNTEGHAKLADFGVAGQLTDTMAKRNTVIGTPFWMAPEVIQEIGYNCVADIWSLGITSI
EMAEGKPPYADIHPMRAIFMIPTNPPPTFRKPELWSDDFTDFVKKCLVKNPEQRATATQL
LQHPFIKNAKPVSILRDLITEAMEIKAKRHEEQQRELEEEEENSDEDELDSHTMVKTSVE
SVGTMRATSTMSEGAQTMIEHNSTMLESDLGTMVINSEDEEEDGTMKRNATSPQVQRPS
FMDYFDKQDFKNKSHENCNQNMHEPFPMSKNVFPDNWKVPQDGDFDFLKNLSLEELQMRL
KALDPMMEREIEELRQRYTAKRQPILDAMDAKKRRQQNF

>Pan troglodytes_STK3(ENSPTR_34983)
MEQPPAPKSKLKKLSEDSLTKQPEEVFDVLEKLGEGSYGSVFKAIHKESGQVVAIKQVPV
ESDLQEIIKEISIMQQCDSPYVVKYYGSYFKNTDLWIVMEYCGAGSVSDIIRLRNKTLTE
DEIATILKSTLKGLEYLHFMRKIHRDIKAGNILLNTEGHAKLADFGVAGQLTDTMAKRNT
VIGTPFWMAPEVIQEIGYNCVADIWSLGITSIEMAEGKPPYADIHPMRAIFMIPTNPPPT
FRKPELWSDDFTDFVKKCLVKNPEQRATATQLLQHPFIKNAKPVSILRDLITEAMEIKAK
RHEEQQRELEEEEENSDEDELDSHTMVKTSVESVGTMRATSTMSEGAQTMIEHNSTMLES
DLGTMVINSEDEEEDGTMKRNATSPQVQRPSFMDYFDKQDFKNKSHENCNQNMHEPFPM
SKNVFPDNWKVPQDGDFDFLKNLSLEELQMRLKALDPMMEREIEELRQRYTAKRQPILDA
MDAKKRRQQNF

>Gorilla gorilla_STK3(ENSGGOP_28648)
MLQLMDGGITICLRNGAASVFKKKEWSTQGEENKQDSKLKKLSEDSLTKQPEEVFDVLEK
LGEGSYGSVFKAIHKESGQVVAIKQVPVESDLQEIIKEISIMQQCDSPYVVKYYGSYFKN
TDLWIVMEYCGAGSVSDIIRLRNKTLTEDEIATILKSTLKGLEYLHFMRKIHRDIKAGNI
LLNTEGHAKLADFGVAGQLTDTMAKRNTVIGTPFWMAPEVIQEIGYNCVADIWSLGITSI
EMAEGKPPYADIHPMRAIFMIPTNPPPTFRKPELWSDDFTDFVKKCLVKNPEQRATATQL
LQHPFIKNAKPVSILRDLITEAMEIKAKRHEEQQRELEEEEENSDEDELDSHTMVKTSVE
SVGTMRATSTMSEGAQTMIEHNSTMLESDLGTMVINSEDEEEDGTMKRNAASPQVQRPS
FMDYFDKQDFKNKSHENCNQNMHEPFPMSKNVFPDNWKVPQDGDFDFLKNLSLEELQMRL
KALDPMMEREIEELRQRYTAKRQPILDAMDAKKRRQQNF

>Mus musculus_STK3(ENSMUSP_18476)
MEQPPASKSKLKKLSEDSLTKQPEEVFDVLEKLGEGSYGSVFKAIHKESGQVVAIKQVPV
ESDLQEIIKEISIMQQCDSPYVVKYYGSYFKNTDLWIVMEYCGAGSVSDIIRLRNKTLTE
DEIATILKSTLKGLEYLHFMRKIHRDIKAGNILLNTEGHAKLADFGVAGQLTDTMAKRNT
VIGTPFWMAPEVIQEIGYNCVADIWSLGITSIEMAEGKPPYADIHPMRAIFMIPTNPPPT
FRKPELWSDDFTDFVKKCLVKSPEQRATATQLLQHPFIKNAKPVSILRDLIAEAMEIKAK
RHEEQQRELEEEEENSDEDELDSHTMVKTSSESVGTMRATSTMSEGAQTMIEHNSTMLES
DLGTMVINSEEEEEEEEEEEEDGTMKRNATSPQVQRPSFMDYFDKQDFKNKSHENCDQSM
REPGPMSNSVFPDNWRVPQDGDFDFLKNLSLEELQMRLKALDPMMEREIEELHQRYSAKR
QPILDAMDAKKRRQQNF

>Rattus norvegicus_STK3(ENSRNOP_62778)
MEQPPAPKSKLKKLSEDSLTKQPEEVFDVLEKLGEGSYGSVFKAIHKESGQVVAIKQVPV
ESDLQEIIKEISIMQQCDSPYVVKYYGSYFKNTDLWIVMEYCGAGSVSDIIRLRNKTLTE
DEIATILKSTLKGLEYLHFMRKIHRDIKAGNILLNTEGHAKLADFGVAGQLTDTMAKRNT
VIGTPFWMAPEVIQEIGYNCVADIWSLGITSIEMAEGKPPYADIHPMRAIFMIPTNPPPT
FRKPELWSDDFTDFVKKCLVKSPEQRATATQLLQHPFIKNAKPVSILRELITEGMEIKAK
RHEEQQRELEDEEENSDEDELDSHTMVKTSSEGVGTMRATSTMSEGAQTMIEHNSTMLES
DLGTMVINSEDEEEDGTMKRNATSPQVQRPSFMDYFDKQDFKNKSHENCDQSMREPCPM

SNNVFPDNWRVPQDGDFDFLKNLSLEELQMRLKALDPMMEREIEELHQRYSAKRQPILDA
MDAKKRRQQNF

>Canis familiaris_STK3(ENSCAFP_711)
MEQPPAPKSKLKKLSEDSLTKQPEEVFDVLEKLGEGSYGSVFKAIHKESGQVVAIKQVPV
ESDLQEIIKEISIMQQCDSPYVVKYYGSYFKNTDLWIVMEYCGAGSVSDIIRLRNKTLTE
DEIATILKSTLKGLEYLHFMRKIHRDIKAGNILLNTEGHAKLADFGVAGQLTDTMAKRNT
VIGTPFWMAPEVIQEIGYNCVADIWSLGITSIEMAEGKPPYADIHPMRAIFMIPTNPPPT
FRKPELWSDDFTDFVKKCLVKNPEQRATATQLLQHPFIKNAKPVSILRDLITEAMEIKAK
RHEEQQRELEEEEENSDEDELDSHTMVKTSSESVGTMKATSTMSEGAQTMIEHNSTMLES
DLGTMVINSEDEEEEDGTMKRNATSPQVQRPSFMDYFDKQDFKNKSHENCNQNMHEPFPM
SKNVFPDNWKVPQDGDFDFLKNLSLEELQMRLKALDPMMEREIEELRQRYTAKRQPILDA
MDAKKRRQQNF

>Felis catus_STK3(ENSFCAP_19309)
MEQPPAPKSKLKKLSEDSLTKQPEEVFDVLEKLGEGSYGSVFKAIHKESGQVVAIKQVPV
ESDLQEIIKEISIMQQCDSPYVVKYYGSYFKNTDLWIVMEYCGAGSVSDIIRLRNKTLTE
DEIATILKSTLKGLEYLHFMRKIHRDIKAGNILLNTEGHAKLADFGVAGQLTDTMAKRNT
VIGTPFWMAPEVIQEIGYNCVADIWSLGITSIEMAEGKPPYADIHPMRAIFMIPTNPPPT
FRKPELWSDDFTDFVKKCLVKNPEQRATATQLLQHAFIKNAKPVSILRDLITEAMEIKAK
RHEEQQRELEEEEENSDEDELDSHTMVKTSSESVGTMKATSTMSEGAQTMIEHNSTMLES
DLGTMVINSEDEEEEDGTMKRNATSPQVQRPSFMDYFDKQDFKNKSHENCNQNMHEPFPM
SKNVFPDNWKVPQDGDFDFGFLFVFVWPNLTMYF

>Monodelphis domestica_STK3(ENSMODP_8035)
SPPTPSPKLKKLSEDSLTKQPEEVFDVLEKLGEGSYGSVFKAIHKESGQVVAIKQVPVES
DLQEIIKEISIMQQCDSPYVVKYYGSYFKNTDLWIVMEYCGAGSVSDIIRLRNKTLTEEE
IATILKSTLKGLEYLHFMRKIHRDIKAGNILLNTEGHAKLADFGVAGQLTDTMAKRNTVI
GTPFWMAPEVIQEIGYNCVADIWSLGITSIEMAEGKPPYADIHPMRAILSHKSPPTFGKQ
KFGTDEFNDLSKKCLVKNPEQRATATQLLQHPFIKNAKAVSILRDLITDAMEIKAKRHEE
QQRELEEVEENSDEDELDSHTMVKNALESVNTMRAASTMSEGAQTMIEHNTTILENDLGT
MVINSEDEEEVDGTMKRNTTSSQAQRPSFMDYFDKQDFKNKSQENSDKNIHEQQVPVSKN
IFPDNWKVPQDGNFDFLKNLSLEELQMRLKALDPMMEREIEELRQSYNAKRQPILDAMDA
KKRRQQNF

>Gallus gallus_STK3(ENSGALP_25754)
MEQAAPKSKLKKLSEDSLTKQPEEVFDVLEKLGEGSYGSVFKAIHKESGQVVAIKQVPVE
SDLQEIIKEISIMQQCDSPYVVKYYGSYFKNTDLWIVMEYCGAGSVSDIIRLRNKRLTED
EIATILKSTLKGLEYLHFMRKIHRDIKAGNILLNTEGHAKLADFGVAGQLTDTMAKRNTV
IGTPFWMAPEVIQEIGYNCVADIWSLGITSIEMAEGKPPYADIHPMRAIFMIPTNPPPTF
RKPELWSDEFTDFVKKCLVKNPEQRATATQLLQHTFIKNAKPVSILRDLITEAMEIKAKR
HEEQQRELEEEEENSDEDELDSHTMVKTNSESASTMRATSTMSEGAQTMIEHNSTMLESD
LGTMVINSDDDEEEDGTMKRNATSPQVQRPSFMDYFDKQDSKNKSPENCNQNMHEPYHIS
KNVFPDNWKVPQDGDFDFLKNLSLEELQMRLKALDPMMEREIEELRQRYTAKRQPILDAM
DAKKRRQQNF

>Anolis carolinensis_STK3(ENSACAP_15922)
MEQAAPKSKLKKLSEDSLTKQPEEVFDVLEKLGEGSYGSVFKAIHKESGQVVAIKQVPVE
SDLQEIIKEISIMQQCDSPYVVKYYGSYFKNTDLWIVMEYCGAGSVSDIIRLRNKTLTED
EIATILKSTLKGLEYLHFMRKIHRDIKAGNILLNTEGHAKLADFGVAGQLTDTMAKRNTV
IGTPFWMAPEVIQEIGYNCVADIWSLGITSIEMAEGKPPYADIHPMRAIFMIPTNPPPTF
RKPELWSDEFTDFVKKCLVKNPEQRATATQLLQHPFIKNAKPVSILRDLITEAMEIKAKR
HEEQQRELEEEDENSEEDELDSHTMVKTGSESAGTMRATSTMSESAQTMIEHNSTMLESD
LGTMVINSDDDDEDEDEDGTMKRNATSPQAQRPSFMDYFDKQDSKNKSHENCNQNMHESY
HISKNVFPDNWKVPQDGDFDFLKNLSLEELQMRLKALDPMMEREIEELRQRYTAKRQPIL
DAMDAKKRRQQNF

>Danio rerio_STK3(ENSDARP_15367)
MEHSVPKNKLKKLSEDSLTKQPEEVFDVLEKLGEGSYGSVFKAIHKESGQVVAIKQVPVE
SDLQEIIKEISIMQQCDSPYVVKYYGSYFKNTDLWIVMEYCGAGSVSDIIRLRNKTLTED
EIATVLKSTLKGLEYLHFMRKIHRDIKAGNILLNTEGHAKLADFGVAGQLTDTMAKRNTV

IGTPFWMAPEVIQEIGYNCVADIWSLGITSIEMAEGKPPYADIHPMRAIFMIPTNPPPTF
RKPEHWSDDFTDFVKKCLVKNPEQRATATQLLQHPFIVGAKPVSILRDLITEAMDMKAKR
QQEQQRELEEDDENSEEEVEVDSHTMVKSGSESAGTMRATGTMSDGAQTMIEHGSTMLES
NLGTMVINSDDEEEEEDLGSMRRNPTSQQIQRPSFMDYFDKQDSNKAQEGFNHNQQDPCL
ISKTAFPDNWKVPQDGDFDFLKNLDFEELQMRLTALDPMMEREIEELRQRYTAKRQPILD
AMDAKKRRQQNF

>Takifugu rubripes_STK3(ENSTRUP_38341)
VLSLYLSSPDSKLKKLSEDSLTKQPEEVFDVLEKLGEGSYGSVFKAIHKESGQVVAIKQV
PVESDLQEIIKEISIMQQCDSPYVVKYYGSYFKNTDLWIVMEYCGAGSVSDIIRLRNKTL
TEEEIATILKSTLKGLEYLHFMRKIHRDIKAGNILLNTEGHAKLADFGVAGQLTDTMAKR
NTVIGTPFWMAPEVIQEIGYNCVADIWSLGITSIEMAEGKPPYADIHPMRAIFMIPTNPP
PTFRKPELWSDEFTDFVKKCLVKNPEQRATATQLLQHPFISQAKPVTILRDLITEAMEMK
AKRQQEQQRELEEEEDNSEEETEVDSHTMVKSGSEGAGTMRATSTMSDGAQTMIEHGSTM
LESDLGTMVINSDDDEEEEEDQRSMKRHATPQQPMRPSFMDYFDKQDSNKAAQQQENYNH
NQAQEPGYHIQSKNVFPDNWKVPQDGDFDFLKNLDFEELQLRLTALDPMMEREIEELRQR
YTAKRQPILDAMDAKKRRQQNF

>Tetraodon nigroviridis_STK3(ENSTNIP_12004)
MEPSAPKSKLKKLSEDSLTKQPEEVFDVLEKLGEGSYGSVFKAIHKESGQVVAIKQVPVE
SDLQEIIKEISIMQQCDSPYVVNYYGSYFKNTDLWIIMEYCGAGSVSDIIRLRNKCGQLT
EEEIATILKSTLKGLEYLHFMRKIHRDIKAGNILLNTEGHAKLADFGVAGQLTDTMAKRN
TVIGTPFWMAPEVIQEIGYNCVADIWSLGITSIEMAEGKPPYADIHPMRAIFMIPTNPPP
TFRKPELWTDEFTDFVKKCLIKNPEQRATATQLLQHPFISQAKPVTILRDLITEAMEMKA
KRQQEQQRELEEDDDNSEEETEVDSHTMVKSGSEGAGTMRATSTMSDGAQTMIEHGSTML
ESDLGTMVINSDEDEEEEEDQGSMRRHATSQQPMRPAFMDYFDKQDSNKAAQQQDNQPQE
PGYHIHAKNVFPDNWKVPQDGDFDFLKNLDFEEQLRLTALDPMMEREIEELRQRYTAKRQ
PILDAMDAKKRRQQNF

>Gasterosteus aculeatus_STK3(ENSGACP_4790)
IMETSAPKSKLKKLSEDSLTKQPEEVFDVLEKLGEGSYGSVFKAIHKESGQVVAIKQVPV
ESDLQEIIKEISIMQQCDSPYVVKYYGSYFKNTDLWIVMEYCGAGSVSDIIRLRNKTLTE
DEIATILKSTLKGLEYLHFMRKIHRDIKAGNILLNTEGHAKLADFGVAGQLTDTMAKRNT
VIGTPFWMAPEVIQEIGYNCVADIWSLGITSIEMAEGKPPYADIHPMRAIFMIPTNPPPT
FRKPELWSDDLTDFVKKCLVKNPEQRATATQLLQHPFISLAKPVTILRDLITEAMEMKAK
RQQEQQRELEEEDDNSEEETEVDSHTMVKSGSEGAGTMRATSTMSDGAQTMIEHGSTMLE
SDLGTMVINSDDDDDEEEDHGSMRRHATSQQPMRPSFMDYFDKQDSNKAAQQQENYNHNQ
PKEQPGFHIQSKNVFPDNWKVPQDGDFDFLKNLDFEELQLRLTALDPMMEREIEELRQRY
TAKRQPILDAMDAKKRRQQNF

>Homo sapiens_STK4(ENSP_361892)
METVQLRNPPRRQLKKLDEDSLTKQPEEVFDVLEKLGEGSYGSVYKAIHKETGQIVAIKQ
VPVESDLQEIIKEISIMQQCDSPHVVKYYGSYFKNTDLWIVMEYCGAGSVSDIIRLRNKT
LTEDEIATILQSTLKGLEYLHFMRKIHRDIKAGNILLNTEGHAKLADFGVAGQLTDTMAK
RNTVIGTPFWMAPEVIQEIGYNCVADIWSLGITAIEMAEGKPPYADIHPMRAIFMIPTNP
PPTFRKPELWSDNFTDFVKQCLVKSPEQRATATQLLQHPFVRSAKGVSILRDLINEAMDV
KLKRQESQQREVDQDDEENSEEDEMDSGTMVRAVGDEMGTVRVASTMTDGANTMIEHDDT
LPSQLGTMVINAEDEEEEGTMKRRDETMQPAKPSFLEYFEQKEKENQINSFGKSVPGPLK
NSSDWKIPQDGDYEFLKSWTVEDLQKRLLALDPMMEQEIEEIRQKYQSKRQPILDAIEAK
KRRQQNF

>Gorilla gorilla_STK4(ENSGGOP_13194)
METVQLRNPPRRQLKKLDEDSLTKQPEEVFDVLEKLGEGSYGSVYKAIHKETGQIVAIKQ
VPVESDLQEIIKEISIMQQCDSPHVVKYYGSYFKNTDLWIVMEYCGAGSVSDIIRLRNKT
LTEDEIATVLQSTLKGLEYLHFMRKIHRDIKAGNILLNTEGHAKLADFGVAGQLTDTMAK
RNTVIGTPFWMAPEVIQEIGYNCVADIWSLGITAIEMAEGKPPYADIHPMRAIFMIPTNP
PPTFRKPELWSDNFTDFVKQCLVKSPEQRATATQLLQHPFVRSAKGVSILRDLINEAMDV
KLKRQESQQREVDQDDEENSEEDEMDSGTMVRAVGDEMGTVRVASTMTDGANTMIEHDDT
LPSQLGTMVINAEDEEEEGTMKRRDETMQPAKPSFLEYFEQKEKENQINSFGKSVPGPLK
NSSDWKIPQDGDYEFLKSWTVEDLQKRLLALDPMMEQEIEEIRQKYQSKRQPILDAIEAK
KRRQQNF

```
>Pongo abelii_STK4(ENSPPYP_12335)
QLKKLDEDSLTKQPEEVFDVLEKLGEGSYGSVYKAIHKETGQIVAIKQVPVESDLQEIIK
EISIMQQCDSPHVVKYYGSYFKNTDLWIVMEYCGAGSVSDIIRLRNKTLTEDEIATILQS
TLKGLEYLHFMRKIHRDIKAGNILLNTEGHAKLADFGVAGQLTDTMAKRNTVIGTPFWMA
PEVIQEIGYNCVADIWSLGITAIEMAEGKPPYADIHPMRAIFMIPTNPPPTFRKPELWSD
NFTDFVKQCLVKSPEQRATATQLLQHPFVKSAKGVSILRDLINEAMDVKLKRQESQQREV
DQDDEENSEEDEMDSGTMVRAVGEEMGTVRVASTMTDGANTMIEHDDTLPSQLGTMVINA
EDEEEEGTMKRRDETMQPAKPSFLEYFEQKEKENQINSFGKSVPPLKNSSDWKIPQDGDY
EFLKSWTVEDLQKRLLALDPMMEQEIEEIRQKYQSKRQPILDAIEAKKRRQQNF

>Rattus norvegicus_STK4(ENSRNOP_61873)
METVQLRNPPRRQLKKLDEDSLTKQPEEVFDVLEKLGEGSYGSVYKAIHKETGQIVAIKQ
VPVESDLQEIIKEISIMQQCDSPHVVKYYGSYFKNTDLWIVMEYCGAGSVSDIIRLRNKT
LTEDEIATILQSTLKGLEYLHFMRKIHRDIKAGNILLNTEGHAKLADFGVAGQLTDTMAK
RNTVIGTPFWMAPEVIQEIGYNCVADIWSLGITAIEMAEGKPPYADIHPMRAIFMIPTNP
PPTFRKPEVWSDNFMDFVKQCLVKSPEQRATATQLLQHPFVKSAKGAAILRDLINEAMDV
KLKRQEAQQRAVDQDDDENSEEDEMDSGTMVRAAGDDMGTVRVASTMSGGANTMIEHGDT
LPSQLGTMVINTEDEEEEGTMKRRDETMQPAKPSFLEYFEQKEKENQINSFGKNVSGSLK
NSSDWKIPQDGDYEFLKSWTVEDLQKRLSALDPMMEQEMEEIRQKYRSKRQPILDAIEAK
KRRQQNF

>Canis familiaris_STK4(ENSCAFP_14145)
METVQLRNPPRRQLKKLDEDSLTKQPEEVFDVLEKLGEGSYGSVYKAIHKETGQIVAIKQ
VPVESDLQEIIKEISIMQQCDSPHVVKYYGSYFKNTDLWIVMEYCGAGSVSDIIRLRNKT
LTEDEIATILQSTLKGLEYLHFMRKIHRDIKAGNILLNTEGHAKLADFGVAGQLTDTMAK
RNTVIGTPFWMAPEVIQEIGYNCVADIWSLGITAIEMAEGKPPYADIHPMRAIFMIPTNP
PPTFRKPELWSDNFMDFVKQCLVKSPEQRATATQLLQHPFVKSAKGVSILRDLINEAMDV
KLKRQEAQQREVDQDDEENSEEDEMDSGTMVRAAGDEMGTVRVASTTSDGASTMIEHGDT
LPSQLGTMVINTEDEEEEGTMKRRDETMQPAKPSFLEYFEQKEKENQIHSFGQSVPGPLR
NSSDWKVPQDGDYEFLKSWTVEDLQKRLLALDPMMEQEIEEIRQKYQSKRQPILDAIEAK
KRRQQNF

>Felis catus_STK4(ENSFCAP_19419)
METVQLRNPPRRQLKKLDEDSLTKQPEEVFDVLEKLGEGSYGSVYKAIHKETGQIVAIKQ
VPVESDLQEIIKEISIMQQCDSPHVVKYYGSYFKNTDLWIVMEYCGAGSVSDIIRLRNKT
LTEDEIATILQSTLKGLEYLHFMRKIHRDIKAGNILLNTEGHAKLADFGVAGQLTDTMAK
RNTVIGTPFWMAPEVIQEIGYNCVADIWSLGITAIEMAEGKPPYADIHPMRAIFMIPTNP
PPTFRKPELWSDNFMDFVKQCLVKSPEQRATATQLLQHPFVKSAKGVSILRDLINEAMDV
KLKRQEAQQREVDQDDEDNSEEDEMDSGTMVRAAGDEMGTVRVASTVTDGASTMIEHGDT
LPSQLGTMVINAEDEEEEGTMKRRDETMQPAKPSFLEYFEQKEKENQINSFGKSVPGPLR
NSSDWKVPQDGDYEFLKSWTVEDLQKRLLALDPMMEQEIEEIRQKYQSKRQPILDAIEAK
KRRQQNF

>Myotis lucifugus_STK4(ENSMLUP_10751)
VCGAGSGRREDGALSGSGRLPGSAMETVQLRNPPRRQLKKLDEDSLTKQPEEVFDVLEKL
GEGSYGSVYKAIHKETGQIVAIKQVPVESDLQEIIKEISIMQQCDSPHVVKYYGSYFKNT
DLWIVMEYCGAGSVSDIIRLRNKTLTEDEIATILQSTLKGLEYLHFMRKIHRDIKAGNIL
LNTEGHAKLADFGVAGQLTDTMAKRNTVIGTPFWMAPEVIQEIGYNCVADIWSLGITAIE
MAEGKPPYADIHPMRAIFMIPTNPPPTFRKPELWSDSFMDFVKQCLVKSPEHRATATQLL
QHPFVKSAKGVSILRDLINEAMDVKLKRQEAQQREVENEDEENSEEDELDSGTMVRAAGD
EMGTVRVASTMSDGANTMIEHDGTLLSQLGTMVINAEDEEEEGTMKRRDETMQPAKPSFL
EYFEQKEKENQINSFGKSVPGPLKNSSDWKVPQDGDYEFLKSWTVEDLRKRLLALDPMME
QEIEEIRQKYQSKRQPILDAIEAKKRRQQNF

>Monodelphis domestica_STK4(ENSMODP_20242)
METVQLRNPPRRQLKKLDEDSLTKQPEEVFDVLEKLGEGSYGSVFKAIHKETGQVVAIKQ
VPVESDLQEIIKEISIMQQCDSPHVVKYYGSYFKNTDLWIVMEYCGAGSVSDIIRLRNKT
LTEDEIATILQSTLKGLEYLHFMRKIHRDIKAGNILLNTEGHAKLADFGVAGQLTDTMAK
RNTVIGTPFWMAPEVIQEIGYNCVADIWSLGITAIEMAEGKPPYADIHPMRAIFMIPTNP
PPTFRKPELWSDSFADFVKQCLVKSPEHRATATQLLQHPFVKSAKGVSILRDLINEAMDV
```

KLKRQEAQQRELDQDDDDNSEEDETDSGTMVRAVGDETGTVRAASTLSDGANTMIEHEDT
LPSQLGTMVINTEEEEEEGTMKRRDETMQPAKPSFLEYFEQKEKENQVNSFGKNVPGPLK
NSSDWKVPQDGDYEFLKSWSVEDLQKRLSALDPMMEQEIEEIRQKYQSKRQPILDAIEAK
KRRQQNF

>Gallus gallus_STK4(ENSGALP_6511)
METVQLRNPRRQLKKLDEDSLTKQPEEVFDVLEKLGEGSYGSVFKAIHKETGQVVAIKQV
PVESDLQEIIKEISIMQQCDSPHVVKYYGSYFKNTDLWIVMEYCGAGSVSDIIRLRNKTL
TEEEIATIVQSTLKGLEYLHFMRKIHRDIKAGNILLNTEGHAKLADFGVAGQLTDTMAKR
NTVIGTPFWMAPEVIQEIGYNCVADIWSLGITAIEMAEGKPPYADIHPMRAIFMIPTNPP
PTFRKPELWSDAFTDFVKQCLVKSPEQRATATQLLQHPFVKSAKGASILRDLINEAMDIK
LKRQEAQQRELDQEDEENSEEDETDSGTMVRASGDETGTIRVVNTMSDGANTMIEHDGTL
ESQLGTMVINTEDEEEEGTMKRRDETMQPARPSFLEYFEQKAKENQVNSFGKNVSGQTKN
SSDWKVPQDGDYEFLKTWSVDELQRRLSALDPMMEQEIEEIRQKYQSKRQPILDAIEAKK
RRQQNF

>Anolis carolinensis_STK4(ENSACAP_11982)
METVQLRHPRRQLRKLDEDSLTKQPEEVFDVLEKLGEGSYGSVFKAIHKETGQVVAIKQV
PVESDLQEIIKEISIMQQCDSPHVVKYYGSYFKNTDLWIVMEYCGAGSVSDIIRLRNKTL
MEDEIATIIQSTLKGLEYLHFMRKIHRDIKAGNILLNTEGHAKLADFGVAGQLTDTMAKR
NTVIGTPFWMAPEVIQEIGYNCLADIWSLGITAIEMAEGKPPYADIHPMRAIFMIPTNPP
PTFRKPELWSDEFTDFVKQCLVKCPEQRATATQLLQHPFVKSAKGVYILRDLINDAMDIK
LKIQEAQQRELYHDDEENSEEDEPDSRTMVRARGQETGTIRASCGMSDGINTMIEYTDTL
ESQLGTMVINTEDEEDEGTMKRRDETMASVRPSFLEYFEQKEKENRRNCVRQVPERYSPD
WRVPQDGDYEFNPAWFRRE

>Tetraodon nigroviridis_STK4(ENSTNIP_7894)
REKVQMRNNPRRQLKKLSEDSLTKQPEEVFDILEKLGEGSYGCVFKAHSKETGEIVAIKQ
VPVESDLQEMIKEISIMQQCNSPHVVQYYGSYFKNSDLLIVMEYCGAGSVADIIRIRNKT
LTEEEVAPILQSTLKGLEYLHFMRKIHRDIKAGNILLNTEGQAKLADFGVAGQLTDTMAK
RNTVIGTPFWMAPEIIQENGYNCVADIWSLGITAIEMAEGKPPYADIHPMRAIFMIPTNP
PPTFRKPELWSESFDFVSQCLVKNPEERATATQLLQHPFIKTSKPRSVLRTLISDAMEIK
LKRRSDENVYKEEEKISTASSDEDEVDQGTMVRAGTADSGTIRPADSLAGTMIHHEDTDT
LLGTLVINSDEEDAGTMKRKWSEETLQPTKPSFLQYFEQKEKEAGVQKDGVHRQTSADER
RPEGDLQVVSTWSVEELHLRLASLDPQMEQEIEEVRQRYQAKRKPILDAIEAKKRQQQRV

>Gasterosteus aculeatus_STK4(ENSGACP_23)
KDRVQMRNHPRRQLKKLSEDSLTKQPEEVFDVLEKLGEGSYGCVFKAHYKETGEIVAIKQ
VPVESDLQEIIKEISIMQQCNSPHVVRYYGSYFKNSDLWIVMEYCGAGSVSDIIRLRNKT
ITEEEICAILQSTLKGLEYLHFMRKIHRDIKAGNILLNSEGQAKLADFGVAGQLTDTMAK
RNTVIGTPFWMAPEVIQEIGYNCVADIWSLGITAIEMAEGKPPYADIHPMRAIFMIPTNP
PPTFRNPDLWSPSFQDFVSQCLVKNPENRATATQLLQHPFIKCPKPSSVLRALITDAMEI
KLKRQEEAEQRQQEDEDNSDEDEVDQGTMVRAGAGDSGTVRVAGSLSGSLGGTARTMIAH
DDDGDTGTMRSQLGTMVINSDEEDEEEAGTMKRRDETMQPAKPSFLQYFEQKEKEANSHR
EGGGDPADHRGLPEGDLQVVSLWSVEELRLRLASLDPQMEQEIEEIRRRYQTKRRPIMDA
IEAKKRRQQNF

>Oryzias latipes_STK4(ENSORLP_24937)
MENTQKVQMRNNPRRQLKKLSEDSLTKQPEEVFDVLEKLGEGSYGCVFKANYKETGEIVA
IKQVPVESDLQEIIKEISIMQQCNSPHVVRYYGSYFKNSDLWIVMEYCGAGSVSDIIRIR
NKTLSEEEIATVVHSTLKGLEYLHFMRKIHRDIKAGNILLNAEGQAKLADFGVAGQLTDT
MAKRNTVIGTPFWMAPEVIQEIGYNCVADIWSLGITAIEMAEGKPPYADIHPMRAIFMIP
TNPPPTFRNPDLWSENFRDFVSQCLVKNPENRATATQLLQHPFIKSAKPNSVLKSMITDA
MEIKLKKQEEALQREQEAEDEDNSDEDEVDQGTMVRAGTGDSGTIRAAGSLARSLSDTCR
TMIEHEDTGTMQSQLGTMVINSDEEDEEECGTMKRRDETMQPVKPSFLEYFEQKEKEANI
QNDGGRKGENHADNRKLPLDGDLEGVMTWSVEELRKRLASLDPQMEQEIEEIRQRYQAKR
QPILDAIQAKKRLQ

>Homo sapiens_STK24(ENSP_442539)
MAHSPVQSGLPGMQNLKADPEELFTKLEKIGKGSFGEVFKGIDNRTQKVVAIKIIDLEEA
EDEIEDIQQEITVLSQCDSPYVTKYYGSYLKDTKLWIIMEYLGGGSALDLLEPGPLDETQ

IATILREILKGLDYLHSEKKIHRDIKAANVLLSEHGEVKLADFGVAGQLTDTQIKRNTFV
GTPFWMAPEVIKQSAYDSKADIWSLGITAIELARGEPPHSELHPMKVLFLIPKNNPPTLE
GNYSKPLKEFVEACLNKEPSFRPTAKELLKHKFILRNAKKTSYLTELIDRYKRWKAEQSH
DDSSSEDSDAETDGQASGGSDSGDWIFTIREKDPKNLENGALQPSDLDRNKMKDIPKRPF
SQCLSTIISPLFAELKEKSQACGGNLGSIEELRGAIYLAEEACPGISDTMVAQLVQRLQR
YSLSGGGTSSH

>Pongo abelii_STK24(ENSPPYP_6227)
NLKADPEELFTKLEKIGKGSFGEVFKGIDNRTQKVVAIKIIDLEEAEDEIEDIQQEITVL
SQCDSPYVTKYYGSYLKDTKLWIIMEYLGGGSALDLLTDTQIKRNTFVGNPFWMAEVIKQ
SAYDSKADIWSLGITAIELARGEPPHSELHPMKVLFLIPKNNPPTLEGNYSKPLKEFVEA
CLNKEPSFRPTAKELLKHKFILRNAKKTSYLTELIDRYKRWKAEQSHDDSSSEDSDAETD
GQASGGSDSGDWIFTIREKDPKNLENGALQPSDLDRNKMKDIPKRPFSQCLSTIISPLFA
ELKEKSQACGGNLGSIEELRGAIYLAEEACPGISDTMVAQLVQRLQRYSLSGGGTSSH

>Rattus norvegicus_STK24(ENSRNOP_31981)
LQTLKADPEELFTKLEKIGKGSFGEVFKGIDNRTQKVVAIKIIDLEEAEDEIEDIQQEIT
VLSQCDSPYVTKYYGSYLKDTKLWIIMEYLGGGSALDLLEPGPLDEIQIATILREILKGL
DYLHSEKKIHRDIKAANVLLSEHGEVKLADFGVAGQLTDTQIKRNTFVGTPFWMAPEVIK
QSAYDSKADIWSLGITAIELAKGEPPHSELHPMKVLFLIPKNNPPTLEGSYSRPLKEFVE
ACLNKEPSFRPTAKELLKHKFIIRNAKKTSYLTELIDRYKRWKAEQSHEDSSSEDSDVET
DSQASGGSDSGDWIFTIREKDPKNLENGTLQPSDLERNKMKDFPKRPFSQCLSTIISPLF
AELKEKSQACGGNLGSIEELRGAIYLAEEACPGISDTMVAQLVQRLQRYSLSGGGASAH

>Dasypus novemcinctus_STK24(ENSDNOP_4811)
MNSRAQLLGLDEDEGHVHPGEKEKNLKADPEELFTKLEKIGKGSFGEVFKGIDNRTQKVV
AIKIIDLEEAEDEIEDIQQEITVLFQCDSGYISKIRHGTFPQDTKLWIIMEYLGGGSALD
LLEPGPLDETQIATILREILKGLDYLHSEKKIHRDIKAANVLLSEHGEVKLADFGVAGQL
TDTQIKRNTFVGTPFWMAPEVIKQSAYDSKADIWSLGITAIELAKGEPPHSELHPMKVLF
LIPKNNPPTLEGNYSKPLKEFVEACLNKEPSFRPTAKELLKHKFIIRNAKKTSYLTELID
RYKRWKAEQSHDDSSSEDSDTETDGQASGGSDSGDWIFTIREKDPKNLENGALQPSDLER
NKMKDIPKRPFSQCLSTIISPLFAELKEKSQACGGNLGSIEELRGAIYLAEEACPGISDT
MVAQLVQRLQRYSLSGGGSSSH

>Anolis carolinensis_STK24(ENSACAP_16226)
MAHSPVQQPGLPGMQNLKADPEELFTKLEKIGKGSFGEVFKGIDNRTQKVVAIKIIDLEE
AEDEIEDIQQEITVLSQCDSPYVTKYYGSYLKDTKLWIIMEYLGGGSALDLLEPGSLDEI
QIATILREILKGLDYLHSEKKIHRDIKAANVLLSEQGEVKLADFGVAGQLTDTQIKRNTF
VGTPFWMAPEVIKQSAYDSKADIWSLGITAIELAKGEPPHSELHPMKVLFLIPKNNPPTL
EGNYSKPLKEFVEACLNKDPSFRPTAKELLKHKFIVRNAKKTSYLTDLIDRHKRWKAEQN
HDNSSSEDSDGETDGQASGGSDSGDWIFTIREKDPKNLENGATQLHEWERNKENTIQRRP
LSRCLSTIISPLFTELKERSQACGGNVGSIEELREAIYLSEEACPGISDTMVTELVERLQ
RYSVAGGSASSH

>Danio rerio_STK24(ENSDARP_135203)
MAHSPVQGSLPGMQNLKADPEELFTKLERIGKGSFGEVFKGIDNRTQKVVAIKIIDLEEA
EDEIEDIQQEITVLSQCDSPFVTKYYGSYLKDTKLWIIMEYLGGGSALDLLEPGSLDETQ
IATILREILKGLEYLHSEKKIHRDIKAANVLLSEQGEVKLADFGVAGQLTDTQIKRNTFV
GTPFWMAPEVIKQSAYDSKADIWSLGITAIELAKGEPPHSDLHPMKVLFLIPKNNPPTLE
GNYCKPLKEFVEACLNKEPSFRPTAKELLKHKLIVRFAKKTSYLTELIDKYKRWKAEQSR
AESSSDESDSEPDGQASGGNDFGNDDWIFTIREKDPKKLQNGASLVGEEEPNKRPLSQSL
STVITPVLTELKEGAGEAAAATTVKPEVLEQLGEAIFLAEEAYPGISDVMVTQLIQRLQR
FSRARTSSSSHQ

>Takifugu rubripes_STK24(ENSTRUP_20094)
MAHSPVQGNLPGMQNCKADPEELFTKLERIGKGSFGEVFKGIDNRTQKVVAIKIIDLEEA
EDEIEDIQQEITVLSQCDSPFITKYYGSYLKGTKLWIIMEYLGGGSALDLMEPGALDETQ
IATILREILKGLEYLHSEKKIHRDIKAANVLLSEQGEVKLADFGVAGQLTDTQIKRNTFV
GTPFWMAPEVIKQSAYDSKADIWSLGITAIELAKGEPPHSELHPMKVLFLIPKNNPPTLE
GNYSKPLKEFVEACLNKEPSFRPTAKELLKHKLIVRHAKKTSYLTELVDKYKRWKAEQSR
TNDSSSDESDSEQDGQASGGNDFGSDDWIFTIREKDPKKLQNGEGQSGAEQTKDIPKRPY

SQSLATVISPALAELKARQEQVNGNPLVLDELRTAILLAEEAFPGISDSLVAHMVHRLQR
Y

>Tetraodon nigroviridis_STK24a(ENSTNIP_16268)
MAHSPVQGNLPGMQNAKADPEELFTKLERIGKGSFGEVFKGIDNRTQKVVAIKIIDLEEA
EDEIEDIQQEITVLSQCDSPFITKYYGSYLKGTKLWIIMEYLGGGSALDLMEPGALDETQ
IATILREILKGLEYLHSEKKIHRDIKAANVLLSEQGEVKLADFGVAGQLTDTQIKRNTFV
GTPFWMAPEVIKQSAYDSKADIWSLGITAIELAKGEPPHSELHPMKVLFLIPKNNPPTLE
GNYSKPLKEFVEACLNKEPSFRPTAKELLKHKLIVRHAKKTSYLTELVDKYKRWKAEQSR
TTESSSDESDSEQDGQASGGNDFGSDDWIFTIREKDPKKLQNGEGQSGTEQQTKDIPKRP
FSQSLATVISPALAELKARQEQVNGNPLVLDELRTAILLAEEAFPGISDSLVAHMVHRLQ
RY

>Tetraodon nigroviridis_STK24b(ENSTNIP_16990)
MAHSPIQSGLPGIQNFRADPEELFTKLERIGKGSFGEVYKGIDNRTQTVVAIKVIDLEEA
EDDIEDIQQEITVLSQCDSPFITKYYGSYLKGTKLWIIMEYLGGGSALDLMEPGALDETQ
IATVLREILKGLEYLHSENKIHRDIKAANVLLSEQGDVKLADFGVAGQLTDTQIKRNTFV
GTPFWMAPEVIKQSAYDSKADIWSLGITAIELAKGEPPHSELHPMKVLFLIPKNNPPTLE
GSYSKPLKEFVEACLNKEPNFRPTAKELLKHKYIVRHAKKTSYLTELIDRFKRWKSEKSR
DESSSDESDGEQDGQASGGGDPDGDKWIFNTIREKDPKKCQNGSAQPEEMETDQVQENPK
RPLSQSLSTIFSPTFTELKAKAGRSNHKPQPAEELQEAIFLAEETYPGICDSLVAELVQR
LQRYSALSGGSSGR

>Gasterosteus aculeatus_STK24(ENSGACP_18309)
MAHSPVQGNLPGMQNTKADPEELFTKLERIGKGSFGEVFKGIDNRTQKVVAIKTIDLEEA
EDEIEDIQQEITVLSQCDSPFVTKYYGSYLKDTKLWIIMEYLGGGSALDLMEPGCLDETQ
IATILREILKGLEYLHSEKKIHRDIKAANVLLSEQGEVKLADFGVAGQLTDTQIKRNTFV
GTPFWMAPEVIKQSAYDSKADIWSLGITAIELAKGEPPHSELHPMKVLFLIPKNTPPTLE
GSYSKPLKEFIEACLNKEPSFRPTAKELLKHKLIVRHAKKTSYLTELVDKYKRWKAEQSR
TAESSSDESDSEQDGQASGGNDFGSDDWIFTIREKDPKKLQNGEGQSGAADQVRASKTKD
IPKRPYSQSLATVISPALAELKARQEQVNGNPMVLDELREAILLAEESYPGIADSLVAHM
VQRLQSFSTSRASASS

>Oryzias latipes_STK24(ENSORLP_3560)
MAHSPVQGNLPGMQNARVDPEELFTKLERIGKGSFGEVFKGIDNRTQKVVAIKIIDLEEA
EDEIEDIQQEITVLSQCDSPFVTKYYGSYLKDTKLWIIMEYLGGGSALDLMEPGPLDETQ
IATILREILKGLEYLHSEKKIHRDIKAANVLLSEQGEVKLADFGVAGQLTDTQIKRNTFV
GTPFWMAPEVIKQSAYDSKADIWSLGITAIELAKGEPPHSDLHPMKVLFLIPKNNPPTLE
GSYCKPLKEFVEACLNKEPSFRPTAKELLKHKLIVRYAKKTSYLTELVDKYKRWKAEQSR
TAESSSDESDSEQDGQASGGNDFGSDDWIFTIREKDPKKLQNGEGQSGVTDQTKDIPKRP
FSQSLAAVISPALAELKARQEKVNGNPMVLDELKEAILLAEEACPGISDSLVAHMVHRLQ
RNMLSMNGVHTR

>Homo sapiens_STK25(ENSP_385687)
MAHLRGFANQHSRVDPEELFTKLDRIGKGSFGEVYKGIDNHTKEVVAIKIIDLEEAEDEI
EDIQQEITVLSQCDSPYITRYFGSYLKSTKLWIIMEYLGGGSALDLLKPGPLEETYIATI
LREILKGLDYLHSERKIHRDIKAANVLLSEQGDVKLADFGVAGQLTDTQIKRNTFVGTPF
WMAPEVIKQSAYDFKADIWSLGITAIELAKGEPPNSDLHPMRVLFLIPKNSPPTLEGQHS
KPFKEFVEACLNKDPRFRPTAKELLKHKFITRYTKKTSFLTELIDRYKRWKSEGHGEESS
SEDSDIDGEAEDGEQGPIWTFPPTIRPSPHSKLHKGTALHSSQKPAEPVKRQPRSQCLST
LVRPVFGELKEKHKQSGGSVGALEELENAFSLAEESCPGISDKLMVHLVERVQRFSHNRN
HLTSTR

>Pan troglodytes_STK25(ENSPTRP_46906)
MAHIRGFANQHSRVDPEELFTKLDRIGKGSFGEVYKGIDNHTKEVVAIKIIDLEEAEDEI
EDIQQEITVLSQCDSPYITRYFGSYLKSTKLWIIMEYLGGGSALDLLKPGPLEETYIATI
LREILKGLDYLHSERKIHRDIKAANVLLSEQGDVKLADFGVAGQLTDTQIKRNTFVGTPF
WMAPEVIKQSAYDFKADIWSLGITAIELAKGEPPNSDLHPMRVLFLIPKNSPPTLEGQHS
KPFKEFVEACLNKDPRFRPTAKELLKHKFITRYTKKTSFLTELIDRYKRWKSEGHGEESS
SEDSDIDGEAEDGEQGPIWTFPPTIRPSPHSKLHKGTALHSSQKPVEPVKRQPRSQCLST
LVRPVFGELKEKHKQSGGSVGALEELENAFSLAEESCPGISDKLMVHLVERVQRFSHNRN

HLTSTR

>Mus musculus_STK25(ENSMUSP_27498)
MAHLRGFAHQHSRVDPEELFTKLDRIGKGSFGEVYKGIDNHTKEVVAIKIIDLEEAEDEI
EDIQQEITVLSQCDSPYITRYFGSYLKSTKLWIIMEYLGGGSALDLLKPGPLEETYIATI
LREILKGLDYLHSERKIHRDIKAANVLLSEQGDVKLADFGVAGQLTDTQIKRNTFVGTPF
WMAPEVIKQSAYDFKADIWSLGITAIELAKGEPPNSDLHPMRVLFLIPKNNPPTLEGHHS
KPFKEFVEACLNKDPRFRPTAKELLKHKFITRYTKKTSFLTELIDRYKRWKSEGHGEESS
SEDSDIDGEAEDGEQGPIWTFPPTIRPSPHSKLHKGTALHSSQKPAEPIKRQPRSQCLST
LVRPVFGELKEKHKQSGGSVGALEELENAFSLAEESCPGISDKLMVHLVERVQRFSHSRN
HLTSTR

>Canis familiaris_STK25(ENSCAFP_8426)
MAHLRGFANQHPRVDPEELFTKLDRIGKGSFGEVYKGIDNRTKEVVAIKIIDLEEAEDEI
EDIQQEITVLSQCDSPYITRYFGSYLKSTKLWIIMEYLGGGSALDLLKPGPLEETYIATI
LREILKGLDYLHSERKIHRDIKAANVLLSEQGDVKLADFGVAGQLTDTQIKRNTFVGTPF
WMAPEVIKQSAYDFKADIWSLGITAIELAKGEPPNSDLHPMRVLFLIPKNSPPTLEGHHS
KPFKEFVEACLNKDPRFRPTAKELLKHKFITRYTRKTSFLTELIDRYKRWKSEGHGEESS
SEDSDIDADAEDGEQGPIWTFPPTIRPSPHSKLHKGTALHGPQKSAEPVKRQPRSQCLST
LVRPVFGELKEKHKQSGGGVGALEELENAFSLAEESCPGISDKLMAHLVERVQRFSHNRN
HLTSTR

>Myotis lucifugus_STK25(ENSMLUP_6530)
MAHLRGFANQHSRVDPEELFTKLDRIGKGSFGEVFKGIDNRTKEVVAIKIIDLEEAEDEI
EDIQQEITVLSQCDSPYITRYFGSYLKSTKLWIIMEYLGGGSALDLLKPGPLEETYIATI
LREILKGLDYLHSERKIHRDIKAANVLLSEQGDVKLADFGVAGQLTDTQIKRNTFVGTPF
WMAPEVIKQSAYDFKADIWSLGITAIELAKGEPPNSDLHPMRVLFLIPKNSPPTLEGHHS
KPFKEFVEACLNKDPRFRPTAKELLKHKFITRYTRKTSFLTELIDRYKRWKSEGHGEESS
SEDSDIDGDAEDGEQGPIWTFPPTIRPSPHSKLHKGTALHGPQKTVESVKRQPRSQCLST
LVRPVFGELKEKHRQSGGGVGALEELENAFSLAEESCPGISDKLMAHLVERVQRFSHNRN
HLPSAR

>Ornithorhynchus anatinus_STK25(ENSOANP_18636)
MAHLREFANQHSRVDPEELFTKLERIGKGSFGEVYKGIDNRTKEVVAIKIIDLEEAEDEI
EDIQQEITVLSQCDSPYITRYFGSYLKSTKLWIIMEYLGGGSALDLLKPGPLEETYIATI
LREILKGLDYLHSERKIHRDIKAANVLLSEQGDVKLADFGVAGQLTDTQIKRNTFVGTPF
WMAPEVIKQSAYDFKADIWSLGITAIELAKGEPPNSDLHPMRVLFLIPKNNPPTLEGHHS
KPFKEFVEACLNKDPRFRPTAKELLKHKFITRYTKKTSFLMELIDRYKRWKSEGHGEDSS
SEDSDIDGDAEDGDQGPIWTFPPTIRPSPLNKLHKGMALNGSQKSAEPIKRQPRSQCLST
LVRPVFGELKDKHKQTGGNVGAMEELENAFSLAEESCPGISDKLIAHMVERVQRFSHSRN
HLTSTR

>Gallus gallus_STK25(ENSGALP_9265)
XPADRAGMAHLRDFASQHSRVDPEELFTKLERIGKGSFGEVYKGIDNRTKEVVAIKIIDL
EEAEDEIEDIQQEITVLSQCDSPYITRYYGSYLKGTKLWIIMEYLGGGSALDLLKPGPLE
ETYIATILREILKGLDYLHSERKIHRDIKAANVLLSEQGDVKLADFGVAGQLTDTQIKRN
TFVGTPFWMAPEVIKQSAYDFKADIWSLGITAIELAKGEPPNSDLHPMRVLFLIPKNNPP
TLEGHHSKPFKEFVEACLNKDPRFRPTAKELLKHKFITRYTKKTSFLMELIDRFKRWKSE
GHGEDSSSGDSDIDGDTEDGDQGPIWTFPPTIRPNSLSKLHKGMALHGSQKPSEPIKRQP
RSQCLSTLVHPVFGELKDKHKQTGGNVGAMEELENAFSLAEESCPGISDKLIAHMVERVQ
RFSHSRNHLTSAR

>Anolis carolinensis_STK25(ENSACAP_1074)
MAHLREFANQHSRVDPEELFTKLERIGKGSFGEVYKGIDNRTKEVVAIKIIDLEEAEDEI
EDIQQEITVLSQCDSPYITRYYGSYLKGTKLWIIMEYLGGGSALDLLKPGPLEETYIATI
LREILKGLDYLHCERKIHRDIKAANVLLSEQGDVKLADFGVAGQLTDTQIKRNTFVGTPF
WMAPEVIKQSAYDFKADIWSLGITAIELAKGEPPNSDLHPMRVLFLIPKNNPPTLEGHHS
KPFKEFVEACLNKDPRFRPTAKELLKHKFITRYTKKTSFLMELIDRYKRWKSEGHGEDSS
SDDSDIDGDADERDQGPMWTFPPTIRPNSLNKLHKGLSHHGSQKPAEPIKRQPRSQSLST
LVRPVFGELKDKHKQTGGNVGAMEELENAFGLAEESCPGISDKLIAHMVERVQRFSHSRN
HLTSAR

>Xenopus tropicalis_STK25(ENSXETP_62780)
VDYPLVVILIVNSLHFQSSRMDPERLFTKLERIGKGSFGEVYKGIENRSKEVVAIKIIDL
EEAEDEIEDIQQEITVLSQCDSPYITRYYGSYLKGSKLWIIMEYLGGGSALDLLKPGPLE
EAYIATILREILKGLDYLHSERKIHRDIKAANVLLSEQGDVKLADFGVAGQLTDTQIKRN
TFVGTPFWMAPEVIKQSAYDFKADIWSLGITAIELAKGEPPYSDLHPMRVLFLIPKNNPP
SLQGQYSKPFKDFVEACLNKDPRSRPTARELLKHRFILRYTKKTSFLVELVERHKRWKSQ
GHREDSSSDESDMDNEDERNQNQIPIWSFPETICAKSMSRMQWGCQKSAEVIHRLPRSHC
LSALVTPVFQELREQRSAGAFGSVQELENAFSLAEESSPGITDQLISHLVQRVQSFIANY
S

>Danio rerio_STK25a(ENSDARP_56962)
MAHLRDMQNQNSRLDPEEYFTKQERIGKGSFGEVYKGINNRTKEVVAIKIIDLEEAEDEI
EDIQQEITVLSQCDSPYVTKYYGSYLKGTKLWIIMEYLGGGSALDLLKPGPLEETYIATI
LREILKGLEYLHSERKIHRDIKAANVLLSEQGDVKLADFGVAGQLTDTQIKRNTFVGTPF
WMAPEVIKQSAYDFKADIWSLGITAIELAKGEPPNSDLHPMRVLFLIPKNTPPTLEGSYS
KPFKEFVEACLNKDPRFRPTAKELLKHKFITRYTKKTSYLTELIDRYRRWKSEGHGDESS
SDDSDMDGDGDDRDHCPVWTFPTVRPSSMNKLQKGYTNTDSEAAETVKRQPKSQCLSALV
SPIFRELKEKRRACGGGMGAIEELENAFNLAEESCPGISDLLVTHIMERVCRFALNGNTT
PSSR

>Danio rerio_STK25b(ENSDARP_123777)
MAHLQNIRNQNSRLNPEEYFTKLERIGKGSFGEVYKGINNRTKEVVAIKIIDLEEAEDEI
EDIQQEITVLSQCDSPYVTKYYGSYLTGSKLWIIMEYLGGGSALDLLRPGTLEEVYIATI
LREILKGLDYLHSERKIHRDIKAANVLLSEHGEVKLADFGVAGQLTDTQIKRNTFVGTPF
WMAPEVIKQSAYDFKADIWSLGITAIELAKGEPPNAELHPMRVLFLIPKNNPPTLEGSYS
KAFKDFVEACLNKEPRFRPTAKELLKHKFITRYTKKTSYLSELIDRYRRWKSEGHGEESS
SDDSDMDGEGDDRDQGPMWTFPTVRPSTINKLNRGLSHLDNKTSDVMKRQPKSSSLTSFI
TPIFKELKEKRRVGGNGVCAVEELENAFSLAEESSPGISDRLLNHVMERVCSFSGHADSM
L

>Takifugu rubripes_STK25(ENSTRUP_963)
GMAHLRDMQNQNTRLDPEEYFTKQERIGKGSFGEVYKGINNRTKEVVAIKIIDLEEAEDE
IEDIQQEITVLSQCDSPFVTKYYGSYLKGTKLWIIMEYLGGGSALDLLRPGPLEETYIAT
ILREILKGLEYLHSERKIHRDIKAANVLLSEQGDVKLADFGVAGQLTDTQIKRNTFVGTP
FWMAPEVIKQSAYDFKADIWSLGITAIELAKGEPPNSDLHPMRVLFLIPKNTPPTLEGPY
SKPFKEFIEACLNKDPRFRPTAKELLKHKFITRYTKKTAYLTELIDRYRRWKSEGHGEES
SSDDSDMDADGDVDTCPMWTFPTVRPTSMNKLQKGYTHTDSESGDSVKRQPKSQSLSALV
TPIFRELKEKRRASGGGVGAIEELENAFNLAEESCPGISDRLVTHMMERVCRFSLNGNTT
PSSR

>Tetraodon nigroviridis_STK25(ENSTNIP_10668)
MAHLRDMQNQNTRLDPEEYFTKQERIGKGFGEVYKGINNRTKEVVAIKIIDLEEAEDEIE
DIQQEITVLSQCDSPFVTKYYGSYLKGTKLWIIMEYLGGGSALDLLRPGPLEETYIATIL
REILKGLEYLHSERKIHRDIKAANVLLSEQGDVKLADFGVAGQLTDTQKRNTFVGTPFWM
APEVIKQSAYDFKVSLWAEGITAIELAKGEPPNSDLHPMRVLFLIPKNTPPTLEGPYSKP
FKEFVEACLNKDPRFRPTAKELLKHKFITRYTKKTAYLTELIDYRRWKSEGHGEESSSDD
SDMDADGDVDTCPMWTFPTVRPTSMNKLQKGYTDTDSESGDSVKRQPKSQSLSALVTPIF
RELKEKRRASGGGVGAIEELENAFNLAEESCPGISDRLVTHMMERVCRFSLNGNTTPSSR

>Gasterosteus aculeatus_STK25(ENSGACP_20761)
MAHLRDMQNQNTRLDPEEYFTKQERIGKGSFGEVYKGINNRTKEVVAIKIIDLEEAEDEI
EDIQQEITVLSQCDSPFVTKYYGSYLKGTKLWIIMEYLGGGSALDLLRPGPLEETYIATI
LREILKGLEYLHSERKIHRDIKAANVLLSEQGEVKLADFGVAGQLTDTQIKRNTFVGTPF
WMAPEVIKQSAYDFKADIWSLGITAIELAKGEPPNSDLHPMRVLFLIPKNTPPTLEGPYS
KPFKEFVEACLNKDPRFRPTAKELLKHKFITRYTKKTAYLTELIDRYRRWKSEGHGEESS
SDDSDMDADGDGDTCPMWTFPTVRPSSLNKLQKGYTHTDSESGDLVKRQPKSQCLSALVT
PIFRELKEKRRASGGGVGAIEELENAFNLAEESCPGISDLLVTHMMERVCRFSLNGNTTQ
SSR

>Oryzias latipes_STK25(ENSORLP_6042)

PATGRTMAHLRDMQNQNTHLDPEEYFTKQERIGKGSFGEVYKGINNRTKEVVAIKIIDLE
EAEDEIEDIQQEITVLSQCDSPYVTKYYGSYLKGTKLWIIMEYLGGGSALDLLRPGPLEE
TYIATILREILKGLEYLHSERKIHRDIKAANVLLSEQGDVKLADFGVAGQLTDTQIKRNT
FVGTPFWMAPEVIKQSAYDFKADIWSLGITAIELAKGEPPNSDLHPMRVLFLIPKNTPPT
LEGPYSKPFKEFVEACLNKDPRFRPTAKELLKHKFITRYTKKTAYLTELIDRYRRWKSEG
HGEESSSDDSDMDADGDGEQCPVWTFPTVRPSSMSQLQMDYAHLESGSGDSMKRQPKSQC
LSALVTPIFRELKDKRRASGGGAGAIEELENAFNLAEESCPGISDRLVTHMMERVCRFSL
NGNTAPSSR

>Homo sapiens_STK26(ENSP_377867)
MAHSPVAVQVPGMQNNIADPEELFTKLERIGKGSFGEVFKGIDNRTQQVVAIKIIDLEEA
EDEIEDIQQEITVLSQCDSSYVTKYYGSYLKGSKLWIIMEYLGGGSALDLLRAGPFDEFQ
IATMLKEILKGLDYLHSEKKIHRDIKAANVLLSEQGDVKLADFGVAGQLTDTQIKRNTFV
GTPFWMAPEVIQQSAYDSKADIWSLGITAIELAKGEPPNSDMHPMRVLFLIPKNNPPTLV
GDFTKSFKEFIDACLNKDPSFRPTAKELLKHKFIVKNSKKTSYLTELIDRFKRWKAEGHS
DDESDSEGSDSESTSRENNTHPEWSFTTVRKKPDPKKVQNGAEQDLVQTLSCLSMIITPA
FAELKQQDENNASRNQAIEELEKSIAVAEAACPGITDKMVKKLIEKFQKCSADESP

>Pan troglodytes_STK26(ENSPTRP_38373)
MAHSPVAVQVPGMQNNIADPEELFTKLERIGKGSFGEVFKGIDNRTQQVVAIKIIDLEEA
EDEIEDIQQEITVLSQCDSSYVTKYYGSYLKDHGISRHLKFQQMEMLLHSRKLGSKLWII
MEYLGGGSALDLLRAGPFDEFQIATMLKEILKGLDYLHSEKKIHRDIKAANVLLSEQGDV
KLADFGVAGQLTDTQIKRNTFVGTPFWMAPEVIQQSAYDSKADIWSLGITAIELAKGEPP
NSDMHPMRVLFLIPKNNPPTLVGDFTKSFKEFIDACLNKDPSFRPTAKELLKHKFIVKNS
KKTSYLTELIDRFKRWKAEGHSDDESDSEGSDSESTSRENNTHPEWSFTTVRKKPDPKKV
QNGAEQDLVQTLSCLSMIITPAFAELKQQDENNASRNQAIEELEKSIAVAEAACPGITDK
MVKKLIEKFQKCSADESP

>Mus musculus_STK26(ENSMUSP_33444)
MAHSPVAVQVPGMQNNIADPEELFTKLERIGKGSFGEVFKGIDNRTQQVVAIKIIDLEEA
EDEIEDIQQEITVLSQCDSSYVTKYYGSYLKGSKLWIIMEYLGGGSALDLLRAGPFDEFQ
IATMLKEILKGLDYLHSEKKIHRDIKAANVLLSEQGDVKLADFGVAGQLTDTQIKRNTFV
GTPFWMAPEVIQQSAYDSKADIWSLGITAIELAKGEPPNSDMHPMRVLFLIPKNNPPTLI
GDFTKSFKEFIDACLNKDPSFRPTAKELLKHKFIVKNSKKTSYLTELIDRFKRWKAEGHS
DEESDSEGSDSESSSRESNPHPEWSFTTVRKKPDPKKLQNGEEQDLVQTLSCLSMIITPA
FAELKQQDENNASRNQAIEELEKSIAVAETACPGITDKMVKKLIEKFQKCSADESP

>Canis familiaris_STK26(ENSCAFP_27770)
MAHSPVAVQVPGMQNNIADPEELFTKLERIGKGSFGEVFKGIDNRTQQVVAIKIIDLEEA
EDEIEDIQQEITVLSQCDSSYVTKYYGSYLKGSKLWIIMEYLGGGSALDLLRAGPFDEFQ
IATMLKEILKGLDYLHSEKKIHRDIKAANVLLSEQGDVKLADFGVAGQLTDTQIKRNTFV
GTPFWMAPEVIQQSAYDSKADIWSLGITAIELAKGEPPNSDMHPMRVLFLIPKNNPPTLV
GDFTKSFKEFIDACLNKDPSFRPTAKELLKHKFIVKNSKKTSYLTELIDRFKRWKAEGHS
DDESDSEGSDSESTSRENNTHPEWSFTTVRKKPDPKKLQNGAEQDLVQTLSCLSMIITPA
FAELKQQDENNASRNQAIEELEKSIAVAEAACPGITDKMVKKLIEKFQKCSADESP

>Myotis lucifugus_STK26(ENSMLUP_1350)
KAHVKSQGNLYALKNNIADPEELFTKLERIGKGSFGEVFKGIDNRTQQVVAIKIIDLEEA
EDEIEDIQQEITVLSQCDSSYVTKYYGSYLKGSKLWIIMEYLGGGSALDLLRAGPFDEFQ
IATMLKEILKGLDYLHSEKKIHRDIKAANVLLSEQGDVKLADFGVAGQLTDTQIKRNTFV
GTPFWMAPEVIQQSAYDSKADIWSLGITAIELAKGEPPNSDMHPMRVLFLIPKSNPPTLV
GEFTKSFKEFIDACLNKDPSFRPTAKELLKHKFIVKNSKKTSYLTELIDRFKRWKAEGHS
DDESDSEGSDSESTSRENNTHPEWSFTTVRKKPDPKKLQNGALEIHIYPKNKEKDLVQTL
SCLSMIITPAFAELKQQDENNASRNQAIEELEKSIAVAEAACPGITDKMVKKLIEKFQK

>Monodelphis domestica_STK26(ENSMODP_19135)
MAHSPVAVQVPGMQAGAASSSSWNTNQRTDPEELFTKLERIGKGSFGEVFKGIDNRTQQV
VAIKIIDLEEAEDEIEDIQQEITVLSQCDSSYVTKYYGSYLKGTKLWIIMEYLGGGSALD
LLRAGPFDEFQIATMLKEILKGLDYLHSEKKIHRDIKAANVLLSEQGEVKLADFGVAGQL
TDTQIKRNTFVGTPFWMAPEVIQQSAYDSKADIWSLGITAIELAKGEPPNSDMHPMRVLF
LIPKNNPPTLLGDFTKPFKEFTDACLNKDPSFRPTAKELLKHKFIIKNAKKTSYLTELID

RFKRWKAEGHSDDESDSDGSDSESSNKENNSHPEWSFTTVRKKPDAKKLQNGTEQEEELA
KTLSCLSTIITPVFAELKEQDTSNASRTKAIEELEKSMTVAETACPGITDKMVKKLIEKF
QKFSVNDSS

>Gallus gallus_STK26(ENSGALP_9728)
MAHSPVAVQVPGMQNHRADPEELFTKLERIGKGSFGEVFKGIDNRTQQVVAIKIIDLEEA
EDEIEDIQQEITVLSQCDSPYVTKYYGSYLKGTKLWIIMEYLGGGSALDLLRAGPFDEFQ
IATMLKEILKGLDYLHSEKKIHRDIKAANVLLSEQGDVKLADFGVAGQLTDTQIKRNTFV
GTPFWMAPEVIQQSAYDSKADIWSLGITAIELAKGEPPNSDMHPMRVLFLIPKNNPPTLL
GEFSKPFKEFIDACLNKDPTFRPTAKELLKHKFIMKNAKKTSYLTELIDRFKRWKAEGHS
SDESDSDGSDSESSNKENNSHPEWSFTTVRKKPDAKKLQNGTDQDLVKTLSCLTMIITPV
FAELKQQDTNNASRKKAIEELEKSINMAEATCPGITDKMVKKLMEKFQKFSVNDSS

>Anolis carolinensis_STK26(ENSACAP_21957)
MAHSPVAVQVPGMQNHRADPEELFTKLERIGKGSFGEVFKGIDNRTQQVVAIKIIDLEEA
EDEIEDIQQEITVLSQCDSPYVTKYYGSYLKGTKLWIIMEYLGGGSALDLLRAGPFDEFQ
IATMLKEILKGLDYLHSEKKIHRDIKAANVLLSEQGDVKLADFGVAGQLTDTQIKRNTFV
GTPFWMAPEVIQQSAYDSKADIWSLGITAIELAKGEPPNSDMHPMRVLFLIPKNNPPTLI
GDFSKPFKEFIDACLNKDPTFRPTAKELLKHKFILKNAKKTSYLTELIDRFKRWKAEGHS
SDETDSDGSDSESSNKENNSHPEWSFTTVRKKPDAKKLQNGTDQDLMKTLSCLSMIITPV
FAELKQQDTNNASRKKAIEELEKSISLAETTCPGITDKMVKKLMEKFQKFSVNESS

>Xenopus tropicalis_STK26(ENSXETP_11925)
ISHSPLRYVYKVPLIQNHRADPEELFTKLERIGKGSFGEVFKGIDNRTQHVVAIKIIDLE
EAEDEIEDIQQEITVLSQCDSPYVTKYYGSYLKVLNFWWITLIYYMGGRTKRAKDLLRVG
PFDEFQIATMLKEILKGLDYLHSENKIHRDIKAANVLLSEQGDVKLADFGVAGQLTDTQI
KRNTFVGTPFWMAPEVIQQSAYDSKADIWSLGITAIELAKGEPPNSDMHPMRVLFLIPKN
NPPSLTGDFSKPFKEFVDACLNKDPSFRPTARELLKHKYIVKNAKRTSYLTELIDRFKRW
KAEGHSDSGSDDSDTESNNKENHTHPEWSFTTVRKKPESKTMQNGTDQDLVKTLSSLSAI
ISPVFAELKQQDRNNTVRKNAIEELEKSINVAEATYPGITDKMVKKLMEKFQNTLKCSTH
SSS

>Danio rerio_STK26(ENSDARP_79474)
MAQSPVAVQVPGMQNSRTDPEELFTKLDRIGKGSFGEVFKGIDRQTQNVVAIKTIDLEEA
EDEIEDIQQEITVLSQCDSPHVTKYYGSYLKGSKLWIIMEYLGGGSALDLLRAGPFDEYQ
IATMLKEILKGLDYLHSEKKIHRDIKAANVLLSESGEVKLADFGVAGQLTDTQIKRETFV
GTPFWMAPEVIQQSAYDSKADIWSLGITAIELAKGEPPNSDMHPMRVLFHIPKNTPPTLN
GDFSKIFKEFVDSCLNKDPAFRPTAKELLKHKFIVKYAKKTSYLTELIDRLKRWKAEGHS
DGESSSDSDSETSSKDNDSSPEWSFTTVRKKKTEKKQPNGTDEELQKKSASLTTVMAPVF
SELKSQHRDNERTKGVIEMLEESLHTAEDLCPGVTDRMITQILDRVQRFSMS

>Takifugu rubripes_STK26(ENSTRUP_15949)
LRHAPQLVKFAHTQSTQIDPEELFTKLDRIGKGSFGEVFKGIDNRTQSVVAIKTIDLEEA
EDEIEDIQQEITVLSQCDSPYITKYYGSYLKGTKLWIIMEYLGGGSALDLLRAGPFDESQ
IATMLKEILKGLDYLHSEKKIHRDIKAANVLLSEHGQVKLADFGVAGQLTDTQIKRETFV
GTPFWMAPEVIQQSAYDSKADIWSLGITAIELAKGEPPNSDMHPMRVLFHIPKFPAPTLT
GDFSKSFKEFTEACLNKDPAFRPTAKELLKHKFIVKHCKKTSYLSELIDRYRRWKEQGHS
DSSSDDSDSESSNKENNESPEWSFTTVRKKKTEGRKVLNGTSPDQMSKSASLTTVISPVF
TELKQQHKEHSQQRLAIEELERNIRLAEDVCPGITDRMVSHIIARYQKPGLKDNV

>Tetraodon nigroviridis_STK26(ENSTNIP_14178)
CPYFKVTMEVPGMQSTQVDPEELFTKLDRIGKGSFGEVFKGIDNRTQSVVAIKTIDLEEA
EDEIEDIQQEITVLSQCDSPYITKYYGSYLKGTKLWIIMEYLGGGSALDLLRAGPFDEFQ
IATMLKEILKGLDYLHSEKKIHRDIKAANVLLSEHGQVKLADFGVAGQLTDTQIKRETFV
GTPFWMAPEVIQQSAYDSKADIWSLGITAIELAKGEPPNSDMHPMRVLFHIPKFPAPTLT
GEFSRNFKEFTEACLNKDPAFRPTAKELLKHKFIVKHCKKTSYLSELIDRYRRWKEEGHS
DSSSDDSDSESSNKENNESPEWSFTTVRKKKTEGRKVLNGTSPDQMSKSASLTTVISPVF
TELKQQHKEHSEQRLAIEELERNIRLAEEVCPGITDRMVSHIIARYQK

>Oryzias latipes_STK26(ENSORLP_4136)
INVLFFFVFQSSQIDPEELFTKLDRIGKGSFGEVFKGIDNRSQSVVAIKTIDLEEAEDEI

EDIQQEITVLSQCDSPYITKYFGSYLKGSKLWIIMEYLGGGSALDLLRAGPFDESQIATM
LKEILKGLDYLHSEKKIHRDIKAANVLLSEHGQVKLADFGVAGQLTDTQIKRETFVGTPF
WMAPEVIQQSAYDSKADIWSLGITAIELAKGEPPNSDMHPMRVLFHIPKSPPPTLSGDFS
KSFKEFTEACLNKDPAFRPTAKELLKHKFITKHCKKTSYLSELIDRYRRWKEEGHSGSSS
DDSDSVSSESSNKENDMHPEWSFTTVRKKKTEGRMVLNGTLQTPEQLSKSASLSTVISPV
FTELKQQHKEHSTQRLAIEQLEHNIQLAEDICPGITDKMVTHIIARCQKYDHRHSVLKDA
V

>Branchiostoma floridae_(XP_002589064)
SELKKLSEESLTRQPEEVFDVLEKLGEGSYGSVFKAMHKESGQVLAIKQVPVDTDLQEIIKEISIMQQCD
RKLLNNGCGTLFYIHVLVIVMEYCGAGSVSDIMKLRNKTLTEDEIAAITSMVLKGLEYLHFMRKIHRDIK
AGNILLNTEGDAKLADFGVAGQLTDTMAKRNTVIGTPFWMAPEVIQEIGYDCLADIWSLGITALEMAEGK
PPYADIHPMRAIFMIPTKPPPTFRNPEKWSQEFIDFVSQCLVKNPQQRPSATQLLQHPFIRAAKPAYILK
EMISEAQVARQRRMEQVEEESPEEEEDDDVDASTMLLGEDNDTGTIKATMVESSTAGTMIEHSDSTMLES
DLGTMVINSDEDTDEDSTMRKTGKVKKEKYRPSFLDHFEKKEQDERDRQSAANGNITAEIQPGPVLRPAS
EGYGFVRPFQDGDFEFLKNLSSDELRRRFETLDQEMEREIEELRRRYQAKRQPILNAMDAKKKRQQHF

>Strongylocentrotus purpuratus_(XP_011667292)
MTSTSTGTCMLKKLSEDALTKEPEEVFDLLEKLGEGSYGSVFKAIHKESGQVLAIKQVPVDTDLQEIIKE
ISIMQQCDSTYVVKYYGSYFKNTDLWIVMEYCGAGSVSDIMRRRNKTLNEAEIATILYSTLKGLEYLHFM
RKIHRDIKAGNILLNNEGNAKLADFGVAGQLTDTMAKRNTVIGTPFWMAPEVIQEIGYDCKADIWSLGIT
ALEMAEGKPPYAEIHPMRAIFMIPTKPPPTFRDPEKWSQDFIDFTSKCLIKSPEDRATATDLLQHPFVRD
AKPVSILNAIINEANCIREREKLKEDDDDEGSDFEDDMYTIVEEEEDTEGRLDEDDVDSGTMVRGRPPVM
NEDDSGTIIISGSAGNTANTFIEHDDVTDIDSGTMIEHGTMIDHDSGTMIEHGTMIDHDSGTMVDHDSGT
MIDHDSGTMVDHHSGTMIDHDSGTMVDHDSGTMINHGSLNDLESNMGTMVINDDDDSGNDGTMKRTDTAK
ADAYKPQYLEHYERQEEAKQAKISSPVTPPINPSNAPTVPSNAPMHIAPGNHRLKQLSIDELRERLTSLD
PQMEAEIEELRKRYQEKRRPIIEAMEVKRKRTQHF

>Drosophila melanogaster_(FBpp0082840)
MSWTEKVDPELIFTKQERIGKGSFGEVFKGIDNRTQQVVAIKIIDLEEAEDEIDDIQQEI
MVLSQCDSPYVTKYYGSFLKGTKLWIIMEYLGGGSALDLMKAGSFEEMHIGIILREVLKG
LDYLHSERKLHRDIKAANVLLSEQGDVKLADFGVAGQLTNTTSKRNTFVGTPFWMAPEVI
KQSQYDAKADIWSLGITAIELAKGEPPNSELHPMRVLFLIPKNNPPQLTGSYTKSFKDFV
EACLNKDPENRPTAKELLKYPFIKKAKKNAYLIDLIDRFKKWKVSKGDESETESENSDSD
SDAKQGGSSQDEPWIMTVKGLHINSAPRAGVNSFQLQEHELTMQKLMQTTHSPAGGGGVQ
ASPAANALSSSVSAVAASASSVSVITANEGVSSASALPPQNSHVHNHNHNNINNNNIRNL
SNKHATTTMLNAAPPPSSVTSSSSASELQQKRSSSKPELRQSRSAAPLDQVEDRRASLGL
PLQSSVPTPSTAEQQQQRRSSQRESFHANNAALVASCSSSSNNHSHEEHVATNHASHVKH
SGAGQRLVQTNSACLNNFLFPVLSDIQRKYSGGGSAGSGGSGGRNIGVGSDIGDLKSVFE
LAERSSPGVSDLFMKELIHMLVPGYSETRINTIMDRAIRNRK

>Apis mellifera_(XP_016771231)
MAFNEKVDPELIFTKQERIGKGSFGEVFKGIDNRTQQVVAIKIIDLEEAEDEIEDIQQEIMVLSQCDSPY
VTKYYGSYLKGTKLWIIMEYLGGGSALDLMKAGNFEEMHIAVILREVLKGLDYLHSERKLHRDIKAANVL
LSEMGDVKLADFGVAGQLTNTTSKRNTFVGTPFWMAPEVIKQASYDSKADIWSLGITAIELAKGEPPNSE
LHPMRVLFLIPKNNPPQLTGNYTKQFKEFVEACLNKDPENRPTAKELLKFQFIRKAKKNSYLIDLIDRYK
KWKSQRSEESETESENSDSEESKQDSDMDDPWIMTVKGPHVVKGPVVPMLPLDEQPPSLTLTHTPNHRSA
NHNQQAKPHANGTSRSPPNDSTLNHYRSESRDSVRDGQAKHTPSRPHEAAPPPPVDTREKREDYRDFNRD
SSLRELKEMRESRESRDRERDREGRERERDREREFSAKEKRNSKPDVPVVPMVDHRPGEDKQRPRSSQEL
KHSRSAALSGVVLPLLSELQRKHQYSARDNNGEGGSGIGKNGGAIEELRSAFETAERTSPGMTEALIREL
LKSLLPANHSEGCLFMLMEKVILRDT

>Anopheles gambiae_(XP_319097)
SLPQKVDPELIFTKQERIGKGSFGEVFKGVDNRTQQVVAIKIIDLEEAEDEIEDIQQEIMVLSQCDSPFV
TKYFGSYLKGTKLWIIMEYLGGGSALDLMKAGLFEEMHIAIILREVLKGLDYLHSERKLHRDIKAANVLL
SELGDVKLADFGVAGQLTNTTSKRNTFVGTPFWMAPEVIKQSMYDSKADIWSLGITAIELAKGEPPNSEL
HPMRVLFLIPKNNPPQLTGSYTKPFKDFVEACLNKDPENRPTAKELLKFPFIKKAKKNSYLIDLIDRYKK
WKSQKGEESETESENSDSEDSKQDSDLDEPWIMTVKGLNFNNFNKNPRPSSSPSHQSSSTLIAANNATNT
SPIIVDSRKERELQNQISALQLQKEKEASLERQQRDHRLERESEWEKDREKERERERDRERERDRERERD
RERERDRERERERERDRDRDRERDRERERDRDRERDRDHRERIDRDHREKDRDNKERKMVASDSLSGSGN
ENEKRSSSLRRERDSPSHNNTSSNGNSRTNSITNVNNNSSKSSSRSSSTTTPTTTATSTNSGCLKAIIFP

LLSEVSIDGWLGNRGYEMQRNYNSGSDMGDLRLAFESAERSSPGVSDLFVKEIIHKLVPGFSETRINKLI
DKVTR

Transcription Elongation factor A (SII) Protein-TCEA

>Homo sapiens_TCEA3(ENSP_406293)
MGQEEELLRIAKKLEKMVARKNTEGALDLLKKLHSCQMSIQLLQTTRIGVAVNGVRKHCS
DKEVVSLAKVLIKNWKRLLDSPGPPKGEKGEEREKAKKKEKGLECSDWKPEAGLSPPRKK
REDPKTRRDSVDSKSSASSSPKRPSVERSNSSKSKAESPKTPSSPLTPTFASSMCLLAPC
YLTGDSVRDKCVEMLSAALKADDDYKDYGVNCDKMASEIEDHIYQELKSTDMKYRNRVRS
RISNLKDPRNPGLRRNVLSGAISAGLIAKMTAEEMASDELRELRNAMTQEAIREHQMAKT
GGTTTDLFQCSKCKKKNCTYNQVQTRSADEPMTTFVLCNECGNRWKFC

>Pan troglodytes_TCEA3(ENSPTRP_44770)
MGQEEELLRIAKKLEKMVARKNTEGALDLLKKLHSCQMSIQLLQTTRIGVAVNGVRKHCS
DKEVVSLAKVLIKNWKRLLDSPGPPKGEKGEEREKAKKKEKGLECSDWKPEAGLSPPRKK
REDPKTRRDSVDSKSSASSSPKRPSVERSNSSKSKAESPKTPSSPLTPTFASSMCLLAPC
YLTGDSVRDKCVEMLSAALKADDDYKDYGVNCDKMASEIEDHIYQELKSTDMKYRNRVRS
RISNLKDPRNPGLRRNVLSGAISAGLIAKMTAEEMASDELRELRNAMTQEAIREHQMAKT
GGTTTDLFQCSKCKKKNCTYNQVQTRSADEPMTTFVLCNECGNRWKFC

>Mus musculus_TCEA3(ENSMUSP_99592)
MGLEEELLRIAKKLEKMVSRKKTEGALDLLKKLNSCQMSIQLLQTTRIGVAVNGVRKHCS
DKEVVSLAKVLIKNWKRLLDSPRTTKGEREEREKAKKEKGLGCSDWKPEAGLSPPRKKGG
GEPKTRRDSVDSRSSTTSSPKRPSLERSNSSKSKVETPTTPSSPSTPTFAPAVCLLAPCY
LTGDSVRDKCVEMLSAALKAEDNFKDYGVNCDKLASEIEDHIYQELKSTDMKYRNRVRSR
ISNLKDPRNPGLRRNVLSGAISPELIAKMTAEEMASDELRELRNAMTQEAIREHQMAKTG
GTTTDLLRCSKCKKKNCTYNQVQTRSADEPMTTFVLCNECGNRWKFC

>Rattus norvegicus_TCEA3(ENSRNOP_15776)
MGLEEELLRIAKKLEKMVARKKTEGALDLLKKLNSCQMSIQLLQTTRIGVAVNGVRKHCS
DKEVVSLAKVLIKNWKRLLDSPRTTKGEREAREKAKKKEKGLGSSGWKPEAGLSPPRKKE
GGEPKTRRDSVDSRSSTTSSPKRPSLERSNSSRSKVETPKTPSSPSTPTFAPAVCLLASC
YLTGDSVRDKCVEMLSAALKAEDDFKDYGVNCDKLASEIEDHIYQELKSTDMKYRNRVRS
RISNLKDPRNPGLRRNVLSGTISPELIAKMTAEEMASDELRELRNAMTQEAIREHQMAKT
GGTTTDLLRCSKCKKKNCTYNQVQTRSADEPMTTFVLCNECGNRWKFC

>Canis familiaris_TCEA3(ENSCAFP_19574)
MGQEEELLRIARKLEKMVARKNSEGALGLLKKLNSCQMSIQLLQTTRIGVAVNGVRKHCS
DKEVVSLAKVLIKNWKRLLDSPGPPKGEKGEERDKAKKEKGLDCSNWKPETGLSPPRKKR
AEEPKERRDSVDSKSSATSSPKRPSMERSNSGKSKAETPKTPTSPSTPTFAPSVCLLAPC
YLTGDSVRDKCVEMLSAALKAEDDYKDYGVNCDKMASEIEDHILEELKSTDMKYRNRVRS
RISNLKDPRNPSLRRNVLSGAISAGLIAKMTAEEMASDELRELRNAMTQEAIREHQMAKT
GGTTTDLFQCSKCKKKNCTYNQVQTRSADEPMTTFVLCNECGNRWKVC

>Felis catus_TCEA3(ENSFCAP_10810)
MGQEEELLRIARKLEKMVARKNTEGALDLLKKLDSCQMSIRLLQTTRIGVAVNGVRKHCS
DKEVVSLAKVLIKNWKRLLDSPGPPKGEKREERDKAKKEKGLNCSDWKPEAGLSPPRKKR
EDPKDRRDSVDSKSSATSSPKRPSMERSNSSKSKAETPKTPTSPSTPTFAPSICLLAPCY
LTGDSVRDKCVEMLSAALKAEDDYKDYGVNCDKMASEIEDHILEELKSTDMKYRNRVRSR
ISNLKDPRNPSLRRNVLSGAISAGLIAKMTAEEMASDELRELRNAMTQEAIREHQMAKTG
GTTTDLFQCSKCKKKNCTYNQVQTRSADEPMTTFVLCNECGNRWKVC

>Myotis lucifugus_TCEA3(ENSMLUP_3196)
MGREEELLRIAKKLEKMVARKHTEGALDLLKKLNSCQMSIQLLQTTRIGVAVNGVRKYCS
DKEVVSLAKVLIKKWKRLLDSPESPKGGKEEEREKAKKKEKELDCSNWKSEAGLSPPRTK
QGEEPKDRRDSVDSKSSSATSSPKRPSMERSNSGKSKAETSPKTPSSPSSPTFASSICLL
APCYLTGDSVRDKCVEMLSAALKADDDYKDYGVNCDKMASEIEDHILEELKSTDMKYRNR
VRSRISNLKDPRNPGLRRNVLSGAISTGLIAKMTAEEMASDELRELRNAMTQEAIREHQM
AKTGGTTTGLFQCKKCKKKNCTYNQVQTRSADEPMTTFVLCNECGNRWKVC

>Monodelphis domestica_TCEA3(ENSMODP_20168)
MGQEEELLRIAKKLDKMVSRKNTEGALDLLKKLSDYKMSIQLLQTTRIGIAVNGVRKHCL
DKEVVALAKILIKNWKRLLDTSETPKKEKAMEREKEKKKEQNVDLSDWKPLEAFSPQSRS
PREEPKDRRDSSESGSSSSVVSSPSPQKRPVERSNSSKGKVETPKTPTSPSSPTFSPSAC
CLAPCYLTGDSVRDKCVEMLSAALKMDDDYKEYGVNCDKMASEIEDHIFQELKGTDMKYR
NRVRSRISNLKDPRNPNLRRNVLCGAISTSLIARMTAEEMASDELKELRNAMTLEAIREH
QMAKTGGTTTDLFQCISVKRRIVPITRCRQEVLMSP

>Taeniopygia guttata_TCEA3(ENSTGUP_953)
MGPAEELVRIAKKLDKMVARKSTEGALDLLKSLTGYTMTIQLLQTTRIGVAVNSVRKHCS
DEEVVASAKILIKNWKRLLGDPNAFWGDREKDTDGDKEKKGLDVSSCPTTSGTAPGQFLK
EGEPLTGRASADSRSSTLSSSSSSQKRLANSSNVKTEVPRSPTSPSFSPSPCFLAPCYLT
GDSVRDKCIEMLTAALRMDDDYKEFGVNCEKMASEIEDHILGLGSTDMKYRNRVRSRISN
LKDPKNPALRRNVLCGAIEPSLIARMTAEEMASDELKKLRNAMTQEAIREHQMAKTGGTV
TDLFQCGKCKKKNCTYNQVQTRSADEPMTTFVLCNECGNRWKVC

>Takifugu rubripes_TCEA3a(ENSTRUP_34730)
MTREEELVRIAKQLDKMVSRNNTDGAMDLLRELKSFNMTLRLLQETRIGVSVNSIRKHCT
DEEVIALAKVLIKDWKRLLETGHQTKNRLRFREKAVHQKRRESSDSKPGPPLKRNSTESK
PERRESQGSRSSQPGPPQRKPSTDGNERKAKPDTPKSPTTPTSPMSPGGALPPHLNTGDS
VRDKCIEMLAAALRTDIYTDDYKEFGTNCDSMAAEIEDHHIYQEIKATDMKYKNRVRSRI
SNLKDPKNPGLRKNVLAGTLALSRIASMSAEEMASDELKQLRNTLTQEAIREHQMAKTGG
TSTDLLQCGKCKKKNCTYNQVQTRSADEPMTTFVLCNECGNRWKFC

>Takifugu rubripes_TCEA3b(ENSTRUP_34735)
MTREEELVRIAKQLDKMVSRNNTDGAMDLLRELKSFNMTLRLLQETRIGVSVNSIRKHCT
DEEVIALAKVLIKDWKRLLASPSSSKFFSRRESQGSRSSQPGPPQRKPSTDGNERKAKPD
TPKSPTTPTSPMSPGGALPPHLNTGDSVRDKCIEMLAAALRTDIYTDDYKEFGTNCDSMA
AEIEDHIYQEIKATDMKYKNRVRSRISNLKDPKNPGLRKNVLAGTLALSRIASMSAEEMA
SDELKQLRNTLTQEAIREHQMAKTGGTSTDLLQCGKCKKKNCTYNQVQTRSADEPMTTFV
LCNECGNRWKFC

>Tetraodon nigroviridis_TCEA3(ENSTNIP_12540)
MTREEELVRIAKKLDKMVSRNTTDGAMDLLRELKSFNMTLKLLQETRIGVSVNSIRKHCA
DEEVIALAKVLIKDWKRLLGSGRRPYAEKPAGLKNGLDPARAAAASPGSSPPDTQNRRDE
KDSSSHPHPHAPPPSSPPPAKRPSLEARKERRDSSSGRGSAKPSDDAQKRDRRESVDSKA
GPPPKRNSTGSKPERRESSDSKKSSSSPAKKLTAERRPKADTPKTPTTPTSPTSPGGLLP
PHLSTGESVRDKCIEMLAAALRTDNDYKEFGTNCDSMAAEIEDHILLEIKATDMKYKNRV
RSRISNLKDPKNPGLRKNVLAGTIALSRIASMSAEEMASDELKQLRNTLTQEAIREHQMA
KTGGTTTDLLQCGKCKKKNCTYNQQVQTRSADEPMTTFVLCNECGNRWKFC

>Gasterosteus aculeatus_TCEA3a(ENSGACP_9658)
MTREEDLTRIAKKLDKMVSRNNTEGALDLLRELKSFNMTLKLLQETRIGMSVNGIRKNST
DEEVIALAKLLIKDWKRLLDLEKAAERNNGLDSSKTAVSPKGPPSETHSRKDSCDSKPGQ
PVRHKSTDSKPGRRESTDSKKSSSPPAKKLTVERESQGLKTSQPGPPQRKPSTDIMERKG
KADTPKTPTSPTSPMSPSGSSSGGPVPPHLATGDSVRDKCIYMLAAALRTDDHYKDFGTN
CDSMAAEIEDYIYQETKATDMKYKNRVRSRISNLKDPKNPGLRRNVLAGSIDLSRIASMS
AQEMASDELKQLRNVLTQEAIREHQMAKTGGTSTDLLQCGKCKKRNCTYNQVQTRSADEP
MTTFVLCNECGNRWKFC

>Gasterosteus aculeatus_TCEA3b(ENSGACP_9668)
MTREEDLTRIAKKLDKMVSRNNTEGALDLLRELKSFNMTLKLLQETRIGMSVNGIRKNST
DEEVIALAKLLIKDWKRLLVSHRRLHVKLKHDSSPEKKQPDKSGKEKHNEERRARRADDP
SDDEKHLEEPGRTESSDSKPPPQKKMPDPVKIKQMFPFVRKDSSDSKPPLLKKPSLGVKK
EKHRCNETKATDMKYKNRVRSRISNLKDPKNPGLRRNVLAGSIDLSRIASMSAQEMASDE
LKQLRNVLTQEAIREHQMAKTGGTSTDLLQCGKCKKRNCTYNQVQTRSADEPMTTFVLCN
ECGNRWKFS

>Homo sapiens_TCEA1(ENSP_428426)
MEDEVVRFAKKMDKMVQKKNAAGALDLLKELKNIPMTLELLQSTRIGMSVNAIRKQSTDE
EVTSLAKSLIKSWKKLLDGPSTEKDLDEKKKEPAITSQNSPEAREESTSSGNVSNRKDET

NARDTYVSSFPRAPSTSDSVRLKCREMLAAALRTGDDYIAIGADEEELGSQIEEAIYQEI
RNTDMKYKNRVRSRISNLKDAKNPNLRKNVLCGNIPPDLFARMTAEEMASDELKEMRKNL
TKEAIREHQMAKTGGTQTDLFTCGKCKKKNCTYTQVQTRSADEPMTTFVVCNECGNRWKF
C

>Macaca mulatta_TCEA1(ENSMMUP_50273)
MEDEVVRFAKKMDKMVQKKNAAGALDLLKELKNIPMTLELLQSTRIGMSVNAIRKQSTDE
EVTSLAKSLIKSWKKLLDGPSTEKDLDEKKKEPAITSQNSPEAREESTSSGNVSNRKDET
NARDTYVSSFPRAPSTSDSVRLKCREMLAAALRTGDDYIAIGADEEELGSQIEEAIYQEI
RNTDMKYKNRVRSRISNLKDAKNPNLRKNVLCGNIPPDLFARMTAEEMASDELKEMRKNL
TKEAIREHQMAKTGGTQTDLFTCGKCKKKNCTYTQVQTRSADEPMTTFVVCNECGNRWKF
C

>Mus musculus_TCEA1(ENSMUSP_80266)
MEDEVVRIAKKMDKMVQKKNAAGALDLLKELKNIPMTLELLQSTRIGMSVNALRKQSTDE
EVTSLAKSLIKSWKKLLDGPSTDKDPEEKKKEPAISSQNSPEAREESSSSSNVSSRKDET
NARDTYVSSFPRAPSTSDSVRLKCREMLAAALRTGDDYVAIGADEEELGSQIEEAIYQEI
RNTDMKYKNRVRSRISNLKDAKNPNLRKNVLCGNIPPDLFARMTAEEMASDELKEMRKNL
TKEAIREHQMAKTGGTQTDLFTCGKCKKKNCTYTQVQTRSADEPMTTFVVCNECGNRWKF
C

>Rattus norvegicus_TCEA1(ENSRNOP_10893)
MEDEVVRIAKKMDKMVQKKNAAGALDLLKELKNIPMTLELLQSTRIGMSVNAIRKQSTDE
EVTSLAKSLIKSWKKLLDGPSTDKDSEEKKKEPAISSQNSPEAREESSSSHVSSRKDET
NARDTYVSSFPRAPSTSDSVRLKCREMLAAALRTGDDYVAIGADEEELGSQIEEAIYQEI
RNTDMKYKNRVRSRISNLKDAKNPNLRKNVLCGNIPPDLFARMTAEEMASDELKEMRKNL
TKEAIREHQMAKTGGTQTDLFTCGKCKKKNCTYTQVQTRSADEPMTTFVVCNECGNRWKF
C

>Felis catus_TCEA1(ENSFCAP_13388)
GALDLLKELKNIPMTLELLQSTRIGMSVNAIRKQSTDEEVTSLAKSLIKSWKKLLDGPST
DKDPEEKKKDTAVTSQNSPEAREERQNKKQDSSIRLTKLSLDAKGSKNKSPLKPRRNSSG
NISSRKEEANARDTYVSSFPRAPSTSDSVRLKCREMLAAALRTGDDYVAIGADEEELGSQ
IEEDILSVYIHDLVPIYQEIRNTDMKYKNRVRSRISNLKDAKNPNLRKNVLCGNIPPDLF
ARMTAEEMASDELKEMRKNLTKEAIREHQMAKTGGTQTDLFTCGKCKKKNCTYTQVQTRS
ADEPMTTFVVCNECGNRWKSVFC

>Bos taurus_TCEA1(ENSBTAP_4499)
AGALDLLKELKNIPMTLELLQSTRIGMSVNAIRKQSTDEEVTSLAKSLIKSWKKLLDGPS
TDKDSEEKKKDTAVTSQNSPEAREESSSSGNMSSRKDETNARDTYVSSFPRAPSTSDSVR
LKCREMLAAALRTGDDYIAIGADEEELGSQIEEAIYQEIRNTDMKYKNRVRSRISNLKDA
KNPNLRKNVLCGNIPPDLFARMTAEEMASDELKEMRKNLTKEAIREHQMAKTGGTQTDLF
TCGKCKKKNCTYTQVQTRSADEPMTTFVVCNECGNRWKFC

>Monodelphis domestica_TCEA1(ENSMODP_17400)
MEDEIIRIAKKMDKMVQKKNAAGALDLLKELKNIPMTLELLQSTRIGMSVNAIRKQSTDE
EVTSLAKSLIKSWKKLLDGPSTDKDSDEKKKEPAISSQNSPEAKEESSSSNASNRKEET
NASDSFIPSFPRAPSTSDSVRMKCREMLAAALRTGDDYIAIGADEEELGSQIEEAIYQEL
RNTDMKYKNRVRSRIANLKDAKNPNLRKNVLCGNIPPDLFARLTAEEMASDELKEMRKNL
TKEAIREHQMAKTGGTQTDLFTCGKCKKKNCTYTQVQTRSADEPMTTFVVCNECGNRWKF
C

>Gallus gallus_TCEA1(ENSGALP_44127)
MTTEDEIIRIAKKMDKMVQKKNAAGALDLLKELKNIPMTLELLQTTRIGMSVNAIRKQST
DEEVTSLAKSLIKSWKKLLDGPSTDKDSEEKKKEPASSSQNSPEAREESSSSSNSSSRKE
EGSAPSNSFIPSFPRAPSTSDSVRVKCREMLSAALRTGDDYIAIGADEEELGSQIEEAIF
QELKNTDMKYKNRVRSRIANLKDAKNPNLRKNVLCGNIPPDKFAKMTAEEMASDELKEMR
KNLTKEAIREHQMAKTGGTQTDLFTCGKCKKKNCTYTQVQTRSADEPMTTFVVCNECGNR
WKFC

>Anolis carolinensis_TCEA1(ENSACAP_17696)

MSAEDEIIRIAKKMDKMVQKKNAAGALDLLKELKNMPMTLELLQSTRIGMSVNAIRKQST
DEEVTSLAKSLIKSWKKLLDGPSNDKDSEDKKKEAASSSQNSPEAREESSSSSNSSNRKE
ESNTGSDAFISSFPRAPITSDSVRMKCREMLAAALKTGDDYIAIGADEEELGSQIEEAPI
FQELKNTDMKYKNRVRSRIANLKDTKNPNLRKNVLCGNIAPDRFAKMTAEEMASDELKEM
RKNLTKEAIREHQMAKTGGTQTDLFSCGKCKKKNCTYTQVQTRSADEPMTTFVVCNECGN
RWKFC

>Xenopus tropicalis_TCEA1(ENSXETP_58861)
MSSAAGDEIIHIAKKMERMVQKKNTAGALDLLKELKNLPMTLELLQSTRIGMSVNAIRKQ
SGEEEVTSLAKSLIKSWKKLLDGPSADMEEKRKDQPAPAQNSPEAREESSSSTNISARKE
ECPAPSDGFITSFPRAPSTSDSVRIKCRELLATALKTGDDHIAIGADVDELGAQIEEAVF
QEFKNTEAKYKNRIRSRIANLKDAKNPNLRRNVLCGNIAPDLFARMSAEEMASDELKEMR
KNLTKEAIREHQMARTGGTETDLFTCGKCKKKNCTYTQVQTRSADEPMTTFVFCNECGNR
WKFC

>Danio rerio_TCEA1a(ENSDARP_92636)
MGKKEEEEIIRIAKKMDKMAQKKNGVGALDLLKELKNIPMTLELLQSTRIGMSVNAIRKQ
STDDEVTSLAKSLIKSWKKLLDEPAADKNSEEKKKERTTPVVSPSETSPEPREESSSSNS
SSKSESVDVTPNTLIATFPRAPGTSDSVRIKCREMLSNALQTGDDYITIGSDCDELGAQI
EECIFLEFKNTDMKYKNRVRSRISNLKDAKNPNLRRNVLCGNVSPDRIAKMTAEEMASDE
LKEMRKNLTKEAIRDHQVATSGGTQTDLFTCGKCKKKKCTYTQVQTRSADEPMTTFVFCN
ECGNRWKFC

>Danio rerio_TCEA1b(ENSDARP_134502)
MGKKEEEEIIRIAKKMDKMAQKKNGVGALDLLKELKNIPMTLELLQSTRIGMSVNAIRKQ
STDDEVTSLAKSLIKSWKKLLDEPAADKNSEEKKKERTTPVVSPSETSPEPREESSSSNS
SSKSESVDVTPNTLIATFPRAPGTSDSVRIKCREMLSNALQTGDYITIGSDCDELGAQIE
EYILFIFMPNFKNTDMKYKNRVRSRISNLKDAKNPNLRRNVLCGNVSPDRIAKMTAEEMA
SDELKEMRKNLTKEAIRDHQVATSGGTQTDLFTCGKCKKKKCTYTQVQTRSADEPMTTFV
FCNECGNRWKFC

>Takifugu rubripes_TCEA1a(ENSTRUP_29649)
MGKKEEEDIIRIAKKIDKMAQKKNGAGALDLLKELRSIPMTLELLQSTRIGMSVNAIRKQ
STDEEVTSLAKSLIKSWKKLLDEPGGGEKTSEEKRKEQSTPVISSSQESPEAREESSCSS
SNNNSRSEPSDDTPSSFVNTFPRAASTSDPIRVKCREMLANALQTGDDYIAIGADCDELG
AQIEDYILSMFKNTDMKYKNRVRSRISNLKDVKNPNLRRTVLCGSITPERMAKMTAEEMA
SDELKEIRKNLTKEAVRDHQMATTGGTQTDLFTCGKCKGKNCTYTQVQTRSADEPMTTFV
FCSGCGNRWKVC

>Takifugu rubripes_TCEA1b(ENSTRUP_29648)
VKMGKKEEEDIIRIAKKIDKMAQKKNGAGALDLLKELRSIPMTLELLQSTRIGMSVNAIR
KQSTDEEVTSLAKSLIKSWKKLLVIKKERSECPRSDFSASPSFRSCSSSNNNSRSEPSDD
TPSSFVNTFPRAASTSDPIRVKCREMLANALQTGDDYIAIGADCDELGAQIEDYILSMFK
NTDMKYKNRVRSRISNLKDVKNPNLRRTVLCGSITPERMAKMTAEEMASDELKEIRKNLT
KEAVRDHQMATTGGTQTDLFTCGKCKGKNCTYTQVQTRSADEPMTTFVFCSGCGNRWKVC

>Tetraodon nigroviridis_TCEA1(ENSTNIP_21813)
MGKKEEEEIIRIAKKIDKMAQKKNGSGALDLLKELRSVPMTLELLQSTRIGMSVNAIRKQ
STDEEVTSLAKSLIKSWKKLLDEPGSGEKTSEEKRKEQSTPIISSSQESPDAREESCSSS
NTNSRSEPLDDTPGSFVNAFPRAASTSDPIRVKCREMLANALQTGDDYIAIGADCDELGA
QIEDFIFQEFKNTDMKYKNRVRSRISNLKDVKNPNLRRTVLCGSITPERMAKMTAEEMAS
DELKEIRKNLTKEAVRDHQMATTGGTQTDLFTCGKCKGKNCTYTQVQTRSADEPMTTFVF
CNGCGNRWKIC

>Gasterosteus aculeatus_TCEA1(ENSGACP_22642)
MGKKEEDEIIRIAKKMDKMAQKKNGAGALDLLKELRSIPMTLELLQSTRIGMSVNAIRKQ
STDDEVTSLAKALIKSWKKLLDEPGSGEKPSDEKRKEPTTPVSPSQGSPEAKEESSSSSN
SSSKSEPAEVSTNSLINTFPRAPRTSDSIRIKCRELLAAALQAGDDHIAIGADCDELGAQ
IEEVIFQEFKNTDPKYKSRVRSRISNLKDMKNPNLRRTVLCGSVTPERMAKMTAEEMASD
ELKEMRKNLTKEAVRDHQMATTGGTQTDLFTCGKCKGKCCTYTQVQTRSADEPMTTFVFC
NDCGNRWKFC

>Oryzias latipes_TCEA1(ENSORLP_22301)
MSKKEEEEIIRIAKKMDKMAQKKNGAGALDLLKELRNIPMSLELLQSTRIGMSVNAIRKQ
STDEEVTSLAKSLIKSWKKLLGSPCPDIKKKAQKHQEQKPVCSLSIHSLKNRTTSSSSNF
SSRSDTTEVAPNSLIHNFPRAPSTSDSIRLKCREMLANALQTGEDYIAIGADCEELGAQI
EEYILHEFKNTDMKYKNRVRSRISNLKDMKNPNLRRTVLCGSVSPERMAKMTAEEMASDE
LKEMRKNLTKEAVRDHQMATTGGTQTDLFTCGKCKGKCCTYTQVQTRSADEPMTTFVFCN
QCGNRWKVC

>Homo sapiens_TCEA2(ENSP_343515)
MMGKEEEIARIARRLDKMVTKKSAEGAMDLLRELKAMPITLHLLQSTRVGMSVNALRKQS
SDEEVIALAKSLIKSWKKLLDASDAKARERGRGMPLPTSSRDASEAPDPSRKRPELPRAP
STPRITTFPPVPVTCDAVRNKCREMLTAALQTDHDHVAIGADCERLSAQIEECIFRDVGN
TDMKYKNRVRSRISNLKDAKNPDLRRNVLCGAITPQQIAVMTSEEMASDELKEIRKAMTK
EAIREHQMARTGGTQTDLFTCGKCRKKNCTYTQVQTRSSDEPMTTFVVCNECGNRWKFC

>Gorilla gorilla_TCEA2(ENSGGOP_3790)
MMGKEEEIARIARRLDKMVTKKSAEGAMDLLRELKAMPITLHLLQSTRVGMSVNALRKQS
SDEEVIALAKSLIKSWKKLLDASDAKARERGRGTPLPTSSRDASEAPDPSRKRPELPRAP
STPRITTFPPVPVTCDAVRNKCREMLTAALQTDHDHVAIGADCERLSAQIEECIFRDVGN
TDMKYKNRVRSRISNLKDAKNPDLRRNVLCGAITPQQIAVMTSEEMASDELKEIRKAMTK
EAIREHQMARTGGTQTDLFTCGKCRKKNCTYTQVQTRSSDEPMTTFVVCNECGNRWKFC

>Mus musculus_TCEA2(ENSMUSP_99331)
MGKEEEIARIARRLDKMVTRKNAEGAMDLLRELKNMPITLHLLQSTRVGMSVNALRKQSS
DEELIALAKSLIKSWKKLLDVSDGKSRNQGRGTPLPTSSSKDASRTTDLSCKKPDPPRTP
STPRITTFPQVPITCDAVRNKCREMLTLALQTDHDHVAVGVNCEHLSSQIEECIFLDVGN
TDMKYKNRVRSRISNLKDAKNPGLRRNVLCGAITPQQIAVMTSEEMASDELKEIRKAMTK
EAIREHQMARTGGTQTDLFTCNKCRKKNCTYTQVQTRSSDEPMTTYVVCNECGNRWKFC

>Canis familiaris_TCEA2(ENSCAFP_22394)
EGAMDLLRELKAMPVTLHLLQSTRVGMSVNALRKQSSDEEVIALAKSLIKSWKKLLDASD
AKAREQRRAGPPPTSSSKEAPEAKDPSRKRPELPRVPSAPRITTFPPVPVTCDAVRNKCR
EMLTAALQTDHDHMAVGADCEGLSAQIEECIFRDVGNTDMKYKNRVRSRISNLKDAKNPD
LRRNVLCGVITPQQIAVMTSEEMASDELKEIRKAMTKEAIREHQMARTGGTQTDLFTCGR
CRRKNCTYTQVQTRSSDEPMTTFVVCNECGNRWK

>Monodelphis domestica_TCEA2(ENSMODP_21286)
GGAGAGTTRPKMQRGGAAEPEARPERAAHGTMMGKEEEIVRIAKRLDKMVTKKSTDGAMD
LLKELKSMPITLDLLQSTRIGMSVNALRKQSTDDEVISLAKSLIKSWKKLLDASEDKNDE
KKRNSSLATSSSKDASDTKDQSSNKKQDPPRTISTPKITTFPPIPITCDAVRNKCREMLT
AALQTDNDHVAIGADCEHLSAQIEEYILKEGLPENTDMKYKNRVRSRISNLKDSKNPDLR
KNVLCGAITPEQIAVMTSEEMASNELKEIRKAMTKEAIREHQMAKTGGTQTDLFTCGKCK
KKNCTYTQVQTRSSDEPMTTFVVCNECGNRWKVG

>Gallus gallus_TCEA2(ENSGALP_49511)
MGGEEEIVRIAKRLDKMVAKKSADGAMDLLKELKSMPMTLDLLQSTRIGMSVNALRKQSS
DEEVIALAKSLIKSWKKLLDASEEKSEEKKKNLSLPTSSSKETGNSRDQSSNKRQEPPKT
PTTPKITTFPPAPVTCDAVRNKCREMLTTALQADDDYIAIGADCEHIAAQIEECIYQDIK
NTDMKYKNRVRSRISNLKDSKNPELKKNVLCGAITPEQIAVMTSEEMASNELKEIRKAMT
KEAIREHQMAKTGGTQTDLFTCGKCKKKNCTYTQVQTRSSDEPMTTFVVCNECGNRWKVA
HQGYYWH

>Anolis carolinensis_TCEA2(ENSACAP_6264)
AMGGEQEIVRIAKRLDKMVAKKSAEGAMDLLKELKSMPITLHLLQSTRIGMSVNALRKQS
TDEDVIALAKSLIKAWKKLLDASEDKGNEKKRNSLPTSSSRESGDSKDQSSIKRQDLPKT
PTTPKITTFPPVPITCDTVRSKCREMLTSALQTDNDYVAIGVDCEEMAAQIEAFELIYQD
VKNTDLKYKNRVRSRISNLKDSKNPDLRKNVLCGVITPEQIAVMTSEEMASNELKEIRKA
MTKEAIREHQMAKTGGTQTDLFTCGKCRKKNCTYTQVQTRSSDEPMTTFVVCNECGNRWK
FC

>Danio rerio_TCEA2(ENSDARP_2994)
MVKDQEVERIAKKLDKMVHKKNTDGAIDLLQELKNMKMSLETLQSTRIGMSVNAVRKQSS
DEEVQTLAKSLIKAWKKLLDGSEGKPEEKKGESPLQSSSSKDCPGSSDTSNKPETPKTPT
TPVTPKFTMFPPAPVTTDSVRTKCRELLVAALQTDGDHQTIGADCEHMAAQIEDYIYQEF
KSTDMKYKTRLRSRISNLKDQKNPDLRRNVLCGNISPERIASMTAEEMASPELKEIRKAL
TKESIREHQLSKVGGTETDMFVCGKCKGKNCTYTQVQTRSADEPMTTFVLCNECGNRWKF
C

>Oryzias latipes_TCEA2(ENSORLP_20995)
TAMAKNQEVERIAKKLDKMVQKENTEGALDLLKELQNIKMSLETLQSTRVGMSVNAVRKQ
SSDEEVQTLAKSLIKSWKRLLDGSEGKSEKKEKKKEGSPLRSSSTSSDSGSSGKRHCSAK
SGDTPTTPNTPSTPTLPPLITSFPPAPVTSDSVRNKCRELLVAALQTDDDYKTIGVDCDH
LAAQIEHHIFQEFKSTDMKYKARLRSRISNLKDQKNPDLRRNVLCGNISAQRIACMTAEE
MASAELKQIREALTKESIREHQLSKVGGTETDMFICSKCHGKNCTYTQVQTRSADEPMTT
FVLCNGCGNRWKFC

>Homo sapiens_TCEANC(ENSP_440038)
MISAHRKLCLPGSSNSPASAFRVAGTTAVKMSDKNQIAARASLIEQLMSKRNFEDLGNHL
TELETIYVTKEHLQETDVVRAVYRVLKNCPSVALKKKAKCLLSKWKAVYKQTHSKARNSP
KLFPVRGNKEENSGPSHDPSQNETLGICSSNSLSSQDVAKLSEMIVPENRAIQLKPKEEH
FGDGDPESTGKRSSELLDPTTPMRTKCIELLYAALTSSSTDQPKADLWQNFAREIEEHVF
TLYSKNIKKYKTCIRSKVANLKNPRNSHLQQNLLSGTTSPREFAEMTVMEMANKELKQLR
ASYTESCIQEHYLPQVIDGTQTNKIKCRRCEKYNCKVTVIDRGTLFLPSWVRNSNPDEQM
MTYVICNECGEQWYHSKWVCW

>Gorilla gorilla_TCEANC(ENSGGOP_4804)
MISAHRKLCLPGSSNSPASAFRVAGTTAVKMSDKNQIAARASLIEQLMSKRNFEDLGNHL
TELETIYVTKEHLQETDVVRAVYRVLKNCPSVALKKKAKCLLSKWKAVYKQTHSKERNSP
KLFPVRGNKEENSGPSHDPSQNETLGICSSNSLSSQDVAKLSEMIVPENRAIQLKPKEEH
FGDGDPESTGKRSSELLDPTTPMRTKCIELLYAALTSSSTDQPKADLWQNFAREIEEHVF
TLYSKNIKKYKTCIRSKVANLKNPKNCHLQQNLLSGTTSPREFAEMTVMEMANKELKQLR
ASYTESCIQEHYLPQVIDGTQTNKIKCRRCEKYNCKVTVIDRGTLFLPSWVRNSNPDEQM
MTYVICNECGEQWYHSKWVCL

>Mus musculus_TCEANC(ENSMUSP_136691)
MSDKNQIIARASLIEQLVSKRYFEDIGKQLTELEMIYVSKEHLQETDVVRAVYRVLKNCP
SVTLKKKAKCLLAKWRGFYKSTHCKPRQSPKVLHTNANKEESAAVSQDVSQDETSGSSHS
EIMGLCSSLSRLLPQDAAKPAAAIGSESSTAQMEINEGYLKGDDSECTRKSSGVFQGTLV
SVRSKCVELLYTALASSCTDHTEVHIWQNLAREIEEHIFTLHSNNIKKYKTSIRSKVANL
KNPRNFHLQQNFLSGTMSAREFAEMSVLDMASQELKQLRASYTESSIQEHCLPQSVDGTW
TNKIKCRRCDKYNCKVTVIARGTLFLPSWVQNSNPDEQMTYVICNECGEQWYHNNWVCL

>Canis familiaris_TCEANC(ENSCAFP_35135)
AVKMSDKNQIAASASLIEQLLSKRNFEDLGNHLTDLETLCVTKEDLQETIVVRAVYRVLK
HCPSVALKKKAKHLLSEWKALYKKDLHFKPRDKSFPTGRNEENSRLSHDPSQDELSGGSS
SNSSSHSVAEAFEMIVPENSTSQTEPKEEHFRSGDPKSTAQRSSELLDPMVPVRAKCTEL
LYEALTSPFTEQPKADLWRNFAREIEEHIFTLHSKNLKKYKTCVRSKVANLRNPKNSHLQ
QNLLSGTMSPREFAKMTVMEMANKELKQLRASYTESCIQEHHLPQAIEGTHTRKIKCRRC
EKFNCKVTVIARGTLFLPSWVRNSNPDEEMMTYVICNECGEQWYHSKWVCL

>Gallus gallus_TCEANC(ENSGALP_26714)
MSDQKNIIYRIHCIEKLLSENNFQDIEDHLKELEDVSMTVEYLQGTEVAKVVYRILKSCP
SAELKKKAKQLLSRWKALYKNNRLQSVQVKKSVSVCVSEATEHLEIPGEQLLSEGPCQQE
VLDAVPSKILVPPRTVKKVASNNADGSRDQLSPFEEGHTDNEGSKPLVNEAGSQQDHMRA
LRCKCTDLLYKALTGSAKDKEETDKWLELSKEIEEHVFALHCKNDKKYKNCIRSKISNLK
NPKSCNLKHNLFSGTLSPKAFAEMTVMEMASDELKQLRALYTESSIQEHQLPQVINGTQT
NKIKCRRCEKSDCTVTMIARGTLFLPGWVRSTNPDEQMLTFVICNECGEQWYHSRWICL

>Anolis carolinensis_TCEANC(ENSACAP_1595)
MSNTKVLTSRADCIEKLLSERKYQDIVIHLTYLETADITIDDLQETDVAKAVFRVSQNCP
SVVLKKKAKHLLSKWKAIYKKHCSPSVQLHLGLSENANEDSGHIQVVSEDVNSIGKLNQQ

DKLCSASPSLASESPSSNPSSCVQKDNTTQPDLGNGSSISETGQQHGPIMAVRGKCTQLL
FEALTDSSTSNEEIAKHHQTAEEIEQHIFALHAGNDRKYKNSIRSKVSNLKNPKNYHLKH
SLHIGVLSPQTFAGMSAVEMAHDELKQLRASYTKSAVQEHQLPQRISGTLTNKIKYGEPT
VLFLILDSS

>Xenopus tropicalis_TCEANC(ENSXETP_56763)
MLHKDINKELVSRALEVENLLAERKYEDLAFHLTYFEKAEVTSGILQGTDIVRVVYRVLK
TCPRGALKLKAKVLLSKWKKLYRKCCHETSCICEHCSKICENKEGPEKLAVDIQDQVHNS
EQKILINKSTSSPSSYKFNLNKSDSSAQDCYGNHEESVTDHEVPKKVNAEDLALRTKCTE
LLYQALREDSECQEKLQNLAKAIEENIYKIHAGNTKKYRNCIRSKISNLKNPKNSHLKMQ
ILSRALSPKVFAEMGVMEMACDELRNLRANYTETCVQEHQLPQGVDGVHTNKIRCRRCDK
FNCTVTMISRGTLFLPGWVRTGNPDEEMMTFVICNECGEQWYHNRWVCL

>Tetraodon nigroviridis_TCEANC(ENSTNIP_214)
AAMDVVHSVLEIERASRERNYRNILALLGDLDKAAITAEQLEMTDVVKVLYRLLKTCPEV
GVRRAAKALLSRWKKEHSQDGDKQENNPELPCRSADVGAEKVHEDASTSGDKCGLRAASG
ASGGFSSVRSKCVQLLLSSLRPEPPEQAQAAELAAAIERHIHERHRSNPLKYKACVRSKV
ANLRNPQNQHLREGLLSASLSPLTFAGMSAEEMAGAALRRLREEYSSRGLSERQLPQAAE
GTPTRKIRCKRCGGRECRVSQVARGALFLPAWVRRGIPGEDAMTFVTCSGCGEQWYHS

>Branchiostoma floridae_(XP_002593577)
MENALDILKSLKELPMTLDILQKTRIGMSVNNLRKSSNSEEVNSLAKSLIKSWKKLLNQNGAPKRSDSTS
SEPSEKSPPEGTKLQRQDSSSTAGSSFSSSQDSFPAVTKTHDSVREKCREMLANALKTDEFKNTEMKYKT
RVRSRIANLKDAKNPGLREKVLHGDVSAEKMAKMTAEEMASPEMKELRQSLTKEAIRDAQMATTGGTQTD
LLKCGKCKKRNVTYNQVQTRSADEPMTTFCYCNECGNRWKENALDILKSLKELPMTLDILQKTRIGMSVN
NLRKSSNSEEVNSLAKSLIKSWKKLLNQNGAPKRSDSTSSEPSEKSPPEGTKLQRQDSSSTAGSSFSSSQ
DSFPAVTKTHDSVREKCREMLANALKTDEFKNTEMKYKTRVRSRIANLKDAKNPGLREKVLHGDVSAEKM
AKMTAEEMASPEMKELRQSLTKEAIRDAQMATTGGTQTDLLKCGKCKKRNVTYNQVQTRSADEPMTTFCY
CNECGNRWKATKTLRCRCIRIITPPCMIYHNAVIISSRTVVATAPEGSIRPKSSLSSDATASADRLSGAE
IRRDAGGIACLAQVPRAQTAGVATATGG

>Saccoglossus kowalevskii_(XP_006818876)
MPGTEEEVIRIGKQLEKMVSKNASDLGNAFDLLKSLKELPMTLEVLQKTRIGMSVNSLRKQSSNEEVISL
AKSLIKSWKKLLPAPSSSKSSNKDNQDSSRDSPTDLSQDGNSINSNNDNSSSSQSKSSSKFQVPKTNDAV
REKCREMLTNALKTPLGDIESVGTEELGEPEEIAATIEDCIFTEFTNTEAKYKTRVRSRVANLKDVRNPI
LRQQVLCGSIPPEKIANMTAEEMASDRLKELRRELTKEAIREAQMSTTGGTKTSLLKCGKCKKRNCTYNQ
VQTRSADEPMTTFVFCNECGNRWKFC

>Hydra magnipapillata_(XP_002166168)
MMITSIKWIFFLYTLIILIGPCDSKKKSVSVALRRNARNVKGGNQNTENLKGRPGQGYYIALEIGTPPQR
INVLVDTGSSNFAVAATPHPYLPVYFHPDRSSTFTDLNRAVAVPYTQGNWEGKLGSDLIRFIDAPNATVR
VNIAAIHSSENFFINGSNWEGILGLGYARLSKPDSSVTPLFDQFVISDVVEDTFSMQLCSTSELDPSSEP
TVAGTMVFGEMDDDLYKGDMLYTPIVKKWYYEVVITDMSVNGISLGVHCKKYNYDKTIVDSGTTNLRVPE
EIFNLILTQLKKYDKLGVPDEFWIGRQMMCWNYDKTPWEEFPHMSISLLSSLSSSKEFVLKIPPQLYLRE
TIERGPLPGQHCYKFAITKAEKGTVIGAVVMEGFYVVFDRENIRVGFAATTCGAEGRLNPRSEVSGPFIR
ADPTDCEYIESELADTALLTVAYVMAGVCGLCLLPVFVLLGQASCKKSVEKAGVPRDY

UBX Domain-Containing Protein-UBXN

>Homo sapiens_UBXN2A(ENSP_312107)
MKDVDNLKSIKEEWVCETGSDNQPLGNNQQSNCEYFVDSLFEEAQKVSSKCVSPAEQKKQ
VDVNIKLWKNGFTVNDDFRSYSDGASQQFLNSIKKGELPSELQGIFDKEEVDVKVEDKKN
EICLSTKPVFQPFSGQGHRLGSATPKIVSKAKNIEVENKNNLSAVPLNNLEPITNIQIWL
ANGKRIVQKFNITHRVSHIKDFIEKYQGSQRSPPFSLATALPVLRLLDETLTLEEADLQN
AVIIQRLQKTASFRELSEH

>Pan troglodytes_UBXN2A(ENSPTRP_20106)
MKDVDNLKSIKEEWVCETGSDNQPLGNNQQSNCEYFVDSLFEEAQKVSSKCVSPAEQKKQ
VDVNIKLWKNGFTVNNDFRSYSDGASQQFLNSIKKGELPSELQGIFDKEEVDVKVEDKKN
EICLSTKPVFQPFSGQGHRLGSATPKIVSKAKNIEIENKNNLSAVPLNNLEPITNIQIWL
ANGKRIVQKFNISHRVSHIKDFIEKYQGSQRSPPFSLATALPVLRLLDETLTLEEADLQN

AVIIQRLQKTASFRELSEH

>Mus musculus_UBXN2A(ENSMUSP_118834)
MKEVDNLDSIKEEWACETGPPDSQPLNDNQQKDCEYFVDSLFEEAGKAGAKCLSPTEQKK
QVDVNIKLWKNGFTVNDDFRSYSDGASQQFLNSIKKGELPSELWGIFDKEEVDVKVEDKK
NEVCMSTKPVFQPFSGQGHRLGSATPRIVSKAKSVEVDNKSTLSAVSLNNLEPITRIQIW
LANGERTVQRFNVSHRVSHIKDFIEKYQGSQRSPPFALATALPFLRFLDETLTLEEADLK
NAVIIQRLQKTAEPFRKL

>Rattus norvegicus_UBXN2A(ENSRNOP_6668)
MKEVDNLDSIKEEWVCETGPPDNQPLNDNPQKDCEYFVDSLFEEAEKAGAKCLSPTEQKK
QVDVNIKLWKNGFTVNDDFRSYSDGASQQFLNSIKKGELPSELQGVFDKDEVDVKVEDKK
NEVCMSTKPVFQPFSGQGHRLGSATPRIVSKAKSIEVDNKSTLSAVSLNNLEPITRIQIW
LANGERTVQRFNISHRVSHIKDFIEKYQGTQRSPPFALATALPFLRFLDETLTLEEADLQ
NAVIIQRLQKTAEPFRKL

>Bos taurus_UBXN2A(ENSBTAP_2634)
MKEVDNLESIKEEWVCETGSDHQPLSDSQQKNCEYFVDSLFEEAQKVGAKCLSPTEQKKQ
VDVSIKLWKNGFTVNDDFRSYSDGASQQFLNSIKKGELPLELQGVFDKEEVDVKVEDKKN
EVCMSTKPVFQPFSGQGHRLGSATPKIVSKAKNIEVENKNKLSAVPLNNLEPITNIQIWL
ANGKRIVQKFNISHRISHIKDFIEKYQGSQRSPPFSLATALPFLKLLDETLTLEEADLQN
AVIIQRLKKTAEPFKELS

>Monodelphis domestica_UBXN2A(ENSMODP_2593)
MAFGPVDCVPNDRTRKENAGDCFLWSCSFSFHFAAGAGRMKEVEKLESLREDWLCESGAE
GQSHRGSQAGSCELFFDSLLEEAQRGGSEHLPSAAAVTQADVSIKLWQNGFTVNGEFRSY
GDGASQQFLNAIRKGELPSELRGRFSQEEVAVRVEDKKEQVFVPRKPAFAPFSGRGHRLG
SATPRIVSKASGEAGPPKPLAAPAVPLNPWEPVTSIQIWLADGRRLVQRFNVSHRVSHVR
DFIRSCEGSPRSAPFSLVTALPGLRPLDDALTLEEAGLRNAVVIQRRPAPRAPAV

>Xenopus tropicalis_UBXN2A(ENSXETP_39482)
MREADKLDNMKSQDRGFRSGGRGSERKRCDSFVNSLFEEAENAGALIASPEDEDSNADVI
IKMWKNGFTINDGYLRDYSGAENRQFMDSVRKGELPEELQKTFDKEEIAVNVEDRKNEEY
LLRKPNIDAFSGLGHRLGSAAPKVITKDMETCNEQSLPSVDLNELEPLTNIKVWLADGKR
IVQKFNTSHRISDVRDFLERIPCKPGNAPFTLATSFPLHDLLDESLTIQEAELQNSVLVQ
KLQRTTEPFRNS

>Danio rerio_UBXN2A(ENSDARP_124898)
MMKNIDSRERHEDDTWCEGAQNEEEEEEEDEEASPIRSSFSVEDLLDEVEKISSVASSGK
KVEIVVRLWKNGFTLNDEDLRSYTQEENQEFLEAIKKGELPLELEGRAEDEELEVNVEDM
KDEVYVPKKKIFHPFTGRGYRLGSVAPRVVARSRSIHEDCSGPPVPAVELNEDLPVTSLQ
IWLADGRRLVQRFNLCHRISDVQRFVEQAQITDTPFILTTSLPFRELTDEAQSLEEADLA
NAVIVQRPVNTHAPFGHS

>Takifugu rubripes_UBXN2A(ENSTRUP_13296)
FSVEDLLDEVEKICYEASGAKQQVEMVVRLWKDGFTVNDEEFRSYSVPENQDFLDAIKRG
ELPGEWESRAEKEELEISVEDLTEENYLPKKKVFHPFSGRGYRLGSVAPRVVARSPSVHE
DGESPPIPMVTLDHALPVTSLQIWLADGRRLVQRFNLSHRIIDVQDFVARSQRSCPPFIL
TTSLPFRELSDKDLSLEEADLANAVIVQRPLNTEAPF

>Tetraodon nigroviridis_UBXN2A(ENSTNIP_6186)
MSRSFSVEDLLDEVEKICYDASGAKVEMVVRLWKDGFTVNDEEFLKIYRSRGGKRESWRS
IGRGYRLGSVAPRVVARSPSVHEDGESPPIPMVTLDHALPVTSLQIWLADGRRLVQRFNL
SHRIVDVQDFVARSQRSCPPFILTTSLPCRELSDKDLSLEEADLANAVIVQRPLNTEAPF
GHS

>Gasterosteus aculeatus_UBXN2A(ENSGACP_16377)
MKEFDSVGGERDDNCEESEECEEQAPIRRSFSVEDLLHEVEKICYDASGTSKVEMVVRLW
KDGFTVNDQEFRSYSVPENQDFLDSIKRGELPKEWESRAEEEELEISVEDLTEENYVPRR
KAFHPFSGQGYRLGSVAPRVVARSPSVHEDGESPPIPMVTLDHALPVTSLQIWLADGRRL
VQRFNLSHRIADVQDFVARCQRSCPPFVLTTSVPFRELNDKELSLGEADLANAVIVQRPL

DTPAPFGHS

>Homo sapiens_UBXN2B(ENSP_382507)
MAEGGGPEPGEQERRSSGPRPPSARDLQLALAELYEDEVKCKSSKSNRPKATVFKSPRTP
PQRFYSSEHEYSGLNIVRPSTGKIVNELFKEAREHGAVPLNEATRASGDDKSKSFTGGGY
RLGSSFCKRSEYIYGENQLQDVQILLKLWSNGFSLDDGELRPYNEPTNAQFLESVKRGEI
PLELQRLVHGGQVNLDMEDHQDQEYIKPRLRFKAFSGEGQKLGSLTPEIVSTPSSPEEED
KSILNAVVLIDDSVPTTKIQIRLADGSRLIQRFNSTHRILDVRNFIVQSRPEFAALDFIL
VTSFPNKELTDESLTLLEADILNTVLLQQLK

>Gorilla gorilla_UBXN2B(ENSGGOP_15686)
MAEGGRPEPGEQERRSSGPRPPSARDLQLALAELYEDEVKCKSSKSNRPKATVFKSPRTP
PQRFYSSEHEYSGLNIVRPSTGKIVNELFKEAREHGAVPLNEATRASGDDKSKSFTGGGY
RLGSSFCKRSEYIYGENQLQDVQILLKLWSNGFSLDDGELRPYNEPTNAQFLESVKRGEI
PLELQRLVHGGQVNLDMEDHQDQEYIKPRLRFKAFSGEGQKLGSLTPEIVSTPSSPEEED
KSILNAVVLIDDSVPTTKIQIRLADGSRLIQRFNSTHRILDVRNFIVQSRPEFAALDFIL
VTSFPNKELTDESLTLLEADILNTVLLQQLK

>Mus musculus_UBXN2B(ENSMUSP_29907)
MAEGGRAEPEEQERGSSRPRPPSARDLQLALAELYEDEMKCKSSKPDRSTPATCRSPRTP
PHRLYSGDHKYDGLHIVQPPTGKIVNELFKEAREHGAVPLNEATRSSREDKTKSFTGGGY
RLGNSFYKRSEYIYGENQLQDVQVLLKLWRNGFSLDDGELRPYSDPTNAQFLESVKRGET
PLELQRLVHGAQVNLDMEDHQDQEYIKPRLRFKAFSGEGQKLGSLTPEIVSTPSSPEEED
KSILNAAVLIDDSMPTTKIQIRLADGSRLVQRFNSTHRILDVRDFIVRSRPEFATTDFIL
VTSFPSKELTDETVTLQEADILNTVILQQLK

>Rattus norvegicus_UBXN2B(ENSRNOP_12551)
MAEGGGAEPEEQERRSSRPRPPSARDLQLALAELYEDEMKCKSSKPDRSTATAFKSPRTP
PLRLYSGDQEYGGLHIAQPPTGKIVNELFKEAREHGAVPLNEATRSSSDDKAKSFTGGGY
RLGSSFYKRSEYIYGENQLQDVQILLRLWSNGFSLDDGELRPYSDPTNAQFLESVKRGEI
PLELQRLVHGSQVSLDMEDHQDQEYIKPRLRFKAFSGEGQKLGSLTPEIVSTPSSPEEED
KSILNAAVLIDDSVPTTKIQIRLADGSRLIQRFNSTHRILDVRDFIVQSRPEFATTDFIL
VTSFPSKELTDESVTLQDADILNTVILQQLK

>Canis familiaris_UBXN2B(ENSCAFP_10519)
MAEGGGADPGEQERSSGPRPPSARDLQLALAELYEDEVKCKSSKSDRPKATVFKSPRTPP
QRFYSSEHEYSGLHIVRPSTGKIVNELFKEAREHGAVPLNEATRASGDNKSKSFTGGGYR
LGNSFCKRSEYIYGENQLQDVQILLKLWSNGFSLDDGELRPYNDPPNAQFLESVKRGEIP
LELQRLVHGGHVNLDMEDHQDQEYIKPRLRFKAFSGEGQKLGSLTPEIVSTPSSPEEEDK
SIFNAVVLIDDSVPTTKIQIRLADGSRLIQRFNSTHRILDVRNFIIQSRPEFATLDFILV
TSFPNKELTDESLTLQEADILNTVILQQLK

>Bos taurus_UBXN2B(ENSBTAP_12043)
MAEGGGGEAGEEEPRAAGPRPPSARDLQLALAELYEDEVKCKASKSDRPKATAFKSPQTA
PQRFYSSEHECSGLHIVQPSTGKIVNELFREARQHGAVPLNEATRASGDDKSKSFTGGGY
RLGNSFCKQSEYIYGENQLQDVQILLKLWSNGFSLDDGELRPYSDPTNAQFLESVKRGEI
PLELQRLVHGGHLNLDMEDHQDQEYVKPRLRFKAFSGEGQKLGSLTPEIVSTPSSPEEEE
KSLFNAVVLIDDSMPTTKIQIRLADGSRLIQRFNSTHRILDVRDFIVQSRPEFATLDFIL
VTSFPNKVLTDESLTLQEADILNTVILQQLK

>Myotis lucifugus_UBXN2B(ENSMLUP_22383)
MAEGGEAGPGEQDRPSGPRPPSARDLQLALAELYKDEVKCKSSKTDRPKASVFKSPRTPP
QRFYSSEHEYSGLHLVRPSPGKIVNELFKEAKEHGAVPLHEATRASGDDKTKSFTGGGYR
LGNSICKQSEYIYGENQMQDVQILLKLWSNGFSLDDGELRPYNDPVNAQFLESVKRGEIP
PELQRLVHGGQVNLDMEDHQDQEYIKPRLRFKAFSGDGQKLGSLTPEIVSTPSSPEEEEK
SILNAAVLIDDSVPTTKIQIRLADGSRLIQRFNSTHRILDVRDFIVQSRPEFANLDFILI
TSFPSKELTDESLTLQEAGIINTVILQQLK

>Gallus gallus_UBXN2B(ENSGALP_24843)
MADGGASPAQQEGEMSAAGPGLRRERQHRGSGRPPSARDLQLALAELYEDEAKRQSLRSD
KPTTTKMSNSKGLKIDSFRSLRKPERSMSDDKENQRFYSGDSEYRGLQISGASNNPSKIV

AELFKEAKEHGAVPLDEASRTSGDFSKAKSFSGGGYRLGDSSQKHSEYIYGNQDVQILLK
LWRNGFSLDDGELRSYSDPINAQFLESVKRGEIPVELQRLVHGGQVNLDMEDHQEQEYVK
PRLRFKAFSGEGQKLGSLTPEIVSTPSSPEEEDKSILNAPVLIDDSVPATKIQIRLADGS
RLIQRFNQTHRIKDIRDFIIQSRPAFATTDFVLVTTFPNKELTDESLTLREADILNTVIL
QQLK

>Anolis carolinensis_UBXN2B(ENSACAP_5211)
VGQGVRATTKGREKESPPSLPPSFSNCTLALAGLYEDEMKRQPLHSEKSTTRVASHPRLP
VQRLASSEYLFGVINIITPLLNKIVDELFKEAREHGAVPLNEVSRASGDCYKAKSFSGGG
YRLGDSTWKRSEYIYGENQFGQDVQILLKLWRNGFSLDDGELRSYTDPVNADFLESVKRG
EIPAELQRLVHGGQINLDMEDHQEQEYVRPRLRFKAFSGEGQKLGSLTPEIVSTPSSPEE
EEKSFADGAVLVDDSVPTTKIQIRLADGSRLIQRFNRTHRIVDIRNFIIQSRPLFANTDF
VLLTTFPHKELTDESMTLQESDILNTVILQQLK

>Homo sapiens_NSFL1C(ENSP_216879)
MAAERQEALREFVAVTGAEEDRARFFLESAGWDLQIALASFYEDGGDEDIVTISQATPSS
VSRGTAPSDNRVTSFRDLIHDQDEDEEEEEGQRFYAGGSERSGQQIVGPPRKKSPNELVD
DLFKGAKEHGAVAVERVTKSPGETSKPRPFAGGGYRLGAAPEEESAYVAGEKRQHSSQDV
HVVLKLWKSGFSLDNGELRSYQDPSNAQFLESIRRGEVPAELRRLAHGGQVNLDMEDHRD
EDFVKPKGAFKAFTGEGQKLGSTAPQVLSTSSPAQQAENEAKASSSILIDESEPTTNIQI
RLADGGRLVQKFNHSHRISDIRLFIVDARPAMAATSFILMTTFPNKELADESQTLKEANL
LNAVIVQRLT

>Pongo abelii_NSFL1C(ENSPPYP_12121)
MAAERQEALREFVAVTGAEEDRARFFLESAGWDLQIALASFYEDGGDEDIVTISQATPSS
VSRGTAPSDNRVTSFRDLIHDQDEDEEEEEGQRFYAGGSERSGQQIVGPPRKKSPNELVD
DLFKGAKEHGAVAVERVTKSPGETSKPRPFAGGGYRLGAAPEEESAYVAGEKRQHSSQDV
HVVLKLWKSGFSLDNGELRSYQDPSNAQFLESIRRGEVPAELRRLAHGGQVNLDMEDHRD
EDFVKPKGAFKAFTGEGQKLGSTAPQVLSTSSPAQQAENEAKASSSILIDESEPTTNIQI
RLADGGRLVQKFNHSHRISDIRLFIVDARPAMAATSFILMTTFPNKELADESQTLKEANL
LNAVIVQRLT

>Macaca mulatta_NSFL1C(ENSMMUP_21035)
MAAERQEALREFVAVTGAEEERARFFLESAGWDLQIALASFYEDGGDEDIVTISQATPSS
VSRGTAPSDNRVTSFRDLIHDQDEDEEEEEGQSSGFYAGGSERSGQQIVGPPRKKSPNEL
VDDLFKGAKEHGAVAVERVTKSPGETSKPRPFAGGGYRLGAAPEEESAYVAGEKRQHSSQ
DVHVVLKLWKSGFSLDNGELRSYQDPSNAQFLESIRRGEVPAELRRLAHGGQVNLDMEDH
RDEDFVKPKGAFKAFTGEGQKLGSTAPQVLSTSSPAQQAENEAKASSSILIDESEPTTNI
QIRLADGGRLVQKFNHSHRISDIRLFIVDARPAMAATSFILMTTFPNKELADESQTLKEA
NLLNAVIVQRLT

>Mus musculus_NSFL1C(ENSMUSP_28949)
MAEERQDALREFVAVTGTEEDRARFFLESAGWDLQIALASFYEDGGDEDIVTISQATPSS
VSRGTAPSDNRVTSFRDLIHDQDEEEEEEEGQRFYAGGSERSGQQIVGPPRKKSPNELVD
DLFKGAKEHGAVAVERVTKSPGETSKPRPFAGGGYRLGAAPEEESAYVAGERRRHSGQDV
HVVLKLWKTGFSLDNGDLRSYQDPSNAQFLESIRRGEVPAELRRLAHGGQVNLDMEDHRD
EDFVKPKGAFKAFTGEGQKLGSTAPQVLNTSSPAQQAENEAKASSSILINEAEPTTNIQI
RLADGGRLVQKFNHSHRISDIRLFIVDARPAMAATSFVLMTTFPNKELADENQTLKEANL
LNAVIVQRLT

>Rattus norvegicus_NSFL1C(ENSRNOP_11654)
MAEERQDALREFVAVTGAEEDRARFFLESAGWDLQIALASFYEDGGDEDIVTISQATPSS
VSRGTAPSDNRVTSFRDLIHDQDEEEEEEEGQRFYAGGSERSGQQIVGPPRKKSPNELVD
DLFKGAKEHGAVAVERVTKSPGETSKPRPFAGGGYRLGAAPEEESAYVAGERRRHSGQDV
HVVLKLWKTGFSLDNGDLRSYQDPSNAQFLESIRRGEVPAELRRLAHGGQVNLDMEDHRD
EDFVKPKGAFKAFTGEGQKLGSTAPQVLNTSSPAQQAENEAKASSSILINEAEPTTNIQI
RLADGGRLVQKFNHSHRISDIRLFIVDARPAMAATSFVLMTTFPNKELADENQTLKEANL
LNAVIVQRLT

>Felis catus_NSFL1C(ENSFCAP_11914)
FQIALASFYEDGGDEDIVTISQATPSSVSRGTAPSDNRVTSFRDLIHDQDEEEEEEEGQR

HRFYAGGSERSGQQIVGPPRKKSPNELVDDLFKGAKEHGAVAVERVTKSPGETSKPRPFA
GGGYRLGAAPEEESAYVAGERRRHSGQDVHVVLKLWKSGFSLDNGELRSYQDPSNAQFLE
SIRRGEVPAELRRLAHGGQVNLDMEDHRDEDFVKPKGAFKAFTGEGQKLGSTAPQVLNTS
SPAQQAENEAKASSSVSIDESQPTTNIQIRLADGGRLVQKFNHSHRISDIRLFIVDARPA
MAATSFVLMTTFPNKELADESQTLKEANLLNAVIVQRLT

>Myotis lucifugus_NSFL1C(ENSMLUP_5784)
KMAERQEALREFVAVTGTEDDRARFFLESAGWDLQIALASFYEDGGDEDIVTISQATPSS
VSRGTVSSISSDNRVTSFKDLIHDQDEDEEEEGQRFYAGGSERSGQQIVGPPRKRSPNE
LVDDLFKGAKEHGAVAVEQMTRSPGETSKPKPFAGGGYRLGAAPEEESAYVAGERRRHSS
QDVHIVLKLWKSGFSLDNGELRSYQDPSNAQFLESIRRGEVPAELRRLAHGGQVNLDMED
HRDEDFVKPKGAFKAFTGEGQKLGSTAPQVLNTVFPAQQAENEAKASSSISIDESQPTTN
IQIRLADGGRLVQKFNHSHRISDIRLFIVDARPAMAATSFVLMTTFPNKELADENQTLKE
ANLLNAVIVQRLT

>Gallus gallus_NSFL1C(ENSGALP_9964)
MADREEALREFVAVTGAEEERARFFLESAGWDLQIALASFYEDGGDEDILTLPQPTPSSV
SRGTAASDHRVTSFRDLVHAQEDDDEEEEGQRFYAGGSERSGQQIVGPPRKKSPNELVED
LFKGAKEHGAVAVDRTAKSSGESSKPKPFAGGGYRLGATPEEESAYVAGERRHNSVQDVH
VVLKLWKTGFSLDSGELRSYQDPSNAQFLDDIRRGEVPAELRRLARGGQVNLDMEDHRDE
EYVKPKSVFKAFTGEGQKLGSTAPQVLSTSSPAQQAENEAKASSAIAIDESEPVTNIQIR
LADGGRLVQKFNHNHRIRDIRLFIVDARPAMAATSFVLMTTFPNKELTDENQTLKEANLL
NAVIVQRLT

>Anolis carolinensis_NSFL1C(ENSACAP_8142)
MADREEALREFVAVTGAEEERARFFLESAGGDLQIALASFYEDGGDEDIITLPQPAPSSV
TRGTTPSDHRVTSFRDLVHAQEEEEEEEEGQRFYAGGSERSGQQIVGPPRKKSPNELVED
LFRGAKEHGAVAVERTAKSPGEGSRPRPFAGGGYRLGAAPEEESAYVAGESRQNAAQDVH
VVLKLWKSGFSLDGGELRSYQDPSNSQFLESIRRGEVPAELRRLARGGQVNLDLEDHRDE
DFVKPRGTFRAFTGEGQKLGSTAPQVMGSGSLAQQAENEAKASSSITIDESEPTTNIQIR
LADGGRLVQKFNHSHRIRDVRLFIADARPAMAATSFVLMTTFPNKELSDEDLTLKEANLL
NAVIVQRLI

>Ciona intestinalis_(ENSCINP_2958)
VMSSQDEMVTEFRGITDASEERARFFLESSGWQLQVALSSFFDSGIAENPTAMSEVEPKR
TTRSSKFASVHDYKNNKNDDSSEEEGQRYYAGGSEHSGELIVGPPRKKNTNQQIKDLFKE
AKEHGAEVVDEPRKHGKEKEKKKYFTGAGYKLGDGGEDSPSVFVPGEVEQQRPGPVNVVL
KLWSNGFTVDDGPLRDFNDPQNQEFLQSVKKGQIPQELIRNAKGGEVHVDMEDHREEDYK
PQKKKLKPFSGQGQMLGSPTPQVETSPAPSISSSVDPPISIDQSKPSTNIQIRLLDGTRI
RQQFNHDHRVSDIRSFILNSQPNMGSRPFVLMTTFPNKELTNENETIAGAQLLNSQVVQK
LK

>Branchiostoma floridae_(XP_002609968)
MADKDELQSQFVAVTGVDQGRAKFFLESAQWDLQVAIAHFYDTAGDDDMEETSPQAAGTSAAPPGDSAKG
GRQESESDEEEGQAYYAGGSEHGGGQQILGPPKKKPNPSTDDVVDKLFQSAKDHGAETVEPEEAAARPKP
LAFKGTGYRLGATEEDTQVVQGERDASRRQEKTIVLRMWKNGFTVDDGELRAYDDPANQEFLNSINKGEV
PLELIRMCRGLEVALNLEDHRHEEWAPPKVAVKAFSGEGHKLGSPTPNVVSAPAAAAGSGDRKTNEAKAQ
SNVGMKDSEPTTSIQIRLADGSRLVAKFNHTNRVSDVRQFIATARPETAVTPFVLMTTFPNKELTDESQT
LKEANLLNAVIVQKMK

>Saccoglossus kowalevskii_(XP_006822016)
MVTEFTNITGSDLERAQFFLESSGWQLQVAIASFYDEAEMDDTPDTLPSGVGGRVSEPEGVAPPPYSATG
GSRFATLSDMMPVGDEDDSSEDEGETFYAGGAERGGSGQQIVGPAKKKKASKIVEEMFQSAKEHGAQEVS
AATPVPSGSSAGASFGGAGYRLGDTEGGDVRPVPGTSRSKQDQPKDMHVVLKLWKNGFTVNDTELRAFSD
PKNAEFLNSVTKGEIPKELHNKAKGGEVNLDMEDHRDEEYVPAKQALKPFTGEGFKLGNPTPKVVTQSTP
SVPANVLNTNPIEVDKNKPITQLQLRLADGSRLSGQFNNTHTVGDIRRFIIYSRPQYSSTVFVLQTTFPN
RELTDDSKTLIEADVWNSLIVQRLK

X Kell Blood Group Precursor-related Family-XKR

>Homo sapiens_XKR4(ENSP_480076)

MAAKSDGRLKMKKSSDVAFTPLQNSDHSGSVQGLAPGLPSGSGAEDEEAAGGGCCPDGGG
CSRCCCCCAGSGGSAGSGGSGGVAGPGGGGAGSAALCLRLGREQRRYSLWDCLWILAAVA
VYFADVGTDVWLAVDYYLRGQRWWFGLTLFFVVLGSLSVQVFSFRWFVHDFSTEDSATAA
AASSCPQPGADCKTVVGGGSAAGEGEARPSTPQRQASNASKSNIAAANSGSNSSGATRAS
GKHRSASCSFCIWLLQSLIHILQLGQIWRYFHTIYLGIRSRQSGENDRWRFYWKMVYEYA
DVSMLHLLATFLESAPQLVLQLCIIVQTHSLQALQGFTAAASLVSLAWALASYQKALRDS
RDDKKPISYMAVIIQFCWHFFTIAARVITFALFASVFQLYFGIFIVLHWCIMTFWIVHCE
TEFCITKWEEIVFDMVVGIIYIFSWFNVKEGRTRCRLFIYYFVILLENTALSALWYLYKA
PQIADAFAIPALCVVFSSFLTGVVFMLMYYAFFHPNGPRFGQSPSCACEDPAAAFTLPPD
VATSTLRSISNNRSVVSDRDQKFAERDGCVPVFQVRPTAPSTPSSRPPRIEESVIKIDLF
RNRYPAWERHVLDRSLRKAILAFECSPSPPRLQYKDDALIQERLEYETTL

>Pan troglodytes_XKR4(ENSPTRP_34691)
RYFHTIYLGIRSRQSGENDRWRFYWKMVYEYADVSMLHLLATFLESAPQLVLQLCIIVQT
HSLQALQGFTAAASLVSLAWALASYQKALRDSRDDKKPISYMAVIIQFCWHFFTIAARVI
TFALFASVFQLYFGIFIVLHWCIMTFWIVHCETEFCITKWEEIVFDMVVGIIYIFSWFNV
KEGRTRCRLFIYYFVILLENTALSALWYLYKAPQIADAFAIPALCVVFSSFLTGVVFMLM
YYAFFHPNGPRFGQSPSCACEDPAAAFPLPPDVATSTLRSISNNRSVVSDRDQKFAERDG
CVPVFQVRPTAPSTPSSRPPRIEESVIKIDLFRNRYPAWERHVLDRSLRKAILAFECSPS
PPRLQYKDDALIQERLEYETTL

>Mus musculus_XKR4(ENSMUSP_70648)
MAAKSDGRLKMKKSSDVAFTPLQNSDNSGSVQGLAPGLPSGSGAEDTEAAGGGCCPDGGG
CSRCCCCCAGSGGSAGSGGSGGGGRGSGAGSAALCLRLGREQRRYSLWDCLWILAAVAVY
FADVGTDIWLAVDYYLRGQRWWFGLTLFFVVLGSLSVQVFSFRWFVHDFSTEDSSTTTTS
SCQQPGADCKTVVSSGSAAGEGEVRPSTPQRQASNASKSNIAATNSGSNSNGATRTSGKH
RSASCSFCIWLLQSLIHILQLGQIWRYLHTIYLGIRSRQSGESGRWRFYWKMVYEYADVS
MLHLLATFLESAPQLVLQLCIIVQTHSLQALQGFTAAASLVSLAWALASYQKALRDSRDD
KKPISYMAVIIQFCWHFFTIAARVITFALFASVFQLYFGIFIVLHWCIMTFWIVHCETEF
CITKWEEIVFDMVVGIIYIFSWFNVKEGRTRCRLFIYYFVILLENTALSALWYLYKAPQI
ADAFAIPALCVVFSSFLTGVVFMLMYYAFFHPNGPRFGQSPSCACDDPATAFSLPPEVAT
STLRSISNNRSVASDRDQKFAERDGCVPVFQVRPTAPPTPSSRPPRIEESVIKIDLFRNR
YPAWERHVLDRSLRKAILAFECSPSPPRLQYKDDALIQERLEYETTL

>Canis familiaris_XKR4(ENSCAFP_10384)
CCAGSAGSGGGSAGGSGGGSGVSGGPGAPDGGGAGSAALCMRLGRERRRYSLWDCLWILA
AVAVYFADVGTDIWLAVDYYLRGQRWWFGLTLFFVVLGSLSVQVFSFRWFVHDFSTEDSA
PAAAASSCPQPGADCKTAAGGGSAAAEGEARPSTPQRQASNASKSNIAAANSGSNSSGST
NIMKKVRSSSCSFCKILYSLISILSYSPCFLSRYFHTIYLGIRSRQSGENDRWRFYWKMV
YEYADVSMLHLLATFLESAPQLVLQLCIIVQTHRLQALQGFTAAASLVSLAWALASYQKA
LRDSRDDKKPISYMAVVIQFCWHFFTIAARVITFALFASVFQLYFGIFIVLHWCIMTFWI
VHCETEFCITKWEEIVFDMVVGIIYIFSWFNVKEGRTRCRLFIYYFVILLENTALSALWY
LYKAPPFADAFAIPALCVVFSSFLTGVVFMLMYYAFFHPNGPRFGQSPSCACEDPVAAFT
LPPEVATSTLRSISNNRSVASDRDQKFVERDGCVPVFQVRPTAPSTPSSRLPRIEESVIK
IDLFRNRYPAWERHVLDRSLRKAILAFECSPSPPRLQYKDDALIQERLEYETTL

>Myotis lucifugus_XKR4(ENSMLUP_9777)
MAAKSDGRLKMKKSSDVAFTPLQNSDHSGSVQGLAPGLPSGSGAEDEEAAGGGCCPDGGG
CSHCCCCCSGSGGSGGGSGGSGGPGGSVGGPGGGASSAALCLRLGGEQRRYSLWDCLWIL
AAVAVYFADVGTDIWLAVDYYLRGQRWWFGLTLFFVVLGSLSVQVFSFRWFVHDFSTEDS
ATAAAAASSCSQPGADCKTLVRSGSAAAEGEVRPSTPQRQASNASKSNIAAPNTGSNSSG
ASGKHRSASCSFCIWLLQSLIHILQLGQIWRYFHTIYLGIRSRQSGENDRWRFYWKMVYE
YADVSMLHLLATFLESAPQLVLQLCIIVQTHSLQALQGFTAAASLVSLAWALASYQKALR
DSRDDKKPISYMAVVIQFCWHFFTIAARVITFALFASVFQLYFGIFIVLHWCIMTFWIVH
CETEFCITKWEEIVFDMVVGIIYIFSWFNVKEGRTRCRLFIYYFVILLENTALSALWYLY
KAPQIADAFAIPALCVVFSSFLTGVVFMLMYYAFFHPNGPRFGQSPSCACEDPVAAFTLP
PEVATSTLRSISNNRSVVSDRDQKFAERDGCVPVFQVRPTAPSTPSSRPPRIEESVIKID
LFRNRYPAWERHVLDRSLRKAILAFECSPSPPRLQYKDDALIQERLEYETTL

>Anolis carolinensis_XKR4(ENSACAP_17702)
IFPQKSFFRYFHTIYLGIRSRRSGDNDRWRFYWKMVYEYADVSMLHLLATFLESAPQLVL

QLCIVVQTRTLQALQGLTAAASLVSLAWALASYQKALRDSRDDKKPISYMAVIIQFCWHF
FTIAARVITFALFASVFQLYFGIFIVLHWCIMTFWIVHCETEFCITKWEEIVFDMVVGII
YIFSWFNVKEGRTRCRLFIYYFVILLENTALSALWYLYKAPMISDAFAIPALCVVFSSFL
TGIVFMLMYYAFFHPNGPRFGQSPSCACEDSVAAFTLPPEVASSTLRSISNNRSVTSDRD
QKFAERDGCVPVFQVRPTAPSTPSSRPPRIEESVIKIDLFRNRYPAWERHVLDRSLRKAI
LAFECSPAPPRLQYKDDALIQERLEYETTL

>Xenopus tropicalis_XKR4(ENSXETP_55756)
MAAKSDGMLKMKKSDVAFTPLQNSDHSGSVQGLATGSQPGSATGDGGCSDGSSRCCCSGG
SSSGRVAGGGGGVGGASPSLCLRLGREQQRYSVWDCLWILAAVAVYFADVGSDIWLSVDY
YLRGQRWWFGLTLFFVVLGSLSVQVFSFRWFVHDFSTENSSQCNPMESKAAAGSAAACEG
DARPSTPQRQASTASSKPATTGTNRANSRDSSSSCSFCIWIMQSLIHILQLGQIWRYFHL
NRLGFRERASGEXERWSFTFNYIHEEFTDRLVLHTLYIYLYTQPQLILQVCHKVKVYHIT
CNIAGLTAAASLVSLAWALASYQKALRDSRDDKKPISYMAVIIQFCWHFFTIAARVITFA
LFASVFQLYFGIFIVLHWCIMTFWIVHCETEFCITKWEEIVFDMVVGIIYIFSWFNVKEG
RTRCRLFIYYFVILLENTALSTLWYLNKAPQILDAFAIPALCVVFSSFLTGIVFMLMYYA
FFHPNGPRFGQSPSCACEDTVTAFPLPPEGASNTLRSMSNNRSVASDRDQKFTERDGCMP
VFQVRPIAPSTPSSRPPRIEESVIKIDLFRNRYPAWERHVLDRSLRKAILAFECSPSPPR
LQYKDDALIQERLEYETTL

>Takifugu rubripes_XKR4(ENSTRUP_24449)
MAAKSDGVLKMKKSDVAFTPLQNSEHSGSVQGLHPGAQLDSAGTGDADFANGESRCCGGG
GSNSTCLRLGREQQKYTVWDCLWILAAVAVYVADVGSDVWLSVDYYLHNDYWWFGLTLFF
VVLGSFSVQLFSFRWFVHDFSTEDGAVSAADCTHMDGNKLLSGSASHGDVTAQQHPATPQ
RQASTASKNTTTNSTASTAVGRSEKRSAYYTFCVWISQSIIHILQLGQIWRYIHTIYLGI
QSHQSVETERWRYYWRMVYEFADVSMLHLLATFLESAPQLVLQLCIIIQTHKLQAVQENA
GMTAAASLVSLAWALASYQKALRDSRDDKKPISYLAVIIQFCWHFFTIAARVITFALFAS
VFQLYFGIFIVLHWCIMTFWIVHCETDFCISKWEEIVFDMVVGIIYIFSWFNVKEGRTRC
RLFIYYLLILVENAALSALWYLYRSPLATDAFAVPALCVIFSSFFTGVVFMLMYYAFFHP
NGPRFGRSLSGHGLNLDPTAQFSTLPSEVATNSLRSNRGATATLERDMGKYSERDGCMPV
FQVRPTVPSTPSSRAPRPEETVIKIDLCRNRYPAWERHVLDRSIRKAILAVDSSQTPPRL
QYKDDALVQERLEYETTL

>Tetraodon nigroviridis_XKR4(ENSTNIP_21301)
GGGSHSTCLRLSREQQKYTVWDCLWIVAAVAVYVADVGSDVWLSVDYYLEEDYWWFGLTL
FFVVLGSFSVQLFSFRWFVHDFSTEDSAEAAADGSHMDGNKLLSGSASHHGQHRAGPQGP
KRPAYYTFCVWGSQSVIHILQLGQIWRYIHTIYLGVQSRQSAETERWRYYWRMVYEFADV
SMLHLLATFLESAPQLVLQLCIIIQTHKLLAVQGMTAAASLVSLAWALASYQKALRDSRD
DKKPISYLAVIIQFCWHFFTIAARVITFALFASVFQLYFGIFIVLHWCIMTFWIVHCETD
FCISKWEEIVFDMVVGIIYIFSWFNVKEGRTRCRLFIYYLLILAENAALSALWYLYRSPL
ATDAFAVPALCVIFSSFLTGVVFMLMYYAFFHPNGPRFGRSMSGHGLNLDPTAQFSTLPS
EVATNSLRSNRGATATLERDAGKYSERDGCMPVFQVRPTVPSTPSSRAPRLEETVIKIDL
CRNRYPAWERHVLDRSIRKAILAVDCSLTPPRLQYKDDALVQERLEYETTL

>Gasterosteus aculeatus_XKR4(ENSGACP_19427)
RSAYYSFCVWFGQSVIHILQMGQIWRYFHTIYLGVRSHQSAETERWRYYWRMVYEFADVS
MLHLLATFLESAPQLVLQLCIIIQTHKLQAVQGMTAAASLVSLAWALASYQKALRESRDD
KKPISYLAVVIQFCWHFFTIAARVITFALFASVFQLYFGIFIVLHWCIMTFWIVHCETDF
CISKWEEIVFDMVVGIIYIFTWFNVKEGRTRCRLFIYYLVILVENAALSALWYLYRSPHT
TDAFAVPALCVIFSSFLTGVVFVLMYYAFFHPNGPRFGRSPSGQGLDLDPSAQFSTLPYE
GTTNSLRSNRGATATLERDGGKYSERDGCMPVFQVRSTVPSTPSSRAPRLEETVIKIDLC
RNRYPAWERHVLDRSIRKAILAVDSSLTPPRLQYKDDALVQERLEYETTL

>Oryzias latipes_XKR4(ENSORLP_5348)
MAAKSDGVLKMKKSDVAFTPLQNSEHSGSVQGLHPGEQPDSACADGDFINGESRCCGGVG
SNSTCLRLGAELQQYSLWDCLWILAAVAVYVADVGTDVWLSVDYFLHGDYWWFSLTLFFV
MLGSFSVQLFSFRWFVHDFSTEDSAESAADCFHVDGKKLLSGSASHGDVTAQQHPGAPQR
QASTASKNTASNSTASAALGGRGTRRSRHYTFCIWFGQSVIHILQLGQIWRYLRTIYLGV
RSRQSAESERWRWHWKMVYEFADVSMLHLLATFLESAPQLVLQLCIIIQTHKLQAVQGMT
AAASLVSLAWALASYQKSLRDSRDDKKPVSYLAVIIQFCWHFFTIAARVITFALFASVFL
YFGIFIVLHWCIMTFWIVHCETDFCISKWEEIVFDMVVGIIYIFSWFNVKEGRTRCRLFI

YYFVILVENAALSALWYLYRIPKTTDAFAVPALCVIFSSFLTGVVFLLMYYAFFHPNGPR
FGRSPSGQGLNLDPTAQFATLPSEGATNSLRSSRGASSTLERDAGKYSERDGCMPVFQVR
PTVPSTPSSRAPRLEETVIKIDLCRNRYPAWERHVLDRGIRKAILAVECSVTPPRLQYKD
DALVQERLEYETTL

>Homo sapiens_XKR5(ENSP_483879)
MHARLLGLSALLQAAEQSARLYTVAYYFTTGRLLWGWLALAVLLPGFLVQALSYLWFRAD
GHPGHCSLMMLHLLQLGVWKRHWDAALTSLQKELEAPHRGWLQLQEADLSALRLLEALLQ
TGPHLLLQTYVFLASDFTDIVPGVSTLFSWSSLSWALVSYTRFMGFMKPGHLAMPWAALF
CQQLWRMGMLGTRVLSLVLFYKAYHFWVFVVAGAHWLVMTFWLVAQQSDIIDSTCHWRLF
NLLVGAVYILCYLSFWDSPSRNRMVTFYMVMLLENIILLLLATDFLQGASWTSLQTIAGV
LSGFLIGSVSLVIYYSLLHPKSTDIWQGCLRKSCGIAGGDKTERRDSPRATDLAGKRTES
SGSCQGASYEPTILGKPPTPEQVPPEAGLGTQVAVEDSFLSHHHWLWVKLALKTGNVSKI
NAAFGDNSPAYCPPAWGLSQQDYLQRKALSAQQELPSSSRDPSTLENSSAFEGVPKAEAD
PLETSSYVSFASDQQDEAPTQNPAATQGEGTPKEGADAVSGTQGKGTGGQQRGGEGQQSS
TLYFSATAEVATSSQQEGSPATLQTAHSGRRLGKSSPAQPASPHPVGLAPFPDTMADISP
ILGTGPCRGFCPSAGFPGRTLSISELEEPLEPKRELSHHAAVGVWVSLPQLRTAHEPCLT
STPKSESIQTDCSCREQMKQEPSFFI

>Pan troglodytes_XKR5(ENSPTRP_54982)
MHAGLLGLSALLQAAEQSARLYTVAYYFTTGRLLWGWLALAVLLPGFLVQALSYLWFRAD
GHPGHCSLVMLHLLQLGVWKRHWDAALTALQKEPEAPHRGWLQLQEADLSALRLLEALLQ
TGPHLLLQTYVFLASDFTDIVPGVSTLFSWSSLSWALVSYTRFMGFMKPGHLAMPWAALF
CQQLWRMGMLGTRVMSLVLFYKAYHFWVFVVAGAHWLVMTFWLVAQQSDIIDSTCHWRLF
NLLVGAVYILCYLSFWDSPSRNRMVTFYMVMLLENTILLLLATDFLQGASWTSLQTIAGV
LSGFLIGSVSLVIYYSLLHPKSTDIWQGCLRKSCGIAGGDKTERRASPRATDLAGKRTES
SGSCQGASYELTILGKPPTPEQVPPEAGLGTQVAVEDSFLSHHHWLWVKLALKTGNMSKI
NAAFGDDNPVYCPPAWGLSQQDDLQRKALSAQRELPSSSRHPSTLENSSAFEGVSKAEAD
PLETSSYVSFASDQQDEAPTQNPAATQGEGTPKEGADAVSGTQGKGTGGQQRGGEGQQSS
TLYFSATAEVATSSQQEGSPATLQTAHSGRRLGKSSPAQPASPHPVGLAPFPATMADISP
ILGTGPCRGFCPSAGFPGRTLSISELEEPLEPTRELSHHAAVGVWMSLPQLRTAHEPCLT
STPKSESIQTDCSCREQMKQEPSFFI

>Gorilla gorilla_XKR5(ENSGGOP_11430)
MHAGLLGLSALLQAAEQSARLYTVAYYFTTGRLLWGWLALAVLLPGFLVQALSYLWFRAD
GYPGHCSLVMLHLLQLGVWKRHWDAALTALQKEPEAPHRGWLQLQEADLSALRLLEALLQ
TGPHLLLQTYVFLASDFTDIVPGVSTLFSWSSLSWALVSYTRFMGFMKPGHLAMPWAALF
CQQLWRMGMLGTRVMSLVLFYKAYHFWVFVVAGAHWLVMTFWLVAQQSDIMDSTCHWRLF
NLLVGAVYILCYLSFWDSPSRNRMVTFYMVMLLENIILLLLATDFLQGASWTSLQTIAGV
LSGFLIGSVSLVIYYSLLHPKSTDIWQGCLRKSCGIAGGDKTERRASPRATDLVGKRTES
SGSCQGASYELTILGKPPTPEQVPPEAGLGTQVAVEDSFLSHHHWLWVKLALKTGNVSKI
NAAFGDDSPVYCPPAWGLSQQDYLQRKALSAQRELPSSSRHPSTLENSSAFEGIPKAEAD
PLETSSYVSFASDQQDEAPTQNPAATQGEGTPKEGADTVSGTQGKGTGGQQRGGEGQQSS
TLYFSATAEVATSSQQEGSPATLQTAHSGRRLGKSSPAQPASPHPVGLAPFPATMADISP
ILGTGPCGGFCPSAGFPGRTLSISELEEPLQPTRELSHHAAVGVWMSLPQMRTAHEPCLT
STPKSESIQTDCSCSEQMKQEPSFFI

>Mus musculus_XKR5(ENSMUSP_93089)
MHAGLLGLSALLQAAEQSARLCSIVFYFATGRLLWGWLALSVLLPGFLVQALSFLWFRAD
GHQGQWWLAVLHLLQLGVWKRHWDSVATALWKGKEAPSWGQLHLQEADLSALRLLEALLQ
TGPHLLLQAYVFLASDFTDIVPGISALLSWSSLSWALVSYNRFLGIMKPGHHTMLWAALL
CQQLWRMGMLGARVLSLVLFCRVYRVWVLVVGGAHWLVMTFWLVAQQSDIVESTCHWRLF
NLLVGAVFILCYINFWDSPSRSRVASFYLVMLLENSILLLLATDFLQGVPGTSLWTVVGV
LSGFLIGCASLVIYYSLLHPKSSDIQQSFMRKCCGPIEDNKPESEPPPRAVDPTGEMPDS
SSWCQEESYELTSLDKAPSPEQNTAEVGLGEQRSGESSFFSHHHWLLLKLALKTGSVSRI
NAALGGDSPGCSCPPLLGSSQHCDLQRKPLFSHQDLPSSPCDPLTLEKGSEYVGAPKAEM
ESLETSSYLSFASELEDNATTQKPPATQEDSPKLAGSKADLAAQGKETEGPLQGKEGQES
TTLYFSAAMDRTTSHQRGSPVVLRISHSETLVESRPGQPALPQAVTKPFPVTVANISPIP
GRNFRPSAELPGRAPDSSECEEWKDAARDPSMQSSLPKMRLKAAEEPCFTSTPKSESIQR
DYSCRDRVRQEMSFFI

>Rattus norvegicus_XKR5(ENSRNOP_32580)
MKPGHRTMLWAALLCQQLWRMGMLGARVLTLVLFCRVYRVWVLVVGGAHWLVMTFWLVAQ
QSDIVESTCHWRLFNLLVGAVFILCYINFWDSPSRSRMASFYLVMLLENSILLLLATDFL
QGAPWTSLWTVVGVLSGFLIGCASLVVYYSLLHPKSSDIQENFMRKCCGPIENNKSDSEP
PPRAMVPTGERPDSSSWCQEKSYELTSLDKAPSPGKSTAELRLEEQRSGEDSFFSHHHWL
LLKLALKTGSISKINAALGGDSPGCSCPPRLGSSQHCDLQRKPLFSQQDLPSSPRDPLTL
EKGPEYVGAPKAEMESLETSSYISFASDLEDNATTQKPPATQEDSPKAGTKADLAAQGKD
TDGPLKGKEGQESTTLYFSATMDRTTCHQKGSPVALRTSHLGTLGESRPVQPALPQAVTK
PFPVALANISPIPGRNFCPDAGLPGRAPESSEWEEWEDPAKNPSTQSSLPKVRLRAAEEP
CVTSTPKSESIQRDYNCRDRVKQEVSFFI

>Canis familiaris_XKR5(ENSCAFP_36968)
QRGGLVFLNARTRCQSRPAGLCCVVHYFTTGQLLWGWLALALLLPGCLVQVLSYLWFRAD
GQQGHGLLVVLHLLQLGVWKRHWDAASSALSKEGEASRRDQLLLQEADLSALRLLEALMQ
TGPHLLLQTHVFLASDFTELVPGVSALCSWAALSWALVCYARLAGLLKPGRLPVPWAALF
CQQLWRMGMLGARVLSLALFSRAYHAWVLVVGGAHWLVMTFWLVAQQSDVVDSTCHWRLF
NLLVGAVYILCYLNFWDSPSRHRMAMFYTIMLLENIVLLLLATDFLQGASQTSLWTIAGV
SSGFLIGSVSLVTSYGLLRPPSAGGRRGRGVAAGDGADAESCPRGPAPARGSPGAGQEDG
GEFTALGRAPGPQWGPPEAGLESLTAREDPFFSHHHWLLLKLALKTGNMSKINAAFGEDS
PGRFGPPARGVGQHSAQQGTPLFPQQELPPSPPGPQASEKGSGIPGVLSAEADLLEASSF
ASLAGDKHDQAPGQKRPPAPTQGRPGEGAGAASPLQGRAAGARTGAQGLEGSTRDFSATA
EGGPSPQQEGGQATPHTCAPGRRPRSPPRPAPRPFPAAVADISPILRPGLSRSFVGTAPG
SSERGEQREPTTDRNLPELLSLPKRSLWPADQPCLTSTPKCE

>Felis catus_XKR5(ENSFCAP_12849)
RHQKTQCRRAWLAAREEPARLCSVVYYFITGQPLWGWLALSVLLPGFLVQGLSYLWFRAD
GHQGHCLLVMLHLLQLGVWKRHWDAASTALQKEREASSRGQLLLQEADLSALRLLEALLQ
TGPHLLLQTHVFLASDFTDVVPGVSALCSWSALSWALVCYACFMGFVKPGHVFTPWAALL
CQQLWRMGMVGARVLSLVLFFGAHRVWVLVFGGAHWLVMTFWLVAQQSDIIDSTCHWRLF
NLLVGAAYILCYLNFWDSPSRNRMAMFYTIMLLENIVLLLLATDFLQGASRTSLWTIAGV
LSGFLIGSISLVVYYSLLHPKSTDIWRGFVTEVRGTAVGDRAERGSSPRASAAAGERPGS
PGDSCELTHLGRAPSPQWGPPEAGLESQIVQEDSFLGHHHWLLVKLALKTGNVSKINAAF
RKDGPGFFGPPEWGLSQHRNHQMTPRFSQKELASSPHDPLSSDKGCESQVVRKAEADLLD
TSSYVSLAGDNPDEAPGRRWSAAPREGSPREGAEAVSPLQGRGAGDRRGRAGQESATLYF
SATAEGATSPQREAGQATSQTPLSGRSSEPSPRPATRPFPATMASISPILGPGRTGSFRP
STGLPGRALGGSERGGQQEPMGDPNLPTIAGTRMALPEVSLRPADEPCLTSTPKCEPAHG
DCGGRARPRTMPSFSM

>Monodelphis domestica_XKR5(ENSMODP_28839)
MHAGYLVICALLLAAEQGARLCTVVYYFHTKQYLWAWLTLSTLLPGCLIQGLSYLWFLAD
GHQGSCSLVIVHLLQLGTWKRHWDTFLTTMTKEEESPDMRKVLLHQGDLFVLRLLEALLQ
IGPHLLLQAYAFISADFQDPVPGVSAVLSWLSLSWSLVSYTRIMSLIKPGHLAMPWAALL
CQLLWRLAMLGTRIITLVLFSQAYHCWVLVVGGAHWLVMTFWLVAQQSDIIDSTCCWRLF
NLLVGALYVFCYINFWDSPSRNRMTTFYTIMLMENIILLLLATDFLQGASWGTMWMTIGI
VSGFIIGCASLVIYYSLLHPKSTEIWQGFIRKFCGSVDSDKTEKEPSSFQCTKPVEEKSE
SLGLYPVEDPKDPKYQGNSLSMDWGASTGSQVVRDEATAKCHHHWLLVKLALKTGNMSKI
NAAFGNGGLDCFCPPIYITSKCFGPQMKLSFLEKEFPLPQKHCGPPCQTSWQKDSQAEDI
PETKEDPLETSSFITLSSHQHVHAPTHKTIQTKVPWRPEKSISESPIGQGGGKGRRQENS
TFYFSATMEGATPSHHEARTAPPVSCRKVDIEKDQQTNSDPNHLVPKPFPISVANISPIL
GTSTAHRLRHNTGLYNGSQCSLPGVSSAGSRRPLELWTAPGNSFLSVGTWVSMIRSKLKP
SGEPYCTSTPKSEPRKDGDSLRERLRQESNLFF

>Xenopus tropicalis_XKR5(ENSXETP_63692)
LTVGKRFSPGRCGMEASGSSVGCAAVSLLLYLMERGAELAVLVHYFLTGQYTWASCTLAL
LIPGCLVQALSFAWFRKEGPAGGFTVTLIHVLQLGILKRQADCLQVALREGKRQKPGNER
ELLMQQGDAALLRLVEALLQALPHLLLQTYIYLALEYTNAYAVASALLSLLSLSWALVSY
SRFLCLLKPGHLSMPWASLLCQLLWRMGMIGTRAMALAVFARVYHFWVFAVAGAHWLVMS
FWLVAQQTDLISRPCYWKLFNALVGAVYVFCFLNVRDGPSRYRVAIFYTVMLLENAILLL
LATDFLQGVLWSNVRLTVVVMCGFLIGENGGLMDLQVNMSSFHISVANVILYCTRSNCLS
IAFSLPPPPATCNMIKGLGEVMRKDMASQPWLAFPTAVRTTAPICVCFCSQYLPYELSFS
IYPTLTDWRHYYIVYSGGILILELLCRIYTVASLPTDYKETYGDQHSKYGNTNLCRKHRA

MGLIQSNKETPIQLMIRLLHSANVEPEKPGAASVEPIQTPTKEIPAQEGARTEVYPAICV
SPILSLATNSNFQRSIAPDVGSLCDDSSMSNDEADVTEAEELPGEKARLLLAPGLLAPSL
RGRLIPEEKPCFTSTPKPQTASAEPGLREDSKTKRKLLEP

>Danio rerio_XKR5a(ENSDARP_134463)
MWMLLTLCLSSSSVVCCVAFYLWQEQLCFAGWTFALLLPGSLVQVLSFRWYRADGDTHTC
HNFIIHTLHLGIFKRFWDCAVCEWTSPGSAGSHAQEVMQQADVSALRLLEVLLMTVPQTL
LQTYALTVTDYGLSSPVALCSGVCLLSLSWALVLYSRACCLIRPGHLQMPPAALLSQLLW
RTGMLAARFTSLALFTRTFGCWLIGVIASHWLMAALWLVSQQTDIYVGQWSWRVFNFILG
GVHVFLFLNVKDGPSRFRMAGFYTVMLLENATLLLAASEILIDASWDSLTIPTAVLCSFL
LGLTSLILYYRFLHPKSTEISQGLHQRAHMGRECLQQADSSFSLGDKHLPPAALPVLFRS
SHPSFSLSGIPGSLLEHPGSCGVKPDGECRHHHWLLIRLALKTGDPVKINSAYGAGGVSG
ILAVDQKAEMIDDGCISASESRDDTMAPLSDCREEFESMSEAKDDDDEDDSLEMESPLES
PVTLSDCKRSSPEGKSVFGDSPEPNYCPTESSSTLYFSADPQSPSSSSNPRLDRETPFSF
GSGTGLETLDELSPISTDGGLHRDXXXXGQRASGPGGSLLHFRTRVKWRGFGITPQGPSQ
AGGFVP

>Danio rerio_XKR5b(ENSDARP_129253)
MQNRKGSEITWSHVCLYSFSGLIVLLEKAALVLCFTHYLITGQELLAWLTAALCLPISAV
QMLSLKWYITDEERPKPSVILLHCLHLGVYHRLWSCMKSQAEQVQFGAVLIQQADVSVLC
LIEALTLSLPQSLLQTYSLFTLHPGLLSPVVVCIGLSLLNVSWFVVLYSRSCFLLRPAHL
HMTPAALLCQFIWRAGMLSARVTSLMLFCSTFHWWTCGVLGFHWLIMMFWQVSQQTDICI
NRGSWRLFNFILGALYIFIFVNVKDGPSRYRMTGFYLLMLLENTFLLLLASDSFSGPSWD
SMSVPLTVSCSFLIGVIFLILYYRFLHPKSTEILQSLRLQMSLTTIDKTDTDCKDTTLES
STHKHGTFSLTGFPPAQHLHPLEGDGHHHHMLIKLALKTGNHDKINSMYGNRGLSVILGK
NDAKDEDLGESGCKRSSVEAGKASENYQIESTSAAEVKQVSLEKIEYNSCPVDCSSTVYF
SAEAQSYSVSDSILEKDKMAAISPIVNGDEVHLIGKQLLNRNPCYTSTPIADFKTMQTVG
DRLGGVRRQVQF

>Tetraodon nigroviridis_XKR5a(ENSTNIP_15267)
LPVIYCIGFYLWNEEMQWAGLTLGLFLPGTAVQLLSMKWYRDDGDDRRCYLSVIHILHLG
IFKRLWDCMRVVLRRQDSVAELGASIMQQADVAALWLLEALVLTLPQSLLQAYVMVSTDV
GFVSPVGCSCGLCVLSISWALVLYNRACCLIRPGHLAMPPAAMLCQLVWRAGMLGARVAC
LMFFARVFNWWVCGVAGFHWLTASFWLISQQPDICTGPWCWRAFNGIMGLVHVFLFLNVK
DGPSRFRMASFYAFMLVENATLLLAASDFLSEASWDNMTLPTTVLCSFVLSATSLILYYR
FLHPKSTEICQGTDHNHVGSTCIEGGESSFSLGDKSLPVPSIQNHGSFSYSGVAGSLLDH
PGTCGGQPNNSCPCYHHHWLLIRLAMKTGDLGKINRAYGAGGAAAILDMEEYAQDIKGQD
SNTLTVGGSCGGISNSVSQEKGLAPLSDCKDEFQTISEPTSIEHPEEDDSLEMESPLESP
MSDFKRSSPEGKSVFGDSPEPYFCPTESSSTLYFSADPQSPSSASNTRLDRDIGGLEQGV
RLNSLPSDPALHRDVRGLMGRVGPRCTSTPRLDSGALDNPPNIPHLTGPRRQLIMSQRDE
DEIF

>Tetraodon nigroviridis_XKR5b(ENSTNIP_20846)
SPAVIYCFVYYLWVGHNYCYAYLAGFTALFLLPGWGPQWLSYLWYLSDGRIRRRSLTWTH
LLHLGLFKRLWECTCQAEEDVYGYIMQQADVSALRLLEALVVTLPETLLHTYVLICTDIG
IKSPASVCFAVCLLSLAWALVLYARACSLIRPGHLQMTPAAVLCRLLWRVGLRMLGSRFA
VLMLFTRVFKQWVLGVVGVHWLGATFWMVSQQSDIIRSTSRWRLFNLVLGAIHIFFFLNV
KYGQSRCRMAGFYLVMFVENAFLLLASSWLFSMVSWDTVGIPAAVLCSFLIAGVISLVLY
YRFLHPKSFEIFQSIRRRGLGGACTERGSTLSLEEKVTPTFHRQASGGTLMDLPLQWEGW
KHHHWLLIRLALKTGAVTRIWSAYGEGGLAGLMGLSEEFHSPDDFRVPQGSPTPPSPQQP
GSLCHIDSNAGTLTEASSSAGSLDIKTPGWSPERRSPFLIGSPEKKPGASGESSTTLYFS
ADAQSPSSGS

>Gasterosteus aculeatus_XKR5(ENSGACP_12330)
VRVLVIYCIGFYLWNEETQWAGITLGLFLPGTAVQLLSMKWYHDDGDDRRCYLSVIHILH
LGIFKRLWDCMRSVLRMQDSVAELGAAVMQQADVAALWLLEALVLTLPQSLLQAHVVVST
DVGIMSPVAYCCGLCVLSISWALVLYSRACCLIRPGHLAMPPAALLCQLVWRAGMLGARV
TCLMFFARVFNWWVCGVAGFHWLTASFWLVSQQPDICTGPWCWRAFNGVMGLVHVFLFLN
VKDGPSRFRMASFYAFMLVENATLLLAASDFLSEASWDSMTLPATVLCSFLLGATSLVLY
YRFLHPKSTEISQGTNRDHMGSTCIERGESSFSFGDKSLPAASVQNHGSFSLSGVAGSLL
EHPGTCRGRANGSCPCYHHHWLLIRLALKTGDLGKINAAILDMEEYRQEFKSNEGMTITA

GGSCDGVSTSESQGKGLAPLSDCKDEFQSVSEPTSTDNQEDEEDSLEMESPMESPASDFK
RSSPEGKSVFGDSPEPYFCPTESSSTLYFSADPQSPSSASTTRLDRDTGGLEPGVRLSSI
PSDPALHRDVRGLIGRVGPRCTSTPKLDSGVLHSPPTVPHLTGPRRQLIMSRRDEEDNF

>Oryzias latipes_XKR5a(ENSORLP_16231)
LCESVIYCIGFYLWNEETQWAGLTLGLFLPGTAVQLLSVKWYYDDGDDRRCYLSVIHILH
LGIFKRLWDCMKSVLRRQGSVSELGVVVMQQADVAALWVLEALVLTLPQSLLQAYVVVST
DVGITSPVAYCCGLCVLSVSWALVLYSRACCLIRPGHLAMPPAALLCQLVWRAGMLGARV
TCLMFFARVFHWWVCGVAGFHWLTASFWLVSQQPDICTGPWCWRAFNGVLGLVHVFLFLN
VKDGPSRYRMASFYAFMLVENATLLLTASDFLSEASWDNMTLPTSVLCCFLLGATSLVLY
YRFLHPKSTEISQGTSRHHMGSTCIEQGESSFSLGDKSLPPPSSQTHGSFSLSGVAGSLL
EHPGNCEGRSNSSCPCYHHHWLLIRLALKTGDLGKINRAYGAGGAAAILDMDDNNQEFKS
NEGITITAGGSCEAVSTSESQGKGLAPLSDCKDEFQSISEPTSTEHPDEEEDSLEMESPM
ESPVSDFKRSSPEGKSVFGDSPEPYFCPTESSSTLYFSADPQSPSSASNPRLDRETGSLE
LGGRLNSIPSDPALHRDVRGLMGRVGQRCISTPKLDLGVLDSASSIPHLTGPRRQLIMSR
RDEDDHF

>Oryzias latipes_XKR5b(ENSORLP_13915)
CMSPVIYCFVYYLWVGHNYCYAHLAGFTALFLLPGWGPQWLSYQWYLSDGRIARKSLRWT
HILHLGIFKRLWECMHLPDEDLYVEIMQQADASALRLFEALVVTLPETLFQTYVLICTDI
GIKSPTSVCFVVCLLSLAWALVLYARACSLIRPGHLHMTPAAILCRLLWRVAMLGSRFAV
LMLFTRVFRQWILGVLGVHWLGATFWMVSQQTDIIRSTSRWRIFNLVLGAIYVFLFLNVK
YGQSRYRMAAFYLVMFVENALLLLASSWLFTMVSWDTVGIPAAVFCSFLIGLIALVLYYR
FLHPKSFEIFQSIRHRGIGGACTDRGSALSLEEKVSPTLHRHATLSVFCSGGGTLMDLPI
QWEGWKHHHWLLIRLALKTGDVVRIWTAYGEGGLAGLMGLPEYSTQTYKTFRETSNRSLT
ASKDSSFQHHSPTQKKRSSNSYPGFKRIPRDYPRPGEKVSLDIKTPGWSPERRSPLLAGS
PEKKSGIPGESSTTLYFSADPQSPSTGS

>Homo sapiens_XKR6(ENSP_416707)
MAAKSDGGGVGVGFAQLHNLDEAVGSGGEEDGEPGGGGCGGGGDGSEPGESSSMHICHCC
NTSSCYWGCRSACLRSLLGRKPRRSAAADGGDQPLQPPAAPGAGRQPPTPSAARPEPPPP
QVERPWLDCLWIVLALLVFFGDVGTDLWLALDYYRKGDYVYFGLTLFFVLVPSLLVQSLS
FRWFVQDYTGGGLGAVEGLTSRGPPMMGAGYVHGAARGGPGVRVSPTPGAQRLCRLSVWI
WQSVIHLLQMGQVWRYIRTMYLGIQSQRRKEHQRRFYWAMMYEYADVNMLRLLETFLESA
PQLVLQLYIMLQKNSAETLPCVSSVTSLMSLAWVLASYHKLLRDSRDDKKSMSYRGAIIQ
VFWRLFTISSRVISFALFASIFQLYFGIFVVVHWCAMAFWIIHGGTDFCMSKWEEILFNM
VVGIVYIFCWFNVKEGRTRYRMFAYYTIVLTENAALTFLWYFYRDPETTDSYAVPALCCV
FISFVAGIAMMLLYYGVLHPTGPRAKILASSCCAELLWGIPLPPDVEPMAPEIPGYRGTQ
VTPTRAVTEQQEDLTADTCLPVFQVRPMGPPTPLGRPYLPEGPLIKIDMPRKRYPAWDAH
FVDRRLRRTINILQYVTPTAVGIRYRDGPLLYELLQYESSL

>Gorilla gorilla_XKR6(ENSGGOP_9164)
LLLPWRHSSREFQYIRTMYLGIQSQRRKEHQRRFYWAMMYEYADVNMLRLLETFLESAPQ
LVLQLYIMLQKNSAETLPCAGVSSVTSLMSLAWVLASYHKLLRDSRDDKKSMSYRGAIIQ
VFWRLFTISSRVISFALFASIFQLYFGIFVVVHWCAMAFWIIHGGTDFCMSKWEEILFNM
VVGIVYIFCWFNVKEGRTRYRMFAYYTIVLTENAALTFLWYFYRDPETTDSYAVPALCCV
FISFVAGIALMLLYYGVLHPTGPRAKILASSCCAELLWGIPLPPDVEPMAPETPGYRGTQ
VTPTRAVTEQQEDLTADTCLPVFQVRPMGPPTPLGRPYLPEGPLIKIDMPRKRYPAWDAH
FVDRRLRRTINILQYVTPTAVGIRYRDGPLLYELLQYESSL

>Mus musculus_XKR6(ENSMUSP_113708)
MAAKSDGGGVGVGFAQLHNLDEAVGSGEEDGEPGGGGCGGGDGSEPGESSSLHICHCCNT
SSCYWGCRSACLRSLLGKKPRRSAAAADGGDQPLQPPGAAGRHPPTPSAGRPQPASPQVE
RPWLDCLWIVLALLVFFGDVGTDLWLALDYYRKGDYGCFGLTLFFVLVPSLLVQSLSFRW
FVQDYTGGGLGAVEGLSSRGPPMMGAGYGHGAARGGPGAGGSATPGAQRLCRLSVWIWQS
VIHLLQMGQVWRYIRTMYLGIQSQRQKEHQRRFYWAMMYEYADVNMLRLLETFLESAPQL
VLQLCIMIQKNSAETLPCVSSVTSLMSLAWVLASYHKLLRDSRDDKKSMSYRGALIHLFW
RLFTISSRVISFALFASIFQLYFGIFVVVHWCAMAFWIIHGGTDFCMSKWEEILFNMVVG
IVYIFCWFNVKEGRTRYRMFAYYTIVLTENAALTFLWYFYRNPESTDSYAVPALCCVFVS
FVAGITLMLLYYGVLHPMGPRAKVFASSCCAELLWGIPLPPDVEPMAPQTPGYRGTQVTP
TRAVTEQQEDLTADTCLPVFQVRPMGPSTPSGRPYHPEGPLIKIDMPRKRYPAWDAHFVD

RRLRRTINILQYVTPTAVGIRYRDGPLLYELLQYESSL

>Canis familiaris_XKR6(ENSCAFP_11914)
RYIRTMYLGIQSQRQKEHQRRFYWAMMYEYADVNMLRLLETFLESAPQLVLQLCIMIQKN
SAETLPCVSSVTSLMSLAWVLASYHKLLRDSRDDKKSMSYRGALIHLFWRLFTISSRVIS
FALFASIFQLYFGIFVVVHWCAMAFWIIHGGTDFCMSKWEEILFNMVVGIVYIFCWFNVK
EGRTRYRMFAYYTIVLTENAALTFLWYFYRNPETTDSYAVPALCCVFISFVAGIALMLLY
YGVLHPMGPRAKIFASSCCAELLWGIPLPPDVEPMAPQTPGYRGTQVTPTRAVTEQQEDL
TADTCLPVFQVRPMVPSTPSGRPYPPEGPLIKIDMPRKRYPAWDAHFVDRRLRRTINILQ
YVTPTAVGIRYRDGPLLYELLQYESSL

>Myotis lucifugus_XKR6(ENSMLUP_22004)
LGRYIRTMYLGIQSQRQKEHQRRFYWAMMYEYADVNMLRLLETFLESAPQLVLQLCIMIQ
ENSAETLPCVSSVTSLMSLAWVLASYHKLLRDSRDDKKSMSYRGALIHLFWRLFTISSRV
ISFALFASIFQLYFGIFVVVHWCAMAFWIIHGGTDFCMSKWEEILFNMVVGIVYIFCWFN
VKEGRTRYRMFAYYTIVLTENAALTFLWYFYRNPETTDFYAVPALCCVFISFVAGIALML
LYYGVLHPMGPRAKIFASSCCAELLWGIPLPPDVEPMAPQTPGYRGTQVTPTRAVTEQQE
DLTADTCLPVFQVRPMGPSTPSGRPYHPEGPLIKIDMPRKRYPAWDAHFVDRRLRRTINI
LQYVTPTAVGIRYRDGPLLYELLQYESSL

>Monodelphis domestica_XKR6(ENSMODP_11108)
MYLGIQSQRQKEHQRRFYWAMMYEYADVNMLRLLETFLESAPQLVLQLCIMIQKNSAETL
PCVSSVTSLMSLAWVLASYHKLLRDSRDDKKSMSYRGALIHLFWRLFTISSRVISFALFA
SIFQLYFGIFVVVHWCAMAFWIIHGGTDFCMSKWEEILFNMVVGIVYIFCWFNVKEGRTR
YRMFAYYTVVLTENAALTFLWYFYRNPETTDSYAVPALCCVFLSFVAGIALMLLYYGVLH
PMGPRAKIFASSCCAELLWGIPLPPDVEPMAPQTPGYRGTQVTPTRAVTEQQEDLTADTC
LPVFQVRPMGPSTPSGRPYHPEGPLIKIDMPRKRYPAWDAHFVDRRLRRTINILQYVTPT
AVGIRYRDGPLLYELLQYESSL

>Gallus gallus_XKR6(ENSGALP_26821)
RVSRFAFPPVSCRYIRTMYLGIQSQRRKEHQRRFYWAMMYEYADVNMLRLLETFLESAPQ
LVLQLCIMIQKNRAETLPCVSSVASLMSLAWVLASYHKLLRDSRDDKKSMSYRGALIHLF
WRLFTISSRVISFALFASIFQLYFGIFVVVHWCAMAFWIIHGGTDFCMSKWEEILFNMVV
GIVYIFCWFNVKEGRTRYRMFAYYTVVLTENAALTLLWYFYRDPDTTDSYAVPALCCVFL
SFAAGIALMLLYYGVLHPMGPRAKIFASSCCAELLWGIPLPPDVEPMAPQTPGYRGAQAT
PTRAVTEQQEELTADTCLPVFQVRAMVPSTPSGRPYPPEGPLIKIDIPRKRYPAWDAHFV
DRRLRRTINILQYVTPTAVGIRYRDGPLLYELLQYESSL

>Anolis carolinensis_XKR6(ENSACAP_7485)
RYIRTMYLGIQSQRQKEHQRRFYWAMMYEYADVNMLRLLETFLESAPQLVLQLCIMIQKN
RAETLPCLSSMASLMSLAWVLASYHKLLRDSRDDKKSMSYRGALIHLFWRLFTISSRVIS
FALFASIFQLYFGIFVVVHWCAMAFWIIHGGTDFCMSKWEEILFNMVVGIVYIFCWFNVK
EGRTRYRMFAYYTVVLTENAALTLLWYWYRDPDTTDSYAVPALCCVFLSFAAGIALMLLY
YGVLHPMGPRAKIFSSSCCAELLWGIPLPPDIEPMAPQTPGYRAAQATPTRAATEQQEEP
TADTCLPVFQVRAMVPSTPSGRPYHPEGPLIKIDIPRKRYPAWDAHFVDRRLRRTINILQ
YVTPTAVGIRYRDGPLLYELLQYESSL

>Xenopus tropicalis_XKR6(ENSXETP_1509)
MAAKSDGMLGVGSGFAQLHNLDEAVAAAAAGERGGGGEDGEPGESSSIHICHCCNTSSCY
WGCRSACLRSLLGKGKGAGMLGGQAGGKGDRAWLDCLWIVLALMVFFWDVGTDLWLAADY
YSKRDYLCSSLTLGFVLVPSLLVQILSFRWFAQDYTGGGLGAVEGLSSRGPPMMGSIHHH
YQRHHPSYTAERFCRISVWLWQSVIHILQMGQVWRYLRTMYLGIQSQRQKEHQRRFYWAM
MYEYADVNMLRLLETFLESAPQLVLQLCIMIQKNRAETLPCVSSVASLMSLAWVLASYHK
LLRDSRDDKKSMSYRGALIHIFWRLFTISSRVISFALFASIFQLYFGIFVVVHWCIMAFW
IIHGGTDFCMSKWEEILFNMVVGIVYIFCWFNVKEGRTRYRMFAYYTVILTENAVLTFLW
YFYRDPTTDSFAVPALCCVFLCFAAGVAFILLYYGVLHPTGPRAKIFASSCCAELLWGIP
LPPDVEPMAPQTPGYRGAEATPTRATTEQQDDLTADTCLPVFQVRPMVPTTPSGRPYYPE
GPLIKIDMPRKRYPAWDAHFVDRRLRRTINILQYITPSAVGIRYRDGPLLYELLQYESSL

>Danio rerio_XKR6a(ENSDARP_63724)
MAAKSDGRGVATGFAQLHNLDEAVGSCEDDPRQGSSFHICHCCNTSSCYWGCRSACLHYL

LDKGRDVRRPPHEERLWLDCLWIVLALLVFFWDVGTDLWLALDYYAKQDYLWFGLTLFFV
LVPSVLVQILSFRWFVQDYTGGGLGSVEGLSSTRRSTSAVVYNRARCCRVSVWVWQTLIH
ILQMGQVWRYIRTMYLGIQSRRQKENQRRFYWAMMYEYADVNMLRLLETFLESAPQLVLQ
LCIMIQKNRAETLQCVSAVTSLLSLSWVLASYHKLLRDSRDDQKSMSYRGALVHLLWRFF
TISSRALSFALFASVFHLYFGIFVVLHWCAMAFWVIHGGTDFCMSKWEEVLFNMVVGVVY
VFCWFNVKEGRTRYRMVFYYSLVLVENTILTALWYAYRDPVSTDSYAASALCGVFLCFAS
GVACMVLYYGVLHPMGPRLRVLASSCCAELLWGLPLPPEAEPMAPTPGPRGSQATPTRGM
ASGDYSEPEEAATDTCLPVFQVRSTEPTDASGRPIPPKGPLIKIDMPRKRYPAWDAHFVD
RRLRRTINILQFISPNAVGIRYRDGPLLYELLQYESSL

>Danio rerio_XKR6b(ENSDARP_22407)
MAAKSDGVGVVTGFAQLHNLDEATGDEEPLDSAAIHICHCCNSSSCYWGCRSACLQPLLG
ERDRPQHQQQHQPEERLWLDCLWIIVALLVFFWDVGTDLLLALDYYSKRDYLWFGLTLFF
VLVPSVLVQILSFRWFVQDYTGGGLGAVEGLSRKATADLSSRIYGRDRCCVISIWIWHAC
VHVLQMGQVWRYIRTMYLGIQSRRQKEHRRRFYWAMMYEYADVNMLRLLETFLESAPQLV
LQLCIMIQKNRAETLQCVSSVASLLSLAWVLASYHKLLRDSRDDKKSMSYRGALIHIFWR
LFTISSRVLSFALFASIFHIYFGIFVVVHWCAMAFWIVHGGTDFCMSKWEEVLFNMVVGI
VYIFCWFNVKEGRTRYRMVIYYFVVLAENTVLTCLWYAYRDPITTDSYAVPVLCGVYLTF
ASGVVFMGVYYGFLHPMGPKIRVLASSCCAELLWGLPLPPEAEPMAPTPGPRASQPTPSR
ASMVDDTTMDTCLPVFQVRSPEPTTPLGRYRPEGPLIKIDMPRKRYPAWDAHFVDRRLRR
TINILQFITPATVGIRYRDGPLLYELLQYESS

>Takifugu rubripes_XKR6(ENSTRUP_13182)
MAAKSDGRGVVTGFAQLHNLDEVVGTGEDDTRNGSSFHICHCCNTSSCYWGCRSACLHYL
RTKGKEKGKDTARPTLEERLWLDCLWIILALLVFFWDVGTDLWLAVDYYHKQDFLWSGLT
LFFVLVPSVLVQILSFRWFVQDYTGGGLGSVEGLSSRRAAACLQRDKCCRLSVWIWQTVI
HIFQLGQVWRYIRTMYLGIQSHRQKENQRRFYWAMMYEYADVNMLRLLETFLESAPQLVL
QLCIMIQSNKAEWLQCVSALSSLLSLAWVLASYHKLLRDSRDDKKSMSYRGALVHLFWRL
FTISSRVLSLALFASVFHIYFGIFVVLHWCAMAFWVIHGGTDFCMSKWEEVLFNMVVGVV
YVFCWFNVREGRTRYRMVAYYIVVLLENVILSSLWYAYRDPATTDAYASFALCGVFLCFA
SGVACMVLYYGVLHPMGPRLRVLASSCCAELLWGLPLPPEAEPMAPTPGPRGSQATPTRG
LTGEYAESDETATDTCLPVFQVRSTEPVDAAGRPIQPEGPFIKIDMPRKRYPAWDAHFVD
RRLRRTINVLQYISPNAAGIRYRDGPLLYELLQYESSF

>Tetraodon nigroviridis_XKR6a(ENSTNIP_17283)
MAAQSDGGKAAVGGGGFAQLYDVDAEEPMDSAAIHICQCCRTSACYWGCRSACLGSLLGG
GQPAGGVGIRETHCPPREQLWLDCLWIILALLVFFWDVGTDLCLAVDYYKRQNYLWFGLT
LFFVLVPSVLVQILSFRWFVQDYTGGGLGEVEGLTKRGAVALGCLYPGRDRLQLATIWMW
QATIHILQLGQVWRYIRTLYLGVMSRRQKEHQRRWYWAMMFEYADVNMLRLLETFLESAP
QLVLQLSIMIQENRAETLQCISSLGSLLSLAWVLASYHKLLRDSRDDQRSMSYRGALLHI
FWRLFTISSRVLSLALFASLFHIYFGIFVVVHWCAMAFWVVHGGTDFCMSKWEEVLFNMV
VGIVYIFCWFNVKEGRTRHRMVTYYMVVLAENSILTGLWYAYRDPVLTDSYAVPALCGVY
LTFAGGVLVMLLYYGFLHPATAHLQPSPASSCCAQLLWGLPLPPSAPPTAPPTPAHMTKS
QTEEDVAETCLPVFQVRSAPPISKPEGPLIKIDMPRKRYPAWDHYVDRRLRRTINILQYI
TPAAVGIRYRDGLLYELLQYESSL

>Tetraodon nigroviridis_XKR6b(ENSTNIP_19426)
MAAKSDGRGVVTGFAQLHNLDEVVGTGEDDARNGSSFHICHCCNTSSCYWGCRSACLHYL
RAKGKGEGKETARPTPEERLWLDCLWIVLALLVFFWDVGTDLWLAVDYYHKQDFLWSGLT
LFFVLVPSVLVQILSFRWFVQDYTGGGLGSVEGLSSRRAAASLQRDKCCRLSVWIWQTVI
HIFQLGQVWRYIRTMYLGIQSHRQKENQRRFYWAMMYEYADVNMLRLLETFLESAPQLVL
QLCIMIQSNKAEWLQCVSALSSLLSLAWVLASYHKLLRDSRDDKKSMSYRGALVHLFWRL
FTISSRVLSLALFASVFHIYFGIFVVLHWCAMAFWVIHGGTDFCMSKWEEVLFNMVVGVV
YVFCWFNVREGRTRYRMVAYYVVVLLENVILTSLWYAYRDPATTDAYASLALC

>Gasterosteus aculeatus_XKR6a(ENSGACP_14390)
MAAKSDGRGVVTGFAQLHNLDEVVGAGEDDTRNSSSFHICHCCNTSSCYWGCRSACLHYL
RGKGKGKGGDAARPPQEERLWLDCLWIILALLVFFWDVGTDLWLAVDYYHKQDFLWSGLT
LFFVLVPSVLVQILSFRWFVQDYTGGGLGSVEGLSRRATATLQRDSCCRLSVWVWQTVIH
IFQLGQVWRYIRTMYLGIQSHRQKENQRRFYWAMMYEYADVNMLRLLETFLESAPQLVLQ
LCIMIQSNKAEWLQCVSAISSLLSLAWVLASYHKLLRDSRDDKKSMSYRGALVHLFWRLF

TISSRVLSLALFASVFHIYFGIFVVLHWCAMAFWVIHGGTDFCMSKWEEVLFNMVVAVVY
VFCWFNVRAGRTRCRMVAYYALVLLENAILSLLWYAYRDPVTTDAYASFALCGVFLCFAS
GVACMVLYYGVLHPMGPRLRVLASSCCAELLWGLPLPPEAEPMAPTPGHRGSQATPTRGL
AGEYEESDETPTDTCLPVFQVRSTEPVDAAGRPIQPEGPLIKIDMPRKRYPAWDAHFVDR
RLRRTINVLQYISPNAVGIRYRDGPLLYELLQYESSL

>Gasterosteus aculeatus_XKR6b(ENSGACP_19719)
MAAQSDGGKAPGGVGAAAPLPATGAAAQPASAPCSAGGQPLGGVGIRETHCPPREQLWLD
CLWIILALIVFFWDVGTDLCLANEYYRRQDYLWFGLTLFFVLVPSVLVQILSFRWFVQDY
TGGGLGEVEGLTKRGAVALGCLYPGRDRLQLATIWLWQATIHILQLGQVWRYIRTLYLGI
MSRRQKEHQRRWYWAMMFEYADVNMLRLLETFLESAPQLVLQLCIMIQENRAETLQCISS
LGSLLSLAWVLASYHKLLRDSRDDQRSMSYRGALLHLFWRLFTISSRVLSLALFASLFHI
YFGIFVVVHWCAMAFWVVHGGTDFCMSKWEEVLFNMVVGIVYIFCWFNVKEGRTRHRMVV
YYIVVLAENTILTGLWYTYRDPALTDSYAVPALCGVYLTFAGGVLVMLLYYGFLHPASAH
LQPSPASSCCAQLLWGLPLPPSAPPTAPPTPAHMSKSQTDEDVAETCLPVFQVRSAPITS
KPEGPLIKIDMPRKRYPAWDAHYVDRRLRRTINILQYITPAAVGIRYRDGPLLYELLQYE
SSF

>Oryzias latipes_XKR6a(ENSORLP_20541)
MAAKSDGRGVVTGFAQLHNLDEVVGTGEDDARNGSSFHICHCCNTSSCYWGCRSACLHYL
RRKGKERDAARPPQEERLWLDCLWIVLALLVFFWDVGTDLWLAVDYYHNQNFLWSSLTLF
FVLVPSVLVQILSFRWFVQDYTGGGLGSVEGLSSSRAVATFQRDRCCRLSVWVWQTIVHI
FQMGQVWRYIRTMYLGIQSHRQKENQRRFYWAMMYEYADVNMLRLLETFLESAPQLVLQL
CIMIQSNKVERLQCLSALSSLLSLAWVLASYHKLLRDSRDDKKSMSYRGALLHLFWRLFT
ISSRVLSFALFASVFHIYFGIFVVLHWCAMAFWVIHGGTDFCMSKWEEVLFNMVVGVVYV
FCWFNVREGRTRYRMVTYYTVVLLENALLTSLWYAYRDPATTDAYASFALCGVFLCFASG
VACMVIYYGVLHPMGPRLRVLASSCCAELLWGLPLPPEAEPMAPTPGPRGSQATPTRGLV
GEHMESDETATDTCLPVFQVRSTEPVDAAGRPIHPEGPLIKIDMPRKRYPAWDAHFVDRR
LRRTINVLQYISPNAVGIRYRDGPLLYELLQYESSL

>Oryzias latipes_XKR6b(ENSORLP_5516)
MAAQSDGGKAAGCGGFAQLYDVDAEEPLDSAAIHICQCCRTSACYWGCRSACLGSLLGGG
QPVGGIGVRETHCPPREQLWLDCLWIILALLVFFWDIGTDLCLAVDYYQRQDYLWFGLTL
FFVLVPSVLVQILSFRWFVQDYTGGGLGDVEGLTKRGTVTLGCLYPGKDRLQLASIWLWQ
ATIHILQLGQVWRYIRTLYLGLMSRRQKEHQRRWYWAMMFEYADVNMLRLLEAFLESAPQ
LVLQLCIMIQENRAETLQCISSLASLLSLAWVLASYHKLLRDSRDDQRSMSYRGALLHLF
WRLLTISSRVLSLALFASLFHIYFGIFVVLHWCAMAFWVVHGGTDFCMSKWEEVLFNMVV
GIVYIFCWFNVKEGRTRYRMVVYYIIVLAENTILTGLWYAYRDPVLTDSYAVPALCGVYF
TFIGGVLIMLLYYGFLHPATAHLQPSPASSCCAQLLWGLPLPPSAPPTAPPTPAHMVKSQ
TDEDVAETCLPVFQVRSTPTTSKPEGPLIKIDMPRKRYPAWDAHYVDRRLRRTINILQYI
TPGAVGIRYRDGPLLYELLQYESSL

>Homo sapiens_XKR7(ENSP_477059)
MAAKSDGAAASASPDPEGAAGGARGSAGGRGEAAAAAGPPGVVGAGGPGPRYELRDCCWV
LCALLVFFSDGATDLWLAASYYLQNQHTYFSLTLLFVLLPSLVVQLLSFRWFVYDYSEPA
GSPGPAVSTKDSVAGGAAISTKDSAGAFRTKEGSPEPGPQPAPSSASAYRRRCCRLCIWL
LQTLVHLLQLGQVWRYLRALYLGLQSRWRGERLRRHFYWQMLFESADVSMLRLLETFLRS
APQLVLQLSLLVHRGGAPDLLPALSTSASLVSLAWTLASYQKVLRDSRDDKRPLSYKGAV
AQVLWHLFSIAARGLAFALFASVYKLYFGIFIVAHWCVMTFWVIQGETDFCMSKWEEIIY
NMVVGIIYIFCWFNVKEGRSRRRMTLYHCIVLLENAALTGFWYSSRNFSTDFYSLIMVCV
VASSFALGIFFMCVYYCLLHPNGPMLGPQAPGCIFRKASEPCGPPADAITSPPRSLPRTT
GAERDGASAGERAGTPTPPVFQVRPGLPPTPVARTLRTEGPVIRIDLPRKKYPAWDAHFI
DRRLRKTILALEYSSPATPRLQYRSVGTSQELLEYETTV

>Gorilla gorilla_XKR7(ENSGGOP_5134)
WLAASYYLQNQHTYFSLTLLFVLLPSLVVQLLSFRWFVYDYSEPPGSPGPAVSTKDSVAG
GAAISTKDSAGAFRTKEGSPEPGPQPAPSSASAYRRRCCRLCIWLLQTLVHLLQLGQVWR
YLRALYLGLQSRWRGERLRRHFYWQMLFESADVSMLRLLETFLRSAPQLVLQLSLLVHRG
GAPDLLPALSTSASLVSLAWTLASYQKVLRDSRDDKRPLSYKGAVAQVLWHLFSIAARGL
AFALFASVYKLYFGIFIVAHWCVMTFWVIQGETDFCMSKWEEIIYNMVVGIIYIFCWFNV
KEGRSRRRMTLYHCIVLLENAALTGFWYSSRNFSTDFYSLIMVCVVASSFALGIFFMCVY

YCLLHPNGPMLGPQAPGCIFRKASEPCGPPADAITSPPRSLPRTTGAERDGASAGERAGT
PTPPVFQVRPGLPPTPVARTLRTEGPVIRIDLPRKKYPAWDAHFIDRRLRKTILALEYSS
PATPRLQYRSVGTPQELLEYETTV

>Pongo abelii_XKR7(ENSPPYP_12174)
MAAKSDGAAVVLLPSLVVQLLSFRWFVYDNSEPAGSPGPAVSTKDSVAGGAAISTKDSAG
AFRTKEGSPEPGPQPAPSSASAYRRRCCRLCIWLLQTLVHLLQLGQVWRYLRALYLGLQS
RWRGERLRRHFYWRMLFESADVSMLRLLETFLRSAPQLVLQLSLLVQRGGAPDLLPALST
SASLVSLAWTLASYQKVLRDSRDDKRPLSYKGAVAQVLWHLFSIAARGLAFALFASVYKL
YFGIFIVAHWCVMTFWVIQGETDFCMSKWEEIIYNMVVGIIYIFCWFNVKEGRSRRRMTL
YHCIVLLENAALTGFWYSSRNFSTDFYSLIMVCVVASSFALGIFFMCVYYCLLHPNGPML
GPQAPGCIFRKASEPCGPPADAITSPPRSLPRTTGAERDGASAGERAGTPTPPVFQVRPG
LPPTPVARTLRTEGPVIRIDLPRKKYPAWDAHFIDRRLRKTILALEYSSPATPRLQYRSV
GTSQELLEYETTV

>Mus musculus_XKR7(ENSMUSP_49346)
MAAKSDGAAAVAGPGPEGPAGADRGGAGGRGEAAAGIAGPGPVEAGCPGPRYELRDCCWV
LCALLVFFSDGATDLWLAASYYLQGQSTYFGLTLLFVLLPSLVVQLLSFRWFVYDYSEPA
GTPGPAVSTKDSDIVGAAISTKDSAVAFRTKEGSAELVPRPAPSSAGTYRRRCCRLCVWL
LQTLVHLLQLGQVWRYLRALYLGLQSRWRGERLRRHFYWQMLFESADVSMLRLLETFLRS
APQLVLQLSLLVHRGREPELLTALSISASLVSLAWTLASYQKVLRDSRDDKRPLSYKGAV
VQVLWHLFTIAARTLAFALFASVYKLYFGIFIVAHWCIMTFWVIQGETDFCMSKWEEIIY
NMVVGIIYIFCWFNVKEGRSRRRVTLYYCIVLLENAALTGFWYSSRNFSTDFYSLILVCV
VASSFALGIFFMCVYYCLLHPNGPMLGPQAPGCIFPEAPGPCGPPADAITSPPRSLPRTT
GTERDGVAVGGERAGTPTLPVFQVRPGLPPTPVARPLRTEGPVIRIDLPRKKYPAWDAHF
IDRRLRKTILALEYSSPATPRLQYRSMGTSQELLEYETTV

>Rattus norvegicus_XKR7(ENSRNOP_12156)
MAAKSDGAAAVAGPGPEGPAGADRGGAGGRGEVAAGIAGPGPVEAGCPGPRYELRDCCWV
LCALLVFFSDGATDLWLAASYYLQGQSTYFGLTLLFVLLPSLVVQLLSFRWFVYDYSEPT
GTPGPAVSTKDSDIVGAAISTKDSAVAFRTKEGSPELVPRPAPSSAGAYRRRCCRLCVWL
LQTLVHLLQLGQVWRYLRALYLGLQSRWRGERLRRHFYWRMLFESADVSMLRLLETFLRS
APQLVLQLSLLVHRGREPELLTALSISASLVSLAWTLASYQKVLRDSRDDKRPLSYKGAV
VQVLWHLFTIAARTLAFALFASVYRLYFGIFIVAHWCIMTFWVIQGETDFCMSKWEEIIY
NMVVGIIYIFCWFNVKEGRSRRRVTLYYCIVLLENAALTGFWYSSRNFSTDFYSLILVCV
VASSFALGIFFMCVYYCLLHPNGPMLGPQAPGCIFPEAPGPCGPPADAITSPPRSLPRTT
GAERDGAAVGGERAGTPTPPVFQVRPGLPPTPVARPLRTEGPVIRIDLPRKKYPAWDAHF
IDRRLRKTILALEYSSPATPRLQYRSMGTSQELLEYETTV

>Canis familiaris_XKR7(ENSCAFP_10553)
MAAKSDGAAAAAGPGPEGAAGGARSEAGGRGEAAAAAAGGPGVTGAGGPGPRYELRDCCW
VLCALLVFFSDGATDLWLAASYYLQGQRTYFGLTLLFVLLPSLVVQLLSFRWFVYDYSEP
AGAPGPAVSTKDSGAGGPSISTKDSAAAFRTKEGGPQLGPGPAPSSASAYRRRCCRLCVW
LLQTLVHLLQLGQVWRYLRAMYLGLQSRWRGERLRRHFYWRMLFESADVSMLRLLETFLR
SAPQLVLQLSLLVHRGGQPDLLPALSTSASLVSLAWTLASYQKVLRDSRDDKRPLSYKGA
VAQVLWHLFTIAARSLAFALFASVYKLYFGIFIVAHWCVMTFWVIQGETDFCMSKWEEII
YNMVVGIIYIFCWFNVKEGRSRRRMTLYYCIVLLENAALTGFWYSSRNFSTDFHSLILVC
VVASSFALGIFFMCVYYCLLHPNGPMLGPQAAGCICPEAPGPCGPPADAVTSPPRSLPRT
TGAEREGPSVGGERAGTPTPPVFQVRPGLPPTPVARPLRTEGPVIRIDLPRKKYPAWDAH
FIDRRLRKTILALEYSSPATPRLQYRSVGTPQELLEYETTV

>Felis catus_XKR7(ENSFCAP_17682)
MAAKSDGAAAAAGPGPEGAAGGARGGAGGRGEXGQRTYFGLTLLFVLLPSLVVQLLSFRW
FVYDYSEPAGAPGPAVSTKDSGTGGPSISTKDSAAAFRTKEGSPQLGPGPAPSSASAYRR
RCCRLCVWLLQTLVHLLQLGQVWSRYKRANYFHLNPRWGSEEEVRSFHFTTIPSSASISE
FRLIKQTSNTKTNKTLQLCMLMSAHSGSPDDSLGTLSTSASLVSLAWTLASYQKVLRDSR
DDKRPLSYKGAVAQVLWHLFTIAARSLAFALFASVYKLYFGIFIVAHWCVMTFWVIQGET
DFCMSKWEEIIYNMVVGIIYIFCWFNVKEGRSRRRMTLYYCIVLLENAALTGFWYSSRNF
STDFHSLILVCVVASSFALGIFFMCVYYCLLHPNGPMLGPQMPGCICPEAPGPCGPPADA
VTSPPRSLPRTTGTERDGASAGGERAGTPTPPVFQVRPGLPPTPVPRTLRTEGPVIRIDL
PRKKYPAWDAHFIDRRLRKTILALEYSSPATPRLQYRSVGTSQEVLEYETSV

>Myotis lucifugus_XKR7(ENSMLUP_19149)
GAAERWGGERLRRHFYWRILFESADVSMLRLLETFLRSASPQLLIVLSWFLHALTVPPSQ
PLSTSASLVSLAWTLASYQKVLRDSRDDKRPLSYKGAVAQVLWHLFTIAARSLAFALFAS
VYKLYFGIFIVAHWCIMTFWVIQGETDFCMSKWEEIIYNMVVGIIYIFCWFNVKEGRSRR
RMTLYYCIILLENAALTGFWYSSRNFSTDFHSLILVCVVASSFALGIFFMCVYYCLLHPN
GPMLGPQAPGCICPEAPEPCGPPADAVTSPPRSLPRTTGAERDGVSVGGERAGTPTPPVF
QVRPGLPPTPVARTSRTEGPVIRIDLPRKKYPAWDAHFIDRRLRKTILALEYSSPVTPRL
QYRSTRTSQELLEYETTV

>Monodelphis domestica_XKR7(ENSMODP_24298)
GGRRPRYSLWDCCWALCALLVFFSDGATDLWLAADYYLQGRRIWFGLTLLFALLPSLVVQ
LLSFRWFVYDYSSAGAGPGAATTAFSTKDSTSASVSASAAFSTKDSGPGPGPAVSAPPAL
SGAPRPHRRCCRLCVWLLQSLVHVLQLGQVWRYLRAMYLGLQSRWHGEQLRRHFYWRMMF
ESADISMLRLLEAFLKSAPQLVLQLSIMVLQGQMEALQGLSASASLVSLAWIIASYQKVL
RDSRDDKRAMSYKGAIVQILWHLFTIAARALAFALFASVFRLYFGIFIVAHWCVMTFWVI
QGETDFCMSKWEEIIYNMVVGIIYIFCCFNVKKRSRCRMATYYCIVLVENVALTGFWYYN
HNFPTDFSALIMVCVVAASYALGIFFMLVYYCLLHPNGPMFGPQAPGGIFPEAVGPGPSG
PPADAVTSPPRSLPRTTGGERDGIPGDRDSGAPAVFQVRPSLPATPVARTPRTEGPVIRI
DLPRKKYPAWDAHFIDRRLRKTILALEYSSPATPRLQYRSMGASRELLEYETTV

>Gallus gallus_XKR7(ENSGALP_428)
GAPASGDAGGGARAAGSGIQPAAGLPGVRAGLRDGCWVLCALLVCFADGASDLWLAADYY
LEGQRWWFGLTLLFVLLPSLVVQLLSFRWFVYDFAVGTKDSAGSTKDSARGRRGCCRLCV
WLLQGCIHLLQLGQVWRYLRTLYLGLQSRWQAEHRRRHFYWRMMFENADISMLRLLETFL
KSAPQLVLQLSIMVQQNNIKALQGFSASASLVSLAWMIASYQKVLRDSREDKMPMSYKGA
VVQILWHLFTIAARAIAFALFASVFQLYFGIFIVTHWCIMTFWIIQGETDFCMSKWEEII
YNMVVGIIYIFCWFNVKEGRSRYRMCIYYIITLSENAALTILWFHYYNRKTMSDFNALIL
VCVVSSSFALGIFFMFIYYCLLHPNGPMFGPSSGGCIFRQRPPPAPSSPADAVTSPPRSL
PRTTGGEREGAPGERDSCVPVFQVRPCAPTPPAARAPRTEGPVIRIDLPRKKYPAWDAHF
IDRRLRKTILALEYASPSTPRLQYRTAGVSQELLEYETTV

>Anolis carolinensis_XKR7(ENSACAP_16774)
IFHLPVLCRYLRTMYLGLQSRWQSENRRRHFYWRMMFESADISMLRLLETFLKSAPQLVL
QLSIMVHQNYIEPLQGLSASASLVSLAWTLASYQKVLRDSREDKLPMSYKGAMVQILWHL
FTIAARAIAFALFASVFQLYFGIFIVTHWCIMTFWIIQGETDFCMSKWEEIIYNMVVGII
YIFCWFNVKEGRSRCRMSIYYIITLSENAAMTGLWYWHRSESISESNALIIVCVVTSSFA
LGVFFMFIYYCLLHPNGTIFATQSTGCMCREEATAVPGGAGDVVTSPPRSLPRTTGAERE
GVPGDRDSCVPVFQVRPSVPPTPVPRAPRTEGPVIRIDLPRKKYPAWDAHFIDRRLRKTI
LALEYSSPTTPRLQYRSVGTSQELLEYETTV

>Danio rerio_XKR7a(ENSDARP_53660)
MAAKSDGTAVSLQNDIPPTCLPGLEKRSPEWRPVGKEYSLLDCCWTLCALLVFFSDGASD
LWLAADYYLRRDYWWFALTLVFIIIPSVVVQVLSFRWFAYDYSDANGSGSAAAAMVAASN
AESHFSTKDSDQRGAGRSAAVTGSRSSAQSCCRVCVWLFQSAVHVLQLAQVWRYVHALYL
GVQSRWRGEHEHKHYYWRTMFENADISMLRLLETFLKSAPQLVLQLSIMVQTGQILPLQG
LSASASLVSLGWMMASYQKALRDSRDDKLPMSYKAVVVHMLWHLFTVGARAMAFALFVSL
FQLYFGIFIVAHWCAMTFWIIQGETDFCMSKWEEIIYNMMVGIVYIFCWFSVREGPTRGR
LLLYSLIIILAENVALTAVWYIYRSPRTSDFYAVVVVCVVACSYALGTFFMFVYYCLLHPD
GPVPGAYLGCCGVQVGAVSDPCVSSPTSAPPLDVVSSPPRTLQRTKGIDSVPMGDIGEVF
KMRTLKASRNAAPRVTPRTEGPVIRIDLPRKQYPAWDAHFIDRRLRKTILVLEGAAPVTP
RIQYRCLGTPKEVMEYETTV

>Danio rerio_XKR7b(ENSDARP_34312)
MVILWKYYQRSIRYVHTLYLGVQSRWHGGPGQRHFYWRLMFESADINMLRLLEAFLKSAP
QLVLQLSIMIHSNHILPLQGLSASASLVSLAWMIASYQKVLRDSRDDKLPMSYKAVIVQM
LWHLFTIGARTIAFALFASVFQLYFGIFIVAHWCAMTFWIIQGETDFCMSKWEEIIYNMV
VGIIYIFCWFNVKEGRARFRLGVYYCVTLFENVALTAAWYIHRGPHTSDFYALIIVCVVV
CSYALGTFFMFVYYCLLHPDGPILGTQWSCVEEGMGVIGLGGIGDLGTPLPQPDVVTSPP
RTLPRTTGGDRENGAGDRDSCMPVFQVRPPSVPPPSSPRPPRTEGPVIRIDLPRKRYPAW
DAHFIDRRLRKTILVLENTSPVTPRIQYQTFSNPKEVMEYETTV

>Takifugu rubripes_XKR7(ENSTRUP_15851)
MAAKSDGAPLSQRNEIPAECPPGPDPRAPRHRPAEQRYSLPDCCWTLCALLVFFSDGASD
LWLAADYYLRTYYWCFALTLVFVIVPSLVVQVLSFRWFAYDFAESVESGSAAAAVVAASG
EEDFSTKDSGERGAGRAAAAEVLPGPGTVGGARGCCRVFVWLFQAIVHIFQLAQVWRYVH
ALYLGVQSRWHRDPERRHFYWRMMFESADISMLRLLESFLKSAPQLVLQLSIMIYTKQVL
PLQGLSASASLISLAWMIASYQKVLRDSRDDKLPMTYKAVVVQILWHLFTIGARTLAFAL
FASVFQLYFGIFIVAHWCIMTFWIIQGETDFCMSKWEEIIYNMMVGVVYIFCWFSVREGR
TRCRMLIYSLTVFVENVALTTIWYLYRGSRTSESYAVIMVCVVASSYALGTFFMFVYYCL
LHPDGPVSGWGYIVEKEVPVESLGSPASSHPPDVVSSPPRTLQRTKGSEREQGAGIDGDV
FQVRPPRGAHAPVNHPTPRTEGPVIRIDLPRKKYPAWDAHFIDRRLRKTILVLESAAPVT
PRIQYRCLGNPKEVMEYETTV

>Tetraodon nigroviridis_XKR7a(ENSTNIP_15450)
QYVHALYLGVQSRWHGDPERRHFYWRMTFASADISMLRLLESFLKSAPQLVLQLSIMVHT
QQVLPLQGLSASASLLSLAWMVASYQKVLRDSRDDKLPMSYKAVAVQILWHLFAIGARTL
AFALFASAFQLYFGIFVVAHWCVMTFWIIQGETDFCMSKWEEIVYNMMVGVVYIFCWFSV
REGRTRCRMLLYSLTVLVENVALTAVWYLYRGAGASESYAVVMVCVVASSYALGTFFMFV
YYCLLHPDGPVSGWGYVVEKELPAEPLASPASSHPPDVVSSPPRTLQRTKGSEREPGAGV
DGDVFQVRPPRGARAPVNPPTPRTEGPVIRIDLPRKKYPAWDAHFVDRRLRKTILVLESA
APATPRIQYRCLGTPKEVMEYETTV

>Tetraodon nigroviridis_XKR7b(ENSTNIP_14283)
KFLNWKYDLRYFHALYLGAQSHLHGSSPGRHFHWRLMFESADITMLRLLEAFLKSAPQLV
LQLSIMIHGNAVLPLQGLSASASLVSLAWMIASYQKVLRDSRDDKLPMSYKAMITQMLWH
FFTIGARTVAFALFASVFQLYFGIFIVSHWCVMTFWISKETDFCMSKWEEIIYNMVVGII
YIFCWFNVKEGPSRFRMIIYYSVTLAENVALTAAWYTYRGPQTSDSSALVVVCLVACSFA
LGTFFMLVYYCWLHPDGPVLGLHWAGCVDEGMVSAGGVVGVIDACLTPSQGSQTDMVITS
PPRTLPRTKDMGEAIPGERDRDSCLPVFQVRPSPSSSAALLQKASSSSRAPEGPVIRIDL
PRKRYPAWDAHFIDRRLRKTILLVEATSTISPRIQYRSSMMGSKEVLEYETTV

>Gasterosteus aculeatus_XKR7a(ENSGACP_15752)
MAAKSDGAAVSLRNEIPAECPSGSDPRPSRHRAAEQRYSVPDCCWTLCALLVFFSDGASD
LWLAADYYLRGSYWCFALTLVFVVIPSVVVQVLSFRWFAYDFTETVESGAAAAAAVVGGG
ASTAGQSDFSTTDSGERGAGRSAEAGSLPGPGTGGGVRGCCRAFVWLFQAIIHIFQLAQV
WRYVHALYLGVQSRWHRDPERRHYYWRMMFESADISMLRLLESFLKSAPQLVLQLSIMNQ
ATQVLPLQGLSASASLISLAWMVASYQKALRDSRDDKLPMTYKAVIVQILWHLFTIGART
LAFALFASVFQLYFGIFIVAHWCIMTFWIIQGETDFCMSKWEEIIYNMMVGIVYVFCWFS
VREGRTRCRMLIYSLTVFIENVALTTAWYLYRDPRTSDFYAVIMVCAVASSYALGTFFMF
VYYCLLHPDGPISGWGYIVAKEVPVETLASPAASLPPDVVSSPPRTLQRTKGSEGERGSG
VDADVFQVRALRGAQAPAPNLTPRTEGPVIRIDLPRKKYPAWDAHFIDRRLRKTVLVLES
AAPVTPRIQYRCLGTPKEVMEYETTV

>Gasterosteus aculeatus_XKR7b(ENSGACP_13894)
ALRYIHALYLGAQSRWHGDGPRRHFHWRLMFESADITMLRLLEAFLKSAPQLVLQLSIMI
HGNIVLPLQGLSASASLVSLAWMIASYQKVLRDSRDDKLPMSYKAVITQMLWHFFTIGAR
TVAFALFASVFQLYFGIFIVSHWCVMTFWIIQGETDFCMSKWEEIIYNMVVGIIYIFCWF
NVKEGPSRLRMTVYYSVTLAENVALTAAWYTYRGPHTSDSYALVVVCLVACSFALGTFFM
LVYYCWLHPDGPVLGSQWGGCVDDGGVSAGGVAGVIDACLTPSQGSQTDMVITSPPRTLP
RTKDTGEPVPGERDKDRDRDSCLPIFQVRPSPSSSPALLHRTSSSSRALDGPVIRIDLPR
KRYPAWDAHFIDRRLRKTILLLETTSAASPRIQYRSSMMGSKEVLEYETTV

>Oryzias latipes_XKR7a(ENSORLP_18662)
RAFQLLSRMPALTRKMKTNRYVHALYLGVQSRWHGDPERRHYYWRMMFETADISMLRLLE
SFLKSAPQLVLQLSIMIHTSQVLPLQGLSASASLMSLAWMISSYQKVLRDSRDDKVPMTY
KAVIVQILWHLFTIAARTLAFALFASVFQLYFGIFIVAHWCIMTFWIIQGETDFCMSKWE
EIIYNMMVGIIYVFCWFSVREGRTHCRLLIYSVTVFVENVALTTIWYMYRGGTDRHGNSD
FYAVMMVCMVASSYALGTFFMFVYYCLLHPEGAVSGWSYTVEKEVPVELLASPASSLPPD
LVSSPPRTLQRTKGSDREQDGDVFKVRPLRGAKPPVPNLKPRTEGPVIRIDLPRKRYPAW
DAHFIDRRLRKTILVLEKAAPVTPRIQYRCLVTPKEVMEYETTV

>Oryzias latipes_XKR7b(ENSORLP_20930)
RYIHALYLGAQSRWHSNGPRRHFHWRLMFESADITMLRLLEAFLKSAPQLVLQLSIMIHG
NGVLPLQGLSASASLVSLAWMIASYQKVLRDSRDDKLPMSYKAVIAQMLWHFFTIGARTV
AFALFASVFQLYFGIFIVSHWCVMTFWIIQGETDFCMSKWEEIIYNMVVGIIYIFCWFNV
KEGPSRFRMALYYSVTLAENVALTAAWYTYRGPQTTDSSALVVVCLVACSFALGTFFMLV
YYCWLHPDGPVLGSQWGGCVDEGVVGAGGVVGVIDACLTPSQGSQTDMVITSPPRTLPRT
KDTGEPVPGERERDRDSCLPVFQVRPSPSASPALLHRNSSLSRAQEGPVIRIDLPRKRYP
AWDAHFIDRRLRKTILLLENTSAVSPRIQYRSSMMGSKEVLEYETTV

>Homo sapiens_XKR8(ENSP_362991)
MPWSSRGALLRDLVLGVLGTAAFLLDLGTDLWAAVQYALGGRYLWAALVLALLGLASVAL
QLFSWLWLRADPAGLHGSQPPRRCLALLHLLQLGYLYRCVQELRQGLLVWQQEEPSEFDL
AYADFLALDISMLRLFETFLETAPQLTLVLAIMLQSGRAEYYQWVGICTSFLGISWALLD
YHRALRTCLPSKPLLGLGSSVIYFLWNLLLLWPRVLAVALFSALFPSYVALHFLGLWLVL
LLWVWLQGTDFMPDPSSEWLYRVTVATILYFSWFNVAEGRTRGRAIIHFAFLLSDSILLV
ATWVTHSSWLPSGIPLQLWLPVGCGCFFLGLALRLVYYHWLHPSCCWKPDPDQVDGARSL
LSPEGYQLPQNRRMTHLAQKFFPKAKDEAASPVKG

>Pan troglodytes_XKR8(ENSPTRP_737)
MPWSSRGALLRDLVLGVLGTAAFLLDLGTDLWAAVQYALGGRYLWAALVLALLGLASVAL
QLFSWLWLRADPAGLHGSQPPRRCLALLHLLQLGYLYRCVQELRQGLLVWQQEEPSEFDL
AYADFLALDISMLRLFETFLETAPQLTLVLAIMLQSGRAEYYQWVGICTSFLGISALLDY
HALRTCLPSKPLLGLGSSVIYFLWNLLLLWPRVLAVALFSALFPSYVALHFLGLWLVLLL
WVWLQGTDFMPDPSSEWLYQVTVATILYFSWFNVAEGRTRGRAIIHFAFLLSDSILLVAT
WVTHSSWLPSGIPLQLWLPVGCGCFFLGLALRLVYYHWLHPSCCWKPDPDQVDGARSLLS
PEGYQLPQNRRMTHLAQNFFPKAKDEAALPVKG

>Gorilla gorilla_XKR8(ENSGGOP_14554)
MPWSSRGALLRDLVLGVLGTAAFLLDLGTDLWAAVQYALGGRYLWAALVLALLGLASVAL
QLFSWLWLRADPAGLHGSQPPRRCLALLHLLQLGYLYRCVQELRQGLLVWQQEEPSEFDL
AYADFLALDISMLRLFETFLETAPQLTLVLAIMLQSGRAEYYQWVGICTSFLGISWALLD
YHRALRTCLPSKPLLGLGSSVIYFLWNLLLLWPRVLAVALFSALFPSYVALHFLGLWLVL
LLWVWLQGTDFMPDPSSEWLYRVTVATILYFSWFNVAEGRTRGRAIIHFAFLLSDSILLV
ATWVTHSSWLPSGIPLQLWLPVGCGCFFLGLALRLVYYHWLHPSCCWKPDPDQVDGARSL
LSPEGYQLPQNRRMTHLAQKFFPKAKDEAASPVKG

>Mus musculus_XKR8(ENSMUSP_41205)
MPLSVHHHVALDVVVGLVSILSFLLDLVADLWAVVQYVLLGRYLWAALVLVLLGQASVLL
QLFSWLWLTADPTELHHSQLSRPFLALLHLLQLGYLYRCLHGMHQGLSMCYQEMPSECDL
AYADFLSLDISMLKLFESFLEATPQLTLVLAIVLQNGQAEYYQWFGISSSFLGISWALLD
YHRSLRTCLPSKPRLGRSSSAIYFLWNLLLLGPRICAIALFSAVFPYYVALHFFSLWLVL
LFWIWLQGTNFMPDSKGEWLYRVTMALILYFSWFNVSGGRTRGRAVIHLIFIFSDSVLLV
TTSWVTHGTWLPSGISLLMWVTIGGACFFLGLALRVIYYLWLHPSCSWDPDLVDGTLGLL
SPHRPPKLIYNRRATLLAENFFAKAKARAVLTEEVQLNGVL

>Rattus norvegicus_XKR8(ENSRNOP_17619)
MPLSVHPQVALDVVIGLVSTLSFLLDLVADLWAVVQYVLVGRYLWAALVVVLLGQASVLL
QLFSWLWLTADPTELHQLQPSRRFLALLHLLQLGYLYRCLHGMRQGLSMCCQEVPSECDL
AYADFLSLDISMLRLFESFLEATPQLTLVLAIVLQSGNAEYYQWFGISSSFLGISWALLD
YHRSLRTCLPSKPRLGWCSSAVYFLWNLLLLGPRICAIATFSVVFPYCLALHFLSLWLVL
LYWVWLQDTKFMPNSNGEWLYRVTVALILYFSWFNVSGGRTRGRATIHLGFILSDSVLLV
TTSWVTDSTWLPGGVLLWAALGGACFSLGLVLRMIYYLRLHPSCSWEPDFVDGTLRLLPP
ERPPKLIYNRRATRLAQNFFAKLKTQAALPQAVQLNGVL

>Canis familiaris_XKR8(ENSCAFP_17596)
MPWLPRAALLWDVVLGFGGTAAFLIDLGADLWAAGQYVVSGRYLWAALVLALLGLASVAL
QLFSWLWLRADPAGLHAPQPPGRCLALLHLLQLGYLYRCVQGLQQGLLVWRQEAPSEFDL
AYADFLTLDISMLRLFETFLETTPQLTLLLAIVLQSGRAESYQWVGICTSFVGISWALLD
YHRALRTCLPSKSLLGLGSSVVYFLWNLLLLWPRVLAVALFSALFPHYVALHFLGLWLVL
LFWVWLQGTDFMPDSCSEWLYRATVATILYFSWFNVAEGRTRGRATIHLVFLLSDSVLLV
VTWVTHIAWLPGGIPLQMLLPVSGICFLLGLALRLVYYRWLHPRCRQEPDRVDGAWGLRS

LEWRRLPQNRRMAQLAQSFFPRVRDEAALPQKGEANGVL

>Felis catus_XKR8(ENSFCAP_16867)
MPWLPRAALLWDLVLGFLGTAAFLVDLGADLWAAVQYVLSGXHLWQMLSTSCFDEAALSL
QPLGSLLTLPWTPCPSLWASPQDPKKFLCLLTFENYHLRQFHSMPRCVQGLQQGLLVWQQ
EAPSDFDLAYADFLTLDISMLRLFETFLETTPQLTLVLAIVLQSGRAESYQWVGICTSFV
GISWALLDYHRALRTCLPSKPPLGPGSSVVYFLWNLLLLWPRVFVVALFSALFPHYVALH
FLGLWLVLLFWVWLQGTDFMPDSCSEWLYRATVATILYFSWFNVAEGRTRGRAAIHLVFL
LSDSVLMAVTWLTHIAWLPSGIPLQTLLPVGGVCVLLGLALRLVYYRWLHPSCRWEPDRV
DGTRGLGSLEWRRLPQNRRMAQLAQNFFPRVRDEAALPQKGEVNGVL

>Myotis lucifugus_XKR8(ENSMLUP_140)
MPCSLRAILLRDLILGVLGISAFLIHLGSDLWAVSQYVLSGRYLWAALVLSLLGVASVAL
QVFSWVWLRADPADLHSSQLSARCLALLHLLQLGYLYRCMQGLQQGLLVWQQEVPSPFDL
AYADFLSLDISMLRLFETFLEKTPQLTLVLAIVLQSGQAEYYQWFGICTSFMGISWALLD
YHRALRTCLPSKPVLGLGSSVIYFLWNLLLLWPRVLAVALFSALFPRYVALHFLGLWLVL
LFWVWCQGTNFMPDPRSEWLYRATVATILYFSWFNVAEGHTRGRATIHLVFLLSDSVLMV
VTWVTQSAWLPSGIPLQMLLPVGGTSFLLGLGLRLVYYRWLHPSYPWEPDQVDGTTAFRS
LEHYQLPQNRRMDHLAQNFFSKARDEAALPWPGEVNGVL

>Monodelphis domestica_XKR8(ENSMODP_13478)
MEWKGRHRRGGVSQGIGGDGMEGAGTPFSDGVPRAGVAPEEAGGSGAMPGYPRVCRLRDL
LLTALGTGAFVLDLGADLWAAGQYALQGHLLWTALQLASLGLASAVLQLFSWAWYRGDPA
GLHPGPSSGRWLAFLHFLHLGYLHRCVRALKVGFSLWRQDEPAEVDLDYANFISMDISML
RLFETFLEATPQLILVLHITLCMGRVEYYQWLGIGTSFLCTAWALLDYHRALRSCLPTKP
PLGYLSSAVYFLWNLLLLCPRILALALFTTVFPWYIAVHFLTLWLVLLLWVWLQNTDFMP
GPTSEWLFRGTVALILYFSWFNVSEGRTRNRSIIHFSFLLMDSALLGGTWLAHNVPFLGS
SLLPWLLPGAALCFLLGLGLRGAYYQWLHPSRQMAALDEGDGRGLTLQDWHQNRRIAKLA
QSFFPRAVPAKRRGVNGAV

>Danio rerio_XKR8(ENSDARP_58512)
MECANISKYSWLDLAFTVIGVCTYLFDVGSDLWVAKEFYVHGEFIWFGVLVGFMLVSSVT
VQLFSWFWLQYDRKLENFECQESAGNAVLFSGAKRVTFSLLLHVFQLGLFIRHISAICQG
FRVWCHGGEGSEYAVYQTHDLSMLRLIETFCESAPQLTLMMYIMLRNNHARIVQCVSVVA
STTSMAWMVVDYHQSLRSFLPDKDKQGCLSSVVYFFWNLFLIAPRVVAVALFASVLPCFI
AVHFLLLWSVFVLWAWRQGTDFMDSPPGEWLYRATIGLIWYFSWFNVSEGHTRDRGFIYH
IFMMADTSIFLTTWWMYRDVEATQSYAVSLIIAIPLCYILGLMIKVLYYCCFHPKLWRPP
DLRVAADASDGLEPFSIVKTVGASFQSINNRMCKHAANFYVTNTSRALKTHECEARM

>Takifugu rubripes_XKR8(ENSTRUP_29820)
DFVFSVIGVFTFFVDWGADVWVATEFYSRGDFFWFGLLVSLMVLSSVLVQMFSWFWLKYD
RVGHHRNQPKRSFLGTLHLLLCHLHLRRHISAIRQGFRVWWRQQEGSEYAVYLTHDLSML
RLIETFSESAPQLTLMVHVMLCTNRARTVQFVSVAASTTSIAWMVVDYHRSLRAFLPDKA
KQGWGSSLIYFLWNFLLIAPRVAALALFASVVGGFVAVHFLLLWCVFVMWAWLQGTEFMD
SVCGEGLYRATVGIIWYFSWFNVAEGQTRGRSIIYHSFITTDGGILLLTWWCYRDPVQTE
PYGLALLVTLLFSYLLGLLFKTVYYCCFHPT

>Tetraodon nigroviridis_XKR8(ENSTNIP_6409)
MEQPSIYNYSWVDFVFSVIGVFTFFVDWGADVWVATEFYRRGDFFWFGVLVGLMVLSSVL
VQMFSWFWLQYDRGLPDLQTGGGTVLFGDRLRLSCLLHVLQLGFLCRHISAIRQGFRVWW
RKEEGSEYAFYLSHDLSMLRLIETFSESAPQLTLMIYVMLRTQRARAVQFVSVAASTTSI
AWMVVDYHRCLRSFLPDKARQGWASSLIYFLWNLLLIAPRVAALALFASVVGGYLGLHLL
LLWLVFVTWAWLQRTHFMDSPGGEWLYRATVGIIWYFSWFNVAEGRTRGRSAIYHAFITA
DGAILLVTWWCCREPVQAEPYALALLLTLLLSYLLGLLFKSLYYCCFHPTLWRPPAREPG
LPDDLPDADVTFRHLSIQDGAPASRPLNRRMALHAADFYSARTS

>Gasterosteus aculeatus_XKR8(ENSGACP_3110)
MEGATFSKYSWIDFVFSVIGACTFLVDWGSDVWLAAEFYRRGDVTWFWVLVGLMALSSFV
VQTFSWFWFQYDRELPGFRAQTEAAAVLLGDRAKLSCAVHVLQLGFLCRHISAIRQGFRV
WWRKEKGSEYAIYQTHDLSMLRLIETFSESAPQLTLMIYVMLHTHKARAVQFVSIAASTT
SIAWMVVDYHRSLRSFLPDKAKQGWGSSLIYFLWNLLLIAPRVAALALCASVLSGYMAAH

FLMLWSAFALWAWRQGTHFMDSVAGEWLYRATVGLIWYFSWFNVKDGQTRSRSAIYHSFI
STDGAILLATWWCHRDPVQTQSYALALLIALPLFHFLGLLFKALYYCCFHPKLWRPPARD
PGLPNDLPDAQVSMRDFPIQDGGLSPKLLNKRMAGQVARFYSDE

>Oryzias latipes_XKR8(ENSORLP_11799)
MQRGCFPRYSWIDFAFSVIGVCTFLMDWGSDLWVAAEFYCRGDLSWFGVLVGLMILSSVV
VQMFSWFWFVYDRKLSGFGSKVGTNSVLCGDRVKIFCLLHVLQLGFFCRHISAIRQGFRV
WWRKEEGSEYAIYLTHDLSMLRLFETFCESAPQLILMMYVMLRTNRARVVQFVSVAASTT
SIAWMVLDYHRSLRSFLPDKAQQGWGSSVVYFLWNLLLIGPRVAALALFSSVLPLLIVPH
FLLLWLAFLLWAWQQQTNFMDSAVGEWLYRATVGLIWYFSWFNVAEGHTRVRSLIYHSFM
TADCCLLLVTWWYLRDAAQTEAYGLALIIALPLSYLLGLLFKAIYYCCFHPKLWRPPPTE
GRLPNDLQIEVTSDLSIQDDSVSAPIFNKRMEHHAARFY

>Homo sapiens_XKR9(ENSP_431088)
MKYTKQNFMMSVLGIIIYVTDLIVDIWVSVRFFHEGQYVFSALALSFMLFGTLVAQCFSY
SWFKADLKKAGQESQHCFLLLHCLQGGVFTRYWFALKRGYHAAFKYDSNTSNFVEEQIDL
HKEVIDRVTDLSMLRLFETYLEGCPQLILQLYILLEHGQANFSQYAAIMVSCCAISWSTV
DYQVALRKSLPDKKLLNGLCPKITYLFYKLFTLLSWMLSVVLLLFLNVKIALFLLLFLWL
LGIIWAFKNNTQFCTCISMEFLYRIVVGFILIFTFFNIKGQNTKCPMSCYYIVRVLGTLG
ILTVFWVCPLTIFNPDYFIPISITIVLTLLLGILFLIVYYGSFHPNRSAETKCDEIDGKP
VLRECRMRYFLME

>Pan troglodytes_XKR9(ENSPTRP_53139)
MKYTKQNFMMSVLGIIIYVTDLIVDIWVSVRFFHEGQYVFSALALSFMLFGTLVAQCFSY
SWFKADLKKAGQESQHCFLLLHCLQGGVFTRYWFALKRGYHAAFKYDSNTSNFVEEQIDL
HKEVIDRVTDLSMLRLFETYLEGCPQLILQLYILLEHGQANFSQYAAIMVSCCAISWSTV
DYQVALRKSLPDKKLLNGLCPKITYLFYKLFTLLSWMLSVVLLLFLNVKIALFLLLFLWL
LGIIWAFKNNTQFCTCISMEFLYRIVVGFILIFTFFNIKGQNTKCPMSCYYIVRVLGTLG
ILTVFWVCPLNIFNPDYFIPISITIVLTLLLGILFLIVYYGSFHPNRSAETKCDEIDGKP
VLRECRMRYFLME

>Gorilla gorilla_XKR9(ENSGGOP_19148)
MKYTKQNFMMSVLGIIIYVTDLIVDIWVSVRFFHEGQYVFSALALSFMLFGTLVAQCFSY
SWFKADLKKAGQESQHCFLLLHCLQGGVFTRYWFALKRGYHAAFKYDSNTSNFVEEQIDL
HKEVIDRVTDLSMLRLFETYLEGCPQLILQLYILLEHGQANFSQFTFFNIKGQNTKCPMS
CYYIVRVLGTLGILTVFWVCPLTIFNPDYFIPISITIVLTLLLGILFLIVYYGTFHPNRS
AETKCDEIDGKPVLRECRMRYFLME

>Mus musculus_XKR9(ENSMUSP_85900)
MKYTKCNFMMSVLGIIIYVTDLVADIVLSVRYFHDGQYVLGVLTLSFVLCGTLIVHCFSY
SWLKADLEKAGQENERYFLLLHCLQGGVFTRYWFALRTGYHVVFKHSDRKSNFMEEQTDP
HKEAIDMATDLSMLRLFETYLEGCPQLILQLYAFLECGQANLSQCMVIMVSCCAISWSTV
DYQIALRKSLPDKNLLRGLWPKLMYLFYKLLTLLSWMLSVVLLLFVDVRVALLLLLFLWI
TGFIWAFINHTQFCNSVSMEFLYRIVVGFILVFTFFNIKGQNTKCPMSCYYTVRVLGTLG
ILTVFWIYPLSIFNSDYFIPISATIVLALLLGIIFLGVYYGNFHPNRNVEPQLDETDGKA
PQRDCRIRYFLMD

>Rattus norvegicus_XKR9(ENSRNOP_58714)
MKYTICNFMMSVLGIIIYVTDLVADIVLTVRYFYDGQYVFGVLTLSFVLCGTLIVHCFSY
SWLKDDLKKAGGENEHYFLLLHCLQGGVFTRYWFVLRTGYHVVFKHSHRTSNFMEEQTDP
HKEAIDMATDLSMLRLFETYLEGCPQLILQLYAFLERGQANFSQYMVIMVSCCAISWSTV
DYQIALRKSLPDKNLLRGFWPKLTYLFYKLFTLLSWMLSVVLLLFVDVRTVLLLLLFLWT
VGFIWAFINHTQFCNSLSMEFLYRLVVGFILVFTFFNIKGQNTKCPMSCYYTVRVLGTLG
ILTVFWIYPLSIFNSDYFIPISATIVLSLLFGIIFLGVYYGTYHPNINAGTQHDEPDGKA
PQRDCRIRYFLMD

>Canis familiaris_XKR9(ENSCAFP_34643)
MKYTKLTFMLSVLGIVIYITDLIVDIWVSVRFFYEGQNVFGILTVSFMLFGTLVVQCFSY
SWFKADLKKAGQESQCCFLLLHCLQGGVFTRYWFALKNGYHVAFKNSKTANFTEEQIDSH
KEVIDRMTDLSMLRLFETYLEGCPQLVLQLYTFLELGQANFSQYAAILVSCCAISWSTVA
YQVALRKSLPDKNLLNGFCPKFTYLFYKLFTLLSWKLSIVLLLFLDVKIALFLLLFLWFL

GILWAFKKQTQFCASVSMEFLYRVVVGFILTFTFFNIKGQNTKCPMFCYYIVRVLATVGI
LVVFWVSPVSIFNSDYFMSISITIALTLVLGIIFLIVYYGTFHPNRNEETKPDEIDGKTT
KRDCRMKYFLME

>Felis catus_XKR9(ENSFCAP_16605)
MKYTKLNFMMSVLGIVIYITDLIVDIWVSVRFFHEGQYVFGILTVSFMFFGTLVVQCFSY
SWFKADLKKAGQESQHCFLLLHCLQGGVFTRYWFALKKGYHVAFKYSSKTDNFMEEQIDQ
HKEVIDRMTDLSMLRLFETYLEGCPQLVLQLYTLLEHGQANFTQYAAIMVSCCAISWSTV
DYQVALRKSLPDKNLLNGFCPKLTYLFYKLFTLLSWMLSVVLLLFINVKIALFLLLFLWF
LGILWAFKKQTQFCASISMEFLYRVVVGFILTFTFFNIKGQNTKYPMFCYYIGRVLVTVG
ILIVFWVCPVSIFNSDYFIPISITIALSLVLGIIFLIVYYGTLHPNRNEETKPDEIDGIA
AERDCRMKYFLME

>Myotis lucifugus_XKR9(ENSMLUP_15612)
MKYTKSNFMMSVVGIIIYITDLIVDIWVSVRFFHEGQYVFGVLTVSFMLVGTLVVQCFSY
SWYKSDLKKAGQESQHRFLLLHCLQGGIFTRYWFALKKGYHVAFKYSSKTDNFIEKQIDP
HKEVIDGVTDLSMLRLFETYLEGCPQLVLQLYTCLEHGQANISQYAALMVSCCAISWSTV
DYQISLRKSLPDKNVLTGPCPKLTYLFYKFFTLLSWMLSVVLLLFLNVKIALCLLSFLWF
LGILWAFKNQTKFCASISMEFLYRIVVGFVLIFTFFNIQGQNTKCPMSCYYIVRVLATLG
ILIVLWFCPLSIFNSDYFIPISIIIALALILGIIFLIVYYGTLHPNKSKERKPDEIDGKP
VQIECRMKYFLME

>Monodelphis domestica_XKR9(ENSMODP_36453)
MGFTKLNFFILVLGILIYITDLIMDIWVSVNFFCKGQHIFGILTMSFMLIAAGIVQCFSF
MWYKEDYQKMGQKGPSCSLLIHCFQGGIFIRYWFALRRGYHLAFQCSSSDGLSGRPALTQ
DPTDLSTPSREEGADAHQTVINEVTDLSMLRLFEIYLEASPQLMIQLYSLMESDQADCIQ
VAAIVTSCCAISSATVDYHIALRKSLPDKKLLSGYFPRFIYFLYKLLTFTSWILTVGLLT
VISINLSLILLIIILWILGIMWACKQKTTFCTSKCMEILYRGVVGFILIFTFFNVKGENTK
FPMSAYYVMRALATLGILVVFWSCIPPDEYQEFFRDFTITIVAAVAFLLGILFLIAYYTS
FHPNRREEKKIFDEVDGRIPPRACRIRNFLMP

>Gallus gallus_XKR9(ENSGALP_35892)
MMKFTKWNFIFLLGGIIVYIADIGVDFWVASKYFYQGQYFWAVLILCFRGLSSLVTQIFS
YHWFKNDWNGTHPGKLDWIFLVHFFHCGIFIRYWFALKYGCQAAFRQDSSGDVSETDPPN
IIQKQAIDVLTDINMLRVFKTFLETTPQLFVQIYVLMEYNNHNFYQYAAIIISFCGISFS
MVDYQISLRKSLPDKEELEALPKLVYLFYKLLTITSWILSISLITLLSVRSSVIVLILLW
SCGFAWTLKQHTTFCKSKEMEYLYRTVVGIILLFTFFNIKGRKTKAYISIYYATHTLVTV
GILLVCLFWKPAVIEKVHLTIVSGLAVISLVLGLIFLAVYYKYFHPTTYCKQRTFFDEVD
RRGGDNMEVGRFQNFIMQ

>Anolis carolinensis_XKR9(ENSACAP_14517)
MIDHESKTMRFTKQDLGMMCFGIVVYVADVGADFWIARTYWCKEQYLWCGLILATGFFSS
VVIQLFSYIWFKEDRAEKLNWKLILLHLFHGGIFTRYWLALNQGFQAACKPKSSENILID
GPTSFIHRKAIYAMADISMLRLFKTYLESTPQLILQVYILMTSDNSTFTQYISITVSFIS
ISCATVDYQIALRKSLPDKKTFVKISSKMMYFSYKLLTLTSWILSIALLAVLSIKTSIIL
VAILWMGGVGWAWKQHTTFCKSKTPEIVYRVTVGIILVFTFFNIKGENTKIPMSVYYVIR
VLIISAISSASLFWKSSFNGKIYLSFMIAAIATALILGIIFLVIYYAFFHPNIYYPQDVV
DGPGSERNENCRMRAFLMQ

>Xenopus tropicalis_XKR9(ENSXETP_58401)
MKFSKWNFLMTIFGIFTLVCDYGVDLWIAVKYFQQGLYIFSLLTILFIVISNAIVNVFSY
AWIKDDCTEGNTRKLRWVWVIHVLMAGTFLRYWHAVKCGYRAAMSTHSEGDFTKTNKKAV
DAMTDLSMLRCFKTFLESTPQLILQIYILMEHGQITLLQYATIIISVFSISWSTVDYHMS
LRSSLENKKEISLGLPAVTYVLYKLLTLTSWILSLVFLLACNVYIFVALLGILWILGLCW
AWKQNTEFCTNMRMEILYRIVVAVILVLTFFNVKGQRTRVPMSVYYTFRIVATIGILILC
FLFKESLTRKLFFAVFSIALVLALGLGLMSLILYYCLFHPTLHSKDQNQDAADGCVDRSK
SHYFLSQ

>Takifugu rubripes_XKR9(ENSTRUP_30989)
MSYFTKLRWFLTIAGLFMYMADICTDAVLVFTYFKEKHFAFAALTLLFVVVGLLVTQLFS
SAWYLDDLNLGLIKAEAETPLPGVSTCGLAALHLLGMGIFVRYYHLLSQGFRLVWRTAEL

PGETLKHQHHLLFSLTADLSMLRLMEAFLESVPQLLLQLYITLGRQECSLIQSVGMTISF
LNAAWALVDYRRCLRKSLPDVNEMPFGFPTVIYLLYKLGTITSHIFGYALFLIFCTYSTV
ALAFIWLVGTIWTHSLHTDFCSSRSLEFLYRAIVGVILTFTFFNVKGQGTRDVMIIYYFF
HSLINILGPLLLFILRPELLAFSVLLCVSVLMASGSVLGLVCLVIYYLKLHPKEACREAD
EVDGLGTKKQPKQRLKNFIHP

>Tetraodon nigroviridis_XKR9(ENSTNIP_11017)
APLRMTEFTKLRWLLSAAGLVMYVADVCTDAALVLTYFKEKHVVCAALTLLFIVVGLLVT
QVFSTAWYWDDMDCDEKREDMKTTLPVVSKRGLATLHLFGVGIFIRYYQMLKRGFGVAWG
TVEPVEEIKNQQHFLFGLAADLSMLRLLEAFLESVPQLLLQLYIVLGQQECSLVQLVGMS
FSFMNAAWALVDYRRCLRRSLPDVRQMPRGLPTAIYLLYKLFTITSRVLGYALLLIFSIY
STVGLAIVWLLGTAWTHRLHTDFCSSQSLEFLYRAIVGVILTFTFFNVKGQGTRDAMITY
YFLHSLINVLSLLLLFVLRPDLLTLAALLCVSTLMAACSVLGLVCLVLYYLLLHPTEACR
EADEVDGLEIKTRSKGRLRDFLYP

>Gasterosteus aculeatus_XKR9(ENSGACP_3687)
IQYTKLRWCLTIAGLLLYVGDIGTDIGLAVRYFQKGDSVWAGLTLAFALAGLLVTQIFSY
AWYEDDRNDPVMSAVGKPTVSGLSKGRLAVLHLSGMGIFSRYYLLLKKGFKVLWTSPHSN
AAAERNDADHCLFCMATDLSMLKLFETFLESVPQLLLQLYILLAHGGSSVLQYLSMAFSF
FNIAWSLVDYRRCLRRSLPRIAAMPSGLPTAIYLLYKFCTITSHILSYTLLLALSPYSSI
GLTVLWLLGTTWTHVLQTDFCSSPKLEPLYRAVIGVILTFTFFNVKGQDAMVPMTIYYLF
YFFVNVTAPLLLGLLKPEWQTDPFLLTVSGVIFGGSVLGLCFLVLYYGFLHPKETQRQAD
EVDGVDVVVKPTRATRRRNNFLQP

>Oryzias latipes_XKR9(ENSORLP_10106)
LQKMNIPWSTVLYFFGFCLYIMDIVTDISLFVNYIKNGQYLWGGMTMMFVVTGTLSTQPF
SYVWYRDDDEEPKTLKAGLAVHLFGLGTIFRYYHLLKESCRVHRTTANSPTAERTGTEHN
LLFGMATDLCMLKMFEAFLESVPQILLQLYIIHDQNEGFFHQWLSVAFSVSNVAYTLMDF
RVCLRRSLPHARQKPSIPSRLVYLFYKLFTITSHILSYSLLLTLSVYSMVALTVLWLLMT
VWVHFLKTDFCTSKGLEFFYRLIVGFILVFTFFNVKGQDTGLAMTVYYMLYAIINIIAPI
LSMFLVPELQTRVFLCITCVIGGTTILGFMCLILYYYYLHPRGMQREPDEVDGLDTKAKA
TRRMRRFLQP

>Ciona savignyi_(ENSCSAVP_4599)
MLSSSHHKESLKTLQHKLLSPMSSHDSDTEIDLQTAMPLLPVHYSPQPKSAGYILAHGIS
AATIEHSNDLSPVELTPENICNKNIQSGDNRKDVVDSAPSREGRILDKENVYDNDCKNSQ
ISNSENNSECSQLSEVNENPKNVTCYSTVCILISCKNICTRFSIVDLVLILGSIGLYIFD
TATDILNGLSYYNKREENKDNLFYFILTMVVIVLPSFIMQAFSIWWEWEDDSKRNEKRKE
NGKMVKNTCCKHFFLCIVHVLQVGPIYRYIRAIHHGVKSRRRELQGNKKRFDFYDKMWKY
QLSDVSFLRVMESYLESAPQVVLQIYIMIKTMQTPFITVLSCCVSIIGMSSMLVSWQRNL
RDTLPKELNKKQISICGTIMLFLYRTCTITARCLLWHYF

>Strongylocentrotus purpuratus_(XP_011680834)
MSALKWITHICQAGPLHRYVNLFVTGMEARRTRDALDFERLFNQQSDVCMLRLFESFMESAPQVVLQLYI
MVATNDENFWTGTSAAVSLFSLCWALGAYSRAHRKVRRDKKVVSWPGLVLQTLWRIGMISSRVMALVLFA
SVFKAYIFLAVGIHWIGMMVWVHLQKTDFCTHPLEERLFNCVVAVIYIFCFFNLKEGKSRKRVLVFYSIM
FVENTVLLLVWYSYRTIGEWYNVAGFTIVFGGYGIGVVSMILYYRYLHPRNDFTLCGPKPEKEPSRSLNT
TPSMSPASSPAALFSPELYRHGHSPSPKQQRRSDVNHNDNAASPLARPLSQTSIHLDDDFDKEHGVPQRS
GKVKGQGHKVGRGDSIVRKSKSESCVSFNENETISPKVHLDKSTAGNSSVLMNASLMASFRCEISGFGTD
LVQKFDDWRDHSRLTNHSVMSAGGKSGNLTRSLGSMLDQSHVPSPAAAHCIDSADAARTPFSPDLQQMPE
IADSPNQIMTAYEAQQIYDSILGIHHPRRNSKRKLKFDDDPGKASGRTRSKSGNAVVHGKPAQKETGNRS
GDGGFGNGGPQRVVVTRKYVKDGCCVGRIGTSGQLLDQSKQSHHPVPNLGTKNRDKCSEGSHKILTHPAN
NAVVETKTKEIYPDNGKKIETETTNFQAVKVDMPIDLEGAKPLQGSKDNHDLGHVINEKQKGIQNQNKPS
ILIVDVNSARAEHETQIGIAAPRLRAALSRDLFKAKERVISRSSHITNKADVHNEGCDKAASVLGCKSDN
VKGIVAYHDEFPIVVGRPAEHNEETAIDASKSEPNAHAQSTKDLQCATTVLNSNDKTKQGHNIYVKHSGK
LASGLEDSRITEREEEDNRDPCENSNAKAQRNSPTKAGKLLGSLSPKAAIFSTSQGRFNGSHSPLIGGER
EPGGSRHKYHGRSPAGKTALKRVRSLKGSVPLDSLSLPQGSSPARDDSGKGHSEHERTPIANLKGKIKRA
LSIKAKIPYEGEVHVDSSPSIKSHLDHDPRRNDNHKTEKALNTISSPQVNVDKASELVMRERPVLVENQQ
SAPKSAFSPQKFEAISQSNKINPPQKTGVEPYSLPTTTLSSPRGEENRGFAPVSGNMIMNTSPQKNTENT
KKYSGSPQKTGKEHLSLSKGTRVRTPGKENRGFQPGTVKGTVSRQTNNKLGSPTGKKPPRRSLNRIPTNL
VADNVARFDSPSK

>Drosophila melanogaster_(FBpp0074004)
MASAEQRTEVDALMMGPISKLSMLLTVVSILWRFVSICINWSLAYVYWMEESYGYCAWTI
GSILVPMVVTSVIYIHTLKSAHAGEKRILERGVYSNAVISYLFRDVYVLNYAFKYSLAKE
RDDKQAEIEYYQKLMTEECNVSFVRLFDSFLESAPQKILQLAILLQSTLEFTYYRHIALI
VYFGNIAWCIQAYNHSNRLAQLDKHDIAAKGRFLQFLFLLCLTVSRTLCIAYVASIFPIE
TLIICATLACFYGTIVFFVDSPMIAKSRPMNYSYCLCFGVVYLFIFTPVKDAPTKYKYAF
YLTFCLLQNIIACALYIPLYLATAIIALYIVGIVLLIIYYTYCHPNTVRTYF

>Capitella teleta_(ELU18097)
VFDLLGNIVGIGSYLSDVVTDCWVAYTHFVRYNWWWFSLTLGFVIIPSLVMTFFSTVWYIQEHRDPHHQH
IKYSLDDHPSESKKKKVSTRRWVSRFILLFFQVAPVLRYLDSIIFCCRSWMANKKGNHRVKRHWYERMLY
EDTDATLLRLMECFMEAAPQLVLQLYIISINGLHDEWYNAAAQVLSCVTSLFSLSWAIAAYHKALRLSLA
DKSNLNWKGFALLILWRVFTIGARISAMALFASEVSPWIFAAAIAIHWLVMFIWCLTKKTEFCHHSWQEP
FFKAVMSVVYIFGFLNLLEGYTRFRYIFFYSLVYLENLAMMMVWFWMGGTDMRWFHKGAVVCIVLGFLVG
VTFQMVYYKFYHPN

>Nematostella vectensis_(XP_001631494)
RYWNVFRTGWRTRRSTASSSEYHSFLSQWRDICMLRLLEAYLESAPQLVLQLYILSYRRRFDMNSDLITA
IAAACSLVSLAWAIVAYQKSLRDVFCDNVSWTGFFLQVMWRLFMVASRVVAMVLFASYFNLWLGVAIALH
WLLMFIFLVTQSTKFCMDNDGRDHPLREMLFDGTIAFIYIFSFFNITEGMTRIRVGIFYSLMFIENSVFV
LLWYPHRSLFGDVAIAAITIVIGGFFLGLLSMFMYYH

>Amphimedon queenslandica_(XP_011409635)
MAIIDSNFTLQLVLSPLLIVFASVVSSAVSLLLTLLVYILSDRLHAERRRVVIPAYIVTFLWHLFVIPSR
IFAMVLFAVAYGPYVAVVVGVHWGAAIIWTLAERTNFCGDLSTTPPKKRYLLELPFVGVVSFIFLFIYFN
VRDGSTMVRIIVYHLLTAVETLLLSALFYVAYPNLSLSPWVFALSVSSYGLGVAFMFLYYAAWHPSRTED
CFLCIGCPKSCECCPCFSDKGELHLGNVDLPLDGERRRDNPRDEERSSGGDIEGEERPVTGRRTPNQNGF
YPSTPHRAGSSSMNNINRSTGGTPTHVRPSLQLNNHFSSAVEIPRNTSLYGGSGGGEENPYSLPFHHVGV
PHTGALSLENIKRVSPYRAPSRGSLREGEEKRWPVKTKTENLPRRMLPLVTNASDPRSTPLPRRGSDSQL
LPGASLNKSKYSNFNGKQRHSMIVHPDLQLSSNINLPSPIGEEPGALDGGRKWSSPLPILHVSSADSPGR
GRGERGRGRGRIAKERRGSGSLLPSPYSSFMYVTPPTPTVNTSMAASSSSLNPSYPVRSSSQLETRFYLE
PGAGIGVKRYRHRSLSPDVHSSEGSGCGYPAYSRSANATPKQSPYSSCRSSSGCAPRKRRSGSSNPLHSG
DPMVEFTQLYTSGASPTHMRRPSSRSSARVSTTCDESSDCGAGRRGRTSACSSGVFSSDKSDTGFSVPPT
GSEVNEPRPVLGVESNDGCGRGEERGRKDKRWQPSVNSDVGVMGGDTLV

YTH Domain-Containing Family Protein-YTHDF

>Homo sapiens_YTHDF1(ENSP_359364)
MSATSVDTQRTKGQDNKVQNGSLHQKDTVHDNDFEPYLTGQSNQSNSYPSMSDPYLSSYY
PPSIGFPYSLNEAPWSTAGDPPIPYLTTYGQLSNGDHHFMHDAVFGQPGGLGNNIYQHRF
NFFPENPAFSAWGTSGSQGQQTQSSAYGSSYTYPPSSLGGTVVDGQPGFHSDTLSKAPGM
NSLEQGMVGLKIGDVSSSAVKTVGSVVSSVALTGVLSGNGGTNVNMPVSKPTSWAAIASK
PAKPQPKMKTKSGPVMGGGLPPPPIKHNMDIGTWDNKGPVPKAPVPQQAPSPQAAPQPQQ
VAQPLPAQPPALAQPQYQSPQQPPQTRWVAPRNRNAAFGQSGGAGSDSNSPGNVQPNSAP
SVESHPVLEKLKAAHSYNPKEFEWNLKSGRVFIIKSYSEDDIHRSIKYSIWCSTEHGNKR
LDSAFRCMSSKGPVYLLFSVNGSGHFCGVAEMKSPVDYGTSAGVWSQDKWKGKFDVQWIF
VKDVPNNQLRHIRLENNDNKPVTNSRDTQEVPLEKAKQVLKIISSYKHTTSIFDDFAHYE
KRQEEEEVVRKERQSRNKQ

>Macaca mulatta_YTHDF1(ENSMMUP_38261)
MSATSVDTQRTKGQDNKVQNGSLHQKDTVHDNDFEPYLTGQSNQSNSYPSMSDPYLSSYY
PPSIGFPYSLNEAPWSTAGDPPIPYLTTYGQLSNGDHHFMHDAVFGQPGGLGNNIYQHRF
NFFPENPAFSAWGTSGSQGQQTQSSAYGSSYTYPPSSLGGTVVDGQPGFHSDTLSKAPGM
NSLEQGMVGLKIGDVSSSAVKTVGSVVSSVALTGVLSGNGGTNVNMPVSKPTSWAAIASK
PAKPQPKMKTKSGPVLGGALPPPPIKHNMDIGTWDNKGPVPKAPVPQQAPSPQAAPQPQQ
VAQPLPAQPPPVAQLQYQSPQQPPQTRWVAPRNRNAAFGQSGGAGSDSNSPGNVQPNSTP
SVESHPVLEKLKAAHSYNPKEFDWNLKSGRVFIIKSYSEDDIHRSIKYSIWCSTEHGNKR
LDSAFRCMSSKGPVYLLFSVNGSGHFCGVAEMKSPVDYGTSAGVWSQDKWKGKFDVKWIF
VKDVPNNQLRHIRLENNDNKPVTNSRDTQEVPLEKAKQVLKIISSYKHTTSIFDDFAHYE
KRQEEEEVVRKERQNRNKQ

>Callithrix jacchus_YTHDF1(ENSCJAP_18479)
MSATSVDAQRTKGQDNKVQNGSLHQKDTVHDGDFEPYLTGQSNQSNSYPSMSDPYLSSYY
PPSIGFPYSLNEAPWSTAGDPPIPYLTTYGQLSNGDHHFMHDAVFGQPGGLGNNIYQHRF
NFFPENPAFSAWGTSGSQGGQTQSSAYGSSYTYPPSSLGGTVVDGQPGFHSDTLSKAPGM
NSLEQGMVGLKIGDVSSSAVKTVGSVVSSVALTGVLSGNGGANVNMPVSKPTSWAAIASK
PAKPQPKMKTKSGPVMGGALPPPPIKHNMDIGTWDNKGPVPKAPVPQQAPSPQAATQPQQ
VAPPVPTQPPPLAQPQYQSPQQPPQTRWVAPRNRNTAFGQSGGAGSDSNSPGNVQPNAAP
SVESHPVLEKLKAAHSYNPKEFDWNLRSGRVFIIKSYSEDDIHRSIKYSIWCSTEHGNKR
LDSAFRCMSSKGPVYLLFSVNGSGHFCGVAEMKSPVDYGTSAGVWSQDKWKGKFDVKWIF
VKDVPNNQLRHIRLENNDNKPVTNSRDTQEVPLEKAKQVLKIISSYKHTTSIFDDFAHYE
KRQEEEEVVRKERQNRNKQ

>Mus musculus_YTHDF1(ENSMUSP_37808)
MSATSVDPQRTKGQDNKVQNGSLHQKDAVHDNDFEPYLSGQSNPSNSYPSMSDPYLSSYY
PPSIGFPYSLSEAPWSTAGDPPIPYLTTYGQLSNGDHHFMHDAVFGQPGGLGNNIYQHRF
NFFPENPAFSAWGTSGSQGGQTQSSAYGSSYTYPPSSLGGTVVDGQTGFHSDSLNKAPGM
NSLEQGMVGLKIGDVTTSAVKTVGSVVNSVALTGVLSGNGGTNVNMPVSKPTSWAAIASK
PAKPQPKMKTKSGPIVGGALPPPPIKHNMDIGTWDNKGPAPKASAPQQTPSPQAAPQPQQ
VAQPLPVQPPPLVQPQYQSPQQPLQPRWVAPRNRNAAFGQSGGANSDSNSVGNAQPTSAP
SVESHPVLEKLKAAHSYNPKEFDWNLKSGRVFIIKSYSEDDIHRSIKYSIWCSTEHGNKR
LDGAFRSMSSKGPVYLLFSVNGSGHFCGVAEMKSPVDYGTSAGVWSQDKWKGKFDVKWIF
VKDVPNNQLRHIRLENNDNKPVTNSRDTQEVPLEKAKQVLKIIASYKHTTSIFDDFSHYE
KRQEEEEVVRKERQNRNKQ

>Rattus norvegicus_YTHDF1(ENSRNOP_37700)
MSATSVDPQRTKGQENKVQNGSLHQKDAVHDNDFEPYLSGQSNPSNSYPSMSDPYLSSYY
PPSIGFPYSLSEAPWSTAGDPPIPYLTTYGQLSNGDHHFMHDAVFGQPGGLGNNIYQHRF
NFFPENPAFSAWGTSGSQGGQTQSSAYGSSYTYPPSSLGGTVVDGQTGFHSDTLNKAPGM
NSLEQGMVGLKIGDVTTSAVKTVGSVVNSVALTGVLSGNGGTNVNMPVSKPTSWAAIASK
PAKPQPKMKTKSGPIVGGTLPPPPIKHNMDIGTWDNKGPAPKASAPQQTPSPQAAPQPQQ
VAQPLPVQPPPLVQPQYQSPQQPLQPRWVAPRNRNAAFGQSGGANSDSNSVGSAQPTSAP
SVESHPVLEKLKAAHSYNPKEFDWNLKSGRVFIIKSYSEDDIHRSIKYSIWCSTEHGNKR
LDGAFRSMSSKGPVYLLFSVNGSGHFCGVAEMKSPVDYGTSAGVWSQDKWKGKFDVKWIF
VKDVPNNQLRHIRLENNDNKPVTNSRDTQEVPLEKAKQVLKIIASYKHTTSIFDDFSHYE
KRQEEEEVVRKERQNRNKQ

>Canis familiaris_YTHDF1(ENSCAFP_18790)
MADPYLSSYYPPSIGFPYSLNEAPWSTGGDPPIPYLTTYGQLSNGDHHFMHDAVFGQPGG
LGNNIYQHRFNFFPENPAFSAWGTSGSQGQQAQSSAYGSSYTYPPSSLGGTIVDGQTGFH
SETLNKAPGMNSLEQGMVGLKIGDVTTSAVKTVGSVVSSVAMTGVLSGNGGTNVNMPVSK
PTSWAAIASKPAKPQPKMKAKSGPVIGAALPPPPIKHNMDIGTWDNQGPVPKAPAPQQAP
APQSAPQPQPVVQPLPTQPPPLAQPQYPSPQQPPQTRWVAPRNRNAAFGQSGGTSSDGNS
PGNTQPSSAPTVESHPVLEKLKAAHSYNPKEFDWNLKSGRVFIIKSYSEDDIHRSIKYSI
WCSTEHGNKRLDGAFRAASSKGPVYLLFSVNGSGHFCGVAEMKSPVDYGTSAGVWSQDKW
KGKFDVKWIFVKDVPNNQLRHIRLENNDNKPVTNSRDTQEVPLEKAKQVLKIIASYKHTT
SIFDDFSHYEKRQEEEEVVRKERQNRNKP

>Felis catus_YTHDF1(ENSFCAP_8264)
MADPYLSSYYPPSIGFPYSLNEAPWSTGGDPPIPYLTTYGQLSNGDHHFMHDAVFGQPGG
LGNSIYQHRFNFFPENPAFSAWGTSGSQGQQAQSSAYGSSYTYPPSSLGGTIVDGQTGFH
SDTLNKAPGMNSLEQGMVGLKIGDVTTSAVKTVGSVVSSVAMAGVLSGSGGTSVNMPVSK
PTSWAAIASKPAKPQPKMKAKSGPVIGAALPPPPIKHNMDIGTWDNKGPVPKPPAPQQAP
APQPAPQPQPVVQPLPAQPPPLAQPQYPSPQQPPQTRWVAPRNRNAAFGQSGGAGGDGNS
PGSAQPHSAPSVESHPVLEKLKAAHSYNPKEFDWNLKSGRVFIIKSYSEDDIHRSIKYSI
WCSTEHGNKRLDGAFRAAGSKGPVYLLFSVNGSGHFCGVAEMKSPVDYGTSAGVWSQDKW
KGKFDVKWIFVKDVPNNQLRHIRLENNDNKPVTNSRDTQEVPLEKAKQVLRIIASYKHTT
SIFDDFSHYEKRQEEEEEVRKERQNRNKQ

>Myotis lucifugus_YTHDF1(ENSMLUP_2032)
FPYLKVIFKNANTIFVSAILVQNGSLHQKDTVHDNDFEPYLSGQSNQSNSYPSMADPYLS

SYYPPSIGFPYSLNEAPWSTGGDPPIPYLTTYGQLSNGDHHFMHDAVFGQPGGLGNNIYQ
HRFNFFPENPAFSAWGTSGSQGQQAQSSAYGSSYTYPPSSLGGTIVDGQTGFHSDTLNKA
PGMNSLEQGMVGLKIGDVTTSAVKTVGSVVSSVAMTGVLSGNGGTNVNMPVSKPTSWAAI
ASKPAKPQPKMKAKGGPVIGAALPPPPIKHNMDIGTWDNKGPVPKALAPQQVPAPQSAPQ
PQPVVQPLSVQPPPLAQPQYQSSQPLPQTRWVAPRNRNAAFGQSSGTGGNSHSPGSTQSG
SALSAESHPVLEKLKAAHSYNPKEFDWNLRSGRVFIIKSYSEDDVHRSIKYSIWCSTEHG
NKRLDGAFRSVGSKGPVYLLFSVNGSGHFCGVAEMKSPVDYGTSAGVWSQDKWKGKFDVK
WIFVKDVPNNQLRHIRLENNDNKPVTNSRDTQEVPLEKAKQVLKIIASYKHTTSIFDDFS
HYEKRQEEEEVVRKERQNRNKQ

>Gallus gallus_YTHDF1(ENSGALP_9213)
MSATSVDPQRPKGQDNKVQNGSLHQKDTVHDNDFEPYLSGQSNQNSSYPSMTDPYLSSYY
PPSIGFPYSLSEAPWSTGGDPPIPYLTTYGQLSNGDHHFMHDAVFGQPGGLGNNIYQHRF
NFFPENPAFSAWGTSGSQGQQTQSSAYGSSYSYPPSSLGGTIVDGQTGFHNDTLNKAPGM
NSIEQGMVGLKIGGDVTTSAVKTVGSVVNSAGMTGALSGNGGSNVNLPVSKPTSWAAIAS
KPAKPQPKMTKTGPVIGGALPPPPIKHNMDIGTWDNKGPVAKVPAPQQIPSPQSVPQPQ
QQIVQPVPAQPPPLTQPQYQSPQQPPQNRWIAPRNRNAAFGQSGGTGNDSNSAGSTQPNP
VPSGESHPVLEKLKAAHSYNPKDFEWNLKNGRVFIIKSYSEDDIHRSIKYSIWCSTEHGN
KRLDSAFRSMNSKGPVYLLFSVNGSGHFCGVAEMKSPVDYGTSAGVWSQDKWKGKFDVKW
IFVKDVPNNQLRHIRLENNDNKPVTNSRDTQEVPLEKAKQVLKIIATYKHTTSIFDDFSH
YEKRQEEEEVVRKERQNRNKQ

>Anolis carolinensis_YTHDF1(ENSACAP_5432)
MSATSVDPQRPKGQDNKVQNGSLHQKDTVHDNDFEPYLSGQSNQNNSYPSMTDPYLSSYY
PHSISFPYSLSEAPWSTGGDPPIPYLTTYGQLSNGDHHFMHDAVFGQPGGLGNNIYPHRF
NFFPENPPFSAWGTSGSQGQQTQNSAYGSSYSYPPSSLGGTIVDGQTGFHNDTLNKAPGM
NSIEQGMVGLKIGGDVTASAVKTVGSVVNSTGLACALSGNGGSSINLPVSKPTSWAAIAS
KPAKPQPKMKAKSGPVVGGALPPPPIKHNMDIGTWDNKGPVAKVPAPQQIPSPQSLPQPQ
IIQPIPTQPPPLTQLPYQNPQPPPPQNRWVAPRNRNAAFGQSGGTGNESNSLGSAPANSI
PGGESHPVLEKLKAAHSYNPKDFEWNLKNGRVFIIKSYSEDDIHRSIKYSIWCSTEHGNK
RLDSAFRSMNSKGPVYLLFSVNGSGHFCGVAEMKSSVDYGTSAGVWSQDKWKGKFDVKWI
FVKDVPNNQLRHIRLENNDNKPVTNSRDTQEVPLEKAKQVLKIIATYKHTTSIFDDFSHY
EKRQEEEEVVRKERQNRNKQ

>Xenopus tropicalis_YTHDF1(ENSXETP_56774)
FSPKVFPLGRSNNDLISVQNGSLHQKDSVHDNDFEQYLSGQSNQSNSYPSMTDPYLSSYY
PPSIGFPYSLSEAPWSTGGDPPIPYLTPYGQLSNGDHHFMHDAVFGQPGGLGNNIYQHRF
NFFPENPAFSAWGTSGSQGQQTQNSAYGGSYSYPPSSLGGTIVDGQTGFHNETLNKAPGM
NSIEQGMVGLKIGSNVTASAVKTVGSVVNSVGMPSALSGNGGTSISMPPSKPTSWAAIAS
KPAKPQPKAKTKPVTIIGGALPPPPIKHNMDIGTWDNKGPVAKPPMPQQIPSPQSMPPPQ
PQLLQPIPAQPPPMSQTPYQNPPPPQQQPQAPQNRWVAPRNRNAAYGQGGGPDGNPLGGA
QSHSAPGNESHPVLEKLKAAHSYNPKDFDWNLKNGRVFIIKSYSEDDIHRSIKYSIWCST
EHGNKRLDSAFRSMNGKGPVYLLFSVNGSGHFCGVAEMKSPVDYGTSAGVWSQDKWKGKF
DVKWLFVKDVPNNQLRHIRLENNDNKPVTNSRDTQEVPLEKAKLVLKIIATYKHTTSIFD
DFSHYEKRQEEEEVVRKVMAKPDSC

>Danio rerio_YTHDF1(ENSDARP_5746)
MSTTSIDPQTSKGQDAKVQNGSLHQKETVHDNDFEPYLTGQSNPNNSYQSMTDPYLSSYY
APSIGFPYPLSEAPWSTGGDPPIPYLTPYAPLSNGDHHFMHDTVFGQPGGLGSSIYPHRF
NFFPENPAFSAWGTSGSQGQQTQSSAYGGSYSYPPSSLGGTLVPDGQTGFPSDTLNKAPG
MNSLEQGMVGLKIGGDVTGGGSGVKTVGSVIGGGPVAAAVATGNGGTPIGLPPPKPTSWA
AIASKPAKPQQLKVKSKPGMPMAGGTLPPPPIKHNMDIGTWDNKGPVTKVASSLPPHHHH
QQQPPLHSQGHLPPHPHGLPPPPQPPMPSAQSLAQQMAMQGPPPPQPYQNHAPAPPPQTR
WVAPRNRNPGYGGGGSVDSSGSSSGGGVGNGGGGGPPGSGAIESHPVLEKLRAAHSYNPK
EFDWNLKNGRVFIIKSYSEDDIHRSIKYSIWCSTEHGNKRLDSAFRAINGKGPVYLLFSV
NGSGHFCGVAEMRSPVDYGTSAGVWAQDKWKGKFDVDWLFVKDVPNSQLRHIRLENNDNK
PVTNSRDTQEVPLEKAKQVLKIIATYKHTTSIFDDFSHYEKRQEEEEVVRKNLEPAPIQN
RSRLDQERQNRNKQ

>Takifugu rubripes_YTHDF1a(ENSTRUP_12114)
MSATSIEPQRSKGQASKVQNGSLHQKETVHDSDFEPYLSGQSTQNNSYQSITDPYLSSYY

APSIGFPYPLSEAPWSTGGDPPIPYLTPYGPLSNGDHHFMPDTVFGQPGGLGSSIYPHRF
NFFPENPAFSAWGTSGSQGQQTQSSAYGGSYSYPPSSLGGTLVPDGQTGFHSETLNKAPG
MNSLEQGMVGLKIGGDVAGQGSGVKAVGSVIGGPAVPTAGNGATPIGMPPPKPTSWAAIA
SKPAKPQQLKTKVKPGMPGSLGGALPPPPIKHNMNIGTWDKGPVTKGPMQQGHMQPPPPQ
SLVQPQMQPMALQPPHHQHHQHHQPPPQPYQNHGQPPQPQTRWIAPRNRNQGYGQGGPGQ
DCNCTMGLISGNGGPPSSISQGPGAESHPVLDKLRASHSYNPKDFDWNLKNGRVFIIKSY
SEDDIHRSIKYSIWCSTEHGNKRLDSAYRAMNAKGPVYLLFSVNGSGHFCGVAEMRSPVD
YGTSAGVWAQDKWKGKFDVDWLFVKDVPNSQLRHIRLENNDNKPVTNSRDTQEVPLEKAK
QVLKIIATYKHTTSIFDDFSHYEKRQEEEEEVRKVRPPPQYC

>Takifugu rubripes_YTHDF1b(ENSTRUP_12113)
MSATSIEPQRSKGQASKVQNGSLHQKETVHDSDFEPYLSGQSTQNNSYQSITDPYLSSYY
APSIGFPYPLSEAPWSTGGDPPIPYLTPYGPLSNGDHHFMPDTVFGQPGGLGSSIYPHRF
NFFPENPAFSAWGTSGSQGQQTQSSAYGGSYSYPPSSLGGTLVPDGQTGFHSETLNKAPG
MNSLEQGMVGLKIGGDVAGQGSGVKAVGSVIGGPAVPTAGNGATPIGMPPPKPTSWAAIA
SKPAKPQQLKTKVKPGMPGSLGGALPPPPIKHNMNIGTWDKGPVTKGPMQQGHMQPPPPQ
SLVQPQMQPMALQPPHHQHHQHHQPPPQPYQNHGQPPQPQTRWIAPRNRNQGYGQGGPGQ
DCNCTMGLISGNGGPPSSISQGPGAESHPVLDKLRASHSYNPKDFDWNLKNGRVFIIKSY
SEDDIHRSIKYSIWCSTEHGNKRLDSAYRAMNAKGPVYLLFSVNGSGHFCGVAEMRSPVD
YGTSAGVWAQDKWKGKFDVDWLFVKDVPNSQLRHIRLENNDNKPVTNSRDTQEVPLEKAK
QVLKIIATYKHTTSIFDDFSHYEKRQEEEEEVRKKKSSLNKL

>Tetraodon nigroviridis_YTHDF1a(ENSTNIP_6584)
MSATSIEPQRSKGQASKVQNGSLHQKETVHDNDFEPYLSGQSTQNNSYQSITDPYMSSYY
APSIGFPYPLSEAPWSTGGDPPIPYLTPYGPLSNGDHHFMPDTVFGQPGGLGSSIYPHRF
NFFPENPAFSAWGTSGSQGQQTQSSAYGGSYSYPPSSLGGTLVPDGQTGFHSETLNKAPG
MNSLQQGMVGLKIGGDVTGQGSGVKAVGSVIGGPTVPTAGNGATPIGMPAPKPTSWAAIA
SKPAKPQQLKTKVKPIKHNMNIGTWDKGPVTKVATVPPPQPLGLPHGLPPQGPMTQGPMQ
TRWIAPRNRNQGYGQGGLGQDCNCTMGLISGNGGPPTSVSQGPGAESHPVLDKLRACHSY
NPKDFDWNLKNGRVFIIKYSEDDIHRSIYSIWCSTEHGNKRLDSAYRAMNAKGPVYLLFS
VNGSGHFCGVAEMRSPVDYGTSAGVWAQDKWKGKFDVDWLFVKDVPNSQLRHIRLENNDN
KPVTNSRDTQEVPLEKAKQVLKIIATYKHTTSIFDDFSHYEKRQEEEEEVRKERQNRHKQ

>Tetraodon nigroviridis_YTHDF1b(ENSTNIP_15460)
MSAASIDPQETSKGQDASKVSVQNGSLHQKDTVHDTDFEPYLSNQSNQNNSYQSMTDPYL
SSYYAPSIGFPYPLSEAPWSTGGDPPVPYLTPYAPLSNGDHHFMHDTVFGQPGGLSSSIY
PHRFNFFPENPAFSAWGTSGSQGQQTQSSAYGGSYSYPPSSLGGTLVPDGQTGFHSDTLS
KAPGMNTIEQGMLGLKLGGDVAGTGSAVKSPGSVIGGGGSVAAAVATPIGMPTPKPTSWA
AIASKPAKLQQQKNKNKPGASIAGGPLPPPPIKHSMDIDTWDNKGTVAKMAAPLPQSLQS
AQSLVQQMTMPGPPPPPPSYQNHSSAPTPQTRWVAPRSRNFCFTPEPGAESHPVLEKLRA
AHSYNPREFDWNLKNGRVFIIKSYSEDDIHRSIKYSIWCSTEHGNKRLDSAFRAMNSKGP
VYLLFSVNGSGHFCGVEMRSPVDYGTSAGVWAQDKWKGKFDVSWLFVKDVPNSQRHIRLE
NNDNKPVTNSRDTQEVPLEKAKQVLKIITQYKHTTSIFDDFSHYEKKQEEEEVVKKERQK
RIKV

>Gasterosteus aculeatus_YTHDF1a(ENSGACP_3428)
MSATSIDPQRSKGQASKVQNGSLHQKEAVHDNDFEPYLTGQSTQNNSYQSITDPYLSSYY
APSIGFPYPLSEAPWSTGGDPPIPYLTPYGPLSNGDHHFMPDTVFGQPGGLGSSIYPHRF
NFFPENPTFSAWGTSGSQGQQTQSSAYGGSYSYPPSSLGGTLVPDGQTGFHSDTLNKAPG
MNSLEQGMVGLKIGGDVGGQCSGVKAVGSVIGGPVVAATGNGATPIGMPPPKPTSWAAIA
SKPAKPQQLKAKLKPGMSNLGGALPPPPIKHNMNIGTWDKGPVTKVATAPMQHQQPMGLP
PHGMPPQGHMQQGPMHPPPSQSMVQPQPYQNHNQPPQPQTRWVAPRNRNQGYGQGGYGQG
GPGHDGGGGVMGMMGSGNCGPPPCAGQGPGGESHPVLDKLRAAHAYNPKDFEWNLKNGRV
FIIKSYSEDDIHRSIKYSIWCSTEHGNKRLDSAFRAMSAKGPVYLLFSVNGSGHFCGVAE
MRSPVDYGTSAGVWAQDKWKGKFDVDWLFVKDVPNSQLRHIRLENNDNKPVTNSRDTQEV
PLEKAKQVLKIIATYKHTTSIFDDFSHYEKRQEEEEEVLKERQNRSKQ

>Gasterosteus aculeatus_YTHDF1b(ENSGACP_15892)
MSAASIDPQTTKGQDASKVSVQNGSLHQKDTVHDNDFEPYLTNQSNQNNSYQSMTDPYLS
SYYAPSIGFPYPLSEAPWSTGGDPPIPYLTPYAPLSNGDHHFMHDTVFGQPGGLSSSIYP
HRFNFFPENPAFSAWGTSGSQGQQTQSSAYGGSYSYPPSSLGGTLVPDGQTGFHSDTLSK

APGMNSLEHGMLGLKIGGDLTGSGLGVKSAGSLGGGSVAAAVATGNGGAPIGMPPPKPTS
WAAIAGKPAKLPQQKAKNKLGAPIVGGGLPPPPVKHNMDIDTWENKGALNKMAPPLPQQP
QLHSHAPSLLPPLQQSLQSAQSLVQQMTMQGPPLPPPQSYQNHNSAPTPQTRWIAPRNRN
LCYGGGSLDSSGSSNGGGVGNGGGGGAPPDPGSESHPVLEKLRAVHSYNPKEFDWNLKNG
RVFIIKSYSEDDIHRSIKYSIWCSTEHGNKRLDSAFRAMNSKGPVYLLFSVNGSGHFCGV
AEMRSPVDYGTSAGVWAQDKWKGKFDVNWLFVKDVPNSQLRHIRLENNDNKPVTNSRDTQ
EVPLEKAKQVLKIIAQYKHTTSLFDDFSHYEKKQEEEEVVKKSYEPAPIQSRSRIDQEKL
SRDQM

>Oryzias latipes_YTHDF1a(ENSORLP_12218)
MSATSIDPQRSKGQASKVQNGSLHQKESVHDNDFEPYLTSQSTQNNSYQSITDPYLSSYY
APSIGFPYPLSEAPWSTGGDPPIPYLTPYGPLSNGDHHFMPDTVFGQPGGLGSSIYPHRF
NFFPENPAFSAWGTSGSQGQQTQNSAYGGSYSYPPSSLGGTLVPDGQTGFHSDTLNKAPG
MNSLEQGMVGLKIGGDVPGQNSGVKAVGSVIGGPVAAAGNGATPIGMPPPKATSWAAIAS
KPAKPQQLKVKMKPGMPNPGGALPPPPIKHNMNIGTWDKGPVTKVAAAPLQQQQQPLGLP
HGMPPQGPMQPPSHQSLVQPQMQPIALQPQPPHHQHHHPPPQAYQNHTQPQQPLTRWVAP
RNRNQGYGQNGPCHDGGGVMGVVGVGHNGPPACGSQGTGGDSHPVLEKLRASHSYNPKDF
DWNLKNGRVFIIKSYSEDDIHRSIKYSIWCSTEHGNKRLDSAFRAMNGKGPVYLLFSVNG
SGHFCGVAEMRSPVDYGTSAGVWAQDKWKGKFDVDWLFVKDVPNSQLRHIRLENNDNKPV
TNSRDTQEVPLEKAKQVLKIIATYKHTTSIFDDFSHYEKRQEEEEEVRKTFEPPQIQNRS
RLDQERQNRNKQ

>Oryzias latipes_YTHDF1b(ENSORLP_12216)
MSATSIDPQRSKGQASKVQNGSLHQKESVHDNDFEPYLTSQSTQNNSYQSITDPYLSSYY
APSIGFPYPLSEAPWSTGGDPPIPYLTPYGPLSNGDHHFMPDTVFGQPGGLGSSIYPHRF
NFFPENPAFSAWGTSGSQGQQTQNSAYGGSYSYPPSSLGGTLVPDGQTGFHSDTLNKAPG
MNSLEQGMVGLKIGGDVPGQNSGVKAVGSVIGGPVAAAGNGATPIGMPPPKATSWAAIAS
KPAKPQQLKVKMKPGMPNPGGALPPPPIKHNMNIGTWDKGPVTKVAAAPLQQQQQPLGLP
HGMPPQGPMQPPSHQSLVQPQMQPIALQPQPPHHQHHHPPPQAYQNHTQPQQPLTRWVAP
RNRNQGYGQNGPCHDGGGVMGVVGVGHNGPPACGSQGTGGDSHPVLEKLRASHSYNPKDF
DWNLKNGRVFIIKSYSEDDIHRSIKYSIWCSTEHGNKRLDSAFRAMNGKGPVYLLFSVNG
SGHFCGVAEMRSPVDYGTSAGVWAQDKWKGKFDVDWLFVKDVPNSQLRHIRLENNDNKPV
TNSRDTQEVPLEKAKQVLKIIATYKHTTSIFDDFSHYEKRQEEEEEVRKISCNPWTPVKI

>Homo sapiens_YTHDF2(ENSP_444660)
MSASSLLEQRPKGQGNKVQNGSVHQKDGLNDDDFEPYLSPQARPNNAYTAMSDSYLPSYY
SPSIGFSYSLGEAAWSTGGDTAMPYLTSYGQLSNGEPHFLPDAMFGQPGALGSTPFLGQH
GFNFFPSGIDFSAWGNNSSQGQSTQSSGYSSNYAYAPSSLGGAMIDGQSAFANETLNKAP
GMNTIDQGMAALKLGSTEVASNVPKVVGSAVGSGSITSNIVASNSLPPATIAPPKPASWA
DIASKPAKQQPKLKTKNGIAGSSLPPPPIKHNMDIGTWDNKGPVAKAPSQALVQNIGQPT
QGSPQPVGQQANNSPPVAQASVGQQTQPLPPPPPQPAQLSVQQQAAQPTRWVAPRNRGSG
FGHNGVDGNGVGQSQAGSGSTPSEPHPVLEKLRSINNYNPKDFDWNLKHGRVFIIKSYSE
DDIHRSIKYNIWCSTEHGNKRLDAAYRSMNGKGPVYLLFSVNGSGHFCGVAEMKSAVDYN
TCAGVWSQDKWKGRFDVRWIFVKDVPNSQLRHIRLENNENKPVTNSRDTQEVPLEKAKQV
LKIIASYKHTTSIFDDFSHYEKRQEEEESVKKERQGRGK

>Callithrix jacchus_YTHDF2(ENSCJAP_18004)
MSASSLLEQRPKGQGNKVQNGSVHQKDGLNDDDFEPYLSPQARPNNAYTAMSDSYLPSYY
SPSIGFSYSLGEAAWSTGGDTAMPYLTSYGQLSNGEPHFLPDAMFGQPGALGSTPFLGQH
GFNFFPSGIDFSAWGNSSSQGQSTQSSGYSSNYAYAPSSLGGAMIDGQSAFANETLNKAP
GMNTIDQGMAALKLGSTEVASNVPKVVGSAVGSGSITSNIVASNSLPPATIAPPKPASWA
DIASKPAKQQPKLKTKNGIAGSSLPPPPIKHNMDIGTWDNKGPVAKAPSQALVQNIGQPT
QGSPQPVGQQANNSPPVAQASVGQQTQPLPPPPPQPAQLSVQQQAAQPTRWVAPRNRGSG
FGHNGVDGNGVGQSQAGSGSAPSEPHPVLEKLRSINNYNPKDFDWNLKHGRVFIIKSYSE
DDIHRSIKYNIWCSTEHGNKRLDAAYRSMNGKGPVYLLFSVNGSGHFCGVAEMKSAVDYN
TCAGVWSQDKWKGRFDVRWIFVKDVPNSQLRHIRLENNENKPVTNSRDTQEVPLEKAKQV
LKIIASYKHTTSIFDDFSHYEKRQEEEESVKKERQGRGK

>Mus musculus_YTHDF2(ENSMUSP_120414)
MSASSLLEQRPKGQGNKVQNGSVHQKDGLNDDDFEPYLSPQARPNNAYTAMSDSYLPSYY
SPSIGFSYSLGEAAWSTGGDTAMPYLTSYGQLSNGEPHFLPDAMFGQPGALGSTPFLGQH

GFNFFPSGIDFSAWGNNSSQGQSTQSSGYSSNYAYAPSSLGGAMIDGQSAFANETLNKAP
GMNTIDQGMAALKLGSTEVASSVPKVVGSAVGSGSITSNIVASSSLPPATIAPPKPASWA
DIASKPAKQQPKLKTKNGIAGSSLPPPPIKHNMDIGTWDNKGPVAKAPSQALVQNIGQPT
QGSPQPVGQQANNSPPVAQASVGQQTQPLPPPPPQPAQLSVQQQAAQPTRWVAPRNRGSG
FGHNGVDGNGVGQSQAGSGSTPSEPHPVLEKLRSINNYNPKDFDWNLKHGRVFIIKSYSE
DDIHRSIKYNIWCSTEHGNKRLDAAYRSMNGKGPVYLLFSVNGSGHFCGVAEMKSAVDYN
TCAGVWSQDKWKGRFDVRWIFVKDVPNSQLRHIRLENNENKPVTNSRDTQEVPLEKAKQV
LKIIASYKHTTSIFDDFSHYEKRQEEEESVKKERQGRGK

>Equus caballus_YTHDF2(ENSECAP_20585)
MSASSLLEQRPKGQGNKVQNGSVHQKDGLNDDDFEPYLSPQARPNNAYTAMSDSYLPSYY
SPSIGFPYSLGEAAWSTGGDTAMPYLTSYGQLSNGEPHFLPDAMFGQPGALGSTPFLGQH
GFNFFPSGIDFSAWGNNSSQGQSTQSSGYSSNYAYAPSSLGGAMIDGQSAFASETLNKAP
GMNTIDQGMAALKLGSTEVASNVPKVVGSAVGSGSITSNIVASNSLPPATIAPPKPASWA
DIASKPAKQQPKLKTKNGIAGSSLPPPPIKHNMDIGTWDNKGPVAKAPSQALVQNIGQQS
TQGSPQPVGQQANNSPPVAQASVGQQTQPLPPPPPQPAQLSVQQQAAQPTRWVAPRNRGS
GFGHNGVDGNGVGQSQAGSGSTPSEPHPVLEKLRSINNYNPKDFDWNLKHGRVFIIKSYS
EDDIHRSIKYNIWCSTEHGNKRLDAAYRSMNGKGPVYLLFSVNGSGHFCGVAEMKSAVDY
NTCAGVWSQDKWKGRFDVRWIFVKDVPNSQLRHIRLENNENKPVTNSRDTQEVPLEKAKQ
VLKIIASYKHTTSIFDDFSHYEKRQEEEESVKKERQGRGK

>Dasypus novemcinctus_YTHDF2(ENSDNOP_29952)
MSASSLLEQRPKGQGNKVQNGSVHQKDGLNDDDFEPYLNPQARPNNAYTAMSDSYLPSYY
SPSIGFSYSLGEAAWSTGGDTAMPYLTSYGQLGNGEPHFLPDAMFGQPGALGSTPFLGQH
GFNFFPSGIDFSAWGNNSSQGQSTQSSGYSSNYAYAPSSLGGAMIDGQSAFANETLNKAP
GMNTIDQGMAALKLGSTEVASSVPKVVGSAVGSGSITSNIVASSSLPPATIAPPKPASWA
DIASKPAKQQPKLKTKNGIAGSSLPPPPIKHNMDIGTWDNKGPVTKAPSQALVQNIGQQP
TQSSPQPVGQQANNSPPVAQASVGQQTQPLPPPPPQPAQLSVQQQAAQPTRWVAPRNRGS
GFGHNGVDGNGVGQSQAGSGSSSSEPHPVLEKLRSINNYNPKDFDWNLKHGRVFIIKSYS
EDDIHRSIKYNIWCSTEHGNKRLDAAYRSMNGKGPVYLLFSVNGSGHFCGVAEMKSAVDY
NTCAGVWSQDKWKGRFDVRWIFVKDVPNSQLRHIRLENNENKPVTNSRDTQEVPLEKAKQ
VLKIIASYKHTTSIFDDFSHYEKRQEEEESVKKVISIPLRFFREGGGL

>Myotis lucifugus_YTHDF2(ENSMLUP_11473)
MSASSLLEQRPKGQGNKVQNGSVHQKDGLNDDDFEPYLSPQARPNNAYTAMSDSYLPSYY
SPSIGFSYSLGEAAWSTGGDTAMPYLTSYGQLSNGEPHFLPDAMFGQPGALGSTPFLGQH
GFNFFPSGIDFSAWGNNSSQGQSTQSSGYSSNYAYAPSSLGGAMIDGQSAFASETLNKAP
GMNTIDQGMAALKLSSTEVASNVPKVVGSAVGSGSITSNIVASNSLPPATIAPPKPASWA
DIASKPAKQQPKLKTKNGIAGSSLPPPPIKHNMDIGTWDNKGPVAKAPSQALVQNIGQQP
TQGSPQPVGQQANNSPPVAQASVGQQTQPLPPPPPQSAQLSVQQQAAQPTRWVAPRNRGS
GFGHNGVDGNGVGQSQAGSGSTPSEPHPVLEKLRSINNYNPKDFDWNLKHGRVFIIKSYS
EDDIHRSIKYNIWCSTEHGNKRLDAAYRSMNGKGPVYLLFSVNGSGHFCGVAEMKSAVDY
NTCAGVWSQDKWKGRFDVRWIFVKDVPNSQLRHIRLENNENKPVTNSRDTQEVPLEKAKQ
VLKIIASYKHTTSIFDDFSHYEKRQEEEESVKKNNKSFNKSSGNN

>Monodelphis domestica_YTHDF2(ENSMODP_13732)
MSASSLLEQRPKGQGNKVQNGSVHQKDGLNDDDFEPYLSPQARPNNAYTAMSDSYLPSYY
SPSIGFSYSLGEAAWSTGGDPPMPYLTSYGQLSNGEPHFLPDAMFGQPGALGSTPFLGQH
GFNFFPSGIDFSAWGNNSSQGQSTQSSGYSSNYAYAPSSLGGAMIDGQSAFANETLNKAP
GMNTIDQGMAALKLGSTDVTSNVPKVVGSAVGSGSITSNIVASNSLPPATIAPPKPTSWA
DIASKPAKQQPKLKTKNGMAGSSLPPPPIKHNMDIGTWDNKGPVAKAPSQALVQNIGQQP
TQVSPQPVGQQINNSPPVVQAAAGQQPQPLPPPPPPQPTQLSVQQQAAQPTRWVAPRNRG
NGFGHNGVDGNGVGQSQNNSGSAPSEPHPVLEKLRSINNYNPKDFDWNLKHGRVFIIKSY
SEDDIHRSIKYNIWCSTEHGNKRLDAAYRSMNGKGPVYLLFSVNGSGHFCGVAEMKSAVD
YNTCAGVWSQDKWKGRFDVRWIFVKDVPNSQLRHIRLENNENKPVTNSRDTQEVPLEKAK
QVLKIIASYKHTTSIFDDFSHYEKRQEEEENVKKERQGRVK

>Gallus gallus_YTHDF2(ENSGALP_1412)
MSDSYLPNYYSPSIGFSYSLGEAAWSTGGDPPMPYLTSYGQLSNGEPHFLPDAMFGQPGA
LGSTPFLGQHGFNFFPSGIDFSAWGNNSSQGQSTQSSGYSSNYAYAPSSLGGAMIDGQSA
FASETLNKAPGMNTIDQGMAALKLGSTDVASNVPKVVGSAVGSGSITSNIVASNSLPPAT

IAPPKPTSWADIASKPAKQQPKLKTKNGIAGSSLPPPPIKHNMDIGTWDNKGPVAKAPSQ
TLVQNMAQQPPQASPQPVAQQMNSSPPVAQAPGGQQSQPLPPPPPQPTPLPVPQPAAQPT
RWVAPRNRGSGFGQNGTEGGGVGAAQPSTGTAPSEPHPVLEKLRSINNYNPKDFDWNPKH
GRVFIIKSYSEDDIHRSIKYNIWCSTEHGNKRLDAAYRSMNGKGPVYLLFSVNGSGHFCG
VAEMKSAVDYNTCAGVWSQDKWKGRFDVRWIFVKDVPNSQLRHIRLENNENKPVTNSRDT
QEVPLEKAKQVLKIIATYKHTTSIFDDFSHYEKRQEEEETVKKEPHGGFGMQGGSEEDVI
AGGFY

>Anolis carolinensis_YTHDF2(ENSACAP_10650)
MSASSLLEQRPKGQGTKVQNGSVHQKDGLNDDDIEPYLSPQARPSNAYTAMSDSYLPNYY
SPSIGFSYSLGEAAWSTGSDPPMPYLTSYGQLSNGEHHFLPEGMFGQPGALGSTPFLGQH
GFNFFPSGIDFSAWGNNSSQGQSTQSSGYSSNYAYAPSSLGGAMIDGQSAFSSETLNKAP
GMNTIDQGMAALKLGSTDVASNVPKVVGSAVGSGSMAGNIVASNSLPPATIAPPKPASWA
DIASKPAKQQPKLKTKNGIAGSSLPPPPIKHNMDIGTWDNKGPVAKNPSQGLVQNIGQPP
TQISPQPLGQQMSNSPPAVQPSTGQPPQPLPPPPTPQPTQLPVQQQAAQPARWVAPRNRG
NGFGQNGVDGTAVGQSPAASGSAPLEPHPVLEKLRSINNYNPKDFDWNPKHGRVFIIKSY
SEDDIHRSIKYNIWCSTEHGNKRLDAAYRSMNGKGPVYLLFSVNGSGHFCGVAEMKSAVD
YNTCAGVWSQDKWKGRFDVRWIFVKDVPNSQLRHIRLENNENKPVTNSRDTQEVPLEKAK
QVLKIIATYKHTTSIFDDFSHYEKRQEEEENVKKERQGRVK

>Danio rerio_YTHDF2(ENSDARP_107692)
MSASSLLEQRPKGQANKVQNGAVTQKDTLNDDEFEPYLNAQPRQSNAYTAMSDSYMPSYY
SPSIGFTYSLNEAAWSTGGDPPMPYLASYGQLSNGEHHFLPDAMFGQSGALGNNPFLGQH
GFNFFPSGIDFPAWGNSSSQGQSTQSSGYSSSYAYAPSTLGGAMIDGQSPFAANEPLNKA
VGMNSLDQGMAGLKIGAGDMAPKVVGSGLPGGPLSQVSTAPTMPPASMAPAKTASWADIA
SKPAKPQPKLKTKGGLGGTNLPPPPIKHNMDIGTWDNKGNMPKPAAPQQTSLPTNGQPPN
QSSPQPGATAGGVPQLPLSNGQLVPPTGQLVQHPLPPGGQPGAVPPQLSQGPPASQPSQP
TRWVPPRNRANGFGDAAGGPGQSPPNSGMGGITVPAEPHPVLEKLRMVNNYNPKDFDWNP
KHGRVFIIKSYSEDDIHRSIKYNIWCSTEHGNKRLDAAYRSLANKGPLYLLFSVNGSGHF
CGVAEMRSPVDYNTCAGVWSQDKWKGRFDVRWIFVKDVPNSQLRHIRLENNENKPVTNSR
DTQEVPLDKARQVLKIIASYKHTTSIFDDFSHYEKRQEEEESVKKVEVQGSDPYSNNSSR
SHYRMQDRQGRVK

>Takifugu rubripes_YTHDF2a(ENSTRUP_30170)
MSASSLLEQRPKGQANKVQNGAVTQKDTLNDDEFEPYLSTQARQSNAYTAMSDSYMPSYY
SPSVGFSYSLNEAAWSTGGDPPMPYLASYGQLSNGEPHYLPDAMFGQPGPLGSNPFLNQH
GFNFFPGGIDFSAWGNSSSQGQSGTPQSSGYSSSYAYAPSSLGGAMIDGQSPFAPGANEP
LNKAPGMNSLDQGMAGLKLGGAAPGGNGDMAPKVVGSGLPGAGPLGPVSSVGTPSMPPVS
IATAKPASWADIASKPAKPQPKLKTKGGMAGANLPPPPIKHNMDIGTWDNKGTMPKAAAP
QQVPLNPSNGQPPIQVSPQPGATALGPHQLPPGQPGMVQIPQVPLSQGPPPPSQQQAPQP
SRWVAPRNRANGFGDPSGNGAGQSPPTSSGVAVLPGVPSEPHPVLEKLRMVNNYNPKDFD
WNPKQGRVFIIKSYSEDDIHRSIKYNIWCSTEHGNKRLDAAYRSLGAKGPLYLLFSVNGS
GHFCGVAEMRSPVDYNTSAGVWSQDKWKGRFDVRWIFVKDVPNSQLRHIRLENNENKPVT
NSRDTQEVPLDKARQVLKIIAGYKHTTSIFDDFSHYEKRQEEEECVKKEPRICAWRLEFG
RS

>Takifugu rubripes_YTHDF2b(ENSTRUP_30168)
MSASSLLEQRPKGQANKVQNGAVTQKDTLNDDEFEPYLSTQARQSNAYTAMSDSYMPSYY
SPSVGFSYSLNEAAWSTGGDPPMPYLASYGQLSNGEPHYLPDAMFGQPGPLGSNPFLNQH
GFNFFPGGIDFSAWGNSSSQGQSGTPQSSGYSSSYAYAPSSLGGAMIDGQSPFAPGANEP
LNKAPGMNSLDQGMAGLKLGGAAPGGNGDMAPKVVGSGLPGAGPLGPVSSVGTPSMPPVS
IATAKPASWADIASKPAKPQPKLKTKGGMAGANLPPPPIKHNMDIGTWDNKGTMPKAAAP
QQVPLNPSNGQPPIQVSPQPGATALGPHQLPPGQPGMVQIPQVPLSQGPPPPSQQQAPQP
SRWVAPRNRANGFGDPSGNGAGQSPPTSSGVAVLPGVPSEPHPVLEKLRMVNNYNPKDFD
WNPKQGRVFIIKSYSEDDIHRSIKYNIWCSTEHGNKRLDAAYRSLGAKGPLYLLFSVNGS
GHFCGVAEMRSPVDYNTSAGVWSQDKWKGRFDVRWIFVKDVPNSQLRHIRLENNENKPVT
NSRDTQEVPLDKARQVLKIIAGYKHTTSIFDDFSHYEKRQEEEECVKKYFKGGKYRYSHD
CRADVSSVS

>Gasterosteus aculeatus_YTHDF2(ENSGACP_12672)
MSASSLLDQRPKGQANKVQNGAVTQKDTLNDDEFEPYLNTQARQSNAYTAMSDSYMPSYY

SPSIGFSYSLNEAAWSTGGDPPMPYLASYGQLSNGEPHYLPDAMFGQPGPLGNNPFMGQH
GFNFFPSGIDFSAWGNNTSQGQSGTPQSSGYSSSYAYAPSSLGGAMIDGPSPFAPAANEP
LNKAPGMNSLDQGMAGLKIGGAAPGGNGDMAPKVVGSGLPGGGPHGPVTSVGPPSMPSVS
IAPPKPASWADIASKPAKPQPKLKTKGGMAGANLPPPPIKHNMDIGTWDNKGTMPKAATP
QQVPPIPSNGQPPNQASPQLASTAVGNPQMPLSNGQLGTPVSQMGQHQLLPGGQPGMGQM
PPLSQGPPPSQQQPSQPTRWVAPRNRANGFGDGSGSGTGQSPLTSSGVGVVPGVPSEPHP
VLEKLRMVNNYNPKDFDWNPKQGRVFIIKSYSEDDIHRSIKYNIWCSTEHGNKRLDAAYR
SLGAKGPLYLLFSVNGSGHFCGVAEMRSPVDYNTSAGVWSQDKWKGRFDVRWIFVKDVPN
SQLRHIRLENNENKPVTNSRDTQEVPLDKARQVLKIIAGYKHTTSIFDDFSHYEKRQEEE
ECVKKVTKSQLNKVNGI

>Oryzias latipes_YTHDF2(ENSORLP_17958)
MSASSLLEQRPKGQANKVQNGPVTQKDTLNDDEFEPYLNSQARLQSNAYTAMSDSYMPSY
YSPSIGFSYSLNEAAWSTGGDPPMPYLASYGQLSNGEPHYLPDAMFGQPGPLGSNPFLSQ
HGFNFFPSGIDFSAWGNSSSQGQSGSSGYSSSYAYPPSSLGGAMIDGQSPFAPAANEPIN
KAPGMNSLDQGMAGLKIGGATPGGNGEMAPKVVGSGLPGGGPLGPVSSVGPTSMPPVSIA
PVKPASWADIASKPAKPQPKLKTKGGMVGVNLPPPPIKHNMDIGTWDNKGTMPKAATPQQ
VPSVPTNGQPPSQASPQPGATAAGNPQMPLSNGQMVPPVSQMGQHQLPPTVQPGVAPMHQ
PPLSQGPPPPPPSQQQQPSQPNRWVPPRNRTNGFGDAGGSGTGHSPPSSGVGVVPGVPSE
PHPVLEKLRMVNNNYNPKDFDWNLKHGRVFIIKSYSEDDIHRSIKYNIWCSTEHGNKRLDG
AYRSLGGKGPLYLLFSVNGSGHFCGVAEMRSPVDYNTSAGVWSQDKWKGRFDVRWIFVKD
VPNSQLRHIRLENNENKPVTNSRDTQEVPLDKARQVLKIIAGYKHTTSIFDDFTHYEKRQ
EEEECVKKVKTFCHQVEVQGS

>Homo sapiens_YTHDF3(ENSP_473496)
MSATSVDQRPKGQGNKVSVQNGSIHQKDAVNDDDFEPYLSSQTNQSNSYPPMSDPYMPSY
YAPSIGFPYSLGEAAWSTAGDQPMPYLTTYGQMSNGEHHYIPDGVFSQPGALGNTPPFLG
QHGFNFFPGNADFSTWGTSGSQGQSTQSSAYSSSYGYPPSSLGRAITDGQAGFGNDTLSK
VPGISSIEQGMTGLKIGGDLTAAVTKTVGTALSSSGMTSIATNSVPPVSSAAPKPTSWAA
IARKPAKPQPKLKPKGNVGIGGSAVPPPPIKHNMNIGTWDEKGSVVKAPPTQPVLPPQTI
IQQPQPLIQPPPLVQSQLPQQQPQPPQPQQQQGPQPQAQPHQVQPQQQQLQNRWVAPRNR
GAGFNQNNGAGSENFGLGVVPVSASPSSVEVHPVLEKLKAINNYNPKDFDWNLKNGRVFI
IKSYSEDDIHRSIKYSIWCSTEHGNKRLDAAYRSLNGKGPLYLLFSVNGSGHFCGVAEMK
SVVDYNAYAGVWSQDKWKGKFEVKWIFVKDVPNNQLRHIRLENNDNKPVTNSRDTQEVPL
EKAKQVLKIIATFKHTTSIFDDFAHYEKRQEEEEAMRRERNRNKQ

>Gorilla gorilla_YTHDF3(ENSGGOP_27387)
QSNSYPPMSDPYMPSYYAPSIGFPYSLGEAAWSTAGDQPMPYLTTYGQMSNGEHHYIPDG
VFSQPGALGNTPPFLGQHGFNFFPGNADFSTWGTSGSQGQSTQSSAYSSSYGYPPSSLGR
AITDGQAGFGNDTLSKVPGISSIEQGMTGLKIGGDLTAAVTKTVGTALSSSGMTSIATNS
VPPVSSAAPKPTSWAAIARKPAKPQPKLKPKGNVGIGGSAVPPPPIKHNMNIGTWDEKGS
VVKAPPTQPVLPPQTIIQQPQPLIQPPPLVQSQLPQQQPQPPQPQQQQGPQPQAQPHQVQ
PQQQQLQNRWVAPRNRGAGFNQNNGAGSENFGLGVVPVSASPSSVEVHPVLEKLKAINNY
NPKDFDWNLKNGRVFIIKSYSEDDIHRSIKYSIWCSTEHGNKRLDAAYRSLNGKGPLYLL
FSVNGSGHFCGVAEMKSVVDYNAYAGVWSQDKWKGKFEVKWIFVKDVPNNQLRHIRLENN
DNKPVTNSRDTQEVPLEKAKQVLKIIATFKHTTSIFDDFAHYEKRQEEEEAMRRETNYLR
NNS

>Pongo abelii_YTHDF3(ENSPPYP_20890)
MSATSVDQRPKGQGNKVSVQNGSIHQKDAVNDDDFEPYLSSQTNQSNSYPPMSDPYMPSY
YAPSIGFPYSLGEAAWSTAGDQPMPYLTTYGQMSLSKVPGISSIEQGMTGLKIGGDLTAA
VTKTVGTALSSSGMTSIATNSVPPVSSAAPKPTSWAAIARKPAKPQPKLKPKGNVGIGGS
AVPPPPIKHNMNIGTWDEKGSVVKAPPTQPVLPPQTIIEQPQPLIQPPPLVQSQLPQQQP
QPPQPQQQQGPQPQAQPHQVQPQQQQLQNRWVAPRNRGAGFNQNNGAGSENFGLGVVPVS
ASPSSVEVHPVLEKLKAINNYNPKDFDWNLKNGRVFIIKSYSEDDIHRSIKYSIWCSTEH
GNKRLDAAYRSLNGKGPLYLLFSVNGSGHFCGVAEMKSVVDYNAYAGVWSQDKWKGKFEV
KWIFVKDVPNNQLRHIRLENNDNKPVTNSRDTQEVPLEKAKQVLKIIATFKHTTSIFDDF
AHYEKRQEEEEAMRRERNRNKQ

>Mus musculus_YTHDF3(ENSMUSP_103983)
MSATSVDQRPKGQGNKVSVQNGSIHQKDAVNDDDFEPYLSSQTNQKYRRAKQLFHCNNSY

PPMSDPYMPSYYAPSIGFPYSLGEAAWSTAGDQPMPYLTTYGQMSNGEHHYIPDGVFSQP
GALGNTPPFLGQHGFNFFPGNADFSTWGTSGSQGQSTQNSAYSSSYGYPPSSLGRAITDG
QAGFGNDTLSKVPGISSIEQGMTGLKIGGDLTAAVTKTVGTALSSSGMTSIATNNVPPVS
SAAPKPTSWAAIARKPAKPQPKLKPKGNVGIGGSAVPPPPIKHNMNIGTWDEKGSVVKAP
PTQPVLPPQTIIQQPQPLIQPPPLVQSQLPQQQPQPPQPQQQQGPQPQAQPHQVQSQQPQ
LQNRWVAPRNRGTGFNQNNGTGSENFGLGVVPVSASPSSVEVHPVLEKLKAINNYNPKDF
DWNLKNGRVFIIKSYSEDDIHRSIKYSIWCSTEHGNKRLDAAYRSLNGKGPLYLLFSVNG
SGHFCGVAEMKSVVDYNAYAGVWSQDKWKGKFEVKWIFVKDVPNNQLRHIRLENNDNKPV
TNSRDTQEVPLEKAKQVLKIIATFKHTTSIFDDFAHYEKRQEEEEAMRRERNRNKQ

>Rattus norvegicus_YTHDF3(ENSRNOP_60081)
MSATSVDQRPKGQGNKVSVQNGSIHQKDAVNDDDFEPYLSSQTNQNNSYPPMSDPYMPSY
YAPSIGFPYSLGEAAWSTAGDQPMPYLTTYGQMSNGEHHYIPDGVFSQPGALGNTPPFLG
QHGFNFFPGNADFSTWGTSGSQGQSTQNSAYSSSYGYPPSSLGRAITDGQAGFGNDTLSK
VPGISSIEQGMTGLKIGGDLTAAVTKTVGTALSSSGMTSIATNNVPPVSSAAPKPTSWAA
IARKPAKPQPKLKPKGSVGIGGSAVPPPPIKHNMNIGTWDEKGSVVKAPPTQPVLPPQTI
IQQPQPLIQPPPLVQSQLPQQQPQPPQPQQQQGPQPQAQPHQVQSQQPQLQNRWVAPRNR
GTGFNQNNGTGSENFGLGVVPVSASPSSVEVHPVLEKLKAINNYNPKDFDWNLKNGRVFI
IKSYSEDDIHRSIKYSIWCSTEHGNKRLDAAYRSLNGKGPLYLLFSVNGSGHFCGVAEMK
SVVDYNAYAGVWSQDKWKGKFEVKWIFVKDVPNNQLRHIRLENNDNKPVTNSRDTQEVPL
EKAKQVLKIIATFKHTTSIFDDFAHYEKRQEEEEAMRRERNRNKQ

>Canis familiaris_YTHDF3(ENSCAFP_10721)
MSATSVDQRPKGQGNKVSVQNGSIHQKDAVNDDDFEPYLSSQTNQSNSYPPMSDPYMPSY
YAPSIGFPYSLGEAAWSTAGDQPMPYLTTYGQMSNGEHHYIPDGVFSQPGALGNTPPFLG
QHGFNFFPGNADFSTWGTSGSQGQSTQSSAYSSSYGYPPSSLGRAITDGQAGFGNDTLSK
VPGISSIEQGMTGLKIGGDLTAAVTKTVGTALSSSGMTSIATNSVPPVSSAAPKPTSWAA
IARKPAKPQPKLKPKGNVGIGGSAVPPPPIKHNMNIGTWDEKGSVVKAPPTQPVLPPQTI
IQQPQPLIQPPPLVQSQLPQQQPQPPQPQQQQGPQPQAQPHQVQPQQQQLQNRWVAPRNR
GAGFNQNNGVGGENFGLGVVPVSASPSNVEVHPVLEKLKAINNYNPKDFDWNLKNGRVFI
IKSYSEDDIHRSIKYSIWCSTEHGNKRLDAAYRSLNGKGPLYLLFSVNGSGHFCGVAEMK
SVVDYNAYAGVWSQDKWKGKFEVKWIFVKDVPNNQLRHIRLENNDNKPVTNSRDTQEVPL
EKAKQVLKIIATFKHTTSIFDDFAHYEKRQEEEEAMRRESETNSF

>Monodelphis domestica_YTHDF3(ENSMODP_10254)
MSATSVDQRPKGQGNKVSVQNGSIHQKDAVNDDDFEPYLSSQTNQSNSYPPMSDPYMPSY
YAPSIGFPYSLGEAAWSTAGDPPMPYLTTYGQMSNGEHHYIPDGVFSQPGALGNTPPFLG
QHGFNFFPGNADFSTWGTSGSQGQSTQSSAYSSSYGYPPSSLGRAITDGQAGFGNDTLSK
VPGINSIEQGMTGLKIGGDMTAAVTKTVGSALSSTGMTSIAANSVPPVSSSAPKPTSWAA
IARKPAKPQPKLKPKGNVGIGGPAVPPPPIKHNMNIGTWDDKGSVVKAPPTQPVLPPQTI
IQQPQPLIQPPPLVQSQLPQQQPQPPQPQQQQGPQPQAQPHQVQQQQPQQLQNRWVAPRN
RGVGFNQNNGAGSENFGLGVVPVSSSPSGVEVHPVLEKLKAINNYNPKDFDWNLKNGRVF
IIKSYSEDDIHRSIKYSIWCSTEHGNKRLDAAYRSLNGKGPLYLLFSVNGSGHFCGVAEM
KSVVDYNAYAGVWSQDKWKGKFDVKWIFVKDVPNNQLRHIRLENNDNKPVTNSRDTQEVP
LEKAKQVLKIIATFKHTTSIFDDFAHYEKRQEEEEAMRRERNRNKQ

>Gallus gallus_YTHDF3(ENSGALP_24954)
FSLLSYVQRPKGQGNKVSVQNGSIHQKDAVNDDDFEPYLSSQTNQSNSYPPMSDPYMPSY
YAPSIGFPYSLGEAAWSTAGDPPMPYLTTYGQMSNGEHHYIPDGVFSQPGALGNTPPFLG
QHGFNFFPGNADFSTWGTSGSQGQSTQSSAYSSSYGYPPSSLGRAIADGQAGFGSDTLSK
VPGISSIEQGMTGLKIGGDMTAAVTKTVGSALSSTGMTSIAANSVPPVSSSAPKPTSWAA
IARKPAKPQPKLKPKGNVGIGGPAVPPPPIKHNMNIGTWDDKGSVVKAPPAQPVLPPQTI
IQQPQPLIQPPTLVQSQLPQQQPQPQQPQQQQGPQQQAQPHQLQQQQLQNRWVAPRNRGV
GFSQNNGAGSENFGLGVVSVSSSPSGVEVHPVLEKLKAINNYNPDFDWNLKNGRVFIIKS
YSEDDIHRSIKYSIWCSTEHGNKRLDAAYRSLNGKGPLLLFSVNGSGHFCGVAEMKSVVD
YNAYAGVWSQDKWKGKFDVKWIFVKDVPNNQLRHIRLENNDNKPVTNSRDTQEVPLEKAK
QVLKIIATFKHTTSIFDDFAHYEKRQEEEEAMRRVRNQYYS

>Pelodiscus sinensis_YTHDF3(ENSPSIP_17846)
MSATSVDQRPKGQGNKVSVQNGSIHQKDAVNDDDFEPYLSSQTNQSNSYPPMSDPYMPSY
YAPSIGFPYSLSEAAWSTAGDPPMPYLTTYGQMSNGEHHYIPDGVFSQPGALGNTPPFLG

QHGFNFFPGNADFSTWGTSGSQGQSTQSSAYSSSYGYPPSSLGRAIADGQAGFGNDTLSK
VPGINSIEQGMTGLKIGGDMTAAVTKTVGSALSSTGMTSIAANSVPPVSSSAPKPTSWAA
IARKPAKPQPKLKPKGNVGIGGPAIPPPPIKHNMNIGTWDDKGSVVKAPPSQPILPPQTI
IQQPQPLIQPPPLVQSQLPQQQPQPPQQQQGPQQQAQNHQLQQQQQLQNRWVAPRNRGV
GFSQNNGGSGSENFGLGVVPVSSSPSGVEVHPVLEKLKAINNYNPKDFDWNLKNGRVFII
KSYSEDDIHRSIKYSIWCSTEHGNKRLDAAYRSLNGKGPLYLLFSVNGSGHFCGVAEMKS
VVDYNAYAGVWSQDKWKGKFDVKWIFVKDVPNNQLRHIRLENNDNKPVTNSRDTQEVPLE
KAKQVLKIIATFKHTTSIFDDFAHYEKRQEEEEAMRRERNRNKQ

>Xenopus tropicalis_YTHDF3(ENSXETP_46817)
MSATSVDQRPKGQGNKVAVQNGSMHQKDAVNDDDFEPYLNSQTNQSNSYPPMSDPYMPSY
YAPSIGFPYSLGEAAWSTAGDPPMPYLYGQMSNGEHHYIPDGVFSQPGALGNTPPFLSQH
GFNFFPGNADFSTWGTSGSQGQSTQSSAYSSSYGYPPSSLGRAIADGQAGFGSDALSKVP
GINSIEQGMTGLKIGGDMTAAVTKTVGSALSSAGMTSIAANSVPPVSSSAPKPTSWAAIA
RKPAKPQPKLKPKNNVGIGSSAVPPPPIKHNINIGTWDDKGAIVKTPPIQSVLPPQPVIQ
QPQPLTQPLPMVQNQLPQQQLQQQGPQQPPHQIQQQLQNRWVAPRNRGAGFNQNNVSGNE
NFSLGVVPVSSSPSGVEVHPVLEKLKAINNYNPKDFDWSLKNGRVFIIKSYSEDDIHRSI
KYTIWCSTEHGNKRLDAAYRSLNGKGPLYLLFSVNGSGHFCGVAEMKSVVDYNAYAGVWS
QDKWKGKFDVKWVFVKDVPNNQLRHIRLENNDNKPVTNSRDTQEVPLEKAKQVLKIIATF
KHTTSIFDDFAHYEKRQEEEEAMRRERNRNKQ

>Takifugu rubripes_YTHDF3(ENSTRUP_19572)
MSATTVDQRPKGQGNKAPVQNGSMHQKDGVNDDDFEPYISGQTNQNNSYPPMSDPYMPSY
YAPSIGFPYSLGEAAWSTAGDPPMPYLTTYGQMSNGEPHFIPDGLFSQPGALGNTPPFLG
QHGFNFFPGNADFSTWGTSVSQGQSTQSSVYSNSYGYAPSSLGRAIADGQAGFGSDTQLS
KVPVLNSIEQGMTGLKLGTEMVSAVTKTVGSPIGGTAGMSSLATNSLPPSVSSSTPKPAS
WAAIAKKPAKLQPKVKPKANMGMVGGATIPPPPIKHNFNIGTWDDKGSLNKPPLAQTMLP
PQPLVQQPLLAQHQPLLQNPLPHQPQHQHQHQPQPPGPPLHHQHPQQPGPPPNRWVAP
RNRGEGFCLGGGVPLSASLCSGELHPMLEKLQALNNYNPKDFDWSLKNGRIFIIKSYSED
DIHRSIKYSIWCSTEHGNKRLDGAYRSLGNKGPLYLLFSVNGSGHFCGVAEMRSPVDYNA
YAGVWSQDKWKGKFEVKWIFIKDVPNNQLRHIRLENNDNKPVTNSRDTQEVPLEKAKQVL
KIIATYKHTTSIFDDFAHYEKRQEEEEILRKERNRNKQ

>Tetraodon nigroviridis_YTHDF3(ENSTNIP_15673)
MSATTVDQRPKGQGNKAPVQNGSMHQKDGVNDDDFEPYISGQTNQNSSYPPMSDPYMPSY
YAPSIGFPYSLGEAAWSTAGDPPMPYLTTYGQMSNGEPHFIPDGVFGQPGTLGNTPPFLG
QHGFNFFPGNADFSTWGTSVSQGQSTQSSVYSNSYGYAPSSLGRAIADGQAGFGSDSQLS
KVPVLNSIEQGMAGLKLGTEMVSAVTKTVGSPVGGTAGMSSLAASGLPPSISSSAPKPAS
WAAIAKKPAKLQPKVKPKANMVGGAAIPPPPIKHNLNIGTWDDKGSLNKPPLAQTMLPPQ
PLVQQPLLAQHQPLLQNPLPHHHSLPPGPPHLQLSSQAGPPLHQQHPQQAGPPPNRWVAP
RNRGEGFCLGGGVPLSASPCSGEVHPVLEKLQALNNYNPKDFDWSLKNGRVFIIKSYSED
DIHRSIKYSIWCSTEHGNKRLDGAYHSLGSKGPLYLLFSVNGSGHFCGVAEMRSPVDYNA
FAGVWSQDKWKGKFEVKWIFIKDVPNNQLRHIRLENNDNKPVTNSRDTQEVPLEKAKQVL
RIIATYKHTTSIFDDFAHYEKRQEEEEVLRKERNRNKQ

>Gasterosteus aculeatus_YTHDF3(ENSGACP_3924)
MSATTVDQRPKGQGNKVQNGSMHQKDGVNDDDFEPYLSGQTNQGNSYPPMSDPYMPSYYA
PSIGFPYSLGEAAWSTAGDPPMPYLTTYGQMSNGEPHFIPDGVFSQAGALGNTPPFLGQH
GFNFFPGNADYSTWGTSVSQGQSTQSSVYSNSYGYAPSSLGRAITDGQAGFGSDTQLSKV
PVLNSIEQGMTGLKLGTDMVAAVTKTVGSPLGGTAGMSSMAANSLPPSVNSSAPKPASWA
AIAKKPAKPQPKVKPKANMGLGGGAIPPPPIKHNMNIGTWDEKGSLNKPPLAQNMMPPQP
MVQQPLLAQPPALTAKPHCLLRLHQPFQLHSLQSPQHPQPMPPGHPHMHLSSQPGPPQHL
HQQPPQQPGPPPNRWVAPRNRGEGFGLGGGLPLSASPCSGEVHPVLEKLRALNNYNPKDF
DWSLKNGRVFIIKSYSEDDIHRSIKYSIWCSTEHGNKRLDGAYRSLGNKGPLYLLFSVNG
SGHFCGVAEMRSPVDYNAYAGVWSQDKWKGKFEVKWAFIKDVPNNQLRHIRLENNDNKPV
TNSRDTQEVPLEKAKQVLKIIATYKHTTSIFDDFAHYEKRQEEEEALRKERNRNKP

>Drosophila melanogaster_(FBpp0084170)
MRFSLFTMPHNAAKSVEERNIPWSQQVDEASYENLSSPTHDEVSGNDNSMQFQYPPFNFK
ENCNTPWNNMNKGRKANANHDSYRRHGHSIPNNTRRDEHQVNPWNSRKPAAQSFRNENDQ
PSTKLDNRTSDEAQNQEVVAAPKKTTWASIASQPAKLTSRAASTTSNSKKKGPGMPPPPM

VPGKHNLDVNVWDLPNNKPPPVPSPPSPIDLGYSDLSSDFSISSNTAPPLGARAEKVHKD
TENFLKYEHKGLGNNNNFSRAKVSPTGPVRRNLGPQQPVHHAAPRPATNAGPFGPPNARR
HDGGPHPSRNSERSGNYSSFRGSEFESASKFEYRDENQSRPVEATSATEELPVDSQLVLD
ELKDKNNYNPKVLDLKKAGSARFFVIKSYSEDDIHRSIKYEIWCSTDHGNKRLDDAFKER
HEEGGNIMLFFSVNGSGHFCGMAQMMTPVDYNSTSSVWSQDKWRGKFKVKWIYVKDVPNG
TLRHIRLENNENKSVTNSRDTQEVPNDKGIEVLQILHSYNHSTSIFDDFFHYEKKQEEEV
SSKRPPMHGPDGNNHAPAPLARSFNRQADDKDRDRDGRGGMSQKHFNSGGGAGFRNPGHR
GGAGSGGSHNNYSEYRRGFDIHKSSSDYQFKDRDGPDFDRENDFGSHYGSNNRKNEYKHN
NTNKARLKTRDRDFSTEQIKIGVRFKDTDKDKMRSNEYS

>Anopheles gambiae_(XP_310378)
MPGNGDYNSWSAQVNHGPGGVGHQKKSDDGYYRNHGAYQSHDSVRNVERGMQGLGLGSASRSGNSEGNSG
VSKYYQQQQQQKEAPKKMTWATIASQPAKPQVNTTSTTVKKKGPGMPPPPMIPGKHNMDIGTWDSPSKNG
PSVIVPTPPPIIPPPVIEPSPLADPVPSKSGGGRESVGGASSQHHGSNSQGMGPAGIQSSRWPTPGQAQI
QQQQPSGTPSSSDRNNGPSAGTNSGSGGSGSNAASGGSMPSGSQHHNQHHQHQQGSHTRNHSSPYQPPQQ
QQPHHADRPGSNYDRAPHHQHQQGGLQHGNSYNQSRNQNAAYSPSGPGGLAATGPSSAMADRQNSGGSGG
DNYRPPHDGRSGPQQHNSMHHHQQTERAPFHHQQRPSNHHGDRVSGGSSYQPADRGHYGSNSSSDAVSHN
SSSTVPPVTTAPAAVKARSPSPTISESEMALKLAESQKILDQLKTKNNYNPASLDLLKTVDLARFFVIKS
YSEDDIHRSIKYEIWCSTEHGNQRLDQAFREREEKGGTVYLFFSVNGSGHFCGVAQMMTAVDYNSNSSVW
SQDKWKGTFKVRWIYVKDVPNSHLRHIRLENNENKSMTNSRDTQEVPNAKGIQVLQIIHSFEHQSSIFDD
FQHYEKRQMEEDTRKHEAPPPSNHSYHNQRDRRENQGGGNYYDGPGKYHGGGGGGGGYNNKYNDRGGDDG
YDGGYGSRGGGGGYHGGYNKGGSSGGYGGYNNRNSYNNRDDPGRGYPSFDRRNNNGNSGGSNNNGDDQHD
AAAPGGGGGGGGYNSRGGYNNRSRDYYRDERRGPNDYGRARGSDFDNGRDSDGGGIYRSGDRGDRGDRGDR
GERGERGDRGDRGDRGDRGDRGDRGDRGDRDRGSSRDHFRDRNDRMMGPGNNRGGSGSNGSGSNIFRNPN
