# Supplementary material for: Evolutionary history of the human multigene families reveals widespread gene duplications throughout the history of animals
Source: BMC Evol Biol. 2019 Jun 20;19:128. doi: 10.1186/s12862-019-1441-0 (PMC6585022; doi:10.1186/s12862-019-1441-0)
Supplement: Supplementary file 2 — Neighbor Joining Trees of gene families (residing on human chromosomes 1/2/8/20) using p-distance method. (PDF 4993 kb) [file 12862_2019_1441_MOESM2_ESM.pdf]

Additional file 2: Neighbor Joining Trees of gene families (residing on human chromosomes 1/2/8/20) using p-distance method.

Co-duplicated group-1

MROH8 and STK

Maestro Heat-like Repeat-containing Protein Family -MROH

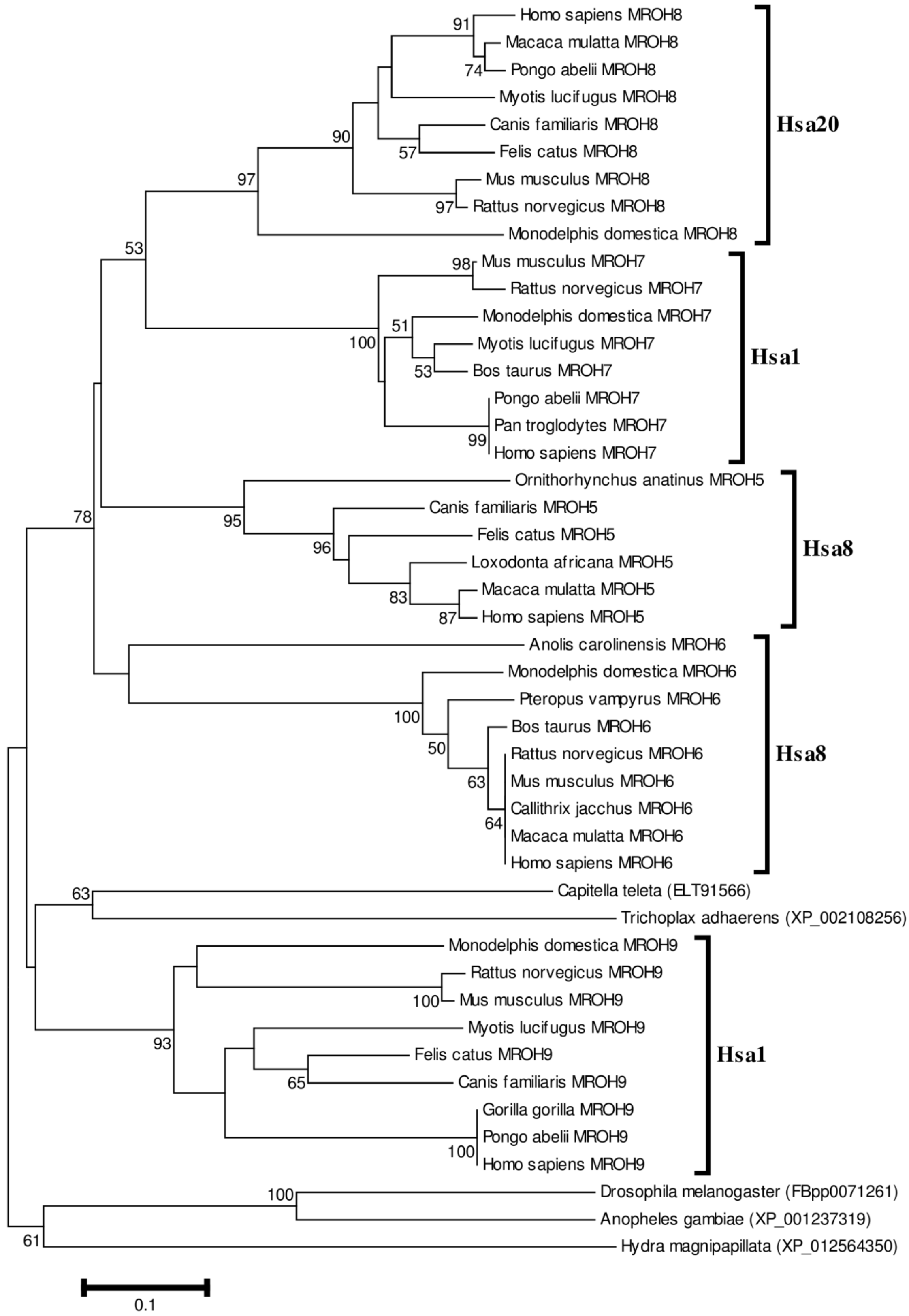

Phylogenetic tree of MROH family.

Neighbor-Joining tree of the MROH family members. Symbols and parameters are the same as described in supplementary Figure 1.1

Serine/Threonine-Protein Kinase-STK

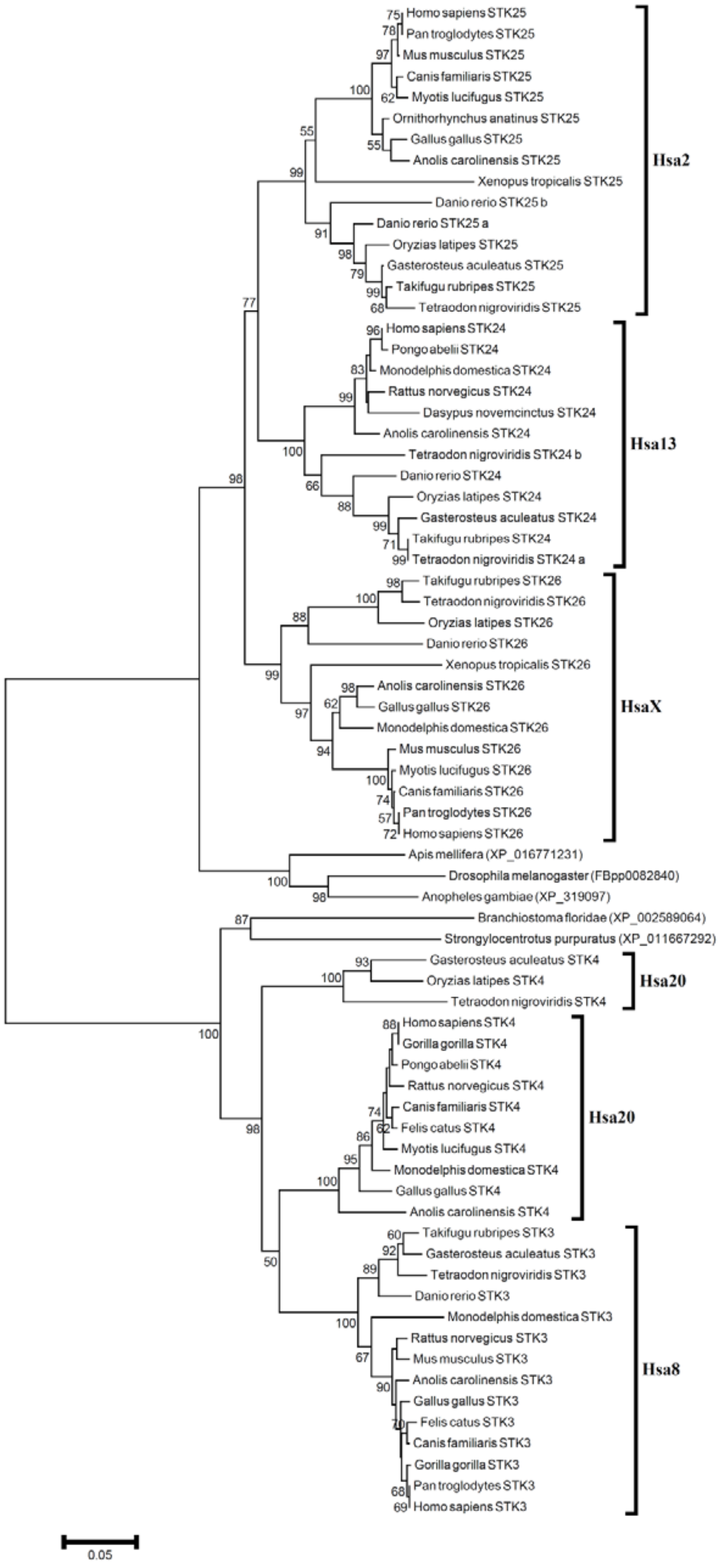

Phylogenetic tree of STK family.

Neighbor-Joining tree of the STK family members. Symbols and parameters are the same as described in supplementary Figure 1.1

Co-duplicated group-2

E2F, STMN1, EYA*

Asterisks (*) represent families published previously by our research group (for details see main text).

E2F Transcription Factor-E2F

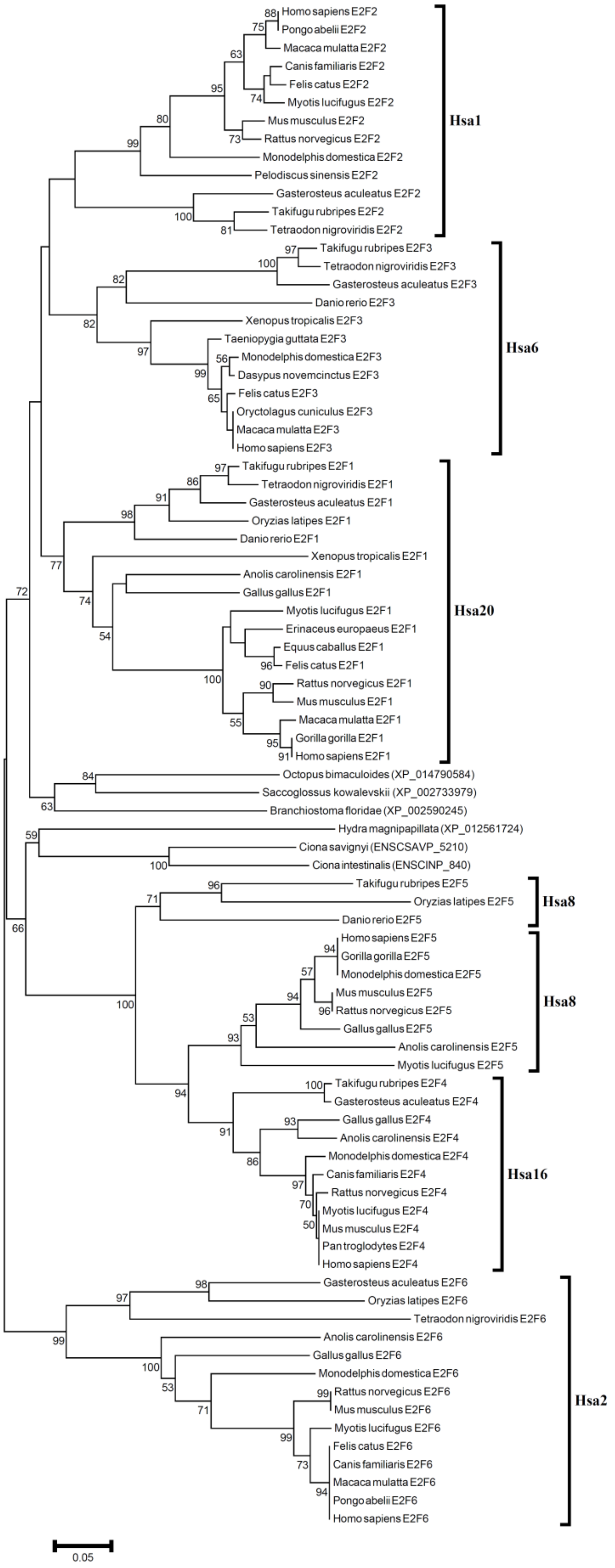

Phylogenetic tree of E2F family.

Neighbor-Joining tree of the E2F family members. Symbols and parameters are the same as described in supplementary Figure 1.1

Stathmin-STMN

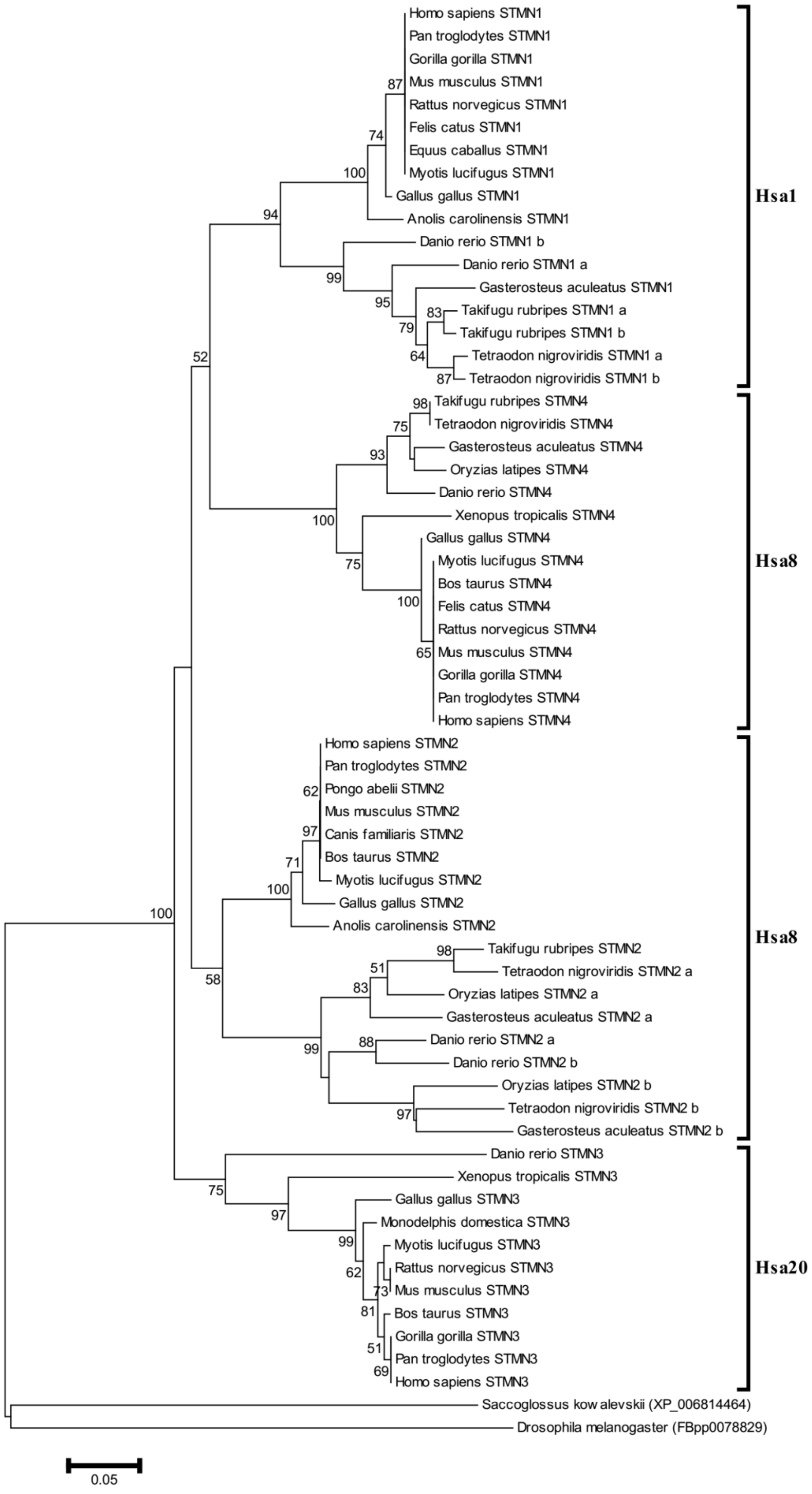

Phylogenetic tree of STMN family.

Neighbor-Joining tree of the STMN family members. Symbols and parameters are the same as described in supplementary Figure 1.1

Co-duplicated group-3

HCK*, DLGAP, NKAIN, KCNQ, MATN4*

Asterisks (*) represent families published previously by our research group (for details see main text).

Discs, large Drosophila Homolog-associated Protein-DLGAP

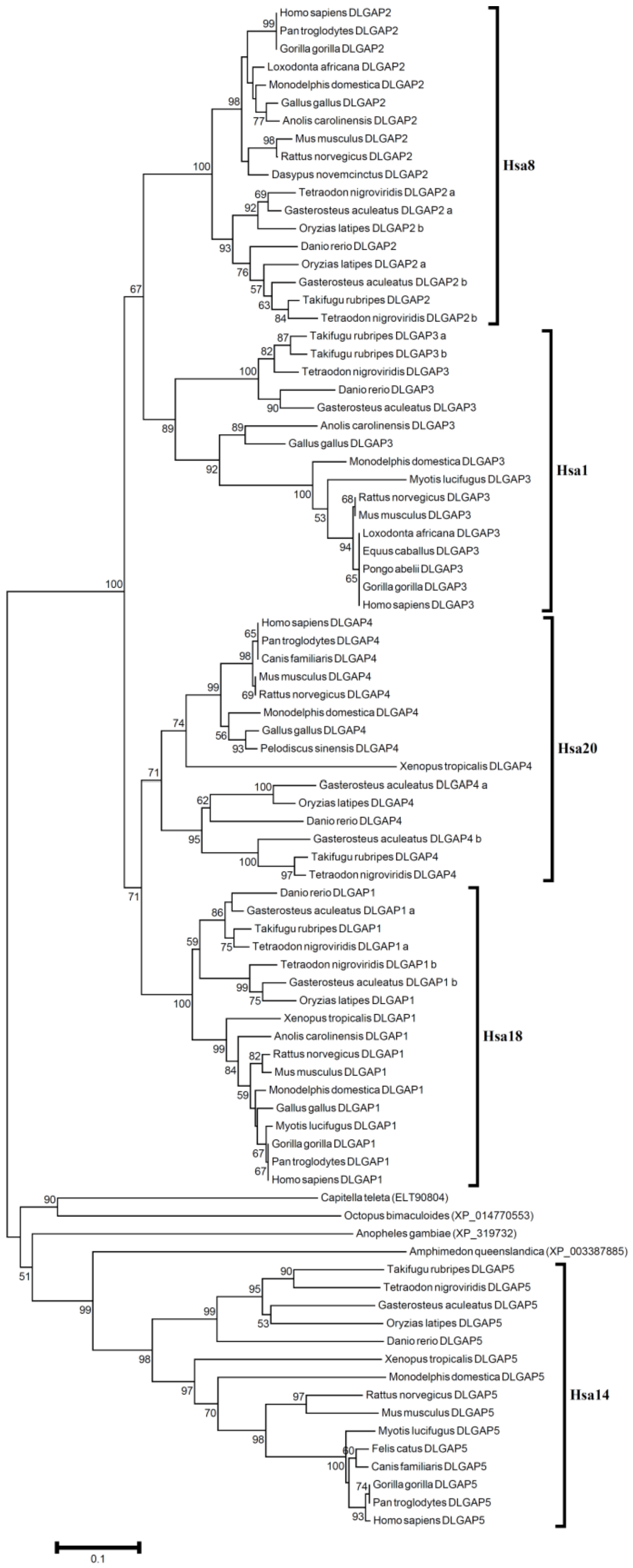

Phylogenetic tree of DLGAP family.

Neighbor-Joining tree of the DLGAP family members. Symbols and parameters are the same as described in supplementary Figure 1.1

Na+/K+ Transporting ATPase Interacting Protein-NKAIN

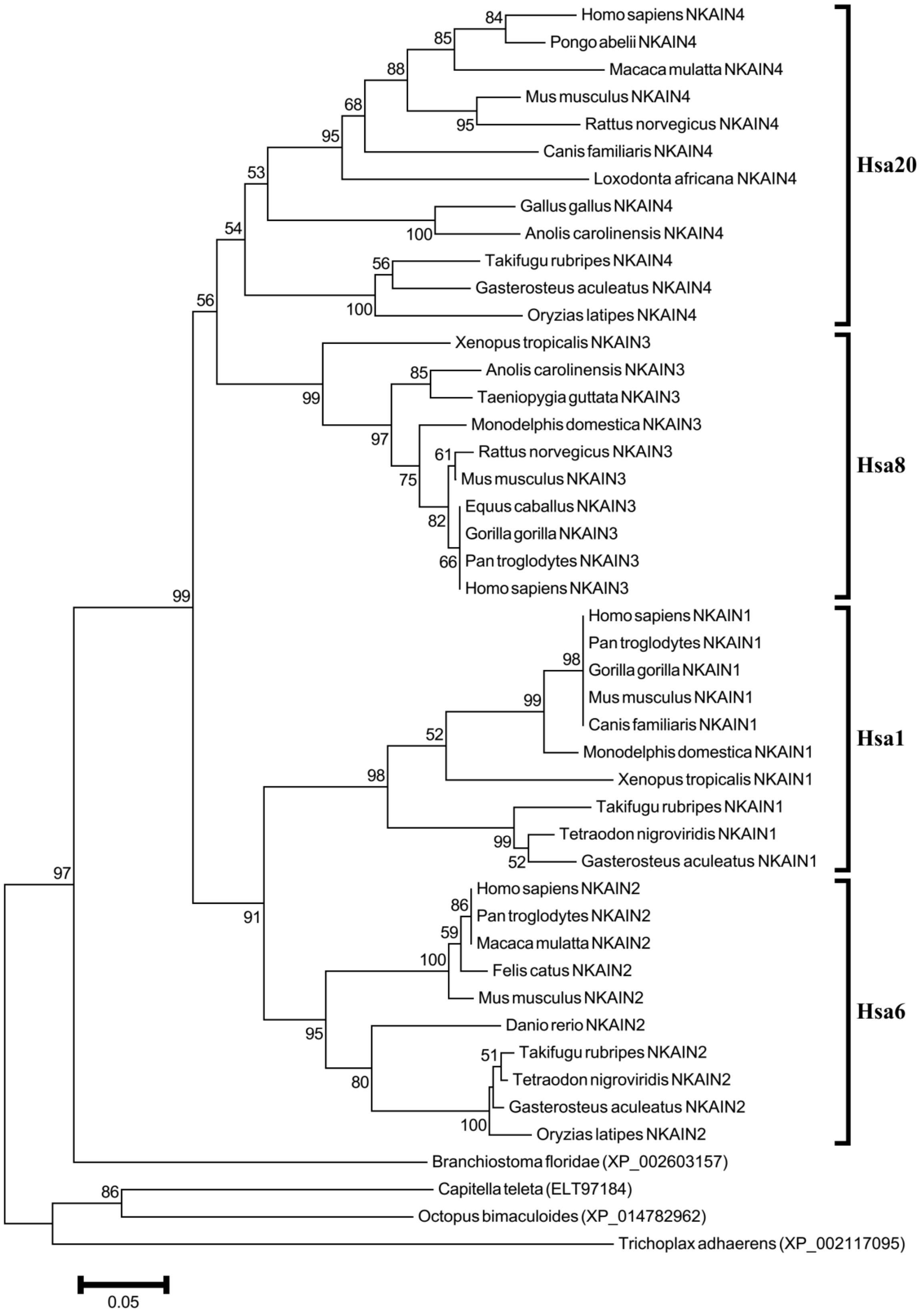

Phylogenetic tree of NKAIN family.

Neighbor-Joining tree of the NKAIN family members. Symbols and parameters are the same as described in supplementary Figure 1.1

Potassium Voltage-Gated Channel subfamily Q-KCNQ

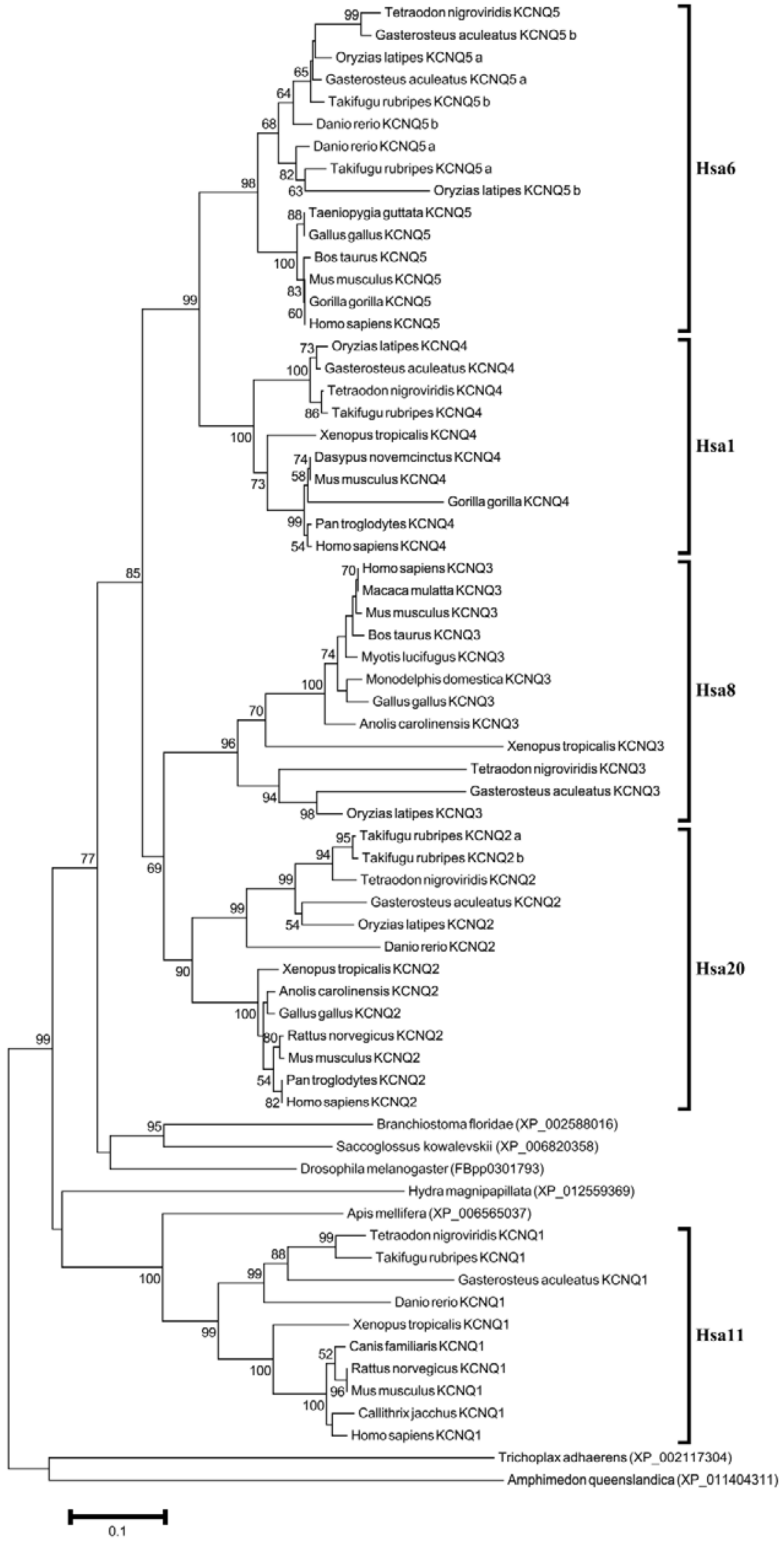

Phylogenetic tree of KCNQ family.

Neighbor-Joining tree of the KCNQ family members. Symbols and parameters are the same as described in supplementary Figure 1.1

Co-duplicated group-4

FAM110, NCOA, KCNS*, YTHDF, XKR, MYT

Asterisks (*) represent families published previously by our research group (for details see main text).

Family with Sequence Similarity 110-FAM110

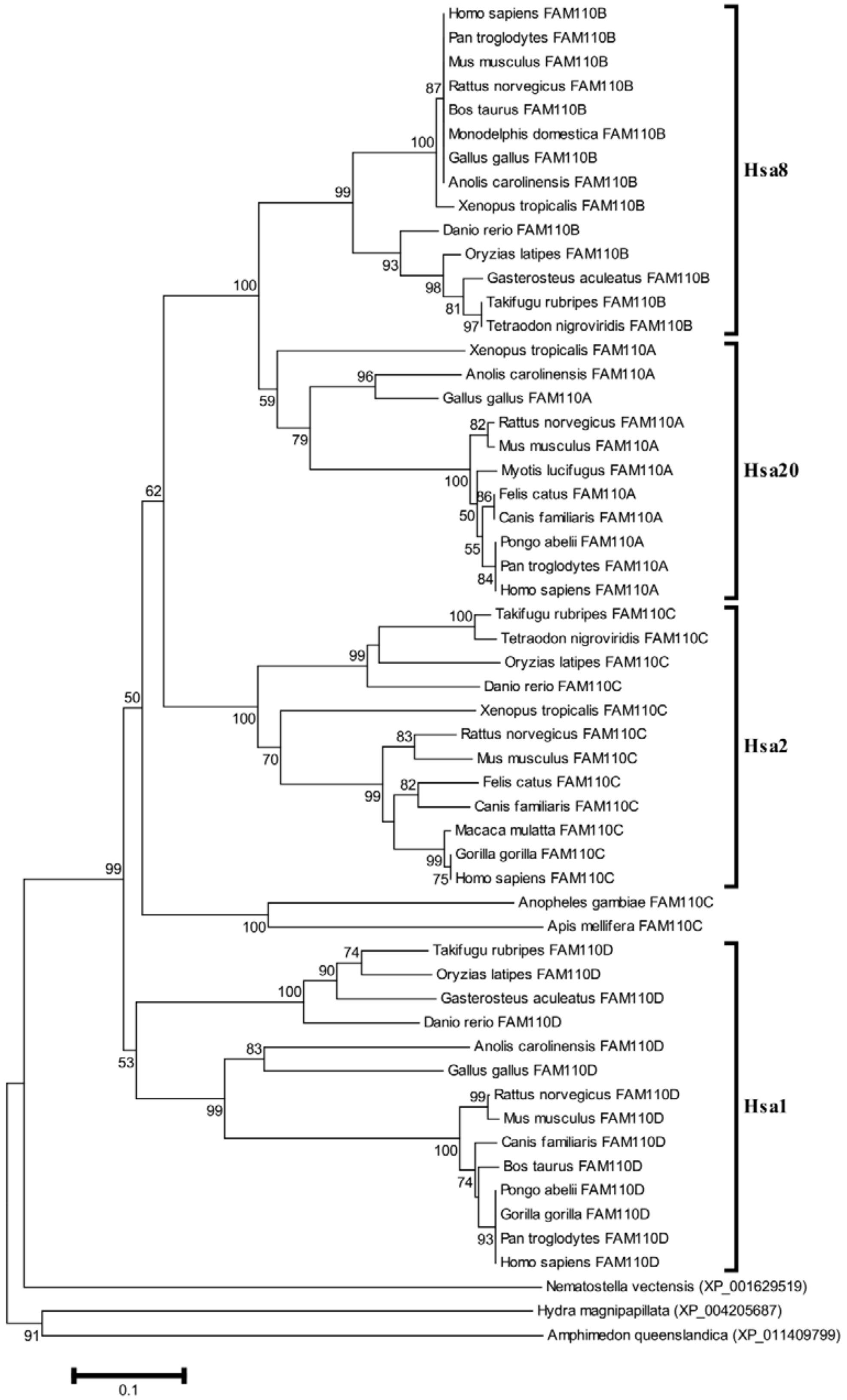

Phylogenetic tree of FAM110 family.

Neighbor-Joining tree of the FAM110 family members. Symbols and parameters are the same as described in supplementary Figure 1.1

Nuclear Receptor Coactivator-NCOA

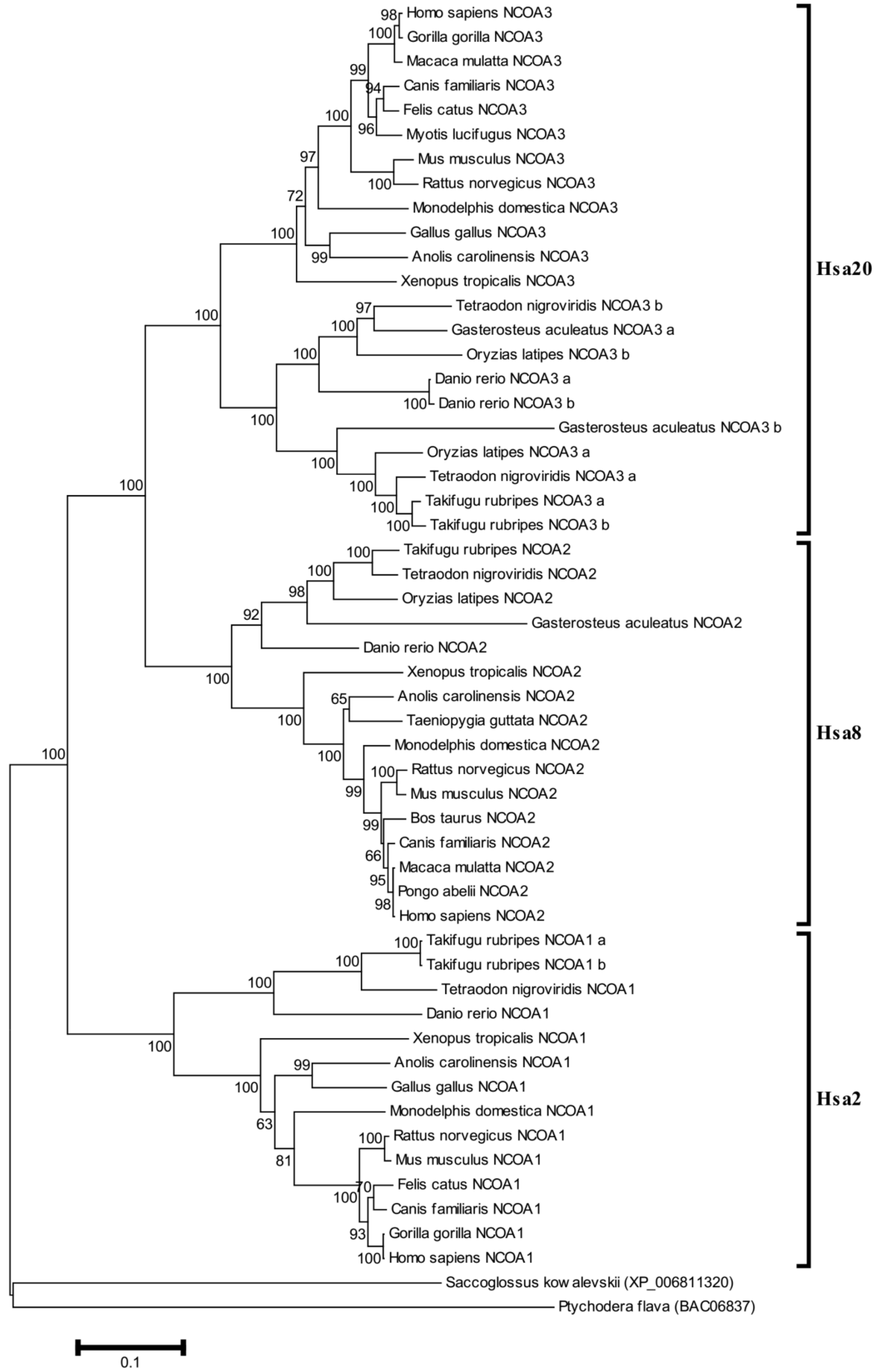

Phylogenetic tree of NCOA family.

Neighbor-Joining tree of the NCOA family members. Symbols and parameters are the same as described in supplementary Figure 1.1

YTH Domain-Containing Family Protein-YTHDF

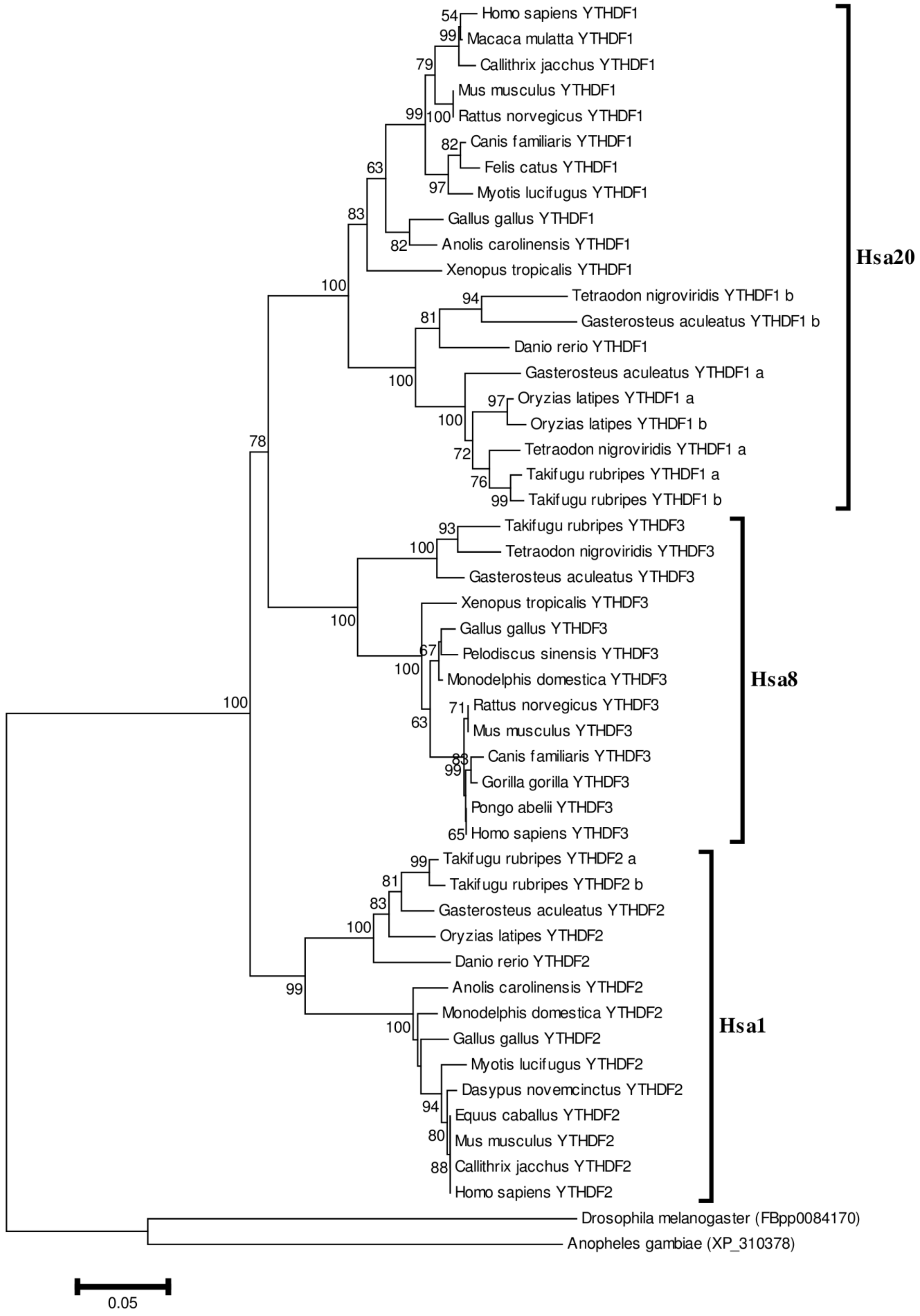

Phylogenetic tree of YTHDF family.

Neighbor-Joining tree of the YTHDF family members. Symbols and parameters are the same as described in supplementary Figure 1.1

X Kell Blood Group Precursor-related Family-XKR

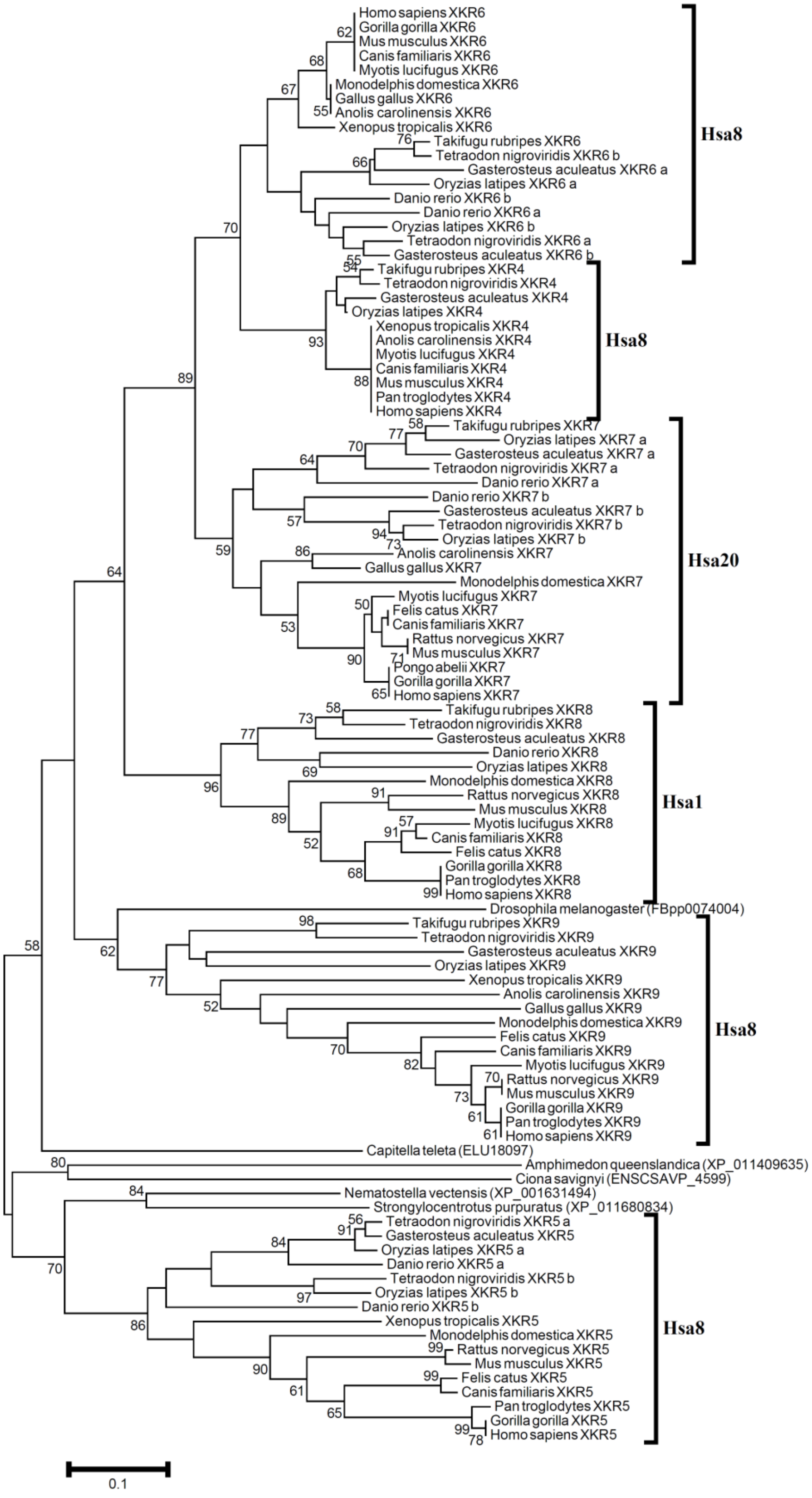

Phylogenetic tree of XKR family.

Neighbor-Joining tree of the XKR family members. Symbols and parameters are the same as described in supplementary Figure 1.1

Myelin Transcription Factor-MYT

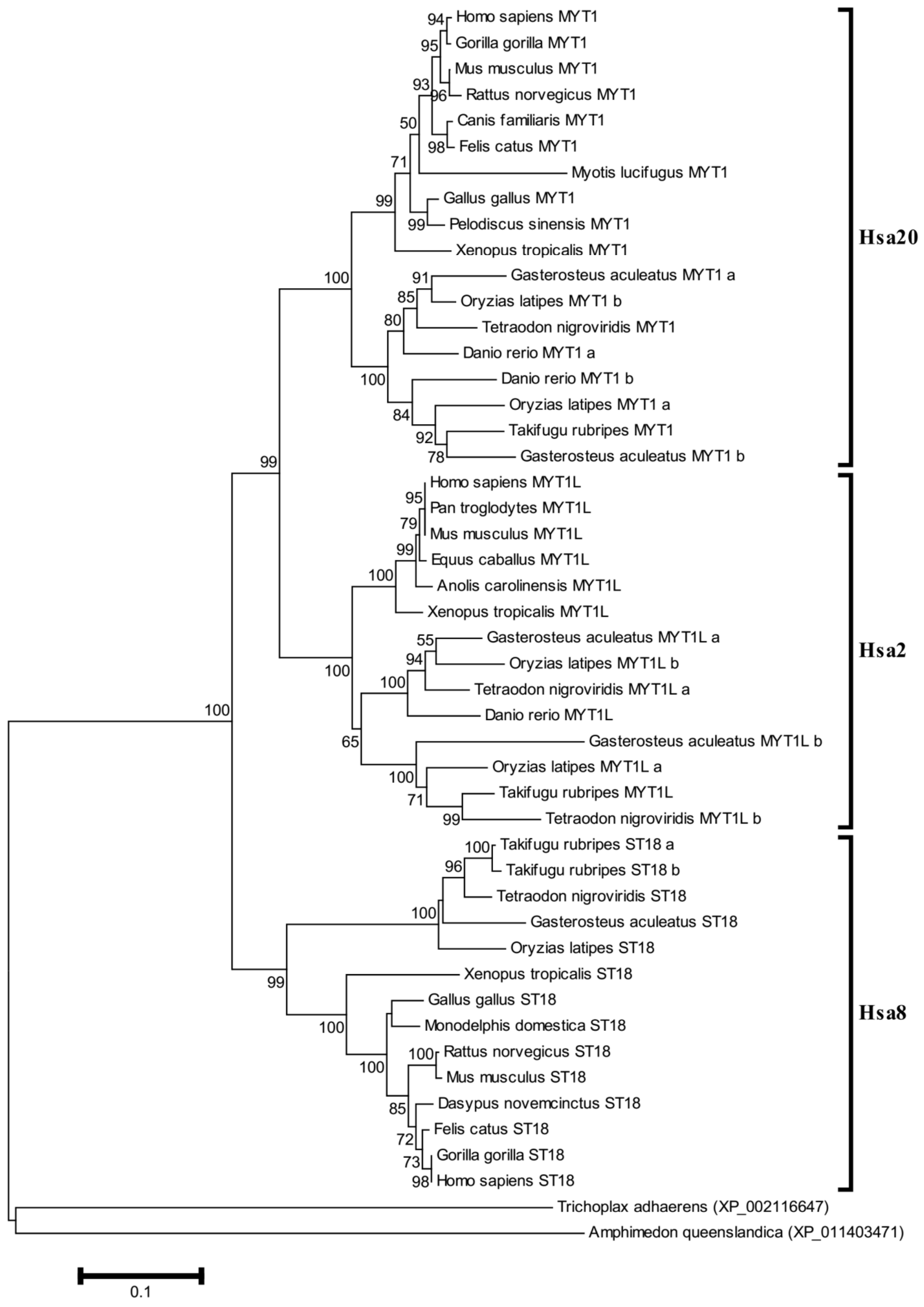

Phylogenetic tree of MYT family.

Neighbor-Joining tree of the MYT family members. Symbols and parameters are the same as described in supplementary Figure 1.1

Other gene families analyzed in the present study

Antizyme Inhibitor-AZIN

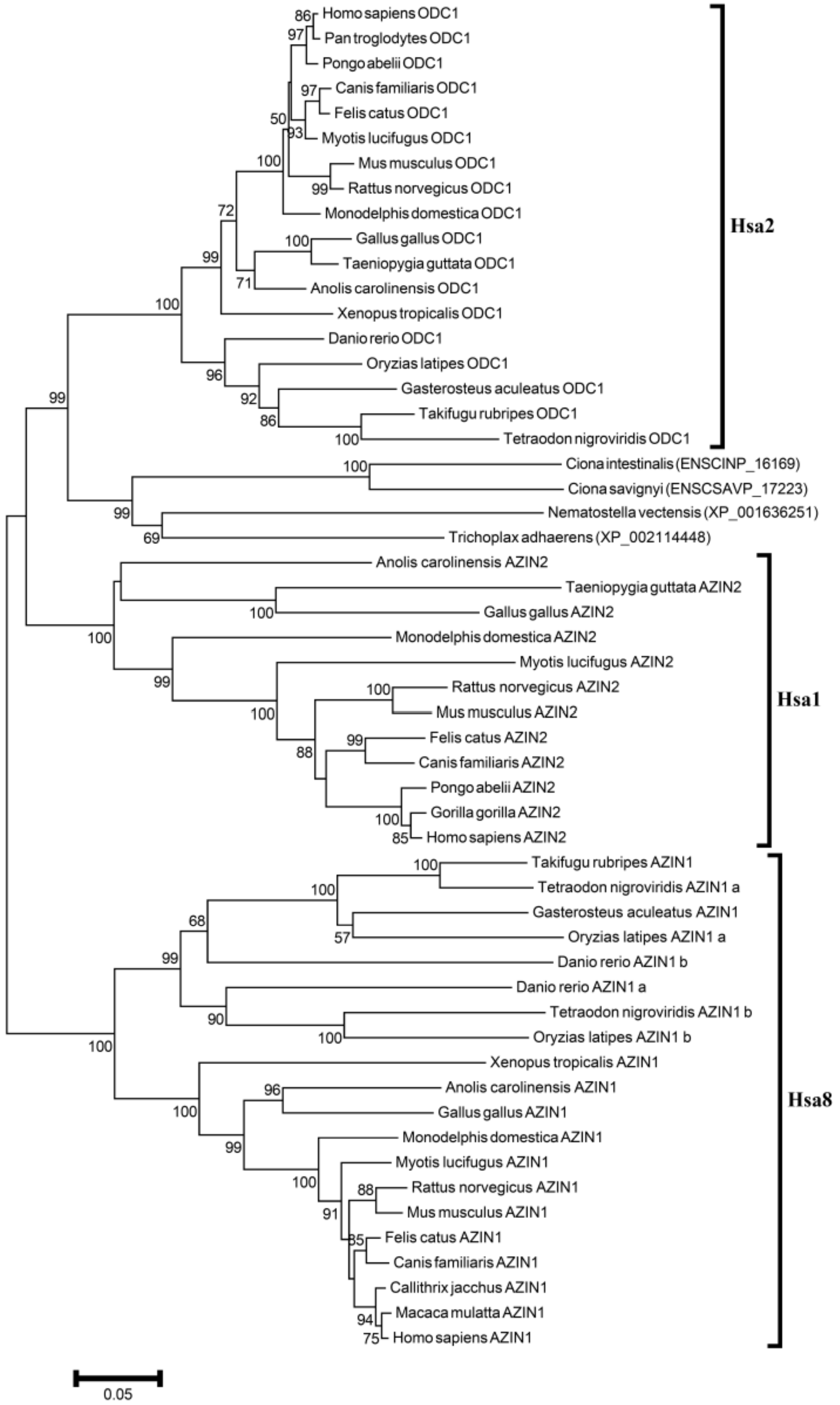

Phylogenetic tree of AZIN family.

Neighbor-Joining tree of the AZIN family members. Uncorrected p-distance was used. Complete-deletion option was used. Numbers on branches represent bootstrap values (based on 1000 replications) supporting that branch; only the values ≥50% are presented here. Scale bar shows amino acid substitution per site.

Cholinergic Receptor Nicotinic Subunits-CHRN

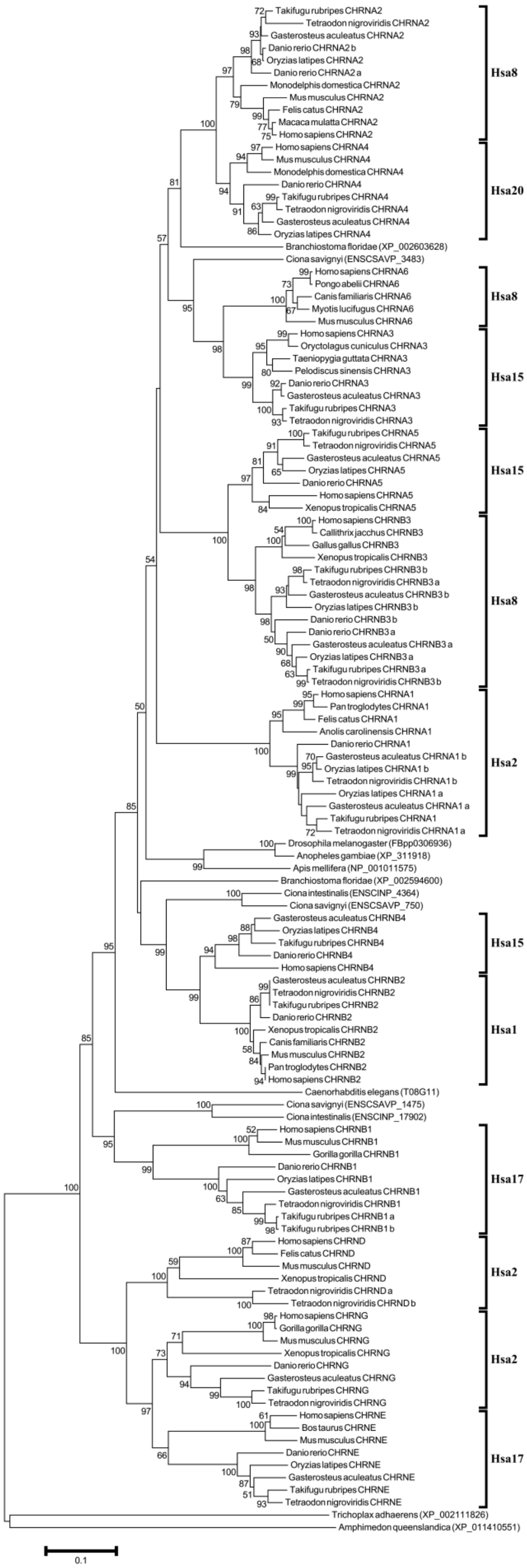

Phylogenetic tree of CHRN family.

Neighbor-Joining tree of the CHRN family members. Symbols and parameters are the same as described in supplementary Figure 1.1

Ciliary Rootlet Coiled-Coil Protein-CRO

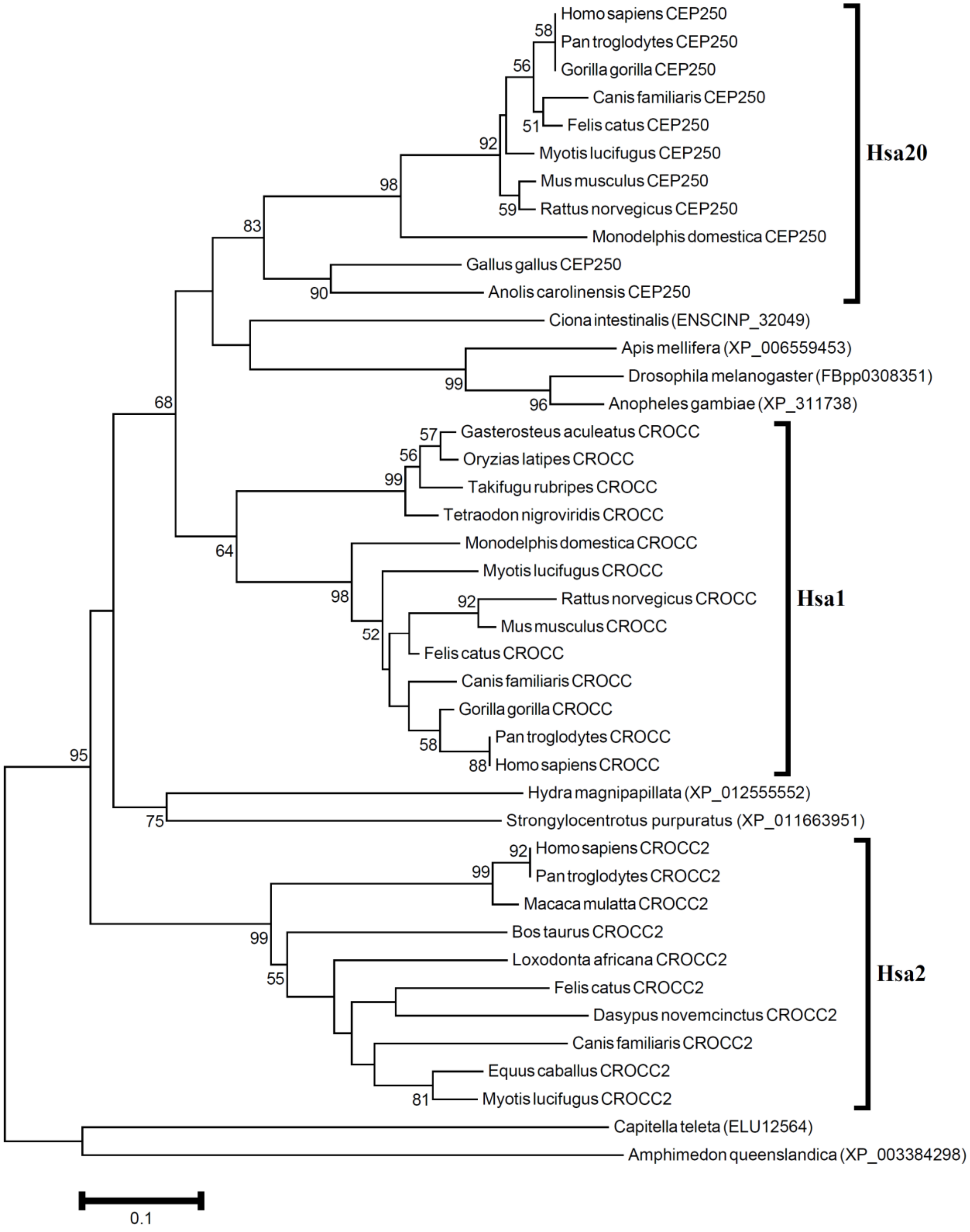

Phylogenetic tree of CRO family.

Neighbor-Joining tree of the CRO family members. Symbols and parameters are the same as described in supplementary Figure 1.1

Grainyhead like Transcription factor-GRHL

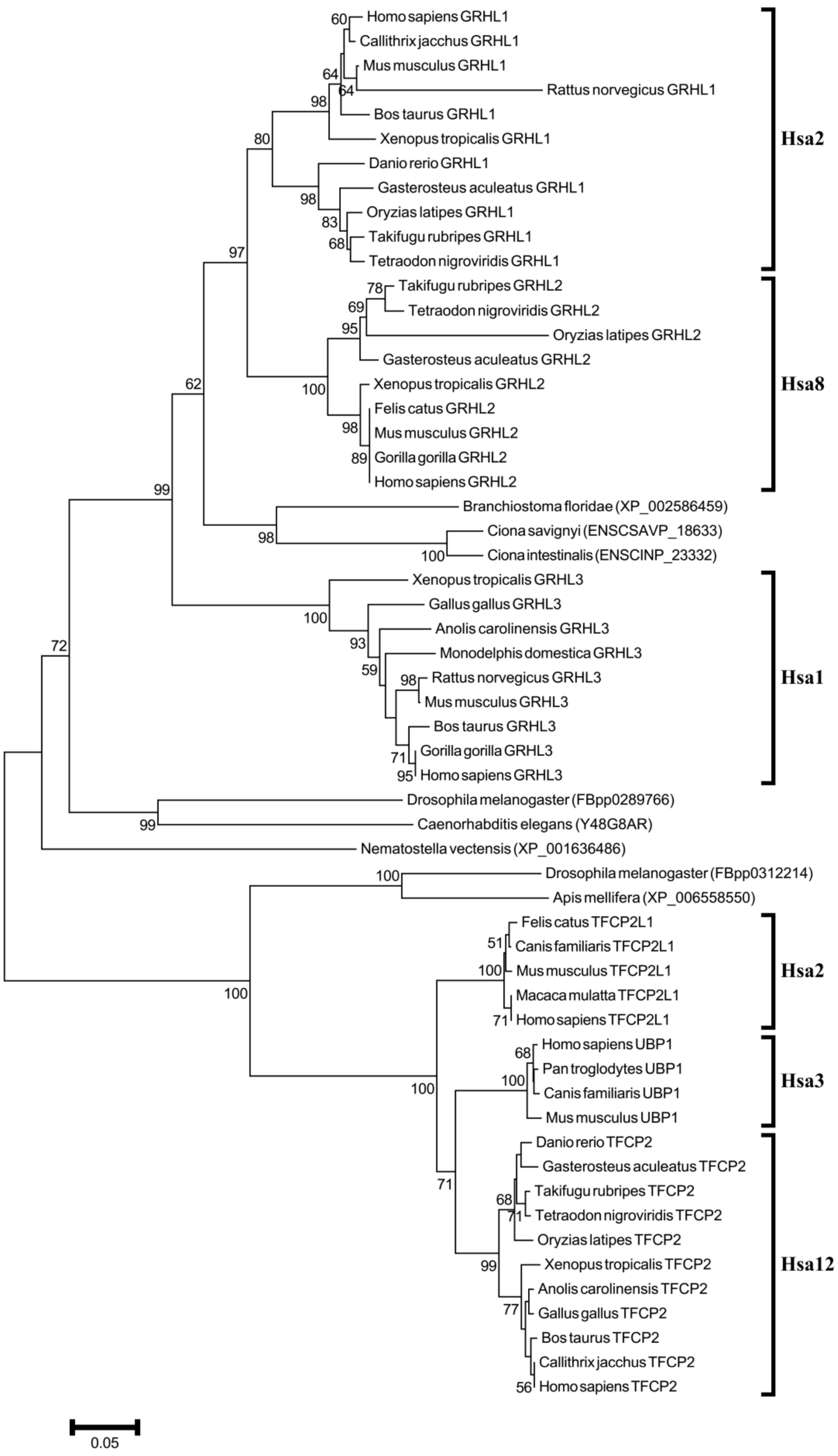

Phylogenetic tree of GRHL family.

Neighbor-Joining tree of the GRHL family members. Symbols and parameters are the same as described in supplementary Figure 1.1

Inhibitor of DNA Binding protein-ID

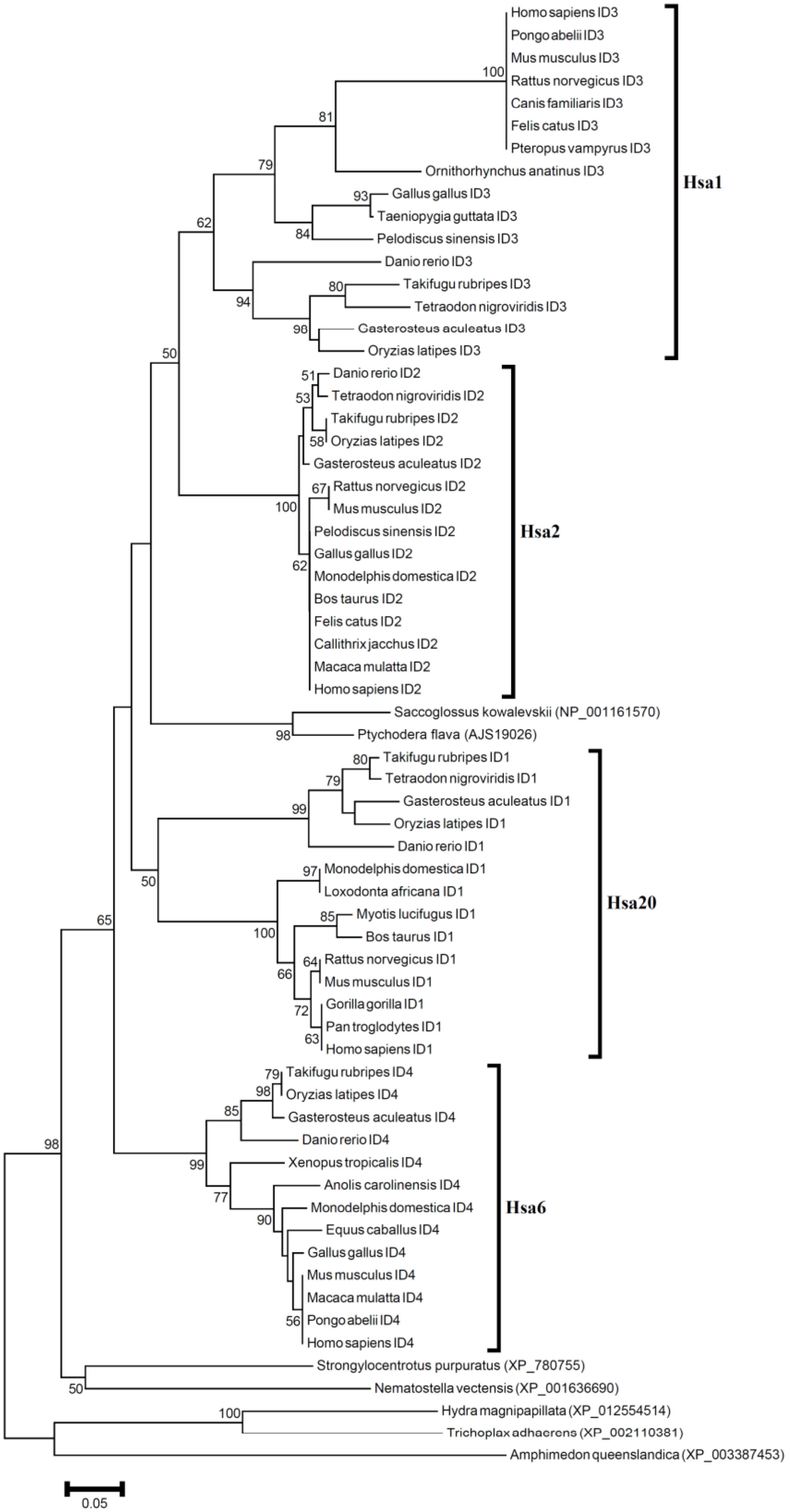

Phylogenetic tree of ID family.

Neighbor-Joining tree of the ID family members. Symbols and parameters are the same as described in supplementary Figure 1.1

Regulator of G-protein Signaling-RGS

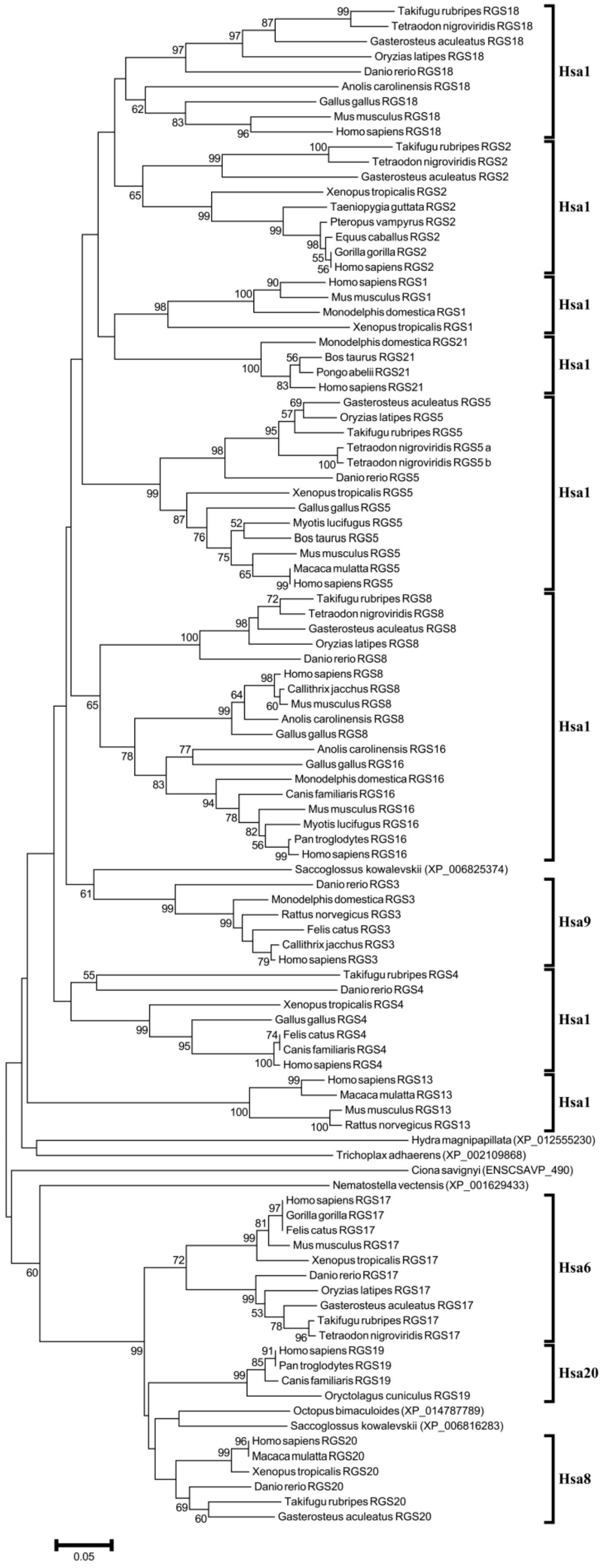

Phylogenetic tree of RGS family.

Neighbor-Joining tree of the RGS family members. Symbols and parameters are the same as described in supplementary Figure 1.1

Regulating Synaptic Membrane Exocytosis Protein -RIMS

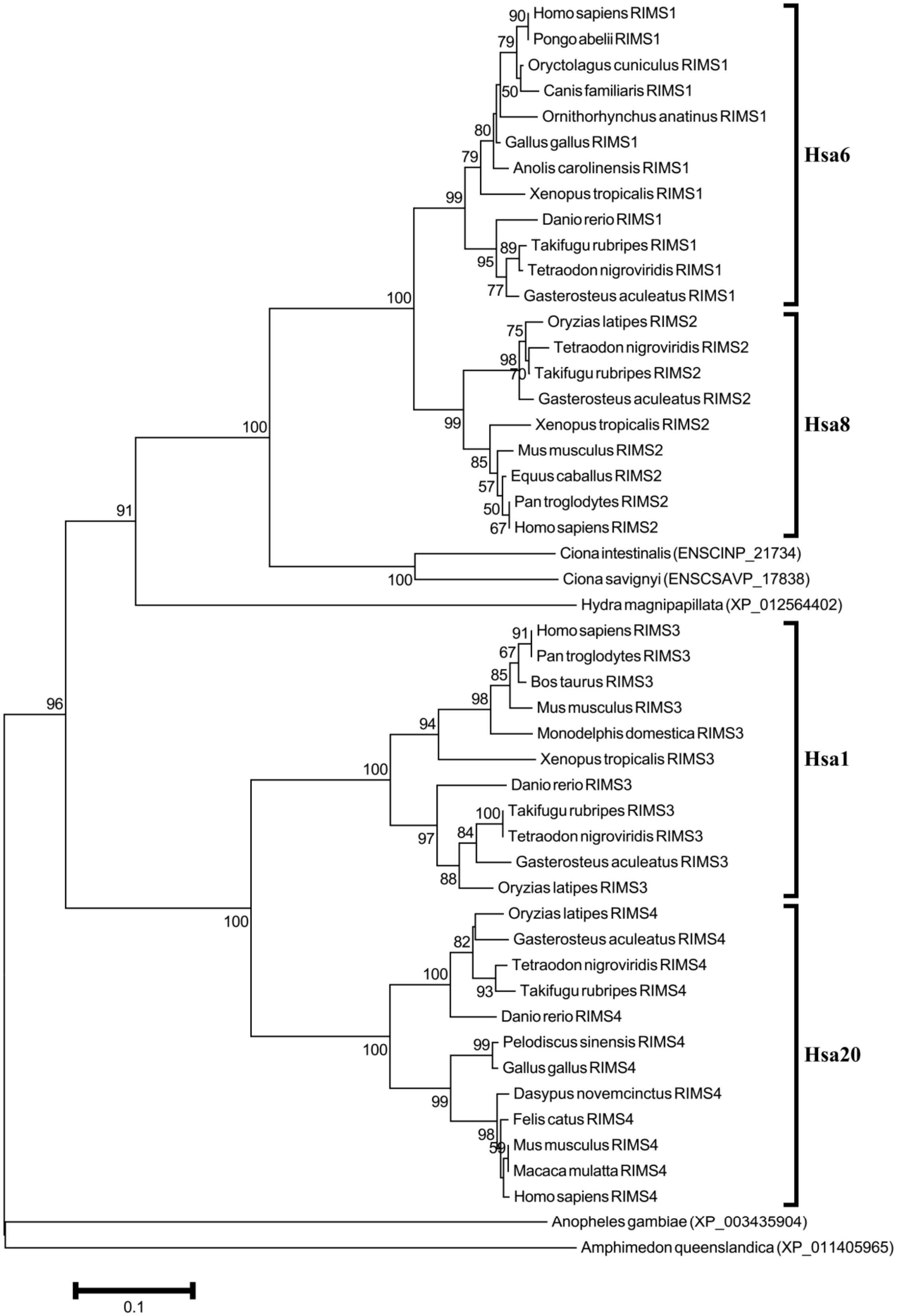

Phylogenetic tree of RIMS family.

Neighbor-Joining tree of the RIMS family members. Symbols and parameters are the same as described in supplementary Figure 1.1

R-Spondin Homolog-RSPO

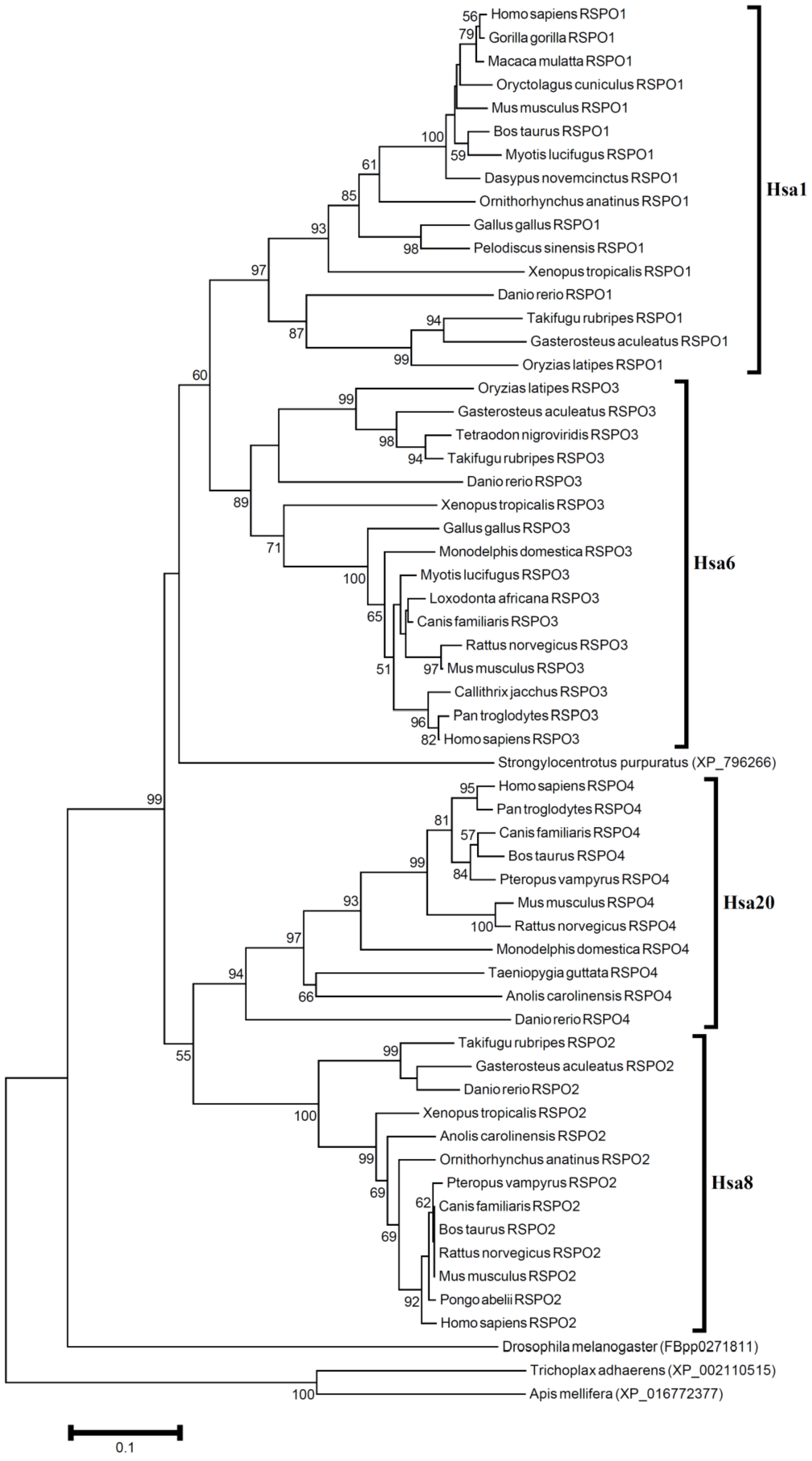

Phylogenetic tree of RSPO family.

Neighbor-Joining tree of the RSPO family members. Symbols and parameters are the same as described in supplementary Figure 1.1

Solute Carrier Family-SLC

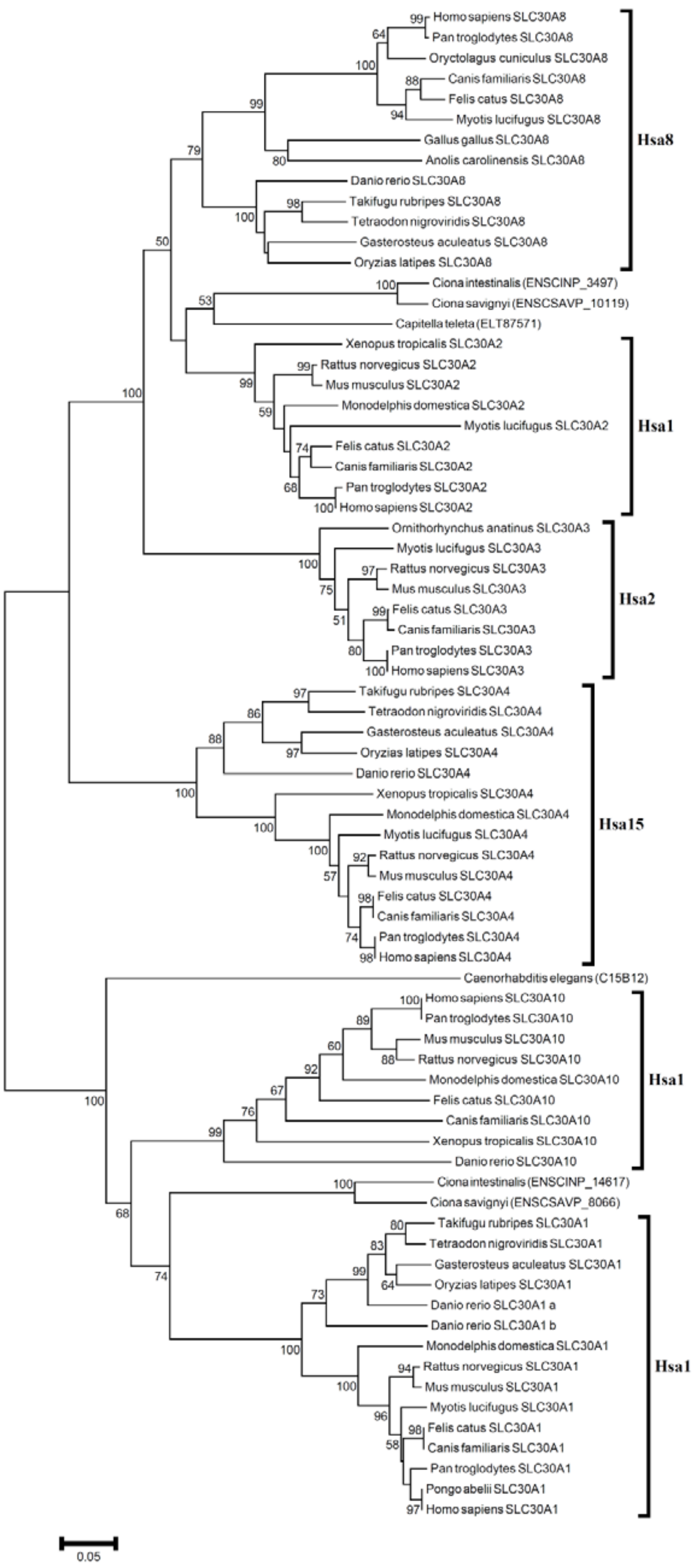

Phylogenetic tree of SLC family.

Neighbor-Joining tree of the SLC family members. Symbols and parameters are the same as described in supplementary Figure 1.1

Syntrophin, Gamma-SNT

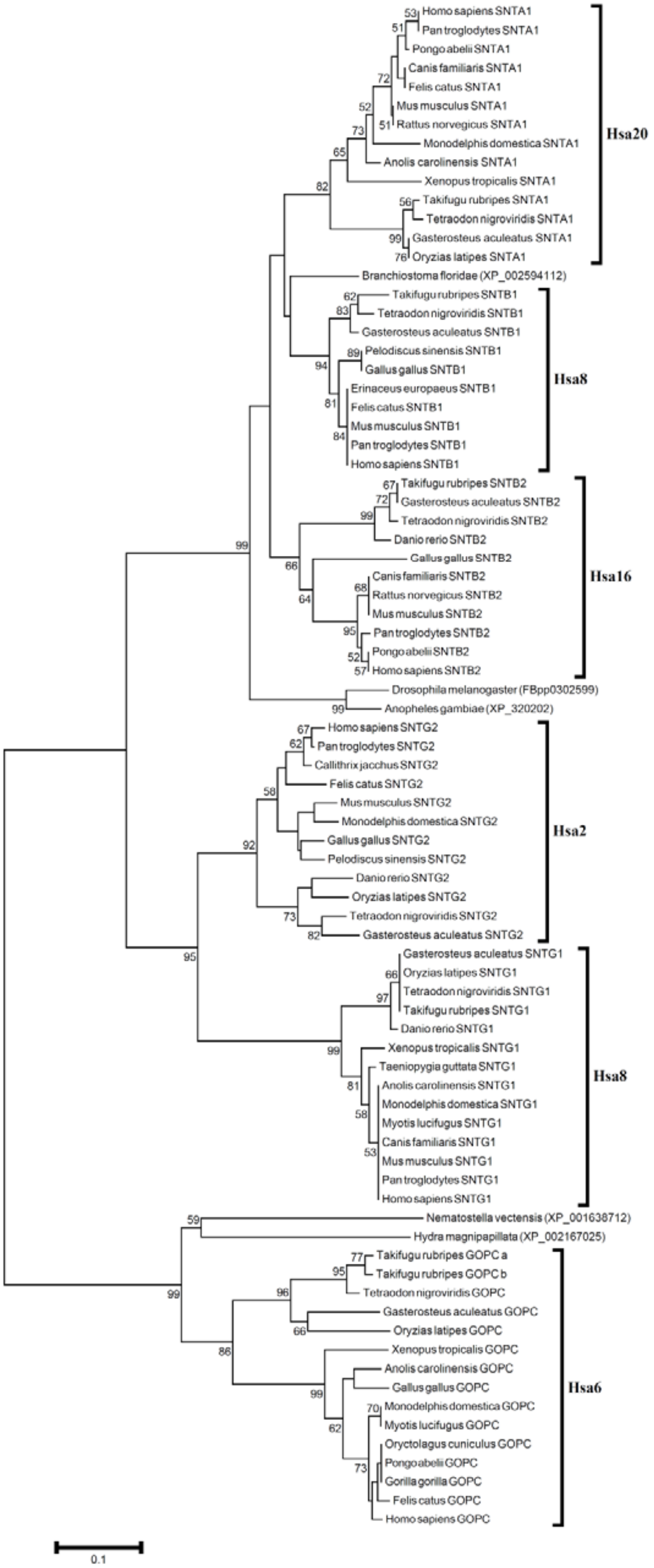

Phylogenetic tree of SNT family.

Neighbor-Joining tree of the SNT family members. Symbols and parameters are the same as described in supplementary Figure 1.1

Sorting Nexin Family-SNX

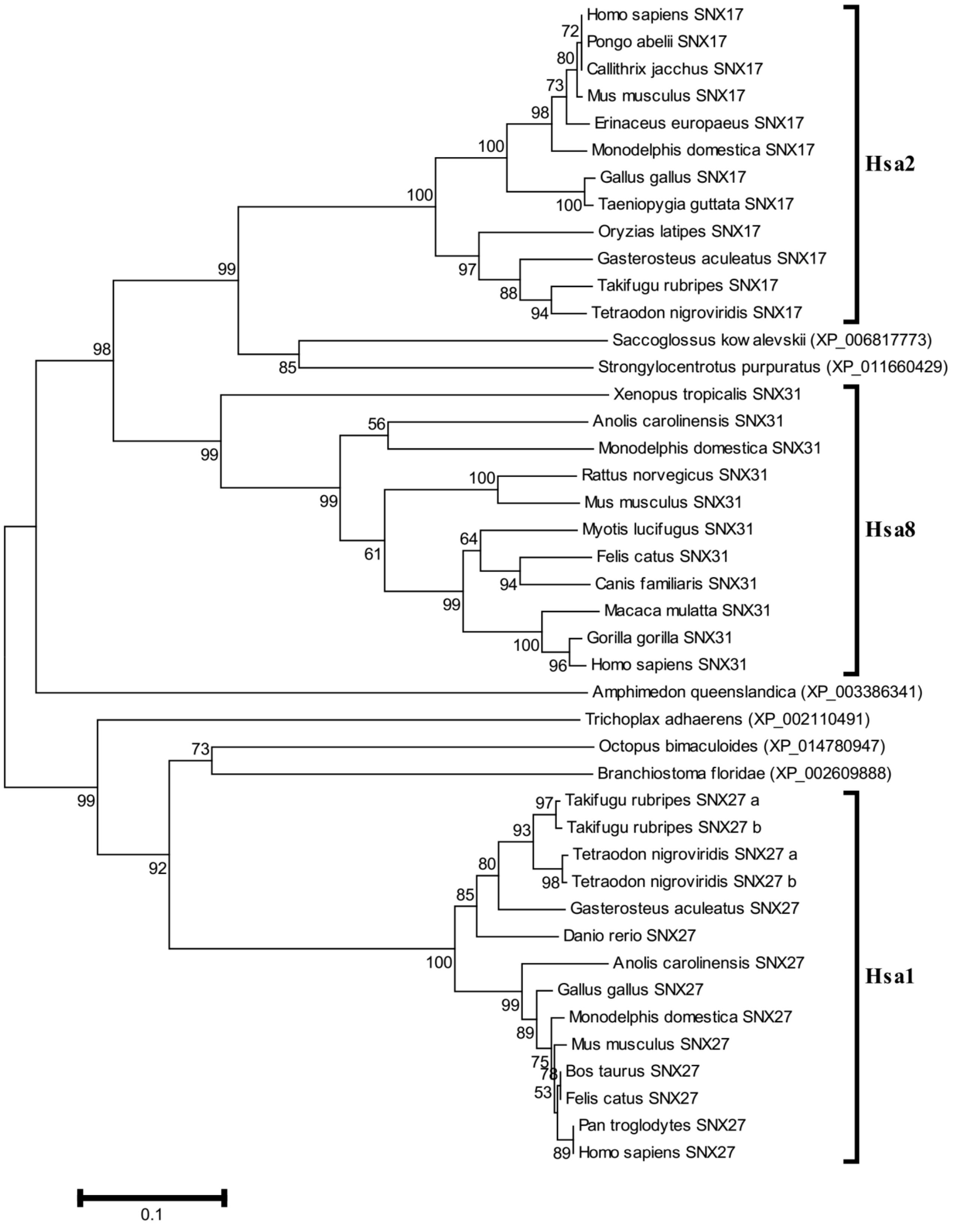

Phylogenetic tree of SNX family.

Neighbor-Joining tree of the SNX family members. Symbols and parameters are the same as described in supplementary Figure 1.1

Transcription Elongation factor A (SII) Protein-TCEA

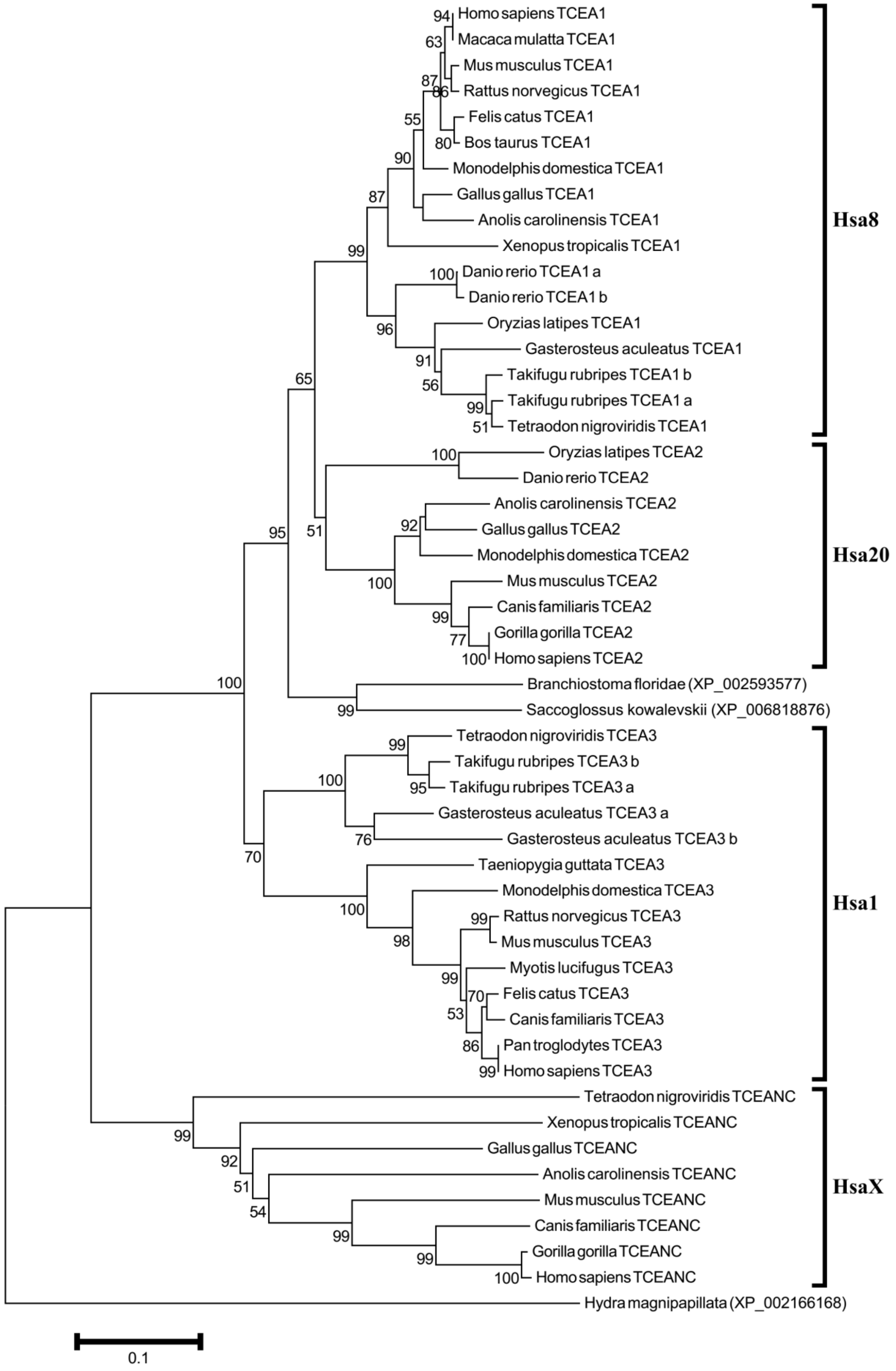

Phylogenetic tree of TCEA family.

Neighbor-Joining tree of the TCEA family members. Symbols and parameters are the same as described in supplementary Figure 1.1

UBX Domain-Containing Protein-UBXN

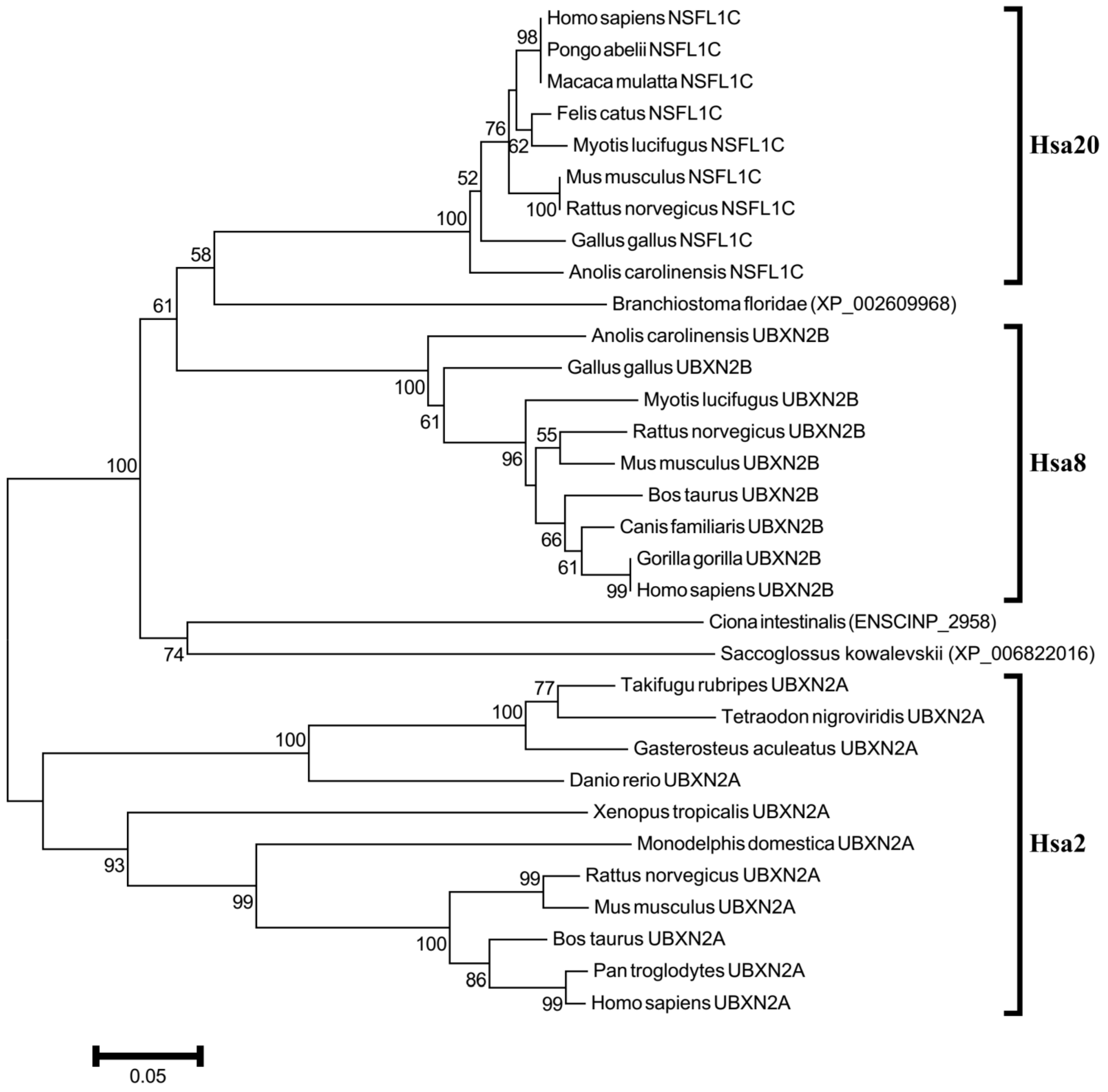

Phylogenetic tree of UBXN family.

Neighbor-Joining tree of the UBXN family members. Symbols and parameters are the same as described in supplementary Figure 1.1
