# Supplementary material for: Evolutionary history of the human multigene families reveals widespread gene duplications throughout the history of animals
Source: BMC Evol Biol. 2019 Jun 20;19:128. doi: 10.1186/s12862-019-1441-0 (PMC6585022; doi:10.1186/s12862-019-1441-0)
Supplement: Supplementary file 4 — Maximum likelihood Trees of gene families (residing on human chromosomes 1/2/8/20) based on WAG model. (PDF 3836 kb) [file 12862_2019_1441_MOESM4_ESM.pdf]

Additional file 4: Maximum Likelihood Trees of gene families (residing on human chromosomes 1/2/8/20) based on WAG model.

Co-duplicated group-1

MROH8 and STK

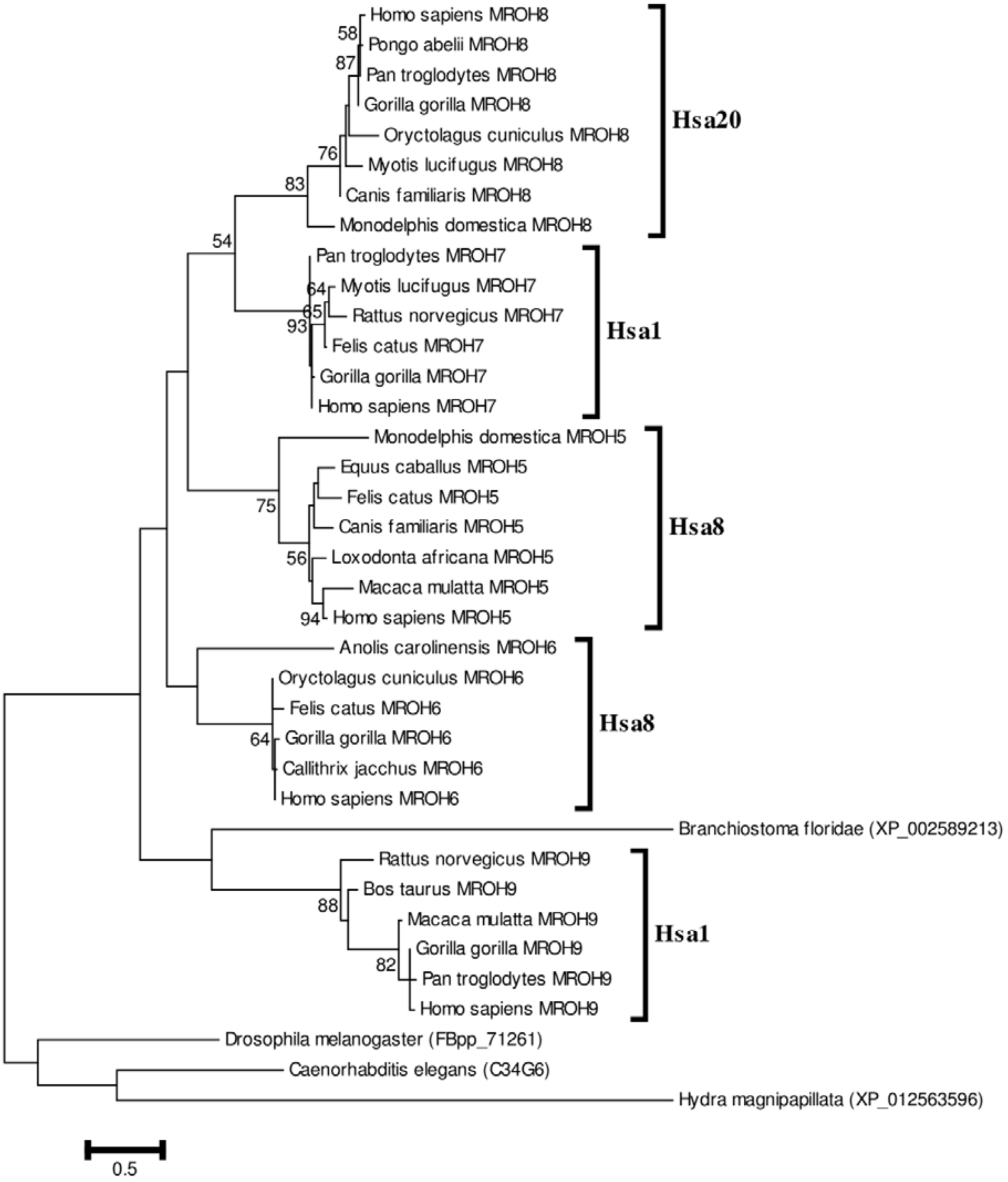

Phylogenetic tree of MROH family.

The evolutionary history was inferred by using the Maximum Likelihood method based on the Whelan And Goldman model. The tree with the highest log likelihood (-3194.2605) is shown. The percentage of trees in which the associated taxa clustered together is shown next to the branches ; only the values ≥ 50% are shown here. A discrete Gamma distribution was used to model evolutionary rate differences among sites (5 categories (+*G*, parameter = 9.5137)). The tree is drawn to scale, with branch lengths measured in the number of substitutions per site. The analysis involved 37 amino acid sequences. All positions containing gaps and missing data were eliminated. There were a total of 80 positions in the final dataset.

Serine/Threonine-Protein Kinase-STK

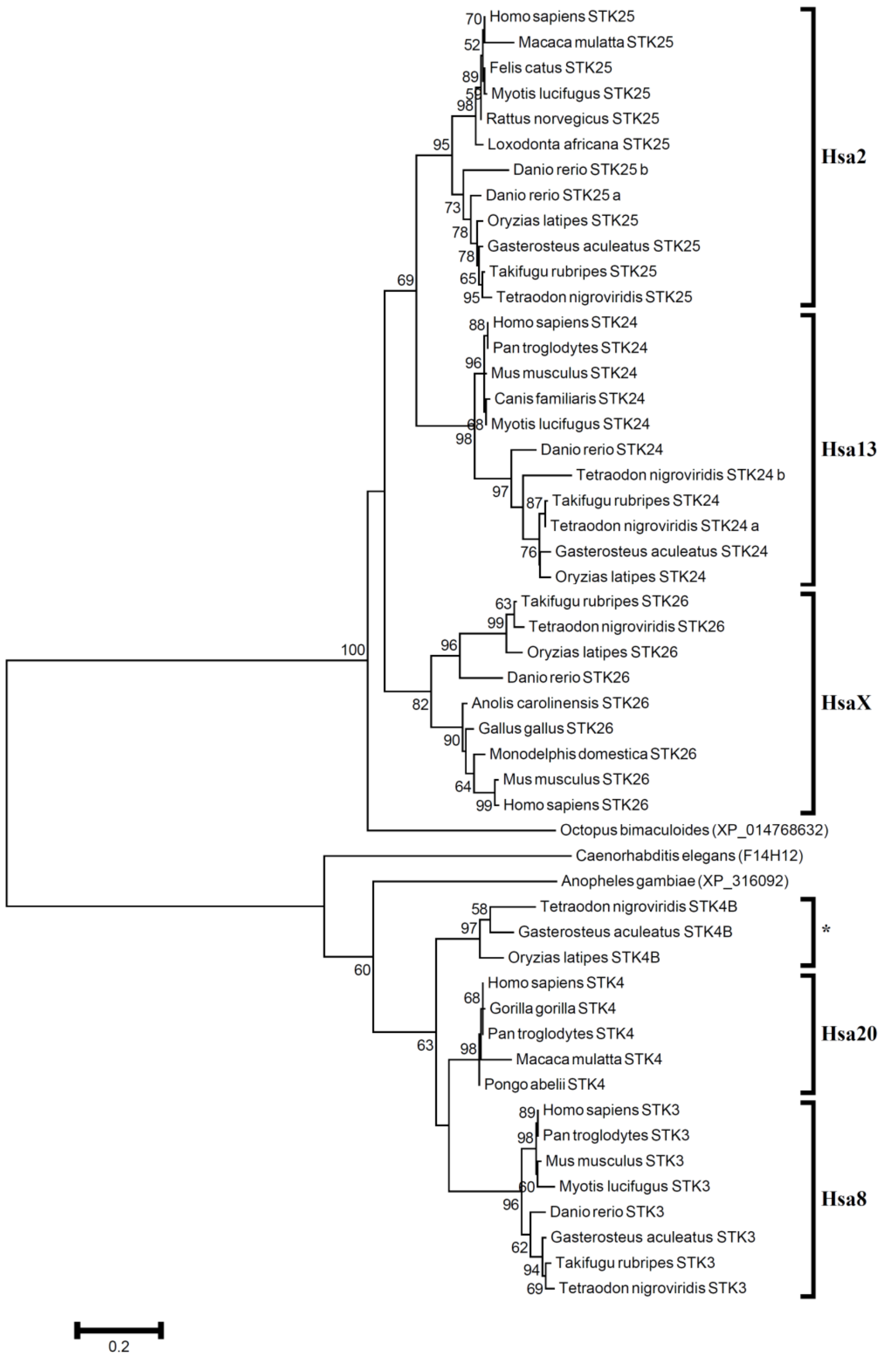

Phylogenetic tree of STK family.

The evolutionary history was inferred by using the Maximum Likelihood method based on the Whelan And Goldman model. The tree with the highest log likelihood (-5708.4638) is shown. The percentage of trees in which the associated taxa clustered together is shown next to the branches ; only the values ≥ 50% are shown here. A discrete Gamma distribution was used to model evolutionary rate differences among sites (5 categories (+*G*, parameter = 1.0610)). The tree is drawn to scale, with branch lengths measured in the number of substitutions per site. The analysis involved 51 amino acid sequences. All positions containing gaps and missing data were eliminated. There were a total of 252 positions in the final dataset.

Co-duplicated group-2

E2F, STMN1, EYA*

Asterisks (*) represent families published previously by our research group (for details see main text).

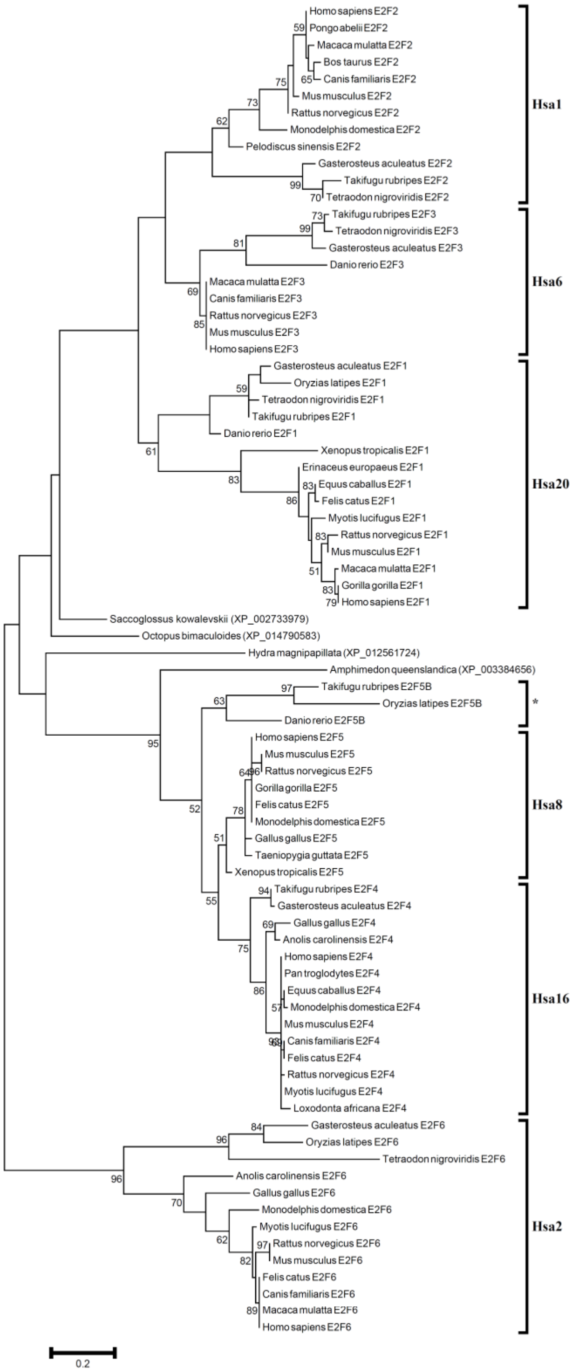

Phylogenetic tree of E2F family.

The evolutionary history was inferred by using the Maximum Likelihood method based on the Whelan And Goldman model. The tree with the highest log likelihood (-3603.2899) is shown. The percentage of trees in which the associated taxa clustered together is shown next to the branches ; only the values ≥ 50% are shown here. A discrete Gamma distribution was used to model evolutionary rate differences among sites (5 categories (+*G*, parameter = 1.4435)). The tree is drawn to scale, with branch lengths measured in the number of substitutions per site. The analysis involved 79 amino acid sequences. All positions containing gaps and missing data were eliminated. There were a total of 84 positions in the final dataset.

Stathmin-STMN

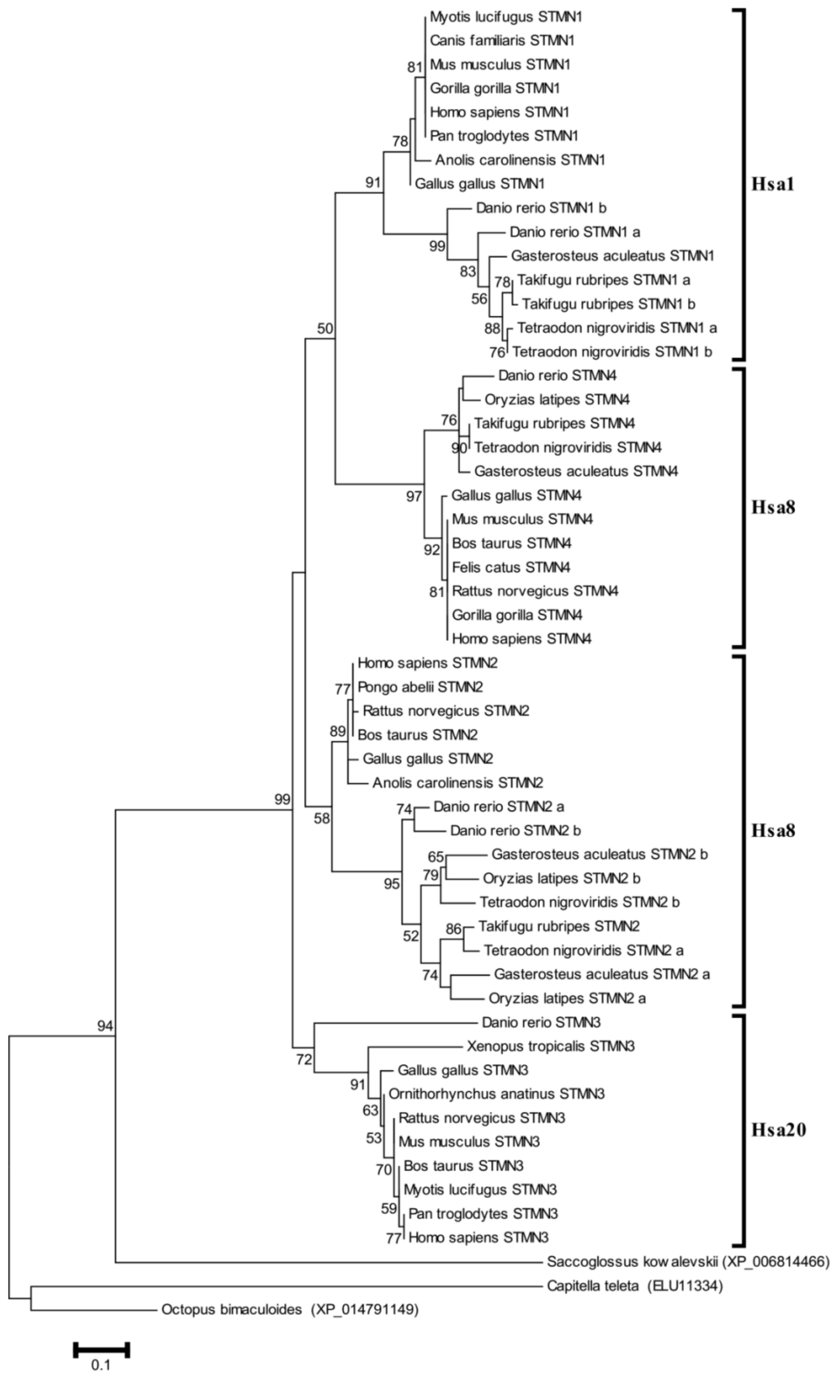

Phylogenetic tree of STMN family.

The evolutionary history was inferred by using the Maximum Likelihood method based on the Whelan And Goldman model. The tree with the highest log likelihood (-2434.5527) is shown. The percentage of trees in which the associated taxa clustered together is shown next to the branches ; only the values ≥ 50% are shown here. A discrete Gamma distribution was used to model evolutionary rate differences among sites (5 categories (+*G*, parameter = 1.7372)). The tree is drawn to scale, with branch lengths measured in the number of substitutions per site. The analysis involved 55 amino acid sequences. All positions containing gaps and missing data were eliminated. There were a total of 107 positions in the final dataset.

Co-duplicated group-3

HCK*, DLGAP, NKAIN, KCNQ, MATN4*

Asterisks (*) represent families published previously by our research group (for details see main text).

Discs, large Drosophila Homolog-associated Protein-DLGAP

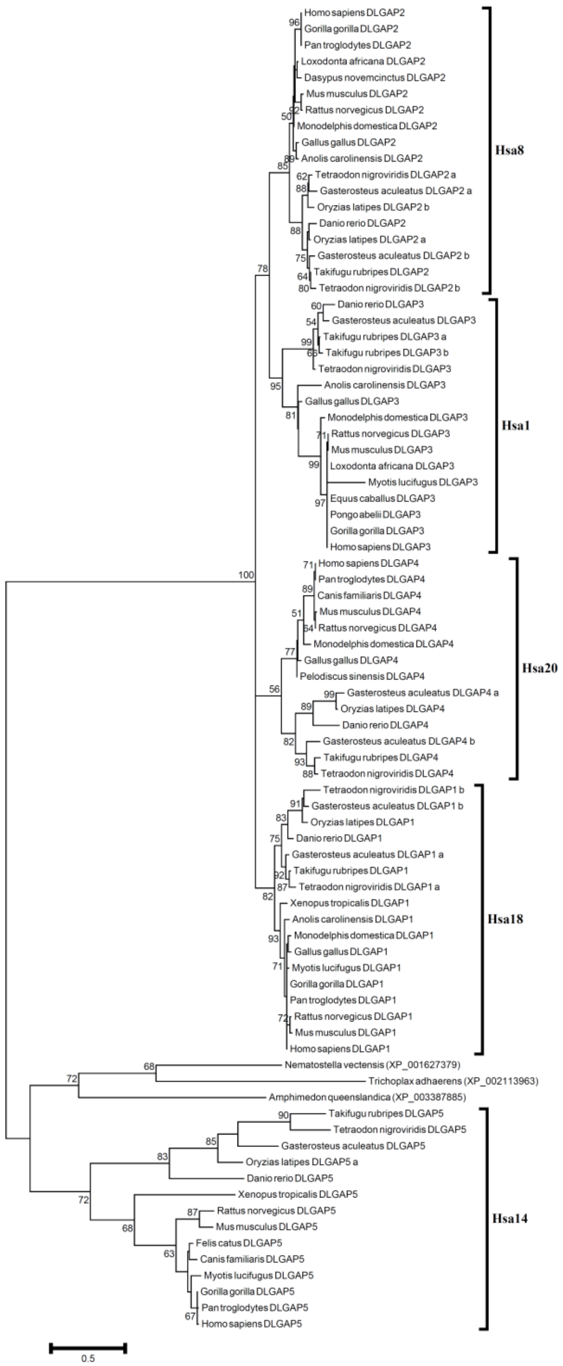

Phylogenetic tree of DLGAP family.

The evolutionary history was inferred by using the Maximum Likelihood method based on the Whelan And Goldman model. The tree with the highest log likelihood (-7367.7106) is shown. The percentage of trees in which the associated taxa clustered together is shown next to the branches ; only the values ≥ 50% are shown here. A discrete Gamma distribution was used to model evolutionary rate differences among sites (5 categories (+*G*, parameter = 4.3659)). The tree is drawn to scale, with branch lengths measured in the number of substitutions per site. The analysis involved 82 amino acid sequences. All positions containing gaps and missing data were eliminated. There were a total of 146 positions in the final dataset.

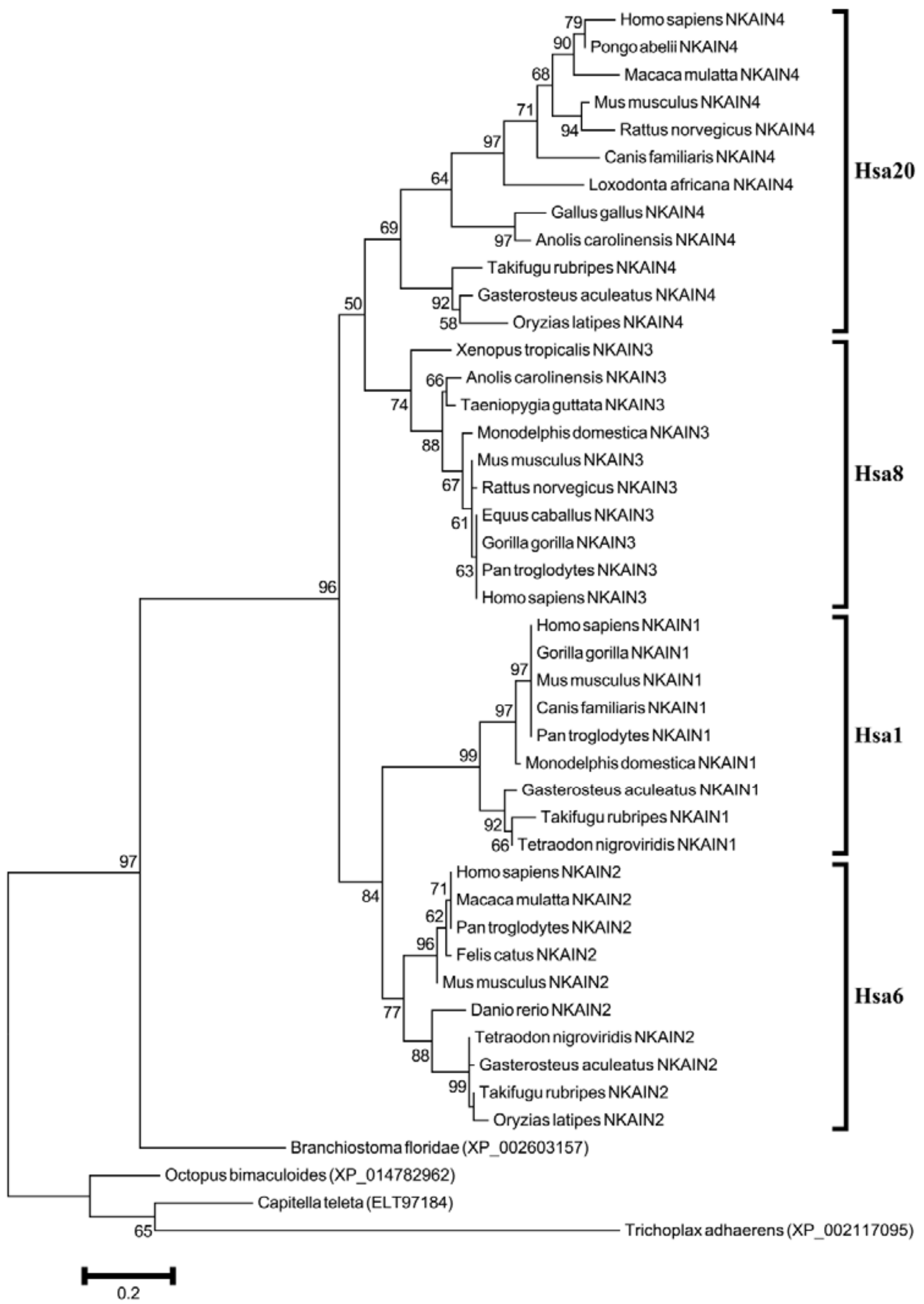

Phylogenetic tree of NKAIN family.

The evolutionary history was inferred by using the Maximum Likelihood method based on the Whelan And Goldman model. The tree with the highest log likelihood (-2308.7286) is shown. The percentage of trees in which the associated taxa clustered together is shown next to the branches ; only the values ≥ 50% are shown here. A discrete Gamma distribution was used to model evolutionary rate differences among sites (5 categories (+*G*, parameter = 1.6036)). The tree is drawn to scale, with branch lengths measured in the number of substitutions per site. The analysis involved 45 amino acid sequences. All positions containing gaps and missing data were eliminated. There were a total of 94 positions in the final dataset.

Potassium Voltage-Gated Channel subfamily Q-KCNQ

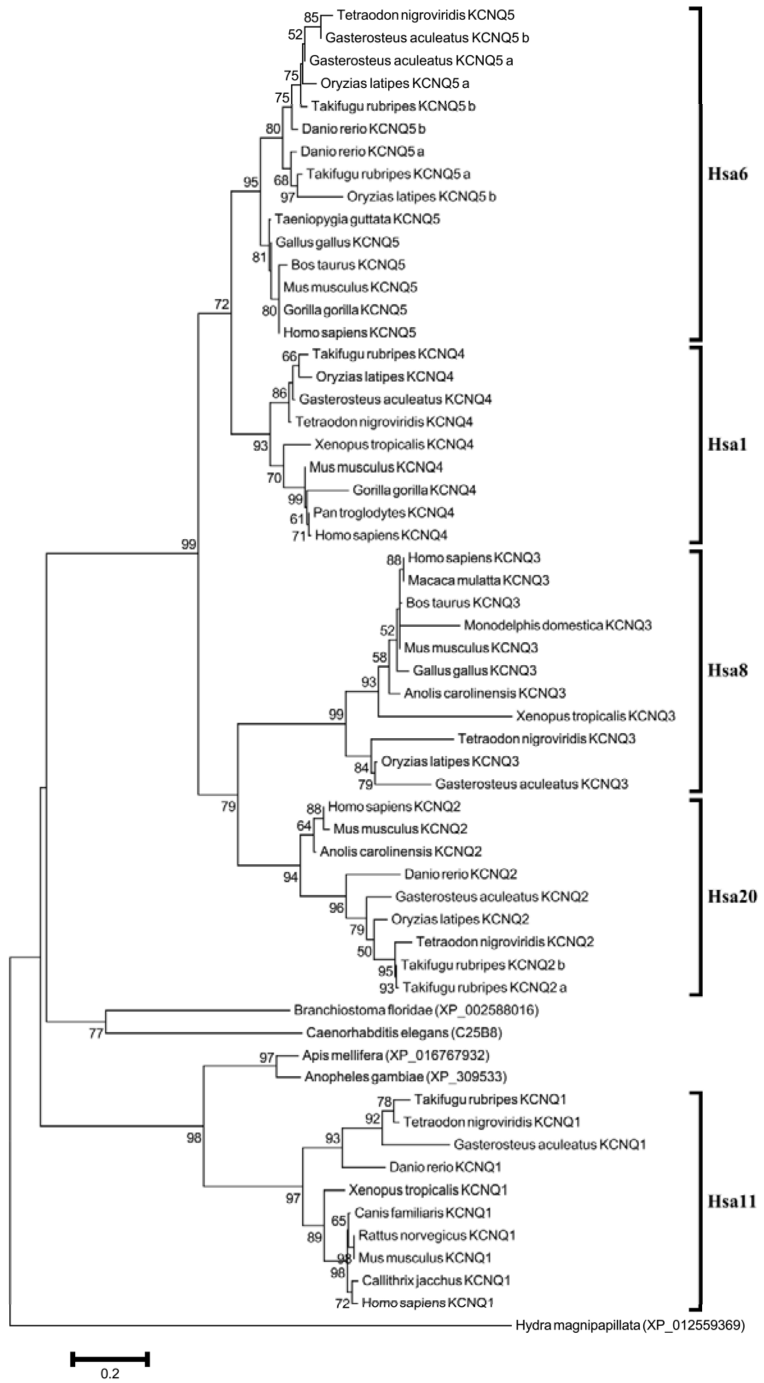

Phylogenetic tree of KCNQ family.

The evolutionary history was inferred by using the Maximum Likelihood method based on the Whelan And Goldman model. The tree with the highest log likelihood (-6689.7208) is shown. The percentage of trees in which the associated taxa clustered together is shown next to the branches ; only the values ≥ 50% are shown here. A discrete Gamma distribution was used to model evolutionary rate differences among sites (5 categories (+*G*, parameter = 1.9925)). The tree is drawn to scale, with branch lengths measured in the number of substitutions per site. The analysis involved 59 amino acid sequences. All positions containing gaps and missing data were eliminated. There were a total of 206 positions in the final dataset.

Co-duplicated group-4

FAM110, NCOA, KCNS*, YTHDF, XKR, MYT

Asterisks (*) represent families published previously by our research group (for details see main text).

Family with Sequence Similarity 110-FAM110

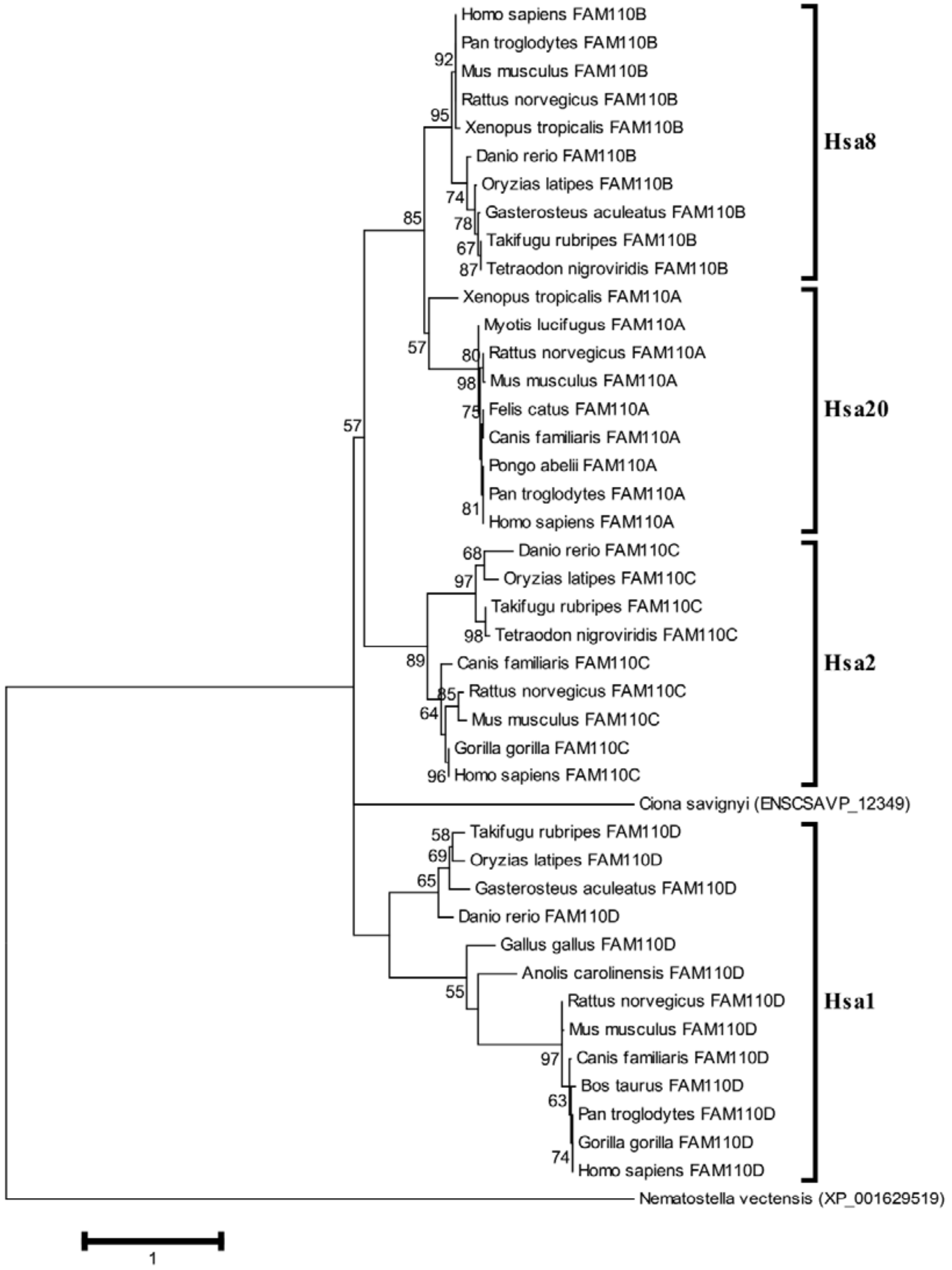

Phylogenetic tree of FAM110 family.

The evolutionary history was inferred by using the Maximum Likelihood method based on the Whelan And Goldman model. The tree with the highest log likelihood (-2939.3885) is shown. The percentage of trees in which the associated taxa clustered together is shown next to the branches ; only the values ≥ 50% are shown here. A discrete Gamma distribution was used to model evolutionary rate differences among sites (5 categories (+*G*, parameter = 4.3955)). The tree is drawn to scale, with branch lengths measured in the number of substitutions per site. The analysis involved 43 amino acid sequences. All positions containing gaps and missing data were eliminated. There were a total of 95 positions in the final dataset.

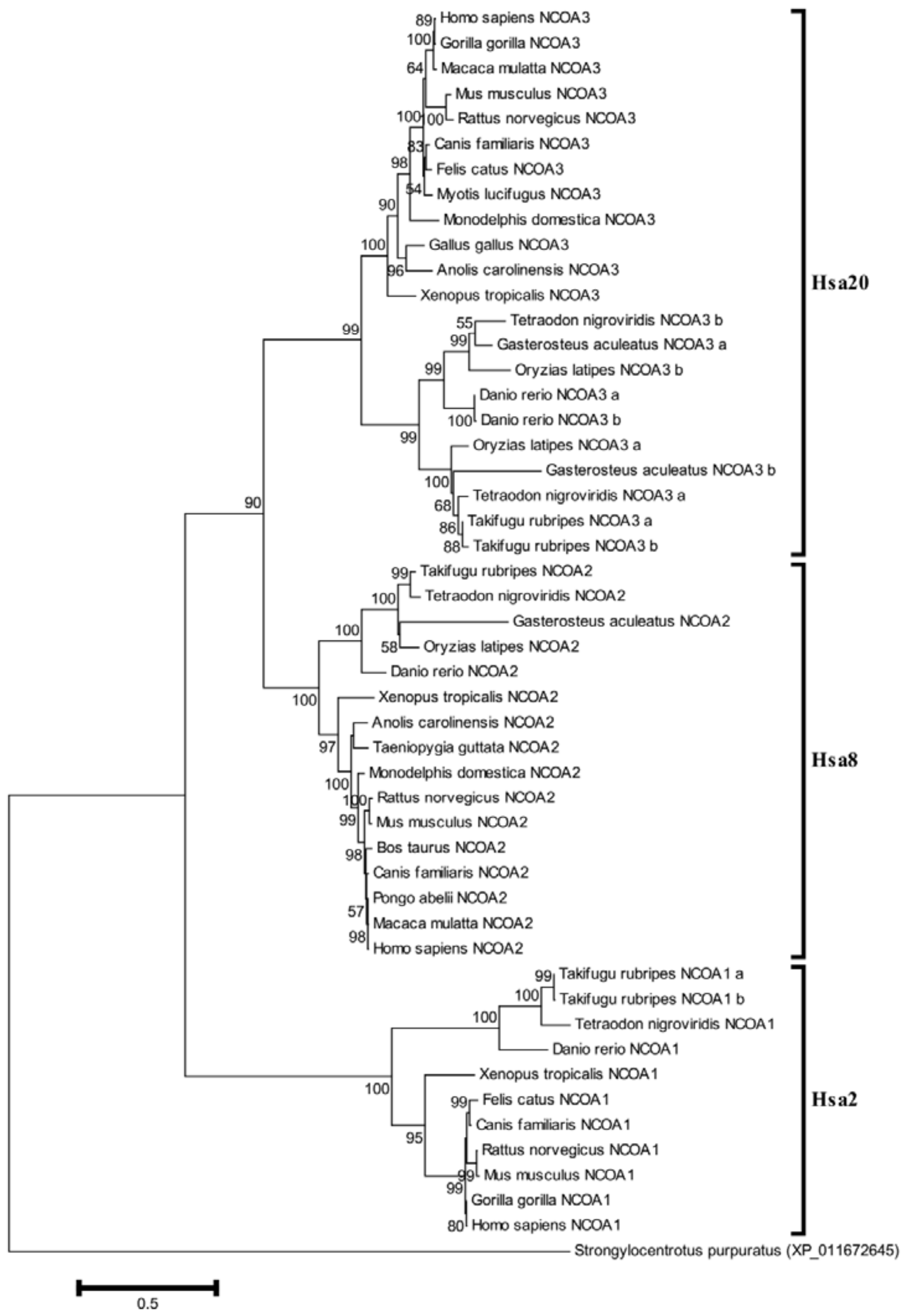

Phylogenetic tree of NCOA family.

The evolutionary history was inferred by using the Maximum Likelihood method based on the Whelan And Goldman model. The tree with the highest log likelihood (-24506.3558) is shown. The percentage of trees in which the associated taxa clustered together is shown next to the branches ; only the values ≥ 50% are shown here. A discrete Gamma distribution was used to model evolutionary rate differences among sites (5 categories (+*G*, parameter = 2.7804)). The tree is drawn to scale, with branch lengths measured in the number of substitutions per site. The analysis involved 50 amino acid sequences. All positions containing gaps and missing data were eliminated. There were a total of 747 positions in the final dataset.

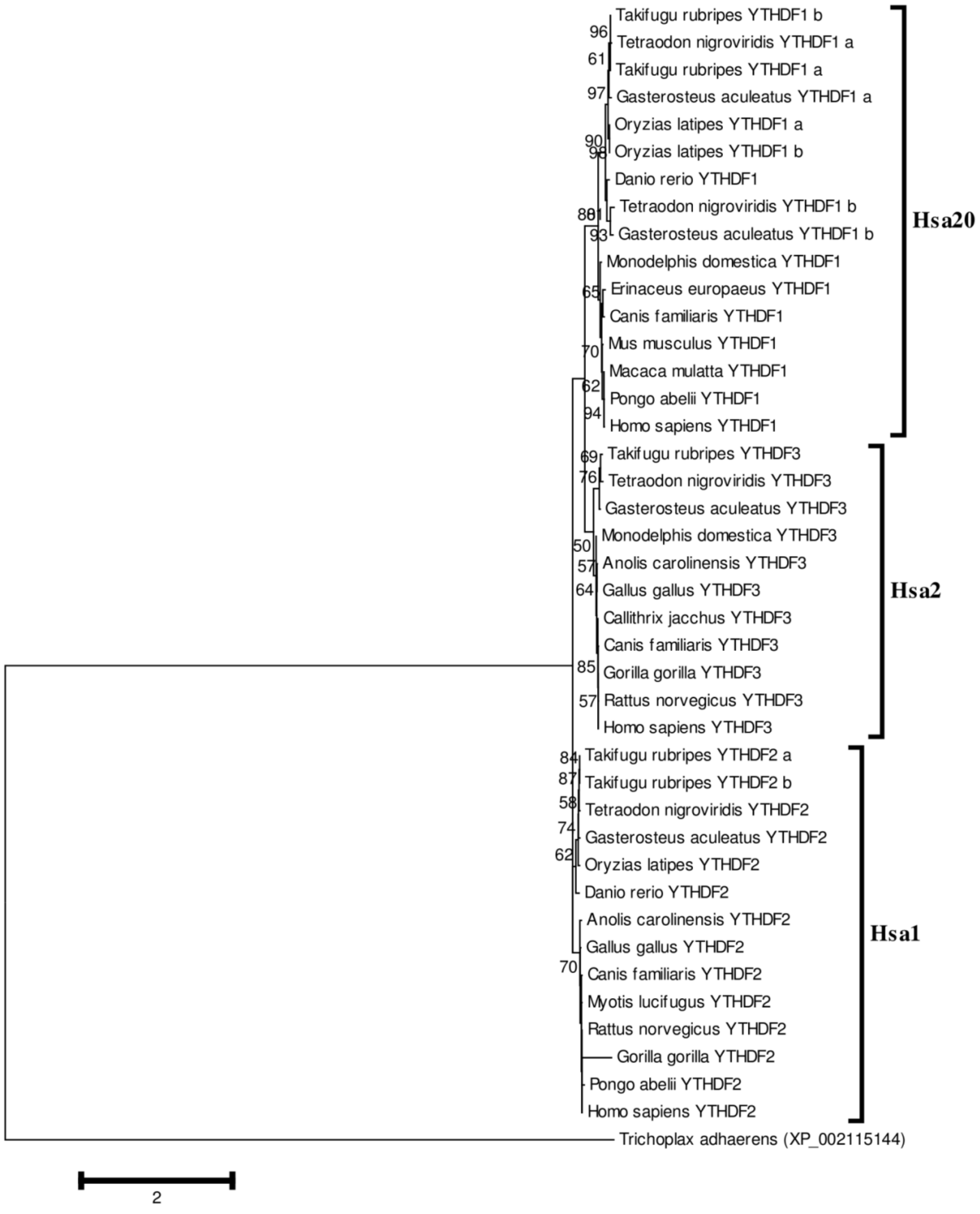

Phylogenetic tree of YTHDF family.

The evolutionary history was inferred by using the Maximum Likelihood method based on the Whelan And Goldman model. The tree with the highest log likelihood (-4540.4192) is shown. The percentage of trees in which the associated taxa clustered together is shown next to the branches ; only the values ≥ 50% are shown here. A discrete Gamma distribution was used to model evolutionary rate differences among sites (5 categories (+*G*, parameter = 3.5033)). The tree is drawn to scale, with branch lengths measured in the number of substitutions per site. The analysis involved 42 amino acid sequences. All positions containing gaps and missing data were eliminated. There were a total of 293 positions in the final dataset.

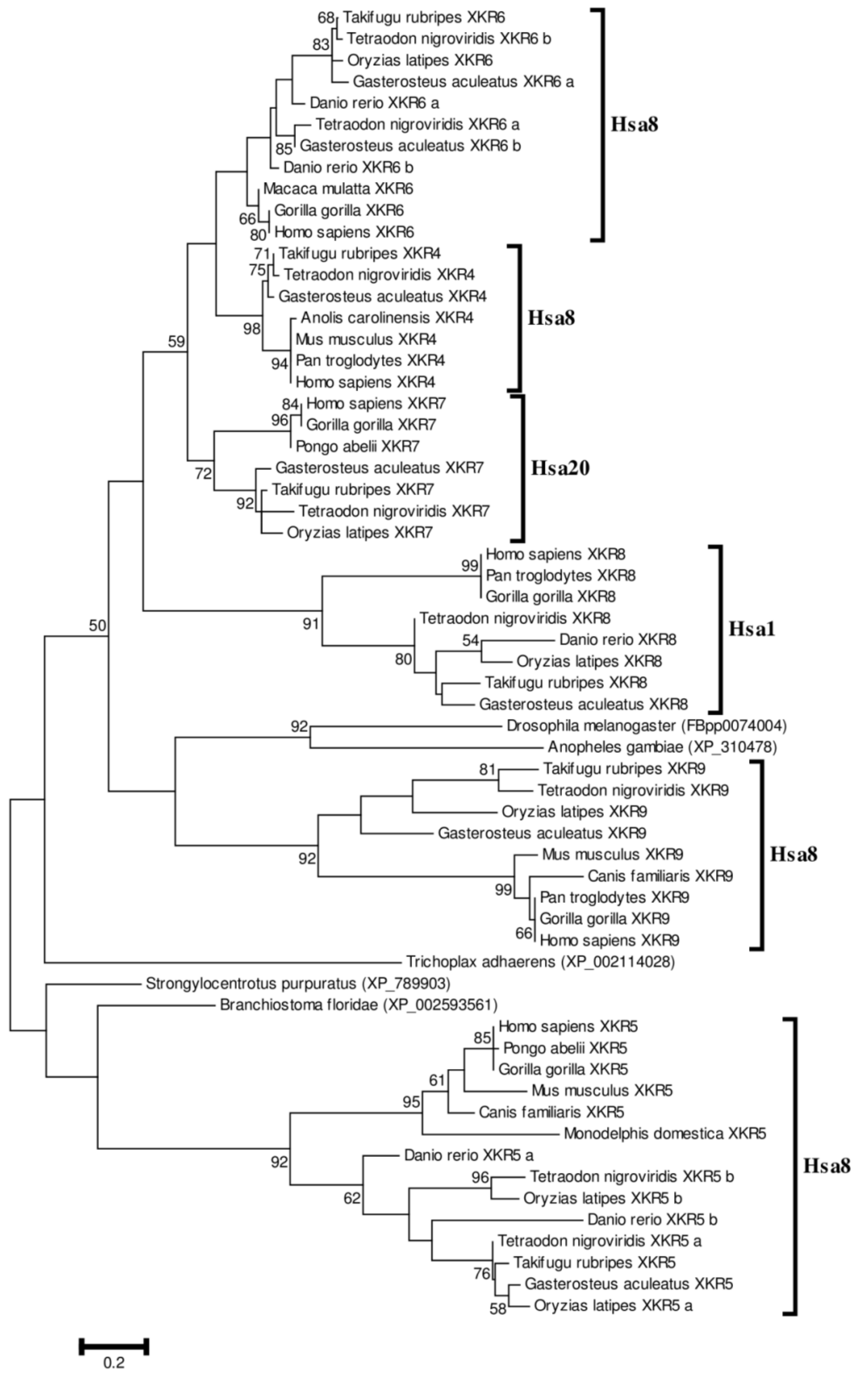

Phylogenetic tree of XKR family.

The evolutionary history was inferred by using the Maximum Likelihood method based on the Whelan And Goldman model. The tree with the highest log likelihood (-3313.2103) is shown. The percentage of trees in which the associated taxa clustered together is shown next to the branches ; only the values ≥ 50% are shown here. A discrete Gamma distribution was used to model evolutionary rate differences among sites (5 categories (+*G*, parameter = 3.1313)). The tree is drawn to scale, with branch lengths measured in the number of substitutions per site. The analysis involved 61 amino acid sequences. All positions containing gaps and missing data were eliminated. There were a total of 67 positions in the final dataset.

Myelin Transcription Factor-MYT

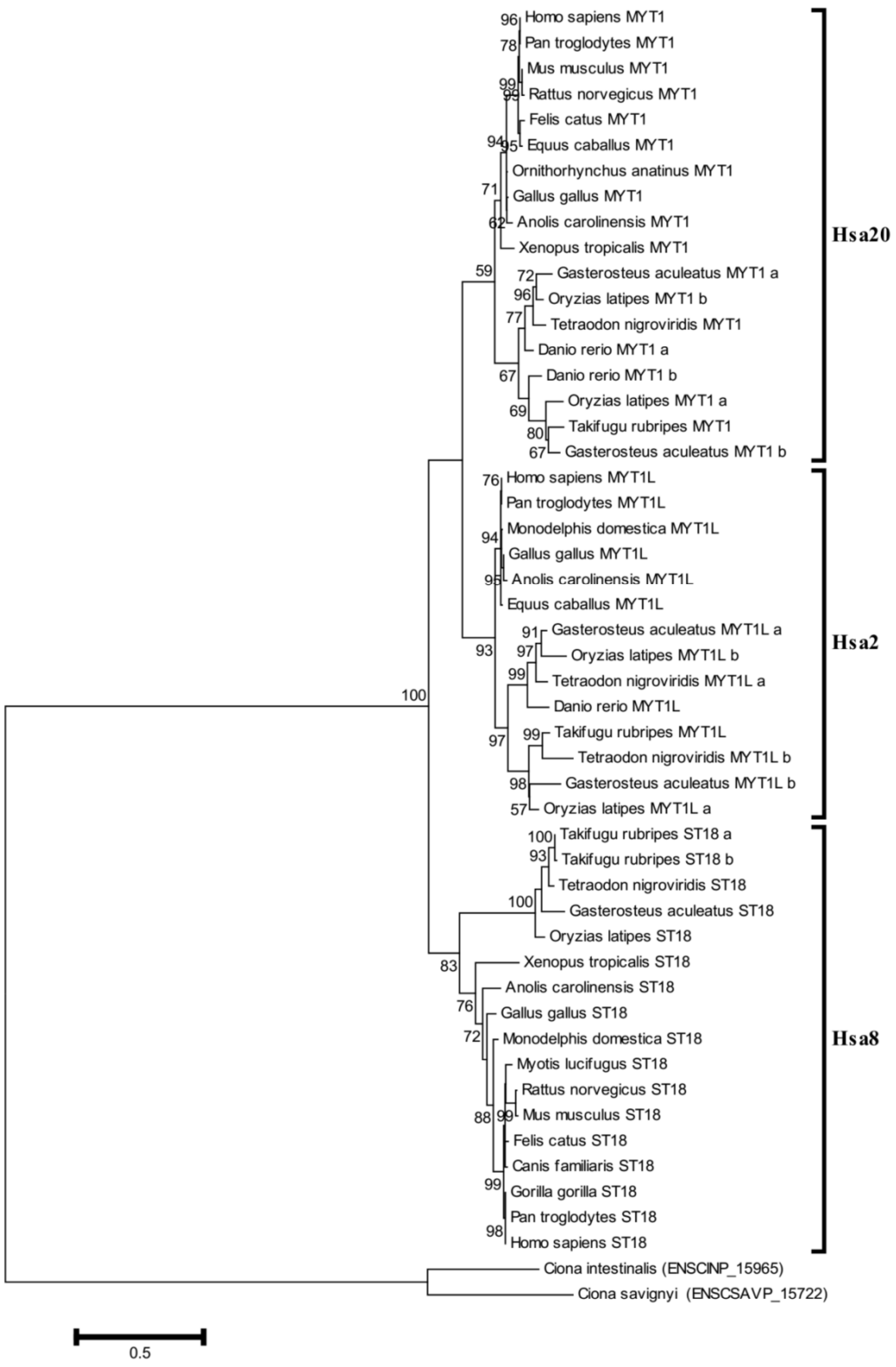

Phylogenetic tree of MYT family.

The evolutionary history was inferred by using the Maximum Likelihood method based on the Whelan And Goldman model. The tree with the highest log likelihood (-11734.7917) is shown. The percentage of trees in which the associated taxa clustered together is shown next to the branches ; only the values ≥ 50% are shown here. A discrete Gamma distribution was used to model evolutionary rate differences among sites (5 categories (+*G*, parameter = 2.3022)). The tree is drawn to scale, with branch lengths measured in the number of substitutions per site. The analysis involved 51 amino acid sequences. All positions containing gaps and missing data were eliminated. There were a total of 469 positions in the final dataset.

Other gene families analyzed in the present study

Antizyme Inhibitor-AZIN

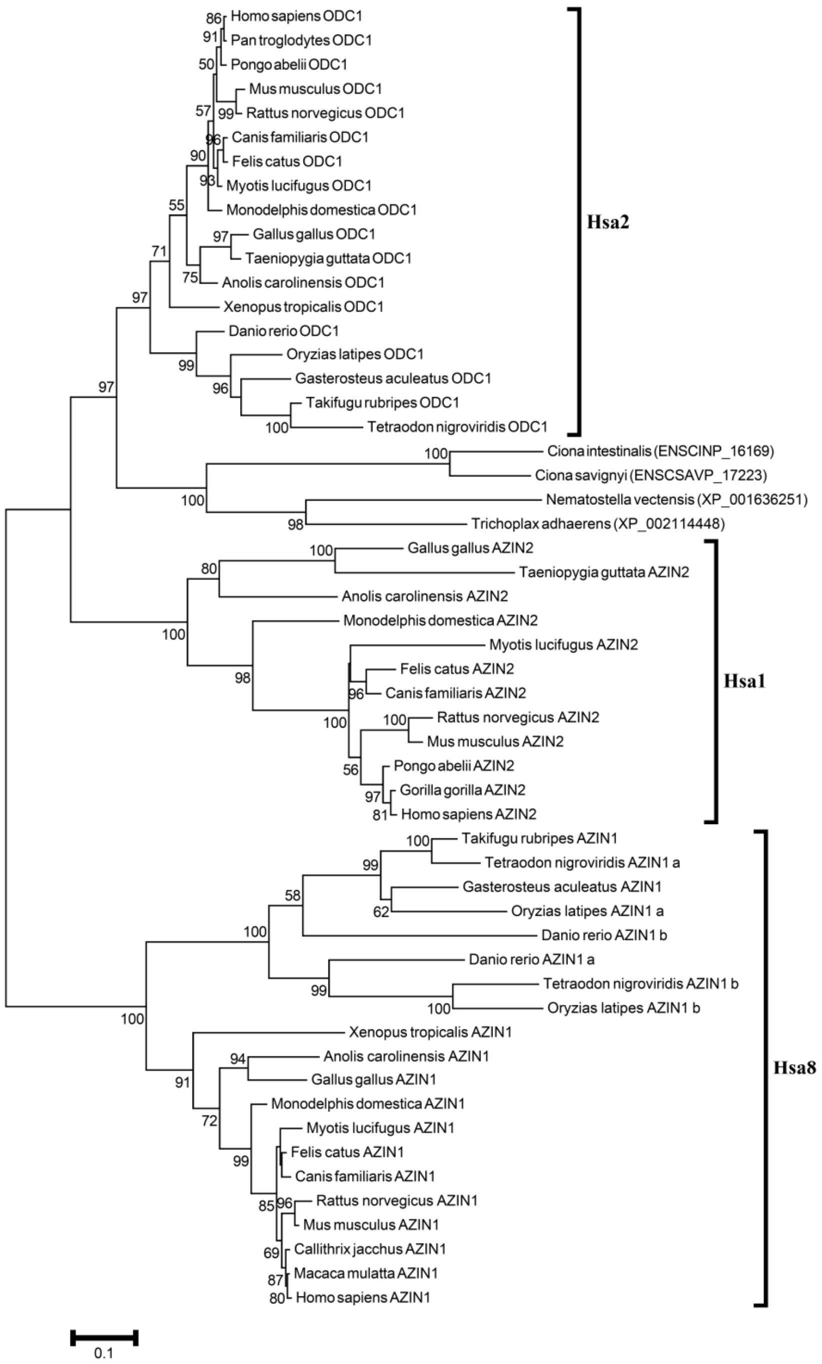

Phylogenetic tree of AZIN family.

The evolutionary history was inferred by using the Maximum Likelihood method based on the Whelan And Goldman model . The tree with the highest log likelihood (-11900.2271) is shown. The percentage of trees in which the associated taxa clustered together is shown next to the branches; only the values ≥ 50% are shown here. A discrete Gamma distribution was used to model evolutionary rate differences among sites (5 categories (+*G*, parameter = 2.2632)). The tree is drawn to scale, with branch lengths measured in the number of substitutions per site. The analysis involved 54 amino acid sequences. All positions containing gaps and missing data were eliminated. There were a total of 327 positions in the final dataset.

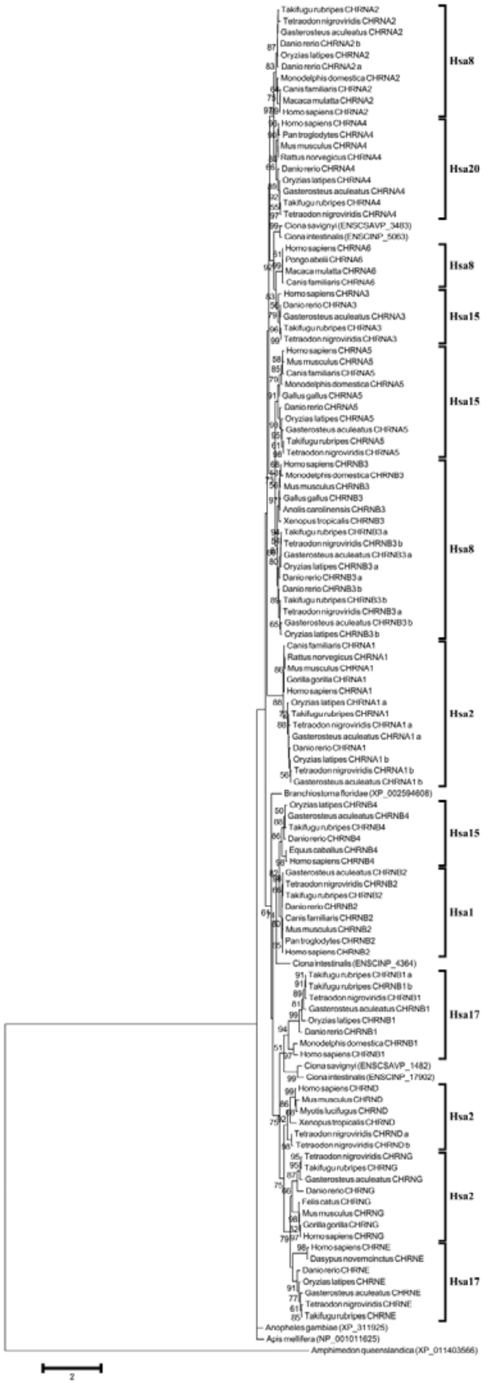

Phylogenetic tree of CHRN family.

The evolutionary history was inferred by using the Maximum Likelihood method based on the Whelan And Goldman model. The tree with the highest log likelihood (-10408.3266) is shown. The percentage of trees in which the associated taxa clustered together is shown next to the branches ; only the values ≥ 50% are shown here. A discrete Gamma distribution was used to model evolutionary rate differences among sites (5 categories (+*G*, parameter = 1.2835)). The tree is drawn to scale, with branch lengths measured in the number of substitutions per site. The analysis involved 119 amino acid sequences. All positions containing gaps and missing data were eliminated. There were a total of 182 positions in the final dataset.

Ciliary Rootlet Coiled-Coil Protein-CRO

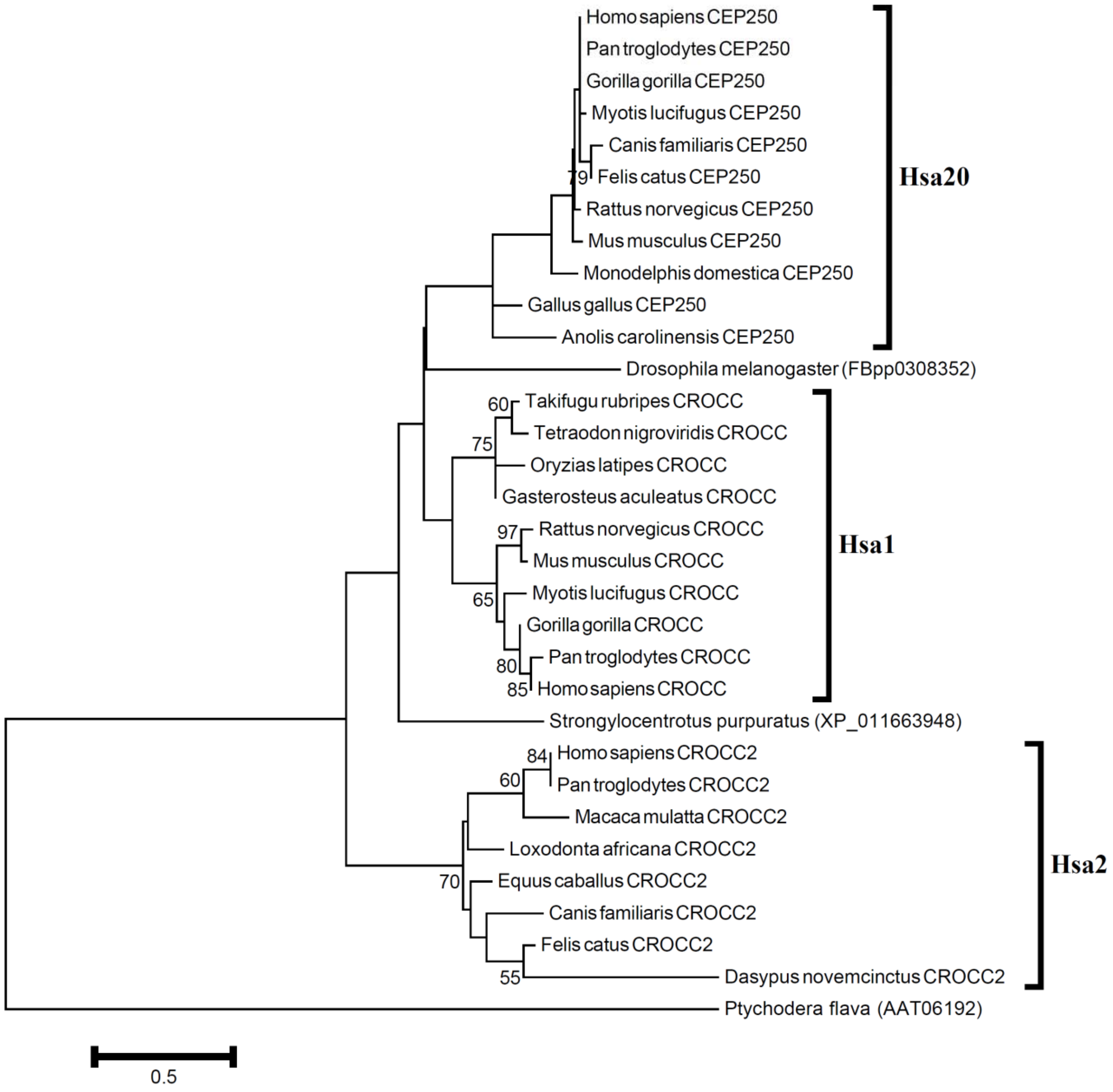

Phylogenetic tree of CRO family.

The evolutionary history was inferred by using the Maximum Likelihood method based on the Whelan And Goldman model. The tree with the highest log likelihood (-1419.2573) is shown. The percentage of trees in which the associated taxa clustered together is shown next to the branches ; only the values ≥ 50% are shown here. A discrete Gamma distribution was used to model evolutionary rate differences among sites (5 categories (+*G*, parameter = 7.5189)). The tree is drawn to scale, with branch lengths measured in the number of substitutions per site. The analysis involved 32 amino acid sequences. All positions containing gaps and missing data were eliminated. There were a total of 49 positions in the final dataset.

Grainyhead like Transcription factor-GRHL

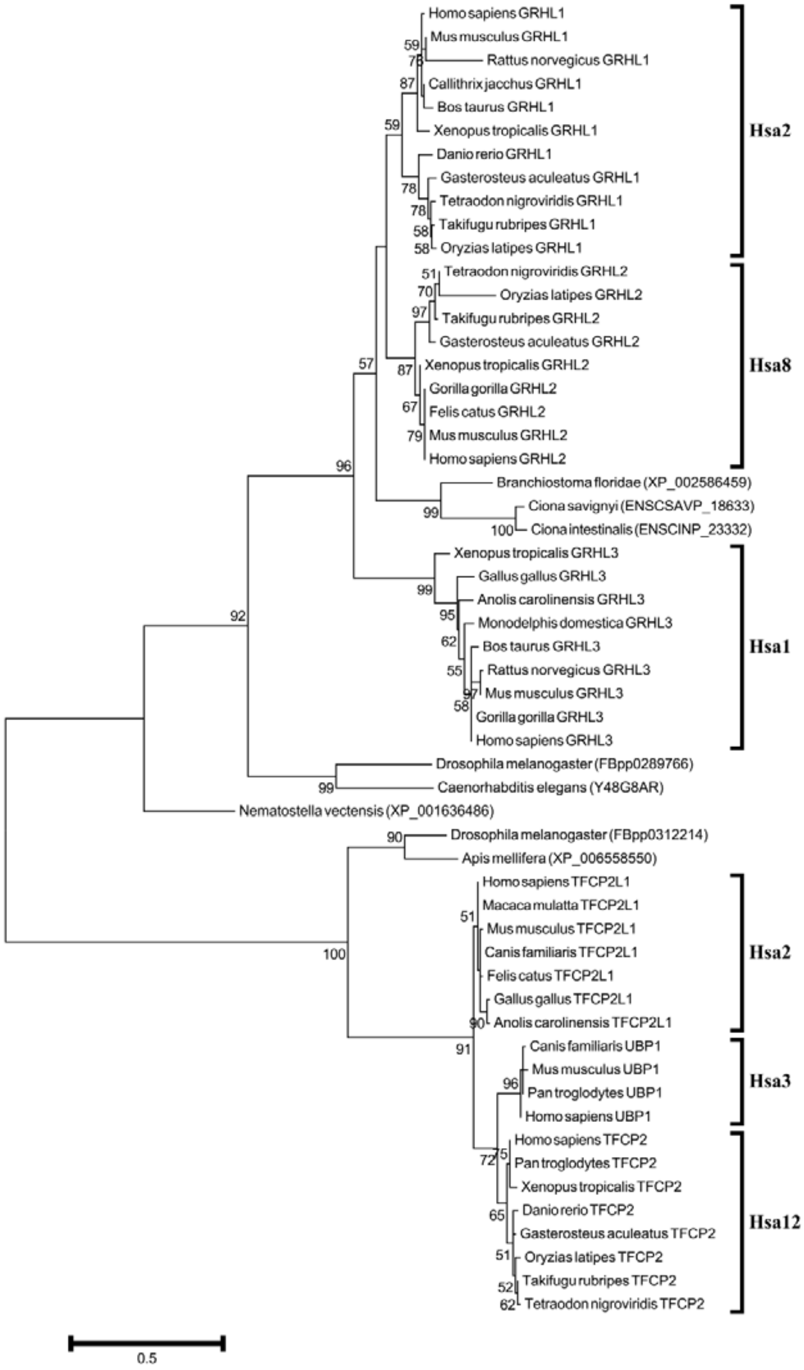

Phylogenetic tree of GRHL family.

The evolutionary history was inferred by using the Maximum Likelihood method based on the Whelan And Goldman model. The tree with the highest log likelihood (-3783.1102) is shown. The percentage of trees in which the associated taxa clustered together is shown next to the branches ; only the values ≥ 50% are shown here. A discrete Gamma distribution was used to model evolutionary rate differences among sites (5 categories (+*G*, parameter = 2.6106)). The tree is drawn to scale, with branch lengths measured in the number of substitutions per site. The analysis involved 56 amino acid sequences. All positions containing gaps and missing data were eliminated. There were a total of 146 positions in the final dataset.

Inhibitor of DNA Binding protein-ID

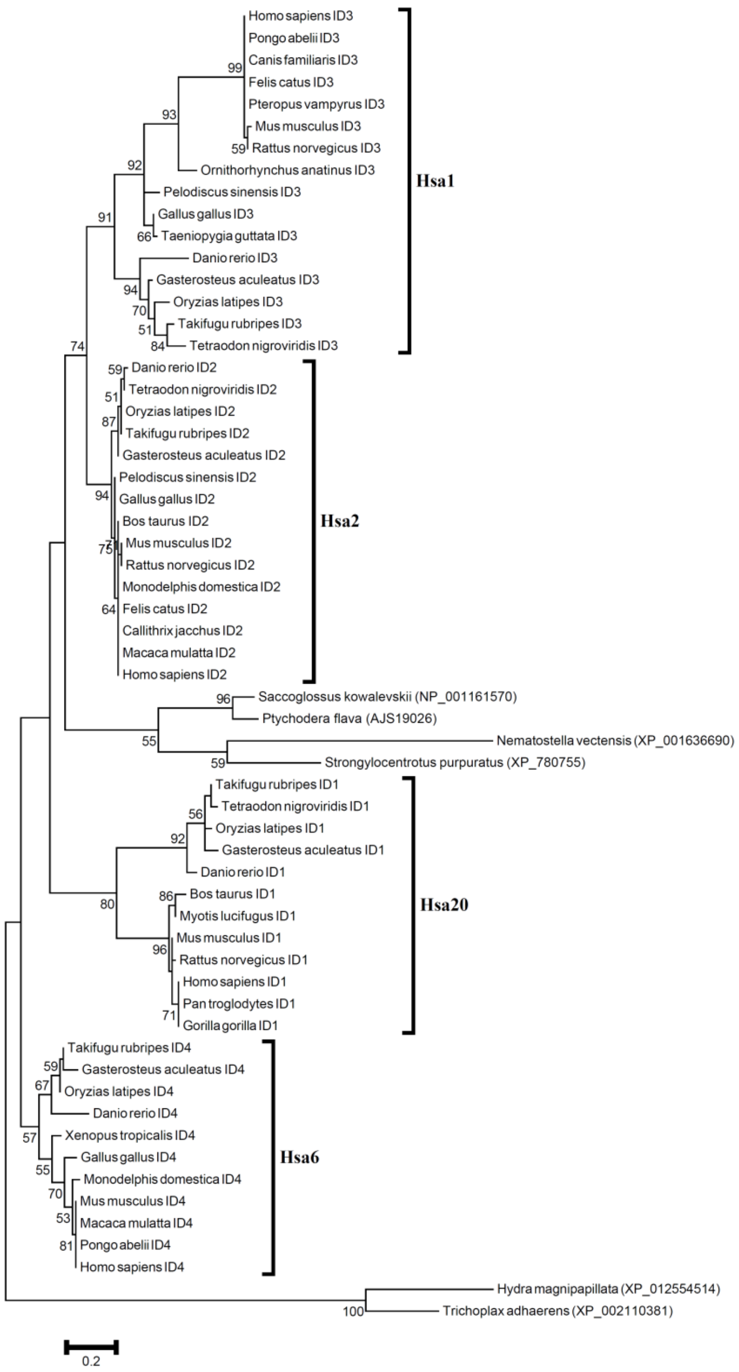

Phylogenetic tree of ID family.

The evolutionary history was inferred by using the Maximum Likelihood method based on the Whelan And Goldman model. The tree with the highest log likelihood (-2160.3041) is shown. The percentage of trees in which the associated taxa clustered together is shown next to the branches ; only the values ≥ 50% are shown here . A discrete Gamma distribution was used to model evolutionary rate differences among sites (5 categories (+*G*, parameter = 2.0164)). The tree is drawn to scale, with branch lengths measured in the number of substitutions per site. The analysis involved 60 amino acid sequences. All positions containing gaps and missing data were eliminated. There were a total of 71 positions in the final dataset.

Regulator of G-protein Signaling-RGS

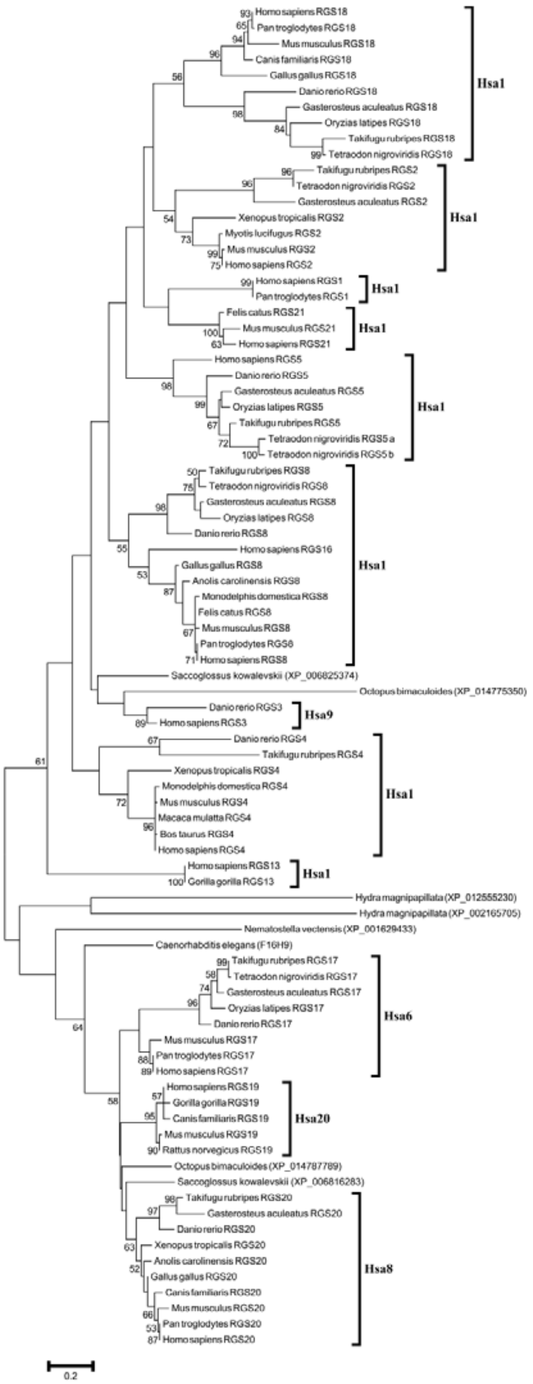

Phylogenetic tree of RGS family.

The evolutionary history was inferred by using the Maximum Likelihood method based on the Whelan And Goldman model. The tree with the highest log likelihood (-7082.8656) is shown. The percentage of trees in which the associated taxa clustered together is shown next to the branches ; only the values ≥ 50% are shown here. A discrete Gamma distribution was used to model evolutionary rate differences among sites (5 categories (+*G*, parameter = 1.9111)). The tree is drawn to scale, with branch lengths measured in the number of substitutions per site. The analysis involved 85 amino acid sequences. All positions containing gaps and missing data were eliminated. There were a total of 115 positions in the final dataset.

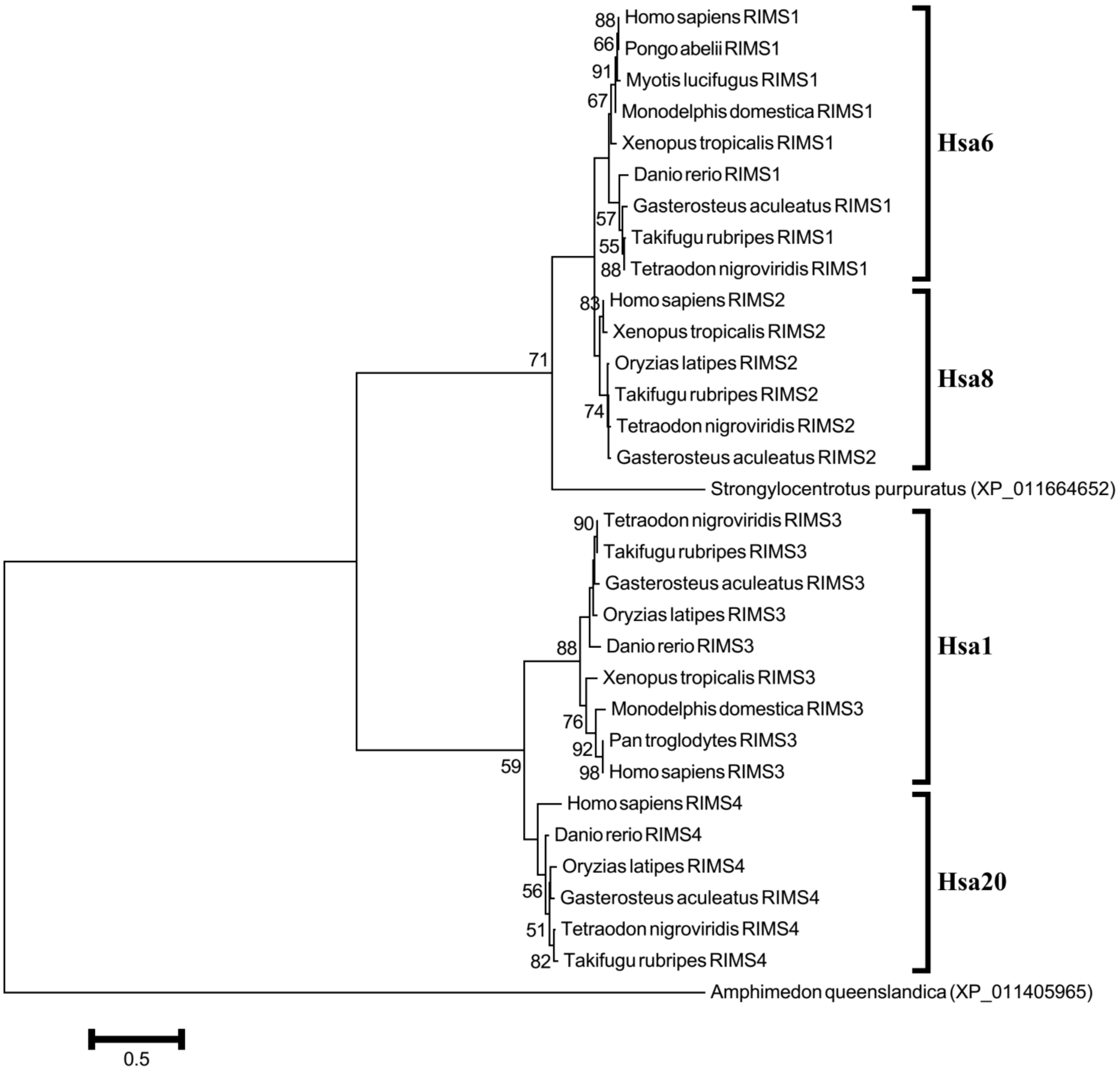

Phylogenetic tree of RIMS family.

The evolutionary history was inferred by using the Maximum Likelihood method based on the Whelan And Goldman model. The tree with the highest log likelihood (-3878.5361) is shown. The percentage of trees in which the associated taxa clustered together is shown next to the branches ; only the values ≥ 50% are shown here. A discrete Gamma distribution was used to model evolutionary rate differences among sites (5 categories (+*G*, parameter = 8.5411)). The tree is drawn to scale, with branch lengths measured in the number of substitutions per site. The analysis involved 32 amino acid sequences. All positions containing gaps and missing data were eliminated. There were a total of 211 positions in the final dataset.

R-Spondin Homolog-RSPO

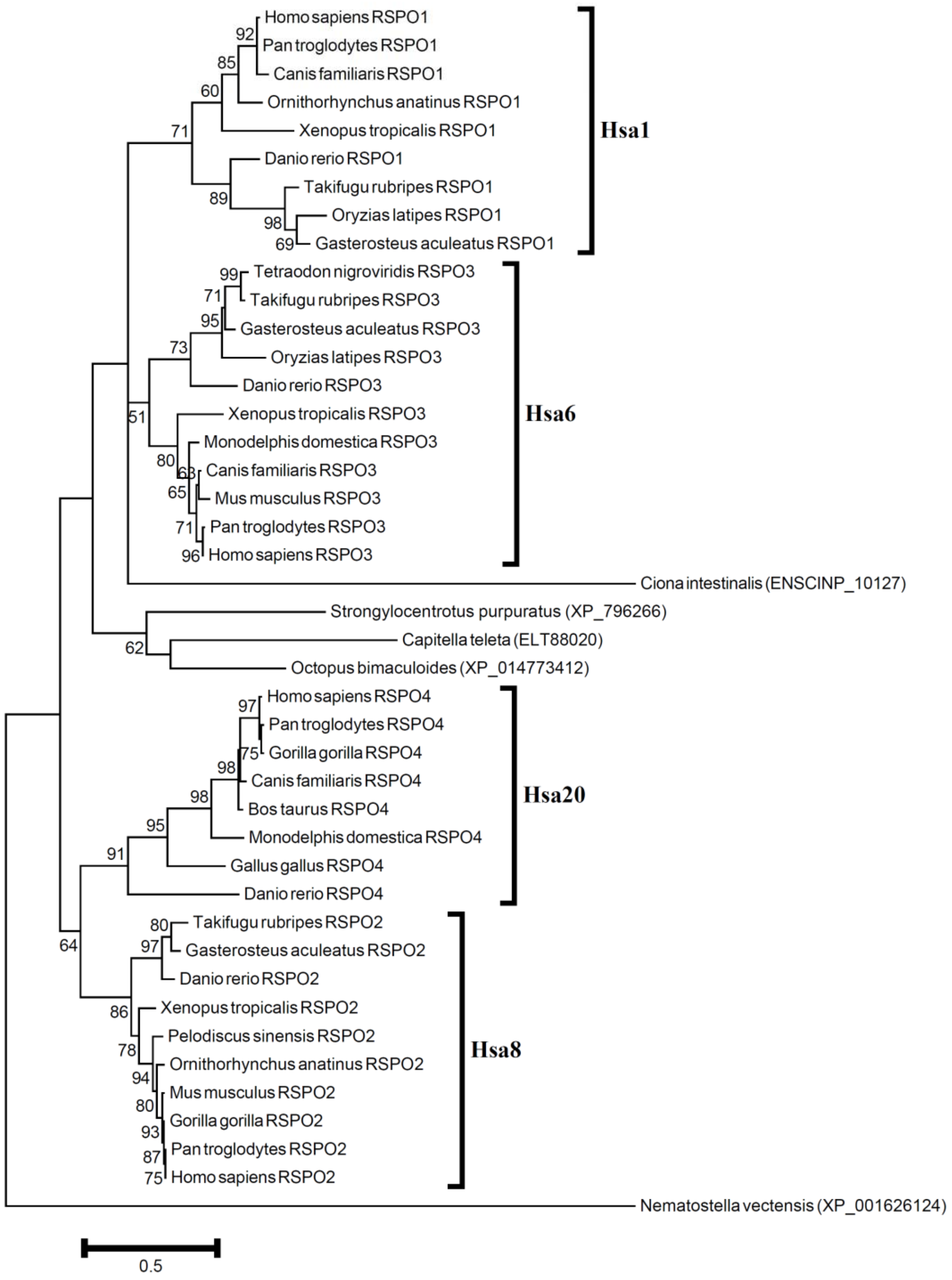

Phylogenetic tree of RSPO family.

The evolutionary history was inferred by using the Maximum Likelihood method based on the Whelan And Goldman model. The tree with the highest log likelihood (-5260.9960) is shown. The percentage of trees in which the associated taxa clustered together is shown next to the branches ; only the values ≥ 50% are shown here. A discrete Gamma distribution was used to model evolutionary rate differences among sites (5 categories (+*G*, parameter = 2.6368)). The tree is drawn to scale, with branch lengths measured in the number of substitutions per site. The analysis involved 43 amino acid sequences. All positions containing gaps and missing data were eliminated. There were a total of 141 positions in the final dataset.

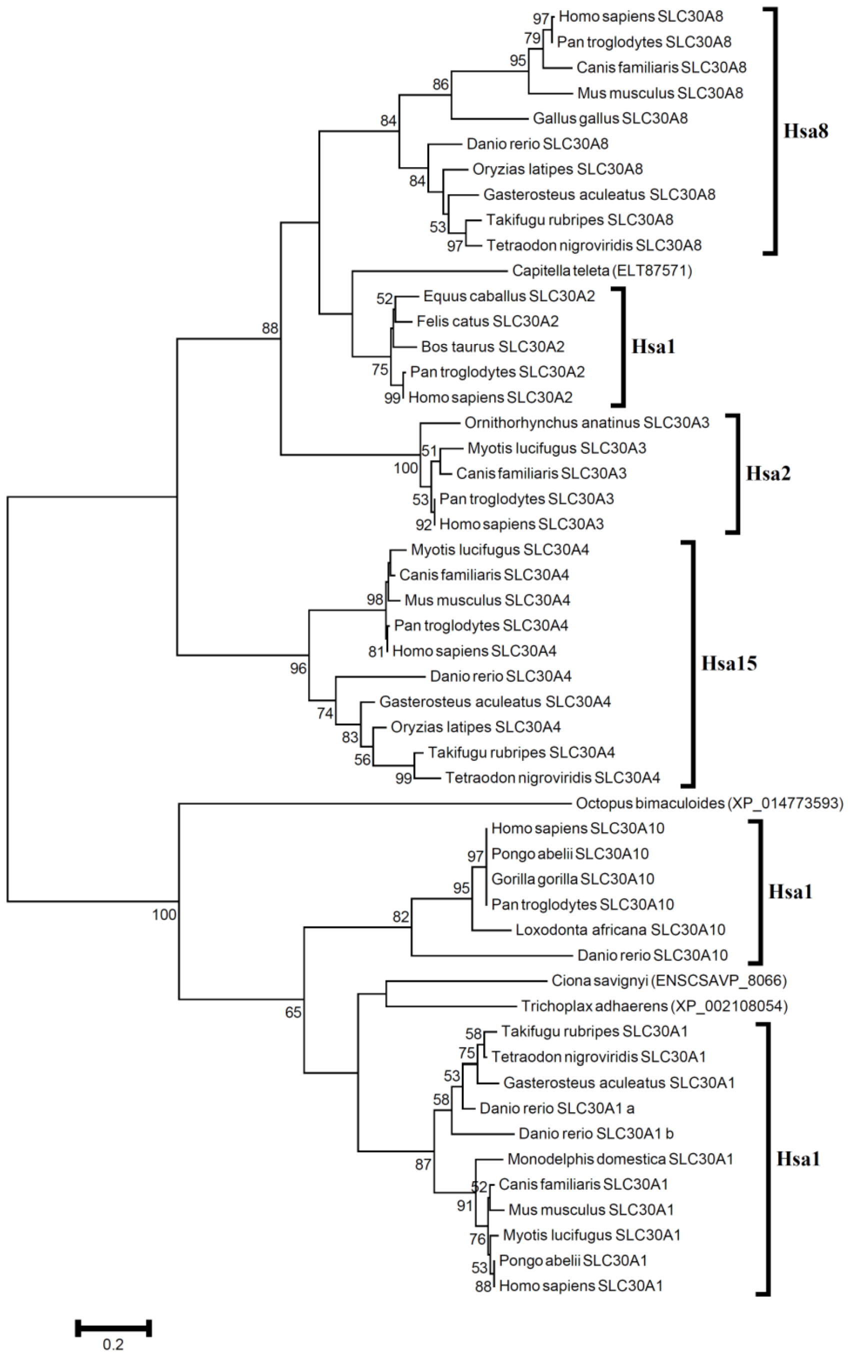

Phylogenetic tree of SLC family.

The evolutionary history was inferred by using the Maximum Likelihood method based on the Whelan And Goldman model. The tree with the highest log likelihood (-4999.4232) is shown. The percentage of trees in which the associated taxa clustered together is shown next to the branches ; only the values ≥ 50% are shown here. A discrete Gamma distribution was used to model evolutionary rate differences among sites (5 categories (+*G*, parameter = 2.3472)). The tree is drawn to scale, with branch lengths measured in the number of substitutions per site. The analysis involved 68 amino acid sequences. All positions containing gaps and missing data were eliminated. There were a total of 131 positions in the final dataset.

Syntrophin, Gamma-SNT

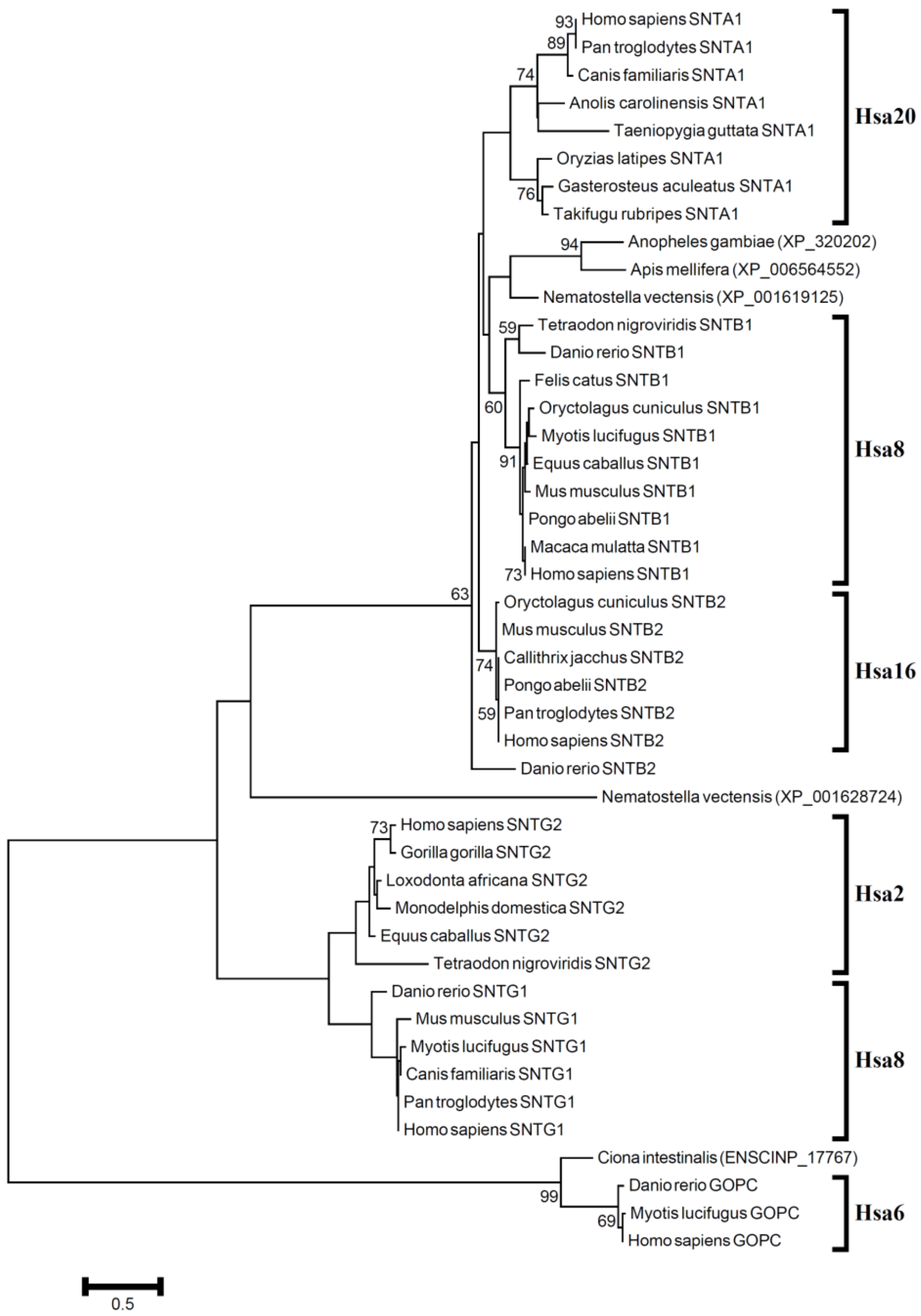

Phylogenetic tree of SNT family.

The evolutionary history was inferred by using the Maximum Likelihood method based on the Whelan And Goldman model. The tree with the highest log likelihood (-2524.4722) is shown. The percentage of trees in which the associated taxa clustered together is shown next to the branches ; only the values ≥ 50% are shown here. A discrete Gamma distribution was used to model evolutionary rate differences among sites (5 categories (+*G*, parameter = 5.9303)). The tree is drawn to scale, with branch lengths measured in the number of substitutions per site. The analysis involved 45 amino acid sequences. All positions containing gaps and missing data were eliminated. There were a total of 67 positions in the final dataset.

Sorting Nexin Family-SNX

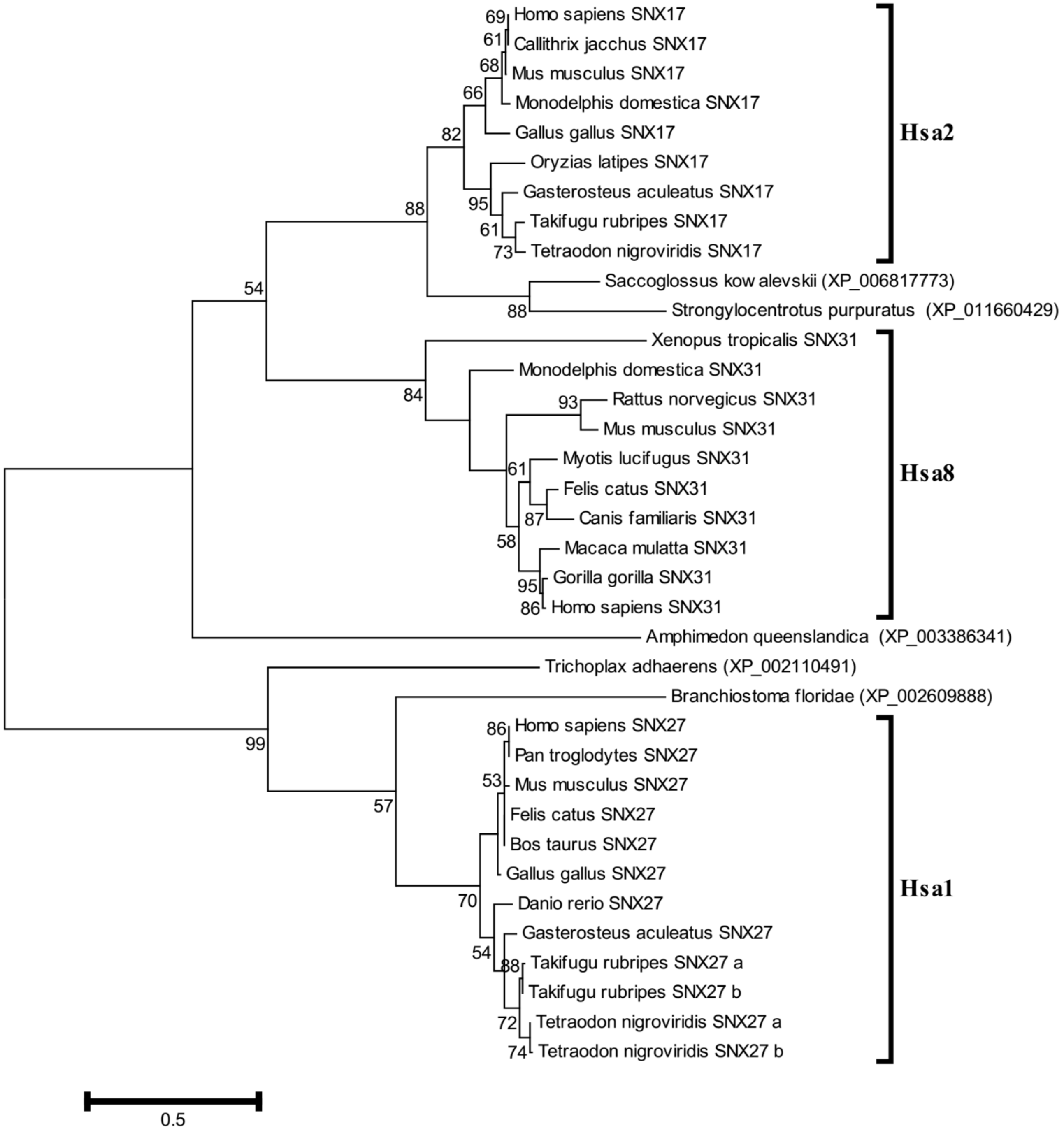

Phylogenetic tree of SNX family.

The evolutionary history was inferred by using the Maximum Likelihood method based on the Whelan And Goldman model. The tree with the highest log likelihood (-4476.6316) is shown. The percentage of trees in which the associated taxa clustered together is shown next to the branches ; only the values ≥ 50% are shown here. A discrete Gamma distribution was used to model evolutionary rate differences among sites (5 categories (+*G*, parameter = 5.5801)). The tree is drawn to scale, with branch lengths measured in the number of substitutions per site. The analysis involved 36 amino acid sequences. All positions containing gaps and missing data were eliminated. There were a total of 153 positions in the final dataset.

Transcription Elongation factor A (SII) Protein-TCEA

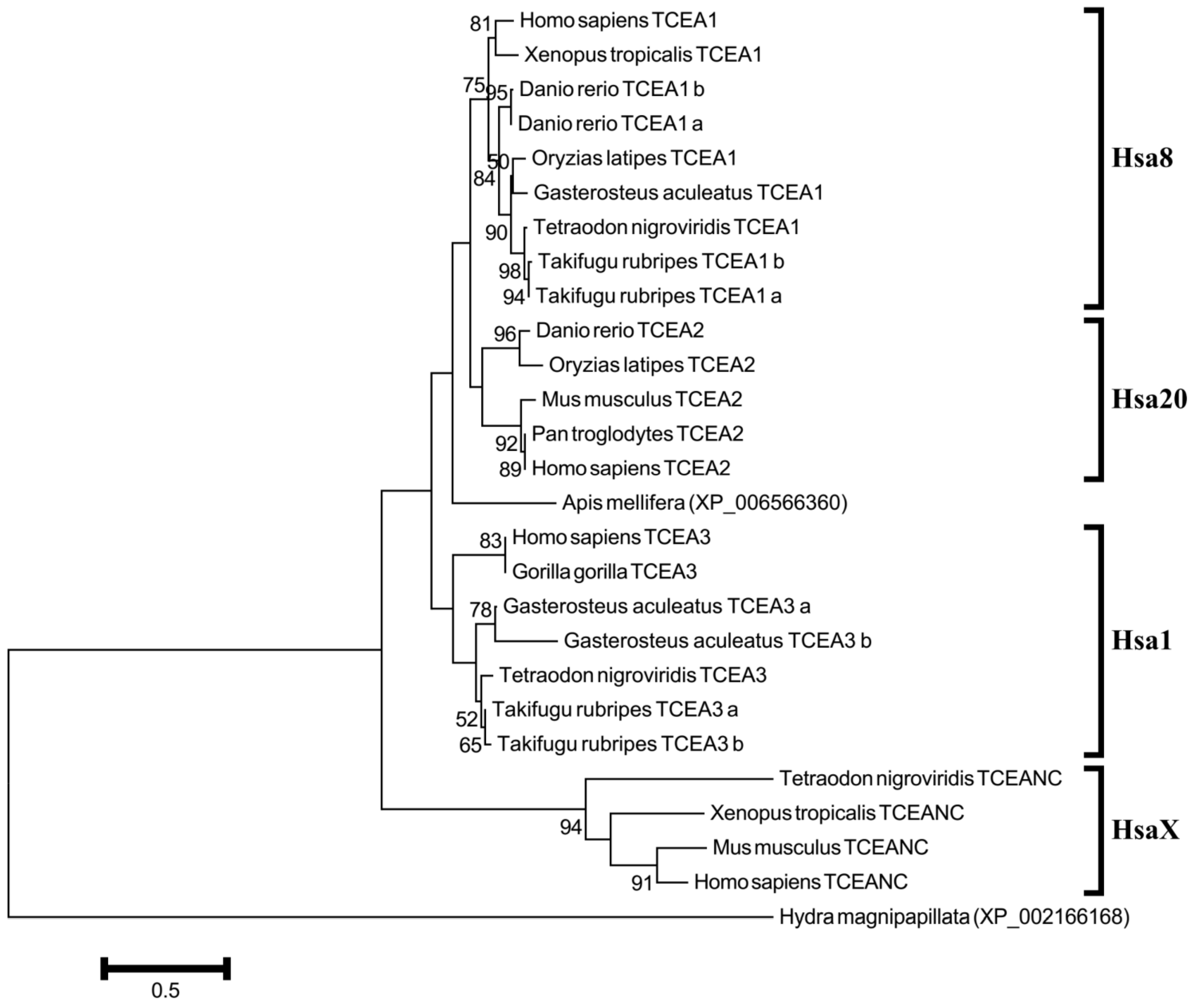

Phylogenetic tree of TCEA family.

The evolutionary history was inferred by using the Maximum Likelihood method based on the Whelan And Goldman model. The tree with the highest log likelihood (-4688.8104) is shown. The percentage of trees in which the associated taxa clustered together is shown next to the branches ; only the values ≥ 50% are shown here. A discrete Gamma distribution was used to model evolutionary rate differences among sites (5 categories (+*G*, parameter = 2.5721)). The tree is drawn to scale, with branch lengths measured in the number of substitutions per site. The analysis involved 27 amino acid sequences. All positions containing gaps and missing data were eliminated. There were a total of 197 positions in the final dataset.

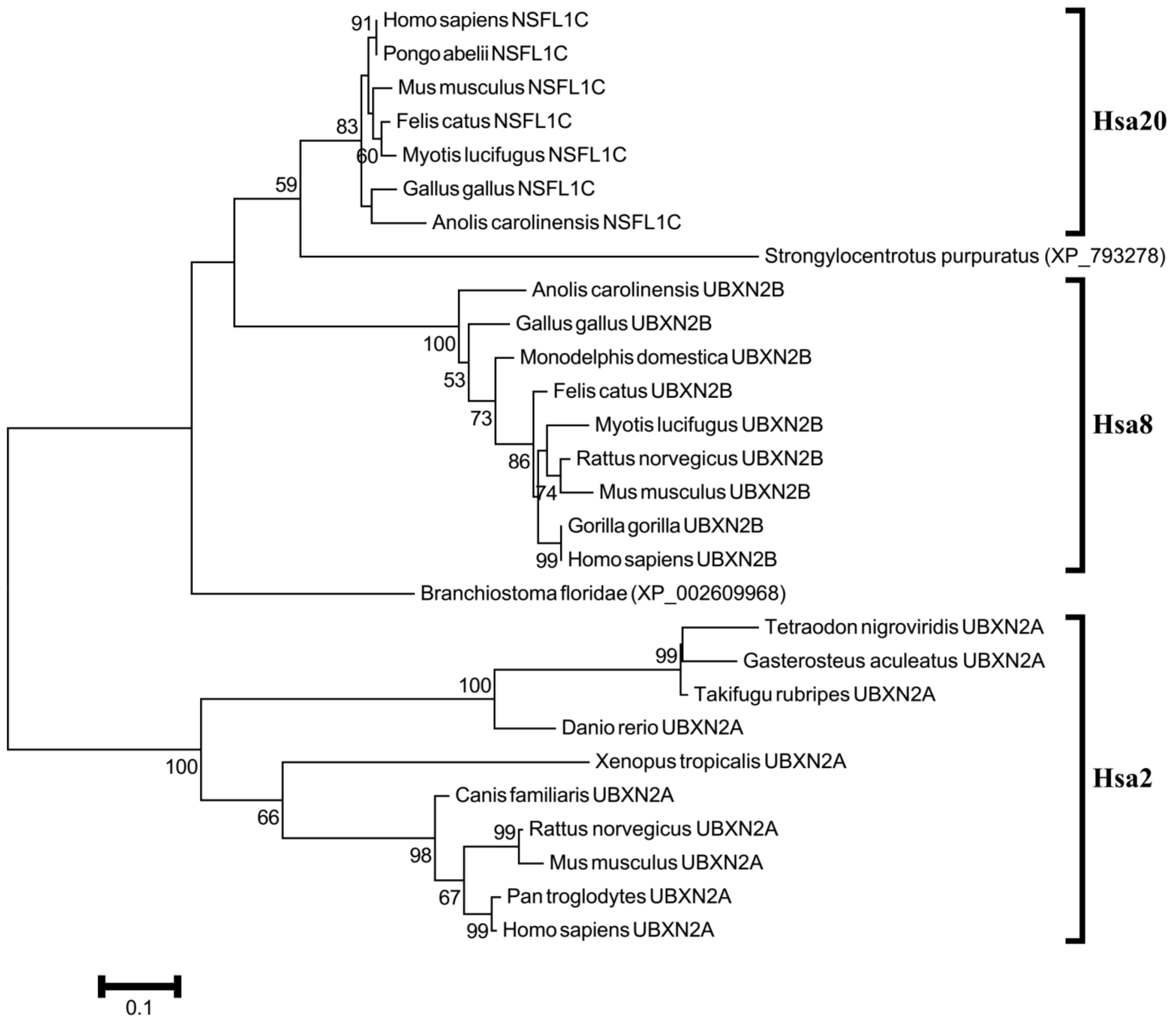

Phylogenetic tree of UBXN family.

The evolutionary history was inferred by using the Maximum Likelihood method based on the Whelan And Goldman model. The tree with the highest log likelihood (-3195.4561) is shown. The percentage of trees in which the associated taxa clustered together is shown next to the branches ; only the values ≥ 50% are shown here. A discrete Gamma distribution was used to model evolutionary rate differences among sites (5 categories (+*G*, parameter = 2.3205)). The tree is drawn to scale, with branch lengths measured in the number of substitutions per site. The analysis involved 28 amino acid sequences. All positions containing gaps and missing data were eliminated. There were a total of 163 positions in the final dataset.
