# Supplementary material for: Evolutionary history of the human multigene families reveals widespread gene duplications throughout the history of animals
Source: BMC Evol Biol. 2019 Jun 20;19:128. doi: 10.1186/s12862-019-1441-0 (PMC6585022; doi:10.1186/s12862-019-1441-0)
Supplement: Supplementary file 5 — Table S1. Summary of the Phylogenetic analysis of gene families with three or more members are residing on human chromosomes 1/2/8/20. (PDF 73 kb) [file 12862_2019_1441_MOESM5_ESM.pdf]

Table S5: Summary of the Phylogenetic analysis of gene families whose three or more members are residing on human chromosomes 1, 2, 8 and 20.

Family name	Hsa1/13[a]	Hsa2/6[b]	Hsa8/X[c]	Hsa20/18[d]	Topology
EYA	EYA3	EYA4[b]	EYA1	EYA2	(((6,8) 20) 1) 99, 76
STMN	STMN1	-	STMN4<br>STMN2	STMN3	(((1,8) 8) 20) 52, 100
E2F	E2F2	E2F3[b]	-	E2F1	((1,6) 20) 82, 77
MROH	MROH7	-	MROH5<br>MROH6	MROH8	(((20,1) 8) 8) 53, 78
STK	STK24[a]	STK25	STK26[c]	-	((2,13) X) 77, 98
HCK	LCK	-	LYN<br>BLK	HCK	(((8,20) (8,1)) 98,85
DLGAP	DLGAP3	-	DLGAP2	DLGAP4<br>DLGAP1[d]	((8,1) (20,18)) 67, 100, 71
NKAIN	NKAIN1	NKAIN2[b]	NKAIN3	NKAIN4	((20,8) (1,6)) 56, 99, 91
KCNQ	KCNQ4	KCNQ5[b]	KCNQ3	KCNQ2	((6,1)(8,20)) 99,85, 69
MATN	MATN1	MATN3	MATN2	MATN4	((20,8) (1,2)) 74
YTHDF	YTHDF2	-	YTHDF3	YTHDF1	((20,8) 1) 78,100
XKR	XKR8	-	XKR6<br>XKR4	XKR7	(((8,8) 20) 1) 70, 89, 64
MYT	-	MYT1L	ST18	MYT1	((20,2) 8) 99, 100
FAM110	-	FAM110C	FAM110B	FAM110A	((8,20) 2) 100, 62
NCOA	-	NCOA1	NCOA2	NCOA3	((20,8) 2) 100, 100
KCNS	-	KCNS3	KCNS2	KCNS1	((20,8)2) 99

For each gene family the chromosomal location and topologies of those genes are given, which arose through duplications after the invertebrates-vertebrates split and before the tetrapods-fishes divergence. In this table only those gene families are given which are present in co-duplicated groups. The percentage bootstrap support of the internal branches is given below each relevant topology.

[a, b, c] Indicates that the gene family members are not positioned on Hsa 1, 2, 8 and 20 respectively.
